# Wuji Wan ameliorates ulcerative colitis by restoring impaired membrane transport

**DOI:** 10.3389/fphar.2026.1718919

**Published:** 2026-01-27

**Authors:** Jianyu Zhang, Yang Zhang, Yuxuan Guo, Yi Sun, Yujie Li, Qi Li, Ying Chen, Yajie Wang, Qing Yang, Meiyu Zhang, Xiaogang Weng, Zhihao Deng

**Affiliations:** 1 Institute of Chinese Materia Medica, China Academy of Chinese Medical Sciences, Beijing, China; 2 Experimental Research Center, China Academy of Chinese Medical Sciences, Beijing, China; 3 Dongzhimen Hospital, Beijing University of Chinese Medicine, Beijing, China

**Keywords:** diarrhea, inflammation, ion channels, membrane transport, ulcerative colitis, voltage-gated potassium channel 1.3, Wuji Wan

## Abstract

**Introduction:**

Wuji Wan (WJW) is a classical Chinese formula traditionally prescribed for diarrhea/dysentery and abdominal pain. In ulcerative colitis (UC), inflammatory diarrhea reflects not only mucosal inflammation but also inflammation-linked disruption of epithelial electrolyte and water handling, highlighting membrane transport as a mechanistic bridge between symptom burden and immune activation. However, it remains unclear whether WJW confers therapeutic benefit in UC and whether any benefit is accompanied by coordinated regulation of membrane-transport–linked pathways. This study therefore asked whether WJW shows therapeutic effects in a UC model and whether these effects are accompanied by changes in epithelial Na^+^/Cl^−^ transport and water-channel programs and by modulation of the T-cell–linked potassium channel Kv1.3.

**Methods:**

We investigated this question in a mouse model of DSS-induced colitis (3% dextran sulfate sodium). Our assessment included disease activity index (DAI) scores, histopathological analysis, ELISA, Western blotting, untargeted metabolomics, and whole-cell patch-clamp electrophysiology.

**Results:**

WJW significantly ameliorated DSS-induced colitis, as reflected by improved colonic pathology and partial normalization of DSS-associated serum metabolic perturbations. Untargeted metabolomics highlighted transport-related pathways. WJW increased/normalized the expression of key epithelial transport proteins involved in Na^+^/Cl^−^ absorption and water handling, including sodium/hydrogen exchanger 3 (NHE3), epithelial sodium channel (ENaC), downregulated in adenoma (DRA), aquaporin-3 (AQP3), and aquaporin-8 (AQP8). In parallel, WJW reduced IL-6, IL-17A, and IFN-γ and dampened ERK/NF-κB pathway activation. WJW also reduced colonic Kv1.3 protein expression, and WJW-containing plasma directly inhibited Kv1.3 currents in Jurkat T cells.

**Conclusion:**

WJW ameliorated DSS-induced colitis and was accompanied by coordinated modulation of epithelial and immune membrane-transport–linked readouts.

## 1 Introduction

Ulcerative colitis (UC) is a chronic, relapsing inflammatory bowel disease in which inflammatory diarrhea is a hallmark manifestation and a major driver of disease burden and impaired quality of life (Knowles et al., 2018; Ungaro et al., 2017). Current first-line therapies—including 5-aminosalicylic acid, glucocorticoids, and biologics—primarily suppress inflammation but are limited by relapse, loss of response, and adverse effects (Rubin et al., 2025; Savelkoul et al., 2023). Importantly, improvement in inflammatory indices does not always translate into rapid or sustained resolution of diarrhea, indicating persistent functional defects that are insufficiently addressed by anti-inflammatory strategies alone (Fairbrass et al., 2020; Savelkoul et al., 2023). Therefore, mechanism-based interventions that can both control inflammation and directly correct diarrhea-related dysfunction remain an unmet clinical need (Rubin et al., 2025; Turner et al., 2021).

A proximal and testable determinant of diarrheal output is intestinal membrane transport, which governs epithelial electrolyte absorption/secretion and transepithelial water movement essential for gut homeostasis (Anbazhagan et al., 2018; Priyamvada et al., 2015). In UC, inflammatory mediators disrupt electroneutral NaCl absorption, promoting fecal electrolyte loss and watery stool (Anbazhagan et al., 2018; Greig et al., 2004). Key absorptive transport proteins—including the sodium–hydrogen exchanger 3 (NHE3), the Cl^−^/HCO_3_^−^ exchanger (DRA), and the epithelial sodium channel (ENaC)—are downregulated and/or functionally impaired under inflammatory conditions, thereby weakening Na^+^ and Cl^−^ uptake and directly contributing to diarrhea (Greig et al., 2004; Yeruva et al., 2010). In parallel, UC-associated diarrhea is also shaped by altered epithelial water handling; aquaporin-3 (AQP3) and aquaporin-8 (AQP8) have been reported to be dysregulated in colitis/UC, potentially amplifying stool water content by disturbing epithelial water permeability (Planell et al., 2013; Ricanek et al., 2015; Zahn et al., 2007).

Beyond epithelial dysfunction, UC pathogenesis is critically shaped by T lymphocytes, and T-cell–driven cytokine networks can propagate mucosal inflammation that secondarily suppresses epithelial transport and barrier function (Ghishan and Kiela, 2014; Musch et al., 2002; Neurath, 2019). T-cell activation programs are intrinsically dependent on ion-channel–regulated membrane potential and Ca^2+^ signaling (Feske et al., 2015; Vaeth et al., 2020). Among these channels, the voltage-gated K^+^ channel Kv1.3 is a key regulator of T-cell excitability and activation, and its aberrant activity can facilitate pro-inflammatory signaling and cytokine release (Koch Hansen et al., 2014; Wulff et al., 2009). In UC/colitis contexts, increased Kv1.3 expression and/or activity has been reported in effector T cells, supporting a channel-linked mechanism that may amplify inflammation and perpetuate epithelial transport dysfunction (Koch Hansen et al., 2014; Unterweger et al., 2021). Collectively, these features position membrane transport proteins as rational therapeutic targets in UC because they mechanistically connect inflammatory signaling to a clinically meaningful functional endpoint (diarrhea) and provide quantifiable readouts for therapeutic evaluation (Ghishan and Kiela, 2014; Magalhães et al., 2016; Priyamvada et al., 2015).

Traditional Chinese medicine (TCM) emphasizes multitarget regulation and is widely applied in UC management (Zhang et al., 2024; Zong et al., 2023). Wuji Wan (WJW), composed of *Coptis chinensis* Franch., *Tetradium ruticarpum* (A.Juss.) T.G.Hartley, and *Paeonia lactiflora* Pall. in a 6:1:6 ratio, has long been used for gastrointestinal disorders characterized by diarrhea and abdominal pain (Jing et al., 2025; Wu et al., 2024). Notably, modern pharmacological evidence suggests that WJW may influence UC not only through broad anti-inflammatory actions but also via membrane-transport–relevant mechanisms, providing a focused rationale that aligns with its traditional antidiarrheal use. Berberine, a representative alkaloid in WJW, has been reported to upregulate NHE3 in intestinal tissue and to modulate K^+^-channel activity in colonic epithelial models, supporting a plausible link to epithelial electrolyte handling and membrane excitability (Alzamora et al., 2011; Zhang et al., 2012; Zhu et al., 2023). In addition, paeoniflorin and related metabolites have been implicated in Ca^2+^-associated signaling processes that intersect with ion-channel regulation and T-cell activation (Fei et al., 2019; Tsai et al., 2005).

Nevertheless, a key research gap persists: existing studies on WJW in UC have predominantly emphasized holistic anti-inflammatory or microbiota-related outcomes (Jing et al., 2025), whereas it remains unclear whether WJW can coordinately restore epithelial electrolyte/water transport and concurrently restrain T-cell ion-channel–dependent activation as an integrated mechanism. The field lacks a coherent evidence chain that bridges (i) phenotypic antidiarrheal efficacy, (ii) system-level pathway signals, and (iii) molecular plus electrophysiological validation of membrane-transport restoration.

To address this gap, we test the falsifiable hypothesis that WJW alleviates UC by coordinately restoring intestinal membrane transport, thereby improving diarrhea-related dysfunction while suppressing T-cell–mediated hyperinflammatory signaling. We establish therapeutic efficacy in a UC mouse model and bridge phenotype to mechanism using serum untargeted metabolomics, in which KEGG enrichment of differential metabolites highlights membrane-transport–related pathways as key perturbed nodes. Guided by this system-level signal, we quantify the expression of colonic epithelial electrolyte and water transport proteins (NHE3, DRA, ENaC; AQP3, AQP8), alongside inflammatory signaling (ERK/NF-κB phosphorylation) and T-cell–linked cytokines (IL-6, IL-1β, IL-17A), and integrate these layers using correlation analysis to test the coherence of a phenotype–metabolome–transport–inflammation framework. To interrogate the T-cell ion-channel arm with functional rigor, we assess Kv1.3 protein expression in colonic tissue *in vivo* and measure Kv1.3 currents in Jurkat T cells by patch-clamp electrophysiology, providing a direct functional readout that bridges the expression–function gap and supports the relevance of ion-channel modulation to UC immunopathology.

## 2 Materials and methods

### 2.1 Chemicals and reagents

Dextran sulfate sodium (DSS; MW 36,000–50,000; MP Biomedicals, cat. no. 0216011080) and mesalazine sustained-release granules (IPSEN) were purchased. ELISA kits for fecal CALP and IL-6/IL-17A/IFN-γ (Ruixin Bio, cat. nos. RXM201855M, RXM203049M, RXW203066M, RXM203097M), Na^+^/Cl^−^ assay kits (Nanjing Jiancheng, cat. nos. C002-1-1, C003-1-1), and margatoxin (MCE, cat. no. HY-P1280) were used. Chemicals were purchased from the following suppliers: NaCl (S9888), KCl (P3911), CaCl_2_·2H_2_O (223506), MgCl_2_·6H_2_O (M2670), glucose (G5767), Na_2_ATP (A2383), Na_2_GTP (V900868), and EGTA (E4378) were from Sigma-Aldrich (St. Louis, MO, United States). HEPES (1112GR025) was from BioFroxx (Einhausen, Germany). K-aspartate (L822242) and KH_2_PO_4_ (P815662) were from Macklin (Shanghai, China). Chemical standards for paeoniflorin (lot no. B21148), evodiamine (lot no. B21315), rutaecarpine (lot no. B21314), berberine hydrochloride (lot no. B21449), and palmatine hydrochloride (lot no. B21433) were purchased from Shanghai Yuanye Bio-Technology Co., Ltd. (Shanghai, China). Primary antibodies were: γ-ENaC (Affinity, DF-8540); NHE3 (Santa Cruz, sc-136368), DRA (sc-376187), AQP3 (sc-518001), AQP8 (sc-81870); ERK1/2 (Proteintech, 11257-1-AP), phospho-ERK1/2 (80031-1-RR), NF-κB p65 (10745-1-AP), phospho-p65 (82335-1-RR), Kv1.3 (14079-1-AP), and GAPDH (10494-1-AP). Secondary antibodies were HRP-conjugated goat anti-rabbit IgG (H+L; Proteintech, SA00001-2) and HRP-conjugated goat anti-mouse IgG (ZSGB-BIO, ZB-2305).

### 2.2 Study drug (commercial Wuji Wan) and preparation for gavage

Wuji Wan (WJW) is a licensed, regulator-approved commercial Chinese polyherbal preparation (CCPP) in a water-pill dosage form manufactured by Li Shizhen Pharmaceutical Group Co., Ltd., compliant with the Chinese Pharmacopoeia (ChP) 2020, Volume I, with NMPA approval no. Z42021264. The product used in this study had batch no. 202401003 (manufacturing date: 2024-01-08; expiry date: 2027-01-07) and was purchased from a licensed retail pharmacy. It was stored at room temperature in a dry environment according to the label instructions. According to ChP 2020 (Vol. I), WJW is composed of three powdered botanical drugs that are mixed at a ratio of 6:1:6 (w/w), made into water pills with water, and dried (i.e., this licensed finished product is not an extract prepared in our laboratory): *C. chinensis* Franch. [Ranunculaceae; Coptidis Rhizoma], *T. ruticarpum* (A.Juss.) T.G.Hartley [Rutaceae; Evodiae Fructus], and *P. lactiflora* Pall. [Paeoniaceae; Paeoniae Radix Alba]. Botanical names were taxonomically verified using Plants of the World Online (POWO; accessed 1 September 2025). As this study used a licensed commercial finished product manufactured under pharmacopeial requirements, detailed proprietary industrial manufacturing parameters are not publicly available. Therefore, a drug-extract ratio is not applicable for the administered preparation. To support reproducibility, we provide batch information and a pharmacopeial-anchored chemical profile (Section 2.3 and Supplementary Material S1). In addition, the major metabolite profile of the same marketed WJW product (same manufacturer) and WJW-containing rat plasma has been characterized previously by our group using UPLC-Q-TOF-MS/MS (Guo et al., 2022).

For animal dosing, WJW pills were weighed, ground into fine powder, passed through a 300-mesh sieve, and suspended in distilled water to obtain a homogeneous gavage preparation. The suspension was mixed thoroughly immediately before administration.

### 2.3 WJW quality control (QC)

WJW quality control was performed by HPLC (Waters 2695-2487, United States) using a Kromasil 100-5 C18 column (4.6 × 250 mm). The mobile phase consisted of A (0.1% phosphoric acid in water) and B (acetonitrile) with gradient elution (0–42 min, A 88%→45%; 42–50 min, A 45%→55%; 50–67 min, A 45%→38%). The flow rate was 1.0 mL min^−1^, the column temperature was 30 °C, and UV detection was monitored at 230 and 330 nm (Geng et al., 2021). For sample preparation, for HPLC analysis, 0.25 g of WJW was ultrasonically extracted with 25 mL of 80% methanol for 40 min, adjusted to the original weight with 80% methanol after cooling, and filtered through a 0.22 μm membrane to obtain the test solution. Paeoniflorin (C_23_H_28_O_11_) and berberine hydrochloride (C_20_H_17_NO_4_·HCl) were quantified using external standards. According to ChP 2020 (Vol. I), berberine hydrochloride and paeoniflorin should be ≥15.0 and ≥7.0 mg/g, respectively; the measured contents in the batch used in this study were 20.29 and 10.76 mg/g (Supplementary Material S1).

In addition to the two pharmacopeial marker metabolites (berberine hydrochloride and paeoniflorin), we quantified three additional abundant metabolites as supplementary batch characterization (Supplementary Material S1). As these metabolites are not specified as mandatory QC markers in ChP 2020 (Vol. I) for WJW, the results are reported descriptively to complement the HPLC fingerprint rather than as pharmacopeial compliance criteria.

### 2.4 Animals and handling

Male C57BL/6J mice [n = 30, 8 weeks old; Beiyou; license SCXK (Beijing) 2024-0016] and male Sprague–Dawley rats [n = 12, 8 weeks old; Vital River; license SCXK (Beijing) 2021-0011] were used. Animals were housed under a 12 h light/dark cycle with *ad libitum* access to food and water and were acclimated for 1 week before experimentation. All procedures complied with national guidelines for the care and use of laboratory animals and were approved by the IACUC of the Institute of Chinese Materia Medica (approval no. 2025B069).

Mice were randomly assigned to five groups using a random number table: control, 3% DSS, 3% DSS + mesalazine (0.25 g/kg/day), 3% DSS + WJW-L (1.56 g/kg/day), and 3% DSS + WJW-H (3.12 g/kg/day). All WJW doses are expressed as g/kg/day of the licensed commercial finished product (water-pill powder), not as crude-drug equivalents and not as laboratory extracts. Dose selection was anchored to the labeled adult regimen (12 g/day for a 70-kg adult) to maintain translational relevance to the marketed product. To additionally provide an API (active pharmaceutical ingredient)–level, quantitative dose description for this CCPP (which does not have a single defined API), we report exposures of representative pharmacopeial marker metabolites (HPLC analytes) based on batch HPLC quantification (Section 2.3). The study batch contained berberine hydrochloride 20.29 mg/g and paeoniflorin 10.76 mg/g; therefore, WJW-L (1.56 g/kg/day) corresponds to 31.65 mg/kg/day berberine hydrochloride and 16.79 mg/kg/day paeoniflorin, and WJW-H (3.12 g/kg/day) corresponds to 63.30 mg/kg/day berberine hydrochloride and 33.57 mg/kg/day paeoniflorin.

Because high nominal mass doses can raise concerns about pharmacological interpretability, we also provide an upper-bound contextual estimate based on a previously measured/established approximate water-extractable fraction (∼20% yield) estimated in our prior extraction for the same marketed product: under this assumption, 3.12 g/kg/day of finished product corresponds to ∼0.62 g/kg/day extract-equivalent (<1 g/kg/day) (Supplementary Material S2). This estimate is provided for context only and does not define the administered preparation as an extract nor constitute a drug–extract ratio. From days 1–7, all groups except control received 3% DSS in drinking water. Mesalazine and WJW were administered twice daily in two divided doses by oral gavage; controls received vehicle. The gavage volume was 0.2 mL/10 g body weight. On day 8, animals were anesthetized with isoflurane and samples were collected. Blood was centrifuged at 3,500 rpm for 15 min at 4 °C to obtain serum, which was stored at −80 °C. Colons were photographed; portions were fixed in 4% paraformaldehyde, and the remainder was stored at −80 °C.

Rats were randomized (random number table) to vehicle plasma or WJW-containing plasma groups. Rats were administered WJW by oral gavage once daily for three consecutive weeks. The rat dose was set at 1.08 g/kg/day (commercial finished product) to generate WJW-containing plasma under a regimen anchored to the labeled adult use; the dose-setting rationale and contextual conversion are provided in Supplementary Material S2. Based on the same batch marker contents (Section 2.3), 1.08 g/kg/day corresponds to 21.91 mg/kg/day berberine hydrochloride and 11.62 mg/kg/day paeoniflorin. After isoflurane anesthesia, blood was collected into EDTA tubes and centrifuged at 3,500 rpm for 15 min at 4 °C to obtain plasma. Plasma was aliquoted and stored at −80 °C for subsequent electrophysiology experiments. For patch-clamp recordings, WJW-containing plasma was added to the extracellular solution to achieve final plasma volume fractions of 5%, 10%, or 20% (v/v).

### 2.5 Assessment of disease activity index

During modeling and treatment, body weight, water intake, stool consistency, and hematochezia were recorded daily. DAI was calculated from weight loss, stool consistency, and hematochezia [DAI = (score weight + score stool + score blood)/3].

### 2.6 Biochemical assays

Fecal Na^+^ and Cl^−^ were measured with commercial kits; total protein in fecal suspensions and colonic homogenates was quantified by BCA assay. Fecal CALP and colonic IL-6/IL-17A/IFN-γ were quantified by ELISA according to the manufacturers’ instructions. Feces and colon were homogenized in PBS at 1:9 (m/v), centrifuged at 5,000 × g for 10 min at 4 °C, and supernatants were collected for assays.

### 2.7 Histopathology

Colons fixed in 4% paraformaldehyde underwent routine H&E staining, including dehydration, paraffin embedding, sectioning, deparaffinization, hematoxylin and eosin staining, and mounting. Three random fields per section were evaluated for inflammatory infiltration and epithelial morphology and were scored according to predefined criteria.

### 2.8 Untargeted serum metabolomics

Serum was thawed at 4 °C and vortexed. Then, 100 µL of serum was mixed with 300 µL pre-chilled methanol (−20 °C), incubated for 1 h, and centrifuged at 2,300 × g for 10 min at 4 °C. The supernatant was injected for LC–MS. Chromatography was performed on a Shimadzu LC-30 UHPLC using an ACQUITY UPLC® HSS T3 column (2.1 × 100 mm, 1.8 µm; Waters). The injection volume was 16 μL; column temperature was 40 °C; flow rate was 0.3 mL/min. Mobile phases were A, 0.1% formic acid in water, and B, 0.1% formic acid in acetonitrile. Gradient: 0–2 min, B 0%; 2–3.3 min, B 0%→48%; 3.3–5.1 min, B 48%→100%; 5.1–7.2 min, B 100%; 7.2–7.3 min, B 100%→0%; 7.3–10 min, B 0%. Each sample was analyzed in electrospray ionization (ESI) positive (+) and negative (−) ion modes. After UPLC separation, an Orbitrap Fusion mass spectrometer (Thermo Scientific) with a HESI source was used: spray voltage 3.8 kV (+)/3.2 kV (−); capillary temperature 320 °C; sheath gas 40; auxiliary gas 15; heater 350 °C; S-Lens RF 50. Quality-control (QC) samples, prepared by pooling aliquots from all study samples, were used for data normalization and system suitability checks. Blanks (75% acetonitrile in water) and QC samples were injected every six samples during acquisition.

Raw data were processed in MS-DIAL for peak alignment, retention time correction, and peak area extraction. Metabolites were annotated by exact-mass matching (with an error tolerance of<10 ppm) and MS/MS spectral matching (with an error tolerance of<0.01 Da) against databases including HMDB, MassBank, GNPS, and an in-house BP-DB. The data obtained in positive and negative ion modes were normalized to total ion current (TIC), merged, and subjected to pattern recognition in Python. Unit variance (UV) scaling was applied before downstream analyses.

R (version 4.0.3) and relevant R packages were used for all multivariate analyses and modeling. PCA and PLS-DA were used to assess group separation in both positive and negative modes. All supervised models were assessed for overfitting by permutation testing (n = 200). Model fit was summarized by R^2^X (cum) and R^2^Y (cum), while predictive performance by Q^2^ (cum) and 200-run permutation tests. For valid permutation results, Y-intercept values of *R* ^2^ and Q^2^ from permuted models should be lower than those of the original (non-permuted) model. Discriminating metabolites were ranked by variable importance in projection (VIP) scores derived from the PLS-DA model. VIP scores quantify each variable’s contribution to between-group discrimination and are computed as weighted sums of squared PLS weights across components. By convention, the mean VIP is 1.0, and variables with VIP > 1.0 are considered influential. Higher VIP scores indicate stronger discriminatory contribution and are commonly used as a feature-selection criterion for candidate biomarkers. Discriminating metabolites were selected using VIP from the PLS-DA model combined with univariate testing (two-tailed Student’s t-test). For multiple-group comparisons, p-values were obtained by one-way ANOVA. Metabolites with VIP > 1.0 and p < 0.05 were considered statistically significant. Fold change (log_2_FC) was calculated as the log_2_ ratio of mean peak areas between two groups. Identified differential metabolites were subjected to hierarchical clustering using R packages.

Differential metabolites were mapped to KEGG pathways (via the KEGG database) to identify perturbed biological processes. KEGG pathway enrichment was evaluated using Fisher’s exact test with Benjamini–Hochberg false-discovery-rate (FDR) correction. Pathways with an FDR-adjusted p-value (q) < 0.05 was considered significantly enriched. Heatmaps and trend plots were generated using the OmicKits web platform.

### 2.9 Western blotting

Colonic protein levels of γ-ENaC, NHE3, DRA, AQP3, AQP8, ERK1/2, phospho-ERK1/2, NF-κB p65, phospho-p65, and Kv1.3 were assessed by Western blotting. Frozen colons (−80 °C) were homogenized in RIPA buffer with protease/phosphatase inhibitors to extract proteins on ice. Lysates were centrifuged at 12,000 rpm for 15 min; protein concentration was measured by BCA assay. Bands were visualized by ECL and quantified in ImageJ.

### 2.10 Cells

Jurkat T cells (E6-1; SUNNCELL) were maintained in RPMI-1640 with 10% (v/v) heat-inactivated FBS, 100 U/mL penicillin, and 100 μg/mL streptomycin at 37 °C in a humidified 5% CO_2_ incubator. Selected Jurkat T cells were in the log-phase, exhibited normal morphology and a phase-bright appearance at 60%–70% confluency. The cells were gently resuspended into a single-cell suspension using a Pasteur pipette. An aliquot of the suspension was then transferred to the recording chamber and allowed to settle and adhere at room temperature for 5–10 min before recording began.

### 2.11 Electrophysiology

Whole-cell patch-clamp recordings were performed on Jurkat T cells. The extracellular solution contained (mM): 136.5 NaCl, 5.4 KCl, 1.8 CaCl_2_, 0.53 MgCl_2_, 5.5 glucose, and 5.5 HEPES; pH was adjusted to 7.4 with NaOH. Patch-clamp electrodes, made from borosilicate glass capillaries (Biospikes, China), had tip resistance of 4–8MΩ.The pipette solution (for K^+^ currents) contained (mM): 130 K-aspartate, 20 KCl, 1 KH_2_PO_4_, 1 MgCl_2_, 3 Na_2_ATP, 0.1 Na_2_GTP, 0.1 EGTA, and 5 HEPES; pH was adjusted to 7.2 with KOH. To minimize Cl^−^ currents, Cl^−^ was replaced with aspartate in the internal solution, which was filtered through a 0.22-µm membrane. Kv1.3 currents were evoked by 250-m depolarizing steps from a holding potential of −80 mV to test potentials between −80 and +60 mV, delivered every 10 s. Currents were amplified and filtered with an Axopatch 700B and digitized at 3 kHz using a Digidata 1440A (Molecular Devices, Union City, CA, United States). Peak currents were normalized to cell capacitance and expressed as current density (pA/pF). Recordings were performed at room temperature. Data were acquired and analyzed with pCLAMP 10.2 and Origin 2021.

### 2.12 Statistical analysis

Data are presented as mean ± SD. Group differences were assessed by one-way ANOVA; when assumptions were violated, the Kruskal-Wallis test was used. Post hoc comparisons used Tukey’s test. Analyses were performed in GraphPad Prism 10.1.2; two-sided p < 0.05 was considered statistically significant.

## 3 Results

### 3.1 WJW improved DSS-induced UC phenotypes in mice

**FIGURE 1:**
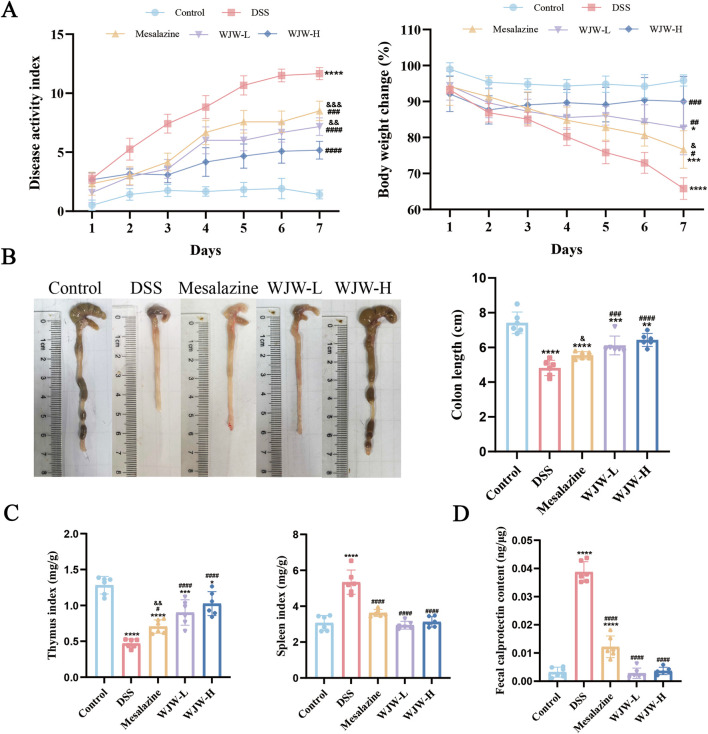
Wuji Wan (WJW) ameliorates phenotypes in mice with ulcerative colitis (UC). **(A)** Change in Disease activity index (DAI) and body-weight. **(B)** Representative gross images of colons and quantification of colon length. **(C)** Spleen and thymus indices. **(D)** Fecal calprotectin (CALP) concentration. Data are mean ± SD (n = 6 per group). *p < 0.05; **p < 0.01; ***p < 0.001; ****p < 0.0001 vs. Control; ^#^p < 0.05; ^##^p < 0.01; ^###^p < 0.001; ^####^p < 0.0001 vs. DSS; ^&^p < 0.05; ^&&^p < 0.01; ^&&&^p < 0.001 vs. WJW-H.

**FIGURE 2:**
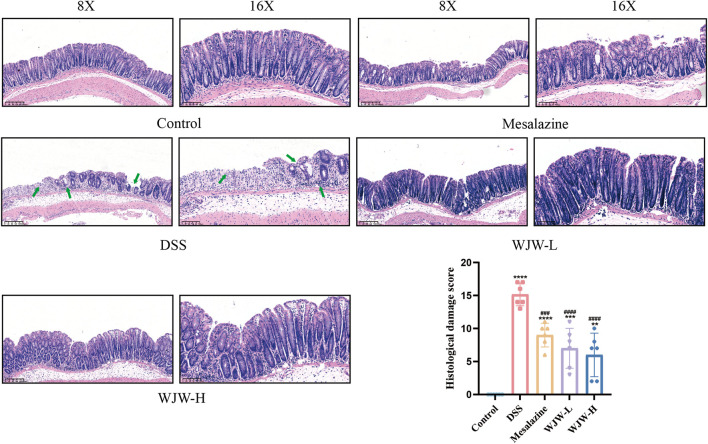
Effect of WJW on colon tissue injury demonstrated by hematoxylin and eosin (H&E) stained sections. (original magnification ×8 and ×16; scale bar, 200 and 100 μm). The epithelial abnormalities are indicated by the green arrows. Data are mean ± SD (n = 6 per group). **p < 0.01; ***p < 0.001; ****p < 0.0001 vs. Control; ^###^p < 0.001; ^####^p < 0.0001 vs. DSS.

To evaluate therapeutic efficacy, we employed a 3% DSS model and assessed multiple parameters: body weight, disease activity index (DAI), colon length, spleen and thymus indices, fecal calprotectin (CALP) levels, and histopathology based on H&E staining. Compared to control mice, DSS-treated mice exhibited significant weight loss, severe diarrhea accompanied by hematochezia, and marked colon shortening (Figures 1A,B). Treatment with either mesalazine or WJW significantly ameliorated these disease phenotypes. Specifically, both treatments reduced the DSS-elevated DAI scores and spleen index, increased the thymus index, and lowered fecal CALP levels (Figures 1C,D). Histological analysis revealed epithelial damage, goblet cell depletion, crypt destruction, and extensive inflammatory infiltration in the DSS group. In contrast, the WJW group exhibited more intact crypt structures, reduced mucosal injury, and a morphology more closely resembling that of the control group (Figure 2). Notably, WJW treatment was more effective than mesalazine in reducing the DAI, restoring colon length, lowering CALP levels, and improving histological scores.

### 3.2 WJW partially reversed serum metabolic disruption in UC mice

**FIGURE 3:**
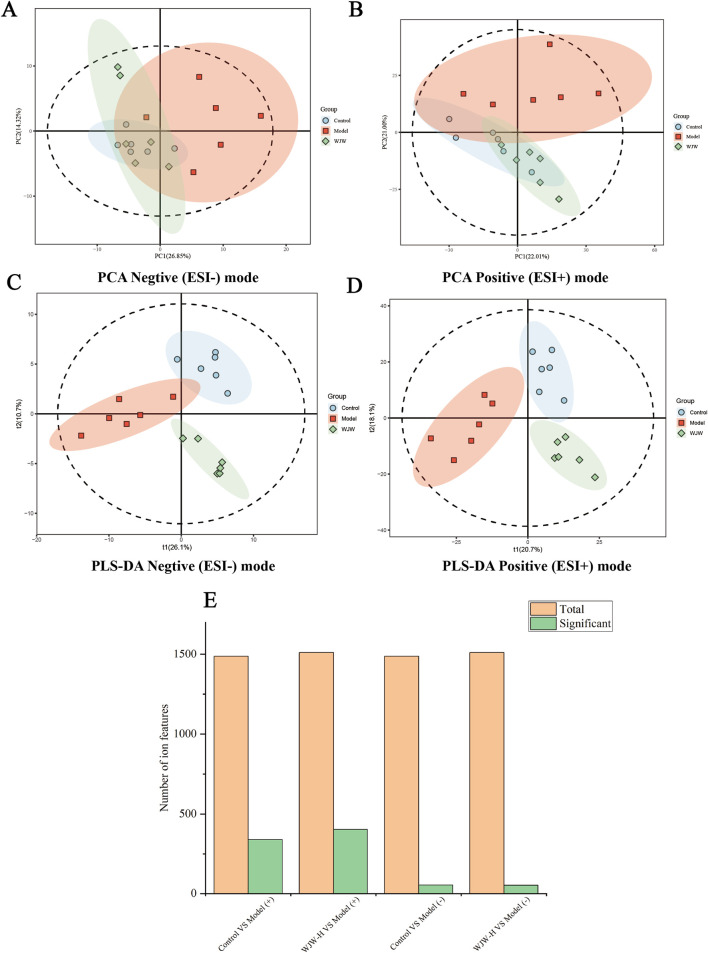
Serum metabolomic profiling. Principal component analysis (PCA) score plots for three groups in **(A)** electrospray-ionization positive (ESI+) and **(B)** negative (ESI−) modes. Partial least squares–discriminant analysis (PLS-DA) score plots for three groups in **(C)** ESI+ and **(D)** ESI− modes. **(E)** Bar Graph of identified and differentially abundant metabolites shared and unique across groups. (n = 6 per group).

To investigate the underlying mechanisms, we conducted untargeted metabolomic profiling on serum samples from the control, DSS, and high-dose WJW (WJW-H) groups. Both principal component analysis (PCA) and partial least-squares-discriminant analysis (PLS-DA) revealed clear separation between the experimental groups. In negative-ion mode, the metabolic profile of the WJW-H group nearly overlapped with that of the control group (Figures 3A,C). A high degree of similarity was also observed in positive-ion mode (Figures 3B,D). These findings indicate that DSS-induced colitis disrupts the serum metabolome and that WJW treatment partially reverses these alterations. As shown in Figure 3E, in positive-ion mode, 339 and 403 metabolites differed significantly between the DSS group and the control or WJW-H groups, respectively. In negative-ion mode, 53 and 52 differential metabolites were identified for these comparisons, respectively. Detailed information on differential metabolites was available in Supplementary Date S1.

**FIGURE 4:**
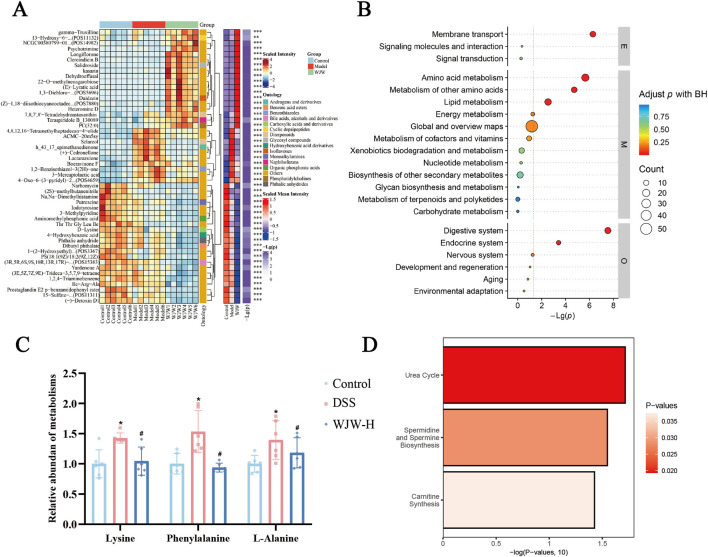
Clustering and pathway enrichment of differentially abundant metabolites. **(A)** Hierarchically clustered heatmap of relative abundances of differentially abundant metabolites in the top 50 across groups; red denotes higher and blue lower abundance. **(B)** Pathway enrichment analysis of altered metabolites using the Kyoto Encyclopedia of Genes and Genomes (KEGG). **(C)** Levels of key differentially abundant serum metabolites across the three groups. **(D)** Pathway enrichment analysis based on the Small Molecule Pathway Database (SMPDB). Data are mean ± SD (n = 6 per group). *p < 0.05 vs. Control; ^#^p < 0.05 vs. DSS.

The top 50 significantly differential metabolites were identified as potential biomarkers distinguishing the Control, DSS and WJW groups and are displayed in the heatmap in Figure 4A. Most of the significantly altered metabolites could be categorized into lipids and lipid-like molecules, plant and microbial-derived secondary metabolites, organic acids and derivatives, among others. The Kyoto Encyclopedia of Genes and Genomes (KEGG) pathway enrichment analysis highlighted several altered pathways, including membrane transport, amino acid metabolism, lipid metabolism, and other amino-acid-related pathways (Figure 4B). Given the close interplay between membrane transport and amino acid metabolism—both implicated in UC pathogenesis—we identified metabolites enriched in these two KEGG categories that were also associated with “digestive system” diseases. This intersection yielded three key metabolites: lysine, phenylalanine, and L-alanine (Figure 4C). The levels of all three metabolites were elevated in the DSS group and shifted towards control levels following WJW-H treatment. The Small Molecule Pathway Database (SMPDB) enrichment also identified the urea cycle as significantly enriched (Figure 4D). Collectively, these data suggest that a reprogramming of the serum metabolome and associated pathways—particularly those involved in membrane transport and amino acid metabolism—contributes to the amelioration of DSS-induced phenotypes and may underlie the therapeutic mechanism of WJW. Given the significant enrichment of the membrane transport pathway in the metabolomic analysis, we hypothesized that WJW might directly regulate the expression of key colonic membrane transport proteins, thereby alleviating diarrhea.

### 3.3 WJW modulated epithelial membrane proteins to alleviate diarrhea

**FIGURE 5:**
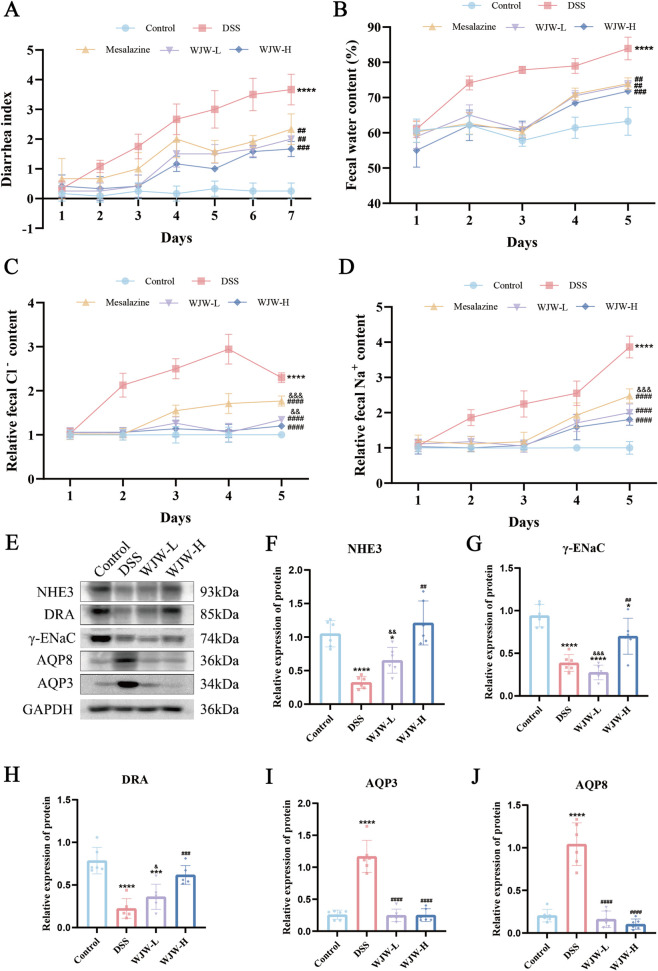
WJW alleviates diarrhea and regulates epithelial transport proteins in UC mice. **(A)** Diarrhea scores over time across groups. **(B)** Fecal water content on days 1–5. **(C)** Fecal Cl^−^ concentration on days 1–5. **(D)** Fecal Na^+^ concentration on days 1–5. **(E)** Representative Western blots. **(F)** Sodium/hydrogen exchanger 3 (NHE3). **(G)** Epithelial sodium channel γ-subunit (γ-ENaC). **(H)** Downregulated in adenoma (DRA). **(I)** Aquaporin-3 (AQP3). **(J)** Aquaporin-8 (AQP8). Data are mean ± SD (n = 6 per group). *p < 0.05; ***p < 0.001; ****p < 0.0001 vs. Control; ^##^p < 0.01; ^###^p < 0.001; ^####^p < 0.0001 vs. DSS; ^&^p < 0.05; ^&&^p < 0.01; ^&&&^p < 0.001 vs. WJW-H.

To quantify diarrhea, we first scored stool consistency throughout the modeling period. The diarrhea index showed a continual rise in the DSS group. In contrast, the treated groups reached a peak on day 4 and subsequently declined. By the end of the modeling period, all treatment groups differed significantly from the DSS group (Figure 5A). Stool water content was significantly higher in DSS mice from day 2 onwards compared to controls during the first 5 days. WJW treatment effectively reduced this elevation (Figure 5B). Biochemical assays revealed that fecal Na^+^ and Cl^−^ levels increased over time in all groups. However, WJW treatment significantly lowered the levels of both ions compared to the DSS group and, overall, was more effective than mesalazine (Figures 5C,D). To identify the molecular correlates of these physiological changes, we measured the protein expression of key transporters—including NHE3, γ-ENaC, DRA, AQP3 and AQP8—by Western blot analysis (Figure 5E). NHE3 and γ-ENaC expression was downregulated in DSS mice, consistent with impaired Na^+^ absorption. WJW treatment upregulated the expression of both transporters in a dose-dependent manner (Figures 5F,G). DRA expression was reduced in DSS mice and returned toward control with WJW (Figure 5H). In contrast, AQP3 and AQP8 expression was upregulated in the DSS group and was suppressed by WJW treatment in a dose-dependent manner (Figures 5I,J). Collectively, these results demonstrate that WJW reshapes the expression of key colonic transport proteins, thereby restoring water-electrolyte homeostasis and ameliorating DSS-induced diarrhea.

### 3.4 WJW downregulated Kv1.3 and mitigated inflammatory responses

**FIGURE 6:**
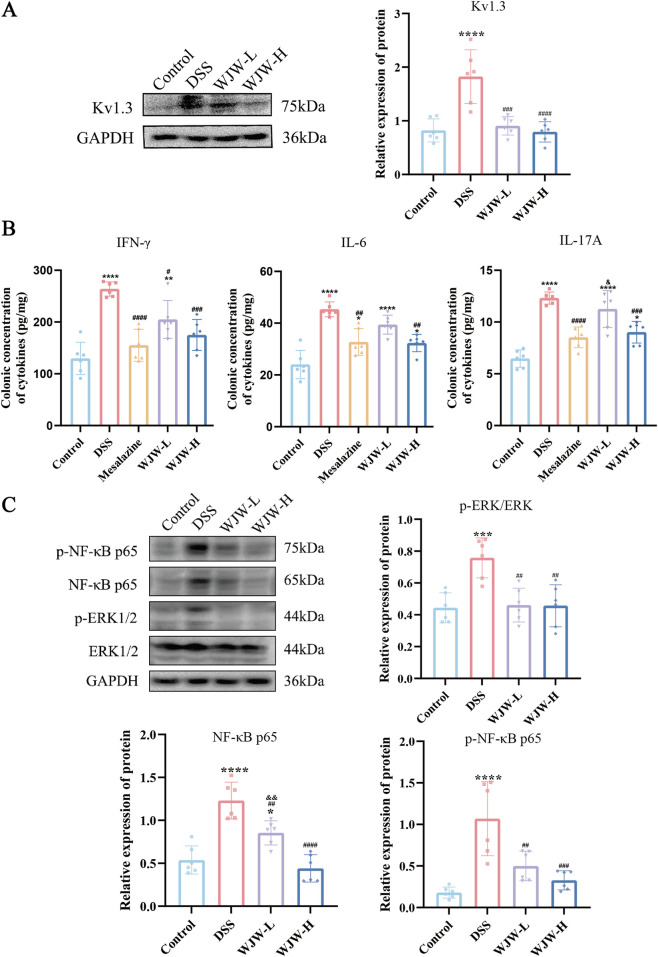
WJW attenuates colonic inflammation by downregulating Kv1.3 and modulating NF-κB/ERK signaling. **(A)** Representative Western blot results and densitometric quantification of Kv1.3 protein expression levels in colonic tissue. **(B)** Cytokine concentrations in colonic homogenates measured by ELISA: interferon-γ (IFN-γ), interleukin-6 (IL-6), and interleukin-17A (IL-17A). **(C)** Representative Western blot results and densitometric quantification of p-ERK/ERK, NF-κB p65 and p-NF-κB p65 protein expression levels in colonic tissue. Data are mean ± SD (n = 6 per group). *p < 0.05; **p < 0.01; ***p < 0.001; ****p < 0.0001 vs. Control; ^#^p < 0.05; ^##^p < 0.01; ^###^p < 0.001; ^####^p < 0.0001 vs. DSS; ^&^p < 0.05; ^&&^p < 0.01; ^&&&^p < 0.001 vs. WJW-H.

Furthermore, since potassium channels (e.g., Kv1.3) are critical membrane proteins involved in both ion transport and T cells activation, we also investigated the anti-inflammatory effects of WJW. Western blot analysis showed that colonic Kv1.3 protein levels were elevated in DSS mice and were significantly reduced by WJW treatment (Figure 6A), suggesting a dampening of the aberrant immune response. ELISA results demonstrated increased levels of the pro-inflammatory cytokines interferon-γ (IFN-γ), interleukin-6 (IL-6), and interleukin-17A (IL-17A) in DSS colon tissue, all of which were suppressed by WJW treatment (Figure 6B). The DSS group exhibited elevated protein levels of NF-κB-p65, phosphorylated p65 (p-p65), and phosphorylated ERK1/2 (p-ERK1/2). WJW treatment reduced the levels of these signaling molecules, with the reductions in p65 and p-p65 showing dose dependence (Figure 6C). Thus, WJW modulates NF-κB/ERK pathway activity and alleviates UC-related inflammation.

### 3.5 Drug-containing plasma directly inhibited Kv1.3 currents in Jurkat T cells

**FIGURE 7:**
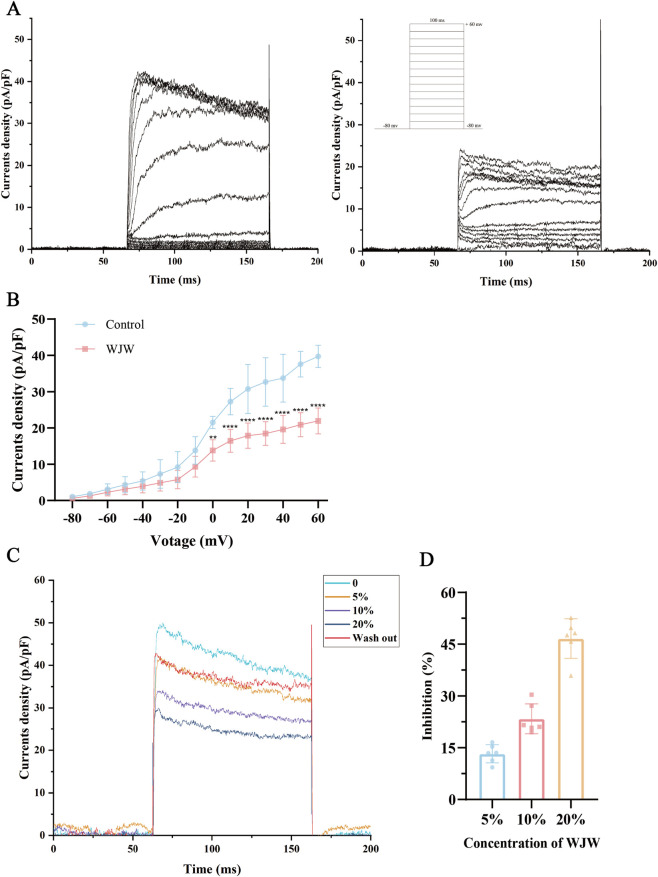
WJW–medicated plasma inhibits Kv1.3 currents in Jurkat T cells. **(A)** Representative Kv1.3 current traces recorded before and after application of 10% WJW-medicated plasma from treated rats; the voltage protocol is shown above. **(B)** Mean current density–voltage (I–V) relationship after application of 10% WJW-medicated plasma. Currents were evoked by 250-m depolarizing steps from a holding potential of −80 mV to test potentials between −80 and +60 mV, delivered every 10 s. **(C)** Superimposed traces recorded before application, during perfusion, and after washout of WJW-medicated plasma. **(D)** Inhibition of Kv1.3 currents at different v/v concentrations of WJW-medicated plasma. Data are mean ± SD (n = 6 per condition). **p < 0.01; ****p < 0.0001 vs. Control.

To test for direct effects on Kv1.3 channel function, we recorded whole-cell patch-clamp currents in Jurkat T cells during exposure to 10% (v/v) plasma from WJW-treated rats. Under control conditions, voltage steps from −80 to +60 mV (in 10 mV increments) evoked rapidly activating currents that inactivated slowly during the 100-m pulse. Representative current traces are shown in Figure 7A. Following application of 10% drug-containing plasma, Kv1.3 currents were markedly suppressed, evident even at a test potential of 0 mV (Figure 7B). Continuous perfusion with 5%, 10%, and 20% drug-containing plasma produced a progressive, time-dependent inhibition of Kv1.3 currents. Representative current traces at each concentration are shown in Figure 7C. The dose-response relationship, summarized from n = 6 cells, is shown in Figure 7D. The mean percent inhibition of Kv1.3 currents at 5%, 10%, and 20% drug-containing plasma was 13.24%, 23.40%, and 45.73%, respectively. These findings indicate that the anti-inflammatory and immunomodulatory actions of WJW are attributable, at least in part, to a direct inhibition of Kv1.3 channel function.

### 3.6 Correlations among serum metabolites, protein expression, and biochemical indices

**FIGURE 8:**
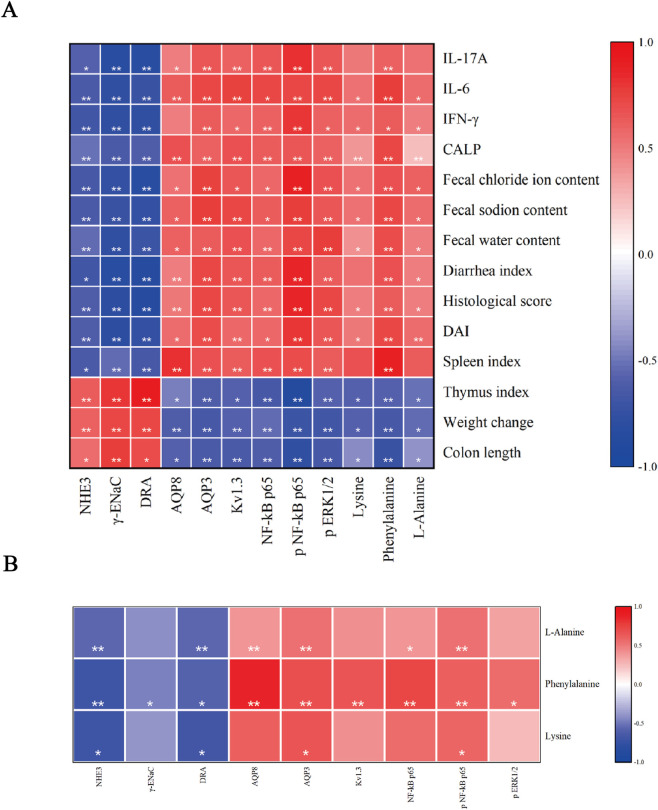
Spearman correlations among serum metabolites, protein expression, and biochemical indices. **(A)** Heatmap of Spearman’s rank correlation coefficients (ρ) between biochemical indices and serum metabolite abundances and protein expression levels. **(B)** Heatmap of Spear-man’s ρ between serum metabolites and protein expression levels. Significance: *p < 0.05; **p < 0.01 (two-tailed). Color scale: red, positive; blue, negative.

We performed Spearman rank correlation analysis among the differential serum metabolites, key membrane and inflammatory proteins, and macroscopic phenotypic measures. The resulting correlation matrix is visualized in the heatmap shown in Figure 8A. The expression levels of NHE3 and DRA showed significant positive correlations with colon length, weight gain, and thymus index, and significant negative correlations with DAI score, stool water content, fecal electrolyte levels, and pro-inflammatory cytokine levels. The three key serum metabolites—lysine, phenylalanine, and L-alanine—exhibited significant negative correlations with the expression of NHE3 and DRA, and significant positive correlations with AQP3 expression and the level of NF-κB phosphorylation (Figure 8B). Phenylalanine was uniquely correlated with ERK activation among the differential metabolites, showing a significant positive correlation with p-ERK levels, suggesting its potential as a key node linking metabolism to pro-inflammatory signaling. Taken together, these correlation patterns suggest that these metabolites may exacerbate colitis by promoting pro-inflammatory cytokine production and activating NF-κB/ERK signaling, ultimately leading to an imbalance in epithelial transport function. The identification of such metabolites provides valuable insights into disease progression and highlights potential therapeutic targets. Overall, the integration of phenotypic, molecular expression, and metabolomic data reveals consistent pathophysiological relationships and provides mechanistic insights into how WJW ameliorates UC.

## 4 Discussion

This study tested whether WJW alleviates DSS-induced UC through coordinated regulation of “membrane transport” across epithelial and immune compartments, thereby improving inflammatory diarrhea while tempering mucosal immune activation. Using a multi-level workflow—phenotypic efficacy, untargeted serum metabolomics, transporter/water-channel profiling, and T-cell electrophysiology—our results converge on a membrane-transport–centered interpretation: WJW treatment is accompanied by restoration of an epithelial absorptive program and suppression of T-cell excitability, with systemic amino-acid signatures nominating candidate nodes that may couple whole-body metabolic state to mucosal signaling.

Consistent with its traditional use, WJW markedly improved core disease manifestations in the DSS model, including overall clinical activity, colon shortening, and histological injury. These efficacy readouts establish that the formula exerts a measurable therapeutic benefit *in vivo* and motivate mechanistic interrogation of how symptom control—especially diarrhea—may be achieved beyond generalized anti-inflammatory effects. Notably, diarrhea can persist despite partial improvement of inflammatory indices (Colombel et al., 2017; Ma et al., 2020), supporting epithelial transport restoration as a mechanistically relevant therapeutic dimension rather than a redundant correlate of inflammation control (Magalhães et al., 2016; Turner et al., 2021).

At the systemic level, untargeted serum metabolomics showed that WJW shifted the global metabolic profile of DSS mice toward that of healthy controls. Pathway enrichment highlighted amino-acid metabolism and membrane transport as prominent categories altered by disease and partially normalized by treatment, providing an unbiased clue aligned with our working hypothesis. Among the differential metabolites, lysine, phenylalanine, and L-alanine emerged as representative nodes. Importantly, we treat these signals as hypothesis-generating rather than causal: serum amino acids integrate inputs from diet, host metabolism, microbiota, and inflammation (Kelly and Pearce, 2020; Li et al., 2024; Nicholson et al., 2012), and their associations with mucosal endpoints do not by themselves specify directionality (Johnson et al., 2016; Long et al., 2020). Mechanistically, amino-acid signatures are intrinsically linked to membrane transport because cellular availability depends on dedicated transporters and because inflammatory remodeling of epithelia often co-regulates metabolic and transport programs (Lin et al., 2015; Schwärzler et al., 2024). In immune cells, amino-acid supply is a recognized constraint on activation and effector polarization (Kelly and Pearce, 2020), providing a plausible route by which systemic amino-acid shifts could track with mucosal inflammatory signaling (Sinclair et al., 2013). In this context, phenylalanine is notable because it showed the strongest association with colonic ERK phosphorylation among the altered amino acids, prioritizing phenylalanine-related pathways as candidates for follow-up tests of metabolic–inflammatory coupling.

Guided by these metabolomic cues and by established diarrhea biology in UC, we focused on epithelial transporters that constitute the core machinery for NaCl absorption and water handling (Anbazhagan et al., 2018; Sullivan et al., 2009). We found that WJW upregulated NHE3, γ-ENaC, and DRA while normalizing dysregulated AQP3 and AQP8 expression in colonic tissue (Hardin et al., 2004; Ricanek et al., 2015; Zahn et al., 2007). The selection of these targets is mechanistically grounded: NHE3 and DRA cooperate to drive electroneutral NaCl absorption (Walker et al., 2008), and ENaC supports electrogenic Na^+^ uptake (Greig et al., 2004); inflammatory cytokine milieus characteristic of colitis are known to disrupt these pathways, contributing directly to impaired salt absorption and diarrheal fluid loss (Amasheh et al., 2004; Saksena et al., 2010). Aquaporins, in turn, often display disease-associated dysregulation that may reflect disturbed epithelial differentiation and water handling (Ricanek et al., 2015; Zahn et al., 2007). Thus, the pattern we observe—restored transporter abundance alongside normalization of water-channel expression—supports the view that WJW is accompanied by re-establishment of epithelial absorptive capacity, a proximal determinant of diarrhea severity.

In parallel, WJW appeared to act on T-cell membrane physiology. We observed reduced Kv1.3 protein abundance in colonic tissue and, more directly, showed that plasma from WJW-treated rats inhibited Kv1.3 currents in Jurkat T cells measured by whole-cell patch clamp. Kv1.3 is a key determinant of T-cell membrane potential and Ca^2+^ signaling during activation, making its functional modulation directly relevant to T-cell–driven mucosal inflammation in colitis (Koch Hansen et al., 2014; Tajti et al., 2020; Unterweger et al., 2021). Jurkat T cells were used as a standardized, reproducible T-cell platform in which ion-channel behavior can be interrogated with minimal confounding from the *in vivo* microenvironment; while they do not fully recapitulate primary mucosal T cells, they enable a clean test of whether WJW-related circulating factor(s) can directly modulate a T-cell Kv1.3 current (Abraham and Weiss, 2004; Matsushita et al., 2009). Patch clamp provides the most direct quantitative assessment of ion-channel function and therefore strengthens inference beyond correlative expression changes (Hamill et al., 1981). The plasma-based inhibition further implies that circulating, soluble factor(s) generated or modulated by WJW exposure can acutely restrain Kv1.3 activity, offering a mechanistic entry point for how WJW could reduce T-cell excitability in a manner consistent with known Kv1.3–Ca^2+^ coupling during T-cell activation (Nicolaou et al., 2009; Tajti et al., 2020).

Integrating across scales, our data support a model in which WJW may mitigate a feed-forward loop linking epithelial dysfunction and immune activation. On one arm, restoration of epithelial ion and water transport would reduce luminal osmotic drivers of diarrhea and improve barrier conditions; on the other, suppression of T-cell Kv1.3 activity would be expected to dampen pathological T-cell activation and cytokine pressure on the epithelium. The observed attenuation of ERK and NF-κB signaling is consistent with an overall reduction in inflammatory tone. “Membrane transport” therefore serves as a unifying conceptual framework that connects epithelial symptom biology (salt/water handling) with immune-cell excitability, providing a coherent and testable explanation for how a multi-component formula may generate coordinated benefits in colitis.

Several limitations temper mechanistic inference and define next steps. First, our epithelial conclusions rely primarily on protein abundance; direct functional assays of net ion transport (e.g., Ussing chamber measurements of Na^+^/Cl^−^ flux) are needed to confirm restored absorptive function. Second, although Kv1.3 current inhibition provides strong functional evidence, the necessity and sufficiency of this pathway for WJW’s immunomodulatory effects should be tested using selective Kv1.3 blockade or genetic loss-of-function, ideally alongside complementary Ca^2+^ and cytokine readouts. Third, the metabolomic findings remain associative; targeted metabolomics combined with perturbation experiments (e.g., phenylalanine manipulation with ERK and transporter readouts) will be required to establish whether specific amino-acid signals causally shape mucosal inflammation or transport programs. Fourth, DSS-induced acute colitis cannot fully model chronic, relapsing UC; organoid–immune co-culture systems and conditional knockout approaches could help disentangle direct epithelial versus T-cell actions and strengthen causal attribution *in vivo*.

In conclusion, this multi-faceted study supports a membrane-transport–centered mechanism whereby WJW ameliorates DSS-induced colitis, accompanied by systemic metabolic normalization, restoration of epithelial ion/water transport protein programs, and functional inhibition of the T-cell Kv1.3 channel. Beyond substantiating a traditional prescription in a modern experimental framework, these findings illustrate how focusing on “membrane transport” can provide an integrative, testable paradigm for dissecting the coordinated epithelial–immune pharmacology of complex botanical drugs.

## Data Availability

The raw data supporting the conclusions of this article will be made available by the authors, without undue reservation.

## References

[B1] Abraham R. T. Weiss A. (2004). Jurkat T cells and development of the T-cell receptor signalling paradigm. Nat. Rev. Immunol. 4 (4), 301–308. 10.1038/nri1330 15057788

[B2] Alzamora R. O'Mahony F. Ko W. H. Yip T. W. Carter D. Irnaten M. (2011). Berberine reduces cAMP-Induced chloride secretion in T84 human colonic carcinoma cells through inhibition of basolateral KCNQ1 channels. Front. Physiol. 2, 33. 10.3389/fphys.2011.00033 21747769 PMC3129074

[B3] Amasheh S. Barmeyer C. Koch C. S. Tavalali S. Mankertz J. Epple H. J. (2004). Cytokine-dependent transcriptional down-regulation of epithelial sodium channel in ulcerative colitis. Gastroenterology 126 (7), 1711–1720. 10.1053/j.gastro.2004.03.010 15188166

[B4] Anbazhagan A. N. Priyamvada S. Alrefai W. A. Dudeja P. K. (2018). Pathophysiology of IBD associated diarrhea. Tissue Barriers 6 (2), e1463897. 10.1080/21688370.2018.1463897 29737913 PMC6179131

[B5] Colombel J. F. Keir M. E. Scherl A. Zhao R. de Hertogh G. Faubion W. A. (2017). Discrepancies between patient-reported outcomes, and endoscopic and histological appearance in UC. Gut 66 (12), 2063–2068. 10.1136/gutjnl-2016-312307 27590995 PMC5749342

[B6] Fairbrass K. M. Costantino S. J. Gracie D. J. Ford A. C. (2020). Prevalence of irritable bowel syndrome-type symptoms in patients with inflammatory bowel disease in remission: a systematic review and meta-analysis. Lancet Gastroenterol. Hepatol. 5 (12), 1053–1062. 10.1016/s2468-1253(20)30300-9 33010814

[B7] Fei F. Aa L. X. Qi Q. Sun R. B. Yan C. X. Aa J. Y. (2019). Paeoniflorin inhibits Th1 and Th17 cells in gut-associated lymphoid tissues to produce anti-arthritis activities. Inflammopharmacology 27 (6), 1193–1203. 10.1007/s10787-019-00615-3 31309485

[B8] Feske S. Wulff H. Skolnik E. Y. (2015). Ion channels in innate and adaptive immunity. Annu. Rev. Immunol. 33, 291–353. 10.1146/annurev-immunol-032414-112212 25861976 PMC4822408

[B9] Geng Y. Chen X. Gong Y. Feng J. (2021). Simultaneous determination of four components in Wuji pills by DAD-HPLC. Drug Stand. China 22 (02), 124–128. 10.19778/j.chp.2021.02.005

[B10] Ghishan F. K. Kiela P. R. (2014). Epithelial transport in inflammatory bowel diseases. Inflamm. Bowel Dis. 20 (6), 1099–1109. 10.1097/mib.0000000000000029 24691115 PMC4103619

[B11] Greig E. R. Boot-Handford R. P. Mani V. Sandle G. I. (2004). Decreased expression of apical Na+ channels and basolateral Na+, K+-ATPase in ulcerative colitis. J. Pathol. 204 (1), 84–92. 10.1002/path.1613 15307141

[B12] Guo Y.-X. Zhang S.-H. Wang A.-Q. Zhu X.-X. Li Y.-J. Chen Y. (2022). Pharmacodynamic substances and therapeutic potential of Wuji pills: based on UPLC-Q-TOF-MS/MS and network pharmacology. Zhongguo Zhong Yao Za Zhi= Zhongguo Zhongyao Zazhi= China J. Chin. Materia Medica 47 (24), 6720–6729. 10.19540/j.cnki.cjcmm.20220727.702 36604922

[B13] Hamill O. P. Marty A. Neher E. Sakmann B. Sigworth F. J. (1981). Improved patch-clamp techniques for high-resolution current recording from cells and cell-free membrane patches. Pflugers Arch. 391 (2), 85–100. 10.1007/bf00656997 6270629

[B14] Hardin J. A. Wallace L. E. Wong J. F. O'Loughlin E. V. Urbanski S. J. Gall D. G. (2004). Aquaporin expression is downregulated in a murine model of colitis and in patients with ulcerative colitis, Crohn's disease and infectious colitis. Cell Tissue Res. 318 (2), 313–323. 10.1007/s00441-004-0932-4 15338270

[B15] Jing W. Dong S. Xu Y. Liu J. Ren J. Liu X. (2025). Gut microbiota-derived tryptophan metabolites regulated by Wuji Wan to attenuate colitis through AhR signaling activation. Acta Pharm. Sin. B 15 (1), 205–223. 10.1016/j.apsb.2024.11.009 40041900 PMC11873645

[B16] Johnson C. H. Ivanisevic J. Siuzdak G. (2016). Metabolomics: beyond biomarkers and towards mechanisms. Nat. Rev. Mol. Cell Biol. 17 (7), 451–459. 10.1038/nrm.2016.25 26979502 PMC5729912

[B17] Kelly B. Pearce E. L. (2020). Amino assets: how amino acids support immunity. Cell Metab. 32 (2), 154–175. 10.1016/j.cmet.2020.06.010 32649859

[B18] Knowles S. R. Keefer L. Wilding H. Hewitt C. Graff L. A. Mikocka-Walus A. (2018). Quality of life in inflammatory bowel disease: a systematic review and meta-analyses-part II. Inflamm. Bowel Dis. 24 (5), 966–976. 10.1093/ibd/izy015 29688466

[B19] Koch Hansen L. Sevelsted-Møller L. Rabjerg M. Larsen D. Hansen T. P. Klinge L. (2014). Expression of T-cell KV1.3 potassium channel correlates with pro-inflammatory cytokines and disease activity in ulcerative colitis. J. Crohns Colitis 8 (11), 1378–1391. 10.1016/j.crohns.2014.04.003 24793818 PMC4216648

[B20] Li T. T. Chen X. Huo D. Arifuzzaman M. Qiao S. Jin W. B. (2024). Microbiota metabolism of intestinal amino acids impacts host nutrient homeostasis and physiology. Cell Host Microbe 32 (5), 661–675.e610. 10.1016/j.chom.2024.04.004 38657606 PMC11636940

[B21] Lin L. Yee S. W. Kim R. B. Giacomini K. M. (2015). SLC transporters as therapeutic targets: emerging opportunities. Nat. Rev. Drug Discov. 14 (8), 543–560. 10.1038/nrd4626 26111766 PMC4698371

[B22] Long N. P. Nghi T. D. Kang Y. P. Anh N. H. Kim H. M. Park S. K. (2020). Toward a standardized strategy of clinical metabolomics for the advancement of precision medicine. Metabolites 10 (2). 10.3390/metabo10020051 32013105 PMC7074059

[B23] Ma C. Sandborn W. J. D'Haens G. R. Zou G. Stitt L. W. Singh S. (2020). Discordance between patient-reported outcomes and mucosal inflammation in patients with mild to moderate ulcerative colitis. Clin. Gastroenterol. Hepatol. 18 (8), 1760–1768.e1761. 10.1016/j.cgh.2019.09.021 31546056 PMC7992966

[B24] Magalhães D. Cabral J. M. Soares-da-Silva P. Magro F. (2016). Role of epithelial ion transports in inflammatory bowel disease. Am. J. Physiol. Gastrointest. Liver Physiol. 310 (7), G460–G476. 10.1152/ajpgi.00369.2015 26744474

[B25] Matsushita Y. Ohya S. Suzuki Y. Itoda H. Kimura T. Yamamura H. (2009). Inhibition of Kv1.3 potassium current by phosphoinositides and stromal-derived factor-1alpha in Jurkat T cells. Am. J. Physiol. Cell Physiol. 296 (5), C1079–C1085. 10.1152/ajpcell.00668.2008 19295169

[B26] Musch M. W. Clarke L. L. Mamah D. Gawenis L. R. Zhang Z. Ellsworth W. (2002). T cell activation causes diarrhea by increasing intestinal permeability and inhibiting epithelial Na+/K+-ATPase. J. Clin. Invest 110 (11), 1739–1747. 10.1172/jci15695 12464679 PMC151630

[B27] Neurath M. F. (2019). Targeting immune cell circuits and trafficking in inflammatory bowel disease. Nat. Immunol. 20 (8), 970–979. 10.1038/s41590-019-0415-0 31235952

[B28] Nicholson J. K. Holmes E. Kinross J. Burcelin R. Gibson G. Jia W. (2012). Host-gut microbiota metabolic interactions. Science 336 (6086), 1262–1267. 10.1126/science.1223813 22674330

[B29] Nicolaou S. A. Neumeier L. Steckly A. Kucher V. Takimoto K. Conforti L. (2009). Localization of Kv1.3 channels in the immunological synapse modulates the calcium response to antigen stimulation in T lymphocytes. J. Immunol. 183 (10), 6296–6302. 10.4049/jimmunol.0900613 19841189 PMC2783516

[B30] Planell N. Lozano J. J. Mora-Buch R. Masamunt M. C. Jimeno M. Ordás I. (2013). Transcriptional analysis of the intestinal mucosa of patients with ulcerative colitis in remission reveals lasting epithelial cell alterations. Gut 62 (7), 967–976. 10.1136/gutjnl-2012-303333 23135761

[B31] Priyamvada S. Gomes R. Gill R. K. Saksena S. Alrefai W. A. Dudeja P. K. (2015). Mechanisms underlying dysregulation of electrolyte absorption in inflammatory bowel disease-associated diarrhea. Inflamm. Bowel Dis. 21 (12), 2926–2935. 10.1097/mib.0000000000000504 26595422 PMC4662046

[B32] Ricanek P. Lunde L. K. Frye S. A. Støen M. Nygård S. Morth J. P. (2015). Reduced expression of aquaporins in human intestinal mucosa in early stage inflammatory bowel disease. Clin. Exp. Gastroenterol. 8, 49–67. 10.2147/ceg.S70119 25624769 PMC4296881

[B33] Rubin D. T. Ananthakrishnan A. N. Siegel C. A. Barnes E. L. Long M. D. (2025). ACG clinical guideline update: ulcerative colitis in adults. Am. J. Gastroenterol. 120 (6), 1187–1224. 10.14309/ajg.0000000000003463 40701556

[B34] Saksena S. Singla A. Goyal S. Katyal S. Bansal N. Gill R. K. (2010). Mechanisms of transcriptional modulation of the human anion exchanger SLC26A3 gene expression by IFN-{gamma}. Am. J. Physiol. Gastrointest. Liver Physiol. 298 (2), G159–G166. 10.1152/ajpgi.00374.2009 19940027 PMC2822505

[B35] Savelkoul E. H. J. Thomas P. W. A. Derikx L. den Broeder N. Römkens T. E. H. Hoentjen F. (2023). Systematic review and meta-analysis: loss of response and need for dose escalation of infliximab and adalimumab in ulcerative colitis. Inflamm. Bowel Dis. 29 (10), 1633–1647. 10.1093/ibd/izac200 36318229 PMC10547237

[B36] Schwärzler J. Mayr L. Grabherr F. Tilg H. Adolph T. E. (2024). Epithelial metabolism as a rheostat for intestinal inflammation and malignancy. Trends Cell Biol. 34 (11), 913–927. 10.1016/j.tcb.2024.01.004 38341347

[B37] Sinclair L. V. Rolf J. Emslie E. Shi Y. B. Taylor P. M. Cantrell D. A. (2013). Control of amino-acid transport by antigen receptors coordinates the metabolic reprogramming essential for T cell differentiation. Nat. Immunol. 14 (5), 500–508. 10.1038/ni.2556 23525088 PMC3672957

[B38] Sullivan S. Alex P. Dassopoulos T. Zachos N. C. Iacobuzio-Donahue C. Donowitz M. (2009). Downregulation of sodium transporters and NHERF proteins in IBD patients and mouse colitis models: potential contributors to IBD-associated diarrhea. Inflamm. Bowel Dis. 15 (2), 261–274. 10.1002/ibd.20743 18942765 PMC2627787

[B39] Tajti G. Wai D. C. C. Panyi G. Norton R. S. (2020). The voltage-gated potassium channel K(V)1.3 as a therapeutic target for venom-derived peptides. Biochem. Pharmacol. 181, 114146. 10.1016/j.bcp.2020.114146 32653588

[B40] Tsai T. Y. Wu S. N. Liu Y. C. Wu A. Z. Tsai Y. C. (2005). Inhibitory action of L-type Ca2+ current by paeoniflorin, a major constituent of peony root, in NG108-15 neuronal cells. Eur. J. Pharmacol. 523 (1-3), 16–24. 10.1016/j.ejphar.2005.08.042 16243310

[B41] Turner D. Ricciuto A. Lewis A. D'Amico F. Dhaliwal J. Griffiths A. M. (2021). STRIDE-II: an update on the selecting therapeutic targets in inflammatory bowel disease (STRIDE) initiative of the international organization for the study of IBD (IOIBD): determining therapeutic goals for treat-to-target strategies in IBD. Gastroenterology 160 (5), 1570–1583. 10.1053/j.gastro.2020.12.031 33359090

[B42] Ungaro R. Mehandru S. Allen P. B. Peyrin-Biroulet L. Colombel J. F. (2017). Ulcerative colitis. Lancet 389 (10080), 1756–1770. 10.1016/s0140-6736(16)32126-2 27914657 PMC6487890

[B43] Unterweger A. L. Jensen M. Giordanetto F. Jogini V. Rüschher A. Seuß M. (2021). Suppressing Kv1.3 ion channel activity with a novel small molecule inhibitor ameliorates inflammation in a humanised mouse model of ulcerative colitis. J. Crohns Colitis 15 (11), 1943–1958. 10.1093/ecco-jcc/jjab078 33891001 PMC8575044

[B44] Vaeth M. Kahlfuss S. Feske S. (2020). CRAC channels and calcium signaling in T cell-mediated immunity. Trends Immunol. 41 (10), 878–901. 10.1016/j.it.2020.06.012 32711944 PMC7985820

[B45] Walker N. M. Simpson J. E. Yen P. F. Gill R. K. Rigsby E. V. Brazill J. M. (2008). Down-regulated in adenoma Cl/HCO3 exchanger couples with Na/H exchanger 3 for NaCl absorption in murine small intestine. Gastroenterology 135 (5), 1645–1653.e1643. 10.1053/j.gastro.2008.07.083 18930060 PMC2673535

[B46] Wu T. Zhang H. Jin Y. Zhang M. Zhao Q. Li H. (2024). The active components and potential mechanisms of Wuji Wan in the treatment of ethanol-induced gastric ulcer: an integrated metabolomics, network pharmacology and experimental validation. J. Ethnopharmacol. 326, 117901. 10.1016/j.jep.2024.117901 38341112

[B47] Wulff H. Castle N. A. Pardo L. A. (2009). Voltage-gated potassium channels as therapeutic targets. Nat. Rev. Drug Discov. 8 (12), 982–1001. 10.1038/nrd2983 19949402 PMC2790170

[B48] Yeruva S. Farkas K. Hubricht J. Rode K. Riederer B. Bachmann O. (2010). Preserved Na(+)/H(+) exchanger isoform 3 expression and localization, but decreased NHE3 function indicate regulatory sodium transport defect in ulcerative colitis. Inflamm. Bowel Dis. 16 (7), 1149–1161. 10.1002/ibd.21183 20027604

[B49] Zahn A. Moehle C. Langmann T. Ehehalt R. Autschbach F. Stremmel W. (2007). Aquaporin-8 expression is reduced in ileum and induced in colon of patients with ulcerative colitis. World J. Gastroenterol. 13 (11), 1687–1695. 10.3748/wjg.v13.i11.1687 17461471 PMC4146947

[B50] Zhang Y. Wang X. Sha S. Liang S. Zhao L. Liu L. (2012). Berberine increases the expression of NHE3 and AQP4 in sennosideA-induced diarrhoea model. Fitoterapia 83 (6), 1014–1022. 10.1016/j.fitote.2012.05.015 22668974

[B51] Zhang S. Zhao L. Shen H. Tang Z. Qin D. Li J. (2024). International clinical practice guideline on the use of traditional Chinese medicine for ulcerative colitis by board of specialty committee of digestive system disease of world Federation of Chinese medicine societies (2023). Phytother. Res. 38 (2), 970–999. 10.1002/ptr.8087 38112572

[B52] Zhu C. Le M. He Z. Bai Y. Yang J. Ye J. (2023). Dietary berberine supplementation improves growth performance and alleviates gut injury in weaned piglets by modulating ileal microbiota and metabolites. Food Funct. 14 (9), 4143–4162. 10.1039/d3fo01044a 37060117

[B53] Zong Y. Meng J. Mao T. Han Q. Zhang P. Shi L. (2023). Repairing the intestinal mucosal barrier of traditional Chinese medicine for ulcerative colitis: a review. Front. Pharmacol. 14, 1273407. 10.3389/fphar.2023.1273407 37942490 PMC10628444

