# Supplementary material for: Wuji Wan ameliorates ulcerative colitis by restoring impaired membrane transport
Source: Front Pharmacol. 2026 Jan 27;17:1718919. doi: 10.3389/fphar.2026.1718919 (PMC12886483; doi:10.3389/fphar.2026.1718919)
Supplement: Supplementary file 1 [file DataSheet2.pdf]

Supplementary Material

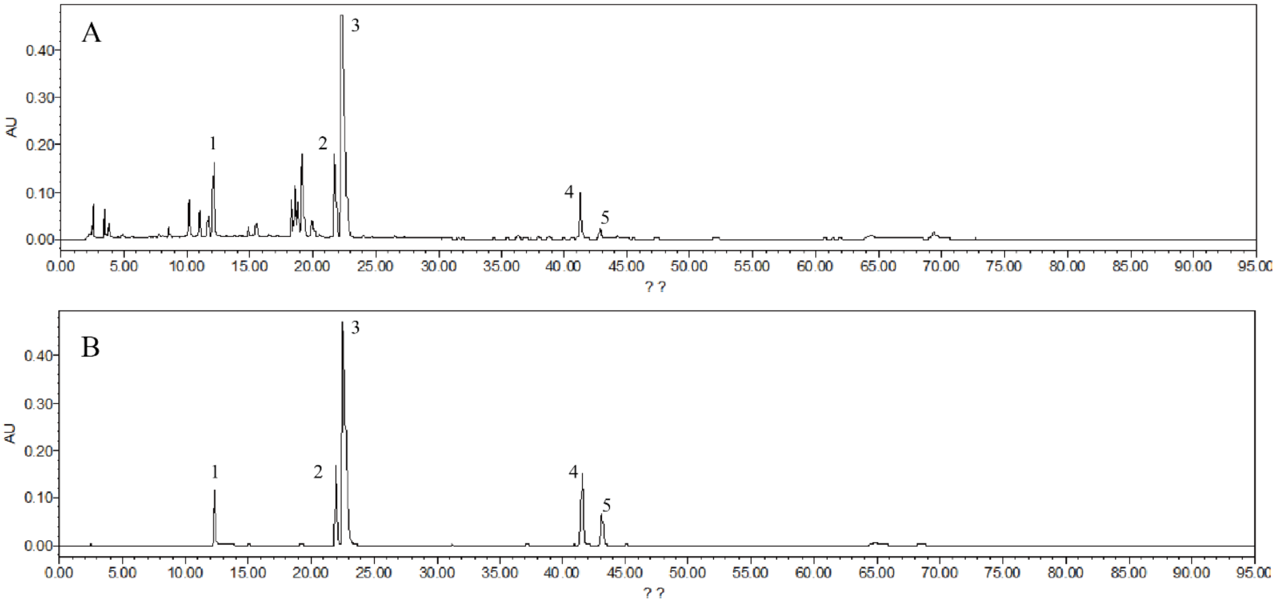

Supplementary Figure 1. Representative HPLC chromatograms of Wuji Wan (WJW) for quality control. A: Sample, B: Standards. (1: Paeoniflorin, 2: Palmatine hydrochloride, 3: Berberine hydrochloride, 4: Evodiamine, 5： Rutaecarpine).

Supplementary Table 1. Pharmacopoeial limits and measured contents of marker compounds in WJW.

Marker compound	Molecular formula	ChP 2020 (Vol. I) limit (mg/g)	Measured content (mg/g)	Compliance	Notes
Berberine hydrochloride	$C_{20}H_{17}NO_4 \cdot HCl$	⩾ 15.0	20.29	Pass	Single determination (n=1)
Paeoniflorin	$C_{23}H_{28}O_{11}$	≥ 7.0	10.76	Pass	Single determination (n=1)
Palmatine hydrochloride	$C_{21}H_{21}NO_4 \cdot HCl$	-	4.15	-	Single determination (n=1)
Evodiamine	$C_{19}H_{17}N_3O$	-	1.09	-	Single determination (n=1)
Rutaecarpine	$C_{18}H_{13}N_3O$	-	0.62	-	Single determination (n=1)
