# Supplementary material for: Wuji Wan ameliorates ulcerative colitis by restoring impaired membrane transport
Source: Front Pharmacol. 2026 Jan 27;17:1718919. doi: 10.3389/fphar.2026.1718919 (PMC12886483; doi:10.3389/fphar.2026.1718919)
Supplement: Supplementary file 2 [file DataSheet4.pdf]

frontiers

Supplementary Material

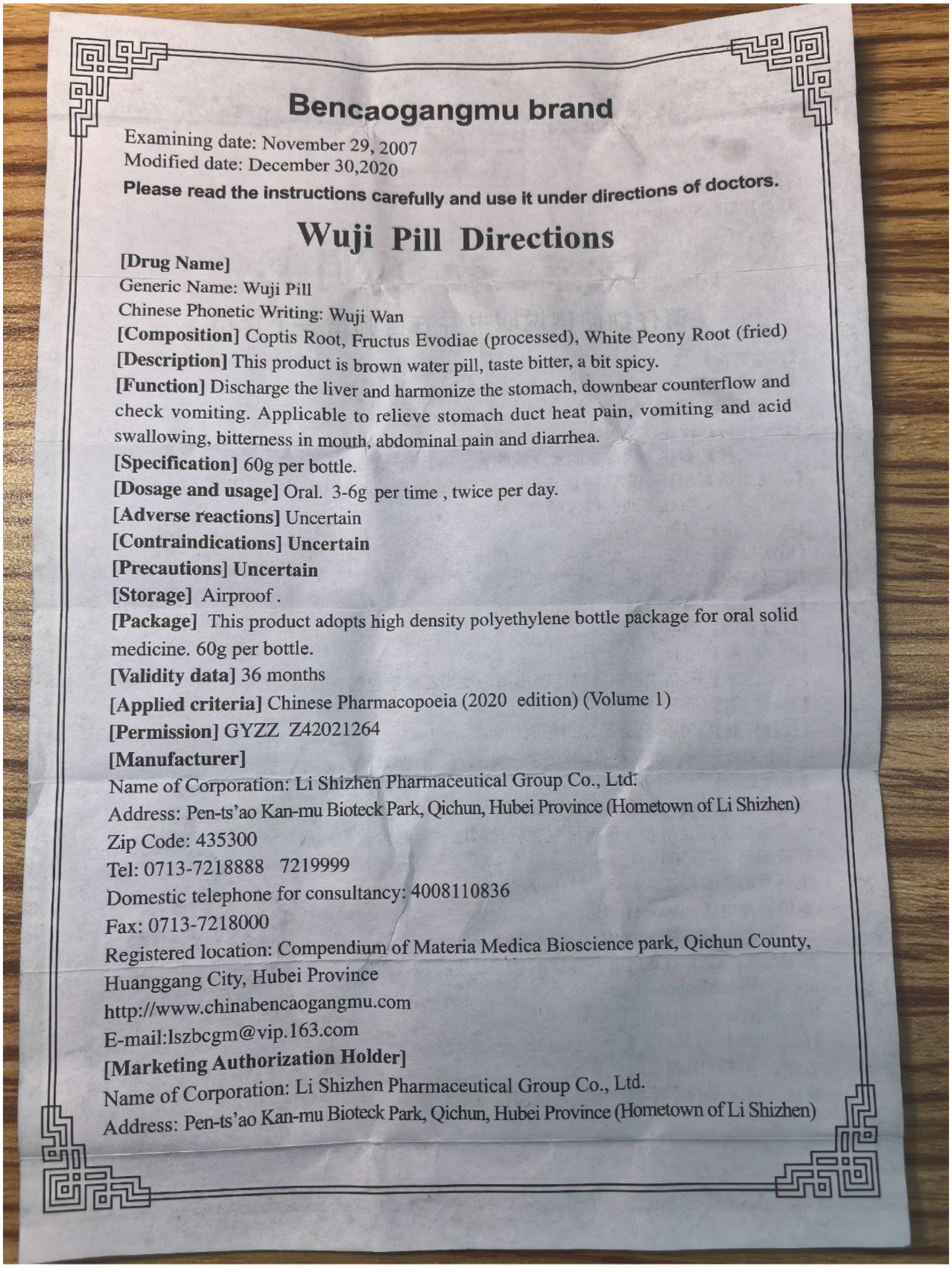

Bencaogangmu brand

Examining date: November 29, 2007

Modified date: December 30,2020

Please read the instructions carefully and use it under directions of doctors.

Wuji Pill Directions

[Drug Name]

Generic Name: Wuji Pill

Chinese Phonetic Writing: Wuji Wan

[Composition] Coptis Root, Fructus Evodiae (processed), White Peony Root (fried)

[Description] This product is brown water pill, taste bitter, a bit spicy.

[Function] Discharge the liver and harmonize the stomach, downbear counterflow and check vomiting. Applicable to relieve stomach duct heat pain, vomiting and acid swallowing, bitterness in mouth, abdominal pain and diarrhea.

[Specification] 60g per bottle.

[Dosage and usage] Oral. 3-6g per time , twice per day.

[Adverse reactions] Uncertain

[Contraindications] Uncertain

[Precautions] Uncertain

[Storage] Airproof .

[Package] This product adopts high density polyethylene bottle package for oral solid medicine. 60g per bottle.

[Validity data] 36 months

[Applied criteria] Chinese Pharmacopoeia (2020 edition) (Volume 1)

[Permission] GYZZ Z42021264

[Manufacturer]

Name of Corporation: Li Shizhen Pharmaceutical Group Co., Ltd.

Address: Pen-ts'ao Kan-mu Bioteck Park, Qichun, Hubei Province (Hometown of Li Shizhen)

Zip Code: 435300

Tel: 0713-7218888 7219999

Domestic telephone for consultancy: 4008110836

Fax: 0713-7218000

Registered location: Compendium of Materia Medica Bioscience park, Qichun County, Huanggang City, Hubei Province

http://www.chinabencaogangmu.com

E-mail:lszbcgm@vip.163.com

[Marketing Authorization Holder]

Name of Corporation: Li Shizhen Pharmaceutical Group Co., Ltd.

Address: Pen-ts'ao Kan-mu Bioteck Park, Qichun, Hubei Province (Hometown of Li Shizhen)

Supplementary Figure 3A. Wuji Pill Directions (package insert), reverse side (English text).

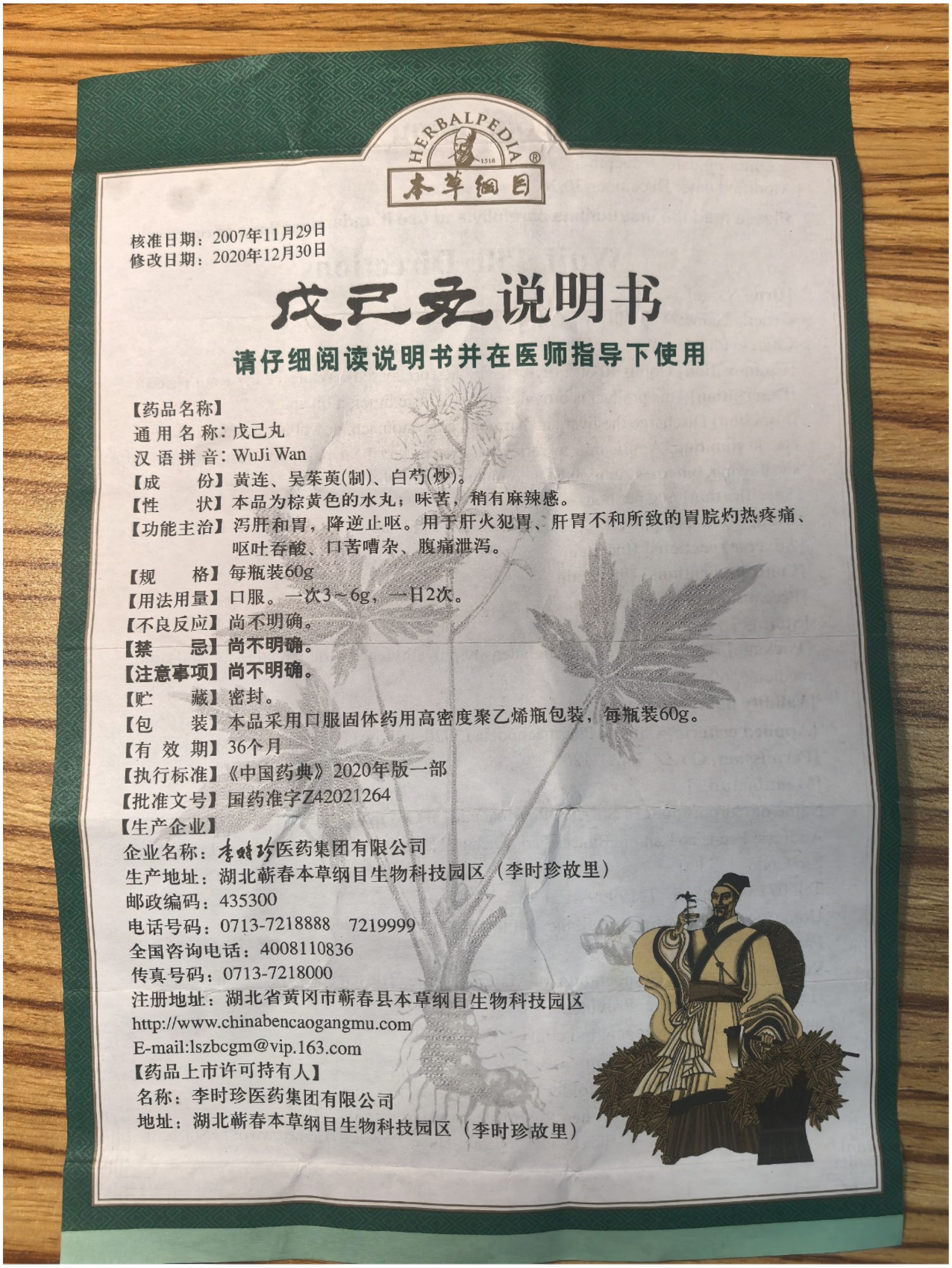

HERBALPEDIA ® 1518
本草纲目

核准日期：2007年11月29日
修改日期：2020年12月30日

戊己丸说明书

请仔细阅读说明书并在医师指导下使用

【药品名称】
通用名称：戊己丸
汉语拼音：WuJi Wan
【成　　份】黄连、吴茱萸(制)、白芍(炒)。
【性　　状】本品为棕黄色的水丸；味苦，稍有麻辣感。
【功能主治】泻肝和胃，降逆止呕。用于肝火犯胃、肝胃不和所致的胃脘灼热疼痛、呕吐吞酸、口苦嘈杂、腹痛泄泻。
【规　　格】每瓶装60g
【用法用量】口服。一次3～6g，一日2次。
【不良反应】尚不明确。
【禁　　忌】尚不明确。
【注意事项】尚不明确。
【贮　　藏】密封。
【包　　装】本品采用口服固体药用高密度聚乙烯瓶包装，每瓶装60g。
【有 效 期】36个月
【执行标准】《中国药典》2020年版一部
【批准文号】国药准字Z42021264
【生产企业】
企业名称：李时珍医药集团有限公司
生产地址：湖北蕲春本草纲目生物科技园区（李时珍故里）
邮政编码：435300
电话号码：0713-7218888　7219999
全国咨询电话：4008110836
传真号码：0713-7218000
注册地址：湖北省黄冈市蕲春县本草纲目生物科技园区
http://www.chinabencaogangmu.com
E-mail:lszbcgm@vip.163.com
【药品上市许可持有人】
名称：李时珍医药集团有限公司
地址：湖北蕲春本草纲目生物科技园区（李时珍故里）

Supplementary Figure 3B. Wuji Pill Directions (package insert), front side (Chinese original).
