# Supplementary material for: Wuji Wan ameliorates ulcerative colitis by restoring impaired membrane transport
Source: Front Pharmacol. 2026 Jan 27;17:1718919. doi: 10.3389/fphar.2026.1718919 (PMC12886483; doi:10.3389/fphar.2026.1718919)
Supplement: Supplementary file 4 [file DataSheet3.pdf]

Supplementary Material

Supplementary Note 1. Dose translation and contextual extract-equivalent estimate

The labeled adult regimen for Wuji Wan (WJW) is 12 g/day. Using a 70-kg adult reference, this corresponds to 12/70 = 0.1714 g/kg/day of the licensed commercial finished product (water pill). To map the labeled regimen to rodents for dosing in this study, we applied commonly used Km-based body-surface-area conversion factors (mouse: 9.1; rat: 6.3). Accordingly, the mouse 1× labeled-regimen dose was 0.1714 × 9.1 = 1.56 g/kg/day, and the mouse 2× dose was 3.12 g/kg/day. The rat 1× labeled-regimen dose was 0.1714 × 6.3 = 1.08 g/kg/day. *These calculations are reported to describe the dosing design relative to the marketed product label, and are not presented as a stand-alone pharmacological justification for high nominal mass dosing.*

To contextualize these finished-product doses relative to extract-focused guidance, an approximate extract-equivalent mass was estimated using a water-extraction yield of ~20% for WJW measured previously under our laboratory conditions (used here as a conservative yield assumption). Under this assumption, the highest mouse dose (3.12 g/kg/day finished product) corresponds to 3.12 × 0.20 = 0.624 g/kg/day extract-equivalent, and the rat dose (1.08 g/kg/day finished product) corresponds to 1.08 × 0.20 = 0.216 g/kg/day extract-equivalent. These extract-equivalent values are provided for contextual reference only, because the primary dosing unit in this study is the mass of the licensed finished product administered by gavage. This extract-equivalent calculation is presented solely as a conservative contextual estimate to support pharmacological interpretability and is not intended as a formal interspecies scaling rule.
