# Supplementary material for: Wuji Wan ameliorates ulcerative colitis by restoring impaired membrane transport
Source: Front Pharmacol. 2026 Jan 27;17:1718919. doi: 10.3389/fphar.2026.1718919 (PMC12886483; doi:10.3389/fphar.2026.1718919)
Supplement: Supplementary file 5 [file DataSheet1.pdf]

**Differences in metabolites between the Model group and the Control group**

| Alignment ID | Metabolite name | Rt(min) | Expreiment Mz | Adduct type | Reference m/z | MS/MS spectrum | PPM |
|---|---|---|---|---|---|---|---|
| POS4386 | Pantothenic acid | 4.396 | 220.11717 | [M+H]+ | 220.11798 | 55.0177:49954 55.05365:39474 56.01259:12366 57.06895:303380 59.04858:241967 60.04425:13005 61.02845:8034 67.05389:184512 69.06867:181612 70.02779:221788 72.04369:495929 73.02811:119645 74.02328:43455 81.06832:15058 83.04769:30535 85.06336:264051 86.0954:52130 87.07997:42329 90.05456:1912377 91.05762:28719 95.04893:165012 96.08018:52378 98.02253:384394 100.03728:43983 103.07443:131626 113.05926:48921 116.03413:187566 124.07427:344107 125.07761:14548 131.06764:15485 142.08383:103294 156.10028:10172 160.0959:19318 166.08405:18475 174.10922:13004 184.0961:69153 202.10344:51304 220.11993:8643 | -3.68E-06 |
| POS2752 | Phenylalanine | 4.381 | 166.08542 | [M+H]+ | 166.08627 | 51.02314:20908 53.03856:12102 77.03801:251645 79.05312:825183 80.04813:27428 91.05389:491642 93.0684:1242536 94.06358:30112 95.04893:214143 102.04611:26303 103.0542:4972212 104.05679:101402 105.044:23711 105.06944:23312 107.04916:486048 118.06288:64796 119.07215:44825 120.08028:14988410 121.08411:298965 131.04831:132634 | -5.12E-06 |
| POS5974 | honadienyl)-3-me | 6.165 | 279.23041 | [M+H]+ | 279.23169 | 53.03856:8408 55.05364:126356 57.06895:28213 65.03803:10419 67.05388:1126282 68.05672:28635 69.06866:117076 71.04815:14044 79.05311:77584 81.06831:947627 82.07299:28072 83.04768:14836 83.08508:58576 85.06504:13836 91.05389:23458 93.06839:89080 95.08475:784001 96.08826:25010 97.09951:25132 105.06944:23707 107.08484:69139 109.06334:16580 109.10004:245262 110.10298:14125 117.06911:12139 119.08331:21450 121.10127:40799 123.11521:94882 131.08374:32588 133.1019:15427 135.11642:38478 137.13217:49228 145.09901:20479 147.11679:15227 149.12999:24142 151.14888:9695 159.1172:11989 163.14491:13367 173.13148:21904 173.43031:9482 | -4.58E-06 |
| POS8713 | Linoleoylcarnitine | 5.468 | 424.34036 | [M+H]+ | 424.34207 | 55.05364:150847 57.03286:65854 57.06895:33783 59.04857:30031 60.08024:486717 67.05388:45969 69.06866:195045 71.08546:8042 81.06831:63957 83.08508:104109 85.02798:1963085 86.03023:37237 89.05894:14804 91.05389:10072 93.06839:18013 95.08475:57991 97.06459:9392 97.09951:58719 99.07937:8237 105.06944:7259 107.08484:11790 109.10004:30690 111.07958:8035 111.11478:21744 119.08331:7051 121.10127:10638 123.11521:13291 133.0986:8555 135.11642:11354 137.13219:10337 139.1123:6562 144.10194:39773 147.11679:10579 161.13194:12289 173.39598:7408 | -4.03E-06 |
| POS1626 | Isoleucine | 1.185 | 132.10175 | [M+H]+ | 132.10188 | 53.01286:7055 55.0177:13773 55.05365:14316 56.04953:22212 56.94233:10269 57.05693:51299 58.06417:43433 61.01003:17182 67.05389:8534 69.06868:586708 72.04369:7239 73.06432:10863 86.09541:3246155 87.02591:7895 87.09918:15027 90.05457:13104 132.10175:10606 | -9.84E-07 |
| POS6396 | nylalanylglutamic | 4.45 | 295.12854 | [M+H]+ | 295.12851 | 56.04862:20747 59.04858:5933 79.05463:6232 84.04315:468398 85.04819:6074 91.05389:7079 93.0684:10304 102.05276:13654 103.05196:53477 107.04916:26937 120.08028:1456756 121.08411:56163 130.04883:64280 131.04831:67332 136.0761:13332 149.05962:22893 166.08405:156041 167.08606:7732 186.09166:34363 232.09715:7410 | 1.017E-07 |
| POS3991 | Indolelactic acid | 4.77 | 206.08014 | [M+H]+ | 206.08121 | 57.06896:30889 67.28918:6299 91.0539:24944 99.71146:5661 103.05421:7142 115.05257:62846 117.05553:47575 118.0629:999360 119.06658:34519 130.06477:564182 131.07088:69125 132.07892:194949 133.08214:8784 142.06566:70979 143.07103:41488 144.07968:99930 146.05721:268826 147.06317:11807 160.07417:155455 170.05659:153698 171.0612:10913 188.07002:80092 194.39003:5958 206.07948:14975 | -5.19E-06 |
| POS5488 | mma-Glutamylleuc | 4.428 | 261.14288 | [M+H]+ | 261.14429 | 56.04952:47760 69.06991:56244 84.04315:1149629 85.02799:15357 85.04652:24617 86.05938:56530 86.0954:2495348 87.09917:64088 102.05498:30532 114.05461:34603 130.04884:207874 132.10173:511789 133.10521:15584 142.04747:64415 152.10725:34450 169.08443:25634 170.11852:16372 198.10959:74884 244.118:14015 | -5.4E-06 |

| Differences in metabolites between the Model group and the Control group | | | | | | | |
|---|---|---|---|---|---|---|---|
| Alignment ID | Metabolite name | Rt(min) | Expreiment Mz | Adduct type | Reference m/z | MS/MS spectrum | PPM |
| POS1617 | Creatine | 1.02 | 132.07649 | [M+H]+ | 132.07678 | 58.06512:10415 68.0483:15458 70.06557:11843 71.04816:6014 72.0542:10250 85.0836:5968 86.05939:18722 87.05381:50090 87.07824:7657 90.05457:746073 114.06509:16834 115.04728:7870 132.07567:150401 | -2.2E-06 |
| POS5117 | mma-Glutamylval | 4.382 | 247.12816 | [M+H]+ | 247.1288 | 55.05365:108200 56.04862:40411 57.03287:7214 69.06992:7648 72.08047:1399870 73.0831:28712 79.05312:10563 83.0477:12338 84.04316:628411 85.02799:8975 85.04652:13994 86.09541:41765 88.03895:16447 102.05276:15880 107.04679:23549 118.08494:251978 119.08612:7749 130.04884:85159 138.08907:11043 141.09964:7876 156.1003:28288 184.09612:60439 230.1053:8228 | -2.59E-06 |
| POS10715 | LysoPC(18:0/0:0) | 8.808 | 524.36963 | [M+H]+ | 524.37109 | 54.19788:89428 57.03287:95803 57.06989:193802 58.06512:106852 60.08025:1369087 66.97967:87274 71.07261:174615 81.0699:101604 86.09541:3593029 98.24385:89434 104.10697:20061236 105.10879:462525 124.99956:987458 163.01524:135640 184.07466:20082530 185.07635:541792 232.21115:92139 258.10977:97197 288.29056:88176 | -2.78E-06 |
| POS1544 | Pipecolic acid | 0.825 | 130.08577 | [M+H]+ | 130.08626 | 56.0486:24527 60.60572:5781 84.07954:132334 99.51071:5700 | -3.77E-06 |
| POS2185 | rans-Cinnamic aci | 4.381 | 149.05949 | [M+H]+ | 149.05991 | 53.32008:5990 56.04953:9270 65.03805:7397 69.61556:6564 74.02328:8517 77.03802:30442 79.05313:145573 79.54906:5949 84.95901:7244 91.0539:36205 93.06841:62515 95.04894:47393 102.09042:7023 103.05422:290493 105.04402:15037 105.07177:6210 107.04918:37790 121.0641:15924 131.04832:7204 149.05965:5875 | -2.82E-06 |
| POS2119 | D-Lysine | 1.052 | 147.11206 | [M+H]+ | 147.1129 | 55.05365:21431 56.04862:311733 60.08025:127686 61.08378:10757 67.05389:41656 70.0643:13652 72.08047:8148 74.02328:13541 84.04316:1430594 84.07957:1365331 85.02799:28264 85.04652:15170 85.08359:10862 87.04334:174690 88.04782:11844 100.07382:9690 101.07021:11646 102.05499:25163 130.04884:137022 130.08389:54141 146.11787:20073 | -5.71E-06 |
| POS2121 | Lysine | 0.829 | 147.1127 | [M+H]+ | 147.11278 | 55.05366:25488 56.04863:205512 58.06512:10718 64.97839:8676 67.05389:71518 68.98124:25102 72.08048:107123 74.02328:9653 84.07958:2397784 85.06337:13968 85.0836:18732 86.99279:33388 105.00241:33210 112.11258:14540 119.03033:16718 130.08389:109110 | -5.44E-07 |
| POS9113 | rachidonoylcarnitir | 5.458 | 448.34097 | [M+H]+ | 448.34207 | 55.05365:37030 57.03287:20498 57.06896:17059 59.04858:34023 60.08025:209223 67.05389:89922 69.06868:53369 73.02811:6666 79.05312:38076 81.06833:79266 83.08509:13748 85.02799:487630 86.03024:7335 87.04334:7280 89.05896:27869 91.0539:16592 93.06841:61372 95.04893:7555 95.08477:71604 105.06945:14911 107.08486:28129 109.10006:22696 119.08333:21630 121.1013:34323 123.11523:10586 131.08377:9976 133.10193:13706 135.11644:14254 144.10197:22296 145.09904:6277 147.11681:6546 149.13002:6498 157.05124:8175 166.11165:5622 | -2.45E-06 |
| POS6276 | Caryophyllen-beta | 4.377 | 290.15833 | [M+H]+ | 290.15979 | 55.05365:24601 56.04862:7940 57.03287:16604 60.08025:107724 70.0643:14590 70.43227:5768 72.08047:8247 81.56439:5745 83.0477:18170 84.07957:26752 85.02799:330220 86.03024:6849 86.09541:9647 101.0593:57444 103.04073:5965 111.04189:107845 129.05446:47478 144.10197:12880 147.06317:9623 | -5.03E-06 |
| POS5916 | coniferonic acid | 5.986 | 277.21527 | [M+H]+ | 277.216 | 55.05366:44631 57.06896:16250 58.06512:5628 65.28091:6331 67.05389:51281 68.14243:5627 69.06992:29253 79.05313:99766 81.06834:45265 83.0851:13430 91.0539:27433 93.06841:123652 95.04894:8984 95.08477:39035 97.10159:6766 105.06946:20093 107.08486:75893 109.10007:12695 119.08334:10177 121.1013:52478 131.08377:9902 133.09863:9803 135.11646:36700 147.11682:6653 149.13002:18174 | -2.63E-06 |
| POS10193 | 27,30-Decaoxadot | 4.529 | 503.30334 | [M+H]+ | 503.30618 | 73.06432:59612 87.04335:267868 89.05896:3006350 90.06191:63603 91.07443:15489 107.06821:24209 117.0909:21221 131.06766:59487 133.08545:1329197 134.09018:36332 173.42545:18521 175.09528:14502 177.11287:152154 | -5.64E-06 |

| Differences in metabolites between the Model group and the Control group | | | | | | | |
|---|---|---|---|---|---|---|---|
| Alignment ID | Metabolite name | Rt(min) | Expreiment Mz | Adduct type | Reference m/z | MS/MS spectrum | PPM |
| POS6038 | Linoleic acid | 6.277 | 281.24734 | [M+H]+ | 281.24731 | 55.05364:125812 57.06895:46625 59.04857:6943 67.05388:53085 69.06866:127913 69.45988:5680 79.05311:13502 81.06831:62368 83.08508:73554 91.05389:13556 93.06839:20966 95.08475:61126 97.06459:10580 97.09951:57009 97.41055:5954 105.06944:7469 107.08484:21791 109.10004:30321 111.07958:5866 111.11478:10256 119.0861:13805 119.28451:5333 121.10127:17172 123.11521:11864 133.1019:14386 135.11642:9635 147.11679:6899 149.12999:6540 161.13194:6625 | 1.067E-07 |
| POS6455 | Epoxyoctadecenoi | 5.771 | 297.24133 | [M+H]+ | 297.24237 | 51.75735:6200 53.80923:6083 55.01683:16956 55.05365:93349 57.03287:13354 57.06896:32501 67.05389:156887 69.06868:92838 71.04816:6667 71.08547:26065 79.05312:36059 81.06834:142837 83.04932:10612 83.08509:42345 85.06337:7733 85.10045:22460 91.0539:14153 93.06841:51028 95.08477:116463 95.10668:6615 97.06461:12573 97.09953:47258 99.07939:8130 105.06945:7673 107.08486:44411 109.10007:58942 111.0796:7031 113.09542:17052 117.06913:6481 119.08333:9755 121.1013:31741 123.11523:23177 125.09565:27342 131.08699:11338 133.09863:15672 135.11646:21771 137.1322:11367 139.11234:7678 147.11681:9530 149.13002:7365 173.13152:6600 | -3.5E-06 |
| POS5099 | Valerylcarnitine | 4.435 | 246.16867 | [M+H]+ | 246.16998 | 55.05365:9061 57.03286:58221 57.06895:137203 58.06511:8810 60.08024:316716 70.02779:20196 72.08047:64540 74.02328:10064 84.04315:17897 84.07957:18389 85.02798:2517008 85.06335:64648 86.03024:51000 86.09539:93092 99.04126:14679 103.07442:9965 114.09124:13155 118.08492:10979 132.10172:12932 144.10196:18704 187.0934:32362 246.17052:12519 | -5.32E-06 |
| POS1243 | ydroxyphenethylar | 4.382 | 120.08065 | [M+H-H2O]+ | 120.081 | 51.02236:20234 53.03856:11115 56.04862:14856 56.05763:10581 56.94139:9320 61.03869:23174 65.03804:22284 72.93572:15278 73.08309:15660 77.03802:204914 79.05312:20551 80.04813:19621 91.05389:311757 93.0684:258589 95.04893:482542 102.04612:38530 103.05421:1672750 105.04401:117492 118.06289:42145 119.07216:26808 120.08029:1302164 121.08411:10697 | -2.91E-06 |
| POS3480 | hydro-2,3-dihydro | 5.059 | 189.09024 | [M+H]+ | 189.091 | 55.0177:49571 55.05365:11699 65.03805:8920 67.05389:70001 79.05312:27394 91.0539:61400 95.04893:28404 105.03246:65584 105.06945:42646 107.04917:170598 115.05257:10935 117.06913:39553 119.08612:8633 121.02692:10609 128.06212:15991 130.06477:36301 133.06566:10016 133.09863:16817 143.08574:13797 145.06526:12545 147.07849:7829 161.09245:9120 173.43034:6694 | -4.02E-06 |
| POS2553 | 2-Aminooctanoic | 4.49 | 160.13257 | [M+H]+ | 160.13318 | 55.05364:520393 57.06987:13520 59.22437:6498 69.06866:57889 72.08046:81235 97.09951:60103 114.12788:635836 115.12945:6748 118.06563:6126 118.11523:5590 | -3.81E-06 |
| POS6500 | s-9,10-Epoxysteari | 6.019 | 299.25647 | [M+H]+ | 299.25781 | 50.23555:5582 55.05364:51847 57.06895:121094 61.54363:6034 67.05388:57802 69.06866:61990 71.08546:90570 79.05311:8757 81.06831:69652 83.08508:35820 85.10043:19202 90.70594:5740 91.05388:7903 93.06838:15439 95.08475:58498 97.09951:26773 105.06943:12217 107.08484:16452 109.10004:32294 111.11478:14801 121.10126:8975 123.1152:15658 133.0986:6310 141.12842:7674 155.14133:7758 | -4.48E-06 |
| POS331 | Sarcosine | 1.035 | 90.0547 | [M+H]+ | 90.05498 | 72.08048:13617 90.05457:7044 | -3.11E-06 |
| POS9307 | 4,27-Nonaoxanon | 4.512 | 459.2782 | [M+H]+ | 459.27997 | 73.06432:38233 87.04335:173401 89.05896:2331928 90.06191:48338 107.07059:22315 111.69564:10559 117.0909:14385 130.08708:15935 131.06766:31613 133.08545:979143 134.08685:25212 173.39603:18287 175.10025:10959 177.11287:82760 404.97165:11585 | -3.85E-06 |

**Differences in metabolites between the Model group and the Control group**

| Alignment ID | Metabolite name | Rt(min) | Expreiment Mz | Adduct type | Reference m/z | MS/MS spectrum | PPM |
|---|---|---|---|---|---|---|---|
| POS6892 | 9,10-DHOME | 5.769 | 315.2522 | [M+H]+ | 315.25269 | 55.05364:85993 57.03286:21650 57.06895:88435 67.05388:336217 68.05793:7065 69.06866:92537 71.04815:23103 71.08546:199392 72.08835:6770 79.05311:50379 81.06989:341412 82.07299:12434 83.04768:10102 83.08508:44796 85.06335:25664 85.10043:139213 86.10397:8183 91.05389:40656 93.06839:65346 95.08475:254246 96.08826:8358 97.06459:16473 97.09951:21453 99.07937:107753 105.06944:28517 107.08484:45978 109.06334:7637 109.10004:89606 111.07958:12368 111.11478:6867 113.09541:97063 117.06911:8496 119.08331:25121 121.10127:43117 123.08001:5879 123.11521:34098 131.08374:14218 133.0986:40235 135.11642:22921 137.13219:20744 139.1123:8036 145.10277:9481 147.11679:46276 149.12999:24990 151.11298:8617 151.14888:6721 153.12355:9442 159.1172:9714 161.13194:8500 163.14491:11530 165.12289:6695 167.14172:11627 173.13148:10005 | -1.55E-06 |
| POS6667 | 11Z,13E-eicosadie | 6.381 | 305.24673 | [M+H-H2O]+ | 305.24701 | 55.05365:79394 57.06988:25526 59.29884:5746 67.05389:119103 67.99528:5835 69.06868:64116 71.08547:8927 79.05312:122237 81.06833:99865 83.04769:7095 83.08509:29936 85.06505:8187 91.05389:50571 93.0684:210821 94.07142:7537 95.04694:7806 95.08476:84318 97.0646:6260 97.09953:11029 105.06944:42027 107.08485:116476 109.10005:33923 117.06912:8314 119.08332:28237 121.10129:84117 122.10348:6832 123.11816:8338 129.07021:9128 131.08376:15071 133.09862:23529 135.07935:6578 135.11644:35966 147.11681:5920 149.13393:7805 161.13196:9924 163.14493:15364 175.14502:6524 | -9.17E-07 |
| POS3491 | 6,N6-Trimethyl-L- | 0.947 | 189.15912 | [M+H]+ | 189.15976 | 54.92054:6198 56.04952:8556 60.08024:117891 67.05389:17722 70.06555:7662 72.08047:36293 75.23055:6300 84.07956:389593 100.07381:21054 101.02003:12643 119.0303:17366 130.08388:48190 147.02486:6465 189.1548:5712 | -3.38E-06 |
| POS7522 | Corticosterone | 5.231 | 347.2215 | [M+H]+ | 347.22168 | 55.05365:19614 67.05389:18172 69.03294:6617 69.06991:7063 79.05312:19613 81.06832:38824 83.04769:42664 87.04333:20494 91.05389:11120 93.0684:42854 95.04893:6980 95.08476:44260 97.0646:117671 99.04338:7680 101.0593:33397 105.06944:37506 107.04678:8476 107.08485:52010 109.06335:49831 109.10005:16612 111.07959:6791 117.06911:9748 119.08332:34740 121.06409:212080 121.09842:20296 122.06581:7709 123.08002:84590 129.07021:6978 131.08376:29713 133.09862:41665 135.07935:43567 135.11644:19583 137.09427:7193 143.08205:20364 145.09903:33114 147.07848:15714 147.11679:20820 149.0948:17200 149.13:8874 155.08327:8941 157.10196:23957 159.08273:7744 159.11722:27993 161.09682:11662 161.12756:7639 163.10913:21910 169.09859:11074 171.11404:28361 173.09726:9739 173.1315:14444 175.11017:12816 177.12296:7805 185.13042:9558 187.10989:7706 189.12688:7723 195.1138:6884 239.6185:6306 | -5.18E-07 |
| POS1655 | Ornithine | 0.825 | 133.09673 | [M+H]+ | 133.09718 | 68.98124:113904 69.03294:17362 69.06992:16352 70.0643:2376008 71.06874:13911 86.09541:57886 86.99279:132144 87.99284:8170 105.0024:128956 115.08438:14438 116.06905:94262 123.01261:10207 | -3.38E-06 |
| POS6503 | Ricinoleic acid | 6.287 | 299.2569 | [M+H]+ | 299.2579 | 53.44488:6044 53.60046:6452 55.05365:103513 57.06896:32207 65.17343:5370 67.05389:38333 69.06992:121672 71.08547:9832 79.05463:11767 81.06834:55123 83.08509:62298 91.0539:6481 93.03371:5882 93.06841:19233 95.08477:52134 97.06461:10400 97.09953:50898 105.06945:10980 107.08486:13158 109.10007:25022 111.0796:7293 111.11481:16280 119.08333:8449 121.09843:18795 123.11523:12748 125.09565:6603 133.09863:12192 135.11646:12812 138.47672:5490 149.13002:8029 161.13197:6575 | -3.34E-06 |
| POS7255 | Glutamyltryptopha | 4.476 | 334.13843 | [M+H]+ | 334.13971 | 59.45607:5788 84.04316:62247 102.05499:6059 118.06566:8077 130.04884:18961 132.07892:9335 144.07968:23359 146.05721:90437 147.05934:5933 159.09137:26060 183.63564:5349 188.07004:140819 189.07664:6173 | -3.83E-06 |
| POS4624 | Isoleucylproline | 4.391 | 229.15327 | [M+H]+ | 229.15469 | 69.06992:30904 70.06431:274902 84.08123:12069 85.02631:5938 86.09541:299671 87.09918:11021 114.05201:9145 116.06905:293098 184.09612:8011 | -6.2E-06 |

**Differences in metabolites between the Model group and the Control group**

| Alignment ID | Metabolite name | Rt(min) | Expreiment Mz | Adduct type | Reference m/z | MS/MS spectrum | PPM |
|---|---|---|---|---|---|---|---|
| POS8775 | Stearoylcarnitine | 5.752 | 428.37057 | [M+H]+ | 428.37338 | 57.03287:34494 57.06896:50800 58.06512:7169 59.04858:13497 60.08025:432255 67.05271:7761 71.08547:34924 81.0699:14444 83.08509:17433 85.028:2250157 85.10045:24065 86.03024:35076 89.05896:11687 95.08477:26836 97.09953:21863 109.10007:14300 123.11523:10734 144.10197:39277 417.58035:6609 | -6.56E-06 |
| POS827 | Benzaldehyde | 4.382 | 107.04906 | [M+H]+ | 107.0495 | 51.02236:9759 56.98664:7080 64.62771:7117 77.03802:23213 79.05312:185056 91.05389:9238 95.04893:61454 105.04401:16870 | -4.11E-06 |
| POS6456 | (10E,12Z)-9-HODE | 6.403 | 297.24136 | [M+H]+ | 297.24219 | 55.01769:6296 55.05364:38162 57.03286:6669 57.06895:22936 67.05388:68804 69.06866:25840 71.08546:240090 79.05311:21698 81.06989:73087 83.04768:6649 83.08508:18579 91.05389:20236 93.06839:25258 95.08475:55215 96.08826:7224 97.06459:7201 97.09951:6251 97.78535:5515 99.07937:96992 105.06944:17698 107.08484:16617 109.10004:20267 111.07958:6676 111.77932:5732 117.06911:6391 119.08331:12852 121.09841:20724 123.11521:6782 127.11191:10806 131.08374:10706 135.11642:11025 147.11679:46501 151.26068:5220 153.12762:6190 163.11359:6140 165.12289:10164 | -2.79E-06 |
| POS6620 | Agallochin G | 6.296 | 303.22992 | [M+H]+ | 303.23169 | 55.0177:6720 55.05366:68190 57.06896:30413 67.05389:114621 69.06869:67332 71.04816:13639 71.08419:8715 79.05313:53904 81.06834:86305 83.0851:8859 85.06337:7039 91.0539:110946 93.06841:60655 95.04894:7086 95.08478:61261 101.05931:8071 105.06946:126382 107.08487:37954 109.10007:12901 117.06913:51039 119.08334:83118 121.0641:8589 121.1013:23180 122.82823:6861 123.11524:9333 129.07022:37621 131.08377:93146 133.09863:39958 141.06726:16166 143.08574:15768 145.09904:47485 147.11682:11172 149.09483:6003 155.08328:7919 157.09775:23872 159.11723:19473 161.13199:14486 169.09862:10174 171.11407:8517 173.13153:16413 173.38622:6334 175.14503:12140 183.11301:11010 197.13113:7165 201.12592:11322 | -5.84E-06 |
| POS3505 | ihydroquinoline-4 | 4.538 | 190.04877 | [M+H]+ | 190.04961 | 55.05365:20905 67.61636:6215 72.08047:142646 89.0373:16348 99.13448:5946 110.25202:6073 115.83308:6206 116.04757:39031 133.35954:6686 144.04256:8430 144.10197:15632 162.05399:418090 163.05547:10310 173.39601:13183 190.04633:30084 | -4.42E-06 |
| POS1399 | Taurine | 1.128 | 126.02174 | [M+H]+ | 126.02198 | 53.03773:10197 55.05365:9503 56.04862:7360 66.53687:5755 68.0495:16281 70.0643:8011 78.98524:6630 80.04968:12502 84.07957:9237 98.05798:7485 108.00944:102751 108.9949:6815 109.07559:31331 126.02023:106282 | -1.9E-06 |
| POS6031 | Stearolic acid | 4.355 | 281.24564 | [M+H]+ | 281.24719 | 55.05364:45826 57.06895:26962 59.04857:6734 67.05388:17571 69.0699:49359 71.08546:9974 81.06989:18077 83.08508:33551 91.05389:5999 93.06839:8555 95.08475:26932 97.09951:17440 98.61504:5824 107.08485:10366 109.10004:9719 111.1173:7898 119.08331:8945 121.10127:8328 123.11521:7154 161.13194:5821 | -5.51E-06 |
| POS1800 | Trigonelline | 4.613 | 138.05453 | [M+H]+ | 138.0551 | 53.03773:15270 55.05365:10182 65.03805:11336 67.05389:27633 68.04829:14746 78.0336:69964 79.04105:37440 79.05162:11264 80.04968:8100 81.06834:51981 94.06554:6644 95.08477:21912 96.04379:228644 108.0432:39978 110.05836:12946 124.03867:25923 138.05421:307052 | -4.13E-06 |
| POS1475 | L-Baikiain | 1.179 | 128.07036 | [M+H]+ | 128.0708 | 53.03856:21195 54.03372:11950 55.0177:17257 55.05453:94553 56.04952:7843 57.04489:12639 58.06511:13196 62.47697:5464 67.0421:17684 70.06556:9392 71.04944:9132 72.58782:6505 82.06502:127867 86.05938:6760 100.07597:11830 110.05835:9058 127.03809:17037 128.07144:17484 | -3.44E-06 |
| NEG253 | mino-2-methylbu | 0.908 | 97.07714 | [M-H]- | 97.0772 | 79.05368:126619 80.05277:612254 94.18431:5963 97.07645:2942376 | -6.18E-07 |
| POS6592 | -Aminooctadecan | 5.507 | 302.30365 | [M+H]+ | 302.30539 | 55.05365:26947 56.04862:6381 57.06896:15481 60.04425:231657 67.05389:30758 69.06868:25997 70.0643:9173 71.08547:6605 81.06989:26805 83.08509:14741 88.07444:7951 95.08476:27072 97.10158:11068 109.10005:11294 120.03226:5724 165.78566:5174 168.79677:5089 183.07042:7081 298.44339:6124 | -5.76E-06 |
| POS8198 | (+)-Diaeudesmin | 5.535 | 387.17865 | [M+H]+ | 387.18011 | 59.04858:7208 79.05312:18069 81.03229:7618 95.04893:7858 103.05421:12348 105.06945:1023412 106.07115:41636 119.04706:18835 121.0641:14413 124.34185:5209 141.08885:5042 163.98055:5395 197.54788:5811 281.13101:5592 | -3.77E-06 |

| Differences in metabolites between the Model group and the Control group | | | | | | | |
|---|---|---|---|---|---|---|---|
| Alignment ID | Metabolite name | Rt(min) | Expreiment Mz | Adduct type | Reference m/z | MS/MS spectrum | PPM |
| POS9645 | oPE(18:2(9Z,12Z)/( | 6.153 | 478.29233 | [M+H]+ | 478.29269 | 51.34163:6667 55.05364:85019 55.21454:5493 57.03286:78181 57.06987:29910 62.06013:11470 67.05388:140674 69.06866:123864 71.08546:10086 79.05311:25298 81.06989:154548 83.08508:80852 85.06335:6763 87.29327:6183 91.05389:13545 93.06839:34365 95.08475:122181 97.06459:13284 97.09951:38596 105.06944:17190 107.08484:27814 109.10004:60872 111.1173:11633 119.0861:16510 121.10127:24420 123.11521:25988 133.0986:16699 135.11642:18706 137.13217:9333 147.11679:13918 149.1339:8839 161.13194:6938 162.26683:6997 163.14938:8087 | -7.53E-07 |
| POS4316 | Propionylcarnitine | 1.092 | 218.13759 | [M+H]+ | 218.13869 | 57.03286:11116 60.08024:37522 61.03971:9927 84.07957:9433 85.02798:259449 | -5.04E-06 |
| POS6404 | (Z),9(Z),12(Z)-octa | 6.243 | 295.22681 | [M+H]+ | 295.22672 | 55.01769:6971 55.05364:28988 57.03286:55182 57.06987:16117 62.27805:5648 67.05388:51800 69.06866:22390 71.08418:17836 79.05311:28301 81.06831:65954 83.04931:7079 83.08508:9164 91.05389:13807 93.06839:42394 95.08475:49425 99.07937:7131 105.06944:8780 107.08484:31933 109.06334:6201 109.10004:20091 119.08331:6609 121.10127:25600 133.0986:8134 135.11642:19060 186.14619:5502 226.35844:5011 | 3.049E-07 |
| POS6403 | 4'Z,8E)-Colneleic | 5.647 | 295.22653 | [M+H]+ | 295.2265 | 52.0545:5624 55.01769:9634 55.05364:34234 57.03286:12621 57.06895:8575 59.04857:8780 67.05388:39854 69.0699:28580 71.04943:7208 71.08546:7824 79.05311:24591 81.06831:37881 83.08508:8951 91.05389:11435 93.06839:30809 93.93243:6190 95.08475:32607 97.06459:9142 97.09951:9384 105.06944:8362 107.08484:28725 109.06334:6243 109.10004:14948 119.08331:11954 121.09841:17574 133.1019:8700 135.11642:10539 145.09901:5857 146.10268:6223 173.39598:9476 | 1.016E-07 |
| POS3359 | midopropyl)pyrro | 4.424 | 185.12825 | [M+H]+ | 185.129 | 53.14906:6051 55.05365:12264 59.64061:5031 70.06429:11845 86.4879:5365 98.06005:86194 100.07381:18263 116.97124:6533 125.10766:20113 126.09013:189282 143.1188:8464 | -4.05E-06 |
| POS5556 | -trans-Bergamoto | 5.875 | 263.20016 | [M+H]+ | 263.2002 | 53.03772:7535 55.01768:18061 55.05364:260452 57.03285:7077 57.06894:58783 59.04856:11671 61.48146:5472 67.05387:164706 69.06866:100524 71.04814:35224 71.08417:15772 77.03799:16605 79.0531:220578 81.06988:155633 82.065:17420 83.04767:15082 83.08507:24513 85.02797:7625 85.06503:19485 87.04332:8695 91.05387:214431 92.05666:5810 93.06838:282421 94.07336:8874 95.0489:18751 95.08474:72032 97.06458:6866 97.10155:11060 105.06942:235724 106.07346:13731 107.08483:105249 109.06332:14729 109.10003:25466 117.06909:49148 119.08329:197641 120.08875:12157 121.06406:5999 121.10126:52704 123.1152:9954 129.06702:29755 131.08372:58224 133.09859:121701 135.11641:21689 142.07654:5827 143.08569:20258 145.099:14880 147.07845:21436 147.11676:60528 149.09477:6374 149.12997:16558 157.10193:16064 161.09679:24467 161.13193:28054 163.1449:8970 171.11401:6749 175.11014:15110 179.10367:7485 203.17516:6551 | -1.52E-07 |
| POS6563 | (+)-Cedronellone | 6.077 | 301.21539 | [M+H]+ | 301.21619 | 55.05366:25368 67.05389:56577 69.06868:18458 71.04816:7128 79.05313:39738 81.0699:50896 85.06506:5491 91.0539:61031 93.06841:58806 95.08477:29793 100.07598:8951 105.06946:56484 107.08486:28652 109.10007:13815 117.06913:59324 119.08334:41936 121.1013:28848 129.06706:5995 131.08377:59156 133.09863:46173 135.11646:18568 143.08574:10898 145.09904:38449 147.11682:18107 157.10197:7542 159.11723:35770 161.13197:10943 171.11888:11544 173.13153:21280 | -2.66E-06 |
| POS1790 | methyl-1,3-buta-( | 6.298 | 137.13211 | [M+H]+ | 137.1324 | 67.05386:16876 79.0546:6534 81.06986:23444 95.08472:7924 | -2.11E-06 |
| POS5559 | Farnesyl acetone | 6.02 | 263.23538 | [M+H]+ | 263.2366 | 55.05365:13059 57.06896:12734 65.03692:8294 67.05389:23489 69.06991:10162 71.08547:9394 79.05312:9136 81.0699:28906 91.0539:7192 93.06841:14666 95.08477:22257 105.06945:8580 107.08486:11834 109.10006:9848 116.69191:5808 119.08333:9577 121.1013:6421 133.09863:8001 | -4.63E-06 |
| POS12629 | ecapropylene glyc | 5.72 | 599.43445 | [M+H]+ | 599.43646 | 57.03287:98999 59.04858:1006998 60.05225:10498 73.02811:17432 87.04334:26531 89.05895:57361 103.07444:19106 115.07378:13303 117.09089:142591 133.08543:25731 175.1301:24920 | -3.35E-06 |

| Differences in metabolites between the Model group and the Control group | | | | | | | |
|---|---|---|---|---|---|---|---|
| Alignment ID | Metabolite name | Rt(min) | Expreiment Mz | Adduct type | Reference m/z | MS/MS spectrum | PPM |
| POS14450 | | 4.642 | 721.41571 | [M+2H]2+ | 721.41571 | 69.03294:64032 73.02811:159873 73.06432:77520 83.45062:47888 87.04334:536056 89.05896:2457454 90.0619:93104 95.04694:86163 98.23338:47352 111.04189:78648 111.44507:43274 113.05927:60756 121.66422:45778 131.07088:82096 133.08543:851508 137.0598:58032 177.11285:88833 200.87497:48388 327.85837:47698 656.01245:49795 | 0 |
| POS11255 | asterone 22-O-su | 4.584 | 545.31409 | [M+2H]2+ | 545.31421 | 67.05389:29798 69.03294:42084 73.02811:194322 73.06432:63047 81.03229:54889 83.04932:51834 87.04334:399634 89.05896:2005170 90.0619:67636 91.04643:47910 95.04893:140429 99.04339:71219 107.07058:39913 108.77773:25939 109.02668:26574 109.06336:35887 111.04189:185371 113.05927:53829 131.07088:53610 133.08543:599344 134.08684:38149 137.0598:75205 155.06668:55882 177.11285:59448 274.02045:23581 311.28952:23464 | -2.2E-07 |
| POS10376 | His Gln Val Lys | 4.662 | 511.29846 | [M+H]+ | 511.29861 | 69.03291:46719 73.02808:224037 73.06429:52427 80.05272:60468 87.0433:471413 88.04601:45781 89.05892:2417979 90.06187:116949 91.04639:54072 95.0489:109640 99.04335:56884 111.04436:90199 113.05922:59512 129.05441:36895 131.07082:54101 133.08539:702772 173.39104:87009 177.10771:62434 323.89127:41408 | -2.93E-07 |
| POS4996 | alpha-Humulene | 4.604 | 243.15111 | [M+2H]2+ | 243.15096 | 73.02811:8617 104.56932:5620 136.52379:5669 173.43034:6491 207.27916:5990 227.04758:5414 | 6.169E-07 |
| POS13566 | Dianthosaponin C | 4.577 | 652.40558 | [2M+H]+ | 652.40552 | 77.99068:37795 87.01893:36948 87.04334:142321 89.05896:1047998 131.06766:38560 133.08543:452202 166.69713:42909 177.11285:79337 400.16666:38883 | 9.197E-08 |
| POS7122 | Boeravinone F | 0.879 | 327.04965 | [M+Na]+ | 327.04971 | 86.16064:5992 102.22125:5057 191.07452:111349 232.32516:5391 | -1.83E-07 |
| POS12311 | Bilirubin | 4.692 | 584.67792 | [M+H]+ | 584.6781 | 73.02811:187672 73.06432:101464 80.05429:116505 87.04334:652252 89.05896:3177115 90.0619:109811 91.04457:60691 95.04893:140279 102.06606:66387 103.98616:58381 111.04441:87383 113.05927:81682 117.09089:58913 131.06766:103725 133.08543:1659876 134.08684:94500 137.05981:68955 155.0667:75801 175.09526:79543 177.11285:344461 210.66563:63524 | -3.08E-07 |
| POS12919 | Puwainaphycin D | 4.706 | 613.35681 | [M+H]+ | 613.35699 | 73.02811:56583 87.04334:169634 89.05896:974096 90.0619:39072 102.56839:25109 113.05927:37699 117.20798:23434 130.75883:29066 133.08543:348689 177.11285:53095 202.06644:32968 | -2.93E-07 |
| POS12917 | Austrobuxusin I | 4.569 | 613.33508 | [2M+H]+ | 613.33502 | 50.28071:22135 76.60421:18230 279.35773:16421 408.97412:18993 449.91245:18538 | 9.783E-08 |
| POS13499 | Lotusine E | 4.542 | 648.37427 | [M+NH4]+ | 648.37433 | 69.03294:40275 83.04769:18316 87.04333:116802 89.05895:355918 99.04338:82046 111.04189:38023 118.28912:15999 125.05659:19656 131.07086:18570 133.08543:149936 155.07082:41003 173.43523:19753 177.11284:22214 377.14182:16945 | -9.25E-08 |
| POS10612 | 180083-23-2 | 4.489 | 521.25366 | [M+H]+ | 521.25378 | 76.65031:7219 86.09541:8095 87.04334:12034 89.05896:183626 90.0619:14901 104.10696:28155 133.08543:76964 134.08684:9642 177.11285:11352 184.06929:12850 466.78955:6213 | -2.3E-07 |
| POS9097 | Mutamicin 5 | 5.214 | 447.29263 | [M+H]+ | 447.29251 | 52.18702:7097 59.04856:8204 75.88783:5461 89.05891:10473 130.28481:6147 149.63971:5515 258.80569:5579 317.15057:5762 330.39728:5447 339.19794:5301 | 2.683E-07 |
| POS14088 | an-2-yl)-17-oxa-1 | 4.554 | 692.4007 | [M+H]+ | 692.401 | 69.03294:34406 83.04932:19091 87.04334:79213 89.05896:243289 99.0434:58198 111.04441:37770 133.08545:97630 137.05981:16046 173.38622:28450 177.11285:22280 319.43127:17043 | -4.33E-07 |
| NEG6553 | etramethylheptade | 0.899 | 323.29568 | [M-H]- | 323.29559 | 77.07738:6812 79.05368:18879 90.50151:5857 103.07947:75414 104.78513:5409 113.12115:24053 119.0854:12567 124.15677:13453 187.15546:156768 | 2.784E-07 |
| POS3472 | 4-Chlorobiphenyl | 0.753 | 189.04665 | M+CH3OH+H] | 189.04649 | 59.59612:5875 68.98123:50815 78.67258:6030 83.00869:42062 86.99104:76636 100.03513:13540 101.01785:244130 102.01955:9461 105.0024:100551 118.0436:34416 119.02752:344739 120.02943:15424 123.0126:13613 129.01353:31462 131.08376:5520 137.03912:139074 138.04027:7743 144.02397:7307 147.02486:159008 161.05292:15694 165.03181:23271 179.06253:7655 | 8.464E-07 |

| Differences in metabolites between the Model group and the Control group | | | | | | | |
|---|---|---|---|---|---|---|---|
| Alignment ID | Metabolite name | Rt(min) | Expreiment Mz | Adduct type | Reference m/z | MS/MS spectrum | PPM |
| POS9531 | olic acid glycine co | 4.473 | 472.27298 | [M+H]+ | 472.27301 | 69.03294:38778 73.02811:13523 73.06432:6329 83.0477:15500 87.04334:72463 87.09045:5532 89.05896:142651 93.32733:6502 95.04694:6716 99.04339:53482 107.07059:5835 111.04189:35667 122.36179:5627 123.80167:6072 125.0596:10348 131.06766:7048 133.08543:49732 155.07083:13830 333.67181:5748 | -6.35E-08 |
| POS13624 | eta-D-glucopyran | 4.717 | 657.38458 | [M+Na]+ | 657.3844 | 73.02678:32745 87.04334:110396 89.05896:562632 90.06374:31628 95.04893:28345 129.8802:24921 133.08543:277951 177.11285:50766 373.06967:24111 | 2.738E-07 |
| POS9326 | BW A868C | 6.157 | 460.28043 | [M+H-H2O]+ | 460.28049 | 50.52047:6237 56.0153:6455 173.39111:6724 | -1.3E-07 |
| POS5433 | ofumesate-2-hydr | 0.88 | 259.06332 | [M+Na]+ | 259.06339 | 75.5243:6317 80.34119:5069 119.49465:5860 150.53221:5378 153.20093:6466 191.07452:69247 | -2.7E-07 |
| POS1644 | THTC | 1.146 | 133.03159 | [2M+H]+ | 133.03169 | 53.03856:10928 55.05365:15188 56.04952:34333 57.05692:121539 58.06416:77048 58.99405:15342 59.04858:12006 61.01003:54938 67.05389:18755 69.06867:1093632 70.06934:24785 70.07311:93038 73.06432:9416 86.0954:2214347 87.0259:32303 87.09917:177128 90.05456:13203 | -7.52E-07 |
| POS7250 | Sarcostolide G | 5.29 | 333.20493 | [M+H]+ | 333.2049 | 72.03056:5233 93.06841:5945 97.06461:14230 107.08486:8269 109.06336:8815 114.88049:5409 121.06409:38398 123.08003:13336 147.11681:7411 180.61118:6398 245.93018:5880 324.12939:6920 | 9.003E-08 |
| POS7280 | dro-15-keto Prost | 5.614 | 335.21899 | [M+Na]+ | 335.21899 | 51.63123:6052 57.83962:6671 76.67337:5677 79.88229:5885 95.08477:6673 109.49762:5725 | 0 |
| POS3471 | 4-Chlorobiphenyl | 1.023 | 189.04648 | [M+H]+ | 189.04649 | 55.17931:6214 58.06416:19840 60.08024:33413 70.06429:25884 72.04369:29659 72.08047:435680 73.08309:21275 83.00869:10138 84.07957:203678 100.07381:216677 101.01785:73082 101.07893:15345 112.11002:5731 117.10176:10318 118.04636:10666 119.0303:116327 126.09013:8868 129.01353:10243 130.08388:20478 136.05563:5951 137.03912:40195 147.02486:57206 161.04852:7674 165.03636:13179 171.14771:6036 | -5.29E-08 |
| POS10232 | Lys Glu Asn Asp | 4.366 | 505.22525 | [2M+H]+ | 505.22519 | 60.04425:13615 72.08047:24447 89.88777:6027 115.0499:6708 118.08492:8113 129.1017:13294 154.0515:7791 175.10519:15609 189.08778:5882 | 1.188E-07 |
| POS7516 | Argophyllin C | 4.821 | 347.18448 | [M+H]+ | 347.18451 | 53.47846:5443 90.58919:5692 102.95767:6152 105.06945:5447 113.36972:5229 121.06409:29767 170.89314:5683 | -8.64E-08 |
| POS14977 | -3-hydroxydecano | 5.943 | 780.54694 | [M+H]+ | 780.547 | 51.74374:10069 55.43724:8301 60.08022:27942 71.07257:10790 80.74166:8042 86.09537:79987 104.10693:11189 124.9995:22929 161.76192:7751 184.07458:204750 464.99683:8789 | -7.69E-08 |
| POS7490 | or-9-carboxy-Del | 5.167 | 345.20386 | [M+H]+ | 345.20389 | 55.05364:8745 57.03286:10758 57.06987:8631 59.04857:156108 60.08023:24029 67.05388:7682 73.0281:9297 79.05311:9225 81.06989:15609 83.04768:7036 85.02798:158970 86.03194:17410 87.04333:10279 89.05894:49265 93.06838:15565 95.08475:14207 97.06458:10748 101.05928:12799 103.07442:9886 105.06943:6111 107.08484:16588 121.06407:72357 123.08001:6829 131.08696:8149 133.1019:9366 135.07932:10998 143.08571:6090 144.25438:6320 145.10277:9176 147.07846:12472 155.08324:6498 157.10194:7401 163.1091:6871 173.09724:13813 297.94568:5678 | -8.69E-08 |
| POS6795 | Estriol | 8.81 | 311.16177 | [M+2H]2+ | 311.16171 | 55.05365:58863 56.96447:7808 57.06895:432831 58.07271:19524 60.08024:27058 67.05389:88310 69.06867:69084 71.08546:254363 72.08836:10565 81.06832:104380 82.073:12499 83.08508:55048 85.10044:123869 86.0954:109906 87.09917:8681 89.05894:6052 95.08476:162792 96.08826:10354 97.09952:57875 102.09041:20834 104.10696:106566 109.10005:71073 111.1148:21223 115.96169:10215 116.98483:6866 123.11522:27162 124.99953:28113 130.99998:7779 150.03761:8254 166.54468:12846 173.43033:15135 181.02368:7195 184.06927:22644 240.09717:13120 | 1.928E-07 |
| POS9465 | 28-Norbrassinolide | 6.426 | 467.33469 | [M+2H]2+ | 467.33459 | 57.03378:8878 57.53098:5601 59.04858:61853 87.04333:8149 122.83405:6319 128.02786:5926 173.42542:6550 | 2.14E-07 |
| POS7879 | Bortezomib | 4.451 | 367.19409 | [M+H]+ | 367.194 | 55.05365:7737 62.02026:6111 69.03294:36387 74.89256:5499 83.04932:16016 86.05939:42416 87.04334:41179 89.05896:33192 93.07034:15025 98.05798:24268 98.65292:5798 99.04339:11100 111.04189:29575 112.07434:26726 113.07734:7178 128.07144:8579 133.08543:6665 152.10727:6122 173.34698:5856 178.11943:16843 317.81894:5571 | 2.451E-07 |

**Differences in metabolites between the Model group and the Control group**

| Alignment ID | Metabolite name | Rt(min) | Expreiment Mz | Adduct type | Reference m/z | MS/MS spectrum | PPM |
|---|---|---|---|---|---|---|---|
| POS3963 | -methyl-2-buteny | 6.297 | 205.1212 | [M+H]+ | 205.1221 | 69.90211:5326 72.95045:5817 91.0539:9489 | -4.39E-06 |
| POS2609 | (R)-Boschniakine | 4.733 | 162.09058 | M+CH3OH+H] | 162.0905 | 55.80161:6038 80.0266:7379 103.05195:17483 120.08028:56350 | 4.936E-07 |
| POS5047 | Pseudouridine | 1.585 | 245.07607 | [M+H]+ | 245.0761 | 54.03372:7226 57.3748:6140 61.03869:8235 68.04829:6953 79.05161:7372 80.04967:7066 82.02831:10593 100.03728:16503 125.03256:34265 155.04594:13881 167.04433:6999 189.8046:5950 191.04048:9591 | -1.22E-07 |
| POS6562 | 4-Oxoretinol | 6.339 | 301.21536 | [M+H-H2O]+ | 301.21539 | 55.0177:11256 55.05365:7648 69.06867:18526 81.06832:6768 95.00317:5636 177.55406:6154 | -9.96E-08 |
| POS6932 | Didemnilactone A | 5.088 | 317.21069 | [M+H-H2O]+ | 317.21069 | 55.01768:7347 55.05452:7069 57.06987:7703 67.05387:6094 69.06866:6327 71.08545:24519 79.05461:6074 81.06831:11657 83.75761:5774 99.07936:7885 105.06942:6155 108.6657:6482 117.06909:6488 129.07018:7550 143.08569:7294 175.81395:5798 | 0 |
| POS2606 | Aminopentanoic a | 1.019 | 162.04997 | [M+NH4]+ | 162.05 | 55.0177:68381 55.05365:34633 56.04862:40127 57.03286:476354 58.06511:99164 59.07295:127286 60.08024:2624557 61.02742:35746 70.0643:36310 84.07957:63394 85.02799:1124644 98.06006:93569 102.09041:716844 103.03848:1458833 162.11159:578657 | -1.85E-07 |
| POS6933 | Didemnilactone A | 5.508 | 317.21069 | M+CH3OH+H] | 317.21069 | 78.49305:6069 86.90919:5969 97.06458:75463 109.06334:59271 153.79721:6272 173.39107:5731 184.94119:6312 232.74396:6119 | 0 |
| POS4735 | Alantolactone | 6.299 | 233.15237 | [M+Na]+ | 233.1524 | 152.50285:5564 | -1.29E-07 |
| POS5496 | -octadeca-5,9,12- | 5.769 | 261.21991 | [M+H-H2O]+ | 261.22 | 67.05388:6690 91.05389:6161 | -3.45E-07 |
| POS428 | Toluene | 4.381 | 93.06992 | [M+H]+ | 93.0698 | 57.03286:10207 70.25361:5628 95.04694:8249 | 1.289E-06 |
| POS7916 | Cortol | 5.88 | 369.26291 | [M+H-H2O]+ | 369.26291 | 67.05388:11127 69.0699:11500 81.06989:13595 95.08475:10779 110.42126:6159 121.09841:8099 147.11679:7654 180.52777:6221 | 0 |
| POS7956 | PARTEINE SULFAT | 5.765 | 371.1701 | [M+H]+ | 371.1702 | 76.54377:5973 173.39111:6509 182.17998:5526 | -2.69E-07 |
| POS337 | L-Alanine | 4.392 | 90.05499 | M+CH3OH+H] | 90.055 | 61.02742:77191 64.32306:6811 69.61306:6290 72.08047:8526 | -1.11E-07 |
| POS3147 | xy-5-phenyl-3-pe | 6.299 | 179.10619 | [M+NH4]+ | 179.1062 | 91.05389:11793 105.06944:10473 119.08332:10252 | -5.58E-08 |
| POS7648 | Grandilobatin A | 6.612 | 353.26736 | [M+H]+ | 353.2674 | 67.05388:6123 91.09308:6433 107.08484:7637 119.67999:6221 140.46465:5768 | -1.13E-07 |
| POS13902 | )-dioic acid 29-me | 4.652 | 677.38928 | [M+2H]2+ | 677.38953 | 69.03294:73562 73.02811:197486 73.06432:65878 81.03229:39471 82.00758:33699 83.04932:46218 87.04334:562341 89.05896:2671234 90.0619:58131 91.04643:49146 95.04893:109715 99.04339:72157 111.04189:137158 113.05927:74273 117.09089:32831 131.07088:47371 133.08543:871621 134.09018:38962 137.0598:50855 155.07083:46456 177.11285:107687 | -3.69E-07 |
| POS10736 | | 4.66 | 525.30688 | [M+H-H2O]+ | 525.30713 | 69.03294:67522 73.02811:187355 73.06432:73247 80.05429:75077 81.03385:33790 83.0477:34944 87.04334:431912 88.04604:27180 89.05896:2465756 90.0619:86112 91.04643:88865 95.04893:88450 99.04339:69196 102.06606:66841 107.06821:25147 111.04441:100871 113.05927:70640 117.08817:29401 124.08022:23947 131.06766:68756 133.08543:860051 134.08684:45384 137.0598:61376 155.07083:33039 177.11285:91886 | -4.76E-07 |
| POS14851 | xy-12,19(29)-ursa | 4.666 | 765.43976 | [M+2H]2+ | 765.44 | 59.04858:189374 69.03294:107946 73.02811:457562 73.06432:231440 81.03229:104140 87.04334:1581831 89.05895:7716564 90.06374:158789 91.04643:139834 95.04893:226545 99.04339:207252 109.06335:110190 111.0444:307058 113.05927:264513 117.09089:189153 131.06764:339911 133.08543:4354042 134.09016:181612 137.0598:223439 146.80688:80855 155.07083:299185 175.09526:154709 177.11284:864362 178.11432:109079 199.09537:129018 221.14047:110403 243.12985:79798 | -3.14E-07 |
| POS5546 | enyl beta-D-gluco | 4.585 | 263.14828 | [M+2H]2+ | 263.1485 | 69.03294:7268 87.04334:5508 171.10925:6106 191.00081:5391 253.66177:6313 | -8.36E-07 |
| POS15066 | )kadaic acid (-H2C | 4.657 | 787.46161 | [M+2H]2+ | 787.46198 | 64.15166:115119 87.04332:165423 89.05893:744046 133.0854:434553 282.94159:115379 783.29553:120371 | -4.7E-07 |
| POS6696 | 4-Gallocatechol | 3.027 | 307.08179 | [M+2H]2+ | 307.0816 | 56.04862:16784 68.02298:7664 76.0215:19080 76.03858:56598 84.04315:369127 96.04378:9515 102.05498:9126 130.04883:145558 131.04509:24300 140.01526:12904 177.03188:30486 179.0471:13981 | 6.187E-07 |
| NEG2957 | Monuron | 1.216 | 197.049 | [M-H]- | 197.0488 | 55.23539:6281 68.33942:6047 73.52995:5450 81.53988:5503 161.03627:460728 162.03664:28802 163.0374:1032564 198.04526:168570 | 1.015E-06 |

**Differences in metabolites between the Model group and the Control group**

| Alignment ID | Metabolite name | Rt(min) | Expreiment Mz | Adduct type | Reference m/z | MS/MS spectrum | PPM |
|---|---|---|---|---|---|---|---|
| POS12097 | ethyl]-10,14,16,16 | 4.692 | 575.00769 | M+CH3OH+H] | 575.00739 | 73.02811:29511 87.04334:93472 89.05896:469726 90.0619:14744 95.04893:13062 99.04339:14744 99.79063:13882 113.05927:17085 131.07088:13450 133.08543:160500 177.11285:21906 366.98584:13541 | 5.217E-07 |
| POS6773 | 6-Hydroxynobiline | 4.717 | 310.20068 | [M+Na]+ | 310.2009 | 55.05452:9349 57.09766:6312 59.04858:12269 60.08024:15333 85.02798:148494 93.06839:6424 121.10128:7372 131.23537:7547 | -7.09E-07 |
| POS9402 | LysoPE(18:0/0:0) | 6.085 | 464.31125 | [M+H-H2O]+ | 464.311 | 57.03286:12124 59.04955:8892 342.63168:5391 | 5.384E-07 |
| POS1671 | L-Alanine | 0.975 | 134.01877 | [M+NH4]+ | 134.019 | 62.98165:6876 70.06429:208419 71.06873:13251 74.02327:22845 90.05455:18754 91.05762:41642 92.05856:6291 93.26151:5477 115.08436:6481 116.06902:6879 133.07881:8893 | -1.72E-06 |
| POS4659 | xo-1,2,3,4-tetrahy | 0.757 | 231.0336 | [M+H]+ | 231.0334 | 75.13534:6435 86.99104:18560 94.8403:5815 101.00695:12948 105.0024:19452 106.35088:5540 109.73916:5246 113.00764:8928 119.01637:16041 129.00409:13318 131.01932:16179 147.01338:23615 | 8.657E-07 |
| POS4682 | Eremanthin | 4.376 | 231.13277 | [M+H-H2O]+ | 231.133 | 55.05365:24699 56.04862:40425 57.03286:18158 57.06988:19215 58.06511:8324 60.08024:104564 69.06991:17024 71.04816:10536 72.08047:78270 84.07957:178283 85.02798:890626 86.0954:77961 100.07381:50362 112.07433:16446 126.09013:85200 129.1017:10055 132.10173:9434 144.10196:9662 168.13461:19759 173.0777:11329 214.14249:8406 | -9.95E-07 |
| POS5564 | Z)-Octadecadien | 6.288 | 263.23578 | [M+H-H2O]+ | 263.23599 | 55.05364:9969 57.06895:5869 62.58315:5822 67.05388:24081 69.0699:7106 81.06831:24764 83.08508:6428 93.06839:10323 95.08475:20730 107.08484:6287 109.10004:13846 121.10127:6250 193.25415:5595 | -7.98E-07 |
| POS10783 | -Indocarbazostati | 1.459 | 527.15625 | [M+H]+ | 527.15601 | 55.55447:5626 127.59034:5621 144.60855:5867 163.10468:6399 | 4.553E-07 |
| POS1158 | -Ethylbutanoic aci | 5.974 | 117.09066 | [M+H]+ | 117.0909 | 55.05365:17360 64.34301:5615 65.67461:6123 70.0643:125691 71.06874:10479 72.08047:30359 | -2.05E-06 |
| POS1657 | t-butyl propyl sulf | 1.18 | 133.10469 | [M+H]+ | 133.10451 | 57.05693:13463 58.06512:8432 61.01003:14805 69.06869:113703 70.07313:16056 86.09541:579528 87.02591:7053 87.09918:66588 90.05457:23341 | 1.352E-06 |
| POS3719 | Lactarazulene | 6.298 | 197.13197 | [M+Na]+ | 197.1322 | 68.17625:5403 80.64205:5329 100.70676:5930 127.82278:5754 136.63005:5533 161.47514:6497 167.6394:5486 | -1.17E-06 |
| POS5077 | 10,11,12,14,15,16,1 | 6.289 | 245.22614 | [2M+H]+ | 245.2263 | 57.05414:6045 59.16666:5253 67.25835:6028 85.66637:6272 113.07216:5864 125.9018:5442 132.26817:5515 214.60085:5742 | -6.52E-07 |
| POS13869 | vernonioside B3 | 4.562 | 675.37421 | [M+NH4]+ | 675.37378 | 59.04858:37087 69.03294:269041 73.02811:92499 73.06432:73920 81.03385:33829 83.0477:105986 85.02631:26682 87.04334:613385 89.05896:1780757 90.0619:30257 95.04893:48746 99.04339:436998 111.04441:239063 113.05927:40765 117.09089:47411 122.54804:27416 125.0596:104342 129.05446:51720 131.07088:62767 133.08543:703142 134.08684:34437 137.05981:51558 155.07083:146177 173.07771:30422 177.11285:80850 | 6.367E-07 |
| POS12824 | Malyngamide J | 4.571 | 608.3797 | [M+NH4]+ | 608.37927 | 73.06432:37063 87.04334:182952 89.05896:1542307 90.0619:39641 117.09089:19249 131.07088:54281 133.08543:670589 134.08684:26788 177.11285:88397 553.66919:19689 | 7.068E-07 |
| POS11942 | Hyperbrasilol A | 4.54 | 569.31042 | [M+Na]+ | 569.31079 | 243.29276:15178 | -6.5E-07 |
| POS10732 | etramethyl-12-oxo | 4.524 | 525.28528 | [M+Na]+ | 525.28497 | 104.10696:90124 116.99842:13251 184.06927:53077 | 5.902E-07 |
| NEG2882 | pholino)-ethanesu | 1.208 | 194.04869 | [M-H]- | 194.0484 | 59.08403:9421 79.05367:22876 80.05276:8272 87.29256:5457 97.07645:5085384 159.03728:26253 | 1.494E-06 |
| POS9325 | BW A868C | 4.53 | 460.28018 | [M+H]+ | 460.28049 | 70.35613:12729 71.03398:12288 72.08044:54452 82.99567:12845 84.07954:17718 86.09536:14624 87.0433:20802 89.05891:358132 90.06187:46690 120.08025:36316 133.08537:85632 173.42535:17198 457.95001:14539 | -6.74E-07 |
| POS12839 | ctyl)-1,4,7,10-tetr | 4.523 | 609.30444 | [M+H]+ | 609.30487 | 68.41991:7554 87.04333:35840 89.05895:261702 90.0619:17221 116.29226:8484 133.08543:93186 134.09016:11636 177.11284:16533 580.45636:8024 | -7.06E-07 |
| NEG2413 | ,2,7,8-Octanetetr | 0.924 | 177.11366 | [M-H]- | 177.1133 | 74.1126:10026 93.03925:30520 128.69928:6126 161.08009:5411 | 2.033E-06 |
| POS5264 | Ribose 5-phospha | 1.199 | 253.00798 | [M+H]+ | 253.00832 | 61.03971:9646 68.36646:5653 68.68076:5543 87.04333:5534 104.36064:5765 120.96402:9389 149.96265:5262 | -1.34E-06 |
| POS7110 | kasarin | 4.438 | 326.17065 | [M+NH4]+ | 326.17099 | 57.06894:28962 70.06554:5563 74.73689:6539 85.06334:9850 87.04332:8115 89.05893:97753 98.51205:5351 110.07072:30468 122.05131:6042 133.0854:19736 | -1.04E-06 |

| Differences in metabolites between the Model group and the Control group | | | | | | | |
|---|---|---|---|---|---|---|---|
| Alignment ID | Metabolite name | Rt(min) | Expreiment Mz | Adduct type | Reference m/z | MS/MS spectrum | PPM |
| POS8621 | EnP(5,8) | 5.227 | 417.33533 | [M+H-H2O]+ | 417.3356 | 57.03286:18918 59.04858:302286 73.02811:18571 81.06832:25269 87.04333:25195 89.05895:135926 99.07938:32106 101.0593:16746 103.07443:36156 107.08485:7418 109.10005:8718 130.55034:6076 133.08543:19907 147.10147:9836 159.40642:5982 173.43523:10533 196.30745:5690 416.92136:6788 | -6.47E-07 |
| POS4936 | Pirbuterol | 4.42 | 241.15355 | [M+H]+ | 241.1539 | 58.06511:13072 69.03294:8028 70.0643:11751 84.07957:7558 95.08477:10598 98.06007:17179 110.09556:5558 134.09351:8895 138.08907:38755 | -1.45E-06 |
| POS13628 | Parisin | 6.429 | 657.49316 | [M+H]+ | 657.49353 | 57.03286:138037 58.03661:6859 58.22031:5838 59.04858:35475 78.02471:7108 99.07938:15568 115.07377:8433 126.9121:6505 126.93359:6413 140.10425:5512 145.29819:5482 157.11888:14588 346.10397:5472 461.44281:6575 652.27557:5618 | -5.63E-07 |
| POS5044 | -11H-dibenzo[b,e | 0.877 | 245.0472 | [M+Na]+ | 245.0475 | 51.55165:7066 52.56477:6209 53.31757:6191 65.14406:6541 65.15309:6011 69.5333:5667 177.06224:34769 224.77242:5997 | -1.22E-06 |
| POS3846 | Lacinilene A | 6.296 | 201.12679 | [M+H-H2O]+ | 201.12711 | 64.187:5540 66.59401:6007 91.05389:9508 105.06944:6709 117.06911:7199 129.06705:13625 131.08376:29337 145.10278:14647 157.10196:18799 173.10216:6734 173.396:8191 | -1.59E-06 |
| POS9643 | ylphenylalanylarg | 6.193 | 478.28867 | [M+H]+ | 478.28839 | 54.62195:6208 62.57891:5577 134.27377:6357 397.29398:5879 | 5.854E-07 |
| POS1062 | i-Dimethylpiperidi | 4.488 | 114.12732 | [M+H]+ | 114.1276 | 53.03857:12167 54.03373:11727 55.0177:189439 55.05365:462522 58.02808:15578 67.05389:110907 68.04829:98581 69.06868:682557 70.06431:86380 71.04816:32870 71.08547:39319 72.08047:69497 77.03802:27084 79.05312:650159 81.05579:13126 84.07957:14653 86.09541:46098 95.04893:44642 96.08018:307102 97.06461:103540 105.04401:13777 114.09126:1768146 115.09234:18092 | -2.45E-06 |
| POS5387 | 3-Deoxyestradiol | 6.297 | 257.18881 | [M+NH4]+ | 257.18909 | 57.53942:7335 57.62482:5391 83.3492:5597 142.84343:5835 | -1.09E-06 |
| POS7211 | Sclareol | 6.5 | 331.26044 | [M+H]+ | 331.26071 | 55.05365:16712 57.06988:10107 65.03015:5403 67.05389:23387 69.06867:18376 79.05312:10309 81.06832:16159 83.08508:6287 91.05389:19068 93.0684:13151 95.08476:16426 105.06944:38936 119.08332:19632 121.10129:9555 131.08376:16476 133.10191:9633 145.10278:7045 155.82838:5614 | -8.15E-07 |
| POS2187 | -Tetrahydro-2-na | 6.3 | 149.09537 | [M+H]+ | 149.0957 | 99.37657:5830 103.77468:5501 | -2.21E-06 |
| POS1414 | SCHEMBL1137702 | 1.639 | 126.091 | M+CH3OH+H] | 126.0913 | 53.03856:20679 55.0177:13306 56.04952:7110 70.06556:11281 80.04813:21980 81.03228:8774 98.05797:15555 108.04319:12716 111.99284:5768 126.09013:7626 127.03809:12474 | -2.38E-06 |
| POS3840 | Matsutakic acid A | 9.749 | 201.11136 | [2M+H]+ | 201.11169 | 55.05365:227960 55.93435:129434 56.9414:111913 59.04858:14787 67.05389:7464 69.06991:6620 70.95692:13366 72.93706:49809 72.95981:12300 73.94402:8057 74.95245:24945 81.06989:9887 83.04769:43832 90.94762:32130 96.95995:11195 97.96836:93609 100.96772:13857 101.0593:69034 111.04189:43725 113.96311:482955 114.97044:68490 116.96038:12450 118.94112:14331 129.05446:14695 131.97466:90264 132.97011:21142 141.95662:110573 155.00035:7223 159.96982:69588 | -1.64E-06 |
| POS6849 | xo-nonadecanoic | 0.736 | 313.27216 | M+CH3OH+H] | 313.27289 | 57.06988:10981 57.52068:6191 124.39247:6272 161.19786:5075 | -2.33E-06 |
| POS7966 | conessine | 1.238 | 371.34229 | [M+H]+ | 371.34201 | 57.06989:9314 59.03105:42755 61.01003:14827 71.08548:6140 73.04555:31922 75.02498:34684 77.04093:20410 91.05577:23723 93.03564:23681 105.07177:9840 237.84406:5418 314.16376:6156 | 7.54E-07 |
| POS9711 | 2-ylanilino)propar | 4.506 | 481.26016 | [M+Na]+ | 481.25977 | 133.00633:5343 173.39601:10702 190.73463:6381 353.4512:6282 | 8.104E-07 |
| POS11926 | hyl-2-[6-(3-meth | 6.186 | 568.4364 | [M+H]+ | 568.43597 | 55.4754:85672 57.03287:352190 58.06511:125360 59.04858:93726 60.08025:1130365 67.05389:84753 69.93349:73463 71.0726:139182 81.06833:84518 86.0954:2759882 87.09918:96145 93.07034:89869 104.10696:12050610 105.10878:331944 109.23234:74300 124.99955:658444 175.14503:80713 184.07465:4111756 185.07634:120884 | 7.565E-07 |
| POS9324 | yptophylvalylargini | 5.752 | 460.2662 | [M+H-H2O]+ | 460.2666 | 59.04858:13547 79.6314:5536 89.05895:13544 119.08332:58896 135.07935:9482 152.16771:5365 173.38618:5401 | -8.69E-07 |
| POS3442 | 4-(pyridin-2-yl)bu | 4.12 | 188.06781 | [M+H]+ | 188.06822 | 55.05365:9874 83.91093:5305 113.4112:6295 131.41315:6112 | -2.18E-06 |
| POS6401 | droxyhexadecano | 5.946 | 295.2247 | [M+H]+ | 295.22433 | 59.04857:7603 67.05388:19576 69.06866:9868 79.05311:13119 81.06831:20876 83.08508:14072 91.05388:6351 93.06838:13704 95.08475:14161 107.08484:9073 121.0984:15495 135.11641:11977 165.01813:6026 258.12753:6279 258.19879:5911 275.03635:6959 | 1.253E-06 |

| Differences in metabolites between the Model group and the Control group | | | | | | | |
|---|---|---|---|---|---|---|---|
| Alignment ID | Metabolite name | Rt(min) | Expreiment Mz | Adduct type | Reference m/z | MS/MS spectrum | PPM |
| POS3075 | hyl-ribo-hexose-a | 4.594 | 177.11166 | [M+NH4]2+ | 177.11211 | 88.02121:34491 134.02684:9513 173.43524:6596 | -2.54E-06 |
| POS10583 | Tenacibactin C | 4.524 | 520.33051 | [M+NH4]+ | 520.33002 | 73.06432:28839 87.04334:132120 89.05896:1572119 90.0619:33301 91.07443:10618 104.10696:19802 107.06821:11937 117.08817:18443 131.06766:32650 133.08543:696134 134.08684:18707 144.3028:8212 173.3813:8593 175.09526:11748 177.11285:97387 197.86429:8397 | 9.417E-07 |
| POS14755 | PC(32:0) | 8.805 | 756.552 | [M+H]+ | 756.55133 | 60.08022:31450 67.54832:12374 73.45212:11172 86.09537:77100 94.06747:11228 101.42462:11288 101.90234:10882 104.10693:10989 111.52592:11379 124.9995:15745 184.06921:205498 222.45992:12398 253.30632:11679 542.04785:14291 | 8.856E-07 |
| POS5478 | 79966-13-5 | 0.759 | 261.00415 | [M+H]+ | 261.00372 | 55.08349:5608 61.03869:70612 62.50561:5753 162.99733:5917 | 1.647E-06 |
| POS13415 | Cyclosquamosin A | 4.367 | 642.32507 | [M+H]+ | 642.32452 | 78.48708:5382 110.16753:6001 118.88821:6000 120.0803:11004 129.1017:14690 284.55325:5294 340.97604:5526 | 8.563E-07 |
| POS1661 | minopropoxy)guar | 2.2 | 133.10878 | [M+H]+ | 133.10831 | 55.01769:9547 55.05364:22978 56.04951:32865 57.05691:117812 58.0651:67808 59.04856:11327 67.05387:14182 69.06989:1012020 70.0731:111514 73.0281:9696 73.0643:16475 86.09538:3664538 87.09916:413410 | 3.531E-06 |
| POS8648 | ptan-2-yl)-10,13-d | 5.825 | 419.35056 | [M+NH4]+ | 419.35101 | 51.32425:6511 53.51543:5580 57.03286:8346 59.04858:18139 59.19405:6231 61.50322:5552 69.06991:15310 81.06832:74198 93.07033:7041 95.08476:13841 99.07938:92604 107.08485:5748 109.10005:24396 118.48554:6358 121.10129:10560 127.11192:42138 128.49895:5709 135.11644:16540 147.11679:11189 149.13:8978 161.13196:9149 177.72186:5742 | -1.07E-06 |
| POS4255 | Oxododecanoic ac | 6.138 | 215.16316 | [M+H]+ | 215.1636 | 51.47779:6184 55.45854:6857 | -2.04E-06 |
| NEG1256 | -Butanesulfonic ac | 8.411 | 137.02744 | [M-H]- | 137.02789 | 93.14729:7280 114.20587:5520 114.93765:5388 | -3.28E-06 |
| POS6069 | Helipandurin | 1.193 | 283.0184 | [M+Na]+ | 283.01889 | 55.05365:23136 55.37881:6720 57.06896:22459 61.0387:36270 67.05389:8412 69.06868:26057 71.08547:8629 81.0699:10292 83.08509:15330 93.06841:6457 95.08477:9999 97.09953:15408 109.10006:8774 110.8261:5504 111.11732:6136 121.1013:6617 162.05843:6212 201.10139:6503 276.58087:4927 | -1.73E-06 |
| POS4658 | aureothricin | 1.204 | 231.02509 | [M+H]+ | 231.0256 | 51.60494:4860 52.34609:5040 52.40226:4955 53.57602:5516 55.51713:4676 59.27923:3988 59.67426:4210 61.0561:4726 61.12378:4139 61.50322:4601 64.35188:4912 70.01521:4499 71.72692:4475 71.8471:4437 79.13621:4928 82.53001:4615 86.48445:4756 90.88618:4138 91.6992:4865 100.39278:4164 101.92226:4489 102.37012:4400 103.79739:5268 105.51961:3989 106.1909:4007 106.89709:4513 119.68001:3814 125.68365:4764 141.86584:4666 158.9062:4620 159.09998:4177 159.43236:4009 161.65155:5065 162.84996:4407 168.66025:4075 180.60074:5156 215.51527:4641 | -2.21E-06 |
| POS6750 | Tripentyl phosphat | 6.215 | 309.21936 | [M+H]+ | 309.21887 | 55.05365:6885 59.04858:8074 67.05389:7179 70.24855:5875 72.41546:5413 81.06989:6388 97.10158:7561 138.85191:5487 173.396:6944 256.55826:5440 | 1.585E-06 |
| POS12620 | active peptide-1\|SC | 4.7 | 599.3504 | [M+H]+ | 599.35107 | 69.03294:38710 73.02811:181858 73.06432:104448 87.04334:467806 89.05896:2369354 90.0619:123719 91.04644:54510 95.04893:88861 99.04339:48918 102.06606:71614 111.04189:82690 113.05927:108419 117.09089:38395 131.06766:90357 133.08543:1302579 134.09018:66776 137.05981:60868 146.55127:41191 155.07083:76725 159.97418:38949 177.10779:196456 | -1.12E-06 |
| POS6633 | 4,5-dimethyl-2-ox | 4.384 | 304.17487 | M+CH3OH+H] | 304.17542 | 51.44688:6234 57.03287:16040 58.06511:8521 60.08025:219606 69.06868:93746 70.02779:12022 70.0643:6306 73.06432:25998 81.0699:7586 84.07957:23066 85.02799:547409 86.03196:12596 86.09541:67413 97.06461:74051 103.03849:8438 115.07378:62989 125.0596:302680 126.06278:8929 132.10173:10240 132.18655:6242 143.06735:108340 144.10197:24769 161.07927:24418 227.09167:8346 | -1.81E-06 |
| POS2500 | Allantoin | 0.763 | 159.04947 | [M+H]+ | 159.05 | 52.15548:6852 55.93345:47177 56.94233:12875 68.98124:35509 69.98251:6275 72.93707:13179 73.06432:23608 86.99105:20833 105.00241:13277 108.27512:5459 113.96312:6903 131.01933:8361 | -3.33E-06 |
| POS4068 | phenyl)-5-(Methyl | 1.583 | 209.05473 | [M+H]+ | 209.0542 | 77.12376:5258 102.64651:6055 162.43123:5468 180.68938:5230 | 2.535E-06 |
| POS3019 | densispicnin D | 5.978 | 175.13226 | [M+H]+ | 175.1328 | 54.46105:5937 59.02229:5374 59.04859:8480 69.72547:5990 133.93025:5405 146.03825:5677 157.05971:6021 165.42868:5228 173.43034:8800 | -3.08E-06 |

| Differences in metabolites between the Model group and the Control group | | | | | | | |
|---|---|---|---|---|---|---|---|
| Alignment ID | Metabolite name | Rt(min) | Expreiment Mz | Adduct type | Reference m/z | MS/MS spectrum | PPM |
| POS10145 | 10,13-dimethylhex | 4.565 | 501.2886 | [M+2H]2+ | 501.28799 | 67.05389:10180 69.03294:24014 73.02811:78849 73.06432:11997 81.03229:36478 83.0477:31797 87.04334:139810 89.05896:695122 90.0619:36670 91.04643:15830 95.04893:52681 99.04339:36235 111.04441:88439 113.05927:24005 129.05132:9701 133.08543:208249 134.09018:9543 137.05981:22338 155.07083:22753 177.10779:13785 349.62927:8779 394.30075:8815 | 1.217E-06 |
| POS13576 | Gnididilatin | 4.539 | 653.33099 | [M+Na]+ | 653.33191 | 73.06432:16337 87.04335:81017 88.42162:21583 89.05896:508404 90.06375:26002 131.07088:22260 133.08545:261973 134.09018:19703 167.93817:14192 173.32739:16029 177.11285:33825 207.07423:17439 | -1.41E-06 |
| NEG6305 | ACMC-20m5sy | 0.912 | 313.25443 | [M-H2O-H]- | 313.25381 | 93.03925:95947 94.70243:6657 95.03764:22058 97.03746:11298 103.07947:53240 124.15677:15567 131.55391:5587 170.74947:6080 177.1145:22416 | 1.979E-06 |
| POS4414 | eta-glucosylbutan | 4.6 | 221.1377 | [M+2H]2+ | 221.13831 | 55.05365:18955 58.04042:7238 61.01003:10168 73.02811:12944 81.0699:6347 89.05896:7078 97.09953:9690 125.09565:8844 173.39111:11760 175.25459:6089 193.96582:5796 | -2.76E-06 |
| POS7123 | riphenyl Phosphat | 6.096 | 327.07718 | [M+H]+ | 327.07779 | 57.78203:5585 95.04893:13692 115.42985:5442 121.75073:5463 131.258:5125 153.06662:12314 | -1.86E-06 |
| POS6316 | Epiandrosterone | 6.166 | 291.21527 | [M+H]+ | 291.21469 | 57.03287:30544 57.06989:8249 59.04858:14080 85.48107:5604 89.87497:5691 | 1.992E-06 |
| POS10231 | Artoindonesianin L | 4.39 | 505.2211 | [M+H]+ | 505.22171 | 60.04426:13514 70.06556:5731 72.08047:15701 72.84618:5567 84.08123:5355 94.80859:6167 118.08494:8652 129.10172:8733 129.49957:5232 175.10521:10225 329.03448:6374 | -1.21E-06 |
| POS6314 | Epiandrosterone | 5.973 | 291.21527 | [M+2H]2+ | 291.21469 | 57.03287:13229 57.06989:11761 59.04858:15163 94.53361:5705 99.57687:5565 104.10696:6784 208.52167:5827 | 1.992E-06 |
| POS8523 | Risperidone | 4.471 | 411.22064 | [M+H]+ | 411.22 | 55.05365:17699 59.04858:11397 67.05389:5960 69.03294:127237 73.02811:24347 81.03229:11864 83.0477:60021 87.04334:171809 89.05896:220269 95.04893:19416 99.04339:81065 107.07059:11021 111.04189:87854 113.05927:8752 125.0596:10837 129.05446:12660 131.06766:18400 133.08543:57860 137.05981:5980 151.09705:10247 155.07083:21651 175.09526:6611 293.59796:5616 316.06183:5958 | 1.556E-06 |
| POS14888 | HMS2873N16 | 4.419 | 770.33722 | [M+2H]2+ | 770.3382 | 72.08047:177076 86.09541:69139 118.08495:95567 129.09856:75262 305.85339:67544 | -1.27E-06 |
| POS10614 | 16-Trihydroxygray | 4.36 | 521.2713 | [M+2H]2+ | 521.27197 | 60.04325:56165 84.04481:14779 84.07957:24844 86.0954:20948 86.4154:9828 102.05276:23592 120.0803:36031 126.05366:11459 129.1017:54961 154.04741:11480 189.0878:20782 217.07977:11623 269.75531:9991 | -1.29E-06 |
| POS14376 | Mycolactone E | 6.036 | 715.5152 | [2M+H]+ | 715.51428 | 54.48004:11175 57.03287:49740 59.04858:412194 115.07642:12631 117.09089:66733 173.3911:19235 191.97964:12295 | 1.286E-06 |
| POS5519 | ydroxyvalerylcarni | 3.958 | 262.16397 | [M+H]+ | 262.16461 | 53.30585:6434 55.40891:6406 59.04858:11721 60.08024:13834 61.03869:45354 76.82643:5531 85.02798:102204 110.94395:5711 111.10222:5649 132.5201:5358 196.49069:5692 228.23517:5695 | -2.44E-06 |
| NEG6724 | Hydroxyprogester | 0.904 | 329.25793 | [M-H]- | 329.25729 | 81.04115:6856 93.03925:49700 95.03764:24921 103.08172:13697 119.0826:16267 126.0732:6496 177.1145:11728 314.51971:6203 | 1.944E-06 |
| POS14103 | coumaroyltetracos | 6.817 | 693.47156 | [M+H]+ | 693.47241 | 62.44623:4860 69.6243:6494 87.19528:6082 184.61758:5548 207.8765:6022 248.67805:5908 | -1.23E-06 |
| POS9109 | yr-pro-leu-gly-NH | 4.624 | 448.25616 | [M+2H]2+ | 448.25549 | 55.05365:43824 59.04858:50330 69.03294:750078 70.0366:21563 71.04816:16042 73.02811:185668 73.06432:84127 78.03804:19506 80.05429:70324 81.03229:24991 83.0477:139718 87.04334:621042 88.04782:23611 89.05896:2186922 90.0619:75419 91.04643:18226 95.04893:47437 99.04339:351123 100.05018:82056 102.06606:48598 107.07058:15750 109.06336:20714 111.04441:180290 113.05927:45693 117.09089:32455 122.06293:132622 122.56553:19196 125.0596:31456 129.05446:73320 131.07088:64470 133.08543:657783 134.08684:34734 137.0598:30580 144.07597:43365 155.07083:73250 166.08865:26780 173.42543:19873 177.11285:46147 | 1.495E-06 |
| POS15254 | 15Z)/22:6(4Z,7Z,10 | 8.757 | 828.55261 | [M+H]+ | 828.55371 | 64.80882:5376 90.13166:5540 90.14452:6178 | -1.33E-06 |
| POS5913 | 13-OxoODE | 6.323 | 277.21069 | [M+H]+ | 277.20999 | 51.60017:5818 55.05365:14852 57.06895:8654 67.05389:11565 68.40289:5850 69.06991:7502 81.06989:8632 91.05389:7189 91.21084:5115 93.0684:13701 120.82984:5609 259.21762:5271 | 2.525E-06 |

| Differences in metabolites between the Model group and the Control group | | | | | | | |
|---|---|---|---|---|---|---|---|
| Alignment ID | Metabolite name | Rt(min) | Expreiment Mz | Adduct type | Reference m/z | MS/MS spectrum | PPM |
| POS10667 | schiprolactone A | 4.747 | 523.30438 | [M+H]+ | 523.30359 | 60.08025:21171 86.09541:53744 87.04335:5583 87.49507:7251 89.05896:20147 104.10697:262831 133.08545:6003 142.02203:6853 184.07466:122621 | 1.51E-06 |
| POS2234 | enzisothiazol-3(2H | 0.764 | 151.00845 | [M+Na]+ | 151.00919 | 56.74443:6504 67.05389:7281 68.98123:155552 82.01235:59734 86.99277:138376 100.02438:116854 105.0024:137716 110.00877:12313 118.03259:28004 123.0126:14057 128.01854:49666 | -4.9E-06 |
| POS3883 | 3-benzodioxole-4 | 1.008 | 203.01422 | M+CH3OH+H] | 203.015 | 57.03287:28735 60.08025:167617 62.98059:44775 70.0643:127962 71.04816:18884 71.05974:54954 82.94377:27979 84.07957:14710 85.02799:894229 87.97689:213884 88.0851:27716 100.07382:27565 105.0024:76477 116.06905:23099 117.00114:14605 135.00183:10803 142.98286:19252 144.10197:18137 145.04651:16065 158.12614:11853 | -3.84E-06 |
| POS8839 | Istamycin C1 | 4.491 | 432.27838 | [M+NH4]+ | 432.27921 | 73.06432:19208 87.04334:58890 89.05896:1171961 90.0619:26224 107.06821:28428 117.08817:8395 130.08389:15416 131.07088:13409 133.08543:480616 134.09018:13108 177.11285:54497 | -1.92E-06 |
| POS11841 | Aristophyll B | 4.506 | 565.2818 | [M+H]+ | 565.28088 | 66.95612:21347 67.26903:21225 87.04334:58740 89.05896:578913 90.0619:37662 98.06215:17836 131.07088:17316 133.08543:227625 140.56842:15987 166.01514:16742 167.74202:14961 173.39111:26395 177.11285:21075 | 1.628E-06 |
| POS6605 | -hydroxy-6-methy | 0.755 | 303.02576 | [M+H]+ | 303.02655 | 53.82195:5647 61.97727:6437 128.94746:5700 132.68735:5070 173.69058:6055 218.98808:8075 236.06581:6082 | -2.61E-06 |
| POS4180 | thyl-1H-benzimid | 0.758 | 213.02388 | [M+Na]+ | 213.0247 | 86.99277:10620 94.43892:6952 105.0024:12319 180.54861:5459 185.02766:11004 | -3.85E-06 |
| POS10242 | Eudesobovatol A | 5.401 | 505.33035 | [M+Na]+ | 505.33121 | 59.04858:22005 60.51869:6391 67.54954:5982 87.04334:9228 89.05896:22581 133.08543:7357 370.6825:5931 | -1.7E-06 |
| POS6837 | nethyllumazine he | 0.878 | 313.03406 | M+CH3OH+H] | 313.0332 | 51.59301:5858 57.06989:7570 64.0778:5520 86.3585:5997 95.34216:5905 145.91704:6573 177.06224:94869 | 2.747E-06 |
| POS4740 | SCHEMBL4748090 | 6.31 | 233.17375 | [M+H]+ | 233.17461 | 50.02441:5649 51.9667:4592 55.5687:5839 57.06989:4761 58.42694:4201 63.52836:5329 65.09892:4186 69.69672:3886 70.41069:4354 71.44855:4437 73.02811:6086 73.10591:3917 73.56042:4965 75.60188:5517 79.3559:7422 82.78501:4928 83.87628:4915 89.71416:4409 92.87972:5020 104.80626:4991 111.92415:5047 123.71887:4982 125.3816:5402 130.29124:4211 136.30479:4052 140.81929:4339 144.29163:4038 147.57776:4511 151.09703:3968 165.68486:3732 179.49561:5680 195.94174:4385 196.09497:4181 210.6328:4450 216.47656:4519 218.19684:4031 230.56325:4532 | -3.69E-06 |
| POS3864 | Pantothenic acid | 4.394 | 202.10612 | [M+H-H2O]+ | 202.10699 | 55.05365:27903 66.91496:6640 67.05389:5866 69.06868:5980 70.02779:6834 70.0643:12287 73.02811:6237 86.09541:9462 88.03895:19339 98.02254:6027 101.0593:7952 104.60148:5565 130.06477:6753 178.33917:5429 | -4.3E-06 |
| POS8122 | JWH-370 | 4.589 | 382.21741 | [M+H]2+ | 382.21649 | 55.05365:38762 58.04137:25107 59.04858:48721 61.02743:24303 67.05389:16343 69.03294:728952 70.0366:20642 71.04816:21413 73.02811:188386 73.06432:52727 78.03804:44417 80.05429:38128 81.03229:25991 81.0699:16770 83.0477:123278 85.02799:19799 87.04334:542064 88.04604:20426 89.05896:1675804 90.0619:53117 91.04643:9790 95.04893:47874 97.06461:10190 99.04339:349853 100.05018:91888 102.06606:26695 103.03849:10777 107.07058:23191 109.06336:18528 111.04189:154115 113.05927:40171 117.09089:15015 122.06293:113519 122.56553:9929 125.0596:38116 127.075:14979 129.05446:73354 131.07088:42454 133.08543:414738 134.09018:15588 137.0598:18181 144.07597:47260 155.07083:57110 166.08865:15737 173.07771:18284 177.11285:28229 | 2.407E-06 |
| POS34 | FLUOROACETONE | 4.383 | 77.03867 | [M+H-H2O]+ | 77.0396 | 53.1757:4496 53.45075:5253 53.69243:4379 54.90918:5249 55.54824:5066 55.82938:5177 60.23557:4648 65.02227:4910 66.54385:3871 67.13178:3795 68.11464:4557 69.44248:4199 69.63427:4563 69.75673:4803 70.04163:4254 71.10993:3927 71.54332:4523 71.62527:4201 72.18309:3845 75.41446:4233 78.97468:5244 79.0169:4167 | -1.21E-05 |
| POS8848 | Glu Arg Glu | 4.46 | 433.20316 | M+CH3OH+H] | 433.2041 | 62.05909:7454 85.02799:8503 87.04334:27083 89.05896:501474 90.0619:43329 110.23711:5445 127.03809:6320 131.06766:6017 133.08543:196828 134.08684:25018 173.43524:12361 177.11285:23861 | -2.17E-06 |
| NEG3943 | dehydroschoberine | 0.757 | 231.18767 | [M-H]- | 231.18671 | 67.90976:5778 97.15044:6255 127.79837:6022 | 4.152E-06 |

| Differences in metabolites between the Model group and the Control group | | | | | | | |
|---|---|---|---|---|---|---|---|
| Alignment ID | Metabolite name | Rt(min) | Expreiment Mz | Adduct type | Reference m/z | MS/MS spectrum | PPM |
| POS13601 | Calenduloside E | 4.625 | 655.38031 | [M+2H]2+ | 655.38159 | 73.02811:264880 87.04334:687350 89.05896:3447120 113.05927:150994 133.08543:1321092 | -1.95E-06 |
| POS5643 | no]-1,3,9-trimethy | 3.201 | 267.13202 | [M+H]+ | 267.133 | 67.05389:7067 78.88884:6173 84.07957:24893 94.57704:6600 95.29619:6594 96.04378:8462 136.06245:11875 | -3.67E-06 |
| POS6938 | -3-Hydroxypregn- | 6.683 | 317.24579 | M+CH3OH+H] | 317.2468 | 67.05389:12161 69.06992:5708 105.06946:10786 119.08613:8711 131.08377:6719 164.12044:5913 173.39111:8186 | -3.18E-06 |
| POS8856 | Salicyloyl-conkurc | 7.323 | 433.28387 | M+CH3OH+H] | 433.28491 | 53.10416:5657 59.07783:5143 62.33404:5331 94.48625:6398 152.66074:7117 | -2.4E-06 |
| POS5602 | 6R)-2-hexoxy-6-( | 4.611 | 265.16293 | [M+2H]2+ | 265.164 | 53.46587:6831 67.6092:6033 72.95446:5688 73.02811:6498 173.43034:11133 | -4.04E-06 |
| POS7128 | ecylbenzenesulfon | 4.648 | 327.19986 | [M+H]+ | 327.19879 | 58.09455:6387 60.16131:5746 73.35352:6188 89.05891:10389 106.76425:5645 | 3.27E-06 |
| POS9231 | Tenovin-6 | 4.488 | 455.24631 | [M+H]+ | 455.24741 | 55.05365:22859 59.04858:15265 69.03294:202679 73.02811:51304 73.06432:14771 81.03229:23595 83.0477:123453 85.02799:13985 87.04334:346734 89.05896:690125 90.0619:9517 95.04893:21427 99.04339:216063 101.0593:10555 103.03849:11945 107.07059:18161 111.04189:187639 113.05927:21910 125.0596:55597 129.05446:26236 131.06766:37816 133.08543:222312 137.05637:19134 151.09305:16517 155.07083:70916 173.0826:12847 195.11966:10895 | -2.42E-06 |
| POS6951 | uberoyl-L-carnitin | 4.365 | 318.18979 | [M+H]+ | 318.19089 | 55.05365:11102 57.03379:10806 60.08025:67546 69.06992:58223 70.0643:14150 71.04816:38446 72.08047:9082 83.08509:22174 84.07957:28562 85.02799:129011 86.09541:91104 91.0539:9058 93.06841:10767 95.08477:6639 97.06461:25073 102.05499:9713 110.07076:8873 111.0796:20090 115.07378:49827 116.06905:8732 136.07611:13535 139.07358:22015 144.10197:9751 157.08505:22809 175.09526:15938 | -3.46E-06 |
| POS15179 | y-16,22,23-trihyd | 4.655 | 809.46619 | [M+2H]2+ | 809.46802 | 73.02811:130332 73.06432:64109 87.04334:322702 89.05896:1307563 131.06766:73103 133.08543:593855 177.895:58002 | -2.26E-06 |
| POS14414 | 2'-O-Acetylactein | 4.578 | 719.40167 | [M+NH4]+ | 719.40002 | 64.37184:55564 67.51974:55907 69.03294:237349 73.02811:120073 73.06297:67230 83.0477:116527 87.04334:450720 89.05896:1374356 95.04893:53932 99.04339:341638 111.04189:201892 122.10059:61744 125.0596:63125 133.08543:561076 150.88187:48487 155.07083:115493 174.20796:58532 362.51266:54235 | 2.294E-06 |
| POS11018 | Phe Leu Asp Lys | 4.711 | 536.30658 | [M+2H]2+ | 536.3078 | 68.92713:6230 69.03295:5334 78.79263:5559 79.1211:5431 87.04335:8284 89.05897:32309 117.82632:5640 133.08545:6548 149.22003:5394 173.38622:8250 303.48926:5258 444.81195:5902 473.62137:6605 | -2.27E-06 |
| POS5181 | Negamycin | 1.131 | 249.15454 | [M+H]+ | 249.15569 | 58.06512:12974 59.07198:13599 79.71541:6195 85.02799:11904 104.24855:6213 118.08494:229739 132.07565:5343 144.95276:6103 | -4.62E-06 |
| POS7558 | Dodecyl glucoside | 6.14 | 349.2569 | [M+H]+ | 349.25809 | 57.03287:18914 59.04858:7832 90.3176:5956 93.2132:5617 165.29622:6592 347.04623:6512 | -3.41E-06 |
| POS5607 | 4-ethylphenyl)-1 | 6.078 | 265.19382 | [M+H-H2O]+ | 265.19501 | 73.36568:5988 173.38618:9266 263.33527:5885 | -4.49E-06 |
| POS4934 | enyl-3-(Pyridin-2- | 4.579 | 241.13478 | [M+H-H2O]2+ | 241.13358 | 55.05365:10048 57.06896:33395 58.06416:1074768 59.06808:27011 67.05389:24511 69.06868:13924 79.05312:12429 81.0699:142001 82.07301:10510 84.07957:53294 95.08477:23404 123.11523:84284 140.14345:14921 | 4.976E-06 |
| POS5160 | -hydroxybutyrylcar | 2.256 | 248.1478 | [M+H]+ | 248.149 | 59.04858:11134 60.08024:14690 72.08047:40565 74.22613:5953 84.04315:15212 85.02799:85597 103.03848:12730 | -4.84E-06 |
| POS7921 | Cholesterol | 0.727 | 369.34979 | [2M+H]+ | 369.35101 | 51.55879:6271 57.06894:6732 67.05387:10748 69.06989:6279 71.08417:7706 81.06831:12099 93.0703:6586 95.08474:9880 107.08483:9799 109.10003:11523 115.62182:6104 135.11641:11067 147.11678:9908 161.13193:8112 | -3.3E-06 |
| POS7422 | Daphnilactone B | 6.078 | 342.24094 | [M+NH4]+ | 342.24219 | 57.06895:7702 57.4271:5912 58.06511:6050 59.05637:5598 67.05389:12496 75.02496:7322 81.06832:7854 84.07957:9365 91.05389:6026 105.06944:6899 107.2755:5846 119.08332:7047 122.09478:8318 131.08376:7998 134.09349:6883 147.11679:6070 171.90515:5738 179.28917:5348 217.66479:5314 | -3.65E-06 |
| POS11944 | Antibiotic WF 3161 | 4.682 | 569.33484 | [M+H]+ | 569.33337 | 69.03294:49433 73.02811:178498 73.06432:57693 80.05429:70046 87.04334:376107 89.05896:1927869 90.0619:74864 91.04643:51479 95.04893:55157 99.04339:59499 111.04441:55463 113.05927:52859 124.08022:31021 131.06766:53555 133.08543:710807 134.09018:65579 155.07083:38486 177.11285:84402 | 2.582E-06 |

| Differences in metabolites between the Model group and the Control group | | | | | | | |
|---|---|---|---|---|---|---|---|
| Alignment ID | Metabolite name | Rt(min) | Expreiment Mz | Adduct type | Reference m/z | MS/MS spectrum | PPM |
| POS3540 | 2H-3,4,5,6-tetrahy | 0.898 | 191.07605 | [M+Na]+ | 191.07739 | 60.08025:22959 61.08378:9675 62.98166:18938 68.05673:6062 72.67956:6599 72.68887:6035 84.07957:166945 85.08359:71411 90.58549:5456 130.08389:15437 131.09021:12874 173.02882:6747 191.0405:10460 191.07452:13864 | -7.01E-06 |
| POS6082 | 17_epimethanedi | 6.075 | 283.20364 | [M+H-H2O]+ | 283.20499 | 53.84824:5745 55.05365:19561 57.06895:16933 67.05389:6858 69.06867:20806 71.08546:11932 79.05312:6546 81.06989:8545 95.08476:9856 105.06944:6845 106.34853:5736 107.08485:6755 171.11885:7386 181.09694:6200 183.11298:8004 188.50836:6093 203.82985:5841 264.63409:5675 | -4.77E-06 |
| POS6239 | Asn Val Gly | 8.817 | 289.1492 | [M+NH4]+ | 289.1506 | 56.96448:7101 57.06989:10926 86.0954:11678 95.08476:7188 104.10696:10945 124.99955:7374 138.52921:7631 185.85204:6748 | -4.84E-06 |
| NEG7454 | lupanacosmine | 1.079 | 356.27225 | [M-H]- | 356.27081 | 93.03925:573915 95.03763:34431 97.03745:8066 102.77911:6475 124.15676:56260 134.97455:6595 151.06711:23835 182.18414:14521 | 4.042E-06 |
| NEG6505 | Aprindine | 6.288 | 321.23227 | [M-H]- | 321.2337 | 53.33165:5608 57.10276:13443 59.08403:341658 60.08905:44248 61.0902:19132 62.78073:6033 67.13445:11165 69.11722:49064 70.1209:15373 71.09898:13863 79.05216:7284 81.16814:10027 83.15008:13115 91.63754:6215 107.2141:39095 108.21955:32842 135.278:45323 136.28323:51715 139.27885:17981 140.28273:17285 153.31172:7172 154.31902:8632 163.30577:17887 164.31381:25905 179.32059:50916 180.32471:76803 181.33206:20822 204.42909:8547 258.54407:7895 280.51889:5748 | -4.45E-06 |
| NEG4258 | -Undecanoylglyci | 9.352 | 242.17418 | [M-H]- | 242.17561 | 64.039:6632 90.04863:14065 | -5.9E-06 |
| POS15252 | Apramide G | 8.762 | 828.54401 | [M+H]+ | 828.5415 | 70.83127:5762 179.4697:5893 213.31702:6425 | 3.029E-06 |
| NEG4801 | SCHEMBL1426937 | 0.906 | 261.18762 | [M-H]- | 261.1861 | 79.05368:19559 93.03925:9310 103.07947:17718 107.06415:5280 118.93213:5943 119.0854:144122 177.1145:7846 190.98895:5499 | 5.82E-06 |
| POS9532 | Malyngamide O | 4.654 | 472.284 | [M+2H]2+ | 472.28241 | 58.04042:41917 69.03294:21966 71.04945:8981 73.02811:171275 73.06432:28295 80.05429:121145 87.04334:97696 89.05896:589103 90.0619:27539 95.04694:10836 102.06606:87038 107.07058:9726 111.06954:10968 117.08817:12382 124.08022:35440 133.08543:219018 134.08684:9092 146.09132:15891 155.09987:16375 173.43524:19122 177.11285:29597 | 3.367E-06 |
| NEG7048 | Galactinol Dihydrat | 0.785 | 341.3584 | [M-H]- | 341.35999 | 80.71797:5797 119.32285:5836 196.84229:5703 | -4.66E-06 |
| POS4324 | mycin monophos | 0.779 | 218.98331 | M+CH3OH+H] | 218.9817 | 51.71658:6445 61.03971:6312 68.98123:7856 73.06431:9481 86.99277:9904 98.97779:5810 101.02003:6761 107.0016:6625 119.0303:12751 121.0269:7406 131.01932:19292 145.04274:6936 149.02837:6261 159.04826:6278 162.99287:6641 190.98946:7110 194.38419:6124 | 7.352E-06 |
| POS10090 | Halisulfate 10 | 4.503 | 499.27396 | [M+Na]+ | 499.27231 | 55.05365:28736 59.04858:18016 69.03294:236843 73.02811:68322 73.06432:23736 81.03229:38010 83.0477:126886 85.02799:18303 87.04334:463670 89.05896:1095471 90.0619:19337 95.04893:42461 99.04339:338329 101.0593:13923 107.06821:35729 111.04189:214311 113.05927:17531 125.0596:71784 129.05446:33904 131.07088:42278 133.08543:348351 137.05981:27478 151.09305:16868 155.07083:97815 173.07771:20199 173.39111:12653 175.09526:13257 177.11285:24897 383.6424:11174 | 3.305E-06 |
| POS7000 | tetrahydroisoquin | 8.805 | 320.1662 | [M+H]2+ | 320.16449 | 57.06989:10949 71.08547:6578 76.01012:5352 86.0954:5684 115.63253:6699 | 5.341E-06 |
| NEG4423 | dopargine | 1.353 | 248.13878 | [M-H]- | 248.1405 | 51.94239:6965 89.13124:21490 97.07645:7956 103.6411:5306 180.61613:5629 | -6.93E-06 |
| POS8439 | yptophylalanyllysi | 4.601 | 404.23093 | [M+H]2+ | 404.22919 | 55.05365:69420 59.04858:82537 61.02743:37435 67.05389:27394 69.03294:1097856 70.0366:35509 71.04816:27370 73.02811:261407 73.06432:105466 78.03804:48174 80.05429:78280 81.03229:38017 81.0699:20906 83.0477:194623 85.02799:23581 87.04334:900676 88.04604:23622 89.05896:2799754 90.0619:89847 95.04893:56691 99.04339:524463 100.05018:130192 102.06606:47082 107.07058:27984 109.06336:26111 111.04189:254387 112.04632:16700 113.05927:72295 117.09089:31407 122.06293:201671 125.0596:59454 129.05446:97910 131.06766:64887 133.08543:726914 134.08684:40704 137.0598:32659 144.07597:79409 144.57867:17736 155.07083:88208 166.08865:22650 173.07771:23871 175.09526:27320 177.10779:45895 | 4.304E-06 |
| POS6131 | Tropisetron | 4.592 | 285.16193 | [2M+H]2+ | 285.16019 | 69.03294:11454 69.72296:6349 89.05896:7488 138.91872:5431 | 6.102E-06 |

**Differences in metabolites between the Model group and the Control group**

| Alignment ID | Metabolite name | Rt(min) | Expreiment Mz | Adduct type | Reference m/z | MS/MS spectrum | PPM |
|---|---|---|---|---|---|---|---|
| POS5659 | etramethyl-1,2-et | 6.296 | 267.20895 | [M+H-H2O]+ | 267.21069 | 55.05365:7454 67.05389:12529 122.52764:5775 141.06725:13587 143.08205:5119 155.08327:6205 159.31569:5686 177.96635:6350 183.1183:7326 | -6.51E-06 |
| POS1262 | Hydroxyphenyletha | 1.133 | 121.07175 | [2M+H]+ | 121.07 | 56.04862:105910 57.03379:9938 60.90679:11689 61.0387:12084809 61.17308:10012 62.04229:11768 72.08047:9141 74.05886:38460 | 1.445E-05 |
| POS14170 | exanoyloxy-6-[(2S | 4.637 | 699.4054 | [M+2H]2+ | 699.4079 | 69.03294:61248 73.02811:190035 87.04334:549712 89.05896:2719911 91.04457:62847 95.04893:142404 111.04441:137877 113.05927:90196 131.06766:69592 133.08543:1072420 134.09018:86522 137.0598:90294 177.11285:104394 539.42859:63564 | -3.57E-06 |
| NEG4443 | Cadabicilone | 1.386 | 249.14725 | [M-H]- | 249.14906 | 50.33427:6968 51.7285:5831 56.11635:5995 89.12944:25875 126.02459:17056 126.341:5377 134.5883:4972 169.09492:6739 | -7.26E-06 |
| POS8459 | Dihydroroseoside | 4.626 | 406.24582 | [M+2H]2+ | 406.24399 | 58.04042:316049 69.03294:26840 71.04816:23644 73.02811:815241 73.06432:76618 74.03149:19535 80.05429:592291 80.55504:35823 87.04334:254484 89.05896:2323191 90.0619:75913 102.06606:456392 102.56839:31231 111.07206:34913 117.09089:24991 124.08022:170757 124.58041:21333 131.07088:32343 133.08543:732108 134.09018:31369 146.09132:51431 155.09572:46962 177.11285:66796 | 4.505E-06 |
| NEG3825 | Pentadecanol | 0.798 | 227.23993 | [M-H]- | 227.2381 | 71.08354:41522 87.09683:59320 88.09991:9791 103.11093:93596 104.11378:13337 115.1256:84508 116.12636:9919 131.14032:348660 132.14209:51706 159.17082:77201 160.17122:17594 177.02853:5517 | 8.053E-06 |
| NEG3179 | -coumaroyl-diketi | 1.208 | 204.04483 | [M-H]- | 204.043 | 79.01295:7636 89.12944:8651 93.03925:39450 124.02621:7196 148.73897:5526 167.03258:17470 | 8.969E-06 |
| NEG3583 | butylphosphine ox | 1.322 | 217.17096 | [M-H]- | 217.17281 | 65.3876:5879 79.05367:11310 97.07645:2343227 98.0778:15686 167.79976:6017 | -8.52E-06 |
| POS2377 | e-desisopropyl-2- | 0.91 | 155.07883 | [M+H]+ | 155.0807 | 50.22104:6700 56.08018:6744 60.34113:6326 76.60997:7373 83.05907:17279 93.04334:12960 110.07076:74578 123.78096:5352 137.58165:5961 155.68228:5672 | -1.21E-05 |
| NEG2933 | noethyl methyl ph | 1.223 | 196.04738 | [M-H]- | 196.0493 | 56.43259:5803 59.08403:5648 79.05368:6140 81.55566:6182 97.07645:1401613 98.0778:45117 99.07355:179528 117.06951:30539 124.15677:12949 153.07974:7956 159.03728:9905 161.03622:529928 162.03658:8897 196.04443:52826 197.04991:6995 | -9.79E-06 |
| POS13082 | hydroxy-12-olean | 5.72 | 621.4173 | [M+Na]+ | 621.4151 | 55.77567:8051 59.04859:32506 73.02812:8587 87.04335:15535 89.05896:51955 90.06191:5907 100.78278:7247 101.05931:14746 103.07444:13967 133.08545:20985 147.10149:8119 173.39111:10285 314.7146:6119 320.94473:5878 322.51874:6938 | 3.54E-06 |
| POS7809 | Bacithrocin C 2 | 4.601 | 362.22061 | [M+NH4]2+ | 362.2186 | 58.04042:568294 69.03294:18110 71.04816:38377 73.02811:1280998 73.06432:84323 74.03149:32832 80.05429:736250 80.55659:42528 87.04334:300222 88.04782:18518 89.05896:3087766 90.0619:93126 102.06606:482204 102.56839:44585 111.07206:35091 115.07378:16474 117.09089:20523 124.08022:192208 124.58041:18554 131.07088:23712 133.08543:780054 134.08684:40726 146.09132:36484 155.09987:25200 177.11285:65140 | 5.549E-06 |
| POS9506 | Ser His Val Lys | 4.633 | 470.27005 | [M+2H]2+ | 470.27209 | 59.04858:28327 61.02743:19094 69.03294:486273 70.0366:11048 71.04816:10393 73.02811:108277 73.06432:64282 78.03804:13932 80.05429:47826 81.03229:17258 83.0477:87647 87.04334:417368 88.04782:14229 89.05896:1414898 90.0619:50529 95.04893:30424 99.04339:216102 100.05018:36082 102.06606:30560 107.07058:14999 109.06336:9811 111.04189:108709 113.05927:36454 117.09089:16338 122.06293:73095 125.0596:25053 129.05446:37538 131.06766:34850 133.08543:424666 134.09018:26651 137.0598:14985 144.07597:36262 155.07083:45272 166.09326:19870 173.07771:12719 177.11285:37390 | -4.34E-06 |
| POS13627 | ene\|21beta-angelo | 5.881 | 657.4751 | [M+H]+ | 657.47241 | 57.03287:56679 59.04858:643011 59.42949:8559 89.05895:18392 115.07378:17871 117.09089:104816 175.13507:13326 | 4.091E-06 |

**Differences in metabolites between the Model group and the Control group**

| Alignment ID | Metabolite name | Rt(min) | Expreiment Mz | Adduct type | Reference m/z | MS/MS spectrum | PPM |
|---|---|---|---|---|---|---|---|
| POS8156 | CHEMBL564302 | 4.614 | 384.23416 | [M+H]2+ | 384.23209 | 58.04136:306083 69.03294:18685 71.04816:28947 73.02811:754821 73.06432:61647 74.03149:13895 80.05429:564688 80.55503:36945 87.04334:225034 89.05895:2058075 90.0619:68475 102.06605:414678 102.56838:31943 111.07205:32339 117.09089:20394 124.08022:162777 131.07086:21136 133.08543:718377 134.09016:25768 146.09132:41706 155.09987:30189 173.40091:13649 177.11284:75759 | 5.387E-06 |
| POS2107 | THTA | 0.766 | 147.04953 | [M+H]+ | 147.04739 | 55.05366:16760 56.94141:14929 58.06512:6982 68.98124:164801 69.98251:7418 72.08048:55584 72.93573:10611 74.95385:5586 84.07958:12888 86.9928:155495 89.81097:6217 100.0244:13701 101.02005:7847 105.00242:161160 106.00315:6326 118.03261:6972 119.03033:20612 123.01263:12071 128.01855:9989 132.57256:5371 137.03914:13565 | 1.455E-05 |
| POS13907 | eudoginsenoside F | 6.451 | 677.42059 | [M+H]+ | 677.42352 | 101.28879:8495 209.72971:9234 482.76108:9774 | -4.33E-06 |
| POS12728 | Janthitrem E | 4.714 | 604.3606 | [M+2H]2+ | 604.36322 | 73.02811:11897 84.39672:9222 87.04334:26743 89.05896:141711 90.0619:9134 107.30415:9620 133.08543:52468 173.43034:19274 | -4.34E-06 |
| POS8802 | 7,8-didehydro-8'- | 5.794 | 430.28433 | [M+H]+ | 430.28662 | 59.04859:7332 60.08025:14503 76.14551:6421 85.028:73655 86.03025:20533 89.05896:6834 165.22324:5583 173.43036:6525 318.39163:7013 | -5.32E-06 |
| POS10117 | Osimertinib | 6.157 | 500.27371 | [M+Na]+ | 500.276 | 59.04858:7297 60.0093:5919 89.05895:6451 120.96402:6568 | -4.58E-06 |
| POS15273 | pteriatoxin B | 4.672 | 831.47833 | [M+2H]2+ | 831.4823 | 84.64203:28986 87.04334:84677 89.05896:302733 133.08543:119601 151.0771:30111 162.6404:26828 173.43524:53055 217.99625:28931 236.25288:28998 316.36374:26634 359.20026:28753 760.00513:29745 | -4.77E-06 |
| POS1292 | -Mercaptolactic ac | 0.763 | 123.01339 | [M+Na]+ | 123.011 | 68.98124:26614 73.66351:6563 80.04815:9105 86.99279:29251 105.00241:38609 111.29868:5666 | 1.943E-05 |
| POS13903 | ahydrogambogic A | 4.624 | 677.39545 | [M+2H]2+ | 677.39215 | 73.02811:224730 77.35265:107361 87.04333:482475 89.05895:2727703 95.04893:120999 99.04338:114120 106.65066:121495 111.04189:180773 111.29109:94109 113.05926:113537 133.08543:1022934 177.11284:128923 182.38081:103717 432.59784:134147 | 4.872E-06 |
| POS10433 | ydrocochlioquinor | 4.649 | 514.29553 | [M+2H]2+ | 514.29303 | 69.03294:203259 73.02811:75934 73.06432:45759 80.05429:31685 83.0477:45819 87.04334:208479 89.05896:751023 90.0619:31274 99.04339:103542 100.05018:21287 102.06606:17963 111.04189:58334 113.05927:26421 122.06293:37257 129.05446:20840 131.07088:19682 133.08543:257239 144.07597:23916 155.07083:25151 163.73279:16963 175.10023:16845 177.10779:34261 | 4.861E-06 |
| POS15065 | indol-3-ylmethyl) | 4.659 | 787.45392 | [2M+H]2+ | 787.45007 | 87.04334:482067 89.05896:1831671 97.71687:87547 113.05927:156148 131.07088:113006 133.08543:857033 177.11285:118860 | 4.889E-06 |
| POS12764 | h_78_metenolone | 6.296 | 605.453 | [2M+H]+ | 605.45599 | 55.05365:18530 57.06895:14888 67.05389:44064 69.06867:27070 78.97769:6644 79.05312:16915 81.06989:42359 83.08508:13350 91.05389:13794 93.0684:29912 95.08476:34396 105.06944:15997 107.08485:21475 109.10005:12111 117.06911:14630 119.08332:20444 121.10129:15391 131.08376:16397 145.09903:7917 147.11296:8111 149.0948:7143 | -4.94E-06 |
| POS9252 | Arachidyl carnitine | 5.93 | 456.4028 | [M+NH4]+ | 456.40527 | 57.06989:6800 60.08025:30331 85.02799:162764 372.31253:6191 | -5.41E-06 |
| POS13883 | Stevastelin D3 | 4.552 | 676.38037 | [M+H]+ | 676.38373 | 69.03293:85440 87.04333:159569 89.05894:513421 89.77805:41723 99.04337:115176 133.08542:183022 155.06667:43362 173.43031:62566 270.54675:37789 544.49554:35321 | -4.97E-06 |
| POS1464 | 5-dihydrooxazole | 0.764 | 128.01889 | M+CH3OH+H] | 128.0164 | 53.03773:43432 54.03372:13280 55.0177:42032 55.05365:56029 57.03286:7783 57.04396:19937 58.99794:11420 67.04092:11162 68.98123:315590 69.06991:9599 69.9913:6332 71.04815:11644 80.04813:7127 81.06989:7155 82.01235:106684 82.06342:70860 86.05938:6850 86.99104:288728 100.02438:233782 101.02003:12023 105.0024:308647 109.02666:6951 110.00877:16486 118.03259:66900 119.02752:30303 123.0126:31600 127.03808:34422 128.01854:61966 128.06833:12319 | 1.945E-05 |
| POS15117 | lucidanin | 6.195 | 795.54449 | [M+H]+ | 795.54053 | 51.78454:6742 59.04857:8131 | 4.978E-06 |

| Differences in metabolites between the Model group and the Control group | | | | | | | |
|---|---|---|---|---|---|---|---|
| Alignment ID | Metabolite name | Rt(min) | Expreiment Mz | Adduct type | Reference m/z | MS/MS spectrum | PPM |
| POS11691 | Homoamericin | 4.506 | 560.32593 | [M+H]+ | 560.32312 | 69.03294:55295 70.76349:11144 73.02811:25559 83.04932:15615 87.04334:117755 89.05896:361677 99.04339:115428 111.04189:54655 125.0596:21098 131.06766:19237 133.08543:129752 155.07083:33561 173.43034:15515 | 5.015E-06 |
| POS13081 | )en-2,28-diol-3-y | 5.736 | 621.4118 | [M+2H]2+ | 621.41492 | 57.03287:56259 59.04859:169509 69.06992:13135 73.02812:43201 73.06432:20588 76.31847:9349 81.0699:10492 85.06337:40862 87.04335:72631 87.07999:14273 89.05896:238665 89.37024:10329 93.17069:9033 99.07939:11249 101.05931:59170 103.07444:35312 115.07378:15233 117.0909:11264 129.08911:29525 133.08545:84440 143.10413:12803 147.10149:18299 155.00865:10393 | -5.02E-06 |
| POS8736 | alpha-rhamnopyra | 4.599 | 426.24603 | [M+2H]2+ | 426.2486 | 55.05363:41015 59.04856:44172 61.02843:28454 69.03291:717016 70.03657:16774 71.04813:16943 73.02808:176514 73.06429:74263 78.038:22960 80.05426:44187 81.03225:26480 83.04766:97931 87.0433:533648 88.04601:24610 89.05891:1840733 90.06187:73354 95.04889:44866 99.04334:301216 100.05013:64558 102.06601:27615 107.06815:17809 111.04185:146249 113.05922:39878 117.09084:31556 122.06287:110193 125.05954:30750 129.05441:61730 131.07082:39951 133.08537:474505 134.08678:16236 137.05974:15891 144.07591:34950 155.07077:56603 173.08252:20539 175.09518:15045 177.11278:38658 | -6.03E-06 |
| NEG7495 | Sinigrin | 1.077 | 358.27255 | [M-H2O-H]- | 358.26999 | 52.36927:5581 68.06616:5950 93.03925:344479 95.03763:297239 97.03745:9331 124.15676:71886 153.06754:8821 182.18414:14831 239.79329:5500 | 7.145E-06 |
| POS2497 | enzenesulfonic aci | 0.759 | 159.01361 | [M+H-H2O]+ | 159.011 | 55.0177:21242 55.05365:12996 55.93434:80591 56.04952:8941 56.94232:9886 67.05389:20318 68.98123:145643 69.06991:10543 69.9825:8849 70.06429:15395 70.97875:7572 71.04815:9243 72.04369:6827 72.93706:22034 73.06431:35320 77.00898:6160 81.06989:6313 85.01283:17215 86.99277:124379 87.99284:8895 90.94576:6641 95.08475:21154 103.02276:78286 105.0024:157077 106.00548:8678 106.99923:15552 113.00764:17669 113.9631:12778 114.97044:11630 117.03919:20402 121.03262:38130 123.0126:23543 131.01932:140857 132.02023:6080 135.049:14033 147.04784:12385 149.02837:49767 149.06354:9678 | 1.641E-05 |
| POS15116 | lucidanin | 6.192 | 795.53638 | [M+Na]+ | 795.54053 | 59.04858:13984 73.63771:5679 74.19592:5868 118.47446:5723 | -5.22E-06 |
| NEG3634 | ioxa-1,13-trideca | 1.32 | 219.16878 | [M-H2O-H]- | 219.17149 | 97.07645:143213 97.36658:5572 99.07355:118894 102.80595:5628 161.03622:6788 | -1.24E-05 |
| POS4373 | nyl-4-oxochromer | 9.18 | 220.01317 | [M+H]+ | 220.01598 | 55.93344:24593 69.45864:6515 70.35236:5317 72.93571:6290 72.9598:36824 89.96286:5966 90.96998:15094 99.74139:5356 116.97396:24667 132.97009:16945 133.97685:15029 134.96141:6939 146.40657:5172 160.96077:8092 178.97508:7638 | -1.28E-05 |
| POS1494 | 4-Thiouracil | 0.761 | 129.01442 | [M+H-H2O]+ | 129.0116 | 55.05365:41874 56.04952:7266 59.04858:6643 68.98123:66361 69.82184:6053 69.9825:11398 82.01235:14051 82.06502:12911 83.00869:7963 84.07957:34310 86.99104:60373 87.99284:8064 100.02438:32180 101.02003:30250 105.0024:83125 106.00314:10898 110.2371:6091 118.03534:9650 119.0303:75677 123.0126:11470 128.01854:10268 129.01982:7432 | 2.186E-05 |
| POS8211 | eucylglutaminyllys | 4.476 | 388.25256 | [M+Na]+ | 388.2554 | 73.06432:11103 87.04334:33327 89.05896:786442 90.0619:16625 107.07059:7810 131.06766:9936 133.08543:294603 134.08684:8135 173.39601:8740 177.11285:39299 | -7.31E-06 |
| POS12918 | melianol | 4.544 | 613.34045 | [M+Na]+ | 613.33698 | 52.72472:15674 57.68122:14530 59.89664:15405 64.38071:16020 69.0884:14292 86.0954:21295 135.45438:14429 148.67734:14215 311.24231:17300 | 5.658E-06 |
| POS10666 | Azadironol | 4.574 | 523.30011 | [M+2H]2+ | 523.3031 | 65.42842:22264 69.03294:42168 73.02811:122011 73.06432:36758 81.03229:60085 83.0477:36457 87.04334:258228 89.05896:1561065 90.0619:25113 91.04643:26116 95.04893:103995 99.04339:36849 111.04189:117303 113.05927:50583 131.07088:41791 133.08543:537202 134.08684:40688 137.0598:44873 151.09305:23499 155.07083:50347 157.08505:26813 177.10779:46777 178.6362:20443 | -5.71E-06 |
| POS8206 | Neoenactin NL2 | 6.197 | 387.28244 | [M+2H]2+ | 387.28531 | 57.03287:41392 59.04858:357778 60.05225:7977 75.11298:5347 87.04334:34951 99.07938:8139 115.07378:10366 117.09089:21867 206.25749:6138 273.48544:5697 | -7.41E-06 |

**Differences in metabolites between the Model group and the Control group**

| Alignment ID | Metabolite name | Rt(min) | Expreiment Mz | Adduct type | Reference m/z | MS/MS spectrum | PPM |
|---|---|---|---|---|---|---|---|
| POS1205 | -Nitrosothiazolidi | 0.763 | 119.03017 | M+CH3OH+H] | 119.0273 | 53.03773:6029 55.05365:293886 55.93434:9302 56.05673:14151 56.94139:140906 57.05692:28162 58.5172:6224 59.04858:7664 72.08047:370484 72.93706:120281 73.08309:20993 74.95245:9104 77.8651:5377 90.94762:48955 91.34943:5251 101.02003:10175 119.0303:32603 | 2.411E-05 |
| POS6728 | Paromomycin | 8.812 | 308.65833 | [M+Na]2+ | 308.65543 | 57.06989:43927 58.06511:11537 59.07198:10592 67.05271:10123 69.06868:6157 71.08418:22973 81.0699:6861 85.10045:8086 86.0954:13526 95.08476:15209 104.10696:9559 149.01665:5558 165.02727:9359 184.06929:6595 | 9.396E-06 |
| POS14654 | -cyclopenta[a]phe | 4.648 | 743.42847 | [M+2H]2+ | 743.42413 | 72.4287:86873 80.15437:81887 87.04334:542368 89.05896:2429276 133.08543:902522 150.55998:93019 156.23862:78811 177.10779:106599 238.40434:82686 461.10242:89324 | 5.838E-06 |
| POS9130 | Malyngamide M | 4.648 | 450.27386 | [M+2H]2+ | 450.27689 | 58.04042:159330 69.03294:25197 71.04816:13779 73.02811:383167 73.06432:44608 74.03149:12087 80.05429:234713 80.55504:12762 87.04334:122674 89.05896:898052 90.0619:49972 102.06606:155110 102.56839:13871 111.07206:13246 117.09089:10217 124.08022:47892 133.08543:271057 134.09018:13289 146.09132:11754 173.43034:13484 177.11285:22689 289.82092:10072 | -6.73E-06 |
| NEG4055 | Dropropizine | 0.932 | 235.14226 | [M-H2O-H]- | 235.14529 | 93.03925:684991 95.03764:5941 103.07947:15903 124.15677:24049 177.1145:28349 | -1.29E-05 |
| POS781 | 2-Cyanopyridine | 0.767 | 105.00304 | [M+Na]+ | 105 | 58.06416:80101 58.99696:15260 61.1515:5169 68.98123:483576 77.00752:6727 86.99104:609953 87.99284:5427 90.89735:6337 105.0024:672241 | 2.895E-05 |
| POS2846 | 2-Furoylglycine | 0.765 | 170.0405 | [M+NH4]+ | 170.04359 | 67.05389:5526 68.98123:128682 71.71257:6216 76.515:5693 81.06832:6663 82.01395:20593 83.00869:31886 86.99277:146306 100.02438:40441 101.01785:118621 102.01955:6718 105.0024:181483 118.03534:14167 119.0303:229980 120.02943:7976 123.0126:18484 123.46803:5846 128.01854:33603 129.01353:15591 137.03912:93193 142.03293:7856 147.02486:59909 165.03636:9211 | -1.82E-05 |
| POS12920 | limnantheoside A | 4.7 | 613.362 | [M+H]+ | 613.35822 | 73.0281:27054 73.06429:10545 79.63444:12220 80.05273:13791 87.04332:72595 89.05893:308858 90.06371:25293 95.0489:11224 113.05666:13771 133.0854:121564 | 6.163E-06 |
| POS8769 | Andrastin D | 4.637 | 428.25958 | [M+H]2+ | 428.25629 | 58.04042:189786 69.03294:19953 71.04816:16872 73.02811:452445 73.06432:55546 74.03149:12153 80.05429:357769 80.55504:20817 87.04334:174921 88.04782:9696 89.05896:1442791 90.0619:64847 91.06323:9601 99.04339:10211 102.06606:253790 102.56839:18867 107.06821:11122 111.07206:13912 113.05927:11019 115.07378:10454 117.09089:16175 124.08022:101969 124.58041:11349 131.07088:20308 133.08543:466748 134.08684:22188 146.09132:30048 155.09987:29479 177.11285:51265 | 7.682E-06 |
| POS9168 | Z)-Eicosadienoylc | 5.645 | 452.3707 | [M+H]+ | 452.37399 | 50.29372:6425 55.05452:8946 60.08024:22619 69.0699:11333 83.08508:9886 85.02798:101310 173.43521:8354 196.08905:5390 260.33258:6729 | -7.27E-06 |
| POS13386 | njaoamine B | 6.449 | 639.46747 | [2M+H]+ | 639.4632 | 53.00208:6937 57.03287:435875 59.04858:140883 73.37244:5391 81.0699:18838 97.06461:7991 99.07938:30953 101.09641:6964 110.6359:6188 115.07378:34385 117.09089:10878 157.12312:27488 489.76151:5649 | 6.677E-06 |
| POS3236 | L-Tyrosine | 2.199 | 182.08022 | [M+H]+ | 182.08118 | 77.03802:15550 91.05391:637734 92.05669:18134 93.06841:8684 95.04894:297246 103.05422:18541 107.04918:17582 109.06337:19536 118.06567:14021 119.04707:396222 120.05206:9361 121.06411:9132 123.04193:399989 124.04758:7692 136.07611:448969 137.07706:13726 147.04404:69719 165.05461:33159 | -5.27E-06 |
| POS1294 | Niacinamide | 1.644 | 123.05486 | [M+H]+ | 123.05528 | 51.02314:10542 53.03856:116635 56.04952:6667 61.03869:8044 67.05389:11350 68.0495:16272 77.03802:8107 77.45795:6885 78.03359:75791 79.05312:6839 80.04813:999731 81.06989:6655 95.04893:65126 96.04379:265895 105.04401:14387 106.02892:33624 123.05364:805984 124.03867:21063 | -3.41E-06 |
| POS5416 | ycerophosphochol | 1.149 | 258.10968 | [M+H]+ | 258.11008 | 60.08025:65541 71.07261:10895 74.4781:5594 86.09541:106029 98.98415:7132 104.10697:588962 105.10879:17453 124.99956:95822 184.06931:20027 | -1.55E-06 |

**Differences in metabolites between the Model group and the Control group**

| Alignment ID | Metabolite name | Rt(min) | Expreiment Mz | Adduct type | Reference m/z | MS/MS spectrum | PPM |
|---|---|---|---|---|---|---|---|
| POS9985 | LysoPC(16:1/0:0) | 6.086 | 494.32361 | [M+H]+ | 494.32468 | 59.04858:17369 60.08025:150052 69.06991:6945 71.0726:15452 73.02811:8417 86.0954:400958 87.09917:8035 89.05895:17857 95.90849:7302 98.98414:8216 101.0593:7508 104.10696:89747 124.99955:112934 184.07465:1936991 185.07634:52290 | -2.16E-06 |
| POS1578 | -Dihydronaphthal | 4.479 | 131.08511 | M+CH3OH+H] | 131.08553 | 53.03773:26525 56.04953:10752 57.06896:28708 65.03805:28931 67.05389:8711 69.06868:6259 74.09583:8711 84.04316:19602 84.07957:34050 85.0836:6501 86.09541:22325 87.00324:18490 88.00348:8670 91.0539:1673423 92.05669:10880 95.04894:8214 97.00918:26390 103.05421:41006 105.06946:7580 115.05257:62450 116.061:176421 128.06212:19383 129.06706:119311 130.06477:31461 130.15721:9880 131.08377:775724 132.08543:8337 133.06566:6404 | -3.2E-06 |
| POS3512 | ole-3-propionic a | 5.052 | 190.08539 | [M+H]+ | 190.08626 | 55.0177:842102 56.02069:8567 57.03379:6661 67.05389:15577 73.02811:9550 91.05389:18599 103.05421:11714 105.03245:17434 105.06945:8788 107.04917:50390 108.05043:9465 130.06477:2285724 131.06764:84330 144.07968:18119 172.07471:75956 190.08571:14212 | -4.58E-06 |
| POS1867 | Betaine | 1.004 | 140.06779 | [M+Na]+ | 140.06822 | 52.9996:41083 58.06512:19829 74.09583:3209492 75.099:20457 80.94776:159718 80.99314:24787 81.05422:29478 82.06183:23694 82.94377:114697 96.07816:16111 114.08864:279681 140.06865:1283927 | -3.07E-06 |
| POS5318 | Actinopolysporin ( | 6.075 | 255.2303 | [M+H]+ | 255.23151 | 55.0177:7581 55.05365:215649 57.03287:22659 57.06896:267334 59.04858:9281 67.05389:59720 69.06868:223862 71.04945:11979 71.08547:109749 73.06432:8417 78.87981:6127 79.05312:31609 81.06834:96833 83.04932:8425 83.08509:156102 85.06337:6585 85.10045:31519 93.06841:68014 95.08477:76990 96.08625:6932 97.06461:39662 97.09953:93929 99.07939:6303 101.05712:9242 107.08486:62857 109.10006:45041 111.0796:24489 111.11481:18312 121.09843:56798 123.11523:17064 125.09565:19197 135.11646:56832 137.1322:6945 139.11234:13492 149.13002:27740 153.12358:9400 157.46205:6511 163.14494:11539 167.14175:8147 219.21098:7793 | -4.74E-06 |
| POS3313 | Carnitine | 0.991 | 184.09396 | [M-H+Na]+ | 184.09399 | 52.47893:5868 68.05794:5483 102.09042:10203 125.02055:275585 184.0961:18894 | -1.63E-07 |
| POS858 | O-Toluidine | 4.135 | 108.08038 | [M+H]+ | 108.08078 | 65.03805:7553 91.05391:21506 93.05685:8486 108.07939:9923 | -3.7E-06 |
| POS587 | Phosphate | 1.183 | 98.98412 | [M+H]+ | 98.98418 | 53.03773:28313 55.0177:12374 55.05365:16439 56.04862:9275 57.06989:19752 58.06416:5947 60.44798:6097 62.96235:14024 70.0643:22148 71.04945:11951 72.03056:5830 79.05312:9777 80.97279:19262 81.0699:16712 81.54541:6228 98.06007:6323 98.09553:6363 98.98415:1035833 | -6.06E-07 |
| POS788 | o[4.2.0]octa-1,3,5- | 5.024 | 105.06965 | [M+H]+ | 105.0702 | 51.02236:25263 57.03286:8617 58.06416:125667 62.05909:6692 70.70855:5422 77.03802:38850 79.05312:91655 95.04893:82298 103.05421:55881 104.10696:6409 105.04401:25295 105.06945:121863 | -5.23E-06 |
| POS10532 | PC(18:3(6Z,9Z,12Z | 5.974 | 518.32239 | [M+H-H2O]+ | 518.32465 | 57.03287:49122 58.06512:52567 59.04859:118975 60.08025:521029 67.05389:32627 71.07261:70802 73.02812:24968 81.06834:41715 86.09541:1216161 87.04335:37495 89.05896:107388 93.06841:43871 101.05931:25951 103.07444:21465 104.10697:6361878 105.10879:177286 121.1013:29052 124.99956:332870 133.08545:28478 173.39111:28858 184.07466:4098194 185.07635:90381 258.10977:33231 | -4.36E-06 |
| POS11290 | C(20:3(8Z,11Z,14Z | 6.473 | 546.35565 | [M+Na]+ | 546.35596 | 50.35427:5837 60.08022:15840 62.15261:4989 86.09536:55689 104.10692:20699 107.79275:5589 124.9995:12059 146.98271:13095 184.07457:167098 185.07626:7511 343.73755:6496 | -5.67E-07 |
| POS3045 | Indoleacetic acid | 4.908 | 176.07011 | [M+H]+ | 176.07001 | 103.05418:12802 112.7732:5731 130.06473:203262 | 5.68E-07 |
| POS5423 | N-Lauroylglycine | 6.021 | 258.20578 | [M+H]+ | 258.2063 | 55.05365:50283 57.06895:494066 58.07271:13376 67.05389:71873 69.06867:15380 71.08546:161806 76.03858:780549 77.04237:11860 81.06833:49184 83.08508:35643 85.10044:94916 95.08476:128147 97.10158:9774 109.10005:74838 123.11523:11927 173.44014:7303 183.17154:17097 | -2.01E-06 |

**Differences in metabolites between the Model group and the Control group**

| Alignment ID | Metabolite name | Rt(min) | Expreiment Mz | Adduct type | Reference m/z | MS/MS spectrum | PPM |
|---|---|---|---|---|---|---|---|
| POS1541 | Quinoline | 5.061 | 130.06508 | [M+H]+ | 130.06509 | 55.05366:19586 56.04863:54437 57.03287:6719 61.02846:6109 67.05389:17018 69.06992:8063 74.02328:7320 77.03802:33228 77.99809:8488 84.04317:57524 84.07957:71176 87.00325:31408 95.04894:44176 96.00742:5359 102.04613:7030 103.05422:111150 105.04402:9914 109.92458:5445 113.96312:10506 128.04967:13874 130.06477:395782 | -7.69E-08 |
| POS11182 | 0:5(5Z,8Z,11Z,14Z | 5.919 | 542.32269 | [M+H-H2O]+ | 542.32465 | 57.03287:45498 59.04858:141114 60.08025:271303 70.5925:37166 86.09541:703530 89.05896:111950 102.30787:38952 104.10697:3619913 105.10879:72451 111.75905:37431 124.99956:188500 181.02895:41554 184.07466:2838886 185.07635:65489 207.51649:42599 528.25073:34308 | -3.61E-06 |
| POS14781 | C(16:0/18:2(9Z,12Z | 8.803 | 758.56696 | [M+H]+ | 758.5694 | 52.5312:81083 60.08022:297747 70.51228:83223 86.09536:1030962 104.10464:110452 124.99949:267361 184.07457:4859117 185.07626:102746 240.23297:89261 | -3.22E-06 |
| POS11231 | [20:4(8Z,11Z,14Z,1 | 6.192 | 544.33997 | [M+H]+ | 544.34033 | 60.08023:744682 86.09538:1786256 94.47636:236946 104.10693:15225237 124.99951:360103 126.55663:278578 184.0746:7584514 226.83458:285865 261.237:259828 341.89731:242410 381.40887:324009 531.4198:284034 | -6.61E-07 |
| POS2409 | ethanolaminium p | 0.991 | 156.04137 | [M+H]+ | 156.0424 | 74.09583:71991 82.05225:11145 83.05907:63042 93.04334:39455 95.05889:27291 96.9661:7112 105.7131:5254 110.07076:223594 112.05141:10357 114.78535:5385 130.06158:42132 146.88715:6278 156.04167:102805 | -6.6E-06 |
| POS4575 | butyl 9-decenoate | 5.693 | 227.20029 | [M+H]+ | 227.20039 | 55.05365:75761 57.06896:54375 67.05389:21621 69.06992:84161 71.08547:15864 76.01581:5873 79.05312:13981 81.0699:28858 83.08509:31734 93.06841:15564 95.08477:25756 97.06461:14665 97.10159:16206 100.11038:9758 107.08486:19222 109.10006:20843 111.0796:8132 121.1013:13856 123.18568:6006 135.11646:11720 135.83438:5483 138.09952:5890 176.64301:6112 177.47789:7268 | -4.4E-07 |
| POS441 | 3-Methylpyridine | 8.783 | 94.0649 | [M+H]+ | 94.065 | 51.02237:7674 53.03774:81905 59.52111:55294 60.02129:6491 65.03917:8304 67.05389:24286 68.52579:13854 71.02888:55394 71.52904:10201 78.0336:16339 80.0343:12790 82.53645:13303 93.05684:11112 94.06359:527473 96.04379:51254 | -1.06E-06 |
| POS11971 | 2:5(4Z,7Z,10Z,13Z | 6.306 | 570.35352 | [2M+H]+ | 570.35596 | 58.06512:26083 60.08025:237833 71.07261:29532 86.09541:566328 91.0539:27831 104.10697:2834781 105.10879:77117 124.99956:129786 184.07466:1870368 185.07635:54585 220.16205:20271 | -4.28E-06 |
| POS6948 | Leu-Ala-Asp | 4.4 | 318.16559 | [M+H-H2O]+ | 318.1655 | 56.04953:7642 60.04426:6621 60.60372:5468 69.04404:8689 84.07958:126264 85.06337:9106 85.0836:6364 86.09541:165517 87.09918:7101 89.96288:5351 91.0539:82896 99.62812:5429 110.07076:109186 110.98161:5239 111.07458:7410 119.04707:94549 120.05206:8496 120.08031:19854 129.10172:102908 130.1062:8360 132.10175:17803 136.07611:178726 137.07704:14016 147.04404:23887 320.41443:6412 | 2.829E-07 |
| POS7530 | -2-ylthio)-N-(4-r | 0.938 | 348.0777 | M+CH3OH+H] | 348.07773 | 80.94776:10779 86.09541:34363 96.92101:111833 104.10697:163656 104.99086:7337 221.01341:33715 | -8.62E-08 |
| POS6179 | Abacavir | 4.402 | 287.15973 | [M+2H]2+ | 287.15961 | 65.06059:7534 67.51974:6101 70.06431:642851 71.06746:23044 71.16019:5951 79.5765:6169 84.07957:14715 93.07034:8096 97.07488:6848 98.06007:16757 103.05197:23197 112.08709:52986 113.58521:38696 115.08704:10591 120.08031:498348 121.08413:37672 124.08617:13476 126.05367:11642 127.08732:15640 140.07935:15476 151.12099:15033 153.10732:7985 155.07913:10428 157.10622:19128 166.08408:38347 167.11856:8890 194.12837:8407 195.11382:30909 246.511:6159 254.16579:6773 | 4.179E-07 |
| POS10527 | Thr Thr Gly Leu Ile | 6.89 | 518.31836 | [M+H]+ | 518.31842 | 59.27237:6310 66.88557:6165 73.49677:5366 83.51614:5136 86.0954:10455 130.33278:5614 146.98276:20141 324.70667:6624 386.10718:5660 | -1.16E-07 |
| POS14978 | -3-hydroxydecan | 6.997 | 780.54718 | [M+NH4]+ | 780.547 | 56.16686:6569 60.65643:4927 69.75047:6196 70.66898:5524 77.57815:5151 85.42:5496 92.73379:5286 148.68123:5243 152.18787:6049 173.3862:10346 345.00034:6225 772.16333:7117 | 2.306E-07 |
| POS4448 | CHEMBL4283703 | 5.497 | 223.06314 | [M+H-H2O]+ | 223.0632 | 55.05365:8100 58.06512:6930 59.04858:16663 67.05389:6855 67.55789:6106 73.02811:8641 73.04555:15009 89.05896:9864 91.05576:12415 93.06841:16797 95.08477:8575 97.51802:7162 107.08486:6847 148.72409:5533 186.02084:6854 209.01401:7058 225.04044:77480 226.04431:8379 | -2.69E-07 |

**Differences in metabolites between the Model group and the Control group**

| Alignment ID | Metabolite name | Rt(min) | Expreiment Mz | Adduct type | Reference m/z | MS/MS spectrum | PPM |
|---|---|---|---|---|---|---|---|
| POS12709 | ne-3alpha,29-dio | 7.894 | 603.40442 | [M+2H]2+ | 603.4043 | 76.80186:5699 145.42993:6409 173.43034:9974 296.44928:6733 | 1.989E-07 |
| POS14041 | E(16:1(9Z)/16:1(9Z | 8.694 | 688.4928 | [M+H]+ | 688.49115 | 116.11739:6684 173.43523:11648 324.71918:5487 384.25632:6124 | 2.397E-06 |
| POS8795 | Leu Arg Leu | 6.871 | 429.31827 | [M+H]+ | 429.3183 | 70.0706:6749 171.49448:6082 | -6.99E-08 |
| POS5266 | 2'-Methoxyflavone | 0.889 | 253.08517 | [M+Na]+ | 253.08521 | 70.98643:6400 79.20729:5666 90.97554:6469 91.85212:4897 173.38123:7053 178.11426:5697 216.29192:6822 225.03308:5615 | -1.58E-07 |
| POS10586 | oPC(0:0/18:2(9Z,1 | 5.3 | 520.33905 | [M+H-H2O]+ | 520.33899 | 57.0338:18046 59.04859:455554 60.05226:11576 73.02812:27370 86.09541:8922 87.04335:53734 89.05897:271589 101.05931:34605 103.07445:89088 104.10697:36869 117.0909:11733 133.08545:61497 147.1015:32816 184.07468:44142 387.79099:10316 | 1.153E-07 |
| NEG1495 | D-Lysine | 8.738 | 145.0985 | [M-H]- | 145.0985 | 50.06327:6758 64.09624:10247 65.09129:6982 77.10647:7974 84.03101:22843 100.0459:104530 101.05441:2798763 102.0561:244824 116.05927:96064 117.06683:50671 118.18127:60178 145.22426:7981 | 0 |
| POS1076 | HLOROMETHYL) E | 9.304 | 114.97112 | [M+NH4]+ | 114.9711 | 55.0177:41067 55.05365:44699 58.06511:8999 59.04858:59962 60.04425:8361 61.01003:6709 62.98917:6479 68.04829:32860 69.03294:20927 69.06868:46368 70.02779:6245 70.0643:18437 72.04369:17074 72.08047:14898 72.93706:194456 73.04554:22057 73.93719:9384 79.05312:24758 80.05737:6212 86.05938:11508 87.04333:22870 90.94762:44448 91.05576:24234 96.08018:17580 97.0646:6942 108.9558:6241 113.96311:8090 114.06508:26326 114.09125:90865 115.09498:7965 | 1.74E-07 |
| POS3367 | -2,2,6-trimethyl-1 | 6.467 | 185.15295 | [M+H]+ | 185.153 | 50.34662:5815 57.06896:31669 58.9425:10792 83.08509:6768 91.78413:5392 96.43512:5643 114.02061:6970 116.97125:33797 117.97753:35894 139.9868:8151 | -2.7E-07 |
| POS247 | 3-Aminopyrrolidin | 8.756 | 87.09145 | [M+NH4]+ | 87.0916 | 86.05938:18741 87.09045:69839 | -1.72E-06 |
| POS2178 | alic acid 4-Me est | 2.683 | 149.04434 | [M+H]+ | 149.0444 | 65.03803:6116 91.05762:6280 103.0542:6043 103.80193:5951 | -4.03E-07 |
| NEG9755 | TAN-1496 E | 6.476 | 591.06091 | [M-H]- | 591.06061 | 79.0522:6446 79.66678:5497 | 5.076E-07 |
| POS2896 | ole-3-propionic a | 5.061 | 172.07523 | [M+H-H2O]+ | 172.075 | 55.0177:67069 55.05366:45425 57.06989:8313 59.04859:10030 67.05389:105780 71.04816:8019 72.04369:39478 81.06834:15025 82.69934:6493 83.0851:13674 89.06979:11754 95.8057:5137 103.05422:7244 109.06336:6309 109.10007:35552 130.06477:157420 144.00543:6544 | 1.337E-06 |
| POS1881 | Na-Dimethylhistan | 8.768 | 140.11795 | [M+Na]+ | 140.1181 | 52.81611:7314 53.03773:8905 54.03287:5940 55.02822:65891 58.06416:292029 69.06868:37091 70.0643:46062 72.08047:17138 80.04967:6099 83.05907:65928 87.20751:5633 88.63091:6419 97.07487:7111 98.083:7589 123.09176:6408 | -1.07E-06 |
| POS607 | mino-2-methylbu | 8.779 | 99.09141 | [M+H-H2O]+ | 99.0916 | 50.09744:5228 50.32975:5594 50.40268:5236 50.46883:5745 52.30545:4833 54.25877:4925 54.44379:5359 55.05365:23090 55.83027:6131 56.27641:5374 58.25276:5211 58.26422:5113 60.29585:5899 60.89658:5148 61.73696:4792 66.15387:3998 74.47533:4653 75.93189:5489 78.03951:4594 84.36342:4230 87.3108:4715 87.74342:4879 91.89187:4448 96.18139:4135 96.32538:3919 96.68372:5059 97.55318:4156 99.13871:4426 | -1.92E-06 |
| POS4443 | Aminoanthraquin | 4.347 | 223.06284 | [M+H-H2O]+ | 223.063 | 58.06414:18982 61.03867:16876 73.02809:6988 73.04685:17831 91.05573:21622 93.06644:6978 118.53261:5827 159.96542:6065 209.01393:10592 225.04034:69868 | -7.17E-07 |
| POS3345 | htho[2,1-b]thioph | 1.326 | 185.04166 | [M+H]+ | 185.0419 | 55.05363:8202 62.56081:6476 113.0902:5627 | -1.3E-06 |
| POS2945 | thyl-1,2-dihydron | 7.21 | 173.132 | [M+H-H2O]+ | 173.1322 | 50.28837:5925 88.32349:6088 | -1.16E-06 |
| POS2649 | Geijeren | 7.249 | 163.1476 | [M+H]+ | 163.1478 | 63.15053:5460 63.16023:5160 119.3293:5345 | -1.23E-06 |
| POS158 | )-methylbutanenit | 5.967 | 84.08052 | [M+H]+ | 84.0807 | 50.23862:6708 52.12395:4311 56.04863:4952 58.34167:3949 58.59901:4274 59.41376:5032 60.71431:4162 62.00978:4429 62.5035:4784 65.93027:5282 70.29284:4345 70.38533:4521 70.97234:3916 71.45245:4370 74.19456:4335 76.51501:4700 80.29636:5403 81.71966:4195 82.41099:4707 84.04317:8207 86.10914:4745 | -2.14E-06 |
| NEG5237 | Fissoldhimine | 0.955 | 277.16745 | [M-H2O-H]- | 277.16711 | 90.03759:5693 93.03925:2699770 97.03745:10799 102.00297:5516 103.07946:40858 141.08502:6409 151.06711:63717 173.78252:6615 | 1.227E-06 |
| NEG5354 | Oprea1_249309 | 0.955 | 281.16565 | [M-H2O-H]- | 281.16599 | 65.52292:5964 75.06394:6474 93.03932:317154 95.0377:1006561 97.03753:253909 103.08179:10856 105.08054:8355 153.06766:13105 155.06664:13855 | -1.21E-06 |
| NEG6992 | Ferocin | 0.963 | 339.19595 | [M-H2O-H]- | 339.19629 | 64.28277:5751 68.55454:6419 69.57709:5841 93.03925:226395 95.03763:278225 97.03745:46623 153.06755:27307 155.06651:12092 | -1E-06 |

**Differences in metabolites between the Model group and the Control group**

| Alignment ID | Metabolite name | Rt(min) | Expreiment Mz | Adduct type | Reference m/z | MS/MS spectrum | PPM |
|---|---|---|---|---|---|---|---|
| POS9013 | Lovastatin | 4.419 | 443.23703 | [M+H]+ | 443.2373 | 70.06431:6500 82.91782:5885 89.05896:176541 118.06566:20414 132.07892:12359 133.08545:90295 136.07611:17369 144.0797:18937 146.061:96159 159.09138:16799 177.11285:8095 188.07004:385374 189.07106:24890 205.09822:73238 239.14922:15919 | -6.09E-07 |
| POS11316 | yl-O-acetylbuxalo | 6.375 | 547.35266 | [M+H]+ | 547.35303 | 55.87151:5746 57.06989:10179 61.21729:5652 62.39856:5583 71.08547:7034 78.10174:6275 86.0954:23001 129.75313:5718 146.98277:20296 184.07465:16940 | -6.76E-07 |
| POS5114 | nyl)methyl]-N-ethy | 1.172 | 247.09932 | [M+H]+ | 247.09959 | 72.08044:6734 95.30415:4812 101.79417:5815 111.04436:6252 125.05955:7002 160.12196:6138 167.10455:13925 | -1.09E-06 |
| POS716 | Triethylamine | 8.459 | 102.12726 | [M+H]+ | 102.1276 | 50.32131:5604 50.48732:4526 51.19893:5189 51.50716:4412 52.29895:5871 52.71486:5036 53.88306:4640 54.18246:5254 55.00543:5181 59.37344:4488 60.23155:4397 60.9456:4849 61.22037:4370 68.22222:4167 70.48689:4791 70.78522:4244 71.1834:5263 71.56672:4789 87.3178:4485 87.85827:3923 88.16148:5554 91.93539:4267 92.01117:4186 92.1326:4756 92.31329:4411 93.96178:5724 96.02963:4223 96.6327:5515 98.03295:4386 | -3.33E-06 |
| POS7953 | trophenyl-L-Trypt | 6.984 | 371.09888 | [M+H]+ | 371.09857 | 173.44016:9933 | 8.354E-07 |
| NEG7218 | oro-10-hydroxyoc | 0.947 | 347.2363 | [M-H]- | 347.2359 | 50.12337:9028 60.17111:6402 76.71121:5469 82.79803:6263 93.03925:337442 95.03763:217009 97.03745:11363 103.07946:11923 151.06711:9117 153.06754:13092 338.8721:6308 | 1.152E-06 |
| POS2926 | Xanthopappin A | 1.019 | 173.04153 | [M+Na]+ | 173.0419 | 70.06431:9695 173.04349:21661 175.89915:6307 | -2.14E-06 |
| POS6152 | Myristoylglycine | 6.484 | 286.23709 | [M+H]+ | 286.23749 | 55.39032:5951 57.06896:39152 67.05389:7861 69.06992:7043 71.08548:23636 76.03859:91189 85.10045:6830 95.08477:13941 121.30764:5824 | -1.4E-06 |
| POS6418 | bimidazole ribonu | 0.949 | 296.0636 | [M+H]+ | 296.06409 | 60.08025:23971 71.07261:19411 86.09541:204355 104.10697:776778 105.10879:24854 112.89422:14757 120.00122:13938 169.9852:20399 236.99554:7672 | -1.66E-06 |
| POS11866 | Leu Tyr Lys Glu | 6.195 | 566.31787 | [M+Na]+ | 566.31842 | 51.65913:10940 58.06512:15513 60.08025:31763 67.05272:14886 71.07261:14371 79.05313:11711 86.09541:230832 93.06841:17973 104.10697:1422411 105.10879:41028 119.08334:13797 146.97896:190666 180.48094:11372 354.95444:10709 | -9.71E-07 |
| NEG1226 | ylpyridin-3-yl)bor | 7.106 | 136.05812 | [M-H]- | 136.0576 | 56.7561:5952 65.09239:8743 89.13125:21010 135.05537:9016 136.05812:8624 137.05487:11191 | 3.822E-06 |
| POS11544 | Juvenimicin A2 | 8.739 | 554.36932 | [M+2H]2+ | 554.36871 | 74.18494:6379 124.93654:5543 173.3911:12067 290.60663:5553 | 1.1E-06 |
| POS678 | Homopiperazine | 8.743 | 101.10677 | [M+Na]+ | 101.10732 | 70.53394:6167 | -5.44E-06 |
| NEG6949 | | 0.963 | 337.19568 | [M-H2O-H]- | 337.19629 | 55.66968:5877 93.03925:612058 95.03763:385042 103.08171:6340 151.06711:31030 153.06754:44012 | -1.81E-06 |
| POS4798 | Callicarpenal | 5.882 | 235.20441 | [M+H-H2O]+ | 235.205 | 55.05365:12582 57.06896:10346 67.05389:19811 69.06992:6651 81.0699:20363 93.06841:13292 95.08477:16563 107.08486:10424 109.10006:8951 121.1013:6810 133.09863:9261 173.39601:9357 189.166:6362 202.80254:6188 | -2.51E-06 |
| POS11920 | Cholylhistidine | 6.474 | 568.33496 | [M+H]+ | 568.33569 | 53.90853:6958 104.10692:24584 130.73308:5764 208.30186:6079 316.26694:5760 355.9762:6039 | -1.28E-06 |
| POS939 | alpha-monochlor | 8.801 | 111.02007 | M+CH3OH+H]2 | 111.0207 | 54.03373:13309 55.04137:24637 55.93435:9953 56.96447:10873 57.9342:423490 67.49115:13985 69.99382:12383 71.05973:21944 72.93706:74402 74.93712:72777 76.49632:41026 77.99808:33349 78.9988:108420 79.49879:9418 84.94554:64956 85.50143:15059 87.00323:120458 88.00347:1912326 88.50381:137503 89.00122:26993 89.50647:13046 90.50591:56403 90.94576:21419 92.94704:11518 95.01113:30420 97.00507:278656 97.50974:30324 97.95379:29597 98.51207:25622 98.96088:91524 99.51075:403118 100.0115:33286 102.95542:46552 106.01485:14048 108.51748:32990 108.95824:12706 110.95901:9218 111.01927:40352 113.96311:112265 | -5.67E-06 |
| POS15031 | PC(16:0e/12-HETE | 8.806 | 784.58392 | [M+Na]+ | 784.58502 | 53.95275:6330 55.18986:7167 57.91145:6853 104.49812:5708 134.72931:5113 151.41658:5085 164.64087:5531 | -1.4E-06 |
| NEG4606 | Tripelennamine | 2.717 | 254.16559 | [M-H]- | 254.16631 | 106.66814:6352 179.69743:5091 182.80327:5982 | -2.83E-06 |
| NEG9857 | ihydro-3-[2-[[(2E | 6.196 | 633.1239 | [M-H]- | 633.12299 | 89.1313:491831 90.13493:24984 171.80325:5295 | 1.437E-06 |
| POS8135 | -methoxyphenyl}- | 1.034 | 383.11487 | [M+H]+ | 383.11569 | 53.2382:5895 53.43983:6521 82.94374:5768 203.05078:79829 238.1199:5854 | -2.14E-06 |

| Differences in metabolites between the Model group and the Control group | | | | | | | |
|---|---|---|---|---|---|---|---|
| Alignment ID | Metabolite name | Rt(min) | Expreiment Mz | Adduct type | Reference m/z | MS/MS spectrum | PPM |
| POS937 | alpha-monochlor | 6.48 | 111.01991 | [M+2H]2+ | 111.0207 | 55.0405:17427 55.93345:11104 57.9342:173904 67.49116:9696 72.93707:81535 74.93713:30982 75.49612:11274 76.4949:17358 77.99808:37259 78.99881:42211 84.94555:24951 85.49975:11244 87.00324:180801 87.50562:13106 88.00348:875251 88.50382:46455 89.00123:17917 89.50648:13792 90.50592:20519 90.94577:26361 96.00943:12156 97.00918:304637 97.50975:12290 98.51208:34880 98.96088:31845 99.51076:172240 100.01151:17327 102.95543:15439 106.01486:16401 108.51749:25855 108.95825:19281 111.01928:19849 113.96312:132436 | -7.12E-06 |
| POS7454 | IC ACID DER (FR. I | 5.815 | 343.28339 | [M+H]+ | 343.28421 | 57.06893:9343 60.08022:5723 67.24886:5568 74.11497:5719 86.09536:13025 163.8858:5687 170.79715:6674 184.07457:13501 188.54721:5212 200.60612:6777 216.05299:5938 | -2.39E-06 |
| NEG1917 | 2403;(E)-Metanico | 0.965 | 161.10941 | [2M-H]- | 161.10851 | 55.08503:9289 57.10279:31440 59.08405:50394 71.099:60111 73.11621:35802 74.11263:10004 85.1313:50907 87.11256:8759 93.03928:677611 95.12722:11837 99.16254:9323 101.14605:5754 103.0795:17885 104.15944:8033 113.15993:18230 | 5.586E-06 |
| NEG3318 | yl-1H-pyrazol-4-y | 1.228 | 208.0872 | [M-H]- | 208.0881 | 59.08407:39933 71.09774:8072 73.11623:69572 74.12088:6859 77.71171:5539 79.05373:15231 85.13134:6082 89.13131:33557 89.14215:7143 97.07652:565952 101.14608:10721 119.08548:7638 120.83963:5409 126.02468:11126 128.02199:17080 146.40347:5377 161.2383:8561 | -4.33E-06 |
| NEG8213 | Kazinol F | 0.992 | 395.22391 | [M-H2O-H]- | 395.22299 | 65.39328:5279 67.67252:5287 80.98167:5345 87.53817:5223 89.13125:10868 93.03925:321897 95.03763:159660 97.03745:18025 101.79763:5129 147.15915:8848 151.06712:23521 153.06755:21269 186.0121:5720 208.17513:5711 333.99783:5874 | 2.328E-06 |
| POS15052 | 18:1(9Z)/18:2(9Z,1 | 8.722 | 786.52936 | [M+2H]2+ | 786.52789 | 52.01259:6679 60.08025:11626 71.0726:6657 86.09541:24623 100.38199:6010 104.10696:10800 124.99955:5935 184.07466:79999 518.55597:6718 | 1.869E-06 |
| NEG3633 | Dehydrocarissone | 0.952 | 219.13754 | [M-H2O-H]- | 219.1385 | 51.07359:5631 93.03929:2280891 103.07951:87869 103.76355:5961 123.10053:12025 151.06718:8102 | -4.38E-06 |
| POS4286 | henylphosphinam | 1.46 | 217.06667 | [M+H]+ | 217.0657 | 52.0803:7392 58.06319:6150 61.18333:6596 66.03267:5109 70.06427:6646 | 4.469E-06 |
| POS3884 | yn-1-one\|trans-5- | 4.268 | 203.05142 | [M+H]+ | 203.0524 | 55.05363:12412 60.04424:5763 72.74344:6130 78.84068:5528 79.36346:5228 82.00914:5838 | -4.83E-06 |
| NEG8216 | squalene | 0.812 | 395.36743 | [M-H]- | 395.36841 | 103.07276:11174 147.11324:14110 174.91417:5283 | -2.48E-06 |
| POS11810 | Ser Leu Pro Tyr Ala | 5.918 | 564.30145 | [M+Na]+ | 564.30267 | 57.03377:6989 59.04856:38234 60.08023:11190 86.09538:35400 87.04506:7924 89.05893:20376 103.0744:6286 104.10693:246669 105.10875:6849 126.15702:5745 146.98273:18779 173.37634:6494 | -2.16E-06 |
| POS7643 | Capillolide | 5.044 | 353.23419 | [M+2H]2+ | 353.23309 | 57.03287:115815 58.04137:39062 59.04859:2607516 60.05226:48531 65.04819:56027 67.05508:14948 69.06992:20878 73.02812:241521 73.06432:163613 80.0543:55733 85.06506:44910 87.04335:225480 87.06254:82433 87.07999:29597 89.05896:1496379 90.06191:47134 94.06947:27298 101.05931:145079 102.06606:28477 103.07444:367553 104.07732:15207 109.0756:38159 115.07643:17702 117.05281:192758 117.0909:49022 129.08911:26939 131.07088:105185 133.08545:425978 134.09018:17067 147.10149:87636 161.1188:22603 177.11285:32119 | 3.114E-06 |
| NEG9707 | Epicatechin | 6.194 | 579.00317 | [M-H]- | 579.00452 | 51.52467:7674 115.89837:5631 125.34409:5780 173.16458:5215 320.50644:7472 | -2.33E-06 |
| NEG1677 | 5-propanoic acid, | 1.081 | 153.06828 | [M-H]- | 153.067 | 60.17114:7239 79.0537:13233 93.03928:807346 93.15691:14844 95.03767:1450379 154.24504:7592 | 8.362E-06 |
| POS11282 | anine-3beta,17,23 | 6.388 | 546.34113 | [M+H]+ | 546.34253 | 61.57684:6459 69.38287:7407 78.57826:5935 97.37959:6004 173.43524:12754 323.24142:5347 333.33173:6626 | -2.56E-06 |
| NEG968 | Flucytosine | 1.381 | 128.02403 | [M-H]- | 128.0253 | 89.13129:16069 127.55685:5690 128.19003:7317 | -9.92E-06 |
| NEG818 | 4-Methylcatechol | 1.229 | 123.0424 | [M-H]- | 123.0437 | 80.0528:71794 87.12827:5272 94.14119:14123 124.15681:26277 | -1.06E-05 |
| NEG6185 | | 0.908 | 309.28989 | [M-H]- | 309.2912 | 93.03925:25532 95.03763:17698 97.03951:8962 103.07947:6493 105.07814:127109 113.12114:13673 124.15676:19737 | -4.24E-06 |
| NEG3877 | Sarmentol A | 0.932 | 229.17865 | [M-H]- | 229.17999 | 79.05367:35423 93.03925:1344531 95.03763:52927 97.03745:12581 97.08466:13376 103.07946:280871 | -5.85E-06 |
| POS9171 | es-arg9)-Bradykin | 4.463 | 452.73608 | [2M+H]2+ | 452.73743 | 60.04426:33393 70.06431:764671 71.06746:21564 86.05939:5841 112.08709:15961 120.08031:56494 157.10199:11559 166.08408:6287 173.39111:10443 263.13351:20543 453.70743:8619 | -2.98E-06 |
| POS11227 | Thr Ile Pro Ile Thr | 7.036 | 544.33527 | [M+H]+ | 544.33398 | 50.03354:5940 100.52903:6793 284.80084:6514 | 2.37E-06 |

**Differences in metabolites between the Model group and the Control group**

| Alignment ID | Metabolite name | Rt(min) | Expreiment Mz | Adduct type | Reference m/z | MS/MS spectrum | PPM |
|---|---|---|---|---|---|---|---|
| NEG9635 | H,2H-Perfluorode | 5.97 | 562.99481 | [M-H]- | 562.99323 | 59.08402:9092 79.05367:13711 93.85946:5239 95.63505:5906 102.93809:5184 277.54752:8793 | 2.806E-06 |
| NEG1638 | Ribitol | 1.081 | 151.0685 | [M-H2O-H]- | 151.07001 | 90.1349:28099 93.03928:2218054 108.14954:29810 136.36186:5906 151.20676:17480 | -1E-05 |
| NEG1302 | Myrothenone B | 7.705 | 138.05797 | [M-H]- | 138.0564 | 57.42459:5554 137.05487:7981 | 1.137E-05 |
| NEG3683 | pyrazin-2-yl)meth | 1.238 | 222.10211 | [M-H]- | 222.1037 | 97.07645:144827 195.65027:6966 | -7.16E-06 |
| POS7824 | ne glycol monodo | 6.877 | 363.30881 | [M+H]+ | 363.31039 | 56.0234:6606 82.39974:5989 286.62631:5919 | -4.35E-06 |
| POS3176 | Phenprobamate | 0.891 | 180.0993 | [M+H]+ | 180.10091 | 50.5035:5940 56.24197:6611 160.02197:6206 | -8.94E-06 |
| POS11350 | n E2 p-benzamido | 6.252 | 548.29883 | [M+2H]2+ | 548.3006 | 51.76935:6327 69.95988:6295 104.10697:31123 184.06931:8699 | -3.23E-06 |
| NEG3663 | SCHEMBL1388932 | 0.955 | 221.13783 | [M-H]- | 221.1395 | 93.03925:792254 95.03763:1454007 103.07947:55862 105.07814:25656 | -7.55E-06 |
| POS10738 | 22alpha,23alpha-e | 6.78 | 525.34564 | [M+2H]2+ | 525.34747 | 55.72471:7451 69.15876:5473 154.17889:5150 171.15733:5928 206.71631:5893 207.01024:5952 | -3.48E-06 |
| POS10472 | Conglobatin | 6.082 | 516.30499 | [M+Na]+ | 516.30682 | 50.25237:7851 53.78294:6299 53.80497:7313 60.08022:6032 86.09536:48983 95.5825:6341 100.53114:5675 104.10692:335300 105.10873:8662 146.97888:29680 171.22461:6543 173.43517:13877 280.15149:7285 499.54236:7580 | -3.54E-06 |
| POS6904 | ctamide semisucc | 4.981 | 316.20999 | [M+2H]2+ | 316.21179 | 56.28908:6220 57.06894:21287 65.11468:5248 71.08544:10108 85.02796:11016 116.069:22489 144.06477:116778 155.23679:5802 158.0791:20443 204.08626:9544 | -5.69E-06 |
| NEG6902 | Istamycin KL1 | 0.963 | 335.19553 | [M-H2O-H]- | 335.1937 | 89.13124:24821 93.03925:542343 95.03763:47169 97.03745:32893 116.0619:6219 133.28078:6024 142.01402:5396 151.06711:47960 167.22272:11081 334.64075:5292 | 5.46E-06 |
| POS11349 | (-)-Detoxin D1 | 6.308 | 548.29456 | [M+H]+ | 548.29657 | 59.04859:7284 76.51501:6913 89.05896:7114 104.10697:43253 183.96208:5542 184.06931:9899 198.2594:6424 338.27423:5626 361.66922:5296 | -3.67E-06 |
| POS979 | omethylphosphon | 6.48 | 112.01766 | [M+2H]2+ | 112.01578 | 54.03373:103496 55.04137:15011 57.9342:140994 59.92951:82930 67.05389:22556 69.04403:12416 70.06431:13730 71.05974:323263 74.93713:25651 76.49633:17608 76.93212:12667 78.99881:19813 79.99587:19222 84.07957:13037 84.94555:17418 85.49975:9229 86.94054:7455 88.00348:610381 88.50382:41697 89.00123:340111 89.50102:31088 90.50592:8788 94.06358:11669 95.01114:12653 97.00918:209536 97.50975:10133 98.00587:132842 98.50788:10097 98.96088:29253 99.51076:99752 100.50954:66568 100.95683:13413 101.01132:12715 102.95543:16278 108.51749:16719 109.51485:11260 111.01928:11083 112.07434:12583 | 1.678E-05 |
| POS4336 | Theobromine | 1.009 | 219.02596 | [M+H]+ | 219.02786 | 57.99675:6674 62.98166:16790 80.94775:20348 82.94376:622374 85.02799:6873 90.97557:117177 131.77626:6205 159.04828:7808 177.06223:11363 | -8.67E-06 |
| POS7450 | Nostocyclyne A | 7.302 | 343.26077 | [M+H]+ | 343.26279 | 55.31514:6243 56.21209:5842 70.43735:6475 92.06237:5683 124.23769:5623 | -5.88E-06 |
| POS1825 | Fumaric acid | 1.867 | 139.00229 | [M+H-H2O]+ | 139.00021 | 52.35179:5638 55.0177:46251 55.05365:17776 62.64914:5860 67.05389:10667 68.996:233036 79.05312:9205 81.06989:9322 82.07301:11076 94.06554:12297 95.04893:8493 95.08476:6105 96.04379:6441 96.08827:7644 105.44981:6102 110.05835:8897 111.00671:27130 112.58314:5851 120.04356:7439 122.06003:9424 138.04724:13404 | 1.496E-05 |
| POS4492 | Cysteinyl-Cysteine | 0.99 | 225.03397 | [M+Na]+ | 225.0361 | 62.98166:93415 66.34612:5813 81.50273:5899 86.9684:6983 96.92101:46571 98.91862:42037 104.99086:895706 105.99377:9747 116.99028:108325 135.00185:16176 | -9.47E-06 |
| NEG1725 | Histidine | 1.079 | 155.06726 | [M-H2O-H]- | 155.06947 | 64.06541:5847 79.0537:191622 93.15691:22481 95.03767:519248 97.03749:252310 97.07648:109035 111.15354:21238 137.08247:9393 156.05357:9694 | -1.43E-05 |
| POS1695 | Iodotyrosine | 8.763 | 135.00243 | [M+H]+ | 135 | 91.13419:5808 111.37939:6078 | 1.8E-05 |
| NEG3252 | droxytrihomometh | 1.228 | 206.08826 | [M-H2O-H]- | 206.08569 | 59.08407:15520 73.11623:33213 75.54928:5876 79.4505:5340 89.13131:7356 95.0377:12043 103.68422:6117 108.77026:5580 126.02468:113253 143.15802:6167 161.03633:9333 181.00244:6086 | 1.247E-05 |
| NEG9525 | Chlorfluazuron | 6.082 | 538.96613 | [M-H2O-H]- | 538.96301 | 173.6057:21503 253.52328:66828 254.53137:9394 284.3291:5692 | 5.789E-06 |
| NEG8825 | 3beta-ol\|4alpha,2 | 0.904 | 443.42911 | [M-H2O-H]- | 443.4259 | 55.6278:5822 93.03925:66331 95.03763:35097 97.03745:12013 103.07947:87112 113.12114:15535 257.57416:5848 | 7.239E-06 |
| NEG8927 | .-Triacontanediol( | 0.864 | 453.47113 | [M-H2O-H]- | 453.4678 | 76.92368:6228 84.98986:6183 86.25851:6162 113.12118:1495906 114.12465:34477 172.76915:6133 181.19069:44372 249.2543:6909 | 7.343E-06 |

**Differences in metabolites between the Model group and the Control group**

| Alignment ID | Metabolite name | Rt(min) | Expreiment Mz | Adduct type | Reference m/z | MS/MS spectrum | PPM |
|---|---|---|---|---|---|---|---|
| POS3961 | L-Tryptophan | 4.419 | 205.09682 | [M+H]+ | 205.09718 | 55.0177:28497 74.02328:473445 91.0539:335248 103.05421:28178 105.06946:27738 115.05257:692070 117.05825:161407 117.06913:685825 118.0629:11985257 118.43848:28942 119.06659:379179 130.06477:708654 131.07088:44960 132.07892:2374151 133.08215:83645 142.06567:905584 143.07104:1194480 144.06113:83329 144.0797:4341700 145.06152:115992 146.05721:17431700 146.54747:29480 146.57033:23767 147.06317:678492 155.0584:63157 159.09137:1192681 160.07417:256210 170.05661:862231 171.0612:37257 188.07004:2923840 189.07106:109007 | -1.76E-06 |
| POS2612 | L-Carnitine | 1.011 | 162.11203 | [M+H]+ | 162.11247 | 55.05363:6803 57.03284:158120 58.06414:36314 59.07293:35311 60.08022:815004 61.02843:9713 84.08119:18494 85.02795:386671 102.09037:217084 103.03844:486962 104.04307:7802 162.11153:194240 | -2.71E-06 |
| POS3448 | lquinolinium-2-ca | 4.418 | 188.07056 | [M+H]+ | 188.0705 | 91.0539:698427 103.05421:33981 115.05257:797674 116.06099:47668 117.06913:479114 118.0629:8225772 119.06659:228830 128.04967:38424 132.07892:89223 142.06566:594714 143.07103:1165095 144.07968:1851898 145.06526:103181 146.05721:5633082 147.06317:168062 155.0584:95603 160.07417:64165 170.06137:422132 188.07004:287089 | 3.19E-07 |
| POS2411 | Histidine | 1.055 | 156.07626 | [M+H]+ | 156.07678 | 56.04861:28400 68.04948:13371 81.04324:6644 82.05224:18332 83.05906:110927 93.04333:76341 95.05887:33226 100.90237:6143 104.79472:5957 110.07073:347611 | -3.33E-06 |
| POS1770 | Hypoxanthine | 4.373 | 137.04532 | [M+H]+ | 137.04578 | 55.02822:373014 57.06896:12667 67.02796:86950 67.05389:58725 72.93707:26646 79.05313:37936 81.06834:159572 82.03949:271530 83.02332:40703 91.0539:79146 92.02255:44135 94.03812:523453 95.08477:94932 110.03357:1091621 112.04887:62715 119.03312:800626 120.01816:13134 128.04344:37191 137.04604:3745190 138.02985:30815 | -3.36E-06 |
| POS1184 | L-Valine | 1.106 | 118.08609 | [M+H]+ | 118.08628 | 53.01368:8607 53.03856:65233 55.05365:3077097 56.04952:54851 57.05414:55938 57.05692:310822 58.06511:25899 59.04858:57039 59.07294:20713 71.06873:8607 72.08047:4019858 73.06431:7744 73.08443:10666 118.08493:16295 | -1.61E-06 |
| POS5832 | auryldiethanolamir | 5.023 | 274.27338 | [M+H]+ | 274.27408 | 55.05365:20481 57.06896:921924 58.06416:58013 58.07272:18651 60.04325:14176 62.05909:51561 68.04829:8100 69.06991:12514 70.0643:945401 71.06745:19545 71.08418:297450 72.08836:10263 74.05885:7639 85.10044:81364 86.05938:14506 88.07444:1278210 89.07881:29560 102.09042:352008 103.09467:7840 106.08522:681112 107.08961:13477 212.23726:7148 230.24779:8332 256.25845:32904 274.2739:217946 275.27167:24002 | -2.55E-06 |
| POS4078 | Kynurenine | 4.336 | 209.09169 | [M+H]+ | 209.09209 | 74.02328:33601 91.0539:6350 94.06359:112013 99.00742:20398 104.04768:13858 118.0629:36202 120.04358:39052 132.04306:15007 136.07611:15142 146.05721:51480 150.05342:7705 174.05498:15598 | -1.91E-06 |
| POS3014 | DL-Arginine | 1.029 | 175.11815 | [M+H]+ | 175.11897 | 60.05526:769510 70.06431:2166182 71.04816:42605 71.06746:29367 72.08048:76556 84.07957:21869 88.07445:9948 97.07488:9417 98.06007:10224 112.08709:35482 113.0696:19749 115.08704:8718 116.06905:236146 130.09663:114071 134.01685:9323 158.09198:33378 175.12015:47736 | -4.68E-06 |
| POS2215 | Methionine | 1.565 | 150.05785 | [M+H]+ | 150.05827 | 53.03773:28598 56.04862:4098100 57.03286:40299 57.05229:26356 58.99405:43779 61.01003:3042040 74.02328:200846 74.05885:141817 74.98869:16092 75.02496:27598 77.00462:19300 84.04315:60120 85.00947:23833 85.02799:62622 87.0259:334150 102.05498:226649 104.05223:569591 105.00008:19749 105.03476:23692 133.02939:316994 150.05736:14543 | -2.8E-06 |
| POS161 | Tetrahydropyridine | 1.102 | 84.08079 | [M+H]+ | 84.08077 | 55.05365:23097 56.04862:215522 57.06988:9418 67.05389:28831 69.05635:8003 82.06502:37404 82.94376:11047 84.04315:141536 84.07957:511719 | 2.379E-07 |
| POS3070 | Serotonin | 3.359 | 177.10153 | [M+H]+ | 177.10223 | 55.93435:49513 67.04092:27707 68.70155:5785 72.93707:17109 72.96115:9613 79.05313:7960 90.94764:6919 91.0539:13002 94.04205:7205 95.9206:5567 105.06946:43974 115.05257:174659 117.05553:76819 130.06477:10756 131.04832:11171 131.07088:9076 132.07892:159173 133.06236:28355 135.94325:6628 142.06567:27246 143.07104:15197 159.06984:16603 160.07417:785549 161.07928:20445 174.8517:5705 | -3.95E-06 |

**Differences in metabolites between the Model group and the Control group**

| Alignment ID | Metabolite name | Rt(min) | Expreiment Mz | Adduct type | Reference m/z | MS/MS spectrum | PPM |
|---|---|---|---|---|---|---|---|
| POS6593 | adecyldiethanolan | 5.238 | 302.30368 | [M+H]+ | 302.30539 | 55.05366:13204 57.06897:611891 58.06512:28764 58.07272:12523 60.04426:8334 62.0591:34532 64.82231:6567 68.0483:9617 69.06869:8540 70.06431:594018 71.08548:215123 72.08837:7588 85.10046:75133 86.05939:9152 88.07445:808188 89.07883:13366 102.09042:211452 106.08524:437557 107.08725:6986 152.76207:6151 194.68724:6670 284.29575:9125 302.30051:69166 303.30765:7273 | -5.66E-06 |
| POS2981 | Hexanoylglycine | 4.763 | 174.11218 | [M+H]+ | 174.11247 | 55.05365:13064 71.08547:378903 76.03858:429061 76.36717:6444 81.06833:31898 86.0954:13485 99.07938:93356 110.10796:5561 | -1.67E-06 |
| POS2296 | Xanthine | 2.151 | 153.0399 | [M+H]+ | 153.04068 | 55.02822:218475 55.05366:12446 55.93435:8781 79.05313:12347 81.00723:29302 82.03949:98438 83.02332:23846 83.04932:7681 92.0491:17941 93.00867:15229 107.04918:12248 108.01909:12309 108.04321:9568 110.03357:754289 110.06084:19264 128.04344:78686 136.01477:29202 153.04224:170907 154.0228:243066 | -5.1E-06 |
| POS10644 | LysoPC(18:1/0:0) | 5.57 | 522.35522 | [M+H]+ | 522.35541 | 58.06512:449133 60.08025:4693632 67.05389:258153 69.06992:217609 71.07261:665162 81.0699:254886 83.0851:210672 86.09541:11328304 89.05896:197113 95.08478:374162 104.10697:67285736 105.10879:1243121 124.99956:3462611 163.01524:388928 184.07466:61771484 185.07635:1554875 258.10977:355226 339.29205:229168 | -3.64E-07 |
| POS769 | Choline | 1.002 | 104.10676 | [M]+ | 104.10699 | 58.06511:824339 59.07295:236863 60.08025:4750052 104.10696:4088644 | -2.21E-06 |
| POS10646 | ysoPC(18:1(11Z)/0: | 8.803 | 522.35553 | [M+H]+ | 522.35596 | 60.08025:221595 71.07261:35196 86.09541:701630 104.10697:335647 124.99956:210444 184.07466:4791878 185.07635:136659 | -8.23E-07 |
| POS11922 | 5(4Z,7Z,10Z,13Z,16 | 6.131 | 568.33942 | M+CH3OH+H] | 568.34033 | 56.04953:11079 59.04858:26899 60.08025:262932 71.0726:47332 86.09541:749017 87.09918:21258 89.05896:15985 98.98203:14355 104.10696:143380 124.99955:252441 133.08543:14529 184.07466:3618798 185.07635:86007 | -1.6E-06 |
| POS1185 | yl-a-aminoisobut | 1.013 | 118.08609 | [M+H]+ | 118.0866 | 53.03773:43769 55.05365:2259418 56.04862:35861 57.05693:188243 58.06416:1108702 59.04858:40500 59.07198:1019120 70.0643:13165 71.06746:29623 72.08047:2998117 73.08444:9731 118.08494:671902 | -4.32E-06 |
| POS1735 | Benzothiazole | 5.225 | 136.02126 | [M+H]+ | 136.02158 | 65.03805:7972 109.00957:24687 136.02158:109387 | -2.35E-06 |
| POS5962 | Dibutyl phthalate | 6.414 | 279.15845 | [M+H]+ | 279.15909 | 57.06896:458324 58.07272:10673 65.03804:35537 67.05389:8404 81.0699:8269 88.65063:6869 91.05203:5475 93.03178:13287 107.08248:6126 121.02692:82243 149.02057:2416805 150.02579:107710 151.60489:5687 167.03043:6447 | -2.29E-06 |
| POS2172 | Phthalic anhydride | 6.414 | 149.02289 | [M+H]+ | 149.02328 | 65.03804:511486 84.07957:7042 93.03371:50982 111.0444:60191 121.02691:156586 132.55287:5859 149.02055:222848 150.02579:6832 | -2.62E-06 |
| POS1188 | D-Valine | 2.589 | 118.08617 | [M+H]+ | 118.08626 | 55.05363:412497 56.9423:10025 57.0569:33033 58.06509:10545 59.04856:11710 72.08044:483927 72.93703:7811 | -7.62E-07 |
| POS1120 | Proline | 1.038 | 116.0703 | [M+H]+ | 116.07058 | 52.41612:6575 54.56131:6288 56.04953:7301 56.55671:5899 58.06512:10102 59.07198:7702 68.0495:25256 70.0643:5008722 71.06874:10617 80.4635:5832 101.43783:6059 116.06905:173371 | -2.41E-06 |
| POS2701 | hol-phenylpyruvat | 2.2 | 165.05429 | [M+H]+ | 165.05479 | 51.02237:6584 57.03287:7438 61.03972:6501 65.03805:19987 67.05389:14565 69.03294:14200 77.03802:40323 91.0539:159150 93.06841:8331 95.04894:567612 96.05188:7459 103.05422:24102 105.04402:33986 109.06337:24149 119.04707:243123 120.08031:7830 121.0641:7408 123.04193:323178 124.04758:6472 147.04404:25713 | -3.03E-06 |
| POS2112 | D-Glutamine | 1.094 | 147.07629 | [M+H]+ | 147.0764 | 55.05365:24853 56.04862:358514 57.03286:10764 58.06511:15396 60.08024:37261 67.05389:20696 69.03294:7830 72.08047:11212 74.02328:23964 82.06502:8848 84.04315:2728692 84.07294:49023 84.07957:583470 85.02799:47516 85.04652:17991 86.05938:28923 87.04333:69607 91.05389:7809 101.07021:39437 102.05498:54518 130.04883:350584 130.08388:31960 | -7.48E-07 |
| POS2818 | Uric acid | 1.747 | 169.03532 | [M+H]+ | 169.03558 | 55.02822:192629 55.05365:10007 62.44093:5208 67.05389:13338 69.00832:71887 70.03912:370193 81.06833:14998 83.02332:8731 91.68036:5230 96.01751:146140 98.03504:216974 99.01799:24070 109.00224:8843 114.03108:5956 123.0302:7455 124.01198:48054 126.02935:290713 127.03194:7306 131.69183:5995 141.03847:532027 142.04385:5920 144.03883:5866 152.00659:160570 169.03252:387130 170.01851:46635 | -1.54E-06 |

| Differences in metabolites between the Model group and the Control group | | | | | | | |
|---|---|---|---|---|---|---|---|
| Alignment ID | Metabolite name | Rt(min) | Expreiment Mz | Adduct type | Reference m/z | MS/MS spectrum | PPM |
| POS3931 | DL-Acetylcarnitine | 1.064 | 204.12248 | [M+H]+ | 204.1232 | 57.03286:122207 58.06511:24310 60.08024:668239 85.02798:3912352 86.03195:55965 144.10196:60948 145.05025:75050 204.1239:36013 | -3.53E-06 |
| POS8739 | Oleoylcarnitine | 5.6 | 426.35623 | [M+H]+ | 426.35779 | 55.05366:23248 57.03287:55325 57.06896:58528 59.04859:9346 60.08025:449291 67.05389:18680 69.06868:35283 71.08419:28706 81.06834:24527 83.0851:38575 85.028:2271848 85.10045:16305 86.03025:37104 93.07034:11432 95.08477:38242 97.09953:26196 107.08487:8926 109.10007:17784 111.11732:8733 121.1013:22005 135.11646:14656 144.10197:46088 149.13394:13348 173.43036:7350 | -3.66E-06 |
| POS4054 | cetyl-DL-phenylala | 4.736 | 208.09615 | [M+H]+ | 208.09679 | 53.41887:5633 57.52442:6482 77.038:6865 79.05311:5655 86.17436:6026 91.05388:7406 93.06838:21384 103.05419:54353 107.04677:9415 118.06287:6317 120.06332:33234 120.08028:1588035 121.0841:55192 131.04829:15870 162.0894:10263 166.08403:36842 173.38617:11598 | -3.08E-06 |
| POS8577 | lonaethylene glyc | 4.509 | 415.25272 | [M+H]+ | 415.25378 | 73.06432:16324 86.09541:11486 87.04334:57259 89.05896:974340 90.0619:14109 107.07059:8721 117.09089:6569 131.07088:8163 133.08543:338896 134.08684:10247 177.11285:27933 207.3625:5970 257.82501:6144 380.27573:5685 | -2.55E-06 |
| POS7979 | yristoyllevocarniti | 5.356 | 372.30908 | [M+H]+ | 372.31079 | 51.56357:6742 57.03287:15133 57.06896:18033 60.08025:185054 67.05389:6094 71.08547:16644 85.02799:971725 86.03024:16129 95.08477:8937 144.10197:17959 244.00337:6547 | -4.59E-06 |
| POS5115 | l-Acetyltryptopha | 4.741 | 247.10725 | [M+H]+ | 247.10768 | 118.0629:26566 130.06477:64358 132.07892:48513 142.06567:9048 144.0797:20273 146.05721:74183 159.09138:159110 170.05661:15699 173.42545:8643 187.08794:9341 188.07004:58967 201.10141:15716 | -1.74E-06 |
| POS8386 | -Palmitoylcarnitin | 5.562 | 400.34106 | [M+H]+ | 400.34207 | 55.05366:9409 57.03287:67078 57.06896:102012 58.06512:12737 60.08025:814513 61.08379:10726 67.05389:14313 69.06868:21933 71.08547:66770 81.0699:39890 83.0851:33708 85.028:4222678 85.10045:39216 86.03025:64784 89.05896:8238 95.08477:49160 97.09953:14544 103.07444:7100 109.10007:31112 123.11524:16860 125.13172:6703 135.66116:6320 137.13222:10360 144.10197:90033 174.76732:6511 239.23662:17393 | -2.52E-06 |
| POS10708 | telet-activating fa | 6.595 | 524.36841 | [M+H]+ | 524.37097 | 53.9196:8265 57.06989:10359 60.08025:51861 64.15609:7760 71.0726:8861 86.0954:182323 101.47952:7156 104.10696:45818 124.99955:59156 160.51437:7745 184.07465:693771 185.07634:14888 | -4.88E-06 |
| POS4571 | cyclotetradecane- | 4.475 | 227.17447 | [M+H]+ | 227.17529 | 55.05365:298983 58.07272:6155 64.70701:6480 69.06992:11014 81.0699:6881 82.06343:5855 83.0851:96790 96.08018:20307 100.11039:1132751 101.11388:29135 109.10007:7135 110.10548:5919 112.84786:6464 114.09126:9909 117.13717:10401 139.08766:7866 209.16336:30335 227.17255:16373 | -3.61E-06 |
| POS1673 | o-Tolyl isocyanate | 4.856 | 134.05936 | [M+H]+ | 134.06 | 77.03802:14145 78.04544:8286 79.05312:146398 79.23306:6396 87.09918:9982 88.02121:9864 95.04893:31499 104.04768:26491 105.03246:75496 106.06411:712941 107.06821:9029 120.69875:6478 133.05247:21390 134.06017:283645 | -4.77E-06 |
| POS2543 | 2721-59-7 | 3.357 | 160.0753 | [M+H]+ | 160.07561 | 52.64342:6544 55.0177:14037 55.05365:20891 55.93435:52322 56.94139:43783 57.06988:6318 65.03804:8303 67.04092:46275 67.05389:7590 69.06991:22072 71.04816:6935 72.08047:7876 72.93706:46772 74.95245:8165 79.05312:25691 90.94762:27540 91.05389:21823 94.04007:9009 95.04893:7258 96.08826:6989 103.05421:9639 105.06944:110273 107.04678:6672 113.9631:30824 114.09124:7417 115.05256:338584 116.97396:6430 117.05552:188538 118.06289:16734 130.06476:23657 131.04831:14488 131.07086:21686 131.97139:9239 132.0789:260377 132.97009:15510 133.06564:43156 142.06566:59329 143.07103:39725 159.06551:36453 160.07416:622196 161.07925:12631 162.91693:5901 | -1.94E-06 |
| POS5704 | Inosine | 4.382 | 269.08707 | [M+H]+ | 269.08801 | 55.0177:37760 55.0291:33671 55.78193:7005 57.03287:64369 59.04859:7084 61.02846:9158 67.02914:10736 69.03294:15844 73.02812:24585 82.03949:29577 85.028:23898 94.04008:62874 110.03357:124679 115.03932:10195 119.0359:90606 133.04918:9042 137.04604:5792410 138.04726:214745 | -3.49E-06 |

**Differences in metabolites between the Model group and the Control group**

| Alignment ID | Metabolite name | Rt(min) | Expreiment Mz | Adduct type | Reference m/z | MS/MS spectrum | PPM |
|---|---|---|---|---|---|---|---|
| POS4708 | Butyrylcarnitine | 4.389 | 232.15369 | [M+H]+ | 232.15421 | 57.03287:82378 58.06512:12276 60.08025:393758 71.04816:65210 72.08047:16666 84.07957:47991 85.028:3262740 86.03024:66696 86.09541:21691 126.09014:13972 144.10197:46354 173.07771:70441 232.15034:32028 | -2.24E-06 |
| POS3508 | Acetyl-L-glutamic | 2.201 | 190.07033 | [M+H]+ | 190.07098 | 56.04861:6674 84.04314:94451 102.05497:10788 126.02325:5684 130.04883:35527 | -3.42E-06 |
| POS1531 | Pyroglutamic acid | 1.096 | 130.04965 | [M+H]+ | 130.05009 | 55.05453:7494 56.04862:128944 58.06511:10979 67.05389:10890 70.0643:61359 74.02328:5937 83.05907:29290 84.04315:318672 84.07957:532844 85.02799:13483 86.05938:34163 102.63087:5424 119.02752:8836 130.04883:17137 130.08388:22824 | -3.38E-06 |
| POS1050 | Creatinine | 1.126 | 114.06573 | [M+H]+ | 114.06618 | 68.0495:10084 70.0643:10541 72.04369:19987 84.48338:6386 86.07139:35948 111.68296:5557 114.06509:147086 | -3.95E-06 |
| POS9749 | yl-sn-glycero-3-p | 6.116 | 482.36169 | [M+H]+ | 482.36047 | 57.03286:28980 57.06895:63700 59.04858:22687 60.08024:71295 71.08547:41875 75.04311:10959 81.06833:9913 83.08509:8698 85.10044:24531 86.0954:111504 89.05895:12491 95.08476:8318 97.10158:6672 104.10696:1097444 105.10878:27839 109.10005:7273 124.99954:36928 173.43033:10886 184.07465:41126 | 2.529E-06 |
| POS1441 | Thymine | 4.432 | 127.04982 | [M+H]+ | 127.05018 | 52.01421:6341 53.03857:8442 54.03373:44442 55.01771:6053 56.04863:31362 75.04592:5839 81.04327:7179 82.02832:13058 84.04317:16026 109.03891:11158 110.02366:31838 127.05041:24780 | -2.83E-06 |
| POS2506 | -Naphthalenediam | 4.419 | 159.09103 | [M+H]+ | 159.0916 | 55.05366:12243 55.93345:53946 56.04953:12704 60.32402:6182 67.05389:16534 69.06992:16412 70.06431:17375 71.04816:7946 72.93573:18955 84.07957:6821 91.0539:13916 95.08477:25721 100.07598:7385 105.06946:21656 112.11003:13647 114.97046:14039 115.05257:162222 116.05025:10474 117.05553:243885 118.0629:69762 130.06477:220108 131.06766:12297 132.07892:561926 133.08215:11112 140.0473:10653 142.06567:92787 158.08345:12692 159.09138:178121 160.07417:53064 | -3.58E-06 |
| POS10032 | nitoylphosphatidyl | 5.109 | 496.33975 | [M+H]+ | 496.33978 | 57.06893:305017 58.06509:151177 60.08022:1846755 64.85479:109228 71.07257:252067 71.08544:138140 82.89832:114492 86.09536:4277546 95.08472:157403 104.10692:23310622 105.10873:612520 124.9995:934110 163.01515:138220 165.84062:115113 184.07457:11627109 185.07626:200921 308.44281:114198 | -6.04E-08 |
| POS7339 | 13-Docosenamide | 8.427 | 338.34024 | [M+H]+ | 338.34167 | 55.0177:8204 55.05365:82802 57.03286:15973 57.06895:169688 58.06416:6364 67.05389:47175 69.06991:155934 71.04816:9116 71.08546:98556 72.08047:14585 73.52791:5775 79.05312:12140 81.06989:81173 83.08509:175609 85.10044:47719 86.05938:22227 93.0684:24825 95.08476:95549 97.0646:36675 97.09953:141619 100.07381:41903 107.08485:30694 109.10005:61223 111.07959:41175 111.1148:59469 114.09125:51521 121.10129:58797 123.11523:34047 125.09564:23458 125.13171:22326 128.10568:30349 135.11644:57188 137.12875:13189 139.11232:13424 142.12386:16195 149.13:41095 153.12764:6558 156.13799:12623 163.14493:14298 167.14174:7100 255.61653:6275 296.59183:5912 | -4.23E-06 |
| POS5467 | -Hexanoylcarnitin | 4.523 | 260.18475 | [M+H]+ | 260.18558 | 57.03286:16321 60.08025:87561 71.08418:20259 85.02799:751638 86.03195:13108 99.07938:16217 144.10197:9767 201.11363:7309 | -3.19E-06 |
| POS5882 | D-Glutarylcarnitine | 3.222 | 276.14291 | [M+H]+ | 276.14417 | 57.03287:8411 60.08025:50595 85.02799:168427 87.04334:33049 103.03849:11218 115.03932:24286 144.10197:6565 235.2888:5383 | -4.56E-06 |
| POS8575 | Gelomulide N | 5.751 | 415.2103 | [M-H2O+H]+ | 415.211 | 65.03805:7164 69.03294:8960 77.03802:6531 79.05313:6739 81.03386:6738 91.0539:42167 91.11925:7534 104.06136:7053 105.06946:7622 107.08487:11777 117.06913:11433 119.08334:2012474 120.08878:79446 133.06236:23733 135.07936:19701 | -1.69E-06 |
| POS7458 | ıramidopropylbeta | 5.351 | 343.29492 | [M]+ | 343.2955 | 57.06895:68496 58.06416:29621 71.08418:38504 76.07277:6028 81.06989:9508 83.08508:10148 85.10044:20196 95.08476:31625 109.10005:30042 123.11523:7812 155.90779:5375 173.43033:7572 183.17154:29559 240.23306:237476 241.23578:19036 | -1.69E-06 |

**Differences in metabolites between the Model group and the Control group**

| Alignment ID | Metabolite name | Rt(min) | Expreiment Mz | Adduct type | Reference m/z | MS/MS spectrum | PPM |
|---|---|---|---|---|---|---|---|
| POS6062 | Oleamide | 8.825 | 282.27826 | [M+H]+ | 282.27908 | 51.70781:6117 55.05365:55737 57.03287:7605 57.06896:66412 57.61167:6213 67.05389:18045 69.06868:96769 71.08547:32424 81.0699:32367 83.08509:53084 85.10045:10326 86.05939:7345 86.2397:5472 93.06841:11368 95.08477:26417 97.06461:14262 97.09953:33756 100.07597:13617 107.08486:22620 109.10006:20293 111.0796:10966 111.11481:20975 114.08864:10737 121.1013:10895 123.11523:8425 135.11646:11592 149.13002:6796 | -2.9E-06 |
| POS5032 | -Undecanoylglycir | 5.123 | 244.18991 | [M+H]+ | 244.19051 | 55.01682:31910 55.05365:249817 56.04862:22095 57.03286:23057 57.06895:138638 58.06416:22317 59.04858:6940 67.05389:128244 69.06867:175357 70.0643:10450 71.04816:25624 71.08418:61457 72.08047:27603 79.05312:63333 81.06833:253740 82.06342:11569 83.04932:10233 83.08508:255996 84.07957:14624 85.06336:23497 85.10044:9918 86.05938:35246 86.0954:13572 91.05389:14000 93.0684:96198 95.08476:202887 96.08018:17514 97.0646:54115 97.09953:140084 98.09552:13043 99.07938:11830 100.07381:44311 100.11037:11172 102.05498:12926 105.06944:10460 107.08485:147261 108.08662:8437 109.10005:39996 110.09555:34732 111.07959:47528 114.09124:36105 119.08332:7507 121.10129:108901 123.11523:22668 124.1099:51772 125.09564:30075 128.10568:13750 131.08376:34733 135.11644:27705 137.13219:8729 138.1274:37965 142.12386:7647 145.10278:5768 147.11681:7541 149.13:6646 156.17151:10797 163.14493:117767 164.15204:7861 167.74667:5342 173.1315:30440 180.17407:189833 181.15976:27629 190.15889:7151 198.18146:15198 226.17574:87363 | -2.46E-06 |
| POS4651 | Lauramine oxide | 5.036 | 230.24738 | [M+H]+ | 230.2476 | 55.05365:16217 57.06896:495944 58.06416:66882 58.07177:10942 62.05909:265085 69.06991:8703 71.08418:155775 85.10044:44003 93.06841:10097 167.10463:8934 173.39601:7315 212.23726:40352 230.24779:246622 231.24898:15788 | -9.55E-07 |
| POS15000 | PC(16:0/18:1(9Z)) | 5.983 | 782.56464 | [M+Na]+ | 782.56702 | 60.08023:54684 64.37959:20354 86.09538:167105 104.10693:27097 124.99951:56726 170.22337:18464 184.0746:721530 185.07629:25982 560.38855:21848 | -3.04E-06 |
| POS1791 | gamma-Terpinene | 1.204 | 137.13217 | [M+H]+ | 137.13251 | 53.03773:18258 55.05365:44628 57.06896:22098 65.03805:13369 67.05389:96747 69.06992:23501 79.05312:56781 81.06834:278486 91.0539:45570 93.06841:14718 95.08477:138938 109.10007:6333 110.03356:10919 119.03311:11075 136.05907:16809 137.04259:13538 137.1322:6631 | -2.48E-06 |
| POS3648 | Phenylacetylglycine | 4.656 | 194.08057 | [M+H]+ | 194.08118 | 53.29583:5855 65.03804:5998 76.03858:359475 91.05389:813066 92.05668:25266 120.0803:7288 135.04227:8015 177.25467:5430 | -3.14E-06 |
| POS2040 | -Methyleneglutara | 9.911 | 145.04929 | [M+H]+ | 145.04939 | 53.03774:301799 55.0177:188558 55.05366:29937 56.04953:11584 56.9414:7899 57.03287:138126 57.06989:6914 59.04858:13138 67.01736:6858 69.03294:30235 70.06431:62151 71.01216:17320 71.04816:385264 72.93707:11418 81.0323:93678 81.0699:14753 84.99097:13324 85.028:42224 98.06007:25728 98.09553:25813 99.0434:540241 101.02222:10793 127.0381:52379 145.04651:18737 | -6.89E-07 |
| POS10710 | enantio-PAF C-16 | 5.717 | 524.36853 | [M+H]+ | 524.37018 | 60.08023:659119 71.0713:114821 86.09538:2224914 99.50859:76156 104.10693:924207 105.65473:68598 124.99951:644511 150.57184:80642 173.4303:120925 180.49649:74015 184.0746:13549719 185.07629:272102 235.66122:75223 | -3.15E-06 |
| POS3510 | ole-3-methyl acet | 5.322 | 190.08531 | [M+H]+ | 190.08627 | 69.89706:5812 103.05418:6452 130.06473:289564 131.06761:12461 | -5.05E-06 |
| POS6792 | nma-Glutamyltyro | 4.376 | 311.12238 | [M+H]+ | 311.1235 | 55.42663:5924 55.52425:6174 70.06431:37661 84.04317:139861 84.07958:22231 91.0539:11376 95.04894:7660 102.05277:7646 119.04707:56794 120.08031:10712 123.04193:85722 130.04886:25579 130.08708:7867 136.07611:194826 137.07704:12027 147.04404:37702 161.81059:5585 163.28825:5993 165.05461:131356 180.08583:8347 182.0797:35967 202.08495:11454 | -3.6E-06 |

**Differences in metabolites between the Model group and the Control group**

| Alignment ID | Metabolite name | Rt(min) | Expreiment Mz | Adduct type | Reference m/z | MS/MS spectrum | PPM |
|---|---|---|---|---|---|---|---|
| POS2076 | ole-3-carboxaldeh | 4.428 | 146.06004 | [M+H]+ | 146.0601 | 53.03774:51781 55.0177:49959 55.05366:156692 57.03287:28205 57.06896:17716 58.06512:8631 59.04859:7799 65.03805:10573 71.04816:83011 72.08048:15333 75.02498:8273 76.03859:10560 81.0323:22008 82.06503:9116 83.0851:53324 85.028:10182 86.05939:18263 86.09541:14054 91.0539:647726 99.0434:103394 100.04588:11014 100.07383:16352 100.11039:168880 112.95607:7029 117.05553:126525 118.0629:1226964 119.06659:13999 127.0381:7106 145.08403:18572 146.05721:81042 146.9101:6684 | -4.11E-07 |
| POS10588 | oPC(0:0/18:2(9Z,1 | 6.252 | 520.33984 | [M+H]+ | 520.33929 | 56.04953:104351 58.06512:138990 60.08025:2771459 67.05389:276732 69.06868:175855 71.07261:293752 81.06834:329192 86.09541:6767502 87.09918:146255 95.08477:191886 104.10697:35008200 105.10879:669086 107.08487:89972 109.10007:124939 124.99956:1800818 163.01524:161429 173.39603:106986 181.02371:83013 184.07466:32080096 185.07635:720869 258.10977:180580 | 1.057E-06 |
| POS1054 | peridinecarboxalde | 4.523 | 114.09103 | [M+H]+ | 114.09189 | 53.03773:22129 54.03373:19684 55.0177:275908 55.05365:720196 56.04952:14814 58.02807:28138 58.06511:13280 65.03804:9965 67.05389:191692 68.04829:166504 69.03294:17596 69.06868:1196464 70.0643:116677 71.04816:59570 71.08547:74695 72.08047:136179 77.03802:45747 79.05312:1238748 81.05579:32851 84.07957:26762 86.05938:14861 86.0954:96980 91.05576:9702 95.04893:72789 96.08018:613010 97.0646:185251 105.04401:16672 114.09125:4150725 115.09233:15965 | -7.54E-06 |
| POS1831 | Urocanic acid | 1.535 | 139.04982 | [M+H]+ | 139.05019 | 55.0177:9414 66.03385:11141 67.05389:7759 68.04949:6381 68.99723:18717 82.0746:5494 93.04333:35198 94.06358:6205 95.05888:8466 112.03867:5948 121.03835:36538 139.0489:13188 | -2.66E-06 |
| POS3462 | tyloxy)propan-1- | 4.786 | 188.19987 | [M+H]+ | 188.2009 | 55.05364:8123 57.06987:499831 58.06511:847825 59.04857:20349 67.88462:6390 69.0699:7033 71.08546:249705 72.08835:5825 76.07561:347996 118.06287:19451 123.48865:5416 146.06096:11333 188.19746:7647 | -5.47E-06 |
| POS1021 | Uracil | 1.557 | 113.03455 | [M+H]+ | 113.03458 | 67.0539:6326 69.04404:15747 70.0278:42926 96.00742:15749 113.03346:21848 | -2.65E-07 |
| POS10030 | LysoPC(0:0/16:0) | 6.521 | 496.33655 | [M+H]+ | 496.3403 | 57.06896:6797 58.06607:7574 60.08025:92779 71.07261:9771 86.09541:301558 98.98415:8214 104.10697:91629 124.99956:81893 184.07466:2157294 185.07635:54194 | -7.56E-06 |
| POS9747 | LysoPC(15:0/0:0) | 6.25 | 482.32437 | [M+H]+ | 482.32407 | 57.06989:8019 59.04858:8869 60.08025:54297 72.65694:7997 82.89674:6261 86.09541:112673 100.42736:5892 104.10696:681901 105.10879:16336 111.15255:5910 124.99955:23582 173.43034:7038 182.06386:7149 184.07466:284068 185.07635:9479 | 6.22E-07 |
| POS6642 | methyl-dodecylar | 5.336 | 304.29819 | [M]+ | 304.2999 | 50.02972:7158 58.06416:92108 62.08849:6043 91.05389:147827 173.4254:6580 212.23723:15942 | -5.62E-06 |
| POS1545 | 2E)-Decenoyl-AC | 1.07 | 130.08577 | [M+H]+ | 130.0864 | 56.04862:118858 58.06511:7521 67.05389:8363 70.0643:49894 83.05907:18019 84.04315:299249 84.07957:499412 85.02799:12389 86.05938:19031 130.04883:13762 130.08388:25923 | -4.84E-06 |
| POS986 | Cytosine | 1.59 | 112.05036 | [M+H]+ | 112.05058 | 55.05365:13088 56.04862:9977 67.02913:10786 69.04403:32435 94.04007:8199 95.02306:57115 112.04886:82686 | -1.96E-06 |
| POS2545 | ndoleacetaldehyd | 4.419 | 160.07535 | [M+H]+ | 160.07568 | 53.03275:7156 55.05364:23287 62.32769:5960 69.0699:10742 72.08046:9774 91.05388:14019 91.74825:5456 104.08642:6121 105.06712:6511 105.79249:5560 113.11348:5554 114.12788:11061 115.05254:31254 115.66189:6374 117.0555:42038 118.06287:86211 130.06474:31119 131.07085:7940 132.07889:76863 133.08542:11895 142.06563:6600 159.09134:9600 160.07414:10775 | -2.06E-06 |
| POS630 | rolidinecarboxalde | 4.404 | 100.07545 | [M+H]+ | 100.07624 | 53.03773:15572 55.0177:33096 55.05365:79980 56.04862:75630 57.06896:14117 58.02807:34226 58.06512:9289 59.04858:12863 62.80278:6011 69.03294:9240 72.04369:11810 72.08047:11866 79.76434:5267 82.06503:14023 83.01195:6788 84.06467:6030 91.50716:5661 97.81029:5782 98.98415:8797 100.03944:6290 100.07382:172510 | -7.89E-06 |

| Differences in metabolites between the Model group and the Control group | | | | | | | |
|---|---|---|---|---|---|---|---|
| Alignment ID | Metabolite name | Rt(min) | Expreiment Mz | Adduct type | Reference m/z | MS/MS spectrum | PPM |
| POS101 | 1,3,5-Hexatriene | 0.646 | 81.07 | [M+H]+ | 81.06988 | 53.03773:68020 55.0177:9358 55.05365:6707 55.92267:5853 56.96447:19258 65.03804:8525 66.04538:8879 67.50902:13644 79.05312:86752 80.04813:8423 81.04482:7777 81.06833:89005 | 1.48E-06 |
| POS10358 | LysoPC(O-18:0/0:0 | 6.057 | 510.38947 | [M+H]+ | 510.39178 | 60.08025:20922 66.76708:5146 70.14877:6543 71.08547:5711 85.028:17314 86.09541:33705 104.10696:321132 105.10879:12888 124.99955:10268 184.06929:22176 297.80222:6396 | -4.53E-06 |
| POS1539 | Isoquinoline | 5.322 | 130.06502 | [M+H]+ | 130.0654 | 55.05451:10840 56.0486:14816 67.05387:6220 77.03799:5908 84.04313:22135 84.07954:42714 95.0489:12653 103.05418:29486 105.04398:6228 130.06473:96836 | -2.92E-06 |
| POS1967 | Kojic acid | 4.376 | 143.03334 | [M+H]+ | 143.03329 | 55.01768:35985 55.05363:31541 56.94137:14899 59.04856:6648 62.4123:6113 69.03291:59530 70.06427:12391 71.04813:16095 72.08044:6174 72.93703:11363 97.0276:9883 98.06002:6243 98.09548:5300 143.03423:122999 | 3.496E-07 |
| POS6089 | adecadiene-1,18- | 0.723 | 283.26205 | [M+H]+ | 283.263 | 55.05365:51287 57.06895:74067 61.03869:14029 67.05389:17077 69.06867:58545 71.08546:26828 81.06832:30109 83.08508:26890 85.10044:10909 93.0684:11862 95.08475:26486 96.94559:5643 97.0646:9364 97.09952:16086 107.08485:12356 109.10005:7087 111.11479:7947 121.10128:11040 135.11642:11242 149.13:6480 171.68271:6646 | -3.35E-06 |
| POS6212 | Octanoylcarnitine | 4.73 | 288.21643 | [M+H]+ | 288.21689 | 54.70615:6900 57.06896:15297 60.08025:41909 75.81551:5321 77.89019:6069 85.02799:316477 102.05719:7244 127.10885:8097 144.10197:9062 | -1.6E-06 |
| POS9770 | ctapropylene glyc | 5.388 | 483.35306 | [M+H]+ | 483.35278 | 57.03287:73776 59.04858:840745 60.05225:12790 60.13429:6466 64.94802:6318 70.09454:7101 76.96691:6548 83.1958:6334 87.04334:10053 89.05896:20193 99.07938:8483 115.07378:9116 117.09089:109783 175.1301:13269 198.19347:5897 305.05081:6387 325.36108:5759 336.14642:6711 | 5.793E-07 |
| POS4456 | Tetraglyme | 4.517 | 223.15384 | [M+H]+ | 223.15398 | 57.03286:17644 59.04858:719164 61.02742:8630 73.02676:7417 84.0448:8180 87.04333:15449 89.05894:137329 90.16662:6172 93.0684:12612 103.07442:34124 117.05279:24353 | -6.27E-07 |
| POS2554 | ninovaleric acid be | 1.024 | 160.1326 | [M+H]+ | 160.13318 | 53.03857:8319 55.05365:1155093 55.0633:28922 56.05674:13139 57.03287:10381 58.06416:57207 59.04858:237063 59.07295:29511 60.08025:840096 70.0643:50259 73.06432:8252 83.0477:172410 84.07957:32714 100.07382:12056 101.0593:460148 102.06162:8367 115.05257:20736 116.04757:7478 117.05553:12796 132.07892:12704 160.07417:29256 160.13072:166982 | -3.62E-06 |
| POS10356 | LysoPC(17:0/0:0) | 5.894 | 510.35648 | [M+H]+ | 510.35538 | 57.03287:86428 59.04859:50801 60.08025:54653 81.0699:7127 86.09541:122606 87.04335:5693 89.05896:8908 95.08477:7326 95.98923:6149 99.07939:13648 104.10697:742077 105.10879:18157 124.99956:30848 133.4489:5693 157.12312:11359 184.07466:368997 185.07635:9702 236.22951:5710 306.14078:5994 | 2.155E-06 |
| POS1177 | Indole | 4.441 | 118.06494 | [M+H]+ | 118.0653 | 53.03857:8410 55.05365:361485 57.05693:29432 58.06512:13322 59.04858:11113 59.07296:9243 70.06557:6328 72.04501:10182 72.08048:568553 77.03802:6876 84.00181:6311 91.0539:163299 95.04894:23475 105.04401:7866 117.05553:22955 118.0629:214646 | -3.05E-06 |
| POS1789 | -alpha-Phellandre | 2.777 | 137.13206 | [M+H]+ | 137.13251 | 50.26157:6555 53.03856:10255 55.05365:24128 56.94232:13348 57.06988:10135 67.05389:39521 69.06991:14085 72.93706:12012 79.05312:32040 81.06833:129502 89.90059:5602 90.94762:9906 91.05389:17447 93.0684:7249 95.08476:58768 103.24319:6600 136.06245:6346 | -3.28E-06 |
| POS5283 | octadienyl ester 4- | 5.89 | 253.21492 | [M+H]+ | 253.2157 | 55.05366:44772 57.06896:14797 64.38515:5835 67.05389:25247 69.06869:39445 79.05313:6614 81.06834:26705 83.0851:16776 93.07034:10381 95.08478:27456 97.06461:7369 97.10159:8873 107.08487:10864 109.10007:10862 112.12533:5510 119.08334:10334 121.09844:7845 133.10193:7679 147.11682:6121 | -3.08E-06 |
| POS9476 | LysoPC(14:0/0:0) | 5.948 | 468.30829 | [M+H]+ | 468.30847 | 57.03284:142001 57.06893:45390 58.03659:30210 59.04856:38273 60.08022:255437 71.08544:26823 86.09536:548739 87.09914:17012 95.08472:17725 104.10692:2605018 105.10873:68251 124.9995:104966 184.07457:971578 185.07626:30284 348.94217:16614 | -3.84E-07 |

| Differences in metabolites between the Model group and the Control group | | | | | | | |
|---|---|---|---|---|---|---|---|
| Alignment ID | Metabolite name | Rt(min) | Expreiment Mz | Adduct type | Reference m/z | MS/MS spectrum | PPM |
| POS1748 | 2-Phenylacetamide | 2.199 | 136.07529 | [M+H]+ | 136.0757 | 55.93345:61193 56.94233:8625 65.03805:41428 67.05389:6790 71.92827:16470 72.93707:55150 79.05313:8527 81.0699:20341 89.93906:9812 90.94764:24493 91.0539:664279 94.04008:7175 107.04918:41799 109.06581:10356 118.06566:13408 119.04707:61620 119.80384:6310 136.02159:13051 136.05907:20846 137.04604:7569 | -3.01E-06 |
| POS7477 | Lauroylcarnitine | 5.14 | 344.27841 | [M+H]+ | 344.27948 | 57.03287:14438 57.06896:18743 59.04858:78071 60.08025:121773 67.05389:10990 69.08101:8178 71.08547:7833 79.05463:9091 81.06833:11027 83.08509:7217 85.028:749086 86.03196:11393 86.61588:6665 88.03895:6369 89.05896:24823 93.06841:16163 95.08477:12929 103.07444:8760 107.08486:11118 109.10006:11058 135.07935:7396 144.10197:12672 163.14494:7803 183.17155:10126 | -3.11E-06 |
| POS206 | Methacrylamide | 4.801 | 86.05978 | [M+H]+ | 86.06 | 58.06416:79400 69.03293:13792 84.03653:5447 86.05937:25114 | -2.56E-06 |
| POS1238 | Isoindoline | 3.962 | 120.08053 | [M+NH4]+ | 120.08077 | 55.05366:9682 56.05764:7025 56.9414:19400 59.60601:5756 61.0387:28501 72.93573:19823 73.08311:14147 77.03802:38079 90.94764:8302 91.0539:63293 93.06841:61439 95.04894:121825 102.04613:6800 103.05422:433237 105.04402:28614 118.0629:7220 120.08031:292394 | -2E-06 |
| POS1440 | 2H-pyran-3-carb | 0.182 | 127.03897 | [M+H]+ | 127.03952 | 50.26004:5651 53.03856:115390 53.93916:7725 55.0177:130637 55.05365:26134 56.04862:10501 56.96447:8503 57.03286:22978 67.05389:9915 70.06556:12345 71.04816:43129 79.05312:8435 80.04813:22549 81.03228:31319 81.06832:17377 82.06502:9874 84.04315:7402 84.95899:8129 99.04338:9133 108.04319:9438 109.02666:25826 127.03809:287876 | -4.33E-06 |
| POS2847 | Indoleacrylic acid | 4.418 | 170.05942 | [M+H-H2O]+ | 170.06004 | 50.93715:6072 55.0177:6911 55.05365:19589 55.93345:27101 67.05389:12955 68.04829:6388 69.06868:8131 72.04369:11242 79.05463:9584 81.06989:11015 106.06411:6356 107.08485:7663 114.97045:25386 115.05257:138336 128.95062:7462 142.06203:38507 146.95981:6089 170.05659:71694 | -3.65E-06 |
| POS1537 | Indole-3-carbinol | 4.423 | 130.06456 | [M+H-H2O]+ | 130.0647 | 54.64017:6537 55.05366:26758 56.04863:30718 57.03287:8398 57.06896:80411 67.05389:15137 74.09583:23880 77.03802:23987 84.04317:38555 84.07957:69391 87.00325:14012 95.04894:33089 103.05422:81566 104.46147:6462 105.04402:6683 107.84089:5992 113.96312:8532 128.04967:9327 129.99472:6496 130.06477:306382 130.15721:27895 132.94377:5972 | -1.08E-06 |
| POS2015 | METHYLQUINOLI | 4.419 | 144.08061 | [M+H]+ | 144.0808 | 52.60244:6370 53.03774:28367 55.0177:33930 55.05366:27428 56.04863:16803 57.03287:18875 57.06989:8537 63.06548:5772 69.03294:31338 70.06431:164053 71.04816:39263 77.03802:7577 79.05313:6114 81.0323:11294 81.0699:16849 84.04482:7076 89.03731:7724 91.0539:29775 91.36633:5304 95.04894:11468 98.06007:87031 98.09554:47486 99.0434:52100 103.05422:41978 115.05257:81645 116.04757:10636 117.06913:139579 118.06566:6036 126.09015:33512 128.04655:8832 142.06567:6482 143.0343:41579 143.07104:94184 144.0797:393521 145.06528:22824 | -1.32E-06 |
| POS2016 | cyclohexanecarbo | 1.135 | 144.1013 | [M+H]+ | 144.10181 | 53.03773:16288 55.0177:23838 55.05365:24757 56.04952:14732 58.06416:96575 61.03869:14347 69.03294:9637 70.06429:72063 71.04815:19953 72.08047:12728 81.06832:10240 84.04315:21596 84.07957:63766 98.06005:49924 98.09551:32076 99.04338:17761 102.05497:7220 112.20184:5816 125.72906:6288 126.09013:10892 143.03059:7747 144.10196:150958 | -3.54E-06 |
| POS1628 | DL-Norleucine | 1.255 | 132.10962 | [M+H]+ | 132.11 | 55.0177:12744 55.05365:12696 56.04952:17870 57.05692:42131 58.06416:46069 61.01003:9893 69.03417:12424 69.06867:466009 72.04369:7878 73.06432:9978 86.0954:2907210 87.09917:12270 90.05456:82747 132.07564:17170 132.10173:22504 | -2.88E-06 |
| POS1619 | 6-Methyl indole | 4.422 | 132.08081 | [M+H]+ | 132.0809 | 58.06512:12753 58.07272:6489 69.03294:22883 69.06992:48831 77.03802:12116 79.05313:14901 86.05939:13680 86.09541:283147 88.00349:10381 91.0539:15793 93.05684:11005 95.04894:24133 97.00919:8706 97.77084:5883 103.05422:70909 105.06946:50995 106.06412:5359 115.05257:103497 117.05553:226174 130.06477:29594 131.07088:29487 132.07892:233975 | -6.81E-07 |

**Differences in metabolites between the Model group and the Control group**

| Alignment ID | Metabolite name | Rt(min) | Expreiment Mz | Adduct type | Reference m/z | MS/MS spectrum | PPM |
|---|---|---|---|---|---|---|---|
| POS5544 | Phenylalanylprolin | 4.435 | 263.13757 | [M+H]+ | 263.13898 | 70.06431:91480 84.04317:22235 85.04652:13408 86.09541:49676 87.09918:29051 116.06905:174861 120.08031:205804 121.08413:11874 123.11524:9314 132.10175:5500 133.10522:7109 | -5.36E-06 |
| POS7887 | exapropylene glyc | 5.061 | 367.26733 | [M+H]+ | 367.26898 | 57.03286:154690 58.18789:5729 59.04858:1746998 60.05225:23401 73.06431:53570 85.02798:7284 87.04333:17183 89.05895:16939 97.06255:5646 99.07938:8648 103.07443:6641 115.07377:10087 117.0528:9869 117.09088:115985 131.07086:19714 167.31822:5942 173.3911:11814 175.13008:6860 226.5341:6140 333.52783:5814 | -4.49E-06 |
| POS211 | Cyclopentylamine | 2.203 | 86.09637 | [M+H]+ | 86.09643 | 50.39038:12062 56.04863:92328 57.05693:146946 58.06512:20445 67.05389:15821 69.06868:112689 86.05939:66632 86.09541:77074 | -6.97E-07 |
| POS1231 | Hydroxybenzonitr | 4.774 | 120.04428 | [M+H]+ | 120.04438 | 56.04952:7750 56.05764:13294 61.03972:18567 65.03804:121006 70.39166:5450 72.08047:8558 73.08444:14919 91.0539:6993 92.0491:83508 103.05421:17259 111.67029:5583 120.04357:20325 120.0803:7590 | -8.33E-07 |
| POS11420 | yl)-sn-glycero-3- | 6.402 | 550.38617 | [M+NH4]+ | 550.38672 | 60.08024:15426 86.0954:27092 104.10696:144095 105.10878:7529 124.99954:7469 184.07465:57400 | -9.99E-07 |
| POS6248 | Testosterone | 5.518 | 289.21558 | [M+H]+ | 289.21619 | 55.05363:9679 67.05386:9397 69.06989:12175 79.05309:7681 81.06986:10555 83.04929:11954 84.03651:6250 86.09537:22617 93.06837:6272 97.06456:156759 98.06837:6410 104.10692:12893 105.0694:9938 109.06331:108771 110.33407:6009 123.07998:6579 130.99672:6339 261.53647:5972 | -2.11E-06 |
| POS6956 | Phytosphingosine | 5.034 | 318.29956 | [M+H]+ | 318.2999 | 55.05365:10813 57.06896:194439 58.06417:28443 69.06992:9206 70.06431:246436 71.08419:70457 72.08048:10266 84.07957:5835 85.10045:16514 86.09541:6291 88.07445:437164 95.08477:6332 102.09042:259378 132.10175:18678 146.11787:10681 150.11266:5960 256.25848:170371 257.26578:9467 318.29404:57728 | -1.07E-06 |
| POS667 | -5-methyl-2(3H)-f | 0.659 | 101.05946 | [M+H]+ | 101.06026 | 53.03773:8754 55.0177:20315 55.05365:102916 56.04861:7906 57.06988:6898 59.04858:34741 60.04425:6534 72.04368:6808 83.04768:6817 100.07381:13531 | -7.92E-06 |
| POS1743 | Adenine | 8.871 | 136.06134 | [M+H]+ | 136.0619 | 76.50495:5886 81.0699:10792 91.05389:60378 94.04008:8470 118.0629:7499 119.0359:17263 136.02158:20705 136.05905:64464 137.04602:8767 | -4.12E-06 |
| POS3818 | Dodecanamide | 6.145 | 200.19971 | [M+H]+ | 200.20081 | 55.05365:18749 57.06895:50255 69.06867:10385 71.08546:7644 74.06022:10785 75.65979:5727 81.06989:6977 88.07444:49109 102.09041:17858 116.10664:6624 124.89156:5987 148.61507:5308 200.19798:42427 | -5.49E-06 |
| POS10162 | 20:4(8Z,11Z,14Z,1 | 6.116 | 502.29059 | [M+H]+ | 502.2926 | 55.01769:23744 55.05364:63581 57.03286:67470 57.06895:32465 61.71925:6370 62.06013:14088 67.05388:168157 69.06866:79820 71.04815:6325 71.08418:14996 73.02811:11585 75.04311:7844 77.03801:6084 79.05311:98005 81.06831:127336 83.04768:6810 83.08508:26961 85.06335:24850 85.10043:7537 91.05389:118244 93.06839:110439 95.04892:7717 95.08475:122202 97.06459:31760 97.10156:10780 105.06944:84821 107.08484:69844 109.10004:38606 117.06911:40537 119.08331:103327 121.10127:61898 123.08001:13187 123.11521:9795 129.05444:51851 131.08374:35621 133.0986:52981 135.07933:6474 135.11642:19200 137.13217:8902 143.08571:12809 145.09901:19486 147.11679:20481 149.12999:8337 157.09772:19947 159.1172:6413 161.13194:9723 171.11403:13829 175.145:6167 203.17519:26631 210.12161:6896 240.12112:5812 | -4E-06 |
| POS9474 | yl-sn-glycero-3-p | 5.96 | 468.3049 | [M+H]+ | 468.3078 | 57.03287:56976 57.06896:50087 58.06512:15779 59.04859:57555 60.08025:282939 67.05389:10296 71.07261:27548 71.08548:30422 73.02812:11311 81.0699:14310 85.10046:11116 86.09541:607746 89.05896:29623 95.08478:18964 97.67747:9594 101.05931:14140 104.10697:3224918 105.10879:76323 109.10007:10339 115.07378:8659 124.99956:154054 144.00543:8513 163.01524:17084 184.07466:1604689 185.07635:49195 285.23489:8885 348.21555:9886 | -6.19E-06 |
| POS2275 | Guanine | 4.375 | 152.05612 | [M+H]+ | 152.05659 | 56.04953:6057 67.05389:8330 79.05312:6622 79.78424:6180 96.08018:5419 107.04917:9919 109.05113:5482 110.03356:28966 110.06084:6528 121.03835:5855 135.02879:17398 152.0549:30094 153.03816:28975 | -3.09E-06 |
| POS6299 | Inosine | 4.194 | 291.06769 | [M+Na]+ | 291.07001 | 103.31759:5482 138.38226:5620 159.02675:44285 | -7.97E-06 |

| Differences in metabolites between the Model group and the Control group | | | | | | | |
|---|---|---|---|---|---|---|---|
| Alignment ID | Metabolite name | Rt(min) | Expreiment Mz | Adduct type | Reference m/z | MS/MS spectrum | PPM |
| POS7731 | racosahexaenoic a | 5.942 | 357.2774 | [M+H]+ | 357.27859 | 55.05365:82340 57.06896:8984 62.52579:6246 67.05389:76133 69.06992:33518 79.05312:27449 81.0699:207516 82.07301:8125 83.04932:10956 83.08509:94266 85.06337:25760 91.0539:24546 93.06841:117068 95.08477:229277 96.08827:6720 99.04339:41540 101.0593:51827 105.06945:86914 107.08486:163615 109.10006:140914 115.07378:9366 119.08333:90308 121.1013:142247 123.08004:8885 123.11523:34384 131.08377:22886 133.09863:107850 135.11646:174219 137.09428:11864 145.09904:32009 147.11681:123491 149.13002:90343 150.13635:7744 151.10901:11390 159.11723:36301 161.13197:136473 162.13377:8170 163.11809:15359 163.14494:41743 167.10463:17815 173.13152:22906 175.14503:66743 177.12804:15544 179.10373:8325 181.12314:20871 187.14839:15478 189.166:24644 193.12155:9220 195.13722:18504 201.16266:44090 207.13823:11848 215.17596:30307 221.1546:10218 229.20091:12362 | -3.33E-06 |
| POS2630 | -Hydroxychromor | 5.279 | 163.03874 | [M+H]+ | 163.039 | 56.07296:6261 57.974:7314 77.03802:18561 79.05312:11146 92.02633:7193 95.04893:39622 105.04401:13257 133.02939:17424 135.04227:13756 163.03757:33028 | -1.59E-06 |
| POS4227 | Tetradecylamine | 5.134 | 214.25204 | [M+H]+ | 214.25288 | 57.06989:31070 71.08547:7581 81.71648:5671 192.96027:5735 214.25024:9807 | -3.92E-06 |
| POS3477 | N-Acetylglutamine | 1.584 | 189.0865 | [M+H]+ | 189.08698 | 55.93435:12320 56.04862:10246 60.04426:12610 61.03972:5803 72.93707:6110 83.05907:8824 84.04316:249315 84.07957:88188 101.07021:7509 102.05499:7050 105.53591:5286 126.09014:23861 129.88974:5816 130.04884:153460 173.39601:7986 | -2.54E-06 |
| POS1335 | Nicotinic acid | 2.61 | 124.03905 | [M+H]+ | 124.03928 | 53.03856:17751 78.03358:12060 80.04813:95435 83.04768:6157 95.04693:8071 96.04377:52175 112.03866:13916 123.05363:35771 124.03866:11491 | -1.85E-06 |
| POS1232 | Hydroxybenzonitr | 4.466 | 120.04428 | [M+H]+ | 120.0447 | 56.04953:7283 56.05674:11034 59.04956:10983 61.03869:30175 61.92805:6124 65.03804:142129 72.08047:7094 73.0831:14251 73.53333:6277 77.03802:14026 91.0539:30732 92.0491:98533 93.06841:16735 95.04893:27270 103.05421:118034 110.06084:7522 120.04357:20188 120.0803:65183 | -3.5E-06 |
| POS4539 | zyldimethyl ammo | 6.524 | 226.15863 | [M]+ | 226.159 | 134.09351:18333 226.16113:12733 | -1.64E-06 |
| POS2555 | ino valeric acid be | 1.373 | 160.1326 | [M+H]+ | 160.13318 | 55.05365:321601 58.06512:7390 59.04858:53601 60.08025:180335 64.59314:6062 70.06557:14093 83.04932:34086 101.0593:79347 116.04757:13912 132.07892:6734 160.07417:6735 160.13509:16309 | -3.62E-06 |
| POS10531 | nitoylphosphatidyl | 8.79 | 518.31934 | [M+Na]+ | 518.32172 | 56.04953:8669 57.03287:23127 57.06896:76608 58.06512:6933 60.08025:35811 67.05389:11238 69.06992:16160 71.0726:25952 71.08547:60934 81.0699:18773 83.08509:27941 85.10045:39284 86.09541:370488 87.09918:9280 95.08477:28970 97.09953:9473 103.07444:6091 104.10696:1537859 105.10879:43020 109.10006:22270 123.11523:10211 126.02023:9904 146.97894:429330 164.99086:6845 260.09827:7101 313.26874:12736 337.16791:6369 | -4.59E-06 |
| POS3580 | ydroxyindoleacetic | 2.976 | 192.06497 | [M+H]+ | 192.06599 | 57.31232:5637 146.05719:13051 | -5.31E-06 |
| POS7939 | -Tetradecenoylcar | 5.229 | 370.29395 | [M+NH4]+ | 370.29575 | 55.05365:12123 57.03286:31568 57.06989:16170 59.04858:7729 60.08025:297746 67.05389:21597 69.06868:27238 81.06989:15191 83.08509:14885 85.02799:1552989 86.03024:22780 89.05895:7308 93.06841:11323 95.08476:17975 97.09953:10649 103.03848:7755 107.08485:12694 109.10006:25355 121.10129:28425 123.11523:8170 135.11644:19502 144.10197:31085 191.17667:11347 209.18933:10597 | -4.86E-06 |
| POS7318 | Subamolide C | 6.06 | 337.27145 | [M+H]+ | 337.2731 | 55.05366:32691 57.03287:20626 57.06989:10268 59.04956:6694 67.05389:48472 69.06868:26923 79.05313:13135 81.06834:40722 83.0851:14560 91.05204:7645 93.07034:9117 95.08477:30326 97.09953:7279 107.08486:10438 109.10007:15247 121.1013:6150 | -4.89E-06 |
| POS5679 | Adenosine | 3.861 | 268.10361 | [M+H]+ | 268.10397 | 55.0177:8560 55.05365:6074 57.03287:16050 84.84476:5419 136.05907:711106 137.04602:243689 180.67375:6017 | -1.34E-06 |
| POS4408 | ydroxy-L-tryptop | 3.106 | 221.09062 | [M+H]+ | 221.0918 | 55.93435:7421 58.06511:9653 74.00551:5496 79.55973:6350 89.96104:8886 102.58624:6205 116.97397:6505 130.06477:35503 132.04306:12425 157.0766:14875 158.05783:16085 175.08531:10770 | -5.34E-06 |

| Differences in metabolites between the Model group and the Control group | | | | | | | |
|---|---|---|---|---|---|---|---|
| Alignment ID | Metabolite name | Rt(min) | Expreiment Mz | Adduct type | Reference m/z | MS/MS spectrum | PPM |
| POS2089 | Spermidine | 0.914 | 146.16499 | [M+H]+ | 146.16518 | 56.04953:17737 58.06512:12252 67.0539:7871 72.08048:77083 73.6934:6841 79.67875:5981 84.04482:6788 84.08124:191994 112.11259:10999 | -1.3E-06 |
| POS1431 | a-4,5-cyclopropyl | 1.204 | 127.03858 | [M+H]+ | 127.0389 | 53.03773:67161 55.0177:76953 55.05365:16891 55.21807:6225 57.03286:15598 57.06896:6209 67.05389:7619 71.04816:11899 78.9069:5679 80.04814:13044 81.03229:20049 81.04325:8220 81.06989:6367 82.06503:15826 108.04319:6029 109.02667:13298 111.38697:4780 127.03809:65632 | -2.52E-06 |
| POS12449 | 33,36-Dodecaoxa | 4.547 | 591.35876 | [M+H]+ | 591.35858 | 73.06432:125073 87.04334:743459 89.05896:5522730 90.0619:104323 117.09089:82210 130.08708:36141 131.06766:189581 133.08543:2375642 134.08684:68181 175.09526:47103 177.11285:303644 | 3.044E-07 |
| POS11312 | | 4.546 | 547.32977 | [M+H]+ | 547.32977 | 73.06432:77080 87.04334:310689 89.05896:3034282 90.0619:56261 91.07443:15872 107.06821:26790 117.09089:29974 131.07088:55766 133.08543:1045644 134.08684:29346 175.09526:16421 177.11285:105917 | 0 |
| POS8525 | ol 3-beta-D-gluco | 5.159 | 411.27438 | [M+2H]2+ | 411.27451 | 57.03287:106701 59.04859:2423900 60.05226:58860 65.04819:49767 69.06868:24429 73.02812:203056 73.06432:45534 80.0543:28441 81.0699:10650 83.04932:11338 85.06337:50911 87.04335:255244 87.06254:93632 87.07999:40629 89.05896:1724694 90.06191:50889 94.06947:59301 101.05931:186742 102.06606:20714 103.07444:447298 104.0796:11406 107.07059:15977 109.0756:40860 115.07378:18653 116.08248:24210 117.05281:16563 117.0909:23711 129.08911:29662 131.06766:24305 133.08545:471122 134.08684:21539 143.1078:10447 145.08403:14879 147.10149:192488 161.11441:12769 177.11285:35988 191.1256:28252 | -3.16E-07 |
| POS10377 | CHEBI:68981 | 4.652 | 511.30283 | [M+H]+ | 511.30289 | 69.03294:54910 73.02811:290002 73.06432:126431 80.05429:70564 81.03229:42121 87.04334:557306 89.05896:2868972 90.0619:147150 91.04643:91641 92.64947:36836 95.04893:132782 97.53043:33536 99.04339:71888 102.06606:61822 104.827:36803 111.04441:53212 113.05927:114186 131.06766:70072 133.08543:985809 134.08684:71800 137.05637:55345 177.11285:107996 288.22748:36591 479.31699:35690 | -1.17E-07 |
| POS8071 | Ile Val Phe | 5.563 | 378.23935 | [M+H]+ | 378.2392 | 53.43733:6285 55.05365:90186 56.04863:21368 57.06896:23453 67.04092:11340 67.05389:134907 69.06868:63033 70.06431:61804 75.76308:6507 79.05312:83881 80.04814:33789 81.06834:101400 82.06343:365326 83.06721:12639 83.08509:32106 84.07957:8502 91.0539:54314 93.06841:110549 94.06359:41593 95.04893:5641 95.08477:93567 96.08018:77879 97.10159:9417 98.98415:8204 105.06945:31175 107.08486:55798 108.07938:16346 109.10007:42081 110.09557:10276 119.08333:18474 119.29852:6083 121.1013:43555 123.11523:9738 124.10991:6794 133.09863:18013 135.11646:16218 138.67992:6402 147.11681:12248 149.13002:16616 161.13197:8614 166.16684:5424 196.27794:6256 208.3924:6424 262.25568:184124 263.25272:19304 | 3.966E-07 |
| NEG3390 | dihydrocanadenso | 0.969 | 211.09708 | [M-H2O-H]- | 211.09711 | 79.05367:6003 93.03925:886889 95.03763:862836 131.2403:23563 154.2491:6626 | -1.42E-07 |
| POS9633 | yonarasterol I | 5.152 | 477.31287 | [M+2H]2+ | 477.3129 | 57.03287:56982 58.04137:15518 59.04859:1226660 60.05226:30691 65.04819:23821 69.06868:19204 73.02812:145377 73.06432:36640 80.0543:48872 83.0477:10988 85.06337:37427 87.04335:209820 87.06079:61516 87.07999:30585 88.04605:9002 89.05896:1451464 90.06191:61655 94.06947:30623 101.05931:131752 102.06606:25430 103.03849:14083 103.07444:283960 104.07732:12882 105.09028:8596 107.07059:14672 109.0756:35061 115.07378:10308 116.08248:15275 117.0909:23098 124.08022:8919 127.07501:8106 129.08911:26603 131.06766:22858 133.08545:470848 134.08684:22171 140.09358:8084 145.08403:12423 147.10149:127214 161.11441:8142 177.1078:47142 191.1256:22526 | -6.29E-08 |
| POS8061 | Desoximetasone | 4.592 | 377.21191 | [M+H]+ | 377.21201 | 74.06023:8026 89.05896:13965 91.90891:6126 377.22052:9604 | -2.65E-07 |

| Differences in metabolites between the Model group and the Control group | | | | | | | |
|---|---|---|---|---|---|---|---|
| Alignment ID | Metabolite name | Rt(min) | Expreiment Mz | Adduct type | Reference m/z | MS/MS spectrum | PPM |
| POS2774 | thyl-2-prenylthiop | 3.379 | 167.08884 | [M+H]+ | 167.08881 | 77.03802:33641 79.05312:119463 80.05737:18155 91.0539:73080 92.05668:7771 93.06841:196037 94.07143:32746 95.04893:37463 103.05421:809050 104.05679:150822 105.04401:8951 107.04917:92188 108.05284:10313 118.0629:8015 120.06335:61653 120.0803:3048589 121.08412:632523 122.0861:7363 131.04832:35670 | 1.795E-07 |
| POS4916 | Securinine | 8.805 | 240.09859 | [M+H-H2O]+ | 240.09869 | 103.37626:5837 116.44865:5990 118.20353:5371 124.99955:6414 173.43526:9234 196.63867:5118 | -4.16E-07 |
| POS8276 | Xanthoangelol I | 4.483 | 393.20648 | [M+Na]+ | 393.20639 | 80.38605:5492 179.43362:6023 183.31004:6171 289.0278:6119 | 2.289E-07 |
| POS9123 | etyl-leu-leu-tyr-a | 4.795 | 449.27011 | [M+H]+ | 449.26999 | 79.14378:5756 87.04335:5503 89.05896:16159 173.38622:10757 326.5238:6411 343.86096:5698 363.62698:6417 | 2.671E-07 |
| POS12359 | Leu Leu Lys Gln Gly | 5.427 | 586.39203 | [2M+H]2+ | 586.39221 | 57.03286:37116 59.04858:574104 65.04817:18765 73.02811:60325 73.06431:19873 85.06504:20511 87.04333:188581 87.06252:27189 87.07997:23055 89.05894:527083 101.05929:118601 103.07442:134199 115.07642:16951 117.09087:15496 129.0891:22851 133.08542:178858 145.08777:13629 147.10147:63098 177.11282:15666 191.12556:18190 192.67273:16708 234.40762:17853 | -3.07E-07 |
| POS9460 | acetildenafil | 4.633 | 467.27637 | [M+H]+ | 467.2764 | 73.02811:107851 73.06432:42993 75.91343:15367 80.05275:28522 81.03229:26734 87.04334:210130 89.05895:984995 90.0619:60965 91.04643:42961 95.04694:39370 99.04339:29260 102.06827:17648 111.04189:42770 113.05927:26667 131.07086:17292 131.7048:16921 133.08543:356423 134.08684:39526 137.0598:27111 155.07083:15947 177.11284:33955 391.74057:15221 | -6.42E-08 |
| POS12534 | | 4.934 | 595.3631 | [M+Na]+ | 595.36292 | 59.82801:13607 62.53004:12277 100.68073:11281 135.33597:12776 148.35861:11993 420.52808:15110 569.88092:12102 | 3.023E-07 |
| POS4287 | lpha-D-galactopy | 1.025 | 217.06792 | [2M+H]+ | 217.06799 | 52.17733:5754 62.98166:16407 70.06431:8139 75.71778:6086 80.94776:1450871 85.028:29720 90.97558:243299 134.10017:5273 160.03503:14015 | -3.22E-07 |
| POS6547 | Salidroside | 4.947 | 301.12735 | [M+Na]+ | 301.12729 | 51.83899:6899 55.01768:66130 57.06894:396680 71.01213:84376 81.06987:12902 83.01192:42239 83.04766:8314 85.06333:185974 86.06792:5770 109.06332:17662 111.04185:16356 113.02309:321047 114.0258:7122 115.03928:25477 125.02351:16579 127.07495:203994 128.07762:7579 139.0383:13773 139.07704:9267 159.09993:459001 160.10455:22396 167.035:13795 167.10457:8249 171.09958:19797 173.38614:7900 | 1.993E-07 |
| POS8731 | 3,4,6,7,8,8a-hexahy | 5.403 | 425.29007 | [M+2H]2+ | 425.29001 | 57.03287:84902 59.04859:1929612 59.05931:49421 60.05226:47566 65.04819:13475 69.06868:16412 73.02812:80386 73.06432:29815 81.0699:7680 83.0851:8116 85.06337:30340 87.04335:141455 87.06254:30547 87.07999:32010 89.05896:676639 90.06191:20312 94.06947:46752 99.08151:8114 101.05931:143792 103.07444:347040 104.07732:9876 115.07378:17640 116.08248:11319 117.05281:13965 117.0909:28290 129.08911:17986 131.06766:13510 133.08545:166479 134.08684:10288 143.1078:6873 147.10149:126930 148.1028:7550 161.11441:18634 191.1256:15710 | 1.411E-07 |
| POS13123 | methylviny l)-17-c | 5.318 | 623.40717 | [M+2H]2+ | 623.40704 | 57.03287:38020 59.04859:691674 73.02812:112119 73.06432:31260 87.04335:166710 87.06254:35440 87.07999:27390 89.05896:1062317 90.06191:33289 94.06947:28494 101.05931:97398 103.07444:200628 107.06821:23051 109.0756:29367 126.93054:21131 130.08708:26378 131.06766:38358 133.08545:382463 147.10149:107107 147.47386:22019 177.1078:49877 | 2.085E-07 |
| POS9694 | PC(15:1(9Z)/0:0) | 8.808 | 480.30655 | [2M+H]+ | 480.30661 | 54.75832:6275 57.06896:7381 62.05909:38228 63.97328:6278 69.06992:10406 83.08509:7427 89.78172:6752 100.4252:5806 104.10696:12415 112.92513:6587 153.58037:5681 155.00865:5704 173.44016:13028 174.6582:5647 204.03627:6123 410.89926:6566 | -1.25E-07 |

| Differences in metabolites between the Model group and the Control group | | | | | | | |
|---|---|---|---|---|---|---|---|
| Alignment ID | Metabolite name | Rt(min) | Expreiment Mz | Adduct type | Reference m/z | MS/MS spectrum | PPM |
| POS10797 | Hemiasterlin | 5.6 | 527.35992 | [M+2H]2+ | 527.35999 | 57.03287:145722 59.04859:2215140 59.05931:56826 60.05226:60017 69.06992:30441 73.02812:165016 73.06432:77054 85.06337:64190 87.04335:285695 87.06079:53136 87.07999:88955 89.05896:1224127 90.06191:73138 94.06947:39396 99.07939:22454 101.05931:325858 101.09641:21453 103.07444:665743 104.10697:119447 105.10879:49069 115.07378:60897 116.08248:44570 117.0909:69733 129.08911:65918 131.06766:44171 131.10634:36730 133.08545:425443 134.08684:38044 143.10413:30270 145.08403:24073 147.10149:350106 161.1188:60345 173.11685:32836 177.1078:49793 184.06931:55314 185.07635:21746 186.07535:28736 191.1256:59552 | -1.33E-07 |
| POS10704 | PD173074 | 5.353 | 524.33429 | [M+2H]2+ | 524.33429 | 59.04859:344152 60.08025:793941 71.07261:123900 73.03751:126679 86.09541:1677589 89.05896:278884 104.10697:10252773 105.1111:233840 124.99956:361402 152.55547:117446 184.07466:3732591 248.77914:103774 | 0 |
| POS14421 | G(16:1(9Z)/16:1(9Z | 6.595 | 719.48578 | [M+2H]2+ | 719.48572 | 59.63764:5914 69.55946:7557 153.83411:6111 | 8.339E-08 |
| POS11476 | Leueantine C | 8.805 | 552.32867 | [M+2H]2+ | 552.32867 | 60.08025:21437 86.0954:54921 104.10696:312401 184.07465:96514 | 0 |
| POS9671 | bipinnatone A | 4.727 | 479.27853 | [M+H]+ | 479.2785 | 69.59312:5999 248.1651:5493 | 6.259E-08 |
| POS10703 | Pyrichalasin H | 8.806 | 524.30072 | [M+2H]2+ | 524.3006 | 60.08022:679045 80.43092:138712 86.09536:1490409 104.10692:7351334 124.99949:304914 184.07457:2358259 293.82486:116712 | 2.289E-07 |
| POS9565 | droxy-5-[(3Z)-5-h | 4.513 | 474.28506 | [M+2H]2+ | 474.28503 | 62.01709:7800 67.75507:8760 70.06428:9745 74.05883:14795 84.04313:19873 86.09538:115987 87.09915:8524 97.10977:7069 101.06799:8509 110.30173:7219 115.08699:8192 120.08026:150606 121.08408:12895 132.10168:25525 161.89456:9129 173.39105:17718 238.93481:8120 | 6.325E-08 |
| POS1651 | Butoxyacetic acid | 4.956 | 133.08571 | [M+H]+ | 133.0858 | 58.06511:9057 69.06867:30295 86.09539:147379 87.09917:10505 89.00121:6080 132.52338:5562 | -6.76E-07 |
| POS5483 | 8-Deoxy-lactucin | 4.64 | 261.11411 | [M+H]+ | 261.11401 | 51.75893:6842 53.03854:10056 55.01768:12436 55.05363:7024 57.03377:15202 57.06894:491758 58.07269:11803 59.04856:6527 61.01:37998 67.05386:8968 69.06989:22516 71.01213:14176 79.05309:11797 81.03226:150374 81.06987:15602 83.04766:31896 85.06333:227721 95.0111:8694 97.06457:9527 107.04913:7432 111.04436:49075 125.05955:159609 127.07495:17739 133.0656:113285 137.09422:7017 167.10457:710584 168.11116:45533 179.10365:8088 185.0979:14064 195.10202:11404 197.1132:11825 213.09639:9178 231.10547:13816 | 3.83E-07 |
| POS9695 | ysoPE(18:1(9Z)/0:0 | 5.375 | 480.30927 | M+CH3OH+H]2 | 480.3093 | 57.03287:22287 59.04859:253173 60.05226:7475 67.015:16773 73.02812:27236 73.06432:11574 78.30522:6307 81.03072:10941 85.06337:11317 87.04335:33369 87.06254:6825 87.07999:6875 89.05896:199777 90.06191:11231 101.05931:19575 103.07445:52426 117.05281:24502 117.0909:5638 131.07088:12578 133.08545:59241 136.66437:5460 147.10149:20882 347.26852:6725 | -6.25E-08 |
| POS10699 | Calcimycin | 5.474 | 524.27399 | [M+H]+ | 524.27399 | 59.04856:33682 60.08023:26828 62.05907:68444 67.05387:32624 69.06989:11172 79.05461:18944 81.06988:20294 86.09538:48259 87.04332:10912 89.05893:38101 91.05387:18221 93.06838:15104 95.08474:12806 104.10693:269808 104.94925:11291 105.06942:14786 184.0746:151074 278.50723:12180 421.39975:12577 | 0 |
| POS14554 | Argentinic acid G | 5.168 | 733.48761 | [M+H]+ | 733.48767 | 57.03379:50785 59.04859:896524 73.06432:52556 87.04335:127946 89.05896:811414 101.05931:153641 103.07444:313097 117.05281:38620 133.08545:230489 147.10149:106157 302.83173:31899 437.43027:38567 712.33484:42054 | -8.18E-08 |
| POS10326 | Stellettin I | 5.493 | 509.32632 | [M+2H]2+ | 509.32639 | 57.03287:24173 59.04859:232241 67.015:16511 73.02812:19835 81.03072:14771 83.22678:5555 87.04335:25750 87.07999:8051 89.05896:150122 90.06191:7309 94.06555:6141 101.05931:30496 103.07444:67063 104.10697:9472 115.07643:5774 117.05281:6304 117.0909:6914 133.08545:34909 147.10149:21165 158.09198:7607 165.10016:7149 211.56157:5429 | -1.37E-07 |
| POS9623 | hyl-but-2-enyl)-d | 4.476 | 477.22696 | [M+H]+ | 477.22711 | 80.10661:5961 87.04333:27250 89.05895:367847 90.0619:23458 112.07433:24636 119.08611:15831 133.08543:138518 134.08682:15450 177.11284:21139 178.11942:7388 | -3.14E-07 |

| Differences in metabolites between the Model group and the Control group | | | | | | | |
|---|---|---|---|---|---|---|---|
| Alignment ID | Metabolite name | Rt(min) | Expreiment Mz | Adduct type | Reference m/z | MS/MS spectrum | PPM |
| POS11020 | PC(16:1(9Z)/2:0) | 5.39 | 536.33325 | [M+H]+ | 536.33313 | 59.04859:185890 60.08025:33364 73.02812:25239 86.09541:77299 87.04335:36444 89.05896:239720 90.06191:16693 101.05931:26606 103.07445:42881 104.10697:422972 124.99956:16040 133.08545:71047 147.10149:19161 184.07466:172538 290.39401:12274 | 2.237E-07 |
| POS8677 | Formacidine | 5.227 | 422.26428 | [M+2H]2+ | 422.26428 | 57.03286:10914 59.04858:108569 67.01499:17543 81.03072:6926 87.04334:7213 89.05895:80268 101.0593:8728 103.07443:22231 133.08543:26469 147.09764:8730 165.10014:6089 180.10658:5913 252.63242:5638 293.42517:5984 | 0 |
| NEG4912 | Hydroprene | 1.052 | 265.21741 | [M-H]- | 265.21741 | 54.30708:6028 59.08406:7086 89.13129:326629 90.13491:13540 93.0393:178610 95.03768:354553 97.0375:16669 121.99403:5086 147.15923:83595 149.16045:35272 154.19572:6567 169.77672:5477 220.4525:5858 | 0 |
| POS6609 | Cumyluron | 4.77 | 303.12589 | [M+H]+ | 303.12589 | 57.06894:245511 58.07269:8180 59.58425:5492 61.01:39462 81.03226:18130 81.06987:16570 85.06333:115257 95.0489:5669 109.06332:9289 111.04185:6901 113.05923:26071 125.05955:13986 127.07495:10276 133.0656:13185 137.05975:93128 167.10457:94935 221.1192:18987 | 0 |
| POS10094 | Cucurbitacin S | 4.964 | 499.30594 | M+CH3OH+H] | 499.30579 | 57.03287:19243 59.04859:263512 60.08025:13730 73.02812:14774 73.06432:10015 85.06337:9587 86.09541:33442 87.04335:970332 87.09918:10552 88.04783:14431 89.05896:214713 101.05931:219429 103.03849:77055 103.07444:60662 104.10697:123746 105.10879:47566 106.11105:13063 115.07643:10213 117.05281:72333 131.06766:30221 133.08545:61663 147.10149:25608 184.07466:32565 185.07635:14610 186.07535:16191 | 3.004E-07 |
| POS10120 | Aconine | 8.901 | 500.28616 | [M+H]+ | 500.2861 | 55.05453:11157 58.30054:10092 81.0699:10628 86.09541:10895 95.08477:10350 104.10696:42753 105.1111:21941 107.08486:12771 119.08333:15483 126.02023:87930 127.02272:11667 128.01544:40502 159.11723:14402 173.43034:13153 209.13087:13199 227.14313:10869 | 1.199E-07 |
| POS9632 | yonarasterol I | 4.5 | 477.31284 | [M+H]+ | 477.3129 | 69.93977:14540 87.04334:26210 89.05896:391634 90.0619:30627 103.72703:8538 112.07434:12685 133.08543:137001 134.08684:29949 177.11285:12063 419.51126:10238 | -1.26E-07 |
| POS8640 | ethoxy-3-(3-meth | 4.839 | 419.22461 | [M+Na]+ | 419.22476 | 58.04042:12495 59.04859:189004 65.04932:9874 73.02812:51785 73.06432:10804 80.0543:7267 85.06337:8721 87.04335:51168 87.06254:10942 89.05896:257374 90.06375:7579 101.05931:18330 102.06606:13027 103.0385:19246 103.07445:23508 109.0756:6560 133.08545:66767 147.10149:7449 | -3.58E-07 |
| POS10619 | quaesitol | 5.068 | 521.32611 | [M+H]+ | 521.32611 | 59.04858:22204 60.08025:63739 86.09541:93852 87.04334:8058 89.05896:17883 104.10696:380594 108.16389:6635 124.99955:14995 173.39111:8455 182.94283:6709 184.07466:79071 239.22867:6583 282.12573:5632 | 0 |
| NEG8080 | o-7,8-dihydropyra | 1.054 | 387.18332 | [M-H]- | 387.1832 | 65.69628:5658 68.01559:5751 93.03928:352337 95.03767:298038 97.03749:33426 124.15681:11693 151.06717:22659 153.0676:49273 155.06657:11867 358.09082:5782 | 3.099E-07 |
| POS9843 | 3,4,6,7,8,8a-hexahy | 4.43 | 487.26398 | [M+H-H2O]+ | 487.26401 | 64.9233:6323 77.04674:5750 87.04335:9197 89.05896:380439 107.06821:6794 111.12991:6008 118.06566:12199 133.08545:173386 144.0797:15248 146.061:74743 151.09705:6603 159.09138:11369 173.42545:8962 177.11285:29188 188.07004:307841 189.07106:13994 205.09822:63379 283.17694:10805 | -6.16E-08 |
| POS9197 | 4-methyl-9,11-sec | 4.583 | 453.33994 | [M+H]+ | 453.34 | 61.49389:5160 96.08015:43369 100.1125:10595 111.08459:5769 113.10571:19788 114.09121:103674 173.39595:7814 209.16328:26759 228.16107:6962 | -1.32E-07 |
| POS8645 | Denticulatin B | 5.002 | 419.27682 | [M+2H]2+ | 419.27682 | 59.04858:171300 73.02811:40529 73.06432:7607 80.0543:12196 87.04334:30343 87.06253:8494 89.05896:235891 90.06191:9288 101.0593:8606 102.06606:6905 103.07444:23919 111.18527:6119 133.08543:44807 137.5574:5651 147.10149:8191 177.11285:9764 188.64743:7025 | 0 |
| POS8140 | Lehualide D | 4.729 | 383.2251 | [M+H]+ | 383.22501 | 57.03287:10409 59.04859:284434 69.79429:8099 70.09203:5685 73.02812:18017 77.05836:5557 83.1274:6299 84.20721:5229 87.04335:481519 89.05896:167555 96.48599:5631 101.05931:64745 103.03849:237185 103.07444:42981 107.06821:7106 117.05281:78851 131.07088:12359 133.08545:40764 147.06319:6251 147.10149:13324 186.17351:5380 | 2.348E-07 |

**Differences in metabolites between the Model group and the Control group**

| Alignment ID | Metabolite name | Rt(min) | Expreiment Mz | Adduct type | Reference m/z | MS/MS spectrum | PPM |
|---|---|---|---|---|---|---|---|
| POS9785 | malyngamide I | 6.112 | 484.28232 | [M+H-H2O]+ | 484.28238 | 59.04856:15166 62.05907:9682 71.34617:6384 104.10693:11735 184.07458:11252 190.59322:5859 | -1.24E-07 |
| POS9100 | nethyl-7,11-dioxo | 5.643 | 447.30978 | [M+H]+ | 447.30981 | 57.06896:13752 59.04858:36661 89.05896:16814 97.06461:16142 101.0593:8015 103.07444:7241 121.0641:6520 159.11293:6657 | -6.71E-08 |
| POS12414 | Nephilatoxin 1 | 5.693 | 589.38202 | [M+2H]2+ | 589.38202 | 57.03287:29722 57.52443:16027 59.04859:194931 65.56047:14424 67.015:16832 70.43989:14676 73.02812:27593 73.06432:18286 87.04335:26774 89.05896:153815 101.05931:29506 103.07444:60804 105.5429:13858 133.08545:52561 147.10149:17220 309.90234:13809 412.1004:13169 | 0 |
| POS7203 | a,4,5,8,9,12,13,15a | 5.222 | 331.18921 | [2M+H]+ | 331.18909 | 53.34515:5668 66.18044:7608 67.05388:8402 81.06989:11972 88.69724:5445 90.57806:4990 95.08475:11984 97.06458:6005 105.06943:6124 107.08484:6942 121.06407:28359 133.0986:6886 147.07846:6495 177.55913:6473 | 3.623E-07 |
| POS10191 | droxy-6-methylhe | 6.221 | 503.29526 | [M+H]+ | 503.29541 | 59.04858:9200 65.70778:5439 86.0954:8650 89.05895:12061 104.10696:22086 484.58868:5896 | -2.98E-07 |
| POS8041 | roxy-6-hydroxyme | 5.259 | 376.173 | [M+NH4]+ | 376.17303 | 55.0177:9572 55.05365:15226 57.03287:38159 59.04858:26096 61.02743:30522 69.03294:335076 71.04816:16089 73.02812:16384 79.05312:17888 81.03229:46270 83.0477:50525 85.028:47738 87.04334:12683 89.05896:9685 91.0539:660279 92.05669:16433 97.0297:10858 99.0434:14089 103.03849:8446 105.03246:62733 107.04917:113719 111.0419:53141 127.03809:14156 129.05447:72112 147.06317:130369 | -7.98E-08 |
| POS12537 | e-(3beta,5beta,11 | 5.902 | 595.38391 | [M+2H]2+ | 595.38397 | 57.03287:38326 59.04859:187299 73.02812:62898 85.06337:25280 87.04509:37664 87.06254:22316 89.05896:185028 101.05931:43827 103.07444:31021 133.08545:49123 | -1.01E-07 |
| POS10431 | hylhexadecahydro | 5.798 | 514.2829 | [M+H]+ | 514.28302 | 59.04857:35530 85.06335:16559 89.05894:28911 93.07031:6606 95.08475:16434 109.10004:9500 121.10126:9594 126.0202:184390 133.0854:11048 157.10194:9082 159.1172:8808 209.13083:7331 | -2.33E-07 |
| POS7719 | Sterebin M | 4.85 | 356.27875 | [M+H]+ | 356.2789 | 58.06511:235047 59.04858:34692 60.32804:6778 73.06432:7429 81.06989:22804 82.43508:5463 84.07957:11302 89.05895:32250 104.79474:5929 108.885:6196 123.11523:24179 133.08543:7052 140.14343:9963 280.99945:5498 341.80237:6580 | -4.21E-07 |
| POS9736 | nethyl-9-oxo-1,2, | 8.887 | 482.2749 | [M+H-H2O]+ | 482.27484 | 53.99451:6296 71.08418:5443 95.08477:8823 95.8601:5344 104.10696:30712 107.08486:6203 126.02023:43718 127.01965:6483 128.01855:16014 130.86165:5197 412.2981:5842 | 1.244E-07 |
| NEG9815 | Cefpiramide | 5.385 | 611.112 | [M-H]- | 611.112 | 79.61639:5075 89.13125:759890 90.13486:35374 94.03929:5042 117.0695:29805 370.36819:6691 412.74951:5783 440.99393:6109 | 0 |
| POS9810 | Communesin H | 4.826 | 485.29111 | [M+H]+ | 485.29099 | 57.03287:9114 59.04859:208773 76.26552:5650 85.06506:6110 87.04335:26542 89.05897:78871 101.05931:16898 103.0385:10071 103.07445:43505 133.08545:10921 147.1015:9228 161.05295:5750 353.60825:5860 365.49567:5915 372.1275:5635 | 2.473E-07 |
| POS8830 | Kuguacin E | 6.151 | 431.31509 | [M+2H]2+ | 431.31509 | 59.04858:9678 95.08477:12758 97.06461:61890 97.10159:10056 107.08486:11493 109.06336:6145 109.10006:12249 112.9767:5473 121.0641:6371 121.1013:7481 133.10193:8005 135.11646:7058 147.11681:6669 171.11887:5707 177.12804:19253 357.97528:5319 | 0 |
| POS8955 | Anisodorin 3 | 5.696 | 439.3053 | [M+2H]2+ | 439.3053 | 57.03287:46780 59.04859:698843 59.05931:17682 60.05226:21029 73.02812:27360 73.06432:29850 85.06337:9506 87.04335:41472 87.07999:7958 89.05896:167857 90.06191:6728 94.06947:8708 99.07939:6574 101.05931:62274 103.07444:111550 104.07732:7553 115.07378:17486 117.05281:7619 117.0909:20451 129.08911:6502 133.08545:32517 147.10149:33827 161.1188:7787 374.9805:6956 393.72964:6196 | 0 |
| POS15004 | PC(16:0/18:1(9Z)) | 6.475 | 782.56622 | [M+H]+ | 782.56616 | 53.69582:11689 67.33069:12777 67.96278:10387 86.09541:15276 184.07466:34009 184.36464:11386 293.25256:10403 351.13647:10588 | 7.667E-08 |
| POS12390 | yl-sn-glycero-3-p | 5.747 | 588.36383 | [M+H]+ | 588.36401 | 59.04856:36489 60.08022:12272 86.09536:9160 87.0433:13002 89.05891:30567 101.05926:7093 103.07439:13733 104.10692:87875 133.08537:11196 173.39102:11056 184.07457:21898 412.29788:6117 | -3.06E-07 |

**Differences in metabolites between the Model group and the Control group**

| Alignment ID | Metabolite name | Rt(min) | Expreiment Mz | Adduct type | Reference m/z | MS/MS spectrum | PPM |
|---|---|---|---|---|---|---|---|
| POS10255 | Talaroconvolutin E | 5.997 | 506.32382 | [M+H]+ | 506.32379 | 51.79336:6660 53.41721:6159 59.04859:22317 60.08025:12511 66.38443:5356 86.09541:31301 89.05896:18294 104.10697:144927 184.07466:37367 185.39581:4931 201.78914:5385 | 5.925E-08 |
| POS1562 | lichloro-1,1-ethan | 9.248 | 130.96594 | [M+H-H2O]+ | 130.966 | 54.03373:7588 55.93435:6275 56.04952:26633 57.06988:6346 61.02845:6356 67.05389:23205 67.93391:13180 70.06556:7088 71.92826:29503 72.93706:41262 74.99706:7067 77.49896:7387 77.99808:64843 78.9988:8127 84.04315:36824 84.07957:76239 85.0061:22638 85.08359:6166 86.09711:6852 86.50518:17245 87.00323:285725 87.50385:31305 88.00347:191576 89.50647:9359 89.93904:12920 90.94762:9840 91.05389:5831 94.01266:8758 96.00942:20700 96.51448:7032 97.00917:70070 98.51207:53196 99.51075:59623 106.37916:6335 107.94919:7916 113.96311:105942 115.96169:38962 116.97124:12352 126.97046:6999 129.99471:7179 131.97466:30121 | -4.58E-07 |
| POS12327 | kirkinine D | 4.852 | 585.27051 | [M+H]+ | 585.27039 | 55.4905:5598 89.05896:11618 106.74535:6542 133.08545:8463 179.12949:6616 299.14221:7438 | 2.05E-07 |
| POS4670 | Norpterphyllin III | 5.997 | 231.10387 | [M+H]+ | 231.104 | 53.69413:5942 57.06989:12373 74.0616:6065 108.67789:5870 167.10463:11847 179.73859:5568 | -5.63E-07 |
| POS4447 | CHEMBL4283703 | 2.625 | 223.06311 | [M+H]+ | 223.0632 | 58.06511:13469 59.04858:7560 61.03868:29019 66.64188:6661 73.02811:9639 73.04553:12324 80.59386:5975 91.05576:17270 93.06839:6086 209.00749:6987 225.04041:63944 | -4.03E-07 |
| POS4449 | CHEMBL4283703 | 0.702 | 223.06323 | M+CH3OH+H] | 223.0632 | 58.02428:6139 58.06417:21920 59.04859:10298 73.02812:10576 73.04555:18931 76.81198:5763 79.05464:8297 90.48373:5205 91.05577:26695 93.06841:11778 119.04707:16973 155.97054:7677 192.70723:5645 209.00754:13773 225.04045:104121 226.04433:7698 | 1.345E-07 |
| POS304 | Butyric acid | 4.952 | 89.05968 | [M+H]+ | 89.05968 | 61.02845:46841 68.67587:6086 72.93705:39696 87.00497:16898 90.94762:6676 | 0 |
| POS6976 | Tomelukast | 6.311 | 319.17535 | [M+NH4]2+ | 319.1752 | 55.0177:30555 55.05365:23184 57.03287:28828 64.85594:6493 67.05389:24674 69.06868:117672 69.80681:5353 71.04816:10347 79.05312:25104 81.06833:19993 91.0539:35686 93.06841:24821 95.04893:5465 95.08477:9970 97.06461:13405 105.03245:10543 105.06945:39949 107.04917:42940 107.08486:8338 107.37579:5076 117.06912:14013 117.28432:6209 119.08333:29494 121.1013:9240 122.76101:6073 131.08377:5982 133.09863:15446 135.11644:13885 139.11232:14733 143.08574:9419 145.09904:11977 147.07849:24923 161.09683:20508 163.10915:28111 165.09103:7358 206.39116:6086 | 4.7E-07 |
| POS244 | Butanal, 2-oxo- | 2.532 | 87.04405 | [M+H]+ | 87.04405 | 69.03294:23471 69.06869:12170 86.05939:124827 87.04335:8207 87.06254:8704 | 0 |
| POS5757 | Doxylamine | 5.121 | 271.18137 | [M+H]+ | 271.1814 | 59.04859:7989 64.42622:5475 69.06868:5858 75.68808:5550 116.85452:5675 | -1.11E-07 |
| POS10170 | Ala Leu Leu Ala Se | 6.274 | 502.32336 | [M+H]+ | 502.32339 | 74.3554:6617 95.82383:5824 183.62494:5457 | -5.97E-08 |
| POS7539 | Mukoenine B | 6.445 | 348.19626 | [M+Na]2+ | 348.19635 | 57.03287:36244 59.67723:6330 74.65649:6367 252.33946:5698 | -2.58E-07 |
| POS8931 | Nudicaulidine | 6.363 | 438.28555 | [M+2H]2+ | 438.2854 | 51.01141:6861 57.03287:7264 59.04858:31380 338.36777:6055 | 3.422E-07 |
| NEG3711 | Sinapic acid | 1.325 | 223.08005 | [M-H]- | 223.08 | 71.09774:6503 79.05373:9090 84.60606:5587 92.02104:34617 97.07652:33898 106.00214:5626 136.06505:14081 140.30067:5684 165.03516:39688 | 2.241E-07 |
| POS2584 | C)OC(C(CC(C)O)C) | 5.244 | 161.11703 | [M+H]+ | 161.1171 | 52.41368:6692 59.04859:8937 | -4.34E-07 |
| POS8562 | eliosupine N-oxic | 5.663 | 414.21255 | [M+Na]+ | 414.21259 | 77.00605:5821 85.02795:15976 119.08327:28298 173.38612:7537 | -9.66E-08 |
| POS9182 | Melleolide M | 5.746 | 453.16736 | [M+H]+ | 453.16739 | 57.03286:72567 57.94083:5677 59.04858:32735 62.03075:6005 85.02798:7600 99.07938:8882 | -6.62E-08 |
| NEG6125 | Sclareol | 0.754 | 307.27808 | [M-H]- | 307.2782 | 76.37918:6321 76.39638:6059 189.36215:6130 | -3.91E-07 |
| POS11311 | , 24E)-Cholest-24 | 6.26 | 547.2934 | [M+2H]2+ | 547.29352 | 52.92344:5911 60.08025:8338 86.09541:12656 104.10696:89387 105.10879:9411 184.07466:15685 308.05939:6095 | -2.19E-07 |
| NEG1909 | Isatinoxim | 0.179 | 161.0356 | [M-H]- | 161.03571 | 55.08417:6740 57.10281:10574 59.08407:77697 67.31788:5529 71.09903:13234 73.11623:150130 82.68986:5427 85.13133:27001 101.14608:15075 110.22124:7649 113.15997:10825 | -6.83E-07 |
| POS9809 | Ardisiaquinone E | 6.66 | 485.28726 | [M+H]+ | 485.28729 | 55.91729:6877 59.04858:18935 91.0539:15434 133.09863:54886 173.39111:11258 317.47849:6127 | -6.18E-08 |

| Differences in metabolites between the Model group and the Control group | | | | | | | |
|---|---|---|---|---|---|---|---|
| Alignment ID | Metabolite name | Rt(min) | Expreiment Mz | Adduct type | Reference m/z | MS/MS spectrum | PPM |
| POS7167 | naH-cheilantha-13 | 2.593 | 329.32031 | [M+H]+ | 329.32019 | 51.93132:6968 55.05365:11566 57.06896:23455 59.03105:90371 61.01003:62247 62.98918:11778 67.05389:7884 69.06992:13425 71.08419:13075 73.04555:73611 75.02498:108072 76.02578:6615 77.00463:7738 77.04093:83916 79.01993:16467 83.0851:9873 87.06253:6298 91.05577:87400 91.52596:5809 93.03564:90181 95.01511:5977 97.09953:15795 99.86346:5875 105.07177:29441 111.11732:14346 119.08891:6894 149.44341:5671 | 3.644E-07 |
| POS5229 | lamino)phenyl]car | 4.8 | 251.10243 | [M+H]+ | 251.10258 | 59.04858:48481 60.04425:81896 69.06991:8564 70.02779:69771 70.09202:6754 74.02328:16012 81.21274:5624 87.04333:12693 88.03894:227375 89.04271:8569 94.06358:167546 98.02461:9232 99.0074:33563 104.04995:9980 116.03413:7494 117.0528:7389 120.04356:59548 123.03605:5659 130.04883:21732 132.04305:8725 136.0761:17339 146.05719:220239 147.06316:7815 150.05341:12704 158.04501:9386 174.05496:49223 192.06534:8392 214.37151:5709 | -5.97E-07 |
| POS236 | Divinyl sulfide | 1.56 | 87.02609 | [M+H]+ | 87.0262 | 56.04863:17014 57.05693:39967 69.03294:10719 69.06868:27926 86.05939:34811 86.09541:16429 | -1.26E-06 |
| POS6698 | phenyl)-3,4-dihyd | 2.76 | 307.08212 | [M+2H]2+ | 307.082 | 76.03857:7299 84.04314:51760 130.04883:21051 | 3.908E-07 |
| NEG4834 | R),5(R)-bis(hydroxy | 0.661 | 262.16595 | [M-H]- | 262.16611 | 84.03101:7416 100.04375:7708 | -6.1E-07 |
| POS10645 | ysoPC(0:0/18:1(9Z | 6.902 | 522.35522 | [M+H]+ | 522.35522 | 128.86884:5368 184.0692:8379 | 0 |
| NEG1882 | Dihydroxynaphtha | 4.314 | 160.05246 | [M-H2O-H]- | 160.05243 | 57.10281:6266 59.08407:42864 62.57202:5617 73.11623:55262 97.1813:5084 154.52921:4889 | 1.874E-07 |
| POS15135 | 11Z,14Z,17Z)/18:3 | 7.002 | 804.5509 | [M+H]+ | 804.55103 | 173.43526:10543 280.09122:6543 635.19501:6346 | -1.62E-07 |
| POS10277 | Asn Phe Ala Arg | 6.663 | 507.26715 | [M+Na]+ | 507.2673 | 55.42575:7476 59.62183:6501 83.06232:5808 107.32325:7394 158.40836:5391 184.34851:5577 | -2.96E-07 |
| POS15090 | PG 38:7 | 6.733 | 793.5011 | [M+H]+ | 793.50128 | 55.14588:6653 60.88638:5143 66.33102:5834 78.19966:6200 143.06367:6466 160.10027:5317 243.58643:6061 359.39038:5954 611.99011:5960 | -2.27E-07 |
| NEG1883 | Dihydroxynaphtha | 1.377 | 160.05307 | [M-H]- | 160.05243 | 51.09473:5957 57.65318:5568 58.00337:6717 59.08403:31394 70.57812:5376 71.09898:10088 73.11619:47205 73.99634:5682 85.13128:5671 101.14601:5693 150.50673:5630 | 3.999E-06 |
| POS10278 | Amoritin | 6.663 | 507.27118 | [M+H]+ | 507.27121 | 76.2698:6746 149.75777:6022 | -5.91E-08 |
| POS15192 | PC(18:0/18:1(9Z)) | 6.665 | 810.59821 | [M+2H]2+ | 810.59833 | 62.37422:6728 207.28555:5488 | -1.48E-07 |
| POS6798 | 3-hydroxypentane | 4.559 | 311.20444 | [M+H-H2O]+ | 311.20441 | 57.03287:52959 59.04859:2111908 60.05226:27540 73.02812:20430 73.06432:14659 85.06506:30420 87.04335:156446 87.07999:9067 89.05896:2519716 90.06191:43693 101.05931:56081 103.03849:67758 103.07444:277993 107.07059:27440 117.05281:22880 129.08911:11014 131.06766:9861 133.08545:399722 134.08684:16435 147.10149:78077 151.09705:15682 | 9.64E-08 |
| POS13939 | -(28),23-dien-3-o | 8.716 | 679.47772 | [M+H]+ | 679.47791 | 53.85504:6025 129.86432:5667 529.3476:5951 | -2.8E-07 |
| POS14521 | Javanicoside K | 7.456 | 729.29626 | [M+H]+ | 729.29639 | 53.35354:5571 | -1.78E-07 |
| POS2874 | Azelaic acid | 5.981 | 171.10107 | M+CH3OH+H] | 171.101 | 53.03772:6060 55.01768:58998 55.05364:20249 57.06894:642833 58.0727:7355 67.05387:8431 72.04367:23066 79.0531:14963 83.01192:449972 83.08669:5889 85.06334:206456 89.06976:6543 91.05387:11880 93.06838:20720 97.06458:7654 111.07957:14641 115.03664:25092 121.06406:8265 139.07353:9037 | 4.091E-07 |
| POS7967 | 5beta-Coprostano | 0.607 | 371.36737 | [M+H]+ | 371.36722 | 57.06989:9920 59.03105:40356 61.01003:10249 73.04689:37856 75.02498:32068 77.04093:20602 91.05577:34151 93.03564:23727 | 4.039E-07 |
| POS6668 | 3-Oxogeranyllinal | 6.936 | 305.24673 | [M+H]+ | 305.2467 | 52.71404:6652 115.55512:5844 | 9.828E-08 |
| POS4258 | Oxododecanoic a | 5.797 | 215.16423 | [M+H]+ | 215.1642 | 57.03287:10104 66.7261:6671 67.05389:5776 122.57718:6670 142.00749:5780 | 1.394E-07 |
| POS7208 | Tianshic acid | 6.156 | 331.24756 | [M+H]+ | 331.24759 | 55.36465:6892 178.25224:6519 | -9.06E-08 |
| POS13403 | Filixic acid pbp | 6.87 | 641.25934 | [M+H]+ | 641.25922 | 67.85222:5791 86.21047:5133 151.98245:6514 223.58301:5499 | 1.871E-07 |
| POS4256 | Putaminoxin E | 5.609 | 215.16414 | [M+H]+ | 215.16409 | 50.61698:4718 50.62858:5628 50.634:5520 52.61634:5988 59.28901:5307 61.05301:4706 63.6503:4244 65.26049:4134 68.36765:4736 69.36423:4484 70.8761:4970 74.7813:4590 77.10919:3789 79.32551:4591 88.21125:5039 88.39301:4773 92.35327:4313 92.43147:4989 96.87998:4409 98.64025:4532 100.98293:4280 103.63856:4294 104.2325:4271 112.76032:3816 116.427:4465 117.93621:4574 120.90114:4925 160.97826:4331 161.43983:4379 164.86342:5118 178.03259:5728 181.50056:4790 182.55013:3901 195.91809:4328 196.93501:4740 203.49271:4507 208.5345:4770 217.56131:4415 217.65092:4551 | 2.324E-07 |

**Differences in metabolites between the Model group and the Control group**

| Alignment ID | Metabolite name | Rt(min) | Expreiment Mz | Adduct type | Reference m/z | MS/MS spectrum | PPM |
|---|---|---|---|---|---|---|---|
| POS3344 | orophenyl)prop-2 | 4.161 | 185.04076 | M+CH3OH+H] | 185.0408 | 55.05363:9739 58.94248:9442 101.24716:5838 116.9712:31405 117.97749:26206 139.85522:5406 147.73953:4866 177.13303:5274 | -2.16E-07 |
| POS4257 | Oxododecanoic a | 5.971 | 215.16423 | [M+H-H2O]+ | 215.1642 | 64.30312:6434 69.00585:5486 74.06844:6177 173.3911:9235 | 1.394E-07 |
| POS1486 | CHLOROACETIC A | 9.179 | 128.95036 | [M+H]+ | 128.95039 | 53.03856:10805 54.03373:10138 55.0177:10933 55.05365:150397 55.93345:179525 56.02069:8500 56.04862:16413 57.03286:41085 57.04396:29066 58.06416:18311 59.04858:7804 60.04425:15010 65.03804:9745 67.04092:12917 67.05389:6669 69.03417:9779 69.06868:26954 69.93349:9102 70.0643:10771 71.04816:6777 72.04237:6227 80.04967:8994 82.06502:99688 83.04769:18093 83.0672:10035 84.04315:20834 84.07957:10450 84.58686:6181 86.05938:12259 100.07597:15920 101.0593:6259 101.36545:5561 110.06083:13659 114.97044:45719 128.04031:16387 128.06833:26616 128.10568:17720 | -2.33E-07 |
| POS2454 | S-HNE | 5.797 | 157.12212 | [M+H-H2O]+ | 157.12219 | 59.829:6147 67.64503:6272 70.0643:11551 74.93713:42569 92.94704:7407 115.9617:16302 122.9072:5297 | -4.46E-07 |
| POS763 | -2-Methylthiazoli | 1.565 | 104.05288 | [M+NH4]+ | 104.0528 | 56.04863:29277 57.03287:24803 58.06512:336618 60.08025:66032 61.01003:36530 61.02846:30831 62.05909:8009 69.03294:6299 86.05939:11487 104.10697:38356 | 7.688E-07 |
| POS17 | Nitroethane | 4.635 | 76.03915 | [M+H]+ | 76.0392 | 51.24459:4285 55.05716:4903 55.30983:7160 57.15702:5153 57.30393:4660 57.6643:4205 57.95031:3573 58.80668:4248 60.95991:4197 61.88621:4240 65.07974:4101 66.60102:5484 69.31342:3955 69.56071:5039 70.01898:4557 71.18597:5216 72.46844:4568 74.39948:4665 | -6.58E-07 |
| POS7696 | Erythrophloin D | 5.256 | 355.26282 | [M+H-H2O]+ | 355.26279 | 55.05365:12225 59.04858:19960 67.05389:9657 70.21569:5375 81.06833:32453 83.4408:5835 85.06506:7493 89.05896:19468 93.06841:22560 95.08477:27711 95.58656:5191 99.04339:10223 101.0593:7161 105.06945:25400 107.08486:16717 109.10006:22608 117.06913:6411 119.08333:19304 121.1013:21053 123.08004:16834 131.08699:13095 133.10193:25328 135.11646:18656 145.09904:14060 147.11681:21592 149.13393:7716 159.11723:32874 161.13197:16357 173.13152:12958 185.13043:8133 256.09998:6970 | 8.444E-08 |
| POS3713 | L-Arginine | 0.948 | 197.10004 | [M+H]+ | 197.10001 | 95.17444:5217 173.43034:11655 | 1.522E-07 |
| POS3956 | Eriosematin F | 2.594 | 205.08528 | M+CH3OH+H] | 205.0854 | 55.93435:11112 57.06989:7322 60.04327:6456 109.32312:5950 149.02449:11007 | -5.85E-07 |
| POS1896 | Phosphonoacetate | 8.79 | 140.99532 | [M+NH4]+ | 140.99519 | 50.61313:7477 51.93935:6905 53.03856:8842 54.03373:7841 55.02909:45250 55.05365:9078 55.94513:44629 56.9414:14445 56.96447:9520 57.9342:627768 58.06511:187980 58.9425:28599 59.92951:71728 67.05389:9024 67.93391:26829 69.06991:20850 70.0643:38305 71.05973:16846 72.08047:9118 75.94611:12554 83.05907:36608 84.94554:12106 85.07516:57226 94.06358:6991 97.07692:10413 98.07049:20568 98.96088:40620 98.98203:8721 102.95542:6715 105.72243:7483 112.08707:64567 112.96638:9198 113.96311:9495 116.97124:453924 117.97478:22373 118.96619:46724 126.96738:10885 139.9868:49409 141.11403:45662 | 9.22E-07 |
| POS2457 | S-HNE | 5.411 | 157.12216 | [2M+H]+ | 157.12219 | 55.0177:8019 55.05365:8181 67.05389:7626 67.55908:5260 69.06992:7577 70.06556:10614 74.93713:27741 142.21487:5011 | -1.91E-07 |
| POS2456 | S-HNE | 6.185 | 157.12212 | [M+H]+ | 157.12219 | 53.70765:6653 55.73544:6473 55.87062:7001 55.8778:7466 70.06431:7513 84.07958:5323 | -4.46E-07 |
| POS3306 | Phosphorylcholine | 5.717 | 184.07246 | [M+H-H2O]+ | 184.07249 | 53.48685:5860 57.93419:6489 58.94249:11718 79.25425:5942 86.09538:11694 99.9063:7164 116.97122:31489 117.9775:41158 | -1.63E-07 |
| POS5956 | Prolyl-Tyrosine | 4.473 | 279.13336 | [M+H-H2O]+ | 279.13339 | 55.76759:6338 56.0486:70977 59.86378:5781 61.01:21312 67.05386:6587 68.04947:12414 70.06427:7602 70.77242:5663 81.06986:10469 84.04312:63320 86.05935:62849 88.02827:5406 95.08472:5874 102.05494:7477 103.05193:7343 104.05219:77849 120.08025:216265 130.04878:6549 132.06256:107539 133.02934:31081 150.0573:14199 | -1.07E-07 |

| Differences in metabolites between the Model group and the Control group | | | | | | | |
|---|---|---|---|---|---|---|---|
| Alignment ID | Metabolite name | Rt(min) | Expreiment Mz | Adduct type | Reference m/z | MS/MS spectrum | PPM |
| POS3810 | cgonine methylest | 4.994 | 200.12769 | [M+H]+ | 200.12759 | 55.0177:20821 55.05365:509425 55.93435:26179 56.46365:6412 60.69907:6153 63.21632:5904 67.05389:54277 69.03294:20509 69.06991:45077 76.03859:206104 79.05312:29338 81.06989:55805 83.08509:62109 85.06336:6453 95.08476:21825 97.09953:121566 97.96837:6897 107.08485:17424 113.96311:40467 114.97045:23147 125.09565:43305 131.97139:7671 141.95662:13195 159.96982:8239 | 4.997E-07 |
| POS194 | lethyl-3-buten-2- | 4.818 | 85.06468 | [M+Na]+ | 85.0647 | 56.0486:32294 84.04313:16884 84.07954:6052 | -2.35E-07 |
| POS6430 | trahydrobungean | 6.202 | 296.25693 | [M+H]+ | 296.25681 | 50.40575:6657 55.05365:9155 57.03286:6456 57.06895:157124 59.75949:6013 67.05389:34264 69.06867:16648 71.08546:12163 81.06832:25536 89.05894:5649 93.0684:9678 95.08475:21343 107.08485:6420 109.10004:19765 112.94058:5981 127.10883:20812 135.11642:11814 169.12219:8839 | 4.051E-07 |
| POS6447 | Nimbidiol | 5.318 | 297.14624 | [M+2H]2+ | 297.14612 | 55.05366:18863 57.06896:140606 67.05389:27585 69.06869:15980 71.08548:78878 81.06834:33064 83.0851:25721 85.10045:44797 86.09541:36444 89.05896:6026 95.08477:48806 97.09953:11422 102.09042:6365 104.10697:25479 109.10007:18973 123.11524:6592 124.99956:5615 184.07466:6961 | 4.038E-07 |
| POS2871 | Azelaic acid | 5.671 | 171.10085 | [M+H]+ | 171.101 | 55.05363:20737 57.06986:32199 67.05386:6138 72.04366:30578 83.01192:20364 85.06502:6532 89.06976:12284 117.02013:5325 173.42537:8162 | -8.77E-07 |
| POS3082 | 9E)-Trideca-3,5,7,9 | 6.409 | 177.16322 | [M+H-H2O]+ | 177.1633 | 55.05366:7912 81.0699:6023 83.68195:5881 88.02122:6673 92.61692:5662 93.06841:9043 102.63982:5740 107.08486:6140 121.1013:12055 | -4.52E-07 |
| POS1077 | HLOROMETHYL) E | 0.146 | 114.97119 | [M+H]+ | 114.9711 | 55.0177:41439 55.05365:39687 55.93435:11379 59.04858:44006 60.04425:8325 61.01002:8971 65.69519:5897 68.04829:22913 69.03294:18476 69.06991:48364 70.02779:10448 70.0643:21124 72.04369:14221 72.08047:17478 72.93706:245053 73.04688:21064 73.93718:9329 76.96111:5797 79.05312:20383 86.06966:7235 87.04333:17434 90.94762:49751 91.05576:36055 96.08018:8058 97.06255:5899 97.50974:5642 113.9631:11952 114.06507:26415 114.09124:80031 115.09232:6779 | 7.828E-07 |
| POS6235 | Sempervilam | 6.28 | 289.13318 | [M+H]+ | 289.1333 | 67.05389:10254 81.06989:6707 86.0954:23349 104.10696:26327 117.12083:5852 124.99955:8917 173.42543:11102 184.06929:12494 | -4.15E-07 |
| POS1575 | 3-Oxobutyl acetate | 4.882 | 131.0701 | [M+H]+ | 131.0701 | 56.04953:8861 59.04859:7283 67.05272:7041 69.06992:8089 73.02812:7622 73.06432:17796 84.04317:12866 84.07958:32323 86.09541:17493 87.00325:10920 88.00349:25491 91.0539:27177 97.00919:20774 103.05197:10683 115.96439:9271 130.06477:10960 130.08708:8023 131.08377:10556 131.97467:6433 | 0 |
| POS1564 | lichloro-1,1-ethan | 0.114 | 130.96603 | [M+H]+ | 130.966 | 55.05365:16079 55.93435:8576 56.04862:30593 57.03286:12117 57.06895:13709 58.05276:5832 60.04425:7659 67.05389:27151 69.03294:8992 70.0643:6230 70.76221:6356 71.04816:7946 71.92825:120858 72.93706:51136 74.02328:6586 75.02496:6958 77.06126:6536 83.04769:7578 84.04315:29053 84.07957:78908 85.06505:10994 85.08358:5625 86.05938:9231 86.0954:8645 89.93904:39558 90.94762:14004 93.03563:6575 107.94919:30535 111.93941:6010 112.07433:11074 125.95948:15501 129.99471:6324 130.08707:7610 130.96458:5561 | 2.291E-07 |
| POS4083 | Heteromine D | 4.832 | 209.12712 | [M+H]+ | 209.127 | 51.89836:5653 60.04323:6426 62.4356:5416 70.06427:22419 81.06829:5421 148.11047:10450 188.13643:5890 | 5.738E-07 |
| NEG1489 | L-LYSINE | 6.807 | 145.09785 | [M-H]- | 145.09801 | 52.09783:7013 84.031:9349 100.04588:44311 101.05439:1161267 102.05608:101844 116.05926:45095 117.06681:15819 118.18125:15751 | -1.1E-06 |
| POS1833 | Suberic acid | 6.058 | 139.07501 | [M+H]+ | 139.075 | 50.5135:6387 55.01768:12965 55.05363:10633 57.06894:14914 58.06509:12052 73.30093:5932 83.01192:63402 93.0703:5907 95.04691:8212 | 7.19E-08 |
| POS1148 | Ethyl pyruvate | 5.392 | 117.0544 | [M+H]+ | 117.0545 | 55.05366:16095 59.04859:15239 64.36852:5225 70.06431:169751 71.04945:5714 71.06875:8396 72.04501:6564 72.08048:16839 101.25597:5752 | -8.54E-07 |
| POS3068 | Allantoic acid | 1.006 | 177.06184 | [M+H]+ | 177.06183 | 60.05523:12147 70.06428:25050 71.06744:23278 82.464:6268 146.761:5011 146.91003:5418 | 5.648E-08 |
| NEG5828 | hinidin 3-sambubi | 5.923 | 297.56561 | [M-H]- | 297.56546 | 92.23355:6520 94.94423:6029 101.05437:6192 173.62036:10440 182.25806:6476 292.43253:5709 | 5.041E-07 |
| POS665 | hyltetrahydrofuran | 5.894 | 101.05945 | [M+H]+ | 101.0596 | 55.05364:12225 60.04425:40639 61.03868:12871 98.69289:5492 100.0738:7191 | -1.48E-06 |
| POS4133 | leodomycin B | 5.797 | 211.13225 | [M+H]+ | 211.1324 | 57.03287:11300 69.06868:6229 70.42085:6023 71.04816:5428 76.21976:5535 81.0699:9033 95.08477:10395 109.06336:13332 119.08333:6266 125.03258:7347 133.10193:10793 151.10901:114635 165.12749:9258 | -7.1E-07 |

**Differences in metabolites between the Model group and the Control group**

| Alignment ID | Metabolite name | Rt(min) | Expreiment Mz | Adduct type | Reference m/z | MS/MS spectrum | PPM |
|---|---|---|---|---|---|---|---|
| POS4451 | lelta-Valerolacton | 0.889 | 223.09393 | M+CH3OH+H] | 223.09399 | 58.27951:5910 62.98166:6700 78.99126:5440 80.94775:8679 96.921:226607 103.89967:5711 104.99084:6436 144.94151:5536 164.92259:37831 202.468:6480 | -2.69E-07 |
| POS149 | al\|Nitrile-(()-3-Hyc | 9.005 | 84.04433 | [M+H-H2O]+ | 84.0443 | 56.0486:120292 56.2347:7436 62.64165:6270 84.04312:82510 | 3.57E-07 |
| POS4675 | Norpterphyllin III | 0.75 | 231.10406 | [M+NH4]+ | 231.104 | 55.93433:12000 57.03284:6762 57.06894:71414 69.06989:8521 79.05309:7886 81.03226:18224 83.04766:10137 85.06333:20585 111.04436:9389 125.05955:49088 133.06889:8965 167.10455:88462 | 2.596E-07 |
| POS3959 | Anofinic acid | 1.176 | 205.0856 | [M+H]+ | 205.0856 | 57.03287:12657 57.06897:11826 60.08025:113265 85.02801:495511 86.03025:37113 149.02058:15997 | 0 |
| POS1258 | Hydroxybenzoic a | 6.414 | 121.02811 | [M+H]+ | 121.028 | 51.98441:6202 52.08194:5974 65.03805:30519 86.61761:5929 120.37191:5659 | 9.089E-07 |
| POS151 | al\|Nitrile-(()-3-Hyc | 0.618 | 84.04436 | [M+H]+ | 84.0443 | 55.05365:16638 56.04861:115979 84.04314:90317 84.07956:9447 | 7.139E-07 |
| POS1778 | 4-Propylphenol | 4.815 | 137.09586 | [M+NH4]+ | 137.0959 | 53.61392:6146 53.8126:5696 67.05386:18617 72.8742:5711 77.83262:6596 79.05309:11525 81.06987:39587 91.05386:9088 95.08473:16437 | -2.92E-07 |
| POS4854 | Palmitoleic acid | 6.324 | 237.22009 | [M+H-H2O]+ | 237.22 | 69.06869:6628 81.0699:9704 107.08487:6414 232.43169:6150 | 3.794E-07 |
| POS672 | hyltetrahydrofuran | 5.335 | 101.05948 | [M+Na]+ | 101.0596 | 55.05366:9573 60.04327:16959 64.96264:6955 70.27892:5149 96.75728:5504 103.46888:5735 | -1.19E-06 |
| POS243 | Butanal, 2-oxo- | 4.902 | 87.04405 | M+CH3OH+H] | 87.04405 | 54.09435:6368 86.05939:24522 87.04335:10728 | 0 |
| POS1740 | ydroxyanthranilic | 3.644 | 136.03891 | [M+H-H2O]+ | 136.039 | 55.93435:66812 71.92825:19297 72.93706:64380 86.21218:5189 89.93904:6074 90.94762:18292 91.05389:29653 108.99001:5988 136.02156:10530 136.05905:19517 | -6.62E-07 |
| POS336 | L-Alanine | 2.49 | 90.05499 | [M+H]+ | 90.05498 | 61.02742:736144 62.03179:24408 72.04368:7129 72.08047:7254 | 1.11E-07 |
| POS7346 | .3Z-Docosenamid | 6.315 | 338.34091 | [M+H]+ | 338.341 | 50.33126:5881 111.81992:5735 173.39104:5693 176.81448:5137 196.18338:6242 | -2.66E-07 |
| POS6847 | MG(16:0/0:0/0:0) | 6.322 | 313.27203 | [M+H]+ | 313.272 | 57.06987:13058 92.41432:6239 110.36397:5778 173.39598:13388 214.98621:5459 260.67572:6780 | 9.576E-08 |
| POS6454 | -6,10,14-trimethyl | 5.097 | 297.2413 | [M+H]+ | 297.2413 | 55.05364:12699 57.06895:78902 67.05388:29424 69.0699:17559 69.81181:6200 71.08546:34550 81.06831:23723 83.08508:9349 84.08121:5730 85.10043:19462 86.09538:15480 89.05894:5805 95.08475:31751 95.21631:6185 104.10694:13999 109.10004:8792 172.32704:5568 | 0 |
| POS330 | Lactamide | 1.48 | 90.05469 | [M+H]+ | 90.05478 | 61.02742:529022 62.03075:13726 72.08047:8393 76.49201:5079 78.29926:5515 | -9.99E-07 |
| POS1868 | BETAINE | 1.318 | 140.0679 | [M+H]+ | 140.06787 | 67.05389:6287 93.9344:5387 94.06358:19246 112.03867:7627 | 2.142E-07 |
| POS4446 | Aminoanthraquinc | 1.523 | 223.0631 | [M+H]+ | 223.063 | 58.06416:18318 59.04858:9237 61.03971:21594 73.02811:13800 73.04688:17204 89.05895:7088 91.05576:21940 93.07033:9530 119.04705:6844 209.00751:10711 225.04042:94361 | 4.483E-07 |
| POS895 | octa-1,3,6-triene | 9.574 | 109.10102 | [M+H]+ | 109.1011 | 67.05386:20167 67.51138:5484 70.40051:5189 72.86618:6126 72.96379:5746 73.08709:7201 74.07662:5161 79.60238:5924 87.0032:8411 109.07554:8961 111.96735:6002 | -7.33E-07 |
| POS593 | lethyl-2(5H)-furan | 9.001 | 99.04386 | [M+H]+ | 99.044 | 52.14494:6029 53.03854:7984 55.93433:11867 57.06894:21089 72.93703:45790 89.961:6771 90.94759:7406 98.9841:14208 | -1.41E-06 |
| POS61 | Benzene | 3.996 | 79.05422 | [M+H]+ | 79.0542 | 51.36534:4811 51.44291:4633 51.60176:4579 55.41333:5178 59.86081:4222 60.2205:3878 61.02845:11184 61.59448:5402 61.78178:4911 64.48734:4628 68.45152:4331 70.77499:5100 71.3941:5594 76.17404:3930 78.70107:4524 | 2.53E-07 |
| POS187 | -butenoic acid ga | 9.595 | 85.02846 | [M+Na]+ | 85.02838 | 53.01286:15435 56.04862:137267 67.05389:6919 84.04315:124379 84.07957:15886 85.02799:6883 85.04652:10727 | 9.409E-07 |
| POS5118 | H-Leu-Asp-OH | 1.668 | 247.12817 | [M+NH4]+ | 247.1282 | 61.03972:6894 72.08047:42287 84.04316:21456 84.07957:9799 85.02799:14644 118.08494:10756 171.03236:5870 187.84866:5711 | -1.21E-07 |
| POS7699 | -Linoleoyl Glycerc | 6.877 | 355.28223 | [M+H]+ | 355.2821 | 101.92889:5706 142.24037:5452 153.16835:5057 173.43524:8239 | 3.659E-07 |
| POS181 | -butenoic acid ga | 9.903 | 85.02835 | [M+H]+ | 85.02838 | 53.77023:6062 55.05363:6463 56.0486:93024 84.04312:65379 | -3.53E-07 |
| POS446 | 2-Methylpyridine | 3.062 | 94.06503 | [M+H]+ | 94.065 | 53.25908:4990 57.03286:6170 95.04893:8674 | 3.189E-07 |
| POS930 | -propan-2-ylpyrrc | 8.752 | 110.09641 | [M+H]+ | 110.0963 | 51.58027:6929 57.9342:33504 71.94005:11487 74.99706:25631 86.50518:14018 87.00323:7748 88.00347:108227 97.00917:47521 99.51075:24002 108.51748:9324 112.96638:11638 | 9.991E-07 |
| POS631 | Pent-4-enamide | 2.585 | 100.07546 | [M+H]+ | 100.0756 | 55.01771:9721 55.05366:19322 58.02808:7424 70.41451:5645 76.19833:5992 98.98415:14159 | -1.4E-06 |
| POS3955 | Eriosematin F | 1.524 | 205.08527 | [M+H]+ | 205.0854 | 57.03287:14839 57.06989:12885 60.08025:91444 85.028:404395 86.03025:32870 121.02692:7917 149.02058:22586 | -6.34E-07 |

| Differences in metabolites between the Model group and the Control group | | | | | | | |
|---|---|---|---|---|---|---|---|
| Alignment ID | Metabolite name | Rt(min) | Expreiment Mz | Adduct type | Reference m/z | MS/MS spectrum | PPM |
| POS8047 | hydroxycrambesc | 5.802 | 376.25919 | [M+NH4]+ | 376.2594 | 57.06896:980735 58.07272:16000 80.38296:5747 83.32634:5984 125.07162:16895 126.05367:46383 209.12439:64788 210.11511:10225 218.12764:23013 219.11343:8727 275.17361:7848 292.19846:9008 293.18784:16395 297.95679:5695 302.18762:21385 343.06805:6336 376.26254:23085 | -5.58E-07 |
| POS8735 | Nafoxidine | 4.614 | 426.24298 | [M+2H]2+ | 426.24277 | 55.05365:50348 59.04858:49053 61.02845:31074 67.05389:20798 69.03294:835472 70.0366:21185 71.04945:25943 73.02811:207204 73.06432:84852 78.03804:39467 80.05429:60806 81.03229:27254 81.0699:14234 83.04932:143834 85.02799:23662 87.04334:669015 88.04782:18084 89.05896:2239860 90.0619:79698 91.04643:17202 95.04893:43332 99.04339:386962 100.05018:102858 102.06606:49288 103.03849:20744 107.06821:25936 109.06336:23486 111.04441:191889 113.05927:53207 117.09089:26953 122.06293:147835 122.56553:16009 125.0596:38296 129.05446:69540 131.07088:66151 133.08543:619741 134.09018:31549 137.0598:20853 144.07597:61997 155.07083:74452 166.08865:23580 173.07771:22267 177.11285:50953 | 4.927E-07 |
| POS9607 | Netilmicin | 4.497 | 476.30658 | [M+H]+ | 476.30679 | 73.06431:16723 82.98758:9562 87.04333:88920 89.05895:1344325 90.0619:26633 107.52888:8753 117.09088:10341 130.08388:8904 131.06764:18442 133.08543:536390 134.09016:13500 175.09525:9280 177.11284:78839 177.44234:9902 | -4.41E-07 |
| POS6878 | 2-methylphenyl)ch | 1.033 | 315.0018 | M+CH3OH+H] | 315.00201 | 62.98163:10330 80.94772:43991 82.94373:11288 133.82373:5799 135.00177:798502 136.00449:11211 | -6.67E-07 |
| POS1643 | THTC | 1.565 | 133.03145 | [M+H]+ | 133.03169 | 55.0177:11503 55.05365:19549 56.04863:22394 57.05693:74716 58.06512:49342 67.05389:8925 69.03294:13369 69.06868:753039 70.07312:57048 86.09541:3624676 87.09918:275236 132.10175:9428 | -1.8E-06 |
| POS3418 | hiophen\|2-<Octat | 1.022 | 187.05727 | M+CH3OH+H] | 187.0575 | 62.55975:6475 67.44471:5435 70.06427:6321 81.06986:8247 101.83167:6262 | -1.23E-06 |
| POS10416 | from Mulder Bioc | 5.345 | 513.34473 | [M+2H]2+ | 513.34497 | 57.03284:27899 59.04856:448896 60.05223:12018 69.06988:6563 73.02808:29925 73.06429:11442 85.06501:14269 87.0433:62729 87.07994:11068 89.05891:367724 90.06187:9711 94.06942:10331 101.05926:60205 103.07439:106553 104.10692:6321 115.07638:11671 117.09084:7292 129.08905:9081 133.08537:99502 147.10141:45184 191.1255:7629 333.50156:7036 379.29004:6238 | -4.68E-07 |
| POS6474 | Stepharine | 8.809 | 298.14288 | [2M+H]2+ | 298.14447 | 55.05366:34647 57.06896:290541 58.07272:17207 60.08025:16111 67.05389:60108 69.06869:44183 71.08419:158150 72.08836:10216 81.06834:70186 83.0851:45791 85.10046:70404 86.09541:80791 95.08478:106599 96.08828:8833 97.09953:26441 99.07939:7069 102.09042:16028 103.09242:6131 104.10697:65978 109.10007:47414 110.10549:5637 123.11524:17088 124.99956:19890 152.28874:5179 173.39111:11392 181.02371:7528 184.07466:13722 189.81023:5380 240.09721:7437 | -5.33E-06 |
| POS10095 | alpha,6alpha,7bet | 5.146 | 499.3259 | [M+2H]2+ | 499.32571 | 57.03287:76137 59.04859:1558574 73.02812:180120 73.06432:77930 80.0543:78842 85.06506:35346 87.04335:275850 87.06079:126498 89.05896:1979272 90.06191:77094 101.05931:196932 102.06606:46237 103.07444:392972 104.10697:220163 105.10879:93223 109.0756:62332 129.08911:44561 131.07088:47159 133.08545:758990 134.09018:37677 138.09604:31302 147.10149:220400 177.11285:76722 184.06931:79134 191.1256:37782 344.14871:30997 | 3.805E-07 |
| POS11993 | hodeoxycholylargi | 5.564 | 571.38263 | [M+2H]2+ | 571.38293 | 57.03287:54088 59.04859:910919 60.05226:26733 69.06992:16959 73.02812:81292 73.06432:36065 74.06708:9281 85.06337:30932 87.04335:141471 87.06254:28789 87.07999:32295 89.05896:563763 90.06191:22655 94.06947:26926 99.07939:13228 101.05931:142150 103.07444:250230 104.10469:18320 107.06821:11190 115.07378:18379 116.08248:9978 117.0909:23833 129.08911:15358 131.06766:15681 131.10634:9048 133.08545:194027 134.08684:17871 145.08403:13019 147.10149:105449 161.11441:12545 171.97781:9731 177.1078:10406 191.1256:13171 205.14238:9301 | -5.25E-07 |
| POS8488 | Val Leu Tyr | 4.962 | 408.25195 | M+CH3OH+H]2 | 408.24921 | 59.04858:44387 87.04334:13409 89.05896:68683 95.42019:6092 101.0593:12186 103.07669:10578 133.08545:26958 | 6.712E-06 |

| Differences in metabolites between the Model group and the Control group | | | | | | | |
|---|---|---|---|---|---|---|---|
| Alignment ID | Metabolite name | Rt(min) | Expreiment Mz | Adduct type | Reference m/z | MS/MS spectrum | PPM |
| POS10966 | Roehybridine | 8.789 | 534.29517 | [M+Na]+ | 534.29541 | 60.08025:12284 65.02566:7665 86.09541:75514 104.10696:321832 105.66643:9116 112.49341:8183 | -4.49E-07 |
| POS4666 | Norpterphyllin III | 5.591 | 231.10378 | [M+Na]+ | 231.104 | 53.03854:9765 55.01768:12449 55.05363:23575 57.03284:29823 57.06894:299791 61.01:36048 67.05386:14375 69.06865:26022 79.05309:12429 81.03226:83789 81.06987:16441 83.04766:36642 85.02796:14007 85.06333:103688 93.06837:6266 95.08473:8876 97.02761:13545 97.06457:11658 107.04913:7903 111.04436:33797 123.0448:6083 125.05955:244129 126.06273:6287 133.06891:40815 167.10457:465402 168.11116:22316 173.39104:7654 | -9.52E-07 |
| POS15389 | PC(18:2(2E,4E)/0:0) | 6.255 | 1039.67102 | [2M+H]+ | 1039.67053 | 56.0486:8800 58.06509:8734 60.08023:132038 63.92054:5359 67.05387:6755 71.07258:26955 86.09538:200052 95.08274:5910 98.98199:7908 104.10693:977993 105.11106:7441 116.77584:6002 121.29324:7112 121.79109:6253 124.9995:39813 173.38614:14816 184.07458:276262 185.0817:7575 914.8385:6452 | 4.713E-07 |
| NEG5130 | Terezine A | 0.991 | 273.12494 | [M-H2O-H]- | 273.12469 | 87.11253:30944 89.13125:90150 93.03925:253037 95.03763:525814 97.03745:222648 145.23549:11469 147.15915:29333 153.06755:11910 155.06651:16051 | 9.153E-07 |
| POS14049 | ethylbutyroyl),22-( | 5.172 | 689.46246 | [M+H]+ | 689.46222 | 57.03287:127973 59.04859:2489306 73.02812:97416 73.06432:81669 85.06337:72944 87.04335:451024 87.07999:84081 89.05896:2099070 99.07939:51889 101.05931:431129 103.07444:982473 115.07378:50248 117.05281:98589 117.0909:64985 131.06766:89739 133.08545:674337 145.08403:49569 147.10149:648273 161.1188:132124 173.39603:93594 177.11285:44225 191.1256:86610 205.14238:50020 355.71689:43311 | 3.481E-07 |
| POS8879 | Suvanine | 4.698 | 435.2565 | [M+H]+ | 435.25629 | 51.50556:7580 67.73112:5330 89.05894:12640 123.58301:5278 183.53943:6587 | 4.825E-07 |
| POS11896 | Cinnamoyl-vulgaro | 4.745 | 567.33191 | [M+Na]+ | 567.3316 | 58.32636:6288 87.04335:6304 89.05896:14008 101.41589:5569 147.08617:5377 148.92294:5751 173.39111:10389 192.58664:6191 392.82523:6321 503.2316:5948 | 5.464E-07 |
| POS11844 | manzamine M | 5.053 | 565.35388 | [2M+H]+ | 565.35358 | 59.04858:259007 73.02811:50874 73.06431:30974 78.36626:16107 80.05274:24239 87.04333:86028 89.05894:518801 101.05929:33520 103.07442:72740 123.34731:18975 133.08542:191946 147.10147:40567 173.43033:40313 177.11282:31289 194.32018:20204 446.87405:18802 | 5.306E-07 |
| POS8786 | Mifepristone | 4.548 | 429.267 | [M+H]+ | 429.26682 | 59.04859:101581 73.02812:9553 73.06432:14635 85.06506:7925 87.04335:104053 89.05896:1139310 90.06191:21204 101.05931:5715 103.07444:21527 107.06821:7909 129.56607:6863 131.06766:16384 133.08545:384629 134.08684:9803 147.10149:14268 154.65695:5826 177.11285:33393 287.56656:5856 | 4.193E-07 |
| POS5025 | Agomelatine | 5.56 | 244.13278 | [M+NH4]+ | 244.133 | 67.05389:23319 69.06868:1558184 70.07312:40942 115.05522:13405 130.06477:303271 131.06766:12601 132.08218:16202 142.06566:259660 143.06737:13584 144.07968:12297 160.07417:332565 161.07927:17557 176.06961:21747 188.07004:192286 189.07663:13067 198.12756:12154 | -9.01E-07 |
| POS11867 | ntibiotic FR 90084 | 5.929 | 566.32208 | [M+H]+ | 566.32239 | 59.04856:25629 60.08023:20202 71.07258:6320 73.02809:5629 86.09538:58974 87.04331:8500 89.05893:18621 104.10693:233342 124.9995:16304 184.07458:77241 355.55829:5667 | -5.47E-07 |
| POS3675 | Hexylresorcinol | 4.373 | 195.12135 | [M+H]+ | 195.1216 | 58.06417:84777 89.05896:127199 95.26823:5724 107.04918:40419 133.08545:6906 135.04228:115350 136.04544:21897 194.11676:14281 | -1.28E-06 |
| POS10211 | PE(0:0/20:3(5Z,8Z, | 6.328 | 504.30774 | [M+H]+ | 504.3075 | 55.84372:5639 55.87241:6045 57.03379:6226 60.15533:6699 62.05909:35627 67.05389:9008 81.0699:10454 89.05896:8405 95.08477:9910 107.08487:6805 115.67261:5821 | 4.759E-07 |
| POS12104 | a-Tocopherol succ | 5.451 | 575.36816 | [M+2H]2+ | 575.36798 | 59.04859:160598 67.015:20277 73.02812:13311 79.78577:10853 87.04335:32101 89.05896:181868 101.05931:14248 103.07444:47691 133.08545:56907 147.10149:15684 | 3.128E-07 |
| POS3346 | htho[2,1-b]thioph | 0.892 | 185.04167 | [M+NH4]+ | 185.0419 | 125.02051:21327 | -1.24E-06 |
| POS10477 | Ile Glu Leu Lys | 5.62 | 516.33887 | [M+2H]2+ | 516.33911 | 57.03287:26206 57.06896:9009 59.04859:219972 67.015:15101 73.02812:19965 73.06432:11710 81.03072:13328 85.06506:7129 87.04335:26028 87.07999:7319 89.05896:145585 90.06191:7778 101.05931:22095 103.07444:54508 117.05281:9146 126.02024:8756 129.08911:7557 133.08545:40360 147.10149:20887 149.97055:7802 161.11441:6022 165.10016:7441 194.85654:5915 340.77332:6237 | -4.65E-07 |

| Differences in metabolites between the Model group and the Control group | | | | | | | |
|---|---|---|---|---|---|---|---|
| Alignment ID | Metabolite name | Rt(min) | Expreiment Mz | Adduct type | Reference m/z | MS/MS spectrum | PPM |
| POS11755 | onjugated chenod | 5.607 | 562.34882 | [M+H]+ | 562.349 | 57.97966:12413 59.04856:44377 60.08022:29247 67.61633:14164 86.09536:69963 89.05891:29635 91.32316:10827 104.10692:295921 105.11105:15458 184.07457:89546 | -3.2E-07 |
| POS12674 | Gly Asn Leu Arg Ly | 5.094 | 601.37762 | M+CH3OH+H] | 601.37793 | 57.03287:14339 59.04859:165112 73.02812:14904 73.06432:10785 87.04335:701428 89.05896:178052 101.05931:156728 103.03849:51479 103.07444:41422 115.07378:12363 117.05281:35948 131.06766:17236 133.08545:55064 147.10149:20260 217.73381:9698 | -5.15E-07 |
| POS12835 | 9-Oxide ; Paecilot | 5.672 | 608.91724 | [M+2H]2+ | 608.91699 | 59.04859:265532 73.02812:20811 85.06337:17145 87.04335:54346 87.06079:14047 89.05896:228246 101.05931:63835 103.07444:93415 133.08545:72130 147.10149:36558 164.48682:14678 | 4.106E-07 |
| POS10700 | Calcimycin | 6.108 | 524.27417 | [M+Na]+ | 524.27399 | 57.03286:1100542 58.03661:42022 59.04858:301784 60.08024:29131 73.58075:13876 81.06989:33230 86.0954:87303 99.07938:87036 101.09422:19774 104.10696:303568 115.07377:73317 117.09088:21560 157.11888:72431 173.43033:28487 184.06927:115013 462.50906:13869 522.58624:12674 | 3.433E-07 |
| POS12148 | PA(15:1(9Z)/12:0) | 5.744 | 577.38403 | [M+2H]2+ | 577.38422 | 55.29747:20168 57.03287:71034 59.04859:217476 67.07867:19889 73.02812:56650 85.06337:37893 87.04335:72367 87.06079:26980 89.05896:315632 101.05931:70320 103.07444:46810 129.08911:41048 133.08545:103642 147.10149:30193 173.38622:22558 | -3.29E-07 |
| POS8252 | Metazin | 4.667 | 391.22971 | [M+Na]+ | 391.22989 | 73.02812:6638 89.05896:17041 133.08545:9736 | -4.6E-07 |
| POS11572 | Muscanone | 5.775 | 555.36932 | [M+2H]2+ | 555.36951 | 55.05366:11162 57.03287:75288 59.04859:163324 65.04931:8099 73.02812:47099 73.06432:9453 85.06337:39725 87.04335:75640 87.06254:15720 87.07999:16998 89.05896:198155 90.06191:8464 101.05931:57031 103.07444:31080 109.0756:6983 115.07378:7632 129.09227:12419 133.08545:57503 143.10413:8150 147.10149:12617 552.71912:6996 | -3.42E-07 |
| POS15383 | ,13-dimethylhexa | 7.329 | 1009.69946 | [M+H-H2O]+ | 1009.70001 | 50.41728:4625 51.16747:4355 67.85581:5258 68.19077:5133 69.09335:4538 69.98:5230 73.86624:4573 87.78227:5144 87.80347:4915 89.77258:4853 102.61972:5708 109.25687:4829 109.90973:5719 111.15759:5039 112.63448:5961 115.85182:6537 175.85406:6312 252.27922:8560 | -5.45E-07 |
| POS2821 | -ureidobutyric aci | 1.049 | 169.05814 | [M+H]+ | 169.05832 | 52.11343:6093 55.02822:24863 67.05389:8183 68.7358:5188 69.00831:10412 70.03912:49662 79.05312:7897 81.06989:12314 96.0175:22582 98.03503:31566 114.06246:6020 116.72981:5296 124.01196:8099 126.02934:37045 141.03847:76945 151.04518:12385 152.00658:21390 169.0325:59061 | -1.06E-06 |
| POS13394 | cynthiaxanthin ac | 6.478 | 640.41254 | [M+H]+ | 640.41278 | 57.03287:116401 59.04859:51566 72.64896:22764 73.15427:20225 86.09541:26475 95.44221:21301 165.65282:20413 173.43034:39705 575.79932:22827 | -3.75E-07 |
| NEG6716 | Thr Pro Asn | 1.086 | 329.14691 | [M-H]- | 329.1467 | 87.11256:12794 89.13128:25800 93.03928:344289 95.03767:312418 97.03749:31470 103.08175:9911 130.59087:5960 145.14165:6423 145.23553:7752 151.06717:7590 153.0676:22636 182.01541:5827 | 6.38E-07 |
| POS10245 | Yardenone A | 7.332 | 505.35059 | [M+2H]2+ | 505.35031 | 173.3911:17792 | 5.541E-07 |
| POS7358 | norpregna-1,3,5,7 | 4.714 | 339.19522 | [M+H]+ | 339.1954 | 57.03287:11041 59.04859:260958 72.71816:7359 73.02812:6847 87.04335:270072 88.04605:5926 89.05896:78140 101.05931:32140 103.03849:103628 103.07444:35871 117.05281:30389 131.06766:6514 133.08545:6862 147.10149:6776 290.02237:5411 | -5.31E-07 |
| POS3669 | 01!(E)-4-hydroxyr | 0.887 | 195.09877 | [M+NH4]+ | 195.099 | 82.34032:6379 82.37885:6615 88.44839:5652 162.27571:6158 173.43031:6117 | -1.18E-06 |
| POS5232 | Paucine | 4.416 | 251.13805 | [M+NH4]+ | 251.13831 | 69.03294:10049 72.77946:5754 73.02945:6132 86.05938:17064 88.03894:18168 101.0593:5852 103.05421:11782 120.04356:14019 120.08029:37074 123.67455:5556 131.04831:6292 147.0555:16864 171.53304:5898 188.10326:14079 | -1.04E-06 |
| POS11802 | Rhodoxanthin | 7.672 | 563.39111 | [M+2H]2+ | 563.39081 | 82.57995:5554 136.10338:5762 136.28088:7128 345.74472:6147 | 5.325E-07 |
| POS680 | Homopiperazine | 6.518 | 101.10713 | [M+H]+ | 101.10732 | 51.99327:6998 70.19422:6915 101.10733:5472 | -1.88E-06 |
| POS3872 | metric dimethylarg | 5.069 | 202.14316 | [M+H-H2O]+ | 202.14296 | 54.00388:6561 55.05365:108846 57.06895:16962 69.06867:15266 76.7874:5737 97.09953:36648 97.24764:5777 101.0593:6591 114.12527:932384 115.12946:22465 130.06476:5500 156.13799:21795 160.13072:36610 | 9.894E-07 |
| NEG4747 | ecyloxypropane-1 | 4.153 | 259.22815 | [M-H]- | 259.22791 | 53.9787:5890 59.0295:9226 60.40223:5661 64.27615:5905 70.36988:6031 92.55002:6256 103.07276:30071 | 9.258E-07 |

| Differences in metabolites between the Model group and the Control group | | | | | | | |
|---|---|---|---|---|---|---|---|
| Alignment ID | Metabolite name | Rt(min) | Expreiment Mz | Adduct type | Reference m/z | MS/MS spectrum | PPM |
| NEG2216 | Glycylproline | 8.782 | 171.07729 | [2M-H]- | 171.07751 | 56.02806:6761 56.26738:6083 61.21238:5515 84.03104:7884 109.19517:7395 136.52611:6194 | -1.29E-06 |
| POS4668 | Norpterphyllin III | 8.925 | 231.10378 | M+CH3OH+H] | 231.104 | 55.01768:8285 55.05363:16588 55.93343:27143 57.03284:14107 57.06894:178894 61.01:23672 61.75778:5917 67.05386:7067 69.06865:17043 73.63633:5686 79.05309:10705 81.03226:47980 81.06987:12417 83.04766:18076 85.02796:14311 85.06333:50272 97.02761:8872 97.06457:8835 111.04436:24819 114.97041:24398 123.0448:6417 125.05955:131348 133.0656:20110 167.10457:251841 168.11116:10679 | -9.52E-07 |
| POS2428 | hosphoglycolic ac | 8.819 | 156.98994 | M+CH3OH+H] | 156.98959 | 52.57622:5798 53.93831:20328 55.0177:6849 55.05365:18289 56.04952:11259 57.9342:16022 70.0643:8385 70.94153:8906 72.93706:21304 73.93719:8762 74.93712:198919 84.07957:7913 86.82748:5832 90.94576:6888 92.94704:32875 97.0646:12623 110.05835:7187 112.97669:6802 113.96311:7086 115.95633:28188 115.96169:84824 116.97124:8238 128.06833:6180 133.97353:17542 | 2.229E-06 |
| POS506 | 2-Dichloroethyler | 0.128 | 96.96085 | [M+H-H2O]+ | 96.9606 | 50.63479:6013 55.0177:9152 55.05365:24431 55.93344:20500 65.42387:6768 68.04949:8679 69.04403:10401 96.04378:19276 97.03996:9028 97.0646:9691 | 2.578E-06 |
| POS502 | 2-Dichloroethyler | 9.291 | 96.96079 | [M+H]+ | 96.9606 | 53.92981:6138 55.05365:20051 55.93435:14013 68.0495:6645 69.04403:7150 84.06301:5123 95.95288:6106 96.04378:13152 97.0379:12825 97.0646:7771 | 1.96E-06 |
| POS1160 | -Ethylbutanoic aci | 4.938 | 117.09067 | M+CH3OH+H] | 117.0909 | 55.05365:34633 70.0643:105869 71.61486:6049 72.08047:36936 98.5709:5404 103.54803:5433 | -1.96E-06 |
| POS3201 | 80638-48-8 | 5.379 | 181.08533 | [M+Na]+ | 181.08549 | 135.07935:33604 | -8.84E-07 |
| POS302 | Acetoin | 5.447 | 89.05939 | [M+H]+ | 89.0596 | 55.93345:7048 61.02845:15928 72.93707:41035 85.18988:5897 90.94763:7938 | -2.36E-06 |
| POS1482 | Galegine | 6.532 | 128.11792 | [M+Na]+ | 128.1181 | 58.06512:7710 62.30235:5532 71.10865:5107 88.24155:5415 128.11816:6668 | -1.4E-06 |
| POS157 | al\|Nitrile-(()-3-Hyc | 1.136 | 84.0445 | [M+NH4]+ | 84.0443 | 55.05365:24761 56.04862:287729 65.03804:6148 66.17352:6014 67.05389:15938 69.05635:7034 80.31335:6038 82.06502:31476 82.94376:8824 83.06069:10527 84.04315:222538 84.07957:435292 | 2.38E-06 |
| POS929 | Brunfelsamidine | 0.855 | 110.07095 | [M+H]+ | 110.0712 | 55.55091:5496 56.04953:7725 62.15791:5484 68.98124:8315 81.96613:5329 86.99279:13087 88.0851:5737 100.02225:7363 105.0024:22770 | -2.27E-06 |
| NEG1147 | _LEUCINE-5,5,5-D | 1.191 | 133.10646 | [M-H]- | 133.10622 | 59.08403:32480 71.09898:515055 73.07996:114427 75.09879:9345 79.05367:981327 79.06424:33752 87.11253:6641 88.14607:14457 89.13124:43366 97.08466:1245166 115.14149:329179 133.05977:14749 133.17186:63786 133.20816:7937 | 1.803E-06 |
| NEG932 | imethylthiazol-2-a | 8.995 | 127.03379 | [M-H]- | 127.0336 | 53.92936:5989 65.28221:6656 | 1.496E-06 |
| POS1854 | yl-2,5-dimethylpy | 6.462 | 139.12263 | [2M+H]+ | 139.12289 | 55.0291:14994 57.9342:17711 58.06417:68347 69.06869:10360 70.06431:14638 83.05908:18365 96.08018:7258 116.97126:9458 122.0948:18950 139.12291:11213 | -1.87E-06 |
| POS3554 | bicyclo[4.3.0]nona | 7.201 | 191.14226 | [M+H]+ | 191.1425 | 55.68453:6124 140.74756:6479 | -1.26E-06 |
| POS1025 | l-imidazol-5-yl)et | 6.475 | 113.07066 | [M+H]+ | 113.0709 | 54.03372:77373 59.92951:49646 61.927:7501 67.05389:20078 69.04403:443480 70.02779:36752 70.0643:9931 71.05973:242969 72.06339:12234 73.56719:6193 86.94053:6018 89.00122:68090 89.50283:10153 89.99953:24537 94.06554:8025 96.0074:17692 98.00586:131554 98.50787:22995 99.00529:32533 100.50954:32851 101.50806:6877 107.01112:12740 109.51484:20907 110.51353:8603 113.03345:19640 113.06959:128467 | -2.12E-06 |
| POS735 | yne\|Ethynylbenzer | 3.787 | 103.05397 | [M+H]+ | 103.0542 | 53.01283:7468 56.0486:15841 57.03284:73042 60.04423:58404 61.0274:97448 85.02795:45406 95.04889:10935 102.05493:8132 103.03844:9101 | -2.23E-06 |
| POS1481 | Galegine | 8.094 | 128.11789 | [M+H]+ | 128.1181 | 51.12348:4462 54.13879:4788 54.93365:4623 56.42904:4269 56.67014:5177 58.83768:4200 61.44732:4207 63.79562:5265 70.97746:5200 73.28477:4825 78.06468:6157 87.17079:5211 102.15912:5638 103.44626:4638 | -1.64E-06 |
| POS3553 | id, 3a,4,5,6,7,7a-h | 7.296 | 191.14224 | [M+H]+ | 191.142 | 55.69256:6028 60.02228:5774 67.29036:4872 70.4183:5808 | 1.256E-06 |
| POS2 | 1-Propene-1-thio | 0.711 | 75.02599 | [M+H]+ | 75.0262 | 50.26234:6873 50.31902:6093 50.34586:4342 52.05128:4041 52.05774:5519 53.90685:4830 53.93237:6118 53.96725:5347 55.43372:4405 56.04863:5089 57.03379:6323 57.98443:4912 57.99202:4277 60.20647:4843 60.25968:5493 60.31799:5181 62.54278:4039 62.55872:5808 62.6055:4660 63.19151:4160 65.00201:4659 67.59607:4722 70.19422:3861 70.68302:4177 73.4035:4236 73.95222:4955 74.05886:5136 74.43671:4852 75.43698:4801 77.62954:4853 | -2.8E-06 |

| Differences in metabolites between the Model group and the Control group | | | | | | | |
|---|---|---|---|---|---|---|---|
| Alignment ID | Metabolite name | Rt(min) | Expriement Mz | Adduct type | Reference m/z | MS/MS spectrum | PPM |
| POS7653 | | 9.493 | 353.34113 | M+CH3OH+H] | 353.34131 | 57.06989:8454 59.03007:18218 61.01003:11569 73.04688:11070 75.02497:19953 77.04092:10995 91.05576:16604 93.03564:21453 105.07176:6055 154.15424:5573 168.61324:5526 | -5.09E-07 |
| POS2116 | -3,4-dimethylpen | 4.537 | 147.10121 | [M+NH4]+ | 147.1015 | 55.05366:16159 59.04859:11035 64.92667:10670 71.04816:8067 72.08048:7752 84.07957:11127 86.05939:7596 90.46526:5589 91.0539:13732 118.0629:12580 | -1.97E-06 |
| POS9970 | Kermadecin C | 4.808 | 493.29453 | [M+H]+ | 493.2948 | 89.05896:14232 111.75651:8839 150.5957:6680 | -5.47E-07 |
| POS7205 | Gly Arg Val | 4.894 | 331.20853 | [M+Na]+ | 331.2088 | 50.59146:6949 89.05891:7436 173.42535:7672 186.45195:6156 | -8.15E-07 |
| POS8912 | Drenison | 4.493 | 437.233 | [M+Na]+ | 437.23331 | 52.80128:6734 68.74682:5393 246.60245:5675 301.61307:5685 | -7.09E-07 |
| POS12827 | Roseocardin | 5.425 | 608.40125 | [M+2H]2+ | 608.40088 | 57.03287:54218 59.04859:969901 60.05226:32435 73.02812:94965 73.06432:38505 75.78008:13408 80.0543:18675 83.0477:14981 85.06337:49265 87.04335:169178 87.06079:40880 87.07999:43729 89.05896:1091298 90.06374:33013 94.06947:26629 101.05931:158968 103.07444:259107 116.08248:23405 117.0909:25837 129.08911:31419 131.06766:21780 133.08545:392229 134.08684:16872 147.10149:119249 161.11441:15337 177.11285:36405 191.1256:26122 | 6.082E-07 |
| POS10867 | Ile Leu Ile Gly Thr | 5.137 | 530.35498 | [M+H]+ | 530.35468 | 59.04859:273368 73.02812:26540 73.06432:14036 87.04335:261049 87.06079:11436 89.05896:394889 90.06191:10841 101.05931:98209 103.03849:10424 103.07444:82931 117.05281:13607 131.06766:20291 133.08545:163177 147.10149:53517 177.11285:17248 191.13127:16924 200.78329:9637 | 5.657E-07 |
| POS5104 | oxyacetaminopher | 1.038 | 247.01546 | [M+NH4]+ | 247.01511 | 62.98163:7152 64.72823:6041 101.51242:6272 104.10692:24282 134.01678:5969 135.00177:247258 | 1.417E-06 |
| POS2118 | -3,4-dimethylpen | 5.027 | 147.10121 | [M+H]+ | 147.1015 | 55.05366:11502 56.04773:6884 59.04859:11893 64.92667:21080 67.82102:6960 69.06869:7496 70.45259:6235 72.08048:7240 76.67482:6051 86.05939:8729 91.0539:9974 100.07383:8196 118.0629:19497 130.08708:5411 | -1.97E-06 |
| POS8878 | Balaenonol | 4.701 | 435.25327 | [M+Na]+ | 435.25299 | 89.05894:16135 133.08542:6624 159.1517:5039 163.68777:5218 252.45139:5891 | 6.433E-07 |
| POS11213 | Val Ile Pro Lys Ser | 5.146 | 543.35034 | [M+2H]2+ | 543.34998 | 59.04858:120187 73.02811:23326 80.05428:13558 87.04333:28569 87.06078:11620 89.05894:233452 101.05929:19089 102.06604:7752 103.07442:30258 131.06763:8023 133.08542:75491 147.10146:16462 173.42542:14482 285.71133:8254 | 6.626E-07 |
| POS13154 | -3,4-dehydro-apo | 4.842 | 625.37299 | [M+Na]+ | 625.37341 | 585.47443:14342 | -6.72E-07 |
| POS2508 | Cleroindicin B | 4.932 | 159.10106 | [M+H]+ | 159.10139 | 53.68312:5904 55.05363:8822 55.93433:23564 56.0486:7073 57.06894:9653 58.07365:5503 66.47277:5330 69.06865:6803 70.06428:9016 71.01213:10431 71.04942:6172 76.93209:8708 104.37206:5536 | -2.07E-06 |
| POS13470 | lbutanoyl)-13-O-( | 5.195 | 645.43555 | [M+H]+ | 645.43597 | 59.04859:242310 66.66176:9597 73.02812:18954 87.04335:24629 89.05896:120566 101.05931:29662 103.07444:54756 117.05281:11269 133.08545:31894 147.10149:17486 155.30751:11078 599.66156:11795 | -6.51E-07 |
| NEG5133 | labda-13(16),14-d | 1.039 | 273.25922 | [M-H]- | 273.25891 | 51.7709:5912 52.92688:7048 87.11259:26638 89.13132:620403 90.13493:24973 91.84886:6244 93.03932:615230 95.0377:199293 97.03753:78373 103.07954:38911 128.18695:6126 136.98956:5688 145.2356:10087 147.15926:225151 209.7704:5888 | 1.134E-06 |
| POS1084 | Siphonodin | 6.062 | 115.03861 | M+CH3OH+H] | 115.0389 | 55.01768:6203 55.05363:10908 68.04948:7986 69.06865:13654 70.06428:11377 75.8084:5719 83.01192:103439 96.24216:6652 107.414:5775 107.7495:5746 114.06504:6739 114.09121:16709 | -2.52E-06 |
| POS10854 | Ala Ala Gln Asn Leu | 4.468 | 530.29352 | [M+2H]2+ | 530.29321 | 65.25937:5692 70.02776:17986 70.06428:5914 86.09537:38615 87.05377:74064 88.03892:7436 89.05892:49134 107.32559:6541 122.51595:5492 133.08539:30002 146.05714:7555 172.06981:10437 188.06996:26492 205.09813:8059 344.03888:5638 | 5.846E-07 |
| POS9165 | Cytochalasin II | 4.969 | 452.27783 | M+CH3OH+H]2 | 452.2775 | 52.35993:6926 59.04859:26203 87.04335:17260 89.05896:52371 101.05931:18825 133.08545:17198 173.43036:7924 321.76129:5831 | 7.296E-07 |
| POS7897 | yltricaprylylammo | 6.231 | 368.42371 | [M+H]+ | 368.42401 | 53.41133:6012 57.06989:9046 60.08025:22375 71.08418:8467 126.59031:6735 | -8.14E-07 |
| POS2503 | ethylene-L-glutar | 2.278 | 159.07599 | [M+H]+ | 159.07629 | 55.05365:7750 55.93345:25019 56.04862:12251 61.03972:9412 62.29601:5395 67.05389:13226 70.0643:38752 72.93572:6851 81.07617:6065 95.08477:17780 115.05257:5547 117.05824:7372 | -1.89E-06 |
| POS11453 | nino)-3beta-phtha | 4.923 | 551.34827 | [M+2H]2+ | 551.3479 | 50.11341:5893 61.73277:5959 89.05892:11118 104.10693:18044 104.21423:6262 133.08539:5544 | 6.711E-07 |

**Differences in metabolites between the Model group and the Control group**

| Alignment ID | Metabolite name | Rt(min) | Expreiment Mz | Adduct type | Reference m/z | MS/MS spectrum | PPM |
|---|---|---|---|---|---|---|---|
| POS12812 | loxanthin 3-aceta | 5.801 | 607.41406 | [M+2H]2+ | 607.41449 | 57.03287:71227 59.04859:547655 69.06868:16950 73.02812:65250 73.06432:23441 85.06337:27886 87.04335:75830 87.07999:18021 89.05896:323129 94.06947:22078 101.05931:67832 103.07444:165300 115.07643:17031 117.0909:26454 129.08911:15801 131.10312:15080 133.08545:87414 147.10149:65990 154.42589:15842 161.1188:17895 568.3938:14728 | -7.08E-07 |
| POS9908 | Mmv676063 | 5.958 | 490.28867 | [M+Na]+ | 490.289 | 59.04859:7609 60.08025:7720 69.77051:5489 86.09541:33568 104.10697:183850 146.97896:17678 190.10825:6852 345.91049:5415 | -6.73E-07 |
| POS13396 | propan-2-yl)-1,7,1 | 5.778 | 640.41736 | [M+2H]2+ | 640.41699 | 57.03287:18330 59.04859:110224 73.02812:21293 73.06432:10910 85.06506:9571 87.04335:33746 87.06254:11364 89.05896:105997 101.05931:33457 103.07445:20257 117.05281:10544 117.0909:9995 133.08545:43526 147.10149:14077 173.38622:9218 213.08313:8803 280.85791:9059 | 5.777E-07 |
| POS7300 | Pipericine | 6.74 | 336.32523 | [M+H]+ | 336.3255 | 50.50196:6109 112.50879:5338 156.31415:6028 218.8768:6308 | -8.03E-07 |
| NEG9902 | Oligomycin F | 8.8 | 803.53241 | [M-H]- | 803.53162 | 105.20542:5352 116.96082:5538 120.79679:5685 146.04282:5948 302.20255:6442 | 9.832E-07 |
| POS6713 | rophenyl)naphtha | 2.993 | 308.08005 | [M+2H]2+ | 308.07971 | 56.04862:7136 59.54773:6225 74.0055:5790 76.0215:8827 76.03858:24778 84.04315:132124 85.04819:7381 130.04883:46154 131.04509:11298 | 1.104E-06 |
| POS6641 | Fenpropimorph | 0.898 | 304.26031 | [M+NH4]+ | 304.26059 | 52.64424:6420 73.14485:5706 115.68862:6183 306.7052:6369 | -9.2E-07 |
| POS4707 | carnitine (isomer | 2.62 | 232.15359 | [M+H]+ | 232.1539 | 51.53652:7216 57.06986:11963 60.08022:18059 73.56039:5627 81.03226:7065 85.02796:114643 167.10455:17058 193.27718:5915 | -1.34E-06 |
| POS3967 | Dihydroxydecanoic | 5.167 | 205.14304 | [M+H]+ | 205.14339 | 59.04859:23058 89.05896:11043 146.061:6498 | -1.71E-06 |
| POS3968 | Dihydroxydecanoic | 5.045 | 205.14304 | [M+2H]2+ | 205.14339 | 53.84231:6575 54.14479:6940 88.4502:5964 102.45471:5068 108.92896:6128 110.70092:5999 173.43036:7271 | -1.71E-06 |
| NEG1818 | ndoleacetaldehyde | 1.412 | 158.06174 | [M-H]- | 158.0614 | 51.81568:5803 58.04419:57416 59.08405:10121 68.85773:6177 71.09771:5955 89.13128:114845 90.1349:6742 99.78008:6799 104.08418:5829 | 2.151E-06 |
| POS747 | lydroxy-2-pentan | 6.013 | 103.075 | [M+H]+ | 103.0753 | 59.04859:14124 61.0387:35706 | -2.91E-06 |
| POS369 | p-Cresol | 3.189 | 91.05404 | [M+H]+ | 91.0537 | 50.38117:6380 61.02845:9666 62.0318:14637 64.66679:6087 | 3.734E-06 |
| POS10761 | eryl-tyrosyl-leucyl- | 6.064 | 526.29065 | [M+H]+ | 526.29108 | 55.05363:43554 57.03284:78227 62.05907:419133 62.19157:40989 67.05386:109530 79.05309:72262 81.06987:91738 91.05386:77026 93.06837:65164 98.98411:42704 104.10693:54144 105.06941:73745 105.22224:45939 119.08607:63324 131.08371:45324 173.39104:98496 | -8.17E-07 |
| POS8037 | .!21-Acetoxypregr | 4.957 | 375.24762 | [M+H]2+ | 375.24799 | 57.03287:52016 58.04042:47946 59.04859:1374553 60.05226:30239 65.04819:56027 69.06992:15300 73.02812:258559 73.06432:31160 80.0543:63820 85.06337:34716 87.04335:197259 87.06079:104272 87.07999:20165 89.05896:1562559 90.06191:56718 94.06947:24597 101.05931:83424 102.06606:31793 103.07444:194860 104.07732:12680 107.07059:12543 109.07315:48915 116.08248:10905 124.08022:10182 129.08911:28066 131.06766:18310 131.08699:17165 133.08545:442284 134.09018:16067 147.10149:67507 173.42545:13363 177.1078:37213 191.1256:10836 | -9.86E-07 |
| NEG3335 | Dimethyl-9H-xant | 0.972 | 209.09691 | [M-H2O-H]- | 209.09731 | 59.08403:16279 71.09898:6542 89.13125:14394 93.03925:1463321 97.03745:18826 97.07645:21359 131.2403:46894 | -1.91E-06 |
| POS12382 | Ganoderic acid Mc | 5.054 | 587.39465 | [M+H]+ | 587.39423 | 57.03286:51786 59.04858:891911 60.05225:20113 73.02811:19863 87.04333:179564 87.07997:23421 89.05894:500972 100.07381:17644 101.05929:119738 103.03848:19811 103.07442:257044 117.09087:24642 129.09224:14715 130.08388:13756 133.08542:91030 147.10147:107219 161.11438:27688 191.12556:17070 203.61127:13223 247.26077:12099 | 7.15E-07 |
| POS8033 | aragylasparagyllys | 4.88 | 375.19821 | [M+H]+ | 375.19861 | 59.04859:47938 73.02812:17314 79.26034:5756 87.04335:17922 87.06254:7271 89.05896:59470 101.05931:8757 103.0385:20065 131.06766:5695 133.08545:12704 168.01765:6012 185.73782:5824 311.60825:5348 374.63766:5259 | -1.07E-06 |
| POS10474 | (18:4(6Z,9Z,12Z,15 | 4.654 | 516.30945 | [M+2H]2+ | 516.30902 | 73.02811:42732 73.06432:11415 80.05429:34236 87.04334:48191 89.05896:279765 90.06374:11849 91.04643:6372 102.06606:18262 124.08022:9198 133.08543:106621 162.54245:7142 177.11285:11255 309.0488:8241 | 8.328E-07 |

| Differences in metabolites between the Model group and the Control group | | | | | | | |
|---|---|---|---|---|---|---|---|
| Alignment ID | Metabolite name | Rt(min) | Expreiment Mz | Adduct type | Reference m/z | MS/MS spectrum | PPM |
| POS5829 | lquinoline 1-oxide | 4.868 | 274.18237 | [M+NH4]+ | 274.18201 | 55.05365:155349 57.03287:30183 57.06896:44646 61.01003:30787 67.05389:217736 68.05673:8080 69.06868:142022 71.04816:36076 79.05312:36470 81.06833:454771 82.07301:19347 83.0477:24995 83.08509:56492 85.00948:11202 85.06337:16910 91.0539:49244 91.93919:5980 93.06841:63061 95.08477:403021 96.08827:17496 97.06461:18138 97.09953:30213 105.06945:67244 106.07349:6510 107.08486:32951 109.06336:18807 109.10006:28658 111.0796:6349 113.05927:8230 115.07378:6267 117.06913:5785 119.08333:131978 120.08878:8567 121.1013:42594 123.08004:34238 127.01965:9520 127.075:10520 133.09863:54900 135.11646:87970 137.09427:11368 137.1322:413534 138.13437:30462 151.14491:5836 161.13197:163509 162.1382:11314 169.10333:28795 173.39111:12062 179.13977:50426 | 1.313E-06 |
| POS8643 | scutione | 4.921 | 419.25839 | [M+H]+ | 419.258 | 57.03287:7353 59.04859:32413 73.02812:7236 87.04335:17096 89.05896:45059 101.05931:9469 103.0385:17268 124.16041:5615 133.08545:14093 173.42545:10300 303.42111:5687 317.40564:5096 | 9.302E-07 |
| POS13578 | ethyl-5-methylide | 5.052 | 653.40283 | [M+H]+ | 653.40234 | 59.04859:22567 70.18664:14842 87.04335:20137 89.05896:97236 95.55244:12756 131.36464:13900 133.08545:17226 268.88205:15103 618.28107:17286 | 7.499E-07 |
| POS10623 | Phe Val Lys Lys | 8.815 | 521.34406 | [M+2H]2+ | 521.34448 | 60.08022:28979 86.09537:76667 87.09914:6393 104.10693:159350 105.10874:18973 124.9995:13415 159.69203:5840 173.42537:6516 184.06921:172363 318.78229:6125 | -8.06E-07 |
| POS14708 | Muronic acid | 5.168 | 750.51654 | [2M+H]+ | 750.51599 | 59.04859:215859 73.06432:32288 87.04335:32286 89.05896:250885 92.87973:18245 101.05931:39315 101.62239:17896 103.07445:71098 117.05281:55112 131.07088:20529 133.08545:96551 147.10149:54381 204.50024:21937 | 7.328E-07 |
| POS12580 | ethylanhydrovilang | 5.437 | 597.38049 | [M+2H]2+ | 597.38 | 59.04859:141038 67.015:20370 73.02812:16096 81.03072:18390 87.04335:27664 88.08155:12357 89.05896:193138 90.06191:13491 101.05931:41205 103.07444:39391 133.08545:75424 147.10149:13880 194.0645:14267 | 8.202E-07 |
| POS11348 | Eurycomaoside | 6.062 | 548.27057 | [M+Na]+ | 548.27014 | 53.59624:5870 59.04858:26783 89.05896:10466 91.0539:6945 104.10696:15133 120.96689:22884 | 7.843E-07 |
| POS4239 | Citric acid | 1.219 | 215.01562 | [M+H-H2O]+ | 215.01601 | 52.46179:6029 69.16247:5332 72.08047:8938 75.66121:6218 81.0699:19912 88.72058:5424 95.08477:6798 | -1.81E-06 |
| POS2777 | (E)-Lyratic acid | 8.914 | 167.10579 | [M+H]+ | 167.1062 | 53.93914:9285 84.95896:29778 103.05193:7594 112.97408:8555 119.39087:5587 120.08025:17724 125.05955:14703 | -2.45E-06 |
| POS12323 | 3a-pentamethyl-4 | 4.865 | 585.26538 | [M+H]+ | 585.26581 | 58.47013:5769 89.05896:14428 173.43034:11705 299.1311:5905 357.3071:6222 | -7.35E-07 |
| POS8335 | a,8-dimethyl-6-ox | 5.75 | 397.19965 | [M+H-H2O]+ | 397.20001 | 59.04858:23504 69.03294:8009 87.04333:5658 89.05895:12635 119.08332:75640 | -9.06E-07 |
| POS10498 | ylnaringenin7-O- | 6.21 | 516.99963 | [M+H]+ | 517 | 52.44873:4856 56.27097:4187 57.98917:5108 58.19838:4594 60.07725:4665 60.36026:5265 60.95378:5076 63.24981:4456 64.00186:4692 64.86604:4564 67.67013:4577 68.34462:4437 68.53918:4719 69.43006:5691 70.26247:4358 70.37391:4217 70.41577:4417 70.42593:4039 70.43608:4755 74.89813:4712 77.04238:4995 80.67318:5405 83.44244:4438 87.48277:4251 89.29951:5035 89.67585:4541 94.42513:4738 95.21034:4112 95.22631:3790 98.21039:4061 98.22293:4514 100.78278:4159 102.74039:4042 102.99358:4351 103.7225:4083 104.58311:3632 107.09676:4168 107.84329:4670 109.57148:4830 111.35164:4010 111.56139:3858 113.12126:4108 136.55806:4286 140.75113:4023 143.19608:5018 153.78905:3666 167.38799:5310 179.18613:4192 179.24796:4594 181.7372:4760 192.49483:5029 195.91818:4660 204.91539:4892 205.36974:5037 220.06386:4828 241.96165:4268 262.63007:4140 266.41821:4031 284.85248:4063 328.84229:4778 330.48773:4968 354.36633:4168 382.86893:4410 403.22705:5486 406.07657:4964 411.92078:4195 432.1152:4902 448.90985:6243 462.74417:4983 480.55896:4326 487.76578:5462 497.17715:4657 501.85251:4493 | -7.16E-07 |
| POS14575 | Mubenin B | 6.597 | 735.4624 | [M+H]+ | 735.46301 | 94.29323:5399 115.30742:6495 117.48097:6924 201.29752:6080 | -8.29E-07 |
| POS13937 | DTXSID60346618 | 8.778 | 679.47156 | [M+H]+ | 679.47198 | 65.3966:5920 198.67972:6253 272.09207:5880 290.05417:6865 | -6.18E-07 |
| POS13936 | DTXSID60346618 | 8.667 | 679.47144 | [M+2H]2+ | 679.47198 | 70.29156:5858 92.95089:5664 105.49634:6093 215.87585:5333 286.19946:6859 304.34259:5523 | -7.95E-07 |
| POS6151 | ncuronium bromi | 7.046 | 286.22839 | [M+NH4]+ | 286.228 | 73.09652:5308 113.8013:6307 | 1.363E-06 |

| Differences in metabolites between the Model group and the Control group | | | | | | | |
|---|---|---|---|---|---|---|---|
| Alignment ID | Metabolite name | Rt(min) | Expreiment Mz | Adduct type | Reference m/z | MS/MS spectrum | PPM |
| POS2031 | Ethephon | 6.504 | 144.9819 | [M+H-H2O]+ | 144.98151 | 53.03773:13579 55.01682:9119 55.05365:10583 57.03286:6811 62.92802:1139831 70.0643:47409 71.06874:6732 80.93837:9232 98.06006:32443 98.09552:15845 99.04338:23092 103.95428:45847 121.96451:462033 131.96162:6944 144.98274:31577 | 2.69E-06 |
| POS370 | p-Cresol | 4.389 | 91.05412 | [M+Na]+ | 91.0537 | 51.59062:7082 57.30673:5757 57.93421:6222 62.03075:6327 65.03805:19051 66.99615:6298 91.0539:28271 | 4.613E-06 |
| POS7164 | Incensole | 6.838 | 329.24548 | [M+H]+ | 329.24512 | 76.35284:6858 107.08486:6808 170.07565:5289 173.3911:10170 328.86789:6140 | 1.093E-06 |
| NEG1560 | Citramalic acid | 8.811 | 147.10043 | [M-H2O-H]- | 147.10001 | 59.08403:8234 74.67177:5613 101.05437:9917 102.05606:16315 103.05476:36319 | 2.855E-06 |
| NEG1185 | Phenyl vinyl sulfide | 7.569 | 135.02791 | [M-H]- | 135.0275 | 65.09129:31817 66.07739:17039 66.08893:7004 79.05219:10746 89.13127:23061 92.12341:6997 92.13479:10820 100.0459:6214 102.05609:9050 107.15221:13537 117.06139:10774 135.0251:24814 135.05542:20732 | 3.036E-06 |
| POS6410 | oleic acid, methyl | 1.207 | 295.26163 | [M+H]+ | 295.26199 | 51.64319:7048 55.05366:19076 57.06989:11474 67.05389:13073 69.06992:20634 69.89835:6757 81.06834:20028 83.0851:14047 95.08477:10860 97.09953:8127 107.08487:7368 121.1013:7297 133.09863:10153 | -1.22E-06 |
| POS1349 | 2,4-Triaminobenze | 8.729 | 124.08641 | [M+H]+ | 124.0868 | 61.37498:5859 77.49311:6051 96.20368:6753 | -3.14E-06 |
| POS12448 | Austrobuxusin I | 4.568 | 591.35358 | [M+2H]2+ | 591.35303 | 73.06431:106240 87.04333:602119 89.05895:4109172 90.0619:73522 117.08816:56878 131.06764:114271 133.08543:1918621 134.08682:60913 173.43523:48508 175.09525:51397 177.11284:220419 296.69055:30865 570.67053:33803 581.78009:35843 | 9.301E-07 |
| POS12196 | odeoxycholyltrypt | 5.331 | 579.38055 | [M+2H]2+ | 579.38 | 57.03287:49203 59.04859:1127110 60.05226:37743 73.02812:124180 73.06432:32703 80.0543:27308 85.06506:35434 87.04335:219358 87.06079:43940 87.07999:42551 89.05896:1293560 90.06191:46038 94.06947:25213 101.05931:167521 103.07444:325522 109.0756:35468 116.08248:25592 117.0909:33581 129.08911:42456 131.06766:25643 133.08545:482817 134.08684:31299 147.10149:172306 161.11441:30490 177.11285:48489 191.1256:36244 | 9.493E-07 |
| POS11050 | ydrocelastryl diace | 4.821 | 537.32159 | [M+Na]+ | 537.32111 | 51.77975:10735 53.44824:10138 172.19113:9965 384.88806:10550 | 8.933E-07 |
| POS11667 | oxy-22,25-oxido- | 4.872 | 559.3634 | [M+H]+ | 559.36292 | 59.04858:164065 87.04334:24241 89.05896:243012 101.0593:19786 103.07444:37613 133.08545:52739 147.10149:11455 184.7793:10282 252.30504:9418 | 8.581E-07 |
| POS11611 | Gln Val Leu Leu Gly | 5.352 | 557.36609 | [M+2H]2+ | 557.3656 | 57.03287:13623 59.04858:238876 69.06992:8568 73.02811:21019 73.06432:8827 85.06505:6937 85.42169:5890 87.04334:38228 87.06078:12802 87.07998:9133 89.05896:238150 90.0619:8125 101.0593:34692 103.07444:61192 109.07559:6788 109.61583:5807 110.70842:6597 129.08911:6293 133.08543:81501 147.10149:31376 173.39601:8739 226.88602:5403 359.24414:5833 | 8.791E-07 |
| POS10524 | lemonnierin | 8.857 | 518.29767 | [M+H]+ | 518.29718 | 65.91531:6574 81.0699:13970 85.06506:15727 93.07034:7524 95.08477:15201 104.10696:29663 105.06945:8505 107.08486:11646 109.10006:13271 121.09843:9822 126.02023:100495 127.02272:19039 128.01544:50965 131.08377:7190 133.09863:8719 135.11646:6467 145.09904:8818 147.11681:9320 157.10197:6557 159.11723:15079 160.12202:11101 199.14969:12618 200.14934:6864 208.06328:12433 209.13087:10801 213.1633:7460 227.14313:9550 505.616:6482 | 9.454E-07 |
| POS7395 | Myrsinoic acid B | 4.502 | 341.21442 | [M+H]+ | 341.21399 | 59.04859:420374 73.06432:26204 85.06506:15104 87.04335:166315 89.05896:2371431 90.06191:49744 96.08018:10842 101.05931:8375 103.03849:13936 103.07444:64185 107.07059:21353 114.09126:11675 129.08911:9317 131.07088:15901 133.08545:565314 134.08684:17578 147.10149:24456 173.43036:8183 177.11285:26297 | 1.26E-06 |
| POS11845 | ïæ¨ å å 4 | 4.528 | 565.35852 | [M+2H]2+ | 565.35822 | 87.04333:35172 89.05895:298107 90.0619:48146 133.08543:136004 134.08684:29761 140.82646:19314 173.3911:40907 176.78931:20847 314.63065:18759 487.51135:21257 | 5.306E-07 |
| POS9389 | Stellettin B | 4.912 | 463.28534 | [M+H]+ | 463.2858 | 59.04859:17473 73.02812:6506 80.0543:6441 87.04335:9064 89.05896:27470 103.0385:7367 133.08545:13761 234.66223:5886 | -9.93E-07 |
| POS8470 | rihydroxy-6-methy | 4.528 | 407.22345 | [M+Na]+ | 407.22299 | 62.14423:6016 86.05939:5706 163.52153:5868 258.34149:6538 | 1.13E-06 |

**Differences in metabolites between the Model group and the Control group**

| Alignment ID | Metabolite name | Rt(min) | Expreiment Mz | Adduct type | Reference m/z | MS/MS spectrum | PPM |
|---|---|---|---|---|---|---|---|
| POS12380 | yloxy)icosanoyl]ox | 5.143 | 587.37946 | [M+2H]2+ | 587.37891 | 59.04859:361571 72.83016:28106 73.02812:91944 73.06432:36273 87.04335:142063 87.06254:31344 89.05896:826186 90.06191:26877 101.05931:88199 103.07444:125138 106.91372:28114 129.08911:28422 133.08545:281272 147.10149:61475 173.42545:34963 177.11285:64965 185.83572:27589 | 9.364E-07 |
| POS9624 | Loperamide | 4.491 | 477.23071 | [M+H]+ | 477.23029 | 72.08047:7460 73.06432:10341 86.05939:12046 86.09541:6800 87.04334:24231 89.05896:357135 90.0619:24425 98.06007:6841 112.07434:29439 119.08612:16395 120.0803:10075 133.08543:163641 134.08684:13166 136.52037:6027 157.00053:5902 177.11285:22950 291.63074:5845 | 8.801E-07 |
| POS11321 | Cholylarginine | 4.913 | 547.38452 | [M+H]+ | 547.38501 | 59.04856:201737 87.0433:19741 89.05891:174987 101.05926:35441 103.07439:54084 117.05275:24089 133.08537:41112 147.10141:25965 173.38612:22581 185.46078:14998 267.18558:15823 | -8.95E-07 |
| POS12977 | Leu Gln Lys Ser Leu | 5.217 | 616.40222 | [M+2H]2+ | 616.40277 | 57.03287:12818 59.04858:170867 73.02812:34173 87.04334:107092 87.06079:10222 88.04782:9812 89.05896:280759 101.0593:50900 102.06606:10401 103.07444:31834 133.08545:113618 145.04276:8984 147.10149:18346 177.11285:11702 348.84436:9617 | -8.92E-07 |
| POS9288 | Gly Val Ile Gly Ile | 5.385 | 458.29684 | M+CH3OH+H]2 | 458.29721 | 57.03286:11500 59.04858:162045 60.05225:6547 61.36362:5549 67.01499:11342 81.03072:11099 87.04333:13940 89.05895:83040 101.0593:12643 103.07443:36718 125.31528:5654 133.08543:16914 141.435:5171 147.10147:11008 158.09196:7295 165.41951:5772 | -8.07E-07 |
| POS12102 | brujavanone D | 4.69 | 575.35828 | M+CH3OH+H] | 575.35779 | 59.04859:11804 64.65227:7554 70.12607:6071 73.02812:13099 79.72459:6653 87.04335:34803 89.05896:179418 94.8423:6089 100.51604:5904 103.07444:6306 111.04441:6606 133.08545:69356 177.11285:11363 189.74847:6204 243.21132:6574 | 8.516E-07 |
| POS12099 | raspiridole B_1300 | 4.457 | 575.31354 | [2M+H]+ | 575.31403 | 88.99577:8921 89.05891:64503 121.77953:7621 133.08537:31569 188.06995:14284 | -8.52E-07 |
| POS13697 | ,18-hexa(propan-. | 6.477 | 662.3974 | [M+Na]+ | 662.39801 | 51.71899:51994 74.05202:54767 155.46558:50627 173.39111:118150 367.89346:58816 | -9.21E-07 |
| NEG6605 | bicyclo[4.1.0]hept- | 1.021 | 325.14819 | [M-H]- | 325.14774 | 55.93273:5822 64.72199:5727 89.13125:15144 93.03925:471780 95.03763:46740 97.03745:13607 105.56303:5625 124.46915:5651 127.20192:7845 134.22708:7003 145.23547:44896 151.06712:33643 | 1.384E-06 |
| POS7904 | erone 3-sulfate so | 5.752 | 369.17142 | [M+H]+ | 369.17099 | 57.06894:21290 59.04953:11239 79.15736:5939 79.33614:5442 85.06333:11476 89.05892:10233 | 1.165E-06 |
| POS9047 | Difenacoum | 3.357 | 445.18066 | [M+H]+ | 445.18109 | 50.11115:6874 137.04604:132977 145.24556:5233 160.07417:39036 179.40268:6166 | -9.66E-07 |
| POS3783 | Tacrine | 4.594 | 199.12416 | [M+H]2+ | 199.1246 | 73.51437:5165 75.64143:6344 81.06833:8050 | -2.21E-06 |
| POS1648 | ethylthiopentan-3 | 4.817 | 133.06772 | [M+Na]+ | 133.06816 | 67.803:5709 69.06989:39772 82.45757:6350 86.09537:158292 87.09914:6806 104.04764:25055 105.03241:6749 133.05241:12601 | -3.31E-06 |
| POS7800 | [12]-Shogaol | 6.047 | 361.27246 | [2M+H]+ | 361.27289 | 55.05366:8866 57.03287:8714 57.06896:5744 57.37107:5850 67.05389:19133 67.47569:5805 69.06869:6543 79.05313:12329 81.0699:19388 91.0539:16121 93.06841:10870 95.08478:10353 105.06946:8554 119.08613:10188 173.39111:10269 | -1.19E-06 |
| POS2369 | ethyl propyl trisulfi | 9.136 | 155.00212 | [M+H]+ | 155.00169 | 51.94016:10242 55.05365:17822 55.93345:133211 67.05389:30009 67.93391:7764 71.94005:16588 72.93706:1124896 73.93582:6224 79.05463:6605 81.06989:14737 90.94762:214569 97.5863:6159 108.08179:9968 108.95824:27118 113.96311:308913 114.97044:26824 126.97046:6325 131.97466:103719 | 2.774E-06 |
| NEG1006 | Agmatine | 0.889 | 129.11505 | [M-H]- | 129.11459 | 62.06304:9145 88.1461:11047 | 3.563E-06 |
| POS4898 | OPC-4:0 | 6.191 | 239.16335 | [M+H]+ | 239.1638 | 55.01769:7056 55.05452:11522 57.06987:16397 67.05388:6518 81.06988:9457 95.08475:19134 107.1801:6125 109.06334:22417 109.10004:6309 119.08609:11449 123.08001:29059 179.13972:130632 | -1.88E-06 |
| POS1215 | de, methyl tert-pe | 1.053 | 119.08923 | [M+H]+ | 119.0888 | 53.03773:49580 55.05365:2183662 56.04951:38541 56.05673:209583 57.05692:204000 59.04858:37663 72.08047:2599418 73.08309:235070 | 3.611E-06 |
| POS7456 | IC ACID DER (FR. I | 6.406 | 343.28378 | [M+2H]2+ | 343.28421 | 60.08125:10091 69.71296:6009 75.02497:9170 82.06503:9029 86.0954:24935 124.99955:8473 145.3998:6233 184.06929:46920 264.26468:14336 | -1.25E-06 |
| NEG1561 | Citramalic acid | 0.646 | 147.10046 | [M-H]- | 147.10001 | 59.0295:16415 59.08405:16129 65.60266:6277 73.20889:6079 79.5448:5428 | 3.059E-06 |
| POS5562 | Z)-Octadecadien | 6.776 | 263.23553 | [M+H]+ | 263.23599 | 201.77066:5538 257.78946:5736 | -1.75E-06 |

| Differences in metabolites between the Model group and the Control group | | | | | | | |
|---|---|---|---|---|---|---|---|
| Alignment ID | Metabolite name | Rt(min) | Expreiment Mz | Adduct type | Reference m/z | MS/MS spectrum | PPM |
| NEG471 | 1,2,4-Butanetriol | 0.337 | 106.06342 | [M-H]- | 106.063 | 53.45897:4091 54.47842:4136 56.19134:5904 59.97625:4719 60.3217:4124 62.3273:4462 62.51997:3862 62.68903:4419 64.66617:4102 64.67733:4571 65.33318:4541 65.69171:4110 68.71078:4153 68.82339:5140 68.90435:4902 69.84071:4193 70.78477:4560 74.1647:4320 76.61039:4261 76.85421:4962 81.62371:4848 82.3423:4234 86.58962:5238 90.25441:4335 92.34967:4441 95.33667:4001 96.94727:5738 101.89474:4618 109.04345:4326 | 3.96E-06 |
| POS12057 | SNF-4794-12 | 4.934 | 573.38245 | [M+H]+ | 573.3819 | 57.03287:155241 59.04859:3207844 60.05226:53694 73.02812:126239 73.06432:67632 85.06337:85421 87.04335:737397 87.07999:92912 89.05896:3782734 90.06191:78200 101.05931:518869 101.09423:32070 103.03849:110417 103.07444:1274112 104.07732:51424 105.09028:30692 107.06821:33453 115.07378:44584 117.05281:62599 117.0909:75995 129.08911:65900 131.06766:93031 131.10634:47827 133.08545:1309605 134.08684:47921 143.10413:30559 145.08403:79916 147.10149:917573 148.1028:29181 161.1188:78016 177.11285:104586 191.1256:143377 205.14238:58272 | 9.592E-07 |
| POS8339 | Rollicosin | 4.953 | 397.259 | [M+2H]2+ | 397.25851 | 57.03284:27986 59.04856:732509 60.05223:15843 65.04928:7615 69.06865:6942 73.02808:110844 73.06429:38310 80.05426:15877 85.06501:17184 87.0433:98442 87.06249:24442 87.07994:13465 89.05891:741093 90.06187:22602 94.06942:8094 100.07593:7046 101.05926:50173 102.06822:11634 103.07439:120326 104.07727:7604 107.06815:8568 109.07554:12543 117.05275:34792 117.09084:10051 129.09219:7225 130.08702:20660 131.07082:25295 133.08537:161299 134.08678:9455 147.10141:40354 161.11433:8025 173.39594:6746 177.11278:11449 | 1.233E-06 |
| POS12516 | Destruxin B | 5.228 | 594.38672 | [M+2H]2+ | 594.38611 | 57.03287:26272 59.04859:538786 73.02812:101193 73.06432:24663 73.49814:17151 80.0543:19289 85.06337:28720 87.04335:178714 87.06254:36547 87.07999:24217 89.05896:1022678 90.06191:35777 101.05931:79315 102.06606:39869 103.07444:150524 109.0756:24944 114.26157:13530 129.08911:22954 131.06766:27389 133.08545:384610 134.09018:27651 147.10149:90130 177.11285:48414 230.37541:13202 233.37059:13654 262.24658:15602 276.94675:14044 | 1.026E-06 |
| POS9151 | -5 trisulfate hexah | 5.267 | 451.28793 | [M+2H]2+ | 451.28741 | 57.03287:15085 59.04859:185668 67.015:17826 73.02812:7320 73.06432:9437 73.48596:7111 81.03072:14132 87.04335:17803 89.05896:148284 101.05931:29580 103.07444:35713 115.07378:6390 133.08545:38886 147.10149:13744 158.08772:5226 226.99617:5283 | 1.152E-06 |
| POS10896 | Ser Ile Arg Arg | 4.69 | 531.33557 | [M+H]+ | 531.33612 | 59.04858:43160 87.04333:65942 87.26875:22683 89.05894:465193 133.08542:152319 173.43033:28266 177.11282:27108 259.75623:19243 530.31659:22636 | -1.04E-06 |
| POS11729 | ntibiotic Sch 2383 | 5.238 | 561.35284 | [M+2H]2+ | 561.35339 | 59.04859:87533 67.015:18922 73.06432:11342 87.04509:14094 89.05896:127367 93.83868:7235 103.07445:20350 117.05553:9711 133.08545:51224 147.10149:11926 428.1304:7850 477.49673:7311 | -9.8E-07 |
| POS13779 | -Dibenzoyl karoun | 5.304 | 667.43494 | [M+2H]2+ | 667.43561 | 59.04859:242078 73.02812:68694 84.68888:23790 87.04335:82863 89.05896:540016 101.05931:60546 103.07444:63152 117.0909:28913 130.72673:29701 133.08545:202787 225.53427:29601 | -1E-06 |
| POS8754 | Arg Asp His | 4.437 | 427.2052 | [M+Na]+ | 427.20471 | 59.04956:6979 86.05939:7605 87.04335:5502 89.05896:15923 103.03849:13665 111.0419:5450 118.0629:26205 132.07892:13821 134.09685:11988 144.0797:16425 146.061:110536 147.06319:5783 159.09138:14633 188.07004:314637 189.07664:16750 205.09822:59622 | 1.147E-06 |
| POS5814 | Alismorientol A | 5.97 | 273.20508 | M+CH3OH+H] | 273.2056 | 55.32309:6444 55.35403:5615 57.03287:8367 57.06989:6506 82.75266:6326 | -1.9E-06 |
| POS12499 | Podototarin | 6.134 | 593.43353 | M+CH3OH+H] | 593.43292 | 50.67118:5669 54.03799:5682 57.03287:294743 59.04858:94944 67.95436:5881 81.06833:6117 99.07938:36638 101.09641:8090 107.9203:5749 115.07378:26630 117.08817:17043 137.96021:5530 157.12312:42351 173.43034:10610 | 1.028E-06 |
| POS4818 | ethoxyphenyl)isoq | 5.91 | 236.10951 | [M+H]+ | 236.11 | 57.06896:21774 141.5977:5960 174.85666:6375 | -2.08E-06 |
| POS5817 | Alismorientol A | 6.138 | 273.20511 | [M+H-H2O]+ | 273.2056 | 180.81987:6144 273.90359:6008 | -1.79E-06 |

| Differences in metabolites between the Model group and the Control group | | | | | | | |
|---|---|---|---|---|---|---|---|
| Alignment ID | Metabolite name | Rt(min) | Expreiment Mz | Adduct type | Reference m/z | MS/MS spectrum | PPM |
| NEG9489 | PFSA-ether | 5.373 | 530.91949 | [M-H]- | 530.92004 | 56.03255:8255 75.12117:5831 123.12397:138146 256.54803:6117 364.29367:5817 | -1.04E-06 |
| POS6744 | -glucopyranosyl-1 | 4.867 | 309.19022 | [M+H]+ | 309.1907 | 55.0177:10556 57.03287:103329 59.04859:1483114 60.05226:15732 71.04816:26645 73.02812:31427 73.06432:408673 87.04335:42986 89.05896:10118 103.07445:11926 117.05281:211554 117.0909:37128 131.06766:139032 | -1.55E-06 |
| POS5815 | Alismorientol A | 5.797 | 273.20508 | [M+H]+ | 273.2056 | 50.07689:6175 72.35727:6141 82.85947:5386 87.14983:5554 182.26979:5537 | -1.9E-06 |
| POS7078 | xanecarbonyltetra | 8.014 | 324.32507 | [M+H]+ | 324.32559 | 51.92889:6355 62.49498:5815 65.37954:5827 87.17076:5573 105.47303:6335 144.18367:5420 221.77029:6139 | -1.6E-06 |
| POS3888 | lydroxy-L-tryptop | 3.068 | 203.08051 | [M+H-H2O]+ | 203.08099 | 55.05365:15338 56.05764:6963 60.04426:13778 61.03972:9841 98.06007:7464 130.06477:8421 134.57501:6771 157.07237:7125 | -2.36E-06 |
| POS4165 | Elaeokanine C | 4.978 | 212.16383 | [M+H-H2O]+ | 212.16431 | 55.05365:14712 57.06988:6819 60.26771:6333 67.05389:7738 69.06991:12367 81.06989:8879 83.08508:10264 93.0684:12155 105.40099:6211 107.08485:9791 | -2.26E-06 |
| NEG398 | Styrene | 8.114 | 103.05589 | [M-H]- | 103.0554 | 50.26:5653 57.10277:13123 58.51401:6316 59.08403:19211 60.29253:5787 61.07175:5855 64.04998:6164 73.11619:8279 88.9059:5414 103.16489:8181 | 4.755E-06 |
| POS1585 | )-2-Pentanol aceta | 5.696 | 131.10608 | [M+H]+ | 131.1066 | 56.04953:7138 66.73078:6452 84.04482:8608 84.07957:26514 86.09541:23548 88.00349:46180 91.0539:15445 97.00918:17650 99.51076:6357 105.87435:5223 115.96171:8913 131.08377:8452 | -3.97E-06 |
| POS7115 | (-)-Spectaline | 6.981 | 326.30392 | [M+H]+ | 326.30441 | 62.06014:7482 83.74775:5349 111.98013:5167 126.20273:5299 156.22183:5575 170.93153:6539 | -1.5E-06 |
| POS6104 | adecanoyloxyprop | 7.722 | 284.29349 | [M+H]+ | 284.29401 | 50.15384:6115 55.83028:5610 70.08572:6014 73.98637:5237 173.38622:15165 205.2939:6562 | -1.83E-06 |
| POS477 | 5Z)-1,3,5-Heptatri | 0.704 | 95.08558 | [M+H]+ | 95.08607 | 55.05365:40419 60.81196:6018 65.03804:9508 67.05389:59072 95.04893:12522 95.08476:16068 | -5.15E-06 |
| POS5136 | Estrane | 7.14 | 247.24088 | [M+H-H2O]+ | 247.24139 | 60.68689:7013 68.0495:6114 101.14882:5995 130.32001:6294 189.62503:5234 | -2.06E-06 |
| POS10064 | 1a,4,4a,5,6,7,7a,10 | 5.543 | 498.28558 | [M+H-H2O]+ | 498.28503 | 57.06989:52798 59.04858:117315 60.08025:319774 61.08378:61635 71.0726:40468 80.26237:38436 86.09541:637366 87.04334:89207 87.09918:212624 89.05896:111737 104.10696:3389616 105.10879:901230 106.11105:71854 124.42527:38870 124.99955:143348 126.02023:198528 143.13353:36345 173.43034:49780 184.07466:1275888 185.07635:350369 186.07533:109538 | 1.104E-06 |
| POS6421 | clopropylethenyl)- | 8.844 | 296.14386 | [M+2H]2+ | 296.14441 | 55.0177:7234 55.05365:61963 57.06896:421288 58.06511:67268 58.07177:17376 59.07198:43990 60.08025:34325 67.05389:84423 69.06868:48493 71.0726:10686 71.08547:201446 72.08836:9254 76.53945:6980 81.06833:84160 83.08509:50908 85.10045:85449 86.0954:100834 95.08477:125856 97.09953:24544 102.09042:12012 104.10696:104389 109.10006:38765 123.11816:12654 124.99955:18668 137.5089:10278 149.01665:18663 156.51999:16360 157.01743:7404 165.52467:12755 184.07465:21663 193.53142:9053 256.00317:23735 | -1.86E-06 |
| POS13106 | homodestcardin | 5.66 | 622.41663 | [M+2H]2+ | 622.4173 | 57.03287:119903 59.04859:1589836 69.06868:36093 73.02812:141734 73.06432:68803 85.06337:71467 87.04335:301380 87.06254:61232 87.07999:69568 89.05896:1353998 90.06191:32242 94.06947:52451 101.05931:317615 102.06384:30548 103.07444:550777 115.07378:36005 116.08248:32758 117.08817:47840 129.08911:66206 131.07088:55611 131.10634:49848 133.08545:577301 134.09018:40175 143.10413:33472 145.08403:32688 147.10149:335780 161.1188:45661 173.11685:31273 177.11285:74070 191.1256:68499 205.14238:45497 | -1.08E-06 |
| POS10119 | tidylethanolamine | 5.495 | 500.27765 | [M+H-H2O]+ | 500.2771 | 59.04857:49635 62.06013:11238 67.21217:5362 87.04333:8936 89.05894:37492 103.07442:9126 104.10694:12132 126.02021:44426 127.02269:6110 128.01852:20755 | 1.099E-06 |
| POS3197 | Nicotinuric acid | 4.505 | 181.05997 | [M+H]+ | 181.0605 | 51.02236:7006 65.03804:6560 68.75417:5609 78.0336:77533 96.04379:539695 97.04613:9670 97.21471:5970 106.02893:12081 115.702:6127 124.03867:318359 135.05576:213309 173.3862:8053 | -2.93E-06 |
| NEG1756 | lethylpseudoconhy | 1.56 | 156.14005 | [M-H]- | 156.1395 | 78.18732:5748 82.16454:20570 89.13125:113313 97.07645:9456 111.15351:7432 151.103:5303 | 3.522E-06 |
| POS11732 | -)-Strempeliopidir | 6.844 | 561.39569 | [M+Na]+ | 561.39508 | 53.71611:5663 95.83591:6661 158.69135:6209 261.57294:5663 273.44662:6200 | 1.087E-06 |
| POS5134 | Estrane | 5.843 | 247.24084 | [M+H]+ | 247.24139 | 79.05312:6558 93.06841:11853 107.08486:7921 | -2.22E-06 |

| Differences in metabolites between the Model group and the Control group | | | | | | | |
|---|---|---|---|---|---|---|---|
| Alignment ID | Metabolite name | Rt(min) | Expreiment Mz | Adduct type | Reference m/z | MS/MS spectrum | PPM |
| POS3017 | densispicnin D | 4.881 | 175.13225 | [M+H-H2O]+ | 175.1328 | 53.40379:6508 59.04858:7105 70.78779:5501 94.23231:6106 104.95851:5791 106.06411:14832 130.06477:32586 134.06017:17831 173.39111:13848 | -3.14E-06 |
| POS2883 | ramethyl-4-piperi | 1.175 | 171.14856 | M+CH3OH+H] | 171.14909 | 55.05365:32473 58.06512:13894 67.05389:27210 69.06992:6107 72.04369:42975 83.08509:7684 89.06979:9185 109.10006:7372 118.74913:5687 | -3.1E-06 |
| POS2917 | anethiosulfonic ac | 9.369 | 172.97636 | [M+H]+ | 172.9758 | 55.05366:23586 55.05892:6892 55.93345:32149 57.73019:6022 67.05389:78850 70.06431:6554 71.04945:6566 71.92827:70420 72.65827:5430 72.93707:64732 81.0699:10823 87.03812:6491 89.93906:65184 90.90295:65891 90.94764:22434 95.08477:6469 107.9492:38478 109.10007:25251 114.97046:10998 125.9595:24131 126.09015:8907 130.9646:25725 131.92909:12921 132.86481:5588 148.97369:38969 149.93901:14493 | 3.237E-06 |
| POS8789 | '-Diapolycopened | 5.797 | 429.27969 | [2M+H]+ | 429.27911 | 57.03287:30988 57.06896:36714 60.08025:325791 71.08547:35847 85.02799:1805681 86.03024:139674 95.08477:25251 144.10197:24246 246.25351:11792 | 1.351E-06 |
| POS10669 | LPE 19:0-d5 | 5.401 | 523.35559 | [M+H]+ | 523.35498 | 60.08022:1087168 86.09537:1949797 87.09914:264323 91.09866:145234 104.10692:9567680 105.10874:1173202 105.7948:130571 124.9995:376109 145.09148:136445 173.43027:289912 184.06921:3053278 185.07626:403079 204.46875:140514 208.62509:134079 261.91843:153349 | 1.166E-06 |
| POS12377 | nagitide A | 4.535 | 587.32336 | [M+NH4]+ | 587.32269 | 59.04858:33429 69.03294:308951 73.02811:92996 73.06432:57698 81.03229:36420 83.0477:130652 85.02799:23950 87.04334:596637 89.05896:1689632 90.0619:30548 95.04893:60869 99.04339:492689 107.06821:29161 111.04189:250668 113.05927:37193 117.09089:32262 124.24364:22068 125.0596:122443 129.05446:44234 131.07088:65241 133.08543:647032 137.05981:42742 151.09705:22558 155.07083:154022 173.07771:39173 175.09526:23181 177.11285:43105 433.12009:19764 531.20953:18946 | 1.141E-06 |
| NEG4961 | -/-)-Apomorphine | 0.986 | 267.12653 | [M-H2O-H]- | 267.12592 | 87.11253:36435 89.13125:15772 93.03925:2156657 95.03763:9071 97.03951:6007 122.80492:5907 151.06712:82487 | 2.284E-06 |
| POS8243 | Bacithrocin A 2 | 4.878 | 390.25101 | [M+2H]2+ | 390.2504 | 57.03287:28517 58.04042:61799 59.04859:810843 60.05226:18014 65.04819:32205 69.06992:12328 73.02812:311303 73.06432:37208 80.0543:152726 85.06337:31220 87.04335:212354 87.06079:100912 87.07999:18149 87.56366:9811 89.05896:1659588 90.06191:47156 94.06947:11847 101.05931:59372 102.06606:90634 103.07444:111592 107.07059:17137 109.0756:40923 111.07206:7925 117.0909:9068 118.07944:8454 124.08022:36657 129.08911:21721 131.06766:22242 131.08699:17347 133.08545:518030 134.08684:24749 140.09358:8830 147.10149:50140 162.10718:10227 173.11685:8866 173.39111:15223 177.11285:46600 191.1256:7760 | 1.563E-06 |
| POS14943 | xy-3-oxochol-4-e | 6.74 | 777.52911 | [M+H]+ | 777.53003 | 62.33298:5719 77.24027:5977 173.43034:14701 | -1.18E-06 |
| POS11315 | 34-oxamanzamin | 6.191 | 547.34375 | [M+H]+ | 547.34308 | 50.72232:13760 60.08022:29099 72.07124:11772 76.34278:12631 76.75559:11365 86.09536:55042 104.10692:204612 105.10873:83869 106.111:29289 112.98438:11424 166.01965:10978 184.06921:58662 185.07626:30063 292.86453:11391 381.84113:11651 419.16068:13625 531.78821:11194 | 1.224E-06 |
| POS8790 | '-Diapolycopened | 5.995 | 429.27969 | [M+Na]+ | 429.27911 | 67.01499:23138 68.83137:5620 85.02631:6440 97.0256:21060 107.27312:5940 140.07933:9545 156.05841:5539 166.37865:6072 213.10983:6838 251.07872:6323 | 1.351E-06 |
| POS10243 | ctapropylene glyc | 5.391 | 505.33411 | [M+Na]+ | 505.33472 | 53.72115:6428 59.04856:11800 87.04504:5356 89.05891:18520 106.9778:6131 307.71118:5652 | -1.21E-06 |
| POS8674 | SCHEMBL9230589 | 5.663 | 421.31528 | [M+H-H2O]+ | 421.31589 | 52.03271:6102 54.90479:5597 57.06893:21297 64.94799:5750 150.03362:6041 173.42535:9497 335.32715:5905 | -1.45E-06 |
| POS10853 | Lucidamine B | 4.455 | 530.28937 | [M+H]+ | 530.28998 | 70.02779:27691 70.25867:6630 86.0954:72075 87.0538:163077 88.03895:13858 88.05669:7621 89.05895:42678 133.08543:20422 136.39027:6852 144.07225:8666 172.06985:29347 188.07002:17350 331.34097:9057 344.17609:6824 | -1.15E-06 |
| POS8201 | Sufentanil | 5.612 | 387.21133 | [M+H]+ | 387.21075 | 66.28812:5293 149.63585:5504 | 1.498E-06 |
| POS6552 | -oxo-3,4,4a,5,6,7- | 1.043 | 301.13873 | [M+H]+ | 301.13931 | 67.55789:6126 74.91763:5349 94.50796:6279 | -1.93E-06 |
| POS14067 | PS(P-16:0/14:1(9Z) | 8.704 | 690.46942 | [M+2H]2+ | 690.46863 | 62.46531:6290 118.79361:5989 173.3911:12671 | 1.144E-06 |

| Differences in metabolites between the Model group and the Control group | | | | | | | |
|---|---|---|---|---|---|---|---|
| Alignment ID | Metabolite name | Rt(min) | Expreiment Mz | Adduct type | Reference m/z | MS/MS spectrum | PPM |
| POS15338 | bicornutin A2 | 8.719 | 859.58398 | [M+2H]2+ | 859.5849 | 62.51623:5267 95.05093:4735 100.53985:5514 196.43747:5891 260.47702:5822 354.30902:5549 376.05884:5837 | -1.07E-06 |
| POS6126 | )-Isopropylidene u | 8.945 | 285.10867 | [M+2H]2+ | 285.10809 | 50.45882:5582 54.38173:5837 57.06989:9494 108.00944:7526 126.02023:100702 133.29341:6084 230.44301:5340 | 2.034E-06 |
| POS8349 | ngoramine\|15-Ace | 6.689 | 398.23312 | [M+H]+ | 398.23251 | 50.02518:5903 53.62407:6384 65.30698:4998 67.58175:6532 102.40796:5349 | 1.532E-06 |
| POS5487 | ,6,7-hexahydro-2 | 5.97 | 261.13385 | [2M+H]2+ | 261.13327 | 57.03287:50469 57.54692:5185 61.26566:5423 109.75152:6913 | 2.221E-06 |
| POS4547 | ty-10-methylunde | 8.399 | 226.21539 | [M+H]+ | 226.216 | 67.77902:6710 87.61996:5760 194.30267:6060 201.20547:6926 | -2.7E-06 |
| POS3548 | SCHEMBL536577 | 4.957 | 191.12712 | [M+2H]2+ | 191.1277 | 59.04859:20309 89.05896:14193 169.03723:6139 | -3.03E-06 |
| POS3547 | SCHEMBL536577 | 5.284 | 191.12712 | M+CH3OH+H] | 191.1277 | 59.04859:16667 78.08692:5601 130.06477:33273 131.21602:5188 131.92909:5869 | -3.03E-06 |
| POS6874 | 4,16-dimethylocta | 5.678 | 314.34061 | [M+H]+ | 314.34119 | 57.06989:29811 58.06417:9153 62.05909:18598 62.34779:5756 67.25955:5549 71.08548:13255 95.08478:6751 162.15594:5457 | -1.85E-06 |
| POS5612 | Vaccenic acid | 0.723 | 265.2514 | [M+H-H2O]+ | 265.25201 | 55.05364:6929 57.06895:7314 64.0822:5705 67.05388:8099 95.08475:7276 128.43639:5448 156.52417:4948 173.39107:8108 | -2.3E-06 |
| NEG6662 | Neurocil | 1.052 | 327.15433 | [M-H2O-H]- | 327.15369 | 52.07363:6494 93.03928:872312 95.03767:416150 112.81192:5661 148.88708:6056 151.06717:36099 153.0676:44578 211.09386:6706 | 1.956E-06 |
| NEG6606 | HYDROQUINIDINE | 1.056 | 325.15448 | [M-H]- | 325.15384 | 64.7287:5833 89.13125:11698 93.03925:628532 95.03763:54615 97.03745:20711 145.23549:26894 151.06712:32256 224.36078:6202 | 1.968E-06 |
| POS9945 | LysoPC 16:2 | 5.8 | 492.30777 | [M+Na]+ | 492.30841 | 59.04859:14322 60.08025:17792 86.09541:33976 89.05896:11013 104.10697:195092 124.99956:13507 184.06931:80539 | -1.3E-06 |
| POS9490 | olin-1-yl)-17-nor | 5.121 | 469.29675 | [M+H]+ | 469.29611 | 59.04858:36831 87.04334:65710 89.05896:30551 101.0593:18277 | 1.364E-06 |
| NEG9467 | Sulfluramid | 5.826 | 526.98547 | [M-H]- | 526.9848 | 56.92723:6923 68.16396:5792 79.05373:35127 98.51506:5653 158.53918:7858 168.244:8458 216.10568:5340 | 1.271E-06 |
| NEG4913 | OCTADEC-16-ENA | 0.878 | 265.25446 | [M-H]- | 265.25381 | 77.54872:6374 101.10021:5444 113.12118:131760 134.21043:6877 143.93689:5017 | 2.45E-06 |
| POS13730 | kanoyl-NBD Chole | 8.766 | 663.44861 | [M+NH4]+ | 663.44788 | 52.42753:6679 174.16846:5838 276.60065:6166 | 1.1E-06 |
| POS14754 | PC(32:0) | 5.984 | 756.55035 | [2M+H]+ | 756.55133 | 60.08022:37579 86.09537:90868 124.9995:29152 184.07457:229178 278.94693:9358 | -1.3E-06 |
| POS7459 | nethylpent-4-enyl | 1.21 | 343.33655 | [M+Na]+ | 343.33591 | 55.05453:10758 57.06896:43638 59.03105:230090 61.01003:106223 62.98918:6912 67.05389:14750 69.06869:29415 70.83771:5507 71.08548:18494 73.04555:155497 75.02498:212522 77.00464:11970 77.04093:139452 79.01993:21569 83.0851:22905 85.10046:10091 87.06079:15150 91.05577:139447 93.03564:124450 95.01512:10207 97.09953:20636 105.07177:51413 111.11733:11258 119.08613:18341 120.30954:5504 125.13172:7294 133.10193:9567 252.17599:5722 343.06805:5855 | 1.864E-06 |
| POS13381 | deferrioxamine E | 4.942 | 639.39178 | [M+NH4]+ | 639.39093 | 51.69823:15774 52.24539:16019 57.26204:16845 57.49446:15405 63.10206:14911 64.38737:17272 66.84683:15535 67.72396:16179 69.98:15275 70.89279:14630 72.48169:14052 81.48377:15286 83.18766:14148 85.52522:14213 106.00784:15084 116.15504:19389 119.128:16230 126.05975:15786 127.80418:13953 130.4415:14237 131.46817:16566 137.12877:14484 145.22301:15836 146.56651:14491 148.21899:17379 149.40419:14346 159.72244:14941 172.44861:17416 181.18074:19832 183.76932:19344 193.33499:17767 198.01981:17442 216.79158:16968 219.92371:18493 251.43799:14919 278.60718:15006 294.77921:16229 316.50882:18242 316.65399:16131 321.06827:17036 323.32883:17358 373.3949:20685 379.43304:18899 503.52267:15397 504.54337:16705 548.30841:15379 584.229:16901 | 1.329E-06 |
| POS8548 | Arg His Thr | 4.42 | 413.22482 | [M+H]+ | 413.22549 | 59.04859:17241 70.06431:7509 72.08048:8153 87.04335:7051 89.05896:45647 103.07444:10459 118.06566:25692 132.07892:8927 133.08545:19008 144.0797:18346 146.061:92134 151.09705:6329 159.09138:12523 188.07004:310236 189.07106:20113 205.09822:54513 209.13737:7734 363.82065:6967 | -1.62E-06 |
| POS2769 | Methylglutaconic a | 0.946 | 167.03081 | M+CH3OH+H] | 167.03148 | 51.87749:7152 53.48768:5939 141.06718:5550 | -4.01E-06 |
| POS5062 | Aspergilliamide B | 4.38 | 245.14882 | [M+NH4]+ | 245.14951 | 50.74328:5710 60.53485:5917 68.04947:19806 69.06865:9987 70.06427:6873 84.08119:7743 85.02796:14854 86.05935:80919 86.09537:175775 132.06581:129317 176.54716:5792 | -2.81E-06 |
| POS12762 | -caffeoyloxyfriede | 6.835 | 605.42078 | [M+H]+ | 605.41998 | 59.86679:6420 | 1.321E-06 |
| POS2033 | Ethephon | 8.766 | 144.98216 | [M+H]+ | 144.98151 | 62.92802:382364 103.95428:12783 121.96451:111242 144.97899:15551 | 4.483E-06 |

| Differences in metabolites between the Model group and the Control group | | | | | | | |
|---|---|---|---|---|---|---|---|
| Alignment ID | Metabolite name | Rt(min) | Expreiment Mz | Adduct type | Reference m/z | MS/MS spectrum | PPM |
| POS5240 | roughanic acid | 5.861 | 251.19934 | [M+H]+ | 251.2 | 55.05366:8995 57.06896:10614 59.04859:13384 67.05389:49762 81.06834:33459 93.06841:13426 95.08478:16153 96.71436:5123 107.08487:8283 119.01638:5881 200.50862:5817 | -2.63E-06 |
| POS7143 | earoylethanolami | 7.516 | 328.31934 | [M+H]+ | 328.32001 | 74.28384:5185 83.54238:5423 168.10655:5885 292.4346:6312 | -2.04E-06 |
| POS3651 | Cyclohexylalanine, | 0.996 | 194.11444 | [M+NH4]+ | 194.11513 | 52.16681:6394 58.21173:6940 65.0448:6380 105.28721:6138 135.04227:12472 | -3.55E-06 |
| POS474 | 5Z)-1,3,5-Heptatri | 1.206 | 95.08541 | [M+H-H2O]+ | 95.08607 | 55.05365:31397 65.03804:11693 67.05389:46139 81.05109:6681 94.06358:28792 95.04893:9914 95.08476:12078 | -6.94E-06 |
| POS4088 | Ethirimol | 7.201 | 209.15213 | [M+H]+ | 209.1528 | 62.28967:5904 95.08477:7367 156.20926:5810 | -3.2E-06 |
| POS5715 | Vitamin A | 7.047 | 269.22433 | [M+H]+ | 269.22501 | 67.57101:5925 97.04408:5224 120.90405:6182 173.43034:10681 | -2.53E-06 |
| POS5489 | Leucyl-Glutamate | 2.399 | 261.14291 | [M+H]+ | 261.14359 | 58.06512:6880 61.0387:112801 90.77464:6157 148.89172:5542 167.25319:5439 215.13528:10629 | -2.6E-06 |
| POS1888 | methylthio)methyl | 9.436 | 140.98665 | [M+H]+ | 140.98599 | 54.94678:16057 55.0177:7569 55.05365:10468 55.93345:120086 55.94513:11345 56.9414:107108 57.9342:29721 58.06511:11469 67.05389:25965 67.93391:10623 70.0643:12048 70.94153:9884 72.93706:54805 90.94762:10952 94.06358:21162 95.04694:7073 95.08476:17165 98.98203:6037 111.96739:9636 113.96311:43964 114.97044:33110 116.97124:22432 131.97466:8216 | 4.681E-06 |
| POS7891 | JNII-5BCD1RO66X | 4.87 | 368.23901 | [M+2H]2+ | 368.23831 | 57.03287:32946 58.04042:81847 59.04859:845961 60.05226:21212 65.04819:47242 69.06869:8507 71.04816:9491 73.02812:288399 73.06432:31626 80.0543:109304 80.55504:8430 85.06337:33913 87.04335:206760 87.06079:92957 87.07999:13240 89.05896:1404834 90.06191:46269 94.06947:13907 101.05931:73293 102.06606:60752 103.07444:122821 107.07059:12879 109.07315:40397 111.07206:7955 115.07378:9574 117.05281:7930 124.08022:18934 129.08911:15292 131.06766:20340 133.08545:402344 134.09018:21942 147.10149:42058 177.11285:31739 191.1256:9253 | 1.901E-06 |
| POS10817 | Pro Lys Lys Val Gly | 5.236 | 528.34955 | [M+2H]2+ | 528.35028 | 57.03378:52435 59.04858:942759 60.05225:24259 73.02811:90964 73.06431:42004 80.05428:17471 85.06504:26185 87.04333:285945 87.06252:45317 87.07997:25489 88.04604:21069 89.05894:1175868 90.0619:30232 94.06945:36619 101.05929:186395 102.06604:24382 103.07442:277642 107.07057:16114 109.07558:18349 129.0891:30027 133.08542:384601 134.08682:17608 147.10147:95883 161.11877:16088 171.94873:14308 177.11282:23848 191.12556:20051 219.33647:14791 | -1.38E-06 |
| POS11452 | A 108836 | 4.929 | 551.33923 | [M+H]+ | 551.34003 | 59.04859:39169 63.23145:11485 73.02812:16131 74.31548:11144 80.0543:16151 87.04335:26564 89.05896:154180 104.10697:19400 133.08545:72250 173.38622:14575 495.42001:11316 | -1.45E-06 |
| POS7519 | reonylalanylargini | 4.648 | 347.203 | [M+Na]+ | 347.2037 | 60.6027:5934 80.86337:6667 89.05896:7305 92.63606:5017 173.39111:15290 | -2.02E-06 |
| POS10834 | methylhexadecahy | 8.806 | 529.32574 | [M+NH4]2+ | 529.32501 | 60.08025:7840 63.63507:6441 86.09541:16018 104.10697:162165 105.11111:9431 132.62503:6264 | 1.379E-06 |
| POS10169 | Ala Leu Leu Ala Se | 5.369 | 502.32269 | [M+2H]2+ | 502.32339 | 57.03287:20505 59.04859:225785 60.05226:8017 67.015:26429 73.02812:7445 73.06432:10946 78.27695:5333 81.03072:15251 85.06506:7810 87.04335:27628 87.07999:12120 89.05896:175188 90.06374:7204 91.40009:5270 101.05931:14949 103.07444:66788 117.05281:5730 117.0909:6618 131.06766:5493 133.08545:48132 147.10149:28884 158.09198:14257 165.0956:8004 177.11285:8119 180.10141:7485 | -1.39E-06 |
| POS11695 | Stellettacholine A | 4.67 | 560.33539 | [M+2H]2+ | 560.33459 | 69.03294:15177 73.02811:35386 73.06432:11459 80.05429:14448 87.04334:85223 88.04604:6778 89.05896:404118 90.0619:15946 91.04643:14813 95.04893:11870 102.06606:13250 111.04189:9243 113.05927:20331 131.06766:11306 133.08543:167499 134.09018:9578 137.0598:8958 155.06668:6020 177.11285:24578 190.08008:7159 223.32474:6305 | 1.428E-06 |
| POS14395 | ulomo'opunalide- | 5.697 | 717.47858 | [2M+H]2+ | 717.47961 | 57.03287:19891 59.04859:221207 73.02812:35528 73.06432:17061 85.06337:12306 87.04335:77540 87.06079:20207 89.05896:270835 94.06947:13494 101.05931:63349 103.07444:78962 129.08911:11617 131.10634:12235 133.08545:89934 147.10149:47843 396.90311:11015 669.2359:11689 | -1.44E-06 |
| NEG4967 | )-6-octadecen-5- | 1.74 | 267.26871 | [M-H]- | 267.26941 | 97.07645:17103 99.07355:76917 122.14449:23477 126.07927:8655 135.1936:9014 165.20354:11966 183.59097:6420 | -2.62E-06 |

**Differences in metabolites between the Model group and the Control group**

| Alignment ID | Metabolite name | Rt(min) | Expreiment Mz | Adduct type | Reference m/z | MS/MS spectrum | PPM |
|---|---|---|---|---|---|---|---|
| POS13938 | (28),23-dien-3-oi | 8.648 | 679.47693 | [M+2H]2+ | 679.47791 | 155.16624:5615 316.72656:5510 389.63431:6421 | -1.44E-06 |
| POS13533 | -en-28-ol\|3??-tra | 6.826 | 649.44531 | [M+H]+ | 649.44623 | 55.45766:7692 66.63255:7260 66.88206:5923 91.21459:5691 110.99668:6065 130.05522:6720 291.0647:5703 517.97321:7532 | -1.42E-06 |
| POS6778 | thro-Sphingosine | 7.672 | 310.30829 | [M+H]+ | 310.30899 | 50.68978:8626 276.54135:5621 | -2.26E-06 |
| POS12710 | ne-3alpha,29-dio | 6.299 | 603.40521 | [M+Na]+ | 603.4043 | 295.258:5816 | 1.508E-06 |
| POS12993 | lene-4-(2-methyl | 4.941 | 617.40582 | [M+H]+ | 617.40491 | 57.03287:111732 59.04859:2946029 73.02812:137628 73.06432:76402 85.06337:118003 87.04335:783200 87.07999:105717 89.05896:4837258 90.06191:77426 101.05931:594982 103.03849:81983 103.07444:1201891 115.07378:47468 117.05281:77075 117.0909:61153 129.08911:124756 131.06766:120960 131.10634:77159 133.08545:1871124 134.09018:72977 145.08403:98662 147.10149:957866 161.11441:76372 177.11285:189428 191.1256:146797 304.17166:44505 | 1.474E-06 |
| POS8016 | Leucocrystal Violet | 5.293 | 374.25827 | [M+2H]2+ | 374.259 | 57.03287:93167 59.04859:2046332 60.05226:51596 65.04819:14339 69.06992:11142 73.02812:116829 73.06432:24672 81.0699:9899 85.06337:32496 87.04335:91908 87.06254:28085 87.07999:22968 89.05896:688187 90.06191:16894 94.06947:42386 99.07939:11622 101.05931:108683 103.07444:305256 104.07732:10915 115.07378:16776 116.08248:17429 117.05281:13299 117.0909:22477 129.08911:17999 131.07088:14247 133.08545:155401 143.1078:10089 147.10149:123765 161.11441:10648 191.1256:11440 | -1.95E-06 |
| POS8668 | Erythrophloin A | 4.606 | 421.23813 | [M+Na]+ | 421.2374 | 89.05896:20748 118.57424:5939 139.98682:6236 173.43526:6276 | 1.733E-06 |
| POS8800 | Val Val Val Gly Gly | 4.948 | 430.26517 | M+CH3OH+H]2 | 430.2659 | 59.04858:42603 67.015:7871 82.13532:5643 87.04334:12522 87.1743:6597 89.05896:85351 103.07444:14768 122.2514:5931 133.08545:26483 221.2818:6249 | -1.7E-06 |
| POS10171 | LysoPC(P-16:0/0:0 | 6.253 | 502.32748 | [M+H-H2O]+ | 502.32672 | 62.06014:11393 68.02178:6959 86.09712:10834 318.39163:5746 | 1.513E-06 |
| POS13012 | Veralosine | 5.804 | 618.40094 | [M+2H]2+ | 618.40002 | 57.03287:16195 59.04859:82528 60.16235:11541 70.74816:10102 73.02812:15396 73.06432:12980 87.04335:28027 89.05896:96925 101.05931:29693 103.07445:22539 129.08911:12432 133.08545:33699 135.46794:9780 147.10149:14249 173.38132:13274 196.63869:10859 | 1.488E-06 |
| POS5109 | yl)methyl]-N-eth | 8.898 | 247.09886 | [2M+H]+ | 247.09959 | 57.06986:10568 69.06989:13126 80.83523:6072 111.04185:26675 125.05955:20055 167.10457:27673 168.4581:6972 229.63365:6215 239.8895:5286 | -2.95E-06 |
| POS4339 | nma-Glutamylalar | 1.483 | 219.09666 | [M+H]+ | 219.0974 | 56.0495:7304 83.04604:5690 84.04313:118986 84.07954:10805 85.02796:8517 90.05453:44206 128.0714:7891 130.0488:15023 156.06255:19326 170.79237:5928 | -3.38E-06 |
| NEG1779 | Pelargonic acid | 1.51 | 157.20074 | [M-H]- | 157.2 | 69.08023:6205 79.71713:5218 89.13125:312981 90.1367:6657 97.07645:7737 113.19607:6950 115.14149:7846 141.94867:5416 144.71094:5617 147.59695:5893 | 4.707E-06 |
| POS13290 | n-3alpha-yl-O-be | 4.616 | 633.3642 | [M+2H]2+ | 633.36322 | 67.05389:62726 69.03294:62783 73.02811:263251 73.06432:93764 81.03229:85335 83.0477:79886 87.04334:608498 89.05896:3244339 90.0619:123708 95.04893:174189 99.04339:100212 111.04189:266453 113.05927:113170 113.1652:62702 133.08543:1007164 137.0598:88154 | 1.547E-06 |
| POS8382 | Cryptomaldamide | 5.19 | 400.25473 | [M+2H]2+ | 400.25549 | 59.04858:46073 67.015:6651 87.04334:8280 89.05896:46653 101.0593:5604 103.07444:10843 127.95014:5198 133.08545:11379 262.60263:6014 307.50278:7369 335.51199:6585 | -1.9E-06 |
| POS8644 | Roxithromycin | 4.963 | 419.27377 | [2M+H]2+ | 419.27301 | 57.03287:36771 58.04042:44168 59.04859:1046584 60.05226:24137 65.04819:44839 67.05389:10654 69.06869:13857 73.02812:213131 73.06432:37940 80.0543:98672 85.06337:40000 87.04335:222274 87.06079:104870 87.07999:21985 89.05896:1663653 90.06191:45792 94.06947:14388 101.05931:74030 102.06606:50977 103.07444:168670 104.0796:10383 107.06821:13701 109.07315:62752 116.0798:9014 117.0909:12747 124.07726:21506 129.08911:25685 131.06766:24749 133.08545:543876 134.08684:29226 138.09604:10695 140.09358:12090 147.10149:69791 153.09918:12457 177.11285:48734 191.1256:13042 | 1.813E-06 |

| Differences in metabolites between the Model group and the Control group | | | | | | | |
|---|---|---|---|---|---|---|---|
| Alignment ID | Metabolite name | Rt(min) | Expreiment Mz | Adduct type | Reference m/z | MS/MS spectrum | PPM |
| POS11613 | CHEBI:69627 | 5.196 | 557.38458 | [M+H]+ | 557.38373 | 57.03287:68192 59.04859:1426531 60.05226:22336 69.06868:16224 73.02812:30907 73.06432:66693 85.06506:16565 87.04335:101496 87.07999:14345 89.05896:331344 101.05931:133579 103.07444:374703 115.07378:27471 117.05281:73537 117.0909:59987 131.07088:55951 133.08545:42112 147.10149:77117 161.11441:46865 | 1.525E-06 |
| POS8199 | Alstovine | 5.725 | 387.19226 | M+CH3OH+H] | 387.1915 | 51.54529:6444 51.69024:6817 55.05365:6559 57.06896:36001 59.04858:6559 71.04816:33403 74.83275:5981 83.0477:43632 101.0593:21270 173.43034:13159 175.01567:53935 211.91226:5915 214.52664:6403 | 1.963E-06 |
| POS12628 | Coriandrinol | 5.713 | 599.42914 | [M+H]+ | 599.42822 | 57.03286:45848 59.04858:470607 67.46854:7742 69.06991:8637 70.37264:7815 70.79802:7299 73.02811:19200 87.04334:21683 89.05895:85190 91.19027:8508 99.07938:9185 101.0593:21007 103.07443:21528 115.07377:11938 117.09089:73108 133.08543:18864 147.10147:8020 155.0584:7607 173.3911:16437 200.63062:7164 480.31015:8204 563.1051:7694 | 1.535E-06 |
| POS13032 | 7',8'-Tetradehydro | 6.297 | 619.37714 | [M+H]+ | 619.37811 | 51.86788:5622 530.44775:5263 | -1.57E-06 |
| POS13958 | cheilocline F | 5.492 | 681.45233 | [M+2H]2+ | 681.45129 | 59.04859:306302 73.02812:48139 87.04335:65890 89.05896:393891 101.05931:62129 103.07445:79381 129.08911:27142 133.08545:134914 147.10149:48421 147.89014:27807 173.43526:39266 | 1.526E-06 |
| POS5228 | Deoxyadenosine | 4.68 | 251.10112 | [M+NH4]+ | 251.10181 | 59.04858:52639 60.04426:70014 69.06992:10529 70.02779:77145 74.02328:15442 87.04334:14689 88.03896:207029 94.06359:180511 95.06884:6101 98.02254:9480 99.00742:32831 104.04768:16240 107.43317:5637 116.03146:11602 117.05281:8812 118.06566:5322 120.04358:56815 130.04884:28260 132.04306:17934 136.07611:13891 146.05721:230585 147.06317:13812 150.05342:17259 158.04503:14454 163.19865:6458 173.38622:10070 174.05498:45442 177.6405:5237 192.06537:12410 | -2.75E-06 |
| POS10530 | Thr Thr Gly Leu Ile | 6.514 | 518.31921 | [M+Na]+ | 518.31842 | 59.04858:12918 60.10926:5981 62.53428:5966 76.55672:6150 80.40308:5481 86.09541:25027 146.97894:35421 331.8078:5767 361.46249:5952 | 1.524E-06 |
| POS4891 | 3,HCl-(??)-N5-Hyd | 4.856 | 239.13814 | [2M+H]+ | 239.1389 | 68.04949:10488 80.04967:18785 84.07957:100441 89.05895:10258 130.08707:9249 134.09683:383236 135.09956:16940 197.1311:14065 | -3.18E-06 |
| POS7961 | Val-Arg-Pro | 5.612 | 371.23929 | [M+H]+ | 371.24011 | 52.73953:5807 59.03397:5750 193.61818:5194 329.18835:6615 371.23489:8925 | -2.21E-06 |
| POS7819 | 1-epoxy-9-fluoro | 4.505 | 363.1973 | [M+Na]+ | 363.1965 | 173.39111:8444 281.01971:6255 289.36584:6283 363.1955:12277 | 2.203E-06 |
| POS9516 | pirost-9(11)-en-1 | 4.84 | 471.31198 | [M+H]+ | 471.31119 | 57.03287:12458 59.04859:331752 73.02812:9897 83.38518:5882 87.04335:30374 89.05896:269482 90.06375:6392 95.44421:5320 101.05931:24970 103.03849:9236 103.07444:66995 133.08545:42039 147.10149:21312 388.28345:5701 | 1.676E-06 |
| POS7551 | Arg Ser Ser | 4.509 | 349.1821 | [M+Na]+ | 349.18289 | 62.11793:5892 67.20391:5960 70.06431:10778 86.05939:8665 165.3967:6089 166.37866:6413 173.43036:6489 | -2.26E-06 |
| POS11797 | -yl)-1H-cyclopent | 5.556 | 563.37207 | [M+Na]+ | 563.37299 | 57.03287:9100 59.04858:24525 66.65124:6273 72.53472:7669 87.04334:11665 89.05896:33263 103.07444:7267 104.10696:14628 104.63825:5839 133.08543:8499 141.77881:5799 165.37384:6538 184.06929:7804 344.60162:5601 350.31827:6336 | -1.63E-06 |
| POS14256 | ta-D-glucopyrand | 4.955 | 705.45593 | [M+H]+ | 705.45709 | 59.04858:88066 73.02811:7844 87.04334:40200 89.05895:236352 101.0593:25592 103.07443:34789 133.08543:94486 147.10147:21517 156.65466:6609 177.11284:12295 184.07465:10663 399.92606:7569 | -1.64E-06 |
| POS12084 | Sevcorine | 5.201 | 574.41101 | [M+H]+ | 574.4101 | 59.04859:415155 73.02812:15506 73.06432:81514 87.04335:36123 89.05896:199439 101.05931:36242 103.07445:124824 117.05281:154756 117.0909:25395 131.06766:75721 133.08545:53150 147.10149:37025 | 1.584E-06 |
| POS7995 | GS-2 | 5.299 | 373.17462 | [M+H-H2O]+ | 373.17542 | 55.01768:9674 57.06894:18595 59.04856:85272 71.1009:6022 73.0281:6516 73.14081:6523 83.04929:11086 85.06334:8841 89.05893:28226 95.08474:23769 101.05709:5455 103.07441:10789 122.09476:11174 130.06473:24991 131.57179:5379 132.04303:6133 146.06094:21617 150.0889:7699 158.05779:26437 160.07413:15754 168.10181:6410 168.88625:6034 175.08525:11309 182.11658:5953 184.0746:22478 186.05348:5870 199.00485:5375 | -2.14E-06 |
| NEG4249 | ethachlor CGA369 | 1.208 | 242.05011 | [M-H]- | 242.0493 | 59.53994:5499 62.06197:10364 71.2459:6079 79.05367:23326 83.19241:5742 97.08672:5531 161.03622:21782 163.03287:17835 | 3.346E-06 |
| POS6274 | )-3-(4-hydroxyph | 6.146 | 290.15469 | [M+H]2+ | 290.1539 | 55.57048:6563 57.03287:35246 110.31171:5599 175.7039:5876 | 2.723E-06 |

| Differences in metabolites between the Model group and the Control group | | | | | | | |
|---|---|---|---|---|---|---|---|
| Alignment ID | Metabolite name | Rt(min) | Expreiment Mz | Adduct type | Reference m/z | MS/MS spectrum | PPM |
| NEG1775 | de, cyclopentyl iso | 1.259 | 157.1049 | [M-H2O-H]- | 157.1057 | 59.09475:28573 71.10927:39130 72.09502:6311 79.05367:106869 97.07645:129535 97.11958:205100 99.07355:13615 114.16649:92565 136.50891:5947 159.91479:5870 | -5.09E-06 |
| POS5650 | eta-D-glucopyran | 4.483 | 267.14297 | [M+H-H2O]+ | 267.1438 | 59.04859:209303 71.04816:7917 73.02812:13696 73.06432:9664 77.05981:7144 87.04335:97005 88.21841:5677 89.05896:187607 101.05931:15789 103.03849:332742 103.07444:17763 104.04312:7029 110.05836:6470 117.05281:11657 119.06938:7902 133.08545:20337 165.7536:5287 173.42545:10359 266.04507:5893 | -3.11E-06 |
| POS6244 | Arginylasparagine | 4.53 | 289.16101 | [M+Na]+ | 289.1618 | 51.45718:6340 70.02779:12580 86.09541:38435 88.03896:7095 156.97519:5914 163.43175:5297 173.39111:5979 232.69829:5819 289.1651:48759 | -2.73E-06 |
| POS2582 | C)OC(C(CC(C)O)C) | 5.496 | 161.11629 | M+CH3OH+H] | 161.1171 | 67.86302:6189 123.64797:5879 | -5.03E-06 |
| POS938 | alpha-monochlo | 5.925 | 111.01991 | [2M+H]+ | 111.0207 | 55.04137:9377 55.93435:9040 57.9342:170588 72.93707:68652 74.93713:31886 76.49633:11064 77.99808:37110 78.99881:31105 84.94386:26990 85.50144:9992 87.00324:147205 87.50562:7534 88.00348:752935 88.50382:34626 89.00123:11667 89.50648:7362 90.94763:19535 92.94897:8194 95.01114:7544 97.00918:268649 97.50975:14659 98.50998:23719 98.96088:19921 99.51076:148819 100.01365:13214 102.95543:19751 106.01486:9065 108.51749:20082 108.95825:15304 111.01928:11217 113.96312:112127 | -7.12E-06 |
| POS935 | alpha-monochlo | 5.758 | 111.0199 | [M+H]+ | 111.0207 | 55.04137:14360 55.93435:18317 57.9342:188736 68.04829:7364 72.93706:89086 74.93713:27482 75.49611:10279 76.49632:25918 77.99808:43985 78.99881:41352 84.94554:25301 85.49974:8835 87.00324:184402 87.50385:18900 88.00348:916425 88.50381:49343 89.00303:12289 89.50647:9252 90.50591:13615 90.94762:27374 92.94704:7548 95.01113:10101 96.00741:8333 97.00917:302326 97.50974:6819 97.95171:9592 98.51208:24318 98.96088:40993 99.51075:168294 100.0115:10693 102.95542:20264 106.01252:9103 108.51748:16989 108.95825:17323 111.01928:11515 113.96311:119832 | -7.21E-06 |
| POS4237 | Citric acid | 1.01 | 215.01515 | [M+H]+ | 215.01601 | 70.0643:6076 96.92099:12553 98.9186:10567 110.9314:6271 131.91931:6299 134.01683:6754 | -4E-06 |
| POS10447 | Hennoxazole A | 8.804 | 515.31238 | [M+NH4]2+ | 515.31152 | 57.06894:18004 60.08023:21856 75.85378:9829 84.26364:10607 86.09538:66938 104.10693:599104 105.10875:29289 112.04374:14156 173.38615:21450 183.12358:10484 312.70972:11045 | 1.669E-06 |
| POS13649 | -alpha-D-acetylri | 5.318 | 659.45282 | [M+H]+ | 659.45172 | 57.03379:66876 59.04956:789822 73.06432:41776 83.54895:27459 87.04335:72894 89.05896:328663 101.05931:96236 103.07444:238795 115.07378:26832 117.05553:55923 131.07088:29420 133.08545:57155 147.10149:64623 161.11441:26692 190.06322:24163 | 1.668E-06 |
| NEG3630 | H-Met-Ala-OH | 1.325 | 219.08076 | [M-H]- | 219.0816 | 58.04516:8286 97.07652:18103 99.07362:24309 118.92107:6107 161.03635:88578 | -3.83E-06 |
| POS12888 | dyl pseudopteran | 5.672 | 611.39319 | [M+2H]2+ | 611.39423 | 57.03286:18838 59.04858:107939 73.02811:18743 85.06504:14420 87.04333:15714 89.05894:100135 101.05929:24600 103.07442:41330 133.08542:26485 147.10147:14782 160.2352:10512 | -1.7E-06 |
| NEG4833 | (-)-Epipolasin A | 0.488 | 262.16446 | [M-H]- | 262.1636 | 84.25964:5170 100.0459:11700 153.12859:5074 | 3.28E-06 |
| POS12675 | olestane-3beta,6a | 5.323 | 601.39343 | [M+2H]2+ | 601.39447 | 57.03287:54435 59.04859:859064 60.05226:35649 73.02812:102762 73.06432:50234 85.06337:38465 87.04335:181364 87.06254:44298 87.07999:44771 89.05896:1238278 90.06374:36665 101.05931:153091 102.06606:21323 103.07444:270837 109.0756:24811 115.07378:19880 117.0909:34380 129.08911:28073 131.06766:23646 133.08545:446052 134.09018:29466 147.10149:143483 177.11285:44140 191.1256:28717 307.53751:16936 | -1.73E-06 |
| POS11521 | His Ile Lys Arg | 5.463 | 553.35583 | [M+2H]2+ | 553.35681 | 59.04859:215099 61.68283:11550 61.80683:13336 67.015:20031 73.02812:24862 73.06432:19312 87.04335:31828 87.07999:13960 89.05896:199596 101.05931:40023 103.07444:60091 104.10697:50848 133.08545:65203 147.10149:36903 158.09198:12769 184.07466:16179 314.30731:14461 | -1.77E-06 |
| NEG2746 | Diethyl L-malate | 1.319 | 189.08087 | [M-H]- | 189.08 | 73.0612:12532 109.03119:7235 125.04041:6868 145.13034:14383 189.08273:7862 | 4.601E-06 |
| POS4736 | SCHEMBL4748090 | 5.798 | 233.17374 | [2M+H]+ | 233.17461 | 55.24187:5609 57.03287:17170 59.04858:21868 | -3.73E-06 |
| POS11284 | anine-3beta,17,23 | 6.111 | 546.34155 | [M+Na]+ | 546.34253 | 86.0954:20507 93.39707:5656 97.71894:5703 104.10696:40334 105.10878:7465 184.06927:20814 273.52429:5481 315.59171:5348 340.05838:6352 | -1.79E-06 |

| Differences in metabolites between the Model group and the Control group | | | | | | | |
|---|---|---|---|---|---|---|---|
| Alignment ID | Metabolite name | Rt(min) | Expreiment Mz | Adduct type | Reference m/z | MS/MS spectrum | PPM |
| POS8085 | DS-007819 | 4.879 | 379.23132 | [M+2H]2+ | 379.22781 | 59.04859:30062 67.015:11734 67.38055:5666 87.04335:11350 89.05896:48424 101.05931:13232 133.08545:14619 137.01158:5262 139.32047:5241 160.3878:5683 | 9.256E-06 |
| POS14377 | anosyl)-2,6-dihydr | 6.592 | 715.53668 | [M+NH4]+ | 715.5354 | 57.03287:232785 57.96547:6274 58.03662:7976 59.04858:58816 99.07938:17533 115.07378:17169 117.09089:8136 157.12312:17195 363.88025:5820 431.01767:5684 715.4892:6396 | 1.789E-06 |
| POS12054 | a-Hydroxymilbem | 4.867 | 573.33826 | [2M+H]+ | 573.33929 | 57.03287:19053 59.04859:212199 73.02812:12020 73.06432:7762 87.04335:295959 87.07999:9202 89.05896:269303 101.05931:92747 103.03849:134737 103.07444:61656 104.3103:7845 115.07378:9094 117.05281:35589 131.06766:13682 131.87703:8857 133.08545:86616 147.10149:33234 173.43036:10327 177.1078:10853 | -1.8E-06 |
| NEG9811 | allocatechin 3,4'-d | 6.248 | 609.0907 | [M-H]- | 609.0896 | 89.1313:907206 90.13492:34712 156.12057:6322 409.70682:8289 591.94617:5607 | 1.806E-06 |
| POS3342 | phohydroxypyruvi | 8.789 | 184.9854 | [M+NH4]+ | 184.9845 | 55.8742:7477 57.9342:157519 58.9425:411639 59.94047:8544 75.94469:93117 76.95241:32865 84.94554:57664 86.0954:11358 93.95591:18053 98.96088:211388 99.96855:133494 100.96555:15784 102.95542:60805 114.95721:18958 115.96169:43297 116.97124:1264344 117.97753:1428314 120.96403:7764 125.97163:17274 126.96738:39663 127.975:18234 130.98712:9043 133.97353:7244 135.99091:9156 139.9868:162464 140.9953:104599 143.95718:16779 161.96983:30649 | 4.865E-06 |
| POS7398 | tadecenyl)-1,3-be | 6.602 | 341.246 | [M+H]+ | 341.24512 | 82.54128:5672 175.02563:6388 305.6582:5038 | 2.579E-06 |
| POS2880 | ramethyl-4-piperi | 0.682 | 171.14819 | [M+H]+ | 171.14909 | 55.05365:61066 57.03287:11399 67.05389:44024 69.06992:11642 70.06431:6126 71.04816:6819 71.92826:6813 72.04369:57363 72.08047:5949 79.05463:8663 81.0699:12644 83.08509:18180 89.06979:19943 93.06841:5858 109.06336:5644 109.10007:18048 123.59779:6135 166.5724:5543 | -5.26E-06 |
| POS7334 | Istamycin AO | 4.561 | 338.19119 | [M+NH4]2+ | 338.19211 | 55.05365:49468 58.04137:31543 59.04858:61426 61.02845:24162 67.05389:26368 69.03294:776238 70.03661:25734 71.04816:22373 73.02811:184930 73.06432:46091 78.03804:83888 80.05429:48495 81.03229:32872 81.0699:20190 83.0477:151707 85.02799:22720 87.04334:640153 88.04604:21083 89.05896:1486336 90.0619:42664 95.04893:39479 97.06461:14050 99.04339:321315 100.05018:94695 101.0593:10558 102.06606:15836 103.03849:8502 107.07059:20344 109.06336:23678 111.04189:174394 112.04632:10697 113.05927:31595 117.09089:10199 122.06293:140023 122.56553:18337 125.0596:42861 129.05446:59089 131.07088:37711 133.08543:289594 134.09018:15252 137.05981:20468 144.07597:41692 151.09305:14173 155.07083:64386 166.08865:9512 173.07771:20122 175.09526:10805 177.11285:16091 | -2.72E-06 |
| POS9493 | Simplexin B | 5.384 | 469.31497 | [M+2H]2+ | 469.31589 | 57.03287:117896 59.04859:2300633 59.05931:59214 60.05226:60339 65.04931:24666 69.06868:28764 73.02812:140915 73.06432:49915 83.0851:15798 85.06337:48771 87.04335:271250 87.06254:50152 87.07999:61435 89.05896:1297080 90.06191:46940 94.06947:73494 99.07939:11795 101.05931:263789 101.09641:11767 103.03849:20538 103.07444:482551 104.07732:28994 105.09028:13361 107.06821:11344 109.07315:13916 115.07378:41262 116.08248:19653 117.05281:23109 117.0909:50237 127.07501:11211 129.08911:37463 130.08708:12384 131.07088:29078 131.10634:18719 133.08545:433862 134.09018:23569 145.08403:24398 147.10149:244411 148.10667:9928 161.1188:28471 177.11285:37578 191.1256:32772 205.14238:12414 | -1.96E-06 |
| POS11697 | Arg Thr Lys Arg | 4.996 | 560.36365 | [M+H]+ | 560.36261 | 59.04856:97321 73.02808:7807 73.06429:9247 76.54948:6580 87.0433:167181 89.05891:92453 101.05926:51732 103.03844:19276 103.07439:28034 117.05275:17101 131.0676:11931 133.08537:28163 145.85263:6712 177.02168:6394 | 1.856E-06 |
| POS9687 | PC-M5' | 4.896 | 480.27539 | [M+H]+ | 480.27448 | 53.64094:6458 65.62093:6215 126.02025:18856 150.00212:6125 | 1.895E-06 |

| Differences in metabolites between the Model group and the Control group | | | | | | | |
|---|---|---|---|---|---|---|---|
| Alignment ID | Metabolite name | Rt(min) | Expreiment Mz | Adduct type | Reference m/z | MS/MS spectrum | PPM |
| POS6422 | Pizotifen | 5.074 | 296.1456 | [M+2H]2+ | 296.14651 | 55.05364:16814 57.06895:174141 58.06415:8749 59.04857:8589 59.07294:6187 60.08023:13534 67.05388:33682 67.2394:5981 68.82522:5669 69.0699:22609 71.08546:90873 75.26418:6891 78.54684:5994 81.06831:35008 83.08508:24588 85.10043:38935 86.09538:38307 95.08475:60267 96.01749:5656 97.09951:13160 104.10694:37141 109.10004:25526 114.91485:6385 156.51996:8986 245.69836:5553 256.00311:7771 290.06473:6318 | -3.07E-06 |
| POS6860 | anthraniloyl-beta- | 1.621 | 314.12149 | [M+H]+ | 314.12241 | 51.39856:6408 54.99667:6656 57.06988:8968 91.59369:6293 121.06409:133554 | -2.93E-06 |
| POS174 | Dichloromethane | 0.763 | 84.95968 | [M+H]+ | 84.9606 | 56.04863:22764 56.96448:23144 59.79324:6337 84.04316:16629 | -1.08E-05 |
| POS175 | Dichloromethane | 4.25 | 84.95968 | [M+H-H2O]+ | 84.9606 | 54.61502:5320 56.04863:26433 56.091:6003 79.57497:5269 84.04482:11852 | -1.08E-05 |
| POS5611 | Vaccenic acid | 7.137 | 265.2511 | [M+H]+ | 265.25201 | 61.67659:5453 84.07957:6347 111.40971:6016 114.95457:6407 173.39601:5858 201.17491:5557 | -3.43E-06 |
| POS7377 | Bursin | 4.589 | 340.20816 | [M+H]2+ | 340.20911 | 58.04137:707509 69.03294:21970 71.04816:62924 73.02811:1839392 73.06432:132891 74.03149:43183 80.05429:962667 80.55504:76281 87.04334:361428 89.05896:3789226 90.0619:124674 102.06606:490082 102.56839:53624 107.07058:26618 111.07206:64520 115.07378:31855 117.09089:22842 124.08022:176716 131.07088:41564 133.08543:1136328 134.09018:34958 146.09511:56660 155.09987:31976 177.11285:87227 | -2.79E-06 |
| POS4947 | -(4-acetylamino-b | 4.534 | 241.20316 | [M+2H]2+ | 241.20219 | 55.05365:7736 57.06896:68681 58.06416:2763218 59.06808:89643 67.05389:63876 69.06868:17389 79.05312:24329 81.06833:379873 82.07301:25212 84.07957:123835 85.06337:14082 95.08477:49800 121.09843:11305 123.11523:306716 124.11882:29187 138.54323:7262 140.14345:69996 | 4.022E-06 |
| POS6840 | Thymol glucoside | 5.258 | 313.16284 | M+CH3OH+H] | 313.16379 | 55.01768:11829 55.05363:64392 57.06894:284785 69.06865:11020 71.01213:23866 81.0683:8650 83.01192:82153 83.04766:774122 84.0514:19074 85.06333:140535 97.06457:7544 99.00737:9122 111.08207:8130 115.03662:36950 127.07495:50870 137.05975:33459 139.07352:23369 155.07077:65777 157.08499:18404 159.09993:94667 171.09958:17877 179.06763:18703 197.07755:10811 | -3.03E-06 |
| POS6395 | purin-6-yl)carbam | 5.751 | 295.11584 | [2M+H]+ | 295.1149 | 55.01682:6428 57.06895:13921 59.04858:10698 67.05389:10906 73.02811:6042 78.6007:4934 81.06989:8065 83.08508:6707 85.02798:8604 93.07033:6373 95.08277:8775 97.09952:5395 104.02032:5922 105.06944:5813 133.06235:21022 163.47661:5188 179.13976:17382 | 3.185E-06 |
| POS3043 | uanidinosuccinic a | 1.091 | 176.06526 | [M+H]+ | 176.06621 | 60.05523:190390 70.06429:845409 71.04814:19765 71.06744:54596 72.08046:24009 84.08121:8979 112.08705:8669 113.06956:61703 114.05197:10024 116.06902:51195 117.07182:6868 130.0966:17345 159.0741:9593 175.12009:8320 | -5.4E-06 |
| POS14929 | Oligomycin C | 8.719 | 775.53864 | [M+NH4]+ | 775.5354 | 78.02028:7026 78.65312:6033 79.51556:6056 82.88864:7619 100.9307:4764 111.60696:5977 130.14764:5777 221.2182:5478 306.94766:6098 353.1517:5441 752.54596:6685 | 4.178E-06 |
| NEG9405 | 6,7,7,8,8-hexadeca | 5.174 | 514.90167 | [M-H]- | 514.9007 | 80.47415:6165 84.43423:5234 106.70364:5642 143.62971:5224 | 1.884E-06 |
| POS4498 | SCHEMBL2268896 | 2.598 | 225.05943 | [M+H]+ | 225.06039 | 58.06512:12460 74.39811:5599 79.05313:11946 91.0539:9945 93.06841:25297 115.93221:5744 121.06696:6799 123.70115:5058 163.1002:5060 227.04024:10378 | -4.27E-06 |
| POS7795 | serylvalylarginine | 4.769 | 361.21835 | [M+Na]+ | 361.2193 | 58.04136:9454 59.04858:48441 73.02811:30879 80.05428:9866 87.04333:12174 89.05894:100509 90.0619:7012 133.08542:19612 253.44498:4951 318.4404:5197 | -2.63E-06 |
| POS6986 | Allopregnanolone | 9.536 | 319.26096 | [M+H]+ | 319.26001 | 51.78613:7408 67.05386:6215 81.0683:6781 93.06837:17456 95.08472:8177 107.08482:7951 119.08607:6210 121.10124:10538 212.5829:6045 316.8718:5836 | 2.976E-06 |
| POS8281 | DTXSID40693955 | 5.056 | 393.24561 | M+CH3OH+H]2 | 393.2446 | 57.03286:8746 59.04858:35580 70.68684:5779 76.29126:5243 87.04333:9185 87.39496:6144 89.05895:49405 101.0593:13576 103.07443:7488 133.08543:18297 138.69392:5703 155.00864:7420 | 2.568E-06 |
| POS12429 | 386093-01_C34H | 6.131 | 590.32086 | [M+Na]+ | 590.32202 | 59.04859:6348 59.73867:5925 67.05389:17007 73.79539:5505 79.05313:13892 81.0699:9701 86.09541:86286 91.0539:12814 93.06841:12853 104.10697:20830 107.08487:7944 119.08334:7685 120.9869:5889 123.31204:5899 146.97896:104460 460.44394:5660 | -1.97E-06 |
| POS12351 | /al-Glu-Ile-Pro-Gl | 8.782 | 586.30707 | [2M+H]+ | 586.30823 | 86.09541:42111 104.10696:279897 105.1111:10117 146.97894:40326 173.39111:14345 | -1.98E-06 |

| Differences in metabolites between the Model group and the Control group | | | | | | | |
|---|---|---|---|---|---|---|---|
| Alignment ID | Metabolite name | Rt(min) | Expreiment Mz | Adduct type | Reference m/z | MS/MS spectrum | PPM |
| POS8787 | Palau'imide | 5.276 | 429.27338 | [M+2H]2+ | 429.27429 | 57.03286:8078 59.04858:169278 60.05225:6477 67.01499:14280 69.03294:6572 73.02811:5794 81.03072:10236 85.06505:5593 87.04333:13633 89.05895:97862 91.05389:12442 101.0593:10057 103.07443:36596 133.08543:32376 147.10147:13437 387.87292:7334 | -2.12E-06 |
| POS7807 | nethoxy-8-(methy | 5.857 | 362.03809 | M+CH3OH+H] | 362.03711 | 51.95142:6460 55.0177:5768 67.46735:6006 82.06343:12142 91.0539:100200 101.25815:5273 119.04706:16269 121.02692:16325 264.27393:7943 | 2.707E-06 |
| POS7299 | Pipericine | 7.722 | 336.32452 | [M+NH4]+ | 336.3255 | 55.48339:6753 55.876:6022 64.58645:6061 66.81867:5411 157.44508:5415 173.39111:13440 335.26138:5837 | -2.91E-06 |
| POS10865 | Cholylvaline | 5.123 | 530.34625 | [M+H]+ | 530.34521 | 57.03287:11319 59.04859:222796 69.06992:5704 73.02812:23499 73.06432:14832 85.06506:9730 87.04335:222920 87.06254:12243 87.07999:17550 89.05896:359254 90.06375:11755 101.05931:83294 103.0385:13641 103.07445:70159 109.0756:6347 117.05281:16226 117.08818:9561 129.08911:11479 131.07088:19808 131.10634:6099 133.08545:146562 134.08685:6598 145.08403:8277 147.10149:34862 173.43036:11283 177.11287:21267 191.1256:7692 282.47211:5720 529.29529:6452 | 1.961E-06 |
| POS9219 | )-Eicoseneoylcarn | 5.772 | 454.38867 | [M+H]+ | 454.38965 | 57.03286:16430 57.06895:6063 57.78485:5819 59.04857:12049 60.08024:26214 61.65057:5446 85.02798:138083 90.2034:5032 152.38158:5777 157.5979:6596 | -2.16E-06 |
| NEG9741 | Bartramiaflavone | 5.139 | 585.06635 | [M-H]- | 585.0675 | 51.88619:5547 57.33037:6239 61.31321:5860 74.51412:6798 89.13125:1512759 90.13486:55324 363.81647:7398 | -1.97E-06 |
| POS8348 | Napelline N-oxide | 6.748 | 398.23111 | [M+H]+ | 398.23013 | 149.02057:11000 214.37152:5354 267.62708:5763 355.32816:6300 | 2.461E-06 |
| POS8046 | tibiotic KA 6606 X | 5.699 | 376.25641 | [M+H-H2O]+ | 376.2554 | 57.06893:699280 58.07269:10234 67.26308:5204 70.55553:5401 116.75143:6984 125.07156:8673 126.0536:21966 140.03654:5666 208.147:6492 209.12428:43279 210.11501:7913 213.08969:6199 218.12753:8382 219.11333:6674 275.17349:7102 293.17691:7592 302.17621:13491 376.26236:14500 | 2.684E-06 |
| POS9507 | Ebastine | 5.062 | 470.3064 | [M+2H]2+ | 470.30539 | 57.03287:43096 58.04042:26597 59.04859:972238 60.05226:23087 65.04819:28614 69.06992:16341 73.02812:179898 73.06432:46282 80.0543:63123 83.04932:11665 85.06337:33685 87.04335:232396 87.06079:83016 87.07999:23623 89.05896:1608846 90.06191:47124 94.06947:17818 101.05931:142289 102.06606:46435 103.07444:213219 109.07315:44856 109.57641:10420 115.07378:11588 117.05281:14388 117.0909:18327 124.08022:10414 127.07501:10495 129.08911:26812 131.06766:25568 133.08545:525393 134.08684:26022 140.09358:10795 145.08403:11520 147.10149:103445 177.11285:52604 191.1256:9549 | 2.148E-06 |
| POS12233 | Manzamine X | 4.833 | 581.34735 | [M+Na]+ | 581.34851 | 104.79475:14431 | -2E-06 |
| POS7958 | Octaethylene glyco | 4.499 | 371.2265 | [M+H]+ | 371.22751 | 59.04859:14098 73.06432:6886 87.04335:41143 89.05896:878039 90.06191:15323 101.05931:6933 107.07059:8062 131.08699:6423 133.08545:237512 177.11285:17984 285.99164:5706 | -2.72E-06 |
| POS8891 | hosphoryl]oxy-2-l | 6.451 | 436.27899 | [M+H-H2O]+ | 436.28 | 84.57517:5229 90.81367:5393 139.2675:5804 226.54878:6242 235.64577:5658 327.74368:6693 | -2.32E-06 |
| NEG9802 | !'-Methoxyvinaxan | 5.522 | 605.05615 | [M-H]- | 605.05737 | 59.08407:15652 79.05373:33699 81.16663:5854 83.12411:15402 107.21418:10491 135.28148:17770 139.27896:9964 163.31036:9103 173.58612:7013 179.32071:34129 180.33003:7254 | -2.02E-06 |
| POS8678 | (-)-Terpendole B | 5.173 | 422.2674 | [M+2H]2+ | 422.2684 | 57.03286:13210 59.04858:182542 67.01499:21936 81.02915:8017 83.87628:5473 87.04333:18935 89.05895:114294 101.0593:18614 103.07443:42228 117.05552:5882 131.06764:6691 133.08543:28848 147.10147:10643 173.42543:9310 177.11284:6188 191.12558:5809 257.68277:5035 | -2.37E-06 |
| POS13644 | gamma-Truxilline | 4.462 | 659.33124 | [M+H]+ | 659.33258 | 62.52151:10420 86.09536:24341 87.0433:29724 89.05891:43916 95.96495:12038 99.04334:21490 133.08537:23260 166.3371:10479 173.43027:13733 188.06995:50777 205.09811:12467 | -2.03E-06 |
| POS10892 | Leu Asp Asn Arg | 4.473 | 531.28741 | [M+H-H2O]+ | 531.28851 | 79.6421:9188 86.09712:12182 87.05381:26044 89.05896:169138 133.08545:96417 143.89046:9582 146.05721:17239 173.42545:10498 177.1078:12586 188.07004:101378 205.09822:21849 247.72908:9404 248.81287:9322 | -2.07E-06 |
| NEG3662 | oisobutyl phthalic | 1.325 | 221.08037 | [M-H]- | 221.08141 | 79.05373:19243 89.13132:6326 97.07652:16499 163.03299:71272 | -4.7E-06 |

| Differences in metabolites between the Model group and the Control group | | | | | | | |
|---|---|---|---|---|---|---|---|
| Alignment ID | Metabolite name | Rt(min) | Expreiment Mz | Adduct type | Reference m/z | MS/MS spectrum | PPM |
| POS10216 | (4R,5R)-antillatoxin | 5.567 | 504.34421 | [M+H-H2O]+ | 504.34317 | 59.04859:55591 89.05896:23572 101.05931:9029 103.07444:12691 133.08545:9579 175.37425:6049 | 2.062E-06 |
| POS12556 | Dihydroxycanthaxa | 5.831 | 596.38879 | [M+2H]2+ | 596.39001 | 57.03379:25446 59.04859:152492 67.015:12316 73.02812:25803 73.06432:22756 87.04335:42468 89.05896:127194 101.05931:15587 103.07445:19902 133.08545:37316 156.90764:11128 166.20825:10197 271.25543:11374 | -2.05E-06 |
| NEG4399 | TADECA-3,6,9-TRI | 0.839 | 247.24217 | [M-H]- | 247.24319 | 64.0225:5693 113.12118:40005 133.25772:5213 | -4.13E-06 |
| POS3868 | AMOZ | 2.237 | 202.11746 | [M+H]+ | 202.1185 | 55.05365:119164 56.05764:6675 59.04858:9776 67.05389:6586 70.0643:136839 71.04816:29730 71.05974:76454 83.0477:21601 101.0593:34106 110.72093:5593 111.04189:13509 113.0696:13377 114.05462:7124 157.88727:6214 | -5.15E-06 |
| POS9718 | Xestobergsterol B | 6.512 | 481.31287 | [M+H]+ | 481.3139 | 135.48819:5754 342.19617:6394 | -2.14E-06 |
| POS55 | Fluoroacetic acid | 8.803 | 79.01788 | [M+NH4]2+ | 79.0189 | 52.70664:5986 56.62345:5510 63.99746:10521 79.01992:7497 | -1.29E-05 |
| POS4543 | Adalinine | 7.196 | 226.17897 | [M+NH4]+ | 226.17999 | 66.62321:5697 67.05389:9229 81.0699:8029 95.08477:9012 171.1862:5397 173.42545:6583 | -4.51E-06 |
| POS14982 | -3-hydroxydecan | 8.793 | 780.54865 | [M+2H]2+ | 780.547 | 55.74166:5505 103.29499:5156 138.22501:5852 173.39102:13069 | 2.114E-06 |
| POS11481 | PE 22:0 | 5.89 | 552.36475 | [M+2H]2+ | 552.36591 | 57.03287:32364 59.04859:160838 73.02812:48004 73.06432:8898 85.06506:15372 86.09541:13243 87.04335:41960 87.06079:6514 87.07999:12448 89.05896:125523 101.05931:29687 103.07444:27711 104.10697:58575 115.07378:7187 129.08911:6066 133.08545:36473 147.10149:14657 173.39111:10247 184.06931:19733 234.51561:6047 | -2.1E-06 |
| POS15372 | Primycin B1 | 7.169 | 946.69574 | [M+NH4]+ | 946.69373 | 57.03287:19356 66.28696:5270 340.01797:5958 | 2.123E-06 |
| POS13354 | Neomycin | 6.753 | 637.30286 | [M+2H]2+ | 637.30151 | 173.43524:9276 317.64862:5666 438.72989:7181 614.59955:6760 | 2.118E-06 |
| POS15149 | LacCer(d18:1/12:0) | 7.051 | 806.56409 | [M+H]+ | 806.56238 | 96.4697:6680 | 2.12E-06 |
| POS13933 | Cimiracemoside D | 4.602 | 679.40662 | [M+2H]2+ | 679.40515 | 87.04334:261744 89.05896:1951863 133.08543:766098 194.5706:85597 342.42755:85462 469.50879:108563 | 2.164E-06 |
| NEG4861 | decylbenzene-1,3 | 1.051 | 263.21698 | [M-H]- | 263.21805 | 87.11257:8198 89.13129:313336 90.13491:14096 93.0393:531192 95.03768:119175 147.15923:120996 148.16444:6571 151.0672:6294 | -4.07E-06 |
| POS10430 | methyl-1-oxo-2-p | 5.784 | 514.27881 | [M+H]+ | 514.27991 | 59.04858:37228 73.06432:26921 85.06506:7012 87.04334:9315 89.05896:29110 95.08477:6550 101.05712:9332 104.10696:6575 106.33912:5185 126.02023:53819 131.10634:7123 | -2.14E-06 |
| POS11941 | phlomiside | 8.96 | 569.20874 | [M+2H]2+ | 569.20752 | 62.99562:5907 79.55058:5110 104.10696:25395 138.2146:5137 184.07466:10556 366.74438:6801 | 2.143E-06 |
| NEG2214 | Glycylproline | 8.998 | 171.07645 | [M-H]- | 171.07751 | 65.77522:6130 79.0537:8499 91.19855:6752 100.76936:5625 127.03277:51219 171.30643:8510 | -6.2E-06 |
| NEG2215 | Glycylproline | 0.143 | 171.07645 | [M-H2O-H]- | 171.07751 | 109.19514:6443 127.03276:23681 134.32732:5374 171.30641:5507 | -6.2E-06 |
| POS5938 | Obscuraminol A | 6.585 | 278.24634 | [M+H]+ | 278.24741 | 98.85948:6284 111.15255:5710 149.02057:21243 | -3.85E-06 |
| POS9849 | caryolanemagnolo | 5.971 | 487.32169 | [M+H]+ | 487.32059 | 59.04858:18644 66.91378:5588 89.05895:19841 105.14582:6132 138.83788:5569 168.3127:5941 170.74451:5189 | 2.257E-06 |
| POS9374 | -3,4-dehydro-apo | 5.261 | 462.3089 | [M+2H]2+ | 462.31 | 57.03287:68483 59.04859:1640159 60.05226:37212 65.04819:28519 67.05389:7945 69.06992:18982 73.02812:108145 73.06432:26699 80.0543:17455 81.06834:9411 85.06337:37671 87.04335:212219 87.06254:50748 87.07999:35953 89.05896:1176095 90.06191:47001 94.06947:35528 99.07939:10329 101.05931:146547 103.03849:16839 103.07444:333822 104.07732:14685 107.07059:12041 109.0756:14204 115.07378:13589 116.08248:20526 117.05281:12288 117.0909:25986 127.07501:9358 129.08911:28441 131.06766:20109 131.10634:11361 133.08545:327602 134.09018:19521 145.08403:8459 147.10149:147866 161.1188:9065 177.11285:24585 191.1256:24974 | -2.38E-06 |
| POS7883 | Betaenone A | 5.159 | 367.24951 | [M+2H]2+ | 367.24841 | 57.03287:82888 59.04859:1792934 60.05226:41220 65.04819:30358 69.06992:10700 73.02812:124861 73.06432:25247 85.06337:32324 87.04335:136399 87.06254:32476 87.07999:23482 89.05896:839407 90.06191:26550 94.06947:35327 101.05931:117040 103.07444:240294 104.07732:10586 109.0756:15513 116.08248:11466 117.05281:10242 117.0909:19812 129.09227:10554 131.07088:12461 133.08545:185155 134.08684:11321 147.10149:97569 177.11285:12571 191.1256:10145 | 2.995E-06 |

| Differences in metabolites between the Model group and the Control group | | | | | | | |
|---|---|---|---|---|---|---|---|
| Alignment ID | Metabolite name | Rt(min) | Expreiment Mz | Adduct type | Reference m/z | MS/MS spectrum | PPM |
| NEG9457 | pferol 3,7,4'-tri-O- | 5.894 | 524.90985 | [M-H]- | 524.91101 | 59.08406:64413 67.13567:8066 79.05222:24424 107.21417:9070 119.23064:5283 140.17944:10809 147.16307:5463 352.116:5702 434.19601:6421 | -2.21E-06 |
| POS5577 | Protriptyline | 4.97 | 264.17349 | [M+Na]+ | 264.17459 | 59.04859:30915 73.06432:6336 77.01915:5714 87.04335:18298 89.05896:14100 101.05931:9161 173.38622:6861 | -4.16E-06 |
| POS12378 | ganorbiformin A | 4.969 | 587.35907 | [2M+H]+ | 587.35779 | 57.03287:19218 59.04859:262800 73.02812:16307 73.06432:11398 85.06337:13281 87.04335:528738 89.05896:240711 101.05931:136286 103.03849:102338 103.07444:68206 115.07378:13795 117.05281:40507 131.06766:19995 133.08545:75808 147.10149:36730 478.21463:10321 | 2.179E-06 |
| POS9696 | ahydro-1H-cyclop | 5.367 | 480.3129 | [M+2H]2+ | 480.314 | 59.04857:103646 60.05124:6194 62.05908:6808 67.01498:9006 73.0281:10117 73.13141:6883 81.06831:8845 85.06335:9736 87.04333:12205 89.05894:73041 90.91968:7216 101.05928:6046 103.07442:20538 105.06943:5431 126.02021:107504 133.0854:12086 159.1172:9335 161.12755:8955 205.14232:6011 205.30017:5483 208.05679:6945 359.60992:5718 401.25186:6022 | -2.29E-06 |
| POS3757 | octylpyrrolidin-2-c | 7.273 | 198.1841 | [M+H]+ | 198.1852 | 75.69515:7077 133.79388:6443 155.98309:6354 168.10655:6377 171.41733:6722 173.39111:11331 | -5.55E-06 |
| NEG3105 | Spermine | 0.809 | 201.2074 | [M-H]- | 201.2085 | 61.06563:9276 62.6869:5420 64.61039:6008 71.497:6145 81.87756:5654 89.09516:25801 89.13128:10589 103.21892:6510 111.10325:24071 149.19171:5498 | -5.47E-06 |
| POS11162 | oxy-5??-cholest-3 | 5.876 | 541.37463 | [M+2H]2+ | 541.37341 | 57.03287:55586 59.04859:377973 60.08025:175833 73.02812:49737 86.09541:411670 87.04335:44070 89.05896:165272 101.05931:55497 103.07444:74900 104.10697:1493231 124.99956:88707 133.08545:44281 184.07466:902685 | 2.254E-06 |
| POS11897 | Hydroxymanzamin | 5.713 | 567.37061 | [M+2H]2+ | 567.36932 | 57.03287:32090 59.04859:225088 65.11697:7883 67.015:9217 69.06868:7730 73.02812:27208 73.06432:12085 80.0543:10143 81.03072:14445 85.06337:6232 87.04335:42018 87.06254:8100 87.07999:15385 89.05896:147403 90.06191:7741 90.55772:6238 101.05931:32599 102.42354:6623 103.07444:62652 115.28882:6132 117.0909:11533 133.08545:48468 147.10149:20078 165.10016:10555 187.26393:6734 224.00723:7255 242.62587:6749 395.24411:6288 | 2.274E-06 |
| POS9944 | -Acetylgentamicin | 5.973 | 492.30383 | [M+H]+ | 492.3027 | 53.31924:7416 59.04858:17883 60.08025:11439 86.09541:18227 89.05896:10776 104.10696:100376 168.57562:4870 173.04839:5391 184.07466:34363 | 2.295E-06 |
| POS14223 | hydroxy-6-(hydro | 4.476 | 703.36108 | [2M+H]+ | 703.36267 | 50.57987:12075 81.83556:12365 89.05891:26074 133.08537:21108 173.42535:18366 | -2.26E-06 |
| POS4790 | tylidene-1-Deoxy | 5.301 | 235.15277 | [M+H]+ | 235.1539 | 59.04859:36308 89.05896:12673 101.05931:7281 | -4.81E-06 |
| POS12572 | Kadsuphilol C | 6.885 | 597.23431 | [M+H]+ | 597.23297 | 68.38469:5406 250.95917:6494 303.30765:6625 | 2.244E-06 |
| POS5064 | Diethyl azelate | 7.266 | 245.17357 | [M+H]+ | 245.17468 | 60.35422:5795 69.06868:7381 72.65029:5639 83.32471:5613 177.83897:6060 | -4.53E-06 |
| POS4789 | tylidene-1-Deoxy | 4.962 | 235.15277 | [M+2H]2+ | 235.1539 | 57.03287:10776 59.04859:40011 75.04173:5838 86.09541:6763 87.04335:6496 89.05896:15004 99.54275:6028 101.05931:12771 104.31946:5300 173.38622:6893 | -4.81E-06 |
| POS7363 | -1-ylidene)-5alph | 5.958 | 339.26678 | [M+H-H2O]+ | 339.26791 | 55.05365:8739 57.06989:9879 59.04858:13318 67.05389:7170 69.06868:11374 76.29413:8746 81.0699:14839 83.08509:6751 93.06841:7072 95.08477:11768 121.09843:6574 180.99232:6405 192.80493:6095 | -3.33E-06 |
| POS11698 | Arg Thr Lys Arg | 5.588 | 560.36389 | [M+2H]2+ | 560.36261 | 57.03287:22512 59.04859:242637 67.015:24194 81.03072:13634 85.90186:12964 87.04335:32407 89.05896:164672 101.05931:33101 103.07444:66674 104.10697:90582 133.08545:45681 147.10149:30558 161.25945:12601 165.10016:17400 168.84389:12040 184.07466:29869 191.1256:13898 239.59464:13538 424.36945:13016 | 2.284E-06 |
| POS14079 | 25-trihydroxy-21a | 4.862 | 691.44312 | [M+H]+ | 691.44153 | 59.04859:232466 73.02812:26440 73.06432:24661 87.04335:121594 89.05896:857079 101.05931:58881 103.07444:87512 131.07088:26065 133.08545:322000 147.10149:51095 173.44016:17372 177.11285:41023 258.35931:15898 268.91992:15496 | 2.3E-06 |
| POS10813 | Mls001140956 | 6.349 | 528.2981 | [M+H]+ | 528.29688 | 59.04856:8319 62.05906:16762 67.05386:6005 73.55632:5951 173.43027:7752 184.07457:6535 195.23669:5533 | 2.309E-06 |
| POS6281 | no-1,3,4-hexadeca | 5.048 | 290.26773 | [M+H]+ | 290.26889 | 55.05366:10786 56.04863:189825 57.06897:135063 58.06417:132218 58.42599:5736 60.04426:25540 68.04951:8877 68.50143:5538 71.08419:38979 74.05887:115193 78.92046:5853 85.10046:11041 86.05939:13852 88.07445:175317 118.08495:35197 122.08031:36038 164.62282:5251 242.24483:35748 290.26651:13312 | -4E-06 |

**Differences in metabolites between the Model group and the Control group**

| Alignment ID | Metabolite name | Rt(min) | Expreiment Mz | Adduct type | Reference m/z | MS/MS spectrum | PPM |
|---|---|---|---|---|---|---|---|
| POS8384 | Uoamine B | 4.787 | 400.28906 | [M+NH4]+ | 400.2879 | 57.03286:47673 59.04858:1348252 60.05225:17710 69.06867:7295 73.02811:10860 73.06431:28986 85.06335:14196 87.04333:55531 87.07997:12585 89.05894:612277 90.0619:10313 100.07596:9477 101.05929:76905 103.07442:379305 107.06819:16122 117.05279:116936 117.09087:34915 130.08707:9278 131.06764:18574 133.08542:88278 145.08401:9514 147.10147:149700 161.11438:29725 191.12556:10875 | 2.898E-06 |
| POS11285 | Arg Ser Lys Arg | 5.349 | 546.3457 | [M+H]+ | 546.34698 | 57.03287:20336 59.04859:239650 67.015:23160 73.02812:21084 81.03072:13272 86.09541:10981 87.04335:27798 87.07999:9468 89.05896:203904 90.06191:10843 101.05931:24012 103.07444:61977 104.10697:83116 117.0909:9549 130.85844:8922 133.08545:77872 147.07468:10073 147.10149:27853 151.08508:14899 158.09198:14052 184.07466:18004 251.92677:8982 466.18372:8502 | -2.34E-06 |
| POS9691 | PC(15:1(9Z)/0:0) | 5.383 | 480.30545 | [M+2H]2+ | 480.30661 | 57.03379:14320 59.04956:151841 67.01499:11221 73.02811:13915 80.43406:5472 81.03072:10483 87.04508:20484 89.05895:111443 101.0593:14821 103.07668:35478 133.08543:33690 147.10147:11830 158.09198:7813 | -2.42E-06 |
| POS3920 | yl)oxy]pyridine-3- | 2.187 | 204.06197 | [M+Na]+ | 204.06313 | 55.93345:6871 60.04426:49156 61.03972:8693 91.0539:6082 204.9343:5201 | -5.68E-06 |
| POS12475 | -Tetradehydroasta | 4.575 | 592.35858 | [M+H]+ | 592.35999 | 87.04333:292582 87.73636:49559 89.05895:2113716 90.0619:265794 133.08543:920538 134.08682:187594 177.10777:66778 188.95941:48504 | -2.38E-06 |
| POS12842 | dyl pseudopteran | 5.051 | 609.37994 | [M+H]+ | 609.37848 | 59.04859:157183 73.02812:36917 80.0543:26331 85.06337:19243 87.04335:74354 89.05896:483808 90.90853:15706 101.05713:40824 103.07444:43739 133.08545:215034 147.10149:33676 549.27478:18568 | 2.396E-06 |
| POS7674 | andamide (20:l, n- | 7.171 | 354.33459 | [M+H]+ | 354.33578 | 51.79896:6119 64.54077:6654 199.06522:5344 352.11353:6186 | -3.36E-06 |
| POS4432 | Carbofuran | 0.688 | 222.11081 | M+CH3OH+H] | 222.112 | 58.06417:33664 62.63849:5800 73.04688:8793 91.0539:29708 114.88578:5683 119.04706:16113 135.04564:8455 209.00752:8800 225.04044:76549 | -5.36E-06 |
| POS13262 | oxowitha-3,5,24-tr | 4.548 | 631.34912 | [M+H]+ | 631.3476 | 69.03294:531950 73.02811:243743 73.06432:134227 76.79319:61089 81.03229:96327 83.0477:276069 87.04334:1322209 89.05896:3918546 90.0619:65491 95.04893:152763 99.04339:1144402 107.07059:83202 109.06336:74333 111.04189:549180 113.05927:78300 125.0596:289524 129.05132:87907 131.07088:170648 133.08543:1947140 137.05981:128580 151.09705:71775 155.07083:464980 173.07771:106829 177.11285:207110 356.07733:73384 | 2.408E-06 |
| POS6902 | kaden-TP NOA 40 | 4.548 | 316.17993 | [M+2H]2+ | 316.17871 | 55.05365:18529 59.04858:24176 67.05389:9614 69.03294:248663 70.03661:6902 71.04816:7926 73.02811:59434 73.06432:11612 78.03804:38009 81.03229:11849 81.0699:28225 83.0477:58645 85.02799:10159 87.04334:192220 88.04782:6438 89.05896:448073 90.0619:13322 94.71547:6485 95.04893:10730 99.04339:104753 99.07938:42340 100.05018:33996 107.07059:6587 109.06336:9850 111.04189:70949 113.05927:7981 117.09089:5997 122.06293:40646 125.0596:14454 129.05446:21948 131.07088:13397 133.08543:80573 137.05981:9095 142.13843:6809 144.07597:11241 155.06668:21540 173.0826:6402 238.23842:6823 | 3.859E-06 |
| NEG1676 | 5-propanoic acid, | 0.976 | 153.06822 | [M-H2O-H]- | 153.067 | 58.00906:6838 79.05367:6097 93.03925:706937 93.15687:31392 95.03763:1316380 154.24498:6898 | 7.97E-06 |
| POS10794 | l-7-oxo-3-oxaper | 5.25 | 527.33826 | [M+2H]2+ | 527.33698 | 57.03287:46245 59.04859:531889 73.02812:105948 73.06432:31040 87.04335:496867 87.06079:42824 89.05896:707738 101.05931:232147 103.07444:118606 104.10697:100247 105.10879:35413 133.08545:221115 147.10149:52471 157.54272:26623 160.78989:23542 173.44016:29715 184.06931:34472 225.7818:24805 | 2.427E-06 |
| POS6384 | Lanyuamide I | 6.014 | 294.24127 | [M+NH4]+ | 294.24249 | 51.58743:5684 55.05365:13873 57.06895:6691 58.06416:16018 59.04858:13208 67.05389:9580 69.06867:6520 69.51213:5415 71.08418:19877 72.06339:5677 79.05312:5969 81.06989:16726 93.0684:10649 95.04893:10753 95.08475:10952 99.07938:9041 103.45982:5507 107.08485:6496 121.10128:6063 151.10899:10128 173.42542:10887 | -4.15E-06 |
| POS3835 | minic acid; Dihydr | 2.265 | 201.08569 | [M+H]+ | 201.0869 | 55.05365:208072 56.94232:6443 59.04858:17433 70.0643:107285 71.05974:20129 73.06432:7664 81.06833:8008 83.0477:29349 101.0593:58942 111.04189:31051 113.0696:30268 113.96312:18595 114.05462:13930 129.05447:6787 | -6.02E-06 |

| Differences in metabolites between the Model group and the Control group | | | | | | | |
|---|---|---|---|---|---|---|---|
| Alignment ID | Metabolite name | Rt(min) | Expreiment Mz | Adduct type | Reference m/z | MS/MS spectrum | PPM |
| POS15091 | S-3466-C | 6.728 | 793.50763 | [M+H]+ | 793.50958 | 87.02763:6464 118.83253:5497 627.29291:5952 661.3858:5900 | -2.46E-06 |
| POS7557 | Dodecyl glucoside | 5.611 | 349.25684 | [2M+H]+ | 349.25809 | 53.00208:46898 55.05365:21485 57.03287:2262521 58.03662:29273 59.04858:645513 60.05225:8515 69.06868:16523 81.0699:61309 97.06461:35786 99.07938:81290 101.09641:13722 115.07378:94912 117.09089:27878 157.12312:40542 | -3.58E-06 |
| POS9429 | R-tetrol 1-O-alpha | 5.97 | 465.34085 | [2M+H]+ | 465.3421 | 53.00125:7212 55.05365:6134 57.03287:1001863 58.03662:16164 59.04858:291038 69.06991:8673 81.06833:29495 84.86993:5864 97.06461:18596 99.07938:66823 101.09641:14815 115.07378:76797 117.09089:25959 157.12312:56319 | -2.69E-06 |
| POS5586 | nethyl-4-n-tridec- | 5.843 | 264.26724 | [M+NH4]+ | 264.26849 | 55.05365:17531 56.04863:6359 57.06896:12214 67.05389:25964 69.06868:16706 70.06431:10714 79.05312:28424 81.05736:7540 81.06834:23025 82.06503:166526 83.08509:8547 87.04334:5763 93.06841:22611 95.07282:8271 95.08477:12848 96.08018:30769 107.08486:9970 107.43556:6034 123.45921:5364 244.9557:5570 264.26468:36412 | -4.73E-06 |
| POS6358 | -beta-D-glucopyr | 5.031 | 293.19446 | [M+NH4]2+ | 293.19571 | 57.03287:7399 59.04859:55202 73.02678:6749 87.04335:11890 89.05896:26747 101.05931:26250 168.88631:5777 184.97366:6137 | -4.26E-06 |
| POS6357 | -beta-D-glucopyr | 5.388 | 293.19446 | [2M+H]+ | 293.19571 | 59.04859:38699 76.22691:6115 87.04335:6678 89.05896:19281 101.05931:11689 122.48106:5725 173.43034:9639 222.31757:5454 | -4.26E-06 |
| POS6356 | -beta-D-glucopyr | 5.168 | 293.19446 | [M+Na]+ | 293.19571 | 57.03287:9277 59.04859:39097 79.92984:5719 87.04335:8527 89.05896:20142 100.119:5361 101.05931:9225 | -4.26E-06 |
| POS7262 | lecatrienoic acid is | 7.32 | 334.30914 | [M+NH4]+ | 334.31039 | 73.8131:5978 173.42545:10635 | -3.74E-06 |
| POS4314 | pha-Acetyl-L-citru | 2.242 | 218.11223 | [M+NH4]+ | 218.11348 | 60.04326:10332 60.08025:11494 70.0643:172582 85.02799:88013 103.29053:5246 103.88376:5663 113.0696:76240 114.05462:66520 115.08438:28031 159.07413:29905 218.09305:6247 | -5.73E-06 |
| POS7263 | lecatrienoic acid is | 7.48 | 334.30914 | [M+H]+ | 334.31039 | 199.73647:5709 | -3.74E-06 |
| POS8229 | lidenebeyer-15-er | 5.054 | 389.24869 | [M+Na]+ | 389.24741 | 59.04858:19214 87.04334:6695 89.05896:9851 101.94215:5459 | 3.288E-06 |
| POS11281 | anine-3beta,17,23 | 5.349 | 546.34113 | [M+2H]2+ | 546.34253 | 57.03287:20336 59.04859:239650 67.015:23160 73.02812:21084 81.03072:13272 86.09541:10981 87.04335:27798 87.07999:9468 89.05896:203904 90.06191:10843 101.05931:24012 103.07444:61977 104.10697:83116 117.0909:9549 130.85844:8922 133.08545:77872 147.07468:10073 147.10149:27853 151.08508:14899 158.09198:14052 184.07466:18004 251.92677:8982 466.18372:8502 | -2.56E-06 |
| POS8915 | legonoxamine D | 5.059 | 437.27228 | M+CH3OH+H]2 | 437.271 | 59.04858:51499 64.85931:7350 67.015:13694 70.60906:5529 73.06432:7510 87.04334:12699 89.05896:82936 90.06191:6787 101.0593:13977 103.07444:11380 109.40909:5558 133.08545:20776 256.47888:5178 | 2.927E-06 |
| POS3073 | ıyl-ribo-hexose-a | 5.004 | 177.11082 | [M+H]+ | 177.11211 | 57.03287:6878 59.04859:13795 75.04312:6009 87.04335:6016 89.05896:7373 130.06477:5905 132.07892:18046 173.39111:13727 | -7.28E-06 |
| POS11380 | beta-Scymnol sulfa | 5.398 | 549.31049 | [M+2H]2+ | 549.30908 | 59.04856:23963 70.72768:5438 86.09537:9585 87.0433:10775 89.05892:37861 104.10692:50456 133.08539:6636 184.06921:49416 270.05994:5654 | 2.567E-06 |
| POS11965 | 4S,5S,6R)-3,4,5-tril | 8.947 | 570.21643 | [M+2H]2+ | 570.2179 | 60.08025:8675 78.17443:5727 86.09541:10618 104.10696:59038 124.64912:5634 184.07466:25470 276.23544:5963 318.97809:6314 | -2.58E-06 |
| NEG7219 | Dodecyl glucoside | 0.951 | 347.24557 | [M-H]- | 347.24429 | 53.43378:6734 93.03925:329943 95.03763:217753 97.03745:17129 103.07946:12491 147.41237:5261 153.06754:11603 164.71239:5910 173.62527:5953 | 3.686E-06 |
| POS13100 | tra-Ac-Glucoerysc | 8.651 | 622.09558 | [M+H]+ | 622.09277 | 57.30859:7273 163.38239:5280 173.3911:15519 184.22493:5325 283.61749:5576 432.82986:5820 | 4.517E-06 |
| POS12383 | tenacibactin G | 5.045 | 587.39984 | [M+H]+ | 587.40137 | 57.03379:49528 59.04859:1150583 73.02812:174833 73.06432:106459 80.0543:36403 85.06337:68389 87.04335:364272 87.06254:45358 87.07999:39762 89.05896:2128959 90.06191:78061 101.05931:212338 102.06606:25877 103.07444:373822 109.0756:31911 115.07378:27184 117.05281:75104 117.08817:28534 129.08911:36362 131.06766:60847 133.08545:963873 134.09018:26638 147.10149:256485 161.11441:42884 173.39111:45294 177.11285:102401 191.1256:39949 205.57848:24855 336.53061:25361 | -2.6E-06 |
| POS12353 | Leu Ser Leu Pro Gl | 5.604 | 586.34613 | [M+H]+ | 586.3446 | 55.58558:7301 59.04856:39906 60.08022:16137 86.09536:32586 87.04504:8769 89.05891:19117 101.47509:6543 104.10692:144929 113.55135:5751 124.99949:8850 137.26674:5675 145.44116:5577 184.07457:48053 | 2.609E-06 |

**Differences in metabolites between the Model group and the Control group**

| Alignment ID | Metabolite name | Rt(min) | Expreiment Mz | Adduct type | Reference m/z | MS/MS spectrum | PPM |
|---|---|---|---|---|---|---|---|
| NEG2861 | ı±)-trans-Nerolid | 0.647 | 193.16119 | [M-H]- | 193.1599 | 55.54954:6353 85.23931:6047 103.05704:52750 | 6.678E-06 |
| POS4060 | Polycanthisine | 7.199 | 208.1682 | [M+NH4]+ | 208.16949 | 52.88708:5961 109.24215:5320 110.50856:7136 124.29422:5920 163.05995:6082 | -6.2E-06 |
| POS9494 | Malonganenone C | 5.369 | 469.31851 | [M+2H]2+ | 469.3172 | 57.03286:56965 59.04858:1142699 59.0593:28750 60.05225:31066 69.0699:12710 73.02811:61570 73.06431:25556 85.06335:27969 87.04333:133959 87.06252:19345 87.07997:25264 89.05894:648978 90.0619:25966 94.06945:23606 101.05929:119793 102.06383:7071 103.07442:246499 107.07057:10209 115.07376:9573 117.05279:11042 117.09087:15522 129.0891:18761 130.08705:6924 131.06764:10362 131.10631:10108 133.08542:178867 134.08682:7983 143.1041:8150 145.08401:10484 147.10147:95276 161.11438:12011 173.396:12762 177.11282:10385 191.12556:12121 | 2.791E-06 |
| POS11700 | gamma-Solanine | 5.05 | 560.39716 | [M+H]+ | 560.39569 | 57.03287:43652 59.04859:819372 73.02812:35597 73.06432:39036 87.04335:92548 87.07999:22864 89.05896:397039 90.06191:14676 101.05931:102712 103.07444:286688 115.07378:16524 117.05281:52576 117.0909:29847 126.69132:14541 131.06766:27036 133.08545:98508 134.34398:12696 147.10149:104265 161.1188:38344 214.75627:13536 325.95432:13449 | 2.623E-06 |
| POS8841 | Fumifungin | 5.541 | 432.2977 | [M+2H]2+ | 432.29639 | 57.03287:73422 59.04859:1232213 60.05226:30514 61.02846:7108 65.04931:8454 69.06992:8820 73.02812:48709 73.06432:18833 85.06337:16643 87.04335:61455 87.06079:17964 87.07999:12595 89.05896:345651 90.06191:9339 94.06947:18745 101.05931:80332 101.1816:6440 103.07444:219893 104.0796:11684 115.07378:15155 117.0909:21544 129.08911:6620 131.07088:11849 133.08545:69052 147.10149:65316 161.11441:15682 191.1256:7175 215.3116:6205 | 3.03E-06 |
| POS13218 | baeckein F | 8.764 | 629.09088 | [M+Na]+ | 629.09253 | 62.18739:7329 70.11976:6194 87.02068:5993 91.66339:5579 141.59047:5065 535.01624:6253 561.61584:6028 | -2.62E-06 |
| POS9544 | Betnesol | 4.411 | 473.17471 | [2M+H]+ | 473.1734 | 53.73895:6162 72.08048:11154 79.14983:5657 86.09541:8131 119.82919:5778 137.04604:1404390 138.04726:40284 188.07004:12017 | 2.769E-06 |
| NEG9081 | -2-yl-2,3,4,5,6,7,7 | 0.865 | 469.4657 | [M-H]- | 469.46701 | 113.12114:215686 181.19063:8714 226.34309:5786 | -2.79E-06 |
| POS15336 | Erythrosine | 6.873 | 858.64624 | [M+H]+ | 858.64398 | 53.67301:5841 90.49667:5979 176.32578:6196 278.14832:5738 287.21078:5204 779.2157:6077 | 2.632E-06 |
| POS7610 | andamide (20:2, n | 6.825 | 352.3187 | [M+NH4]+ | 352.32001 | 86.98582:7061 267.80582:6085 | -3.72E-06 |
| POS12957 | pha-cholestane-3 | 5.537 | 615.41187 | [M+2H]2+ | 615.41022 | 57.03287:44080 59.04859:834913 73.02812:47441 73.06432:44656 85.06337:39158 87.04335:140754 87.06254:22146 87.07999:33512 89.05896:715880 90.06191:22219 94.06947:23019 98.14563:19219 101.05931:127727 103.07444:256990 110.08812:18889 115.07378:23200 117.0909:25901 129.09227:24319 131.06766:23162 133.08545:219415 136.89111:17692 147.10149:101774 335.16913:18091 387.0867:18684 611.11322:18948 | 2.681E-06 |
| POS13648 | leptasteroside L | 5.508 | 659.43463 | [M+2H]2+ | 659.4364 | 57.03379:45047 59.04859:610398 73.02812:59204 73.06432:39891 85.06506:23196 87.04335:119176 87.06254:32428 87.07999:21949 89.05896:665859 90.06191:27727 101.05931:133712 103.07444:178434 117.0909:37598 121.28181:21722 129.08911:30531 133.08545:245690 147.10149:94194 170.73015:25919 415.89944:21779 | -2.68E-06 |
| POS7792 | anylaspartylarginir | 4.765 | 361.18155 | [M+Na]+ | 361.18289 | 58.04042:23351 59.04859:104682 65.04819:12460 73.02812:91447 80.0543:31045 80.41393:6032 87.04335:34073 87.06254:8323 89.05896:240925 90.06191:5522 101.05931:7642 102.06606:16955 124.07726:8817 131.06766:8963 131.90631:5727 133.08545:60119 173.43036:8546 300.88321:5418 | -3.71E-06 |
| POS4086 | epoxy-15R-hydro | 4.436 | 209.13698 | [M+NH4]+ | 209.13831 | 59.04859:329315 70.06431:58044 84.07958:16457 85.06506:6163 87.04335:14626 89.05896:314430 94.06359:27752 96.08018:9538 103.07445:14247 107.06821:7843 118.06566:9683 133.08545:25822 146.061:9335 148.11055:35581 187.04947:5586 | -6.36E-06 |
| POS5183 | 5-methylsulfinylhe | 4.956 | 249.16536 | [M+H]+ | 249.164 | 59.04859:32759 73.02812:6702 89.05896:12184 208.26324:6382 | 5.458E-06 |
| POS11847 | Valnemulin | 5.139 | 565.36841 | [2M+H]2+ | 565.36688 | 59.04859:518046 73.02812:56453 87.04335:163613 89.05896:1044131 96.30509:36186 101.05931:63679 103.07444:124619 109.0756:44440 133.08545:456674 147.10149:79674 177.11285:51930 | 2.706E-06 |

| Differences in metabolites between the Model group and the Control group | | | | | | | |
|---|---|---|---|---|---|---|---|
| Alignment ID | Metabolite name | Rt(min) | Expreiment Mz | Adduct type | Reference m/z | MS/MS spectrum | PPM |
| POS5280 | 3,4,6-Penta-Me-A | 4.464 | 253.16315 | [M+H]+ | 253.16451 | 59.04859:545051 85.06337:9005 87.04335:61951 89.05896:1103382 90.06191:21774 103.03849:19756 103.07444:43522 107.07059:20743 112.07435:6289 133.08545:122674 146.87569:6869 151.09306:11128 177.1078:5932 | -5.37E-06 |
| NEG744 | Dimethoxypropan- | 9.109 | 119.07005 | [M-H]- | 119.0714 | 59.08403:37295 61.75145:6227 73.11752:6672 97.57933:4913 100.04586:6139 101.04565:74731 102.04721:49298 105.74018:5590 118.07099:59549 119.06866:51500 | -1.13E-05 |
| POS8909 | isovaleryl-blastmy | 5.754 | 437.19043 | [M+Na]+ | 437.1918 | 59.04858:13381 99.09844:5843 115.8652:5791 173.44014:7355 | -3.13E-06 |
| POS5303 | Iodine | 1.063 | 254.81473 | [M+H]+ | 254.8161 | 61.03867:23704 67.29745:7271 80.94772:593200 138.90459:11091 | -5.38E-06 |
| NEG3376 | Zalcitabine | 4.322 | 210.08705 | [M-H]- | 210.08842 | 66.15359:4819 68.75729:6831 69.07782:4508 74.63445:4580 85.09258:4703 86.00461:4641 93.35798:4824 94.57208:3693 95.05959:4105 95.53884:4481 104.05004:4949 105.25879:5141 106.06308:4313 106.9953:4225 107.84846:5108 110.26845:4463 115.0144:4439 119.9493:4524 122.31573:4279 125.98219:5389 126.38684:4694 126.5578:4420 126.81796:4924 138.25093:4513 141.14272:4189 151.39058:4107 155.47362:3909 167.15324:4146 181.72069:4445 189.89471:4348 198.03937:5441 198.21295:4317 202.14603:4163 207.77614:5262 | -6.52E-06 |
| POS12436 | -2,3,5,6,7,11,12,15 | 4.934 | 590.40765 | [M+NH4]+ | 590.40601 | 59.04859:838808 87.04335:134216 89.05896:939187 101.05931:114236 103.07444:326499 114.22485:21803 117.05281:51142 117.0909:25332 131.07088:30137 133.08545:324380 147.10149:179621 161.11441:25466 177.11285:34271 191.1256:33417 298.89792:23738 347.28244:25441 393.26031:24812 | 2.778E-06 |
| POS12415 | dro-1H-cyclopent | 4.749 | 589.38702 | [M+H]+ | 589.38867 | 59.04858:22575 87.04333:30022 89.05894:167008 91.48834:9489 103.07442:10588 133.08542:58874 165.20952:8194 186.57237:8493 | -2.8E-06 |
| POS15029 | PC(16:0e/12-HETE | 5.901 | 784.5824 | [M+H]+ | 784.58502 | 50.02442:27649 60.08025:49787 86.09541:132816 105.14583:23793 124.99956:27804 173.43526:46783 184.06931:433022 623.297:25599 688.9975:26388 690.32001:28995 | -3.34E-06 |
| NEG2059 | Fluorocinnamic ac | 2.365 | 165.03433 | [M-H]- | 165.03572 | 55.9624:4941 58.11544:4593 59.91349:4666 60.95618:4588 63.27049:4503 64.56695:4678 65.45237:4508 66.11316:4603 70.95638:4896 71.53596:5115 74.69258:4643 76.7502:4013 81.75526:4291 84.90416:4658 88.45958:4754 89.58092:4436 93.14533:4997 94.02954:4403 95.4747:4193 97.07649:23371 97.18539:4177 109.36189:4477 111.2215:4581 115.00906:5117 119.16917:4485 125.9518:4829 136.76608:4327 137.42764:4511 156.04103:4502 159.0804:3900 165.81146:3944 | -8.42E-06 |
| POS11693 | Leu Ser Ala Leu Glu | 5.52 | 560.33051 | [M+2H]2+ | 560.32892 | 53.19652:9330 57.84054:8613 59.04856:55066 60.08023:59508 71.07258:14030 86.09538:143371 87.04332:23037 89.05893:58555 101.05927:12562 103.07441:9952 104.10693:430735 105.11107:11428 124.99951:32392 133.0854:13643 184.0746:298269 325.12164:7536 560.04681:8055 | 2.838E-06 |
| POS11127 | 3R,4R)-3,4-dihydro | 5.978 | 540.30292 | [M+Na]+ | 540.30139 | 57.77541:5576 59.04856:15019 60.08023:6218 86.09538:23974 89.05893:6018 96.25027:5780 104.10693:110546 146.98273:12025 | 2.832E-06 |
| POS5185 | COOH-2But-A7EC | 5.148 | 249.16818 | [M+2H]2+ | 249.1696 | 59.04859:43293 80.82903:5246 89.05896:13447 120.44286:6777 173.39111:7545 | -5.7E-06 |
| POS5184 | COOH-2But-A7EC | 5.326 | 249.16818 | [M+H]+ | 249.1696 | 59.04859:34217 73.56042:5763 89.05896:14240 102.36123:6026 | -5.7E-06 |
| POS9048 | Mevinolinic acid | 5.796 | 445.25461 | [M+H]+ | 445.25601 | 59.04858:9104 86.5484:5603 89.05895:5840 149.04791:5048 173.42543:11955 268.03186:5439 326.53644:5422 | -3.14E-06 |
| POS14930 | PG 36:2 | 8.745 | 775.54608 | [M+2H]2+ | 775.54828 | 68.68565:6044 75.29504:4935 | -2.84E-06 |
| NEG1524 | ,2,3,4-tetrahydrois | 7.486 | 146.09901 | [M-H]- | 146.0976 | 100.04586:8539 101.05436:159156 102.05606:168511 103.057:27012 144.21524:6429 | 9.651E-06 |
| NEG5078 | Sempervirine | 0.991 | 271.12555 | [M-H2O-H]- | 271.12411 | 89.13125:22548 90.1367:8192 93.03925:648444 95.03763:1175372 97.03745:192475 145.23549:9570 153.06755:50462 155.06651:25311 | 5.311E-06 |

| Differences in metabolites between the Model group and the Control group | | | | | | | |
|---|---|---|---|---|---|---|---|
| Alignment ID | Metabolite name | Rt(min) | Expreiment Mz | Adduct type | Reference m/z | MS/MS spectrum | PPM |
| POS10415 | Ala Leu Ala Pro Lys | 5.358 | 513.34088 | [M+2H]2+ | 513.33942 | 57.03379:102069 59.04956:2082748 60.05226:59262 65.04931:17320 69.06992:27439 73.02812:152526 73.06567:64356 80.0543:29008 83.04932:12528 85.06506:58920 87.04509:335997 87.06254:67164 87.07999:63058 89.05896:1542368 90.06374:50734 94.06947:63915 99.08151:17238 101.05931:272991 102.06606:21560 103.04074:17742 103.07444:472059 104.0796:14513 107.07059:25414 109.0756:32055 115.07378:21387 116.08248:25829 117.05553:19112 117.0909:50235 129.09227:45088 131.07088:37560 131.10634:27178 133.08545:580697 134.09018:35650 138.09604:15243 143.1078:17884 145.08403:28035 147.10149:303286 148.1028:18751 161.1188:25219 169.11278:15132 173.43034:24680 177.11285:64152 191.13127:40275 | 2.844E-06 |
| POS6843 | Ficellomycin | 5.412 | 313.19678 | [M+Na]+ | 313.19821 | 57.06989:6684 62.84341:5471 271.15952:10932 313.1973:23428 | -4.57E-06 |
| POS8109 | -1-(2,4,6-trimetho | 5.259 | 381.12848 | [M+Na]+ | 381.12991 | 59.04858:24008 70.1021:6931 73.06432:6414 73.32253:5950 87.04334:10393 89.05896:18119 101.0593:15519 255.73947:5295 | -3.75E-06 |
| POS9766 | Euryspongiol A1 | 5.653 | 483.33017 | [M+2H]2+ | 483.3316 | 57.03287:14812 59.04858:207015 60.05225:6586 62.06014:6340 73.02812:13751 87.04334:13053 89.05896:90974 94.06947:7405 101.0593:21132 103.07444:45253 104.10696:78496 115.07378:7008 133.08545:19303 147.10149:17951 147.60472:5190 173.43034:12231 199.8517:6654 308.36151:5312 | -2.96E-06 |
| POS9769 | Complanadine A | 6.313 | 483.34955 | [M+H]+ | 483.34811 | 59.04856:76678 95.27219:5765 104.10693:24816 117.09086:9704 161.69566:5716 173.4303:10129 184.0746:7882 | 2.979E-06 |
| POS15316 | Antibiotic TM 531 | 6.872 | 851.54895 | [M+H]+ | 851.55139 | 53.28831:7958 54.97478:6765 105.15044:6230 254.47932:5572 438.78909:7185 631.97534:7426 | -2.87E-06 |
| POS12886 | H-3,12-epoxy[1,2]d | 4.61 | 611.35553 | [M+2H]2+ | 611.35376 | 69.03294:60483 73.02811:301618 73.06432:88196 81.03229:85977 83.0477:56965 87.04334:632549 89.05896:3386812 90.0619:114237 91.04457:79164 95.04893:177748 99.04339:106397 111.04189:192093 113.05927:149944 131.06766:63385 133.08543:1148567 134.08684:76949 137.0598:73368 155.07083:93591 164.50946:43316 173.39111:50584 175.10023:43853 177.11285:137209 442.12436:47115 | 2.895E-06 |
| POS10705 | dimine B\|gymnody | 5.355 | 524.3385 | [M+2H]2+ | 524.33698 | 60.08024:562938 71.07259:89143 72.72482:79104 75.99161:84194 86.0954:1219011 89.05895:152949 100.47708:77300 104.10696:6883640 105.10877:170021 118.89655:80900 124.99954:278323 172.39996:81033 184.06927:2543607 253.02956:86323 360.09375:93576 390.51123:104774 | 2.899E-06 |
| POS10859 | leopolic acid A | 6.498 | 530.32086 | [M+H]+ | 530.32239 | 59.04858:7175 67.40431:6374 86.38091:6041 183.17155:5455 277.32327:5659 | -2.89E-06 |
| POS8983 | ommunesin F\|com | 4.842 | 441.26627 | [M+H]+ | 441.2648 | 57.03287:16432 59.04859:379092 73.02812:16020 73.06432:10834 85.06337:7376 87.04335:950837 88.04605:19752 89.05896:261383 97.06461:6118 101.05931:178688 103.03849:176796 103.07444:67373 115.07378:15655 117.05281:107968 131.07088:19277 133.08545:62974 145.08403:6911 147.06319:6605 147.10149:23804 173.38622:6797 | 3.331E-06 |
| POS7836 | Inumakilactone A | 4.814 | 365.12537 | [M+2H]2+ | 365.1239 | 70.19419:5737 70.58992:6560 99.70287:6753 126.41608:6569 162.04507:6534 204.09253:9393 227.08423:22130 250.07343:9381 | 4.026E-06 |
| POS13122 | 3-methoxybenzoy | 5.184 | 623.39606 | [M+Na]+ | 623.39423 | 53.5634:6773 57.68123:8495 59.04859:10286 87.04335:9461 89.05896:24055 115.29946:6531 122.35307:7124 418.62692:6913 | 2.936E-06 |
| POS6626 | -3,15-Docosadien | 8.426 | 303.30313 | [M+H]+ | 303.3046 | 78.00248:5855 173.43027:11384 | -4.85E-06 |

**Differences in metabolites between the Model group and the Control group**

| Alignment ID | Metabolite name | Rt(min) | Expreiment Mz | Adduct type | Reference m/z | MS/MS spectrum | PPM |
|---|---|---|---|---|---|---|---|
| POS13362 | Antioxidant 1098 | 6.565 | 637.49188 | [M+H]+ | 637.49377 | 51.36454:6747 53.10913:5163 53.34431:4667 55.26744:4753 55.4603:4592 57.16814:4529 57.60226:4841 59.38129:5185 59.70594:4757 59.73666:4807 60.54902:4772 61.71716:4371 62.1821:4951 63.79452:4673 67.00437:4054 67.07275:4297 68.12308:4600 72.93169:4974 79.71844:4418 81.57861:4400 93.94611:4260 95.96295:4347 96.63064:5221 99.46171:4827 100.33014:4970 101.19031:4252 107.37337:4320 109.60594:4511 110.68337:3835 111.24065:3892 111.91395:4893 116.4918:4230 119.59566:4429 121.4167:4181 126.0597:4394 127.47894:4810 138.11342:3916 149.29829:4425 154.5083:4636 155.32823:4680 168.06438:3975 171.64886:5563 173.42538:3826 174.58382:5302 186.85178:4110 190.22644:4278 199.62126:4291 204.95943:4169 224.4037:4408 230.90944:5263 276.89719:3772 288.78519:4333 289.20724:5025 311.63177:4714 321.98441:4252 326.27039:5364 337.58038:4794 337.66031:4594 339.78909:4637 342.22339:4357 365.4505:4522 383.93292:4938 418.79239:4649 445.98264:4205 453.12668:4803 552.55151:5532 555.49207:5100 609.55859:5993 | -2.96E-06 |
| POS10837 | etyl-7,8-didehydro | 4.929 | 529.35529 | [M+H]+ | 529.3537 | 57.03287:122787 59.04859:2515289 60.05226:35061 73.02812:49060 73.06432:43538 83.04932:16974 85.06337:48027 87.04335:323380 87.07999:46630 89.05896:1727090 90.06191:39242 99.07939:18467 101.05931:253110 101.09641:16173 103.03849:52425 103.07444:806262 105.09028:15142 115.07378:33323 117.05281:46176 117.0909:45960 129.08911:28993 131.06766:25724 131.10634:22433 133.08545:409248 143.10413:16687 145.08403:30187 147.10149:320582 161.11441:51390 177.1078:18989 191.1256:41225 205.14238:19176 | 3.004E-06 |
| POS6680 | Benzosimuline | 8.807 | 306.1503 | [M+H]2+ | 306.1488 | 103.46206:5460 218.17601:6104 | 4.9E-06 |
| POS12019 | Kinetensin 4-7 | 6.178 | 572.28857 | [M+2H]2+ | 572.29028 | 59.04859:6540 60.08025:10112 76.07848:6124 86.09541:27595 104.10697:101438 105.10879:10998 184.06931:69282 185.07635:9413 186.07535:7044 | -2.99E-06 |
| POS9447 | ethanolamine lyso | 6.406 | 466.32761 | [M+H]+ | 466.3291 | 51.75655:5860 57.03287:13613 59.04858:45287 | -3.2E-06 |
| POS10620 | nigramide I | 5.149 | 521.33887 | [M+2H]2+ | 521.33728 | 59.04859:1043720 60.05226:27521 65.04931:28196 73.02812:149483 73.06432:49144 80.0543:55029 85.06506:39103 87.04335:217216 87.06079:100088 87.07999:44362 89.05896:1615024 90.06191:61559 94.06947:20830 101.05931:118585 102.06606:39513 103.07444:265622 104.10697:19272 109.07315:34636 116.08248:18047 117.0909:18739 129.08911:30466 131.07088:34386 133.08545:555306 134.09018:31190 145.08403:23317 147.10149:146270 173.43034:27456 177.11285:53251 184.06931:72285 191.1256:33343 448.9303:17013 | 3.05E-06 |
| POS7757 | Ile-Arg-Ala | 6.022 | 359.23859 | [M+H]+ | 359.24011 | 52.452:5979 54.32751:8237 69.1946:5060 88.05315:6284 106.14863:6019 180.57471:6387 341.74814:5899 | -4.23E-06 |
| POS8173 | Persicaxanthin | 6.014 | 385.27136 | [M+H]+ | 385.27289 | 67.05389:19140 79.05312:16632 81.06989:7775 91.05389:11767 93.0684:7734 105.06713:5753 180.40804:5330 | -3.97E-06 |
| POS9848 | Ile Val Leu Glu | 5.51 | 487.31403 | [M+2H]2+ | 487.3125 | 57.03287:11754 59.04859:178044 67.015:12952 73.02812:7761 81.03072:9289 87.04335:14631 87.07999:6587 89.05896:107639 101.05931:16636 103.07444:35327 133.08545:34823 147.10149:13984 221.12637:5452 | 3.14E-06 |
| NEG6661 | CCG-47657 | 1.066 | 327.14783 | [M-H]- | 327.1463 | 54.53023:5918 93.03925:570753 95.03763:280756 151.06711:26429 153.06754:25377 211.29141:6546 267.29996:5797 291.4368:5469 | 4.677E-06 |
| POS8760 | ircinic acid A | 6.353 | 427.29388 | [M+2H]2+ | 427.29541 | 57.03287:29050 59.04858:206712 81.03072:22710 115.8304:5330 117.09089:26084 173.43034:8558 | -3.58E-06 |

| Differences in metabolites between the Model group and the Control group | | | | | | | |
|---|---|---|---|---|---|---|---|
| Alignment ID | Metabolite name | Rt(min) | Expreiment Mz | Adduct type | Reference m/z | MS/MS spectrum | PPM |
| POS8932 | hoethanolamine (F | 6.689 | 438.29608 | [M+H]+ | 438.29761 | 52.20243:4490 53.61563:5270 53.66457:4977 60.84661:4453 62.38481:4801 65.33078:4671 65.99124:5305 66.74718:4625 67.60562:4701 70.60014:4795 76.86985:4876 78.83771:5031 82.89027:4843 83.98693:4172 86.43439:4401 87.24602:3800 92.00359:4432 95.965:4148 103.80876:4389 105.82056:4366 112.09728:4502 116.57019:4230 118.66303:4819 120.83271:4774 128.8532:4697 129.61676:4410 132.38916:3731 134.03683:4933 134.87729:3885 137.11153:4585 137.99503:4415 139.85175:4222 141.37001:4694 155.07085:5265 155.99983:4850 156.95407:4549 158.97508:4801 173.45488:5520 174.03526:4117 175.35431:4045 175.96431:4479 198.67374:4813 203.77364:4810 205.12976:5112 222.55981:5364 242.70706:4240 257.70947:3820 271.49545:4172 273.60205:4675 278.77704:4638 293.27414:3851 313.62628:4549 314.39111:7065 352.8526:5080 401.8049:4986 423.26389:4703 | -3.49E-06 |
| POS1260 | Hydroxyphenyletha | 1.517 | 121.07151 | [M+H]+ | 121.07 | 56.04952:14227 61.03869:6216516 72.08047:22252 | 1.247E-05 |
| POS13319 | 3,36,39-Tridecaoxa | 4.563 | 635.38287 | [M+2H]2+ | 635.38483 | 87.04334:469308 89.05896:3297967 90.06374:82939 117.09089:67942 131.07088:76644 133.08543:1125547 177.11285:102442 216.40135:69223 369.65768:67791 | -3.08E-06 |
| POS12958 | achidonic acid-bic | 5.328 | 615.42828 | [M+H]+ | 615.43018 | 55.33458:16317 57.03287:42628 59.04859:680263 69.15135:12526 73.02812:17714 73.06432:47268 83.45882:10977 87.04335:62743 89.05896:223262 101.05931:64144 103.07444:201452 117.05281:48062 117.0909:33547 131.06766:22156 133.08545:41216 147.10149:33225 161.1188:18984 580.7569:13279 | -3.09E-06 |
| POS13273 | palmitoyl-sn-glyce | 4.565 | 632.35535 | [M+H]+ | 632.35339 | 69.03294:129341 73.02811:35006 73.06431:30610 83.04769:63903 87.04333:227547 88.04781:24677 89.05895:619305 90.0619:60743 99.04338:155461 100.04802:31763 111.04189:78529 117.09088:32560 125.05659:34261 129.0513:30956 133.08543:238546 134.08682:38132 155.07082:38177 173.42543:33366 | 3.1E-06 |
| POS14295 | -4a-methyl-3,4,4a | 4.861 | 708.47028 | [2M+H]+ | 708.46808 | 59.04956:128458 67.2335:25062 83.81858:19368 87.04335:67545 89.05896:420225 101.05931:36987 103.07444:52472 131.06766:19661 133.08545:208250 147.10149:30929 177.11285:29017 490.83408:20972 | 3.105E-06 |
| POS8788 | Palau'imide | 5.276 | 429.27655 | [M+H]+ | 429.27509 | 59.04858:78891 62.70562:6008 67.01499:7043 81.03072:6086 87.04334:7795 89.05895:63827 89.54285:5633 101.0593:6206 103.07443:18299 133.08543:19503 133.1481:6079 147.10147:5292 169.53391:6042 296.42734:6526 | 3.401E-06 |
| POS14893 | poline-3r-carboxyl | 4.581 | 771.29462 | [M+H]+ | 771.297 | 66.49142:46233 223.17427:48411 310.88882:50981 | -3.09E-06 |
| POS14229 | thyl)oxan-2-yl]oxy | 5.306 | 703.48169 | [M+H]+ | 703.47949 | 57.03287:69866 59.04859:845872 65.52287:39937 68.4929:35657 87.04335:78830 89.05896:398245 98.69503:47696 101.05931:115384 103.07444:239877 117.05281:55079 133.08545:119557 147.10149:122421 184.06931:55139 | 3.127E-06 |
| NEG296 | heamino)-N-meth | 7.709 | 99.05807 | [M-H]- | 99.0565 | 52.83277:6460 58.19922:5807 | 1.585E-05 |
| POS9971 | 1-(prop-1-en-2-y | 4.799 | 493.2984 | [M+H]+ | 493.29999 | 51.77015:6467 89.05895:10948 109.86272:5593 | -3.22E-06 |
| POS8733 | eptapropylene glyc | 5.228 | 425.3092 | [M+H]+ | 425.31079 | 57.03287:102488 59.04858:1181776 60.05225:14621 87.04334:10245 89.05896:20238 103.96567:5605 115.07378:10823 117.09089:124968 173.3862:6020 175.1301:13398 274.86017:6186 | -3.74E-06 |
| POS9235 | Blazeispirol Z | 4.956 | 455.2811 | [M+H]+ | 455.27951 | 59.04858:74319 73.02812:7384 73.06566:6187 73.29153:6055 87.04334:236414 89.05896:49675 98.93763:4991 101.0593:62978 103.03849:17002 103.07444:13917 115.07378:5845 117.05281:10844 133.08545:14029 161.34744:5438 172.78468:5846 231.72554:5815 345.12427:5506 | 3.492E-06 |
| POS11082 | Val Leu His Arg | 5.602 | 538.34418 | [M+2H]2+ | 538.34589 | 57.03286:9390 59.04858:114031 67.01499:6481 73.02811:14208 76.64165:6153 87.04333:18041 87.06252:9217 89.05895:88988 101.0593:11741 103.07443:25937 104.10696:45449 115.07377:5394 133.08543:26685 147.09764:9660 184.06927:14473 | -3.18E-06 |
| POS8892 | Leu Leu Tyr | 6.411 | 436.28207 | [M+H]+ | 436.28049 | 51.60652:6803 160.33104:6165 173.43027:13779 198.89635:5400 | 3.622E-06 |
| POS13777 | | 5.18 | 667.42255 | [2M+H]+ | 667.42041 | 50.11649:13235 59.04859:12614 89.05896:21060 490.27408:12572 | 3.206E-06 |

| Differences in metabolites between the Model group and the Control group | | | | | | | |
|---|---|---|---|---|---|---|---|
| Alignment ID | Metabolite name | Rt(min) | Expreiment Mz | Adduct type | Reference m/z | MS/MS spectrum | PPM |
| POS8967 | (?)-agelasidine E | 5.267 | 440.29572 | [M+2H]2+ | 440.2941 | 57.03287:114316 59.04859:2189595 60.05226:54308 65.04819:34113 69.06992:23163 73.02812:163661 73.06432:40475 80.0543:20518 85.06337:43061 87.04335:292393 87.06254:57682 87.07999:43790 88.04783:13107 89.05896:1435500 90.06191:45631 94.06947:51671 99.07939:14391 101.05931:236192 102.06384:21944 103.03849:23308 103.07444:457049 104.07732:18845 107.06821:11388 109.0756:26532 115.07378:19339 116.08248:16834 117.05281:13209 117.0909:40443 127.07501:9301 129.08911:31106 131.06766:23576 131.10634:12972 133.08545:406086 134.09018:23270 138.09604:14415 143.10413:10586 145.08403:17173 147.10149:185349 161.11441:13617 177.11285:33361 191.1256:26315 | 3.679E-06 |
| POS10008 | Norselic acid E | 5.253 | 495.31253 | [M+2H]2+ | 495.31091 | 57.03287:31592 57.06896:126130 58.06512:54802 59.04859:95775 60.08025:892944 67.05389:28807 71.07261:99655 71.08419:76540 81.06834:31475 83.0851:34036 85.10045:65301 86.09541:1777225 89.05896:124465 95.08477:35028 104.10697:9197985 109.10007:26533 124.99956:409778 133.08545:25773 143.24394:21408 163.01524:25461 184.07466:3315038 424.03168:20732 | 3.271E-06 |
| POS9355 | navirine | 5.671 | 461.31781 | [M+2H]2+ | 461.31619 | 57.03287:59935 59.04859:897027 60.05226:17523 65.04819:8818 69.06868:11381 73.02812:41278 73.06432:16588 87.04335:50952 87.06254:8955 87.07999:14598 89.05896:251265 94.06947:19965 101.05931:62550 103.07444:172294 115.07378:10213 117.0909:25079 129.08911:8562 133.08545:51831 147.10149:63720 161.11441:14604 173.42545:8420 | 3.512E-06 |
| POS9772 | aroten-4'-al; beta | 5.39 | 483.36047 | [2M+H]+ | 483.36209 | 57.03286:60035 59.04857:639142 60.05224:10273 69.8093:6211 73.0643:7539 87.04333:13400 89.05894:31742 99.07937:5985 101.0571:7897 103.07442:7199 115.07375:8980 117.09087:72617 118.09319:5757 133.0854:7041 175.13007:7639 277.59119:5874 418.71881:5733 | -3.35E-06 |
| POS14351 | -acetyltrichagmali | 7.121 | 713.31439 | [M+H]+ | 713.31671 | 110.02861:5819 173.39111:16903 193.88463:5749 | -3.25E-06 |
| POS10701 | oylglycerophosphc | 8.804 | 524.29651 | [M+2H]2+ | 524.29822 | 50.22102:49994 60.08023:159852 69.78801:43132 86.09538:275146 99.47875:45190 104.10693:1657209 105.10875:61902 184.06923:1175168 185.07629:113468 343.16348:49365 428.68253:52781 | -3.26E-06 |
| POS8170 | MLS001074091-01 | 4.518 | 385.24164 | [M+H]+ | 385.23999 | 59.04859:198724 73.06432:23292 85.06506:9454 87.04335:135996 89.05896:1684771 90.06191:27626 101.05931:10071 103.07444:42261 107.07059:17644 118.46617:7133 129.08911:8882 131.06766:22653 133.08545:532233 134.09018:12455 147.10149:19174 177.11285:33121 | 4.283E-06 |
| POS6992 | 3-Hexadecylphenc | 7.728 | 319.29785 | [M+H]+ | 319.2995 | 55.44436:6228 152.06296:5795 | -5.17E-06 |
| POS5965 | 2-Heptanol gluco: | 4.96 | 279.17847 | [M+H]+ | 279.18011 | 57.03287:7884 59.04859:34718 60.22052:7271 87.04335:20299 89.05896:26744 101.05931:55446 103.07444:8419 133.08545:7181 149.02057:9892 | -5.87E-06 |
| NEG9735 | PFSM-amine | 5.075 | 585.06122 | [M-H]- | 585.05927 | 89.13124:1344418 90.13486:49481 91.69405:5157 173.586:6204 236.26202:6039 238.19133:5688 | 3.333E-06 |
| NEG2818 | Cyprotene | 5.414 | 191.17894 | [M-H]- | 191.1806 | 59.02949:33858 87.11256:5863 103.07276:50878 111.14096:13856 134.77959:5929 173.61551:12363 | -8.68E-06 |
| POS9238 | Palmonine A | 5.153 | 455.30197 | [M+2H]2+ | 455.30029 | 57.03287:68998 59.04859:1527596 60.05226:45674 65.04819:25475 69.06868:17124 73.02812:146998 73.06432:42980 80.0543:33719 85.06337:37843 87.04335:245819 87.06079:71965 87.07999:33428 88.04783:10096 89.05896:1523596 90.06191:55922 94.06947:27000 101.05931:149161 102.06606:22085 103.03849:15588 103.07444:321698 104.07732:17927 107.06821:15137 109.07315:36192 115.07378:17028 116.08248:17508 117.05281:25268 117.0909:22044 127.07501:9960 129.08911:27742 131.06766:29751 131.10634:16879 133.08545:448558 134.08684:21470 138.09604:13135 145.08403:11129 147.10149:139436 177.11285:40433 191.1256:18424 | 3.69E-06 |

**Differences in metabolites between the Model group and the Control group**

| Alignment ID | Metabolite name | Rt(min) | Expreiment Mz | Adduct type | Reference m/z | MS/MS spectrum | PPM |
|---|---|---|---|---|---|---|---|
| POS10068 | Tumonoic Acid I | 5.466 | 498.34079 | [M+2H]2+ | 498.34247 | 57.03286:38459 57.06988:22198 59.04858:684673 60.08024:89957 61.08377:15770 65.0493:16494 69.0699:12064 71.07259:12954 71.7504:10678 73.02811:62351 73.06431:11042 75.86941:10828 85.06335:15657 86.09539:198595 87.04333:149263 87.06252:30276 87.07997:13244 87.09917:39303 89.05894:384471 90.0619:13226 94.06945:16235 101.05929:79931 103.07442:110724 104.10695:892489 105.10877:292476 106.11103:40248 111.16512:11085 124.99953:42653 133.08542:94051 147.10147:40128 184.07463:249382 185.07632:98694 186.07532:41872 | -3.37E-06 |
| POS13361 | milicifoline C | 5.521 | 637.42297 | [M+2H]2+ | 637.42511 | 57.03287:62586 59.04859:825992 73.02812:69609 87.04335:158241 87.06079:40926 89.05896:817897 101.05931:144391 103.07444:246986 122.71721:27694 133.08545:284362 147.10149:133885 177.11285:41117 298.98672:31467 | -3.36E-06 |
| POS9308 | SCHEMBL1271357 | 5.06 | 459.28525 | [M+2H]2+ | 459.2869 | 59.04858:34977 59.05443:7569 67.01499:6994 87.04334:11196 89.05895:55091 95.59659:5571 103.07443:11088 133.08543:19402 147.10147:7609 173.3911:11204 270.74762:5613 | -3.59E-06 |
| NEG5611 | ethyl-4-n-pentade | 1.872 | 290.28708 | [M-H]- | 290.2854 | 50.17372:5483 74.03466:7673 90.04866:10248 99.07362:6553 101.05444:6378 104.0432:7910 105.05279:24208 106.2744:5517 114.11422:8284 128.07486:6858 128.10909:6768 130.12769:71969 146.14146:21199 156.13734:53912 157.14728:6726 162.1563:5780 166.15576:7130 174.17671:6195 200.18018:12158 | 5.787E-06 |
| POS6825 | SCHEMBL1021325 | 8.576 | 312.32361 | [M+H]+ | 312.32529 | 55.52958:5945 59.48562:5526 130.17635:5828 236.2997:5516 | -5.38E-06 |
| POS10621 | nigramide I | 6.135 | 521.33905 | [M+H]+ | 521.33728 | 56.04953:14607 60.08025:237305 61.08378:19303 67.05389:10716 71.0726:40524 86.09541:677397 87.09918:72619 98.98415:28602 104.10696:130559 105.1111:17278 124.99955:215033 126.00201:11723 173.43034:12835 184.07466:2453926 185.07635:387950 186.07533:29304 | 3.395E-06 |
| POS14227 | ulomo'opunalide- | 5.483 | 703.46161 | [M+2H]2+ | 703.46399 | 59.04859:390085 73.02946:39071 87.04335:113928 89.05896:523676 101.05931:107492 103.07444:96810 104.81318:28701 116.56748:27100 133.08545:200510 147.10149:61869 177.11285:34186 184.06931:41318 211.92552:23765 328.8551:31348 | -3.38E-06 |
| POS3138 | se, 6-deoxy-3-O- | 0.947 | 179.0896 | [M+H-H2O]+ | 179.09129 | 64.6556:6685 69.68045:5681 79.08632:5474 | -9.44E-06 |
| POS9788 | Poricoic acid B | 5.253 | 484.32059 | [M+2H]2+ | 484.31888 | 57.03287:68054 58.04042:17563 59.04859:1518611 59.05931:38277 60.05226:44010 65.04819:26666 67.05389:10380 69.06992:24349 73.02812:140028 73.06432:39950 80.0543:31676 85.06337:40551 87.04335:266839 87.06079:73682 87.07999:38629 89.05896:1348141 90.06191:47112 94.06947:36153 99.07939:9958 101.05931:204703 102.06384:22097 103.03849:13150 103.07444:343966 104.07732:14918 107.07059:14090 109.0756:29523 115.07378:19859 116.08248:13343 117.05281:17225 117.0909:23356 129.08911:28205 131.06766:22913 131.10634:13401 133.08545:420011 134.08684:29316 143.10413:9866 145.08403:14646 147.10149:155668 148.10667:10781 161.11441:15132 177.11285:33982 191.1256:14469 | 3.531E-06 |
| POS9771 | aroten-4'-al; beta | 5.875 | 483.36038 | [M+H]+ | 483.36209 | 57.03284:903255 58.03659:71234 59.04856:265367 60.05223:21377 60.08022:42360 69.06989:10621 81.0683:24088 86.09537:44687 99.07935:82589 100.08238:14040 101.09636:14544 104.10692:476680 105.10874:72281 115.07373:53297 117.09084:31735 124.9995:17849 157.12306:60753 158.12607:17816 173.42537:15916 184.07457:19753 | -3.54E-06 |
| POS10282 | ethyl-2H-chromen | 4.92 | 507.31213 | [M+Na]+ | 507.31039 | 89.05896:28389 133.08545:8527 328.20291:8787 | 3.43E-06 |
| POS10506 | abloxime / IC202C | 5.249 | 517.32721 | [M+2H]2+ | 517.32898 | 57.03287:9752 59.04859:166769 67.015:27063 73.02812:11806 73.06432:9301 81.03072:6908 86.09541:9540 87.04335:20078 87.07999:7269 89.05896:186589 90.06374:9987 101.05931:11965 103.07444:48519 104.10697:66890 117.93352:5956 133.08545:62091 147.10149:28620 151.08508:9196 177.11285:5900 | -3.42E-06 |
| POS7642 | syl-(1->4)-2,6-dic | 4.817 | 353.21518 | [M+Na]+ | 353.21689 | 57.03287:14908 59.04859:426669 60.05226:7114 71.04816:6970 73.02812:14269 73.06432:9159 87.04335:1004828 88.04605:21520 89.05896:117182 94.90382:7466 101.05931:210115 103.03849:70058 103.07444:29014 117.05281:109733 131.07088:17596 133.08545:19675 | -4.84E-06 |

| Differences in metabolites between the Model group and the Control group | | | | | | | |
|---|---|---|---|---|---|---|---|
| Alignment ID | Metabolite name | Rt(min) | Expreiment Mz | Adduct type | Reference m/z | MS/MS spectrum | PPM |
| POS7524 | oscr#20 | 5.046 | 347.2446 | [M+H]+ | 347.24289 | 55.05364:6974 67.05388:22365 69.0699:10099 79.05311:9369 81.06831:25682 83.08508:9356 93.06838:14687 95.08475:23665 105.06943:9346 107.08484:10309 109.10004:18336 121.10127:13071 293.7276:5209 | 4.925E-06 |
| POS11799 | DTXSID10275904 | 5.859 | 563.38623 | [M+2H]2+ | 563.38831 | 57.03287:26132 59.04859:208545 73.02812:12773 73.06432:14312 87.04335:28288 87.07824:12119 89.05896:134492 101.05931:35914 103.07445:56449 104.10697:22666 112.35517:11952 117.05281:12725 117.0909:14243 133.08545:35059 173.43036:22893 184.81708:13305 244.06889:10416 | -3.69E-06 |
| POS8447 | Glu Lys Glu | 4.377 | 405.19617 | [2M+H]+ | 405.19791 | 53.77364:5498 60.04425:44760 70.06556:11702 86.0954:127568 87.0538:42449 88.03894:25101 90.05456:27388 115.48846:6684 124.03867:12775 128.10568:15187 170.0423:7647 173.12662:170473 205.07927:25285 | -4.29E-06 |
| POS10281 | -8-methoxy-10,12 | 4.779 | 507.30383 | [M+H]+ | 507.3056 | 59.04857:10016 73.0281:6517 89.05894:28411 133.0854:6429 143.883:5278 177.48293:7160 | -3.49E-06 |
| NEG6279 | Arachidic acid | 4.943 | 312.30453 | [M-H]- | 312.3028 | 80.05276:7612 84.76994:6113 97.07439:6550 100.04586:23098 101.05437:45655 167.23665:6156 180.09624:28321 | 5.539E-06 |
| POS8429 | Enactin Ib | 5.415 | 403.27847 | [M+2H]2+ | 403.28021 | 57.03287:82442 59.04859:1698368 60.05226:36274 61.02743:9456 65.04819:10553 69.06868:10839 73.02812:79429 73.06432:24878 85.06337:21941 87.04335:72156 87.06254:16414 87.07999:20441 89.05896:561635 90.06191:17750 94.06947:39409 99.07939:9733 101.05931:95659 103.07444:279788 104.07732:12398 115.07378:14869 117.0909:23914 131.07088:12124 133.08545:117425 147.10149:89823 161.11441:14249 177.1078:7988 | -4.31E-06 |
| POS7880 | 2-Dihydro-(Z,Z)-1 | 4.827 | 367.22177 | M+CH3OH+H] | 367.22351 | 59.04856:37874 70.06428:148208 73.02809:10618 84.07954:43110 87.04331:13396 89.05892:47022 94.06355:14508 96.08015:42066 116.069:13440 133.08539:8421 148.11049:302496 149.11432:17073 165.13654:37938 209.1243:8955 298.54297:5790 | -4.74E-06 |
| POS10566 | Psychotrimine | 7.26 | 519.32117 | [M+H]+ | 519.323 | 50.16373:6887 173.39102:12142 190.7968:5946 313.43536:5778 443.14413:5879 | -3.52E-06 |
| POS8441 | an-2-yl]propanoyl | 5.07 | 404.26877 | [M+2H]2+ | 404.267 | 57.03287:58837 58.04137:29486 59.04859:1616627 60.05226:35988 65.04819:36403 69.06992:16574 73.02812:189531 73.06432:37543 80.0543:43942 85.06337:37458 87.04335:213136 87.06079:98341 87.07999:25048 87.56366:9658 89.05896:1617776 90.06191:48771 94.06947:38166 99.07939:10110 101.05931:120398 102.06606:31638 103.03849:13374 103.07444:284408 107.07059:17747 109.07315:50609 116.08248:18777 117.0909:19918 129.08911:33830 131.06766:24497 131.08699:18428 133.08545:484706 134.09018:23158 140.09358:10265 147.10149:112636 177.11285:42992 191.1256:16952 | 4.378E-06 |
| POS4702 | hylideneamino)-5 | 5.789 | 232.11319 | [M+2H]2+ | 232.11142 | 57.03287:26397 59.04858:8583 73.45755:5578 104.44542:5183 | 7.626E-06 |
| POS10501 | (2S,3R,4R,5R,6S)-3 | 6.171 | 517.01233 | [M+H]+ | 517.01416 | 57.88688:5908 66.74015:5527 173.43526:8482 259.29834:5302 323.72876:5833 345.17938:6032 | -3.54E-06 |
| POS6132 | Boldione | 5.072 | 285.18777 | [M+H]+ | 285.186 | 55.05453:6606 55.1802:5646 64.45844:6128 67.05389:7664 81.06833:10869 104.02033:5184 106.8283:5606 144.31024:5418 | 6.206E-06 |
| POS3415 | prosulfonyl)ethyl a | 8.788 | 186.98083 | M+CH3OH+H] | 186.9826 | 55.93345:21983 57.9342:7293 58.94153:16957 59.92951:71616 59.94047:26872 60.93742:204263 68.94187:20280 75.94469:6384 77.94041:52171 78.94756:11954 81.06989:8968 86.94053:31410 100.95683:85024 101.96426:52086 104.95158:35621 116.97124:39376 117.95827:18006 117.97753:41988 118.96619:651313 119.97298:735529 120.97546:8423 127.96568:11266 128.96005:20319 129.96925:8824 137.98456:11401 141.98206:61670 142.9902:57285 145.9549:7332 163.96249:17008 | -9.47E-06 |
| POS5070 | 10,11,12,14,15,16,1 | 6.775 | 245.22453 | [M+H]+ | 245.2263 | 107.41644:6377 | -7.22E-06 |
| NEG1727 | -2-Hexenyl propa | 1.257 | 155.10521 | [M-H]- | 155.10699 | 57.10276:10240 59.08403:14502 69.11722:22841 79.05367:640348 80.05276:9872 81.14304:13137 83.15008:8291 93.15687:46848 95.03763:308253 96.13688:8700 97.03745:157327 97.08466:514540 97.10725:17037 109.17062:13483 111.1535:9824 111.17866:27566 119.17749:7602 137.08243:25627 147.46233:5920 154.24498:8477 | -1.15E-05 |

| Differences in metabolites between the Model group and the Control group | | | | | | | |
|---|---|---|---|---|---|---|---|
| Alignment ID | Metabolite name | Rt(min) | Expreiment Mz | Adduct type | Reference m/z | MS/MS spectrum | PPM |
| POS11179 | hexoxy)pentan-2-y | 6.253 | 542.31909 | [M+Na]+ | 542.31714 | 51.5143:6374 54.3645:6571 57.03286:9626 59.04858:18997 60.08024:28518 67.05389:7207 69.0699:7761 71.07259:10684 81.06989:16238 86.09539:168554 95.08475:10387 104.10695:982143 105.10877:23540 146.97893:103052 150.06129:7659 167.15565:6181 173.43031:6585 393.3775:6571 | 3.596E-06 |
| POS10668 | Methylepiamauron | 4.723 | 523.30859 | [M+H]+ | 523.3067 | 60.08024:39206 86.09539:83178 89.05894:24975 104.10695:432274 124.99953:18719 131.77299:5289 184.06927:171092 | 3.612E-06 |
| NEG8043 | Arg Pro Asp | 1.051 | 385.1824 | [M-H2O-H]- | 385.1842 | 59.79719:5582 93.03928:417381 94.34543:5411 95.03767:189130 97.03749:6539 151.06717:41243 153.0676:34035 211.09386:6038 249.88901:7290 296.67526:5707 374.81418:5789 | -4.67E-06 |
| POS14140 | odermin glycolipid | 5.389 | 696.45532 | [M+2H]2+ | 696.45282 | 59.04859:140904 68.52091:34557 87.04335:55010 89.05896:318027 101.05931:43050 103.07444:68044 133.08545:143489 242.30151:31183 382.90112:33882 512.59747:36556 623.69818:38147 | 3.59E-06 |
| POS13845 | inaginsenoside R1 | 5.733 | 673.44965 | [M+2H]2+ | 673.45209 | 57.03287:85024 59.04859:784371 60.05126:27631 64.73385:24077 69.06868:27228 73.02812:76311 73.06432:39863 80.05276:20891 82.95837:15141 85.06337:45652 87.04335:179937 87.06079:38342 87.07999:55866 89.05896:756533 90.06374:30229 94.06947:43568 99.07939:21152 101.05931:189201 103.07444:251639 115.07378:31749 117.05281:32922 117.08817:36493 129.08911:41739 131.07088:26483 131.10634:31873 133.08545:338218 134.09018:24566 147.10149:190411 161.11441:20361 177.1078:45478 181.53217:18532 191.1256:51214 205.1487:16869 211.9454:17643 226.3:16723 340.80035:16961 380.6423:17731 397.44708:19957 | -3.62E-06 |
| NEG948 | yl-2,3-dimethylpe | 1.102 | 127.15112 | [M-H]- | 127.1493 | 82.05115:58658 83.85929:5676 89.13124:245074 90.13486:13532 97.09287:11531 99.88496:5881 126.15527:9329 128.18996:18336 | 1.431E-05 |
| POS1940 | yl]methyl 2-methy | 4.418 | 142.06476 | [M+H-H2O]+ | 142.063 | 51.89838:6403 55.0177:12582 55.05366:20095 56.9414:23620 57.68123:6654 62.30868:6446 64.73273:6578 67.05389:9188 69.03294:11704 69.06992:9261 70.06431:11796 70.44497:5969 72.93707:13392 95.08477:8545 96.08018:8232 113.96312:7614 115.05257:35396 143.0343:35726 | 1.239E-05 |
| POS12302 | Shearinine F | 5.495 | 584.33398 | [M+NH4]+ | 584.33612 | 59.04858:61152 60.08025:92369 62.04125:18600 74.02055:19986 86.09541:196362 89.05896:85023 103.07444:23999 104.10696:871256 124.99955:34437 173.43034:42850 184.07466:412600 | -3.66E-06 |
| POS10435 | Maraviroc | 5.065 | 514.33313 | [M+2H]2+ | 514.33502 | 58.04137:18704 59.04859:457688 60.05226:19809 69.06992:13175 73.02812:109044 73.06432:33504 80.0543:49272 85.06506:17945 87.04335:222579 87.06254:43340 87.07999:14094 88.04605:17976 89.05896:978529 90.06191:29299 94.06947:10053 101.05931:128383 102.06606:39990 103.07444:120581 107.06821:9358 109.0756:27888 116.08248:11130 117.05281:18551 117.0909:14302 124.07726:18119 129.08911:17802 131.06766:31383 133.08545:392702 134.08684:18607 140.09358:9025 147.10149:54001 153.09918:9300 177.11285:40338 191.1256:12368 | -3.67E-06 |
| POS13977 | Rubescensin M | 4.947 | 683.41779 | [M+H]+ | 683.41528 | 71.70737:10859 86.33093:11542 88.84815:11253 105.48704:12829 150.76653:13188 173.39111:27333 | 3.673E-06 |
| POS6704 | Ruscopine | 5.437 | 307.21088 | [M+NH4]+ | 307.21271 | 57.03286:9458 59.04858:17416 | -5.96E-06 |
| POS6451 | dodeca-2,6,10-tri | 4.487 | 297.19003 | [M+H]+ | 297.1882 | 59.04859:673313 73.06432:21185 85.06337:18211 87.04335:153095 89.05896:2518169 90.06191:56779 103.03849:14835 103.07444:72495 107.06821:28876 131.06766:10369 133.08545:485030 134.08684:16965 147.10149:19674 151.09705:15676 177.11285:10663 | 6.158E-06 |
| POS7026 | e-(3Z,6Z,9Z)-3,6,9 | 8.429 | 321.31326 | [M+H]+ | 321.31509 | 70.24474:7366 76.22115:6057 158.60115:5338 319.11258:6577 | -5.7E-06 |
| POS13677 | Eupassofilin | 4.946 | 661.43335 | [M+H]+ | 661.43091 | 57.03287:41420 59.04859:805266 73.02812:28269 73.06432:22540 85.06337:22492 87.04335:219622 87.07999:37638 89.05896:1216031 90.06375:24636 101.05931:162048 103.07444:273206 117.05281:18477 129.08911:26497 131.10634:29001 133.08545:406824 134.09018:20388 147.10149:153326 161.1188:17418 177.11285:22391 191.1256:28133 271.96683:16540 | 3.689E-06 |

| Differences in metabolites between the Model group and the Control group | | | | | | | |
|---|---|---|---|---|---|---|---|
| Alignment ID | Metabolite name | Rt(min) | Expreiment Mz | Adduct type | Reference m/z | MS/MS spectrum | PPM |
| POS10414 | rynamine A\|Pseud | 5.1 | 513.32422 | [M+Na]+ | 513.32233 | 57.03287:25815 59.04859:295939 73.02812:54974 73.06432:16633 80.05276:16281 85.06337:12561 87.04335:987143 87.06254:29520 88.04605:16722 89.05896:348376 101.05931:308362 102.06606:14154 103.03849:24348 103.07444:75383 115.07643:9455 117.05281:44277 117.0909:8690 130.0807:9060 131.07088:24750 131.10634:7846 133.08545:113121 147.10149:23943 177.1078:11328 406.83331:7597 | 3.682E-06 |
| POS4727 | rcero-pentulose) 1 | 4.815 | 233.10005 | [M+NH4]+ | 233.1019 | 53.03772:63084 54.04139:13798 55.01768:123942 55.05363:106245 56.02068:24513 56.05671:34031 57.03284:185372 57.06894:2077693 58.03659:32060 58.07269:616833 59.04856:33323 59.07586:10628 61.01:169423 62.01289:26131 63.00633:121529 67.01733:18516 67.05386:80745 68.05671:15839 68.99598:18621 69.03291:13742 69.06865:168111 70.0731:52136 71.01213:13405 71.04942:10780 73.00933:9853 75.00541:8774 79.05309:85527 80.05733:38082 81.03226:538095 81.06987:96583 82.03626:199772 82.07298:37309 83.04766:220530 84.0514:89592 85.02796:84541 85.06333:542437 86.03192:21388 86.06792:206682 87.06947:19218 91.05386:14044 93.06837:30923 94.07335:15087 95.0489:38375 95.08473:40722 96.05183:12069 96.08823:22827 97.02761:62571 97.06457:76830 98.03084:28852 98.06837:34211 99.04335:10673 103.03844:9134 107.04913:39429 108.0528:21900 109.06332:13291 109.10002:22181 111.04436:205374 112.04627:92888 113.0489:14727 121.10125:9485 123.0448:26997 124.05049:37016 125.05955:1076340 126.06273:628918 127.06264:86861 131.03217:11939 133.06891:78873 134.07011:101645 135.06244:119802 137.09422:21919 138.09946:13984 139.10875:21397 140.1149:18014 150.10074:9414 167.08138:22464 167.10457:1270805 168.11116:1294474 169.1127:210337 186.10252:11168 187.09335:14999 | -7.94E-06 |
| POS7608 | 2-(3-furanyl)ethyl] | 5.516 | 352.25009 | [M+H]+ | 352.24823 | 57.06894:50991 71.08544:19760 83.08505:5694 84.07954:64985 85.10041:5780 86.09537:12881 88.0744:5945 166.05182:15901 184.06384:35843 | 5.28E-06 |
| POS6075 | CHEBI:70570 | 4.972 | 283.17126 | [M+H]+ | 283.1694 | 55.05365:31218 57.06896:33562 67.05389:13975 69.06868:42773 71.08547:9772 79.05463:10750 81.06833:18709 83.08509:22840 89.05896:7788 93.06841:26898 95.08477:16820 97.06461:7051 97.09953:16189 100.07597:6934 107.08486:13321 109.10006:7549 111.0796:6733 121.1013:12432 135.11307:6930 163.14494:5752 173.43034:16278 | 6.569E-06 |
| NEG1977 | ylsulfanyl-propyl)- | 0.192 | 163.03513 | [M-H]- | 163.037 | 59.08501:6264 101.95877:5287 102.12029:6051 112.62181:5924 | -1.15E-05 |
| NEG298 | neamino)-N-meth | 7.485 | 99.05838 | [M-H2O-H]- | 99.0565 | 50.45867:5282 50.63686:4731 52.5896:5478 54.41459:5216 54.5553:4341 55.59398:5477 55.64116:4879 57.96262:4576 60.51724:5215 62.46269:4877 63.83049:4119 66.61794:5225 67.90253:3883 70.18143:4460 70.47387:6502 72.58765:4299 72.85787:4634 75.44221:4565 75.67907:4878 77.01496:4716 81.56989:4984 87.99705:3960 89.88375:5045 94.98595:4363 96.0257:5005 96.65898:4448 | 1.898E-05 |
| POS9605 | Progeldanamycin | 4.507 | 476.30289 | [M+NH4]+ | 476.30099 | 73.06432:18860 87.04334:121661 89.05896:1662491 90.0619:35266 107.07059:10722 112.07434:13270 117.08817:17685 131.06766:26392 133.08543:736677 134.09018:30300 173.39111:23393 177.11285:97579 | 3.989E-06 |
| POS11816 | anoyl]-hydroxyam | 5.455 | 564.37366 | [M+2H]2+ | 564.37152 | 57.03379:24448 59.04956:417829 60.05325:13891 73.02812:45138 73.06566:15355 85.06506:15845 87.04334:70351 87.06253:15152 87.07999:14180 89.05896:356656 90.06374:14936 99.72215:8955 101.0593:66806 103.07444:125605 109.07559:10320 123.51226:8200 129.09225:11722 130.08708:11693 133.08545:136882 147.10149:68488 177.11285:14320 191.13127:15101 551.74365:8931 | 3.792E-06 |
| POS13171 | Enniatin-B2 | 5.54 | 626.39862 | [M+2H]2+ | 626.401 | 57.03287:25847 59.04859:309793 67.015:22678 69.06992:13608 73.02812:35115 73.06432:25919 85.06337:17718 87.04335:68475 89.05896:400755 90.06191:14581 99.92779:12315 101.05931:62306 103.07444:92552 109.0756:12974 117.08817:21424 131.06766:20017 133.08545:188559 147.10149:65896 173.39111:28819 177.11285:17788 225.6507:12906 | -3.8E-06 |

| Differences in metabolites between the Model group and the Control group | | | | | | | |
|---|---|---|---|---|---|---|---|
| Alignment ID | Metabolite name | Rt(min) | Expreiment Mz | Adduct type | Reference m/z | MS/MS spectrum | PPM |
| POS7380 | cyclohenicosane- | 4.542 | 340.25748 | [M+H]+ | 340.2594 | 59.04856:31007 69.06866:84019 71.08545:8408 73.06429:9314 79.0531:16901 79.1861:6788 87.04331:11348 89.05893:184400 96.08015:372017 97.06458:18211 97.08306:9037 113.10571:162547 114.09121:358557 115.09229:11966 133.08539:41046 173.43028:8567 174.75732:5879 195.7886:6075 209.16328:129619 210.16083:8006 226.19028:7461 227.17245:35459 321.50101:7261 322.24512:7990 | -5.64E-06 |
| POS14697 | zamamidine A | 4.955 | 749.48724 | M+CH3OH+H] | 749.49011 | 59.04859:193782 87.04335:65241 89.05896:529070 101.05713:36432 103.07444:51241 109.67995:36455 133.08545:207760 147.09766:35842 404.62183:33904 474.819:35804 | -3.83E-06 |
| NEG3341 | pentadec-2-ene | 1.342 | 209.22568 | [M-H]- | 209.2276 | 59.08403:13174 79.05367:23100 80.04047:5185 89.13125:25835 93.50523:6111 97.07645:787088 119.08539:8472 128.02501:9076 | -9.18E-06 |
| POS1263 | Dimethoxypropan- | 3.17 | 121.08389 | [M+H]+ | 121.0858 | 56.96447:10960 61.03869:542954 77.03802:17207 84.959:22201 91.05389:21343 93.03563:11806 93.06841:25035 95.04893:44023 103.05421:116503 104.05679:19980 120.0803:84862 121.08412:12230 | -1.58E-05 |
| POS2235 | Aminobenzothiaz | 0.763 | 151.03493 | [M+H]+ | 151.033 | 58.99696:9747 67.05389:7667 68.98123:290371 72.04237:6093 81.06989:9024 82.01395:151616 86.99278:262824 87.99461:6195 91.05389:5746 95.08476:6405 95.43819:6763 96.04378:6050 100.02438:330463 101.02657:9117 105.0024:291039 105.06944:9573 110.00877:47923 110.03355:8945 118.03534:92609 123.0126:44635 123.03898:8102 128.01854:202653 129.01982:7852 132.99644:9000 146.02687:21046 151.00931:6500 151.06114:22776 | 1.278E-05 |
| POS11894 | Thr Tyr Arg Lys | 4.592 | 567.32703 | [M+2H]2+ | 567.32483 | 69.03417:53726 73.02811:220069 73.06432:42869 81.03385:46702 83.0477:46845 87.04334:445389 89.05896:2303357 90.06374:59990 95.04893:148172 99.04339:77710 111.04189:168038 113.05927:63343 131.06766:65612 133.08543:796132 134.09018:48601 137.0598:39987 155.06668:80457 173.39111:53708 177.11285:72286 294.94229:40929 562.07343:45543 | 3.878E-06 |
| POS11924 | His Gly Lys Lys Va | 5.341 | 568.3587 | [M+2H]2+ | 568.35651 | 57.03379:15383 59.04859:190882 67.015:20860 73.02812:32846 73.06432:15990 81.03072:14575 85.06337:15280 87.04335:34936 89.05896:244115 90.06374:13454 97.61116:9353 101.05931:32276 103.07444:65960 133.08545:91691 147.10149:32142 157.22046:9896 158.09198:12284 177.11285:14461 409.98849:9933 | 3.853E-06 |
| POS10589 | Fenestin B | 5.471 | 520.34729 | [M+2H]2+ | 520.3493 | 57.03284:30269 59.04856:486377 60.05223:17744 65.04928:8935 73.02808:33974 73.06429:9876 81.70692:6584 85.06333:12744 86.09536:9245 87.0433:50634 87.06075:10561 87.07994:7390 89.05891:288461 90.06187:7405 96.95786:7399 101.05926:37328 103.07439:85359 104.10692:41217 117.09084:11496 133.08537:66665 147.10141:31501 168.12987:6619 184.07457:22551 240.41701:6519 | -3.86E-06 |
| POS14752 | Microcolin H | 5.991 | 756.54291 | [M+H]+ | 756.53998 | 60.04424:13397 60.08022:24316 86.09537:83658 124.9995:19123 184.06921:140597 | 3.873E-06 |
| POS5068 | 0,11,12,14,15,16,1 | 9.918 | 245.22437 | [M+H-H2O]+ | 245.2263 | 109.76384:5522 194.7397:5349 | -7.87E-06 |
| POS9517 | Terfenadine | 4.807 | 471.31564 | [M+H]+ | 471.31369 | 57.03287:65988 59.04859:1819779 60.05226:33917 61.02846:12331 69.06992:14219 73.02812:38060 73.06432:24521 83.04932:12352 85.06337:36044 87.04335:223799 87.07999:33804 89.05896:1708307 90.06191:29641 99.07939:9761 101.05931:180879 103.03849:32028 103.07444:524725 104.07732:13567 105.09028:11927 107.07059:14424 115.07378:10759 117.05281:32226 117.0909:27327 129.08911:23706 131.07088:27981 131.10634:12667 133.08545:416039 134.08684:13790 145.08403:18900 147.10149:247631 161.11441:20221 177.11285:18828 191.1256:24494 | 4.137E-06 |
| POS11351 | asterone C 22-nic | 4.537 | 548.33484 | [M+H]+ | 548.33698 | 73.06431:42322 87.04333:107792 87.3213:27235 89.05895:1227742 90.0619:142684 117.09088:27070 131.07086:28767 133.08543:418955 134.09016:65235 177.11284:46088 | -3.9E-06 |
| POS295 | Putrescine | 6.474 | 89.00195 | [M+H]+ | 89 | 50.37579:6720 55.05365:10358 55.93435:10232 56.04952:8560 61.02743:22531 61.0346:6141 70.0643:16085 72.93706:86397 74.99706:9477 77.99807:10994 87.00323:63718 88.07444:42139 90.94762:25573 | 2.191E-05 |

| Differences in metabolites between the Model group and the Control group | | | | | | | |
|---|---|---|---|---|---|---|---|
| Alignment ID | Metabolite name | Rt(min) | Expreiment Mz | Adduct type | Reference m/z | MS/MS spectrum | PPM |
| POS10869 | Lys Lys Leu Ala Gly | 5.359 | 530.3681 | [M+2H]2+ | 530.36603 | 59.04858:93051 69.77426:6928 73.02812:7024 73.06432:11558 85.06337:6334 87.04334:36853 87.07998:5721 89.05896:121367 101.0593:25695 103.07444:31208 117.0909:7133 133.08545:41338 147.10149:13551 364.68661:6553 | 3.903E-06 |
| NEG9325 | Maltotriose | 6.334 | 502.90198 | [M-H]- | 502.89999 | 56.04784:6105 67.74068:6038 | 3.957E-06 |
| NEG3004 | N-Glyceryltaurine | 1.205 | 198.04619 | [M-H]- | 198.0442 | 79.05367:22192 81.06151:5960 161.03622:312986 163.03734:726720 164.03838:13700 198.04518:121900 | 1.005E-05 |
| NEG242 | mino-2H-pyrrol-2 | 0.962 | 97.03882 | [M-H2O-H]- | 97.0408 | 50.8479:6218 79.05367:208009 80.05276:81134 81.286:4924 88.2065:5166 97.03745:211881 97.07645:435261 | -2.04E-05 |
| POS6589 | CHEMBL2229478 | 5.069 | 302.21338 | [M+H]+ | 302.2114 | 55.05365:129180 57.03287:9962 57.06896:49742 59.04858:7324 61.01003:8355 67.05389:185932 69.06868:156592 71.04816:31271 71.08547:23748 79.05312:30386 81.06833:309800 82.07301:7658 83.0477:20895 83.08509:116967 85.06337:28484 85.10045:13174 91.0539:49071 93.06841:48134 95.08477:318163 96.08827:11507 97.06461:12086 97.09953:65192 99.07939:5623 105.06945:32571 107.08486:75967 109.06336:16497 109.10006:243459 110.103:11340 111.0796:8286 111.11481:15745 114.59279:5540 119.08333:76117 121.1013:35163 123.08004:23880 123.11523:69731 125.09565:11049 128.06834:25619 129.07336:16376 133.09863:98182 134.10684:6890 135.11646:15927 137.09427:30717 142.08385:6653 144.09827:8149 147.11681:47253 148.11829:5573 149.13002:19925 151.10901:10614 161.13197:18900 163.14494:33349 165.16396:237099 166.16684:15773 167.3136:6215 170.48605:6220 173.39111:6772 178.52345:5523 189.16042:86633 190.16454:6522 207.17024:25460 | 6.552E-06 |
| POS9812 | Tobramycin | 4.847 | 485.29492 | [M+Na]+ | 485.29294 | 57.03287:15398 59.04859:273494 73.02812:14205 73.06432:10434 76.4834:5527 87.04335:649412 88.04605:9919 89.05896:235728 101.05931:130095 103.03849:171420 103.07444:66416 110.9791:5381 115.07378:9879 117.05281:83480 131.06766:16572 133.08545:75681 147.06319:8732 147.10149:25839 173.43526:10832 196.16579:6213 202.72192:6580 | 4.08E-06 |
| POS12173 | NBD-dihydro-Cera | 5.691 | 578.39343 | [M+2H]2+ | 578.39111 | 55.05366:14753 57.03287:53323 59.04859:671995 60.05226:17840 69.06992:10379 73.02812:68294 73.06432:42721 80.0543:8628 81.0699:9920 85.06337:35636 87.04335:91351 87.06079:22020 87.07999:30506 89.05896:411190 90.06375:17210 94.06947:16227 96.50431:9577 99.07939:15179 101.05931:104704 103.07445:194301 115.07378:22064 117.05281:52219 117.0909:29782 119.23139:8095 129.08911:19768 131.06766:41126 131.10634:13377 133.08545:172080 143.10413:9771 147.10149:98957 161.11441:21054 177.11287:21305 191.1256:12890 205.14238:11902 429.96268:8644 | 4.011E-06 |
| POS9714 | Pharboside B | 4.645 | 481.28281 | [M+NH4]+ | 481.28079 | 58.04041:23872 59.04858:35887 69.03294:54111 73.02811:189832 73.06432:62878 80.05429:73051 81.03229:41889 83.04769:28918 87.04334:390027 88.04782:21281 89.05895:2047538 90.0619:89012 91.04643:68448 91.07442:10828 95.04893:82738 99.04339:69170 101.0593:11472 102.06605:41337 107.0682:24784 109.06335:12856 111.0444:82514 113.05927:81471 117.09089:25129 124.08022:17131 125.05959:13639 131.07086:51757 133.08543:782893 134.09016:43064 137.0598:43303 151.09703:24787 155.06668:39977 157.08505:17409 173.39601:11618 177.11284:87990 | 4.197E-06 |
| POS10293 | Val Phe Lys Asp | 6.067 | 508.27853 | [M+H-H2O]+ | 508.27649 | 60.08022:57792 69.06989:10630 86.09537:75225 104.10693:815147 105.10874:13218 124.9995:27646 158.71704:6638 184.07458:32309 335.11627:9683 | 4.014E-06 |
| NEG3492 | N-Nonanoylglycine | 1.45 | 214.14711 | [M-H]- | 214.1451 | 55.67414:6437 65.62659:7043 84.0806:6578 89.13124:8733 126.02458:336706 127.0235:9115 165.97191:5996 | 9.386E-06 |
| POS13266 | ethyl-5-methylide | 5.051 | 631.42297 | [2M+H]+ | 631.42041 | 57.03287:150773 59.04859:2764982 69.06869:38455 73.02812:91875 73.06432:60500 85.06337:66679 85.2186:31195 87.04335:632992 87.07999:84893 89.05896:2537144 90.06191:53195 99.08151:36128 101.05931:507606 103.03849:76130 103.07444:947555 105.09028:34377 107.07059:32935 115.07378:40858 117.05281:76758 117.0909:69998 129.08911:67531 131.06766:89552 133.08545:787532 145.08403:54317 147.10149:647766 161.11441:72138 177.1078:56457 191.1256:119385 264.8284:34663 | 4.054E-06 |

**Differences in metabolites between the Model group and the Control group**

| Alignment ID | Metabolite name | Rt(min) | Expreiment Mz | Adduct type | Reference m/z | MS/MS spectrum | PPM |
|---|---|---|---|---|---|---|---|
| POS4831 | ene-1,3-disulfona | 1.009 | 236.99767 | [M+Na]+ | 236.99969 | 51.28637:5659 62.98058:33092 69.63676:6189 103.01153:22380 132.98657:34966 143.15192:5232 168.37367:5230 169.98517:21779 | -8.52E-06 |
| POS11985 | Cymarine | 4.436 | 571.28479 | [M+Na]+ | 571.28717 | 67.41739:6578 132.3401:5945 146.061:6037 188.07004:25305 205.09822:8072 231.13576:6047 | -4.17E-06 |
| POS6528 | xy-C12 homoserin | 5.084 | 300.21484 | [M+NH4]+ | 300.21689 | 55.53489:6784 57.06894:103329 61.96259:6471 70.06429:18750 71.08545:62549 82.065:18059 95.22827:5435 100.07379:159104 128.0683:699485 129.07332:21185 142.0838:174223 188.09213:79339 254.20921:7240 275.32065:6325 | -6.83E-06 |
| POS11759 | Rhodoxanthin | 4.751 | 562.37769 | [M+NH4]+ | 562.38 | 59.04858:142885 73.02811:8122 73.06431:10740 87.04333:66884 89.05894:514488 90.0619:9773 101.05929:24945 103.07442:58253 129.0891:7427 131.07086:9705 133.08542:221969 147.10147:33828 173.38129:5567 177.11282:26216 | -4.11E-06 |
| POS9670 | -8,10-dioxapenta | 4.554 | 479.27475 | [M+2H]2+ | 479.27682 | 67.05389:8360 69.03294:12926 73.02811:43288 73.06432:9890 81.03229:12823 83.0477:9371 86.09541:21134 87.04334:67828 89.05896:387738 90.0619:11879 95.04893:30411 99.04339:18889 111.04189:45463 113.05927:14954 133.08543:146203 134.08684:9891 137.05981:10721 144.71318:6682 155.07083:10098 173.39111:9454 177.11285:7148 195.11966:8453 388.89212:7036 406.26993:7750 449.97394:7982 | -4.32E-06 |
| POS12677 | tenacibactin H | 5.182 | 601.41455 | [M+H]+ | 601.41705 | 57.03287:78289 59.04859:1548720 73.02812:30158 73.06432:42032 85.06506:26493 87.04335:130026 87.07824:25916 89.05896:502687 101.05931:146764 103.07444:383406 115.07378:48419 117.05281:41343 117.08817:63891 131.07088:41107 133.08545:66329 147.10149:124154 161.11441:40724 173.42545:23346 417.47043:20268 | -4.16E-06 |
| POS7201 | Epijasminoside A | 4.879 | 331.17203 | [M+Na]+ | 331.1741 | 59.04859:12242 81.48219:5523 89.05896:10799 98.88059:5631 107.7904:5492 161.48398:5151 331.17267:9964 | -6.25E-06 |
| POS13453 | NPTX-643 | 5.638 | 644.42688 | [M+2H]2+ | 644.42419 | 59.04856:99508 73.02808:12471 87.0433:26044 89.05892:105064 95.53635:9875 101.05926:14035 103.07439:34219 133.08539:24962 | 4.174E-06 |
| POS11132 | yl-15-nonyl-9-pr | 5.581 | 540.36176 | [M+H]+ | 540.36401 | 51.79255:6873 57.03286:8263 59.04858:62176 60.08024:16632 70.71621:5496 83.32796:6701 86.0954:23095 87.04333:9356 89.05895:22252 104.10696:169093 117.09088:8898 173.43524:6501 184.07465:40385 339.21149:6492 | -4.16E-06 |
| NEG5909 | netamine L | 0.999 | 300.24249 | [M-H]- | 300.2446 | 72.52529:5988 84.68958:5811 93.03925:186879 95.03763:283775 97.07645:7399 110.58968:5112 124.15676:183779 182.18414:20774 291.46884:5531 | -7.03E-06 |
| POS9813 | Pubeside B | 4.98 | 485.3064 | [M+2H]2+ | 485.3085 | 59.04858:79486 60.05225:6663 61.8371:6356 73.02812:27787 73.06432:7785 80.0543:15872 87.04334:33021 89.05896:162685 92.46779:5702 101.0593:23513 102.06606:12967 103.07444:18479 124.07726:7576 133.08545:59366 147.10149:10895 177.11285:5607 | -4.33E-06 |
| NEG3613 | Cimaterol | 9.081 | 218.1279 | [M-H]- | 218.13 | 128.02194:71183 129.03217:6533 133.70459:5598 | -9.63E-06 |
| POS5059 | -5-hydroxy-1-he | 4.501 | 245.1351 | [M+Na]+ | 245.133 | 58.06512:9572 86.09541:13427 122.86917:5221 143.72749:5680 146.99809:6036 173.43526:6088 218.00317:6136 237.56071:5529 245.13695:37659 | 8.567E-06 |
| POS9459 | farneside A | 4.614 | 467.27295 | [M+H]+ | 467.27509 | 73.02812:51321 80.0543:16473 87.04335:104693 89.05896:517633 90.06191:29742 95.04894:19450 107.06821:13535 111.0419:32094 113.05927:15459 133.08545:160752 137.05637:15123 173.39111:37168 173.87758:17191 399.6514:12759 | -4.58E-06 |
| POS13563 | ermoactinoamide | 6.305 | 651.4762 | M+CH3OH+H] | 651.47998 | 57.03379:42684 59.04956:13713 164.73628:5198 650.09869:5839 | -5.8E-06 |
| NEG210 | 4-Pyridinol | 0.963 | 95.03929 | [M-H2O-H]- | 95.03712 | 58.87597:5863 90.95226:5704 95.03763:651935 | 2.283E-05 |
| POS13492 | xy]-19-hydroxyurs | 4.853 | 647.41809 | [M+H]+ | 647.41528 | 59.04859:528102 73.02812:37374 85.06337:36406 87.04335:215381 89.05896:1493702 95.46825:25189 101.05931:125037 103.07444:212478 124.09804:30390 131.07088:38314 131.10634:28086 133.08545:545537 147.10149:101782 161.69572:23650 227.7915:22995 446.18515:25857 | 4.34E-06 |
| POS7512 | s-19-Ene-1,2,4-T | 5.478 | 346.32935 | [M+H]+ | 346.33151 | 56.04953:18614 57.06989:9248 58.06512:15486 74.05886:12861 88.07445:24774 99.30854:6438 | -6.24E-06 |
| POS12731 | 4,9,13,14-pentame | 5.551 | 604.38763 | [M+2H]2+ | 604.38501 | 59.04859:78126 67.015:9397 81.03072:6475 87.04335:8618 87.07824:6789 89.05896:85814 99.40212:5879 101.05931:13335 103.07445:23943 133.08545:24141 154.89267:5869 | 4.335E-06 |

| Differences in metabolites between the Model group and the Control group | | | | | | | |
|---|---|---|---|---|---|---|---|
| Alignment ID | Metabolite name | Rt(min) | Expreiment Mz | Adduct type | Reference m/z | MS/MS spectrum | PPM |
| POS4681 | benzo<a>fluoren | 4.446 | 231.11897 | [2M+H]+ | 231.11681 | 51.75735:6450 56.04863:14775 60.08025:29441 72.08048:25120 84.07958:13355 85.028:226357 86.09541:26358 100.07383:16396 231.12067:29570 | 9.346E-06 |
| POS10946 | Taurocholic acid | 8.928 | 533.32312 | [M+NH4]+ | 533.32544 | 85.06337:13517 104.10696:45688 126.02023:120155 145.09904:11422 160.20474:11112 184.07466:33281 208.06328:16524 209.13087:24366 227.13577:17589 | -4.35E-06 |
| POS9391 | yonarasterol G | 6.563 | 463.29947 | [M+H]+ | 463.2973 | 55.12037:6647 147.88623:5357 180.24678:5464 | 4.684E-06 |
| POS11637 | taumycin A | 5.068 | 558.35614 | [M+2H]2+ | 558.3537 | 59.04859:317628 73.02812:77858 73.06432:28561 76.76718:17249 80.0543:37095 87.04335:342822 87.06254:27695 88.04605:28998 89.05896:611285 90.06191:18244 96.08221:17910 101.05931:127267 102.06606:28792 103.03849:18756 103.07444:54981 109.07315:19595 117.05281:42712 133.08545:194131 147.10149:32335 161.22427:18152 170.08043:20278 173.21472:22817 | 4.37E-06 |
| POS8857 | Salicyloyl-conkurc | 5.156 | 433.28711 | [M+2H]2+ | 433.28491 | 57.03287:122648 59.04859:3019070 59.05931:77019 60.05226:74211 65.04931:33097 69.06992:24835 73.02812:264160 73.06432:66059 80.0543:46306 85.06337:83297 87.04335:410670 87.06254:135765 87.07999:58686 89.05896:2636898 90.06191:104028 94.06947:95750 101.05931:266741 102.06606:26984 103.07444:610018 109.0756:42692 115.07378:33155 116.08248:45168 117.0909:43486 129.08911:70544 131.06766:38930 133.08545:929040 134.09018:38699 138.09604:22047 145.08403:24259 147.10149:303882 177.11285:83305 191.1256:74282 | 5.077E-06 |
| POS9946 | Morusimic acid C | 5.061 | 492.31949 | [M+2H]2+ | 492.31729 | 58.04042:32820 59.04859:673719 60.05226:22571 65.04819:30212 69.06992:13995 73.02812:150374 73.06432:40802 80.0543:70867 85.06337:26951 87.04335:162683 87.06254:76816 87.07999:25394 89.05896:1259871 90.06191:47778 101.05931:88979 102.06606:40417 103.07444:144945 107.06821:13208 109.07315:43866 117.0909:22869 124.08022:12762 129.08911:22447 131.06766:24791 133.08545:464620 134.09018:26379 138.09604:11087 140.09358:16099 147.10149:74442 169.11278:10513 177.11285:54784 191.1256:15896 | 4.469E-06 |
| POS12237 | copadiol decanoat | 6.3 | 581.42255 | [2M+H]+ | 581.41998 | 57.03287:583029 59.04858:187706 81.0699:15293 97.06461:6372 99.07938:49343 101.09422:14097 115.07378:50363 117.09089:20887 157.12312:40594 173.3911:10101 | 4.42E-06 |
| POS13872 | Giganteumgenin D | 5.053 | 675.44958 | [M+H]+ | 675.44659 | 57.03287:53724 59.04859:1120360 73.02812:38305 87.04335:224956 87.07999:22732 89.05896:1106176 90.06191:21493 101.05931:168575 103.07444:380565 107.0254:19690 117.05553:24756 118.83812:18633 129.08911:21403 131.06766:41651 133.08545:349481 145.08403:19166 147.10149:165995 161.1188:22588 177.1078:20334 205.86365:21003 231.24901:24255 | 4.427E-06 |
| NEG1724 | Histidine | 0.976 | 155.06726 | [M-H]- | 155.06947 | 58.04514:5773 59.83095:5668 79.0537:106586 80.66509:5785 81.14307:7922 87.11256:70161 93.15691:34894 95.03767:465257 97.03749:232436 97.07648:103337 111.15354:35922 120.89378:6292 121.51244:5309 137.19968:6037 151.60265:6598 154.24504:10632 | -1.43E-05 |
| NEG1570 | ropenyl)-5-vinylcy | 8.966 | 147.11578 | [M-H]- | 147.118 | 57.0112:14206 59.0295:55214 59.08405:27695 103.07276:20890 103.67512:5608 112.83764:5624 119.54141:5832 | -1.51E-05 |
| POS11021 | Leu Tyr Val Lys | 5.063 | 536.34656 | [M+2H]2+ | 536.34418 | 58.04137:17266 59.04859:301936 73.02812:94452 73.06432:21392 80.0543:43703 85.06337:21301 87.04335:130609 87.06079:36121 87.07999:15848 89.05896:783413 90.06191:27599 101.05931:45697 102.06606:45551 103.07444:85751 109.0756:23540 117.0909:18548 124.08022:22265 129.08911:16082 131.06766:18971 133.08545:313664 134.08684:17615 147.10149:39057 173.42545:16622 177.11285:32828 | 4.437E-06 |
| NEG2568 | 1-Undecanolactor | 8.285 | 183.14153 | [M-H]- | 183.1393 | 55.92466:5540 83.12405:23497 116.58435:5303 142.57878:5698 | 1.218E-05 |

**Differences in metabolites between the Model group and the Control group**

| Alignment ID | Metabolite name | Rt(min) | Expreiment Mz | Adduct type | Reference m/z | MS/MS spectrum | PPM |
|---|---|---|---|---|---|---|---|
| POS13228 | Ferensimycin A | 5.773 | 629.42297 | [M+2H]2+ | 629.42578 | 57.03287:57295 59.04859:461322 69.06992:14182 73.02812:52708 73.06432:29320 85.06337:26788 87.04335:101916 87.06254:20498 87.07999:18603 89.05896:318566 94.06947:24179 101.05931:72536 103.07444:132810 115.07378:17623 129.08911:19577 133.08545:91906 141.32309:13970 147.10149:54603 193.21381:14327 | -4.46E-06 |
| NEG9602 | PFCA-H | 5.373 | 556.98877 | [M-H]- | 556.98627 | 62.79358:5873 79.05221:8913 81.14308:5793 89.53181:5702 181.16978:6607 | 4.488E-06 |
| POS9449 | voretin C\|olovoreti | 5.967 | 466.34497 | [M+H]+ | 466.34271 | 53.00208:10126 55.05365:9939 57.03287:1568713 58.03662:138237 59.04858:423905 60.05225:27359 69.06992:14513 81.0699:41265 87.04334:8140 97.06461:21210 99.07939:98160 100.08458:16083 101.09641:17642 115.07378:97125 116.0771:20054 117.0909:35484 157.12312:76096 158.12614:31178 | 4.846E-06 |
| POS8539 | Lys Lys His | 4.885 | 412.26434 | [M+2H]2+ | 412.2666 | 57.03287:26233 58.04042:51039 59.04859:530500 65.04819:22388 69.06869:9260 71.04816:10028 73.02812:211329 73.06432:27828 80.0543:117431 85.06337:26355 87.04335:191352 87.06079:60341 87.07999:11348 88.04783:9405 89.05896:1205068 90.06191:36132 101.05931:68762 102.06606:69439 103.03849:14021 103.07444:110287 107.07059:8640 109.0756:34035 111.07206:13151 117.08817:12291 124.08022:28268 129.08911:16022 131.06766:24895 133.08545:404612 134.08684:21362 140.09358:15864 147.10149:40341 173.43036:11455 177.11285:39558 | -5.48E-06 |
| POS10244 | Ergosterol Acetate | 5.62 | 505.34656 | [M+2H]2+ | 505.34427 | 57.03287:66480 59.04859:1008826 60.05226:28304 69.06868:10573 73.02812:56892 73.06432:28375 85.06337:26026 87.04335:95828 87.06079:24889 87.07999:30373 89.05896:400585 90.06191:14719 94.06947:29000 99.07939:8330 101.05931:99646 103.07444:229806 104.07732:12755 109.0756:7315 115.07378:20926 117.05281:15195 117.0909:28429 129.08911:15724 131.07088:11503 131.10634:13149 133.08545:109593 147.10149:84254 161.11441:18592 205.14238:9040 | 4.532E-06 |
| NEG6244 | 5-Diamino-eikosa | 1.852 | 311.34546 | [M-H]- | 311.3432 | 85.13134:7977 87.1126:11482 111.14101:55249 | 7.259E-06 |
| POS9814 | Liquoric acid | 4.921 | 485.32938 | [M+H]+ | 485.32709 | 57.03287:125270 59.04859:2843541 59.05931:71617 60.05226:39477 69.06992:16250 73.02812:37788 73.06432:36680 81.0699:15102 85.06337:51433 87.04335:258376 87.07999:53761 89.05896:1359929 90.06191:23239 99.07939:14334 101.05931:256138 101.09423:18458 103.03849:49684 103.07444:781300 104.07732:24005 105.09028:15188 115.07378:46380 117.05281:66066 117.0909:57051 129.08911:19021 131.06766:26963 133.08545:268187 143.1078:19690 145.08403:15776 147.10149:276400 161.11441:52580 | 4.718E-06 |
| POS5532 | promethyl)chrome | 1.033 | 262.94507 | [M+H]+ | 262.94278 | 62.57782:6309 80.94772:11668 82.94373:53067 85.02795:14817 103.21838:7255 105.81583:6000 110.58839:5851 135.00177:100440 173.43027:5741 | 8.709E-06 |
| POS9672 | Terragine D | 4.717 | 479.28229 | [M+H]+ | 479.28 | 62.54703:5555 89.05896:11415 98.65503:5455 193.53143:7135 243.06476:5573 | 4.778E-06 |
| POS2839 | minoethylarsonic | 9.075 | 169.97691 | [M+H]+ | 169.9792 | 50.41959:6511 55.0177:8175 55.05365:12372 55.93345:449948 65.27977:5757 67.05389:7732 69.93349:5878 70.95692:38693 72.93706:21210 79.05312:9070 81.06989:5857 87.94499:8464 88.96877:12257 96.95995:72113 100.07382:6120 110.05835:6676 111.98266:7744 113.96311:13822 114.97044:527374 128.95062:102406 137.98456:17548 146.95981:36415 | -1.35E-05 |
| POS10622 | nigramide I | 8.81 | 521.33966 | [2M+H]+ | 521.33728 | 50.45496:6193 51.83419:6802 60.08023:43348 67.37577:5809 86.09538:89886 95.44818:6482 104.10693:163753 105.10874:19782 124.9995:22076 128.15237:5616 184.07458:227376 185.07628:8154 195.01427:6031 | 4.565E-06 |
| POS8593 | tadecanedioylcarn | 6.351 | 416.30289 | [M+2H]2+ | 416.3006 | 57.03287:79220 59.04858:714507 60.05225:16413 87.04334:48032 99.07938:16584 101.09422:6412 115.07378:14586 117.09089:53473 175.1301:8154 | 5.501E-06 |
| POS12076 | Cycloposine | 5.121 | 574.3764 | [M+H]+ | 574.37378 | 59.04858:79125 69.92596:5668 73.02811:8315 87.04333:146374 89.05895:88243 101.0593:47183 103.03848:7634 103.07443:16938 117.0528:7801 131.06764:7907 133.08543:24716 147.10147:7331 175.0853:6157 180.86687:5702 198.75192:5900 270.37485:5190 | 4.561E-06 |

**Differences in metabolites between the Model group and the Control group**

| Alignment ID | Metabolite name | Rt(min) | Expreiment Mz | Adduct type | Reference m/z | MS/MS spectrum | PPM |
|---|---|---|---|---|---|---|---|
| POS9651 | cyclo[10.8.0.02,9.04 | 8.809 | 478.32684 | [M+H-H2O]+ | 478.32913 | 50.00464:6662 62.23794:5641 86.09537:7955 87.28099:5024 88.32702:6885 111.99535:7242 119.55073:5597 192.5636:6034 428.54907:6149 | -4.79E-06 |
| NEG5636 | -2-enyl]cyclopent | 1.985 | 291.22513 | [M-H]- | 291.22742 | 60.69548:5592 77.46813:7272 108.77018:6596 126.02459:14571 163.03734:12093 167.73914:5356 285.31952:6500 | -7.86E-06 |
| POS8630 | anodipyrido[1,2-A | 5.274 | 418.28296 | [M+H]2+ | 418.28528 | 55.05366:22078 57.03287:125034 59.04859:2525705 60.05226:57428 65.04819:25872 67.05389:10159 69.06868:20235 73.02812:148140 73.06432:31841 80.0543:9936 81.06834:12473 85.06337:42060 87.04335:225535 87.06254:50750 87.07999:43845 89.05896:1302131 90.06191:37053 94.06947:61475 99.07939:9880 101.05931:181628 103.07444:478159 104.07732:19685 107.06821:12884 109.07315:21801 115.07378:23777 116.08248:26758 117.05281:13252 117.0909:36600 129.08911:35198 130.08389:12682 131.07088:18517 131.10634:10495 133.08545:347346 134.09018:17711 143.1078:13060 145.08403:17356 147.10149:199987 148.1028:10108 161.11441:21964 173.11685:8694 177.1078:26012 191.1256:18382 | -5.55E-06 |
| POS9392 | yonarasterol G | 4.971 | 463.29962 | [M+2H]2+ | 463.2973 | 58.04042:63295 59.04859:504811 65.04819:21997 73.02812:167294 73.06432:25138 80.0543:96970 85.06506:25089 87.04335:177305 87.06079:66151 89.05896:1213248 90.06191:44246 101.05931:69166 102.06606:88313 103.07444:117189 109.07315:38830 111.07206:15858 117.08817:17058 124.08022:34782 129.08911:15408 131.06766:18088 133.08545:425593 134.08684:19498 147.10149:65143 154.63217:12540 173.43036:18277 177.11285:50763 | 5.008E-06 |
| POS8411 | Leu Lys Gln | 4.526 | 402.26868 | [M+NH4]+ | 402.271 | 59.04859:82599 73.06432:9263 85.06337:7422 87.04335:61379 89.05896:874148 90.06375:11985 101.05931:6852 103.07444:22527 107.06821:8809 124.58938:5510 130.08389:5964 131.06766:16379 133.08545:359856 134.09018:8764 147.10149:17029 177.11285:35710 241.19559:6116 | -5.77E-06 |
| POS9847 | Ile Val Leu Glu | 4.639 | 487.31018 | [M+H]+ | 487.3125 | 59.04859:292906 73.02812:30849 73.06432:28687 79.13319:12788 85.06506:12807 87.04335:175797 89.05896:1561067 90.06191:30701 101.05931:34060 103.07444:83540 107.60078:10197 117.6151:9986 129.08911:19638 131.06766:23717 133.08545:549733 134.09018:23234 147.10149:41072 173.43036:22546 177.11285:47516 | -4.76E-06 |
| POS13468 | Fucoxanthin | 5.319 | 645.41791 | [M+2H]2+ | 645.41492 | 59.04859:479514 73.02812:81248 73.06432:51689 87.04335:120059 89.05896:855098 101.05931:131167 103.07444:167082 131.10312:32544 133.08545:362154 134.09018:28192 147.10149:79929 173.44016:26547 177.11285:50734 | 4.633E-06 |
| POS12195 | Hugonone B | 5.191 | 579.37061 | [M+Na]+ | 579.36792 | 59.04859:32268 87.04335:11722 89.05896:47033 96.43513:8825 114.28255:8750 133.08545:19594 274.88956:8878 | 4.643E-06 |
| POS5381 | 4-hydroxyphenyl) | 4.875 | 257.15601 | [M+H]+ | 257.15369 | 55.05365:14169 57.06896:19251 67.05389:17654 69.06868:10464 71.08547:6875 77.8651:5469 81.06833:33341 88.07445:17903 95.08477:27614 102.09264:9084 119.08333:8294 137.1322:7120 | 9.022E-06 |
| NEG6396 | cavernene B | 1.849 | 316.26703 | [M-H]- | 316.26471 | 96.82237:6153 148.11794:11831 182.01009:5761 | 7.336E-06 |
| POS12122 | 2-yl]-18-norpregn | 4.835 | 576.39209 | [M+NH4]+ | 576.3894 | 59.04859:1065958 73.02678:55432 73.06432:42095 85.06337:36108 87.04335:323799 87.07999:47369 89.05896:2615279 90.06375:44507 101.05931:177454 101.09423:22210 103.07444:531423 105.08796:24071 117.05281:53286 117.0909:39218 129.08911:65461 131.06766:92997 131.10634:31421 133.08545:1296119 145.08403:55253 147.10149:480032 161.1188:34809 177.11285:190329 189.11574:24983 191.1256:129740 205.14238:31715 341.20605:24596 | 4.667E-06 |
| POS10173 | oxypoly-angioic ac | 4.92 | 502.35495 | [M+NH4]+ | 502.3526 | 57.03287:43776 59.04859:1181274 73.02812:24017 73.06432:28593 81.0699:9594 85.06337:19759 87.04335:112047 87.07999:18800 89.05896:666070 90.06191:15021 101.05931:129104 101.09423:14091 103.07444:446719 104.07732:10150 115.07378:16237 117.05281:87278 117.0909:38393 129.08911:10081 131.06766:36632 131.10634:12713 133.08545:169742 145.08403:12324 147.10149:206987 148.1028:8780 161.11441:54314 163.5305:8100 167.5183:10008 173.43036:15006 191.1256:24685 205.14238:19739 485.25378:8436 | 4.678E-06 |

| Differences in metabolites between the Model group and the Control group | | | | | | | |
|---|---|---|---|---|---|---|---|
| Alignment ID | Metabolite name | Rt(min) | Expreiment Mz | Adduct type | Reference m/z | MS/MS spectrum | PPM |
| POS12078 | Cycloposine | 5.857 | 574.37646 | [M+2H]2+ | 574.37378 | 57.03287:33910 59.04859:136280 73.02812:28360 81.03072:9795 85.06506:14046 87.04335:41005 87.07999:8481 89.05896:132471 101.05931:27133 103.07444:28370 115.07378:8781 129.08911:12956 133.08545:35650 147.10149:15380 | 4.666E-06 |
| POS9634 | (-)-Asbestinine 2 | 5.142 | 477.31635 | [M+2H]2+ | 477.3187 | 59.04856:188902 60.05123:8391 73.02808:14107 73.06429:6009 85.06333:6872 87.0433:35492 87.06249:7482 89.05891:211438 90.06187:7440 101.05926:18100 103.07439:35720 117.0854:5972 118.5326:5493 133.08537:62620 147.10141:15339 256.90271:6885 462.67981:5708 | -4.92E-06 |
| POS9491 | Ebastine | 5.095 | 469.30042 | [M+Na]+ | 469.29807 | 57.03287:26413 58.04137:13386 59.04859:331842 65.04819:7736 69.06992:9934 73.02812:68830 73.06432:19481 80.0543:23919 85.06337:13467 87.04335:816974 87.06254:31098 88.04605:14580 89.05896:377655 101.05931:311944 102.06384:21137 103.03849:9401 103.07444:63120 109.0756:10877 115.07378:10911 117.05281:29543 117.08817:7011 131.06766:15910 133.08545:112635 145.08403:6826 147.10149:23166 | 5.007E-06 |
| POS9112 | CHEMBL470965 | 5.06 | 448.29343 | [M+2H]2+ | 448.29581 | 57.03287:68804 58.04137:27871 59.04859:1628296 60.05226:39454 65.04819:30631 69.06868:25025 73.02812:244775 73.06432:63531 80.0543:67590 85.06337:54377 87.04335:304086 87.06079:140329 87.07999:39268 87.56366:13261 88.04605:17000 89.05896:2262140 90.06191:80290 94.06947:36830 101.05931:156011 102.06606:47961 103.07444:331285 104.07732:16371 105.09028:10549 107.07059:17179 109.07315:66728 115.07378:15284 116.08248:23736 117.0909:23972 124.08022:24049 129.08911:43356 130.08389:22054 131.06766:42495 131.10634:17943 133.08545:784664 134.08684:42267 138.09604:9933 140.09358:13738 145.08403:15352 146.09132:11834 147.10149:176709 177.11285:79602 191.1256:33857 | -5.31E-06 |
| POS10067 | Poricoic acid A | 5.496 | 498.33691 | [M+2H]2+ | 498.33453 | 57.03287:287249 59.04859:4624726 60.05226:121201 60.08025:118506 65.04819:79492 69.06992:81700 73.02812:449603 73.06432:126714 80.0543:89781 85.06337:157684 86.09541:264607 87.04335:567264 87.06079:199582 87.07999:106976 89.05896:3489491 90.06191:134469 94.06947:103107 101.05931:522818 102.06606:46573 103.07444:1024088 104.10697:1308416 105.10879:365901 106.11105:46620 109.0756:56872 115.07378:52093 117.05281:102652 117.0909:87329 124.99956:93343 129.08911:80034 131.06766:72005 133.08545:1056324 134.08684:51277 143.1078:55076 145.08778:54046 147.10149:505908 161.1188:83836 173.39111:83174 177.11285:126451 184.07466:896408 185.07635:309374 186.07535:146422 191.1256:114480 205.14238:48011 | 4.776E-06 |
| POS8077 | 4-bromophenyl)ch | 0.882 | 378.89838 | [M+Na]+ | 378.896 | 90.97556:16093 106.94932:100680 174.93611:8732 | 6.281E-06 |
| POS8759 | Crustecdysone | 4.798 | 427.28738 | [M+H]+ | 427.285 | 57.03287:96412 59.04859:2660545 60.05226:41071 69.06992:13467 73.02812:38638 73.06432:30020 81.0699:8670 85.06337:43292 87.04335:254559 87.07999:30664 89.05896:1612168 90.06191:30564 99.07939:9460 101.05931:212539 101.09641:11841 103.03849:74057 103.07444:696734 104.07732:15618 105.09028:12907 107.07059:17053 115.07378:13781 117.05281:58399 117.0909:30087 129.08911:22180 131.06766:23475 131.10634:9323 133.08545:323802 143.10413:11097 145.08403:20325 147.10149:280810 161.11441:35992 191.1256:26189 | 5.57E-06 |
| POS8592 | Salmeterol | 4.605 | 416.28442 | [M+NH4]+ | 416.2821 | 59.04859:257618 69.03294:18634 73.02812:10737 73.06432:9467 85.06337:8628 87.04335:70807 89.05896:1019619 90.06191:24733 101.05931:23922 103.07444:79191 107.07059:9047 117.05281:10679 131.07088:14556 133.08545:402513 134.09018:12916 147.10149:58041 173.39111:8539 177.11285:42085 191.1256:10972 | 5.573E-06 |
| NEG5800 | ZINC2171653 | 8.938 | 296.97531 | [M-2H]2- | 296.97769 | 63.70482:5931 97.07649:38057 227.58353:6403 | -8.01E-06 |
| POS1363 | roxy-1,4-benzoqu | 0.991 | 125.02081 | [M+Na]+ | 125.0232 | 101.17503:6058 117.59318:5031 | -1.91E-05 |
| POS11968 | eu Ala Pro Leu Gl | 5.879 | 570.35242 | [M+H]+ | 570.34967 | 59.04858:23768 66.82805:6713 72.18837:5650 86.0954:18019 87.04334:7782 89.05895:14613 94.83833:6230 101.0593:7966 104.10696:112184 146.98277:12615 249.88678:6454 301.56808:5957 | 4.822E-06 |

| Differences in metabolites between the Model group and the Control group | | | | | | | |
|---|---|---|---|---|---|---|---|
| Alignment ID | Metabolite name | Rt(min) | Expreiment Mz | Adduct type | Reference m/z | MS/MS spectrum | PPM |
| POS11188 | Pro Lys Val Lys Ala | 5.459 | 542.3634 | [2M+H]2+ | 542.36603 | 57.03287:86391 59.04859:1612797 60.05226:37101 65.04819:23288 69.06868:20449 73.02812:122944 73.06432:40164 80.0543:32205 83.04932:16424 85.06337:38825 87.04335:284572 87.06254:57144 87.07999:39721 89.05896:1251753 90.06191:29175 91.494:19192 94.06947:41465 101.05931:275969 102.06606:14246 103.07444:386000 104.07732:23684 109.07315:36497 116.0798:27127 117.05281:20293 117.0909:43102 129.09227:40122 131.06766:24424 133.08545:350447 134.08684:14581 147.10149:157161 148.1028:22169 161.1188:29090 177.11285:31342 191.1256:33280 267.83408:14932 311.62006:14301 328.30508:16016 407.45084:15379 | -4.85E-06 |
| NEG3242 | droxytrihomometh | 3.201 | 206.0881 | [M-H]- | 206.08569 | 59.08403:43411 69.11722:14541 73.11618:107827 80.05276:11726 99.1286:9273 101.14601:9415 126.02459:50621 161.03183:6211 161.23819:8132 | 1.169E-05 |
| NEG2671 | Gongrine | 1.501 | 187.08127 | [M-H]- | 187.08369 | 58.0983:58602 62.21653:5617 79.05367:93919 89.09513:6340 97.08466:229408 97.18124:5655 114.16125:6013 115.15741:29718 123.03893:6622 125.22068:17593 125.24475:15819 127.20192:17880 145.23547:7066 | -1.29E-05 |
| POS10256 | Ile Phe Val Lys | 5.247 | 506.33606 | [M+2H]2+ | 506.33359 | 57.03287:71605 59.04859:1823837 60.05226:56884 65.04819:17257 69.06992:25750 73.02812:167496 73.06432:57882 80.05276:35937 85.06337:52209 87.04335:315450 87.06079:100667 87.07999:59629 89.05896:1945502 90.06191:67040 94.06947:47589 99.07939:16286 101.05931:212129 103.03849:24585 103.07444:477548 104.07732:22879 105.09028:12167 107.06821:14019 109.0756:51116 115.07378:18939 116.08248:25256 117.0909:37772 127.07501:16344 129.08911:43925 131.06766:37075 133.08545:727718 134.08684:52970 138.09604:23348 140.09358:12996 143.1078:13173 145.08403:14221 147.10149:272258 148.1028:14220 161.1188:20799 173.11685:15015 177.11285:68870 191.1256:53325 | 4.878E-06 |
| POS4291 | Longiflorone | 5.696 | 217.10446 | [M+NH4]+ | 217.1069 | 55.01768:16464 55.05363:17945 57.03284:43864 57.06986:19536 58.03659:7789 61.01:13364 63.00633:9905 67.01733:8009 67.05269:7271 69.06989:6962 75.02634:22691 77.02202:15905 79.05309:15500 80.05733:12623 81.06987:15537 82.07138:6386 83.04766:31121 84.0514:11510 85.02796:29634 86.03021:6940 97.06457:16772 107.04913:8384 111.04185:21558 112.04627:14442 124.05049:13923 125.05955:275719 126.06273:164950 127.06264:21246 167.10457:283170 168.11116:288651 169.1127:49499 | -1.12E-05 |
| POS11752 | etraen-1-yl]-5,6-d | 5.605 | 562.33954 | [M+H]+ | 562.3374 | 59.04859:14791 60.08025:13667 86.09541:19358 89.05896:9053 104.10697:110067 133.08545:7633 184.07466:28801 | 3.806E-06 |
| POS10996 | Cyclomicrosin | 5.97 | 535.39191 | [2M+H]+ | 535.38928 | 57.03287:347741 59.04858:111809 75.69939:6349 81.0699:7431 86.09711:7236 87.04334:6061 89.05895:13908 99.07938:32438 104.10696:6865 115.07378:24910 117.09089:13262 157.12312:41837 184.06929:20632 192.94875:5606 | 4.912E-06 |
| POS10168 | Delaminomycin A | 5.361 | 502.31866 | [M+2H]2+ | 502.31619 | 57.03287:11288 59.04859:205091 60.05226:11892 67.015:23236 73.02812:8479 73.06432:15896 81.03072:8298 87.04335:23478 89.05896:155828 101.05931:16909 103.07445:53432 117.05281:17865 131.07088:13840 133.08545:41663 147.10149:13157 151.0811:10240 177.11287:6631 204.758:6350 316.92041:6433 | 4.917E-06 |
| POS2577 | Monomethyl adipat | 2.601 | 161.07834 | [M+H]+ | 161.0808 | 56.94137:11350 67.67966:6202 72.93703:15146 105.0694:7747 115.05251:19043 117.05547:13777 118.06284:12438 132.07886:15843 132.97005:6489 160.0741:24901 161.07481:6579 | -1.53E-05 |
| POS8986 | Grevirobstol B | 4.913 | 441.30276 | [M+H]+ | 441.30029 | 57.03287:91869 59.04859:2216200 60.05226:38474 69.06869:14908 73.02812:28853 73.06432:28137 85.06337:23678 87.04335:285232 87.07999:26585 89.05896:622334 90.06191:14456 99.07939:13362 101.05931:162838 101.09423:9388 103.03849:55808 103.07444:518486 104.07732:11131 107.07059:9778 115.07378:27765 117.05281:52568 117.0909:56797 129.09227:9778 131.06766:18558 133.08545:70772 147.10149:131341 161.11441:37325 | 5.597E-06 |
| POS7812 | N-Palmitoyltaurine | 5.844 | 362.24283 | [M+H-H2O]+ | 362.2453 | 57.06989:6537 67.05389:14997 69.06992:8783 79.05312:19566 81.06834:11710 82.06503:43602 91.0539:13094 93.06841:20613 95.08477:9569 96.08018:11956 107.08486:11704 214.23677:6474 264.26468:32965 264.41238:5526 350.88223:5713 | -6.82E-06 |

**Differences in metabolites between the Model group and the Control group**

| Alignment ID | Metabolite name | Rt(min) | Expreiment Mz | Adduct type | Reference m/z | MS/MS spectrum | PPM |
|---|---|---|---|---|---|---|---|
| POS13018 | -D-glucosyl betul | 5.182 | 618.43945 | [M+NH4]+ | 618.4364 | 59.04859:448789 73.06432:32302 87.04335:50417 89.05896:304779 90.06191:27372 101.05931:63464 103.07444:146189 117.05281:60982 117.0909:24036 131.07088:26206 133.08545:75904 147.10149:72940 163.85435:20030 173.39603:31478 474.44147:19340 | 4.932E-06 |
| POS9508 | tumonoic acid G | 5.05 | 470.31366 | [M+H]+ | 470.31119 | 59.04856:210663 64.28428:6020 73.02809:39989 73.06429:12336 80.05426:16006 87.04331:50212 87.0625:18074 87.0782:6990 89.05892:304845 90.06371:9022 101.05927:28035 102.06602:6567 103.07439:47648 109.07555:7757 133.08539:95973 147.10143:14785 177.11278:14535 | 5.252E-06 |
| NEG9597 | efpodoxime?proxe | 8.148 | 556.11475 | [M-H]- | 556.11749 | 58.03277:6804 95.15706:5698 370.90424:6061 | -4.93E-06 |
| POS9269 | Chaxalactin B | 4.713 | 457.29761 | [M+H]+ | 457.2951 | 57.03287:16353 59.04859:529559 73.02812:14687 73.06432:12692 85.06506:15007 87.04335:97771 87.07999:15167 89.05896:994740 90.06191:21788 101.05931:51281 103.03849:11047 103.07444:149086 104.07732:7846 107.06821:9425 129.08911:11107 131.06766:10006 131.10634:8119 133.08545:293362 134.09018:9588 147.10149:73929 177.11285:19039 191.1256:8962 | 5.489E-06 |
| POS12032 | PC-M4 | 5.227 | 572.37628 | [M+2H]2+ | 572.37341 | 57.03286:9966 59.04858:221267 73.02811:28340 73.06431:7596 85.06505:8617 87.04333:77892 87.06252:8328 87.07997:6421 89.05895:322147 90.0619:11336 94.06946:7232 99.61956:6140 101.0593:44944 102.06383:8446 103.07443:60197 104.10696:21277 109.07558:9611 115.77686:5817 131.06764:7148 133.08543:104042 134.08682:6520 147.10147:28222 177.11284:14357 184.07465:6833 415.7355:5473 | 5.014E-06 |
| POS8799 | Mebeverine | 4.958 | 430.26196 | [M+2H]2+ | 430.25946 | 59.04859:51989 67.015:8720 86.36885:5594 87.04335:13277 89.05896:76175 101.05931:6941 103.07445:10649 131.06766:6788 133.08545:16632 135.28865:6161 173.43036:7527 | 5.81E-06 |
| POS13293 | Scaphopetalumate | 5.659 | 633.40973 | [M+2H]2+ | 633.4129 | 59.04859:127985 80.32882:16383 87.04335:24626 89.05896:122135 101.05931:27054 103.07444:37583 103.53445:14955 133.08545:45083 147.10149:17551 162.54245:15598 173.39111:24998 189.11574:16272 209.53416:14642 | -5E-06 |
| POS12430 | ngeloylzygadenin | 5.333 | 590.3717 | [2M+H]2+ | 590.36871 | 59.04859:142035 87.04335:29038 89.05896:142146 103.07444:33614 130.18591:17652 133.08545:65233 308.32666:18721 436.48868:16883 | 5.065E-06 |
| NEG6717 | Schultesine | 1.055 | 329.15332 | [M-H]- | 329.15079 | 80.64178:6422 93.03928:74284 95.03767:32222 124.15681:27274 177.93195:6010 | 7.686E-06 |
| POS13718 | NAD | 8.769 | 663.11243 | [M+H]+ | 663.10907 | 126.32156:5312 166.99335:5442 173.43034:13072 256.35541:5511 | 5.067E-06 |
| POS11488 | Aliskiren-[d3] | 6.453 | 552.39777 | [M+Na]+ | 552.40057 | 60.08122:5983 72.73411:5317 73.3144:6683 76.72816:5385 104.10692:20045 139.75227:5697 184.06921:7460 | -5.07E-06 |
| NEG9583 | (E)-Ceftriaxone | 6.016 | 553.03607 | [M-H]- | 553.03888 | 76.75741:7106 79.0537:6830 121.28275:5667 172.79352:5907 210.16214:6500 250.67091:6197 270.48953:5435 | -5.08E-06 |
| POS7510 | thylnonanedioylca | 4.862 | 346.22552 | [M+2H]2+ | 346.22296 | 57.03287:29942 58.04042:76672 59.04859:875504 60.05226:16977 65.04819:46132 69.06992:7833 73.02812:301787 73.06432:24861 80.0543:87657 85.06337:31198 87.04335:155382 87.06079:98721 89.05896:1272129 90.06191:48954 94.06947:11967 101.05931:49615 102.06606:58963 103.07444:119498 107.07059:17606 109.07315:34147 124.08022:24388 129.08911:18888 131.06766:12967 133.08545:358139 134.08684:19133 147.10149:39424 177.11285:33961 | 7.394E-06 |
| POS9239 | Ilekudinol A | 5.051 | 455.31937 | [M+H]+ | 455.3168 | 57.03287:102228 59.04859:1811090 60.05226:29341 69.06992:11679 73.02812:24976 73.06432:91429 85.06337:16261 87.04335:99042 87.07999:12841 89.05896:216625 90.06374:9159 99.07939:10766 101.05931:96706 101.09423:12097 103.03849:13055 103.07444:357020 115.07378:35759 117.05281:92245 117.0909:73378 131.06766:64145 133.08545:19462 147.10149:47996 161.11441:43254 | 5.644E-06 |
| POS9028 | Nakijiquinone B | 5.157 | 444.27756 | 1+CH3OH+H]2 | 444.27499 | 57.03286:10691 59.04858:120785 67.01498:18566 74.60526:5510 81.03071:8738 87.04333:11223 89.05894:108782 101.05929:7136 103.07442:25807 126.29716:5232 133.08542:32302 147.10147:10585 156.52419:6353 177.11282:6748 | 5.785E-06 |

**Differences in metabolites between the Model group and the Control group**

| Alignment ID | Metabolite name | Rt(min) | Expreiment Mz | Adduct type | Reference m/z | MS/MS spectrum | PPM |
|---|---|---|---|---|---|---|---|
| POS7359 | U 6796 | 4.768 | 339.23547 | [M+H]+ | 339.23291 | 57.03287:107158 59.04859:2537525 60.05226:41069 69.06992:7865 73.02812:29093 73.06432:57512 85.06337:17847 87.04335:129956 87.07999:13310 89.05896:412178 90.06375:8792 99.07939:7426 101.05931:68198 103.03849:39782 103.07444:344578 104.07732:8444 115.07378:11308 117.05281:145545 117.0909:37751 131.07088:31810 133.08545:17737 147.10149:47546 161.11441:15716 | 7.546E-06 |
| POS9551 | yl apo-6'-lycoper | 6.863 | 473.34387 | [M+H]+ | 473.34131 | 51.72378:6127 53.48267:6634 91.91269:5447 97.12214:6040 219.6438:5558 337.36746:5935 381.37701:5360 | 5.408E-06 |
| POS10297 | PC(17:1(10Z)/0:0) | 6.412 | 508.33618 | [M+H]+ | 508.33881 | 55.6239:5924 59.04858:6614 60.08025:18263 86.09541:45672 104.10697:293443 105.1111:10635 124.99956:13625 148.10667:5865 173.38622:11353 184.06931:103155 295.92383:6376 | -5.17E-06 |
| NEG4148 | pentadecanone C | 2.49 | 238.22028 | [M-H]- | 238.2177 | 101.31654:6837 108.03613:9828 108.22439:5969 124.04993:7581 180.11699:6444 | 1.083E-05 |
| POS8350 | hylethyl)phenyl]su | 5.664 | 398.23859 | [M+Na]+ | 398.23599 | 59.04856:6062 71.14339:6012 130.37427:5976 | 6.529E-06 |
| POS9933 | Herbasterol | 5.372 | 491.33173 | [M+2H]2+ | 491.33432 | 55.05366:26013 57.03287:120930 59.04859:2295520 60.05226:72763 65.04931:26636 69.06868:24696 73.02812:137599 73.06432:47595 80.05276:16213 81.06834:12172 83.0851:15285 85.06337:63016 87.04335:313542 87.06254:72873 87.07999:72223 88.04605:17042 89.05896:1561190 90.06191:56459 94.06947:70155 99.07939:11920 101.05931:283891 103.04074:16838 103.07444:528793 104.07732:29033 107.07059:11558 109.07315:26968 115.07378:31105 116.08248:30635 117.05281:20081 117.0909:46183 123.08884:12106 129.08911:44046 131.07088:32080 131.10634:29423 133.08545:521258 134.09018:28042 138.09604:18277 145.08403:25715 147.10149:286550 148.1028:17335 161.11441:33870 177.11285:45030 191.1256:49261 205.14238:11468 | -5.27E-06 |
| POS8528 | Flabellinone | 5.051 | 411.2923 | M+CH3OH+H] | 411.2897 | 57.03287:44003 59.04859:859429 59.05931:20887 60.05226:12857 71.04816:8536 73.02812:24879 73.06432:171605 85.06337:7432 87.04335:100162 87.07999:7724 89.05896:129800 101.05931:40620 103.0385:28408 103.07445:135499 115.07378:9791 117.05281:193132 117.0909:28669 131.06766:88047 133.08545:30173 161.11441:9903 | 6.322E-06 |
| NEG1841 | Tricholomic acid | 1.226 | 159.03804 | [M-H]- | 159.04059 | 55.08413:10747 59.08403:18302 69.11721:6679 73.11618:8054 99.07355:8781 159.03728:13510 | -1.6E-05 |
| POS10348 | Narbomycin | 8.759 | 510.34515 | [M+2H]2+ | 510.3425 | 70.88638:5356 87.14983:6076 108.37923:4924 313.50699:5615 353.19443:5993 | 5.193E-06 |
| POS11608 | Revandchinone 4 | 5.097 | 557.34216 | [M+H]+ | 557.34509 | 57.03287:23495 59.04859:229775 73.02812:38939 73.06432:14431 80.05276:16103 87.04335:946634 88.04783:16418 89.05896:252195 101.05931:259222 103.03849:54702 103.07444:55777 115.07378:17740 117.05281:41938 131.07088:24598 133.08545:89166 147.10149:29268 | -5.26E-06 |
| POS5314 | Russulanorol | 5.197 | 255.15587 | [M+Na]+ | 255.15849 | 57.06988:6670 78.09728:6172 98.81725:5879 149.29048:5845 216.18271:5881 255.16071:8834 | -1.03E-05 |
| POS10624 | cdysone-20,22-m | 6.142 | 521.35156 | [M+H]+ | 521.34882 | 51.77413:6985 56.04951:9219 60.08023:146083 69.06989:7297 71.07258:29433 86.09538:428126 87.09915:50395 95.08474:5568 98.98412:10856 104.10693:69240 105.10875:9331 124.99951:119508 126.00197:6355 184.0746:1123078 185.07629:177466 186.07527:13060 | 5.256E-06 |
| POS7978 | lysylprolyllysine | 4.576 | 372.25784 | [M+H]+ | 372.2605 | 59.04859:448099 69.03294:16725 73.02812:11457 73.06432:9359 85.06337:15701 87.04335:73439 87.07999:7755 89.05896:1144522 90.06191:28135 101.05931:27742 103.07444:127799 105.09028:7020 107.06821:9930 117.05281:19430 129.08911:7743 131.07088:12052 133.08545:401910 134.08684:12480 147.10149:78159 151.09705:8489 177.11285:28023 191.1256:15673 | -7.15E-06 |
| NEG4220 | decyltetrahydroth | 1.413 | 241.20226 | [M-H]- | 241.1996 | 55.8771:6330 70.7528:6757 73.42717:5996 74.81602:5965 82.7948:5707 87.11253:6721 89.07528:10927 161.14594:13424 204.73056:5733 232.00209:6128 | 1.103E-05 |
| NEG2928 | -Phenyl-L-cystein | 3.148 | 196.04639 | [M-H]- | 196.04372 | 99.32591:5903 153.19771:5391 153.42589:6134 161.03622:34233 161.28214:5408 | 1.362E-05 |
| POS13541 | Quinovin | 4.775 | 650.42969 | [M+NH4]+ | 650.42621 | 59.04858:50737 87.04333:31364 89.05894:259203 103.07442:10850 133.08542:111104 147.10147:18945 147.1283:10857 173.38618:22166 191.7171:12443 192.78191:12033 473.44424:10940 | 5.35E-06 |

| Differences in metabolites between the Model group and the Control group | | | | | | | |
|---|---|---|---|---|---|---|---|
| Alignment ID | Metabolite name | Rt(min) | Expreiment Mz | Adduct type | Reference m/z | MS/MS spectrum | PPM |
| NEG2806 | hyl-6-(2-methylb | 5.066 | 191.15807 | [M-H]- | 191.1554 | 57.01118:21492 59.02947:116695 85.13128:6248 87.04276:7689 87.11253:11088 91.05847:6309 101.05437:13474 103.07272:208186 104.07503:7912 111.14092:14668 135.65366:5751 147.117:10377 | 1.397E-05 |
| POS9867 | SCHEMBL1643131 | 5.969 | 488.32437 | [M+H]+ | 488.32169 | 59.04858:23033 64.29315:5737 82.54288:7031 89.05895:11043 94.77686:5356 101.0593:6085 151.42064:6293 | 5.488E-06 |
| POS6820 | Adiphenine | 4.652 | 312.19916 | [M+NH4]+ | 312.19647 | 59.04859:833715 60.05226:67039 73.06432:8571 85.06506:15342 87.04335:65677 88.04605:6379 89.05896:1032050 90.06191:127384 91.06323:7664 101.05931:25769 103.07445:99740 104.07732:13123 107.07059:10487 117.05281:9556 133.08545:147176 134.09018:24780 147.10149:25984 | 8.616E-06 |
| POS9650 | cyclo[10.8.0.02,9.0 | 6.48 | 478.32645 | [M+2H]2+ | 478.32913 | 92.10029:5338 271.37048:5825 308.74551:5511 | -5.6E-06 |
| POS6640 | Salicin | 0.892 | 304.25275 | [M+H]+ | 304.2554 | 110.79853:5377 118.67968:5474 | -8.71E-06 |
| POS3354 | thyl-1-azulenecar | 4.815 | 185.09868 | [M+H]+ | 185.09599 | 53.03854:7116 55.01768:6571 55.05363:8614 57.06894:89617 57.93418:13230 61.01:267803 63.99744:6028 67.05386:23601 75.56093:6006 81.03226:12174 81.06987:10338 85.06333:11562 87.02412:6764 91.05386:6057 95.0489:29208 109.10002:11649 | 1.453E-05 |
| POS11187 | Pro Lys Val Lys Ala | 5.675 | 542.3631 | [M+H]+ | 542.36603 | 57.03284:35229 59.04856:470812 73.02808:47687 73.06429:21024 85.06333:14921 87.0433:71874 89.05891:394650 94.06942:17086 101.05926:67976 103.07439:118733 129.08905:13049 133.08537:92892 147.10141:43828 177.11278:14554 | -5.4E-06 |
| POS14038 | MONENSIN | 5.599 | 688.45917 | [M+2H]2+ | 688.46289 | 59.04859:447999 73.02812:75560 85.06506:27413 87.04335:96532 87.06254:39067 89.05896:532809 101.05931:67741 103.07444:129273 133.08545:188542 147.10149:60605 177.11285:27077 | -5.4E-06 |
| POS9549 | Val Ile Leu Asp | 5.261 | 473.29962 | M+CH3OH+H]2 | 473.29691 | 57.03287:13304 59.04859:167174 67.015:23341 73.02812:6643 81.03072:7557 87.04335:29208 89.05896:170959 101.05931:12395 103.07444:44551 133.08545:57064 147.10149:8679 158.09198:8681 | 5.726E-06 |
| POS9356 | an-2-yl)-10,13-di | 5.677 | 461.32129 | [M+2H]2+ | 461.32401 | 57.03286:34728 59.04858:503341 60.05225:12222 63.82409:6740 73.02811:22106 73.06431:9679 85.06335:6543 87.04333:29484 87.07997:10785 89.05894:144769 90.0619:6752 94.06945:9436 101.05929:31698 103.07442:97789 117.09087:12654 133.08542:26507 147.10147:25154 161.11877:7539 | -5.9E-06 |
| POS10743 | Axillarine C | 6.387 | 525.37146 | [M+H]+ | 525.36859 | 60.08024:31618 70.04918:5882 71.07259:7051 86.09539:95816 87.09917:9695 104.10695:40976 120.02661:5949 124.99953:23066 163.24342:5148 167.61609:5305 184.07463:315921 185.07632:48014 293.36038:6355 | 5.463E-06 |
| POS11994 | bengamide G | 5.561 | 571.39209 | [M+2H]2+ | 571.3952 | 59.04856:119837 87.0433:16278 89.05892:99058 101.05926:20124 103.07439:30982 133.08539:21094 145.95105:6874 146.86797:6536 147.09758:7523 173.43027:15425 280.6557:6597 311.86816:7253 | -5.44E-06 |
| POS11320 | seudotrienic acid | 6.138 | 547.37109 | [M+H]+ | 547.37408 | 69.62679:6764 86.0954:16902 88.90752:6739 104.10696:19850 116.29496:6737 168.67908:6925 184.07465:34999 185.07634:14910 186.07532:14030 318.24524:6938 476.40054:8121 | -5.46E-06 |
| POS8117 | Methoxybutyrfenta | 5.846 | 381.25635 | [M+H]+ | 381.2536 | 55.05366:59197 56.04863:36764 57.06896:87857 58.07272:9338 67.04092:12889 67.05389:116800 68.04951:8904 68.05674:18427 69.06868:54600 70.06431:37694 70.07313:11344 71.08419:35277 79.05313:141014 80.05737:23471 81.05579:16697 81.06834:107621 82.06343:589187 83.06721:102276 83.0851:42505 84.08786:9734 85.10045:12190 91.0539:21022 93.06841:205623 94.07143:43152 95.04894:10832 95.08477:71566 96.08018:105786 97.0831:24824 97.09953:25755 98.98415:8786 107.08487:72153 108.08663:20951 109.10007:29213 110.09557:9670 121.09844:49051 122.10349:12156 123.11524:11544 135.11646:42561 136.11703:15726 149.13002:7634 263.25275:9608 264.26471:164125 265.27322:189890 266.2782:7267 | 7.213E-06 |
| POS8449 | Celerioside E | 4.781 | 405.24515 | [M+Na]+ | 405.24789 | 59.04858:12415 73.02811:5900 87.04333:5505 89.05894:18713 108.92649:5637 133.08542:6137 173.58739:6753 176.6682:5636 216.32614:5671 354.49524:6265 | -6.76E-06 |
| POS9215 | o-teleocidin A1\|JB | 5.548 | 454.30905 | [M+2H]2+ | 454.3063 | 57.03287:18774 59.04859:295508 73.02812:18046 87.04335:17452 89.05896:89480 101.05931:15349 102.5617:5680 103.07444:37998 133.08545:14549 147.10149:7192 158.81593:5572 173.43036:11397 | 6.053E-06 |

| Differences in metabolites between the Model group and the Control group | | | | | | | |
|---|---|---|---|---|---|---|---|
| Alignment ID | Metabolite name | Rt(min) | Expreiment Mz | Adduct type | Reference m/z | MS/MS spectrum | PPM |
| POS13742 | voninin 6\|pavonini | 4.854 | 664.44543 | [M+NH4]+ | 664.44177 | 59.04859:245674 73.02812:21700 85.06506:16253 87.04335:104584 87.07999:14111 87.48628:13562 89.05896:727168 90.06375:20733 101.05931:49600 103.07444:99762 131.06766:14181 133.08545:315046 147.10149:67503 173.38132:15537 177.1078:44899 191.13127:18117 | 5.508E-06 |
| NEG1702 | Antibiotic BN 227 | 1.207 | 154.05396 | [M-H]- | 154.05119 | 67.07545:6313 79.05367:305536 95.03962:13577 97.07645:243799 137.08243:14250 154.05191:25417 | 1.798E-05 |
| NEG3554 | Maybridge4_00094 | 4.314 | 216.12827 | [M-H]- | 216.1255 | 54.79307:5975 62.37378:5944 62.44574:6431 126.02459:83638 127.03272:8735 128.0219:33050 131.66733:5889 | 1.282E-05 |
| POS10096 | o-ethyl)-1H-indol | 5.052 | 499.34589 | [M+H]+ | 499.34311 | 57.03287:86547 59.04859:1682228 60.05226:28452 60.08025:22141 73.02812:27972 73.06432:34939 85.06506:20956 86.09541:22343 87.04335:225468 87.07999:22317 87.09918:10002 89.05896:368289 99.08151:16498 101.05931:141151 101.09641:13981 103.03849:26235 103.07444:394975 104.10697:155502 105.10879:48503 106.11105:12970 115.07378:30682 117.05281:36056 117.0909:66113 131.07088:29743 133.08545:50670 147.10149:88991 161.1188:40566 184.07466:38011 185.07635:29515 186.07535:21842 | 5.567E-06 |
| POS9492 | Simplexin B | 5.947 | 469.31122 | [M+H]+ | 469.314 | 57.03286:13280 59.04857:8582 60.08023:42136 61.08377:5509 86.09539:78112 87.09917:7598 101.05711:5882 104.10695:351710 105.10876:52893 124.99953:13642 158.75143:5535 184.07462:122027 185.07631:24648 215.35905:5728 | -5.92E-06 |
| POS7473 | Piperolein B | 4.464 | 344.22577 | [M+NH4]+ | 344.22299 | 87.04335:17462 89.05896:404679 90.06375:6687 97.43122:6500 133.08545:143591 177.11285:12752 | 8.076E-06 |
| POS9371 | S,14aS)-4b-deoxy | 8.922 | 462.26657 | [M+Na]+ | 462.26379 | 55.05365:11330 67.05389:7267 79.05312:6647 81.0699:21159 85.06337:14778 91.85783:5756 93.06841:17016 95.08477:15604 105.06945:18444 107.08486:28719 109.10006:12051 117.06913:8309 119.08612:16590 121.09843:11999 126.02023:204008 131.08377:6468 133.09863:20057 135.11646:11219 143.08206:5647 145.09904:15259 147.11681:16331 157.10197:13389 159.11723:23023 161.13197:7431 173.43034:8036 189.12691:6211 199.14366:13544 201.16266:6546 208.06328:19880 209.13087:18352 210.6722:5690 213.1633:9412 227.13577:9477 | 6.014E-06 |
| POS9869 | Pro-aMCA | 8.777 | 488.33322 | [M+2H]2+ | 488.336 | 91.88431:5876 149.21219:6338 | -5.69E-06 |
| NEG1138 | nopropyl)amino)e | 8.349 | 133.08339 | [M-H]- | 133.0806 | 59.12987:5186 65.09239:9815 73.30846:5403 74.24288:5666 101.05437:61494 102.54467:6000 116.05923:91887 117.06135:6233 133.08284:6874 | 2.096E-05 |
| POS10148 | p-20(29)-en-3-ox | 4.727 | 501.3248 | [M+H]+ | 501.32199 | 59.04859:428126 73.02812:17540 73.06432:15485 85.06337:15767 87.04335:140079 87.07999:11619 89.05896:1095919 90.06191:26964 101.05931:57188 103.03849:10138 103.07444:128774 104.68195:7630 107.06821:11142 117.05281:7244 129.08911:13689 131.06766:23962 133.08545:371861 134.08684:7068 145.08403:7231 147.10149:74325 173.39111:7151 177.11285:22029 191.1256:8284 | 5.605E-06 |
| POS9870 | ydroxydocosa-4,7, | 4.81 | 488.33981 | [M+NH4]+ | 488.33701 | 57.03286:24280 59.04858:739728 60.05225:10445 73.02811:16689 73.06431:10252 85.06335:13911 87.04333:98766 87.07997:18103 89.05894:805009 90.0619:14342 101.05929:82125 103.03848:10202 103.07442:282660 103.96793:6582 107.07057:7414 117.05279:26650 117.09087:13071 129.0891:12148 131.07086:12641 131.10631:7236 133.08542:227288 145.08401:9229 147.10147:140270 160.1046:6894 161.11438:15337 177.11282:10423 191.12556:21180 | 5.734E-06 |
| POS11210 | Milbemycin A4 | 4.968 | 543.33673 | [M+H]+ | 543.33368 | 57.03287:14949 59.04859:239886 73.06432:11215 87.04335:780433 89.05896:221587 95.14056:9272 101.05931:168653 102.06384:12893 103.03849:121933 103.07444:70587 115.07378:11425 117.05281:73307 131.07088:21478 133.08545:66765 147.10149:23043 | 5.613E-06 |
| POS7672 | s-Cyclosuffrobuxi | 6.034 | 354.2821 | [M+H]+ | 354.2793 | 55.05366:20769 57.06897:29820 59.03105:17005 59.04859:185439 61.01003:7553 73.04689:9508 75.02637:12812 87.04335:29207 89.05897:6684 91.05764:9443 103.07445:209723 131.08055:5746 | 7.903E-06 |

| Differences in metabolites between the Model group and the Control group | | | | | | | |
|---|---|---|---|---|---|---|---|
| Alignment ID | Metabolite name | Rt(min) | Expreiment Mz | Adduct type | Reference m/z | MS/MS spectrum | PPM |
| POS10886 | ;,6R)-3,4,5-trihydr | 4.653 | 530.98499 | [M+H]+ | 530.98798 | 70.42718:6286 72.86754:5890 73.02811:7430 87.04333:17348 89.05894:109585 113.05925:5994 133.08542:36838 173.39598:9305 364.62674:6157 | -5.63E-06 |
| POS10893 | Leu Asp Asn Arg | 4.448 | 531.2915 | [M+2H]2+ | 531.28851 | 72.4194:7773 86.09536:17901 87.05376:24342 89.05891:81013 133.08537:43158 146.06093:8085 188.06995:30052 303.14871:8317 | 5.628E-06 |
| NEG166 | Glycerol | 0.634 | 91.04296 | [M-H]- | 91.0401 | 90.13486:71847 91.13872:16344 | 3.141E-05 |
| POS9268 | Communesin A | 4.548 | 457.2626 | [M+2H]2+ | 457.25977 | 69.03294:7465 73.02811:24149 83.04932:7884 87.04334:40421 89.05896:183975 95.04893:15484 99.04339:10666 111.04189:25470 113.05927:14135 133.08543:49846 155.07083:7130 168.79677:5700 173.42543:5884 | 6.189E-06 |
| POS13291 | Rubuside D | 4.768 | 633.40399 | [M+H]+ | 633.40039 | 59.04859:129463 73.02812:15925 73.06432:14343 87.04335:100421 89.05896:635802 90.06191:15554 101.05931:27993 103.07444:34781 109.3968:10547 129.08911:10945 131.06766:19760 133.08545:258954 146.70763:11522 147.10149:23064 171.92455:11422 177.11285:28269 | 5.684E-06 |
| POS10476 | Delaminomycin B | 5.631 | 516.3349 | [M+2H]2+ | 516.33191 | 57.03379:10046 59.04858:86059 73.02812:6818 87.04334:6345 89.05896:65314 101.0593:11654 103.07444:22657 117.05553:6404 126.02024:28145 133.08545:22148 137.23573:6146 137.4812:5640 147.10149:9745 166.02432:6052 170.09471:5838 319.87314:5954 | 5.791E-06 |
| POS8367 | ihydroxy-4,6-dim | 4.604 | 399.25629 | [M+H]+ | 399.25339 | 59.04859:1010931 73.02812:28224 73.06432:32070 85.06337:29569 87.04335:253725 87.07999:24881 89.05896:3000530 90.06191:61240 101.05931:73680 103.03849:27828 103.07444:237082 107.06821:20239 117.05281:16405 129.08911:20548 131.07088:33861 133.08545:866283 134.08684:26756 147.10149:118198 177.11285:54567 | 7.264E-06 |
| POS9327 | Verdine | 4.611 | 460.30869 | [M+H]+ | 460.30579 | 59.04859:114620 69.03294:12691 73.06432:6779 74.03423:6222 87.04335:50910 89.05896:563675 90.06191:12397 98.87637:6070 101.05931:12596 103.07444:33731 107.07059:8782 117.36894:5773 131.06766:15377 133.08545:233407 134.09018:11356 147.10149:29190 173.43036:12408 177.11285:25475 | 6.3E-06 |
| POS8566 | Metachromin S | 4.905 | 414.30331 | [M+NH4]+ | 414.30042 | 57.03287:50975 59.04859:1296740 60.05226:20845 73.02812:16496 73.06432:173960 85.06506:6581 87.04335:32499 87.07999:13341 89.05896:289834 90.06191:7439 99.07939:11105 101.05931:68599 101.09423:10172 103.07445:356011 104.07732:7444 115.07378:12790 117.05281:405628 117.0909:62243 118.0574:9386 131.06766:159921 132.0724:6890 133.08545:18985 145.08403:11452 147.10149:83947 161.07928:16601 161.11441:45642 173.39111:11217 175.09528:13656 | 6.976E-06 |
| POS12083 | esteryl-Phosphoch | 5.341 | 574.39618 | [M+2H]2+ | 574.39954 | 59.04858:74523 65.13841:8808 87.04334:16033 89.05896:105291 101.0593:19257 103.07444:17919 104.18001:8297 117.97754:7694 133.08545:39980 147.10149:14681 177.11285:9324 | -5.85E-06 |
| POS12413 | 1alpha,25-dimeth | 4.757 | 589.37689 | [2M+H]+ | 589.37341 | 59.04859:207411 73.02812:28959 73.06432:17706 83.31817:11252 87.04335:127032 87.07999:13074 89.05896:909512 90.06191:18586 92.93743:10793 101.05931:55264 103.07444:76697 115.78223:12321 131.07088:21915 133.08545:333416 147.10149:45428 173.43526:12231 177.11285:26196 183.19818:10830 265.44962:12614 358.28094:12755 | 5.905E-06 |
| POS8184 | cosahexaenoyl Gly | 4.669 | 386.27216 | [M+NH4]+ | 386.2692 | 59.04859:166957 87.04335:13037 89.05896:192528 99.55555:6222 101.05931:10841 103.07444:50844 117.05281:11954 133.08545:55275 147.10149:27949 277.32327:6236 | 7.663E-06 |
| POS11846 | Valnemulin | 5.139 | 565.36353 | [M+2H]2+ | 565.36688 | 59.04858:259007 73.02811:50874 73.06431:30974 78.36626:16107 80.05274:24239 87.04333:86028 89.05894:518801 101.05929:33520 103.07442:72740 123.34731:18975 133.08542:191946 147.10147:40567 173.43033:40313 177.11282:31289 194.32018:20204 446.87405:18802 | -5.93E-06 |
| POS15168 | PC(17:1/12-HEPE) | 8.623 | 808.54535 | [M+2H]2+ | 808.54858 | 50.21645:5920 54.8856:6263 57.75751:6358 173.39111:9749 497.72522:5572 | -3.99E-06 |

| Differences in metabolites between the Model group and the Control group | | | | | | | |
|---|---|---|---|---|---|---|---|
| Alignment ID | Metabolite name | Rt(min) | Expreiment Mz | Adduct type | Reference m/z | MS/MS spectrum | PPM |
| POS8515 | Marineosin A | 5.563 | 410.285 | [M+2H]2+ | 410.28201 | 57.03287:49031 59.04859:876259 60.05226:18268 65.04931:7682 67.85582:5822 73.02812:41810 73.06432:19938 85.06506:12180 87.04335:46344 87.06254:8629 87.07999:9658 89.05896:216433 90.06191:9081 94.06947:14511 101.05931:57353 103.07444:141593 105.61979:6301 115.07378:9548 116.08248:7404 117.0909:18444 131.07088:6858 133.08545:42086 147.10149:37057 161.1188:8715 173.39111:10399 185.21703:6740 195.57704:6851 211.2645:5911 | 7.288E-06 |
| NEG2901 | ethyl-1-cyclohexer | 1.277 | 195.17252 | [M-H]- | 195.17551 | 59.08406:6652 75.09884:7763 79.05372:22833 97.07651:682352 159.03737:12622 161.03632:573850 196.04454:63048 | -1.53E-05 |
| POS13957 | horosyloxydocosar | 5.5 | 681.44598 | [M+2H]2+ | 681.44189 | 57.03287:42599 59.04859:477045 73.02812:59066 74.02876:28013 87.04335:110902 89.05896:615241 101.05931:83280 103.07444:203065 133.08545:260993 147.10149:91056 153.90381:33434 200.93614:29460 332.91391:27498 387.02127:27199 551.54889:30427 555.54846:31138 | 6.002E-06 |
| POS11256 | ]-1-oxopropan-2- | 5.527 | 545.33667 | [M+H]+ | 545.33337 | 59.04856:17063 60.08022:9496 69.95734:5940 86.09537:31472 89.05892:17490 104.10693:201760 105.10874:26047 146.98271:20803 191.11984:5359 319.55377:6838 | 6.051E-06 |
| NEG2410 | ylsulfanyl-butyl)-t | 1.209 | 177.04959 | [M-H]- | 177.0526 | 79.05367:6822 119.17749:7333 142.03583:7854 | -1.7E-05 |
| POS6925 | ,6-Dimethyl-2,5-h | 4.716 | 317.15616 | [M+Na]+ | 317.15921 | 58.04042:9487 59.04859:41583 73.02812:39470 80.0543:10378 89.05896:57821 133.08545:13578 173.43036:13731 173.4745:5458 176.86003:5563 207.79285:5325 | -9.62E-06 |
| POS11727 | side, 3,17-dihydro | 6.137 | 561.33856 | [M+H]+ | 561.34198 | 59.04858:6808 71.33195:6263 89.05895:8051 98.42394:5281 104.10696:5405 539.50934:6287 | -6.09E-06 |
| POS13108 | Antibiotic M 230B | 5.663 | 622.42749 | [M+H]+ | 622.43127 | 59.04856:123729 87.04331:19210 89.05893:108340 101.05927:22135 103.0744:32797 115.92144:9283 133.08539:26790 147.10143:9544 | -6.07E-06 |
| POS7737 | Songorine | 4.504 | 358.24142 | [M+NH4]+ | 358.23837 | 59.04859:187338 73.06432:14123 87.04335:81153 89.05896:1400826 90.06191:26297 101.05931:5973 103.07444:37700 107.07059:15584 129.08911:7096 131.07088:12867 133.08545:486213 134.09018:12227 147.10149:31980 173.39603:8242 177.1078:51863 | 8.514E-06 |
| POS12976 | Arg Leu Lys Trp | 5.233 | 616.39667 | [M+2H]2+ | 616.39288 | 57.03287:32646 59.04859:466780 73.02812:84005 73.06432:23669 80.05276:22272 85.06337:22002 87.04335:305077 87.07999:25052 88.04783:31059 89.05896:844076 90.06374:38897 101.05931:189509 103.07444:144583 117.0909:21604 133.08545:324054 134.08684:20407 145.08778:22085 147.10149:57823 173.43034:32907 175.88412:21732 177.11285:45332 191.1256:28014 424.63242:19913 | 6.149E-06 |
| POS7780 | Lavoltidine | 5.053 | 360.24237 | [M+2H]2+ | 360.23929 | 57.03287:60582 58.04137:15193 59.04859:1675242 60.05226:41959 65.04819:49136 69.06868:12856 71.04945:7803 73.02812:166575 73.06432:21791 80.0543:17844 85.06337:32565 87.04335:150591 87.06079:69315 87.07999:19379 89.05896:1115052 90.06191:37919 94.06947:40386 101.05931:95564 102.06606:9818 103.07444:234132 104.07732:12819 105.09259:7544 107.07059:9096 109.07315:31309 116.08248:19999 117.0909:14133 129.08911:19810 131.08699:10183 133.08545:285434 147.10149:86939 177.11285:15536 191.1256:13700 | 8.55E-06 |
| POS11259 | spicatin | 4.74 | 545.35059 | [M+H]+ | 545.34723 | 59.04859:622712 73.02812:57318 73.06432:44968 85.06506:20813 87.04335:345886 87.07999:33732 89.05896:2375766 89.07882:41091 90.06191:41952 101.05931:115064 103.07444:226217 107.07059:34198 129.08911:32429 130.08708:18221 131.07088:54070 133.08545:1103812 134.09018:32979 147.10149:182907 173.43526:23820 177.11285:122377 191.1256:26938 | 6.161E-06 |
| POS12732 | ı-D-glucopyranos | 5.051 | 604.42444 | [M+H]+ | 604.42072 | 57.03287:33024 59.04859:715256 73.02812:24066 73.06432:21369 87.04335:79713 89.05896:540829 90.06191:24670 101.05931:93361 103.07444:301644 106.69563:20633 117.05281:69426 117.07185:24236 117.0909:29699 131.06766:39433 131.10634:25272 133.08545:178801 147.10149:147013 147.56622:18270 161.11441:37347 173.43034:49754 183.02257:18996 342.37314:23317 343.1636:21411 | 6.155E-06 |

**Differences in metabolites between the Model group and the Control group**

| Alignment ID | Metabolite name | Rt(min) | Expreiment Mz | Adduct type | Reference m/z | MS/MS spectrum | PPM |
|---|---|---|---|---|---|---|---|
| POS9216 | )-Deoxymethymyc | 5.52 | 454.31253 | [M+2H]2+ | 454.31561 | 57.03287:81565 59.04859:1490384 60.05226:42113 65.04819:17652 69.06992:15052 73.02812:92610 73.06432:32519 80.0543:7485 83.0851:9630 85.06337:28999 87.04335:111027 87.06079:23074 87.07999:26781 89.05896:621674 90.06191:21255 94.06947:40236 99.07939:11675 101.05931:127551 103.07444:278122 104.07732:11744 115.07378:21818 117.05553:9391 117.0909:23259 129.08911:15064 131.06766:18057 131.10312:8823 133.08545:152206 145.08403:8057 147.10149:98787 161.11441:19346 173.43034:8036 177.1078:10809 191.1256:10621 | -6.78E-06 |
| POS13580 | opyranosyl-6'-pim | 5.044 | 653.40875 | [M+H]+ | 653.40472 | 57.87836:7624 74.77715:7412 173.43031:12962 565.78345:6817 | 6.168E-06 |
| POS10665 | Azadironol | 4.732 | 523.29987 | [M+H]+ | 523.3031 | 60.08022:20622 69.03291:6328 81.09808:5146 86.09536:34227 89.05891:9720 104.10692:177748 124.9995:9959 173.43027:11599 184.06921:65670 293.67346:5616 | -6.17E-06 |
| POS13219 | baeckein F | 8.78 | 629.09644 | [M+H]+ | 629.09253 | 67.19205:6851 69.94729:7347 95.36812:6350 100.12971:5204 282.84961:6618 | 6.215E-06 |
| POS1957 | Iodomethane | 1.23 | 142.9382 | [M+H]+ | 142.9351 | 55.01682:24101 55.05365:43290 56.94231:9903 57.06895:7810 64.52962:5774 68.04829:7093 69.03294:65214 70.06555:11197 70.07311:6937 71.04815:20688 83.04768:6460 97.02763:12463 98.05796:6868 143.03427:91947 | 2.169E-05 |
| NEG4317 | imycin: Oxazinom | 1.208 | 244.04961 | [M-H]- | 244.0464 | 79.05367:6909 97.07645:11114 163.03734:11778 175.2373:5440 | 1.315E-05 |
| NEG635 | Isocaproic acid | 5.528 | 115.07261 | [M-H2O-H]- | 115.0757 | 59.08403:7579 69.07899:6232 98.04861:26594 100.04587:57877 103.87704:5463 115.07258:24219 116.05923:61702 | -2.69E-05 |
| NEG5018 | -)-Quinolactacin A | 0.985 | 269.12595 | [M-H2O-H]- | 269.12909 | 59.08403:22810 89.13125:479299 90.13486:14166 93.03925:1773201 95.03763:1125557 151.06712:48152 153.06755:73754 | -1.17E-05 |
| POS11669 | Cholyllysine | 4.829 | 559.36816 | [M+H]+ | 559.3717 | 57.03287:53343 59.04859:1266598 73.02812:47890 73.06432:36520 83.0477:18243 85.06337:49893 87.04335:307887 87.07999:49722 89.05896:2215204 90.06191:44290 101.05931:198639 103.07444:401994 107.07059:17771 117.05281:25782 129.08911:34704 131.06766:55594 133.08545:772419 134.08684:22090 145.08403:25622 147.10149:237818 164.1385:16358 173.43036:39536 177.1078:47093 191.1256:39333 372.55954:16853 | -6.33E-06 |
| NEG1145 | 3-Trimethoxyprop | 8.741 | 133.08394 | [M-H]- | 133.0871 | 55.03414:5771 56.21574:6244 65.09239:15039 67.10966:7483 79.05367:8124 91.13873:7797 100.04587:14211 101.05437:131678 102.05606:12560 109.54619:6082 116.05923:190376 117.06135:15043 133.08284:8208 | -2.37E-05 |
| POS10994 | )10-natafuranamin | 5.346 | 535.35645 | [2M+H]2+ | 535.35303 | 57.03287:58664 59.04859:1185472 60.05226:28537 69.06992:18596 73.02812:105121 73.06432:35161 85.06337:35816 87.04335:220489 87.06254:56060 87.07999:54007 89.05896:1072132 90.06191:31375 94.06947:34509 101.05931:145346 103.03849:19681 103.07444:333188 104.07732:15184 109.0756:17790 117.0909:30649 129.08911:23306 131.06766:17923 133.08545:354329 134.08684:24095 143.10413:15855 147.10149:166337 173.38622:19003 177.11285:38861 191.1256:25034 314.09201:16848 | 6.388E-06 |
| POS10449 | 11-Ketofusidic acid | 4.823 | 515.34082 | [M+H]+ | 515.33752 | 57.03287:49450 59.04859:1449929 60.05226:28917 73.02812:46166 73.06432:39569 83.0477:12608 85.06337:38266 87.04335:237243 87.07999:34396 89.05896:1809246 90.06191:40554 101.05931:169273 103.03849:32397 103.07444:433003 104.07732:12532 115.07378:14554 117.05281:30054 117.0909:22782 129.08911:30253 131.07088:40680 133.08545:538518 134.08684:22924 145.08403:18900 147.10149:231683 161.11441:17324 177.11285:35046 191.1256:24259 338.48813:11314 | 6.404E-06 |
| POS12665 | orodecane sulfoni | 7.882 | 600.93445 | [M+H]+ | 600.93829 | 68.48682:5486 90.24759:6837 173.43526:12346 293.1124:5001 369.65771:5913 554.7619:6490 | -6.39E-06 |
| POS10097 | o-ethyl)-1H-indol | 5.375 | 499.34631 | [M+H]2+ | 499.34311 | 57.06989:6828 59.04859:39692 60.08025:63374 73.02812:7840 86.09541:121291 87.04335:54077 87.09918:40902 89.05896:41583 101.05931:12329 103.07445:11368 104.10697:595170 105.10879:221811 106.11105:73269 110.11542:7478 124.99956:26496 127.00428:7786 131.83476:7331 133.08545:14332 184.07466:188160 185.07635:108954 186.07535:100589 187.07695:9257 318.11121:7126 | 6.408E-06 |
| POS11690 | Chrysopentamine | 6.214 | 560.29828 | [M+2H]2+ | 560.30188 | 84.90689:5295 104.10469:17228 133.38271:6134 171.91002:5442 405.91846:5953 | -6.43E-06 |

| Differences in metabolites between the Model group and the Control group | | | | | | | |
|---|---|---|---|---|---|---|---|
| Alignment ID | Metabolite name | Rt(min) | Expreiment Mz | Adduct type | Reference m/z | MS/MS spectrum | PPM |
| POS11664 | Sepaconitine | 6.214 | 559.29767 | [M+2H]2+ | 559.30127 | 50.40268:8965 52.1749:6583 53.81686:5946 68.18956:5778 104.10696:18576 224.66388:6301 240.11317:5511 245.61572:5266 266.03574:5919 502.1665:7462 | -6.44E-06 |
| POS3633 | Vitispirane | 5.393 | 193.16249 | [M+H]+ | 193.15924 | 55.05365:9474 58.06512:6990 60.2657:5893 67.05389:17243 79.05312:6183 81.06834:19681 93.07034:7061 95.08477:11928 105.06945:11578 109.10007:9376 147.11681:15425 147.72034:5289 | 1.683E-05 |
| POS13035 | hydroxyl acyl DFC | 5.428 | 619.39294 | [M+2H]2+ | 619.39697 | 59.04859:85033 81.03072:14718 87.04335:23911 89.05896:107737 103.07445:20833 109.73177:12931 133.08545:35925 173.39111:25291 | -6.51E-06 |
| POS12658 | yl)-deoxysphing-4 | 6.837 | 600.46539 | [M+H]+ | 600.46149 | 55.53491:6757 83.89444:5594 89.05896:6132 199.65767:5901 203.06952:5435 | 6.495E-06 |
| POS5740 | Diuron | 0.883 | 270.97675 | M+CH3OH+H] | 270.98001 | 57.06986:13553 71.08544:8173 79.61764:5963 135.00177:139311 263.71185:6056 | -1.2E-05 |
| POS5741 | Diuron | 1.025 | 270.97675 | [M+Na]+ | 270.98001 | 62.98163:22976 74.09579:5637 76.16116:5345 80.94772:29281 82.94373:6478 90.97554:17180 96.92096:10587 104.99081:108276 116.99022:15700 135.00177:368785 136.00449:6799 140.06859:13752 | -1.2E-05 |
| NEG3854 | Axinynitrile A | 2.089 | 228.17908 | [M-H]- | 228.1758 | 79.05367:26955 84.02768:5510 97.08672:9662 224.09412:6638 | 1.437E-05 |
| POS5588 | Pentachloropheno | 0.958 | 264.85098 | M+CH3OH+H] | 264.85428 | 50.29755:6809 62.46424:5260 77.06998:5493 80.94775:1146872 81.04482:13340 82.94376:26061 83.67702:6314 84.91025:6728 90.97557:17328 104.10696:13956 122.07161:108671 138.90465:7942 144.04625:26688 | -1.25E-05 |
| POS12412 | rmectin B2b aglyc | 4.601 | 589.341 | [2M+H]2+ | 589.3371 | 69.03294:85645 73.02811:270464 79.66347:74724 81.03229:100442 81.9725:68091 87.04334:605848 89.05896:3143008 90.0619:150195 95.04893:184828 99.04339:77337 111.04189:266398 113.05927:86412 133.08543:931530 137.0598:126393 155.07083:95403 155.12476:64704 199.10141:68832 | 6.618E-06 |
| POS10671 | ydrokarounidiol di | 6.136 | 523.38159 | [M+H]+ | 523.37811 | 53.00208:11481 55.05365:13411 57.03287:1944042 58.03662:30775 59.04858:574645 69.06868:19389 81.06833:58211 86.0954:20230 97.06461:30371 99.07938:156839 101.09422:33453 104.10696:110452 115.07378:141694 117.09089:61504 157.12312:161664 184.06929:48328 | 6.649E-06 |
| POS8125 | obutyryl bakkenoli | 5.055 | 382.25488 | [M+H]2+ | 382.25821 | 57.03287:59844 58.04137:17722 59.04859:1631943 59.05931:39530 60.05226:41665 65.04819:48386 69.06868:14164 71.04816:8664 73.02812:166879 73.06432:24983 74.03149:8137 80.0543:25562 85.06337:41089 87.04335:187063 87.06079:65798 87.07999:23344 87.56366:8138 89.05896:1267394 90.06191:33992 94.06947:29465 101.05931:98242 102.06606:12479 103.03849:10363 103.07444:233574 104.07732:12470 107.07059:12391 109.07315:29079 116.08248:14366 117.0909:17535 129.08911:21471 131.06766:19420 131.10634:10398 133.08545:342321 134.08684:11035 147.10149:90922 177.11285:26646 191.1256:12000 | -8.71E-06 |
| POS9032 | Yibeissine | 4.8 | 444.31442 | [M+NH4]+ | 444.3111 | 57.03286:35610 59.04858:1024461 59.0593:26431 60.05225:16286 73.02811:12469 73.06431:18139 83.04768:7020 85.06504:16909 87.04333:86153 87.07997:17599 89.05894:779869 90.0619:15131 99.07938:6169 101.05929:79470 103.03848:14076 103.07442:363566 105.09026:8277 115.07376:6370 117.05279:68029 117.09087:20251 129.09224:7563 131.07086:11202 131.10631:6301 133.08542:179811 145.08401:9892 147.10147:178089 161.11438:20425 173.396:8609 191.12556:18245 205.14235:7523 | 7.472E-06 |
| POS9286 | adecahydro-3,5-d | 5.395 | 458.29333 | [M+2H]2+ | 458.29001 | 57.03379:7869 59.04859:141324 67.015:16456 73.06432:13343 81.02916:6543 87.04335:13792 89.05896:83653 101.05931:11880 103.07445:29819 117.05281:18938 131.06766:11334 133.08545:21246 147.10149:13494 158.092:5847 191.1256:6630 439.38196:6118 | 7.244E-06 |
| POS9287 | adecahydro-3,5-d | 4.85 | 458.29333 | [M+NH4]+ | 458.29001 | 59.04859:127912 62.9291:6736 87.04335:275102 89.05896:95178 101.05931:69613 103.0385:62724 103.07445:31513 110.84615:5616 117.05281:34988 131.07088:8119 133.08545:33611 136.56493:5515 147.10149:8298 180.89824:5683 | 7.244E-06 |
| POS15209 | Ganosinensin C | 6.569 | 815.55103 | [M+2H]2+ | 815.54559 | 52.05935:5830 397.22595:5531 610.95099:6742 | 6.67E-06 |

**Differences in metabolites between the Model group and the WJW group**

| Alignment ID | Metabolite name | Rt(min) | Expreiment Mz | Adduct type | Reference m/z | MS/MS spectrum | PPM |
|---|---|---|---|---|---|---|---|
| POS3961 | L-Tryptophan | 4.419 | 205.09682 | [M+H]+ | 205.09718 | 55.0177:28497 74.02328:473445 91.0539:335248 103.05421:28178 105.06946:27738 115.05257:692070 117.05825:161407 117.06913:685825 118.0629:11985257 118.43848:28942 119.06659:379179 130.06477:708654 131.07088:44960 132.07892:2374151 133.08215:83645 142.06567:905584 143.07104:1194480 144.06113:83329 144.0797:4341700 145.06152:115992 146.05721:17431700 146.54747:29480 146.57033:23767 147.06317:678492 155.0584:63157 159.09137:1192681 160.07417:256210 170.05661:862231 171.0612:37257 188.07004:2923840 189.07106:109007 | -1.75527E-06 |
| POS3448 | lquinolinium-2-ca | 4.418 | 188.07056 | [M+H]+ | 188.0705 | 91.0539:698427 103.05421:33981 115.05257:797674 116.06099:47668 117.06913:479114 118.0629:8225772 119.06659:228830 128.04967:38424 132.07892:89223 142.06566:594714 143.07103:1165095 144.07968:1851898 145.06526:103181 146.05721:5633082 147.06317:168062 155.0584:95603 160.07417:64165 170.06137:422132 188.07004:287089 | 3.19029E-07 |
| POS1294 | Niacinamide | 1.644 | 123.05486 | [M+H]+ | 123.05528 | 51.02314:10542 53.03856:116635 56.04952:6667 61.03869:8044 67.05389:11350 68.0495:16272 77.03802:8107 77.45795:6885 78.03359:75791 79.05312:6839 80.04813:999731 81.06989:6655 95.04893:65126 96.04379:265895 105.04401:14387 106.02892:33624 123.05364:805984 124.03867:21063 | -3.4131E-06 |
| POS5416 | ycerophosphochol | 1.149 | 258.10968 | [M+H]+ | 258.11008 | 60.08025:65541 71.07261:10895 74.4781:5594 86.09541:106029 98.98415:7132 104.10697:588962 105.10879:17453 124.99956:95822 184.06931:20027 | -1.54973E-06 |
| POS9985 | LysoPC(16:1/0:0) | 6.086 | 494.32361 | [M+H]+ | 494.32468 | 59.04858:17369 60.08025:150052 69.06991:6945 71.0726:15452 73.02811:8417 86.0954:400958 87.09917:8035 89.05895:17857 95.90849:7302 98.98414:8216 101.0593:7508 104.10696:89747 124.99955:112934 184.07465:1936991 185.07634:52290 | -2.16457E-06 |
| POS1867 | Betaine | 1.004 | 140.06779 | [M+Na]+ | 140.06822 | 52.9996:41083 58.06512:19829 74.09583:3209492 75.099:20457 80.94776:159718 80.99314:24787 81.05422:29478 82.06183:23694 82.94377:114697 96.07816:16111 114.08864:279681 140.06865:1283927 | -3.06993E-06 |
| POS2506 | -Naphthalenediam | 4.419 | 159.09103 | [M+H]+ | 159.0916 | 55.05366:12243 55.93345:53946 56.04953:12704 60.32402:6182 67.05389:16534 69.06992:16412 70.06431:17375 71.04816:7946 72.93573:18955 84.07957:6821 91.0539:13916 95.08477:25721 100.07598:7385 105.06946:21656 112.11003:13647 114.97046:14039 115.05257:162222 116.05025:10474 117.05553:243885 118.0629:69762 130.06477:220108 131.06766:12297 132.07892:561926 133.08215:11112 140.0473:10653 142.06567:92787 158.08345:12692 159.09138:178121 160.07417:53064 | -3.58284E-06 |
| POS3313 | Carnitine | 0.991 | 184.09396 | [M-H+Na]+ | 184.09399 | 52.47893:5868 68.05794:5483 102.09042:10203 125.02055:275585 184.0961:18894 | -1.6296E-07 |
| POS15000 | PC(16:0/18:1(9Z)) | 5.983 | 782.56464 | [M+Na]+ | 782.56702 | 60.08023:54684 64.37959:20354 86.09538:167105 104.10693:27097 124.99951:56726 170.22337:18464 184.0746:721530 185.07629:25982 560.38855:21848 | -3.04127E-06 |
| POS2076 | ble-3-carboxaldeh | 4.428 | 146.06004 | [M+H]+ | 146.0601 | 53.03774:51781 55.0177:49959 55.05366:156692 57.03287:28205 57.06896:17716 58.06512:8631 59.04859:7799 65.03805:10573 71.04816:83011 72.08048:15333 75.02498:8273 76.03859:10560 81.0323:22008 82.06503:9116 83.0851:53324 85.028:10182 86.05939:18263 86.09541:14054 91.0539:647726 99.0434:103394 100.04588:11014 100.07383:16352 100.11039:168880 112.95607:7029 117.05553:126525 118.0629:1226964 119.06659:13999 127.0381:7106 145.08403:18572 146.05721:81042 146.9101:6684 | -4.1079E-07 |
| POS10532 | PC(18:3(6Z,9Z,12Z | 5.974 | 518.32239 | [M+H-H2O]+ | 518.32465 | 57.03287:49122 58.06512:52567 59.04859:118975 60.08025:521029 67.05389:32627 71.07261:70802 73.02812:24968 81.06834:41715 86.09541:1216161 87.04335:37495 89.05896:107388 93.06841:43871 101.05931:25951 103.07444:21465 104.10697:6361878 105.10879:177286 121.1013:29052 124.99956:332870 133.08545:28478 173.39111:28858 184.07466:4098194 185.07635:90381 258.10977:33231 | -4.3602E-06 |
| POS11290 | PC(20:3(8Z,11Z,14Z | 6.473 | 546.35565 | [M+Na]+ | 546.35596 | 50.35427:5837 60.08022:15840 62.15261:4989 86.09536:55689 104.10692:20699 107.79275:5589 124.9995:12059 146.98271:13095 184.07457:167098 185.07626:7511 343.73755:6496 | -5.67396E-07 |
| POS5423 | N-Lauroylglycine | 6.021 | 258.20578 | [M+H]+ | 258.2063 | 55.05365:50283 57.06895:494066 58.07271:13376 67.05389:71873 69.06867:15380 71.08546:161806 76.03858:780549 77.04237:11860 81.06833:49184 83.08508:35643 85.10044:94916 95.08476:128147 97.10158:9774 109.10005:74838 123.11523:11927 173.44014:7303 183.17154:17097 | -2.01389E-06 |

**Differences in metabolites between the Model group and the WJW group**

| Alignment ID | Metabolite name | Rt(min) | Expreiment Mz | Adduct type | Reference m/z | MS/MS spectrum | PPM |
|---|---|---|---|---|---|---|---|
| POS2545 | ndoleacetaldehyd | 4.419 | 160.07535 | [M+H]+ | 160.07568 | 53.03275:7156 55.05364:23287 62.32769:5960 69.0699:10742 72.08046:9774 91.05388:14019 91.74825:5456 104.08642:6121 105.06712:6511 105.79249:5560 113.11348:5554 114.12788:11061 115.05254:31254 115.66189:6374 117.0555:42038 118.06287:86211 130.06474:31119 131.07085:7940 132.07889:76863 133.08542:11895 142.06563:6600 159.09134:9600 160.07414:10775 | -2.06152E-06 |
| POS11182 | 0:5(5Z,8Z,11Z,14Z | 5.919 | 542.32269 | [M+H-H2O]+ | 542.32465 | 57.03287:45498 59.04858:141114 60.08025:271303 70.5925:37166 86.09541:703530 89.05896:111950 102.30787:38952 104.10697:3619913 105.10879:72451 111.75905:37431 124.99956:188500 181.02895:41554 184.07466:2838886 185.07635:65489 207.51649:42599 528.25073:34308 | -3.61407E-06 |
| POS14781 | C(16:0/18:2(9Z,12Z | 8.803 | 758.56696 | [M+H]+ | 758.5694 | 52.5312:81083 60.08022:297747 70.51228:83223 86.09536:1030962 104.10464:110452 124.99949:267361 184.07457:4859117 185.07626:102746 240.23297:89261 | -3.21658E-06 |
| POS2847 | Indoleacrylic acid | 4.418 | 170.05942 | [M+H-H2O]+ | 170.06004 | 50.93715:6072 55.0177:6911 55.05365:19589 55.93345:27101 67.05389:12955 68.04829:6388 69.06868:8131 72.04369:11242 79.05463:9584 81.06989:11015 106.06411:6356 107.08485:7663 114.97045:25386 115.05257:138336 128.95062:7462 142.06203:38507 146.95981:6089 170.05659:71694 | -3.64577E-06 |
| POS1537 | Indole-3-carbinol | 4.423 | 130.06456 | [M+H-H2O]+ | 130.0647 | 54.64017:6537 55.05366:26758 56.04863:30718 57.03287:8398 57.06896:80411 67.05389:15137 74.09583:23880 77.03802:23987 84.04317:38555 84.07957:69391 87.00325:14012 95.04894:33089 103.05422:81566 104.46147:6462 105.04402:6683 107.84089:5992 113.96312:8532 128.04967:9327 129.99472:6496 130.06477:306382 130.15721:27895 132.94377:5972 | -1.07639E-06 |
| POS2015 | METHYLQUINOLI | 4.419 | 144.08061 | [M+H]+ | 144.0808 | 52.60244:6370 53.03774:28367 55.0177:33930 55.05366:27428 56.04863:16803 57.03287:18875 57.06989:8537 63.06548:5772 69.03294:31338 70.06431:164053 71.04816:39263 77.03802:7577 79.05313:6114 81.0323:11294 81.0699:16849 84.04482:7076 89.03731:7724 91.0539:29775 91.36633:5304 95.04894:11468 98.06007:87031 98.09554:47486 99.0434:52100 103.05422:41978 115.05257:81645 116.04757:10636 117.06913:139579 118.06566:6036 126.09015:33512 128.04655:8832 142.06567:6482 143.0343:41579 143.07104:94184 144.0797:393521 145.06528:22824 | -1.3187E-06 |
| POS1619 | 6-Methyl indole | 4.422 | 132.08081 | [M+H]+ | 132.0809 | 58.06512:12753 58.07272:6489 69.03294:22883 69.06992:48831 77.03802:12116 79.05313:14901 86.05939:13680 86.09541:283147 88.00349:10381 91.0539:15793 93.05684:11005 95.04894:24133 97.00919:8706 97.77084:5883 103.05422:70909 105.06946:50995 106.06412:5359 115.05257:103497 117.05553:226174 130.06477:29594 131.07088:29487 132.07892:233975 | -6.81401E-07 |
| POS6248 | Testosterone | 5.518 | 289.21558 | [M+H]+ | 289.21619 | 55.05363:9679 67.05386:9397 69.06989:12175 79.05309:7681 81.06986:10555 83.04929:11954 84.03651:6250 86.09537:22617 93.06837:6272 97.06456:156759 98.06837:6410 104.10692:12893 105.0694:9938 109.06331:108771 110.33407:6009 123.07998:6579 130.99672:6339 261.53647:5972 | -2.10915E-06 |
| POS2409 | ethanolaminium p | 0.991 | 156.04137 | [M+H]+ | 156.0424 | 74.09583:71991 82.05225:11145 83.05907:63042 93.04334:39455 95.05889:27291 96.9661:7112 105.7131:5254 110.07076:223594 112.05141:10357 114.78535:5385 130.06158:42132 146.88715:6278 156.04167:102805 | -6.60077E-06 |
| POS10531 | nitoylphosphatidyl | 8.79 | 518.31934 | [M+Na]+ | 518.32172 | 56.04953:8669 57.03287:23127 57.06896:76608 58.06512:6933 60.08025:35811 67.05389:11238 69.06992:16160 71.0726:25952 71.08547:60934 81.0699:18773 83.08509:27941 85.10045:39284 86.09541:370488 87.09918:9280 95.08477:28970 97.09953:9473 103.07444:6091 104.10696:1537859 105.10879:43020 109.10006:22270 123.11523:10211 126.02023:9904 146.97894:429330 164.99086:6845 260.09827:7101 313.26874:12736 337.16791:6369 | -4.59174E-06 |
| POS11971 | 2:5(4Z,7Z,10Z,13Z | 6.306 | 570.35352 | [2M+H]+ | 570.35596 | 58.06512:26083 60.08025:237833 71.07261:29532 86.09541:566328 91.0539:27831 104.10697:2834781 105.10879:77117 124.99956:129786 184.07466:1870368 185.07635:54585 220.16205:20271 | -4.27803E-06 |
| POS9758 | yl)oxolan-2-yl]oxy | 4.813 | 483.18228 | [M+Na]+ | 483.1824 | 55.71933:7792 67.26189:6694 68.4661:6352 76.087:7454 81.38747:8313 95.49025:8769 132.05931:6142 142.10197:5732 162.06279:45742 189.08772:593454 190.09126:33628 207.09973:27956 230.1127:6781 253.08136:356252 254.08736:22405 | -2.48353E-07 |

| Differences in metabolites between the Model group and the WJW group | | | | | | | |
|---|---|---|---|---|---|---|---|
| Alignment ID | Metabolite name | Rt(min) | Expreiment Mz | Adduct type | Reference m/z | MS/MS spectrum | PPM |
| POS6547 | Salidroside | 4.947 | 301.12735 | [M+Na]+ | 301.12729 | 51.83899:6899 55.01768:66130 57.06894:396680 71.01213:84376 81.06987:12902 83.01192:42239 83.04766:8314 85.06333:185974 86.06792:5770 109.06332:17662 111.04185:16356 113.02309:321047 114.0258:7122 115.03928:25477 125.02351:16579 127.07495:203994 128.07762:7579 139.0383:13773 139.07704:9267 159.09993:459001 160.10455:22396 167.035:13795 167.10457:8249 171.09958:19797 173.38614:7900 | 1.99251E-07 |
| POS6948 | Leu-Ala-Asp | 4.4 | 318.16559 | [M+H-H2O]+ | 318.1655 | 56.04953:7642 60.04426:6621 60.60372:5468 69.04404:8689 84.07958:126264 85.06337:9106 85.0836:6364 86.09541:165517 87.09918:7101 89.96288:5351 91.0539:82896 99.62812:5429 110.07076:109186 110.98161:5239 111.07458:7410 119.04707:94549 120.05206:8496 120.08031:19854 129.10172:102908 130.1062:8360 132.10175:17803 136.07611:178726 137.07704:14016 147.04404:23887 320.41443:6412 | 2.82872E-07 |
| POS9565 | droxy-5-[(3Z)-5-h | 4.513 | 474.28506 | [M+2H]2+ | 474.28503 | 62.01709:7800 67.75507:8760 70.06428:9745 74.05883:14795 84.04313:19873 86.09538:115987 87.09915:8524 97.10977:7069 101.06799:8509 110.30173:7219 115.08699:8192 120.08026:150606 121.08408:12895 132.10168:25525 161.89456:9129 173.39105:17718 238.93481:8120 | 6.32531E-08 |
| POS5483 | 8-Deoxy-lactucin | 4.64 | 261.11411 | [M+H]+ | 261.11401 | 51.75893:6842 53.03854:10056 55.01768:12436 55.05363:7024 57.03377:15202 57.06894:491758 58.07269:11803 59.04856:6527 61.01:37998 67.05386:8968 69.06989:22516 71.01213:14176 79.05309:11797 81.03226:150374 81.06987:15602 83.04766:31896 85.06333:227721 95.0111:8694 97.06457:9527 107.04913:7432 111.04436:49075 125.05955:159609 127.07495:17739 133.0656:113285 137.09422:7017 167.10457:710584 168.11116:45533 179.10365:8088 185.0979:14064 195.10202:11404 197.1132:11825 213.09639:9178 231.10547:13816 | 3.82974E-07 |
| NEG4912 | Hydroprene | 1.052 | 265.21741 | [M-H]- | 265.21741 | 54.30708:6028 59.08406:7086 89.13129:326629 90.13491:13540 93.0393:178610 95.03768:354553 97.0375:16669 121.99403:5086 147.15923:83595 149.16045:35272 154.19572:6567 169.77672:5477 220.4525:5858 | 0 |
| POS6609 | Cumyluron | 4.77 | 303.12589 | [M+H]+ | 303.12589 | 57.06894:245511 58.07269:8180 59.58425:5492 61.01:39462 81.03226:18130 81.06987:16570 85.06333:115257 95.0489:5669 109.06332:9289 111.04185:6901 113.05923:26071 125.05955:13986 127.07495:10276 133.0656:13185 137.05975:93128 167.10457:94935 221.1192:18987 | 0 |
| POS4246 | Atrazine | 4.819 | 215.08989 | [2M+H]+ | 215.09 | 50.42034:6599 55.05363:9548 57.06894:113538 58.07269:33225 61.01:8257 63.00633:9057 70.39037:6693 81.03226:25529 82.03626:7456 85.06333:15536 | -5.11414E-07 |
| POS7530 | -2-ylthio)-N-(4-r | 0.938 | 348.0777 | M+CH3OH+H] | 348.07773 | 80.94776:10779 86.09541:34363 96.92101:111833 104.10697:163656 104.99086:7337 221.01341:33715 | -8.61876E-08 |
| POS9145 | Arnamiol | 4.456 | 451.18823 | [M+H]+ | 451.18811 | 55.0168:14414 55.05363:23165 57.03377:7112 59.04856:76132 61.01:9500 67.05386:6581 75.02494:11045 79.05309:8651 81.03226:82662 81.0683:8136 83.04766:73302 85.02796:17255 91.02029:179714 95.0489:37929 97.06457:14619 110.53846:6260 123.04187:33697 125.05955:827176 126.06273:33212 127.03804:14552 129.03552:11116 143.0526:34766 146.05714:18834 165.09097:73302 171.04672:35155 183.10228:34063 188.06996:43084 189.05423:202614 190.0575:11211 247.10213:10098 379.40112:6607 | 2.65964E-07 |
| POS6179 | Abacavir | 4.402 | 287.15973 | [M+2H]2+ | 287.15961 | 65.06059:7534 67.51974:6101 70.06431:642851 71.06746:23044 71.16019:5951 79.5765:6169 84.07957:14715 93.07034:8096 97.07488:6848 98.06007:16757 103.05197:23197 112.08709:52986 113.58521:38696 115.08704:10591 120.08031:498348 121.08413:37672 124.08617:13476 126.05367:11642 127.08732:15640 140.07935:15476 151.12099:15033 153.10732:7985 155.07913:10428 157.10622:19128 166.08408:38347 167.11856:8890 194.12837:8407 195.11382:30909 246.511:6159 254.16579:6773 | 4.17886E-07 |
| NEG9815 | Cefpiramide | 5.385 | 611.112 | [M-H]- | 611.112 | 79.61639:5075 89.13125:759890 90.13486:35374 94.03929:5042 117.0695:29805 370.36819:6691 412.74951:5783 440.99393:6109 | 0 |
| POS12390 | yl-sn-glycero-3-p | 5.747 | 588.36383 | [M+H]+ | 588.36401 | 59.04856:36489 60.08022:12272 86.09536:9160 87.0433:13002 89.05891:30567 101.05926:7093 103.07439:13733 104.10692:87875 133.08537:11196 173.39102:11056 184.07457:21898 412.29788:6117 | -3.05933E-07 |
| POS6127 | lbut-3-en-2-yl)fu | 4.77 | 285.11392 | [M+H]+ | 285.11401 | 57.06986:9585 110.07567:6062 | -3.15663E-07 |
| POS14977 | -3-hydroxydecan | 5.943 | 780.54694 | [M+H]+ | 780.547 | 51.74374:10069 55.43724:8301 60.08022:27942 71.07257:10790 80.74166:8042 86.09537:79987 104.10693:11189 124.9995:22929 161.76192:7751 184.07458:204750 464.99683:8789 | -7.68692E-08 |

| Differences in metabolites between the Model group and the WJW group | | | | | | | |
|---|---|---|---|---|---|---|---|
| Alignment ID | Metabolite name | Rt(min) | Expreiment Mz | Adduct type | Reference m/z | MS/MS spectrum | PPM |
| NEG3711 | Sinapic acid | 1.325 | 223.08005 | [M-H]- | 223.08 | 71.09774:6503 79.05373:9090 84.60606:5587 92.02104:34617 97.07652:33898 106.00214:5626 136.06505:14081 140.30067:5684 165.03516:39688 | 2.24135E-07 |
| NEG4834 | ),5(R)-bis(hydroxy | 0.661 | 262.16595 | [M-H]- | 262.16611 | 84.03101:7416 100.04375:7708 | -6.103E-07 |
| POS10645 | ysoPC(0:0/18:1(9Z | 6.902 | 522.35522 | [M+H]+ | 522.35522 | 128.86884:5368 184.0692:8379 | 0 |
| NEG1883 | Dihydroxynaphtha | 1.377 | 160.05307 | [M-H]- | 160.05243 | 51.09473:5957 57.65318:5568 58.00337:6717 59.08403:31394 70.57812:5376 71.09898:10088 73.11619:47205 73.99634:5682 85.13128:5671 101.14601:5693 150.50673:5630 | 3.99869E-06 |
| POS5266 | 2'-Methoxyflavone | 0.889 | 253.08517 | [M+Na]+ | 253.08521 | 70.98643:6400 79.20729:5666 90.97554:6469 91.85212:4897 173.38123:7053 178.11426:5697 216.29192:6822 225.03308:5615 | -1.5805E-07 |
| POS2874 | Azelaic acid | 5.981 | 171.10107 | M+CH3OH+H] | 171.101 | 53.03772:6060 55.01768:58998 55.05364:20249 57.06894:642833 58.0727:7355 67.05387:8431 72.04367:23066 79.0531:14963 83.01192:449972 83.08669:5889 85.06334:206456 89.06976:6543 91.05387:11880 93.06838:20720 97.06458:7654 111.07957:14641 115.03664:25092 121.06406:8265 139.07353:9037 | 4.09115E-07 |
| POS5271 | 2'-Methoxyflavone | 8.761 | 253.08577 | [M+H]+ | 253.08549 | 70.40687:5541 73.03882:6438 133.87697:5330 213.12314:6659 242.1476:5910 | 1.10635E-06 |
| POS5270 | Dehydroeffusal | 6.458 | 253.08565 | [M+H]+ | 253.08569 | 53.29498:7137 95.92661:5268 129.79753:5642 150.35779:5993 251.99538:6043 | -1.58049E-07 |
| POS3344 | orophenyl)prop-2 | 4.161 | 185.04076 | M+CH3OH+H] | 185.0408 | 55.05363:9739 58.94248:9442 101.24716:5838 116.9712:31405 117.97749:26206 139.85522:5406 147.73953:4866 177.13303:5274 | -2.16169E-07 |
| POS4643 | hydro-3H-pyrano[ | 4.824 | 230.11755 | [M+H]+ | 230.11758 | 55.01768:438388 55.05363:539904 57.03284:886288 57.06894:11108241 61.01:1178954 67.05386:506047 69.06989:1050960 79.05309:479473 81.03226:2900829 81.06987:401629 83.04766:1292620 85.02796:423432 85.06333:3656297 94.01654:219990 95.0489:253486 95.08473:223981 97.02761:297533 97.06457:372030 98.426:228350 107.04913:280327 111.04436:1397226 119.08328:196404 123.0448:183343 125.04754:457597 125.05955:6812293 133.06891:1254960 167.10457:14973471 | -1.30368E-07 |
| POS3810 | cgonine methylest | 4.994 | 200.12769 | [M+H]+ | 200.12759 | 55.0177:20821 55.05365:509425 55.93435:26179 56.46365:6412 60.69907:6153 63.21632:5904 67.05389:54277 69.03294:20509 69.06991:45077 76.03859:206104 79.05312:29338 81.06989:55805 83.08509:62109 85.06336:6453 95.08476:21825 97.09953:121566 97.96837:6897 107.08485:17424 113.96311:40467 114.97045:23147 125.09565:43305 131.97139:7671 141.95662:13195 159.96982:8239 | 4.99681E-07 |
| POS194 | lethyl-3-buten-2- | 4.818 | 85.06468 | [M+Na]+ | 85.0647 | 56.0486:32294 84.04313:16884 84.07954:6052 | -2.35115E-07 |
| POS2871 | Azelaic acid | 5.671 | 171.10085 | [M+H]+ | 171.101 | 55.05363:20737 57.06986:32199 67.05386:6138 72.04366:30578 83.01192:20364 85.06502:6532 89.06976:12284 117.02013:5325 173.42537:8162 | -8.76675E-07 |
| POS4083 | Heteromine D | 4.832 | 209.12712 | [M+H]+ | 209.127 | 51.89836:5653 60.04323:6426 62.4356:5416 70.06427:22419 81.06829:5421 148.11047:10450 188.13643:5890 | 5.73814E-07 |
| POS4675 | Norpterphyllin III | 0.75 | 231.10406 | [M+NH4]+ | 231.104 | 55.93433:12000 57.03284:6762 57.06894:71414 69.06989:8521 79.05309:7886 81.03226:18224 83.04766:10137 85.06333:20585 111.04436:9389 125.05955:49088 133.06889:8965 167.10455:88462 | 2.59623E-07 |
| POS1778 | 4-Propylphenol | 4.815 | 137.09586 | [M+NH4]+ | 137.0959 | 53.61392:6146 53.8126:5696 67.05386:18617 72.8742:5711 77.83262:6596 79.05309:11525 81.06987:39587 91.05386:9088 95.08473:16437 | -2.91767E-07 |
| POS3418 | hiophen\|2-<Octat | 1.022 | 187.05727 | M+CH3OH+H] | 187.0575 | 62.55975:6475 67.44471:5435 70.06427:6321 81.06986:8247 101.83167:6262 | -1.22957E-06 |
| POS8693 | Ugonin P | 5.456 | 423.1788 | [M+Na]+ | 423.17899 | 59.04856:6510 69.68794:6085 85.02796:14425 96.01747:71660 108.74114:5448 138.02629:170491 173.43518:6079 180.07538:49427 193.08113:254384 194.08763:12209 213.98772:5948 218.55022:5748 338.6218:5873 | -4.48983E-07 |
| POS4666 | Norpterphyllin III | 5.591 | 231.10378 | [M+Na]+ | 231.104 | 53.03854:9765 55.01768:12449 55.05363:23575 57.03284:29823 57.06894:299791 61.01:36048 67.05386:14375 69.06865:26022 79.05309:12429 81.03226:83789 81.06987:16441 83.04766:36642 85.02796:14007 85.06333:103688 93.06837:6266 95.08473:8876 97.02761:13545 97.06457:11658 107.04913:7903 111.04436:33797 123.0448:6083 125.05955:244129 126.06273:6287 133.06891:40815 167.10457:465402 168.11116:22316 173.39104:7654 | -9.51952E-07 |
| POS11867 | ntibiotic FR 90084 | 5.929 | 566.32208 | [M+H]+ | 566.32239 | 59.04856:25629 60.08023:20202 71.07258:6320 73.02809:5629 86.09538:58974 87.04331:8500 89.05893:18621 104.10693:233342 124.9995:16304 184.07458:77241 355.55829:5667 | -5.47391E-07 |
| POS10211 | PE(0:0/20:3(5Z,8Z, | 6.328 | 504.30774 | [M+H]+ | 504.3075 | 55.84372:5639 55.87241:6045 57.03379:6226 60.15533:6699 62.05909:35627 67.05389:9008 81.0699:10454 89.05896:8405 95.08477:9910 107.08487:6805 115.67261:5821 | 4.759E-07 |
| NEG9755 | TAN-1496 E | 6.476 | 591.06091 | [M-H]- | 591.06061 | 79.0522:6446 79.66678:5497 | 5.07562E-07 |

| Differences in metabolites between the Model group and the WJW group | | | | | | | |
|---|---|---|---|---|---|---|---|
| Alignment ID | Metabolite name | Rt(min) | Expreiment Mz | Adduct type | Reference m/z | MS/MS spectrum | PPM |
| POS4668 | Norpterphyllin III | 8.925 | 231.10378 | M+CH3OH+H] | 231.104 | 55.01768:8285 55.05363:16588 55.93343:27143 57.03284:14107 57.06894:178894 61.01:23672 61.75778:5917 67.05386:7067 69.06865:17043 73.63633:5686 79.05309:10705 81.03226:47980 81.06987:12417 83.04766:18076 85.02796:14311 85.06333:50272 97.02761:8872 97.06457:8835 111.04436:24819 114.97041:24398 123.0448:6417 125.05955:131348 133.0656:20110 167.10457:251841 168.11116:10679 | -9.51952E-07 |
| POS4443 | Aminoanthraquinc | 4.347 | 223.06284 | [M+H-H2O]+ | 223.063 | 58.06414:18982 61.03867:16876 73.02809:6988 73.04685:17831 91.05573:21622 93.06644:6978 118.53261:5827 159.96542:6065 209.01393:10592 225.04034:69868 | -7.17286E-07 |
| POS3345 | ohtho[2,1-b]thioph | 1.326 | 185.04166 | [M+H]+ | 185.0419 | 55.05363:8202 62.56081:6476 113.0902:5627 | -1.297E-06 |
| POS4186 | Thiolactomycin ?? | 4.814 | 213.09402 | [M+H-H2O]+ | 213.0943 | 53.03772:39754 55.01768:27279 55.05363:8960 57.06894:755510 58.07269:14496 61.01:111636 64.67905:6122 67.05386:16043 68.99598:7803 73.01067:9754 81.03226:159019 85.06333:93695 87.02587:8795 91.02029:8021 95.0489:22413 95.08473:9010 97.02761:11801 101.03963:9227 109.10002:8852 123.04187:13448 129.03552:6515 133.0656:31378 137.09767:9771 166.09779:11435 | -1.31397E-06 |
| NEG5237 | Fissoldhimine | 0.955 | 277.16745 | [M-H2O-H]- | 277.16711 | 90.03759:5693 93.03925:2699770 97.03745:10799 102.00297:5516 103.07946:40858 141.08502:6409 151.06711:63717 173.78252:6615 | 1.2267E-06 |
| NEG5354 | Oprea1_249309 | 0.955 | 281.16565 | [M-H2O-H]- | 281.16599 | 65.52292:5964 75.06394:6474 93.03932:317154 95.0377:1006561 97.03753:253909 103.08179:10856 105.08054:8355 153.06766:13105 155.06664:13855 | -1.20925E-06 |
| POS3951 | ethylsulfanyl)propy | 4.535 | 205.05252 | [2M+H]+ | 205.0528 | 55.01768:78959 57.03284:19077 60.04324:9173 69.03291:7160 74.02325:8180 79.05309:53662 81.0683:31984 83.04929:7404 85.02796:34856 91.05386:8945 93.06837:10499 95.0489:7755 97.06457:63387 108.05521:12513 108.25813:6309 115.05253:9964 117.06908:12983 118.06285:184366 121.06405:7455 125.05955:449338 126.06273:14118 130.06154:7599 132.04301:8610 132.07886:32021 142.06561:8152 143.07098:14918 144.07964:52961 146.05714:223572 159.09131:17284 170.06129:9758 173.38614:9934 178.04791:8678 188.06996:43958 | -1.3655E-06 |
| NEG6992 | Ferocin | 0.963 | 339.19595 | [M-H2O-H]- | 339.19629 | 64.28277:5751 68.55454:6419 69.57709:5841 93.03925:226395 95.03763:278225 97.03745:46623 153.06755:27307 155.06651:12092 | -1.00237E-06 |
| POS2508 | Cleroindicin B | 4.932 | 159.10106 | [M+H]+ | 159.10139 | 53.68312:5904 55.05363:8822 55.93433:23564 56.0486:7073 57.06894:9653 58.07365:5503 66.47277:5330 69.06865:6803 70.06428:9016 71.01213:10431 71.04942:6172 76.93209:8708 104.37206:5536 | -2.07415E-06 |
| NEG5133 | labda-13(16),14-c | 1.039 | 273.25922 | [M-H]- | 273.25891 | 51.7709:5912 52.92688:7048 87.11259:26638 89.13132:620403 90.13493:24973 91.84886:6244 93.03932:615230 95.0377:199293 97.03753:78373 103.07954:38911 128.18695:6126 136.98956:5688 145.2356:10087 147.15926:225151 209.7704:5888 | 1.13446E-06 |
| POS9013 | Lovastatin | 4.419 | 443.23703 | [M+H]+ | 443.2373 | 70.06431:6500 82.91782:5885 89.05896:176541 118.06566:20414 132.07892:12359 133.08545:90295 136.07611:17369 144.0797:18937 146.061:96159 159.09138:16799 177.11285:8095 188.07004:385374 189.07106:24890 205.09822:73238 239.14922:15919 | -6.09155E-07 |
| POS11316 | yl-O-acetylbuxalc | 6.375 | 547.35266 | [M+H]+ | 547.35303 | 55.87151:5746 57.06989:10179 61.21729:5652 62.39856:5583 71.08547:7034 78.10174:6275 86.0954:23001 129.75313:5718 146.98277:20296 184.07465:16940 | -6.75981E-07 |
| POS7110 | kasarin | 4.438 | 326.17065 | [M+NH4]+ | 326.17099 | 57.06894:28962 70.06554:5563 74.73689:6539 85.06334:9850 87.04332:8115 89.05893:97753 98.51205:5351 110.07072:30468 122.05131:6042 133.0854:19736 | -1.0424E-06 |
| POS5114 | nyl)methyl]-N-ethy | 1.172 | 247.09932 | [M+H]+ | 247.09959 | 72.08044:6734 95.30415:4812 101.79417:5815 111.04436:6252 125.05955:7002 160.12196:6138 167.10455:13925 | -1.09268E-06 |
| NEG7218 | oro-10-hydroxyoc | 0.947 | 347.2363 | [M-H]- | 347.2359 | 50.12337:9028 60.17111:6402 76.71121:5469 82.79803:6263 93.03925:337442 95.03763:217009 97.03745:11363 103.07946:11923 151.06711:9117 153.06754:13092 338.8721:6308 | 1.15195E-06 |
| POS5829 | ylquinoline 1-oxide | 4.868 | 274.18237 | [M+NH4]+ | 274.18201 | 55.05365:155349 57.03287:30183 57.06896:44646 61.01003:30787 67.05389:217736 68.05673:8080 69.06868:142022 71.04816:36076 79.05312:36470 81.06833:454771 82.07301:19347 83.0477:24995 83.08509:56492 85.00948:11202 85.06337:16910 91.0539:49244 91.93919:5980 93.06841:63061 95.08477:403021 96.08827:17496 97.06461:18138 97.09953:30213 105.06945:67244 106.07349:6510 107.08486:32951 109.06336:18807 109.10006:28658 111.0796:6349 113.05927:8230 115.07378:6267 117.06913:5785 119.08333:131978 120.08878:8567 121.1013:42594 123.08004:34238 127.01965:9520 127.075:10520 133.09863:54900 135.11646:87970 137.09427:11368 137.1322:413534 138.13437:30462 151.14491:5836 161.13197:163509 162.1382:11314 169.10333:28795 173.39111:12062 179.13977:50426 | 1.313E-06 |

**Differences in metabolites between the Model group and the WJW group**

| Alignment ID | Metabolite name | Rt(min) | Expreiment Mz | Adduct type | Reference m/z | MS/MS spectrum | PPM |
|---|---|---|---|---|---|---|---|
| POS2926 | Xanthopappin A | 1.019 | 173.04153 | [M+Na]+ | 173.0419 | 70.06431:9695 173.04349:21661 175.89915:6307 | -2.13821E-06 |
| POS2777 | (E)-Lyratic acid | 8.914 | 167.10579 | [M+H]+ | 167.1062 | 53.93914:9285 84.95896:29778 103.05193:7594 112.97408:8555 119.39087:5587 120.08025:17724 125.05955:14703 | -2.45353E-06 |
| POS14755 | PC(32:0) | 8.805 | 756.552 | [M+H]+ | 756.55133 | 60.08022:31450 67.54832:12374 73.45212:11172 86.09537:77100 94.06747:11228 101.42462:11288 101.90234:10882 104.10693:10989 111.52592:11379 124.9995:15745 184.06921:205498 222.45992:12398 253.30632:11679 542.04785:14291 | 8.85598E-07 |
| POS12099 | raspiridole B_1300 | 4.457 | 575.31354 | [2M+H]+ | 575.31403 | 88.99577:8921 89.05891:64503 121.77953:7621 133.08537:31569 188.06995:14284 | -8.51709E-07 |
| POS1648 | ethylthiopentan-3 | 4.817 | 133.06772 | [M+Na]+ | 133.06816 | 67.803:5709 69.06989:39772 82.45757:6350 86.09537:158292 87.09914:6806 104.04764:25055 105.03241:6749 133.05241:12601 | -3.30658E-06 |
| NEG1561 | Citramalic acid | 0.646 | 147.10046 | [M-H]- | 147.10001 | 59.0295:16415 59.08405:16129 65.60266:6277 73.20889:6079 79.5448:5428 | 3.05914E-06 |
| POS6418 | bimidazole ribonu | 0.949 | 296.0636 | [M+H]+ | 296.06409 | 60.08025:23971 71.07261:19411 86.09541:204355 104.10697:776778 105.10879:24854 112.89422:14757 120.00122:13938 169.9852:20399 236.99554:7672 | -1.65505E-06 |
| POS5696 | 12-Hexahydroaze | 4.825 | 269.06018 | [M+H]+ | 269.06067 | 53.90258:5675 183.7211:6412 | -1.82115E-06 |
| POS7357 | -methylneoagaro | 4.731 | 339.12799 | [M+H]+ | 339.12851 | 55.01768:8163 57.06894:54867 59.04856:50167 65.94633:5708 83.01192:150502 85.06333:49011 87.04331:15571 89.05892:8066 101.02218:7596 103.03844:10054 103.07439:7378 111.07955:9845 115.03662:63315 139.07352:34259 141.03842:7937 152.00653:11651 157.08499:16901 158.7514:5029 169.03244:82317 171.09958:30901 217.64404:6682 | -1.53334E-06 |
| NEG6949 | | 0.963 | 337.19568 | [M-H2O-H]- | 337.19629 | 55.66968:5877 93.03925:612058 95.03763:385042 103.08171:6340 151.06711:31030 153.06754:44012 | -1.80904E-06 |
| POS8025 | nzylidene,2-mesy | 4.811 | 375.11008 | [M+2H]2+ | 375.11069 | 50.22026:7631 57.06894:14736 81.03226:6824 85.06503:6672 109.48528:6124 122.25717:6253 125.05956:8737 144.07964:10290 149.09477:5550 166.09781:6158 167.10458:58280 168.11118:9279 173.4303:8431 221.47981:6724 259.06525:6603 260.06213:9506 | -1.62619E-06 |
| POS11315 | 34-oxamanzamin | 6.191 | 547.34375 | [M+H]+ | 547.34308 | 50.72232:13760 60.08022:29099 72.07124:11772 76.34278:12631 76.75559:11365 86.09536:55042 104.10692:204612 105.10873:83869 106.111:29289 112.98438:11424 166.01965:10978 184.06921:58662 185.07626:30063 292.86453:11391 381.84113:11651 419.16068:13625 531.78821:11194 | 1.2241E-06 |
| POS15031 | PC(16:0e/12-HETE | 8.806 | 784.58392 | [M+Na]+ | 784.58502 | 53.95275:6330 55.18986:7167 57.91145:6853 104.49812:5708 134.72931:5113 151.41658:5085 164.64087:5531 | -1.40202E-06 |
| NEG9857 | ihydro-3-[2-[[(2E | 6.196 | 633.1239 | [M-H]- | 633.12299 | 89.1313:491831 90.13493:24984 171.80325:5295 | 1.43732E-06 |
| POS5109 | nyl)methyl]-N-eth | 8.898 | 247.09886 | [2M+H]+ | 247.09959 | 57.06986:10568 69.06989:13126 80.83523:6072 111.04185:26675 125.05955:20055 167.10457:27673 168.4581:6972 229.63365:6215 239.8895:5286 | -2.95427E-06 |
| POS7995 | GS-2 | 5.299 | 373.17462 | [M+H-H2O]+ | 373.17542 | 55.01768:9674 57.06894:18595 59.04856:85272 71.1009:6022 73.0281:6516 73.14081:6523 83.04929:11086 85.06334:8841 89.05893:28226 95.08474:23769 101.05709:5455 103.07441:10789 122.09476:11174 130.06473:24991 131.57179:5379 132.04303:6133 146.06094:21617 150.0889:7699 158.05779:26437 160.07413:15754 168.10181:6410 168.88625:6034 175.08525:11309 182.11658:5953 184.0746:22478 186.05348:5870 199.00485:5375 | -2.14376E-06 |
| POS7454 | IC ACID DER (FR. L | 5.815 | 343.28339 | [M+H]+ | 343.28421 | 57.06893:9343 60.08022:5723 67.24886:5568 74.11497:5719 86.09536:13025 163.8858:5687 170.79715:6674 184.07457:13501 188.54721:5212 200.60612:6777 216.05299:5938 | -2.38869E-06 |
| NEG3630 | H-Met-Ala-OH | 1.325 | 219.08076 | [M-H]- | 219.0816 | 58.04516:8286 97.07652:18103 99.07362:24309 118.92107:6107 161.03635:88578 | -3.83419E-06 |
| NEG1917 | 2403;(E)-Metanic | 0.965 | 161.10941 | [2M-H]- | 161.10851 | 55.08503:9289 57.10279:31440 59.08405:50394 71.099:60111 73.11621:35802 74.11263:10004 85.1313:50907 87.11256:8759 93.03928:677611 95.12722:11837 99.16254:9323 101.14605:5754 103.0795:17885 104.15944:8033 113.15993:18230 | 5.5863E-06 |
| NEG3318 | yl-1H-pyrazol-4-y | 1.228 | 208.0872 | [M-H]- | 208.0881 | 59.08407:39933 71.09774:8072 73.11623:69572 74.12088:6859 77.71171:5539 79.05373:15231 85.13134:6082 89.13131:33557 89.14215:7143 97.07652:565952 101.14608:10721 119.08548:7638 120.83963:5409 126.02468:11126 128.02199:17080 146.40347:5377 161.2383:8561 | -4.32509E-06 |
| NEG9811 | allocatechin 3,4'-d | 6.248 | 609.0907 | [M-H]- | 609.0896 | 89.1313:907206 90.13492:34712 156.12057:6322 409.70682:8289 591.94617:5607 | 1.80597E-06 |
| NEG3633 | Dehydrocarissone | 0.952 | 219.13754 | [M-H2O-H]- | 219.1385 | 51.07359:5631 93.03929:2280891 103.07951:87869 103.76355:5961 123.10053:12025 151.06718:8102 | -4.38079E-06 |

**Differences in metabolites between the Model group and the WJW group**

| Alignment ID | Metabolite name | Rt(min) | Expreiment Mz | Adduct type | Reference m/z | MS/MS spectrum | PPM |
|---|---|---|---|---|---|---|---|
| POS8010 | DIMBOA-Glc | 4.814 | 374.1109 | [M+2H]2+ | 374.10999 | 57.06894:43731 59.26746:6120 81.03226:19435 83.04929:13791 85.06334:20640 123.04189:8169 125.05956:16727 133.06561:19267 148.95413:8290 149.09477:15906 166.09781:17216 166.96548:11569 167.10458:129679 168.11118:10385 173.4303:11710 175.29941:6665 227.06221:21572 231.1055:9013 235.06412:6081 236.07355:23268 242.04243:11663 247.57834:7085 259.06525:22297 260.05313:5796 | 2.43244E-06 |
| POS4286 | phenylphosphinam | 1.46 | 217.06667 | [M+H]+ | 217.0657 | 52.0803:7392 58.06319:6150 61.18333:6596 66.03267:5109 70.06427:6646 | 4.46869E-06 |
| POS3884 | yn-1-one\|trans-5- | 4.268 | 203.05142 | [M+H]+ | 203.0524 | 55.05363:12412 60.04424:5763 72.74344:6130 78.84068:5528 79.36346:5228 82.00914:5838 | -4.82634E-06 |
| NEG8216 | squalene | 0.812 | 395.36743 | [M-H]- | 395.36841 | 103.07276:11174 147.11324:14110 174.91417:5283 | -2.4787E-06 |
| NEG9741 | Bartramiaflavone | 5.139 | 585.06635 | [M-H]- | 585.0675 | 51.88619:5547 57.33037:6239 61.31321:5860 74.51412:6798 89.13125:1512759 90.13486:55324 363.81647:7398 | -1.96559E-06 |
| NEG9802 | ''-Methoxyvinaxan | 5.522 | 605.05615 | [M-H]- | 605.05737 | 59.08407:15652 79.05373:33699 81.16663:5854 83.12411:15402 107.21418:10491 135.28148:17770 139.27896:9964 163.31036:9103 173.58612:7013 179.32071:34129 180.33003:7254 | -2.01634E-06 |
| POS13644 | gamma-Truxilline | 4.462 | 659.33124 | [M+H]+ | 659.33258 | 62.52151:10420 86.09536:24341 87.0433:29724 89.05891:43916 95.96495:12038 99.04334:21490 133.08537:23260 166.3371:10479 173.43027:13733 188.06995:50777 205.09811:12467 | -2.03236E-06 |
| NEG3662 | oisobutyl phthalic | 1.325 | 221.08037 | [M-H]- | 221.08141 | 79.05373:19243 89.13132:6326 97.07652:16499 163.03299:71272 | -4.70415E-06 |
| POS14982 | -3-hydroxydecano | 8.793 | 780.54865 | [M+2H]2+ | 780.547 | 55.74166:5505 103.29499:5156 138.22501:5852 173.39102:13069 | 2.1139E-06 |
| POS11810 | Ser Leu Pro Tyr Ala | 5.918 | 564.30145 | [M+Na]+ | 564.30267 | 57.03377:6989 59.04856:38234 60.08023:11190 86.09538:35400 87.04506:7924 89.05893:20376 103.0744:6286 104.10693:246669 105.10875:6849 126.15702:5745 146.98273:18779 173.37634:6494 | -2.16196E-06 |
| NEG4861 | decylbenzene-1,3 | 1.051 | 263.21698 | [M-H]- | 263.21805 | 87.11257:8198 89.13129:313336 90.13491:14096 93.0393:531192 95.03768:119175 147.15923:120996 148.16444:6571 151.0672:6294 | -4.06507E-06 |
| POS4249 | Marindinin | 6.416 | 215.10892 | [M+H]+ | 215.11 | 55.90113:6879 57.98917:6670 60.19744:7268 61.5167:5501 62.53429:6215 70.83131:5585 123.52111:6118 | -5.02069E-06 |
| POS5310 | Daidzein | 4.815 | 255.08171 | [M+H]+ | 255.08058 | 53.44067:6108 57.06986:7541 162.06279:6037 188.56947:5542 189.08772:11988 190.09126:9945 | 4.42997E-06 |
| POS14223 | hydroxy-6-(hydro | 4.476 | 703.36108 | [2M+H]+ | 703.36267 | 50.57987:12075 81.83556:12365 89.05891:26074 133.08537:21108 173.42535:18366 | -2.26057E-06 |
| POS10813 | Mls001140956 | 6.349 | 528.2981 | [M+H]+ | 528.29688 | 59.04856:8319 62.05906:16762 67.05386:6005 73.55632:5951 173.43027:7752 184.07457:6535 195.23669:5533 | 2.30931E-06 |
| POS12475 | -Tetradehydroasta | 4.575 | 592.35858 | [M+H]+ | 592.35999 | 87.04333:292582 87.73636:49559 89.05895:2113716 90.0619:265794 133.08543:920538 134.08682:187594 177.10777:66778 188.95941:48504 | -2.38031E-06 |
| POS11282 | anine-3beta,17,23 | 6.388 | 546.34113 | [M+H]+ | 546.34253 | 61.57684:6459 69.38287:7407 78.57826:5935 97.37959:6004 173.43524:12754 323.24142:5347 333.33173:6626 | -2.56249E-06 |
| POS11380 | beta-Scymnol sulfa | 5.398 | 549.31049 | [M+2H]2+ | 549.30908 | 59.04856:23963 70.72768:5438 86.09537:9585 87.0433:10775 89.05892:37861 104.10692:50456 133.08539:6636 184.06921:49416 270.05994:5654 | 2.56686E-06 |
| NEG7219 | Dodecyl glucoside | 0.951 | 347.24557 | [M-H]- | 347.24429 | 53.43378:6734 93.03925:329943 95.03763:217753 97.03745:17129 103.07946:12491 147.41237:5261 153.06754:11603 164.71239:5910 173.62527:5953 | 3.68617E-06 |
| NEG968 | Flucytosine | 1.381 | 128.02403 | [M-H]- | 128.0253 | 89.13129:16069 127.55685:5690 128.19003:7317 | -9.91991E-06 |
| NEG6185 | | 0.908 | 309.28989 | [M-H]- | 309.2912 | 93.03925:25532 95.03763:17698 97.03951:8962 103.07947:6493 105.07814:127109 113.12114:13673 124.15676:19737 | -4.23549E-06 |
| NEG3877 | Sarmentol A | 0.932 | 229.17865 | [M-H]- | 229.17999 | 79.05367:35423 93.03925:1344531 95.03763:52927 97.03745:12581 97.08466:13376 103.07946:280871 | -5.84693E-06 |
| POS9171 | es-arg9)-Bradykin | 4.463 | 452.73608 | [2M+H]2+ | 452.73743 | 60.04426:33393 70.06431:764671 71.06746:21564 86.05939:5841 112.08709:15961 120.08031:56494 157.10199:11559 166.08408:6287 173.39111:10443 263.13351:20543 453.70743:8619 | -2.98186E-06 |
| NEG2059 | Fluorocinnamic ac | 2.365 | 165.03433 | [M-H]- | 165.03572 | 55.9624:4941 58.11544:4593 59.91349:4666 60.95618:4588 63.27049:4503 64.56695:4678 65.45237:4508 66.11316:4603 70.95638:4896 71.53596:5115 74.69258:4643 76.7502:4013 81.75526:4291 84.90416:4658 88.45958:4754 89.58092:4436 93.14533:4997 94.02954:4403 95.4747:4193 97.07649:23371 97.18539:4177 109.36189:4477 111.2215:4581 115.00906:5117 119.16917:4485 125.9518:4829 136.76608:4327 137.42764:4511 156.04103:4502 159.0804:3900 165.81146:3944 | -8.42242E-06 |
| NEG9635 | H,2H-Perfluorode | 5.97 | 562.99481 | [M-H]- | 562.99323 | 59.08402:9092 79.05367:13711 93.85946:5239 95.63505:5906 102.93809:5184 277.54752:8793 | 2.80643E-06 |

| Differences in metabolites between the Model group and the WJW group | | | | | | | |
|---|---|---|---|---|---|---|---|
| Alignment ID | Metabolite name | Rt(min) | Expreiment Mz | Adduct type | Reference m/z | MS/MS spectrum | PPM |
| POS7836 | Inumakilactone A | 4.814 | 365.12537 | [M+2H]2+ | 365.1239 | 70.19419:5737 70.58992:6560 99.70287:6753 126.41608:6569 162.04507:6534 204.09253:9393 227.08423:22130 250.07343:9381 | 4.02603E-06 |
| NEG3683 | pyrazin-2-yl)meth | 1.238 | 222.10211 | [M-H]- | 222.1037 | 97.07645:144827 195.65027:6966 | -7.15882E-06 |
| NEG3663 | SCHEMBL1388932 | 0.955 | 221.13783 | [M-H]- | 221.1395 | 93.03925:792254 95.03763:1454007 103.07947:55862 105.07814:25656 | -7.55179E-06 |
| POS7880 | 2-Dihydro-(Z,Z)-1 | 4.827 | 367.22177 | M+CH3OH+H] | 367.22351 | 59.04856:37874 70.06428:148208 73.02809:10618 84.07954:43110 87.04331:13396 89.05892:47022 94.06355:14508 96.08015:42066 116.069:13440 133.08539:8421 148.11049:302496 149.11432:17073 165.13654:37938 209.1243:8955 298.54297:5790 | -4.73826E-06 |
| POS10566 | Psychotrimine | 7.26 | 519.32117 | [M+H]+ | 519.323 | 50.16373:6887 173.39102:12142 190.7968:5946 313.43536:5778 443.14413:5879 | -3.52382E-06 |
| POS6904 | ctamide semisucc | 4.981 | 316.20999 | [M+2H]2+ | 316.21179 | 56.28908:6220 57.06894:21287 65.11468:5248 71.08544:10108 85.02796:11016 116.069:22489 144.06477:116778 155.23679:5802 158.0791:20443 204.08626:9544 | -5.69239E-06 |
| NEG948 | yl-2,3-dimethylpe | 1.102 | 127.15112 | [M-H]- | 127.1493 | 82.05115:58658 83.85929:5676 89.13124:245074 90.13486:13532 97.09287:11531 99.88496:5881 126.15527:9329 128.18996:18336 | 1.43139E-05 |
| POS1940 | yl]methyl 2-methy | 4.418 | 142.06476 | [M+H-H2O]+ | 142.063 | 51.89838:6403 55.0177:12582 55.05366:20095 56.9414:23620 57.68123:6654 62.30868:6446 64.73273:6578 67.05389:9188 69.03294:11704 69.06992:9261 70.06431:11796 70.44497:5969 72.93707:13392 95.08477:8545 96.08018:8232 113.96312:7614 115.05257:35396 143.0343:35726 | 1.23889E-05 |
| NEG6902 | Istamycin KL1 | 0.963 | 335.19553 | [M-H2O-H]- | 335.1937 | 89.13124:24821 93.03925:542343 95.03763:47169 97.03745:32893 116.0619:6219 133.28078:6024 142.01402:5396 151.06711:47960 167.22272:11081 334.64075:5292 | 5.45953E-06 |
| POS4727 | ycero-pentulose) 1 | 4.815 | 233.10005 | [M+NH4]+ | 233.1019 | 53.03772:63084 54.04139:13798 55.01768:123942 55.05363:106245 56.02068:24513 56.05671:34031 57.03284:185372 57.06894:2077693 58.03659:32060 58.07269:616833 59.04856:33323 59.07586:10628 61.01:169423 62.01289:26131 63.00633:121529 67.01733:18516 67.05386:80745 68.05671:15839 68.99598:18621 69.03291:13742 69.06865:168111 70.0731:52136 71.01213:13405 71.04942:10780 73.00933:9853 75.00541:8774 79.05309:85527 80.05733:38082 81.03226:538095 81.06987:96583 82.03626:199772 82.07298:37309 83.04766:220530 84.0514:89592 85.02796:84541 85.06333:542437 86.03192:21388 86.06792:206682 87.06947:19218 91.05386:14044 93.06837:30923 94.07335:15087 95.0489:38375 95.08473:40722 96.05183:12069 96.08823:22827 97.02761:62571 97.06457:76830 98.03084:28852 98.06837:34211 99.04335:10673 103.03844:9134 107.04913:39429 108.0528:21900 109.06332:13291 109.10002:22181 111.04436:205374 112.04627:92888 113.0489:14727 121.10125:9485 123.0448:26997 124.05049:37016 125.05955:1076340 126.06273:628918 127.06264:86861 131.03217:11939 133.06891:78873 134.07011:101645 135.06244:119802 137.09422:21919 138.09946:13984 139.10875:21397 140.1149:18014 150.10074:9414 167.08138:22464 167.10457:1270805 168.11116:1294474 169.1127:210337 186.10252:11168 187.09335:14999 | -7.93644E-06 |
| POS7608 | 2-(3-furanyl)ethyl] | 5.516 | 352.25009 | [M+H]+ | 352.24823 | 57.06894:50991 71.08544:19760 83.08505:5694 84.07954:64985 85.10041:5780 86.09537:12881 88.0744:5945 166.05182:15901 184.06384:35843 | 5.28037E-06 |
| POS4336 | Theobromine | 1.009 | 219.02596 | [M+H]+ | 219.02786 | 57.99675:6674 62.98166:16790 80.94775:20348 82.94376:622374 85.02799:6873 90.97557:117177 131.77626:6205 159.04828:7808 177.06223:11363 | -8.6747E-06 |
| NEG9325 | Maltotriose | 6.334 | 502.90198 | [M-H]- | 502.89999 | 56.04784:6105 67.74068:6038 | 3.95705E-06 |
| NEG3492 | N-Nonanoylglycin | 1.45 | 214.14711 | [M-H]- | 214.1451 | 55.67414:6437 65.62659:7043 84.0806:6578 89.13124:8733 126.02458:336706 127.0235:9115 165.97191:5996 | 9.38616E-06 |
| POS1825 | Fumaric acid | 1.867 | 139.00229 | [M+H-H2O]+ | 139.00021 | 52.35179:5638 55.0177:46251 55.05365:17776 62.64914:5860 67.05389:10667 68.996:233036 79.05312:9205 81.06989:9322 82.07301:11076 94.06554:12297 95.04893:8493 95.08476:6105 96.04379:6441 96.08827:7644 105.44981:6102 110.05835:8897 111.00671:27130 112.58314:5851 120.04356:7439 122.06003:9424 138.04724:13404 | 1.4964E-05 |
| POS11132 | yl-15-nonyl-9-pr | 5.581 | 540.36176 | [M+H]+ | 540.36401 | 51.79255:6873 57.03286:8263 59.04858:62176 60.08024:16632 70.71621:5496 83.32796:6701 86.0954:23095 87.04333:9356 89.05895:22252 104.10696:169093 117.09088:8898 173.43524:6501 184.07465:40385 339.21149:6492 | -4.16386E-06 |
| POS4492 | Cysteinyl-Cysteine | 0.99 | 225.03397 | [M+Na]+ | 225.0361 | 62.98166:93415 66.34612:5813 81.50273:5899 86.9684:6983 96.92101:46571 98.91862:42037 104.99086:895706 105.99377:9747 116.99028:108325 135.00185:16176 | -9.46515E-06 |
| NEG9602 | PFCA-H | 5.373 | 556.98877 | [M-H]- | 556.98627 | 62.79358:5873 79.05221:8913 81.14308:5793 89.53181:5702 181.16978:6607 | 4.48844E-06 |
| POS9651 | cyclo[10.8.0.02,9.04 | 8.809 | 478.32684 | [M+H-H2O]+ | 478.32913 | 50.00464:6662 62.23794:5641 86.09537:7955 87.28099:5024 88.32702:6885 111.99535:7242 119.55073:5597 192.5636:6034 428.54907:6149 | -4.7875E-06 |
| POS1363 | roxy-1,4-benzoqu | 0.991 | 125.02081 | [M+Na]+ | 125.0232 | 101.17503:6058 117.59318:5031 | -1.91165E-05 |

**Differences in metabolites between the Model group and the WJW group**

| Alignment ID | Metabolite name | Rt(min) | Expreiment Mz | Adduct type | Reference m/z | MS/MS spectrum | PPM |
|---|---|---|---|---|---|---|---|
| POS4291 | Longiflorone | 5.696 | 217.10446 | [M+NH4]+ | 217.1069 | 55.01768:16464 55.05363:17945 57.03284:43864 57.06986:19536 58.03659:7789 61.01:13364 63.00633:9905 67.01733:8009 67.05269:7271 69.06989:6962 75.02634:22691 77.02202:15905 79.05309:15500 80.05733:12623 81.06987:15537 82.07138:6386 83.04766:31121 84.0514:11510 85.02796:29634 86.03021:6940 97.06457:16772 107.04913:8384 111.04185:21558 112.04627:14442 124.05049:13923 125.05955:275719 126.06273:164950 127.06264:21246 167.10457:283170 168.11116:288651 169.1127:49499 | -1.12387E-05 |
| POS11752 | etraen-1-yl]-5,6-d | 5.605 | 562.33954 | [M+H]+ | 562.3374 | 59.04859:14791 60.08025:13667 86.09541:19358 89.05896:9053 104.10697:110067 133.08545:7633 184.07466:28801 | 3.80554E-06 |
| NEG3252 | droxytrihomometh | 1.228 | 206.08826 | [M-H2O-H]- | 206.08569 | 59.08407:15520 73.11623:33213 75.54928:5876 79.4505:5340 89.13131:7356 95.0377:12043 103.68422:6117 108.77026:5580 126.02468:113253 143.15802:6167 161.03633:9333 181.00244:6086 | 1.24705E-05 |
| POS2928 | hyl-1,4-naphthoq | 4.922 | 173.06264 | [M+Na]+ | 173.06 | 55.05363:9834 67.05386:25503 71.71907:6162 86.84657:5966 109.10002:10689 125.05955:51217 173.39104:10989 | 1.52548E-05 |
| POS3354 | thyl-1-azulenecarl | 4.815 | 185.09868 | [M+H]+ | 185.09599 | 53.03854:7116 55.01768:6571 55.05363:8614 57.06894:89617 57.93418:13230 61.01:267803 63.99744:6028 67.05386:23601 75.56093:6006 81.03226:12174 81.06987:10338 85.06333:11562 87.02412:6764 91.05386:6057 95.0489:29208 109.10002:11649 | 1.4533E-05 |
| NEG9525 | Chlorfluazuron | 6.082 | 538.96613 | [M-H2O-H]- | 538.96301 | 173.6057:21503 253.52328:66828 254.53137:9394 284.3291:5692 | 5.78889E-06 |
| NEG8825 | 3beta-ol\|4alpha,2 | 0.904 | 443.42911 | [M-H2O-H]- | 443.4259 | 55.6278:5822 93.03925:66331 95.03763:35097 97.03745:12013 103.07947:87112 113.12114:15535 257.57416:5848 | 7.23909E-06 |
| POS5741 | Diuron | 1.025 | 270.97675 | [M+Na]+ | 270.98001 | 62.98163:22976 74.09579:5637 76.16116:5345 80.94772:29281 82.94373:6478 90.97554:17180 96.92096:10587 104.99081:108276 116.99022:15700 135.00177:368785 136.00449:6799 140.06859:13752 | -1.20304E-05 |
| POS2612 | L-Carnitine | 1.011 | 162.11203 | [M+H]+ | 162.11247 | 55.05363:6803 57.03284:158120 58.06414:36314 59.07293:35311 60.08022:815004 61.02843:9713 84.08119:18494 85.02795:386671 102.09037:217084 103.03844:486962 104.04307:7802 162.11153:194240 | -2.71417E-06 |
| POS4386 | Pantothenic acid | 4.396 | 220.11717 | [M+H]+ | 220.11798 | 55.0177:49954 55.05365:39474 56.01259:12366 57.06895:303380 59.04858:241967 60.04425:13005 61.02845:8034 67.05389:184512 69.06867:181612 70.02779:221788 72.04369:495929 73.02811:119645 74.02328:43455 81.06832:15058 83.04769:30535 85.06336:264051 86.0954:52130 87.07997:42329 90.05456:1912377 91.05762:28719 95.04893:165012 96.08018:52378 98.02253:384394 100.03728:43983 103.07443:131626 113.05926:48921 116.03413:187566 124.07427:344107 125.07761:14548 131.06764:15485 142.08383:103294 156.10028:10172 160.0959:19318 166.08405:18475 174.10922:13004 184.0961:69153 202.10344:51304 220.11993:8643 | -3.67984E-06 |
| POS2752 | Phenylalanine | 4.381 | 166.08542 | [M+H]+ | 166.08627 | 51.02314:20908 53.03856:12102 77.03801:251645 79.05312:825183 80.04813:27428 91.05389:491642 93.0684:1242536 94.06358:30112 95.04893:214143 102.04611:26303 103.0542:4972212 104.05679:101402 105.044:23711 105.06944:23312 107.04916:486048 118.06288:64796 119.07215:44825 120.08028:14988410 121.08411:298965 131.04831:132634 | -5.11782E-06 |
| POS5974 | nonadienyl)-3-me | 6.165 | 279.23041 | [M+H]+ | 279.23169 | 53.03856:8408 55.05364:126356 57.06895:28213 65.03803:10419 67.05388:1126282 68.05672:28635 69.06866:117076 71.04815:14044 79.05311:77584 81.06831:947627 82.07299:28072 83.04768:14836 83.08508:58576 85.06504:13836 91.05389:23458 93.06839:89080 95.08475:784001 96.08826:25010 97.09951:25132 105.06944:23707 107.08484:69139 109.06334:16580 109.10004:245262 110.10298:14125 117.06911:12139 119.08331:21450 121.10127:40799 123.11521:94882 131.08374:32588 133.1019:15427 135.11642:38478 137.13217:49228 145.09901:20479 147.11679:15227 149.12999:24142 151.14888:9695 159.1172:11989 163.14491:13367 173.13148:21904 173.43031:9482 | -4.58401E-06 |
| POS8713 | Linoleoylcarnitine | 5.468 | 424.34036 | [M+H]+ | 424.34207 | 55.05364:150847 57.03286:65854 57.06895:33783 59.04857:30031 60.08024:486717 67.05388:45969 69.06866:195045 71.08546:8042 81.06831:63957 83.08508:104109 85.02798:1963085 86.03023:37237 89.05894:14804 91.05389:10072 93.06839:18013 95.08475:57991 97.06459:9392 97.09951:58719 99.07937:8237 105.06944:7259 107.08484:11790 109.10004:30690 111.07958:8035 111.11478:21744 119.08331:7051 121.10127:10638 123.11521:13291 133.0986:8555 135.11642:11354 137.13219:10337 139.1123:6562 144.10194:39773 147.11679:10579 161.13194:12289 173.39598:7408 | -4.02977E-06 |
| POS769 | Choline | 1.002 | 104.10676 | [M]+ | 104.10699 | 58.06511:824339 59.07295:236863 60.08025:4750052 104.10696:4088644 | -2.20927E-06 |

| Differences in metabolites between the Model group and the WJW group | | | | | | | |
|---|---|---|---|---|---|---|---|
| Alignment ID | Metabolite name | Rt(min) | Expreiment Mz | Adduct type | Reference m/z | MS/MS spectrum | PPM |
| POS1626 | Isoleucine | 1.185 | 132.10175 | [M+H]+ | 132.10188 | 53.01286:7055 55.0177:13773 55.05365:14316 56.04953:22212 56.94233:10269 57.05693:51299 58.06417:43433 61.01003:17182 67.05389:8534 69.06868:586708 72.04369:7239 73.06432:10863 86.09541:3246155 87.02591:7895 87.09918:15027 90.05457:13104 132.10175:10606 | -9.84089E-07 |
| POS6396 | nylalanylglutamic | 4.45 | 295.12854 | [M+H]+ | 295.12851 | 56.04862:20747 59.04858:5933 79.05463:6232 84.04315:468398 85.04819:6074 91.05389:7079 93.0684:10304 102.05276:13654 103.05196:53477 107.04916:26937 120.08028:1456756 121.08411:56163 130.04883:64280 131.04831:67332 136.0761:13332 149.05962:22893 166.08405:156041 167.08606:7732 186.09166:34363 232.09715:7410 | 1.01651E-07 |
| POS3991 | Indolelactic acid | 4.77 | 206.08014 | [M+H]+ | 206.08121 | 57.06896:30889 67.28918:6299 91.0539:24944 99.71146:5661 103.05421:7142 115.05257:62846 117.05553:47575 118.0629:999360 119.06658:34519 130.06477:564182 131.07088:69125 132.07892:194949 133.08214:8784 142.06566:70979 143.07103:41488 144.07968:99930 146.05721:268826 147.06317:11807 160.07417:155455 170.05659:153698 171.0612:10913 188.07002:80092 194.39003:5958 206.07948:14975 | -5.19213E-06 |
| POS1185 | yl-a-aminoisobut | 1.013 | 118.08609 | [M+H]+ | 118.0866 | 53.03773:43769 55.05365:2259418 56.04862:35861 57.05693:188243 58.06416:1108702 59.04858:40500 59.07198:1019120 70.0643:13165 71.06746:29623 72.08047:2998117 73.08444:9731 118.08494:671902 | -4.31886E-06 |
| POS5962 | Dibutyl phthalate | 6.414 | 279.15845 | [M+H]+ | 279.15909 | 57.06896:458324 58.07272:10673 65.03804:35537 67.05389:8404 81.0699:8269 88.65063:6869 91.05203:5475 93.03178:13287 107.08248:6126 121.02692:82243 149.02057:2416805 150.02579:107710 151.60489:5687 167.03043:6447 | -2.2926E-06 |
| POS2172 | Phthalic anhydride | 6.414 | 149.02289 | [M+H]+ | 149.02328 | 65.03804:511486 84.07957:7042 93.03371:50982 111.0444:60191 121.02691:156586 132.55287:5859 149.02055:222848 150.02579:6832 | -2.61704E-06 |
| POS2112 | D-Glutamine | 1.094 | 147.07629 | [M+H]+ | 147.0764 | 55.05365:24853 56.04862:358514 57.03286:10764 58.06511:15396 60.08024:37261 67.05389:20696 69.03294:7830 72.08047:11212 74.02328:23964 82.06502:8848 84.04315:2728692 84.07294:49023 84.07957:583470 85.02799:47516 85.04652:17991 86.05938:28923 87.04333:69607 91.05389:7809 101.07021:39437 102.05498:54518 130.04883:350584 130.08388:31960 | -7.47911E-07 |
| POS3931 | DL-Acetylcarnitine | 1.064 | 204.12248 | [M+H]+ | 204.1232 | 57.03286:122207 58.06511:24310 60.08024:668239 85.02798:3912352 86.03195:55965 144.10196:60948 145.05025:75050 204.1239:36013 | -3.52728E-06 |
| POS8739 | Oleoylcarnitine | 5.6 | 426.35623 | [M+H]+ | 426.35779 | 55.05366:23248 57.03287:55325 57.06896:58528 59.04859:9346 60.08025:449291 67.05389:18680 69.06868:35283 71.08419:28706 81.06834:24527 83.0851:38575 85.028:2271848 85.10045:16305 86.03025:37104 93.07034:11432 95.08477:38242 97.09953:26196 107.08487:8926 109.10007:17784 111.11732:8733 121.1013:22005 135.11646:14656 144.10197:46088 149.13394:13348 173.43036:7350 | -3.6589E-06 |
| POS4054 | cetyl-DL-phenylala | 4.736 | 208.09615 | [M+H]+ | 208.09679 | 53.41887:5633 57.52442:6482 77.038:6865 79.05311:5655 86.17436:6026 91.05388:7406 93.06838:21384 103.05419:54353 107.04677:9415 118.06287:6317 120.06332:33234 120.08028:1588035 121.0841:55192 131.04829:15870 162.0894:10263 166.08403:36842 173.38617:11598 | -3.07549E-06 |
| POS1617 | Creatine | 1.02 | 132.07649 | [M+H]+ | 132.07678 | 58.06512:10415 68.0483:15458 70.06557:11843 71.04816:6014 72.0542:10250 85.0836:5968 86.05939:18722 87.05381:50090 87.07824:7657 90.05457:746073 114.06509:16834 115.04728:7870 132.07567:150401 | -2.19569E-06 |
| POS5117 | mma-Glutamylval | 4.382 | 247.12816 | [M+H]+ | 247.1288 | 55.05365:108200 56.04862:40411 57.03287:7214 69.06992:7648 72.08047:1399870 73.0831:28712 79.05312:10563 83.0477:12338 84.04316:628411 85.02799:8975 85.04652:13994 86.09541:41765 88.03895:16447 102.05276:15880 107.04679:23549 118.08494:251978 119.08612:7749 130.04884:85159 138.08907:11043 141.09964:7876 156.1003:28288 184.09612:60439 230.1053:8228 | -2.58974E-06 |
| POS1544 | Pipecolic acid | 0.825 | 130.08577 | [M+H]+ | 130.08626 | 56.0486:24527 60.60572:5781 84.07954:132334 99.51071:5700 | -3.76673E-06 |
| POS2185 | rans-Cinnamic aci | 4.381 | 149.05949 | [M+H]+ | 149.05991 | 53.32008:5990 56.04953:9270 65.03805:7397 69.61556:6564 74.02328:8517 77.03802:30442 79.05313:145573 79.54906:5949 84.95901:7244 91.0539:36205 93.06841:62515 95.04894:47393 102.09042:7023 103.05422:290493 105.04402:15037 105.07177:6210 107.04918:37790 121.0641:15924 131.04832:7204 149.05965:5875 | -2.81766E-06 |

| Differences in metabolites between the Model group and the WJW group | | | | | | | |
|---|---|---|---|---|---|---|---|
| Alignment ID | Metabolite name | Rt(min) | Expreiment Mz | Adduct type | Reference m/z | MS/MS spectrum | PPM |
| POS2121 | Lysine | 0.829 | 147.1127 | [M+H]+ | 147.11278 | 55.05366:25488 56.04863:205512 58.06512:10718 64.97839:8676 67.05389:71518 68.98124:25102 72.08048:107123 74.02328:9653 84.07958:2397784 85.06337:13968 85.0836:18732 86.99279:33388 105.00241:33210 112.11258:14540 119.03033:16718 130.08389:109110 | -5.438E-07 |
| POS9113 | rachidonoylcarniti | 5.458 | 448.34097 | [M+H]+ | 448.34207 | 55.05365:37030 57.03287:20498 57.06896:17059 59.04858:34023 60.08025:209223 67.05389:89922 69.06868:53369 73.02811:6666 79.05312:38076 81.06833:79266 83.08509:13748 85.02799:487630 86.03024:7335 87.04334:7280 89.05896:27869 91.0539:16592 93.06841:61372 95.04893:7555 95.08477:71604 105.06945:14911 107.08486:28129 109.10006:22696 119.08333:21630 121.1013:34323 123.11523:10586 131.08377:9976 133.10193:13706 135.11644:14254 144.10197:22296 145.09904:6277 147.11681:6546 149.13002:6498 157.05124:8175 166.11165:5622 | -2.45348E-06 |
| POS6276 | Caryophyllen-beta | 4.377 | 290.15833 | [M+H]+ | 290.15979 | 55.05365:24601 56.04862:7940 57.03287:16604 60.08025:107724 70.0643:14590 70.43227:5768 72.08047:8247 81.56439:5745 83.0477:18170 84.07957:26752 85.02799:330220 86.03024:6849 86.09541:9647 101.0593:57444 103.04073:5965 111.04189:107845 129.05446:47478 144.10197:12880 147.06317:9623 | -5.03171E-06 |
| POS1531 | Pyroglutamic acid | 1.096 | 130.04965 | [M+H]+ | 130.05009 | 55.05453:7494 56.04862:128944 58.06511:10979 67.05389:10890 70.0643:61359 74.02328:5937 83.05907:29290 84.04315:318672 84.07957:532844 85.02799:13483 86.05938:34163 102.63087:5424 119.02752:8836 130.04883:17137 130.08388:22824 | -3.38331E-06 |
| POS1441 | Thymine | 4.432 | 127.04982 | [M+H]+ | 127.05018 | 52.01421:6341 53.03857:8442 54.03373:44442 55.01771:6053 56.04863:31362 75.04592:5839 81.04327:7179 82.02832:13058 84.04317:16026 109.03891:11158 110.02366:31838 127.05041:24780 | -2.83353E-06 |
| POS5916 | coniferonic acid | 5.986 | 277.21527 | [M+H]+ | 277.216 | 55.05366:44631 57.06896:16250 58.06512:5628 65.28091:6331 67.05389:51281 68.14243:5627 69.06992:29253 79.05313:99766 81.06834:45265 83.0851:13430 91.0539:27433 93.06841:123652 95.04894:8984 95.08477:39035 97.10159:6766 105.06946:20093 107.08486:75893 109.10007:12695 119.08334:10177 121.1013:52478 131.08377:9902 133.09863:9803 135.11646:36700 147.11682:6653 149.13002:18174 | -2.63333E-06 |
| POS6038 | Linoleic acid | 6.277 | 281.24734 | [M+H]+ | 281.24731 | 55.05364:125812 57.06895:46625 59.04857:6943 67.05388:53085 69.06866:127913 69.45988:5680 79.05311:13502 81.06831:62368 83.08508:73554 91.05389:13556 93.06839:20966 95.08475:61126 97.06459:10580 97.09951:57009 97.41055:5954 105.06944:7469 107.08484:21791 109.10004:30321 111.07958:5866 111.11478:10256 119.0861:13805 119.28451:5333 121.10127:17172 123.11521:11864 133.1019:14386 135.11642:9635 147.11679:6899 149.12999:6540 161.13194:6625 | 1.06668E-07 |
| POS6455 | Epoxyoctadecenoi | 5.771 | 297.24133 | [M+H]+ | 297.24237 | 51.75735:6200 53.80923:6083 55.01683:16956 55.05365:93349 57.03287:13354 57.06896:32501 67.05389:156887 69.06868:92838 71.04816:6667 71.08547:26065 79.05312:36059 81.06834:142837 83.04932:10612 83.08509:42345 85.06337:7733 85.10045:22460 91.0539:14153 93.06841:51028 95.08477:116463 95.10668:6615 97.06461:12573 97.09953:47258 99.07939:8130 105.06945:7673 107.08486:44411 109.10007:58942 111.0796:7031 113.09542:17052 117.06913:6481 119.08333:9755 121.1013:31741 123.11523:23177 125.09565:27342 131.08699:11338 133.09863:15672 135.11646:21771 137.1322:11367 139.11234:7678 147.11681:9530 149.13002:7365 173.13152:6600 | -3.49883E-06 |
| POS5099 | Valerylcarnitine | 4.435 | 246.16867 | [M+H]+ | 246.16998 | 55.05365:9061 57.03286:58221 57.06895:137203 58.06511:8810 60.08024:316716 70.02779:20196 72.08047:64540 74.02328:10064 84.04315:17897 84.07957:18389 85.02798:2517008 85.06335:64648 86.03024:51000 86.09539:93092 99.04126:14679 103.07442:9965 114.09124:13155 118.08492:10979 132.10172:12932 144.10196:18704 187.0934:32362 246.17052:12519 | -5.32153E-06 |
| POS1243 | ydroxyphenethylar | 4.382 | 120.08065 | [M+H-H2O]+ | 120.081 | 51.02236:20234 53.03856:11115 56.04862:14856 56.05763:10581 56.94139:9320 61.03869:23174 65.03804:22284 72.93572:15278 73.08309:15660 77.03802:204914 79.05312:20551 80.04813:19621 91.05389:311757 93.0684:258589 95.04893:482542 102.04612:38530 103.05421:1672750 105.04401:117492 118.06289:42145 119.07216:26808 120.08029:1302164 121.08411:10697 | -2.9147E-06 |
| POS2553 | 2-Aminooctanoic | 4.49 | 160.13257 | [M+H]+ | 160.13318 | 55.05364:520393 57.06987:13520 59.22437:6498 69.06866:57889 72.08046:81235 97.09951:60103 114.12788:635836 115.12945:6748 118.06563:6126 118.11523:5590 | -3.80933E-06 |

| Differences in metabolites between the Model group and the WJW group | | | | | | | |
|---|---|---|---|---|---|---|---|
| Alignment ID | Metabolite name | Rt(min) | Expreiment Mz | Adduct type | Reference m/z | MS/MS spectrum | PPM |
| POS6500 | s-9,10-Epoxysteari | 6.019 | 299.25647 | [M+H]+ | 299.25781 | 50.23555:5582 55.05364:51847 57.06895:121094 61.54363:6034 67.05388:57802 69.06866:61990 71.08546:90570 79.05311:8757 81.06831:69652 83.08508:35820 85.10043:19202 90.70594:5740 91.05388:7903 93.06838:15439 95.08475:58498 97.09951:26773 105.06943:12217 107.08484:16452 109.10004:32294 111.11478:14801 121.10126:8975 123.1152:15658 133.0986:6310 141.12842:7674 155.14133:7758 | -4.47774E-06 |
| POS331 | Sarcosine | 1.035 | 90.0547 | [M+H]+ | 90.05498 | 72.08048:13617 90.05457:7044 | -3.10921E-06 |
| POS9307 | 4,27-Nonaoxanon | 4.512 | 459.2782 | [M+H]+ | 459.27997 | 73.06432:38233 87.04335:173401 89.05896:2331928 90.06191:48338 107.07059:22315 111.69564:10559 117.0909:14385 130.08708:15935 131.06766:31613 133.08545:979143 134.08685:25212 173.39603:18287 175.10025:10959 177.11287:82760 404.97165:11585 | -3.85386E-06 |
| POS6892 | 9,10-DHOME | 5.769 | 315.2522 | [M+H]+ | 315.25269 | 55.05364:85993 57.03286:21650 57.06895:88435 67.05388:336217 68.05793:7065 69.06866:92537 71.04815:23103 71.08546:199392 72.08835:6770 79.05311:50379 81.06989:341412 82.07299:12434 83.04768:10102 83.08508:44796 85.06335:25664 85.10043:139213 86.10397:8183 91.05389:40656 93.06839:65346 95.08475:254246 96.08826:8358 97.06459:16473 97.09951:21453 99.07937:107753 105.06944:28517 107.08484:45978 109.06334:7637 109.10004:89606 111.07958:12368 111.11478:6867 113.09541:97063 117.06911:8496 119.08331:25121 121.10127:43117 123.08001:5879 123.11521:34098 131.08374:14218 133.0986:40235 135.11642:22921 137.13219:20744 139.1123:8036 145.10277:9481 147.11679:46276 149.12999:24990 151.11298:8617 151.14888:6721 153.12355:9442 159.1172:9714 161.13194:8500 163.14491:11530 165.12289:6695 167.14172:11627 173.13148:10005 | -1.55431E-06 |
| POS6667 | 11Z,13E-eicosadie | 6.381 | 305.24673 | [M+H-H2O]+ | 305.24701 | 55.05365:79394 57.06988:25526 59.29884:5746 67.05389:119103 67.99528:5835 69.06868:64116 71.08547:8927 79.05312:122237 81.06833:99865 83.04769:7095 83.08509:29936 85.06505:8187 91.05389:50571 93.0684:210821 94.07142:7537 95.04694:7806 95.08476:84318 97.0646:6260 97.09953:11029 105.06944:42027 107.08485:116476 109.10005:33923 117.06912:8314 119.08332:28237 121.10129:84117 122.10348:6832 123.11816:8338 129.07021:9128 131.08376:15071 133.09862:23529 135.07935:6578 135.11644:35966 147.11681:5920 149.13393:7805 161.13196:9924 163.14493:15364 175.14502:6524 | -9.1729E-07 |
| POS3491 | 5,N6-Trimethyl-L- | 0.947 | 189.15912 | [M+H]+ | 189.15976 | 54.92054:6198 56.04952:8556 60.08024:117891 67.05389:17722 70.06555:7662 72.08047:36293 75.23055:6300 84.07956:389593 100.07381:21054 101.02003:12643 119.0303:17366 130.08388:48190 147.02486:6465 189.1548:5712 | -3.38338E-06 |
| POS7522 | Corticosterone | 5.231 | 347.2215 | [M+H]+ | 347.22168 | 55.05365:19614 67.05389:18172 69.03294:6617 69.06991:7063 79.05312:19613 81.06832:38824 83.04769:42664 87.04333:20494 91.05389:11120 93.0684:42854 95.04893:6980 95.08476:44260 97.0646:117671 99.04338:7680 101.0593:33397 105.06944:37506 107.04678:8476 107.08485:52010 109.06335:49831 109.10005:16612 111.07959:6791 117.06911:9748 119.08332:34740 121.06409:212080 121.09842:20296 122.06581:7709 123.08002:84590 129.07021:6978 131.08376:29713 133.09862:41665 135.07935:43567 135.11644:19583 137.09427:7193 143.08205:20364 145.09903:33114 147.07848:15714 147.11679:20820 149.0948:17200 149.13:8874 155.08327:8941 157.10196:23957 159.08273:7744 159.11722:27993 161.09682:11662 161.12756:7639 163.10913:21910 169.09859:11074 171.11404:28361 173.09726:9739 173.1315:14444 175.11017:12816 177.12296:7805 185.13042:9558 187.10989:7706 189.12688:7723 195.1138:6884 239.6185:6306 | -5.18401E-07 |
| POS1655 | Ornithine | 0.825 | 133.09673 | [M+H]+ | 133.09718 | 68.98124:113904 69.03294:17362 69.06992:16352 70.0643:2376008 71.06874:13911 86.09541:57886 86.99279:132144 87.99284:8170 105.0024:128956 115.08438:14438 116.06905:94262 123.01261:10207 | -3.38099E-06 |
| POS6503 | Ricinoleic acid | 6.287 | 299.2569 | [M+H]+ | 299.2579 | 53.44488:6044 53.60046:6452 55.05365:103513 57.06896:32207 65.17343:5370 67.05389:38333 69.06992:121672 71.08547:9832 79.05463:11767 81.06834:55123 83.08509:62298 91.0539:6481 93.03371:5882 93.06841:19233 95.08477:52134 97.06461:10400 97.09953:50898 105.06945:10980 107.08486:13158 109.10007:25022 111.0796:7293 111.11481:16280 119.08333:8449 121.09843:18795 123.11523:12748 125.09565:6603 133.09863:12192 135.11646:12812 138.47672:5490 149.13002:8029 161.13197:6575 | -3.3416E-06 |
| POS4624 | Isoleucylproline | 4.391 | 229.15327 | [M+H]+ | 229.15469 | 69.06992:30904 70.06431:274902 84.08123:12069 85.02631:5938 86.09541:299671 87.09918:11021 114.05201:9145 116.06905:293098 184.09612:8011 | -6.19669E-06 |

| Differences in metabolites between the Model group and the WJW group | | | | | | | |
|---|---|---|---|---|---|---|---|
| Alignment ID | Metabolite name | Rt(min) | Expreiment Mz | Adduct type | Reference m/z | MS/MS spectrum | PPM |
| POS8775 | Stearoylcarnitine | 5.752 | 428.37057 | [M+H]+ | 428.37338 | 57.03287:34494 57.06896:50800 58.06512:7169 59.04858:13497 60.08025:432255 67.05271:7761 71.08547:34924 81.0699:14444 83.08509:17433 85.028:2250157 85.10045:24065 86.03024:35076 89.05896:11687 95.08477:26836 97.09953:21863 109.10007:14300 123.11523:10734 144.10197:39277 417.58035:6609 | -6.5597E-06 |
| POS827 | Benzaldehyde | 4.382 | 107.04906 | [M+H]+ | 107.0495 | 51.02236:9759 56.98664:7080 64.62771:7117 77.03802:23213 79.05312:185056 91.05389:9238 95.04893:61454 105.04401:16870 | -4.11025E-06 |
| POS6456 | (10E,12Z)-9-HODE | 6.403 | 297.24136 | [M+H]+ | 297.24219 | 55.01769:6296 55.05364:38162 57.03286:6669 57.06895:22936 67.05388:68804 69.06866:25840 71.08546:240090 79.05311:21698 81.06989:73087 83.04768:6649 83.08508:18579 91.05389:20236 93.06839:25258 95.08475:55215 96.08826:7224 97.06459:7201 97.09951:6251 97.78535:5515 99.07937:96992 105.06944:17698 107.08484:16617 109.10004:20267 111.07958:6676 111.77932:5732 117.06911:6391 119.08331:12852 121.09841:20724 123.11521:6782 127.11191:10806 131.08374:10706 135.11642:11025 147.11679:46501 151.26068:5220 153.12762:6190 163.11359:6140 165.12289:10164 | -2.79234E-06 |
| POS6620 | Agallochin G | 6.296 | 303.22992 | [M+H]+ | 303.23169 | 55.0177:6720 55.05366:68190 57.06896:30413 67.05389:114621 69.06869:67332 71.04816:13639 71.08419:8715 79.05313:53904 81.06834:86305 83.0851:8859 85.06337:7039 91.0539:110946 93.06841:60655 95.04894:7086 95.08478:61261 101.05931:8071 105.06946:126382 107.08487:37954 109.10007:12901 117.06913:51039 119.08334:83118 121.0641:8589 121.1013:23180 122.82823:6861 123.11524:9333 129.07022:37621 131.08377:93146 133.09863:39958 141.06726:16166 143.08574:15768 145.09904:47485 147.11682:11172 149.09483:6003 155.08328:7919 157.09775:23872 159.11723:19473 161.13199:14486 169.09862:10174 171.11407:8517 173.13153:16413 173.38622:6334 175.14503:12140 183.11301:11010 197.13113:7165 201.12592:11322 | -5.83712E-06 |
| POS2554 | ninovaleric acid be | 1.024 | 160.1326 | [M+H]+ | 160.13318 | 53.03857:8319 55.05365:1155093 55.0633:28922 56.05674:13139 57.03287:10381 58.06416:57207 59.04858:237063 59.07295:29511 60.08025:840096 70.0643:50259 73.06432:8252 83.0477:172410 84.07957:32714 100.07382:12056 101.0593:460148 102.06162:8367 115.05257:20736 116.04757:7478 117.05553:12796 132.07892:12704 160.07417:29256 160.13072:166982 | -3.62199E-06 |
| POS1399 | Taurine | 1.128 | 126.02174 | [M+H]+ | 126.02198 | 53.03773:10197 55.05365:9503 56.04862:7360 66.53687:5755 68.0495:16281 70.0643:8011 78.98524:6630 80.04968:12502 84.07957:9237 98.05798:7485 108.00944:102751 108.9949:6815 109.07559:31331 126.02023:106282 | -1.90443E-06 |
| POS1800 | Trigonelline | 4.613 | 138.05453 | [M+H]+ | 138.0551 | 53.03773:15270 55.05365:10182 65.03805:11336 67.05389:27633 68.04829:14746 78.0336:69964 79.04105:37440 79.05162:11264 80.04968:8100 81.06834:51981 94.06554:6644 95.08477:21912 96.04379:228644 108.0432:39978 110.05836:12946 124.03867:25923 138.05421:307052 | -4.12879E-06 |
| POS1231 | Hydroxybenzonitr | 4.774 | 120.04428 | [M+H]+ | 120.04438 | 56.04952:7750 56.05764:13294 61.03972:18567 65.03804:121006 70.39166:5450 72.08047:8558 73.08444:14919 91.0539:6993 92.0491:83508 103.05421:17259 111.67029:5583 120.04357:20325 120.0803:7590 | -8.33025E-07 |
| NEG253 | mino-2-methylbu | 0.908 | 97.07714 | [M-H]- | 97.0772 | 79.05368:126619 80.05277:612254 94.18431:5963 97.07645:2942376 | -6.18065E-07 |
| POS9645 | oPE(18:2(9Z,12Z)/( | 6.153 | 478.29233 | [M+H]+ | 478.29269 | 51.34163:6667 55.05364:85019 55.21454:5493 57.03286:78181 57.06987:29910 62.06013:11470 67.05388:140674 69.06866:123864 71.08546:10086 79.05311:25298 81.06989:154548 83.08508:80852 85.06335:6763 87.29327:6183 91.05389:13545 93.06839:34365 95.08475:122181 97.06459:13284 97.09951:38596 105.06944:17190 107.08484:27814 109.10004:60872 111.1173:11633 119.0861:16510 121.10127:24420 123.11521:25988 133.0986:16699 135.11642:18706 137.13217:9333 147.11679:13918 149.1339:8839 161.13194:6938 162.26683:6997 163.14938:8087 | -7.52677E-07 |
| POS3818 | Dodecanamide | 6.145 | 200.19971 | [M+H]+ | 200.20081 | 55.05365:18749 57.06895:50255 69.06867:10385 71.08546:7644 74.06022:10785 75.65979:5727 81.06989:6977 88.07444:49109 102.09041:17858 116.10664:6624 124.89156:5987 148.61507:5308 200.19798:42427 | -5.49448E-06 |
| POS4316 | Propionylcarnitine | 1.092 | 218.13759 | [M+H]+ | 218.13869 | 57.03286:11116 60.08024:37522 61.03971:9927 84.07957:9433 85.02798:259449 | -5.04266E-06 |

| Differences in metabolites between the Model group and the WJW group | | | | | | | |
|---|---|---|---|---|---|---|---|
| Alignment ID | Metabolite name | Rt(min) | Expreiment Mz | Adduct type | Reference m/z | MS/MS spectrum | PPM |
| POS6404 | (Z),9(Z),12(Z)-octa | 6.243 | 295.22681 | [M+H]+ | 295.22672 | 55.01769:6971 55.05364:28988 57.03286:55182 57.06987:16117 62.27805:5648 67.05388:51800 69.06866:22390 71.08418:17836 79.05311:28301 81.06831:65954 83.04931:7079 83.08508:9164 91.05389:13807 93.06839:42394 95.08475:49425 99.07937:7131 105.06944:8780 107.08484:31933 109.06334:6201 109.10004:20091 119.08331:6609 121.10127:25600 133.0986:8134 135.11642:19060 186.14619:5502 226.35844:5011 | 3.0485E-07 |
| POS6403 | ,4'Z,8E)-Colneleic | 5.647 | 295.22653 | [M+H]+ | 295.2265 | 52.0545:5624 55.01769:9634 55.05364:34234 57.03286:12621 57.06895:8575 59.04857:8780 67.05388:39854 69.0699:28580 71.04943:7208 71.08546:7824 79.05311:24591 81.06831:37881 83.08508:8951 91.05389:11435 93.06839:30809 93.93243:6190 95.08475:32607 97.06459:9142 97.09951:9384 105.06944:8362 107.08484:28725 109.06334:6243 109.10004:14948 119.08331:11954 121.09841:17574 133.1019:8700 135.11642:10539 145.09901:5857 146.10268:6223 173.39598:9476 | 1.01617E-07 |
| POS3359 | midopropyl)pyrro | 4.424 | 185.12825 | [M+H]+ | 185.129 | 53.14906:6051 55.05365:12264 59.64061:5031 70.06429:11845 86.4879:5365 98.06005:86194 100.07381:18263 116.97124:6533 125.10766:20113 126.09013:189282 143.1188:8464 | -4.05123E-06 |
| POS6563 | (+)-Cedronellone | 6.077 | 301.21539 | [M+H]+ | 301.21619 | 55.05366:25368 67.05389:56577 69.06868:18458 71.04816:7128 79.05313:39738 81.0699:50896 85.06506:5491 91.0539:61031 93.06841:58806 95.08477:29793 100.07598:8951 105.06946:56484 107.08486:28652 109.10007:13815 117.06913:59324 119.08334:41936 121.1013:28848 129.06706:5995 131.08377:59156 133.09863:46173 135.11646:18568 143.08574:10898 145.09904:38449 147.11682:18107 157.10197:7542 159.11723:35770 161.13197:10943 171.11888:11544 173.13153:21280 | -2.6559E-06 |
| POS1790 | nethyl-1,3-buta-c | 6.298 | 137.13211 | [M+H]+ | 137.1324 | 67.05386:16876 79.0546:6534 81.06986:23444 95.08472:7924 | -2.11474E-06 |
| POS441 | 3-Methylpyridine | 8.783 | 94.0649 | [M+H]+ | 94.065 | 51.02237:7674 53.03774:81905 59.52111:55294 60.02129:6491 65.03917:8304 67.05389:24286 68.52579:13854 71.02888:55394 71.52904:10201 78.0336:16339 80.0343:12790 82.53645:13303 93.05684:11112 94.06359:527473 96.04379:51254 | -1.06309E-06 |
| POS1232 | Hydroxybenzonitr | 4.466 | 120.04428 | [M+H]+ | 120.0447 | 56.04953:7283 56.05674:11034 59.04956:10983 61.03869:30175 61.92805:6124 65.03804:142129 72.08047:7094 73.0831:14251 73.53333:6277 77.03802:14026 91.0539:30732 92.0491:98533 93.06841:16735 95.04893:27270 103.05421:118034 110.06084:7522 120.04357:20188 120.0803:65183 | -3.4987E-06 |
| POS4539 | zyldimethyl ammo | 6.524 | 226.15863 | [M]+ | 226.159 | 134.09351:18333 226.16113:12733 | -1.63602E-06 |
| POS5559 | Farnesyl acetone | 6.02 | 263.23538 | [M+H]+ | 263.2366 | 55.05365:13059 57.06896:12734 65.03692:8294 67.05389:23489 69.06991:10162 71.08547:9394 79.05312:9136 81.0699:28906 91.0539:7192 93.06841:14666 95.08477:22257 105.06945:8580 107.08486:11834 109.10006:9848 116.69191:5808 119.08333:9577 121.1013:6421 133.09863:8001 | -4.63461E-06 |
| POS2555 | iino valeric acid be | 1.373 | 160.1326 | [M+H]+ | 160.13318 | 55.05365:321601 58.06512:7390 59.04858:53601 60.08025:180335 64.59314:6062 70.06557:14093 83.04932:34086 101.0593:79347 116.04757:13912 132.07892:6734 160.07417:6735 160.13509:16309 | -3.62199E-06 |
| POS7318 | Subamolide C | 6.06 | 337.27145 | [M+H]+ | 337.2731 | 55.05366:32691 57.03287:20626 57.06989:10268 59.04956:6694 67.05389:48472 69.06868:26923 79.05313:13135 81.06834:40722 83.0851:14560 91.05204:7645 93.07034:9117 95.08477:30326 97.09953:7279 107.08486:10438 109.10007:15247 121.1013:6150 | -4.89218E-06 |
| POS13566 | Dianthosaponin C | 4.577 | 652.40558 | [2M+H]+ | 652.40552 | 77.99068:37795 87.01893:36948 87.04334:142321 89.05896:1047998 131.06766:38560 133.08543:452202 166.69713:42909 177.11285:79337 400.16666:38883 | 9.19673E-08 |
| POS12311 | Bilirubin | 4.692 | 584.67792 | [M+H]+ | 584.6781 | 73.02811:187672 73.06432:101464 80.05429:116505 87.04334:652252 89.05896:3177115 90.0619:109811 91.04457:60691 95.04893:140279 102.06606:66387 103.98616:58381 111.04441:87383 113.05927:81682 117.09089:58913 131.06766:103725 133.08543:1659876 134.08684:94500 137.05981:68955 155.0667:75801 175.09526:79543 177.11285:344461 210.66563:63524 | -3.07862E-07 |
| POS12919 | Puwainaphycin D | 4.706 | 613.35681 | [M+H]+ | 613.35699 | 73.02811:56583 87.04334:169634 89.05896:974096 90.0619:39072 102.56839:25109 113.05927:37699 117.20798:23434 130.75883:29066 133.08543:348689 177.11285:53095 202.06644:32968 | -2.93467E-07 |
| POS13499 | Lotusine E | 4.542 | 648.37427 | [M+NH4]+ | 648.37433 | 69.03294:40275 83.04769:18316 87.04333:116802 89.05895:355918 99.04338:82046 111.04189:38023 118.28912:15999 125.05659:19656 131.07086:18570 133.08543:149936 155.07082:41003 173.43523:19753 177.11284:22214 377.14182:16945 | -9.25391E-08 |

**Differences in metabolites between the Model group and the WJW group**

| Alignment ID | Metabolite name | Rt(min) | Expreiment Mz | Adduct type | Reference m/z | MS/MS spectrum | PPM |
|---|---|---|---|---|---|---|---|
| POS10612 | 180083-23-2 | 4.489 | 521.25366 | [M+H]+ | 521.25378 | 76.65031:7219 86.09541:8095 87.04334:12034 89.05896:183626 90.0619:14901 104.10696:28155 133.08543:76964 134.08684:9642 177.11285:11352 184.06929:12850 466.78955:6213 | -2.30214E-07 |
| POS14088 | an-2-yl)-17-oxa-1 | 4.554 | 692.4007 | [M+H]+ | 692.401 | 69.03294:34406 83.04932:19091 87.04334:79213 89.05896:243289 99.0434:58198 111.04441:37770 133.08545:97630 137.05981:16046 173.38622:28450 177.11285:22280 319.43127:17043 | -4.33275E-07 |
| NEG6553 | etramethylheptade | 0.899 | 323.29568 | [M-H]- | 323.29559 | 77.07738:6812 79.05368:18879 90.50151:5857 103.07947:75414 104.78513:5409 113.12115:24053 119.0854:12567 124.15677:13453 187.15546:156768 | 2.78383E-07 |
| POS9531 | olic acid glycine co | 4.473 | 472.27298 | [M+H]+ | 472.27301 | 69.03294:38778 73.02811:13523 73.06432:6329 83.0477:15500 87.04334:72463 87.09045:5532 89.05896:142651 93.32733:6502 95.04694:6716 99.04339:53482 107.07059:5835 111.04189:35667 122.36179:5627 123.80167:6072 125.0596:10348 131.06766:7048 133.08543:49732 155.07083:13830 333.67181:5748 | -6.35226E-08 |
| POS13624 | beta-D-glucopyrar | 4.717 | 657.38458 | [M+Na]+ | 657.3844 | 73.02678:32745 87.04334:110396 89.05896:562632 90.06374:31628 95.04893:28345 129.8802:24921 133.08543:277951 177.11285:50766 373.06967:24111 | 2.73812E-07 |
| POS9326 | BW A868C | 6.157 | 460.28043 | [M+H-H2O]+ | 460.28049 | 50.52047:6237 56.0153:6455 173.39111:6724 | -1.30355E-07 |
| POS5433 | ofumesate-2-hydr | 0.88 | 259.06332 | [M+Na]+ | 259.06339 | 75.5243:6317 80.34119:5069 119.49465:5860 150.53221:5378 153.20093:6466 191.07452:69247 | -2.70204E-07 |
| POS1644 | THTC | 1.146 | 133.03159 | [2M+H]+ | 133.03169 | 53.03856:10928 55.05365:15188 56.04952:34333 57.05692:121539 58.06416:77048 58.99405:15342 59.04858:12006 61.01003:54938 67.05389:18755 69.06867:1093632 70.06934:24785 70.07311:93038 73.06432:9416 86.0954:2214347 87.0259:32303 87.09917:177128 90.05456:13203 | -7.51701E-07 |
| POS7250 | Sarcostolide G | 5.29 | 333.20493 | [M+H]+ | 333.2049 | 72.03056:5233 93.06841:5945 97.06461:14230 107.08486:8269 109.06336:8815 114.88049:5409 121.06409:38398 123.08003:13336 147.11681:7411 180.61118:6398 245.93018:5880 324.12939:6920 | 9.00347E-08 |
| POS7280 | dro-15-keto Prost | 5.614 | 335.21899 | [M+Na]+ | 335.21899 | 51.63123:6052 57.83962:6671 76.67337:5677 79.88229:5885 95.08477:6673 109.49762:5725 | 0 |
| POS3471 | 4-Chlorobiphenyl | 1.023 | 189.04648 | [M+H]+ | 189.04649 | 55.17931:6214 58.06416:19840 60.08024:33413 70.06429:25884 72.04369:29659 72.08047:435680 73.08309:21275 83.00869:10138 84.07957:203678 100.07381:216677 101.01785:73082 101.07893:15345 112.11002:5731 117.10176:10318 118.04636:10666 119.0303:116327 126.09013:8868 129.01353:10243 130.08388:20478 136.05563:5951 137.03912:40195 147.02486:57206 161.04852:7674 165.03636:13179 171.14771:6036 | -5.2897E-08 |
| POS6976 | Tomelukast | 6.311 | 319.17535 | [M+NH4]2+ | 319.1752 | 55.0177:30555 55.05365:23184 57.03287:28828 64.85594:6493 67.05389:24674 69.06868:117672 69.80681:5353 71.04816:10347 79.05312:25104 81.06833:19993 91.0539:35686 93.06841:24821 95.04893:5465 95.08477:9970 97.06461:13405 105.03245:10543 105.06945:39949 107.04917:42940 107.08486:8338 107.37579:5076 117.06912:14013 117.28432:6209 119.08333:29494 121.1013:9240 122.76101:6073 131.08377:5982 133.09863:15446 135.11644:13885 139.11232:14733 143.08574:9419 145.09904:11977 147.07849:24923 161.09683:20508 163.10915:28111 165.09103:7358 206.39116:6086 | 4.69961E-07 |
| POS10232 | Lys Glu Asn Asp | 4.366 | 505.22525 | [2M+H]+ | 505.22519 | 60.04425:13615 72.08047:24447 89.88777:6027 115.0499:6708 118.08492:8113 129.1017:13294 154.0515:7791 175.10519:15609 189.08778:5882 | 1.18759E-07 |
| POS7539 | Mukoenine B | 6.445 | 348.19626 | [M+Na]2+ | 348.19635 | 57.03287:36244 59.67723:6330 74.65649:6367 252.33946:5698 | -2.58475E-07 |
| POS7516 | Argophyllin C | 4.821 | 347.18448 | [M+H]+ | 347.18451 | 53.47846:5443 90.58919:5692 102.95767:6152 105.06945:5447 113.36972:5229 121.06409:29767 170.89314:5683 | -8.64094E-08 |
| POS8931 | Nudicaulidine | 6.363 | 438.28555 | [M+2H]2+ | 438.2854 | 51.01141:6861 57.03287:7264 59.04858:31380 338.36777:6055 | 3.42243E-07 |
| POS7490 | or-9-carboxy-Del | 5.167 | 345.20386 | [M+H]+ | 345.20389 | 55.05364:8745 57.03286:10758 57.06987:8631 59.04857:156108 60.08023:24029 67.05388:7682 73.0281:9297 79.05311:9225 81.06989:15609 83.04768:7036 85.02798:158970 86.03194:17410 87.04333:10279 89.05894:49265 93.06838:15565 95.08475:14207 97.06458:10748 101.05928:12799 103.07442:9886 105.06943:6111 107.08484:16588 121.06407:72357 123.08001:6829 131.08696:8149 133.1019:9366 135.07932:10998 143.08571:6090 144.25438:6320 145.10277:9176 147.07846:12472 155.08324:6498 157.10194:7401 163.1091:6871 173.09724:13813 297.94568:5678 | -8.69052E-08 |
| POS9182 | Melleolide M | 5.746 | 453.16736 | [M+H]+ | 453.16739 | 57.03286:72567 57.94083:5677 59.04858:32735 62.03075:6005 85.02798:7600 99.07938:8882 | -6.62007E-08 |
| POS9465 | 28-Norbrassinolide | 6.426 | 467.33469 | [M+2H]2+ | 467.33459 | 57.03378:8878 57.53098:5601 59.04858:61853 87.04333:8149 122.83405:6319 128.02786:5926 173.42542:6550 | 2.13979E-07 |

| Differences in metabolites between the Model group and the WJW group | | | | | | | |
|---|---|---|---|---|---|---|---|
| Alignment ID | Metabolite name | Rt(min) | Expreiment Mz | Adduct type | Reference m/z | MS/MS spectrum | PPM |
| POS11311 | , 24E)-Cholest-24 | 6.26 | 547.2934 | [M+2H]2+ | 547.29352 | 52.92344:5911 60.08025:8338 86.09541:12656 104.10696:89387 105.10879:9411 184.07466:15685 308.05939:6095 | -2.19261E-07 |
| POS7167 | naH-cheilantha-13 | 2.593 | 329.32031 | [M+H]+ | 329.32019 | 51.93132:6968 55.05365:11566 57.06896:23455 59.03105:90371 61.01003:62247 62.98918:11778 67.05389:7884 69.06992:13425 71.08419:13075 73.04555:73611 75.02498:108072 76.02578:6615 77.00463:7738 77.04093:83916 79.01993:16467 83.0851:9873 87.06253:6298 91.05577:87400 91.52596:5809 93.03564:90181 95.01511:5977 97.09953:15795 99.86346:5875 105.07177:29441 111.11732:14346 119.08891:6894 149.44341:5671 | 3.64387E-07 |
| POS3963 | -methyl-2-buteny | 6.297 | 205.1212 | [M+H]+ | 205.1221 | 69.90211:5326 72.95045:5817 91.0539:9489 | -4.38763E-06 |
| POS2609 | (R)-Boschniakine | 4.733 | 162.09058 | M+CH3OH+H] | 162.0905 | 55.80161:6038 80.0266:7379 103.05195:17483 120.08028:56350 | 4.93551E-07 |
| POS15090 | PG 38:7 | 6.733 | 793.5011 | [M+H]+ | 793.50128 | 55.14588:6653 60.88638:5143 66.33102:5834 78.19966:6200 143.06367:6466 160.10027:5317 243.58643:6061 359.39038:5954 611.99011:5960 | -2.26843E-07 |
| POS6562 | 4-Oxoretinol | 6.339 | 301.21536 | [M+H-H2O]+ | 301.21539 | 55.0177:11256 55.05365:7648 69.06867:18526 81.06832:6768 95.00317:5636 177.55406:6154 | -9.95965E-08 |
| POS13939 | -(28),23-dien-3-oi | 8.716 | 679.47772 | [M+H]+ | 679.47791 | 53.85504:6025 129.86432:5667 529.3476:5951 | -2.79626E-07 |
| POS14521 | Javanicoside K | 7.456 | 729.29626 | [M+H]+ | 729.29639 | 53.35354:5571 | -1.78254E-07 |
| POS6668 | 3-Oxogeranyllinal | 6.936 | 305.24673 | [M+H]+ | 305.2467 | 52.71404:6652 115.55512:5844 | 9.82812E-08 |
| POS3956 | Eriosematin F | 2.594 | 205.08528 | M+CH3OH+H] | 205.0854 | 55.93435:11112 57.06989:7322 60.04327:6456 109.32312:5950 149.02449:11007 | -5.85122E-07 |
| POS4735 | Alantolactone | 6.299 | 233.15237 | [M+Na]+ | 233.1524 | 152.50285:5564 | -1.28671E-07 |
| POS5496 | -octadeca-5,9,12- | 5.769 | 261.21991 | [M+H-H2O]+ | 261.22 | 67.05388:6690 91.05389:6161 | -3.44537E-07 |
| POS428 | Toluene | 4.381 | 93.06992 | [M+H]+ | 93.0698 | 57.03286:10207 70.25361:5628 95.04694:8249 | 1.28935E-06 |
| POS7916 | Cortol | 5.88 | 369.26291 | [M+H-H2O]+ | 369.26291 | 67.05388:11127 69.0699:11500 81.06989:13595 95.08475:10779 110.42126:6159 121.09841:8099 147.11679:7654 180.52777:6221 | 0 |
| POS7956 | PARTEINE SULFAT | 5.765 | 371.1701 | [M+H]+ | 371.1702 | 76.54377:5973 173.39111:6509 182.17998:5526 | -2.69418E-07 |
| POS3082 | 9E)-Trideca-3,5,7,9 | 6.409 | 177.16322 | [M+H-H2O]+ | 177.1633 | 55.05366:7912 81.0699:6023 83.68195:5881 88.02122:6673 92.61692:5662 93.06841:9043 102.63982:5740 107.08486:6140 121.1013:12055 | -4.51561E-07 |
| NEG1489 | L-LYSINE | 6.807 | 145.09785 | [M-H]- | 145.09801 | 52.09783:7013 84.031:9349 100.04588:44311 101.05439:1161267 102.05608:101844 116.05926:45095 117.06681:15819 118.18125:15751 | -1.1027E-06 |
| POS337 | L-Alanine | 4.392 | 90.05499 | M+CH3OH+H] | 90.055 | 61.02742:77191 64.32306:6811 69.61306:6290 72.08047:8526 | -1.11043E-07 |
| POS3147 | xy-5-phenyl-3-pe | 6.299 | 179.10619 | [M+NH4]+ | 179.1062 | 91.05389:11793 105.06944:10473 119.08332:10252 | -5.58328E-08 |
| POS3367 | -2,2,6-trimethyl-1 | 6.467 | 185.15295 | [M+H]+ | 185.153 | 50.34662:5815 57.06896:31669 58.9425:10792 83.08509:6768 91.78413:5392 96.43512:5643 114.02061:6970 116.97125:33797 117.97753:35894 139.9868:8151 | -2.70047E-07 |
| POS1258 | Hydroxybenzoic a | 6.414 | 121.02811 | [M+H]+ | 121.028 | 51.98441:6202 52.08194:5974 65.03805:30519 86.61761:5929 120.37191:5659 | 9.08881E-07 |
| POS151 | al\|Nitrile-(()-3-Hyd | 0.618 | 84.04436 | [M+H]+ | 84.0443 | 55.05365:16638 56.04861:115979 84.04314:90317 84.07956:9447 | 7.13909E-07 |
| POS7648 | Grandilobatin A | 6.612 | 353.26736 | [M+H]+ | 353.2674 | 67.05388:6123 91.09308:6433 107.08484:7637 119.67999:6221 140.46465:5768 | -1.13229E-07 |
| POS4854 | Palmitoleic acid | 6.324 | 237.22009 | [M+H-H2O]+ | 237.22 | 69.06869:6628 81.0699:9704 107.08487:6414 232.43169:6150 | 3.79395E-07 |
| POS330 | Lactamide | 1.48 | 90.05469 | [M+H]+ | 90.05478 | 61.02742:529022 62.03075:13726 72.08047:8393 76.49201:5079 78.29926:5515 | -9.99392E-07 |
| POS7699 | -Linoleoyl Glycero | 6.877 | 355.28223 | [M+H]+ | 355.2821 | 101.92889:5706 142.24037:5452 153.16835:5057 173.43524:8239 | 3.65906E-07 |
| POS10736 | | 4.66 | 525.30688 | [M+H-H2O]+ | 525.30713 | 69.03294:67522 73.02811:187355 73.06432:73247 80.05429:75077 81.03385:33790 83.0477:34944 87.04334:431912 88.04604:27180 89.05896:2465756 90.0619:86112 91.04643:88865 95.04893:88450 99.04339:69196 102.06606:66841 107.06821:25147 111.04441:100871 113.05927:70640 117.08817:29401 124.08022:23947 131.06766:68756 133.08543:860051 134.08684:45384 137.0598:61376 155.07083:33039 177.11285:91886 | -4.75912E-07 |
| POS14851 | xy-12,19(29)-ursad | 4.666 | 765.43976 | [M+2H]2+ | 765.44 | 59.04858:189374 69.03294:107946 73.02811:457562 73.06432:231440 81.03229:104140 87.04334:1581831 89.05895:7716564 90.06374:158789 91.04643:139834 95.04893:226545 99.04339:207252 109.06335:110190 111.0444:307058 113.05927:264513 117.09089:189153 131.06764:339911 133.08543:4354042 134.09016:181612 137.0598:223439 146.80688:80855 155.07083:299185 175.09526:154709 177.11284:864362 178.11432:109079 199.09537:129018 221.14047:110403 243.12985:79798 | -3.13545E-07 |
| POS15066 | )kadaic acid (-H2O | 4.657 | 787.46161 | [M+2H]2+ | 787.46198 | 64.15166:115119 87.04332:165423 89.05893:744046 133.0854:434553 282.94159:115379 783.29553:120371 | -4.69864E-07 |
| POS10416 | e from Mulder Bioc | 5.345 | 513.34473 | [M+2H]2+ | 513.34497 | 57.03284:27899 59.04856:448896 60.05223:12018 69.06988:6563 73.02808:29925 73.06429:11442 85.06501:14269 87.0433:62729 87.07994:11068 89.05891:367724 90.06187:9711 94.06942:10331 101.05926:60205 103.07439:106553 104.10692:6321 115.07638:11671 117.09084:7292 129.08905:9081 133.08537:99502 147.10141:45184 191.1255:7629 333.50156:7036 379.29004:6238 | -4.67522E-07 |

**Differences in metabolites between the Model group and the WJW group**

| Alignment ID | Metabolite name | Rt(min) | Expreiment Mz | Adduct type | Reference m/z | MS/MS spectrum | PPM |
|---|---|---|---|---|---|---|---|
| POS11896 | Cinnamoyl-vulgaro | 4.745 | 567.33191 | [M+Na]+ | 567.3316 | 58.32636:6288 87.04335:6304 89.05896:14008 101.41589:5569 147.08617:5377 148.92294:5751 173.39111:10389 192.58664:6191 392.82523:6321 503.2316:5948 | 5.46418E-07 |
| POS12097 | ethyl]-10,14,16,16 | 4.692 | 575.00769 | M+CH3OH+H] | 575.00739 | 73.02811:29511 87.04334:93472 89.05896:469726 90.0619:14744 95.04893:13062 99.04339:14744 99.79063:13882 113.05927:17085 131.07088:13450 133.08543:160500 177.11285:21906 366.98584:13541 | 5.21732E-07 |
| POS6773 | 6-Hydroxynobiline | 4.717 | 310.20068 | [M+Na]+ | 310.2009 | 55.05452:9349 57.09766:6312 59.04858:12269 60.08024:15333 85.02798:148494 93.06839:6424 121.10128:7372 131.23537:7547 | -7.09218E-07 |
| POS9402 | LysoPE(18:0/0:0) | 6.085 | 464.31125 | [M+H-H2O]+ | 464.311 | 57.03286:12124 59.04955:8892 342.63168:5391 | 5.38432E-07 |
| POS15383 | ,13-dimethylhexa | 7.329 | 1009.69946 | [M+H-H2O]+ | 1009.70001 | 50.41728:4625 51.16747:4355 67.85581:5258 68.19077:5133 69.09335:4538 69.98:5230 73.86624:4573 87.78227:5144 87.80347:4915 89.77258:4853 102.61972:5708 109.25687:4829 109.90973:5719 111.15759:5039 112.63448:5961 115.85182:6537 175.85406:6312 252.27922:8560 | -5.44716E-07 |
| POS2821 | -ureidobutyric aci | 1.049 | 169.05814 | [M+H]+ | 169.05832 | 52.11343:6093 55.02822:24863 67.05389:8183 68.7358:5188 69.00831:10412 70.03912:49662 79.05312:7897 81.06989:12314 96.0175:22582 98.03503:31566 114.06246:6020 116.72981:5296 124.01196:8099 126.02934:37045 141.03847:76945 151.04518:12385 152.00658:21390 169.0325:59061 | -1.06472E-06 |
| POS10245 | Yardenone A | 7.332 | 505.35059 | [M+2H]2+ | 505.35031 | 173.3911:17792 | 5.54071E-07 |
| POS5564 | Z)-Octadecadieno | 6.288 | 263.23578 | [M+H-H2O]+ | 263.23599 | 55.05364:9969 57.06895:5869 62.58315:5822 67.05388:24081 69.0699:7106 81.06831:24764 83.08508:6428 93.06839:10323 95.08475:20730 107.08484:6287 109.10004:13846 121.10127:6250 193.25415:5595 | -7.97763E-07 |
| POS10783 | -Indocarbazostati | 1.459 | 527.15625 | [M+H]+ | 527.15601 | 55.55447:5626 127.59034:5621 144.60855:5867 163.10468:6399 | 4.55273E-07 |
| POS2428 | hosphoglycolic ac | 8.819 | 156.98994 | M+CH3OH+H] | 156.98959 | 52.57622:5798 53.93831:20328 55.0177:6849 55.05365:18289 56.04952:11259 57.9342:16022 70.0643:8385 70.94153:8906 72.93706:21304 73.93719:8762 74.93712:198919 84.07957:7913 86.82748:5832 90.94576:6888 92.94704:32875 97.0646:12623 110.05835:7187 112.97669:6802 113.96311:7086 115.95633:28188 115.96169:84824 116.97124:8238 128.06833:6180 133.97353:17542 | 2.22945E-06 |
| POS3719 | Lactarazulene | 6.298 | 197.13197 | [M+Na]+ | 197.1322 | 68.17625:5403 80.64205:5329 100.70676:5930 127.82278:5754 136.63005:5533 161.47514:6497 167.6394:5486 | -1.16673E-06 |
| POS1881 | Na-Dimethylhistan | 8.768 | 140.11795 | [M+Na]+ | 140.1181 | 52.81611:7314 53.03773:8905 54.03287:5940 55.02822:65891 58.06416:292029 69.06868:37091 70.0643:46062 72.08047:17138 80.04967:6099 83.05907:65928 87.20751:5633 88.63091:6419 97.07487:7111 98.083:7589 123.09176:6408 | -1.07053E-06 |
| POS5077 | 10,11,12,14,15,16,1 | 6.289 | 245.22614 | [2M+H]+ | 245.2263 | 57.05414:6045 59.16666:5253 67.25835:6028 85.66637:6272 113.07216:5864 125.9018:5442 132.26817:5515 214.60085:5742 | -6.52459E-07 |
| POS1025 | l-imidazol-5-yl)et | 6.475 | 113.07066 | [M+H]+ | 113.0709 | 54.03372:77373 59.92951:49646 61.927:7501 67.05389:20078 69.04403:443480 70.02779:36752 70.0643:9931 71.05973:242969 72.06339:12234 73.56719:6193 86.94053:6018 89.00122:68090 89.50283:10153 89.99953:24537 94.06554:8025 96.0074:17692 98.00586:131554 98.50787:22995 99.00529:32533 100.50954:32851 101.50806:6877 107.01112:12740 109.51484:20907 110.51353:8603 113.03345:19640 113.06959:128467 | -2.12256E-06 |
| POS12824 | Malyngamide J | 4.571 | 608.3797 | [M+NH4]+ | 608.37927 | 73.06432:37063 87.04334:182952 89.05896:1542307 90.0619:39641 117.09089:19249 131.07088:54281 133.08543:670589 134.08684:26788 177.11285:88397 553.66919:19689 | 7.06796E-07 |
| POS8912 | Drenison | 4.493 | 437.233 | [M+Na]+ | 437.23331 | 52.80128:6734 68.74682:5393 246.60245:5675 301.61307:5685 | -7.09004E-07 |
| POS12839 | ctyl)-1,4,7,10-tetr | 4.523 | 609.30444 | [M+H]+ | 609.30487 | 68.41991:7554 87.04333:35840 89.05895:261702 90.0619:17221 116.29226:8484 133.08543:93186 134.09016:11636 177.11284:16533 580.45636:8024 | -7.05722E-07 |
| NEG2413 | ,2,7,8-Octanetetro | 0.924 | 177.11366 | [M-H]- | 177.1133 | 74.1126:10026 93.03925:30520 128.69928:6126 161.08009:5411 | 2.0326E-06 |
| POS5264 | Ribose 5-phospha | 1.199 | 253.00798 | [M+H]+ | 253.00832 | 61.03971:9646 68.36646:5653 68.68076:5543 87.04333:5534 104.36064:5765 120.96402:9389 149.96265:5262 | -1.34383E-06 |
| POS8621 | EnP(5,8) | 5.227 | 417.33533 | [M+H-H2O]+ | 417.3356 | 57.03286:18918 59.04858:302286 73.02811:18571 81.06832:25269 87.04333:25195 89.05895:135926 99.07938:32106 101.0593:16746 103.07443:36156 107.08485:7418 109.10005:8718 130.55034:6076 133.08543:19907 147.10147:9836 159.40642:5982 173.43523:10533 196.30745:5690 416.92136:6788 | -6.46961E-07 |
| POS4936 | Pirbuterol | 4.42 | 241.15355 | [M+H]+ | 241.1539 | 58.06511:13072 69.03294:8028 70.0643:11751 84.07957:7558 95.08477:10598 98.06007:17179 110.09556:5558 134.09351:8895 138.08907:38755 | -1.45136E-06 |

| Differences in metabolites between the Model group and the WJW group | | | | | | | |
|---|---|---|---|---|---|---|---|
| Alignment ID | Metabolite name | Rt(min) | Expreiment Mz | Adduct type | Reference m/z | MS/MS spectrum | PPM |
| POS13628 | Parisin | 6.429 | 657.49316 | [M+H]+ | 657.49353 | 57.03286:138037 58.03661:6859 58.22031:5838 59.04858:35475 78.02471:7108 99.07938:15568 115.07377:8433 126.9121:6505 126.93359:6413 140.10425:5512 145.29819:5482 157.11888:14588 346.10397:5472 461.44281:6575 652.27557:5618 | -5.62743E-07 |
| POS7300 | Pipericine | 6.74 | 336.32523 | [M+H]+ | 336.3255 | 50.50196:6109 112.50879:5338 156.31415:6028 218.8768:6308 | -8.02794E-07 |
| POS5044 | -11H-dibenzo[b,e | 0.877 | 245.0472 | [M+Na]+ | 245.0475 | 51.55165:7066 52.56477:6209 53.31757:6191 65.14406:6541 65.15309:6011 69.5333:5667 177.06224:34769 224.77242:5997 | -1.22425E-06 |
| POS3846 | Lacinilene A | 6.296 | 201.12679 | [M+H-H2O]+ | 201.12711 | 64.187:5540 66.59401:6007 91.05389:9508 105.06944:6709 117.06911:7199 129.06705:13625 131.08376:29337 145.10278:14647 157.10196:18799 173.10216:6734 173.396:8191 | -1.59103E-06 |
| POS9643 | ylphenylalanylarg | 6.193 | 478.28867 | [M+H]+ | 478.28839 | 54.62195:6208 62.57891:5577 134.27377:6357 397.29398:5879 | 5.85421E-07 |
| POS6641 | Fenpropimorph | 0.898 | 304.26031 | [M+NH4]+ | 304.26059 | 52.64424:6420 73.14485:5706 115.68862:6183 306.7052:6369 | -9.20264E-07 |
| POS1062 | i-Dimethylpiperidi | 4.488 | 114.12732 | [M+H]+ | 114.1276 | 53.03857:12167 54.03373:11727 55.0177:189439 55.05365:462522 58.02808:15578 67.05389:110907 68.04829:98581 69.06868:682557 70.06431:86380 71.04816:32870 71.08547:39319 72.08047:69497 77.03802:27084 79.05312:650159 81.05579:13126 84.07957:14653 86.09541:46098 95.04893:44642 96.08018:307102 97.06461:103540 105.04401:13777 114.09126:1768146 115.09234:18092 | -2.45339E-06 |
| POS5387 | 3-Deoxyestradiol | 6.297 | 257.18881 | [M+NH4]+ | 257.18909 | 57.53942:7335 57.62482:5391 83.3492:5597 142.84343:5835 | -1.08869E-06 |
| POS7211 | Sclareol | 6.5 | 331.26044 | [M+H]+ | 331.26071 | 55.05365:16712 57.06988:10107 65.03015:5403 67.05389:23387 69.06867:18376 79.05312:10309 81.06832:16159 83.08508:6287 91.05389:19068 93.0684:13151 95.08476:16426 105.06944:38936 119.08332:19632 121.10129:9555 131.08376:16476 133.10191:9633 145.10278:7045 155.82838:5614 | -8.15068E-07 |
| POS2187 | -Tetrahydro-2-na | 6.3 | 149.09537 | [M+H]+ | 149.0957 | 99.37657:5830 103.77468:5501 | -2.21334E-06 |
| POS1414 | SCHEMBL1137702 | 1.639 | 126.091 | M+CH3OH+H] | 126.0913 | 53.03856:20679 55.0177:13306 56.04952:7110 70.06556:11281 80.04813:21980 81.03228:8774 98.05797:15555 108.04319:12716 111.99284:5768 126.09013:7626 127.03809:12474 | -2.37923E-06 |
| POS7966 | conessine | 1.238 | 371.34229 | [M+H]+ | 371.34201 | 57.06989:9314 59.03105:42755 61.01003:14827 71.08548:6140 73.04555:31922 75.02498:34684 77.04093:20410 91.05577:23723 93.03564:23681 105.07177:9840 237.84406:5418 314.16376:6156 | 7.54022E-07 |
| POS9711 | 2-ylanilino)propar | 4.506 | 481.26016 | [M+Na]+ | 481.25977 | 133.00633:5343 173.39601:10702 190.73463:6381 353.4512:6282 | 8.10373E-07 |
| POS4239 | Citric acid | 1.219 | 215.01562 | [M+H-H2O]+ | 215.01601 | 52.46179:6029 69.16247:5332 72.08047:8938 75.66121:6218 81.0699:19912 88.72058:5424 95.08477:6798 | -1.81382E-06 |
| POS9324 | yptophylvalylargini | 5.752 | 460.2662 | [M+H-H2O]+ | 460.2666 | 59.04858:13547 79.6314:5536 89.05895:13544 119.08332:58896 135.07935:9482 152.16771:5365 173.38618:5401 | -8.69062E-07 |
| POS13937 | DTXSID60346618 | 8.778 | 679.47156 | [M+H]+ | 679.47198 | 65.3966:5920 198.67972:6253 272.09207:5880 290.05417:6865 | -6.18127E-07 |
| POS2031 | Ethephon | 6.504 | 144.9819 | [M+H-H2O]+ | 144.98151 | 53.03773:13579 55.01682:9119 55.05365:10583 57.03286:6811 62.92802:1139831 70.0643:47409 71.06874:6732 80.93837:9232 98.06006:32443 98.09552:15845 99.04338:23092 103.95428:45847 121.96451:462033 131.96162:6944 144.98274:31577 | 2.69E-06 |
| POS370 | p-Cresol | 4.389 | 91.05412 | [M+Na]+ | 91.0537 | 51.59062:7082 57.30673:5757 57.93421:6222 62.03075:6327 65.03805:19051 66.99615:6298 91.0539:28271 | 4.61266E-06 |
| POS7164 | Incensole | 6.838 | 329.24548 | [M+H]+ | 329.24512 | 76.35284:6858 107.08486:6808 170.07565:5289 173.3911:10170 328.86789:6140 | 1.09341E-06 |
| POS6401 | droxyhexadecano | 5.946 | 295.2247 | [M+H]+ | 295.22433 | 59.04857:7603 67.05388:19576 69.06866:9868 79.05311:13119 81.06831:20876 83.08508:14072 91.05388:6351 93.06838:13704 95.08475:14161 107.08484:9073 121.0984:15495 135.11641:11977 165.01813:6026 258.12753:6279 258.19879:5911 275.03635:6959 | 1.25328E-06 |
| POS1349 | 2,4-Triaminobenze | 8.729 | 124.08641 | [M+H]+ | 124.0868 | 61.37498:5859 77.49311:6051 96.20368:6753 | -3.14296E-06 |
| POS10583 | Tenacibactin C | 4.524 | 520.33051 | [M+NH4]+ | 520.33002 | 73.06432:28839 87.04334:132120 89.05896:1572119 90.0619:33301 91.07443:10618 104.10696:19802 107.06821:11937 117.08817:18443 131.06766:32650 133.08543:696134 134.08684:18707 144.3028:8212 173.3813:8593 175.09526:11748 177.11285:97387 197.86429:8397 | 9.4171E-07 |
| POS9624 | Loperamide | 4.491 | 477.23071 | [M+H]+ | 477.23029 | 72.08047:7460 73.06432:10341 86.05939:12046 86.09541:6800 87.04334:24231 89.05896:357135 90.0619:24425 98.06007:6841 112.07434:29439 119.08612:16395 120.0803:10075 133.08543:163641 134.08684:13166 136.52037:6027 157.00053:5902 177.11285:22950 291.63074:5845 | 8.80078E-07 |
| POS12102 | brujavanone D | 4.69 | 575.35828 | M+CH3OH+H] | 575.35779 | 59.04859:11804 64.65227:7554 70.12607:6071 73.02812:13099 79.72459:6653 87.04335:34803 89.05896:179418 94.8423:6089 100.51604:5904 103.07444:6306 111.04441:6606 133.08545:69356 177.11285:11363 189.74847:6204 243.21132:6574 | 8.51644E-07 |

**Differences in metabolites between the Model group and the WJW group**

| Alignment ID | Metabolite name | Rt(min) | Expreiment Mz | Adduct type | Reference m/z | MS/MS spectrum | PPM |
|---|---|---|---|---|---|---|---|
| POS13415 | Cyclosquamosin A | 4.367 | 642.32507 | [M+H]+ | 642.32452 | 78.48708:5382 110.16753:6001 118.88821:6000 120.0803:11004 129.1017:14690 284.55325:5294 340.97604:5526 | 8.56265E-07 |
| POS1661 | minopropoxy)guar | 2.2 | 133.10878 | [M+H]+ | 133.10831 | 55.01769:9547 55.05364:22978 56.04951:32865 57.05691:117812 58.0651:67808 59.04856:11327 67.05387:14182 69.06989:1012020 70.0731:111514 73.0281:9696 73.0643:16475 86.09538:3664538 87.09916:413410 | 3.53096E-06 |
| POS8648 | ptan-2-yl)-10,13-( | 5.825 | 419.35056 | [M+NH4]+ | 419.35101 | 51.32425:6511 53.51543:5580 57.03286:8346 59.04858:18139 59.19405:6231 61.50322:5552 69.06991:15310 81.06832:74198 93.07033:7041 95.08476:13841 99.07938:92604 107.08485:5748 109.10005:24396 118.48554:6358 121.10129:10560 127.11192:42138 128.49895:5709 135.11644:16540 147.11679:11189 149.13:8978 161.13196:9149 177.72186:5742 | -1.07309E-06 |
| POS6069 | Helipandurin | 1.193 | 283.0184 | [M+Na]+ | 283.01889 | 55.05365:23136 55.37881:6720 57.06896:22459 61.0387:36270 67.05389:8412 69.06868:26057 71.08547:8629 81.0699:10292 83.08509:15330 93.06841:6457 95.08477:9999 97.09953:15408 109.10006:8774 110.8261:5504 111.11732:6136 121.1013:6617 162.05843:6212 201.10139:6503 276.58087:4927 | -1.73133E-06 |
| POS4658 | aureothricin | 1.204 | 231.02509 | [M+H]+ | 231.0256 | 51.60494:4860 52.34609:5040 52.40226:4955 53.57602:5516 55.51713:4676 59.27923:3988 59.67426:4210 61.0561:4726 61.12378:4139 61.50322:4601 64.35188:4912 70.01521:4499 71.72692:4475 71.8471:4437 79.13621:4928 82.53001:4615 86.48445:4756 90.88618:4138 91.6992:4865 100.39278:4164 101.92226:4489 102.37012:4400 103.79739:5268 105.51961:3989 106.1909:4007 106.89709:4513 119.68001:3814 125.68365:4764 141.86584:4666 158.9062:4620 159.09998:4177 159.43236:4009 161.65155:5065 162.84996:4407 168.66025:4075 180.60074:5156 215.51527:4641 | -2.20755E-06 |
| POS6750 | Tripentyl phosphat | 6.215 | 309.21936 | [M+H]+ | 309.21887 | 55.05365:6885 59.04858:8074 67.05389:7179 70.24855:5875 72.41546:5413 81.06989:6388 97.10158:7561 138.85191:5487 173.396:6944 256.55826:5440 | 1.58464E-06 |
| POS12620 | active peptide-1\|S( | 4.7 | 599.3504 | [M+H]+ | 599.35107 | 69.03294:38710 73.02811:181858 73.06432:104448 87.04334:467806 89.05896:2369354 90.0619:123719 91.04644:54510 95.04893:88861 99.04339:48918 102.06606:71614 111.04189:82690 113.05927:108419 117.09089:38395 131.06766:90357 133.08543:1302579 134.09018:66776 137.05981:60868 146.55127:41191 155.07083:76725 159.97418:38949 177.10779:196456 | -1.11788E-06 |
| POS6633 | 4,5-dimethyl-2-ox | 4.384 | 304.17487 | M+CH3OH+H] | 304.17542 | 51.44688:6234 57.03287:16040 58.06511:8521 60.08025:219606 69.06868:93746 70.02779:12022 70.0643:6306 73.06432:25998 81.0699:7586 84.07957:23066 85.02799:547409 86.03196:12596 86.09541:67413 97.06461:74051 103.03849:8438 115.07378:62989 125.0596:302680 126.06278:8929 132.10173:10240 132.18655:6242 143.06735:108340 144.10197:24769 161.07927:24418 227.09167:8346 | -1.80817E-06 |
| POS2500 | Allantoin | 0.763 | 159.04947 | [M+H]+ | 159.05 | 52.15548:6852 55.93345:47177 56.94233:12875 68.98124:35509 69.98251:6275 72.93707:13179 73.06432:23608 86.99105:20833 105.00241:13277 108.27512:5459 113.96312:6903 131.01933:8361 | -3.33229E-06 |
| POS3019 | densispicnin D | 5.978 | 175.13226 | [M+H]+ | 175.1328 | 54.46105:5937 59.02229:5374 59.04859:8480 69.72547:5990 133.93025:5405 146.03825:5677 157.05971:6021 165.42868:5228 173.43034:8800 | -3.08337E-06 |
| POS2883 | ramethyl-4-piperi( | 1.175 | 171.14856 | M+CH3OH+H] | 171.14909 | 55.05365:32473 58.06512:13894 67.05389:27210 69.06992:6107 72.04369:42975 83.08509:7684 89.06979:9185 109.10006:7372 118.74913:5687 | -3.09672E-06 |
| POS14943 | xy-3-oxochol-4-e | 6.74 | 777.52911 | [M+H]+ | 777.53003 | 62.33298:5719 77.24027:5977 173.43034:14701 | -1.18323E-06 |
| NEG6305 | ACMC-20m5sy | 0.912 | 313.25443 | [M-H2O-H]- | 313.25381 | 93.03925:95947 94.70243:6657 95.03764:22058 97.03746:11298 103.07947:53240 124.15677:15567 131.55391:5587 170.74947:6080 177.1145:22416 | 1.97923E-06 |
| POS4414 | eta-glucosylbutan | 4.6 | 221.1377 | [M+2H]2+ | 221.13831 | 55.05365:18955 58.04042:7238 61.01003:10168 73.02811:12944 81.0699:6347 89.05896:7078 97.09953:9690 125.09565:8844 173.39111:11760 175.25459:6089 193.96582:5796 | -2.75845E-06 |
| POS7123 | riphenyl Phosphat | 6.096 | 327.07718 | [M+H]+ | 327.07779 | 57.78203:5585 95.04893:13692 115.42985:5442 121.75073:5463 131.258:5125 153.06662:12314 | -1.865E-06 |
| POS14067 | PS(P-16:0/14:1(9Z) | 8.704 | 690.46942 | [M+2H]2+ | 690.46863 | 62.46531:6290 118.79361:5989 173.3911:12671 | 1.14415E-06 |
| POS6316 | Epiandrosterone | 6.166 | 291.21527 | [M+H]+ | 291.21469 | 57.03287:30544 57.06989:8249 59.04858:14080 85.48107:5604 89.87497:5691 | 1.99166E-06 |
| POS10231 | Artoindonesianin l | 4.39 | 505.2211 | [M+H]+ | 505.22171 | 60.04426:13514 70.06556:5731 72.08047:15701 72.84618:5567 84.08123:5355 94.80859:6167 118.08494:8652 129.10172:8733 129.49957:5232 175.10521:10225 329.03448:6374 | -1.20739E-06 |
| POS15338 | bicornutin A2 | 8.719 | 859.58398 | [M+2H]2+ | 859.5849 | 62.51623:5267 95.05093:4735 100.53985:5514 196.43747:5891 260.47702:5822 354.30902:5549 376.05884:5837 | -1.07028E-06 |
| POS5487 | ,6,7-hexahydro-2 | 5.97 | 261.13385 | [2M+H]2+ | 261.13327 | 57.03287:50469 57.54692:5185 61.26566:5423 109.75152:6913 | 2.22109E-06 |

**Differences in metabolites between the Model group and the WJW group**

| Alignment ID | Metabolite name | Rt(min) | Expreiment Mz | Adduct type | Reference m/z | MS/MS spectrum | PPM |
|---|---|---|---|---|---|---|---|
| POS14888 | HMS2873N16 | 4.419 | 770.33722 | [M+2H]2+ | 770.3382 | 72.08047:177076 86.09541:69139 118.08495:95567 129.09856:75262 305.85339:67544 | -1.27217E-06 |
| NEG4913 | OCTADEC-16-ENA | 0.878 | 265.25446 | [M-H]- | 265.25381 | 77.54872:6374 101.10021:5444 113.12118:131760 134.21043:6877 143.93689:5017 | 2.45048E-06 |
| NEG6724 | Hydroxyprogester | 0.904 | 329.25793 | [M-H]- | 329.25729 | 81.04115:6856 93.03925:49700 95.03764:24921 103.08172:13697 119.0826:16267 126.0732:6496 177.1145:11728 314.51971:6203 | 1.94377E-06 |
| POS939 | alpha-monochlor | 8.801 | 111.02007 | M+CH3OH+H]2 | 111.0207 | 54.03373:13309 55.04137:24637 55.93435:9953 56.96447:10873 57.9342:423490 67.49115:13985 69.99382:12383 71.05973:21944 72.93706:74402 74.93712:72777 76.49632:41026 77.99808:33349 78.9988:108420 79.49879:9418 84.94554:64956 85.50143:15059 87.00323:120458 88.00347:1912326 88.50381:137503 89.00122:26993 89.50647:13046 90.50591:56403 90.94576:21419 92.94704:11518 95.01113:30420 97.00507:278656 97.50974:30324 97.95379:29597 98.51207:25622 98.96088:91524 99.51075:403118 100.0115:33286 102.95542:46552 106.01485:14048 108.51748:32990 108.95824:12706 110.95901:9218 111.01927:40352 113.96311:112265 | -5.67462E-06 |
| POS9109 | yr-pro-leu-gly-NH | 4.624 | 448.25616 | [M+2H]2+ | 448.25549 | 55.05365:43824 59.04858:50330 69.03294:750078 70.0366:21563 71.04816:16042 73.02811:185668 73.06432:84127 78.03804:19506 80.05429:70324 81.03229:24991 83.0477:139718 87.04334:621042 88.04782:23611 89.05896:2186922 90.0619:75419 91.04643:18226 95.04893:47437 99.04339:351123 100.05018:82056 102.06606:48598 107.07058:15750 109.06336:20714 111.04441:180290 113.05927:45693 117.09089:32455 122.06293:132622 122.56553:19196 125.0596:31456 129.05446:73320 131.07088:64470 133.08543:657783 134.08684:34734 137.0598:30580 144.07597:43365 155.07083:73250 166.08865:26780 173.42543:19873 177.11285:46147 | 1.49468E-06 |
| POS12762 | -caffeoyloxyfriede | 6.835 | 605.42078 | [M+H]+ | 605.41998 | 59.86679:6420 | 1.3214E-06 |
| POS2033 | Ethephon | 8.766 | 144.98216 | [M+H]+ | 144.98151 | 62.92802:382364 103.95428:12783 121.96451:111242 144.97899:15551 | 4.48333E-06 |
| POS5240 | roughanic acid | 5.861 | 251.19934 | [M+H]+ | 251.2 | 55.05366:8995 57.06896:10614 59.04859:13384 67.05389:49762 81.06834:33459 93.06841:13426 95.08478:16153 96.71436:5123 107.08487:8283 119.01638:5881 200.50862:5817 | -2.62739E-06 |
| POS3651 | Cyclohexylalanine, | 0.996 | 194.11444 | [M+NH4]+ | 194.11513 | 52.16681:6394 58.21173:6940 65.0448:6380 105.28721:6138 135.04227:12472 | -3.55459E-06 |
| POS5913 | 13-OxoODE | 6.323 | 277.21069 | [M+H]+ | 277.20999 | 51.60017:5818 55.05365:14852 57.06895:8654 67.05389:11565 68.40289:5850 69.06991:7502 81.06989:8632 91.05389:7189 91.21084:5115 93.0684:13701 120.82984:5609 259.21762:5271 | 2.52516E-06 |
| POS8800 | Val Val Val Gly Gly | 4.948 | 430.26517 | M+CH3OH+H]2 | 430.2659 | 59.04858:42603 67.015:7871 82.13532:5643 87.04334:12522 87.1743:6597 89.05896:85351 103.07444:14768 122.2514:5931 133.08545:26483 221.2818:6249 | -1.69663E-06 |
| POS2234 | enzisothiazol-3(2H | 0.764 | 151.00845 | [M+Na]+ | 151.00919 | 56.74443:6504 67.05389:7281 68.98123:155552 82.01235:59734 86.99277:138376 100.02438:116854 105.0024:137716 110.00877:12313 118.03259:28004 123.0126:14057 128.01854:49666 | -4.90036E-06 |
| POS3883 | 3-benzodioxole-4 | 1.008 | 203.01422 | M+CH3OH+H] | 203.015 | 57.03287:28735 60.08025:167617 62.98059:44775 70.0643:127962 71.04816:18884 71.05974:54954 82.94377:27979 84.07957:14710 85.02799:894229 87.97689:213884 88.0851:27716 100.07382:27565 105.0024:76477 116.06905:23099 117.00114:14605 135.00183:10803 142.98286:19252 144.10197:18137 145.04651:16065 158.12614:11853 | -3.84208E-06 |
| POS4891 | 3,HCl-(??)-N5-Hyd | 4.856 | 239.13814 | [2M+H]+ | 239.1389 | 68.04949:10488 80.04967:18785 84.07957:100441 89.05895:10258 130.08707:9249 134.09683:383236 135.09956:16940 197.1311:14065 | -3.17807E-06 |
| POS8839 | Istamycin C1 | 4.491 | 432.27838 | [M+NH4]+ | 432.27921 | 73.06432:19208 87.04334:58890 89.05896:1171961 90.0619:26224 107.06821:28428 117.08817:8395 130.08389:15416 131.07088:13409 133.08543:480616 134.09018:13108 177.11285:54497 | -1.92006E-06 |
| POS9516 | pirost-9(11)-en-1 | 4.84 | 471.31198 | [M+H]+ | 471.31119 | 57.03287:12458 59.04859:331752 73.02812:9897 83.38518:5882 87.04335:30374 89.05896:269482 90.06375:6392 95.44421:5320 101.05931:24970 103.03849:9236 103.07444:66995 133.08545:42039 147.10149:21312 388.28345:5701 | 1.67617E-06 |
| POS11841 | Aristophyll B | 4.506 | 565.2818 | [M+H]+ | 565.28088 | 66.95612:21347 67.26903:21225 87.04334:58740 89.05896:578913 90.0619:37662 98.06215:17836 131.07088:17316 133.08543:227625 140.56842:15987 166.01514:16742 167.74202:14961 173.39111:26395 177.11285:21075 | 1.62751E-06 |
| POS6274 | )-3-(4-hydroxyph | 6.146 | 290.15469 | [M+H]2+ | 290.1539 | 55.57048:6563 57.03287:35246 110.31171:5599 175.7039:5876 | 2.72269E-06 |

| Differences in metabolites between the Model group and the WJW group | | | | | | | |
|---|---|---|---|---|---|---|---|
| Alignment ID | Metabolite name | Rt(min) | Expreiment Mz | Adduct type | Reference m/z | MS/MS spectrum | PPM |
| POS937 | alpha-monochlor | 6.48 | 111.01991 | [M+2H]2+ | 111.0207 | 55.0405:17427 55.93345:11104 57.9342:173904 67.49116:9696 72.93707:81535 74.93713:30982 75.49612:11274 76.4949:17358 77.99808:37259 78.99881:42211 84.94555:24951 85.49975:11244 87.00324:180801 87.50562:13106 88.00348:875251 88.50382:46455 89.00123:17917 89.50648:13792 90.50592:20519 90.94577:26361 96.00943:12156 97.00918:304637 97.50975:12290 98.51208:34880 98.96088:31845 99.51076:172240 100.01151:17327 102.95543:15439 106.01486:16401 108.51749:25855 108.95825:19281 111.01928:19849 113.96312:132436 | -7.11579E-06 |
| POS935 | alpha-monochlor | 5.758 | 111.0199 | [M+H]+ | 111.0207 | 55.04137:14360 55.93435:18317 57.9342:188736 68.04829:7364 72.93706:89086 74.93713:27482 75.49611:10279 76.49632:25918 77.99808:43985 78.99881:41352 84.94554:25301 85.49974:8835 87.00324:184402 87.50385:18900 88.00348:916425 88.50381:49343 89.00303:12289 89.50647:9252 90.50591:13615 90.94762:27374 92.94704:7548 95.01113:10101 96.00741:8333 97.00917:302326 97.50974:6819 97.95171:9592 98.51208:24318 98.96088:40993 99.51075:168294 100.0115:10693 102.95542:20264 106.01252:9103 108.51748:16989 108.95825:17323 111.01928:11515 113.96311:119832 | -7.20586E-06 |
| POS6837 | nethyllumazine he | 0.878 | 313.03406 | M+CH3OH+H] | 313.0332 | 51.59301:5858 57.06989:7570 64.0778:5520 86.3585:5997 95.34216:5905 145.91704:6573 177.06224:94869 | 2.74731E-06 |
| POS4740 | SCHEMBL4748090 | 6.31 | 233.17375 | [M+H]+ | 233.17461 | 50.02441:5649 51.9667:4592 55.5687:5839 57.06989:4761 58.42694:4201 63.52836:5329 65.09892:4186 69.69672:3886 70.41069:4354 71.44855:4437 73.02811:6086 73.10591:3917 73.56042:4965 75.60188:5517 79.3559:7422 82.78501:4928 83.87628:4915 89.71416:4409 92.87972:5020 104.80626:4991 111.92415:5047 123.71887:4982 125.3816:5402 130.29124:4211 136.30479:4052 140.81929:4339 144.29163:4038 147.57776:4511 151.09703:3968 165.68486:3732 179.49561:5680 195.94174:4385 196.09497:4181 210.6328:4450 216.47656:4519 218.19684:4031 230.56325:4532 | -3.68822E-06 |
| POS3864 | Pantothenic acid | 4.394 | 202.10612 | [M+H-H2O]+ | 202.10699 | 55.05365:27903 66.91496:6640 67.05389:5866 69.06868:5980 70.02779:6834 70.0643:12287 73.02811:6237 86.09541:9462 88.03895:19339 98.02254:6027 101.0593:7952 104.60148:5565 130.06477:6753 178.33917:5429 | -4.30465E-06 |
| POS8085 | DS-007819 | 4.879 | 379.23132 | [M+2H]2+ | 379.22781 | 59.04859:30062 67.015:11734 67.38055:5666 87.04335:11350 89.05896:48424 101.05931:13232 133.08545:14619 137.01158:5262 139.32047:5241 160.3878:5683 | 9.25565E-06 |
| POS14377 | anosyl)-2,6-dihydr | 6.592 | 715.53668 | [M+NH4]+ | 715.5354 | 57.03287:232785 57.96547:6274 58.03662:7976 59.04858:58816 99.07938:17533 115.07378:17169 117.09089:8136 157.12312:17195 363.88025:5820 431.01767:5684 715.4892:6396 | 1.78887E-06 |
| POS2880 | ramethyl-4-piperi | 0.682 | 171.14819 | [M+H]+ | 171.14909 | 55.05365:61066 57.03287:11399 67.05389:44024 69.06992:11642 70.06431:6126 71.04816:6819 71.92826:6813 72.04369:57363 72.08047:5949 79.05463:8663 81.0699:12644 83.08509:18180 89.06979:19943 93.06841:5858 109.06336:5644 109.10007:18048 123.59779:6135 166.5724:5543 | -5.25857E-06 |
| POS8122 | JWH-370 | 4.589 | 382.21741 | [M+H]2+ | 382.21649 | 55.05365:38762 58.04137:25107 59.04858:48721 61.02743:24303 67.05389:16343 69.03294:728952 70.0366:20642 71.04816:21413 73.02811:188386 73.06432:52727 78.03804:44417 80.05429:38128 81.03229:25991 81.0699:16770 83.0477:123278 85.02799:19799 87.04334:542064 88.04604:20426 89.05896:1675804 90.0619:53117 91.04643:9790 95.04893:47874 97.06461:10190 99.04339:349853 100.05018:91888 102.06606:26695 103.03849:10777 107.07058:23191 109.06336:18528 111.04189:154115 113.05927:40171 117.09089:15015 122.06293:113519 122.56553:9929 125.0596:38116 127.075:14979 129.05446:73354 131.07088:42454 133.08543:414738 134.09018:15588 137.0598:18181 144.07597:47260 155.07083:57110 166.08865:15737 173.07771:18284 177.11285:28229 | 2.40701E-06 |
| POS15052 | 18:1(9Z)/18:2(9Z,1 | 8.722 | 786.52936 | [M+2H]2+ | 786.52789 | 52.01259:6679 60.08025:11626 71.0726:6657 86.09541:24623 100.38199:6010 104.10696:10800 124.99955:5935 184.07466:79999 518.55597:6718 | 1.86897E-06 |
| POS34 | FLUOROACETONE | 4.383 | 77.03867 | [M+H-H2O]+ | 77.0396 | 53.1757:4496 53.45075:5253 53.69243:4379 54.90918:5249 55.54824:5066 55.82938:5177 60.23557:4648 65.02227:4910 66.54385:3871 67.13178:3795 68.11464:4557 69.44248:4199 69.63427:4563 69.75673:4803 70.04163:4254 71.10993:3927 71.54332:4523 71.62527:4201 72.18309:3845 75.41446:4233 78.97468:5244 79.0169:4167 | -1.20717E-05 |

**Differences in metabolites between the Model group and the WJW group**

| Alignment ID | Metabolite name | Rt(min) | Expreiment Mz | Adduct type | Reference m/z | MS/MS spectrum | PPM |
|---|---|---|---|---|---|---|---|
| POS8848 | Glu Arg Glu | 4.46 | 433.20316 | M+CH3OH+H] | 433.2041 | 62.05909:7454 85.02799:8503 87.04334:27083 89.05896:501474 90.0619:43329 110.23711:5445 127.03809:6320 131.06766:6017 133.08543:196828 134.08684:25018 173.43524:12361 177.11285:23861 | -2.16988E-06 |
| POS5643 | no]-1,3,9-trimethy | 3.201 | 267.13202 | [M+H]+ | 267.133 | 67.05389:7067 78.88884:6173 84.07957:24893 94.57704:6600 95.29619:6594 96.04378:8462 136.06245:11875 | -3.66858E-06 |
| POS6938 | -3-Hydroxypregn- | 6.683 | 317.24579 | M+CH3OH+H] | 317.2468 | 67.05389:12161 69.06992:5708 105.06946:10786 119.08613:8711 131.08377:6719 164.12044:5913 173.39111:8186 | -3.18364E-06 |
| POS9718 | Xestobergsterol B | 6.512 | 481.31287 | [M+H]+ | 481.3139 | 135.48819:5754 342.19617:6394 | -2.13998E-06 |
| POS8856 | Salicyloyl-conkurc | 7.323 | 433.28387 | M+CH3OH+H] | 433.28491 | 53.10416:5657 59.07783:5143 62.33404:5331 94.48625:6398 152.66074:7117 | -2.40027E-06 |
| POS55 | Fluoroacetic acid | 8.803 | 79.01788 | [M+NH4]2+ | 79.0189 | 52.70664:5986 56.62345:5510 63.99746:10521 79.01992:7497 | -1.29083E-05 |
| POS5602 | 6R)-2-hexoxy-6-( | 4.611 | 265.16293 | [M+2H]2+ | 265.164 | 53.46587:6831 67.6092:6033 72.95446:5688 73.02811:6498 173.43034:11133 | -4.03524E-06 |
| POS7128 | ecylbenzenesulfon | 4.648 | 327.19986 | [M+H]+ | 327.19879 | 58.09455:6387 60.16131:5746 73.35352:6188 89.05891:10389 106.76425:5645 | 3.27018E-06 |
| POS9231 | Tenovin-6 | 4.488 | 455.24631 | [M+H]+ | 455.24741 | 55.05365:22859 59.04858:15265 69.03294:202679 73.02811:51304 73.06432:14771 81.03229:23595 83.0477:123453 85.02799:13985 87.04334:346734 89.05896:690125 90.0619:9517 95.04893:21427 99.04339:216063 101.0593:10555 103.03849:11945 107.07059:18161 111.04189:187639 113.05927:21910 125.0596:55597 129.05446:26236 131.06766:37816 133.08543:222312 137.05637:19134 151.09305:16517 155.07083:70916 173.0826:12847 195.11966:10895 | -2.41627E-06 |
| POS6951 | uberoyl-L-carnitin | 4.365 | 318.18979 | [M+H]+ | 318.19089 | 55.05365:11102 57.03379:10806 60.08025:67546 69.06992:58223 70.0643:14150 71.04816:38446 72.08047:9082 83.08509:22174 84.07957:28562 85.02799:129011 86.09541:91104 91.0539:9058 93.06841:10767 95.08477:6639 97.06461:25073 102.05499:9713 110.07076:8873 111.0796:20090 115.07378:49827 116.06905:8732 136.07611:13535 139.07358:22015 144.10197:9751 157.08505:22809 175.09526:15938 | -3.45704E-06 |
| POS5181 | Negamycin | 1.131 | 249.15454 | [M+H]+ | 249.15569 | 58.06512:12974 59.07198:13599 79.71541:6195 85.02799:11904 104.24855:6213 118.08494:229739 132.07565:5343 144.95276:6103 | -4.61559E-06 |
| POS8384 | Uoamine B | 4.787 | 400.28906 | [M+NH4]+ | 400.2879 | 57.03286:47673 59.04858:1348252 60.05225:17710 69.06867:7295 73.02811:10860 73.06431:28986 85.06335:14196 87.04333:55531 87.07997:12585 89.05894:612277 90.0619:10313 100.07596:9477 101.05929:76905 103.07442:379305 107.06819:16122 117.05279:116936 117.09087:34915 130.08707:9278 131.06764:18574 133.08542:88278 145.08401:9514 147.10147:149700 161.11438:29725 191.12556:10875 | 2.89791E-06 |
| POS11285 | Arg Ser Lys Arg | 5.349 | 546.3457 | [M+H]+ | 546.34698 | 57.03287:20336 59.04859:239650 67.015:23160 73.02812:21084 81.03072:13272 86.09541:10981 87.04335:27798 87.07999:9468 89.05896:203904 90.06191:10843 101.05931:24012 103.07444:61977 104.10697:83116 117.0909:9549 130.85844:8922 133.08545:77872 147.07468:10073 147.10149:27853 151.08508:14899 158.09198:14052 184.07466:18004 251.92677:8982 466.18372:8502 | -2.34283E-06 |
| POS7558 | Dodecyl glucoside | 6.14 | 349.2569 | [M+H]+ | 349.25809 | 57.03287:18914 59.04858:7832 90.3176:5956 93.2132:5617 165.29622:6592 347.04623:6512 | -3.40722E-06 |
| POS5607 | 4-ethylphenyl)-1- | 6.078 | 265.19382 | [M+H-H2O]+ | 265.19501 | 73.36568:5988 173.38618:9266 263.33527:5885 | -4.48726E-06 |
| POS6902 | kaden-TP NOA 40 | 4.548 | 316.17993 | [M+2H]2+ | 316.17871 | 55.05365:18529 59.04858:24176 67.05389:9614 69.03294:248663 70.03661:6902 71.04816:7926 73.02811:59434 73.06432:11612 78.03804:38009 81.03229:11849 81.0699:28225 83.0477:58645 85.02799:10159 87.04334:192220 88.04782:6438 89.05896:448073 90.0619:13322 94.71547:6485 95.04893:10730 99.04339:104753 99.07938:42340 100.05018:33996 107.07059:6587 109.06336:9850 111.04189:70949 113.05927:7981 117.09089:5997 122.06293:40646 125.0596:14454 129.05446:21948 131.07088:13397 133.08543:80573 137.05981:9095 142.13843:6809 144.07597:11241 155.06668:21540 173.0826:6402 238.23842:6823 | 3.85858E-06 |
| POS5160 | -hydroxybutyrylcar | 2.256 | 248.1478 | [M+H]+ | 248.149 | 59.04858:11134 60.08024:14690 72.08047:40565 74.22613:5953 84.04315:15212 85.02799:85597 103.03848:12730 | -4.8358E-06 |
| POS15091 | S-3466-C | 6.728 | 793.50763 | [M+H]+ | 793.50958 | 87.02763:6464 118.83253:5497 627.29291:5952 661.3858:5900 | -2.45744E-06 |
| POS7422 | Daphnilactone B | 6.078 | 342.24094 | [M+NH4]+ | 342.24219 | 57.06895:7702 57.4271:5912 58.06511:6050 59.05637:5598 67.05389:12496 75.02496:7322 81.06832:7854 84.07957:9365 91.05389:6026 105.06944:6899 107.2755:5846 119.08332:7047 122.09478:8318 131.08376:7998 134.09349:6883 147.11679:6070 171.90515:5738 179.28917:5348 217.66479:5314 | -3.65238E-06 |

| Differences in metabolites between the Model group and the WJW group | | | | | | | |
|---|---|---|---|---|---|---|---|
| Alignment ID | Metabolite name | Rt(min) | Expreiment Mz | Adduct type | Reference m/z | MS/MS spectrum | PPM |
| POS11944 | Antibiotic WF 3161 | 4.682 | 569.33484 | [M+H]+ | 569.33337 | 69.03294:49433 73.02811:178498 73.06432:57693 80.05429:70046 87.04334:376107 89.05896:1927869 90.0619:74864 91.04643:51479 95.04893:55157 99.04339:59499 111.04441:55463 113.05927:52859 124.08022:31021 131.06766:53555 133.08543:710807 134.09018:65579 155.07083:38486 177.11285:84402 | 2.58197E-06 |
| POS7610 | andamide (20:2, n | 6.825 | 352.3187 | [M+NH4]+ | 352.32001 | 86.98582:7061 267.80582:6085 | -3.71821E-06 |
| POS7792 | anylaspartylarginir | 4.765 | 361.18155 | [M+Na]+ | 361.18289 | 58.04042:23351 59.04859:104682 65.04819:12460 73.02812:91447 80.0543:31045 80.41393:6032 87.04335:34073 87.06254:8323 89.05896:240925 90.06191:5522 101.05931:7642 102.06606:16955 124.07726:8817 131.06766:8963 131.90631:5727 133.08545:60119 173.43036:8546 300.88321:5418 | -3.71003E-06 |
| POS3540 | 2H-3,4,5,6-tetrahy | 0.898 | 191.07605 | [M+Na]+ | 191.07739 | 60.08025:22959 61.08378:9675 62.98166:18938 68.05673:6062 72.67956:6599 72.68887:6035 84.07957:166945 85.08359:71411 90.58549:5456 130.08389:15437 131.09021:12874 173.02882:6747 191.0405:10460 191.07452:13864 | -7.01287E-06 |
| POS6082 | 17_epimethanedie | 6.075 | 283.20364 | [M+H-H2O]+ | 283.20499 | 53.84824:5745 55.05365:19561 57.06895:16933 67.05389:6858 69.06867:20806 71.08546:11932 79.05312:6546 81.06989:8545 95.08476:9856 105.06944:6845 106.34853:5736 107.08485:6755 171.11885:7386 181.09694:6200 183.11298:8004 188.50836:6093 203.82985:5841 264.63409:5675 | -4.76687E-06 |
| POS6239 | Asn Val Gly | 8.817 | 289.1492 | [M+NH4]+ | 289.1506 | 56.96448:7101 57.06989:10926 86.0954:11678 95.08476:7188 104.10696:10945 124.99955:7374 138.52921:7631 185.85204:6748 | -4.84177E-06 |
| NEG4258 | -Undecanoylglycir | 9.352 | 242.17418 | [M-H]- | 242.17561 | 64.039:6632 90.04863:14065 | -5.90481E-06 |
| POS15316 | Antibiotic TM 531 | 6.872 | 851.54895 | [M+H]+ | 851.55139 | 53.28831:7958 54.97478:6765 105.15044:6230 254.47932:5572 438.78909:7185 631.97534:7426 | -2.86536E-06 |
| POS7757 | Ile-Arg-Ala | 6.022 | 359.23859 | [M+H]+ | 359.24011 | 52.452:5979 54.32751:8237 69.1946:5060 88.05315:6284 106.14863:6019 180.57471:6387 341.74814:5899 | -4.23115E-06 |
| NEG4801 | SCHEMBL1426937( | 0.906 | 261.18762 | [M-H]- | 261.1861 | 79.05368:19559 93.03925:9310 103.07947:17718 107.06415:5280 118.93213:5943 119.0854:144122 177.1145:7846 190.98895:5499 | 5.81961E-06 |
| POS8760 | ircinic acid A | 6.353 | 427.29388 | [M+2H]2+ | 427.29541 | 57.03287:29050 59.04858:206712 81.03072:22710 115.8304:5330 117.09089:26084 173.43034:8558 | -3.58066E-06 |
| POS9532 | Malyngamide O | 4.654 | 472.284 | [M+2H]2+ | 472.28241 | 58.04042:41917 69.03294:21966 71.04945:8981 73.02811:171275 73.06432:28295 80.05429:121145 87.04334:97696 89.05896:589103 90.0619:27539 95.04694:10836 102.06606:87038 107.07058:9726 111.06954:10968 117.08817:12382 124.08022:35440 133.08543:219018 134.08684:9092 146.09132:15891 155.09987:16375 173.43524:19122 177.11285:29597 | 3.36663E-06 |
| POS9235 | Blazeispirol Z | 4.956 | 455.2811 | [M+H]+ | 455.27951 | 59.04858:74319 73.02812:7384 73.06566:6187 73.29153:6055 87.04334:236414 89.05896:49675 98.93763:4991 101.0593:62978 103.03849:17002 103.07444:13917 115.07378:5845 117.05281:10844 133.08545:14029 161.34744:5438 172.78468:5846 231.72554:5815 345.12427:5506 | 3.49236E-06 |
| POS4324 | mycin monophos | 0.779 | 218.98331 | M+CH3OH+H] | 218.9817 | 51.71658:6445 61.03971:6312 68.98123:7856 73.06431:9481 86.99277:9904 98.97779:5810 101.02003:6761 107.0016:6625 119.0303:12751 121.0269:7406 131.01932:19292 145.04274:6936 149.02837:6261 159.04826:6278 162.99287:6641 190.98946:7110 194.38419:6124 | 7.35221E-06 |
| POS11350 | n E2 p-benzamidc | 6.252 | 548.29883 | [M+2H]2+ | 548.3006 | 51.76935:6327 69.95988:6295 104.10697:31123 184.06931:8699 | -3.22816E-06 |
| POS14351 | -acetyltrichagmali | 7.121 | 713.31439 | [M+H]+ | 713.31671 | 110.02861:5819 173.39111:16903 193.88463:5749 | -3.25241E-06 |
| POS10090 | Halisulfate 10 | 4.503 | 499.27396 | [M+Na]+ | 499.27231 | 55.05365:28736 59.04858:18016 69.03294:236843 73.02811:68322 73.06432:23736 81.03229:38010 83.0477:126886 85.02799:18303 87.04334:463670 89.05896:1095471 90.0619:19337 95.04893:42461 99.04339:338329 101.0593:13923 107.06821:35729 111.04189:214311 113.05927:17531 125.0596:71784 129.05446:33904 131.07088:42278 133.08543:348351 137.05981:27478 151.09305:16868 155.07083:97815 173.07771:20199 173.39111:12653 175.09526:13257 177.11285:24897 383.6424:11174 | 3.30481E-06 |
| POS9771 | aroten-4'-al; beta | 5.875 | 483.36038 | [M+H]+ | 483.36209 | 57.03284:903255 58.03659:71234 59.04856:265367 60.05223:21377 60.08022:42360 69.06989:10621 81.0683:24088 86.09537:44687 99.07935:82589 100.08238:14040 101.09636:14544 104.10692:476680 105.10874:72281 115.07373:53297 117.09084:31735 124.9995:17849 157.12306:60753 158.12607:17816 173.42537:15916 184.07457:19753 | -3.53772E-06 |
| POS7000 | tetrahydroisoquin | 8.805 | 320.1662 | [M+H]2+ | 320.16449 | 57.06989:10949 71.08547:6578 76.01012:5352 86.0954:5684 115.63253:6699 | 5.341E-06 |
| NEG4423 | dopargine | 1.353 | 248.13878 | [M-H]- | 248.1405 | 51.94239:6965 89.13124:21490 97.07645:7956 103.6411:5306 180.61613:5629 | -6.93156E-06 |

| Differences in metabolites between the Model group and the WJW group | | | | | | | |
|---|---|---|---|---|---|---|---|
| Alignment ID | Metabolite name | Rt(min) | Expreiment Mz | Adduct type | Reference m/z | MS/MS spectrum | PPM |
| POS8439 | yptophylalanyllysi | 4.601 | 404.23093 | [M+H]2+ | 404.22919 | 55.05365:69420 59.04858:82537 61.02743:37435 67.05389:27394 69.03294:1097856 70.0366:35509 71.04816:27370 73.02811:261407 73.06432:105466 78.03804:48174 80.05429:78280 81.03229:38017 81.0699:20906 83.0477:194623 85.02799:23581 87.04334:900676 88.04604:23622 89.05896:2799754 90.0619:89847 95.04893:56691 99.04339:524463 100.05018:130192 102.06606:47082 107.07058:27984 109.06336:26111 111.04189:254387 112.04632:16700 113.05927:72295 117.09089:31407 122.06293:201671 125.0596:59454 129.05446:97910 131.06766:64887 133.08543:726914 134.08684:40704 137.0598:32659 144.07597:79409 144.57867:17736 155.07083:88208 166.08865:22650 173.07771:23871 175.09526:27320 177.10779:45895 | 4.30449E-06 |
| POS8447 | Glu Lys Glu | 4.377 | 405.19617 | [2M+H]+ | 405.19791 | 53.77364:5498 60.04425:44760 70.06556:11702 86.0954:127568 87.0538:42449 88.03894:25101 90.05456:27388 115.48846:6684 124.03867:12775 128.10568:15187 170.0423:7647 173.12662:170473 205.07927:25285 | -4.2942E-06 |
| POS5659 | etramethyl-1,2-et | 6.296 | 267.20895 | [M+H-H2O]+ | 267.21069 | 55.05365:7454 67.05389:12529 122.52764:5775 141.06725:13587 143.08205:5119 155.08327:6205 159.31569:5686 177.96635:6350 183.1183:7326 | -6.51172E-06 |
| POS1262 | ydroxyphenyletha | 1.133 | 121.07175 | [2M+H]+ | 121.07 | 56.04862:105910 57.03379:9938 60.90679:11689 61.0387:12084809 61.17308:10012 62.04229:11768 72.08047:9141 74.05886:38460 | 1.44544E-05 |
| POS11179 | hexoxy)pentan-2- | 6.253 | 542.31909 | [M+Na]+ | 542.31714 | 51.5143:6374 54.3645:6571 57.03286:9626 59.04858:18997 60.08024:28518 67.05389:7207 69.0699:7761 71.07259:10684 81.06989:16238 86.09539:168554 95.08475:10387 104.10695:982143 105.10877:23540 146.97893:103052 150.06129:7659 167.15565:6181 173.43031:6585 393.3775:6571 | 3.59568E-06 |
| NEG4443 | Cadabicilone | 1.386 | 249.14725 | [M-H]- | 249.14906 | 50.33427:6968 51.7285:5831 56.11635:5995 89.12944:25875 126.02459:17056 126.341:5377 134.5883:4972 169.09492:6739 | -7.26473E-06 |
| POS8459 | Dihydroroseoside | 4.626 | 406.24582 | [M+2H]2+ | 406.24399 | 58.04042:316049 69.03294:26840 71.04816:23644 73.02811:815241 73.06432:76618 74.03149:19535 80.05429:592291 80.55504:35823 87.04334:254484 89.05896:2323191 90.0619:75913 102.06606:456392 102.56839:31231 111.07206:34913 117.09089:24991 124.08022:170757 124.58041:21333 131.07088:32343 133.08543:732108 134.09018:31369 146.09132:51431 155.09572:46962 177.11285:66796 | 4.50468E-06 |
| POS11349 | (-)-Detoxin D1 | 6.308 | 548.29456 | [M+H]+ | 548.29657 | 59.04859:7284 76.51501:6913 89.05896:7114 104.10697:43253 183.96208:5542 184.06931:9899 198.2594:6424 338.27423:5626 361.66922:5296 | -3.6659E-06 |
| NEG3583 | butylphosphine ox | 1.322 | 217.17096 | [M-H]- | 217.17281 | 65.3876:5879 79.05367:11310 97.07645:2343227 98.0778:15686 167.79976:6017 | -8.51856E-06 |
| POS2377 | -desisopropyl-2- | 0.91 | 155.07883 | [M+H]+ | 155.0807 | 50.22104:6700 56.08018:6744 60.34113:6326 76.60997:7373 83.05907:17279 93.04334:12960 110.07076:74578 123.78096:5352 137.58165:5961 155.68228:5672 | -1.20582E-05 |
| POS11816 | anoyl]-hydroxyam | 5.455 | 564.37366 | [M+2H]2+ | 564.37152 | 57.03379:24448 59.04956:417829 60.05325:13891 73.02812:45138 73.06566:15355 85.06506:15845 87.04334:70351 87.06253:15152 87.07999:14180 89.05896:356656 90.06374:14936 99.72215:8955 101.0593:66806 103.07444:125605 109.07559:10320 123.51226:8200 129.09225:11722 130.08708:11693 133.08545:136882 147.10149:68488 177.11285:14320 191.13127:15101 551.74365:8931 | 3.79183E-06 |
| POS979 | methylphosphon | 6.48 | 112.01766 | [M+2H]2+ | 112.01578 | 54.03373:103496 55.04137:15011 57.9342:140994 59.92951:82930 67.05389:22556 69.04403:12416 70.06431:13730 71.05974:323263 74.93713:25651 76.49633:17608 76.93212:12667 78.99881:19813 79.99587:19222 84.07957:13037 84.94555:17418 85.49975:9229 86.94054:7455 88.00348:610381 88.50382:41697 89.00123:340111 89.50102:31088 90.50592:8788 94.06358:11669 95.01114:12653 97.00918:209536 97.50975:10133 98.00587:132842 98.50788:10097 98.96088:29253 99.51076:99752 100.50954:66568 100.95683:13413 101.01132:12715 102.95543:16278 108.51749:16719 109.51485:11260 111.01928:11083 112.07434:12583 | 1.67833E-05 |
| POS295 | Putrescine | 6.474 | 89.00195 | [M+H]+ | 89 | 50.37579:6720 55.05365:10358 55.93435:10232 56.04952:8560 61.02743:22531 61.0346:6141 70.0643:16085 72.93706:86397 74.99706:9477 77.99807:10994 87.00323:63718 88.07444:42139 90.94762:25573 | 2.19101E-05 |
| POS7809 | Bacithrocin C 2 | 4.601 | 362.22061 | [M+NH4]2+ | 362.2186 | 58.04042:568294 69.03294:18110 71.04816:38377 73.02811:1280998 73.06432:84323 74.03149:32832 80.05429:736250 80.55659:42528 87.04334:300222 88.04782:18518 89.05896:3087766 90.0619:93126 102.06606:482204 102.56839:44585 111.07206:35091 115.07378:16474 117.09089:20523 124.08022:192208 124.58041:18554 131.07088:23712 133.08543:780054 134.08684:40726 146.09132:36484 155.09987:25200 177.11285:65140 | 5.54914E-06 |

| Differences in metabolites between the Model group and the WJW group | | | | | | | |
|---|---|---|---|---|---|---|---|
| Alignment ID | Metabolite name | Rt(min) | Expreiment Mz | Adduct type | Reference m/z | MS/MS spectrum | PPM |
| POS9714 | Pharboside B | 4.645 | 481.28281 | [M+NH4]+ | 481.28079 | 58.04041:23872 59.04858:35887 69.03294:54111 73.02811:189832 73.06432:62878 80.05429:73051 81.03229:41889 83.04769:28918 87.04334:390027 88.04782:21281 89.05895:2047538 90.0619:89012 91.04643:68448 91.07442:10828 95.04893:82738 99.04339:69170 101.0593:11472 102.06605:41337 107.0682:24784 109.06335:12856 111.0444:82514 113.05927:81471 117.09089:25129 124.08022:17131 125.05959:13639 131.07086:51757 133.08543:782893 134.09016:43064 137.0598:43303 151.09703:24787 155.06668:39977 157.08505:17409 173.39601:11618 177.11284:87990 | 4.19713E-06 |
| POS9506 | Ser His Val Lys | 4.633 | 470.27005 | [M+2H]2+ | 470.27209 | 59.04858:28327 61.02743:19094 69.03294:486273 70.0366:11048 71.04816:10393 73.02811:108277 73.06432:64282 78.03804:13932 80.05429:47826 81.03229:17258 83.0477:87647 87.04334:417368 88.04782:14229 89.05896:1414898 90.0619:50529 95.04893:30424 99.04339:216102 100.05018:36082 102.06606:30560 107.07058:14999 109.06336:9811 111.04189:108709 113.05927:36454 117.09089:16338 122.06293:73095 125.0596:25053 129.05446:37538 131.06766:34850 133.08543:424666 134.09018:26651 137.0598:14985 144.07597:36262 155.07083:45272 166.09326:19870 173.07771:12719 177.11285:37390 | -4.33791E-06 |
| POS11759 | Rhodoxanthin | 4.751 | 562.37769 | [M+NH4]+ | 562.38 | 59.04858:142885 73.02811:8122 73.06431:10740 87.04333:66884 89.05894:514488 90.0619:9773 101.05929:24945 103.07442:58253 129.0891:7427 131.07086:9705 133.08542:221969 147.10147:33828 173.38129:5567 177.11282:26216 | -4.10754E-06 |
| POS8156 | CHEMBL564302 | 4.614 | 384.23416 | [M+H]2+ | 384.23209 | 58.04136:306083 69.03294:18685 71.04816:28947 73.02811:754821 73.06432:61647 74.03149:13895 80.05429:564688 80.55503:36945 87.04334:225034 89.05895:2058075 90.0619:68475 102.06605:414678 102.56838:31943 111.07205:32339 117.09089:20394 124.08022:162777 131.07086:21136 133.08543:718377 134.09016:25768 146.09132:41706 155.09987:30189 173.40091:13649 177.11284:75759 | 5.38737E-06 |
| POS2107 | THTA | 0.766 | 147.04953 | [M+H]+ | 147.04739 | 55.05366:16760 56.94141:14929 58.06512:6982 68.98124:164801 69.98251:7418 72.08048:55584 72.93573:10611 74.95385:5586 84.07958:12888 86.9928:155495 89.81097:6217 100.0244:13701 101.02005:7847 105.00242:161160 106.00315:6326 118.03261:6972 119.03033:20612 123.01263:12071 128.01855:9989 132.57256:5371 137.03914:13565 | 1.45531E-05 |
| POS12728 | Janthitrem E | 4.714 | 604.3606 | [M+2H]2+ | 604.36322 | 73.02811:11897 84.39672:9222 87.04334:26743 89.05896:141711 90.0619:9134 107.30415:9620 133.08543:52468 173.43034:19274 | -4.33514E-06 |
| POS8593 | tadecanedioylcarn | 6.351 | 416.30289 | [M+2H]2+ | 416.3006 | 57.03287:79220 59.04858:714507 60.05225:16413 87.04334:48032 99.07938:16584 101.09422:6412 115.07378:14586 117.09089:53473 175.1301:8154 | 5.50083E-06 |
| POS10117 | Osimertinib | 6.157 | 500.27371 | [M+Na]+ | 500.276 | 59.04858:7297 60.0093:5919 89.05895:6451 120.96402:6568 | -4.57747E-06 |
| POS12122 | 2-yl]-18-norpregn | 4.835 | 576.39209 | [M+NH4]+ | 576.3894 | 59.04859:1065958 73.02678:55432 73.06432:42095 85.06337:36108 87.04335:323799 87.07999:47369 89.05896:2615279 90.06375:44507 101.05931:177454 101.09423:22210 103.07444:531423 105.08796:24071 117.05281:53286 117.0909:39218 129.08911:65461 131.06766:92997 131.10634:31421 133.08545:1296119 145.08403:55253 147.10149:480032 161.1188:34809 177.11285:190329 189.11574:24983 191.1256:129740 205.14238:31715 341.20605:24596 | 4.66698E-06 |
| POS8077 | 4-bromophenyl)ch | 0.882 | 378.89838 | [M+Na]+ | 378.896 | 90.97556:16093 106.94932:100680 174.93611:8732 | 6.28141E-06 |
| POS15273 | pteriatoxin B | 4.672 | 831.47833 | [M+2H]2+ | 831.4823 | 84.64203:28986 87.04334:84677 89.05896:302733 133.08543:119601 151.0771:30111 162.6404:26828 173.43524:53055 217.99625:28931 236.25288:28998 316.36374:26634 359.20026:28753 760.00513:29745 | -4.77461E-06 |
| POS1292 | -Mercaptolactic ac | 0.763 | 123.01339 | [M+Na]+ | 123.011 | 68.98124:26614 73.66351:6563 80.04815:9105 86.99279:29251 105.00241:38609 111.29868:5666 | 1.94292E-05 |
| POS1695 | Iodotyrosine | 8.763 | 135.00243 | [M+H]+ | 135 | 91.13419:5808 111.37939:6078 | 1.8E-05 |
| POS10433 | ydrocochlioquinor | 4.649 | 514.29553 | [M+2H]2+ | 514.29303 | 69.03294:203259 73.02811:75934 73.06432:45759 80.05429:31685 83.0477:45819 87.04334:208479 89.05896:751023 90.0619:31274 99.04339:103542 100.05018:21287 102.06606:17963 111.04189:58334 113.05927:26421 122.06293:37257 129.05446:20840 131.07088:19682 133.08543:257239 144.07597:23916 155.07083:25151 163.73279:16963 175.10023:16845 177.10779:34261 | 4.86104E-06 |
| POS15065 | indol-3-ylmethyl) | 4.659 | 787.45392 | [2M+H]2+ | 787.45007 | 87.04334:482067 89.05896:1831671 97.71687:87547 113.05927:156148 131.07088:113006 133.08543:857033 177.11285:118860 | 4.8892E-06 |

**Differences in metabolites between the Model group and the WJW group**

| Alignment ID | Metabolite name | Rt(min) | Expreiment Mz | Adduct type | Reference m/z | MS/MS spectrum | PPM |
|---|---|---|---|---|---|---|---|
| POS12764 | h_78_metenolone | 6.296 | 605.453 | [2M+H]+ | 605.45599 | 55.05365:18530 57.06895:14888 67.05389:44064 69.06867:27070 78.97769:6644 79.05312:16915 81.06989:42359 83.08508:13350 91.05389:13794 93.0684:29912 95.08476:34396 105.06944:15997 107.08485:21475 109.10005:12111 117.06911:14630 119.08332:20444 121.10129:15391 131.08376:16397 145.09903:7917 147.11296:8111 149.0948:7143 | -4.93843E-06 |
| POS9252 | Arachidyl carnitine | 5.93 | 456.4028 | [M+NH4]+ | 456.40527 | 57.06989:6800 60.08025:30331 85.02799:162764 372.31253:6191 | -5.41186E-06 |
| POS1464 | 5-dihydrooxazole | 0.764 | 128.01889 | M+CH3OH+H] | 128.0164 | 53.03773:43432 54.03372:13280 55.0177:42032 55.05365:56029 57.03286:7783 57.04396:19937 58.99794:11420 67.04092:11162 68.98123:315590 69.06991:9599 69.9913:6332 71.04815:11644 80.04813:7127 81.06989:7155 82.01235:106684 82.06342:70860 86.05938:6850 86.99104:288728 100.02438:233782 101.02003:12023 105.0024:308647 109.02666:6951 110.00877:16486 118.03259:66900 119.02752:30303 123.0126:31600 127.03808:34422 128.01854:61966 128.06833:12319 | 1.94506E-05 |
| POS11691 | Homoamericin | 4.506 | 560.32593 | [M+H]+ | 560.32312 | 69.03294:55295 70.76349:11144 73.02811:25559 83.04932:15615 87.04334:117755 89.05896:361677 99.04339:115428 111.04189:54655 125.0596:21098 131.06766:19237 133.08543:129752 155.07083:33561 173.43034:15515 | 5.01496E-06 |
| POS13081 | )en-2,28-diol-3-y | 5.736 | 621.4118 | [M+2H]2+ | 621.41492 | 57.03287:56259 59.04859:169509 69.06992:13135 73.02812:43201 73.06432:20588 76.31847:9349 81.0699:10492 85.06337:40862 87.04335:72631 87.07999:14273 89.05896:238665 89.37024:10329 93.17069:9033 99.07939:11249 101.05931:59170 103.07444:35312 115.07378:15233 117.0909:11264 129.08911:29525 133.08545:84440 143.10413:12803 147.10149:18299 155.00865:10393 | -5.0208E-06 |
| POS13718 | NAD | 8.769 | 663.11243 | [M+H]+ | 663.10907 | 126.32156:5312 166.99335:5442 173.43034:13072 256.35541:5511 | 5.06704E-06 |
| POS8736 | alpha-rhamnopyra | 4.599 | 426.24603 | [M+2H]2+ | 426.2486 | 55.05363:41015 59.04856:44172 61.02843:28454 69.03291:717016 70.03657:16774 71.04813:16943 73.02808:176514 73.06429:74263 78.038:22960 80.05426:44187 81.03225:26480 83.04766:97931 87.0433:533648 88.04601:24610 89.05891:1840733 90.06187:73354 95.04889:44866 99.04334:301216 100.05013:64558 102.06601:27615 107.06815:17809 111.04185:146249 113.05922:39878 117.09084:31556 122.06287:110193 125.05954:30750 129.05441:61730 131.07082:39951 133.08537:474505 134.08678:16236 137.05974:15891 144.07591:34950 155.07077:56603 173.08252:20539 175.09518:15045 177.11278:38658 | -6.02935E-06 |
| POS7359 | U 6796 | 4.768 | 339.23547 | [M+H]+ | 339.23291 | 57.03287:107158 59.04859:2537525 60.05226:41069 69.06992:7865 73.02812:29093 73.06432:57512 85.06337:17847 87.04335:129956 87.07999:13310 89.05896:412178 90.06375:8792 99.07939:7426 101.05931:68198 103.03849:39782 103.07444:344578 104.07732:8444 115.07378:11308 117.05281:145545 117.0909:37751 131.07088:31810 133.08545:17737 147.10149:47546 161.11441:15716 | 7.54644E-06 |
| POS2497 | enzenesulfonic aci | 0.759 | 159.01361 | [M+H-H2O]+ | 159.011 | 55.0177:21242 55.05365:12996 55.93434:80591 56.04952:8941 56.94232:9886 67.05389:20318 68.98123:145643 69.06991:10543 69.9825:8849 70.06429:15395 70.97875:7572 71.04815:9243 72.04369:6827 72.93706:22034 73.06431:35320 77.00898:6160 81.06989:6313 85.01283:17215 86.99277:124379 87.99284:8895 90.94576:6641 95.08475:21154 103.02276:78286 105.0024:157077 106.00548:8678 106.99923:15552 113.00764:17669 113.9631:12778 114.97044:11630 117.03919:20402 121.03262:38130 123.0126:23543 131.01932:140857 132.02023:6080 135.049:14033 147.04784:12385 149.02837:49767 149.06354:9678 | 1.6414E-05 |
| POS10348 | Narbomycin | 8.759 | 510.34515 | [M+2H]2+ | 510.3425 | 70.88638:5356 87.14983:6076 108.37923:4924 313.50699:5615 353.19443:5993 | 5.19259E-06 |
| POS13541 | Quinovin | 4.775 | 650.42969 | [M+NH4]+ | 650.42621 | 59.04858:50737 87.04333:31364 89.05894:259203 103.07442:10850 133.08542:111104 147.10147:18945 147.1283:10857 173.38618:22166 191.7171:12443 192.78191:12033 473.44424:10940 | 5.35034E-06 |
| POS6820 | Adiphenine | 4.652 | 312.19916 | [M+NH4]+ | 312.19647 | 59.04859:833715 60.05226:67039 73.06432:8571 85.06506:15342 87.04335:65677 88.04605:6379 89.05896:1032050 90.06191:127384 91.06323:7664 101.05931:25769 103.07445:99740 104.07732:13123 107.07059:10487 117.05281:9556 133.08545:147176 134.09018:24780 147.10149:25984 | 8.61637E-06 |
| POS6640 | Salicin | 0.892 | 304.25275 | [M+H]+ | 304.2554 | 110.79853:5377 118.67968:5474 | -8.70979E-06 |

**Differences in metabolites between the Model group and the WJW group**

| Alignment ID | Metabolite name | Rt(min) | Expriment Mz | Adduct type | Reference m/z | MS/MS spectrum | PPM |
|---|---|---|---|---|---|---|---|
| POS9356 | an-2-yl)-10,13-di | 5.677 | 461.32129 | [M+2H]2+ | 461.32401 | 57.03286:34728 59.04858:503341 60.05225:12222 63.82409:6740 73.02811:22106 73.06431:9679 85.06335:6543 87.04333:29484 87.07997:10785 89.05894:144769 90.0619:6752 94.06945:9436 101.05929:31698 103.07442:97789 117.09087:12654 133.08542:26507 147.10147:25154 161.11877:7539 | -5.89607E-06 |
| NEG3634 | ioxa-1,13-trideca | 1.32 | 219.16878 | [M-H2O-H]- | 219.17149 | 97.07645:143213 97.36658:5572 99.07355:118894 102.80595:5628 161.03622:6788 | -1.23647E-05 |
| POS8449 | Celerioside E | 4.781 | 405.24515 | [M+Na]+ | 405.24789 | 59.04858:12415 73.02811:5900 87.04333:5505 89.05894:18713 108.92649:5637 133.08542:6137 173.58739:6753 176.6682:5636 216.32614:5671 354.49524:6265 | -6.76129E-06 |
| POS7473 | Piperolein B | 4.464 | 344.22577 | [M+NH4]+ | 344.22299 | 87.04335:17462 89.05896:404679 90.06375:6687 97.43122:6500 133.08545:143591 177.11285:12752 | 8.07616E-06 |
| POS9869 | Pro-aMCA | 8.777 | 488.33322 | [M+2H]2+ | 488.336 | 91.88431:5876 149.21219:6338 | -5.6928E-06 |
| POS9870 | ydroxydocosa-4,7, | 4.81 | 488.33981 | [M+NH4]+ | 488.33701 | 57.03286:24280 59.04858:739728 60.05225:10445 73.02811:16689 73.06431:10252 85.06335:13911 87.04333:98766 87.07997:18103 89.05894:805009 90.0619:14342 101.05929:82125 103.03848:10202 103.07442:282660 103.96793:6582 107.07057:7414 117.05279:26650 117.09087:13071 129.0891:12148 131.07086:12641 131.10631:7236 133.08542:227288 145.08401:9229 147.10147:140270 160.1046:6894 161.11438:15337 177.11282:10423 191.12556:21180 | 5.73375E-06 |
| POS7672 | s-Cyclosuffrobuxi | 6.034 | 354.2821 | [M+H]+ | 354.2793 | 55.05366:20769 57.06897:29820 59.03105:17005 59.04859:185439 61.01003:7553 73.04689:9508 75.02637:12812 87.04335:29207 89.05897:6684 91.05764:9443 103.07445:209723 131.08055:5746 | 7.90337E-06 |
| POS8211 | eucylglutaminyllys | 4.476 | 388.25256 | [M+Na]+ | 388.2554 | 73.06432:11103 87.04334:33327 89.05896:786442 90.0619:16625 107.07059:7810 131.06766:9936 133.08543:294603 134.08684:8135 173.39601:8740 177.11285:39299 | -7.31477E-06 |
| POS8206 | Neoenactin NL2 | 6.197 | 387.28244 | [M+2H]2+ | 387.28531 | 57.03287:41392 59.04858:357778 60.05225:7977 75.11298:5347 87.04334:34951 99.07938:8139 115.07378:10366 117.09089:21867 206.25749:6138 273.48544:5697 | -7.41056E-06 |
| POS14654 | -cyclopenta[a]phe | 4.648 | 743.42847 | [M+2H]2+ | 743.42413 | 72.4287:86873 80.15437:81887 87.04334:542368 89.05896:2429276 133.08543:902522 150.55998:93019 156.23862:78811 177.10779:106599 238.40434:82686 461.10242:89324 | 5.83785E-06 |
| POS12413 | 1alpha,25-dimeth | 4.757 | 589.37689 | [2M+H]+ | 589.37341 | 59.04859:207411 73.02812:28959 73.06432:17706 83.31817:11252 87.04335:127032 87.07999:13074 89.05896:909512 90.06191:18586 92.93743:10793 101.05931:55264 103.07444:76697 115.78223:12321 131.07088:21915 133.08545:333416 147.10149:45428 173.43526:12231 177.11285:26196 183.19818:10830 265.44962:12614 358.28094:12755 | 5.90458E-06 |
| POS8184 | osahexaenoyl Gly | 4.669 | 386.27216 | [M+NH4]+ | 386.2692 | 59.04859:166957 87.04335:13037 89.05896:192528 99.55555:6222 101.05931:10841 103.07444:50844 117.05281:11954 133.08545:55275 147.10149:27949 277.32327:6236 | 7.66305E-06 |
| POS15168 | PC(17:1/12-HEPE) | 8.623 | 808.54535 | [M+2H]2+ | 808.54858 | 50.21645:5920 54.8856:6263 57.75751:6358 173.39111:9749 497.72522:5572 | -3.99481E-06 |
| POS9130 | Malyngamide M | 4.648 | 450.27386 | [M+2H]2+ | 450.27689 | 58.04042:159330 69.03294:25197 71.04816:13779 73.02811:383167 73.06432:44608 74.03149:12087 80.05429:234713 80.55504:12762 87.04334:122674 89.05896:898052 90.0619:49972 102.06606:155110 102.56839:13871 111.07206:13246 117.09089:10217 124.08022:47892 133.08543:271057 134.09018:13289 146.09132:11754 173.43034:13484 177.11285:22689 289.82092:10072 | -6.72919E-06 |
| POS6925 | ,6-Dimethyl-2,5-h | 4.716 | 317.15616 | [M+Na]+ | 317.15921 | 58.04042:9487 59.04859:41583 73.02812:39470 80.0543:10378 89.05896:57821 133.08545:13578 173.43036:13731 173.4745:5458 176.86003:5563 207.79285:5325 | -9.61662E-06 |
| NEG4055 | Dropropizine | 0.932 | 235.14226 | [M-H2O-H]- | 235.14529 | 93.03925:684991 95.03764:5941 103.07947:15903 124.15677:24049 177.1145:28349 | -1.28857E-05 |
| POS781 | 2-Cyanopyridine | 0.767 | 105.00304 | [M+Na]+ | 105 | 58.06416:80101 58.99696:15260 61.1515:5169 68.98123:483576 77.00752:6727 86.99104:609953 87.99284:5427 90.89735:6337 105.0024:672241 | 2.89524E-05 |
| POS13219 | baeckein F | 8.78 | 629.09644 | [M+H]+ | 629.09253 | 67.19205:6851 69.94729:7347 95.36812:6350 100.12971:5204 282.84961:6618 | 6.2153E-06 |
| NEG635 | Isocaproic acid | 5.528 | 115.07261 | [M-H2O-H]- | 115.0757 | 59.08403:7579 69.07899:6232 98.04861:26594 100.04587:57877 103.87704:5463 115.07258:24219 116.05923:61702 | -2.68519E-05 |
| POS12920 | limnantheoside A | 4.7 | 613.362 | [M+H]+ | 613.35822 | 73.0281:27054 73.06429:10545 79.63444:12220 80.05273:13791 87.04332:72595 89.05893:308858 90.06371:25293 95.0489:11224 113.05666:13771 133.0854:121564 | 6.16279E-06 |
| POS10097 | )-ethyl)-1H-indol | 5.375 | 499.34631 | [M+H]2+ | 499.34311 | 57.06989:6828 59.04859:39692 60.08025:63374 73.02812:7840 86.09541:121291 87.04335:54077 87.09918:40902 89.05896:41583 101.05931:12329 103.07445:11368 104.10697:595170 105.10879:221811 106.11105:73269 110.11542:7478 124.99956:26496 127.00428:7786 131.83476:7331 133.08545:14332 184.07466:188160 185.07635:108954 186.07535:100589 187.07695:9257 318.11121:7126 | 6.40842E-06 |
| POS11664 | Sepaconitine | 6.214 | 559.29767 | [M+2H]2+ | 559.30127 | 50.40268:8965 52.1749:6583 53.81686:5946 68.18956:5778 104.10696:18576 224.66388:6301 240.11317:5511 245.61572:5266 266.03574:5919 502.1665:7462 | -6.4366E-06 |

| Differences in metabolites between the Model group and the WJW group | | | | | | | |
|---|---|---|---|---|---|---|---|
| Alignment ID | Metabolite name | Rt(min) | Expreiment Mz | Adduct type | Reference m/z | MS/MS spectrum | PPM |
| POS8769 | Andrastin D | 4.637 | 428.25958 | [M+H]2+ | 428.25629 | 58.04042:189786 69.03294:19953 71.04816:16872 73.02811:452445 73.06432:55546 74.03149:12153 80.05429:357769 80.55504:20817 87.04334:174921 88.04782:9696 89.05896:1442791 90.0619:64847 91.06323:9601 99.04339:10211 102.06606:253790 102.56839:18867 107.06821:11122 111.07206:13912 113.05927:11019 115.07378:10454 117.09089:16175 124.08022:101969 124.58041:11349 131.07088:20308 133.08543:466748 134.08684:22188 146.09132:30048 155.09987:29479 177.11285:51265 | 7.68232E-06 |
| POS9032 | Yibeissine | 4.8 | 444.31442 | [M+NH4]+ | 444.3111 | 57.03286:35610 59.04858:1024461 59.0593:26431 60.05225:16286 73.02811:12469 73.06431:18139 83.04768:7020 85.06504:16909 87.04333:86153 87.07997:17599 89.05894:779869 90.0619:15131 99.07938:6169 101.05929:79470 103.03848:14076 103.07442:363566 105.09026:8277 115.07376:6370 117.05279:68029 117.09087:20251 129.09224:7563 131.07086:11202 131.10631:6301 133.08542:179811 145.08401:9892 147.10147:178089 161.11438:20425 173.396:8609 191.12556:18245 205.14235:7523 | 7.47224E-06 |
| POS9287 | adecahydro-3,5-d | 4.85 | 458.29333 | [M+NH4]+ | 458.29001 | 59.04859:127912 62.9291:6736 87.04335:275102 89.05896:95178 101.05931:69613 103.0385:62724 103.07445:31513 110.84615:5616 117.05281:34988 131.07088:8119 133.08545:33611 136.56493:5515 147.10149:8298 180.89824:5683 | 7.24432E-06 |
| POS3236 | L-Tyrosine | 2.199 | 182.08022 | [M+H]+ | 182.08118 | 77.03802:15550 91.05391:637734 92.05669:18134 93.06841:8684 95.04894:297246 103.05422:18541 107.04918:17582 109.06337:19536 118.06567:14021 119.04707:396222 120.05206:9361 121.06411:9132 123.04193:399989 124.04758:7692 136.07611:448969 137.07706:13726 147.04404:69719 165.05461:33159 | -5.27237E-06 |
| POS2411 | Histidine | 1.055 | 156.07626 | [M+H]+ | 156.07678 | 56.04861:28400 68.04948:13371 81.04324:6644 82.05224:18332 83.05906:110927 93.04333:76341 95.05887:33226 100.90237:6143 104.79472:5957 110.07073:347611 | -3.33169E-06 |
| POS1770 | Hypoxanthine | 4.373 | 137.04532 | [M+H]+ | 137.04578 | 55.02822:373014 57.06896:12667 67.02796:86950 67.05389:58725 72.93707:26646 79.05313:37936 81.06834:159572 82.03949:271530 83.02332:40703 91.0539:79146 92.02255:44135 94.03812:523453 95.08477:94932 110.03357:1091621 112.04887:62715 119.03312:800626 120.01816:13134 128.04344:37191 137.04604:3745190 138.02985:30815 | -3.35654E-06 |
| POS1184 | L-Valine | 1.106 | 118.08609 | [M+H]+ | 118.08628 | 53.01368:8607 53.03856:65233 55.05365:3077097 56.04952:54851 57.05414:55938 57.05692:310822 58.06511:25899 59.04858:57039 59.07294:20713 71.06873:8607 72.08047:4019858 73.06431:7744 73.08443:10666 118.08493:16295 | -1.60899E-06 |
| POS5832 | auryldiethanolamir | 5.023 | 274.27338 | [M+H]+ | 274.27408 | 55.05365:20481 57.06896:921924 58.06416:58013 58.07272:18651 60.04325:14176 62.05909:51561 68.04829:8100 69.06991:12514 70.0643:945401 71.06745:19545 71.08418:297450 72.08836:10263 74.05885:7639 85.10044:81364 86.05938:14506 88.07444:1278210 89.07881:29560 102.09042:352008 103.09467:7840 106.08522:681112 107.08961:13477 212.23726:7148 230.24779:8332 256.25845:32904 274.2739:217946 275.27167:24002 | -2.55219E-06 |
| POS4078 | Kynurenine | 4.336 | 209.09169 | [M+H]+ | 209.09209 | 74.02328:33601 91.0539:6350 94.06359:112013 99.00742:20398 104.04768:13858 118.0629:36202 120.04358:39052 132.04306:15007 136.07611:15142 146.05721:51480 150.05342:7705 174.05498:15598 | -1.91303E-06 |
| POS3014 | DL-Arginine | 1.029 | 175.11815 | [M+H]+ | 175.11897 | 60.05526:769510 70.06431:2166182 71.04816:42605 71.06746:29367 72.08048:76556 84.07957:21869 88.07445:9948 97.07488:9417 98.06007:10224 112.08709:35482 113.0696:19749 115.08704:8718 116.06905:236146 130.09663:114071 134.01685:9323 158.09198:33378 175.12015:47736 | -4.68253E-06 |
| POS2215 | Methionine | 1.565 | 150.05785 | [M+H]+ | 150.05827 | 53.03773:28598 56.04862:4098100 57.03286:40299 57.05229:26356 58.99405:43779 61.01003:3042040 74.02328:200846 74.05885:141817 74.98869:16092 75.02496:27598 77.00462:19300 84.04315:60120 85.00947:23833 85.02799:62622 87.0259:334150 102.05498:226649 104.05223:569591 105.00008:19749 105.03476:23692 133.02939:316994 150.05736:14543 | -2.79891E-06 |
| POS161 | Tetrahydropyridine | 1.102 | 84.08079 | [M+H]+ | 84.08077 | 55.05365:23097 56.04862:215522 57.06988:9418 67.05389:28831 69.05635:8003 82.06502:37404 82.94376:11047 84.04315:141536 84.07957:511719 | 2.37867E-07 |
| POS3070 | Serotonin | 3.359 | 177.10153 | [M+H]+ | 177.10223 | 55.93435:49513 67.04092:27707 68.70155:5785 72.93707:17109 72.96115:9613 79.05313:7960 90.94764:6919 91.0539:13002 94.04205:7205 95.9206:5567 105.06946:43974 115.05257:174659 117.05553:76819 130.06477:10756 131.04832:11171 131.07088:9076 132.07892:159173 133.06236:28355 135.94325:6628 142.06567:27246 143.07104:15197 159.06984:16603 160.07417:785549 161.07928:20445 174.8517:5705 | -3.95252E-06 |

**Differences in metabolites between the Model group and the WJW group**

| Alignment ID | Metabolite name | Rt(min) | Expreiment Mz | Adduct type | Reference m/z | MS/MS spectrum | PPM |
|---|---|---|---|---|---|---|---|
| POS6593 | adecyldiethanolar | 5.238 | 302.30368 | [M+H]+ | 302.30539 | 55.05366:13204 57.06897:611891 58.06512:28764 58.07272:12523 60.04426:8334 62.0591:34532 64.82231:6567 68.0483:9617 69.06869:8540 70.06431:594018 71.08548:215123 72.08837:7588 85.10046:75133 86.05939:9152 88.07445:808188 89.07883:13366 102.09042:211452 106.08524:437557 107.08725:6986 152.76207:6151 194.68724:6670 284.29575:9125 302.30051:69166 303.30765:7273 | -5.65653E-06 |
| POS2981 | Hexanoylglycine | 4.763 | 174.11218 | [M+H]+ | 174.11247 | 55.05365:13064 71.08547:378903 76.03858:429061 76.36717:6444 81.06833:31898 86.0954:13485 99.07938:93356 110.10796:5561 | -1.66559E-06 |
| POS2296 | Xanthine | 2.151 | 153.0399 | [M+H]+ | 153.04068 | 55.02822:218475 55.05366:12446 55.93435:8781 79.05313:12347 81.00723:29302 82.03949:98438 83.02332:23846 83.04932:7681 92.0491:17941 93.00867:15229 107.04918:12248 108.01909:12309 108.04321:9568 110.03357:754289 110.06084:19264 128.04344:78686 136.01477:29202 153.04224:170907 154.0228:243066 | -5.09668E-06 |
| POS10644 | LysoPC(18:1/0:0) | 5.57 | 522.35522 | [M+H]+ | 522.35541 | 58.06512:449133 60.08025:4693632 67.05389:258153 69.06992:217609 71.07261:665162 81.0699:254886 83.0851:210672 86.09541:11328304 89.05896:197113 95.08478:374162 104.10697:67285736 105.10879:1243121 124.99956:3462611 163.01524:388928 184.07466:61771484 185.07635:1554875 258.10977:355226 339.29205:229168 | -3.63737E-07 |
| POS10646 | ysoPC(18:1(11Z)/0: | 8.803 | 522.35553 | [M+H]+ | 522.35596 | 60.08025:221595 71.07261:35196 86.09541:701630 104.10697:335647 124.99956:210444 184.07466:4791878 185.07635:136659 | -8.23193E-07 |
| POS11922 | 5(4Z,7Z,10Z,13Z,16 | 6.131 | 568.33942 | M+CH3OH+H] | 568.34033 | 56.04953:11079 59.04858:26899 60.08025:262932 71.0726:47332 86.09541:749017 87.09918:21258 89.05896:15985 98.98203:14355 104.10696:143380 124.99955:252441 133.08543:14529 184.07466:3618798 185.07635:86007 | -1.60115E-06 |
| POS1735 | Benzothiazole | 5.225 | 136.02126 | [M+H]+ | 136.02158 | 65.03805:7972 109.00957:24687 136.02158:109387 | -2.35257E-06 |
| POS1188 | D-Valine | 2.589 | 118.08617 | [M+H]+ | 118.08626 | 55.05363:412497 56.9423:10025 57.0569:33033 58.06509:10545 59.04856:11710 72.08044:483927 72.93703:7811 | -7.62155E-07 |
| POS5488 | nma-Glutamylleuc | 4.428 | 261.14288 | [M+H]+ | 261.14429 | 56.04952:47760 69.06991:56244 84.04315:1149629 85.02799:15357 85.04652:24617 86.05938:56530 86.0954:2495348 87.09917:64088 102.05498:30532 114.05461:34603 130.04884:207874 132.10173:511789 133.10521:15584 142.04747:64415 152.10725:34450 169.08443:25634 170.11852:16372 198.10959:74884 244.118:14015 | -5.39931E-06 |
| POS1120 | Proline | 1.038 | 116.0703 | [M+H]+ | 116.07058 | 52.41612:6575 54.56131:6288 56.04953:7301 56.55671:5899 58.06512:10102 59.07198:7702 68.0495:25256 70.0643:5008722 71.06874:10617 80.4635:5832 101.43783:6059 116.06905:173371 | -2.41233E-06 |
| POS2701 | nol-phenylpyruvat | 2.2 | 165.05429 | [M+H]+ | 165.05479 | 51.02237:6584 57.03287:7438 61.03972:6501 65.03805:19987 67.05389:14565 69.03294:14200 77.03802:40323 91.0539:159150 93.06841:8331 95.04894:567612 96.05188:7459 103.05422:24102 105.04402:33986 109.06337:24149 119.04707:243123 120.08031:7830 121.0641:7408 123.04193:323178 124.04758:6472 147.04404:25713 | -3.0293E-06 |
| POS2818 | Uric acid | 1.747 | 169.03532 | [M+H]+ | 169.03558 | 55.02822:192629 55.05365:10007 62.44093:5208 67.05389:13338 69.00832:71887 70.03912:370193 81.06833:14998 83.02332:8731 91.68036:5230 96.01751:146140 98.03504:216974 99.01799:24070 109.00224:8843 114.03108:5956 123.0302:7455 124.01198:48054 126.02935:290713 127.03194:7306 131.69183:5995 141.03847:532027 142.04385:5920 144.03883:5866 152.00659:160570 169.03252:387130 170.01851:46635 | -1.53814E-06 |
| POS8577 | lonaethylene glyco | 4.509 | 415.25272 | [M+H]+ | 415.25378 | 73.06432:16324 86.09541:11486 87.04334:57259 89.05896:974340 90.0619:14109 107.07059:8721 117.09089:6569 131.07088:8163 133.08543:338896 134.08684:10247 177.11285:27933 207.3625:5970 257.82501:6144 380.27573:5685 | -2.55266E-06 |
| POS7979 | yristoyllevocarnitir | 5.356 | 372.30908 | [M+H]+ | 372.31079 | 51.56357:6742 57.03287:15133 57.06896:18033 60.08025:185054 67.05389:6094 71.08547:16644 85.02799:971725 86.03024:16129 95.08477:8937 144.10197:17959 244.00337:6547 | -4.59294E-06 |
| POS1578 | -Dihydronaphthal | 4.479 | 131.08511 | M+CH3OH+H] | 131.08553 | 53.03773:26525 56.04953:10752 57.06896:28708 65.03805:28931 67.05389:8711 69.06868:6259 74.09583:8711 84.04316:19602 84.07957:34050 85.0836:6501 86.09541:22325 87.00324:18490 88.00348:8670 91.0539:1673423 92.05669:10880 95.04894:8214 97.00918:26390 103.05421:41006 105.06946:7580 115.05257:62450 116.061:176421 128.06212:19383 129.06706:119311 130.06477:31461 130.15721:9880 131.08377:775724 132.08543:8337 133.06566:6404 | -3.20401E-06 |

| Differences in metabolites between the Model group and the WJW group | | | | | | | |
|---|---|---|---|---|---|---|---|
| Alignment ID | Metabolite name | Rt(min) | Expreiment Mz | Adduct type | Reference m/z | MS/MS spectrum | PPM |
| POS10715 | LysoPC(18:0/0:0) | 8.808 | 524.36963 | [M+H]+ | 524.37109 | 54.19788:89428 57.03287:95803 57.06989:193802 58.06512:106852 60.08025:1369087 66.97967:87274 71.07261:174615 81.0699:101604 86.09541:3593029 98.24385:89434 104.10697:20061236 105.10879:462525 124.99956:987458 163.01524:135640 184.07466:20082530 185.07635:541792 232.21115:92139 258.10977:97197 288.29056:88176 | -2.78429E-06 |
| POS3512 | ole-3-propionic a | 5.052 | 190.08539 | [M+H]+ | 190.08626 | 55.0177:842102 56.02069:8567 57.03379:6661 67.05389:15577 73.02811:9550 91.05389:18599 103.05421:11714 105.03245:17434 105.06945:8788 107.04917:50390 108.05043:9465 130.06477:2285724 131.06764:84330 144.07968:18119 172.07471:75956 190.08571:14212 | -4.57687E-06 |
| POS5115 | I-Acetyltryptopha | 4.741 | 247.10725 | [M+H]+ | 247.10768 | 118.0629:26566 130.06477:64358 132.07892:48513 142.06567:9048 144.0797:20273 146.05721:74183 159.09138:159110 170.05661:15699 173.42545:8643 187.08794:9341 188.07004:58967 201.10141:15716 | -1.74013E-06 |
| POS8386 | -Palmitoylcarnitin | 5.562 | 400.34106 | [M+H]+ | 400.34207 | 55.05366:9409 57.03287:67078 57.06896:102012 58.06512:12737 60.08025:814513 61.08379:10726 67.05389:14313 69.06868:21933 71.08547:66770 81.0699:39890 83.0851:33708 85.028:4222678 85.10045:39216 86.03025:64784 89.05896:8238 95.08477:49160 97.09953:14544 103.07444:7100 109.10007:31112 123.11524:16860 125.13172:6703 135.66116:6320 137.13222:10360 144.10197:90033 174.76732:6511 239.23662:17393 | -2.52284E-06 |
| POS10708 | telet-activating fa | 6.595 | 524.36841 | [M+H]+ | 524.37097 | 53.9196:8265 57.06989:10359 60.08025:51861 64.15609:7760 71.0726:8861 86.0954:182323 101.47952:7156 104.10696:45818 124.99955:59156 160.51437:7745 184.07465:693771 185.07634:14888 | -4.88204E-06 |
| POS2119 | D-Lysine | 1.052 | 147.11206 | [M+H]+ | 147.1129 | 55.05365:21431 56.04862:311733 60.08025:127686 61.08378:10757 67.05389:41656 70.0643:13652 72.08047:8148 74.02328:13541 84.04316:1430594 84.07957:1365331 85.02799:28264 85.04652:15170 85.08359:10862 87.04334:174690 88.04782:11844 100.07382:9690 101.07021:11646 102.05499:25163 130.04884:137022 130.08389:54141 146.11787:20073 | -5.7099E-06 |
| POS4571 | cyclotetradecane- | 4.475 | 227.17447 | [M+H]+ | 227.17529 | 55.05365:298983 58.07272:6155 64.70701:6480 69.06992:11014 81.0699:6881 82.06343:5855 83.0851:96790 96.08018:20307 100.11039:1132751 101.11388:29135 109.10007:7135 110.10548:5919 112.84786:6464 114.09126:9909 117.13717:10401 139.08766:7866 209.16336:30335 227.17255:16373 | -3.60955E-06 |
| POS1673 | o-Tolyl isocyanate | 4.856 | 134.05936 | [M+H]+ | 134.06 | 77.03802:14145 78.04544:8286 79.05312:146398 79.23306:6396 87.09918:9982 88.02121:9864 95.04893:31499 104.04768:26491 105.03246:75496 106.06411:712941 107.06821:9029 120.69875:6478 133.05247:21390 134.06017:283645 | -4.77398E-06 |
| POS2543 | 2721-59-7 | 3.357 | 160.0753 | [M+H]+ | 160.07561 | 52.64342:6544 55.0177:14037 55.05365:20891 55.93435:52322 56.94139:43783 57.06988:6318 65.03804:8303 67.04092:46275 67.05389:7590 69.06991:22072 71.04816:6935 72.08047:7876 72.93706:46772 74.95245:8165 79.05312:25691 90.94762:27540 91.05389:21823 94.04007:9009 95.04893:7258 96.08826:6989 103.05421:9639 105.06944:110273 107.04678:6672 113.9631:30824 114.09124:7417 115.05256:338584 116.97396:6430 117.05552:188538 118.06289:16734 130.06476:23657 131.04831:14488 131.07086:21686 131.97139:9239 132.0789:260377 132.97009:15510 133.06564:43156 142.06566:59329 143.07103:39725 159.06551:36453 160.07416:622196 161.07925:12631 162.91693:5901 | -1.93658E-06 |
| POS5704 | Inosine | 4.382 | 269.08707 | [M+H]+ | 269.08801 | 55.0177:37760 55.0291:33671 55.78193:7005 57.03287:64369 59.04859:7084 61.02846:9158 67.02914:10736 69.03294:15844 73.02812:24585 82.03949:29577 85.028:23898 94.04008:62874 110.03357:124679 115.03932:10195 119.0359:90606 133.04918:9042 137.04604:5792410 138.04726:214745 | -3.49328E-06 |
| POS4708 | Butyrylcarnitine | 4.389 | 232.15369 | [M+H]+ | 232.15421 | 57.03287:82378 58.06512:12276 60.08025:393758 71.04816:65210 72.08047:16666 84.07957:47991 85.028:3262740 86.03024:66696 86.09541:21691 126.09014:13972 144.10197:46354 173.07771:70441 232.15034:32028 | -2.23989E-06 |
| POS3508 | Acetyl-L-glutamic | 2.201 | 190.07033 | [M+H]+ | 190.07098 | 56.04861:6674 84.04314:94451 102.05497:10788 126.02325:5684 130.04883:35527 | -3.41978E-06 |

**Differences in metabolites between the Model group and the WJW group**

| Alignment ID | Metabolite name | Rt(min) | Expreiment Mz | Adduct type | Reference m/z | MS/MS spectrum | PPM |
|---|---|---|---|---|---|---|---|
| POS5318 | Actinopolysporin ( | 6.075 | 255.2303 | [M+H]+ | 255.23151 | 55.0177:7581 55.05365:215649 57.03287:22659 57.06896:267334 59.04858:9281 67.05389:59720 69.06868:223862 71.04945:11979 71.08547:109749 73.06432:8417 78.87981:6127 79.05312:31609 81.06834:96833 83.04932:8425 83.08509:156102 85.06337:6585 85.10045:31519 93.06841:68014 95.08477:76990 96.08625:6932 97.06461:39662 97.09953:93929 99.07939:6303 101.05712:9242 107.08486:62857 109.10006:45041 111.0796:24489 111.11481:18312 121.09843:56798 123.11523:17064 125.09565:19197 135.11646:56832 137.1322:6945 139.11234:13492 149.13002:27740 153.12358:9400 157.46205:6511 163.14494:11539 167.14175:8147 219.21098:7793 | -4.74079E-06 |
| POS1050 | Creatinine | 1.126 | 114.06573 | [M+H]+ | 114.06618 | 68.0495:10084 70.0643:10541 72.04369:19987 84.48338:6386 86.07139:35948 111.68296:5557 114.06509:147086 | -3.94508E-06 |
| POS9749 | yl-sn-glycero-3-p | 6.116 | 482.36169 | [M+H]+ | 482.36047 | 57.03286:28980 57.06895:63700 59.04858:22687 60.08024:71295 71.08547:41875 75.04311:10959 81.06833:9913 83.08509:8698 85.10044:24531 86.0954:111504 89.05895:12491 95.08476:8318 97.10158:6672 104.10696:1097444 105.10878:27839 109.10005:7273 124.99954:36928 173.43033:10886 184.07465:41126 | 2.52923E-06 |
| POS10032 | nitoylphosphatidyl | 5.109 | 496.33975 | [M+H]+ | 496.33978 | 57.06893:305017 58.06509:151177 60.08022:1846755 64.85479:109228 71.07257:252067 71.08544:138140 82.89832:114492 86.09536:4277546 95.08472:157403 104.10692:23310622 105.10873:612520 124.9995:934110 163.01515:138220 165.84062:115113 184.07457:11627109 185.07626:200921 308.44281:114198 | -6.04425E-08 |
| POS7339 | 13-Docosenamide | 8.427 | 338.34024 | [M+H]+ | 338.34167 | 55.0177:8204 55.05365:82802 57.03286:15973 57.06895:169688 58.06416:6364 67.05389:47175 69.06991:155934 71.04816:9116 71.08546:98556 72.08047:14585 73.52791:5775 79.05312:12140 81.06989:81173 83.08509:175609 85.10044:47719 86.05938:22227 93.0684:24825 95.08476:95549 97.0646:36675 97.09953:141619 100.07381:41903 107.08485:30694 109.10005:61223 111.07959:41175 111.1148:59469 114.09125:51521 121.10129:58797 123.11523:34047 125.09564:23458 125.13171:22326 128.10568:30349 135.11644:57188 137.12875:13189 139.11232:13424 142.12386:16195 149.13:41095 153.12764:6558 156.13799:12623 163.14493:14298 167.14174:7100 255.61653:6275 296.59183:5912 | -4.2265E-06 |
| POS5467 | -Hexanoylcarnitin | 4.523 | 260.18475 | [M+H]+ | 260.18558 | 57.03286:16321 60.08025:87561 71.08418:20259 85.02799:751638 86.03195:13108 99.07938:16217 144.10197:9767 201.11363:7309 | -3.19003E-06 |
| POS5882 | D-Glutarylcarnitine | 3.222 | 276.14291 | [M+H]+ | 276.14417 | 57.03287:8411 60.08025:50595 85.02799:168427 87.04334:33049 103.03849:11218 115.03932:24286 144.10197:6565 235.2888:5383 | -4.56283E-06 |
| POS8575 | Gelomulide N | 5.751 | 415.2103 | [M-H2O+H]+ | 415.211 | 65.03805:7164 69.03294:8960 77.03802:6531 79.05313:6739 81.03386:6738 91.0539:42167 91.11925:7534 104.06136:7053 105.06946:7622 107.08487:11777 117.06913:11433 119.08334:2012474 120.08878:79446 133.06236:23733 135.07936:19701 | -1.68589E-06 |
| POS7458 | uramidopropylbeta | 5.351 | 343.29492 | [M]+ | 343.2955 | 57.06895:68496 58.06416:29621 71.08418:38504 76.07277:6028 81.06989:9508 83.08508:10148 85.10044:20196 95.08476:31625 109.10005:30042 123.11523:7812 155.90779:5375 173.43033:7572 183.17154:29559 240.23306:237476 241.23578:19036 | -1.68951E-06 |
| POS6062 | Oleamide | 8.825 | 282.27826 | [M+H]+ | 282.27908 | 51.70781:6117 55.05365:55737 57.03287:7605 57.06896:66412 57.61167:6213 67.05389:18045 69.06868:96769 71.08547:32424 81.0699:32367 83.08509:53084 85.10045:10326 86.05939:7345 86.2397:5472 93.06841:11368 95.08477:26417 97.06461:14262 97.09953:33756 100.07597:13617 107.08486:22620 109.10006:20293 111.0796:10966 111.11481:20975 114.08864:10737 121.1013:10895 123.11523:8425 135.11646:11592 149.13002:6796 | -2.90493E-06 |

**Differences in metabolites between the Model group and the WJW group**

| Alignment ID | Metabolite name | Rt(min) | Expreiment Mz | Adduct type | Reference m/z | MS/MS spectrum | PPM |
|---|---|---|---|---|---|---|---|
| POS5032 | -Undecanoylglycir | 5.123 | 244.18991 | [M+H]+ | 244.19051 | 55.01682:31910 55.05365:249817 56.04862:22095 57.03286:23057 57.06895:138638 58.06416:22317 59.04858:6940 67.05389:128244 69.06867:175357 70.0643:10450 71.04816:25624 71.08418:61457 72.08047:27603 79.05312:63333 81.06833:253740 82.06342:11569 83.04932:10233 83.08508:255996 84.07957:14624 85.06336:23497 85.10044:9918 86.05938:35246 86.0954:13572 91.05389:14000 93.0684:96198 95.08476:202887 96.08018:17514 97.0646:54115 97.09953:140084 98.09552:13043 99.07938:11830 100.07381:44311 100.11037:11172 102.05498:12926 105.06944:10460 107.08485:147261 108.08662:8437 109.10005:39996 110.09555:34732 111.07959:47528 114.09124:36105 119.08332:7507 121.10129:108901 123.11523:22668 124.1099:51772 125.09564:30075 128.10568:13750 131.08376:34733 135.11644:27705 137.13219:8729 138.1274:37965 142.12386:7647 145.10278:5768 147.11681:7541 149.13:6646 156.17151:10797 163.14493:117767 164.15204:7861 167.74667:5342 173.1315:30440 180.17407:189833 181.15976:27629 190.15889:7151 198.18146:15198 226.17574:87363 | -2.4571E-06 |
| POS4651 | Lauramine oxide | 5.036 | 230.24738 | [M+H]+ | 230.2476 | 55.05365:16217 57.06896:495944 58.06416:66882 58.07177:10942 62.05909:265085 69.06991:8703 71.08418:155775 85.10044:44003 93.06841:10097 167.10463:8934 173.39601:7315 212.23726:40352 230.24779:246622 231.24898:15788 | -9.55493E-07 |
| POS858 | O-Toluidine | 4.135 | 108.08038 | [M+H]+ | 108.08078 | 65.03805:7553 91.05391:21506 93.05685:8486 108.07939:9923 | -3.70094E-06 |
| POS1791 | gamma-Terpinene | 1.204 | 137.13217 | [M+H]+ | 137.13251 | 53.03773:18258 55.05365:44628 57.06896:22098 65.03805:13369 67.05389:96747 69.06992:23501 79.05312:56781 81.06834:278486 91.0539:45570 93.06841:14718 95.08477:138938 109.10007:6333 110.03356:10919 119.03311:11075 136.05907:16809 137.04259:13538 137.1322:6631 | -2.47935E-06 |
| POS3648 | Phenylacetylglycine | 4.656 | 194.08057 | [M+H]+ | 194.08118 | 53.29583:5855 65.03804:5998 76.03858:359475 91.05389:813066 92.05668:25266 120.0803:7288 135.04227:8015 177.25467:5430 | -3.14301E-06 |
| POS587 | Phosphate | 1.183 | 98.98412 | [M+H]+ | 98.98418 | 53.03773:28313 55.0177:12374 55.05365:16439 56.04862:9275 57.06989:19752 58.06416:5947 60.44798:6097 62.96235:14024 70.0643:22148 71.04945:11951 72.03056:5830 79.05312:9777 80.97279:19262 81.0699:16712 81.54541:6228 98.06007:6323 98.09553:6363 98.98415:1035833 | -6.06157E-07 |
| POS2040 | -Methyleneglutara | 9.911 | 145.04929 | [M+H]+ | 145.04939 | 53.03774:301799 55.0177:188558 55.05366:29937 56.04953:11584 56.9414:7899 57.03287:138126 57.06989:6914 59.04858:13138 67.01736:6858 69.03294:30235 70.06431:62151 71.01216:17320 71.04816:385264 72.93707:11418 81.0323:93678 81.0699:14753 84.99097:13324 85.028:42224 98.06007:25728 98.09553:25813 99.0434:540241 101.02222:10793 127.0381:52379 145.04651:18737 | -6.8942E-07 |
| POS10710 | enantio-PAF C-16 | 5.717 | 524.36853 | [M+H]+ | 524.37018 | 60.08023:659119 71.0713:114821 86.09538:2224914 99.50859:76156 104.10693:924207 105.65473:68598 124.99951:644511 150.57184:80642 173.4303:120925 180.49649:74015 184.0746:13549719 185.07629:272102 235.66122:75223 | -3.14663E-06 |
| POS10193 | 27,30-Decaoxadot | 4.529 | 503.30334 | [M+H]+ | 503.30618 | 73.06432:59612 87.04335:267868 89.05896:3006350 90.06191:63603 91.07443:15489 107.06821:24209 117.0909:21221 131.06766:59487 133.08545:1329197 134.09018:36332 173.42545:18521 175.09528:14502 177.11287:152154 | -5.64269E-06 |
| POS3510 | ole-3-methyl acet | 5.322 | 190.08531 | [M+H]+ | 190.08627 | 69.89706:5812 103.05418:6452 130.06473:289564 131.06761:12461 | -5.05034E-06 |
| POS6792 | nma-Glutamyltyro | 4.376 | 311.12238 | [M+H]+ | 311.1235 | 55.42663:5924 55.52425:6174 70.06431:37661 84.04317:139861 84.07958:22231 91.0539:11376 95.04894:7660 102.05277:7646 119.04707:56794 120.08031:10712 123.04193:85722 130.04886:25579 130.08708:7867 136.07611:194826 137.07704:12027 147.04404:37702 161.81059:5585 163.28825:5993 165.05461:131356 180.08583:8347 182.0797:35967 202.08495:11454 | -3.59986E-06 |
| POS10588 | oPC(0:0/18:2(9Z,1 | 6.252 | 520.33984 | [M+H]+ | 520.33929 | 56.04953:104351 58.06512:138990 60.08025:2771459 67.05389:276732 69.06868:175855 71.07261:293752 81.06834:329192 86.09541:6767502 87.09918:146255 95.08477:191886 104.10697:35008200 105.10879:669086 107.08487:89972 109.10007:124939 124.99956:1800818 163.01524:161429 173.39603:106986 181.02371:83013 184.07466:32080096 185.07635:720869 258.10977:180580 | 1.057E-06 |

**Differences in metabolites between the Model group and the WJW group**

| Alignment ID | Metabolite name | Rt(min) | Expreiment Mz | Adduct type | Reference m/z | MS/MS spectrum | PPM |
|---|---|---|---|---|---|---|---|
| POS1054 | peridinecarboxalde | 4.523 | 114.09103 | [M+H]+ | 114.09189 | 53.03773:22129 54.03373:19684 55.0177:275908 55.05365:720196 56.04952:14814 58.02807:28138 58.06511:13280 65.03804:9965 67.05389:191692 68.04829:166504 69.03294:17596 69.06868:1196464 70.0643:116677 71.04816:59570 71.08547:74695 72.08047:136179 77.03802:45747 79.05312:1238748 81.05579:32851 84.07957:26762 86.05938:14861 86.0954:96980 91.05576:9702 95.04893:72789 96.08018:613010 97.0646:185251 105.04401:16672 114.09125:4150725 115.09233:15965 | -7.53778E-06 |
| POS788 | o[4.2.0]octa-1,3,5- | 5.024 | 105.06965 | [M+H]+ | 105.0702 | 51.02236:25263 57.03286:8617 58.06416:125667 62.05909:6692 70.70855:5422 77.03802:38850 79.05312:91655 95.04893:82298 103.05421:55881 104.10696:6409 105.04401:25295 105.06945:121863 | -5.2346E-06 |
| POS1831 | Urocanic acid | 1.535 | 139.04982 | [M+H]+ | 139.05019 | 55.0177:9414 66.03385:11141 67.05389:7759 68.04949:6381 68.99723:18717 82.0746:5494 93.04333:35198 94.06358:6205 95.05888:8466 112.03867:5948 121.03835:36538 139.0489:13188 | -2.66091E-06 |
| POS3462 | tyloxy)propan-1-a | 4.786 | 188.19987 | [M+H]+ | 188.2009 | 55.05364:8123 57.06987:499831 58.06511:847825 59.04857:20349 67.88462:6390 69.0699:7033 71.08546:249705 72.08835:5825 76.07561:347996 118.06287:19451 123.48865:5416 146.06096:11333 188.19746:7647 | -5.47287E-06 |
| POS1021 | Uracil | 1.557 | 113.03455 | [M+H]+ | 113.03458 | 67.0539:6326 69.04404:15747 70.0278:42926 96.00742:15749 113.03346:21848 | -2.65406E-07 |
| POS10030 | LysoPC(0:0/16:0) | 6.521 | 496.33655 | [M+H]+ | 496.3403 | 57.06896:6797 58.06607:7574 60.08025:92779 71.07261:9771 86.09541:301558 98.98415:8214 104.10697:91629 124.99956:81893 184.07466:2157294 185.07635:54194 | -7.5553E-06 |
| POS9747 | LysoPC(15:0/0:0) | 6.25 | 482.32437 | [M+H]+ | 482.32407 | 57.06989:8019 59.04858:8869 60.08025:54297 72.65694:7997 82.89674:6261 86.09541:112673 100.42736:5892 104.10696:681901 105.10879:16336 111.15255:5910 124.99955:23582 173.43034:7038 182.06386:7149 184.07466:284068 185.07635:9479 | 6.21988E-07 |
| POS6642 | methyl-dodecylar | 5.336 | 304.29819 | [M]+ | 304.2999 | 50.02972:7158 58.06416:92108 62.08849:6043 91.05389:147827 173.4254:6580 212.23723:15942 | -5.61946E-06 |
| POS3480 | hydro-2,3-dihydro | 5.059 | 189.09024 | [M+H]+ | 189.091 | 55.0177:49571 55.05365:11699 65.03805:8920 67.05389:70001 79.05312:27394 91.0539:61400 95.04893:28404 105.03246:65584 105.06945:42646 107.04917:170598 115.05257:10935 117.06913:39553 119.08612:8633 121.02692:10609 128.06212:15991 130.06477:36301 133.06566:10016 133.09863:16817 143.08574:13797 145.06526:12545 147.07849:7829 161.09245:9120 173.43034:6694 | -4.01923E-06 |
| POS1545 | 2E)-Decenoyl-ACI | 1.07 | 130.08577 | [M+H]+ | 130.0864 | 56.04862:118858 58.06511:7521 67.05389:8363 70.0643:49894 83.05907:18019 84.04315:299249 84.07957:499412 85.02799:12389 86.05938:19031 130.04883:13762 130.08388:25923 | -4.84294E-06 |
| POS3045 | Indoleacetic acid | 4.908 | 176.07011 | [M+H]+ | 176.07001 | 103.05418:12802 112.7732:5731 130.06473:203262 | 5.67956E-07 |
| POS986 | Cytosine | 1.59 | 112.05036 | [M+H]+ | 112.05058 | 55.05365:13088 56.04862:9977 67.02913:10786 69.04403:32435 94.04007:8199 95.02306:57115 112.04886:82686 | -1.9634E-06 |
| POS1541 | Quinoline | 5.061 | 130.06508 | [M+H]+ | 130.06509 | 55.05366:19586 56.04863:54437 57.03287:6719 61.02846:6109 67.05389:17018 69.06992:8063 74.02328:7320 77.03802:33228 77.99809:8488 84.04317:57524 84.07957:71176 87.00325:31408 95.04894:44176 96.00742:5359 102.04613:7030 103.05422:111150 105.04402:9914 109.92458:5445 113.96312:10506 128.04967:13874 130.06477:395782 | -7.68846E-08 |
| POS630 | rolidinecarboxalde | 4.404 | 100.07545 | [M+H]+ | 100.07624 | 53.03773:15572 55.0177:33096 55.05365:79980 56.04862:75630 57.06896:14117 58.02807:34226 58.06512:9289 59.04858:12863 62.80278:6011 69.03294:9240 72.04369:11810 72.08047:11866 79.76434:5267 82.06503:14023 83.01195:6788 84.06467:6030 91.50716:5661 97.81029:5782 98.98415:8797 100.03944:6290 100.07382:172510 | -7.89398E-06 |
| POS101 | 1,3,5-Hexatriene | 0.646 | 81.07 | [M+H]+ | 81.06988 | 53.03773:68020 55.0177:9358 55.05365:6707 55.92267:5853 56.96447:19258 65.03804:8525 66.04538:8879 67.50902:13644 79.05312:86752 80.04813:8423 81.04482:7777 81.06833:89005 | 1.4802E-06 |
| POS10358 | LysoPC(O-18:0/0:0 | 6.057 | 510.38947 | [M+H]+ | 510.39178 | 60.08025:20922 66.76708:5146 70.14877:6543 71.08547:5711 85.028:17314 86.09541:33705 104.10696:321132 105.10879:12888 124.99955:10268 184.06929:22176 297.80222:6396 | -4.52593E-06 |
| POS1539 | Isoquinoline | 5.322 | 130.06502 | [M+H]+ | 130.0654 | 55.05451:10840 56.0486:14816 67.05387:6220 77.03799:5908 84.04313:22135 84.07954:42714 95.0489:12653 103.05418:29486 105.04398:6228 130.06473:96836 | -2.92161E-06 |
| POS1967 | Kojic acid | 4.376 | 143.03334 | [M+H]+ | 143.03329 | 55.01768:35985 55.05363:31541 56.94137:14899 59.04856:6648 62.4123:6113 69.03291:59530 70.06427:12391 71.04813:16095 72.08044:6174 72.93703:11363 97.0276:9883 98.06002:6243 98.09548:5300 143.03423:122999 | 3.49569E-07 |

| Differences in metabolites between the Model group and the WJW group | | | | | | | |
|---|---|---|---|---|---|---|---|
| Alignment ID | Metabolite name | Rt(min) | Expreiment Mz | Adduct type | Reference m/z | MS/MS spectrum | PPM |
| POS6089 | adecadiene-1,18- | 0.723 | 283.26205 | [M+H]+ | 283.263 | 55.05365:51287 57.06895:74067 61.03869:14029 67.05389:17077 69.06867:58545 71.08546:26828 81.06832:30109 83.08508:26890 85.10044:10909 93.0684:11862 95.08475:26486 96.94559:5643 97.0646:9364 97.09952:16086 107.08485:12356 109.10005:7087 111.11479:7947 121.10128:11040 135.11642:11242 149.13:6480 171.68271:6646 | -3.35377E-06 |
| POS6212 | Octanoylcarnitine | 4.73 | 288.21643 | [M+H]+ | 288.21689 | 54.70615:6900 57.06896:15297 60.08025:41909 75.81551:5321 77.89019:6069 85.02799:316477 102.05719:7244 127.10885:8097 144.10197:9062 | -1.59602E-06 |
| POS7255 | Glutamyltryptopha | 4.476 | 334.13843 | [M+H]+ | 334.13971 | 59.45607:5788 84.04316:62247 102.05499:6059 118.06566:8077 130.04884:18961 132.07892:9335 144.07968:23359 146.05721:90437 147.05934:5933 159.09137:26060 183.63564:5349 188.07004:140819 189.07664:6173 | -3.83073E-06 |
| POS9770 | ctapropylene glyc | 5.388 | 483.35306 | [M+H]+ | 483.35278 | 57.03287:73776 59.04858:840745 60.05225:12790 60.13429:6466 64.94802:6318 70.09454:7101 76.96691:6548 83.1958:6334 87.04334:10053 89.05896:20193 99.07938:8483 115.07378:9116 117.09089:109783 175.1301:13269 198.19347:5897 305.05081:6387 325.36108:5759 336.14642:6711 | 5.79287E-07 |
| POS4456 | Tetraglyme | 4.517 | 223.15384 | [M+H]+ | 223.15398 | 57.03286:17644 59.04858:719164 61.02742:8630 73.02676:7417 84.0448:8180 87.04333:15449 89.05894:137329 90.16662:6172 93.0684:12612 103.07442:34124 117.05279:24353 | -6.27369E-07 |
| POS3505 | ihydroquinoline-4 | 4.538 | 190.04877 | [M+H]+ | 190.04961 | 55.05365:20905 67.61636:6215 72.08047:142646 89.0373:16348 99.13448:5946 110.25202:6073 115.83308:6206 116.04757:39031 133.35954:6686 144.04256:8430 144.10197:15632 162.05399:418090 163.05547:10310 173.39601:13183 190.04633:30084 | -4.4199E-06 |
| POS10356 | LysoPC(17:0/0:0) | 5.894 | 510.35648 | [M+H]+ | 510.35538 | 57.03287:86428 59.04859:50801 60.08025:54653 81.0699:7127 86.09541:122606 87.04335:5693 89.05896:8908 95.08477:7326 95.98923:6149 99.07939:13648 104.10697:742077 105.10879:18157 124.99956:30848 133.4489:5693 157.12312:11359 184.07466:368997 185.07635:9702 236.22951:5710 306.14078:5994 | 2.15536E-06 |
| POS1177 | Indole | 4.441 | 118.06494 | [M+H]+ | 118.0653 | 53.03857:8410 55.05365:361485 57.05693:29432 58.06512:13322 59.04858:11113 59.07296:9243 70.06557:6328 72.04501:10182 72.08048:568553 77.03802:6876 84.00181:6311 91.0539:163299 95.04894:23475 105.04401:7866 117.05553:22955 118.0629:214646 | -3.04916E-06 |
| POS6031 | Stearolic acid | 4.355 | 281.24564 | [M+H]+ | 281.24719 | 55.05364:45826 57.06895:26962 59.04857:6734 67.05388:17571 69.0699:49359 71.08546:9974 81.06989:18077 83.08508:33551 91.05389:5999 93.06839:8555 95.08475:26932 97.09951:17440 98.61504:5824 107.08485:10366 109.10004:9719 111.1173:7898 119.08331:8945 121.10127:8328 123.11521:7154 161.13194:5821 | -5.51117E-06 |
| POS1789 | -alpha-Phellandre | 2.777 | 137.13206 | [M+H]+ | 137.13251 | 50.26157:6555 53.03856:10255 55.05365:24128 56.94232:13348 57.06988:10135 67.05389:39521 69.06991:14085 72.93706:12012 79.05312:32040 81.06833:129502 89.90059:5602 90.94762:9906 91.05389:17447 93.0684:7249 95.08476:58768 103.24319:6600 136.06245:6346 | -3.2815E-06 |
| POS5283 | octadienyl ester 4- | 5.89 | 253.21492 | [M+H]+ | 253.2157 | 55.05366:44772 57.06896:14797 64.38515:5835 67.05389:25247 69.06869:39445 79.05313:6614 81.06834:26705 83.0851:16776 93.07034:10381 95.08478:27456 97.06461:7369 97.10159:8873 107.08487:10864 109.10007:10862 112.12533:5510 119.08334:10334 121.09844:7845 133.10193:7679 147.11682:6121 | -3.08038E-06 |
| POS9476 | LysoPC(14:0/0:0) | 5.948 | 468.30829 | [M+H]+ | 468.30847 | 57.03284:142001 57.06893:45390 58.03659:30210 59.04856:38273 60.08022:255437 71.08544:26823 86.09536:548739 87.09914:17012 95.08472:17725 104.10692:2605018 105.10873:68251 124.9995:104966 184.07457:971578 185.07626:30284 348.94217:16614 | -3.84362E-07 |
| POS1748 | 2-Phenylacetamide | 2.199 | 136.07529 | [M+H]+ | 136.0757 | 55.93345:61193 56.94233:8625 65.03805:41428 67.05389:6790 71.92827:16470 72.93707:55150 79.05313:8527 81.0699:20341 89.93906:9812 90.94764:24493 91.0539:664279 94.04008:7175 107.04918:41799 109.06581:10356 118.06566:13408 119.04707:61620 119.80384:6310 136.02159:13051 136.05907:20846 137.04604:7569 | -3.01303E-06 |
| POS7477 | Lauroylcarnitine | 5.14 | 344.27841 | [M+H]+ | 344.27948 | 57.03287:14438 57.06896:18743 59.04858:78071 60.08025:121773 67.05389:10990 69.08101:8178 71.08547:7833 79.05463:9091 81.06833:11027 83.08509:7217 85.028:749086 86.03196:11393 86.61588:6665 88.03895:6369 89.05896:24823 93.06841:16163 95.08477:12929 103.07444:8760 107.08486:11118 109.10006:11058 135.07935:7396 144.10197:12672 163.14494:7803 183.17155:10126 | -3.10794E-06 |
| POS206 | Methacrylamide | 4.801 | 86.05978 | [M+H]+ | 86.06 | 58.06416:79400 69.03293:13792 84.03653:5447 86.05937:25114 | -2.55636E-06 |

| Differences in metabolites between the Model group and the WJW group | | | | | | | |
|---|---|---|---|---|---|---|---|
| Alignment ID | Metabolite name | Rt(min) | Expreiment Mz | Adduct type | Reference m/z | MS/MS spectrum | PPM |
| POS1238 | Isoindoline | 3.962 | 120.08053 | [M+NH4]+ | 120.08077 | 55.05366:9682 56.05764:7025 56.9414:19400 59.60601:5756 61.0387:28501 72.93573:19823 73.08311:14147 77.03802:38079 90.94764:8302 91.0539:63293 93.06841:61439 95.04894:121825 102.04613:6800 103.05422:433237 105.04402:28614 118.0629:7220 120.08031:292394 | -1.99865E-06 |
| POS1440 | -2H-pyran-3-carb | 0.182 | 127.03897 | [M+H]+ | 127.03952 | 50.26004:5651 53.03856:115390 53.93916:7725 55.0177:130637 55.05365:26134 56.04862:10501 56.96447:8503 57.03286:22978 67.05389:9915 70.06556:12345 71.04816:43129 79.05312:8435 80.04813:22549 81.03228:31319 81.06832:17377 82.06502:9874 84.04315:7402 84.95899:8129 99.04338:9133 108.04319:9438 109.02666:25826 127.03809:287876 | -4.32936E-06 |
| POS2016 | cyclohexanecarbo | 1.135 | 144.1013 | [M+H]+ | 144.10181 | 53.03773:16288 55.0177:23838 55.05365:24757 56.04952:14732 58.06416:96575 61.03869:14347 69.03294:9637 70.06429:72063 71.04815:19953 72.08047:12728 81.06832:10240 84.04315:21596 84.07957:63766 98.06005:49924 98.09551:32076 99.04338:17761 102.05497:7220 112.20184:5816 125.72906:6288 126.09013:10892 143.03059:7747 144.10196:150958 | -3.53916E-06 |
| POS1628 | DL-Norleucine | 1.255 | 132.10962 | [M+H]+ | 132.11 | 55.0177:12744 55.05365:12696 56.04952:17870 57.05692:42131 58.06416:46069 61.01003:9893 69.03417:12424 69.06867:466009 72.04369:7878 73.06432:9978 86.0954:2907210 87.09917:12270 90.05456:82747 132.07564:17170 132.10173:22504 | -2.87639E-06 |
| POS5544 | Phenylalanylproline | 4.435 | 263.13757 | [M+H]+ | 263.13898 | 70.06431:91480 84.04317:22235 85.04652:13408 86.09541:49676 87.09918:29051 116.06905:174861 120.08031:205804 121.08413:11874 123.11524:9314 132.10175:5500 133.10522:7109 | -5.35839E-06 |
| POS7887 | exapropylene glyc | 5.061 | 367.26733 | [M+H]+ | 367.26898 | 57.03286:154690 58.18789:5729 59.04858:1746998 60.05225:23401 73.06431:53570 85.02798:7284 87.04333:17183 89.05895:16939 97.06255:5646 99.07938:8648 103.07443:6641 115.07377:10087 117.0528:9869 117.09088:115985 131.07086:19714 167.31822:5942 173.3911:11814 175.13008:6860 226.5341:6140 333.52783:5814 | -4.49262E-06 |
| POS211 | Cyclopentylamine | 2.203 | 86.09637 | [M+H]+ | 86.09643 | 50.39038:12062 56.04863:92328 57.05693:146946 58.06512:20445 67.05389:15821 69.06868:112689 86.05939:66632 86.09541:77074 | -6.96893E-07 |
| POS1475 | L-Baikiain | 1.179 | 128.07036 | [M+H]+ | 128.0708 | 53.03856:21195 54.03372:11950 55.0177:17257 55.05453:94553 56.04952:7843 57.04489:12639 58.06511:13196 62.47697:5464 67.0421:17684 70.06556:9392 71.04944:9132 72.58782:6505 82.06502:127867 86.05938:6760 100.07597:11830 110.05835:9058 127.03809:17037 128.07144:17484 | -3.4356E-06 |
| POS11231 | (20:4(8Z,11Z,14Z,1 | 6.192 | 544.33997 | [M+H]+ | 544.34033 | 60.08023:744682 86.09538:1786256 94.47636:236946 104.10693:15225237 124.99951:360103 126.55663:278578 184.0746:7584514 226.83458:285865 261.237:259828 341.89731:242410 381.40887:324009 531.4198:284034 | -6.61351E-07 |
| POS11420 | yl)-sn-glycero-3- | 6.402 | 550.38617 | [M+NH4]+ | 550.38672 | 60.08024:15426 86.0954:27092 104.10696:144095 105.10878:7529 124.99954:7469 184.07465:57400 | -9.99297E-07 |
| POS6592 | -Aminooctadecan | 5.507 | 302.30365 | [M+H]+ | 302.30539 | 55.05365:26947 56.04862:6381 57.06896:15481 60.04425:231657 67.05389:30758 69.06868:25997 70.0643:9173 71.08547:6605 81.06989:26805 83.08509:14741 88.07444:7951 95.08476:27072 97.10158:11068 109.10005:11294 120.03226:5724 165.78566:5174 168.79677:5089 183.07042:7081 298.44339:6124 | -5.75577E-06 |
| POS6956 | Phytosphingosine | 5.034 | 318.29956 | [M+H]+ | 318.2999 | 55.05365:10813 57.06896:194439 58.06417:28443 69.06992:9206 70.06431:246436 71.08419:70457 72.08048:10266 84.07957:5835 85.10045:16514 86.09541:6291 88.07445:437164 95.08477:6332 102.09042:259378 132.10175:18678 146.11787:10681 150.11266:5960 256.25848:170371 257.26578:9467 318.29404:57728 | -1.06818E-06 |
| POS8198 | (+)-Diaeudesmin | 5.535 | 387.17865 | [M+H]+ | 387.18011 | 59.04858:7208 79.05312:18069 81.03229:7618 95.04893:7858 103.05421:12348 105.06945:1023412 106.07115:41636 119.04706:18835 121.0641:14413 124.34185:5209 141.08885:5042 163.98055:5395 197.54788:5811 281.13101:5592 | -3.77085E-06 |
| POS667 | -5-methyl-2(3H)-f | 0.659 | 101.05946 | [M+H]+ | 101.06026 | 53.03773:8754 55.0177:20315 55.05365:102916 56.04861:7906 57.06988:6898 59.04858:34741 60.04425:6534 72.04368:6808 83.04768:6817 100.07381:13531 | -7.91607E-06 |
| POS1743 | Adenine | 8.871 | 136.06134 | [M+H]+ | 136.0619 | 76.50495:5886 81.0699:10792 91.05389:60378 94.04008:8470 118.0629:7499 119.0359:17263 136.02158:20705 136.05905:64464 137.04602:8767 | -4.11577E-06 |

| Differences in metabolites between the Model group and the WJW group | | | | | | | |
|---|---|---|---|---|---|---|---|
| Alignment ID | Metabolite name | Rt(min) | Expreiment Mz | Adduct type | Reference m/z | MS/MS spectrum | PPM |
| POS10162 | 20:4(8Z,11Z,14Z,1 | 6.116 | 502.29059 | [M+H]+ | 502.2926 | 55.01769:23744 55.05364:63581 57.03286:67470 57.06895:32465 61.71925:6370 62.06013:14088 67.05388:168157 69.06866:79820 71.04815:6325 71.08418:14996 73.02811:11585 75.04311:7844 77.03801:6084 79.05311:98005 81.06831:127336 83.04768:6810 83.08508:26961 85.06335:24850 85.10043:7537 91.05389:118244 93.06839:110439 95.04892:7717 95.08475:122202 97.06459:31760 97.10156:10780 105.06944:84821 107.08484:69844 109.10004:38606 117.06911:40537 119.08331:103327 121.10127:61898 123.08001:13187 123.11521:9795 129.05444:51851 131.08374:35621 133.0986:52981 135.07933:6474 135.11642:19200 137.13217:8902 143.08571:12809 145.09901:19486 147.11679:20481 149.12999:8337 157.09772:19947 159.1172:6413 161.13194:9723 171.11403:13829 175.145:6167 203.17519:26631 210.12161:6896 240.12112:5812 | -4.00165E-06 |
| POS4575 | butyl 9-decenoate | 5.693 | 227.20029 | [M+H]+ | 227.20039 | 55.05365:75761 57.06896:54375 67.05389:21621 69.06992:84161 71.08547:15864 76.01581:5873 79.05312:13981 81.0699:28858 83.08509:31734 93.06841:15564 95.08477:25756 97.06461:14665 97.10159:16206 100.11038:9758 107.08486:19222 109.10006:20843 111.0796:8132 121.1013:13856 123.18568:6006 135.11646:11720 135.83438:5483 138.09952:5890 176.64301:6112 177.47789:7268 | -4.4014E-07 |
| POS9474 | yl-sn-glycero-3-p | 5.96 | 468.3049 | [M+H]+ | 468.3078 | 57.03287:56976 57.06896:50087 58.06512:15779 59.04859:57555 60.08025:282939 67.05389:10296 71.07261:27548 71.08548:30422 73.02812:11311 81.0699:14310 85.10046:11116 86.09541:607746 89.05896:29623 95.08478:18964 97.67747:9594 101.05931:14140 104.10697:3224918 105.10879:76323 109.10007:10339 115.07378:8659 124.99956:154054 144.00543:8513 163.01524:17084 184.07466:1604689 185.07635:49195 285.23489:8885 348.21555:9886 | -6.19251E-06 |
| POS2275 | Guanine | 4.375 | 152.05612 | [M+H]+ | 152.05659 | 56.04953:6057 67.05389:8330 79.05312:6622 79.78424:6180 96.08018:5419 107.04917:9919 109.05113:5482 110.03356:28966 110.06084:6528 121.03835:5855 135.02879:17398 152.0549:30094 153.03816:28975 | -3.09095E-06 |
| POS6299 | Inosine | 4.194 | 291.06769 | [M+Na]+ | 291.07001 | 103.31759:5482 138.38226:5620 159.02675:44285 | -7.97059E-06 |
| POS5556 | -trans-Bergamotc | 5.875 | 263.20016 | [M+H]+ | 263.2002 | 53.03772:7535 55.01768:18061 55.05364:260452 57.03285:7077 57.06894:58783 59.04856:11671 61.48146:5472 67.05387:164706 69.06866:100524 71.04814:35224 71.08417:15772 77.03799:16605 79.0531:220578 81.06988:155633 82.065:17420 83.04767:15082 83.08507:24513 85.02797:7625 85.06503:19485 87.04332:8695 91.05387:214431 92.05666:5810 93.06838:282421 94.07336:8874 95.0489:18751 95.08474:72032 97.06458:6866 97.10155:11060 105.06942:235724 106.07346:13731 107.08483:105249 109.06332:14729 109.10003:25466 117.06909:49148 119.08329:197641 120.08875:12157 121.06406:5999 121.10126:52704 123.1152:9954 129.06702:29755 131.08372:58224 133.09859:121701 135.11641:21689 142.07654:5827 143.08569:20258 145.099:14880 147.07845:21436 147.11676:60528 149.09477:6374 149.12997:16558 157.10193:16064 161.09679:24467 161.13193:28054 163.1449:8970 171.11401:6749 175.11014:15110 179.10367:7485 203.17516:6551 | -1.51976E-07 |
| POS7731 | racosahexaenoic a | 5.942 | 357.2774 | [M+H]+ | 357.27859 | 55.05365:82340 57.06896:8984 62.52579:6246 67.05389:76133 69.06992:33518 79.05312:27449 81.0699:207516 82.07301:8125 83.04932:10956 83.08509:94266 85.06337:25760 91.0539:24546 93.06841:117068 95.08477:229277 96.08827:6720 99.04339:41540 101.0593:51827 105.06945:86914 107.08486:163615 109.10006:140914 115.07378:9366 119.08333:90308 121.1013:142247 123.08004:8885 123.11523:34384 131.08377:22886 133.09863:107850 135.11646:174219 137.09428:11864 145.09904:32009 147.11681:123491 149.13002:90343 150.13635:7744 151.10901:11390 159.11723:36301 161.13197:136473 162.13377:8170 163.11809:15359 163.14494:41743 167.10463:17815 173.13152:22906 175.14503:66743 177.12804:15544 179.10373:8325 181.12314:20871 187.14839:15478 189.166:24644 193.12155:9220 195.13722:18504 201.16266:44090 207.13823:11848 215.17596:30307 221.1546:10218 229.20091:12362 | -3.33073E-06 |
| POS2630 | -Hydroxychromor | 5.279 | 163.03874 | [M+H]+ | 163.039 | 56.07296:6261 57.974:7314 77.03802:18561 79.05312:11146 92.02633:7193 95.04893:39622 105.04401:13257 133.02939:17424 135.04227:13756 163.03757:33028 | -1.59471E-06 |
| POS4227 | Tetradecylamine | 5.134 | 214.25204 | [M+H]+ | 214.25288 | 57.06989:31070 71.08547:7581 81.71648:5671 192.96027:5735 214.25024:9807 | -3.9206E-06 |

**Differences in metabolites between the Model group and the WJW group**

| Alignment ID | Metabolite name | Rt(min) | Expreiment Mz | Adduct type | Reference m/z | MS/MS spectrum | PPM |
|---|---|---|---|---|---|---|---|
| POS3477 | N-Acetylglutamine | 1.584 | 189.0865 | [M+H]+ | 189.08698 | 55.93435:12320 56.04862:10246 60.04426:12610 61.03972:5803 72.93707:6110 83.05907:8824 84.04316:249315 84.07957:88188 101.07021:7509 102.05499:7050 105.53591:5286 126.09014:23861 129.88974:5816 130.04884:153460 173.39601:7986 | -2.53851E-06 |
| POS1335 | Nicotinic acid | 2.61 | 124.03905 | [M+H]+ | 124.03928 | 53.03856:17751 78.03358:12060 80.04813:95435 83.04768:6157 95.04693:8071 96.04377:52175 112.03866:13916 123.05363:35771 124.03866:11491 | -1.85425E-06 |
| POS3580 | ydroxyindoleacetic | 2.976 | 192.06497 | [M+H]+ | 192.06599 | 57.31232:5637 146.05719:13051 | -5.31067E-06 |
| POS7939 | -Tetradecenoylcar | 5.229 | 370.29395 | [M+NH4]+ | 370.29575 | 55.05365:12123 57.03286:31568 57.06989:16170 59.04858:7729 60.08025:297746 67.05389:21597 69.06868:27238 81.06989:15191 83.08509:14885 85.02799:1552989 86.03024:22780 89.05895:7308 93.06841:11323 95.08476:17975 97.09953:10649 103.03848:7755 107.08485:12694 109.10006:25355 121.10129:28425 123.11523:8170 135.11644:19502 144.10197:31085 191.17667:11347 209.18933:10597 | -4.86098E-06 |
| POS12629 | ecapropylene glyc | 5.72 | 599.43445 | [M+H]+ | 599.43646 | 57.03287:98999 59.04858:1006998 60.05225:10498 73.02811:17432 87.04334:26531 89.05895:57361 103.07444:19106 115.07378:13303 117.09089:142591 133.08543:25731 175.1301:24920 | -3.35315E-06 |
| POS5679 | Adenosine | 3.861 | 268.10361 | [M+H]+ | 268.10397 | 55.0177:8560 55.05365:6074 57.03287:16050 84.84476:5419 136.05907:711106 137.04602:243689 180.67375:6017 | -1.34276E-06 |
| POS4408 | ydroxy-L-tryptop | 3.106 | 221.09062 | [M+H]+ | 221.0918 | 55.93435:7421 58.06511:9653 74.00551:5496 79.55973:6350 89.96104:8886 102.58624:6205 116.97397:6505 130.06477:35503 132.04306:12425 157.0766:14875 158.05783:16085 175.08531:10770 | -5.33715E-06 |
| POS2089 | Spermidine | 0.914 | 146.16499 | [M+H]+ | 146.16518 | 56.04953:17737 58.06512:12252 67.0539:7871 72.08048:77083 73.6934:6841 79.67875:5981 84.04482:6788 84.08124:191994 112.11259:10999 | -1.2999E-06 |
| POS1431 | a-4,5-cyclopropyl- | 1.204 | 127.03858 | [M+H]+ | 127.0389 | 53.03773:67161 55.0177:76953 55.05365:16891 55.21807:6225 57.03286:15598 57.06896:6209 67.05389:7619 71.04816:11899 78.9069:5679 80.04814:13044 81.03229:20049 81.04325:8220 81.06989:6367 82.06503:15826 108.04319:6029 109.02667:13298 111.38697:4780 127.03809:65632 | -2.51891E-06 |
| POS14450 | | 4.642 | 721.41571 | [M+2H]2+ | 721.41571 | 69.03294:64032 73.02811:159873 73.06432:77520 83.45062:47888 87.04334:536056 89.05896:2457454 90.0619:93104 95.04694:86163 98.23338:47352 111.04189:78648 111.44507:43274 113.05927:60756 121.66422:45778 131.07088:82096 133.08543:851508 137.0598:58032 177.11285:88833 200.87497:48388 327.85837:47698 656.01245:49795 | 0 |
| POS12449 | 33,36-Dodecaoxa | 4.547 | 591.35876 | [M+H]+ | 591.35858 | 73.06432:125073 87.04334:743459 89.05896:5522730 90.0619:104323 117.09089:82210 130.08708:36141 131.06766:189581 133.08543:2375642 134.08684:68181 175.09526:47103 177.11285:303644 | 3.04384E-07 |
| POS11255 | asterone 22-O-su | 4.584 | 545.31409 | [M+2H]2+ | 545.31421 | 67.05389:29798 69.03294:42084 73.02811:194322 73.06432:63047 81.03229:54889 83.04932:51834 87.04334:399634 89.05896:2005170 90.0619:67636 91.04643:47910 95.04893:140429 99.04339:71219 107.07058:39913 108.77773:25939 109.02668:26574 109.06336:35887 111.04189:185371 113.05927:53829 131.07088:53610 133.08543:599344 134.08684:38149 137.0598:75205 155.06668:55882 177.11285:59448 274.02045:23581 311.28952:23464 | -2.20057E-07 |
| POS11312 | | 4.546 | 547.32977 | [M+H]+ | 547.32977 | 73.06432:77080 87.04334:310689 89.05896:3034282 90.0619:56261 91.07443:15872 107.06821:26790 117.09089:29974 131.07088:55766 133.08543:1045644 134.08684:29346 175.09526:16421 177.11285:105917 | 0 |
| POS8525 | ol 3-beta-D-gluco | 5.159 | 411.27438 | [M+2H]2+ | 411.27451 | 57.03287:106701 59.04859:2423900 60.05226:58860 65.04819:49767 69.06868:24429 73.02812:203056 73.06432:45534 80.0543:28441 81.0699:10650 83.04932:11338 85.06337:50911 87.04335:255244 87.06254:93632 87.07999:40629 89.05896:1724694 90.06191:50889 94.06947:59301 101.05931:186742 102.06606:20714 103.07444:447298 104.0796:11406 107.07059:15977 109.0756:40860 115.07378:18653 116.08248:24210 117.05281:16563 117.0909:23711 129.08911:29662 131.06766:24305 133.08545:471122 134.08684:21539 143.1078:10447 145.08403:14879 147.10149:192488 161.11441:12769 177.11285:35988 191.1256:28252 | -3.16091E-07 |

| Differences in metabolites between the Model group and the WJW group | | | | | | | |
|---|---|---|---|---|---|---|---|
| Alignment ID | Metabolite name | Rt(min) | Expreiment Mz | Adduct type | Reference m/z | MS/MS spectrum | PPM |
| POS10377 | CHEBI:68981 | 4.652 | 511.30283 | [M+H]+ | 511.30289 | 69.03294:54910 73.02811:290002 73.06432:126431 80.05429:70564 81.03229:42121 87.04334:557306 89.05896:2868972 90.0619:147150 91.04643:91641 92.64947:36836 95.04893:132782 97.53043:33536 99.04339:71888 102.06606:61822 104.827:36803 111.04441:53212 113.05927:114186 131.06766:70072 133.08543:985809 134.08684:71800 137.05637:55345 177.11285:107996 288.22748:36591 479.31699:35690 | -1.17347E-07 |
| POS8071 | Ile Val Phe | 5.563 | 378.23935 | [M+H]+ | 378.2392 | 53.43733:6285 55.05365:90186 56.04863:21368 57.06896:23453 67.04092:11340 67.05389:134907 69.06868:63033 70.06431:61804 75.76308:6507 79.05312:83881 80.04814:33789 81.06834:101400 82.06343:365326 83.06721:12639 83.08509:32106 84.07957:8502 91.0539:54314 93.06841:110549 94.06359:41593 95.04893:5641 95.08477:93567 96.08018:77879 97.10159:9417 98.98415:8204 105.06945:31175 107.08486:55798 108.07938:16346 109.10007:42081 110.09557:10276 119.08333:18474 119.29852:6083 121.1013:43555 123.11523:9738 124.10991:6794 133.09863:18013 135.11646:16218 138.67992:6402 147.11681:12248 149.13002:16616 161.13197:8614 166.16684:5424 196.27794:6256 208.3924:6424 262.25568:184124 263.25272:19304 | 3.96574E-07 |
| POS10376 | His Gln Val Lys | 4.662 | 511.29846 | [M+H]+ | 511.29861 | 69.03291:46719 73.02808:224037 73.06429:52427 80.05272:60468 87.0433:471413 88.04601:45781 89.05892:2417979 90.06187:116949 91.04639:54072 95.0489:109640 99.04335:56884 111.04436:90199 113.05922:59512 129.05441:36895 131.07082:54101 133.08539:702772 173.39104:87009 177.10771:62434 323.89127:41408 | -2.93371E-07 |
| NEG3390 | dihydrocanadenso | 0.969 | 211.09708 | [M-H2O-H]- | 211.09711 | 79.05367:6003 93.03925:886889 95.03763:862836 131.2403:23563 154.2491:6626 | -1.42115E-07 |
| POS9633 | yonarasterol I | 5.152 | 477.31287 | [M+2H]2+ | 477.3129 | 57.03287:56982 58.04137:15518 59.04859:1226660 60.05226:30691 65.04819:23821 69.06868:19204 73.02812:145377 73.06432:36640 80.0543:48872 83.0477:10988 85.06337:37427 87.04335:209820 87.06079:61516 87.07999:30585 88.04605:9002 89.05896:1451464 90.06191:61655 94.06947:30623 101.05931:131752 102.06606:25430 103.03849:14083 103.07444:283960 104.07732:12882 105.09028:8596 107.07059:14672 109.0756:35061 115.07378:10308 116.08248:15275 117.0909:23098 124.08022:8919 127.07501:8106 129.08911:26603 131.06766:22858 133.08545:470848 134.08684:22171 140.09358:8084 145.08403:12423 147.10149:127214 161.11441:8142 177.1078:47142 191.1256:22526 | -6.28519E-08 |
| POS8061 | Desoximetasone | 4.592 | 377.21191 | [M+H]+ | 377.21201 | 74.06023:8026 89.05896:13965 91.90891:6126 377.22052:9604 | -2.65103E-07 |
| POS4996 | alpha-Humulene | 4.604 | 243.15111 | [M+2H]2+ | 243.15096 | 73.02811:8617 104.56932:5620 136.52379:5669 173.43034:6491 207.27916:5990 227.04758:5414 | 6.16901E-07 |
| POS2774 | thyl-2-prenylthiop | 3.379 | 167.08884 | [M+H]+ | 167.08881 | 77.03802:33641 79.05312:119463 80.05737:18155 91.0539:73080 92.05668:7771 93.06841:196037 94.07143:32746 95.04893:37463 103.05421:809050 104.05679:150822 105.04401:8951 107.04917:92188 108.05284:10313 118.0629:8015 120.06335:61653 120.0803:3048589 121.08412:632523 122.0861:7363 131.04832:35670 | 1.79545E-07 |
| POS4916 | Securinine | 8.805 | 240.09859 | [M+H-H2O]+ | 240.09869 | 103.37626:5837 116.44865:5990 118.20353:5371 124.99955:6414 173.43526:9234 196.63867:5118 | -4.16495E-07 |
| POS7122 | Boeravinone F | 0.879 | 327.04965 | [M+Na]+ | 327.04971 | 86.16064:5992 102.22125:5057 191.07452:111349 232.32516:5391 | -1.83458E-07 |
| POS8276 | Xanthoangelol I | 4.483 | 393.20648 | [M+Na]+ | 393.20639 | 80.38605:5492 179.43362:6023 183.31004:6171 289.0278:6119 | 2.28887E-07 |
| POS9123 | etyl-leu-leu-tyr-a | 4.795 | 449.27011 | [M+H]+ | 449.26999 | 79.14378:5756 87.04335:5503 89.05896:16159 173.38622:10757 326.5238:6411 343.86096:5698 363.62698:6417 | 2.671E-07 |
| POS12359 | Leu Leu Lys Gln Gl | 5.427 | 586.39203 | [2M+H]2+ | 586.39221 | 57.03286:37116 59.04858:574104 65.04817:18765 73.02811:60325 73.06431:19873 85.06504:20511 87.04333:188581 87.06252:27189 87.07997:23055 89.05894:527083 101.05929:118601 103.07442:134199 115.07642:16951 117.09087:15496 129.0891:22851 133.08542:178858 145.08777:13629 147.10147:63098 177.11282:15666 191.12556:18190 192.67273:16708 234.40762:17853 | -3.06962E-07 |
| POS12917 | Austrobuxusin I | 4.569 | 613.33508 | [2M+H]+ | 613.33502 | 50.28071:22135 76.60421:18230 279.35773:16421 408.97412:18993 449.91245:18538 | 9.78258E-08 |
| POS9460 | acetildenafil | 4.633 | 467.27637 | [M+H]+ | 467.2764 | 73.02811:107851 73.06432:42993 75.91343:15367 80.05275:28522 81.03229:26734 87.04334:210130 89.05895:984995 90.0619:60965 91.04643:42961 95.04694:39370 99.04339:29260 102.06827:17648 111.04189:42770 113.05927:26667 131.07086:17292 131.7048:16921 133.08543:356423 134.08684:39526 137.0598:27111 155.07083:15947 177.11284:33955 391.74057:15221 | -6.42018E-08 |

| Differences in metabolites between the Model group and the WJW group | | | | | | | |
|---|---|---|---|---|---|---|---|
| Alignment ID | Metabolite name | Rt(min) | Expreiment Mz | Adduct type | Reference m/z | MS/MS spectrum | PPM |
| POS8166 | sartorymensin | 5.686 | 385.16568 | [M+NH4]+ | 385.1658 | 55.01768:108421 57.06894:137002 83.01192:8474 85.06333:50239 111.49811:5934 155.18697:6350 204.95941:6868 | -3.11554E-07 |
| POS12534 | | 4.934 | 595.3631 | [M+Na]+ | 595.36292 | 59.82801:13607 62.53004:12277 100.68073:11281 135.33597:12776 148.35861:11993 420.52808:15110 569.88092:12102 | 3.02337E-07 |
| POS4287 | lpha-D-galactopy | 1.025 | 217.06792 | [2M+H]+ | 217.06799 | 52.17733:5754 62.98166:16407 70.06431:8139 75.71778:6086 80.94776:1450871 85.028:29720 90.97558:243299 134.10017:5273 160.03503:14015 | -3.2248E-07 |
| POS8731 | 3,4,6,7,8,8a-hexahy | 5.403 | 425.29007 | [M+2H]2+ | 425.29001 | 57.03287:84902 59.04859:1929612 59.05931:49421 60.05226:47566 65.04819:13475 69.06868:16412 73.02812:80386 73.06432:29815 81.0699:7680 83.0851:8116 85.06337:30340 87.04335:141455 87.06254:30547 87.07999:32010 89.05896:676639 90.06191:20312 94.06947:46752 99.08151:8114 101.05931:143792 103.07444:347040 104.07732:9876 115.07378:17640 116.08248:11319 117.05281:13965 117.0909:28290 129.08911:17986 131.06766:13510 133.08545:166479 134.08684:10288 143.1078:6873 147.10149:126930 148.1028:7550 161.11441:18634 191.1256:15710 | 1.4108E-07 |
| POS9097 | Mutamicin 5 | 5.214 | 447.29263 | [M+H]+ | 447.29251 | 52.18702:7097 59.04856:8204 75.88783:5461 89.05891:10473 130.28481:6147 149.63971:5515 258.80569:5579 317.15057:5762 330.39728:5447 339.19794:5301 | 2.68281E-07 |
| POS13123 | nethylviny l)-17-c | 5.318 | 623.40717 | [M+2H]2+ | 623.40704 | 57.03287:38020 59.04859:691674 73.02812:112119 73.06432:31260 87.04335:166710 87.06254:35440 87.07999:27390 89.05896:1062317 90.06191:33289 94.06947:28494 101.05931:97398 103.07444:200628 107.06821:23051 109.0756:29367 126.93054:21131 130.08708:26378 131.06766:38358 133.08545:382463 147.10149:107107 147.47386:22019 177.1078:49877 | 2.08531E-07 |
| POS8391 | Lintetralin | 5.686 | 401.19635 | M+CH3OH+H] | 401.1962 | 57.06894:233706 58.07269:7607 61.01:15858 81.03226:61737 83.01192:78113 85.02796:10233 85.06333:169177 111.04436:9805 115.03928:45062 125.05955:8504 127.07495:13433 133.06891:58049 135.04222:15256 137.05975:9002 139.07352:21032 147.04396:13973 157.08499:7303 167.10457:243647 168.11116:14351 171.09958:19267 213.09639:6366 221.1192:22467 231.10547:21423 | 3.73882E-07 |
| POS9694 | PC(15:1(9Z)/0:0) | 8.808 | 480.30655 | [2M+H]+ | 480.30661 | 54.75832:6275 57.06896:7381 62.05909:38228 63.97328:6278 69.06992:10406 83.08509:7427 89.78172:6752 100.4252:5806 104.10696:12415 112.92513:6587 153.58037:5681 155.00865:5704 173.44016:13028 174.6582:5647 204.03627:6123 410.89926:6566 | -1.2492E-07 |
| POS10797 | Hemiasterlin | 5.6 | 527.35992 | [M+2H]2+ | 527.35999 | 57.03287:145722 59.04859:2215140 59.05931:56826 60.05226:60017 69.06992:30441 73.02812:165016 73.06432:77054 85.06337:64190 87.04335:285695 87.06079:53136 87.07999:88955 89.05896:1224127 90.06191:73138 94.06947:39396 99.07939:22454 101.05931:325858 101.09641:21453 103.07444:665743 104.10697:119447 105.10879:49069 115.07378:60897 116.08248:44570 117.0909:69733 129.08911:65918 131.06766:44171 131.10634:36730 133.08545:425443 134.08684:38044 143.10413:30270 145.08403:24073 147.10149:350106 161.1188:60345 173.11685:32836 177.1078:49793 184.06931:55314 185.07635:21746 186.07535:28736 191.1256:59552 | -1.32737E-07 |
| POS10704 | PD173074 | 5.353 | 524.33429 | [M+2H]2+ | 524.33429 | 59.04859:344152 60.08025:793941 71.07261:123900 73.03751:126679 86.09541:1677589 89.05896:278884 104.10697:10252773 105.1111:233840 124.99956:361402 152.55547:117446 184.07466:3732591 248.77914:103774 | 0 |
| POS14421 | G(16:1(9Z)/16:1(9Z | 6.595 | 719.48578 | [M+2H]2+ | 719.48572 | 59.63764:5914 69.55946:7557 153.83411:6111 | 8.33929E-08 |
| POS11476 | Leueantine C | 8.805 | 552.32867 | [M+2H]2+ | 552.32867 | 60.08025:21437 86.0954:54921 104.10696:312401 184.07465:96514 | 0 |
| POS9671 | bipinnatone A | 4.727 | 479.27853 | [M+H]+ | 479.2785 | 69.59312:5999 248.1651:5493 | 6.25941E-08 |
| POS10703 | Pyrichalasin H | 8.806 | 524.30072 | [M+2H]2+ | 524.3006 | 60.08022:679045 80.43092:138712 86.09536:1490409 104.10692:7351334 124.99949:304914 184.07457:2358259 293.82486:116712 | 2.28876E-07 |
| POS1651 | Butoxyacetic acid | 4.956 | 133.08571 | [M+H]+ | 133.0858 | 58.06511:9057 69.06867:30295 86.09539:147379 87.09917:10505 89.00121:6080 132.52338:5562 | -6.76255E-07 |
| POS9695 | ysoPE(18:1(9Z)/0:( | 5.375 | 480.30927 | M+CH3OH+H]2 | 480.3093 | 57.03287:22287 59.04859:253173 60.05226:7475 67.015:16773 73.02812:27236 73.06432:11574 78.30522:6307 81.03072:10941 85.06337:11317 87.04335:33369 87.06254:6825 87.07999:6875 89.05896:199777 90.06191:11231 101.05931:19575 103.07445:52426 117.05281:24502 117.0909:5638 131.07088:12578 133.08545:59241 136.66437:5460 147.10149:20882 347.26852:6725 | -6.24598E-08 |

| Differences in metabolites between the Model group and the WJW group | | | | | | | |
|---|---|---|---|---|---|---|---|
| Alignment ID | Metabolite name | Rt(min) | Expreiment Mz | Adduct type | Reference m/z | MS/MS spectrum | PPM |
| POS10699 | Calcimycin | 5.474 | 524.27399 | [M+H]+ | 524.27399 | 59.04856:33682 60.08023:26828 62.05907:68444 67.05387:32624 69.06989:11172 79.05461:18944 81.06988:20294 86.09538:48259 87.04332:10912 89.05893:38101 91.05387:18221 93.06838:15104 95.08474:12806 104.10693:269808 104.94925:11291 105.06942:14786 184.0746:151074 278.50723:12180 421.39975:12577 | 0 |
| POS14554 | Argentinic acid G | 5.168 | 733.48761 | [M+H]+ | 733.48767 | 57.03379:50785 59.04859:896524 73.06432:52556 87.04335:127946 89.05896:811414 101.05931:153641 103.07444:313097 117.05281:38620 133.08545:230489 147.10149:106157 302.83173:31899 437.43027:38567 712.33484:42054 | -8.1801E-08 |
| POS10326 | Stellettin I | 5.493 | 509.32632 | [M+2H]2+ | 509.32639 | 57.03287:24173 59.04859:232241 67.015:16511 73.02812:19835 81.03072:14771 83.22678:5555 87.04335:25750 87.07999:8051 89.05896:150122 90.06191:7309 94.06555:6141 101.05931:30496 103.07444:67063 104.10697:9472 115.07643:5774 117.05281:6304 117.0909:6914 133.08545:34909 147.10149:21165 158.09198:7607 165.10016:7149 211.56157:5429 | -1.37436E-07 |
| POS9623 | hyl-but-2-enyl)-d | 4.476 | 477.22696 | [M+H]+ | 477.22711 | 80.10661:5961 87.04333:27250 89.05895:367847 90.0619:23458 112.07433:24636 119.08611:15831 133.08543:138518 134.08682:15450 177.11284:21139 178.11942:7388 | -3.14316E-07 |
| POS11020 | PC(16:1(9Z)/2:0) | 5.39 | 536.33325 | [M+H]+ | 536.33313 | 59.04859:185890 60.08025:33364 73.02812:25239 86.09541:77299 87.04335:36444 89.05896:239720 90.06191:16693 101.05931:26606 103.07445:42881 104.10697:422972 124.99956:16040 133.08545:71047 147.10149:19161 184.07466:172538 290.39401:12274 | 2.23742E-07 |
| POS8677 | Formacidine | 5.227 | 422.26428 | [M+2H]2+ | 422.26428 | 57.03286:10914 59.04858:108569 67.01499:17543 81.03072:6926 87.04334:7213 89.05895:80268 101.0593:8728 103.07443:22231 133.08543:26469 147.09764:8730 165.10014:6089 180.10658:5913 252.63242:5638 293.42517:5984 | 0 |
| POS3472 | 4-Chlorobiphenyl | 0.753 | 189.04665 | M+CH3OH+H] | 189.04649 | 59.59612:5875 68.98123:50815 78.67258:6030 83.00869:42062 86.99104:76636 100.03513:13540 101.01785:244130 102.01955:9461 105.0024:100551 118.0436:34416 119.02752:344739 120.02943:15424 123.0126:13613 129.01353:31462 131.08376:5520 137.03912:139074 138.04027:7743 144.02397:7307 147.02486:159008 161.05292:15694 165.03181:23271 179.06253:7655 | 8.46353E-07 |
| POS10094 | Cucurbitacin S | 4.964 | 499.30594 | M+CH3OH+H] | 499.30579 | 57.03287:19243 59.04859:263512 60.08025:13730 73.02812:14774 73.06432:10015 85.06337:9587 86.09541:33442 87.04335:970332 87.09918:10552 88.04783:14431 89.05896:214713 101.05931:219429 103.03849:77055 103.07444:60662 104.10697:123746 105.10879:47566 106.11105:13063 115.07643:10213 117.05281:72333 131.06766:30221 133.08545:61663 147.10149:25608 184.07466:32565 185.07635:14610 186.07535:16191 | 3.00417E-07 |
| POS10120 | Aconine | 8.901 | 500.28616 | [M+H]+ | 500.2861 | 55.05453:11157 58.30054:10092 81.0699:10628 86.09541:10895 95.08477:10350 104.10696:42753 105.1111:21941 107.08486:12771 119.08333:15483 126.02023:87930 127.02272:11667 128.01544:40502 159.11723:14402 173.43034:13153 209.13087:13199 227.14313:10869 | 1.19931E-07 |
| POS9632 | yonarasterol I | 4.5 | 477.31284 | [M+H]+ | 477.3129 | 69.93977:14540 87.04334:26210 89.05896:391634 90.0619:30627 103.72703:8538 112.07434:12685 133.08543:137001 134.08684:29949 177.11285:12063 419.51126:10238 | -1.25704E-07 |
| POS8640 | ethoxy-3-(3-meth | 4.839 | 419.22461 | [M+Na]+ | 419.22476 | 58.04042:12495 59.04859:189004 65.04932:9874 73.02812:51785 73.06432:10804 80.0543:7267 85.06337:8721 87.04335:51168 87.06254:10942 89.05896:257374 90.06375:7579 101.05931:18330 102.06606:13027 103.0385:19246 103.07445:23508 109.0756:6560 133.08545:66767 147.10149:7449 | -3.57803E-07 |
| POS10619 | quaesitol | 5.068 | 521.32611 | [M+H]+ | 521.32611 | 59.04858:22204 60.08025:63739 86.09541:93852 87.04334:8058 89.05896:17883 104.10696:380594 108.16389:6635 124.99955:14995 173.39111:8455 182.94283:6709 184.07466:79071 239.22867:6583 282.12573:5632 | 0 |
| NEG8080 | o-7,8-dihydropyra | 1.054 | 387.18332 | [M-H]- | 387.1832 | 65.69628:5658 68.01559:5751 93.03928:352337 95.03767:298038 97.03749:33426 124.15681:11693 151.06717:22659 153.0676:49273 155.06657:11867 358.09082:5782 | 3.09931E-07 |
| POS9843 | 3,4,6,7,8,8a-hexahy | 4.43 | 487.26398 | [M+H-H2O]+ | 487.26401 | 64.9233:6323 77.04674:5750 87.04335:9197 89.05896:380439 107.06821:6794 111.12991:6008 118.06566:12199 133.08545:173386 144.0797:15248 146.061:74743 151.09705:6603 159.09138:11369 173.42545:8962 177.11285:29188 188.07004:307841 189.07106:13994 205.09822:63379 283.17694:10805 | -6.15683E-08 |
| POS9197 | 4-methyl-9,11-sec | 4.583 | 453.33994 | [M+H]+ | 453.34 | 61.49389:5160 96.08015:43369 100.1125:10595 111.08459:5769 113.10571:19788 114.09121:103674 173.39595:7814 209.16328:26759 228.16107:6962 | -1.32351E-07 |

| Differences in metabolites between the Model group and the WJW group | | | | | | | |
|---|---|---|---|---|---|---|---|
| Alignment ID | Metabolite name | Rt(min) | Expreiment Mz | Adduct type | Reference m/z | MS/MS spectrum | PPM |
| POS8645 | Denticulatin B | 5.002 | 419.27682 | [M+2H]2+ | 419.27682 | 59.04858:171300 73.02811:40529 73.06432:7607 80.0543:12196 87.04334:30343 87.06253:8494 89.05896:235891 90.06191:9288 101.0593:8606 102.06606:6905 103.07444:23919 111.18527:6119 133.08543:44807 137.5574:5651 147.10149:8191 177.11285:9764 188.64743:7025 | 0 |
| POS8140 | Lehualide D | 4.729 | 383.2251 | [M+H]+ | 383.22501 | 57.03287:10409 59.04859:284434 69.79429:8099 70.09203:5685 73.02812:18017 77.05836:5557 83.1274:6299 84.20721:5229 87.04335:481519 89.05896:167555 96.48599:5631 101.05931:64745 103.03849:237185 103.07444:42981 107.06821:7106 117.05281:78851 131.07088:12359 133.08545:40764 147.06319:6251 147.10149:13324 186.17351:5380 | 2.34849E-07 |
| POS9785 | malyngamide I | 6.112 | 484.28232 | [M+H-H2O]+ | 484.28238 | 59.04856:15166 62.05907:9682 71.34617:6384 104.10693:11735 184.07458:11252 190.59322:5859 | -1.23895E-07 |
| POS9100 | nethyl-7,11-dioxc | 5.643 | 447.30978 | [M+H]+ | 447.30981 | 57.06896:13752 59.04858:36661 89.05896:16814 97.06461:16142 101.0593:8015 103.07444:7241 121.0641:6520 159.11293:6657 | -6.70676E-08 |
| POS12414 | Nephilatoxin 1 | 5.693 | 589.38202 | [M+2H]2+ | 589.38202 | 57.03287:29722 57.52443:16027 59.04859:194931 65.56047:14424 67.015:16832 70.43989:14676 73.02812:27593 73.06432:18286 87.04335:26774 89.05896:153815 101.05931:29506 103.07444:60804 105.5429:13858 133.08545:52561 147.10149:17220 309.90234:13809 412.1004:13169 | 0 |
| POS7203 | a,4,5,8,9,12,13,15a | 5.222 | 331.18921 | [2M+H]+ | 331.18909 | 53.34515:5668 66.18044:7608 67.05388:8402 81.06989:11972 88.69724:5445 90.57806:4990 95.08475:11984 97.06458:6005 105.06943:6124 107.08484:6942 121.06407:28359 133.0986:6886 147.07846:6495 177.55913:6473 | 3.62331E-07 |
| POS10191 | droxy-6-methylhe | 6.221 | 503.29526 | [M+H]+ | 503.29541 | 59.04858:9200 65.70778:5439 86.0954:8650 89.05895:12061 104.10696:22086 484.58868:5896 | -2.98036E-07 |
| POS8041 | roxy-6-hydroxyme | 5.259 | 376.173 | [M+NH4]+ | 376.17303 | 55.0177:9572 55.05365:15226 57.03287:38159 59.04858:26096 61.02743:30522 69.03294:335076 71.04816:16089 73.02812:16384 79.05312:17888 81.03229:46270 83.0477:50525 85.028:47738 87.04334:12683 89.05896:9685 91.0539:660279 92.05669:16433 97.0297:10858 99.0434:14089 103.03849:8446 105.03246:62733 107.04917:113719 111.0419:53141 127.03809:14156 129.05447:72112 147.06317:130369 | -7.97505E-08 |
| POS12537 | e-(3beta,5beta,11 | 5.902 | 595.38391 | [M+2H]2+ | 595.38397 | 57.03287:38326 59.04859:187299 73.02812:62898 85.06337:25280 87.04509:37664 87.06254:22316 89.05896:185028 101.05931:43827 103.07444:31021 133.08545:49123 | -1.00775E-07 |
| POS10431 | hylhexadecahydro | 5.798 | 514.2829 | [M+H]+ | 514.28302 | 59.04857:35530 85.06335:16559 89.05894:28911 93.07031:6606 95.08475:16434 109.10004:9500 121.10126:9594 126.0202:184390 133.0854:11048 157.10194:9082 159.1172:8808 209.13083:7331 | -2.33335E-07 |
| POS7719 | Sterebin M | 4.85 | 356.27875 | [M+H]+ | 356.2789 | 58.06511:235047 59.04858:34692 60.32804:6778 73.06432:7429 81.06989:22804 82.43508:5463 84.07957:11302 89.05895:32250 104.79474:5929 108.885:6196 123.11523:24179 133.08543:7052 140.14343:9963 280.99945:5498 341.80237:6580 | -4.21018E-07 |
| POS9736 | nethyl-9-oxo-1,2, | 8.887 | 482.2749 | [M+H-H2O]+ | 482.27484 | 53.99451:6296 71.08418:5443 95.08477:8823 95.8601:5344 104.10696:30712 107.08486:6203 126.02023:43718 127.01965:6483 128.01855:16014 130.86165:5197 412.2981:5842 | 1.2441E-07 |
| POS9810 | Communesin H | 4.826 | 485.29111 | [M+H]+ | 485.29099 | 57.03287:9114 59.04859:208773 76.26552:5650 85.06506:6110 87.04335:26542 89.05897:78871 101.05931:16898 103.0385:10071 103.07445:43505 133.08545:10921 147.1015:9228 161.05295:5750 353.60825:5860 365.49567:5915 372.1275:5635 | 2.47274E-07 |
| POS8830 | Kuguacin E | 6.151 | 431.31509 | [M+2H]2+ | 431.31509 | 59.04858:9678 95.08477:12758 97.06461:61890 97.10159:10056 107.08486:11493 109.06336:6145 109.10006:12249 112.9767:5473 121.0641:6371 121.1013:7481 133.10193:8005 135.11646:7058 147.11681:6669 171.11887:5707 177.12804:19253 357.97528:5319 | 0 |
| POS8955 | Anisodorin 3 | 5.696 | 439.3053 | [M+2H]2+ | 439.3053 | 57.03287:46780 59.04859:698843 59.05931:17682 60.05226:21029 73.02812:27360 73.06432:29850 85.06337:9506 87.04335:41472 87.07999:7958 89.05896:167857 90.06191:6728 94.06947:8708 99.07939:6574 101.05931:62274 103.07444:111550 104.07732:7553 115.07378:17486 117.05281:7619 117.0909:20451 129.08911:6502 133.08545:32517 147.10149:33827 161.1188:7787 374.9805:6956 393.72964:6196 | 0 |
| POS15004 | PC(16:0/18:1(9Z)) | 6.475 | 782.56622 | [M+H]+ | 782.56616 | 53.69582:11689 67.33069:12777 67.96278:10387 86.09541:15276 184.07466:34009 184.36464:11386 293.25256:10403 351.13647:10588 | 7.66708E-08 |
| POS10255 | Talaroconvolutin E | 5.997 | 506.32382 | [M+H]+ | 506.32379 | 51.79336:6660 53.41721:6159 59.04859:22317 60.08025:12511 66.38443:5356 86.09541:31301 89.05896:18294 104.10697:144927 184.07466:37367 185.39581:4931 201.78914:5385 | 5.92506E-08 |

| Differences in metabolites between the Model group and the WJW group | | | | | | | |
|---|---|---|---|---|---|---|---|
| Alignment ID | Metabolite name | Rt(min) | Expreiment Mz | Adduct type | Reference m/z | MS/MS spectrum | PPM |
| POS1562 | lichloro-1,1-ethan | 9.248 | 130.96594 | [M+H-H2O]+ | 130.966 | 54.03373:7588 55.93435:6275 56.04952:26633 57.06988:6346 61.02845:6356 67.05389:23205 67.93391:13180 70.06556:7088 71.92826:29503 72.93706:41262 74.99706:7067 77.49896:7387 77.99808:64843 78.9988:8127 84.04315:36824 84.07957:76239 85.0061:22638 85.08359:6166 86.09711:6852 86.50518:17245 87.00323:285725 87.50385:31305 88.00347:191576 89.50647:9359 89.93904:12920 90.94762:9840 91.05389:5831 94.01266:8758 96.00942:20700 96.51448:7032 97.00917:70070 98.51207:53196 99.51075:59623 106.37916:6335 107.94919:7916 113.96311:105942 115.96169:38962 116.97124:12352 126.97046:6999 129.99471:7179 131.97466:30121 | -4.58134E-07 |
| POS10527 | Thr Thr Gly Leu Ile | 6.89 | 518.31836 | [M+H]+ | 518.31842 | 59.27237:6310 66.88557:6165 73.49677:5366 83.51614:5136 86.0954:10455 130.33278:5614 146.98276:20141 324.70667:6624 386.10718:5660 | -1.15759E-07 |
| POS12327 | kirkinine D | 4.852 | 585.27051 | [M+H]+ | 585.27039 | 55.4905:5598 89.05896:11618 106.74535:6542 133.08545:8463 179.12949:6616 299.14221:7438 | 2.05033E-07 |
| POS4670 | Norpterphyllin III | 5.997 | 231.10387 | [M+H]+ | 231.104 | 53.69413:5942 57.06989:12373 74.0616:6065 108.67789:5870 167.10463:11847 179.73859:5568 | -5.62517E-07 |
| POS4447 | CHEMBL4283703 | 2.625 | 223.06311 | [M+H]+ | 223.0632 | 58.06511:13469 59.04858:7560 61.03868:29019 66.64188:6661 73.02811:9639 73.04553:12324 80.59386:5975 91.05576:17270 93.06839:6086 209.00749:6987 225.04041:63944 | -4.03473E-07 |
| POS4449 | CHEMBL4283703 | 0.702 | 223.06323 | M+CH3OH+H] | 223.0632 | 58.02428:6139 58.06417:21920 59.04859:10298 73.02812:10576 73.04555:18931 76.81198:5763 79.05464:8297 90.48373:5205 91.05577:26695 93.06841:11778 119.04707:16973 155.97054:7677 192.70723:5645 209.00754:13773 225.04045:104121 226.04433:7698 | 1.34491E-07 |
| POS304 | Butyric acid | 4.952 | 89.05968 | [M+H]+ | 89.05968 | 61.02845:46841 68.67587:6086 72.93705:39696 87.00497:16898 90.94762:6676 | 0 |
| POS244 | Butanal, 2-oxo- | 2.532 | 87.04405 | [M+H]+ | 87.04405 | 69.03294:23471 69.06869:12170 86.05939:124827 87.04335:8207 87.06254:8704 | 0 |
| POS5757 | Doxylamine | 5.121 | 271.18137 | [M+H]+ | 271.1814 | 59.04859:7989 64.42622:5475 69.06868:5858 75.68808:5550 116.85452:5675 | -1.10627E-07 |
| POS10170 | Ala Leu Leu Ala Se | 6.274 | 502.32336 | [M+H]+ | 502.32339 | 74.3554:6617 95.82383:5824 183.62494:5457 | -5.97225E-08 |
| POS2584 | C)OC(C(CC(C)O)C) | 5.244 | 161.11703 | [M+H]+ | 161.1171 | 52.41368:6692 59.04859:8937 | -4.34467E-07 |
| POS8562 | eliosupine N-oxid | 5.663 | 414.21255 | [M+Na]+ | 414.21259 | 77.00605:5821 85.02795:15976 119.08327:28298 173.38612:7537 | -9.65688E-08 |
| POS6795 | Estriol | 8.81 | 311.16177 | [M+2H]2+ | 311.16171 | 55.05365:58863 56.96447:7808 57.06895:432831 58.07271:19524 60.08024:27058 67.05389:88310 69.06867:69084 71.08546:254363 72.08836:10565 81.06832:104380 82.073:12499 83.08508:55048 85.10044:123869 86.0954:109906 87.09917:8681 89.05894:6052 95.08476:162792 96.08826:10354 97.09952:57875 102.09041:20834 104.10696:106566 109.10005:71073 111.1148:21223 115.96169:10215 116.98483:6866 123.11522:27162 124.99953:28113 130.99998:7779 150.03761:8254 166.54468:12846 173.43033:15135 181.02368:7195 184.06927:22644 240.09717:13120 | 1.92826E-07 |
| NEG6125 | Sclareol | 0.754 | 307.27808 | [M-H]- | 307.2782 | 76.37918:6321 76.39638:6059 189.36215:6130 | -3.90526E-07 |
| POS5599 | Enokipodin C | 6.059 | 265.14273 | [M+Na]+ | 265.1427 | 57.06894:271096 67.16013:5953 83.01192:16628 85.06333:68407 139.07352:6696 | 1.13147E-07 |
| NEG1909 | Isatinoxim | 0.179 | 161.0356 | [M-H]- | 161.03571 | 55.08417:6740 57.10281:10574 59.08407:77697 67.31788:5529 71.09903:13234 73.11623:150130 82.68986:5427 85.13133:27001 101.14608:15075 110.22124:7649 113.15997:10825 | -6.83078E-07 |
| POS9809 | Ardisiaquinone E | 6.66 | 485.28726 | [M+H]+ | 485.28729 | 55.91729:6877 59.04858:18935 91.0539:15434 133.09863:54886 173.39111:11258 317.47849:6127 | -6.18191E-08 |
| POS14978 | -3-hydroxydecan | 6.997 | 780.54718 | [M+NH4]+ | 780.547 | 56.16686:6569 60.65643:4927 69.75047:6196 70.66898:5524 77.57815:5151 85.42:5496 92.73379:5286 148.68123:5243 152.18787:6049 173.3862:10346 345.00034:6225 772.16333:7117 | 2.30608E-07 |
| POS5229 | lamino)phenyl]car | 4.8 | 251.10243 | [M+H]+ | 251.10258 | 59.04858:48481 60.04425:81896 69.06991:8564 70.02779:69771 70.09202:6754 74.02328:16012 81.21274:5624 87.04333:12693 88.03894:227375 89.04271:8569 94.06358:167546 98.02461:9232 99.0074:33563 104.04995:9980 116.03413:7494 117.0528:7389 120.04356:59548 123.03605:5659 130.04883:21732 132.04305:8725 136.0761:17339 146.05719:220239 147.06316:7815 150.05341:12704 158.04501:9386 174.05496:49223 192.06534:8392 214.37151:5709 | -5.97365E-07 |
| POS236 | Divinyl sulfide | 1.56 | 87.02609 | [M+H]+ | 87.0262 | 56.04863:17014 57.05693:39967 69.03294:10719 69.06868:27926 86.05939:34811 86.09541:16429 | -1.26399E-06 |
| POS7879 | Bortezomib | 4.451 | 367.19409 | [M+H]+ | 367.194 | 55.05365:7737 62.02026:6111 69.03294:36387 74.89256:5499 83.04932:16016 86.05939:42416 87.04334:41179 89.05896:33192 93.07034:15025 98.05798:24268 98.65292:5798 99.04339:11100 111.04189:29575 112.07434:26726 113.07734:7178 128.07144:8579 133.08543:6665 152.10727:6122 173.34698:5856 178.11943:16843 317.81894:5571 | 2.45102E-07 |
| POS6698 | phenyl)-3,4-dihyd | 2.76 | 307.08212 | [M+2H]2+ | 307.082 | 76.03857:7299 84.04314:51760 130.04883:21051 | 3.90775E-07 |

**Differences in metabolites between the Model group and the WJW group**

| Alignment ID | Metabolite name | Rt(min) | Expreiment Mz | Adduct type | Reference m/z | MS/MS spectrum | PPM |
|---|---|---|---|---|---|---|---|
| POS4448 | CHEMBL4283703 | 5.497 | 223.06314 | [M+H-H2O]+ | 223.0632 | 55.05365:8100 58.06512:6930 59.04858:16663 67.05389:6855 67.55789:6106 73.02811:8641 73.04555:15009 89.05896:9864 91.05576:12415 93.06841:16797 95.08477:8575 97.51802:7162 107.08486:6847 148.72409:5533 186.02084:6854 209.01401:7058 225.04044:77480 226.04431:8379 | -2.68982E-07 |
| NEG1882 | Dihydroxynaphtha | 4.314 | 160.05246 | [M-H2O-H]- | 160.05243 | 57.10281:6266 59.08407:42864 62.57202:5617 73.11623:55262 97.1813:5084 154.52921:4889 | 1.87439E-07 |
| POS15135 | 11Z,14Z,17Z)/18:3 | 7.002 | 804.5509 | [M+H]+ | 804.55103 | 173.43526:10543 280.09122:6543 635.19501:6346 | -1.61581E-07 |
| POS12709 | ne-3alpha,29-dio | 7.894 | 603.40442 | [M+2H]2+ | 603.4043 | 76.80186:5699 145.42993:6409 173.43034:9974 296.44928:6733 | 1.98872E-07 |
| POS14041 | E(16:1(9Z)/16:1(9Z | 8.694 | 688.4928 | [M+H]+ | 688.49115 | 116.11739:6684 173.43523:11648 324.71918:5487 384.25632:6124 | 2.39654E-06 |
| POS10277 | Asn Phe Ala Arg | 6.663 | 507.26715 | [M+Na]+ | 507.2673 | 55.42575:7476 59.62183:6501 83.06232:5808 107.32325:7394 158.40836:5391 184.34851:5577 | -2.95702E-07 |
| POS5047 | Pseudouridine | 1.585 | 245.07607 | [M+H]+ | 245.0761 | 54.03372:7226 57.3748:6140 61.03869:8235 68.04829:6953 79.05161:7372 80.04967:7066 82.02831:10593 100.03728:16503 125.03256:34265 155.04594:13881 167.04433:6999 189.8046:5950 191.04048:9591 | -1.22411E-07 |
| POS8795 | Leu Arg Leu | 6.871 | 429.31827 | [M+H]+ | 429.3183 | 70.0706:6749 171.49448:6082 | -6.98782E-08 |
| POS10278 | Amoritin | 6.663 | 507.27118 | [M+H]+ | 507.27121 | 76.2698:6746 149.75777:6022 | -5.914E-08 |
| POS15192 | PC(18:0/18:1(9Z)) | 6.665 | 810.59821 | [M+2H]2+ | 810.59833 | 62.37422:6728 207.28555:5488 | -1.48039E-07 |
| POS6798 | 3-hydroxypentane | 4.559 | 311.20444 | [M+H-H2O]+ | 311.20441 | 57.03287:52959 59.04859:2111908 60.05226:27540 73.02812:20430 73.06432:14659 85.06506:30420 87.04335:156446 87.07999:9067 89.05896:2519716 90.06191:43693 101.05931:56081 103.03849:67758 103.07444:277993 107.07059:27440 117.05281:22880 129.08911:11014 131.06766:9861 133.08545:399722 134.08684:16435 147.10149:78077 151.09705:15682 | 9.63997E-08 |
| POS6932 | Didemnilactone A | 5.088 | 317.21069 | [M+H-H2O]+ | 317.21069 | 55.01768:7347 55.05452:7069 57.06987:7703 67.05387:6094 69.06866:6327 71.08545:24519 79.05461:6074 81.06831:11657 83.75761:5774 99.07936:7885 105.06942:6155 108.6657:6482 117.06909:6488 129.07018:7550 143.08569:7294 175.81395:5798 | 0 |
| POS7967 | 5beta-Coprostano | 0.607 | 371.36737 | [M+H]+ | 371.36722 | 57.06989:9920 59.03105:40356 61.01003:10249 73.04689:37856 75.02498:32068 77.04093:20602 91.05577:34151 93.03564:23727 | 4.03913E-07 |
| POS4258 | Oxododecanoic a | 5.797 | 215.16423 | [M+H]+ | 215.1642 | 57.03287:10104 66.7261:6671 67.05389:5776 122.57718:6670 142.00749:5780 | 1.39428E-07 |
| POS10586 | oPC(0:0/18:2(9Z,1 | 5.3 | 520.33905 | [M+H-H2O]+ | 520.33899 | 57.0338:18046 59.04859:455554 60.05226:11576 73.02812:27370 86.09541:8922 87.04335:53734 89.05897:271589 101.05931:34605 103.07445:89088 104.10697:36869 117.0909:11733 133.08545:61497 147.1015:32816 184.07468:44142 387.79099:10316 | 1.15309E-07 |
| POS7208 | Tianshic acid | 6.156 | 331.24756 | [M+H]+ | 331.24759 | 55.36465:6892 178.25224:6519 | -9.05667E-08 |
| POS13403 | Filixic acid pbp | 6.87 | 641.25934 | [M+H]+ | 641.25922 | 67.85222:5791 86.21047:5133 151.98245:6514 223.58301:5499 | 1.87132E-07 |
| POS4256 | Putaminoxin E | 5.609 | 215.16414 | [M+H]+ | 215.16409 | 50.61698:4718 50.62858:5628 50.634:5520 52.61634:5988 59.28901:5307 61.05301:4706 63.6503:4244 65.26049:4134 68.36765:4736 69.36423:4484 70.8761:4970 74.7813:4590 77.10919:3789 79.32551:4591 88.21125:5039 88.39301:4773 92.35327:4313 92.43147:4989 96.87998:4409 98.64025:4532 100.98293:4280 103.63856:4294 104.2325:4271 112.76032:3816 116.427:4465 117.93621:4574 120.90114:4925 160.97826:4331 161.43983:4379 164.86342:5118 178.03259:5728 181.50056:4790 182.55013:3901 195.91809:4328 196.93501:4740 203.49271:4507 208.5345:4770 217.56131:4415 217.65092:4551 | 2.32381E-07 |
| POS4257 | Oxododecanoic a | 5.971 | 215.16423 | [M+H-H2O]+ | 215.1642 | 64.30312:6434 69.00585:5486 74.06844:6177 173.3911:9235 | 1.39428E-07 |
| POS1486 | CHLOROACETIC A | 9.179 | 128.95036 | [M+H]+ | 128.95039 | 53.03856:10805 54.03373:10138 55.0177:10933 55.05365:150397 55.93345:179525 56.02069:8500 56.04862:16413 57.03286:41085 57.04396:29066 58.06416:18311 59.04858:7804 60.04425:15010 65.03804:9745 67.04092:12917 67.05389:6669 69.03417:9779 69.06868:26954 69.93349:9102 70.0643:10771 71.04816:6777 72.04237:6227 80.04967:8994 82.06502:99688 83.04769:18093 83.0672:10035 84.04315:20834 84.07957:10450 84.58686:6181 86.05938:12259 100.07597:15920 101.0593:6259 101.36545:5561 110.06083:13659 114.97044:45719 128.04031:16387 128.06833:26616 128.10568:17720 | -2.32648E-07 |
| POS2454 | S-HNE | 5.797 | 157.12212 | [M+H-H2O]+ | 157.12219 | 59.829:6147 67.64503:6272 70.0643:11551 74.93713:42569 92.94704:7407 115.9617:16302 122.9072:5297 | -4.45513E-07 |
| POS763 | -2-Methylthiazoli | 1.565 | 104.05288 | [M+NH4]+ | 104.0528 | 56.04863:29277 57.03287:24803 58.06512:336618 60.08025:66032 61.01003:36530 61.02846:30831 62.05909:8009 69.03294:6299 86.05939:11487 104.10697:38356 | 7.6884E-07 |
| POS17 | Nitroethane | 4.635 | 76.03915 | [M+H]+ | 76.0392 | 51.24459:4285 55.05716:4903 55.30983:7160 57.15702:5153 57.30393:4660 57.6643:4205 57.95031:3573 58.80668:4248 60.95991:4197 61.88621:4240 65.07974:4101 66.60102:5484 69.31342:3955 69.56071:5039 70.01898:4557 71.18597:5216 72.46844:4568 74.39948:4665 | -6.57556E-07 |

| Differences in metabolites between the Model group and the WJW group | | | | | | | |
|---|---|---|---|---|---|---|---|
| Alignment ID | Metabolite name | Rt(min) | Expreiment Mz | Adduct type | Reference m/z | MS/MS spectrum | PPM |
| POS7696 | Erythrophloin D | 5.256 | 355.26282 | [M+H-H2O]+ | 355.26279 | 55.05365:12225 59.04858:19960 67.05389:9657 70.21569:5375 81.06833:32453 83.4408:5835 85.06506:7493 89.05896:19468 93.06841:22560 95.08477:27711 95.58656:5191 99.04339:10223 101.0593:7161 105.06945:25400 107.08486:16717 109.10006:22608 117.06913:6411 119.08333:19304 121.1013:21053 123.08004:16834 131.08699:13095 133.10193:25328 135.11646:18656 145.09904:14060 147.11681:21592 149.13393:7716 159.11723:32874 161.13197:16357 173.13152:12958 185.13043:8133 256.09998:6970 | 8.44445E-08 |
| POS3713 | L-Arginine | 0.948 | 197.10004 | [M+H]+ | 197.10001 | 95.17444:5217 173.43034:11655 | 1.52207E-07 |
| POS2606 | Aminopentanoic a | 1.019 | 162.04997 | [M+NH4]+ | 162.05 | 55.0177:68381 55.05365:34633 56.04862:40127 57.03286:476354 58.06511:99164 59.07295:127286 60.08024:2624557 61.02742:35746 70.0643:36310 84.07957:63394 85.02799:1124644 98.06006:93569 102.09041:716844 103.03848:1458833 162.11159:578657 | -1.85128E-07 |
| POS1896 | Phosphonoacetate | 8.79 | 140.99532 | [M+NH4]+ | 140.99519 | 50.61313:7477 51.93935:6905 53.03856:8842 54.03373:7841 55.02909:45250 55.05365:9078 55.94513:44629 56.9414:14445 56.96447:9520 57.9342:627768 58.06511:187980 58.9425:28599 59.92951:71728 67.05389:9024 67.93391:26829 69.06991:20850 70.0643:38305 71.05973:16846 72.08047:9118 75.94611:12554 83.05907:36608 84.94554:12106 85.07516:57226 94.06358:6991 97.07692:10413 98.07049:20568 98.96088:40620 98.98203:8721 102.95542:6715 105.72243:7483 112.08707:64567 112.96638:9198 113.96311:9495 116.97124:453924 117.97478:22373 118.96619:46724 126.96738:10885 139.9868:49409 141.11403:45662 | 9.22017E-07 |
| POS2457 | S-HNE | 5.411 | 157.12216 | [2M+H]+ | 157.12219 | 55.0177:8019 55.05365:8181 67.05389:7626 67.55908:5260 69.06992:7577 70.06556:10614 74.93713:27741 142.21487:5011 | -1.90934E-07 |
| POS6933 | Didemnilactone A | 5.508 | 317.21069 | M+CH3OH+H] | 317.21069 | 78.49305:6069 86.90919:5969 97.06458:75463 109.06334:59271 153.79721:6272 173.39107:5731 184.94119:6312 232.74396:6119 | 0 |
| POS2456 | S-HNE | 6.185 | 157.12212 | [M+H]+ | 157.12219 | 53.70765:6653 55.73544:6473 55.87062:7001 55.8778:7466 70.06431:7513 84.07958:5323 | -4.45513E-07 |
| POS3306 | Phosphorylcholine | 5.717 | 184.07246 | [M+H-H2O]+ | 184.07249 | 53.48685:5860 57.93419:6489 58.94249:11718 79.25425:5942 86.09538:11694 99.9063:7164 116.97122:31489 117.9775:41158 | -1.62979E-07 |
| POS5956 | Prolyl-Tyrosine | 4.473 | 279.13336 | [M+H-H2O]+ | 279.13339 | 55.76759:6338 56.0486:70977 59.86378:5781 61.01:21312 67.05386:6587 68.04947:12414 70.06427:7602 70.77242:5663 81.06986:10469 84.04312:63320 86.05935:62849 88.02827:5406 95.08472:5874 102.05494:7477 103.05193:7343 104.05219:77849 120.08025:216265 130.04878:6549 132.06256:107539 133.02934:31081 150.0573:14199 | -1.07475E-07 |
| NEG1495 | D-Lysine | 8.738 | 145.0985 | [M-H]- | 145.0985 | 50.06327:6758 64.09624:10247 65.09129:6982 77.10647:7974 84.03101:22843 100.0459:104530 101.05441:2798763 102.0561:244824 116.05927:96064 117.06683:50671 118.18127:60178 145.22426:7981 | 0 |
| POS6430 | trahydrobungeanc | 6.202 | 296.25693 | [M+H]+ | 296.25681 | 50.40575:6657 55.05365:9155 57.03286:6456 57.06895:157124 59.75949:6013 67.05389:34264 69.06867:16648 71.08546:12163 81.06832:25536 89.05894:5649 93.0684:9678 95.08475:21343 107.08485:6420 109.10004:19765 112.94058:5981 127.10883:20812 135.11642:11814 169.12219:8839 | 4.05054E-07 |
| POS6447 | Nimbidiol | 5.318 | 297.14624 | [M+2H]2+ | 297.14612 | 55.05366:18863 57.06896:140606 67.05389:27585 69.06869:15980 71.08548:78878 81.06834:33064 83.0851:25721 85.10045:44797 86.09541:36444 89.05896:6026 95.08477:48806 97.09953:11422 102.09042:6365 104.10697:25479 109.10007:18973 123.11524:6592 124.99956:5615 184.07466:6961 | 4.03842E-07 |
| POS1076 | HLOROMETHYL) E | 9.304 | 114.97112 | [M+NH4]+ | 114.9711 | 55.0177:41067 55.05365:44699 58.06511:8999 59.04858:59962 60.04425:8361 61.01003:6709 62.98917:6479 68.04829:32860 69.03294:20927 69.06868:46368 70.02779:6245 70.0643:18437 72.04369:17074 72.08047:14898 72.93706:194456 73.04554:22057 73.93719:9384 79.05312:24758 80.05737:6212 86.05938:11508 87.04333:22870 90.94762:44448 91.05576:24234 96.08018:17580 97.0646:6942 108.9558:6241 113.96311:8090 114.06508:26326 114.09125:90865 115.09498:7965 | 1.73957E-07 |
| POS1077 | HLOROMETHYL) E | 0.146 | 114.97119 | [M+H]+ | 114.9711 | 55.0177:41439 55.05365:39687 55.93435:11379 59.04858:44006 60.04425:8325 61.01002:8971 65.69519:5897 68.04829:22913 69.03294:18476 69.06991:48364 70.02779:10448 70.0643:21124 72.04369:14221 72.08047:17478 72.93706:245053 73.04688:21064 73.93718:9329 76.96111:5797 79.05312:20383 86.06966:7235 87.04333:17434 90.94762:49751 91.05576:36055 96.08018:8058 97.06255:5899 97.50974:5642 113.9631:11952 114.06507:26415 114.09124:80031 115.09232:6779 | 7.82805E-07 |
| POS6235 | Sempervilam | 6.28 | 289.13318 | [M+H]+ | 289.1333 | 67.05389:10254 81.06989:6707 86.0954:23349 104.10696:26327 117.12083:5852 124.99955:8917 173.42543:11102 184.06929:12494 | -4.15033E-07 |

| Differences in metabolites between the Model group and the WJW group | | | | | | | |
|---|---|---|---|---|---|---|---|
| Alignment ID | Metabolite name | Rt(min) | Expreiment Mz | Adduct type | Reference m/z | MS/MS spectrum | PPM |
| POS1575 | 3-Oxobutyl acetat | 4.882 | 131.0701 | [M+H]+ | 131.0701 | 56.04953:8861 59.04859:7283 67.05272:7041 69.06992:8089 73.02812:7622 73.06432:17796 84.04317:12866 84.07958:32323 86.09541:17493 87.00325:10920 88.00349:25491 91.0539:27177 97.00919:20774 103.05197:10683 115.96439:9271 130.06477:10960 130.08708:8023 131.08377:10556 131.97467:6433 | 0 |
| POS1564 | lichloro-1,1-ethan | 0.114 | 130.96603 | [M+H]+ | 130.966 | 55.05365:16079 55.93435:8576 56.04862:30593 57.03286:12117 57.06895:13709 58.05276:5832 60.04425:7659 67.05389:27151 69.03294:8992 70.0643:6230 70.76221:6356 71.04816:7946 71.92825:120858 72.93706:51136 74.02328:6586 75.02496:6958 77.06126:6536 83.04769:7578 84.04315:29053 84.07957:78908 85.06505:10994 85.08358:5625 86.05938:9231 86.0954:8645 89.93904:39558 90.94762:14004 93.03563:6575 107.94919:30535 111.93941:6010 112.07433:11074 125.95948:15501 129.99471:6324 130.08707:7610 130.96458:5561 | 2.29067E-07 |
| POS1833 | Suberic acid | 6.058 | 139.07501 | [M+H]+ | 139.075 | 50.5135:6387 55.01768:12965 55.05363:10633 57.06894:14914 58.06509:12052 73.30093:5932 83.01192:63402 93.0703:5907 95.04691:8212 | 7.19036E-08 |
| POS1148 | Ethyl pyruvate | 5.392 | 117.0544 | [M+H]+ | 117.0545 | 55.05366:16095 59.04859:15239 64.36852:5225 70.06431:169751 71.04945:5714 71.06875:8396 72.04501:6564 72.08048:16839 101.25597:5752 | -8.54303E-07 |
| POS3068 | Allantoic acid | 1.006 | 177.06184 | [M+H]+ | 177.06183 | 60.05523:12147 70.06428:25050 71.06744:23278 82.464:6268 146.761:5011 146.91003:5418 | 5.64774E-08 |
| NEG5828 | hinidin 3-sambubi | 5.923 | 297.56561 | [M-H]- | 297.56546 | 92.23355:6520 94.94423:6029 101.05437:6192 173.62036:10440 182.25806:6476 292.43253:5709 | 5.04091E-07 |
| POS665 | hyltetrahydrofuran | 5.894 | 101.05945 | [M+H]+ | 101.0596 | 55.05364:12225 60.04425:40639 61.03868:12871 98.69289:5492 100.0738:7191 | -1.48427E-06 |
| POS4133 | leodomycin B | 5.797 | 211.13225 | [M+H]+ | 211.1324 | 57.03287:11300 69.06868:6229 70.42085:6023 71.04816:5428 76.21976:5535 81.0699:9033 95.08477:10395 109.06336:13332 119.08333:6266 125.03258:7347 133.10193:10793 151.10901:114635 165.12749:9258 | -7.10455E-07 |
| POS4451 | lelta-Valerolacton | 0.889 | 223.09393 | M+CH3OH+H] | 223.09399 | 58.27951:5910 62.98166:6700 78.99126:5440 80.94775:8679 96.921:226607 103.89967:5711 104.99084:6436 144.94151:5536 164.92259:37831 202.468:6480 | -2.68945E-07 |
| POS149 | al\|Nitrile-(()-3-Hyc | 9.005 | 84.04433 | [M+H-H2O]+ | 84.0443 | 56.0486:120292 56.2347:7436 62.64165:6270 84.04312:82510 | 3.56955E-07 |
| POS3959 | Anofinic acid | 1.176 | 205.0856 | [M+H]+ | 205.0856 | 57.03287:12657 57.06897:11826 60.08025:113265 85.02801:495511 86.03025:37113 149.02058:15997 | 0 |
| POS247 | 3-Aminopyrrolidin | 8.756 | 87.09145 | [M+NH4]+ | 87.0916 | 86.05938:18741 87.09045:69839 | -1.72232E-06 |
| POS672 | hyltetrahydrofuran | 5.335 | 101.05948 | [M+Na]+ | 101.0596 | 55.05366:9573 60.04327:16959 64.96264:6955 70.27892:5149 96.75728:5504 103.46888:5735 | -1.18742E-06 |
| POS243 | Butanal, 2-oxo- | 4.902 | 87.04405 | M+CH3OH+H] | 87.04405 | 54.09435:6368 86.05939:24522 87.04335:10728 | 0 |
| POS1740 | ydroxyanthranilic | 3.644 | 136.03891 | [M+H-H2O]+ | 136.039 | 55.93435:66812 71.92825:19297 72.93706:64380 86.21218:5189 89.93904:6074 90.94762:18292 91.05389:29653 108.99001:5988 136.02156:10530 136.05905:19517 | -6.61575E-07 |
| POS336 | L-Alanine | 2.49 | 90.05499 | [M+H]+ | 90.05498 | 61.02742:736144 62.03179:24408 72.04368:7129 72.08047:7254 | 1.11043E-07 |
| POS7346 | 13Z-Docosenamid | 6.315 | 338.34091 | [M+H]+ | 338.341 | 50.33126:5881 111.81992:5735 173.39104:5693 176.81448:5137 196.18338:6242 | -2.66004E-07 |
| POS6847 | MG(16:0/0:0/0:0) | 6.322 | 313.27203 | [M+H]+ | 313.272 | 57.06987:13058 92.41432:6239 110.36397:5778 173.39598:13388 214.98621:5459 260.67572:6780 | 9.57634E-08 |
| POS6454 | -6,10,14-trimethyl | 5.097 | 297.2413 | [M+H]+ | 297.2413 | 55.05364:12699 57.06895:78902 67.05388:29424 69.0699:17559 69.81181:6200 71.08546:34550 81.06831:23723 83.08508:9349 84.08121:5730 85.10043:19462 86.09538:15480 89.05894:5805 95.08475:31751 95.21631:6185 104.10694:13999 109.10004:8792 172.32704:5568 | 0 |
| POS1868 | BETAINE | 1.318 | 140.0679 | [M+H]+ | 140.06787 | 67.05389:6287 93.9344:5387 94.06358:19246 112.03867:7627 | 2.14182E-07 |
| POS4446 | Aminoanthraquinc | 1.523 | 223.0631 | [M+H]+ | 223.063 | 58.06416:18318 59.04858:9237 61.03971:21594 73.02811:13800 73.04688:17204 89.05895:7088 91.05576:21940 93.07033:9530 119.04705:6844 209.00751:10711 225.04042:94361 | 4.48304E-07 |
| POS2178 | alic acid 4-Me est | 2.683 | 149.04434 | [M+H]+ | 149.0444 | 65.03803:6116 91.05762:6280 103.0542:6043 103.80193:5951 | -4.02565E-07 |
| POS895 | octa-1,3,6-triene | 9.574 | 109.10102 | [M+H]+ | 109.1011 | 67.05386:20167 67.51138:5484 70.40051:5189 72.86618:6126 72.96379:5746 73.08709:7201 74.07662:5161 79.60238:5924 87.0032:8411 109.07554:8961 111.96735:6002 | -7.33265E-07 |
| POS593 | lethyl-2(5H)-furan | 9.001 | 99.04386 | [M+H]+ | 99.044 | 52.14494:6029 53.03854:7984 55.93433:11867 57.06894:21089 72.93703:45790 89.961:6771 90.94759:7406 98.9841:14208 | -1.41351E-06 |
| POS61 | Benzene | 3.996 | 79.05422 | [M+H]+ | 79.0542 | 51.36534:4811 51.44291:4633 51.60176:4579 55.41333:5178 59.86081:4222 60.2205:3878 61.02845:11184 61.59448:5402 61.78178:4911 64.48734:4628 68.45152:4331 70.77499:5100 71.3941:5594 76.17404:3930 78.70107:4524 | 2.52991E-07 |
| POS187 | -butenoic acid ga | 9.595 | 85.02846 | [M+Na]+ | 85.02838 | 53.01286:15435 56.04862:137267 67.05389:6919 84.04315:124379 84.07957:15886 85.02799:6883 85.04652:10727 | 9.40862E-07 |
| POS5118 | H-Leu-Asp-OH | 1.668 | 247.12817 | [M+NH4]+ | 247.1282 | 61.03972:6894 72.08047:42287 84.04316:21456 84.07957:9799 85.02799:14644 118.08494:10756 171.03236:5870 187.84866:5711 | -1.21394E-07 |
| POS181 | -butenoic acid ga | 9.903 | 85.02835 | [M+H]+ | 85.02838 | 53.77023:6062 55.05363:6463 56.0486:93024 84.04312:65379 | -3.52823E-07 |

| Differences in metabolites between the Model group and the WJW group | | | | | | | |
|---|---|---|---|---|---|---|---|
| Alignment ID | Metabolite name | Rt(min) | Expreiment Mz | Adduct type | Reference m/z | MS/MS spectrum | PPM |
| POS446 | 2-Methylpyridine | 3.062 | 94.06503 | [M+H]+ | 94.065 | 53.25908:4990 57.03286:6170 95.04893:8674 | 3.18928E-07 |
| POS930 | -propan-2-ylpyrro | 8.752 | 110.09641 | [M+H]+ | 110.0963 | 51.58027:6929 57.9342:33504 71.94005:11487 74.99706:25631 86.50518:14018 87.00323:7748 88.00347:108227 97.00917:47521 99.51075:24002 108.51748:9324 112.96638:11638 | 9.99125E-07 |
| POS631 | Pent-4-enamide | 2.585 | 100.07546 | [M+H]+ | 100.0756 | 55.01771:9721 55.05366:19322 58.02808:7424 70.41451:5645 76.19833:5992 98.98415:14159 | -1.39894E-06 |
| POS3955 | Eriosematin F | 1.524 | 205.08527 | [M+H]+ | 205.0854 | 57.03287:14839 57.06989:12885 60.08025:91444 85.028:404395 86.03025:32870 121.02692:7917 149.02058:22586 | -6.33882E-07 |
| POS8047 | hydroxycrambesc | 5.802 | 376.25919 | [M+NH4]+ | 376.2594 | 57.06896:980735 58.07272:16000 80.38296:5747 83.32634:5984 125.07162:16895 126.05367:46383 209.12439:64788 210.11511:10225 218.12764:23013 219.11343:8727 275.17361:7848 292.19846:9008 293.18784:16395 297.95679:5695 302.18762:21385 343.06805:6336 376.26254:23085 | -5.58126E-07 |
| POS8735 | Nafoxidine | 4.614 | 426.24298 | [M+2H]2+ | 426.24277 | 55.05365:50348 59.04858:49053 61.02845:31074 67.05389:20798 69.03294:835472 70.0366:21185 71.04945:25943 73.02811:207204 73.06432:84852 78.03804:39467 80.05429:60806 81.03229:27254 81.0699:14234 83.04932:143834 85.02799:23662 87.04334:669015 88.04782:18084 89.05896:2239860 90.0619:79698 91.04643:17202 95.04893:43332 99.04339:386962 100.05018:102858 102.06606:49288 103.03849:20744 107.06821:25936 109.06336:23486 111.04441:191889 113.05927:53207 117.09089:26953 122.06293:147835 122.56553:16009 125.0596:38296 129.05446:69540 131.07088:66151 133.08543:619741 134.09018:31549 137.0598:20853 144.07597:61997 155.07083:74452 166.08865:23580 173.07771:22267 177.11285:50953 | 4.92677E-07 |
| POS13902 | )-dioic acid 29-me | 4.652 | 677.38928 | [M+2H]2+ | 677.38953 | 69.03294:73562 73.02811:197486 73.06432:65878 81.03229:39471 82.00758:33699 83.04932:46218 87.04334:562341 89.05896:2671234 90.0619:58131 91.04643:49146 95.04893:109715 99.04339:72157 111.04189:137158 113.05927:74273 117.09089:32831 131.07088:47371 133.08543:871621 134.09018:38962 137.0598:50855 155.07083:46456 177.11285:107687 | -3.69064E-07 |
| POS9607 | Netilmicin | 4.497 | 476.30658 | [M+H]+ | 476.30679 | 73.06431:16723 82.98758:9562 87.04333:88920 89.05895:1344325 90.0619:26633 107.52888:8753 117.09088:10341 130.08388:8904 131.06764:18442 133.08543:536390 134.09016:13500 175.09525:9280 177.11284:78839 177.44234:9902 | -4.40892E-07 |
| POS5546 | enyl beta-D-gluco | 4.585 | 263.14828 | [M+2H]2+ | 263.1485 | 69.03294:7268 87.04334:5508 171.10925:6106 191.00081:5391 253.66177:6313 | -8.3603E-07 |
| POS6878 | 2-methylphenyl)ch | 1.033 | 315.0018 | M+CH3OH+H] | 315.00201 | 62.98163:10330 80.94772:43991 82.94373:11288 133.82373:5799 135.00177:798502 136.00449:11211 | -6.66662E-07 |
| POS1643 | THTC | 1.565 | 133.03145 | [M+H]+ | 133.03169 | 55.0177:11503 55.05365:19549 56.04863:22394 57.05693:74716 58.06512:49342 67.05389:8925 69.03294:13369 69.06868:753039 70.07312:57048 86.09541:3624676 87.09918:275236 132.10175:9428 | -1.80408E-06 |
| POS6474 | Stepharine | 8.809 | 298.14288 | [2M+H]2+ | 298.14447 | 55.05366:34647 57.06896:290541 58.07272:17207 60.08025:16111 67.05389:60108 69.06869:44183 71.08419:158150 72.08836:10216 81.06834:70186 83.0851:45791 85.10046:70404 86.09541:80791 95.08478:106599 96.08828:8833 97.09953:26441 99.07939:7069 102.09042:16028 103.09242:6131 104.10697:65978 109.10007:47414 110.10549:5637 123.11524:17088 124.99956:19890 152.28874:5179 173.39111:11392 181.02371:7528 184.07466:13722 189.81023:5380 240.09721:7437 | -5.33299E-06 |
| POS10095 | alpha,6alpha,7bet | 5.146 | 499.3259 | [M+2H]2+ | 499.32571 | 57.03287:76137 59.04859:1558574 73.02812:180120 73.06432:77930 80.0543:78842 85.06506:35346 87.04335:275850 87.06079:126498 89.05896:1979272 90.06191:77094 101.05931:196932 102.06606:46237 103.07444:392972 104.10697:220163 105.10879:93223 109.0756:62332 129.08911:44561 131.07088:47159 133.08545:758990 134.09018:37677 138.09604:31302 147.10149:220400 177.11285:76722 184.06931:79134 191.1256:37782 344.14871:30997 | 3.80513E-07 |
| POS11993 | hodeoxycholylargi | 5.564 | 571.38263 | [M+2H]2+ | 571.38293 | 57.03287:54088 59.04859:910919 60.05226:26733 69.06992:16959 73.02812:81292 73.06432:36065 74.06708:9281 85.06337:30932 87.04335:141471 87.06254:28789 87.07999:32295 89.05896:563763 90.06191:22655 94.06947:26926 99.07939:13228 101.05931:142150 103.07444:250230 104.10469:18320 107.06821:11190 115.07378:18379 116.08248:9978 117.0909:23833 129.08911:15358 131.06766:15681 131.10634:9048 133.08545:194027 134.08684:17871 145.08403:13019 147.10149:105449 161.11441:12545 171.97781:9731 177.1078:10406 191.1256:13171 205.14238:9301 | -5.25042E-07 |

**Differences in metabolites between the Model group and the WJW group**

| Alignment ID | Metabolite name | Rt(min) | Expreiment Mz | Adduct type | Reference m/z | MS/MS spectrum | PPM |
|---|---|---|---|---|---|---|---|
| POS8488 | Val Leu Tyr | 4.962 | 408.25195 | M+CH3OH+H]2 | 408.24921 | 59.04858:44387 87.04334:13409 89.05896:68683 95.42019:6092 101.0593:12186 103.07669:10578 133.08545:26958 | 6.71159E-06 |
| POS10966 | Roehybridine | 8.789 | 534.29517 | [M+Na]+ | 534.29541 | 60.08025:12284 65.02566:7665 86.09541:75514 104.10696:321832 105.66643:9116 112.49341:8183 | -4.4919E-07 |
| POS15389 | PC(18:2(2E,4E)/0:0 | 6.255 | 1039.67102 | [2M+H]+ | 1039.67053 | 56.0486:8800 58.06509:8734 60.08023:132038 63.92054:5359 67.05387:6755 71.07258:26955 86.09538:200052 95.08274:5910 98.98199:7908 104.10693:977993 105.11106:7441 116.77584:6002 121.29324:7112 121.79109:6253 124.9995:39813 173.38614:14816 184.07458:276262 185.0817:7575 914.8385:6452 | 4.71303E-07 |
| NEG5130 | Terezine A | 0.991 | 273.12494 | [M-H2O-H]- | 273.12469 | 87.11253:30944 89.13125:90150 93.03925:253037 95.03763:525814 97.03745:222648 145.23549:11469 147.15915:29333 153.06755:11910 155.06651:16051 | 9.15333E-07 |
| POS14049 | ethylbutyroyl),22-( | 5.172 | 689.46246 | [M+H]+ | 689.46222 | 57.03287:127973 59.04859:2489306 73.02812:97416 73.06432:81669 85.06337:72944 87.04335:451024 87.07999:84081 89.05896:2099070 99.07939:51889 101.05931:431129 103.07444:982473 115.07378:50248 117.05281:98589 117.0909:64985 131.06766:89739 133.08545:674337 145.08403:49569 147.10149:648273 161.1188:132124 173.39603:93594 177.11285:44225 191.1256:86610 205.14238:50020 355.71689:43311 | 3.48097E-07 |
| POS8879 | Suvanine | 4.698 | 435.2565 | [M+H]+ | 435.25629 | 51.50556:7580 67.73112:5330 89.05894:12640 123.58301:5278 183.53943:6587 | 4.82474E-07 |
| POS11844 | manzamine M | 5.053 | 565.35388 | [2M+H]+ | 565.35358 | 59.04858:259007 73.02811:50874 73.06431:30974 78.36626:16107 80.05274:24239 87.04333:86028 89.05894:518801 101.05929:33520 103.07442:72740 123.34731:18975 133.08542:191946 147.10147:40567 173.43033:40313 177.11282:31289 194.32018:20204 446.87405:18802 | 5.30641E-07 |
| POS6696 | 4-Gallocatechol | 3.027 | 307.08179 | [M+2H]2+ | 307.0816 | 56.04862:16784 68.02298:7664 76.0215:19080 76.03858:56598 84.04315:369127 96.04378:9515 102.05498:9126 130.04883:145558 131.04509:24300 140.01526:12904 177.03188:30486 179.0471:13981 | 6.18728E-07 |
| POS8786 | Mifepristone | 4.548 | 429.267 | [M+H]+ | 429.26682 | 59.04859:101581 73.02812:9553 73.06432:14635 85.06506:7925 87.04335:104053 89.05896:1139310 90.06191:21204 101.05931:5715 103.07444:21527 107.06821:7909 129.56607:6863 131.06766:16384 133.08545:384629 134.08684:9803 147.10149:14268 154.65695:5826 177.11285:33393 287.56656:5856 | 4.1932E-07 |
| POS5025 | Agomelatine | 5.56 | 244.13278 | [M+NH4]+ | 244.133 | 67.05389:23319 69.06868:1558184 70.07312:40942 115.05522:13405 130.06477:303271 131.06766:12601 132.08218:16202 142.06566:259660 143.06737:13584 144.07968:12297 160.07417:332565 161.07927:17557 176.06961:21747 188.07004:192286 189.07663:13067 198.12756:12154 | -9.01148E-07 |
| POS3675 | Hexylresorcinol | 4.373 | 195.12135 | [M+H]+ | 195.1216 | 58.06417:84777 89.05896:127199 95.26823:5724 107.04918:40419 133.08545:6906 135.04228:115350 136.04544:21897 194.11676:14281 | -1.28125E-06 |
| POS12104 | a-Tocopherol succ | 5.451 | 575.36816 | [M+2H]2+ | 575.36798 | 59.04859:160598 67.015:20277 73.02812:13311 79.78577:10853 87.04335:32101 89.05896:181868 101.05931:14248 103.07444:47691 133.08545:56907 147.10149:15684 | 3.12843E-07 |
| POS3346 | htho[2,1-b]thioph | 0.892 | 185.04167 | [M+NH4]+ | 185.0419 | 125.02051:21327 | -1.24296E-06 |
| POS10477 | Ile Glu Leu Lys | 5.62 | 516.33887 | [M+2H]2+ | 516.33911 | 57.03287:26206 57.06896:9009 59.04859:219972 67.015:15101 73.02812:19965 73.06432:11710 81.03072:13328 85.06506:7129 87.04335:26028 87.07999:7319 89.05896:145585 90.06191:7778 101.05931:22095 103.07444:54508 117.05281:9146 126.02024:8756 129.08911:7557 133.08545:40360 147.10149:20887 149.97055:7802 161.11441:6022 165.10016:7441 194.85654:5915 340.77332:6237 | -4.64811E-07 |
| POS11755 | onjugated chenod | 5.607 | 562.34882 | [M+H]+ | 562.349 | 57.97966:12413 59.04856:44377 60.08022:29247 67.61633:14164 86.09536:69963 89.05891:29635 91.32316:10827 104.10692:295921 105.11105:15458 184.07457:89546 | -3.20086E-07 |
| NEG2957 | Monuron | 1.216 | 197.049 | [M-H]- | 197.0488 | 55.23539:6281 68.33942:6047 73.52995:5450 81.53988:5503 161.03627:460728 162.03664:28802 163.0374:1032564 198.04526:168570 | 1.01498E-06 |
| POS12674 | Gly Asn Leu Arg Ly | 5.094 | 601.37762 | M+CH3OH+H] | 601.37793 | 57.03287:14339 59.04859:165112 73.02812:14904 73.06432:10785 87.04335:701428 89.05896:178052 101.05931:156728 103.03849:51479 103.07444:41422 115.07378:12363 117.05281:35948 131.06766:17236 133.08545:55064 147.10149:20260 217.73381:9698 | -5.15483E-07 |
| POS12835 | 9-Oxide ; Paecilot | 5.672 | 608.91724 | [M+2H]2+ | 608.91699 | 59.04859:265532 73.02812:20811 85.06337:17145 87.04335:54346 87.06079:14047 89.05896:228246 101.05931:63835 103.07444:93415 133.08545:72130 147.10149:36558 164.48682:14678 | 4.10565E-07 |

**Differences in metabolites between the Model group and the WJW group**

| Alignment ID | Metabolite name | Rt(min) | Expreiment Mz | Adduct type | Reference m/z | MS/MS spectrum | PPM |
|---|---|---|---|---|---|---|---|
| POS10700 | Calcimycin | 6.108 | 524.27417 | [M+Na]+ | 524.27399 | 57.03286:1100542 58.03661:42022 59.04858:301784 60.08024:29131 73.58075:13876 81.06989:33230 86.0954:87303 99.07938:87036 101.09422:19774 104.10696:303568 115.07377:73317 117.09088:21560 157.11888:72431 173.43033:28487 184.06927:115013 462.50906:13869 522.58624:12674 | 3.43332E-07 |
| POS12148 | PA(15:1(9Z)/12:0) | 5.744 | 577.38403 | [M+2H]2+ | 577.38422 | 55.29747:20168 57.03287:71034 59.04859:217476 67.07867:19889 73.02812:56650 85.06337:37893 87.04335:72367 87.06079:26980 89.05896:315632 101.05931:70320 103.07444:46810 129.08911:41048 133.08545:103642 147.10149:30193 173.38622:22558 | -3.2907E-07 |
| POS1671 | L-Alanine | 0.975 | 134.01877 | [M+NH4]+ | 134.019 | 62.98165:6876 70.06429:208419 71.06873:13251 74.02327:22845 90.05455:18754 91.05762:41642 92.05856:6291 93.26151:5477 115.08436:6481 116.06902:6879 133.07881:8893 | -1.71617E-06 |
| POS4659 | ko-1,2,3,4-tetrahy | 0.757 | 231.0336 | [M+H]+ | 231.0334 | 75.13534:6435 86.99104:18560 94.8403:5815 101.00695:12948 105.0024:19452 106.35088:5540 109.73916:5246 113.00764:8928 119.01637:16041 129.00409:13318 131.01932:16179 147.01338:23615 | 8.65676E-07 |
| POS8252 | Metazin | 4.667 | 391.22971 | [M+Na]+ | 391.22989 | 73.02812:6638 89.05896:17041 133.08545:9736 | -4.60088E-07 |
| POS4682 | Eremanthin | 4.376 | 231.13277 | [M+H-H2O]+ | 231.133 | 55.05365:24699 56.04862:40425 57.03286:18158 57.06988:19215 58.06511:8324 60.08024:104564 69.06991:17024 71.04816:10536 72.08047:78270 84.07957:178283 85.02798:890626 86.0954:77961 100.07381:50362 112.07433:16446 126.09013:85200 129.1017:10055 132.10173:9434 144.10196:9662 168.13461:19759 173.0777:11329 214.14249:8406 | -9.95098E-07 |
| POS11572 | Muscanone | 5.775 | 555.36932 | [M+2H]2+ | 555.36951 | 55.05366:11162 57.03287:75288 59.04859:163324 65.04931:8099 73.02812:47099 73.06432:9453 85.06337:39725 87.04335:75640 87.06254:15720 87.07999:16998 89.05896:198155 90.06191:8464 101.05931:57031 103.07444:31080 109.0756:6983 115.07378:7632 129.09227:12419 133.08545:57503 143.10413:8150 147.10149:12617 552.71912:6996 | -3.42115E-07 |
| POS13394 | cynthiaxanthin ac | 6.478 | 640.41254 | [M+H]+ | 640.41278 | 57.03287:116401 59.04859:51566 72.64896:22764 73.15427:20225 86.09541:26475 95.44221:21301 165.65282:20413 173.43034:39705 575.79932:22827 | -3.74758E-07 |
| NEG6716 | Thr Pro Asn | 1.086 | 329.14691 | [M-H]- | 329.1467 | 87.11256:12794 89.13128:25800 93.03928:344289 95.03767:312418 97.03749:31470 103.08175:9911 130.59087:5960 145.14165:6423 145.23553:7752 151.06717:7590 153.0676:22636 182.01541:5827 | 6.38013E-07 |
| POS7358 | norpregna-1,3,5,7 | 4.714 | 339.19522 | [M+H]+ | 339.1954 | 57.03287:11041 59.04859:260958 72.71816:7359 73.02812:6847 87.04335:270072 88.04605:5926 89.05896:78140 101.05931:32140 103.03849:103628 103.07444:35871 117.05281:30389 131.06766:6514 133.08545:6862 147.10149:6776 290.02237:5411 | -5.30668E-07 |
| POS3669 | 01!(E)-4-hydroxyr | 0.887 | 195.09877 | [M+NH4]+ | 195.099 | 82.34032:6379 82.37885:6615 88.44839:5652 162.27571:6158 173.43031:6117 | -1.17889E-06 |
| POS5232 | Paucine | 4.416 | 251.13805 | [M+NH4]+ | 251.13831 | 69.03294:10049 72.77946:5754 73.02945:6132 86.05938:17064 88.03894:18168 101.0593:5852 103.05421:11782 120.04356:14019 120.08029:37074 123.67455:5556 131.04831:6292 147.0555:16864 171.53304:5898 188.10326:14079 | -1.03529E-06 |
| POS11802 | Rhodoxanthin | 7.672 | 563.39111 | [M+2H]2+ | 563.39081 | 82.57995:5554 136.10338:5762 136.28088:7128 345.74472:6147 | 5.3249E-07 |
| POS680 | Homopiperazine | 6.518 | 101.10713 | [M+H]+ | 101.10732 | 51.99327:6998 70.19422:6915 101.10733:5472 | -1.87919E-06 |
| POS3872 | metric dimethylar | 5.069 | 202.14316 | [M+H-H2O]+ | 202.14296 | 54.00388:6561 55.05365:108846 57.06895:16962 69.06867:15266 76.7874:5737 97.09953:36648 97.24764:5777 101.0593:6591 114.12527:932384 115.12946:22465 130.06476:5500 156.13799:21795 160.13072:36610 | 9.89399E-07 |
| NEG4747 | ecyloxypropane-1 | 4.153 | 259.22815 | [M-H]- | 259.22791 | 53.9787:5890 59.0295:9226 60.40223:5661 64.27615:5905 70.36988:6031 92.55002:6256 103.07276:30071 | 9.25826E-07 |
| NEG2216 | Glycylproline | 8.782 | 171.07729 | [2M-H]- | 171.07751 | 56.02806:6761 56.26738:6083 61.21238:5515 84.03104:7884 109.19517:7395 136.52611:6194 | -1.28597E-06 |
| POS506 | 2-Dichloroethyler | 0.128 | 96.96085 | [M+H-H2O]+ | 96.9606 | 50.63479:6013 55.0177:9152 55.05365:24431 55.93344:20500 65.42387:6768 68.04949:8679 69.04403:10401 96.04378:19276 97.03996:9028 97.0646:9691 | 2.57837E-06 |
| POS502 | 2-Dichloroethyler | 9.291 | 96.96079 | [M+H]+ | 96.9606 | 53.92981:6138 55.05365:20051 55.93435:14013 68.0495:6645 69.04403:7150 84.06301:5123 95.95288:6106 96.04378:13152 97.0379:12825 97.0646:7771 | 1.95956E-06 |
| POS1158 | -Ethylbutanoic aci | 5.974 | 117.09066 | [M+H]+ | 117.0909 | 55.05365:17360 64.34301:5615 65.67461:6123 70.0643:125691 71.06874:10479 72.08047:30359 | -2.04969E-06 |
| POS1657 | t-butyl propyl sulf | 1.18 | 133.10469 | [M+H]+ | 133.10451 | 57.05693:13463 58.06512:8432 61.01003:14805 69.06869:113703 70.07313:16056 86.09541:579528 87.02591:7053 87.09918:66588 90.05457:23341 | 1.35232E-06 |
| POS1160 | -Ethylbutanoic aci | 4.938 | 117.09067 | M+CH3OH+H] | 117.0909 | 55.05365:34633 70.0643:105869 71.61486:6049 72.08047:36936 98.5709:5404 103.54803:5433 | -1.96429E-06 |
| POS3201 | 80638-48-8 | 5.379 | 181.08533 | [M+Na]+ | 181.08549 | 135.07935:33604 | -8.83561E-07 |

| Differences in metabolites between the Model group and the WJW group | | | | | | | |
|---|---|---|---|---|---|---|---|
| Alignment ID | Metabolite name | Rt(min) | Expreiment Mz | Adduct type | Reference m/z | MS/MS spectrum | PPM |
| POS2896 | ole-3-propionic a | 5.061 | 172.07523 | [M+H-H2O]+ | 172.075 | 55.0177:67069 55.05366:45425 57.06989:8313 59.04859:10030 67.05389:105780 71.04816:8019 72.04369:39478 81.06834:15025 82.69934:6493 83.0851:13674 89.06979:11754 95.8057:5137 103.05422:7244 109.06336:6309 109.10007:35552 130.06477:157420 144.00543:6544 | 1.33663E-06 |
| POS302 | Acetoin | 5.447 | 89.05939 | [M+H]+ | 89.0596 | 55.93345:7048 61.02845:15928 72.93707:41035 85.18988:5897 90.94763:7938 | -2.35797E-06 |
| POS1482 | Galegine | 6.532 | 128.11792 | [M+Na]+ | 128.1181 | 58.06512:7710 62.30235:5532 71.10865:5107 88.24155:5415 128.11816:6668 | -1.40495E-06 |
| POS157 | al\|Nitrile-(()-3-Hyd | 1.136 | 84.0445 | [M+NH4]+ | 84.0443 | 55.05365:24761 56.04862:287729 65.03804:6148 66.17352:6014 67.05389:15938 69.05635:7034 80.31335:6038 82.06502:31476 82.94376:8824 83.06069:10527 84.04315:222538 84.07957:435292 | 2.3797E-06 |
| POS929 | Brunfelsamidine | 0.855 | 110.07095 | [M+H]+ | 110.0712 | 55.55091:5496 56.04953:7725 62.15791:5484 68.98124:8315 81.96613:5329 86.99279:13087 88.0851:5737 100.02225:7363 105.0024:22770 | -2.27126E-06 |
| NEG1147 | _LEUCINE-5,5,5-D | 1.191 | 133.10646 | [M-H]- | 133.10622 | 59.08403:32480 71.09898:515055 73.07996:114427 75.09879:9345 79.05367:981327 79.06424:33752 87.11253:6641 88.14607:14457 89.13124:43366 97.08466:1245166 115.14149:329179 133.05977:14749 133.17186:63786 133.20816:7937 | 1.80307E-06 |
| POS607 | mino-2-methylbu | 8.779 | 99.09141 | [M+H-H2O]+ | 99.0916 | 50.09744:5228 50.32975:5594 50.40268:5236 50.46883:5745 52.30545:4833 54.25877:4925 54.44379:5359 55.05365:23090 55.83027:6131 56.27641:5374 58.25276:5211 58.26422:5113 60.29585:5899 60.89658:5148 61.73696:4792 66.15387:3998 74.47533:4653 75.93189:5489 78.03951:4594 84.36342:4230 87.3108:4715 87.74342:4879 91.89187:4448 96.18139:4135 96.32538:3919 96.68372:5059 97.55318:4156 99.13871:4426 | -1.91742E-06 |
| NEG932 | imethylthiazol-2-a | 8.995 | 127.03379 | [M-H]- | 127.0336 | 53.92936:5989 65.28221:6656 | 1.49567E-06 |
| POS1854 | yl-2,5-dimethylpy | 6.462 | 139.12263 | [2M+H]+ | 139.12289 | 55.0291:14994 57.9342:17711 58.06417:68347 69.06869:10360 70.06431:14638 83.05908:18365 96.08018:7258 116.97126:9458 122.0948:18950 139.12291:11213 | -1.86885E-06 |
| POS3554 | bicyclo[4.3.0]nona | 7.201 | 191.14226 | [M+H]+ | 191.1425 | 55.68453:6124 140.74756:6479 | -1.25561E-06 |
| POS735 | yne\|Ethynylbenzer | 3.787 | 103.05397 | [M+H]+ | 103.0542 | 53.01283:7468 56.0486:15841 57.03284:73042 60.04423:58404 61.0274:97448 85.02795:45406 95.04889:10935 102.05493:8132 103.03844:9101 | -2.23184E-06 |
| POS1481 | Galegine | 8.094 | 128.11789 | [M+H]+ | 128.1181 | 51.12348:4462 54.13879:4788 54.93365:4623 56.42904:4269 56.67014:5177 58.83768:4200 61.44732:4207 63.79562:5265 70.97746:5200 73.28477:4825 78.06468:6157 87.17079:5211 102.15912:5638 103.44626:4638 | -1.63911E-06 |
| POS3553 | id, 3a,4,5,6,7,7a-h | 7.296 | 191.14224 | [M+H]+ | 191.142 | 55.69256:6028 60.02228:5774 67.29036:4872 70.4183:5808 | 1.25561E-06 |
| POS2945 | thyl-1,2-dihydron | 7.21 | 173.132 | [M+H-H2O]+ | 173.1322 | 50.28837:5925 88.32349:6088 | -1.15519E-06 |
| POS2649 | Geijeren | 7.249 | 163.1476 | [M+H]+ | 163.1478 | 63.15053:5460 63.16023:5160 119.3293:5345 | -1.22588E-06 |
| POS2 | 1-Propene-1-thio | 0.711 | 75.02599 | [M+H]+ | 75.0262 | 50.26234:6873 50.31902:6093 50.34586:4342 52.05128:4041 52.05774:5519 53.90685:4830 53.93237:6118 53.96725:5347 55.43372:4405 56.04863:5089 57.03379:6323 57.98443:4912 57.99202:4277 60.20647:4843 60.25968:5493 60.31799:5181 62.54278:4039 62.55872:5808 62.6055:4660 63.19151:4160 65.00201:4659 67.59607:4722 70.19422:3861 70.68302:4177 73.4035:4236 73.95222:4955 74.05886:5136 74.43671:4852 75.43698:4801 77.62954:4853 | -2.79902E-06 |
| POS7653 | | 9.493 | 353.34113 | M+CH3OH+H] | 353.34131 | 57.06989:8454 59.03007:18218 61.01003:11569 73.04688:11070 75.02497:19953 77.04092:10995 91.05576:16604 93.03564:21453 105.07176:6055 154.15424:5573 168.61324:5526 | -5.09422E-07 |
| POS158 | )-methylbutanenit | 5.967 | 84.08052 | [M+H]+ | 84.0807 | 50.23862:6708 52.12395:4311 56.04863:4952 58.34167:3949 58.59901:4274 59.41376:5032 60.71431:4162 62.00978:4429 62.5035:4784 65.93027:5282 70.29284:4345 70.38533:4521 70.97234:3916 71.45245:4370 74.19456:4335 76.51501:4700 80.29636:5403 81.71966:4195 82.41099:4707 84.04317:8207 86.10914:4745 | -2.1408E-06 |
| POS2116 | -3,4-dimethylpen | 4.537 | 147.10121 | [M+NH4]+ | 147.1015 | 55.05366:16159 59.04859:11035 64.92667:10670 71.04816:8067 72.08048:7752 84.07957:11127 86.05939:7596 90.46526:5589 91.0539:13732 118.0629:12580 | -1.97143E-06 |
| POS13869 | vernonioside B3 | 4.562 | 675.37421 | [M+NH4]+ | 675.37378 | 59.04858:37087 69.03294:269041 73.02811:92499 73.06432:73920 81.03385:33829 83.0477:105986 85.02631:26682 87.04334:613385 89.05896:1780757 90.0619:30257 95.04893:48746 99.04339:436998 111.04441:239063 113.05927:40765 117.09089:47411 122.54804:27416 125.0596:104342 129.05446:51720 131.07088:62767 133.08543:703142 134.08684:34437 137.05981:51558 155.07083:146177 173.07771:30422 177.11285:80850 | 6.36684E-07 |
| POS11942 | Hyperbrasilol A | 4.54 | 569.31042 | [M+Na]+ | 569.31079 | 243.29276:15178 | -6.49909E-07 |
| POS10732 | etramethyl-12-oxo | 4.524 | 525.28528 | [M+Na]+ | 525.28497 | 104.10696:90124 116.99842:13251 184.06927:53077 | 5.90156E-07 |
| POS9970 | Kermadecin C | 4.808 | 493.29453 | [M+H]+ | 493.2948 | 89.05896:14232 111.75651:8839 150.5957:6680 | -5.4734E-07 |
| POS7205 | Gly Arg Val | 4.894 | 331.20853 | [M+Na]+ | 331.2088 | 50.59146:6949 89.05891:7436 173.42535:7672 186.45195:6156 | -8.15196E-07 |

**Differences in metabolites between the Model group and the WJW group**

| Alignment ID | Metabolite name | Rt(min) | Expreiment Mz | Adduct type | Reference m/z | MS/MS spectrum | PPM |
|---|---|---|---|---|---|---|---|
| POS12827 | Roseocardin | 5.425 | 608.40125 | [M+2H]2+ | 608.40088 | 57.03287:54218 59.04859:969901 60.05226:32435 73.02812:94965 73.06432:38505 75.78008:13408 80.0543:18675 83.0477:14981 85.06337:49265 87.04335:169178 87.06079:40880 87.07999:43729 89.05896:1091298 90.06374:33013 94.06947:26629 101.05931:158968 103.07444:259107 116.08248:23405 117.0909:25837 129.08911:31419 131.06766:21780 133.08545:392229 134.08684:16872 147.10149:119249 161.11441:15337 177.11285:36405 191.1256:26122 | 6.08152E-07 |
| NEG2882 | pholino)-ethanesu | 1.208 | 194.04869 | [M-H]- | 194.0484 | 59.08403:9421 79.05367:22876 80.05276:8272 87.29256:5457 97.07645:5085384 159.03728:26253 | 1.49447E-06 |
| POS10867 | Ile Leu Ile Gly Thr | 5.137 | 530.35498 | [M+H]+ | 530.35468 | 59.04859:273368 73.02812:26540 73.06432:14036 87.04335:261049 87.06079:11436 89.05896:394889 90.06191:10841 101.05931:98209 103.03849:10424 103.07444:82931 117.05281:13607 131.06766:20291 133.08545:163177 147.10149:53517 177.11285:17248 191.13127:16924 200.78329:9637 | 5.65659E-07 |
| POS9325 | BW A868C | 4.53 | 460.28018 | [M+H]+ | 460.28049 | 70.35613:12729 71.03398:12288 72.08044:54452 82.99567:12845 84.07954:17718 86.09536:14624 87.0433:20802 89.05891:358132 90.06187:46690 120.08025:36316 133.08537:85632 173.42535:17198 457.95001:14539 | -6.73502E-07 |
| POS5104 | oxyacetaminopher | 1.038 | 247.01546 | [M+NH4]+ | 247.01511 | 62.98163:7152 64.72823:6041 101.51242:6272 104.10692:24282 134.01678:5969 135.00177:247258 | 1.41692E-06 |
| POS2118 | -3,4-dimethylpen | 5.027 | 147.10121 | [M+H]+ | 147.1015 | 55.05366:11502 56.04773:6884 59.04859:11893 64.92667:21080 67.82102:6960 69.06869:7496 70.45259:6235 72.08048:7240 76.67482:6051 86.05939:8729 91.0539:9974 100.07383:8196 118.0629:19497 130.08708:5411 | -1.97143E-06 |
| POS8878 | Balaenonol | 4.701 | 435.25327 | [M+Na]+ | 435.25299 | 89.05894:16135 133.08542:6624 159.1517:5039 163.68777:5218 252.45139:5891 | 6.43304E-07 |
| POS11213 | Val Ile Pro Lys Ser | 5.146 | 543.35034 | [M+2H]2+ | 543.34998 | 59.04858:120187 73.02811:23326 80.05428:13558 87.04333:28569 87.06078:11620 89.05894:233452 101.05929:19089 102.06604:7752 103.07442:30258 131.06763:8023 133.08542:75491 147.10146:16462 173.42542:14482 285.71133:8254 | 6.62556E-07 |
| POS13154 | -3,4-dehydro-apo | 4.842 | 625.37299 | [M+Na]+ | 625.37341 | 585.47443:14342 | -6.71599E-07 |
| POS13470 | lbutanoyl)-13-O-( | 5.195 | 645.43555 | [M+H]+ | 645.43597 | 59.04859:242310 66.66176:9597 73.02812:18954 87.04335:24629 89.05896:120566 101.05931:29662 103.07444:54756 117.05281:11269 133.08545:31894 147.10149:17486 155.30751:11078 599.66156:11795 | -6.50723E-07 |
| POS1084 | Siphonodin | 6.062 | 115.03861 | M+CH3OH+H] | 115.0389 | 55.01768:6203 55.05363:10908 68.04948:7986 69.06865:13654 70.06428:11377 75.8084:5719 83.01192:103439 96.24216:6652 107.414:5775 107.7495:5746 114.06504:6739 114.09121:16709 | -2.52089E-06 |
| POS10854 | Ala Ala Gln Asn Leu | 4.468 | 530.29352 | [M+2H]2+ | 530.29321 | 65.25937:5692 70.02776:17986 70.06428:5914 86.09537:38615 87.05377:74064 88.03892:7436 89.05892:49134 107.32559:6541 122.51595:5492 133.08539:30002 146.05714:7555 172.06981:10437 188.06996:26492 205.09813:8059 344.03888:5638 | 5.84582E-07 |
| POS9165 | Cytochalasin II | 4.969 | 452.27783 | M+CH3OH+H]2 | 452.2775 | 52.35993:6926 59.04859:26203 87.04335:17260 89.05896:52371 101.05931:18825 133.08545:17198 173.43036:7924 321.76129:5831 | 7.29641E-07 |
| POS7897 | yltricaprylylammo | 6.231 | 368.42371 | [M+H]+ | 368.42401 | 53.41133:6012 57.06989:9046 60.08025:22375 71.08418:8467 126.59031:6735 | -8.14279E-07 |
| POS8027 | Acetoxypregnenol | 5.196 | 375.1463 | M+CH3OH+H] | 375.14603 | 55.01768:11003 57.06894:23731 59.04856:18964 83.01192:36653 85.06333:16538 89.05892:15921 115.03928:16748 171.09958:11834 | 7.1972E-07 |
| POS2503 | ethylene-L-glutar | 2.278 | 159.07599 | [M+H]+ | 159.07629 | 55.05365:7750 55.93345:25019 56.04862:12251 61.03972:9412 62.29601:5395 67.05389:13226 70.0643:38752 72.93572:6851 81.07617:6065 95.08477:17780 115.05257:5547 117.05824:7372 | -1.88589E-06 |
| POS11453 | nino)-3beta-phtha | 4.923 | 551.34827 | [M+2H]2+ | 551.3479 | 50.11341:5893 61.73277:5959 89.05892:11118 104.10693:18044 104.21423:6262 133.08539:5544 | 6.71083E-07 |
| POS12812 | loxanthin 3-aceta | 5.801 | 607.41406 | [M+2H]2+ | 607.41449 | 57.03287:71227 59.04859:547655 69.06868:16950 73.02812:65250 73.06432:23441 85.06337:27886 87.04335:75830 87.07999:18021 89.05896:323129 94.06947:22078 101.05931:67832 103.07444:165300 115.07643:17031 117.0909:26454 129.08911:15801 131.10312:15080 133.08545:87414 147.10149:65990 154.42589:15842 161.1188:17895 568.3938:14728 | -7.07919E-07 |
| POS9908 | Mmv676063 | 5.958 | 490.28867 | [M+Na]+ | 490.289 | 59.04859:7609 60.08025:7720 69.77051:5489 86.09541:33568 104.10697:183850 146.97896:17678 190.10825:6852 345.91049:5415 | -6.73072E-07 |
| POS13396 | propan-2-yl)-1,7,1 | 5.778 | 640.41736 | [M+2H]2+ | 640.41699 | 57.03287:18330 59.04859:110224 73.02812:21293 73.06432:10910 85.06506:9571 87.04335:33746 87.06254:11364 89.05896:105997 101.05931:33457 103.07445:20257 117.05281:10544 117.0909:9995 133.08545:43526 147.10149:14077 173.38622:9218 213.08313:8803 280.85791:9059 | 5.77749E-07 |

| Differences in metabolites between the Model group and the WJW group | | | | | | | |
|---|---|---|---|---|---|---|---|
| Alignment ID | Metabolite name | Rt(min) | Expreiment Mz | Adduct type | Reference m/z | MS/MS spectrum | PPM |
| NEG9902 | Oligomycin F | 8.8 | 803.53241 | [M-H]- | 803.53162 | 105.20542:5352 116.96082:5538 120.79679:5685 146.04282:5948 302.20255:6442 | 9.8316E-07 |
| POS6713 | rophenyl)naphtha | 2.993 | 308.08005 | [M+2H]2+ | 308.07971 | 56.04862:7136 59.54773:6225 74.0055:5790 76.0215:8827 76.03858:24778 84.04315:132124 85.04819:7381 130.04883:46154 131.04509:11298 | 1.10361E-06 |
| POS4707 | carnitine (isomer | 2.62 | 232.15359 | [M+H]+ | 232.1539 | 51.53652:7216 57.06986:11963 60.08022:18059 73.56039:5627 81.03226:7065 85.02796:114643 167.10455:17058 193.27718:5915 | -1.33532E-06 |
| POS3967 | Dihydroxydecanoic | 5.167 | 205.14304 | [M+H]+ | 205.14339 | 59.04859:23058 89.05896:11043 146.061:6498 | -1.70612E-06 |
| POS3968 | Dihydroxydecanoic | 5.045 | 205.14304 | [M+2H]2+ | 205.14339 | 53.84231:6575 54.14479:6940 88.4502:5964 102.45471:5068 108.92896:6128 110.70092:5999 173.43036:7271 | -1.70612E-06 |
| NEG1818 | ndoleacetaldehyde | 1.412 | 158.06174 | [M-H]- | 158.0614 | 51.81568:5803 58.04419:57416 59.08405:10121 68.85773:6177 71.09771:5955 89.13128:114845 90.1349:6742 99.78008:6799 104.08418:5829 | 2.15106E-06 |
| POS747 | lydroxy-2-pentan | 6.013 | 103.075 | [M+H]+ | 103.0753 | 59.04859:14124 61.0387:35706 | -2.91049E-06 |
| POS716 | Triethylamine | 8.459 | 102.12726 | [M+H]+ | 102.1276 | 50.32131:5604 50.48732:4526 51.19893:5189 51.50716:4412 52.29895:5871 52.71486:5036 53.88306:4640 54.18246:5254 55.00543:5181 59.37344:4488 60.23155:4397 60.9456:4849 61.22037:4370 68.22222:4167 70.48689:4791 70.78522:4244 71.1834:5263 71.56672:4789 87.3178:4485 87.85827:3923 88.16148:5554 91.93539:4267 92.01117:4186 92.1326:4756 92.31329:4411 93.96178:5724 96.02963:4223 96.6327:5515 98.03295:4386 | -3.32917E-06 |
| POS3840 | Matsutakic acid A | 9.749 | 201.11136 | [2M+H]+ | 201.11169 | 55.05365:227960 55.93435:129434 56.9414:111913 59.04858:14787 67.05389:7464 69.06991:6620 70.95692:13366 72.93706:49809 72.95981:12300 73.94402:8057 74.95245:24945 81.06989:9887 83.04769:43832 90.94762:32130 96.95995:11195 97.96836:93609 100.96772:13857 101.0593:69034 111.04189:43725 113.96311:482955 114.97044:68490 116.96038:12450 118.94112:14331 129.05446:14695 131.97466:90264 132.97011:21142 141.95662:110573 155.00035:7223 159.96982:69588 | -1.64088E-06 |
| POS369 | p-Cresol | 3.189 | 91.05404 | [M+H]+ | 91.0537 | 50.38117:6380 61.02845:9666 62.0318:14637 64.66679:6087 | 3.73406E-06 |
| POS7953 | trophenyl-L-Trypt | 6.984 | 371.09888 | [M+H]+ | 371.09857 | 173.44016:9933 | 8.35358E-07 |
| POS6849 | xo-nonadecanoic | 0.736 | 313.27216 | M+CH3OH+H] | 313.27289 | 57.06988:10981 57.52068:6191 124.39247:6272 161.19786:5075 | -2.33024E-06 |
| POS10761 | eryl-tyrosyl-leucyl | 6.064 | 526.29065 | [M+H]+ | 526.29108 | 55.05363:43554 57.03284:78227 62.05907:419133 62.19157:40989 67.05386:109530 79.05309:72262 81.06987:91738 91.05386:77026 93.06837:65164 98.98411:42704 104.10693:54144 105.06941:73745 105.22224:45939 119.08607:63324 131.08371:45324 173.39104:98496 | -8.17038E-07 |
| POS8037 | .!21-Acetoxypregr | 4.957 | 375.24762 | [M+H]2+ | 375.24799 | 57.03287:52016 58.04042:47946 59.04859:1374553 60.05226:30239 65.04819:56027 69.06992:15300 73.02812:258559 73.06432:31160 80.0543:63820 85.06337:34716 87.04335:197259 87.06079:104272 87.07999:20165 89.05896:1562559 90.06191:56718 94.06947:24597 101.05931:83424 102.06606:31793 103.07444:194860 104.07732:12680 107.07059:12543 109.07315:48915 116.08248:10905 124.08022:10182 129.08911:28066 131.06766:18310 131.08699:17165 133.08545:442284 134.09018:16067 147.10149:67507 173.42545:13363 177.1078:37213 191.1256:10836 | -9.86015E-07 |
| NEG3335 | Dimethyl-9H-xant | 0.972 | 209.09691 | [M-H2O-H]- | 209.09731 | 59.08403:16279 71.09898:6542 89.13125:14394 93.03925:1463321 97.03745:18826 97.07645:21359 131.2403:46894 | -1.91298E-06 |
| POS12382 | Ganoderic acid Mc | 5.054 | 587.39465 | [M+H]+ | 587.39423 | 57.03286:51786 59.04858:891911 60.05225:20113 73.02811:19863 87.04333:179564 87.07997:23421 89.05894:500972 100.07381:17644 101.05929:119738 103.03848:19811 103.07442:257044 117.09087:24642 129.09224:14715 130.08388:13756 133.08542:91030 147.10147:107219 161.11438:27688 191.12556:17070 203.61127:13223 247.26077:12099 | 7.15022E-07 |
| POS8033 | aragylasparagyllys | 4.88 | 375.19821 | [M+H]+ | 375.19861 | 59.04859:47938 73.02812:17314 79.26034:5756 87.04335:17922 87.06254:7271 89.05896:59470 101.05931:8757 103.0385:20065 131.06766:5695 133.08545:12704 168.01765:6012 185.73782:5824 311.60825:5348 374.63766:5259 | -1.0661E-06 |
| POS10474 | (18:4(6Z,9Z,12Z,15 | 4.654 | 516.30945 | [M+2H]2+ | 516.30902 | 73.02811:42732 73.06432:11415 80.05429:34236 87.04334:48191 89.05896:279765 90.06374:11849 91.04643:6372 102.06606:18262 124.08022:9198 133.08543:106621 162.54245:7142 177.11285:11255 309.0488:8241 | 8.32835E-07 |
| POS8643 | scutione | 4.921 | 419.25839 | [M+H]+ | 419.258 | 57.03287:7353 59.04859:32413 73.02812:7236 87.04335:17096 89.05896:45059 101.05931:9469 103.0385:17268 124.16041:5615 133.08545:14093 173.42545:10300 303.42111:5687 317.40564:5096 | 9.30215E-07 |
| POS13578 | ethyl-5-methylide | 5.052 | 653.40283 | [M+H]+ | 653.40234 | 59.04859:22567 70.18664:14842 87.04335:20137 89.05896:97236 95.55244:12756 131.36464:13900 133.08545:17226 268.88205:15103 618.28107:17286 | 7.49921E-07 |

| Differences in metabolites between the Model group and the WJW group | | | | | | | |
|---|---|---|---|---|---|---|---|
| Alignment ID | Metabolite name | Rt(min) | Expreiment Mz | Adduct type | Reference m/z | MS/MS spectrum | PPM |
| POS11926 | hyl-2-[6-(3-meth | 6.186 | 568.4364 | [M+H]+ | 568.43597 | 55.4754:85672 57.03287:352190 58.06511:125360 59.04858:93726 60.08025:1130365 67.05389:84753 69.93349:73463 71.0726:139182 81.06833:84518 86.0954:2759882 87.09918:96145 93.07034:89869 104.10696:12050610 105.10878:331944 109.23234:74300 124.99955:658444 175.14503:80713 184.07465:4111756 185.07634:120884 | 7.56462E-07 |
| POS10623 | Phe Val Lys Lys | 8.815 | 521.34406 | [M+2H]2+ | 521.34448 | 60.08022:28979 86.09537:76667 87.09914:6393 104.10693:159350 105.10874:18973 124.9995:13415 159.69203:5840 173.42537:6516 184.06921:172363 318.78229:6125 | -8.05609E-07 |
| POS14708 | Muronic acid | 5.168 | 750.51654 | [2M+H]+ | 750.51599 | 59.04859:215859 73.06432:32288 87.04335:32286 89.05896:250885 92.87973:18245 101.05931:39315 101.62239:17896 103.07445:71098 117.05281:55112 131.07088:20529 133.08545:96551 147.10149:54381 204.50024:21937 | 7.32829E-07 |
| POS12580 | ethylanhydrovilang | 5.437 | 597.38049 | [M+2H]2+ | 597.38 | 59.04859:141038 67.015:20370 73.02812:16096 81.03072:18390 87.04335:27664 88.08155:12357 89.05896:193138 90.06191:13491 101.05931:41205 103.07444:39391 133.08545:75424 147.10149:13880 194.0645:14267 | 8.20248E-07 |
| POS11348 | Eurycomaoside | 6.062 | 548.27057 | [M+Na]+ | 548.27014 | 53.59624:5870 59.04858:26783 89.05896:10466 91.0539:6945 104.10696:15133 120.96689:22884 | 7.84285E-07 |
| POS6152 | Myristoylglycine | 6.484 | 286.23709 | [M+H]+ | 286.23749 | 55.39032:5951 57.06896:39152 67.05389:7861 69.06992:7043 71.08548:23636 76.03859:91189 85.10045:6830 95.08477:13941 121.30764:5824 | -1.39744E-06 |
| POS12323 | 3a-pentamethyl-4 | 4.865 | 585.26538 | [M+H]+ | 585.26581 | 58.47013:5769 89.05896:14428 173.43034:11705 299.1311:5905 357.3071:6222 | -7.34709E-07 |
| POS8335 | a,8-dimethyl-6-ox | 5.75 | 397.19965 | [M+H-H2O]+ | 397.20001 | 59.04858:23504 69.03294:8009 87.04333:5658 89.05895:12635 119.08332:75640 | -9.06344E-07 |
| POS3442 | 4-(pyridin-2-yl)bu | 4.12 | 188.06781 | [M+H]+ | 188.06822 | 55.05365:9874 83.91093:5305 113.4112:6295 131.41315:6112 | -2.18006E-06 |
| POS10498 | ylnaringenin7-O-( | 6.21 | 516.99963 | [M+H]+ | 517 | 52.44873:4856 56.27097:4187 57.98917:5108 58.19838:4594 60.07725:4665 60.36026:5265 60.95378:5076 63.24981:4456 64.00186:4692 64.86604:4564 67.67013:4577 68.34462:4437 68.53918:4719 69.43006:5691 70.26247:4358 70.37391:4217 70.41577:4417 70.42593:4039 70.43608:4755 74.89813:4712 77.04238:4995 80.67318:5405 83.44244:4438 87.48277:4251 89.29951:5035 89.67585:4541 94.42513:4738 95.21034:4112 95.22631:3790 98.21039:4061 98.22293:4514 100.78278:4159 102.74039:4042 102.99358:4351 103.7225:4083 104.58311:3632 107.09676:4168 107.84329:4670 109.57148:4830 111.35164:4010 111.56139:3858 113.12126:4108 136.55806:4286 140.75113:4023 143.19608:5018 153.78905:3666 167.38799:5310 179.18613:4192 179.24796:4594 181.7372:4760 192.49483:5029 195.91818:4660 204.91539:4892 205.36974:5037 220.06386:4828 241.96165:4268 262.63007:4140 266.41821:4031 284.85248:4063 328.84229:4778 330.48773:4968 354.36633:4168 382.86893:4410 403.22705:5486 406.07657:4964 411.92078:4195 432.1152:4902 448.90985:6243 462.74417:4983 480.55896:4326 487.76578:5462 497.17715:4657 501.85251:4493 | -7.15667E-07 |
| POS14575 | Mubenin B | 6.597 | 735.4624 | [M+H]+ | 735.46301 | 94.29323:5399 115.30742:6495 117.48097:6924 201.29752:6080 | -8.29409E-07 |
| POS13936 | DTXSID60346618 | 8.667 | 679.47144 | [M+2H]2+ | 679.47198 | 70.29156:5858 92.95089:5664 105.49634:6093 215.87585:5333 286.19946:6859 304.34259:5523 | -7.94735E-07 |
| POS6151 | ncuronium bromi | 7.046 | 286.22839 | [M+NH4]+ | 286.228 | 73.09652:5308 113.8013:6307 | 1.36255E-06 |
| NEG1560 | Citramalic acid | 8.811 | 147.10043 | [M-H2O-H]- | 147.10001 | 59.08403:8234 74.67177:5613 101.05437:9917 102.05606:16315 103.05476:36319 | 2.8552E-06 |
| NEG1185 | Phenyl vinyl sulfide | 7.569 | 135.02791 | [M-H]- | 135.0275 | 65.09129:31817 66.07739:17039 66.08893:7004 79.05219:10746 89.13127:23061 92.12341:6997 92.13479:10820 100.0459:6214 102.05609:9050 107.15221:13537 117.06139:10774 135.0251:24814 135.05542:20732 | 3.03642E-06 |
| POS6410 | oleic acid, methyl | 1.207 | 295.26163 | [M+H]+ | 295.26199 | 51.64319:7048 55.05366:19076 57.06989:11474 67.05389:13073 69.06992:20634 69.89835:6757 81.06834:20028 83.0851:14047 95.08477:10860 97.09953:8127 107.08487:7368 121.1013:7297 133.09863:10153 | -1.21926E-06 |
| POS12448 | Austrobuxusin I | 4.568 | 591.35358 | [M+2H]2+ | 591.35303 | 73.06431:106240 87.04333:602119 89.05895:4109172 90.0619:73522 117.08816:56878 131.06764:114271 133.08543:1918621 134.08682:60913 173.43523:48508 175.09525:51397 177.11284:220419 296.69055:30865 570.67053:33803 581.78009:35843 | 9.3007E-07 |
| POS3075 | nyl-ribo-hexose-a | 4.594 | 177.11166 | [M+NH4]2+ | 177.11211 | 88.02121:34491 134.02684:9513 173.43524:6596 | -2.54076E-06 |
| POS12196 | odeoxycholyltrypto | 5.331 | 579.38055 | [M+2H]2+ | 579.38 | 57.03287:49203 59.04859:1127110 60.05226:37743 73.02812:124180 73.06432:32703 80.0543:27308 85.06506:35434 87.04335:219358 87.06079:43940 87.07999:42551 89.05896:1293560 90.06191:46038 94.06947:25213 101.05931:167521 103.07444:325522 109.0756:35468 116.08248:25592 117.0909:33581 129.08911:42456 131.06766:25643 133.08545:482817 134.08684:31299 147.10149:172306 161.11441:30490 177.11285:48489 191.1256:36244 | 9.49291E-07 |

| Differences in metabolites between the Model group and the WJW group | | | | | | | |
|---|---|---|---|---|---|---|---|
| Alignment ID | Metabolite name | Rt(min) | Expreiment Mz | Adduct type | Reference m/z | MS/MS spectrum | PPM |
| POS11050 | ydrocelastryl diace | 4.821 | 537.32159 | [M+Na]+ | 537.32111 | 51.77975:10735 53.44824:10138 172.19113:9965 384.88806:10550 | 8.93321E-07 |
| POS11667 | oxy-22,25-oxido- | 4.872 | 559.3634 | [M+H]+ | 559.36292 | 59.04858:164065 87.04334:24241 89.05896:243012 101.0593:19786 103.07444:37613 133.08545:52739 147.10149:11455 184.7793:10282 252.30504:9418 | 8.58119E-07 |
| POS11611 | Gln Val Leu Leu Gl | 5.352 | 557.36609 | [M+2H]2+ | 557.3656 | 57.03287:13623 59.04858:238876 69.06992:8568 73.02811:21019 73.06432:8827 85.06505:6937 85.42169:5890 87.04334:38228 87.06078:12802 87.07998:9133 89.05896:238150 90.0619:8125 101.0593:34692 103.07444:61192 109.07559:6788 109.61583:5807 110.70842:6597 129.08911:6293 133.08543:81501 147.10149:31376 173.39601:8739 226.88602:5403 359.24414:5833 | 8.79136E-07 |
| POS10524 | lemonnierin | 8.857 | 518.29767 | [M+H]+ | 518.29718 | 65.91531:6574 81.0699:13970 85.06506:15727 93.07034:7524 95.08477:15201 104.10696:29663 105.06945:8505 107.08486:11646 109.10006:13271 121.09843:9822 126.02023:100495 127.02272:19039 128.01544:50965 131.08377:7190 133.09863:8719 135.11646:6467 145.09904:8818 147.11681:9320 157.10197:6557 159.11723:15079 160.12202:11101 199.14969:12618 200.14934:6864 208.06328:12433 209.13087:10801 213.1633:7460 227.14313:9550 505.616:6482 | 9.45404E-07 |
| POS7395 | Myrsinoic acid B | 4.502 | 341.21442 | [M+H]+ | 341.21399 | 59.04859:420374 73.06432:26204 85.06506:15104 87.04335:166315 89.05896:2371431 90.06191:49744 96.08018:10842 101.05931:8375 103.03849:13936 103.07444:64185 107.07059:21353 114.09126:11675 129.08911:9317 131.07088:15901 133.08545:565314 134.08684:17578 147.10149:24456 173.43036:8183 177.11285:26297 | 1.26021E-06 |
| POS11845 | [æ¨ å å 4 | 4.528 | 565.35852 | [M+2H]2+ | 565.35822 | 87.04333:35172 89.05895:298107 90.0619:48146 133.08543:136004 134.08684:29761 140.82646:19314 173.3911:40907 176.78931:20847 314.63065:18759 487.51135:21257 | 5.30637E-07 |
| POS9389 | Stellettin B | 4.912 | 463.28534 | [M+H]+ | 463.2858 | 59.04859:17473 73.02812:6506 80.0543:6441 87.04335:9064 89.05896:27470 103.0385:7367 133.08545:13761 234.66223:5886 | -9.92908E-07 |
| POS8470 | rihydroxy-6-meth | 4.528 | 407.22345 | [M+Na]+ | 407.22299 | 62.14423:6016 86.05939:5706 163.52153:5868 258.34149:6538 | 1.1296E-06 |
| POS12380 | yloxy)icosanoyl]ox | 5.143 | 587.37946 | [M+2H]2+ | 587.37891 | 59.04859:361571 72.83016:28106 73.02812:91944 73.06432:36273 87.04335:142063 87.06254:31344 89.05896:826186 90.06191:26877 101.05931:88199 103.07444:125138 106.91372:28114 129.08911:28422 133.08545:281272 147.10149:61475 173.42545:34963 177.11285:64965 185.83572:27589 | 9.36363E-07 |
| POS11321 | Cholylarginine | 4.913 | 547.38452 | [M+H]+ | 547.38501 | 59.04856:201737 87.0433:19741 89.05891:174987 101.05926:35441 103.07439:54084 117.05275:24089 133.08537:41112 147.10141:25965 173.38612:22581 185.46078:14998 267.18558:15823 | -8.95165E-07 |
| POS12977 | Leu Gln Lys Ser Le | 5.217 | 616.40222 | [M+2H]2+ | 616.40277 | 57.03287:12818 59.04858:170867 73.02812:34173 87.04334:107092 87.06079:10222 88.04782:9812 89.05896:280759 101.0593:50900 102.06606:10401 103.07444:31834 133.08545:113618 145.04276:8984 147.10149:18346 177.11285:11702 348.84436:9617 | -8.92274E-07 |
| POS9288 | Gly Val Ile Gly Ile | 5.385 | 458.29684 | M+CH3OH+H]2 | 458.29721 | 57.03286:11500 59.04858:162045 60.05225:6547 61.36362:5549 67.01499:11342 81.03072:11099 87.04333:13940 89.05895:83040 101.0593:12643 103.07443:36718 125.31528:5654 133.08543:16914 141.435:5171 147.10147:11008 158.09196:7295 165.41951:5772 | -8.07336E-07 |
| POS5478 | 79966-13-5 | 0.759 | 261.00415 | [M+H]+ | 261.00372 | 55.08349:5608 61.03869:70612 62.50561:5753 162.99733:5917 | 1.64749E-06 |
| POS13697 | ,18-hexa(propan- | 6.477 | 662.3974 | [M+Na]+ | 662.39801 | 51.71899:51994 74.05202:54767 155.46558:50627 173.39111:118150 367.89346:58816 | -9.20896E-07 |
| NEG6605 | bicyclo[4.1.0]hept- | 1.021 | 325.14819 | [M-H]- | 325.14774 | 55.93273:5822 64.72199:5727 89.13125:15144 93.03925:471780 95.03763:46740 97.03745:13607 105.56303:5625 124.46915:5651 127.20192:7845 134.22708:7003 145.23547:44896 151.06712:33643 | 1.38399E-06 |
| POS7904 | erone 3-sulfate so | 5.752 | 369.17142 | [M+H]+ | 369.17099 | 57.06894:21290 59.04953:11239 79.15736:5939 79.33614:5442 85.06333:11476 89.05892:10233 | 1.16477E-06 |
| POS9047 | Difenacoum | 3.357 | 445.18066 | [M+H]+ | 445.18109 | 50.11115:6874 137.04604:132977 145.24556:5233 160.07417:39036 179.40268:6166 | -9.65899E-07 |
| POS3783 | Tacrine | 4.594 | 199.12416 | [M+H]2+ | 199.1246 | 73.51437:5165 75.64143:6344 81.06833:8050 | -2.20967E-06 |
| POS4255 | Oxododecanoic a | 6.138 | 215.16316 | [M+H]+ | 215.1636 | 51.47779:6184 55.45854:6857 | -2.04496E-06 |
| POS7800 | [12]-Shogaol | 6.047 | 361.27246 | [2M+H]+ | 361.27289 | 55.05366:8866 57.03287:8714 57.06896:5744 57.37107:5850 67.05389:19133 67.47569:5805 69.06869:6543 79.05313:12329 81.0699:19388 91.0539:16121 93.06841:10870 95.08478:10353 105.06946:8554 119.08613:10188 173.39111:10269 | -1.19024E-06 |
| POS2369 | ethyl propyl trisulf | 9.136 | 155.00212 | [M+H]+ | 155.00169 | 51.94016:10242 55.05365:17822 55.93345:133211 67.05389:30009 67.93391:7764 71.94005:16588 72.93706:1124896 73.93582:6224 79.05463:6605 81.06989:14737 90.94762:214569 97.5863:6159 108.08179:9968 108.95824:27118 113.96311:308913 114.97044:26824 126.97046:6325 131.97466:103719 | 2.77416E-06 |

| Differences in metabolites between the Model group and the WJW group | | | | | | | |
|---|---|---|---|---|---|---|---|
| Alignment ID | Metabolite name | Rt(min) | Expreiment Mz | Adduct type | Reference m/z | MS/MS spectrum | PPM |
| NEG1006 | Agmatine | 0.889 | 129.11505 | [M-H]- | 129.11459 | 62.06304:9145 88.1461:11047 | 3.56273E-06 |
| POS4898 | OPC-4:0 | 6.191 | 239.16335 | [M+H]+ | 239.1638 | 55.01769:7056 55.05452:11522 57.06987:16397 67.05388:6518 81.06988:9457 95.08475:19134 107.1801:6125 109.06334:22417 109.10004:6309 119.08609:11449 123.08001:29059 179.13972:130632 | -1.88156E-06 |
| POS1215 | de, methyl tert-pe | 1.053 | 119.08923 | [M+H]+ | 119.0888 | 53.03773:49580 55.05365:2183662 56.04951:38541 56.05673:209583 57.05692:204000 59.04858:37663 72.08047:2599418 73.08309:235070 | 3.61075E-06 |
| POS7456 | IC ACID DER (FR. L | 6.406 | 343.28378 | [M+2H]2+ | 343.28421 | 60.08125:10091 69.71296:6009 75.02497:9170 82.06503:9029 86.0954:24935 124.99955:8473 145.3998:6233 184.06929:46920 264.26468:14336 | -1.25261E-06 |
| NEG1256 | -Butanesulfonic ac | 8.411 | 137.02744 | [M-H]- | 137.02789 | 93.14729:7280 114.20587:5520 114.93765:5388 | -3.284E-06 |
| POS5562 | Z)-Octadecadiene | 6.776 | 263.23553 | [M+H]+ | 263.23599 | 201.77066:5538 257.78946:5736 | -1.74748E-06 |
| NEG471 | 1,2,4-Butanetriol | 0.337 | 106.06342 | [M-H]- | 106.063 | 53.45897:4091 54.47842:4136 56.19134:5904 59.97625:4719 60.3217:4124 62.3273:4462 62.51997:3862 62.68903:4419 64.66617:4102 64.67733:4571 65.33318:4541 65.69171:4110 68.71078:4153 68.82339:5140 68.90435:4902 69.84071:4193 70.78477:4560 74.1647:4320 76.61039:4261 76.85421:4962 81.62371:4848 82.3423:4234 86.58962:5238 90.25441:4335 92.34967:4441 95.33667:4001 96.94727:5738 101.89474:4618 109.04345:4326 | 3.95991E-06 |
| POS12057 | SNF-4794-12 | 4.934 | 573.38245 | [M+H]+ | 573.3819 | 57.03287:155241 59.04859:3207844 60.05226:53694 73.02812:126239 73.06432:67632 85.06337:85421 87.04335:737397 87.07999:92912 89.05896:3782734 90.06191:78200 101.05931:518869 101.09423:32070 103.03849:110417 103.07444:1274112 104.07732:51424 105.09028:30692 107.06821:33453 115.07378:44584 117.05281:62599 117.0909:75995 129.08911:65900 131.06766:93031 131.10634:47827 133.08545:1309605 134.08684:47921 143.10413:30559 145.08403:79916 147.10149:917573 148.1028:29181 161.1188:78016 177.11285:104586 191.1256:143377 205.14238:58272 | 9.59221E-07 |
| POS11866 | Leu Tyr Lys Glu | 6.195 | 566.31787 | [M+Na]+ | 566.31842 | 51.65913:10940 58.06512:15513 60.08025:31763 67.05272:14886 71.07261:14371 79.05313:11711 86.09541:230832 93.06841:17973 104.10697:1422411 105.10879:41028 119.08334:13797 146.97896:190666 180.48094:11372 354.95444:10709 | -9.71185E-07 |
| POS8339 | Rollicosin | 4.953 | 397.259 | [M+2H]2+ | 397.25851 | 57.03284:27986 59.04856:732509 60.05223:15843 65.04928:7615 69.06865:6942 73.02808:110844 73.06429:38310 80.05426:15877 85.06501:17184 87.0433:98442 87.06249:24442 87.07994:13465 89.05891:741093 90.06187:22602 94.06942:8094 100.07593:7046 101.05926:50173 102.06822:11634 103.07439:120326 104.07727:7604 107.06815:8568 109.07554:12543 117.05275:34792 117.09084:10051 129.09219:7225 130.08702:20660 131.07082:25295 133.08537:161299 134.08678:9455 147.10141:40354 161.11433:8025 173.39594:6746 177.11278:11449 | 1.23345E-06 |
| POS12516 | Destruxin B | 5.228 | 594.38672 | [M+2H]2+ | 594.38611 | 57.03287:26272 59.04859:538786 73.02812:101193 73.06432:24663 73.49814:17151 80.0543:19289 85.06337:28720 87.04335:178714 87.06254:36547 87.07999:24217 89.05896:1022678 90.06191:35777 101.05931:79315 102.06606:39869 103.07444:150524 109.0756:24944 114.26157:13530 129.08911:22954 131.06766:27389 133.08545:384610 134.09018:27651 147.10149:90130 177.11285:48414 230.37541:13202 233.37059:13654 262.24658:15602 276.94675:14044 | 1.02627E-06 |
| POS9151 | -5 trisulfate hexah | 5.267 | 451.28793 | [M+2H]2+ | 451.28741 | 57.03287:15085 59.04859:185668 67.015:17826 73.02812:7320 73.06432:9437 73.48596:7111 81.03072:14132 87.04335:17803 89.05896:148284 101.05931:29580 103.07444:35713 115.07378:6390 133.08545:38886 147.10149:13744 158.08772:5226 226.99617:5283 | 1.15226E-06 |
| POS7905 | erone 3-sulfate so | 5.468 | 369.17148 | [M+H-H2O]+ | 369.17099 | 53.51121:5726 57.06894:147905 59.04856:9290 62.43773:6368 67.10578:5489 83.04766:6463 85.06333:84177 89.05892:7449 107.04913:6400 121.02687:5973 127.07495:12709 133.0656:7208 137.05975:15837 147.04396:10677 167.10457:23500 173.43027:10163 179.7023:6258 221.1192:18449 286.83472:5713 | 1.3273E-06 |
| POS10896 | Ser Ile Arg Arg | 4.69 | 531.33557 | [M+H]+ | 531.33612 | 59.04858:43160 87.04333:65942 87.26875:22683 89.05894:465193 133.08542:152319 173.43033:28266 177.11282:27108 259.75623:19243 530.31659:22636 | -1.03513E-06 |
| POS11729 | ntibiotic Sch 2383 | 5.238 | 561.35284 | [M+2H]2+ | 561.35339 | 59.04859:87533 67.015:18922 73.06432:11342 87.04509:14094 89.05896:127367 93.83868:7235 103.07445:20350 117.05553:9711 133.08545:51224 147.10149:11926 428.1304:7850 477.49673:7311 | -9.79775E-07 |
| POS13779 | -Dibenzoyl karour | 5.304 | 667.43494 | [M+2H]2+ | 667.43561 | 59.04859:242078 73.02812:68694 84.68888:23790 87.04335:82863 89.05896:540016 101.05931:60546 103.07444:63152 117.0909:28913 130.72673:29701 133.08545:202787 225.53427:29601 | -1.00384E-06 |

| Differences in metabolites between the Model group and the WJW group | | | | | | | |
|---|---|---|---|---|---|---|---|
| Alignment ID | Metabolite name | Rt(min) | Expreiment Mz | Adduct type | Reference m/z | MS/MS spectrum | PPM |
| POS8754 | Arg Asp His | 4.437 | 427.2052 | [M+Na]+ | 427.20471 | 59.04956:6979 86.05939:7605 87.04335:5502 89.05896:15923 103.03849:13665 111.0419:5450 118.0629:26205 132.07892:13821 134.09685:11988 144.0797:16425 146.061:110536 147.06319:5783 159.09138:14633 188.07004:314637 189.07664:16750 205.09822:59622 | 1.14699E-06 |
| POS5814 | Alismorientol A | 5.97 | 273.20508 | M+CH3OH+H] | 273.2056 | 55.32309:6444 55.35403:5615 57.03287:8367 57.06989:6506 82.75266:6326 | -1.90333E-06 |
| POS12499 | Podototarin | 6.134 | 593.43353 | M+CH3OH+H] | 593.43292 | 50.67118:5669 54.03799:5682 57.03287:294743 59.04858:94944 67.95436:5881 81.06833:6117 99.07938:36638 101.09641:8090 107.9203:5749 115.07378:26630 117.08817:17043 137.96021:5530 157.12312:42351 173.43034:10610 | 1.02792E-06 |
| POS4818 | ethoxyphenyl)isoq | 5.91 | 236.10951 | [M+H]+ | 236.11 | 57.06896:21774 141.5977:5960 174.85666:6375 | -2.0753E-06 |
| POS5817 | Alismorientol A | 6.138 | 273.20511 | [M+H-H2O]+ | 273.2056 | 180.81987:6144 273.90359:6008 | -1.79352E-06 |
| NEG9489 | PFSA-ether | 5.373 | 530.91949 | [M-H]- | 530.92004 | 56.03255:8255 75.12117:5831 123.12397:138146 256.54803:6117 364.29367:5817 | -1.03594E-06 |
| POS6744 | -glucopyranosyl-1 | 4.867 | 309.19022 | [M+H]+ | 309.1907 | 55.0177:10556 57.03287:103329 59.04859:1483114 60.05226:15732 71.04816:26645 73.02812:31427 73.06432:408673 87.04335:42986 89.05896:10118 103.07445:11926 117.05281:211554 117.0909:37128 131.06766:139032 | -1.55244E-06 |
| POS5815 | Alismorientol A | 5.797 | 273.20508 | [M+H]+ | 273.2056 | 50.07689:6175 72.35727:6141 82.85947:5386 87.14983:5554 182.26979:5537 | -1.90333E-06 |
| POS7078 | xanecarbonyltetra | 8.014 | 324.32507 | [M+H]+ | 324.32559 | 51.92889:6355 62.49498:5815 65.37954:5827 87.17076:5573 105.47303:6335 144.18367:5420 221.77029:6139 | -1.60333E-06 |
| POS3888 | lydroxy-L-tryptop | 3.068 | 203.08051 | [M+H-H2O]+ | 203.08099 | 55.05365:15338 56.05764:6963 60.04426:13778 61.03972:9841 98.06007:7464 130.06477:8421 134.57501:6771 157.07237:7125 | -2.36359E-06 |
| POS4165 | Elaeokanine C | 4.978 | 212.16383 | [M+H-H2O]+ | 212.16431 | 55.05365:14712 57.06988:6819 60.26771:6333 67.05389:7738 69.06991:12367 81.06989:8879 83.08508:10264 93.0684:12155 105.40099:6211 107.08485:9791 | -2.2624E-06 |
| NEG398 | Styrene | 8.114 | 103.05589 | [M-H]- | 103.0554 | 50.26:5653 57.10277:13123 58.51401:6316 59.08403:19211 60.29253:5787 61.07175:5855 64.04998:6164 73.11619:8279 88.9059:5414 103.16489:8181 | 4.75472E-06 |
| POS1585 | )-2-Pentanol aceta | 5.696 | 131.10608 | [M+H]+ | 131.1066 | 56.04953:7138 66.73078:6452 84.04482:8608 84.07957:26514 86.09541:23548 88.00349:46180 91.0539:15445 97.00918:17650 99.51076:6357 105.87435:5223 115.96171:8913 131.08377:8452 | -3.96624E-06 |
| NEG1226 | ylpyridin-3-yl)bor | 7.106 | 136.05812 | [M-H]- | 136.0576 | 56.7561:5952 65.09239:8743 89.13125:21010 135.05537:9016 136.05812:8624 137.05487:11191 | 3.82191E-06 |
| POS7115 | (-)-Spectaline | 6.981 | 326.30392 | [M+H]+ | 326.30441 | 62.06014:7482 83.74775:5349 111.98013:5167 126.20273:5299 156.22183:5575 170.93153:6539 | -1.50167E-06 |
| POS6104 | adecanoyloxyprop | 7.722 | 284.29349 | [M+H]+ | 284.29401 | 50.15384:6115 55.83028:5610 70.08572:6014 73.98637:5237 173.38622:15165 205.2939:6562 | -1.82909E-06 |
| POS477 | 5Z)-1,3,5-Heptatr | 0.704 | 95.08558 | [M+H]+ | 95.08607 | 55.05365:40419 60.81196:6018 65.03804:9508 67.05389:59072 95.04893:12522 95.08476:16068 | -5.15323E-06 |
| POS5136 | Estrane | 7.14 | 247.24088 | [M+H-H2O]+ | 247.24139 | 60.68689:7013 68.0495:6114 101.14882:5995 130.32001:6294 189.62503:5234 | -2.06276E-06 |
| POS10064 | 1a,4,4a,5,6,7,7a,10 | 5.543 | 498.28558 | [M+H-H2O]+ | 498.28503 | 57.06989:52798 59.04858:117315 60.08025:319774 61.08378:61635 71.0726:40468 80.26237:38436 86.09541:637366 87.04334:89207 87.09918:212624 89.05896:111737 104.10696:3389616 105.10879:901230 106.11105:71854 124.42527:38870 124.99955:143348 126.02023:198528 143.13353:36345 173.43034:49780 184.07466:1275888 185.07635:350369 186.07533:109538 | 1.10379E-06 |
| POS6421 | clopropylethenyl)- | 8.844 | 296.14386 | [M+2H]2+ | 296.14441 | 55.0177:7234 55.05365:61963 57.06896:421288 58.06511:67268 58.07177:17376 59.07198:43990 60.08025:34325 67.05389:84423 69.06868:48493 71.0726:10686 71.08547:201446 72.08836:9254 76.53945:6980 81.06833:84160 83.08509:50908 85.10045:85449 86.0954:100834 95.08477:125856 97.09953:24544 102.09042:12012 104.10696:104389 109.10006:38765 123.11816:12654 124.99955:18668 137.5089:10278 149.01665:18663 156.51999:16360 157.01743:7404 165.52467:12755 184.07465:21663 193.53142:9053 256.00317:23735 | -1.8572E-06 |
| POS13106 | homodestcardin | 5.66 | 622.41663 | [M+2H]2+ | 622.4173 | 57.03287:119903 59.04859:1589836 69.06868:36093 73.02812:141734 73.06432:68803 85.06337:71467 87.04335:301380 87.06254:61232 87.07999:69568 89.05896:1353998 90.06191:32242 94.06947:52451 101.05931:317615 102.06384:30548 103.07444:550777 115.07378:36005 116.08248:32758 117.08817:47840 129.08911:66206 131.07088:55611 131.10634:49848 133.08545:577301 134.09018:40175 143.10413:33472 145.08403:32688 147.10149:335780 161.1188:45661 173.11685:31273 177.11285:74070 191.1256:68499 205.14238:45497 | -1.07645E-06 |
| POS10119 | tidylethanolamine | 5.495 | 500.27765 | [M+H-H2O]+ | 500.2771 | 59.04857:49635 62.06013:11238 67.21217:5362 87.04333:8936 89.05894:37492 103.07442:9126 104.10694:12132 126.02021:44426 127.02269:6110 128.01852:20755 | 1.09939E-06 |
| POS3197 | Nicotinuric acid | 4.505 | 181.05997 | [M+H]+ | 181.0605 | 51.02236:7006 65.03804:6560 68.75417:5609 78.0336:77533 96.04379:539695 97.04613:9670 97.21471:5970 106.02893:12081 115.702:6127 124.03867:318359 135.05576:213309 173.3862:8053 | -2.9272E-06 |
| POS11544 | Juvenimicin A2 | 8.739 | 554.36932 | [M+2H]2+ | 554.36871 | 74.18494:6379 124.93654:5543 173.3911:12067 290.60663:5553 | 1.10035E-06 |

**Differences in metabolites between the Model group and the WJW group**

| Alignment ID | Metabolite name | Rt(min) | Expreiment Mz | Adduct type | Reference m/z | MS/MS spectrum | PPM |
|---|---|---|---|---|---|---|---|
| NEG1756 | ethylpseudoconhy | 1.56 | 156.14005 | [M-H]- | 156.1395 | 78.18732:5748 82.16454:20570 89.13125:113313 97.07645:9456 111.15351:7432 151.103:5303 | 3.52249E-06 |
| POS11732 | -)-Strempeliopidir | 6.844 | 561.39569 | [M+Na]+ | 561.39508 | 53.71611:5663 95.83591:6661 158.69135:6209 261.57294:5663 273.44662:6200 | 1.08658E-06 |
| POS678 | Homopiperazine | 8.743 | 101.10677 | [M+Na]+ | 101.10732 | 70.53394:6167 | -5.43976E-06 |
| POS4068 | phenyl)-5-(Methyl | 1.583 | 209.05473 | [M+H]+ | 209.0542 | 77.12376:5258 102.64651:6055 162.43123:5468 180.68938:5230 | 2.53523E-06 |
| POS5134 | Estrane | 5.843 | 247.24084 | [M+H]+ | 247.24139 | 79.05312:6558 93.06841:11853 107.08486:7921 | -2.22455E-06 |
| POS3017 | densispicnin D | 4.881 | 175.13225 | [M+H-H2O]+ | 175.1328 | 53.40379:6508 59.04858:7105 70.78779:5501 94.23231:6106 104.95851:5791 106.06411:14832 130.06477:32586 134.06017:17831 173.39111:13848 | -3.14047E-06 |
| POS2917 | anethiosulfonic ac | 9.369 | 172.97636 | [M+H]+ | 172.9758 | 55.05366:23586 55.05892:6892 55.93345:32149 57.73019:6022 67.05389:78850 70.06431:6554 71.04945:6566 71.92827:70420 72.65827:5430 72.93707:64732 81.0699:10823 87.03812:6491 89.93906:65184 90.90295:65891 90.94764:22434 95.08477:6469 107.9492:38478 109.10007:25251 114.97046:10998 125.9595:24131 126.09015:8907 130.9646:25725 131.92909:12921 132.86481:5588 148.97369:38969 149.93901:14493 | 3.23745E-06 |
| POS8789 | '-Diapolycopened | 5.797 | 429.27969 | [2M+H]+ | 429.27911 | 57.03287:30988 57.06896:36714 60.08025:325791 71.08547:35847 85.02799:1805681 86.03024:139674 95.08477:25251 144.10197:24246 246.25351:11792 | 1.3511E-06 |
| POS10669 | LPE 19:0-d5 | 5.401 | 523.35559 | [M+H]+ | 523.35498 | 60.08022:1087168 86.09537:1949797 87.09914:264323 91.09866:145234 104.10692:9567680 105.10874:1173202 105.7948:130571 124.9995:376109 145.09148:136445 173.43027:289912 184.06921:3053278 185.07626:403079 204.46875:140514 208.62509:134079 261.91843:153349 | 1.16556E-06 |
| POS12377 | nagitide A | 4.535 | 587.32336 | [M+NH4]+ | 587.32269 | 59.04858:33429 69.03294:308951 73.02811:92996 73.06432:57698 81.03229:36420 83.0477:130652 85.02799:23950 87.04334:596637 89.05896:1689632 90.0619:30548 95.04893:60869 99.04339:492689 107.06821:29161 111.04189:250668 113.05927:37193 117.09089:32262 124.24364:22068 125.0596:122443 129.05446:44234 131.07088:65241 133.08543:647032 137.05981:42742 151.09705:22558 155.07083:154022 173.07771:39173 175.09526:23181 177.11285:43105 433.12009:19764 531.20953:18946 | 1.14077E-06 |
| NEG4961 | -/-)-Apomorphine | 0.986 | 267.12653 | [M-H2O-H]- | 267.12592 | 87.11253:36435 89.13125:15772 93.03925:2156657 95.03763:9071 97.03951:6007 122.80492:5907 151.06712:82487 | 2.28357E-06 |
| POS10145 | 0,13-dimethylhex | 4.565 | 501.2886 | [M+2H]2+ | 501.28799 | 67.05389:10180 69.03294:24014 73.02811:78849 73.06432:11997 81.03229:36478 83.0477:31797 87.04334:139810 89.05896:695122 90.0619:36670 91.04643:15830 95.04893:52681 99.04339:36235 111.04441:88439 113.05927:24005 129.05132:9701 133.08543:208249 134.09018:9543 137.05981:22338 155.07083:22753 177.10779:13785 349.62927:8779 394.30075:8815 | 1.21687E-06 |
| POS8243 | Bacithrocin A 2 | 4.878 | 390.25101 | [M+2H]2+ | 390.2504 | 57.03287:28517 58.04042:61799 59.04859:810843 60.05226:18014 65.04819:32205 69.06992:12328 73.02812:311303 73.06432:37208 80.0543:152726 85.06337:31220 87.04335:212354 87.06079:100912 87.07999:18149 87.56366:9811 89.05896:1659588 90.06191:47156 94.06947:11847 101.05931:59372 102.06606:90634 103.07444:111592 107.07059:17137 109.0756:40923 111.07206:7925 117.0909:9068 118.07944:8454 124.08022:36657 129.08911:21721 131.06766:22242 131.08699:17347 133.08545:518030 134.08684:24749 140.09358:8830 147.10149:50140 162.10718:10227 173.11685:8866 173.39111:15223 177.11285:46600 191.1256:7760 | 1.5631E-06 |
| POS13576 | Gnididilatin | 4.539 | 653.33099 | [M+Na]+ | 653.33191 | 73.06432:16337 87.04335:81017 88.42162:21583 89.05896:508404 90.06375:26002 131.07088:22260 133.08545:261973 134.09018:19703 167.93817:14192 173.32739:16029 177.11285:33825 207.07423:17439 | -1.40817E-06 |
| POS8790 | '-Diapolycopened | 5.995 | 429.27969 | [M+Na]+ | 429.27911 | 67.01499:23138 68.83137:5620 85.02631:6440 97.0256:21060 107.27312:5940 140.07933:9545 156.05841:5539 166.37865:6072 213.10983:6838 251.07872:6323 | 1.3511E-06 |
| POS10243 | ctapropylene glyc | 5.391 | 505.33411 | [M+Na]+ | 505.33472 | 53.72115:6428 59.04856:11800 87.04504:5356 89.05891:18520 106.9778:6131 307.71118:5652 | -1.20712E-06 |
| POS8674 | SCHEMBL9230589 | 5.663 | 421.31528 | [M+H-H2O]+ | 421.31589 | 52.03271:6102 54.90479:5597 57.06893:21297 64.94799:5750 150.03362:6041 173.42535:9497 335.32715:5905 | -1.44784E-06 |
| POS10853 | Lucidamine B | 4.455 | 530.28937 | [M+H]+ | 530.28998 | 70.02779:27691 70.25867:6630 86.0954:72075 87.0538:163077 88.03895:13858 88.05669:7621 89.05895:42678 133.08543:20422 136.39027:6852 144.07225:8666 172.06985:29347 188.07002:17350 331.34097:9057 344.17609:6824 | -1.15031E-06 |
| POS8201 | Sufentanil | 5.612 | 387.21133 | [M+H]+ | 387.21075 | 66.28812:5293 149.63585:5504 | 1.49789E-06 |
| POS6552 | -oxo-3,4,4a,5,6,7- | 1.043 | 301.13873 | [M+H]+ | 301.13931 | 67.55789:6126 74.91763:5349 94.50796:6279 | -1.92602E-06 |
| POS6314 | Epiandrosterone | 5.973 | 291.21527 | [M+2H]2+ | 291.21469 | 57.03287:13229 57.06989:11761 59.04858:15163 94.53361:5705 99.57687:5565 104.10696:6784 208.52167:5827 | 1.99166E-06 |

**Differences in metabolites between the Model group and the WJW group**

| Alignment ID | Metabolite name | Rt(min) | Expreiment Mz | Adduct type | Reference m/z | MS/MS spectrum | PPM |
|---|---|---|---|---|---|---|---|
| POS6126 | )-Isopropylidene u | 8.945 | 285.10867 | [M+2H]2+ | 285.10809 | 50.45882:5582 54.38173:5837 57.06989:9494 108.00944:7526 126.02023:100702 133.29341:6084 230.44301:5340 | 2.03432E-06 |
| POS8349 | ngoramine\|15-Ace | 6.689 | 398.23312 | [M+H]+ | 398.23251 | 50.02518:5903 53.62407:6384 65.30698:4998 67.58175:6532 102.40796:5349 | 1.53177E-06 |
| POS4547 | ky-10-methylunde | 8.399 | 226.21539 | [M+H]+ | 226.216 | 67.77902:6710 87.61996:5760 194.30267:6060 201.20547:6926 | -2.69654E-06 |
| POS3548 | SCHEMBL536577 | 4.957 | 191.12712 | [M+2H]2+ | 191.1277 | 59.04859:20309 89.05896:14193 169.03723:6139 | -3.03462E-06 |
| POS3547 | SCHEMBL536577 | 5.284 | 191.12712 | M+CH3OH+H] | 191.1277 | 59.04859:16667 78.08692:5601 130.06477:33273 131.21602:5188 131.92909:5869 | -3.03462E-06 |
| POS6874 | 4,16-dimethylocta | 5.678 | 314.34061 | [M+H]+ | 314.34119 | 57.06989:29811 58.06417:9153 62.05909:18598 62.34779:5756 67.25955:5549 71.08548:13255 95.08478:6751 162.15594:5457 | -1.84513E-06 |
| POS4798 | Callicarpenal | 5.882 | 235.20441 | [M+H-H2O]+ | 235.205 | 55.05365:12582 57.06896:10346 67.05389:19811 69.06992:6651 81.0699:20363 93.06841:13292 95.08477:16563 107.08486:10424 109.10006:8951 121.1013:6810 133.09863:9261 173.39601:9357 189.166:6362 202.80254:6188 | -2.50845E-06 |
| POS5612 | Vaccenic acid | 0.723 | 265.2514 | [M+H-H2O]+ | 265.25201 | 55.05364:6929 57.06895:7314 64.0822:5705 67.05388:8099 95.08475:7276 128.43639:5448 156.52417:4948 173.39107:8108 | -2.2997E-06 |
| NEG6662 | Neurocil | 1.052 | 327.15433 | [M-H2O-H]- | 327.15369 | 52.07363:6494 93.03928:872312 95.03767:416150 112.81192:5661 148.88708:6056 151.06717:36099 153.0676:44578 211.09386:6706 | 1.95627E-06 |
| POS8523 | Risperidone | 4.471 | 411.22064 | [M+H]+ | 411.22 | 55.05365:17699 59.04858:11397 67.05389:5960 69.03294:127237 73.02811:24347 81.03229:11864 83.0477:60021 87.04334:171809 89.05896:220269 95.04893:19416 99.04339:81065 107.07059:11021 111.04189:87854 113.05927:8752 125.0596:10837 129.05446:12660 131.06766:18400 133.08543:57860 137.05981:5980 151.09705:10247 155.07083:21651 175.09526:6611 293.59796:5616 316.06183:5958 | 1.55634E-06 |
| NEG6606 | HYDROQUINIDINE | 1.056 | 325.15448 | [M-H]- | 325.15384 | 64.7287:5833 89.13125:11698 93.03925:628532 95.03763:54615 97.03745:20711 145.23549:26894 151.06712:32256 224.36078:6202 | 1.9683E-06 |
| POS10614 | 16-Trihydroxygray | 4.36 | 521.2713 | [M+2H]2+ | 521.27197 | 60.04325:56165 84.04481:14779 84.07957:24844 86.0954:20948 86.4154:9828 102.05276:23592 120.0803:36031 126.05366:11459 129.1017:54961 154.04741:11480 189.0878:20782 217.07977:11623 269.75531:9991 | -1.28532E-06 |
| POS9945 | LysoPC 16:2 | 5.8 | 492.30777 | [M+Na]+ | 492.30841 | 59.04859:14322 60.08025:17792 86.09541:33976 89.05896:11013 104.10697:195092 124.99956:13507 184.06931:80539 | -1.3E-06 |
| POS9490 | nolin-1-yl)-17-nor | 5.121 | 469.29675 | [M+H]+ | 469.29611 | 59.04858:36831 87.04334:65710 89.05896:30551 101.0593:18277 | 1.36374E-06 |
| NEG9467 | Sulfluramid | 5.826 | 526.98547 | [M-H]- | 526.9848 | 56.92723:6923 68.16396:5792 79.05373:35127 98.51506:5653 158.53918:7858 168.244:8458 216.10568:5340 | 1.27138E-06 |
| POS14376 | Mycolactone E | 6.036 | 715.5152 | [2M+H]+ | 715.51428 | 54.48004:11175 57.03287:49740 59.04858:412194 115.07642:12631 117.09089:66733 173.3911:19235 191.97964:12295 | 1.28579E-06 |
| POS11920 | Cholylhistidine | 6.474 | 568.33496 | [M+H]+ | 568.33569 | 53.90853:6958 104.10692:24584 130.73308:5764 208.30186:6079 316.26694:5760 355.9762:6039 | -1.28445E-06 |
| POS13730 | kanoyl-NBD Chole | 8.766 | 663.44861 | [M+NH4]+ | 663.44788 | 52.42753:6679 174.16846:5838 276.60065:6166 | 1.10031E-06 |
| POS14754 | PC(32:0) | 5.984 | 756.55035 | [2M+H]+ | 756.55133 | 60.08022:37579 86.09537:90868 124.9995:29152 184.07457:229178 278.94693:9358 | -1.29535E-06 |
| POS5519 | ydroxyvalerylcarni | 3.958 | 262.16397 | [M+H]+ | 262.16461 | 53.30585:6434 55.40891:6406 59.04858:11721 60.08024:13834 61.03869:45354 76.82643:5531 85.02798:102204 110.94395:5711 111.10222:5649 132.5201:5358 196.49069:5692 228.23517:5695 | -2.44121E-06 |
| POS14103 | coumaroyltetracos | 6.817 | 693.47156 | [M+H]+ | 693.47241 | 62.44623:4860 69.6243:6494 87.19528:6082 184.61758:5548 207.8765:6022 248.67805:5908 | -1.22572E-06 |
| POS7459 | nethylpent-4-enyl | 1.21 | 343.33655 | [M+Na]+ | 343.33591 | 55.05453:10758 57.06896:43638 59.03105:230090 61.01003:106223 62.98918:6912 67.05389:14750 69.06869:29415 70.83771:5507 71.08548:18494 73.04555:155497 75.02498:212522 77.00464:11970 77.04093:139452 79.01993:21569 83.0851:22905 85.10046:10091 87.06079:15150 91.05577:139447 93.03564:124450 95.01512:10207 97.09953:20636 105.07177:51413 111.11733:11258 119.08613:18341 120.30954:5504 125.13172:7294 133.10193:9567 252.17599:5722 343.06805:5855 | 1.86406E-06 |
| POS8606 | Epigomisin O | 5.68 | 417.19174 | [2M+H]+ | 417.19241 | 55.01768:174743 57.06894:393506 69.06989:9178 79.05309:16715 83.01192:882887 84.015:11910 85.06333:274529 93.0703:31073 97.06457:46170 101.02218:44803 111.04436:16485 111.07955:46585 115.03928:542901 116.04215:7881 121.06405:26848 137.05975:7557 139.07352:234912 140.07928:11300 149.09476:8913 150.10469:6037 153.09097:10085 157.08499:131414 167.10457:37600 171.09958:352444 172.10374:14078 221.1192:19923 222.12537:16691 247.10213:10619 | -1.60597E-06 |

**Differences in metabolites between the Model group and the WJW group**

| Alignment ID | Metabolite name | Rt(min) | Expreiment Mz | Adduct type | Reference m/z | MS/MS spectrum | PPM |
|---|---|---|---|---|---|---|---|
| POS13381 | deferrioxamine E | 4.942 | 639.39178 | [M+NH4]+ | 639.39093 | 51.69823:15774 52.24539:16019 57.26204:16845 57.49446:15405 63.10206:14911 64.38737:17272 66.84683:15535 67.72396:16179 69.98:15275 70.89279:14630 72.48169:14052 81.48377:15286 83.18766:14148 85.52522:14213 106.00784:15084 116.15504:19389 119.128:16230 126.05975:15786 127.80418:13953 130.4415:14237 131.46817:16566 137.12877:14484 145.22301:15836 146.56651:14491 148.21899:17379 149.40419:14346 159.72244:14941 172.44861:17416 181.18074:19832 183.76932:19344 193.33499:17767 198.01981:17442 216.79158:16968 219.92371:18493 251.43799:14919 278.60718:15006 294.77921:16229 316.50882:18242 316.65399:16131 321.06827:17036 323.32883:17358 373.3949:20685 379.43304:18899 503.52267:15397 504.54337:16705 548.30841:15379 584.229:16901 | 1.32939E-06 |
| POS8548 | Arg His Thr | 4.42 | 413.22482 | [M+H]+ | 413.22549 | 59.04859:17241 70.06431:7509 72.08048:8153 87.04335:7051 89.05896:45647 103.07444:10459 118.06566:25692 132.07892:8927 133.08545:19008 144.0797:18346 146.061:92134 151.09705:6329 159.09138:12523 188.07004:310236 189.07106:20113 205.09822:54513 209.13737:7734 363.82065:6967 | -1.62139E-06 |
| POS2769 | Methylglutaconic a | 0.946 | 167.03081 | M+CH3OH+H] | 167.03148 | 51.87749:7152 53.48768:5939 141.06718:5550 | -4.01122E-06 |
| POS15254 | 15Z)/22:6(4Z,7Z,10 | 8.757 | 828.55261 | [M+H]+ | 828.55371 | 64.80882:5376 90.13166:5540 90.14452:6178 | -1.32761E-06 |
| POS5062 | Aspergilliamide B | 4.38 | 245.14882 | [M+NH4]+ | 245.14951 | 50.74328:5710 60.53485:5917 68.04947:19806 69.06865:9987 70.06427:6873 84.08119:7743 85.02796:14854 86.05935:80919 86.09537:175775 132.06581:129317 176.54716:5792 | -2.81461E-06 |
| POS7143 | earoylethanolamid | 7.516 | 328.31934 | [M+H]+ | 328.32001 | 74.28384:5185 83.54238:5423 168.10655:5885 292.4346:6312 | -2.04069E-06 |
| POS474 | 5Z)-1,3,5-Heptatri | 1.206 | 95.08541 | [M+H-H2O]+ | 95.08607 | 55.05365:31397 65.03804:11693 67.05389:46139 81.05109:6681 94.06358:28792 95.04893:9914 95.08476:12078 | -6.94108E-06 |
| POS4088 | Ethirimol | 7.201 | 209.15213 | [M+H]+ | 209.1528 | 62.28967:5904 95.08477:7367 156.20926:5810 | -3.2034E-06 |
| POS5715 | Vitamin A | 7.047 | 269.22433 | [M+H]+ | 269.22501 | 67.57101:5925 97.04408:5224 120.90405:6182 173.43034:10681 | -2.52577E-06 |
| POS5489 | Leucyl-Glutamate | 2.399 | 261.14291 | [M+H]+ | 261.14359 | 58.06512:6880 61.0387:112801 90.77464:6157 148.89172:5542 167.25319:5439 215.13528:10629 | -2.60393E-06 |
| POS1888 | methylthio)methyl | 9.436 | 140.98665 | [M+H]+ | 140.98599 | 54.94678:16057 55.0177:7569 55.05365:10468 55.93345:120086 55.94513:11345 56.9414:107108 57.9342:29721 58.06511:11469 67.05389:25965 67.93391:10623 70.0643:12048 70.94153:9884 72.93706:54805 90.94762:10952 94.06358:21162 95.04694:7073 95.08476:17165 98.98203:6037 111.96739:9636 113.96311:43964 114.97044:33110 116.97124:22432 131.97466:8216 | 4.68132E-06 |
| POS7891 | JNII-5BCD1RO66X | 4.87 | 368.23901 | [M+2H]2+ | 368.23831 | 57.03287:32946 58.04042:81847 59.04859:845961 60.05226:21212 65.04819:47242 69.06869:8507 71.04816:9491 73.02812:288399 73.06432:31626 80.0543:109304 80.55504:8430 85.06337:33913 87.04335:206760 87.06079:92957 87.07999:13240 89.05896:1404834 90.06191:46269 94.06947:13907 101.05931:73293 102.06606:60752 103.07444:122821 107.07059:12879 109.07315:40397 111.07206:7955 115.07378:9574 117.05281:7930 124.08022:18934 129.08911:15292 131.06766:20340 133.08545:402344 134.09018:21942 147.10149:42058 177.11285:31739 191.1256:9253 | 1.90094E-06 |
| POS10817 | Pro Lys Lys Val Gly | 5.236 | 528.34955 | [M+2H]2+ | 528.35028 | 57.03378:52435 59.04858:942759 60.05225:24259 73.02811:90964 73.06431:42004 80.05428:17471 85.06504:26185 87.04333:285945 87.06252:45317 87.07997:25489 88.04604:21069 89.05894:1175868 90.0619:30232 94.06945:36619 101.05929:186395 102.06604:24382 103.07442:277642 107.07057:16114 109.07558:18349 129.0891:30027 133.08542:384601 134.08682:17608 147.10147:95883 161.11877:16088 171.94873:14308 177.11282:23848 191.12556:20051 219.33647:14791 | -1.38166E-06 |
| POS11452 | A 108836 | 4.929 | 551.33923 | [M+H]+ | 551.34003 | 59.04859:39169 63.23145:11485 73.02812:16131 74.31548:11144 80.0543:16151 87.04335:26564 89.05896:154180 104.10697:19400 133.08545:72250 173.38622:14575 495.42001:11316 | -1.45101E-06 |
| POS7519 | reonylalanylargini | 4.648 | 347.203 | [M+Na]+ | 347.2037 | 60.6027:5934 80.86337:6667 89.05896:7305 92.63606:5017 173.39111:15290 | -2.01611E-06 |
| POS10834 | methylhexadecahy | 8.806 | 529.32574 | [M+NH4]2+ | 529.32501 | 60.08025:7840 63.63507:6441 86.09541:16018 104.10697:162165 105.11111:9431 132.62503:6264 | 1.37911E-06 |

| Differences in metabolites between the Model group and the WJW group | | | | | | | |
|---|---|---|---|---|---|---|---|
| Alignment ID | Metabolite name | Rt(min) | Expreiment Mz | Adduct type | Reference m/z | MS/MS spectrum | PPM |
| POS10169 | Ala Leu Leu Ala Se | 5.369 | 502.32269 | [M+2H]2+ | 502.32339 | 57.03287:20505 59.04859:225785 60.05226:8017 67.015:26429 73.02812:7445 73.06432:10946 78.27695:5333 81.03072:15251 85.06506:7810 87.04335:27628 87.07999:12120 89.05896:175188 90.06374:7204 91.40009:5270 101.05931:14949 103.07444:66788 117.05281:5730 117.0909:6618 131.06766:5493 133.08545:48132 147.10149:28884 158.09198:14257 165.0956:8004 177.11285:8119 180.10141:7485 | -1.39352E-06 |
| POS11695 | Stellettacholine A | 4.67 | 560.33539 | [M+2H]2+ | 560.33459 | 69.03294:15177 73.02811:35386 73.06432:11459 80.05429:14448 87.04334:85223 88.04604:6778 89.05896:404118 90.0619:15946 91.04643:14813 95.04893:11870 102.06606:13250 111.04189:9243 113.05927:20331 131.06766:11306 133.08543:167499 134.09018:9578 137.0598:8958 155.06668:6020 177.11285:24578 190.08008:7159 223.32474:6305 | 1.42772E-06 |
| POS14395 | ulomo'opunalide- | 5.697 | 717.47858 | [2M+H]2+ | 717.47961 | 57.03287:19891 59.04859:221207 73.02812:35528 73.06432:17061 85.06337:12306 87.04335:77540 87.06079:20207 89.05896:270835 94.06947:13494 101.05931:63349 103.07444:78962 129.08911:11617 131.10634:12235 133.08545:89934 147.10149:47843 396.90311:11015 669.2359:11689 | -1.43558E-06 |
| NEG4967 | )-6-octadecen-5- | 1.74 | 267.26871 | [M-H]- | 267.26941 | 97.07645:17103 99.07355:76917 122.14449:23477 126.07927:8655 135.1936:9014 165.20354:11966 183.59097:6420 | -2.61908E-06 |
| NEG4606 | Tripelennamine | 2.717 | 254.16559 | [M-H]- | 254.16631 | 106.66814:6352 179.69743:5091 182.80327:5982 | -2.83279E-06 |
| POS13938 | (28),23-dien-3-ol | 8.648 | 679.47693 | [M+2H]2+ | 679.47791 | 155.16624:5615 316.72656:5510 389.63431:6421 | -1.44228E-06 |
| POS13533 | -en-28-ol\|3??-trar | 6.826 | 649.44531 | [M+H]+ | 649.44623 | 55.45766:7692 66.63255:7260 66.88206:5923 91.21459:5691 110.99668:6065 130.05522:6720 291.0647:5703 517.97321:7532 | -1.41659E-06 |
| POS6778 | ythro-Sphingosine | 7.672 | 310.30829 | [M+H]+ | 310.30899 | 50.68978:8626 276.54135:5621 | -2.25582E-06 |
| POS12710 | ne-3alpha,29-dio | 6.299 | 603.40521 | [M+Na]+ | 603.4043 | 295.258:5816 | 1.50811E-06 |
| POS12993 | lene-4-(2-methyl | 4.941 | 617.40582 | [M+H]+ | 617.40491 | 57.03287:111732 59.04859:2946029 73.02812:137628 73.06432:76402 85.06337:118003 87.04335:783200 87.07999:105717 89.05896:4837258 90.06191:77426 101.05931:594982 103.03849:81983 103.07444:1201891 115.07378:47468 117.05281:77075 117.0909:61153 129.08911:124756 131.06766:120960 131.10634:77159 133.08545:1871124 134.09018:72977 145.08403:98662 147.10149:957866 161.11441:76372 177.11285:189428 191.1256:146797 304.17166:44505 | 1.47391E-06 |
| POS8016 | Leucocrystal Violet | 5.293 | 374.25827 | [M+2H]2+ | 374.259 | 57.03287:93167 59.04859:2046332 60.05226:51596 65.04819:14339 69.06992:11142 73.02812:116829 73.06432:24672 81.0699:9899 85.06337:32496 87.04335:91908 87.06254:28085 87.07999:22968 89.05896:688187 90.06191:16894 94.06947:42386 99.07939:11622 101.05931:108683 103.07444:305256 104.07732:10915 115.07378:16776 116.08248:17429 117.05281:13299 117.0909:22477 129.08911:17999 131.07088:14247 133.08545:155401 143.1078:10089 147.10149:123765 161.11441:10648 191.1256:11440 | -1.95052E-06 |
| POS10667 | schiprolactone A | 4.747 | 523.30438 | [M+H]+ | 523.30359 | 60.08025:21171 86.09541:53744 87.04335:5583 87.49507:7251 89.05896:20147 104.10697:262831 133.08545:6003 142.02203:6853 184.07466:122621 | 1.50964E-06 |
| POS8668 | Erythrophloin A | 4.606 | 421.23813 | [M+Na]+ | 421.2374 | 89.05896:20748 118.57424:5939 139.98682:6236 173.43526:6276 | 1.73299E-06 |
| POS10171 | LysoPC(P-16:0/0:0 | 6.253 | 502.32748 | [M+H-H2O]+ | 502.32672 | 62.06014:11393 68.02178:6959 86.09712:10834 318.39163:5746 | 1.51296E-06 |
| POS13012 | Veralosine | 5.804 | 618.40094 | [M+2H]2+ | 618.40002 | 57.03287:16195 59.04859:82528 60.16235:11541 70.74816:10102 73.02812:15396 73.06432:12980 87.04335:28027 89.05896:96925 101.05931:29693 103.07445:22539 129.08911:12432 133.08545:33699 135.46794:9780 147.10149:14249 173.38132:13274 196.63869:10859 | 1.48771E-06 |
| POS4339 | nma-Glutamylalar | 1.483 | 219.09666 | [M+H]+ | 219.0974 | 56.0495:7304 83.04604:5690 84.04313:118986 84.07954:10805 85.02796:8517 90.05453:44206 128.0714:7891 130.0488:15023 156.06255:19326 170.79237:5928 | -3.37749E-06 |
| NEG1779 | Pelargonic acid | 1.51 | 157.20074 | [M-H]- | 157.2 | 69.08023:6205 79.71713:5218 89.13125:312981 90.1367:6657 97.07645:7737 113.19607:6950 115.14149:7846 141.94867:5416 144.71094:5617 147.59695:5893 | 4.70738E-06 |
| POS13290 | n-3alpha-yl-O-be | 4.616 | 633.3642 | [M+2H]2+ | 633.36322 | 67.05389:62726 69.03294:62783 73.02811:263251 73.06432:93764 81.03229:85335 83.0477:79886 87.04334:608498 89.05896:3244339 90.0619:123708 95.04893:174189 99.04339:100212 111.04189:266453 113.05927:113170 113.1652:62702 133.08543:1007164 137.0598:88154 | 1.5473E-06 |
| POS8382 | Cryptomaldamide | 5.19 | 400.25473 | [M+2H]2+ | 400.25549 | 59.04858:46073 67.015:6651 87.04334:8280 89.05896:46653 101.0593:5604 103.07444:10843 127.95014:5198 133.08545:11379 262.60263:6014 307.50278:7369 335.51199:6585 | -1.89879E-06 |

| Differences in metabolites between the Model group and the WJW group | | | | | | | |
|---|---|---|---|---|---|---|---|
| Alignment ID | Metabolite name | Rt(min) | Expreiment Mz | Adduct type | Reference m/z | MS/MS spectrum | PPM |
| POS8644 | Roxithromycin | 4.963 | 419.27377 | [2M+H]2+ | 419.27301 | 57.03287:36771 58.04042:44168 59.04859:1046584 60.05226:24137 65.04819:44839 67.05389:10654 69.06869:13857 73.02812:213131 73.06432:37940 80.0543:98672 85.06337:40000 87.04335:222274 87.06079:104870 87.07999:21985 89.05896:1663653 90.06191:45792 94.06947:14388 101.05931:74030 102.06606:50977 103.07444:168670 104.0796:10383 107.06821:13701 109.07315:62752 116.0798:9014 117.0909:12747 124.07726:21506 129.08911:25685 131.06766:24749 133.08545:543876 134.08684:29226 138.09604:10695 140.09358:12090 147.10149:69791 153.09918:12457 177.11285:48734 191.1256:13042 | 1.81266E-06 |
| POS11613 | CHEBI:69627 | 5.196 | 557.38458 | [M+H]+ | 557.38373 | 57.03287:68192 59.04859:1426531 60.05226:22336 69.06868:16224 73.02812:30907 73.06432:66693 85.06506:16565 87.04335:101496 87.07999:14345 89.05896:331344 101.05931:133579 103.07444:374703 115.07378:27471 117.05281:73537 117.0909:59987 131.07088:55951 133.08545:42112 147.10149:77117 161.11441:46865 | 1.52498E-06 |
| POS8199 | Alstovine | 5.725 | 387.19226 | M+CH3OH+H] | 387.1915 | 51.54529:6444 51.69024:6817 55.05365:6559 57.06896:36001 59.04858:6559 71.04816:33403 74.83275:5981 83.0477:43632 101.0593:21270 173.43034:13159 175.01567:53935 211.91226:5915 214.52664:6403 | 1.96285E-06 |
| POS12628 | Coriandrinol | 5.713 | 599.42914 | [M+H]+ | 599.42822 | 57.03286:45848 59.04858:470607 67.46854:7742 69.06991:8637 70.37264:7815 70.79802:7299 73.02811:19200 87.04334:21683 89.05895:85190 91.19027:8508 99.07938:9185 101.0593:21007 103.07443:21528 115.07377:11938 117.09089:73108 133.08543:18864 147.10147:8020 155.0584:7607 173.3911:16437 200.63062:7164 480.31015:8204 563.1051:7694 | 1.5348E-06 |
| POS13032 | 7',8'-Tetradehydro | 6.297 | 619.37714 | [M+H]+ | 619.37811 | 51.86788:5622 530.44775:5263 | -1.56609E-06 |
| POS13958 | cheilocline F | 5.492 | 681.45233 | [M+2H]2+ | 681.45129 | 59.04859:306302 73.02812:48139 87.04335:65890 89.05896:393891 101.05931:62129 103.07445:79381 129.08911:27142 133.08545:134914 147.10149:48421 147.89014:27807 173.43526:39266 | 1.52615E-06 |
| POS5228 | Deoxyadenosine | 4.68 | 251.10112 | [M+NH4]+ | 251.10181 | 59.04858:52639 60.04426:70014 69.06992:10529 70.02779:77145 74.02328:15442 87.04334:14689 88.03896:207029 94.06359:180511 95.06884:6101 98.02254:9480 99.00742:32831 104.04768:16240 107.43317:5637 116.03146:11602 117.05281:8812 118.06566:5322 120.04358:56815 130.04884:28260 132.04306:17934 136.07611:13891 146.05721:230585 147.06317:13812 150.05342:17259 158.04503:14454 163.19865:6458 173.38622:10070 174.05498:45442 177.6405:5237 192.06537:12410 | -2.74789E-06 |
| POS10530 | Thr Thr Gly Leu Ile | 6.514 | 518.31921 | [M+Na]+ | 518.31842 | 59.04858:12918 60.10926:5981 62.53428:5966 76.55672:6150 80.40308:5481 86.09541:25027 146.97894:35421 331.8078:5767 361.46249:5952 | 1.52416E-06 |
| POS7961 | Val-Arg-Pro | 5.612 | 371.23929 | [M+H]+ | 371.24011 | 52.73953:5807 59.03397:5750 193.61818:5194 329.18835:6615 371.23489:8925 | -2.20881E-06 |
| POS8135 | -methoxyphenyl}- | 1.034 | 383.11487 | [M+H]+ | 383.11569 | 53.2382:5895 53.43983:6521 82.94374:5768 203.05078:79829 238.1199:5854 | -2.14035E-06 |
| POS7819 | 1-epoxy-9-fluoro | 4.505 | 363.1973 | [M+Na]+ | 363.1965 | 173.39111:8444 281.01971:6255 289.36584:6283 363.1955:12277 | 2.20266E-06 |
| POS7551 | Arg Ser Ser | 4.509 | 349.1821 | [M+Na]+ | 349.18289 | 62.11793:5892 67.20391:5960 70.06431:10778 86.05939:8665 165.3967:6089 166.37866:6413 173.43036:6489 | -2.26242E-06 |
| POS11797 | -yl)-1H-cyclopent | 5.556 | 563.37207 | [M+Na]+ | 563.37299 | 57.03287:9100 59.04858:24525 66.65124:6273 72.53472:7669 87.04334:11665 89.05896:33263 103.07444:7267 104.10696:14628 104.63825:5839 133.08543:8499 141.77881:5799 165.37384:6538 184.06929:7804 344.60162:5601 350.31827:6336 | -1.63302E-06 |
| POS14256 | ta-D-glucopyranc | 4.955 | 705.45593 | [M+H]+ | 705.45709 | 59.04858:88066 73.02811:7844 87.04334:40200 89.05895:236352 101.0593:25592 103.07443:34789 133.08543:94486 147.10147:21517 156.65466:6609 177.11284:12295 184.07465:10663 399.92606:7569 | -1.64432E-06 |
| POS12084 | Sevcorine | 5.201 | 574.41101 | [M+H]+ | 574.4101 | 59.04859:415155 73.02812:15506 73.06432:81514 87.04335:36123 89.05896:199439 101.05931:36242 103.07445:124824 117.05281:154756 117.0909:25395 131.06766:75721 133.08545:53150 147.10149:37025 | 1.58423E-06 |
| NEG4249 | ethachlor CGA369 | 1.208 | 242.05011 | [M-H]- | 242.0493 | 59.53994:5499 62.06197:10364 71.2459:6079 79.05367:23326 83.19241:5742 97.08672:5531 161.03622:21782 163.03287:17835 | 3.34643E-06 |
| NEG1775 | de, cyclopentyl iso | 1.259 | 157.1049 | [M-H2O-H]- | 157.1057 | 59.09475:28573 71.10927:39130 72.09502:6311 79.05367:106869 97.07645:129535 97.11958:205100 99.07355:13615 114.16649:92565 136.50891:5947 159.91479:5870 | -5.09211E-06 |
| POS6605 | -hydroxy-6-methy | 0.755 | 303.02576 | [M+H]+ | 303.02655 | 53.82195:5647 61.97727:6437 128.94746:5700 132.68735:5070 173.69058:6055 218.98808:8075 236.06581:6082 | -2.60703E-06 |

| Differences in metabolites between the Model group and the WJW group | | | | | | | |
|---|---|---|---|---|---|---|---|
| Alignment ID | Metabolite name | Rt(min) | Expreiment Mz | Adduct type | Reference m/z | MS/MS spectrum | PPM |
| POS5650 | eta-D-glucopyrand | 4.483 | 267.14297 | [M+H-H2O]+ | 267.1438 | 59.04859:209303 71.04816:7917 73.02812:13696 73.06432:9664 77.05981:7144 87.04335:97005 88.21841:5677 89.05896:187607 101.05931:15789 103.03849:332742 103.07444:17763 104.04312:7029 110.05836:6470 117.05281:11657 119.06938:7902 133.08545:20337 165.7536:5287 173.42545:10359 266.04507:5893 | -3.10694E-06 |
| POS4180 | thyl-1H-benzimid | 0.758 | 213.02388 | [M+Na]+ | 213.0247 | 86.99277:10620 94.43892:6952 105.0024:12319 180.54861:5459 185.02766:11004 | -3.84932E-06 |
| POS6244 | Arginylasparagine | 4.53 | 289.16101 | [M+Na]+ | 289.1618 | 51.45718:6340 70.02779:12580 86.09541:38435 88.03896:7095 156.97519:5914 163.43175:5297 173.39111:5979 232.69829:5819 289.1651:48759 | -2.73203E-06 |
| POS2582 | C)OC(C(CC(C)O)C) | 5.496 | 161.11629 | M+CH3OH+H] | 161.1171 | 67.86302:6189 123.64797:5879 | -5.0274E-06 |
| POS938 | alpha-monochlo | 5.925 | 111.01991 | [2M+H]+ | 111.0207 | 55.04137:9377 55.93435:9040 57.9342:170588 72.93707:68652 74.93713:31886 76.49633:11064 77.99808:37110 78.99881:31105 84.94386:26990 85.50144:9992 87.00324:147205 87.50562:7534 88.00348:752935 88.50382:34626 89.00123:11667 89.50648:7362 90.94763:19535 92.94897:8194 95.01114:7544 97.00918:268649 97.50975:14659 98.50998:23719 98.96088:19921 99.51076:148819 100.01365:13214 102.95543:19751 106.01486:9065 108.51749:20082 108.95825:15304 111.01928:11217 113.96312:112127 | -7.11579E-06 |
| POS4237 | Citric acid | 1.01 | 215.01515 | [M+H]+ | 215.01601 | 70.0643:6076 96.92099:12553 98.9186:10567 110.9314:6271 131.91931:6299 134.01683:6754 | -3.9997E-06 |
| POS10447 | Hennoxazole A | 8.804 | 515.31238 | [M+NH4]2+ | 515.31152 | 57.06894:18004 60.08023:21856 75.85378:9829 84.26364:10607 86.09538:66938 104.10693:599104 105.10875:29289 112.04374:14156 173.38615:21450 183.12358:10484 312.70972:11045 | 1.66889E-06 |
| POS10242 | Eudesobovatol A | 5.401 | 505.33035 | [M+Na]+ | 505.33121 | 59.04858:22005 60.51869:6391 67.54954:5982 87.04334:9228 89.05896:22581 133.08543:7357 370.6825:5931 | -1.70185E-06 |
| POS13649 | )-alpha-D-acetylri | 5.318 | 659.45282 | [M+H]+ | 659.45172 | 57.03379:66876 59.04956:789822 73.06432:41776 83.54895:27459 87.04335:72894 89.05896:328663 101.05931:96236 103.07444:238795 115.07378:26832 117.05553:55923 131.07088:29420 133.08545:57155 147.10149:64623 161.11441:26692 190.06322:24163 | 1.66805E-06 |
| POS12888 | dyl pseudopteran | 5.672 | 611.39319 | [M+2H]2+ | 611.39423 | 57.03286:18838 59.04858:107939 73.02811:18743 85.06504:14420 87.04333:15714 89.05894:100135 101.05929:24600 103.07442:41330 133.08542:26485 147.10147:14782 160.2352:10512 | -1.70103E-06 |
| NEG4833 | (-)-Epipolasin A | 0.488 | 262.16446 | [M-H]- | 262.1636 | 84.25964:5170 100.0459:11700 153.12859:5074 | 3.28039E-06 |
| POS12675 | olestane-3beta,6a | 5.323 | 601.39343 | [M+2H]2+ | 601.39447 | 57.03287:54435 59.04859:859064 60.05226:35649 73.02812:102762 73.06432:50234 85.06337:38465 87.04335:181364 87.06254:44298 87.07999:44771 89.05896:1238278 90.06374:36665 101.05931:153091 102.06606:21323 103.07444:270837 109.0756:24811 115.07378:19880 117.0909:34380 129.08911:28073 131.06766:23646 133.08545:446052 134.09018:29466 147.10149:143483 177.11285:44140 191.1256:28717 307.53751:16936 | -1.72931E-06 |
| POS11521 | His Ile Lys Arg | 5.463 | 553.35583 | [M+2H]2+ | 553.35681 | 59.04859:215099 61.68283:11550 61.80683:13336 67.015:20031 73.02812:24862 73.06432:19312 87.04335:31828 87.07999:13960 89.05896:199596 101.05931:40023 103.07444:60091 104.10697:50848 133.08545:65203 147.10149:36903 158.09198:12769 184.07466:16179 314.30731:14461 | -1.77101E-06 |
| NEG2746 | Diethyl L-malate | 1.319 | 189.08087 | [M-H]- | 189.08 | 73.0612:12532 109.03119:7235 125.04041:6868 145.13034:14383 189.08273:7862 | 4.60123E-06 |
| POS4736 | SCHEMBL4748090 | 5.798 | 233.17374 | [2M+H]+ | 233.17461 | 55.24187:5609 57.03287:17170 59.04858:21868 | -3.73111E-06 |
| POS11284 | anine-3beta,17,23 | 6.111 | 546.34155 | [M+Na]+ | 546.34253 | 86.0954:20507 93.39707:5656 97.71894:5703 104.10696:40334 105.10878:7465 184.06927:20814 273.52429:5481 315.59171:5348 340.05838:6352 | -1.79375E-06 |
| POS12054 | a-Hydroxymilbem | 4.867 | 573.33826 | [2M+H]+ | 573.33929 | 57.03287:19053 59.04859:212199 73.02812:12020 73.06432:7762 87.04335:295959 87.07999:9202 89.05896:269303 101.05931:92747 103.03849:134737 103.07444:61656 104.3103:7845 115.07378:9094 117.05281:35589 131.06766:13682 131.87703:8857 133.08545:86616 147.10149:33234 173.43036:10327 177.1078:10853 | -1.79649E-06 |
| POS3342 | phohydroxypyruvi | 8.789 | 184.9854 | [M+NH4]+ | 184.9845 | 55.8742:7477 57.9342:157519 58.9425:411639 59.94047:8544 75.94469:93117 76.95241:32865 84.94554:57664 86.0954:11358 93.95591:18053 98.96088:211388 99.96855:133494 100.96555:15784 102.95542:60805 114.95721:18958 115.96169:43297 116.97124:1264344 117.97753:1428314 120.96403:7764 125.97163:17274 126.96738:39663 127.975:18234 130.98712:9043 133.97353:7244 135.99091:9156 139.9868:162464 140.9953:104599 143.95718:16779 161.96983:30649 | 4.86527E-06 |
| POS7398 | tadecenyl)-1,3-be | 6.602 | 341.246 | [M+H]+ | 341.24512 | 82.54128:5672 175.02563:6388 305.6582:5038 | 2.57879E-06 |

| Differences in metabolites between the Model group and the WJW group | | | | | | | |
|---|---|---|---|---|---|---|---|
| Alignment ID | Metabolite name | Rt(min) | Expreiment Mz | Adduct type | Reference m/z | MS/MS spectrum | PPM |
| POS7334 | Istamycin AO | 4.561 | 338.19119 | [M+NH4]2+ | 338.19211 | 55.05365:49468 58.04137:31543 59.04858:61426 61.02845:24162 67.05389:26368 69.03294:776238 70.03661:25734 71.04816:22373 73.02811:184930 73.06432:46091 78.03804:83888 80.05429:48495 81.03229:32872 81.0699:20190 83.0477:151707 85.02799:22720 87.04334:640153 88.04604:21083 89.05896:1486336 90.0619:42664 95.04893:39479 97.06461:14050 99.04339:321315 100.05018:94695 101.0593:10558 102.06606:15836 103.03849:8502 107.07059:20344 109.06336:23678 111.04189:174394 112.04632:10697 113.05927:31595 117.09089:10199 122.06293:140023 122.56553:18337 125.0596:42861 129.05446:59089 131.07088:37711 133.08543:289594 134.09018:15252 137.05981:20468 144.07597:41692 151.09305:14173 155.07083:64386 166.08865:9512 173.07771:20122 175.09526:10805 177.11285:16091 | -2.72035E-06 |
| POS9493 | Simplexin B | 5.384 | 469.31497 | [M+2H]2+ | 469.31589 | 57.03287:117896 59.04859:2300633 59.05931:59214 60.05226:60339 65.04931:24666 69.06868:28764 73.02812:140915 73.06432:49915 83.0851:15798 85.06337:48771 87.04335:271250 87.06254:50152 87.07999:61435 89.05896:1297080 90.06191:46940 94.06947:73494 99.07939:11795 101.05931:263789 101.09641:11767 103.03849:20538 103.07444:482551 104.07732:28994 105.09028:13361 107.06821:11344 109.07315:13916 115.07378:41262 116.08248:19653 117.05281:23109 117.0909:50237 127.07501:11211 129.08911:37463 130.08708:12384 131.07088:29078 131.10634:18719 133.08545:433862 134.09018:23569 145.08403:24398 147.10149:244411 148.10667:9928 161.1188:28471 177.11285:37578 191.1256:32772 205.14238:12414 | -1.9603E-06 |
| POS11697 | Arg Thr Lys Arg | 4.996 | 560.36365 | [M+H]+ | 560.36261 | 59.04856:97321 73.02808:7807 73.06429:9247 76.54948:6580 87.0433:167181 89.05891:92453 101.05926:51732 103.03844:19276 103.07439:28034 117.05275:17101 131.0676:11931 133.08537:28163 145.85263:6712 177.02168:6394 | 1.85594E-06 |
| NEG8213 | Kazinol F | 0.992 | 395.22391 | [M-H2O-H]- | 395.22299 | 65.39328:5279 67.67252:5287 80.98167:5345 87.53817:5223 89.13125:10868 93.03925:321897 95.03763:159660 97.03745:18025 101.79763:5129 147.15915:8848 151.06712:23521 153.06755:21269 186.0121:5720 208.17513:5711 333.99783:5874 | 2.3278E-06 |
| POS9687 | PC-M5' | 4.896 | 480.27539 | [M+H]+ | 480.27448 | 53.64094:6458 65.62093:6215 126.02025:18856 150.00212:6125 | 1.89475E-06 |
| POS6422 | Pizotifen | 5.074 | 296.1456 | [M+2H]2+ | 296.14651 | 55.05364:16814 57.06895:174141 58.06415:8749 59.04857:8589 59.07294:6187 60.08023:13534 67.05388:33682 67.2394:5981 68.82522:5669 69.0699:22609 71.08546:90873 75.26418:6891 78.54684:5994 81.06831:35008 83.08508:24588 85.10043:38935 86.09538:38307 95.08475:60267 96.01749:5656 97.09951:13160 104.10694:37141 109.10004:25526 114.91485:6385 156.51996:8986 245.69836:5553 256.00311:7771 290.06473:6318 | -3.0728E-06 |
| POS6860 | anthraniloyl-beta- | 1.621 | 314.12149 | [M+H]+ | 314.12241 | 51.39856:6408 54.99667:6656 57.06988:8968 91.59369:6293 121.06409:133554 | -2.92879E-06 |
| POS174 | Dichloromethane | 0.763 | 84.95968 | [M+H]+ | 84.9606 | 56.04863:22764 56.96448:23144 59.79324:6337 84.04316:16629 | -1.08285E-05 |
| POS175 | Dichloromethane | 4.25 | 84.95968 | [M+H-H2O]+ | 84.9606 | 54.61502:5320 56.04863:26433 56.091:6003 79.57497:5269 84.04482:11852 | -1.08285E-05 |
| POS5611 | Vaccenic acid | 7.137 | 265.2511 | [M+H]+ | 265.25201 | 61.67659:5453 84.07957:6347 111.40971:6016 114.95457:6407 173.39601:5858 201.17491:5557 | -3.4307E-06 |
| POS7377 | Bursin | 4.589 | 340.20816 | [M+H]2+ | 340.20911 | 58.04137:707509 69.03294:21970 71.04816:62924 73.02811:1839392 73.06432:132891 74.03149:43183 80.05429:962667 80.55504:76281 87.04334:361428 89.05896:3789226 90.0619:124674 102.06606:490082 102.56839:53624 107.07058:26618 111.07206:64520 115.07378:31855 117.09089:22842 124.08022:176716 131.07088:41564 133.08543:1136328 134.09018:34958 146.09511:56660 155.09987:31976 177.11285:87227 | -2.7924E-06 |
| POS4947 | -(4-acetylamino-b | 4.534 | 241.20316 | [M+2H]2+ | 241.20219 | 55.05365:7736 57.06896:68681 58.06416:2763218 59.06808:89643 67.05389:63876 69.06868:17389 79.05312:24329 81.06833:379873 82.07301:25212 84.07957:123835 85.06337:14082 95.08477:49800 121.09843:11305 123.11523:306716 124.11882:29187 138.54323:7262 140.14345:69996 | 4.02152E-06 |
| POS6840 | Thymol glucoside | 5.258 | 313.16284 | M+CH3OH+H] | 313.16379 | 55.01768:11829 55.05363:64392 57.06894:284785 69.06865:11020 71.01213:23866 81.0683:8650 83.01192:82153 83.04766:774122 84.0514:19074 85.06333:140535 97.06457:7544 99.00737:9122 111.08207:8130 115.03662:36950 127.07495:50870 137.05975:33459 139.07352:23369 155.07077:65777 157.08499:18404 159.09993:94667 171.09958:17877 179.06763:18703 197.07755:10811 | -3.03356E-06 |
| POS6395 | purin-6-yl)carbamo | 5.751 | 295.11584 | [2M+H]+ | 295.1149 | 55.01682:6428 57.06895:13921 59.04858:10698 67.05389:10906 73.02811:6042 78.6007:4934 81.06989:8065 83.08508:6707 85.02798:8604 93.07033:6373 95.08277:8775 97.09952:5395 104.02032:5922 105.06944:5813 133.06235:21022 163.47661:5188 179.13976:17382 | 3.1852E-06 |

**Differences in metabolites between the Model group and the WJW group**

| Alignment ID | Metabolite name | Rt(min) | Expreiment Mz | Adduct type | Reference m/z | MS/MS spectrum | PPM |
|---|---|---|---|---|---|---|---|
| POS3043 | uanidinosuccinic ac | 1.091 | 176.06526 | [M+H]+ | 176.06621 | 60.05523:190390 70.06429:845409 71.04814:19765 71.06744:54596 72.08046:24009 84.08121:8979 112.08705:8669 113.06956:61703 114.05197:10024 116.06902:51195 117.07182:6868 130.0966:17345 159.0741:9593 175.12009:8320 | -5.3957E-06 |
| NEG3943 | dehydroschoberine | 0.757 | 231.18767 | [M-H]- | 231.18671 | 67.90976:5778 97.15044:6255 127.79837:6022 | 4.15249E-06 |
| POS14929 | Oligomycin C | 8.719 | 775.53864 | [M+NH4]+ | 775.5354 | 78.02028:7026 78.65312:6033 79.51556:6056 82.88864:7619 100.9307:4764 111.60696:5977 130.14764:5777 221.2182:5478 306.94766:6098 353.1517:5441 752.54596:6685 | 4.17776E-06 |
| NEG9405 | 6,7,7,8,8-hexadeca | 5.174 | 514.90167 | [M-H]- | 514.9007 | 80.47415:6165 84.43423:5234 106.70364:5642 143.62971:5224 | 1.88386E-06 |
| POS4498 | SCHEMBL2268896 | 2.598 | 225.05943 | [M+H]+ | 225.06039 | 58.06512:12460 74.39811:5599 79.05313:11946 91.0539:9945 93.06841:25297 115.93221:5744 121.06696:6799 123.70115:5058 163.1002:5060 227.04024:10378 | -4.26552E-06 |
| POS7795 | serylvalylarginine | 4.769 | 361.21835 | [M+Na]+ | 361.2193 | 58.04136:9454 59.04858:48441 73.02811:30879 80.05428:9866 87.04333:12174 89.05894:100509 90.0619:7012 133.08542:19612 253.44498:4951 318.4404:5197 | -2.62998E-06 |
| POS6986 | Allopregnanolone | 9.536 | 319.26096 | [M+H]+ | 319.26001 | 51.78613:7408 67.05386:6215 81.0683:6781 93.06837:17456 95.08472:8177 107.08482:7951 119.08607:6210 121.10124:10538 212.5829:6045 316.8718:5836 | 2.97563E-06 |
| POS13601 | Calenduloside E | 4.625 | 655.38031 | [M+2H]2+ | 655.38159 | 73.02811:264880 87.04334:687350 89.05896:3447120 113.05927:150994 133.08543:1321092 | -1.95306E-06 |
| POS8281 | DTXSID40693955 | 5.056 | 393.24561 | M+CH3OH+H]2 | 393.2446 | 57.03286:8746 59.04858:35580 70.68684:5779 76.29126:5243 87.04333:9185 87.39496:6144 89.05895:49405 101.0593:13576 103.07443:7488 133.08543:18297 138.69392:5703 155.00864:7420 | 2.56838E-06 |
| POS12429 | 386093-01_C34H4 | 6.131 | 590.32086 | [M+Na]+ | 590.32202 | 59.04859:6348 59.73867:5925 67.05389:17007 73.79539:5505 79.05313:13892 81.0699:9701 86.09541:86286 91.0539:12814 93.06841:12853 104.10697:20830 107.08487:7944 119.08334:7685 120.9869:5889 123.31204:5899 146.97896:104460 460.44394:5660 | -1.96503E-06 |
| POS12351 | Val-Glu-Ile-Pro-Gl | 8.782 | 586.30707 | [2M+H]+ | 586.30823 | 86.09541:42111 104.10696:279897 105.1111:10117 146.97894:40326 173.39111:14345 | -1.97848E-06 |
| POS8787 | Palau'imide | 5.276 | 429.27338 | [M+2H]2+ | 429.27429 | 57.03286:8078 59.04858:169278 60.05225:6477 67.01499:14280 69.03294:6572 73.02811:5794 81.03072:10236 85.06505:5593 87.04333:13633 89.05895:97862 91.05389:12442 101.0593:10057 103.07443:36596 133.08543:32376 147.10147:13437 387.87292:7334 | -2.11986E-06 |
| POS7807 | nethoxy-8-(methy | 5.857 | 362.03809 | M+CH3OH+H] | 362.03711 | 51.95142:6460 55.0177:5768 67.46735:6006 82.06343:12142 91.0539:100200 101.25815:5273 119.04706:16269 121.02692:16325 264.27393:7943 | 2.7069E-06 |
| POS7299 | Pipericine | 7.722 | 336.32452 | [M+NH4]+ | 336.3255 | 55.48339:6753 55.876:6022 64.58645:6061 66.81867:5411 157.44508:5415 173.39111:13440 335.26138:5837 | -2.91384E-06 |
| POS10865 | Cholylvaline | 5.123 | 530.34625 | [M+H]+ | 530.34521 | 57.03287:11319 59.04859:222796 69.06992:5704 73.02812:23499 73.06432:14832 85.06506:9730 87.04335:222920 87.06254:12243 87.07999:17550 89.05896:359254 90.06375:11755 101.05931:83294 103.0385:13641 103.07445:70159 109.0756:6347 117.05281:16226 117.08818:9561 129.08911:11479 131.07088:19808 131.10634:6099 133.08545:146562 134.08685:6598 145.08403:8277 147.10149:34862 173.43036:11283 177.11287:21267 191.1256:7692 282.47211:5720 529.29529:6452 | 1.96099E-06 |
| POS9219 | )-Eicoseneoylcarn | 5.772 | 454.38867 | [M+H]+ | 454.38965 | 57.03286:16430 57.06895:6063 57.78485:5819 59.04857:12049 60.08024:26214 61.65057:5446 85.02798:138083 90.2034:5032 152.38158:5777 157.5979:6596 | -2.15674E-06 |
| POS8348 | Napelline N-oxide | 6.748 | 398.23111 | [M+H]+ | 398.23013 | 149.02057:11000 214.37152:5354 267.62708:5763 355.32816:6300 | 2.46089E-06 |
| POS8046 | tibiotic KA 6606 X | 5.699 | 376.25641 | [M+H-H2O]+ | 376.2554 | 57.06893:699280 58.07269:10234 67.26308:5204 70.55553:5401 116.75143:6984 125.07156:8673 126.0536:21966 140.03654:5666 208.147:6492 209.12428:43279 210.11501:7913 213.08969:6199 218.12753:8382 219.11333:6674 275.17349:7102 293.17691:7592 302.17621:13491 376.26236:14500 | 2.68435E-06 |
| POS9507 | Ebastine | 5.062 | 470.3064 | [M+2H]2+ | 470.30539 | 57.03287:43096 58.04042:26597 59.04859:972238 60.05226:23087 65.04819:28614 69.06992:16341 73.02812:179898 73.06432:46282 80.0543:63123 83.04932:11665 85.06337:33685 87.04335:232396 87.06079:83016 87.07999:23623 89.05896:1608846 90.06191:47124 94.06947:17818 101.05931:142289 102.06606:46435 103.07444:213219 109.07315:44856 109.57641:10420 115.07378:11588 117.05281:14388 117.0909:18327 124.08022:10414 127.07501:10495 129.08911:26812 131.06766:25568 133.08545:525393 134.08684:26022 140.09358:10795 145.08403:11520 147.10149:103445 177.11285:52604 191.1256:9549 | 2.14754E-06 |
| POS12233 | Manzamine X | 4.833 | 581.34735 | [M+Na]+ | 581.34851 | 104.79475:14431 | -1.99536E-06 |
| POS7958 | Octaethylene glyco | 4.499 | 371.2265 | [M+H]+ | 371.22751 | 59.04859:14098 73.06432:6886 87.04335:41143 89.05896:878039 90.06191:15323 101.05931:6933 107.07059:8062 131.08699:6423 133.08545:237512 177.11285:17984 285.99164:5706 | -2.7207E-06 |
| POS8891 | hosphoryl]oxy-2-l | 6.451 | 436.27899 | [M+H-H2O]+ | 436.28 | 84.57517:5229 90.81367:5393 139.2675:5804 226.54878:6242 235.64577:5658 327.74368:6693 | -2.31503E-06 |

| Differences in metabolites between the Model group and the WJW group | | | | | | | |
|---|---|---|---|---|---|---|---|
| Alignment ID | Metabolite name | Rt(min) | Expreiment Mz | Adduct type | Reference m/z | MS/MS spectrum | PPM |
| POS8678 | (-)-Terpendole B | 5.173 | 422.2674 | [M+2H]2+ | 422.2684 | 57.03286:13210 59.04858:182542 67.01499:21936 81.02915:8017 83.87628:5473 87.04333:18935 89.05895:114294 101.0593:18614 103.07443:42228 117.05552:5882 131.06764:6691 133.08543:28848 147.10147:10643 173.42543:9310 177.11284:6188 191.12558:5809 257.68277:5035 | -2.36816E-06 |
| POS10892 | Leu Asp Asn Arg | 4.473 | 531.28741 | [M+H-H2O]+ | 531.28851 | 79.6421:9188 86.09712:12182 87.05381:26044 89.05896:169138 133.08545:96417 143.89046:9582 146.05721:17239 173.42545:10498 177.1078:12586 188.07004:101378 205.09822:21849 247.72908:9404 248.81287:9322 | -2.07044E-06 |
| POS10216 | 4R,5R)-antillatoxir | 5.567 | 504.34421 | [M+H-H2O]+ | 504.34317 | 59.04859:55591 89.05896:23572 101.05931:9029 103.07444:12691 133.08545:9579 175.37425:6049 | 2.06209E-06 |
| POS12556 | Dihydroxycanthaxa | 5.831 | 596.38879 | [M+2H]2+ | 596.39001 | 57.03379:25446 59.04859:152492 67.015:12316 73.02812:25803 73.06432:22756 87.04335:42468 89.05896:127194 101.05931:15587 103.07445:19902 133.08545:37316 156.90764:11128 166.20825:10197 271.25543:11374 | -2.04564E-06 |
| NEG4399 | TADECA-3,6,9-TRI | 0.839 | 247.24217 | [M-H]- | 247.24319 | 64.0225:5693 113.12118:40005 133.25772:5213 | -4.12549E-06 |
| POS3868 | AMOZ | 2.237 | 202.11746 | [M+H]+ | 202.1185 | 55.05365:119164 56.05764:6675 59.04858:9776 67.05389:6586 70.0643:136839 71.04816:29730 71.05974:76454 83.0477:21601 101.0593:34106 110.72093:5593 111.04189:13509 113.0696:13377 114.05462:7124 157.88727:6214 | -5.1455E-06 |
| POS4543 | Adalinine | 7.196 | 226.17897 | [M+NH4]+ | 226.17999 | 66.62321:5697 67.05389:9229 81.0699:8029 95.08477:9012 171.1862:5397 173.42545:6583 | -4.50968E-06 |
| POS11481 | PE 22:0 | 5.89 | 552.36475 | [M+2H]2+ | 552.36591 | 57.03287:32364 59.04859:160838 73.02812:48004 73.06432:8898 85.06506:15372 86.09541:13243 87.04335:41960 87.06079:6514 87.07999:12448 89.05896:125523 101.05931:29687 103.07444:27711 104.10697:58575 115.07378:7187 129.08911:6066 133.08545:36473 147.10149:14657 173.39111:10247 184.06931:19733 234.51561:6047 | -2.10006E-06 |
| POS15372 | Primycin B1 | 7.169 | 946.69574 | [M+NH4]+ | 946.69373 | 57.03287:19356 66.28696:5270 340.01797:5958 | 2.12318E-06 |
| POS13354 | Neomycin | 6.753 | 637.30286 | [M+2H]2+ | 637.30151 | 173.43524:9276 317.64862:5666 438.72989:7181 614.59955:6760 | 2.11831E-06 |
| POS15149 | LacCer(d18:1/12:0) | 7.051 | 806.56409 | [M+H]+ | 806.56238 | 96.4697:6680 | 2.12011E-06 |
| POS13933 | Cimiracemoside D | 4.602 | 679.40662 | [M+2H]2+ | 679.40515 | 87.04334:261744 89.05896:1951863 133.08543:766098 194.5706:85597 342.42755:85462 469.50879:108563 | 2.16366E-06 |
| POS10430 | nethyl-1-oxo-2-p | 5.784 | 514.27881 | [M+H]+ | 514.27991 | 59.04858:37228 73.06432:26921 85.06506:7012 87.04334:9315 89.05896:29110 95.08477:6550 101.05712:9332 104.10696:6575 106.33912:5185 126.02023:53819 131.10634:7123 | -2.13891E-06 |
| POS11941 | phlomiside | 8.96 | 569.20874 | [M+2H]2+ | 569.20752 | 62.99562:5907 79.55058:5110 104.10696:25395 138.2146:5137 184.07466:10556 366.74438:6801 | 2.14333E-06 |
| NEG2214 | Glycylproline | 8.998 | 171.07645 | [M-H]- | 171.07751 | 65.77522:6130 79.0537:8499 91.19855:6752 100.76936:5625 127.03277:51219 171.30643:8510 | -6.19602E-06 |
| NEG2215 | Glycylproline | 0.143 | 171.07645 | [M-H2O-H]- | 171.07751 | 109.19514:6443 127.03276:23681 134.32732:5374 171.30641:5507 | -6.19602E-06 |
| POS5938 | Obscuraminol A | 6.585 | 278.24634 | [M+H]+ | 278.24741 | 98.85948:6284 111.15255:5710 149.02057:21243 | -3.8455E-06 |
| POS5106 | nethoxyphenyl)-2 | 4.905 | 247.08507 | [M+H]+ | 247.084 | 61.811:5658 66.62203:5495 130.02655:5917 173.43034:9058 | 4.33051E-06 |
| POS9849 | caryolanemagnolo | 5.971 | 487.32169 | [M+H]+ | 487.32059 | 59.04858:18644 66.91378:5588 89.05895:19841 105.14582:6132 138.83788:5569 168.3127:5941 170.74451:5189 | 2.25724E-06 |
| POS9374 | -3,4-dehydro-apo | 5.261 | 462.3089 | [M+2H]2+ | 462.31 | 57.03287:68483 59.04859:1640159 60.05226:37212 65.04819:28519 67.05389:7945 69.06992:18982 73.02812:108145 73.06432:26699 80.0543:17455 81.06834:9411 85.06337:37671 87.04335:212219 87.06254:50748 87.07999:35953 89.05896:1176095 90.06191:47001 94.06947:35528 99.07939:10329 101.05931:146547 103.03849:16839 103.07444:333822 104.07732:14685 107.07059:12041 109.0756:14204 115.07378:13589 116.08248:20526 117.05281:12288 117.0909:25986 127.07501:9358 129.08911:28441 131.06766:20109 131.10634:11361 133.08545:327602 134.09018:19521 145.08403:8459 147.10149:147866 161.1188:9065 177.11285:24585 191.1256:24974 | -2.37936E-06 |
| POS7883 | Betaenone A | 5.159 | 367.24951 | [M+2H]2+ | 367.24841 | 57.03287:82888 59.04859:1792934 60.05226:41220 65.04819:30358 69.06992:10700 73.02812:124861 73.06432:25247 85.06337:32324 87.04335:136399 87.06254:32476 87.07999:23482 89.05896:839407 90.06191:26550 94.06947:35327 101.05931:117040 103.07444:240294 104.07732:10586 109.0756:15513 116.08248:11466 117.05281:10242 117.0909:19812 129.09227:10554 131.07088:12461 133.08545:185155 134.08684:11321 147.10149:97569 177.11285:12571 191.1256:10145 | 2.99525E-06 |
| NEG9457 | pferol 3,7,4'-tri-O- | 5.894 | 524.90985 | [M-H]- | 524.91101 | 59.08406:64413 67.13567:8066 79.05222:24424 107.21417:9070 119.23064:5283 140.17944:10809 147.16307:5463 352.116:5702 434.19601:6421 | -2.2099E-06 |

**Differences in metabolites between the Model group and the WJW group**

| Alignment ID | Metabolite name | Rt(min) | Expreiment Mz | Adduct type | Reference m/z | MS/MS spectrum | PPM |
|---|---|---|---|---|---|---|---|
| POS7643 | Capillolide | 5.044 | 353.23419 | [M+2H]2+ | 353.23309 | 57.03287:115815 58.04137:39062 59.04859:2607516 60.05226:48531 65.04819:56027 67.05508:14948 69.06992:20878 73.02812:241521 73.06432:163613 80.0543:55733 85.06506:44910 87.04335:225480 87.06254:82433 87.07999:29597 89.05896:1496379 90.06191:47134 94.06947:27298 101.05931:145079 102.06606:28477 103.07444:367553 104.07732:15207 109.0756:38159 115.07643:17702 117.05281:192758 117.0909:49022 129.08911:26939 131.07088:105185 133.08545:425978 134.09018:17067 147.10149:87636 161.1188:22603 177.11285:32119 | 3.11409E-06 |
| POS5577 | Protriptyline | 4.97 | 264.17349 | [M+Na]+ | 264.17459 | 59.04859:30915 73.06432:6336 77.01915:5714 87.04335:18298 89.05896:14100 101.05931:9161 173.38622:6861 | -4.16391E-06 |
| POS12378 | ganorbiformin A | 4.969 | 587.35907 | [2M+H]+ | 587.35779 | 57.03287:19218 59.04859:262800 73.02812:16307 73.06432:11398 85.06337:13281 87.04335:528738 89.05896:240711 101.05931:136286 103.03849:102338 103.07444:68206 115.07378:13795 117.05281:40507 131.06766:19995 133.08545:75808 147.10149:36730 478.21463:10321 | 2.17925E-06 |
| POS9696 | ahydro-1H-cyclop | 5.367 | 480.3129 | [M+2H]2+ | 480.314 | 59.04857:103646 60.05124:6194 62.05908:6808 67.01498:9006 73.0281:10117 73.13141:6883 81.06831:8845 85.06335:9736 87.04333:12205 89.05894:73041 90.91968:7216 101.05928:6046 103.07442:20538 105.06943:5431 126.02021:107504 133.0854:12086 159.1172:9335 161.12755:8955 205.14232:6011 205.30017:5483 208.05679:6945 359.60992:5718 401.25186:6022 | -2.29017E-06 |
| POS3757 | octylpyrrolidin-2-c | 7.273 | 198.1841 | [M+H]+ | 198.1852 | 75.69515:7077 133.79388:6443 155.98309:6354 168.10655:6377 171.41733:6722 173.39111:11331 | -5.55036E-06 |
| NEG3105 | Spermine | 0.809 | 201.2074 | [M-H]- | 201.2085 | 61.06563:9276 62.6869:5420 64.61039:6008 71.497:6145 81.87756:5654 89.09516:25801 89.13128:10589 103.21892:6510 111.10325:24071 149.19171:5498 | -5.46697E-06 |
| POS15179 | ty-16,22,23-trihyd | 4.655 | 809.46619 | [M+2H]2+ | 809.46802 | 73.02811:130332 73.06432:64109 87.04334:322702 89.05896:1307563 131.06766:73103 133.08543:593855 177.895:58002 | -2.26074E-06 |
| POS11162 | oxy-5??-cholest-3 | 5.876 | 541.37463 | [M+2H]2+ | 541.37341 | 57.03287:55586 59.04859:377973 60.08025:175833 73.02812:49737 86.09541:411670 87.04335:44070 89.05896:165272 101.05931:55497 103.07444:74900 104.10697:1493231 124.99956:88707 133.08545:44281 184.07466:902685 | 2.25353E-06 |
| POS11897 | Hydroxymanzamin | 5.713 | 567.37061 | [M+2H]2+ | 567.36932 | 57.03287:32090 59.04859:225088 65.11697:7883 67.015:9217 69.06868:7730 73.02812:27208 73.06432:12085 80.0543:10143 81.03072:14445 85.06337:6232 87.04335:42018 87.06254:8100 87.07999:15385 89.05896:147403 90.06191:7741 90.55772:6238 101.05931:32599 102.42354:6623 103.07444:62652 115.28882:6132 117.0909:11533 133.08545:48468 147.10149:20078 165.10016:10555 187.26393:6734 224.00723:7255 242.62587:6749 395.24411:6288 | 2.27365E-06 |
| POS9944 | -Acetylgentamicin | 5.973 | 492.30383 | [M+H]+ | 492.3027 | 53.31924:7416 59.04858:17883 60.08025:11439 86.09541:18227 89.05896:10776 104.10696:100376 168.57562:4870 173.04839:5391 184.07466:34363 | 2.29534E-06 |
| POS4790 | tylidene-1-Deoxy | 5.301 | 235.15277 | [M+H]+ | 235.1539 | 59.04859:36308 89.05896:12673 101.05931:7281 | -4.80536E-06 |
| POS12572 | Kadsuphilol C | 6.885 | 597.23431 | [M+H]+ | 597.23297 | 68.38469:5406 250.95917:6494 303.30765:6625 | 2.24368E-06 |
| POS5064 | Diethyl azelate | 7.266 | 245.17357 | [M+H]+ | 245.17468 | 60.35422:5795 69.06868:7381 72.65029:5639 83.32471:5613 177.83897:6060 | -4.52738E-06 |
| POS4789 | tylidene-1-Deoxy | 4.962 | 235.15277 | [M+2H]2+ | 235.1539 | 57.03287:10776 59.04859:40011 75.04173:5838 86.09541:6763 87.04335:6496 89.05896:15004 99.54275:6028 101.05931:12771 104.31946:5300 173.38622:6893 | -4.80536E-06 |
| POS7363 | -1-ylidene)-5alph | 5.958 | 339.26678 | [M+H-H2O]+ | 339.26791 | 55.05365:8739 57.06989:9879 59.04858:13318 67.05389:7170 69.06868:11374 76.29413:8746 81.0699:14839 83.08509:6751 93.06841:7072 95.08477:11768 121.09843:6574 180.99232:6405 192.80493:6095 | -3.3307E-06 |
| POS14414 | 2'-O-Acetylactein | 4.578 | 719.40167 | [M+NH4]+ | 719.40002 | 64.37184:55564 67.51974:55907 69.03294:237349 73.02811:120073 73.06297:67230 83.0477:116527 87.04334:450720 89.05896:1374356 95.04893:53932 99.04339:341638 111.04189:201892 122.10059:61744 125.0596:63125 133.08543:561076 150.88187:48487 155.07083:115493 174.20796:58532 362.51266:54235 | 2.29358E-06 |
| POS11698 | Arg Thr Lys Arg | 5.588 | 560.36389 | [M+2H]2+ | 560.36261 | 57.03287:22512 59.04859:242637 67.015:24194 81.03072:13634 85.90186:12964 87.04335:32407 89.05896:164672 101.05931:33101 103.07444:66674 104.10697:90582 133.08545:45681 147.10149:30558 161.25945:12601 165.10016:17400 168.84389:12040 184.07466:29869 191.1256:13898 239.59464:13538 424.36945:13016 | 2.28424E-06 |

**Differences in metabolites between the Model group and the WJW group**

| Alignment ID | Metabolite name | Rt(min) | Expreiment Mz | Adduct type | Reference m/z | MS/MS spectrum | PPM |
|---|---|---|---|---|---|---|---|
| POS11018 | Phe Leu Asp Lys | 4.711 | 536.30658 | [M+2H]2+ | 536.3078 | 68.92713:6230 69.03295:5334 78.79263:5559 79.1211:5431 87.04335:8284 89.05897:32309 117.82632:5640 133.08545:6548 149.22003:5394 173.38622:8250 303.48926:5258 444.81195:5902 473.62137:6605 | -2.27481E-06 |
| POS14079 | 25-trihydroxy-21a | 4.862 | 691.44312 | [M+H]+ | 691.44153 | 59.04859:232466 73.02812:26440 73.06432:24661 87.04335:121594 89.05896:857079 101.05931:58881 103.07444:87512 131.07088:26065 133.08545:322000 147.10149:51095 173.44016:17372 177.11285:41023 258.35931:15898 268.91992:15496 | 2.29954E-06 |
| POS6281 | no-1,3,4-hexadeca | 5.048 | 290.26773 | [M+H]+ | 290.26889 | 55.05366:10786 56.04863:189825 57.06897:135063 58.06417:132218 58.42599:5736 60.04426:25540 68.04951:8877 68.50143:5538 71.08419:38979 74.05887:115193 78.92046:5853 85.10046:11041 86.05939:13852 88.07445:175317 118.08495:35197 122.08031:36038 164.62282:5251 242.24483:35748 290.26651:13312 | -3.99629E-06 |
| POS9691 | PC(15:1(9Z)/0:0) | 5.383 | 480.30545 | [M+2H]2+ | 480.30661 | 57.03379:14320 59.04956:151841 67.01499:11221 73.02811:13915 80.43406:5472 81.03072:10483 87.04508:20484 89.05895:111443 101.0593:14821 103.07668:35478 133.08543:33690 147.10147:11830 158.09198:7813 | -2.41512E-06 |
| POS3920 | yl)oxy]pyridine-3- | 2.187 | 204.06197 | [M+Na]+ | 204.06313 | 55.93345:6871 60.04426:49156 61.03972:8693 91.0539:6082 204.9343:5201 | -5.68452E-06 |
| NEG9707 | Epicatechin | 6.194 | 579.00317 | [M-H]- | 579.00452 | 51.52467:7674 115.89837:5631 125.34409:5780 173.16458:5215 320.50644:7472 | -2.33159E-06 |
| POS12842 | dyl pseudopteran | 5.051 | 609.37994 | [M+H]+ | 609.37848 | 59.04859:157183 73.02812:36917 80.0543:26331 85.06337:19243 87.04335:74354 89.05896:483808 90.90853:15706 101.05713:40824 103.07444:43739 133.08545:215034 147.10149:33676 549.27478:18568 | 2.39588E-06 |
| POS7674 | andamide (20:l, n- | 7.171 | 354.33459 | [M+H]+ | 354.33578 | 51.79896:6119 64.54077:6654 199.06522:5344 352.11353:6186 | -3.3584E-06 |
| POS4432 | Carbofuran | 0.688 | 222.11081 | M+CH3OH+H] | 222.112 | 58.06417:33664 62.63849:5800 73.04688:8793 91.0539:29708 114.88578:5683 119.04706:16113 135.04564:8455 209.00752:8800 225.04044:76549 | -5.35766E-06 |
| POS13262 | oxowitha-3,5,24-tr | 4.548 | 631.34912 | [M+H]+ | 631.3476 | 69.03294:531950 73.02811:243743 73.06432:134227 76.79319:61089 81.03229:96327 83.0477:276069 87.04334:1322209 89.05896:3918546 90.0619:65491 95.04893:152763 99.04339:1144402 107.07059:83202 109.06336:74333 111.04189:549180 113.05927:78300 125.0596:289524 129.05132:87907 131.07088:170648 133.08543:1947140 137.05981:128580 151.09705:71775 155.07083:464980 173.07771:106829 177.11285:207110 356.07733:73384 | 2.40755E-06 |
| NEG1676 | 5-propanoic acid, | 0.976 | 153.06822 | [M-H2O-H]- | 153.067 | 58.00906:6838 79.05367:6097 93.03925:706937 93.15687:31392 95.03763:1316380 154.24498:6898 | 7.97037E-06 |
| POS4934 | enyl-3-(Pyridin-2- | 4.579 | 241.13478 | [M+H-H2O]2+ | 241.13358 | 55.05365:10048 57.06896:33395 58.06416:1074768 59.06808:27011 67.05389:24511 69.06868:13924 79.05312:12429 81.0699:142001 82.07301:10510 84.07957:53294 95.08477:23404 123.11523:84284 140.14345:14921 | 4.97649E-06 |
| POS10794 | l-7-oxo-3-oxaper | 5.25 | 527.33826 | [M+2H]2+ | 527.33698 | 57.03287:46245 59.04859:531889 73.02812:105948 73.06432:31040 87.04335:496867 87.06079:42824 89.05896:707738 101.05931:232147 103.07444:118606 104.10697:100247 105.10879:35413 133.08545:221115 147.10149:52471 157.54272:26623 160.78989:23542 173.44016:29715 184.06931:34472 225.7818:24805 | 2.42729E-06 |
| POS6384 | Lanyuamide I | 6.014 | 294.24127 | [M+NH4]+ | 294.24249 | 51.58743:5684 55.05365:13873 57.06895:6691 58.06416:16018 59.04858:13208 67.05389:9580 69.06867:6520 69.51213:5415 71.08418:19877 72.06339:5677 79.05312:5969 81.06989:16726 93.0684:10649 95.04893:10753 95.08475:10952 99.07938:9041 103.45982:5507 107.08485:6496 121.10128:6063 151.10899:10128 173.42542:10887 | -4.14624E-06 |
| POS7921 | Cholesterol | 0.727 | 369.34979 | [2M+H]+ | 369.35101 | 51.55879:6271 57.06894:6732 67.05387:10748 69.06989:6279 71.08417:7706 81.06831:12099 93.0703:6586 95.08474:9880 107.08483:9799 109.10003:11523 115.62182:6104 135.11641:11067 147.11678:9908 161.13193:8112 | -3.30309E-06 |
| POS3835 | minic acid; Dihydr | 2.265 | 201.08569 | [M+H]+ | 201.0869 | 55.05365:208072 56.94232:6443 59.04858:17433 70.0643:107285 71.05974:20129 73.06432:7664 81.06833:8008 83.0477:29349 101.0593:58942 111.04189:31051 113.0696:30268 113.96312:18595 114.05462:13930 129.05447:6787 | -6.0173E-06 |
| POS7557 | Dodecyl glucoside | 5.611 | 349.25684 | [2M+H]+ | 349.25809 | 53.00208:46898 55.05365:21485 57.03287:2262521 58.03662:29273 59.04858:645513 60.05225:8515 69.06868:16523 81.0699:61309 97.06461:35786 99.07938:81290 101.09641:13722 115.07378:94912 117.09089:27878 157.12312:40542 | -3.57902E-06 |
| POS9429 | R-tetrol 1-O-alpha | 5.97 | 465.34085 | [2M+H]+ | 465.3421 | 53.00125:7212 55.05365:6134 57.03287:1001863 58.03662:16164 59.04858:291038 69.06991:8673 81.06833:29495 84.86993:5864 97.06461:18596 99.07938:66823 101.09641:14815 115.07378:76797 117.09089:25959 157.12312:56319 | -2.6862E-06 |

**Differences in metabolites between the Model group and the WJW group**

| Alignment ID | Metabolite name | Rt(min) | Expreiment Mz | Adduct type | Reference m/z | MS/MS spectrum | PPM |
|---|---|---|---|---|---|---|---|
| POS5586 | nethyl-4-n-tridec- | 5.843 | 264.26724 | [M+NH4]+ | 264.26849 | 55.05365:17531 56.04863:6359 57.06896:12214 67.05389:25964 69.06868:16706 70.06431:10714 79.05312:28424 81.05736:7540 81.06834:23025 82.06503:166526 83.08509:8547 87.04334:5763 93.06841:22611 95.07282:8271 95.08477:12848 96.08018:30769 107.08486:9970 107.43556:6034 123.45921:5364 244.9557:5570 264.26468:36412 | -4.73004E-06 |
| POS6358 | -beta-D-glucopyr | 5.031 | 293.19446 | [M+NH4]2+ | 293.19571 | 57.03287:7399 59.04859:55202 73.02678:6749 87.04335:11890 89.05896:26747 101.05931:26250 168.88631:5777 184.97366:6137 | -4.26336E-06 |
| POS6357 | -beta-D-glucopyr | 5.388 | 293.19446 | [2M+H]+ | 293.19571 | 59.04859:38699 76.22691:6115 87.04335:6678 89.05896:19281 101.05931:11689 122.48106:5725 173.43034:9639 222.31757:5454 | -4.26336E-06 |
| POS6356 | -beta-D-glucopyr | 5.168 | 293.19446 | [M+Na]+ | 293.19571 | 57.03287:9277 59.04859:39097 79.92984:5719 87.04335:8527 89.05896:20142 100.119:5361 101.05931:9225 | -4.26336E-06 |
| POS7262 | lecatrienoic acid is | 7.32 | 334.30914 | [M+NH4]+ | 334.31039 | 73.8131:5978 173.42545:10635 | -3.73904E-06 |
| POS4314 | pha-Acetyl-L-citru | 2.242 | 218.11223 | [M+NH4]+ | 218.11348 | 60.04326:10332 60.08025:11494 70.0643:172582 85.02799:88013 103.29053:5246 103.88376:5663 113.0696:76240 114.05462:66520 115.08438:28031 159.07413:29905 218.09305:6247 | -5.73096E-06 |
| POS7263 | lecatrienoic acid is | 7.48 | 334.30914 | [M+H]+ | 334.31039 | 199.73647:5709 | -3.73904E-06 |
| POS8229 | lidenebeyer-15-er | 5.054 | 389.24869 | [M+Na]+ | 389.24741 | 59.04858:19214 87.04334:6695 89.05896:9851 101.94215:5459 | 3.2884E-06 |
| NEG1677 | 5-propanoic acid, | 1.081 | 153.06828 | [M-H]- | 153.067 | 60.17114:7239 79.0537:13233 93.03928:807346 93.15691:14844 95.03767:1450379 154.24504:7592 | 8.36235E-06 |
| POS11281 | anine-3beta,17,23 | 5.349 | 546.34113 | [M+2H]2+ | 546.34253 | 57.03287:20336 59.04859:239650 67.015:23160 73.02812:21084 81.03072:13272 86.09541:10981 87.04335:27798 87.07999:9468 89.05896:203904 90.06191:10843 101.05931:24012 103.07444:61977 104.10697:83116 117.0909:9549 130.85844:8922 133.08545:77872 147.07468:10073 147.10149:27853 151.08508:14899 158.09198:14052 184.07466:18004 251.92677:8982 466.18372:8502 | -2.56249E-06 |
| POS8915 | legonoxamine D | 5.059 | 437.27228 | 1+CH3OH+H]2 | 437.271 | 59.04858:51499 64.85931:7350 67.015:13694 70.60906:5529 73.06432:7510 87.04334:12699 89.05896:82936 90.06191:6787 101.0593:13977 103.07444:11380 109.40909:5558 133.08545:20776 256.47888:5178 | 2.92725E-06 |
| POS3073 | nyl-ribo-hexose-a | 5.004 | 177.11082 | [M+H]+ | 177.11211 | 57.03287:6878 59.04859:13795 75.04312:6009 87.04335:6016 89.05896:7373 130.06477:5905 132.07892:18046 173.39111:13727 | -7.28352E-06 |
| POS11965 | 4S,5S,6R)-3,4,5-tri | 8.947 | 570.21643 | [M+2H]2+ | 570.2179 | 60.08025:8675 78.17443:5727 86.09541:10618 104.10696:59038 124.64912:5634 184.07466:25470 276.23544:5963 318.97809:6314 | -2.57796E-06 |
| POS13100 | tra-Ac-Glucoerysc | 8.651 | 622.09558 | [M+H]+ | 622.09277 | 57.30859:7273 163.38239:5280 173.3911:15519 184.22493:5325 283.61749:5576 432.82986:5820 | 4.51701E-06 |
| POS12383 | tenacibactin G | 5.045 | 587.39984 | [M+H]+ | 587.40137 | 57.03379:49528 59.04859:1150583 73.02812:174833 73.06432:106459 80.0543:36403 85.06337:68389 87.04335:364272 87.06254:45358 87.07999:39762 89.05896:2128959 90.06191:78061 101.05931:212338 102.06606:25877 103.07444:373822 109.0756:31911 115.07378:27184 117.05281:75104 117.08817:28534 129.08911:36362 131.06766:60847 133.08545:963873 134.09018:26638 147.10149:256485 161.11441:42884 173.39111:45294 177.11285:102401 191.1256:39949 205.57848:24855 336.53061:25361 | -2.60469E-06 |
| POS12353 | Leu Ser Leu Pro Gl | 5.604 | 586.34613 | [M+H]+ | 586.3446 | 55.58558:7301 59.04856:39906 60.08022:16137 86.09536:32586 87.04504:8769 89.05891:19117 101.47509:6543 104.10692:144929 113.55135:5751 124.99949:8850 137.26674:5675 145.44116:5577 184.07457:48053 | 2.60939E-06 |
| NEG818 | 4-Methylcatechol | 1.229 | 123.0424 | [M-H]- | 123.0437 | 80.0528:71794 87.12827:5272 94.14119:14123 124.15681:26277 | -1.05654E-05 |
| NEG2861 | ı±)-trans-Nerolid | 0.647 | 193.16119 | [M-H]- | 193.1599 | 55.54954:6353 85.23931:6047 103.05704:52750 | 6.6784E-06 |
| POS4060 | Polycanthisine | 7.199 | 208.1682 | [M+NH4]+ | 208.16949 | 52.88708:5961 109.24215:5320 110.50856:7136 124.29422:5920 163.05995:6082 | -6.19687E-06 |
| POS9494 | Malonganenone C | 5.369 | 469.31851 | [M+2H]2+ | 469.3172 | 57.03286:56965 59.04858:1142699 59.0593:28750 60.05225:31066 69.0699:12710 73.02811:61570 73.06431:25556 85.06335:27969 87.04333:133959 87.06252:19345 87.07997:25264 89.05894:648978 90.0619:25966 94.06945:23606 101.05929:119793 102.06383:7071 103.07442:246499 107.07057:10209 115.07376:9573 117.05279:11042 117.09087:15522 129.0891:18761 130.08705:6924 131.06764:10362 131.10631:10108 133.08542:178867 134.08682:7983 143.1041:8150 145.08401:10484 147.10147:95276 161.11438:12011 173.396:12762 177.11282:10385 191.12556:12121 | 2.79129E-06 |

**Differences in metabolites between the Model group and the WJW group**

| Alignment ID | Metabolite name | Rt(min) | Expreiment Mz | Adduct type | Reference m/z | MS/MS spectrum | PPM |
|---|---|---|---|---|---|---|---|
| POS11700 | gamma-Solanine | 5.05 | 560.39716 | [M+H]+ | 560.39569 | 57.03287:43652 59.04859:819372 73.02812:35597 73.06432:39036 87.04335:92548 87.07999:22864 89.05896:397039 90.06191:14676 101.05931:102712 103.07444:286688 115.07378:16524 117.05281:52576 117.0909:29847 126.69132:14541 131.06766:27036 133.08545:98508 134.34398:12696 147.10149:104265 161.1188:38344 214.75627:13536 325.95432:13449 | 2.62315E-06 |
| POS8841 | Fumifungin | 5.541 | 432.2977 | [M+2H]2+ | 432.29639 | 57.03287:73422 59.04859:1232213 60.05226:30514 61.02846:7108 65.04931:8454 69.06992:8820 73.02812:48709 73.06432:18833 85.06337:16643 87.04335:61455 87.06079:17964 87.07999:12595 89.05896:345651 90.06191:9339 94.06947:18745 101.05931:80332 101.1816:6440 103.07444:219893 104.0796:11684 115.07378:15155 117.0909:21544 129.08911:6620 131.07088:11849 133.08545:69052 147.10149:65316 161.11441:15682 191.1256:7175 215.3116:6205 | 3.03033E-06 |
| POS13218 | baeckein F | 8.764 | 629.09088 | [M+Na]+ | 629.09253 | 62.18739:7329 70.11976:6194 87.02068:5993 91.66339:5579 141.59047:5065 535.01624:6253 561.61584:6028 | -2.62283E-06 |
| POS9544 | Betnesol | 4.411 | 473.17471 | [2M+H]+ | 473.1734 | 53.73895:6162 72.08048:11154 79.14983:5657 86.09541:8131 119.82919:5778 137.04604:1404390 138.04726:40284 188.07004:12017 | 2.76854E-06 |
| NEG9081 | -2-yl-2,3,4,5,6,7,7 | 0.865 | 469.4657 | [M-H]- | 469.46701 | 113.12114:215686 181.19063:8714 226.34309:5786 | -2.7904E-06 |
| POS15336 | Erythrosine | 6.873 | 858.64624 | [M+H]+ | 858.64398 | 53.67301:5841 90.49667:5979 176.32578:6196 278.14832:5738 287.21078:5204 779.2157:6077 | 2.63206E-06 |
| POS12957 | pha-cholestane-3 | 5.537 | 615.41187 | [M+2H]2+ | 615.41022 | 57.03287:44080 59.04859:834913 73.02812:47441 73.06432:44656 85.06337:39158 87.04335:140754 87.06254:22146 87.07999:33512 89.05896:715880 90.06191:22219 94.06947:23019 98.14563:19219 101.05931:127727 103.07444:256990 110.08812:18889 115.07378:23200 117.0909:25901 129.09227:24319 131.06766:23162 133.08545:219415 136.89111:17692 147.10149:101774 335.16913:18091 387.0867:18684 611.11322:18948 | 2.68114E-06 |
| POS13648 | leptasteroside L | 5.508 | 659.43463 | [M+2H]2+ | 659.4364 | 57.03379:45047 59.04859:610398 73.02812:59204 73.06432:39891 85.06506:23196 87.04335:119176 87.06254:32428 87.07999:21949 89.05896:665859 90.06191:27727 101.05931:133712 103.07444:178434 117.0909:37598 121.28181:21722 129.08911:30531 133.08545:245690 147.10149:94194 170.73015:25919 415.89944:21779 | -2.68411E-06 |
| POS4086 | epoxy-15R-hydro | 4.436 | 209.13698 | [M+NH4]+ | 209.13831 | 59.04859:329315 70.06431:58044 84.07958:16457 85.06506:6163 87.04335:14626 89.05896:314430 94.06359:27752 96.08018:9538 103.07445:14247 107.06821:7843 118.06566:9683 133.08545:25822 146.061:9335 148.11055:35581 187.04947:5586 | -6.35943E-06 |
| POS5183 | 5-methylsulfinylhe | 4.956 | 249.16536 | [M+H]+ | 249.164 | 59.04859:32759 73.02812:6702 89.05896:12184 208.26324:6382 | 5.45825E-06 |
| POS11847 | Valnemulin | 5.139 | 565.36841 | [2M+H]2+ | 565.36688 | 59.04859:518046 73.02812:56453 87.04335:163613 89.05896:1044131 96.30509:36186 101.05931:63679 103.07444:124619 109.0756:44440 133.08545:456674 147.10149:79674 177.11285:51930 | 2.70621E-06 |
| POS5280 | 3,4,6-Penta-Me-A | 4.464 | 253.16315 | [M+H]+ | 253.16451 | 59.04859:545051 85.06337:9005 87.04335:61951 89.05896:1103382 90.06191:21774 103.03849:19756 103.07444:43522 107.07059:20743 112.07435:6289 133.08545:122674 146.87569:6869 151.09306:11128 177.1078:5932 | -5.372E-06 |
| POS11227 | Thr Ile Pro Ile Thr | 7.036 | 544.33527 | [M+H]+ | 544.33398 | 50.03354:5940 100.52903:6793 284.80084:6514 | 2.36987E-06 |
| NEG744 | Dimethoxypropan- | 9.109 | 119.07005 | [M-H]- | 119.0714 | 59.08403:37295 61.75145:6227 73.11752:6672 97.57933:4913 100.04586:6139 101.04565:74731 102.04721:49298 105.74018:5590 118.07099:59549 119.06866:51500 | -1.13377E-05 |
| POS8909 | isovaleryl-blastmy | 5.754 | 437.19043 | [M+Na]+ | 437.1918 | 59.04858:13381 99.09844:5843 115.8652:5791 173.44014:7355 | -3.13364E-06 |
| POS5303 | Iodine | 1.063 | 254.81473 | [M+H]+ | 254.8161 | 61.03867:23704 67.29745:7271 80.94772:593200 138.90459:11091 | -5.37643E-06 |
| NEG3376 | Zalcitabine | 4.322 | 210.08705 | [M-H]- | 210.08842 | 66.15359:4819 68.75729:6831 69.07782:4508 74.63445:4580 85.09258:4703 86.00461:4641 93.35798:4824 94.57208:3693 95.05959:4105 95.53884:4481 104.05004:4949 105.25879:5141 106.06308:4313 106.9953:4225 107.84846:5108 110.26845:4463 115.0144:4439 119.9493:4524 122.31573:4279 125.98219:5389 126.38684:4694 126.5578:4420 126.81796:4924 138.25093:4513 141.14272:4189 151.39058:4107 155.47362:3909 167.15324:4146 181.72069:4445 189.89471:4348 198.03937:5441 198.21295:4317 202.14603:4163 207.77614:5262 | -6.52106E-06 |
| POS12436 | -2,3,5,6,7,11,12,15 | 4.934 | 590.40765 | [M+NH4]+ | 590.40601 | 59.04859:838808 87.04335:134216 89.05896:939187 101.05931:114236 103.07444:326499 114.22485:21803 117.05281:51142 117.0909:25332 131.07088:30137 133.08545:324380 147.10149:179621 161.11441:25466 177.11285:34271 191.1256:33417 298.89792:23738 347.28244:25441 393.26031:24812 | 2.77775E-06 |

| Differences in metabolites between the Model group and the WJW group | | | | | | | |
|---|---|---|---|---|---|---|---|
| Alignment ID | Metabolite name | Rt(min) | Expreiment Mz | Adduct type | Reference m/z | MS/MS spectrum | PPM |
| POS12415 | dro-1H-cyclopent | 4.749 | 589.38702 | [M+H]+ | 589.38867 | 59.04858:22575 87.04333:30022 89.05894:167008 91.48834:9489 103.07442:10588 133.08542:58874 165.20952:8194 186.57237:8493 | -2.79951E-06 |
| POS15029 | PC(16:0e/12-HETE | 5.901 | 784.5824 | [M+H]+ | 784.58502 | 50.02442:27649 60.08025:49787 86.09541:132816 105.14583:23793 124.99956:27804 173.43526:46783 184.06931:433022 623.297:25599 688.9975:26388 690.32001:28995 | -3.33934E-06 |
| POS11693 | Leu Ser Ala Leu Glu | 5.52 | 560.33051 | [M+2H]2+ | 560.32892 | 53.19652:9330 57.84054:8613 59.04856:55066 60.08023:59508 71.07258:14030 86.09538:143371 87.04332:23037 89.05893:58555 101.05927:12562 103.07441:9952 104.10693:430735 105.11107:11428 124.99951:32392 133.0854:13643 184.0746:298269 325.12164:7536 560.04681:8055 | 2.83762E-06 |
| POS11127 | 3R,4R)-3,4-dihydro | 5.978 | 540.30292 | [M+Na]+ | 540.30139 | 57.77541:5576 59.04856:15019 60.08023:6218 86.09538:23974 89.05893:6018 96.25027:5780 104.10693:110546 146.98273:12025 | 2.83175E-06 |
| POS5185 | COOH-2But-A7EC | 5.148 | 249.16818 | [M+2H]2+ | 249.1696 | 59.04859:43293 80.82903:5246 89.05896:13447 120.44286:6777 173.39111:7545 | -5.69893E-06 |
| POS5184 | COOH-2But-A7EC | 5.326 | 249.16818 | [M+H]+ | 249.1696 | 59.04859:34217 73.56042:5763 89.05896:14240 102.36123:6026 | -5.69893E-06 |
| POS9048 | Mevinolinic acid | 5.796 | 445.25461 | [M+H]+ | 445.25601 | 59.04858:9104 86.5484:5603 89.05895:5840 149.04791:5048 173.42543:11955 268.03186:5439 326.53644:5422 | -3.14426E-06 |
| POS14930 | PG 36:2 | 8.745 | 775.54608 | [M+2H]2+ | 775.54828 | 68.68565:6044 75.29504:4935 | -2.8367E-06 |
| NEG1524 | 1,2,3,4-tetrahydroi | 7.486 | 146.09901 | [M-H]- | 146.0976 | 100.04586:8539 101.05436:159156 102.05606:168511 103.057:27012 144.21524:6429 | 9.65108E-06 |
| NEG5078 | Sempervirine | 0.991 | 271.12555 | [M-H2O-H]- | 271.12411 | 89.13125:22548 90.1367:8192 93.03925:648444 95.03763:1175372 97.03745:192475 145.23549:9570 153.06755:50462 155.06651:25311 | 5.31122E-06 |
| POS10415 | Ala Leu Ala Pro Lys | 5.358 | 513.34088 | [M+2H]2+ | 513.33942 | 57.03379:102069 59.04956:2082748 60.05226:59262 65.04931:17320 69.06992:27439 73.02812:152526 73.06567:64356 80.0543:29008 83.04932:12528 85.06506:58920 87.04509:335997 87.06254:67164 87.07999:63058 89.05896:1542368 90.06374:50734 94.06947:63915 99.08151:17238 101.05931:272991 102.06606:21560 103.04074:17742 103.07444:472059 104.0796:14513 107.07059:25414 109.0756:32055 115.07378:21387 116.08248:25829 117.05553:19112 117.0909:50235 129.09227:45088 131.07088:37560 131.10634:27178 133.08545:580697 134.09018:35650 138.09604:15243 143.1078:17884 145.08403:28035 147.10149:303286 148.1028:18751 161.1188:25219 169.11278:15132 173.43034:24680 177.11285:64152 191.13127:40275 | 2.84412E-06 |
| POS6843 | Ficellomycin | 5.412 | 313.19678 | [M+Na]+ | 313.19821 | 57.06989:6684 62.84341:5471 271.15952:10932 313.1973:23428 | -4.5658E-06 |
| POS8109 | )-1-(2,4,6-trimetho | 5.259 | 381.12848 | [M+Na]+ | 381.12991 | 59.04858:24008 70.1021:6931 73.06432:6414 73.32253:5950 87.04334:10393 89.05896:18119 101.0593:15519 255.73947:5295 | -3.752E-06 |
| POS9766 | Euryspongiol A1 | 5.653 | 483.33017 | [M+2H]2+ | 483.3316 | 57.03287:14812 59.04858:207015 60.05225:6586 62.06014:6340 73.02812:13751 87.04334:13053 89.05896:90974 94.06947:7405 101.0593:21132 103.07444:45253 104.10696:78496 115.07378:7008 133.08545:19303 147.10149:17951 147.60472:5190 173.43034:12231 199.8517:6654 308.36151:5312 | -2.95863E-06 |
| NEG7454 | lupanacosmine | 1.079 | 356.27225 | [M-H]- | 356.27081 | 93.03925:573915 95.03763:34431 97.03745:8066 102.77911:6475 124.15676:56260 134.97455:6595 151.06711:23835 182.18414:14521 | 4.04187E-06 |
| POS9769 | Complanadine A | 6.313 | 483.34955 | [M+H]+ | 483.34811 | 59.04856:76678 95.27219:5765 104.10693:24816 117.09086:9704 161.69566:5716 173.4303:10129 184.0746:7882 | 2.97922E-06 |
| NEG6505 | Aprindine | 6.288 | 321.23227 | [M-H]- | 321.2337 | 53.33165:5608 57.10276:13443 59.08403:341658 60.08905:44248 61.0902:19132 62.78073:6033 67.13445:11165 69.11722:49064 70.1209:15373 71.09898:13863 79.05216:7284 81.16814:10027 83.15008:13115 91.63754:6215 107.2141:39095 108.21955:32842 135.278:45323 136.28323:51715 139.27885:17981 140.28273:17285 153.31172:7172 154.31902:8632 163.30577:17887 164.31381:25905 179.32059:50916 180.32471:76803 181.33206:20822 204.42909:8547 258.54407:7895 280.51889:5748 | -4.45159E-06 |
| POS12886 | H-3,12-epoxy[1,2]c | 4.61 | 611.35553 | [M+2H]2+ | 611.35376 | 69.03294:60483 73.02811:301618 73.06432:88196 81.03229:85977 83.0477:56965 87.04334:632549 89.05896:3386812 90.0619:114237 91.04457:79164 95.04893:177748 99.04339:106397 111.04189:192093 113.05927:149944 131.06766:63385 133.08543:1148567 134.08684:76949 137.0598:73368 155.07083:93591 164.50946:43316 173.39111:50584 175.10023:43853 177.11285:137209 442.12436:47115 | 2.89521E-06 |
| POS10705 | dimine B\|gymnody | 5.355 | 524.3385 | [M+2H]2+ | 524.33698 | 60.08024:562938 71.07259:89143 72.72482:79104 75.99161:84194 86.0954:1219011 89.05895:152949 100.47708:77300 104.10696:6883640 105.10877:170021 118.89655:80900 124.99954:278323 172.39996:81033 184.06927:2543607 253.02956:86323 360.09375:93576 390.51123:104774 | 2.8989E-06 |
| POS10859 | leopolic acid A | 6.498 | 530.32086 | [M+H]+ | 530.32239 | 59.04858:7175 67.40431:6374 86.38091:6041 183.17155:5455 277.32327:5659 | -2.88504E-06 |

| Differences in metabolites between the Model group and the WJW group | | | | | | | |
|---|---|---|---|---|---|---|---|
| Alignment ID | Metabolite name | Rt(min) | Expreiment Mz | Adduct type | Reference m/z | MS/MS spectrum | PPM |
| POS8983 | ommunesin F\|com | 4.842 | 441.26627 | [M+H]+ | 441.2648 | 57.03287:16432 59.04859:379092 73.02812:16020 73.06432:10834 85.06337:7376 87.04335:950837 88.04605:19752 89.05896:261383 97.06461:6118 101.05931:178688 103.03849:176796 103.07444:67373 115.07378:15655 117.05281:107968 131.07088:19277 133.08545:62974 145.08403:6911 147.06319:6605 147.10149:23804 173.38622:6797 | 3.33133E-06 |
| POS13122 | 3-methoxybenzoy | 5.184 | 623.39606 | [M+Na]+ | 623.39423 | 53.5634:6773 57.68123:8495 59.04859:10286 87.04335:9461 89.05896:24055 115.29946:6531 122.35307:7124 418.62692:6913 | 2.93554E-06 |
| POS6626 | )-3,15-Docosadier | 8.426 | 303.30313 | [M+H]+ | 303.3046 | 78.00248:5855 173.43027:11384 | -4.84661E-06 |
| POS13362 | Antioxidant 1098 | 6.565 | 637.49188 | [M+H]+ | 637.49377 | 51.36454:6747 53.10913:5163 53.34431:4667 55.26744:4753 55.4603:4592 57.16814:4529 57.60226:4841 59.38129:5185 59.70594:4757 59.73666:4807 60.54902:4772 61.71716:4371 62.1821:4951 63.79452:4673 67.00437:4054 67.07275:4297 68.12308:4600 72.93169:4974 79.71844:4418 81.57861:4400 93.94611:4260 95.96295:4347 96.63064:5221 99.46171:4827 100.33014:4970 101.19031:4252 107.37337:4320 109.60594:4511 110.68337:3835 111.24065:3892 111.91395:4893 116.4918:4230 119.59566:4429 121.4167:4181 126.0597:4394 127.47894:4810 138.11342:3916 149.29829:4425 154.5083:4636 155.32823:4680 168.06438:3975 171.64886:5563 173.42538:3826 174.58382:5302 186.85178:4110 190.22644:4278 199.62126:4291 204.95943:4169 224.4037:4408 230.90944:5263 276.89719:3772 288.78519:4333 289.20724:5025 311.63177:4714 321.98441:4252 326.27039:5364 337.58038:4794 337.66031:4594 339.78909:4637 342.22339:4357 365.4505:4522 383.93292:4938 418.79239:4649 445.98264:4205 453.12668:4803 552.55151:5532 555.49207:5100 609.55859:5993 | -2.96473E-06 |
| POS10837 | etyl-7,8-didehydro | 4.929 | 529.35529 | [M+H]+ | 529.3537 | 57.03287:122787 59.04859:2515289 60.05226:35061 73.02812:49060 73.06432:43538 83.04932:16974 85.06337:48027 87.04335:323380 87.07999:46630 89.05896:1727090 90.06191:39242 99.07939:18467 101.05931:253110 101.09641:16173 103.03849:52425 103.07444:806262 105.09028:15142 115.07378:33323 117.05281:46176 117.0909:45960 129.08911:28993 131.06766:25724 131.10634:22433 133.08545:409248 143.10413:16687 145.08403:30187 147.10149:320582 161.11441:51390 177.1078:18989 191.1256:41225 205.14238:19176 | 3.00366E-06 |
| POS6680 | Benzosimuline | 8.807 | 306.1503 | [M+H]2+ | 306.1488 | 103.46206:5460 218.17601:6104 | 4.89958E-06 |
| POS12019 | Kinetensin 4-7 | 6.178 | 572.28857 | [M+2H]2+ | 572.29028 | 59.04859:6540 60.08025:10112 76.07848:6124 86.09541:27595 104.10697:101438 105.10879:10998 184.06931:69282 185.07635:9413 186.07535:7044 | -2.98799E-06 |
| POS9447 | ethanolamine lysc | 6.406 | 466.32761 | [M+H]+ | 466.3291 | 51.75655:5860 57.03287:13613 59.04858:45287 | -3.19517E-06 |
| NEG1638 | Ribitol | 1.081 | 151.0685 | [M-H2O-H]- | 151.07001 | 90.1349:28099 93.03928:2218054 108.14954:29810 136.36186:5906 151.20676:17480 | -9.99537E-06 |
| POS10620 | nigramide I | 5.149 | 521.33887 | [M+2H]2+ | 521.33728 | 59.04859:1043720 60.05226:27521 65.04931:28196 73.02812:149483 73.06432:49144 80.0543:55029 85.06506:39103 87.04335:217216 87.06079:100088 87.07999:44362 89.05896:1615024 90.06191:61559 94.06947:20830 101.05931:118585 102.06606:39513 103.07444:265622 104.10697:19272 109.07315:34636 116.08248:18047 117.0909:18739 129.08911:30466 131.07088:34386 133.08545:555306 134.09018:31190 145.08403:23317 147.10149:146270 173.43034:27456 177.11285:53251 184.06931:72285 191.1256:33343 448.9303:17013 | 3.04985E-06 |
| POS8173 | Persicaxanthin | 6.014 | 385.27136 | [M+H]+ | 385.27289 | 67.05389:19140 79.05312:16632 81.06989:7775 91.05389:11767 93.0684:7734 105.06713:5753 180.40804:5330 | -3.97121E-06 |
| POS9848 | Ile Val Leu Glu | 5.51 | 487.31403 | [M+2H]2+ | 487.3125 | 57.03287:11754 59.04859:178044 67.015:12952 73.02812:7761 81.03072:9289 87.04335:14631 87.07999:6587 89.05896:107639 101.05931:16636 103.07444:35327 133.08545:34823 147.10149:13984 221.12637:5452 | 3.13967E-06 |
| NEG6661 | CCG-47657 | 1.066 | 327.14783 | [M-H]- | 327.1463 | 54.53023:5918 93.03925:570753 95.03763:280756 151.06711:26429 153.06754:25377 211.29141:6546 267.29996:5797 291.4368:5469 | 4.67681E-06 |
| POS15252 | Apramide G | 8.762 | 828.54401 | [M+H]+ | 828.5415 | 70.83127:5762 179.4697:5893 213.31702:6425 | 3.02942E-06 |

| Differences in metabolites between the Model group and the WJW group | | | | | | | |
|---|---|---|---|---|---|---|---|
| Alignment ID | Metabolite name | Rt(min) | Expreiment Mz | Adduct type | Reference m/z | MS/MS spectrum | PPM |
| POS8932 | hoethanolamine (F | 6.689 | 438.29608 | [M+H]+ | 438.29761 | 52.20243:4490 53.61563:5270 53.66457:4977 60.84661:4453 62.38481:4801 65.33078:4671 65.99124:5305 66.74718:4625 67.60562:4701 70.60014:4795 76.86985:4876 78.83771:5031 82.89027:4843 83.98693:4172 86.43439:4401 87.24602:3800 92.00359:4432 95.965:4148 103.80876:4389 105.82056:4366 112.09728:4502 116.57019:4230 118.66303:4819 120.83271:4774 128.8532:4697 129.61676:4410 132.38916:3731 134.03683:4933 134.87729:3885 137.11153:4585 137.99503:4415 139.85175:4222 141.37001:4694 155.07085:5265 155.99983:4850 156.95407:4549 158.97508:4801 173.45488:5520 174.03526:4117 175.35431:4045 175.96431:4479 198.67374:4813 203.77364:4810 205.12976:5112 222.55981:5364 242.70706:4240 257.70947:3820 271.49545:4172 273.60205:4675 278.77704:4638 293.27414:3851 313.62628:4549 314.39111:7065 352.8526:5080 401.8049:4986 423.26389:4703 | -3.49078E-06 |
| POS1260 | ydroxyphenyletha | 1.517 | 121.07151 | [M+H]+ | 121.07 | 56.04952:14227 61.03869:6216516 72.08047:22252 | 1.24721E-05 |
| POS13319 | 3,36,39-Tridecaoxa | 4.563 | 635.38287 | [M+2H]2+ | 635.38483 | 87.04334:469308 89.05896:3297967 90.06374:82939 117.09089:67942 131.07088:76644 133.08543:1125547 177.11285:102442 216.40135:69223 369.65768:67791 | -3.08474E-06 |
| POS12958 | achidonic acid-bio | 5.328 | 615.42828 | [M+H]+ | 615.43018 | 55.33458:16317 57.03287:42628 59.04859:680263 69.15135:12526 73.02812:17714 73.06432:47268 83.45882:10977 87.04335:62743 89.05896:223262 101.05931:64144 103.07444:201452 117.05281:48062 117.0909:33547 131.06766:22156 133.08545:41216 147.10149:33225 161.1188:18984 580.7569:13279 | -3.08727E-06 |
| POS13273 | palmitoyl-sn-glyce | 4.565 | 632.35535 | [M+H]+ | 632.35339 | 69.03294:129341 73.02811:35006 73.06431:30610 83.04769:63903 87.04333:227547 88.04781:24677 89.05895:619305 90.0619:60743 99.04338:155461 100.04802:31763 111.04189:78529 117.09088:32560 125.05659:34261 129.0513:30956 133.08543:238546 134.08682:38132 155.07082:38177 173.42543:33366 | 3.09953E-06 |
| POS14295 | -4a-methyl-3,4,4a | 4.861 | 708.47028 | [2M+H]+ | 708.46808 | 59.04956:128458 67.2335:25062 83.81858:19368 87.04335:67545 89.05896:420225 101.05931:36987 103.07444:52472 131.06766:19661 133.08545:208250 147.10149:30929 177.11285:29017 490.83408:20972 | 3.10529E-06 |
| POS8788 | Palau'imide | 5.276 | 429.27655 | [M+H]+ | 429.27509 | 59.04858:78891 62.70562:6008 67.01499:7043 81.03072:6086 87.04334:7795 89.05895:63827 89.54285:5633 101.0593:6206 103.07443:18299 133.08543:19503 133.1481:6079 147.10147:5292 169.53391:6042 296.42734:6526 | 3.40108E-06 |
| POS14893 | oline-3r-carboxyl | 4.581 | 771.29462 | [M+H]+ | 771.297 | 66.49142:46233 223.17427:48411 310.88882:50981 | -3.08571E-06 |
| POS14229 | thyl)oxan-2-yl]oxy | 5.306 | 703.48169 | [M+H]+ | 703.47949 | 57.03287:69866 59.04859:845872 65.52287:39937 68.4929:35657 87.04335:78830 89.05896:398245 98.69503:47696 101.05931:115384 103.07444:239877 117.05281:55079 133.08545:119557 147.10149:122421 184.06931:55139 | 3.12731E-06 |
| NEG1302 | Myrothenone B | 7.705 | 138.05797 | [M-H]- | 138.0564 | 57.42459:5554 137.05487:7981 | 1.13722E-05 |
| NEG296 | eamino)-N-meth | 7.709 | 99.05807 | [M-H]- | 99.0565 | 52.83277:6460 58.19922:5807 | 1.58495E-05 |
| POS9971 | 1-(prop-1-en-2-y | 4.799 | 493.2984 | [M+H]+ | 493.29999 | 51.77015:6467 89.05895:10948 109.86272:5593 | -3.22319E-06 |
| POS8733 | eptapropylene glyc | 5.228 | 425.3092 | [M+H]+ | 425.31079 | 57.03287:102488 59.04858:1181776 60.05225:14621 87.04334:10245 89.05896:20238 103.96567:5605 115.07378:10823 117.09089:124968 173.3862:6020 175.1301:13398 274.86017:6186 | -3.73844E-06 |
| POS11082 | Val Leu His Arg | 5.602 | 538.34418 | [M+2H]2+ | 538.34589 | 57.03286:9390 59.04858:114031 67.01499:6481 73.02811:14208 76.64165:6153 87.04333:18041 87.06252:9217 89.05895:88988 101.0593:11741 103.07443:25937 104.10696:45449 115.07377:5394 133.08543:26685 147.09764:9660 184.06927:14473 | -3.1764E-06 |
| POS8892 | Leu Leu Tyr | 6.411 | 436.28207 | [M+H]+ | 436.28049 | 51.60652:6803 160.33104:6165 173.43027:13779 198.89635:5400 | 3.62152E-06 |
| NEG7048 | alactinol Dihydrat | 0.785 | 341.3584 | [M-H]- | 341.35999 | 80.71797:5797 119.32285:5836 196.84229:5703 | -4.65784E-06 |
| POS7824 | ne glycol monodo | 6.877 | 363.30881 | [M+H]+ | 363.31039 | 56.0234:6606 82.39974:5989 286.62631:5919 | -4.3489E-06 |
| POS13777 | | 5.18 | 667.42255 | [2M+H]+ | 667.42041 | 50.11649:13235 59.04859:12614 89.05896:21060 490.27408:12572 | 3.20637E-06 |
| POS3176 | Phenprobamate | 0.891 | 180.0993 | [M+H]+ | 180.10091 | 50.5035:5940 56.24197:6611 160.02197:6206 | -8.93943E-06 |

| Differences in metabolites between the Model group and the WJW group | | | | | | | |
|---|---|---|---|---|---|---|---|
| Alignment ID | Metabolite name | Rt(min) | Expreiment Mz | Adduct type | Reference m/z | MS/MS spectrum | PPM |
| POS8967 | (?)-agelasidine E | 5.267 | 440.29572 | [M+2H]2+ | 440.2941 | 57.03287:114316 59.04859:2189595 60.05226:54308 65.04819:34113 69.06992:23163 73.02812:163661 73.06432:40475 80.0543:20518 85.06337:43061 87.04335:292393 87.06254:57682 87.07999:43790 88.04783:13107 89.05896:1435500 90.06191:45631 94.06947:51671 99.07939:14391 101.05931:236192 102.06384:21944 103.03849:23308 103.07444:457049 104.07732:18845 107.06821:11388 109.0756:26532 115.07378:19339 116.08248:16834 117.05281:13209 117.0909:40443 127.07501:9301 129.08911:31106 131.06766:23576 131.10634:12972 133.08545:406086 134.09018:23270 138.09604:14415 143.10413:10586 145.08403:17173 147.10149:185349 161.11441:13617 177.11285:33361 191.1256:26315 | 3.67936E-06 |
| POS10008 | Norselic acid E | 5.253 | 495.31253 | [M+2H]2+ | 495.31091 | 57.03287:31592 57.06896:126130 58.06512:54802 59.04859:95775 60.08025:892944 67.05389:28807 71.07261:99655 71.08419:76540 81.06834:31475 83.0851:34036 85.10045:65301 86.09541:1777225 89.05896:124465 95.08477:35028 104.10697:9197985 109.10007:26533 124.99956:409778 133.08545:25773 143.24394:21408 163.01524:25461 184.07466:3315038 424.03168:20732 | 3.27067E-06 |
| POS9355 | navirine | 5.671 | 461.31781 | [M+2H]2+ | 461.31619 | 57.03287:59935 59.04859:897027 60.05226:17523 65.04819:8818 69.06868:11381 73.02812:41278 73.06432:16588 87.04335:50952 87.06254:8955 87.07999:14598 89.05896:251265 94.06947:19965 101.05931:62550 103.07444:172294 115.07378:10213 117.0909:25079 129.08911:8562 133.08545:51831 147.10149:63720 161.11441:14604 173.42545:8420 | 3.51169E-06 |
| POS9772 | aroten-4'-al; beta | 5.39 | 483.36047 | [2M+H]+ | 483.36209 | 57.03286:60035 59.04857:639142 60.05224:10273 69.8093:6211 73.0643:7539 87.04333:13400 89.05894:31742 99.07937:5985 101.0571:7897 103.07442:7199 115.07375:8980 117.09087:72617 118.09319:5757 133.0854:7041 175.13007:7639 277.59119:5874 418.71881:5733 | -3.35152E-06 |
| POS10701 | ylglycerophosph | 8.804 | 524.29651 | [M+2H]2+ | 524.29822 | 50.22102:49994 60.08023:159852 69.78801:43132 86.09538:275146 99.47875:45190 104.10693:1657209 105.10875:61902 184.06923:1175168 185.07629:113468 343.16348:49365 428.68253:52781 | -3.2615E-06 |
| POS8170 | MLS001074091-01 | 4.518 | 385.24164 | [M+H]+ | 385.23999 | 59.04859:198724 73.06432:23292 85.06506:9454 87.04335:135996 89.05896:1684771 90.06191:27626 101.05931:10071 103.07444:42261 107.07059:17644 118.46617:7133 129.08911:8882 131.06766:22653 133.08545:532233 134.09018:12455 147.10149:19174 177.11285:33121 | 4.28304E-06 |
| POS6992 | 3-Hexadecylpheno | 7.728 | 319.29785 | [M+H]+ | 319.2995 | 55.44436:6228 152.06296:5795 | -5.16756E-06 |
| POS5965 | 2-Heptanol gluco | 4.96 | 279.17847 | [M+H]+ | 279.18011 | 57.03287:7884 59.04859:34718 60.22052:7271 87.04335:20299 89.05896:26744 101.05931:55446 103.07444:8419 133.08545:7181 149.02057:9892 | -5.87434E-06 |
| NEG9735 | PFSM-amine | 5.075 | 585.06122 | [M-H]- | 585.05927 | 89.13124:1344418 90.13486:49481 91.69405:5157 173.586:6204 236.26202:6039 238.19133:5688 | 3.333E-06 |
| NEG2818 | Cyprotene | 5.414 | 191.17894 | [M-H]- | 191.1806 | 59.02949:33858 87.11256:5863 103.07276:50878 111.14096:13856 134.77959:5929 173.61551:12363 | -8.68289E-06 |
| POS9238 | Palmonine A | 5.153 | 455.30197 | [M+2H]2+ | 455.30029 | 57.03287:68998 59.04859:1527596 60.05226:45674 65.04819:25475 69.06868:17124 73.02812:146998 73.06432:42980 80.0543:33719 85.06337:37843 87.04335:245819 87.06079:71965 87.07999:33428 88.04783:10096 89.05896:1523596 90.06191:55922 94.06947:27000 101.05931:149161 102.06606:22085 103.03849:15588 103.07444:321698 104.07732:17927 107.06821:15137 109.07315:36192 115.07378:17028 116.08248:17508 117.05281:25268 117.0909:22044 127.07501:9960 129.08911:27742 131.06766:29751 131.10634:16879 133.08545:448558 134.08684:21470 138.09604:13135 145.08403:11129 147.10149:139436 177.11285:40433 191.1256:18424 | 3.68987E-06 |
| POS10068 | Tumonoic Acid I | 5.466 | 498.34079 | [M+2H]2+ | 498.34247 | 57.03286:38459 57.06988:22198 59.04858:684673 60.08024:89957 61.08377:15770 65.0493:16494 69.0699:12064 71.07259:12954 71.7504:10678 73.02811:62351 73.06431:11042 75.86941:10828 85.06335:15657 86.09539:198595 87.04333:149263 87.06252:30276 87.07997:13244 87.09917:39303 89.05894:384471 90.0619:13226 94.06945:16235 101.05929:79931 103.07442:110724 104.10695:892489 105.10877:292476 106.11103:40248 111.16512:11085 124.99953:42653 133.08542:94051 147.10147:40128 184.07463:249382 185.07632:98694 186.07532:41872 | -3.37118E-06 |

| Differences in metabolites between the Model group and the WJW group | | | | | | | |
|---|---|---|---|---|---|---|---|
| Alignment ID | Metabolite name | Rt(min) | Expreiment Mz | Adduct type | Reference m/z | MS/MS spectrum | PPM |
| POS13361 | milicifoline C | 5.521 | 637.42297 | [M+2H]2+ | 637.42511 | 57.03287:62586 59.04859:825992 73.02812:69609 87.04335:158241 87.06079:40926 89.05896:817897 101.05931:144391 103.07444:246986 122.71721:27694 133.08545:284362 147.10149:133885 177.11285:41117 298.98672:31467 | -3.35726E-06 |
| POS9308 | SCHEMBL1271357 | 5.06 | 459.28525 | [M+2H]2+ | 459.2869 | 59.04858:34977 59.05443:7569 67.01499:6994 87.04334:11196 89.05895:55091 95.59659:5571 103.07443:11088 133.08543:19402 147.10147:7609 173.3911:11204 270.74762:5613 | -3.59253E-06 |
| NEG5611 | ethyl-4-n-pentade | 1.872 | 290.28708 | [M-H]- | 290.2854 | 50.17372:5483 74.03466:7673 90.04866:10248 99.07362:6553 101.05444:6378 104.0432:7910 105.05279:24208 106.2744:5517 114.11422:8284 128.07486:6858 128.10909:6768 130.12769:71969 146.14146:21199 156.13734:53912 157.14728:6726 162.1563:5780 166.15576:7130 174.17671:6195 200.18018:12158 | 5.78741E-06 |
| POS6825 | SCHEMBL1021325 | 8.576 | 312.32361 | [M+H]+ | 312.32529 | 55.52958:5945 59.48562:5526 130.17635:5828 236.2997:5516 | -5.37901E-06 |
| POS10621 | nigramide I | 6.135 | 521.33905 | [M+H]+ | 521.33728 | 56.04953:14607 60.08025:237305 61.08378:19303 67.05389:10716 71.0726:40524 86.09541:677397 87.09918:72619 98.98415:28602 104.10696:130559 105.1111:17278 124.99955:215033 126.00201:11723 173.43034:12835 184.07466:2453926 185.07635:387950 186.07533:29304 | 3.39511E-06 |
| POS14227 | ulomo'opunalide- | 5.483 | 703.46161 | [M+2H]2+ | 703.46399 | 59.04859:390085 73.02946:39071 87.04335:113928 89.05896:523676 101.05931:107492 103.07444:96810 104.81318:28701 116.56748:27100 133.08545:200510 147.10149:61869 177.11285:34186 184.06931:41318 211.92552:23765 328.8551:31348 | -3.38326E-06 |
| POS3138 | se, 6-deoxy-3-O- | 0.947 | 179.0896 | [M+H-H2O]+ | 179.09129 | 64.6556:6685 69.68045:5681 79.08632:5474 | -9.43653E-06 |
| POS9788 | Poricoic acid B | 5.253 | 484.32059 | [M+2H]2+ | 484.31888 | 57.03287:68054 58.04042:17563 59.04859:1518611 59.05931:38277 60.05226:44010 65.04819:26666 67.05389:10380 69.06992:24349 73.02812:140028 73.06432:39950 80.0543:31676 85.06337:40551 87.04335:266839 87.06079:73682 87.07999:38629 89.05896:1348141 90.06191:47112 94.06947:36153 99.07939:9958 101.05931:204703 102.06384:22097 103.03849:13150 103.07444:343966 104.07732:14918 107.07059:14090 109.0756:29523 115.07378:19859 116.08248:13343 117.05281:17225 117.0909:23356 129.08911:28205 131.06766:22913 131.10634:13401 133.08545:420011 134.08684:29316 143.10413:9866 145.08403:14646 147.10149:155668 148.10667:10781 161.11441:15132 177.11285:33982 191.1256:14469 | 3.53073E-06 |
| POS10282 | ethyl-2H-chromer | 4.92 | 507.31213 | [M+Na]+ | 507.31039 | 89.05896:28389 133.08545:8527 328.20291:8787 | 3.42985E-06 |
| POS10506 | abloxime / IC202C | 5.249 | 517.32721 | [M+2H]2+ | 517.32898 | 57.03287:9752 59.04859:166769 67.015:27063 73.02812:11806 73.06432:9301 81.03072:6908 86.09541:9540 87.04335:20078 87.07999:7269 89.05896:186589 90.06374:9987 101.05931:11965 103.07444:48519 104.10697:66890 117.93352:5956 133.08545:62091 147.10149:28620 151.08508:9196 177.11285:5900 | -3.42142E-06 |
| POS7642 | syl-(1->4)-2,6-dic | 4.817 | 353.21518 | [M+Na]+ | 353.21689 | 57.03287:14908 59.04859:426669 60.05226:7114 71.04816:6970 73.02812:14269 73.06432:9159 87.04335:1004828 88.04605:21520 89.05896:117182 94.90382:7466 101.05931:210115 103.03849:70058 103.07444:29014 117.05281:109733 131.07088:17596 133.08545:19675 | -4.84122E-06 |
| POS7524 | oscr#20 | 5.046 | 347.2446 | [M+H]+ | 347.24289 | 55.05364:6974 67.05388:22365 69.0699:10099 79.05311:9369 81.06831:25682 83.08508:9356 93.06838:14687 95.08475:23665 105.06943:9346 107.08484:10309 109.10004:18336 121.10127:13071 293.7276:5209 | 4.92451E-06 |
| POS11799 | DTXSID10275904 | 5.859 | 563.38623 | [M+2H]2+ | 563.38831 | 57.03287:26132 59.04859:208545 73.02812:12773 73.06432:14312 87.04335:28288 87.07824:12119 89.05896:134492 101.05931:35914 103.07445:56449 104.10697:22666 112.35517:11952 117.05281:12725 117.0909:14243 133.08545:35059 173.43036:22893 184.81708:13305 244.06889:10416 | -3.69195E-06 |
| POS6131 | Tropisetron | 4.592 | 285.16193 | [2M+H]2+ | 285.16019 | 69.03294:11454 69.72296:6349 89.05896:7488 138.91872:5431 | 6.10183E-06 |
| POS10281 | -8-methoxy-10,12 | 4.779 | 507.30383 | [M+H]+ | 507.3056 | 59.04857:10016 73.0281:6517 89.05894:28411 133.0854:6429 143.883:5278 177.48293:7160 | -3.48902E-06 |
| NEG6279 | Arachidic acid | 4.943 | 312.30453 | [M-H]- | 312.3028 | 80.05276:7612 84.76994:6113 97.07439:6550 100.04586:23098 101.05437:45655 167.23665:6156 180.09624:28321 | 5.5395E-06 |
| POS10738 | 22alpha,23alpha-e | 6.78 | 525.34564 | [M+2H]2+ | 525.34747 | 55.72471:7451 69.15876:5473 154.17889:5150 171.15733:5928 206.71631:5893 207.01024:5952 | -3.48341E-06 |
| POS8429 | Enactin Ib | 5.415 | 403.27847 | [M+2H]2+ | 403.28021 | 57.03287:82442 59.04859:1698368 60.05226:36274 61.02743:9456 65.04819:10553 69.06868:10839 73.02812:79429 73.06432:24878 85.06337:21941 87.04335:72156 87.06254:16414 87.07999:20441 89.05896:561635 90.06191:17750 94.06947:39409 99.07939:9733 101.05931:95659 103.07444:279788 104.07732:12398 115.07378:14869 117.0909:23914 131.07088:12124 133.08545:117425 147.10149:89823 161.11441:14249 177.1078:7988 | -4.31462E-06 |

| Differences in metabolites between the Model group and the WJW group | | | | | | | |
|---|---|---|---|---|---|---|---|
| Alignment ID | Metabolite name | Rt(min) | Expreiment Mz | Adduct type | Reference m/z | MS/MS spectrum | PPM |
| POS8441 | n-2-yl]propanoyl | 5.07 | 404.26877 | [M+2H]2+ | 404.267 | 57.03287:58837 58.04137:29486 59.04859:1616627 60.05226:35988 65.04819:36403 69.06992:16574 73.02812:189531 73.06432:37543 80.0543:43942 85.06337:37458 87.04335:213136 87.06079:98341 87.07999:25048 87.56366:9658 89.05896:1617776 90.06191:48771 94.06947:38166 99.07939:10110 101.05931:120398 102.06606:31638 103.03849:13374 103.07444:284408 107.07059:17747 109.07315:50609 116.08248:18777 117.0909:19918 129.08911:33830 131.06766:24497 131.08699:18428 133.08545:484706 134.09018:23158 140.09358:10265 147.10149:112636 177.11285:42992 191.1256:16952 | 4.37829E-06 |
| POS10472 | Conglobatin | 6.082 | 516.30499 | [M+Na]+ | 516.30682 | 50.25237:7851 53.78294:6299 53.80497:7313 60.08022:6032 86.09536:48983 95.5825:6341 100.53114:5675 104.10692:335300 105.10873:8662 146.97888:29680 171.22461:6543 173.43517:13877 280.15149:7285 499.54236:7580 | -3.5444E-06 |
| POS4702 | hylideneamino)-5 | 5.789 | 232.11319 | [M+2H]2+ | 232.11142 | 57.03287:26397 59.04858:8583 73.45755:5578 104.44542:5183 | 7.62565E-06 |
| POS10501 | (2S,3R,4R,5R,6S)-3 | 6.171 | 517.01233 | [M+H]+ | 517.01416 | 57.88688:5908 66.74015:5527 173.43526:8482 259.29834:5302 323.72876:5833 345.17938:6032 | -3.53955E-06 |
| POS6132 | Boldione | 5.072 | 285.18777 | [M+H]+ | 285.186 | 55.05453:6606 55.1802:5646 64.45844:6128 67.05389:7664 81.06833:10869 104.02033:5184 106.8283:5606 144.31024:5418 | 6.20648E-06 |
| POS3415 | prosulfonyl)ethyl a | 8.788 | 186.98083 | M+CH3OH+H] | 186.9826 | 55.93345:21983 57.9342:7293 58.94153:16957 59.92951:71616 59.94047:26872 60.93742:204263 68.94187:20280 75.94469:6384 77.94041:52171 78.94756:11954 81.06989:8968 86.94053:31410 100.95683:85024 101.96426:52086 104.95158:35621 116.97124:39376 117.95827:18006 117.97753:41988 118.96619:651313 119.97298:735529 120.97546:8423 127.96568:11266 128.96005:20319 129.96925:8824 137.98456:11401 141.98206:61670 142.9902:57285 145.9549:7332 163.96249:17008 | -9.46612E-06 |
| POS5070 | 0,11,12,14,15,16,1 | 6.775 | 245.22453 | [M+H]+ | 245.2263 | 107.41644:6377 | -7.21782E-06 |
| POS14170 | exanoyloxy-6-[(2S | 4.637 | 699.4054 | [M+2H]2+ | 699.4079 | 69.03294:61248 73.02811:190035 87.04334:549712 89.05896:2719911 91.04457:62847 95.04893:142404 111.04441:137877 113.05927:90196 131.06766:69592 133.08543:1072420 134.09018:86522 137.0598:90294 177.11285:104394 539.42859:63564 | -3.57445E-06 |
| NEG1727 | -2-Hexenyl propa | 1.257 | 155.10521 | [M-H]- | 155.10699 | 57.10276:10240 59.08403:14502 69.11722:22841 79.05367:640348 80.05276:9872 81.14304:13137 83.15008:8291 93.15687:46848 95.03763:308253 96.13688:8700 97.03745:157327 97.08466:514540 97.10725:17037 109.17062:13483 111.1535:9824 111.17866:27566 119.17749:7602 137.08243:25627 147.46233:5920 154.24498:8477 | -1.14759E-05 |
| POS10668 | Methylepiamauron | 4.723 | 523.30859 | [M+H]+ | 523.3067 | 60.08024:39206 86.09539:83178 89.05894:24975 104.10695:432274 124.99953:18719 131.77299:5289 184.06927:171092 | 3.61165E-06 |
| NEG8043 | Arg Pro Asp | 1.051 | 385.1824 | [M-H2O-H]- | 385.1842 | 59.79719:5582 93.03928:417381 94.34543:5411 95.03767:189130 97.03749:6539 151.06717:41243 153.0676:34035 211.09386:6038 249.88901:7290 296.67526:5707 374.81418:5789 | -4.67309E-06 |
| POS14140 | odermin glycolipi | 5.389 | 696.45532 | [M+2H]2+ | 696.45282 | 59.04859:140904 68.52091:34557 87.04335:55010 89.05896:318027 101.05931:43050 103.07444:68044 133.08545:143489 242.30151:31183 382.90112:33882 512.59747:36556 623.69818:38147 | 3.58962E-06 |
| POS13845 | inaginsenoside R1 | 5.733 | 673.44965 | [M+2H]2+ | 673.45209 | 57.03287:85024 59.04859:784371 60.05126:27631 64.73385:24077 69.06868:27228 73.02812:76311 73.06432:39863 80.05276:20891 82.95837:15141 85.06337:45652 87.04335:179937 87.06079:38342 87.07999:55866 89.05896:756533 90.06374:30229 94.06947:43568 99.07939:21152 101.05931:189201 103.07444:251639 115.07378:31749 117.05281:32922 117.08817:36493 129.08911:41739 131.07088:26483 131.10634:31873 133.08545:338218 134.09018:24566 147.10149:190411 161.11441:20361 177.1078:45478 181.53217:18532 191.1256:51214 205.1487:16869 211.9454:17643 226.3:16723 340.80035:16961 380.6423:17731 397.44708:19957 | -3.62312E-06 |
| POS12302 | Shearinine F | 5.495 | 584.33398 | [M+NH4]+ | 584.33612 | 59.04858:61152 60.08025:92369 62.04125:18600 74.02055:19986 86.09541:196362 89.05896:85023 103.07444:23999 104.10696:871256 124.99955:34437 173.43034:42850 184.07466:412600 | -3.66228E-06 |
| NEG3825 | Pentadecanol | 0.798 | 227.23993 | [M-H]- | 227.2381 | 71.08354:41522 87.09683:59320 88.09991:9791 103.11093:93596 104.11378:13337 115.1256:84508 116.12636:9919 131.14032:348660 132.14209:51706 159.17082:77201 160.17122:17594 177.02853:5517 | 8.05323E-06 |

| Differences in metabolites between the Model group and the WJW group | | | | | | | |
|---|---|---|---|---|---|---|---|
| Alignment ID | Metabolite name | Rt(min) | Expreiment Mz | Adduct type | Reference m/z | MS/MS spectrum | PPM |
| POS10435 | Maraviroc | 5.065 | 514.33313 | [M+2H]2+ | 514.33502 | 58.04137:18704 59.04859:457688 60.05226:19809 69.06992:13175 73.02812:109044 73.06432:33504 80.0543:49272 85.06506:17945 87.04335:222579 87.06254:43340 87.07999:14094 88.04605:17976 89.05896:978529 90.06191:29299 94.06947:10053 101.05931:128383 102.06606:39990 103.07444:120581 107.06821:9358 109.0756:27888 116.08248:11130 117.05281:18551 117.0909:14302 124.07726:18119 129.08911:17802 131.06766:31383 133.08545:392702 134.08684:18607 140.09358:9025 147.10149:54001 153.09918:9300 177.11285:40338 191.1256:12368 | -3.67465E-06 |
| POS13977 | Rubescensin M | 4.947 | 683.41779 | [M+H]+ | 683.41528 | 71.70737:10859 86.33093:11542 88.84815:11253 105.48704:12829 150.76653:13188 173.39111:27333 | 3.67273E-06 |
| POS6704 | Ruscopine | 5.437 | 307.21088 | [M+NH4]+ | 307.21271 | 57.03286:9458 59.04858:17416 | -5.95678E-06 |
| NEG3179 | -coumaroyl-diketi | 1.208 | 204.04483 | [M-H]- | 204.043 | 79.01295:7636 89.12944:8651 93.03925:39450 124.02621:7196 148.73897:5526 167.03258:17470 | 8.9687E-06 |
| POS6451 | dodeca-2,6,10-tri | 4.487 | 297.19003 | [M+H]+ | 297.1882 | 59.04859:673313 73.06432:21185 85.06337:18211 87.04335:153095 89.05896:2518169 90.06191:56779 103.03849:14835 103.07444:72495 107.06821:28876 131.06766:10369 133.08545:485030 134.08684:16965 147.10149:19674 151.09705:15676 177.11285:10663 | 6.15771E-06 |
| POS7026 | e-(3Z,6Z,9Z)-3,6,9 | 8.429 | 321.31326 | [M+H]+ | 321.31509 | 70.24474:7366 76.22115:6057 158.60115:5338 319.11258:6577 | -5.69534E-06 |
| POS13677 | Eupassofilin | 4.946 | 661.43335 | [M+H]+ | 661.43091 | 57.03287:41420 59.04859:805266 73.02812:28269 73.06432:22540 85.06337:22492 87.04335:219622 87.07999:37638 89.05896:1216031 90.06375:24636 101.05931:162048 103.07444:273206 117.05281:18477 129.08911:26497 131.10634:29001 133.08545:406824 134.09018:20388 147.10149:153326 161.1188:17418 177.11285:22391 191.1256:28133 271.96683:16540 | 3.68897E-06 |
| POS10414 | rynamine A\|Pseud | 5.1 | 513.32422 | [M+Na]+ | 513.32233 | 57.03287:25815 59.04859:295939 73.02812:54974 73.06432:16633 80.05276:16281 85.06337:12561 87.04335:987143 87.06254:29520 88.04605:16722 89.05896:348376 101.05931:308362 102.06606:14154 103.03849:24348 103.07444:75383 115.07643:9455 117.05281:44277 117.0909:8690 130.0807:9060 131.07088:24750 131.10634:7846 133.08545:113121 147.10149:23943 177.1078:11328 406.83331:7597 | 3.6819E-06 |
| POS6075 | CHEBI:70570 | 4.972 | 283.17126 | [M+H]+ | 283.1694 | 55.05365:31218 57.06896:33562 67.05389:13975 69.06868:42773 71.08547:9772 79.05463:10750 81.06833:18709 83.08509:22840 89.05896:7788 93.06841:26898 95.08477:16820 97.06461:7051 97.09953:16189 100.07597:6934 107.08486:13321 109.10006:7549 111.0796:6733 121.1013:12432 135.11307:6930 163.14494:5752 173.43034:16278 | 6.56851E-06 |
| NEG1977 | ylsulfanyl-propyl)- | 0.192 | 163.03513 | [M-H]- | 163.037 | 59.08501:6264 101.95877:5287 102.12029:6051 112.62181:5924 | -1.14698E-05 |
| NEG298 | ieamino)-N-meth | 7.485 | 99.05838 | [M-H2O-H]- | 99.0565 | 50.45867:5282 50.63686:4731 52.5896:5478 54.41459:5216 54.5553:4341 55.59398:5477 55.64116:4879 57.96262:4576 60.51724:5215 62.46269:4877 63.83049:4119 66.61794:5225 67.90253:3883 70.18143:4460 70.47387:6502 72.58765:4299 72.85787:4634 75.44221:4565 75.67907:4878 77.01496:4716 81.56989:4984 87.99705:3960 89.88375:5045 94.98595:4363 96.0257:5005 96.65898:4448 | 1.89791E-05 |
| POS9605 | Progeldanamycin | 4.507 | 476.30289 | [M+NH4]+ | 476.30099 | 73.06432:18860 87.04334:121661 89.05896:1662491 90.0619:35266 107.07059:10722 112.07434:13270 117.08817:17685 131.06766:26392 133.08543:736677 134.09018:30300 173.39111:23393 177.11285:97579 | 3.98907E-06 |
| POS13171 | Enniatin-B2 | 5.54 | 626.39862 | [M+2H]2+ | 626.401 | 57.03287:25847 59.04859:309793 67.015:22678 69.06992:13608 73.02812:35115 73.06432:25919 85.06337:17718 87.04335:68475 89.05896:400755 90.06191:14581 99.92779:12315 101.05931:62306 103.07444:92552 109.0756:12974 117.08817:21424 131.06766:20017 133.08545:188559 147.10149:65896 173.39111:28819 177.11285:17788 225.6507:12906 | -3.79948E-06 |
| NEG2933 | noethyl methyl ph | 1.223 | 196.04738 | [M-H]- | 196.0493 | 56.43259:5803 59.08403:5648 79.05368:6140 81.55566:6182 97.07645:1401613 98.0778:45117 99.07355:179528 117.06951:30539 124.15677:12949 153.07974:7956 159.03728:9905 161.03622:529928 162.03658:8897 196.04443:52826 197.04991:6995 | -9.79346E-06 |
| POS7380 | cyclohenicosane-: | 4.542 | 340.25748 | [M+H]+ | 340.2594 | 59.04856:31007 69.06866:84019 71.08545:8408 73.06429:9314 79.0531:16901 79.1861:6788 87.04331:11348 89.05893:184400 96.08015:372017 97.06458:18211 97.08306:9037 113.10571:162547 114.09121:358557 115.09229:11966 133.08539:41046 173.43028:8567 174.75732:5879 195.7886:6075 209.16328:129619 210.16083:8006 226.19028:7461 227.17245:35459 321.50101:7261 322.24512:7990 | -5.64275E-06 |

| Differences in metabolites between the Model group and the WJW group | | | | | | | |
|---|---|---|---|---|---|---|---|
| Alignment ID | Metabolite name | Rt(min) | Expreiment Mz | Adduct type | Reference m/z | MS/MS spectrum | PPM |
| POS14697 | zamamidine A | 4.955 | 749.48724 | M+CH3OH+H] | 749.49011 | 59.04859:193782 87.04335:65241 89.05896:529070 101.05713:36432 103.07444:51241 109.67995:36455 133.08545:207760 147.09766:35842 404.62183:33904 474.819:35804 | -3.82927E-06 |
| NEG3341 | pentadec-2-ene | 1.342 | 209.22568 | [M-H]- | 209.2276 | 59.08403:13174 79.05367:23100 80.04047:5185 89.13125:25835 93.50523:6111 97.07645:787088 119.08539:8472 128.02501:9076 | -9.17661E-06 |
| POS1263 | Dimethoxypropan- | 3.17 | 121.08389 | [M+H]+ | 121.0858 | 56.96447:10960 61.03869:542954 77.03802:17207 84.959:22201 91.05389:21343 93.03563:11806 93.06841:25035 95.04893:44023 103.05421:116503 104.05679:19980 120.0803:84862 121.08412:12230 | -1.57739E-05 |
| POS2235 | Aminobenzothiazo | 0.763 | 151.03493 | [M+H]+ | 151.033 | 58.99696:9747 67.05389:7667 68.98123:290371 72.04237:6093 81.06989:9024 82.01395:151616 86.99278:262824 87.99461:6195 91.05389:5746 95.08476:6405 95.43819:6763 96.04378:6050 100.02438:330463 101.02657:9117 105.0024:291039 105.06944:9573 110.00877:47923 110.03355:8945 118.03534:92609 123.0126:44635 123.03898:8102 128.01854:202653 129.01982:7852 132.99644:9000 146.02687:21046 151.00931:6500 151.06114:22776 | 1.27787E-05 |
| POS11894 | Thr Tyr Arg Lys | 4.592 | 567.32703 | [M+2H]2+ | 567.32483 | 69.03417:53726 73.02811:220069 73.06432:42869 81.03385:46702 83.0477:46845 87.04334:445389 89.05896:2303357 90.06374:59990 95.04893:148172 99.04339:77710 111.04189:168038 113.05927:63343 131.06766:65612 133.08543:796132 134.09018:48601 137.0598:39987 155.06668:80457 173.39111:53708 177.11285:72286 294.94229:40929 562.07343:45543 | 3.87785E-06 |
| POS11924 | His Gly Lys Lys Val | 5.341 | 568.3587 | [M+2H]2+ | 568.35651 | 57.03379:15383 59.04859:190882 67.015:20860 73.02812:32846 73.06432:15990 81.03072:14575 85.06337:15280 87.04335:34936 89.05896:244115 90.06374:13454 97.61116:9353 101.05931:32276 103.07444:65960 133.08545:91691 147.10149:32142 157.22046:9896 158.09198:12284 177.11285:14461 409.98849:9933 | 3.85322E-06 |
| POS10589 | Fenestin B | 5.471 | 520.34729 | [M+2H]2+ | 520.3493 | 57.03284:30269 59.04856:486377 60.05223:17744 65.04928:8935 73.02808:33974 73.06429:9876 81.70692:6584 85.06333:12744 86.09536:9245 87.0433:50634 87.06075:10561 87.07994:7390 89.05891:288461 90.06187:7405 96.95786:7399 101.05926:37328 103.07439:85359 104.10692:41217 117.09084:11496 133.08537:66665 147.10141:31501 168.12987:6619 184.07457:22551 240.41701:6519 | -3.86279E-06 |
| POS14752 | Microcolin H | 5.991 | 756.54291 | [M+H]+ | 756.53998 | 60.04424:13397 60.08022:24316 86.09537:83658 124.9995:19123 184.06921:140597 | 3.8729E-06 |
| POS5068 | 10,11,12,14,15,16,1 | 9.918 | 245.22437 | [M+H-H2O]+ | 245.2263 | 109.76384:5522 194.7397:5349 | -7.87028E-06 |
| POS9517 | Terfenadine | 4.807 | 471.31564 | [M+H]+ | 471.31369 | 57.03287:65988 59.04859:1819779 60.05226:33917 61.02846:12331 69.06992:14219 73.02812:38060 73.06432:24521 83.04932:12352 85.06337:36044 87.04335:223799 87.07999:33804 89.05896:1708307 90.06191:29641 99.07939:9761 101.05931:180879 103.03849:32028 103.07444:524725 104.07732:13567 105.09028:11927 107.07059:14424 115.07378:10759 117.05281:32226 117.0909:27327 129.08911:23706 131.07088:27981 131.10634:12667 133.08545:416039 134.08684:13790 145.08403:18900 147.10149:247631 161.11441:20221 177.11285:18828 191.1256:24494 | 4.13737E-06 |
| POS11351 | asterone C 22-nic | 4.537 | 548.33484 | [M+H]+ | 548.33698 | 73.06431:42322 87.04333:107792 87.3213:27235 89.05895:1227742 90.0619:142684 117.09088:27070 131.07086:28767 133.08543:418955 134.09016:65235 177.11284:46088 | -3.90271E-06 |
| POS13082 | hydroxy-12-olean | 5.72 | 621.4173 | [M+Na]+ | 621.4151 | 55.77567:8051 59.04859:32506 73.02812:8587 87.04335:15535 89.05896:51955 90.06191:5907 100.78278:7247 101.05931:14746 103.07444:13967 133.08545:20985 147.10149:8119 173.39111:10285 314.7146:6119 320.94473:5878 322.51874:6938 | 3.54031E-06 |
| POS10869 | Lys Lys Leu Ala Gly | 5.359 | 530.3681 | [M+2H]2+ | 530.36603 | 59.04858:93051 69.77426:6928 73.02812:7024 73.06432:11558 85.06337:6334 87.04334:36853 87.07998:5721 89.05896:121367 101.0593:25695 103.07444:31208 117.0909:7133 133.08545:41338 147.10149:13551 364.68661:6553 | 3.90296E-06 |
| NEG3004 | N-Glyceryltaurine | 1.205 | 198.04619 | [M-H]- | 198.0442 | 79.05367:22192 81.06151:5960 161.03622:312986 163.03734:726720 164.03838:13700 198.04518:121900 | 1.00483E-05 |
| NEG242 | mino-2H-pyrrol-2 | 0.962 | 97.03882 | [M-H2O-H]- | 97.0408 | 50.8479:6218 79.05367:208009 80.05276:81134 81.286:4924 88.2065:5166 97.03745:211881 97.07645:435261 | -2.04038E-05 |

| Differences in metabolites between the Model group and the WJW group | | | | | | | |
|---|---|---|---|---|---|---|---|
| Alignment ID | Metabolite name | Rt(min) | Expreiment Mz | Adduct type | Reference m/z | MS/MS spectrum | PPM |
| POS6589 | CHEMBL2229478 | 5.069 | 302.21338 | [M+H]+ | 302.2114 | 55.05365:129180 57.03287:9962 57.06896:49742 59.04858:7324 61.01003:8355 67.05389:185932 69.06868:156592 71.04816:31271 71.08547:23748 79.05312:30386 81.06833:309800 82.07301:7658 83.0477:20895 83.08509:116967 85.06337:28484 85.10045:13174 91.0539:49071 93.06841:48134 95.08477:318163 96.08827:11507 97.06461:12086 97.09953:65192 99.07939:5623 105.06945:32571 107.08486:75967 109.06336:16497 109.10006:243459 110.103:11340 111.0796:8286 111.11481:15745 114.59279:5540 119.08333:76117 121.1013:35163 123.08004:23880 123.11523:69731 125.09565:11049 128.06834:25619 129.07336:16376 133.09863:98182 134.10684:6890 135.11646:15927 137.09427:30717 142.08385:6653 144.09827:8149 147.11681:47253 148.11829:5573 149.13002:19925 151.10901:10614 161.13197:18900 163.14494:33349 165.16396:237099 166.16684:15773 167.3136:6215 170.48605:6220 173.39111:6772 178.52345:5523 189.16042:86633 190.16454:6522 207.17024:25460 | 6.55171E-06 |
| POS9812 | Tobramycin | 4.847 | 485.29492 | [M+Na]+ | 485.29294 | 57.03287:15398 59.04859:273494 73.02812:14205 73.06432:10434 76.4834:5527 87.04335:649412 88.04605:9919 89.05896:235728 101.05931:130095 103.03849:171420 103.07444:66416 110.9791:5381 115.07378:9879 117.05281:83480 131.06766:16572 133.08545:75681 147.06319:8732 147.10149:25839 173.43526:10832 196.16579:6213 202.72192:6580 | 4.08001E-06 |
| POS12173 | NBD-dihydro-Cera | 5.691 | 578.39343 | [M+2H]2+ | 578.39111 | 55.05366:14753 57.03287:53323 59.04859:671995 60.05226:17840 69.06992:10379 73.02812:68294 73.06432:42721 80.0543:8628 81.0699:9920 85.06337:35636 87.04335:91351 87.06079:22020 87.07999:30506 89.05896:411190 90.06375:17210 94.06947:16227 96.50431:9577 99.07939:15179 101.05931:104704 103.07445:194301 115.07378:22064 117.05281:52219 117.0909:29782 119.23139:8095 129.08911:19768 131.06766:41126 131.10634:13377 133.08545:172080 143.10413:9771 147.10149:98957 161.11441:21054 177.11287:21305 191.1256:12890 205.14238:11902 429.96268:8644 | 4.01113E-06 |
| POS10293 | Val Phe Lys Asp | 6.067 | 508.27853 | [M+H-H2O]+ | 508.27649 | 60.08022:57792 69.06989:10630 86.09537:75225 104.10693:815147 105.10874:13218 124.9995:27646 158.71704:6638 184.07458:32309 335.11627:9683 | 4.01356E-06 |
| POS7450 | Nostocyclyne A | 7.302 | 343.26077 | [M+H]+ | 343.26279 | 55.31514:6243 56.21209:5842 70.43735:6475 92.06237:5683 124.23769:5623 | -5.8847E-06 |
| POS13266 | ethyl-5-methylide | 5.051 | 631.42297 | [2M+H]+ | 631.42041 | 57.03287:150773 59.04859:2764982 69.06869:38455 73.02812:91875 73.06432:60500 85.06337:66679 85.2186:31195 87.04335:632992 87.07999:84893 89.05896:2537144 90.06191:53195 99.08151:36128 101.05931:507606 103.03849:76130 103.07444:947555 105.09028:34377 107.07059:32935 115.07378:40858 117.05281:76758 117.0909:69998 129.08911:67531 131.06766:89552 133.08545:787532 145.08403:54317 147.10149:647766 161.11441:72138 177.1078:56457 191.1256:119385 264.8284:34663 | 4.05435E-06 |
| POS4831 | ene-1,3-disulfona | 1.009 | 236.99767 | [M+Na]+ | 236.99969 | 51.28637:5659 62.98058:33092 69.63676:6189 103.01153:22380 132.98657:34966 143.15192:5232 168.37367:5230 169.98517:21779 | -8.52322E-06 |
| POS11985 | Cymarine | 4.436 | 571.28479 | [M+Na]+ | 571.28717 | 67.41739:6578 132.3401:5945 146.061:6037 188.07004:25305 205.09822:8072 231.13576:6047 | -4.16603E-06 |
| POS6528 | xy-C12 homoserin | 5.084 | 300.21484 | [M+NH4]+ | 300.21689 | 55.53489:6784 57.06894:103329 61.96259:6471 70.06429:18750 71.08545:62549 82.065:18059 95.22827:5435 100.07379:159104 128.0683:699485 129.07332:21185 142.0838:174223 188.09213:79339 254.20921:7240 275.32065:6325 | -6.8284E-06 |
| POS13627 | ene\|21beta-angelo | 5.881 | 657.4751 | [M+H]+ | 657.47241 | 57.03287:56679 59.04858:643011 59.42949:8559 89.05895:18392 115.07378:17871 117.09089:104816 175.13507:13326 | 4.09143E-06 |
| POS9670 | -8,10-dioxapenta | 4.554 | 479.27475 | [M+2H]2+ | 479.27682 | 67.05389:8360 69.03294:12926 73.02811:43288 73.06432:9890 81.03229:12823 83.0477:9371 86.09541:21134 87.04334:67828 89.05896:387738 90.0619:11879 95.04893:30411 99.04339:18889 111.04189:45463 113.05927:14954 133.08543:146203 134.08684:9891 137.05981:10721 144.71318:6682 155.07083:10098 173.39111:9454 177.11285:7148 195.11966:8453 388.89212:7036 406.26993:7750 449.97394:7982 | -4.31901E-06 |
| POS12677 | tenacibactin H | 5.182 | 601.41455 | [M+H]+ | 601.41705 | 57.03287:78289 59.04859:1548720 73.02812:30158 73.06432:42032 85.06506:26493 87.04335:130026 87.07824:25916 89.05896:502687 101.05931:146764 103.07444:383406 115.07378:48419 117.05281:41343 117.08817:63891 131.07088:41107 133.08545:66329 147.10149:124154 161.11441:40724 173.42545:23346 417.47043:20268 | -4.15685E-06 |
| POS7201 | Epijasminoside A | 4.879 | 331.17203 | [M+Na]+ | 331.1741 | 59.04859:12242 81.48219:5523 89.05896:10799 98.88059:5631 107.7904:5492 161.48398:5151 331.17267:9964 | -6.25049E-06 |

**Differences in metabolites between the Model group and the WJW group**

| Alignment ID | Metabolite name | Rt(min) | Expreiment Mz | Adduct type | Reference m/z | MS/MS spectrum | PPM |
|---|---|---|---|---|---|---|---|
| POS13453 | NPTX-643 | 5.638 | 644.42688 | [M+2H]2+ | 644.42419 | 59.04856:99508 73.02808:12471 87.0433:26044 89.05892:105064 95.53635:9875 101.05926:14035 103.07439:34219 133.08539:24962 | 4.17427E-06 |
| NEG5909 | netamine L | 0.999 | 300.24249 | [M-H]- | 300.2446 | 72.52529:5988 84.68958:5811 93.03925:186879 95.03763:283775 97.07645:7399 110.58968:5112 124.15676:183779 182.18414:20774 291.46884:5531 | -7.0276E-06 |
| POS9813 | Pubeside B | 4.98 | 485.3064 | [M+2H]2+ | 485.3085 | 59.04858:79486 60.05225:6663 61.8371:6356 73.02812:27787 73.06432:7785 80.0543:15872 87.04334:33021 89.05896:162685 92.46779:5702 101.0593:23513 102.06606:12967 103.07444:18479 124.07726:7576 133.08545:59366 147.10149:10895 177.11285:5607 | -4.32714E-06 |
| NEG3613 | Cimaterol | 9.081 | 218.1279 | [M-H]- | 218.13 | 128.02194:71183 129.03217:6533 133.70459:5598 | -9.62729E-06 |
| POS5059 | -5-hydroxy-1-he | 4.501 | 245.1351 | [M+Na]+ | 245.133 | 58.06512:9572 86.09541:13427 122.86917:5221 143.72749:5680 146.99809:6036 173.43526:6088 218.00317:6136 237.56071:5529 245.13695:37659 | 8.56678E-06 |
| POS4725 | cero-pentulose) 1 | 5.566 | 233.09978 | [M+H]+ | 233.1019 | 55.01768:8584 57.03377:7507 57.06894:49350 57.18206:6569 58.07269:10056 67.05505:6206 81.03226:14436 83.08506:8660 85.06333:15404 103.12611:5356 111.04185:6677 112.5395:5665 121.06405:13022 125.05955:25938 126.06273:14620 167.10457:32994 167.83998:6425 168.11116:38598 173.43027:7378 183.91914:5373 | -9.09473E-06 |
| POS9459 | farneside A | 4.614 | 467.27295 | [M+H]+ | 467.27509 | 73.02812:51321 80.0543:16473 87.04335:104693 89.05896:517633 90.06191:29742 95.04894:19450 107.06821:13535 111.0419:32094 113.05927:15459 133.08545:160752 137.05637:15123 173.39111:37168 173.87758:17191 399.6514:12759 | -4.57974E-06 |
| POS13907 | eudoginsenoside F | 6.451 | 677.42059 | [M+H]+ | 677.42352 | 101.28879:8495 209.72971:9234 482.76108:9774 | -4.32521E-06 |
| POS13563 | ermoactinoamide | 6.305 | 651.4762 | M+CH3OH+H] | 651.47998 | 57.03379:42684 59.04956:13713 164.73628:5198 650.09869:5839 | -5.80217E-06 |
| NEG210 | 4-Pyridinol | 0.963 | 95.03929 | [M-H2O-H]- | 95.03712 | 58.87597:5863 90.95226:5704 95.03763:651935 | 2.28332E-05 |
| POS13492 | xy]-19-hydroxyurs | 4.853 | 647.41809 | [M+H]+ | 647.41528 | 59.04859:528102 73.02812:37374 85.06337:36406 87.04335:215381 89.05896:1493702 95.46825:25189 101.05931:125037 103.07444:212478 124.09804:30390 131.07088:38314 131.10634:28086 133.08545:545537 147.10149:101782 161.69572:23650 227.7915:22995 446.18515:25857 | 4.34034E-06 |
| POS7512 | s-19-Ene-1,2,4-T | 5.478 | 346.32935 | [M+H]+ | 346.33151 | 56.04953:18614 57.06989:9248 58.06512:15486 74.05886:12861 88.07445:24774 99.30854:6438 | -6.2368E-06 |
| POS12731 | 4,9,13,14-pentame | 5.551 | 604.38763 | [M+2H]2+ | 604.38501 | 59.04859:78126 67.015:9397 81.03072:6475 87.04335:8618 87.07824:6789 89.05896:85814 99.40212:5879 101.05931:13335 103.07445:23943 133.08545:24141 154.89267:5869 | 4.33499E-06 |
| POS4681 | benzo<a>fluoren | 4.446 | 231.11897 | [2M+H]+ | 231.11681 | 51.75735:6450 56.04863:14775 60.08025:29441 72.08048:25120 84.07958:13355 85.028:226357 86.09541:26358 100.07383:16396 231.12067:29570 | 9.34592E-06 |
| POS10946 | Taurocholic acid | 8.928 | 533.32312 | [M+NH4]+ | 533.32544 | 85.06337:13517 104.10696:45688 126.02023:120155 145.09904:11422 160.20474:11112 184.07466:33281 208.06328:16524 209.13087:24366 227.13577:17589 | -4.35006E-06 |
| POS9391 | yonarasterol G | 6.563 | 463.29947 | [M+H]+ | 463.2973 | 55.12037:6647 147.88623:5357 180.24678:5464 | 4.68382E-06 |
| POS11637 | taumycin A | 5.068 | 558.35614 | [M+2H]2+ | 558.3537 | 59.04859:317628 73.02812:77858 73.06432:28561 76.76718:17249 80.0543:37095 87.04335:342822 87.06254:27695 88.04605:28998 89.05896:611285 90.06191:18244 96.08221:17910 101.05931:127267 102.06606:28792 103.03849:18756 103.07444:54981 109.07315:19595 117.05281:42712 133.08545:194131 147.10149:32335 161.22427:18152 170.08043:20278 173.21472:22817 | 4.36999E-06 |
| POS8857 | Salicyloyl-conkurc | 5.156 | 433.28711 | [M+2H]2+ | 433.28491 | 57.03287:122648 59.04859:3019070 59.05931:77019 60.05226:74211 65.04931:33097 69.06992:24835 73.02812:264160 73.06432:66059 80.0543:46306 85.06337:83297 87.04335:410670 87.06254:135765 87.07999:58686 89.05896:2636898 90.06191:104028 94.06947:95750 101.05931:266741 102.06606:26984 103.07444:610018 109.0756:42692 115.07378:33155 116.08248:45168 117.0909:43486 129.08911:70544 131.06766:38930 133.08545:929040 134.09018:38699 138.09604:22047 145.08403:24259 147.10149:303882 177.11285:83305 191.1256:74282 | 5.07749E-06 |
| POS9946 | Morusimic acid C | 5.061 | 492.31949 | [M+2H]2+ | 492.31729 | 58.04042:32820 59.04859:673719 60.05226:22571 65.04819:30212 69.06992:13995 73.02812:150374 73.06432:40802 80.0543:70867 85.06337:26951 87.04335:162683 87.06254:76816 87.07999:25394 89.05896:1259871 90.06191:47778 101.05931:88979 102.06606:40417 103.07444:144945 107.06821:13208 109.07315:43866 117.0909:22869 124.08022:12762 129.08911:22447 131.06766:24791 133.08545:464620 134.09018:26379 138.09604:11087 140.09358:16099 147.10149:74442 169.11278:10513 177.11285:54784 191.1256:15896 | 4.46866E-06 |
| POS12237 | copadiol decanoat | 6.3 | 581.42255 | [2M+H]+ | 581.41998 | 57.03287:583029 59.04858:187706 81.0699:15293 97.06461:6372 99.07938:49343 101.09422:14097 115.07378:50363 117.09089:20887 157.12312:40594 173.3911:10101 | 4.42021E-06 |

**Differences in metabolites between the Model group and the WJW group**

| Alignment ID | Metabolite name | Rt(min) | Expreiment Mz | Adduct type | Reference m/z | MS/MS spectrum | PPM |
|---|---|---|---|---|---|---|---|
| POS13872 | Giganteumgenin D | 5.053 | 675.44958 | [M+H]+ | 675.44659 | 57.03287:53724 59.04859:1120360 73.02812:38305 87.04335:224956 87.07999:22732 89.05896:1106176 90.06191:21493 101.05931:168575 103.07444:380565 107.0254:19690 117.05553:24756 118.83812:18633 129.08911:21403 131.06766:41651 133.08545:349481 145.08403:19166 147.10149:165995 161.1188:22588 177.1078:20334 205.86365:21003 231.24901:24255 | 4.4267E-06 |
| NEG1724 | Histidine | 0.976 | 155.06726 | [M-H]- | 155.06947 | 58.04514:5773 59.83095:5668 79.0537:106586 80.66509:5785 81.14307:7922 87.11256:70161 93.15691:34894 95.03767:465257 97.03749:232436 97.07648:103337 111.15354:35922 120.89378:6292 121.51244:5309 137.19968:6037 151.60265:6598 154.24504:10632 | -1.42517E-05 |
| NEG1725 | Histidine | 1.079 | 155.06726 | [M-H2O-H]- | 155.06947 | 64.06541:5847 79.0537:191622 93.15691:22481 95.03767:519248 97.03749:252310 97.07648:109035 111.15354:21238 137.08247:9393 156.05357:9694 | -1.42517E-05 |
| NEG1570 | ropenyl)-5-vinylcy | 8.966 | 147.11578 | [M-H]- | 147.118 | 57.0112:14206 59.0295:55214 59.08405:27695 103.07276:20890 103.67512:5608 112.83764:5624 119.54141:5832 | -1.50899E-05 |
| POS11021 | Leu Tyr Val Lys | 5.063 | 536.34656 | [M+2H]2+ | 536.34418 | 58.04137:17266 59.04859:301936 73.02812:94452 73.06432:21392 80.0543:43703 85.06337:21301 87.04335:130609 87.06079:36121 87.07999:15848 89.05896:783413 90.06191:27599 101.05931:45697 102.06606:45551 103.07444:85751 109.0756:23540 117.0909:18548 124.08022:22265 129.08911:16082 131.06766:18971 133.08545:313664 134.08684:17615 147.10149:39057 173.42545:16622 177.11285:32828 | 4.43745E-06 |
| NEG2568 | 1-Undecanolactor | 8.285 | 183.14153 | [M-H]- | 183.1393 | 55.92466:5540 83.12405:23497 116.58435:5303 142.57878:5698 | 1.21765E-05 |
| POS13228 | Ferensimycin A | 5.773 | 629.42297 | [M+2H]2+ | 629.42578 | 57.03287:57295 59.04859:461322 69.06992:14182 73.02812:52708 73.06432:29320 85.06337:26788 87.04335:101916 87.06254:20498 87.07999:18603 89.05896:318566 94.06947:24179 101.05931:72536 103.07444:132810 115.07378:17623 129.08911:19577 133.08545:91906 141.32309:13970 147.10149:54603 193.21381:14327 | -4.46439E-06 |
| POS9449 | voretin C\|olovoreti | 5.967 | 466.34497 | [M+H]+ | 466.34271 | 53.00208:10126 55.05365:9939 57.03287:1568713 58.03662:138237 59.04858:423905 60.05225:27359 69.06992:14513 81.0699:41265 87.04334:8140 97.06461:21210 99.07939:98160 100.08458:16083 101.09641:17642 115.07378:97125 116.0771:20054 117.0909:35484 157.12312:76096 158.12614:31178 | 4.84622E-06 |
| POS8539 | Lys Lys His | 4.885 | 412.26434 | [M+2H]2+ | 412.2666 | 57.03287:26233 58.04042:51039 59.04859:530500 65.04819:22388 69.06869:9260 71.04816:10028 73.02812:211329 73.06432:27828 80.0543:117431 85.06337:26355 87.04335:191352 87.06079:60341 87.07999:11348 88.04783:9405 89.05896:1205068 90.06191:36132 101.05931:68762 102.06606:69439 103.03849:14021 103.07444:110287 107.07059:8640 109.0756:34035 111.07206:13151 117.08817:12291 124.08022:28268 129.08911:16022 131.06766:24895 133.08545:404612 134.08684:21362 140.09358:15864 147.10149:40341 173.43036:11455 177.11285:39558 | -5.48189E-06 |
| POS10244 | Ergosterol Acetate | 5.62 | 505.34656 | [M+2H]2+ | 505.34427 | 57.03287:66480 59.04859:1008826 60.05226:28304 69.06868:10573 73.02812:56892 73.06432:28375 85.06337:26026 87.04335:95828 87.06079:24889 87.07999:30373 89.05896:400585 90.06191:14719 94.06947:29000 99.07939:8330 101.05931:99646 103.07444:229806 104.07732:12755 109.0756:7315 115.07378:20926 117.05281:15195 117.0909:28429 129.08911:15724 131.07088:11503 131.10634:13149 133.08545:109593 147.10149:84254 161.11441:18592 205.14238:9040 | 4.53156E-06 |
| NEG6244 | 5-Diamino-eikosa | 1.852 | 311.34546 | [M-H]- | 311.3432 | 85.13134:7977 87.1126:11482 111.14101:55249 | 7.25887E-06 |
| POS9814 | Liquoric acid | 4.921 | 485.32938 | [M+H]+ | 485.32709 | 57.03287:125270 59.04859:2843541 59.05931:71617 60.05226:39477 69.06992:16250 73.02812:37788 73.06432:36680 81.0699:15102 85.06337:51433 87.04335:258376 87.07999:53761 89.05896:1359929 90.06191:23239 99.07939:14334 101.05931:256138 101.09423:18458 103.03849:49684 103.07444:781300 104.07732:24005 105.09028:15188 115.07378:46380 117.05281:66066 117.0909:57051 129.08911:19021 131.06766:26963 133.08545:268187 143.1078:19690 145.08403:15776 147.10149:276400 161.11441:52580 | 4.71847E-06 |
| POS8802 | 7,8-didehydro-8'- | 5.794 | 430.28433 | [M+H]+ | 430.28662 | 59.04859:7332 60.08025:14503 76.14551:6421 85.028:73655 86.03025:20533 89.05896:6834 165.22324:5583 173.43036:6525 318.39163:7013 | -5.32203E-06 |
| POS5532 | promethyl)chrome | 1.033 | 262.94507 | [M+H]+ | 262.94278 | 62.57782:6309 80.94772:11668 82.94373:53067 85.02795:14817 103.21838:7255 105.81583:6000 110.58839:5851 135.00177:100440 173.43027:5741 | 8.70912E-06 |
| POS9672 | Terragine D | 4.717 | 479.28229 | [M+H]+ | 479.28 | 62.54703:5555 89.05896:11415 98.65503:5455 193.53143:7135 243.06476:5573 | 4.778E-06 |

**Differences in metabolites between the Model group and the WJW group**

| Alignment ID | Metabolite name | Rt(min) | Expreiment Mz | Adduct type | Reference m/z | MS/MS spectrum | PPM |
|---|---|---|---|---|---|---|---|
| POS2839 | minoethylarsonic | 9.075 | 169.97691 | [M+H]+ | 169.9792 | 50.41959:6511 55.0177:8175 55.05365:12372 55.93345:449948 65.27977:5757 67.05389:7732 69.93349:5878 70.95692:38693 72.93706:21210 79.05312:9070 81.06989:5857 87.94499:8464 88.96877:12257 96.95995:72113 100.07382:6120 110.05835:6676 111.98266:7744 113.96311:13822 114.97044:527374 128.95062:102406 137.98456:17548 146.95981:36415 | -1.34722E-05 |
| POS10622 | nigramide I | 8.81 | 521.33966 | [2M+H]+ | 521.33728 | 50.45496:6193 51.83419:6802 60.08023:43348 67.37577:5809 86.09538:89886 95.44818:6482 104.10693:163753 105.10874:19782 124.9995:22076 128.15237:5616 184.07458:227376 185.07628:8154 195.01427:6031 | 4.56518E-06 |
| POS12076 | Cycloposine | 5.121 | 574.3764 | [M+H]+ | 574.37378 | 59.04858:79125 69.92596:5668 73.02811:8315 87.04333:146374 89.05895:88243 101.0593:47183 103.03848:7634 103.07443:16938 117.0528:7801 131.06764:7907 133.08543:24716 147.10147:7331 175.0853:6157 180.86687:5702 198.75192:5900 270.37485:5190 | 4.56149E-06 |
| NEG5636 | -2-enyl]cyclopent | 1.985 | 291.22513 | [M-H]- | 291.22742 | 60.69548:5592 77.46813:7272 108.77018:6596 126.02459:14571 163.03734:12093 167.73914:5356 285.31952:6500 | -7.86327E-06 |
| POS8630 | anodipyrido[1,2-A | 5.274 | 418.28296 | [M+H]2+ | 418.28528 | 55.05366:22078 57.03287:125034 59.04859:2525705 60.05226:57428 65.04819:25872 67.05389:10159 69.06868:20235 73.02812:148140 73.06432:31841 80.0543:9936 81.06834:12473 85.06337:42060 87.04335:225535 87.06254:50750 87.07999:43845 89.05896:1302131 90.06191:37053 94.06947:61475 99.07939:9880 101.05931:181628 103.07444:478159 104.07732:19685 107.06821:12884 109.07315:21801 115.07378:23777 116.08248:26758 117.05281:13252 117.0909:36600 129.08911:35198 130.08389:12682 131.07088:18517 131.10634:10495 133.08545:347346 134.09018:17711 143.1078:13060 145.08403:17356 147.10149:199987 148.1028:10108 161.11441:21964 173.11685:8694 177.1078:26012 191.1256:18382 | -5.54645E-06 |
| POS9392 | yonarasterol G | 4.971 | 463.29962 | [M+2H]2+ | 463.2973 | 58.04042:63295 59.04859:504811 65.04819:21997 73.02812:167294 73.06432:25138 80.0543:96970 85.06506:25089 87.04335:177305 87.06079:66151 89.05896:1213248 90.06191:44246 101.05931:69166 102.06606:88313 103.07444:117189 109.07315:38830 111.07206:15858 117.08817:17058 124.08022:34782 129.08911:15408 131.06766:18088 133.08545:425593 134.08684:19498 147.10149:65143 154.63217:12540 173.43036:18277 177.11285:50763 | 5.00758E-06 |
| POS8411 | Leu Lys Gln | 4.526 | 402.26868 | [M+NH4]+ | 402.271 | 59.04859:82599 73.06432:9263 85.06337:7422 87.04335:61379 89.05896:874148 90.06375:11985 101.05931:6852 103.07444:22527 107.06821:8809 124.58938:5510 130.08389:5964 131.06766:16379 133.08545:359856 134.09018:8764 147.10149:17029 177.11285:35710 241.19559:6116 | -5.76726E-06 |
| POS9847 | Ile Val Leu Glu | 4.639 | 487.31018 | [M+H]+ | 487.3125 | 59.04859:292906 73.02812:30849 73.06432:28687 79.13319:12788 85.06506:12807 87.04335:175797 89.05896:1561067 90.06191:30701 101.05931:34060 103.07444:83540 107.60078:10197 117.6151:9986 129.08911:19638 131.06766:23717 133.08545:549733 134.09018:23234 147.10149:41072 173.43036:22546 177.11285:47516 | -4.76081E-06 |
| POS13468 | Fucoxanthin | 5.319 | 645.41791 | [M+2H]2+ | 645.41492 | 59.04859:479514 73.02812:81248 73.06432:51689 87.04335:120059 89.05896:855098 101.05931:131167 103.07444:167082 131.10312:32544 133.08545:362154 134.09018:28192 147.10149:79929 173.44016:26547 177.11285:50734 | 4.63268E-06 |
| POS12195 | Hugonone B | 5.191 | 579.37061 | [M+Na]+ | 579.36792 | 59.04859:32268 87.04335:11722 89.05896:47033 96.43513:8825 114.28255:8750 133.08545:19594 274.88956:8878 | 4.64299E-06 |
| POS5381 | 4-hydroxyphenyl) | 4.875 | 257.15601 | [M+H]+ | 257.15369 | 55.05365:14169 57.06896:19251 67.05389:17654 69.06868:10464 71.08547:6875 77.8651:5469 81.06833:33341 88.07445:17903 95.08477:27614 102.09264:9084 119.08333:8294 137.1322:7120 | 9.02184E-06 |
| NEG6396 | cavernene B | 1.849 | 316.26703 | [M-H]- | 316.26471 | 96.82237:6153 148.11794:11831 182.01009:5761 | 7.33563E-06 |
| POS10173 | oxypoly-angioic ac | 4.92 | 502.35495 | [M+NH4]+ | 502.3526 | 57.03287:43776 59.04859:1181274 73.02812:24017 73.06432:28593 81.0699:9594 85.06337:19759 87.04335:112047 87.07999:18800 89.05896:666070 90.06191:15021 101.05931:129104 101.09423:14091 103.07444:446719 104.07732:10150 115.07378:16237 117.05281:87278 117.0909:38393 129.08911:10081 131.06766:36632 131.10634:12713 133.08545:169742 145.08403:12324 147.10149:206987 148.1028:8780 161.11441:54314 163.5305:8100 167.5183:10008 173.43036:15006 191.1256:24685 205.14238:19739 485.25378:8436 | 4.67799E-06 |

| Differences in metabolites between the Model group and the WJW group | | | | | | | |
|---|---|---|---|---|---|---|---|
| Alignment ID | Metabolite name | Rt(min) | Expreiment Mz | Adduct type | Reference m/z | MS/MS spectrum | PPM |
| POS12078 | Cycloposine | 5.857 | 574.37646 | [M+2H]2+ | 574.37378 | 57.03287:33910 59.04859:136280 73.02812:28360 81.03072:9795 85.06506:14046 87.04335:41005 87.07999:8481 89.05896:132471 101.05931:27133 103.07444:28370 115.07378:8781 129.08911:12956 133.08545:35650 147.10149:15380 | 4.66595E-06 |
| POS9634 | (-)-Asbestinine 2 | 5.142 | 477.31635 | [M+2H]2+ | 477.3187 | 59.04856:188902 60.05123:8391 73.02808:14107 73.06429:6009 85.06333:6872 87.0433:35492 87.06249:7482 89.05891:211438 90.06187:7440 101.05926:18100 103.07439:35720 117.0854:5972 118.5326:5493 133.08537:62620 147.10141:15339 256.90271:6885 462.67981:5708 | -4.92334E-06 |
| POS9491 | Ebastine | 5.095 | 469.30042 | [M+Na]+ | 469.29807 | 57.03287:26413 58.04137:13386 59.04859:331842 65.04819:7736 69.06992:9934 73.02812:68830 73.06432:19481 80.0543:23919 85.06337:13467 87.04335:816974 87.06254:31098 88.04605:14580 89.05896:377655 101.05931:311944 102.06384:21137 103.03849:9401 103.07444:63120 109.0756:10877 115.07378:10911 117.05281:29543 117.08817:7011 131.06766:15910 133.08545:112635 145.08403:6826 147.10149:23166 | 5.00748E-06 |
| POS9112 | CHEMBL470965 | 5.06 | 448.29343 | [M+2H]2+ | 448.29581 | 57.03287:68804 58.04137:27871 59.04859:1628296 60.05226:39454 65.04819:30631 69.06868:25025 73.02812:244775 73.06432:63531 80.0543:67590 85.06337:54377 87.04335:304086 87.06079:140329 87.07999:39268 87.56366:13261 88.04605:17000 89.05896:2262140 90.06191:80290 94.06947:36830 101.05931:156011 102.06606:47961 103.07444:331285 104.07732:16371 105.09028:10549 107.07059:17179 109.07315:66728 115.07378:15284 116.08248:23736 117.0909:23972 124.08022:24049 129.08911:43356 130.08389:22054 131.06766:42495 131.10634:17943 133.08545:784664 134.08684:42267 138.09604:9933 140.09358:13738 145.08403:15352 146.09132:11834 147.10149:176709 177.11285:79602 191.1256:33857 | -5.30899E-06 |
| POS10067 | Poricoic acid A | 5.496 | 498.33691 | [M+2H]2+ | 498.33453 | 57.03287:287249 59.04859:4624726 60.05226:121201 60.08025:118506 65.04819:79492 69.06992:81700 73.02812:449603 73.06432:126714 80.0543:89781 85.06337:157684 86.09541:264607 87.04335:567264 87.06079:199582 87.07999:106976 89.05896:3489491 90.06191:134469 94.06947:103107 101.05931:522818 102.06606:46573 103.07444:1024088 104.10697:1308416 105.10879:365901 106.11105:46620 109.0756:56872 115.07378:52093 117.05281:102652 117.0909:87329 124.99956:93343 129.08911:80034 131.06766:72005 133.08545:1056324 134.08684:51277 143.1078:55076 145.08778:54046 147.10149:505908 161.1188:83836 173.39111:83174 177.11285:126451 184.07466:896408 185.07635:309374 186.07535:146422 191.1256:114480 205.14238:48011 | 4.77591E-06 |
| POS8759 | Crustecdysone | 4.798 | 427.28738 | [M+H]+ | 427.285 | 57.03287:96412 59.04859:2660545 60.05226:41071 69.06992:13467 73.02812:38638 73.06432:30020 81.0699:8670 85.06337:43292 87.04335:254559 87.07999:30664 89.05896:1612168 90.06191:30564 99.07939:9460 101.05931:212539 101.09641:11841 103.03849:74057 103.07444:696734 104.07732:15618 105.09028:12907 107.07059:17053 115.07378:13781 117.05281:58399 117.0909:30087 129.08911:22180 131.06766:23475 131.10634:9323 133.08545:323802 143.10413:11097 145.08403:20325 147.10149:280810 161.11441:35992 191.1256:26189 | 5.57005E-06 |
| POS8592 | Salmeterol | 4.605 | 416.28442 | [M+NH4]+ | 416.2821 | 59.04859:257618 69.03294:18634 73.02812:10737 73.06432:9467 85.06337:8628 87.04335:70807 89.05896:1019619 90.06191:24733 101.05931:23922 103.07444:79191 107.07059:9047 117.05281:10679 131.07088:14556 133.08545:402513 134.09018:12916 147.10149:58041 173.39111:8539 177.11285:42085 191.1256:10972 | 5.57314E-06 |
| NEG5800 | ZINC2171653 | 8.938 | 296.97531 | [M-2H]2- | 296.97769 | 63.70482:5931 97.07649:38057 227.58353:6403 | -8.01407E-06 |
| POS11968 | ̶Leu Ala Pro Leu Gl | 5.879 | 570.35242 | [M+H]+ | 570.34967 | 59.04858:23768 66.82805:6713 72.18837:5650 86.0954:18019 87.04334:7782 89.05895:14613 94.83833:6230 101.0593:7966 104.10696:112184 146.98277:12615 249.88678:6454 301.56808:5957 | 4.8216E-06 |
| POS11188 | Pro Lys Val Lys Ala | 5.459 | 542.3634 | [2M+H]2+ | 542.36603 | 57.03287:86391 59.04859:1612797 60.05226:37101 65.04819:23288 69.06868:20449 73.02812:122944 73.06432:40164 80.0543:32205 83.04932:16424 85.06337:38825 87.04335:284572 87.06254:57144 87.07999:39721 89.05896:1251753 90.06191:29175 91.494:19192 94.06947:41465 101.05931:275969 102.06606:14246 103.07444:386000 104.07732:23684 109.07315:36497 116.0798:27127 117.05281:20293 117.0909:43102 129.09227:40122 131.06766:24424 133.08545:350447 134.08684:14581 147.10149:157161 148.1028:22169 161.1188:29090 177.11285:31342 191.1256:33280 267.83408:14932 311.62006:14301 328.30508:16016 407.45084:15379 | -4.84912E-06 |

**Differences in metabolites between the Model group and the WJW group**

| Alignment ID | Metabolite name | Rt(min) | Expreiment Mz | Adduct type | Reference m/z | MS/MS spectrum | PPM |
|---|---|---|---|---|---|---|---|
| NEG3242 | droxytrihomometh | 3.201 | 206.0881 | [M-H]- | 206.08569 | 59.08403:43411 69.11722:14541 73.11618:107827 80.05276:11726 99.1286:9273 101.14601:9415 126.02459:50621 161.03183:6211 161.23819:8132 | 1.16942E-05 |
| NEG2671 | Gongrine | 1.501 | 187.08127 | [M-H]- | 187.08369 | 58.0983:58602 62.21653:5617 79.05367:93919 89.09513:6340 97.08466:229408 97.18124:5655 114.16125:6013 115.15741:29718 123.03893:6622 125.22068:17593 125.24475:15819 127.20192:17880 145.23547:7066 | -1.29354E-05 |
| POS13903 | ahydrogambogic / | 4.624 | 677.39545 | [M+2H]2+ | 677.39215 | 73.02811:224730 77.35265:107361 87.04333:482475 89.05895:2727703 95.04893:120999 99.04338:114120 106.65066:121495 111.04189:180773 111.29109:94109 113.05926:113537 133.08543:1022934 177.11284:128923 182.38081:103717 432.59784:134147 | 4.87162E-06 |
| POS10256 | Ile Phe Val Lys | 5.247 | 506.33606 | [M+2H]2+ | 506.33359 | 57.03287:71605 59.04859:1823837 60.05226:56884 65.04819:17257 69.06992:25750 73.02812:167496 73.06432:57882 80.05276:35937 85.06337:52209 87.04335:315450 87.06079:100667 87.07999:59629 89.05896:1945502 90.06191:67040 94.06947:47589 99.07939:16286 101.05931:212129 103.03849:24585 103.07444:477548 104.07732:22879 105.09028:12167 107.06821:14019 109.0756:51116 115.07378:18939 116.08248:25256 117.0909:37772 127.07501:16344 129.08911:43925 131.06766:37075 133.08545:727718 134.08684:52970 138.09604:23348 140.09358:12996 143.1078:13173 145.08403:14221 147.10149:272258 148.1028:14220 161.1188:20799 173.11685:15015 177.11285:68870 191.1256:53325 | 4.87821E-06 |
| POS10996 | Cyclomicrosin | 5.97 | 535.39191 | [2M+H]+ | 535.38928 | 57.03287:347741 59.04858:111809 75.69939:6349 81.0699:7431 86.09711:7236 87.04334:6061 89.05895:13908 99.07938:32438 104.10696:6865 115.07378:24910 117.09089:13262 157.12312:41837 184.06929:20632 192.94875:5606 | 4.91231E-06 |
| POS10168 | Delaminomycin A | 5.361 | 502.31866 | [M+2H]2+ | 502.31619 | 57.03287:11288 59.04859:205091 60.05226:11892 67.015:23236 73.02812:8479 73.06432:15896 81.03072:8298 87.04335:23478 89.05896:155828 101.05931:16909 103.07445:53432 117.05281:17865 131.07088:13840 133.08545:41663 147.10149:13157 151.0811:10240 177.11287:6631 204.758:6350 316.92041:6433 | 4.91722E-06 |
| POS2577 | lonomethyl adipat | 2.601 | 161.07834 | [M+H]+ | 161.0808 | 56.94137:11350 67.67966:6202 72.93703:15146 105.0694:7747 115.05251:19043 117.05547:13777 118.06284:12438 132.07886:15843 132.97005:6489 160.0741:24901 161.07481:6579 | -1.52718E-05 |
| POS8986 | Grevirobstol B | 4.913 | 441.30276 | [M+H]+ | 441.30029 | 57.03287:91869 59.04859:2216200 60.05226:38474 69.06869:14908 73.02812:28853 73.06432:28137 85.06337:23678 87.04335:285232 87.07999:26585 89.05896:622334 90.06191:14456 99.07939:13362 101.05931:162838 101.09423:9388 103.03849:55808 103.07444:518486 104.07732:11131 107.07059:9778 115.07378:27765 117.05281:52568 117.0909:56797 129.09227:9778 131.06766:18558 133.08545:70772 147.10149:131341 161.11441:37325 | 5.5971E-06 |
| POS7812 | N-Palmitoyltaurine | 5.844 | 362.24283 | [M+H-H2O]+ | 362.2453 | 57.06989:6537 67.05389:14997 69.06992:8783 79.05312:19566 81.06834:11710 82.06503:43602 91.0539:13094 93.06841:20613 95.08477:9569 96.08018:11956 107.08486:11704 214.23677:6474 264.26468:32965 264.41238:5526 350.88223:5713 | -6.81858E-06 |
| POS13018 | -D-glucosyl betul | 5.182 | 618.43945 | [M+NH4]+ | 618.4364 | 59.04859:448789 73.06432:32302 87.04335:50417 89.05896:304779 90.06191:27372 101.05931:63464 103.07444:146189 117.05281:60982 117.0909:24036 131.07088:26206 133.08545:75904 147.10149:72940 163.85435:20030 173.39603:31478 474.44147:19340 | 4.93179E-06 |
| POS9508 | tumonoic acid G | 5.05 | 470.31366 | [M+H]+ | 470.31119 | 59.04856:210663 64.28428:6020 73.02809:39989 73.06429:12336 80.05426:16006 87.04331:50212 87.0625:18074 87.0782:6990 89.05892:304845 90.06371:9022 101.05927:28035 102.06602:6567 103.07439:47648 109.07555:7757 133.08539:95973 147.10143:14785 177.11278:14535 | 5.25184E-06 |
| NEG9597 | efpodoxime?proxe | 8.148 | 556.11475 | [M-H]- | 556.11749 | 58.03277:6804 95.15706:5698 370.90424:6061 | -4.92702E-06 |
| POS13883 | Stevastelin D3 | 4.552 | 676.38037 | [M+H]+ | 676.38373 | 69.03293:85440 87.04333:159569 89.05894:513421 89.77805:41723 99.04337:115176 133.08542:183022 155.06667:43362 173.43031:62566 270.54675:37789 544.49554:35321 | -4.96759E-06 |
| POS15117 | lucidanin | 6.195 | 795.54449 | [M+H]+ | 795.54053 | 51.78454:6742 59.04857:8131 | 4.97775E-06 |
| POS9269 | Chaxalactin B | 4.713 | 457.29761 | [M+H]+ | 457.2951 | 57.03287:16353 59.04859:529559 73.02812:14687 73.06432:12692 85.06506:15007 87.04335:97771 87.07999:15167 89.05896:994740 90.06191:21788 101.05931:51281 103.03849:11047 103.07444:149086 104.07732:7846 107.06821:9425 129.08911:11107 131.06766:10006 131.10634:8119 133.08545:293362 134.09018:9588 147.10149:73929 177.11285:19039 191.1256:8962 | 5.4888E-06 |

| Differences in metabolites between the Model group and the WJW group | | | | | | | |
|---|---|---|---|---|---|---|---|
| Alignment ID | Metabolite name | Rt(min) | Expreiment Mz | Adduct type | Reference m/z | MS/MS spectrum | PPM |
| POS12032 | PC-M4 | 5.227 | 572.37628 | [M+2H]2+ | 572.37341 | 57.03286:9966 59.04858:221267 73.02811:28340 73.06431:7596 85.06505:8617 87.04333:77892 87.06252:8328 87.07997:6421 89.05895:322147 90.0619:11336 94.06946:7232 99.61956:6140 101.0593:44944 102.06383:8446 103.07443:60197 104.10696:21277 109.07558:9611 115.77686:5817 131.06764:7148 133.08543:104042 134.08682:6520 147.10147:28222 177.11284:14357 184.07465:6833 415.7355:5473 | 5.01421E-06 |
| POS8799 | Mebeverine | 4.958 | 430.26196 | [M+2H]2+ | 430.25946 | 59.04859:51989 67.015:8720 86.36885:5594 87.04335:13277 89.05896:76175 101.05931:6941 103.07445:10649 131.06766:6788 133.08545:16632 135.28865:6161 173.43036:7527 | 5.81045E-06 |
| POS13293 | Scaphopetalumate | 5.659 | 633.40973 | [M+2H]2+ | 633.4129 | 59.04859:127985 80.32882:16383 87.04335:24626 89.05896:122135 101.05931:27054 103.07444:37583 103.53445:14955 133.08545:45083 147.10149:17551 162.54245:15598 173.39111:24998 189.11574:16272 209.53416:14642 | -5.00463E-06 |
| POS12430 | ngeoylzygadenin | 5.333 | 590.3717 | [2M+H]2+ | 590.36871 | 59.04859:142035 87.04335:29038 89.05896:142146 103.07444:33614 130.18591:17652 133.08545:65233 308.32666:18721 436.48868:16883 | 5.06463E-06 |
| NEG6717 | Schultesine | 1.055 | 329.15332 | [M-H]- | 329.15079 | 80.64178:6422 93.03928:74284 95.03767:32222 124.15681:27274 177.93195:6010 | 7.68645E-06 |
| POS11488 | Aliskiren-[d3] | 6.453 | 552.39777 | [M+Na]+ | 552.40057 | 60.08122:5983 72.73411:5317 73.3144:6683 76.72816:5385 104.10692:20045 139.75227:5697 184.06921:7460 | -5.06879E-06 |
| NEG9583 | (E)-Ceftriaxone | 6.016 | 553.03607 | [M-H]- | 553.03888 | 76.75741:7106 79.0537:6830 121.28275:5667 172.79352:5907 210.16214:6500 250.67091:6197 270.48953:5435 | -5.08102E-06 |
| POS7510 | thylnonanedioylca | 4.862 | 346.22552 | [M+2H]2+ | 346.22296 | 57.03287:29942 58.04042:76672 59.04859:875504 60.05226:16977 65.04819:46132 69.06992:7833 73.02812:301787 73.06432:24861 80.0543:87657 85.06337:31198 87.04335:155382 87.06079:98721 89.05896:1272129 90.06191:48954 94.06947:11967 101.05931:49615 102.06606:58963 103.07444:119498 107.07059:17606 109.07315:34147 124.08022:24388 129.08911:18888 131.06766:12967 133.08545:358139 134.08684:19133 147.10149:39424 177.11285:33961 | 7.39408E-06 |
| POS9239 | Ilekudinol A | 5.051 | 455.31937 | [M+H]+ | 455.3168 | 57.03287:102228 59.04859:1811090 60.05226:29341 69.06992:11679 73.02812:24976 73.06432:91429 85.06337:16261 87.04335:99042 87.07999:12841 89.05896:216625 90.06374:9159 99.07939:10766 101.05931:96706 101.09423:12097 103.03849:13055 103.07444:357020 115.07378:35759 117.05281:92245 117.0909:73378 131.06766:64145 133.08545:19462 147.10149:47996 161.11441:43254 | 5.64442E-06 |
| POS9028 | Nakijiquinone B | 5.157 | 444.27756 | M+CH3OH+H]2 | 444.27499 | 57.03286:10691 59.04858:120785 67.01498:18566 74.60526:5510 81.03071:8738 87.04333:11223 89.05894:108782 101.05929:7136 103.07442:25807 126.29716:5232 133.08542:32302 147.10147:10585 156.52419:6353 177.11282:6748 | 5.78471E-06 |
| NEG7495 | Sinigrin | 1.077 | 358.27255 | [M-H2O-H]- | 358.26999 | 52.36927:5581 68.06616:5950 93.03925:344479 95.03763:297239 97.03745:9331 124.15676:71886 153.06754:8821 182.18414:14831 239.79329:5500 | 7.14545E-06 |
| POS9551 | yl apo-6'-lycoper | 6.863 | 473.34387 | [M+H]+ | 473.34131 | 51.72378:6127 53.48267:6634 91.91269:5447 97.12214:6040 219.6438:5558 337.36746:5935 381.37701:5360 | 5.40836E-06 |
| POS10297 | PC(17:1(10Z)/0:0) | 6.412 | 508.33618 | [M+H]+ | 508.33881 | 55.6239:5924 59.04858:6614 60.08025:18263 86.09541:45672 104.10697:293443 105.1111:10635 124.99956:13625 148.10667:5865 173.38622:11353 184.06931:103155 295.92383:6376 | -5.17371E-06 |
| NEG4148 | pentadecanone C | 2.49 | 238.22028 | [M-H]- | 238.2177 | 101.31654:6837 108.03613:9828 108.22439:5969 124.04993:7581 180.11699:6444 | 1.08304E-05 |
| POS8350 | hylethyl)phenyl]su | 5.664 | 398.23859 | [M+Na]+ | 398.23599 | 59.04856:6062 71.14339:6012 130.37427:5976 | 6.52879E-06 |
| POS9933 | Herbasterol | 5.372 | 491.33173 | [M+2H]2+ | 491.33432 | 55.05366:26013 57.03287:120930 59.04859:2295520 60.05226:72763 65.04931:26636 69.06868:24696 73.02812:137599 73.06432:47595 80.05276:16213 81.06834:12172 83.0851:15285 85.06337:63016 87.04335:313542 87.06254:72873 87.07999:72223 88.04605:17042 89.05896:1561190 90.06191:56459 94.06947:70155 99.07939:11920 101.05931:283891 103.04074:16838 103.07444:528793 104.07732:29033 107.07059:11558 109.07315:26968 115.07378:31105 116.08248:30635 117.05281:20081 117.0909:46183 123.08884:12106 129.08911:44046 131.07088:32080 131.10634:29423 133.08545:521258 134.09018:28042 138.09604:18277 145.08403:25715 147.10149:286550 148.1028:17335 161.11441:33870 177.11285:45030 191.1256:49261 205.14238:11468 | -5.27136E-06 |
| POS8528 | Flabellinone | 5.051 | 411.2923 | M+CH3OH+H] | 411.2897 | 57.03287:44003 59.04859:859429 59.05931:20887 60.05226:12857 71.04816:8536 73.02812:24879 73.06432:171605 85.06337:7432 87.04335:100162 87.07999:7724 89.05896:129800 101.05931:40620 103.0385:28408 103.07445:135499 115.07378:9791 117.05281:193132 117.0909:28669 131.06766:88047 133.08545:30173 161.11441:9903 | 6.32158E-06 |

| Differences in metabolites between the Model group and the WJW group | | | | | | | |
|---|---|---|---|---|---|---|---|
| Alignment ID | Metabolite name | Rt(min) | Expreiment Mz | Adduct type | Reference m/z | MS/MS spectrum | PPM |
| NEG1841 | Tricholomic acid | 1.226 | 159.03804 | [M-H]- | 159.04059 | 55.08413:10747 59.08403:18302 69.11721:6679 73.11618:8054 99.07355:8781 159.03728:13510 | -1.60336E-05 |
| POS15116 | lucidanin | 6.192 | 795.53638 | [M+Na]+ | 795.54053 | 59.04858:13984 73.63771:5679 74.19592:5868 118.47446:5723 | -5.21658E-06 |
| POS11608 | Revandchinone 4 | 5.097 | 557.34216 | [M+H]+ | 557.34509 | 57.03287:23495 59.04859:229775 73.02812:38939 73.06432:14431 80.05276:16103 87.04335:946634 88.04783:16418 89.05896:252195 101.05931:259222 103.03849:54702 103.07444:55777 115.07378:17740 117.05281:41938 131.07088:24598 133.08545:89166 147.10149:29268 | -5.25707E-06 |
| POS5314 | Russulanorol | 5.197 | 255.15587 | [M+Na]+ | 255.15849 | 57.06988:6670 78.09728:6172 98.81725:5879 149.29048:5845 216.18271:5881 255.16071:8834 | -1.02681E-05 |
| POS10624 | cdysone-20,22-m | 6.142 | 521.35156 | [M+H]+ | 521.34882 | 51.77413:6985 56.04951:9219 60.08023:146083 69.06989:7297 71.07258:29433 86.09538:428126 87.09915:50395 95.08474:5568 98.98412:10856 104.10693:69240 105.10875:9331 124.99951:119508 126.00197:6355 184.0746:1123078 185.07629:177466 186.07527:13060 | 5.2556E-06 |
| POS7978 | lysylprolyllysine | 4.576 | 372.25784 | [M+H]+ | 372.2605 | 59.04859:448099 69.03294:16725 73.02812:11457 73.06432:9359 85.06337:15701 87.04335:73439 87.07999:7755 89.05896:1144522 90.06191:28135 101.05931:27742 103.07444:127799 105.09028:7020 107.06821:9930 117.05281:19430 129.08911:7743 131.07088:12052 133.08545:401910 134.08684:12480 147.10149:78159 151.09705:8489 177.11285:28023 191.1256:15673 | -7.14553E-06 |
| NEG4220 | decyltetrahydrothi | 1.413 | 241.20226 | [M-H]- | 241.1996 | 55.8771:6330 70.7528:6757 73.42717:5996 74.81602:5965 82.7948:5707 87.11253:6721 89.07528:10927 161.14594:13424 204.73056:5733 232.00209:6128 | 1.10282E-05 |
| NEG2928 | -Phenyl-L-cystein | 3.148 | 196.04639 | [M-H]- | 196.04372 | 99.32591:5903 153.19771:5391 153.42589:6134 161.03622:34233 161.28214:5408 | 1.36194E-05 |
| NEG2806 | hyl-6-(2-methylb | 5.066 | 191.15807 | [M-H]- | 191.1554 | 57.01118:21492 59.02947:116695 85.13128:6248 87.04276:7689 87.11253:11088 91.05847:6309 101.05437:13474 103.07272:208186 104.07503:7912 111.14092:14668 135.65366:5751 147.117:10377 | 1.39677E-05 |
| POS9867 | SCHEMBL1643131 | 5.969 | 488.32437 | [M+H]+ | 488.32169 | 59.04858:23033 64.29315:5737 82.54288:7031 89.05895:11043 94.77686:5356 101.0593:6085 151.42064:6293 | 5.48819E-06 |
| POS9650 | cyclo[10.8.0.02,9.04 | 6.48 | 478.32645 | [M+2H]2+ | 478.32913 | 92.10029:5338 271.37048:5825 308.74551:5511 | -5.60284E-06 |
| POS11187 | Pro Lys Val Lys Ala | 5.675 | 542.3631 | [M+H]+ | 542.36603 | 57.03284:35229 59.04856:470812 73.02808:47687 73.06429:21024 85.06333:14921 87.0433:71874 89.05891:394650 94.06942:17086 101.05926:67976 103.07439:118733 129.08905:13049 133.08537:92892 147.10141:43828 177.11278:14554 | -5.40226E-06 |
| POS14038 | MONENSIN | 5.599 | 688.45917 | [M+2H]2+ | 688.46289 | 59.04859:447999 73.02812:75560 85.06506:27413 87.04335:96532 87.06254:39067 89.05896:532809 101.05931:67741 103.07444:129273 133.08545:188542 147.10149:60605 177.11285:27077 | -5.40334E-06 |
| POS9549 | Val Ile Leu Asp | 5.261 | 473.29962 | M+CH3OH+H]2 | 473.29691 | 57.03287:13304 59.04859:167174 67.015:23341 73.02812:6643 81.03072:7557 87.04335:29208 89.05896:170959 101.05931:12395 103.07444:44551 133.08545:57064 147.10149:8679 158.09198:8681 | 5.72579E-06 |
| POS10743 | Axillarine C | 6.387 | 525.37146 | [M+H]+ | 525.36859 | 60.08024:31618 70.04918:5882 71.07259:7051 86.09539:95816 87.09917:9695 104.10695:40976 120.02661:5949 124.99953:23066 163.24342:5148 167.61609:5305 184.07463:315921 185.07632:48014 293.36038:6355 | 5.46283E-06 |
| POS11994 | bengamide G | 5.561 | 571.39209 | [M+2H]2+ | 571.3952 | 59.04856:119837 87.0433:16278 89.05892:99058 101.05926:20124 103.07439:30982 133.08539:21094 145.95105:6874 146.86797:6536 147.09758:7523 173.43027:15425 280.6557:6597 311.86816:7253 | -5.44282E-06 |
| POS11320 | seudotrienic acid | 6.138 | 547.37109 | [M+H]+ | 547.37408 | 69.62679:6764 86.0954:16902 88.90752:6739 104.10696:19850 116.29496:6737 168.67908:6925 184.07465:34999 185.07634:14910 186.07532:14030 318.24524:6938 476.40054:8121 | -5.46244E-06 |
| POS8117 | Methoxybutyrfenta | 5.846 | 381.25635 | [M+H]+ | 381.2536 | 55.05366:59197 56.04863:36764 57.06896:87857 58.07272:9338 67.04092:12889 67.05389:116800 68.04951:8904 68.05674:18427 69.06868:54600 70.06431:37694 70.07313:11344 71.08419:35277 79.05313:141014 80.05737:23471 81.05579:16697 81.06834:107621 82.06343:589187 83.06721:102276 83.0851:42505 84.08786:9734 85.10045:12190 91.0539:21022 93.06841:205623 94.07143:43152 95.04894:10832 95.08477:71566 96.08018:105786 97.0831:24824 97.09953:25755 98.98415:8786 107.08487:72153 108.08663:20951 109.10007:29213 110.09557:9670 121.09844:49051 122.10349:12156 123.11524:11544 135.11646:42561 136.11703:15726 149.13002:7634 263.25275:9608 264.26471:164125 265.27322:189890 266.2782:7267 | 7.21305E-06 |

**Differences in metabolites between the Model group and the WJW group**

| Alignment ID | Metabolite name | Rt(min) | Expreiment Mz | Adduct type | Reference m/z | MS/MS spectrum | PPM |
|---|---|---|---|---|---|---|---|
| POS9215 | o-teleocidin A1\|JB | 5.548 | 454.30905 | [M+2H]2+ | 454.3063 | 57.03287:18774 59.04859:295508 73.02812:18046 87.04335:17452 89.05896:89480 101.05931:15349 102.5617:5680 103.07444:37998 133.08545:14549 147.10149:7192 158.81593:5572 173.43036:11397 | 6.05318E-06 |
| POS13742 | voninin 6\|pavonini | 4.854 | 664.44543 | [M+NH4]+ | 664.44177 | 59.04859:245674 73.02812:21700 85.06506:16253 87.04335:104584 87.07999:14111 87.48628:13562 89.05896:727168 90.06375:20733 101.05931:49600 103.07444:99762 131.06766:14181 133.08545:315046 147.10149:67503 173.38132:15537 177.1078:44899 191.13127:18117 | 5.50838E-06 |
| NEG1702 | Antibiotic BN 227 | 1.207 | 154.05396 | [M-H]- | 154.05119 | 67.07545:6313 79.05367:305536 95.03962:13577 97.07645:243799 137.08243:14250 154.05191:25417 | 1.7981E-05 |
| NEG3554 | Maybridge4_00094 | 4.314 | 216.12827 | [M-H]- | 216.1255 | 54.79307:5975 62.37378:5944 62.44574:6431 126.02459:83638 127.03272:8735 128.0219:33050 131.66733:5889 | 1.28166E-05 |
| POS10096 | o-ethyl)-1H-indol | 5.052 | 499.34589 | [M+H]+ | 499.34311 | 57.03287:86547 59.04859:1682228 60.05226:28452 60.08025:22141 73.02812:27972 73.06432:34939 85.06506:20956 86.09541:22343 87.04335:225468 87.07999:22317 87.09918:10002 89.05896:368289 99.08151:16498 101.05931:141151 101.09641:13981 103.03849:26235 103.07444:394975 104.10697:155502 105.10879:48503 106.11105:12970 115.07378:30682 117.05281:36056 117.0909:66113 131.07088:29743 133.08545:50670 147.10149:88991 161.1188:40566 184.07466:38011 185.07635:29515 186.07535:21842 | 5.56731E-06 |
| POS9492 | Simplexin B | 5.947 | 469.31122 | [M+H]+ | 469.314 | 57.03286:13280 59.04857:8582 60.08023:42136 61.08377:5509 86.09539:78112 87.09917:7598 101.05711:5882 104.10695:351710 105.10876:52893 124.99953:13642 158.75143:5535 184.07462:122027 185.07631:24648 215.35905:5728 | -5.92354E-06 |
| POS9371 | S,14aS)-4b-deoxy | 8.922 | 462.26657 | [M+Na]+ | 462.26379 | 55.05365:11330 67.05389:7267 79.05312:6647 81.0699:21159 85.06337:14778 91.85783:5756 93.06841:17016 95.08477:15604 105.06945:18444 107.08486:28719 109.10006:12051 117.06913:8309 119.08612:16590 121.09843:11999 126.02023:204008 131.08377:6468 133.09863:20057 135.11646:11219 143.08206:5647 145.09904:15259 147.11681:16331 157.10197:13389 159.11723:23023 161.13197:7431 173.43034:8036 189.12691:6211 199.14366:13544 201.16266:6546 208.06328:19880 209.13087:18352 210.6722:5690 213.1633:9412 227.13577:9477 | 6.01388E-06 |
| NEG1138 | nopropyl)amino)e | 8.349 | 133.08339 | [M-H]- | 133.0806 | 59.12987:5186 65.09239:9815 73.30846:5403 74.24288:5666 101.05437:61494 102.54467:6000 116.05923:91887 117.06135:6233 133.08284:6874 | 2.09647E-05 |
| POS10148 | o-20(29)-en-3-ox | 4.727 | 501.3248 | [M+H]+ | 501.32199 | 59.04859:428126 73.02812:17540 73.06432:15485 85.06337:15767 87.04335:140079 87.07999:11619 89.05896:1095919 90.06191:26964 101.05931:57188 103.03849:10138 103.07444:128774 104.68195:7630 107.06821:11142 117.05281:7244 129.08911:13689 131.06766:23962 133.08545:371861 134.08684:7068 145.08403:7231 147.10149:74325 173.39111:7151 177.11285:22029 191.1256:8284 | 5.60518E-06 |
| POS11210 | Milbemycin A4 | 4.968 | 543.33673 | [M+H]+ | 543.33368 | 57.03287:14949 59.04859:239886 73.06432:11215 87.04335:780433 89.05896:221587 95.14056:9272 101.05931:168653 102.06384:12893 103.03849:121933 103.07444:70587 115.07378:11425 117.05281:73307 131.07088:21478 133.08545:66765 147.10149:23043 | 5.61349E-06 |
| POS4373 | hyl-4-oxochromer | 9.18 | 220.01317 | [M+H]+ | 220.01598 | 55.93344:24593 69.45864:6515 70.35236:5317 72.93571:6290 72.9598:36824 89.96286:5966 90.96998:15094 99.74139:5356 116.97396:24667 132.97009:16945 133.97685:15029 134.96141:6939 146.40657:5172 160.96077:8092 178.97508:7638 | -1.27718E-05 |
| POS10886 | 5,6R)-3,4,5-trihydr | 4.653 | 530.98499 | [M+H]+ | 530.98798 | 70.42718:6286 72.86754:5890 73.02811:7430 87.04333:17348 89.05894:109585 113.05925:5994 133.08542:36838 173.39598:9305 364.62674:6157 | -5.63101E-06 |
| POS10893 | Leu Asp Asn Arg | 4.448 | 531.2915 | [M+2H]2+ | 531.28851 | 72.4194:7773 86.09536:17901 87.05376:24342 89.05891:81013 133.08537:43158 146.06093:8085 188.06995:30052 303.14871:8317 | 5.62783E-06 |
| POS1494 | 4-Thiouracil | 0.761 | 129.01442 | [M+H-H2O]+ | 129.0116 | 55.05365:41874 56.04952:7266 59.04858:6643 68.98123:66361 69.82184:6053 69.9825:11398 82.01235:14051 82.06502:12911 83.00869:7963 84.07957:34310 86.99104:60373 87.99284:8064 100.02438:32180 101.02003:30250 105.0024:83125 106.00314:10898 110.2371:6091 118.03534:9650 119.0303:75677 123.0126:11470 128.01854:10268 129.01982:7432 | 2.18585E-05 |
| NEG166 | Glycerol | 0.634 | 91.04296 | [M-H]- | 91.0401 | 90.13486:71847 91.13872:16344 | 3.14147E-05 |
| POS12918 | melianol | 4.544 | 613.34045 | [M+Na]+ | 613.33698 | 52.72472:15674 57.68122:14530 59.89664:15405 64.38071:16020 69.0884:14292 86.0954:21295 135.45438:14429 148.67734:14215 311.24231:17300 | 5.65758E-06 |

| Differences in metabolites between the Model group and the WJW group | | | | | | | |
|---|---|---|---|---|---|---|---|
| Alignment ID | Metabolite name | Rt(min) | Expreiment Mz | Adduct type | Reference m/z | MS/MS spectrum | PPM |
| POS9268 | Communesin A | 4.548 | 457.2626 | [M+2H]2+ | 457.25977 | 69.03294:7465 73.02811:24149 83.04932:7884 87.04334:40421 89.05896:183975 95.04893:15484 99.04339:10666 111.04189:25470 113.05927:14135 133.08543:49846 155.07083:7130 168.79677:5700 173.42543:5884 | 6.18904E-06 |
| POS13291 | Rubuside D | 4.768 | 633.40399 | [M+H]+ | 633.40039 | 59.04859:129463 73.02812:15925 73.06432:14343 87.04335:100421 89.05896:635802 90.06191:15554 101.05931:27993 103.07444:34781 109.3968:10547 129.08911:10945 131.06766:19760 133.08545:258954 146.70763:11522 147.10149:23064 171.92455:11422 177.11285:28269 | 5.68361E-06 |
| POS10666 | Azadironol | 4.574 | 523.30011 | [M+2H]2+ | 523.3031 | 65.42842:22264 69.03294:42168 73.02811:122011 73.06432:36758 81.03229:60085 83.0477:36457 87.04334:258228 89.05896:1561065 90.0619:25113 91.04643:26116 95.04893:103995 99.04339:36849 111.04189:117303 113.05927:50583 131.07088:41791 133.08543:537202 134.08684:40688 137.0598:44873 151.09305:23499 155.07083:50347 157.08505:26813 177.10779:46777 178.6362:20443 | -5.71371E-06 |
| POS1205 | -Nitrosothiazolidi | 0.763 | 119.03017 | M+CH3OH+H] | 119.0273 | 53.03773:6029 55.05365:293886 55.93434:9302 56.05673:14151 56.94139:140906 57.05692:28162 58.5172:6224 59.04858:7664 72.08047:370484 72.93706:120281 73.08309:20993 74.95245:9104 77.8651:5377 90.94762:48955 91.34943:5251 101.02003:10175 119.0303:32603 | 2.41121E-05 |
| POS10476 | Delaminomycin B | 5.631 | 516.3349 | [M+2H]2+ | 516.33191 | 57.03379:10046 59.04858:86059 73.02812:6818 87.04334:6345 89.05896:65314 101.0593:11654 103.07444:22657 117.05553:6404 126.02024:28145 133.08545:22148 137.23573:6146 137.4812:5640 147.10149:9745 166.02432:6052 170.09471:5838 319.87314:5954 | 5.79085E-06 |
| POS8367 | ihydroxy-4,6-dim | 4.604 | 399.25629 | [M+H]+ | 399.25339 | 59.04859:1010931 73.02812:28224 73.06432:32070 85.06337:29569 87.04335:253725 87.07999:24881 89.05896:3000530 90.06191:61240 101.05931:73680 103.03849:27828 103.07444:237082 107.06821:20239 117.05281:16405 129.08911:20548 131.07088:33861 133.08545:866283 134.08684:26756 147.10149:118198 177.11285:54567 | 7.26356E-06 |
| POS9327 | Verdine | 4.611 | 460.30869 | [M+H]+ | 460.30579 | 59.04859:114620 69.03294:12691 73.06432:6779 74.03423:6222 87.04335:50910 89.05896:563675 90.06191:12397 98.87637:6070 101.05931:12596 103.07444:33731 107.07059:8782 117.36894:5773 131.06766:15377 133.08545:233407 134.09018:11356 147.10149:29190 173.43036:12408 177.11285:25475 | 6.30016E-06 |
| POS6728 | Paromomycin | 8.812 | 308.65833 | [M+Na]2+ | 308.65543 | 57.06989:43927 58.06511:11537 59.07198:10592 67.05271:10123 69.06868:6157 71.08418:22973 81.0699:6861 85.10045:8086 86.0954:13526 95.08476:15209 104.10696:9559 149.01665:5558 165.02727:9359 184.06929:6595 | 9.39559E-06 |
| POS8566 | Metachromin S | 4.905 | 414.30331 | [M+NH4]+ | 414.30042 | 57.03287:50975 59.04859:1296740 60.05226:20845 73.02812:16496 73.06432:173960 85.06506:6581 87.04335:32499 87.07999:13341 89.05896:289834 90.06191:7439 99.07939:11105 101.05931:68599 101.09423:10172 103.07445:356011 104.07732:7444 115.07378:12790 117.05281:405628 117.0909:62243 118.0574:9386 131.06766:159921 132.0724:6890 133.08545:18985 145.08403:11452 147.10149:83947 161.07928:16601 161.11441:45642 173.39111:11217 175.09528:13656 | 6.97561E-06 |
| POS12083 | esteryl-Phosphoch | 5.341 | 574.39618 | [M+2H]2+ | 574.39954 | 59.04858:74523 65.13841:8808 87.04334:16033 89.05896:105291 101.0593:19257 103.07444:17919 104.18001:8297 117.97754:7694 133.08545:39980 147.10149:14681 177.11285:9324 | -5.84959E-06 |
| POS11846 | Valnemulin | 5.139 | 565.36353 | [M+2H]2+ | 565.36688 | 59.04858:259007 73.02811:50874 73.06431:30974 78.36626:16107 80.05274:24239 87.04333:86028 89.05894:518801 101.05929:33520 103.07442:72740 123.34731:18975 133.08542:191946 147.10147:40567 173.43033:40313 177.11282:31289 194.32018:20204 446.87405:18802 | -5.92536E-06 |
| POS8515 | Marineosin A | 5.563 | 410.285 | [M+2H]2+ | 410.28201 | 57.03287:49031 59.04859:876259 60.05226:18268 65.04931:7682 67.85582:5822 73.02812:41810 73.06432:19938 85.06506:12180 87.04335:46344 87.06254:8629 87.07999:9658 89.05896:216433 90.06191:9081 94.06947:14511 101.05931:57353 103.07444:141593 105.61979:6301 115.07378:9548 116.08248:7404 117.0909:18444 131.07088:6858 133.08545:42086 147.10149:37057 161.1188:8715 173.39111:10399 185.21703:6740 195.57704:6851 211.2645:5911 | 7.28767E-06 |
| NEG2901 | ethyl-1-cyclohexer | 1.277 | 195.17252 | [M-H]- | 195.17551 | 59.08406:6652 75.09884:7763 79.05372:22833 97.07651:682352 159.03737:12622 161.03632:573850 196.04454:63048 | -1.53195E-05 |

**Differences in metabolites between the Model group and the WJW group**

| Alignment ID | Metabolite name | Rt(min) | Expreiment Mz | Adduct type | Reference m/z | MS/MS spectrum | PPM |
|---|---|---|---|---|---|---|---|
| POS13957 | horosyloxydocosar | 5.5 | 681.44598 | [M+2H]2+ | 681.44189 | 57.03287:42599 59.04859:477045 73.02812:59066 74.02876:28013 87.04335:110902 89.05896:615241 101.05931:83280 103.07444:203065 133.08545:260993 147.10149:91056 153.90381:33434 200.93614:29460 332.91391:27498 387.02127:27199 551.54889:30427 555.54846:31138 | 6.00198E-06 |
| POS11256 | ]-1-oxopropan-2- | 5.527 | 545.33667 | [M+H]+ | 545.33337 | 59.04856:17063 60.08022:9496 69.95734:5940 86.09537:31472 89.05892:17490 104.10693:201760 105.10874:26047 146.98271:20803 191.11984:5359 319.55377:6838 | 6.05134E-06 |
| NEG2410 | ylsulfanyl-butyl)-1 | 1.209 | 177.04959 | [M-H]- | 177.0526 | 79.05367:6822 119.17749:7333 142.03583:7854 | -1.70006E-05 |
| POS11727 | side, 3,17-dihydro | 6.137 | 561.33856 | [M+H]+ | 561.34198 | 59.04858:6808 71.33195:6263 89.05895:8051 98.42394:5281 104.10696:5405 539.50934:6287 | -6.09254E-06 |
| POS13108 | Antibiotic M 230B | 5.663 | 622.42749 | [M+H]+ | 622.43127 | 59.04856:123729 87.04331:19210 89.05893:108340 101.05927:22135 103.0744:32797 115.92144:9283 133.08539:26790 147.10143:9544 | -6.07296E-06 |
| POS7737 | Songorine | 4.504 | 358.24142 | [M+NH4]+ | 358.23837 | 59.04859:187338 73.06432:14123 87.04335:81153 89.05896:1400826 90.06191:26297 101.05931:5973 103.07444:37700 107.07059:15584 129.08911:7096 131.07088:12867 133.08545:486213 134.09018:12227 147.10149:31980 173.39603:8242 177.1078:51863 | 8.51388E-06 |
| POS12976 | Arg Leu Lys Trp | 5.233 | 616.39667 | [M+2H]2+ | 616.39288 | 57.03287:32646 59.04859:466780 73.02812:84005 73.06432:23669 80.05276:22272 85.06337:22002 87.04335:305077 87.07999:25052 88.04783:31059 89.05896:844076 90.06374:38897 101.05931:189509 103.07444:144583 117.0909:21604 133.08545:324054 134.08684:20407 145.08778:22085 147.10149:57823 173.43034:32907 175.88412:21732 177.11285:45332 191.1256:28014 424.63242:19913 | 6.14868E-06 |
| POS7780 | Lavoltidine | 5.053 | 360.24237 | [M+2H]2+ | 360.23929 | 57.03287:60582 58.04137:15193 59.04859:1675242 60.05226:41959 65.04819:49136 69.06868:12856 71.04945:7803 73.02812:166575 73.06432:21791 80.0543:17844 85.06337:32565 87.04335:150591 87.06079:69315 87.07999:19379 89.05896:1115052 90.06191:37919 94.06947:40386 101.05931:95564 102.06606:9818 103.07444:234132 104.07732:12819 105.09259:7544 107.07059:9096 109.07315:31309 116.08248:19999 117.0909:14133 129.08911:19810 131.08699:10183 133.08545:285434 147.10149:86939 177.11285:15536 191.1256:13700 | 8.54987E-06 |
| POS11259 | spicatin | 4.74 | 545.35059 | [M+H]+ | 545.34723 | 59.04859:622712 73.02812:57318 73.06432:44968 85.06506:20813 87.04335:345886 87.07999:33732 89.05896:2375766 89.07882:41091 90.06191:41952 101.05931:115064 103.07444:226217 107.07059:34198 129.08911:32429 130.08708:18221 131.07088:54070 133.08545:1103812 134.09018:32979 147.10149:182907 173.43526:23820 177.11285:122377 191.1256:26938 | 6.16121E-06 |
| POS12732 | ı-D-glucopyranos | 5.051 | 604.42444 | [M+H]+ | 604.42072 | 57.03287:33024 59.04859:715256 73.02812:24066 73.06432:21369 87.04335:79713 89.05896:540829 90.06191:24670 101.05931:93361 103.07444:301644 106.69563:20633 117.05281:69426 117.07185:24236 117.0909:29699 131.06766:39433 131.10634:25272 133.08545:178801 147.10149:147013 147.56622:18270 161.11441:37347 173.43034:49754 183.02257:18996 342.37314:23317 343.1636:21411 | 6.15465E-06 |
| POS9216 | )-Deoxymethymyc | 5.52 | 454.31253 | [M+2H]2+ | 454.31561 | 57.03287:81565 59.04859:1490384 60.05226:42113 65.04819:17652 69.06992:15052 73.02812:92610 73.06432:32519 80.0543:7485 83.0851:9630 85.06337:28999 87.04335:111027 87.06079:23074 87.07999:26781 89.05896:621674 90.06191:21255 94.06947:40236 99.07939:11675 101.05931:127551 103.07444:278122 104.07732:11744 115.07378:21818 117.05553:9391 117.0909:23259 129.08911:15064 131.06766:18057 131.10312:8823 133.08545:152206 145.08403:8057 147.10149:98787 161.11441:19346 173.43034:8036 177.1078:10809 191.1256:10621 | -6.77943E-06 |
| POS13580 | opyranosyl-6'-pim | 5.044 | 653.40875 | [M+H]+ | 653.40472 | 57.87836:7624 74.77715:7412 173.43031:12962 565.78345:6817 | 6.16769E-06 |
| POS10665 | Azadironol | 4.732 | 523.29987 | [M+H]+ | 523.3031 | 60.08022:20622 69.03291:6328 81.09808:5146 86.09536:34227 89.05891:9720 104.10692:177748 124.9995:9959 173.43027:11599 184.06921:65670 293.67346:5616 | -6.17233E-06 |
| POS1957 | Iodomethane | 1.23 | 142.9382 | [M+H]+ | 142.9351 | 55.01682:24101 55.05365:43290 56.94231:9903 57.06895:7810 64.52962:5774 68.04829:7093 69.03294:65214 70.06555:11197 70.07311:6937 71.04815:20688 83.04768:6460 97.02763:12463 98.05796:6868 143.03427:91947 | 2.16882E-05 |
| POS2846 | 2-Furoylglycine | 0.765 | 170.0405 | [M+NH4]+ | 170.04359 | 67.05389:5526 68.98123:128682 71.71257:6216 76.515:5693 81.06832:6663 82.01395:20593 83.00869:31886 86.99277:146306 100.02438:40441 101.01785:118621 102.01955:6718 105.0024:181483 118.03534:14167 119.0303:229980 120.02943:7976 123.0126:18484 123.46803:5846 128.01854:33603 129.01353:15591 137.03912:93193 142.03293:7856 147.02486:59909 165.03636:9211 | -1.81718E-05 |

**Differences in metabolites between the Model group and the WJW group**

| Alignment ID | Metabolite name | Rt(min) | Expreiment Mz | Adduct type | Reference m/z | MS/MS spectrum | PPM |
|---|---|---|---|---|---|---|---|
| NEG4317 | imycin: Oxazinom | 1.208 | 244.04961 | [M-H]- | 244.0464 | 79.05367:6909 97.07645:11114 163.03734:11778 175.2373:5440 | 1.31532E-05 |
| NEG5018 | -)-Quinolactacin A | 0.985 | 269.12595 | [M-H2O-H]- | 269.12909 | 59.08403:22810 89.13125:479299 90.13486:14166 93.03925:1773201 95.03763:1125557 151.06712:48152 153.06755:73754 | -1.16673E-05 |
| POS11669 | Cholyllysine | 4.829 | 559.36816 | [M+H]+ | 559.3717 | 57.03287:53343 59.04859:1266598 73.02812:47890 73.06432:36520 83.0477:18243 85.06337:49893 87.04335:307887 87.07999:49722 89.05896:2215204 90.06191:44290 101.05931:198639 103.07444:401994 107.07059:17771 117.05281:25782 129.08911:34704 131.06766:55594 133.08545:772419 134.08684:22090 145.08403:25622 147.10149:237818 164.1385:16358 173.43036:39536 177.1078:47093 191.1256:39333 372.55954:16853 | -6.32853E-06 |
| NEG1145 | 3-Trimethoxyprop | 8.741 | 133.08394 | [M-H]- | 133.0871 | 55.03414:5771 56.21574:6244 65.09239:15039 67.10966:7483 79.05367:8124 91.13873:7797 100.04587:14211 101.05437:131678 102.05606:12560 109.54619:6082 116.05923:190376 117.06135:15043 133.08284:8208 | -2.37438E-05 |
| POS10994 | )10-natafuranamir | 5.346 | 535.35645 | [2M+H]2+ | 535.35303 | 57.03287:58664 59.04859:1185472 60.05226:28537 69.06992:18596 73.02812:105121 73.06432:35161 85.06337:35816 87.04335:220489 87.06254:56060 87.07999:54007 89.05896:1072132 90.06191:31375 94.06947:34509 101.05931:145346 103.03849:19681 103.07444:333188 104.07732:15184 109.0756:17790 117.0909:30649 129.08911:23306 131.06766:17923 133.08545:354329 134.08684:24095 143.10413:15855 147.10149:166337 173.38622:19003 177.11285:38861 191.1256:25034 314.09201:16848 | 6.38831E-06 |
| POS10449 | 11-Ketofusidic acid | 4.823 | 515.34082 | [M+H]+ | 515.33752 | 57.03287:49450 59.04859:1449929 60.05226:28917 73.02812:46166 73.06432:39569 83.0477:12608 85.06337:38266 87.04335:237243 87.07999:34396 89.05896:1809246 90.06191:40554 101.05931:169273 103.03849:32397 103.07444:433003 104.07732:12532 115.07378:14554 117.05281:30054 117.0909:22782 129.08911:30253 131.07088:40680 133.08545:538518 134.08684:22924 145.08403:18900 147.10149:231683 161.11441:17324 177.11285:35046 191.1256:24259 338.48813:11314 | 6.40357E-06 |
| POS12665 | orodecane sulfoni | 7.882 | 600.93445 | [M+H]+ | 600.93829 | 68.48682:5486 90.24759:6837 173.43526:12346 293.1124:5001 369.65771:5913 554.7619:6490 | -6.39001E-06 |
| POS11690 | Chrysopentamine | 6.214 | 560.29828 | [M+2H]2+ | 560.30188 | 84.90689:5295 104.10469:17228 133.38271:6134 171.91002:5442 405.91846:5953 | -6.42511E-06 |
| POS3633 | Vitispirane | 5.393 | 193.16249 | [M+H]+ | 193.15924 | 55.05365:9474 58.06512:6990 60.2657:5893 67.05389:17243 79.05312:6183 81.06834:19681 93.07034:7061 95.08477:11928 105.06945:11578 109.10007:9376 147.11681:15425 147.72034:5289 | 1.68255E-05 |
| POS13035 | hydroxyl acyl DFC | 5.428 | 619.39294 | [M+2H]2+ | 619.39697 | 59.04859:85033 81.03072:14718 87.04335:23911 89.05896:107737 103.07445:20833 109.73177:12931 133.08545:35925 173.39111:25291 | -6.50633E-06 |
| POS12658 | yl)-deoxysphing-4 | 6.837 | 600.46539 | [M+H]+ | 600.46149 | 55.53491:6757 83.89444:5594 89.05896:6132 199.65767:5901 203.06952:5435 | 6.495E-06 |
| POS5740 | Diuron | 0.883 | 270.97675 | M+CH3OH+H] | 270.98001 | 57.06986:13553 71.08544:8173 79.61764:5963 135.00177:139311 263.71185:6056 | -1.20304E-05 |
| NEG3854 | Axinynitrile A | 2.089 | 228.17908 | [M-H]- | 228.1758 | 79.05367:26955 84.02768:5510 97.08672:9662 224.09412:6638 | 1.43749E-05 |
| POS5588 | Pentachloropheno | 0.958 | 264.85098 | M+CH3OH+H] | 264.85428 | 50.29755:6809 62.46424:5260 77.06998:5493 80.94775:1146872 81.04482:13340 82.94376:26061 83.67702:6314 84.91025:6728 90.97557:17328 104.10696:13956 122.07161:108671 138.90465:7942 144.04625:26688 | -1.24597E-05 |
| POS9168 | Z)-Eicosadienoylc | 5.645 | 452.3707 | [M+H]+ | 452.37399 | 50.29372:6425 55.05452:8946 60.08024:22619 69.0699:11333 83.08508:9886 85.02798:101310 173.43521:8354 196.08905:5390 260.33258:6729 | -7.27274E-06 |
| POS12412 | rmectin B2b aglyc | 4.601 | 589.341 | [2M+H]2+ | 589.3371 | 69.03294:85645 73.02811:270464 79.66347:74724 81.03229:100442 81.9725:68091 87.04334:605848 89.05896:3143008 90.0619:150195 95.04893:184828 99.04339:77337 111.04189:266398 113.05927:86412 133.08543:931530 137.0598:126393 155.07083:95403 155.12476:64704 199.10141:68832 | 6.6176E-06 |
| POS10671 | ydrokarounidiol di | 6.136 | 523.38159 | [M+H]+ | 523.37811 | 53.00208:11481 55.05365:13411 57.03287:1944042 58.03662:30775 59.04858:574645 69.06868:19389 81.06833:58211 86.0954:20230 97.06461:30371 99.07938:156839 101.09422:33453 104.10696:110452 115.07378:141694 117.09089:61504 157.12312:161664 184.06929:48328 | 6.64911E-06 |

| Differences in metabolites between the Model group and the WJW group | | | | | | | |
|---|---|---|---|---|---|---|---|
| Alignment ID | Metabolite name | Rt(min) | Expreiment Mz | Adduct type | Reference m/z | MS/MS spectrum | PPM |
| POS8125 | obutyryl bakkenoli | 5.055 | 382.25488 | [M+H]2+ | 382.25821 | 57.03287:59844 58.04137:17722 59.04859:1631943 59.05931:39530 60.05226:41665 65.04819:48386 69.06868:14164 71.04816:8664 73.02812:166879 73.06432:24983 74.03149:8137 80.0543:25562 85.06337:41089 87.04335:187063 87.06079:65798 87.07999:23344 87.56366:8138 89.05896:1267394 90.06191:33992 94.06947:29465 101.05931:98242 102.06606:12479 103.03849:10363 103.07444:233574 104.07732:12470 107.07059:12391 109.07315:29079 116.08248:14366 117.0909:17535 129.08911:21471 131.06766:19420 131.10634:10398 133.08545:342321 134.08684:11035 147.10149:90922 177.11285:26646 191.1256:12000 | -8.71139E-06 |
| NEG8927 | L-Triacontanediol( | 0.864 | 453.47113 | [M-H2O-H]- | 453.4678 | 76.92368:6228 84.98986:6183 86.25851:6162 113.12118:1495906 114.12465:34477 172.76915:6133 181.19069:44372 249.2543:6909 | 7.34341E-06 |
| POS9286 | adecahydro-3,5-d | 5.395 | 458.29333 | [M+2H]2+ | 458.29001 | 57.03379:7869 59.04859:141324 67.015:16456 73.06432:13343 81.02916:6543 87.04335:13792 89.05896:83653 101.05931:11880 103.07445:29819 117.05281:18938 131.06766:11334 133.08545:21246 147.10149:13494 158.092:5847 191.1256:6630 439.38196:6118 | 7.24432E-06 |
| POS15209 | Ganosinensin C | 6.569 | 815.55103 | [M+2H]2+ | 815.54559 | 52.05935:5830 397.22595:5531 610.95099:6742 | 6.67038E-06 |
| POS13386 | njaoamine B | 6.449 | 639.46747 | [2M+H]+ | 639.4632 | 53.00208:6937 57.03287:435875 59.04858:140883 73.37244:5391 81.0699:18838 97.06461:7991 99.07938:30953 101.09641:6964 110.6359:6188 115.07378:34385 117.09089:10878 157.12312:27488 489.76151:5649 | 6.67748E-06 |

**Differences in metabolites between the WJW group, the Model group and the Control group**

| Alignment ID | Metabolite name | Rt(min) | Expreiment Mz | Adduct type | Reference m/z | MS/MS spectrum | PPM |
|---|---|---|---|---|---|---|---|
| POS3961 | L-Tryptophan | 4.419 | 205.09682 | [M+H]+ | 205.09718 | 55.0177:28497 74.02328:473445 91.0539:335248 103.05421:28178 105.06946:27738 115.05257:692070 117.05825:161407 117.06913:685825 118.0629:11985257 118.43848:28942 119.06659:379179 130.06477:708654 131.07088:44960 132.07892:2374151 133.08215:83645 142.06567:905584 143.07104:1194480 144.06113:83329 144.0797:4341700 145.06152:115992 146.05721:17431700 146.54747:29480 146.57033:23767 147.06317:678492 155.0584:63157 159.09137:1192681 160.07417:256210 170.05661:862231 171.0612:37257 188.07004:2923840 189.07106:109007 | -1.7553E-06 |
| POS2612 | L-Carnitine | 1.011 | 162.11203 | [M+H]+ | 162.11247 | 55.05363:6803 57.03284:158120 58.06414:36314 59.07293:35311 60.08022:815004 61.02843:9713 84.08119:18494 85.02795:386671 102.09037:217084 103.03844:486962 104.04307:7802 162.11153:194240 | -2.7142E-06 |
| POS3448 | lquinolinium-2-ca | 4.418 | 188.07056 | [M+H]+ | 188.0705 | 91.0539:698427 103.05421:33981 115.05257:797674 116.06099:47668 117.06913:479114 118.0629:8225772 119.06659:228830 128.04967:38424 132.07892:89223 142.06566:594714 143.07103:1165095 144.07968:1851898 145.06526:103181 146.05721:5633082 147.06317:168062 155.0584:95603 160.07417:64165 170.06137:422132 188.07004:287089 | 3.19029E-07 |
| POS4386 | Pantothenic acid | 4.396 | 220.11717 | [M+H]+ | 220.11798 | 55.0177:49954 55.05365:39474 56.01259:12366 57.06895:303380 59.04858:241967 60.04425:13005 61.02845:8034 67.05389:184512 69.06867:181612 70.02779:221788 72.04369:495929 73.02811:119645 74.02328:43455 81.06832:15058 83.04769:30535 85.06336:264051 86.0954:52130 87.07997:42329 90.05456:1912377 91.05762:28719 95.04893:165012 96.08018:52378 98.02253:384394 100.03728:43983 103.07443:131626 113.05926:48921 116.03413:187566 124.07427:344107 125.07761:14548 131.06764:15485 142.08383:103294 156.10028:10172 160.0959:19318 166.08405:18475 174.10922:13004 184.0961:69153 202.10344:51304 220.11993:8643 | -3.6798E-06 |
| POS2752 | Phenylalanine | 4.381 | 166.08542 | [M+H]+ | 166.08627 | 51.02314:20908 53.03856:12102 77.03801:251645 79.05312:825183 80.04813:27428 91.05389:491642 93.0684:1242536 94.06358:30112 95.04893:214143 102.04611:26303 103.0542:4972212 104.05679:101402 105.044:23711 105.06944:23312 107.04916:486048 118.06288:64796 119.07215:44825 120.08028:14988410 121.08411:298965 131.04831:132634 | -5.1178E-06 |
| POS1294 | Niacinamide | 1.644 | 123.05486 | [M+H]+ | 123.05528 | 51.02314:10542 53.03856:116635 56.04952:6667 61.03869:8044 67.05389:11350 68.0495:16272 77.03802:8107 77.45795:6885 78.03359:75791 79.05312:6839 80.04813:999731 81.06989:6655 95.04893:65126 96.04379:265895 105.04401:14387 106.02892:33624 123.05364:805984 124.03867:21063 | -3.4131E-06 |

**Differences in metabolites between the WJW group, the Model group and the Control group**

| Alignment ID | Metabolite name | Rt(min) | Expreiment Mz | Adduct type | Reference m/z | MS/MS spectrum | PPM |
|---|---|---|---|---|---|---|---|
| POS5974 | nonadienyl)-3-me | 6.165 | 279.23041 | [M+H]+ | 279.23169 | 53.03856:8408 55.05364:126356 57.06895:28213 65.03803:10419 67.05388:1126282 68.05672:28635 69.06866:117076 71.04815:14044 79.05311:77584 81.06831:947627 82.07299:28072 83.04768:14836 83.08508:58576 85.06504:13836 91.05389:23458 93.06839:89080 95.08475:784001 96.08826:25010 97.09951:25132 105.06944:23707 107.08484:69139 109.06334:16580 109.10004:245262 110.10298:14125 117.06911:12139 119.08331:21450 121.10127:40799 123.11521:94882 131.08374:32588 133.1019:15427 135.11642:38478 137.13217:49228 145.09901:20479 147.11679:15227 149.12999:24142 151.14888:9695 159.1172:11989 163.14491:13367 173.13148:21904 173.43031:9482 | -4.584E-06 |
| POS8713 | Linoleoylcarnitine | 5.468 | 424.34036 | [M+H]+ | 424.34207 | 55.05364:150847 57.03286:65854 57.06895:33783 59.04857:30031 60.08024:486717 67.05388:45969 69.06866:195045 71.08546:8042 81.06831:63957 83.08508:104109 85.02798:1963085 86.03023:37237 89.05894:14804 91.05389:10072 93.06839:18013 95.08475:57991 97.06459:9392 97.09951:58719 99.07937:8237 105.06944:7259 107.08484:11790 109.10004:30690 111.07958:8035 111.11478:21744 119.08331:7051 121.10127:10638 123.11521:13291 133.0986:8555 135.11642:11354 137.13219:10337 139.1123:6562 144.10194:39773 147.11679:10579 161.13194:12289 173.39598:7408 | -4.0298E-06 |
| POS769 | Choline | 1.002 | 104.10676 | [M]+ | 104.10699 | 58.06511:824339 59.07295:236863 60.08025:4750052 104.10696:4088644 | -2.2093E-06 |
| POS1626 | Isoleucine | 1.185 | 132.10175 | [M+H]+ | 132.10188 | 53.01286:7055 55.0177:13773 55.05365:14316 56.04953:22212 56.94233:10269 57.05693:51299 58.06417:43433 61.01003:17182 67.05389:8534 69.06868:586708 72.04369:7239 73.06432:10863 86.09541:3246155 87.02591:7895 87.09918:15027 90.05457:13104 132.10175:10606 | -9.8409E-07 |
| POS6396 | nylalanylglutamic | 4.45 | 295.12854 | [M+H]+ | 295.12851 | 56.04862:20747 59.04858:5933 79.05463:6232 84.04315:468398 85.04819:6074 91.05389:7079 93.0684:10304 102.05276:13654 103.05196:53477 107.04916:26937 120.08028:1456756 121.08411:56163 130.04883:64280 131.04831:67332 136.0761:13332 149.05962:22893 166.08405:156041 167.08606:7732 186.09166:34363 232.09715:7410 | 1.01651E-07 |
| POS3991 | Indolelactic acid | 4.77 | 206.08014 | [M+H]+ | 206.08121 | 57.06896:30889 67.28918:6299 91.0539:24944 99.71146:5661 103.05421:7142 115.05257:62846 117.05553:47575 118.0629:999360 119.06658:34519 130.06477:564182 131.07088:69125 132.07892:194949 133.08214:8784 142.06566:70979 143.07103:41488 144.07968:99930 146.05721:268826 147.06317:11807 160.07417:155455 170.05659:153698 171.0612:10913 188.07002:80092 194.39003:5958 206.07948:14975 | -5.1921E-06 |
| POS5962 | Dibutyl phthalate | 6.414 | 279.15845 | [M+H]+ | 279.15909 | 57.06896:458324 58.07272:10673 65.03804:35537 67.05389:8404 81.0699:8269 88.65063:6869 91.05203:5475 93.03178:13287 107.08248:6126 121.02692:82243 149.02057:2416805 150.02579:107710 151.60489:5687 167.03043:6447 | -2.2926E-06 |
| POS2172 | Phthalic anhydride | 6.414 | 149.02289 | [M+H]+ | 149.02328 | 65.03804:511486 84.07957:7042 93.03371:50982 111.0444:60191 121.02691:156586 132.55287:5859 149.02055:222848 150.02579:6832 | -2.617E-06 |

**Differences in metabolites between the WJW group, the Model group and the Control group**

| Alignment ID | Metabolite name | Rt(min) | Expreiment Mz | Adduct type | Reference m/z | MS/MS spectrum | PPM |
|---|---|---|---|---|---|---|---|
| POS9985 | LysoPC(16:1/0:0) | 6.086 | 494.32361 | [M+H]+ | 494.32468 | 59.04858:17369 60.08025:150052 69.06991:6945 71.0726:15452 73.02811:8417 86.0954:400958 87.09917:8035 89.05895:17857 95.90849:7302 98.98414:8216 101.0593:7508 104.10696:89747 124.99955:112934 184.07465:1936991 185.07634:52290 | -2.1646E-06 |
| POS2112 | D-Glutamine | 1.094 | 147.07629 | [M+H]+ | 147.0764 | 55.05365:24853 56.04862:358514 57.03286:10764 58.06511:15396 60.08024:37261 67.05389:20696 69.03294:7830 72.08047:11212 74.02328:23964 82.06502:8848 84.04315:2728692 84.07294:49023 84.07957:583470 85.02799:47516 85.04652:17991 86.05938:28923 87.04333:69607 91.05389:7809 101.07021:39437 102.05498:54518 130.04883:350584 130.08388:31960 | -7.4791E-07 |
| POS3931 | DL-Acetylcarnitine | 1.064 | 204.12248 | [M+H]+ | 204.1232 | 57.03286:122207 58.06511:24310 60.08024:668239 85.02798:3912352 86.03195:55965 144.10196:60948 145.05025:75050 204.1239:36013 | -3.5273E-06 |
| POS4054 | cetyl-DL-phenylala | 4.736 | 208.09615 | [M+H]+ | 208.09679 | 53.41887:5633 57.52442:6482 77.038:6865 79.05311:5655 86.17436:6026 91.05388:7406 93.06838:21384 103.05419:54353 107.04677:9415 118.06287:6317 120.06332:33234 120.08028:1588035 121.0841:55192 131.04829:15870 162.0894:10263 166.08403:36842 173.38617:11598 | -3.0755E-06 |
| POS1617 | Creatine | 1.02 | 132.07649 | [M+H]+ | 132.07678 | 58.06512:10415 68.0483:15458 70.06557:11843 71.04816:6014 72.0542:10250 85.0836:5968 86.05939:18722 87.05381:50090 87.07824:7657 90.05457:746073 114.06509:16834 115.04728:7870 132.07567:150401 | -2.1957E-06 |
| POS5117 | mma-Glutamylvali | 4.382 | 247.12816 | [M+H]+ | 247.1288 | 55.05365:108200 56.04862:40411 57.03287:7214 69.06992:7648 72.08047:1399870 73.0831:28712 79.05312:10563 83.0477:12338 84.04316:628411 85.02799:8975 85.04652:13994 86.09541:41765 88.03895:16447 102.05276:15880 107.04679:23549 118.08494:251978 119.08612:7749 130.04884:85159 138.08907:11043 141.09964:7876 156.1003:28288 184.09612:60439 230.1053:8228 | -2.5897E-06 |
| POS1578 | -Dihydronaphthale | 4.479 | 131.08511 | [M+CH3OH+H]+ | 131.08553 | 53.03773:26525 56.04953:10752 57.06896:28708 65.03805:28931 67.05389:8711 69.06868:6259 74.09583:8711 84.04316:19602 84.07957:34050 85.0836:6501 86.09541:22325 87.00324:18490 88.00348:8670 91.0539:1673423 92.05669:10880 95.04894:8214 97.00918:26390 103.05421:41006 105.06946:7580 115.05257:62450 116.061:176421 128.06212:19383 129.06706:119311 130.06477:31461 130.15721:9880 131.08377:775724 132.08543:8337 133.06566:6404 | -3.204E-06 |
| POS10715 | LysoPC(18:0/0:0) | 8.808 | 524.36963 | [M+H]+ | 524.37109 | 54.19788:89428 57.03287:95803 57.06989:193802 58.06512:106852 60.08025:1369087 66.97967:87274 71.07261:174615 81.0699:101604 86.09541:3593029 98.24385:89434 104.10697:20061236 105.10879:462525 124.99956:987458 163.01524:135640 184.07466:20082530 185.07635:541792 232.21115:92139 258.10977:97197 288.29056:88176 | -2.7843E-06 |
| POS3512 | lole-3-propionic a | 5.052 | 190.08539 | [M+H]+ | 190.08626 | 55.0177:842102 56.02069:8567 57.03379:6661 67.05389:15577 73.02811:9550 91.05389:18599 103.05421:11714 105.03245:17434 105.06945:8788 107.04917:50390 108.05043:9465 130.06477:2285724 131.06764:84330 144.07968:18119 172.07471:75956 190.08571:14212 | -4.5769E-06 |
| POS1544 | Pipecolic acid | 0.825 | 130.08577 | [M+H]+ | 130.08626 | 56.0486:24527 60.60572:5781 84.07954:132334 99.51071:5700 | -3.7667E-06 |

**Differences in metabolites between the WJW group, the Model group and the Control group**

| Alignment ID | Metabolite name | Rt(min) | Expreiment Mz | Adduct type | Reference m/z | MS/MS spectrum | PPM |
|---|---|---|---|---|---|---|---|
| POS2185 | rans-Cinnamic aci | 4.381 | 149.05949 | [M+H]+ | 149.05991 | 53.32008:5990 56.04953:9270 65.03805:7397 69.61556:6564 74.02328:8517 77.03802:30442 79.05313:145573 79.54906:5949 84.95901:7244 91.0539:36205 93.06841:62515 95.04894:47393 102.09042:7023 103.05422:290493 105.04402:15037 105.07177:6210 107.04918:37790 121.0641:15924 131.04832:7204 149.05965:5875 | -2.8177E-06 |
| POS2121 | Lysine | 0.829 | 147.1127 | [M+H]+ | 147.11278 | 55.05366:25488 56.04863:205512 58.06512:10718 64.97839:8676 67.05389:71518 68.98124:25102 72.08048:107123 74.02328:9653 84.07958:2397784 85.06337:13968 85.0836:18732 86.99279:33388 105.00241:33210 112.11258:14540 119.03033:16718 130.08389:109110 | -5.438E-07 |
| POS1867 | Betaine | 1.004 | 140.06779 | [M+Na]+ | 140.06822 | 52.9996:41083 58.06512:19829 74.09583:3209492 75.099:20457 80.94776:159718 80.99314:24787 81.05422:29478 82.06183:23694 82.94377:114697 96.07816:16111 114.08864:279681 140.06865:1283927 | -3.0699E-06 |
| POS9113 | rachidonoylcarniti | 5.458 | 448.34097 | [M+H]+ | 448.34207 | 55.05365:37030 57.03287:20498 57.06896:17059 59.04858:34023 60.08025:209223 67.05389:89922 69.06868:53369 73.02811:6666 79.05312:38076 81.06833:79266 83.08509:13748 85.02799:487630 86.03024:7335 87.04334:7280 89.05896:27869 91.0539:16592 93.06841:61372 95.04893:7555 95.08477:71604 105.06945:14911 107.08486:28129 109.10006:22696 119.08333:21630 121.1013:34323 123.11523:10586 131.08377:9976 133.10193:13706 135.11644:14254 144.10197:22296 145.09904:6277 147.11681:6546 149.13002:6498 157.05124:8175 166.11165:5622 | -2.4535E-06 |
| POS5318 | Actinopolysporin C | 6.075 | 255.2303 | [M+H]+ | 255.23151 | 55.0177:7581 55.05365:215649 57.03287:22659 57.06896:267334 59.04858:9281 67.05389:59720 69.06868:223862 71.04945:11979 71.08547:109749 73.06432:8417 78.87981:6127 79.05312:31609 81.06834:96833 83.04932:8425 83.08509:156102 85.06337:6585 85.10045:31519 93.06841:68014 95.08477:76990 96.08625:6932 97.06461:39662 97.09953:93929 99.07939:6303 101.05712:9242 107.08486:62857 109.10006:45041 111.0796:24489 111.11481:18312 121.09843:56798 123.11523:17064 125.09565:19197 135.11646:56832 137.1322:6945 139.11234:13492 149.13002:27740 153.12358:9400 157.46205:6511 163.14494:11539 167.14175:8147 219.21098:7793 | -4.7408E-06 |
| POS6276 | Caryophyllen-beta | 4.377 | 290.15833 | [M+H]+ | 290.15979 | 55.05365:24601 56.04862:7940 57.03287:16604 60.08025:107724 70.0643:14590 70.43227:5768 72.08047:8247 81.56439:5745 83.0477:18170 84.07957:26752 85.02799:330220 86.03024:6849 86.09541:9647 101.0593:57444 103.04073:5965 111.04189:107845 129.05446:47478 144.10197:12880 147.06317:9623 | -5.0317E-06 |
| POS1531 | Pyroglutamic acid | 1.096 | 130.04965 | [M+H]+ | 130.05009 | 55.05453:7494 56.04862:128944 58.06511:10979 67.05389:10890 70.0643:61359 74.02328:5937 83.05907:29290 84.04315:318672 84.07957:532844 85.02799:13483 86.05938:34163 102.63087:5424 119.02752:8836 130.04883:17137 130.08388:22824 | -3.3833E-06 |

**Differences in metabolites between the WJW group, the Model group and the Control group**

| Alignment ID | Metabolite name | Rt(min) | Expreiment Mz | Adduct type | Reference m/z | MS/MS spectrum | PPM |
|---|---|---|---|---|---|---|---|
| POS1441 | Thymine | 4.432 | 127.04982 | [M+H]+ | 127.05018 | 52.01421:6341 53.03857:8442 54.03373:44442 55.01771:6053 56.04863:31362 75.04592:5839 81.04327:7179 82.02832:13058 84.04317:16026 109.03891:11158 110.02366:31838 127.05041:24780 | -2.8335E-06 |
| POS2506 | -Naphthalenediam | 4.419 | 159.09103 | [M+H]+ | 159.0916 | 55.05366:12243 55.93345:53946 56.04953:12704 60.32402:6182 67.05389:16534 69.06992:16412 70.06431:17375 71.04816:7946 72.93573:18955 84.07957:6821 91.0539:13916 95.08477:25721 100.07598:7385 105.06946:21656 112.11003:13647 114.97046:14039 115.05257:162222 116.05025:10474 117.05553:243885 118.0629:69762 130.06477:220108 131.06766:12297 132.07892:561926 133.08215:11112 140.0473:10653 142.06567:92787 158.08345:12692 159.09138:178121 160.07417:53064 | -3.5828E-06 |
| POS3313 | Carnitine | 0.991 | 184.09396 | [M-H+Na]+ | 184.09399 | 52.47893:5868 68.05794:5483 102.09042:10203 125.02055:275585 184.0961:18894 | -1.6296E-07 |
| POS5882 | O-Glutarylcarnitine | 3.222 | 276.14291 | [M+H]+ | 276.14417 | 57.03287:8411 60.08025:50595 85.02799:168427 87.04334:33049 103.03849:11218 115.03932:24286 144.10197:6565 235.2888:5383 | -4.5628E-06 |
| POS5916 | coniferonic acid | 5.986 | 277.21527 | [M+H]+ | 277.216 | 55.05366:44631 57.06896:16250 58.06512:5628 65.28091:6331 67.05389:51281 68.14243:5627 69.06992:29253 79.05313:99766 81.06834:45265 83.0851:13430 91.0539:27433 93.06841:123652 95.04894:8984 95.08477:39035 97.10159:6766 105.06946:20093 107.08486:75893 109.10007:12695 119.08334:10177 121.1013:52478 131.08377:9902 133.09863:9803 135.11646:36700 147.11682:6653 149.13002:18174 | -2.6333E-06 |
| POS15000 | PC(16:0/18:1(9Z)) | 5.983 | 782.56464 | [M+Na]+ | 782.56702 | 60.08023:54684 64.37959:20354 86.09538:167105 104.10693:27097 124.99951:56726 170.22337:18464 184.0746:721530 185.07629:25982 560.38855:21848 | -3.0413E-06 |
| POS858 | O-Toluidine | 4.135 | 108.08038 | [M+H]+ | 108.08078 | 65.03805:7553 91.05391:21506 93.05685:8486 108.07939:9923 | -3.7009E-06 |
| POS1791 | gamma-Terpinene | 1.204 | 137.13217 | [M+H]+ | 137.13251 | 53.03773:18258 55.05365:44628 57.06896:22098 65.03805:13369 67.05389:96747 69.06992:23501 79.05312:56781 81.06834:278486 91.0539:45570 93.06841:14718 95.08477:138938 109.10007:6333 110.03356:10919 119.03311:11075 136.05907:16809 137.04259:13538 137.1322:6631 | -2.4794E-06 |
| POS587 | Phosphate | 1.183 | 98.98412 | [M+H]+ | 98.98418 | 53.03773:28313 55.0177:12374 55.05365:16439 56.04862:9275 57.06989:19752 58.06416:5947 60.44798:6097 62.96235:14024 70.0643:22148 71.04945:11951 72.03056:5830 79.05312:9777 80.97279:19262 81.0699:16712 81.54541:6228 98.06007:6323 98.09553:6363 98.98415:1035833 | -6.0616E-07 |
| POS10193 | 27,30-Decaoxadot | 4.529 | 503.30334 | [M+H]+ | 503.30618 | 73.06432:59612 87.04335:267868 89.05896:3006350 90.06191:63603 91.07443:15489 107.06821:24209 117.0909:21221 131.06766:59487 133.08545:1329197 134.09018:36332 173.42545:18521 175.09528:14502 177.11287:152154 | -5.6427E-06 |

**Differences in metabolites between the WJW group, the Model group and the Control group**

| Alignment ID | Metabolite name | Rt(min) | Expreiment Mz | Adduct type | Reference m/z | MS/MS spectrum | PPM |
|---|---|---|---|---|---|---|---|
| POS2076 | ole-3-carboxaldeh | 4.428 | 146.06004 | [M+H]+ | 146.0601 | 53.03774:51781 55.0177:49959 55.05366:156692 57.03287:28205 57.06896:17716 58.06512:8631 59.04859:7799 65.03805:10573 71.04816:83011 72.08048:15333 75.02498:8273 76.03859:10560 81.0323:22008 82.06503:9116 83.0851:53324 85.028:10182 86.05939:18263 86.09541:14054 91.0539:647726 99.0434:103394 100.04588:11014 100.07383:16352 100.11039:168880 112.95607:7029 117.05553:126525 118.0629:1226964 119.06659:13999 127.0381:7106 145.08403:18572 146.05721:81042 146.9101:6684 | -4.1079E-07 |
| POS6038 | Linoleic acid | 6.277 | 281.24734 | [M+H]+ | 281.24731 | 55.05364:125812 57.06895:46625 59.04857:6943 67.05388:53085 69.06866:127913 69.45988:5680 79.05311:13502 81.06831:62368 83.08508:73554 91.05389:13556 93.06839:20966 95.08475:61126 97.06459:10580 97.09951:57009 97.41055:5954 105.06944:7469 107.08484:21791 109.10004:30321 111.07958:5866 111.11478:10256 119.0861:13805 119.28451:5333 121.10127:17172 123.11521:11864 133.1019:14386 135.11642:9635 147.11679:6899 149.12999:6540 161.13194:6625 | 1.06668E-07 |
| POS6455 | Epoxyoctadecenoi | 5.771 | 297.24133 | [M+H]+ | 297.24237 | 51.75735:6200 53.80923:6083 55.01683:16956 55.05365:93349 57.03287:13354 57.06896:32501 67.05389:156887 69.06868:92838 71.04816:6667 71.08547:26065 79.05312:36059 81.06834:142837 83.04932:10612 83.08509:42345 85.06337:7733 85.10045:22460 91.0539:14153 93.06841:51028 95.08477:116463 95.10668:6615 97.06461:12573 97.09953:47258 99.07939:8130 105.06945:7673 107.08486:44411 109.10007:58942 111.0796:7031 113.09542:17052 117.06913:6481 119.08333:9755 121.1013:31741 123.11523:23177 125.09565:27342 131.08699:11338 133.09863:15672 135.11646:21771 137.1322:11367 139.11234:7678 147.11681:9530 149.13002:7365 173.13152:6600 | -3.4988E-06 |
| POS5099 | Valerylcarnitine | 4.435 | 246.16867 | [M+H]+ | 246.16998 | 55.05365:9061 57.03286:58221 57.06895:137203 58.06511:8810 60.08024:316716 70.02779:20196 72.08047:64540 74.02328:10064 84.04315:17897 84.07957:18389 85.02798:2517008 85.06335:64648 86.03024:51000 86.09539:93092 99.04126:14679 103.07442:9965 114.09124:13155 118.08492:10979 132.10172:12932 144.10196:18704 187.0934:32362 246.17052:12519 | -5.3215E-06 |
| POS788 | o[4.2.0]octa-1,3,5- | 5.024 | 105.06965 | [M+H]+ | 105.0702 | 51.02236:25263 57.03286:8617 58.06416:125667 62.05909:6692 70.70855:5422 77.03802:38850 79.05312:91655 95.04893:82298 103.05421:55881 104.10696:6409 105.04401:25295 105.06945:121863 | -5.2346E-06 |
| POS1243 | ydroxyphenethylar | 4.382 | 120.08065 | [M+H-H2O]+ | 120.081 | 51.02236:20234 53.03856:11115 56.04862:14856 56.05763:10581 56.94139:9320 61.03869:23174 65.03804:22284 72.93572:15278 73.08309:15660 77.03802:204914 79.05312:20551 80.04813:19621 91.05389:311757 93.0684:258589 95.04893:482542 102.04612:38530 103.05421:1672750 105.04401:117492 118.06289:42145 119.07216:26808 120.08029:1302164 121.08411:10697 | -2.9147E-06 |

**Differences in metabolites between the WJW group, the Model group and the Control group**

| Alignment ID | Metabolite name | Rt(min) | Expreiment Mz | Adduct type | Reference m/z | MS/MS spectrum | PPM |
|---|---|---|---|---|---|---|---|
| POS10532 | PC(18:3(6Z,9Z,12Z) | 5.974 | 518.32239 | [M+H-H2O]+ | 518.32465 | 57.03287:49122 58.06512:52567 59.04859:118975 60.08025:521029 67.05389:32627 71.07261:70802 73.02812:24968 81.06834:41715 86.09541:1216161 87.04335:37495 89.05896:107388 93.06841:43871 101.05931:25951 103.07444:21465 104.10697:6361878 105.10879:177286 121.1013:29052 124.99956:332870 133.08545:28478 173.39111:28858 184.07466:4098194 185.07635:90381 258.10977:33231 | -4.3602E-06 |
| POS3480 | hydro-2,3-dihydro | 5.059 | 189.09024 | [M+H]+ | 189.091 | 55.0177:49571 55.05365:11699 65.03805:8920 67.05389:70001 79.05312:27394 91.0539:61400 95.04893:28404 105.03246:65584 105.06945:42646 107.04917:170598 115.05257:10935 117.06913:39553 119.08612:8633 121.02692:10609 128.06212:15991 130.06477:36301 133.06566:10016 133.09863:16817 143.08574:13797 145.06526:12545 147.07849:7829 161.09245:9120 173.43034:6694 | -4.0192E-06 |
| POS2553 | 2-Aminooctanoic | 4.49 | 160.13257 | [M+H]+ | 160.13318 | 55.05364:520393 57.06987:13520 59.22437:6498 69.06866:57889 72.08046:81235 97.09951:60103 114.12788:635836 115.12945:6748 118.06563:6126 118.11523:5590 | -3.8093E-06 |
| POS6500 | s-9,10-Epoxysteari | 6.019 | 299.25647 | [M+H]+ | 299.25781 | 50.23555:5582 55.05364:51847 57.06895:121094 61.54363:6034 67.05388:57802 69.06866:61990 71.08546:90570 79.05311:8757 81.06831:69652 83.08508:35820 85.10043:19202 90.70594:5740 91.05388:7903 93.06838:15439 95.08475:58498 97.09951:26773 105.06943:12217 107.08484:16452 109.10004:32294 111.11478:14801 121.10126:8975 123.1152:15658 133.0986:6310 141.12842:7674 155.14133:7758 | -4.4777E-06 |
| POS331 | Sarcosine | 1.035 | 90.0547 | [M+H]+ | 90.05498 | 72.08048:13617 90.05457:7044 | -3.1092E-06 |
| POS9307 | 4,27-Nonaoxanon | 4.512 | 459.2782 | [M+H]+ | 459.27997 | 73.06432:38233 87.04335:173401 89.05896:2331928 90.06191:48338 107.07059:22315 111.69564:10559 117.0909:14385 130.08708:15935 131.06766:31613 133.08545:979143 134.08685:25212 173.39603:18287 175.10025:10959 177.11287:82760 404.97165:11585 | -3.8539E-06 |
| POS6892 | 9,10-DHOME | 5.769 | 315.2522 | [M+H]+ | 315.25269 | 55.05364:85993 57.03286:21650 57.06895:88435 67.05388:336217 68.05793:7065 69.06866:92537 71.04815:23103 71.08546:199392 72.08835:6770 79.05311:50379 81.06989:341412 82.07299:12434 83.04768:10102 83.08508:44796 85.06335:25664 85.10043:139213 86.10397:8183 91.05389:40656 93.06839:65346 95.08475:254246 96.08826:8358 97.06459:16473 97.09951:21453 99.07937:107753 105.06944:28517 107.08484:45978 109.06334:7637 109.10004:89606 111.07958:12368 111.11478:6867 113.09541:97063 117.06911:8496 119.08331:25121 121.10127:43117 123.08001:5879 123.11521:34098 131.08374:14218 133.0986:40235 135.11642:22921 137.13219:20744 139.1123:8036 145.10277:9481 147.11679:46276 149.12999:24990 151.11298:8617 151.14888:6721 153.12355:9442 159.1172:9714 161.13194:8500 163.14491:11530 165.12289:6695 167.14172:11627 173.13148:10005 | -1.5543E-06 |

**Differences in metabolites between the WJW group, the Model group and the Control group**

| Alignment ID | Metabolite name | Rt(min) | Expreiment Mz | Adduct type | Reference m/z | MS/MS spectrum | PPM |
|---|---|---|---|---|---|---|---|
| POS5423 | N-Lauroylglycine | 6.021 | 258.20578 | [M+H]+ | 258.2063 | 55.05365:50283 57.06895:494066 58.07271:13376 67.05389:71873 69.06867:15380 71.08546:161806 76.03858:780549 77.04237:11860 81.06833:49184 83.08508:35643 85.10044:94916 95.08476:128147 97.10158:9774 109.10005:74838 123.11523:11927 173.44014:7303 183.17154:17097 | -2.0139E-06 |
| POS6667 | 11Z,13E-eicosadie | 6.381 | 305.24673 | [M+H-H2O]+ | 305.24701 | 55.05365:79394 57.06988:25526 59.29884:5746 67.05389:119103 67.99528:5835 69.06868:64116 71.08547:8927 79.05312:122237 81.06833:99865 83.04769:7095 83.08509:29936 85.06505:8187 91.05389:50571 93.0684:210821 94.07142:7537 95.04694:7806 95.08476:84318 97.0646:6260 97.09953:11029 105.06944:42027 107.08485:116476 109.10005:33923 117.06912:8314 119.08332:28237 121.10129:84117 122.10348:6832 123.11816:8338 129.07021:9128 131.08376:15071 133.09862:23529 135.07935:6578 135.11644:35966 147.11681:5920 149.13393:7805 161.13196:9924 163.14493:15364 175.14502:6524 | -9.1729E-07 |
| POS1541 | Quinoline | 5.061 | 130.06508 | [M+H]+ | 130.06509 | 55.05366:19586 56.04863:54437 57.03287:6719 61.02846:6109 67.05389:17018 69.06992:8063 74.02328:7320 77.03802:33228 77.99809:8488 84.04317:57524 84.07957:71176 87.00325:31408 95.04894:44176 96.00742:5359 102.04613:7030 103.05422:111150 105.04402:9914 109.92458:5445 113.96312:10506 128.04967:13874 130.06477:395782 | -7.6885E-08 |
| POS3491 | 5,N6-Trimethyl-L- | 0.947 | 189.15912 | [M+H]+ | 189.15976 | 54.92054:6198 56.04952:8556 60.08024:117891 67.05389:17722 70.06555:7662 72.08047:36293 75.23055:6300 84.07956:389593 100.07381:21054 101.02003:12643 119.0303:17366 130.08388:48190 147.02486:6465 189.1548:5712 | -3.3834E-06 |
| POS2545 | ndoleacetaldehyde | 4.419 | 160.07535 | [M+H]+ | 160.07568 | 53.03275:7156 55.05364:23287 62.32769:5960 69.0699:10742 72.08046:9774 91.05388:14019 91.74825:5456 104.08642:6121 105.06712:6511 105.79249:5560 113.11348:5554 114.12788:11061 115.05254:31254 115.66189:6374 117.0555:42038 118.06287:86211 130.06474:31119 131.07085:7940 132.07889:76863 133.08542:11895 142.06563:6600 159.09134:9600 160.07414:10775 | -2.0615E-06 |
| POS11182 | 0:5(5Z,8Z,11Z,14Z, | 5.919 | 542.32269 | [M+H-H2O]+ | 542.32465 | 57.03287:45498 59.04858:141114 60.08025:271303 70.5925:37166 86.09541:703530 89.05896:111950 102.30787:38952 104.10697:3619913 105.10879:72451 111.75905:37431 124.99956:188500 181.02895:41554 184.07466:2838886 185.07635:65489 207.51649:42599 528.25073:34308 | -3.6141E-06 |

**Differences in metabolites between the WJW group, the Model group and the Control group**

| Alignment ID | Metabolite name | Rt(min) | Expreiment Mz | Adduct type | Reference m/z | MS/MS spectrum | PPM |
|---|---|---|---|---|---|---|---|
| POS7522 | Corticosterone | 5.231 | 347.2215 | [M+H]+ | 347.22168 | 55.05365:19614 67.05389:18172 69.03294:6617 69.06991:7063 79.05312:19613 81.06832:38824 83.04769:42664 87.04333:20494 91.05389:11120 93.0684:42854 95.04893:6980 95.08476:44260 97.0646:117671 99.04338:7680 101.0593:33397 105.06944:37506 107.04678:8476 107.08485:52010 109.06335:49831 109.10005:16612 111.07959:6791 117.06911:9748 119.08332:34740 121.06409:212080 121.09842:20296 122.06581:7709 123.08002:84590 129.07021:6978 131.08376:29713 133.09862:41665 135.07935:43567 135.11644:19583 137.09427:7193 143.08205:20364 145.09903:33114 147.07848:15714 147.11679:20820 149.0948:17200 149.13:8874 155.08327:8941 157.10196:23957 159.08273:7744 159.11722:27993 161.09682:11662 161.12756:7639 163.10913:21910 169.09859:11074 171.11404:28361 173.09726:9739 173.1315:14444 175.11017:12816 177.12296:7805 185.13042:9558 187.10989:7706 189.12688:7723 195.1138:6884 239.6185:6306 | -5.184E-07 |
| POS1655 | Ornithine | 0.825 | 133.09673 | [M+H]+ | 133.09718 | 68.98124:113904 69.03294:17362 69.06992:16352 70.0643:2376008 71.06874:13911 86.09541:57886 86.99279:132144 87.99284:8170 105.0024:128956 115.08438:14438 116.06905:94262 123.01261:10207 | -3.381E-06 |
| POS14781 | C(16:0/18:2(9Z,12Z | 8.803 | 758.56696 | [M+H]+ | 758.5694 | 52.5312:81083 60.08022:297747 70.51228:83223 86.09536:1030962 104.10464:110452 124.99949:267361 184.07457:4859117 185.07626:102746 240.23297:89261 | -3.2166E-06 |
| POS6503 | Ricinoleic acid | 6.287 | 299.2569 | [M+H]+ | 299.2579 | 53.44488:6044 53.60046:6452 55.05365:103513 57.06896:32207 65.17343:5370 67.05389:38333 69.06992:121672 71.08547:9832 79.05463:11767 81.06834:55123 83.08509:62298 91.0539:6481 93.03371:5882 93.06841:19233 95.08477:52134 97.06461:10400 97.09953:50898 105.06945:10980 107.08486:13158 109.10007:25022 111.0796:7293 111.11481:16280 119.08333:8449 121.09843:18795 123.11523:12748 125.09565:6603 133.09863:12192 135.11646:12812 138.47672:5490 149.13002:8029 161.13197:6575 | -3.3416E-06 |
| POS7255 | Glutamyltryptophar | 4.476 | 334.13843 | [M+H]+ | 334.13971 | 59.45607:5788 84.04316:62247 102.05499:6059 118.06566:8077 130.04884:18961 132.07892:9335 144.07968:23359 146.05721:90437 147.05934:5933 159.09137:26060 183.63564:5349 188.07004:140819 189.07664:6173 | -3.8307E-06 |
| POS4624 | Isoleucylproline | 4.391 | 229.15327 | [M+H]+ | 229.15469 | 69.06992:30904 70.06431:274902 84.08123:12069 85.02631:5938 86.09541:299671 87.09918:11021 114.05201:9145 116.06905:293098 184.09612:8011 | -6.1967E-06 |
| POS8775 | Stearoylcarnitine | 5.752 | 428.37057 | [M+H]+ | 428.37338 | 57.03287:34494 57.06896:50800 58.06512:7169 59.04858:13497 60.08025:432255 67.05271:7761 71.08547:34924 81.0699:14444 83.08509:17433 85.028:2250157 85.10045:24065 86.03024:35076 89.05896:11687 95.08477:26836 97.09953:21863 109.10007:14300 123.11523:10734 144.10197:39277 417.58035:6609 | -6.5597E-06 |

**Differences in metabolites between the WJW group, the Model group and the Control group**

| Alignment ID | Metabolite name | Rt(min) | Expreiment Mz | Adduct type | Reference m/z | MS/MS spectrum | PPM |
|---|---|---|---|---|---|---|---|
| POS827 | Benzaldehyde | 4.382 | 107.04906 | [M+H]+ | 107.0495 | 51.02236:9759 56.98664:7080 64.62771:7117 77.03802:23213 79.05312:185056 91.05389:9238 95.04893:61454 105.04401:16870 | -4.1102E-06 |
| POS6456 | (10E,12Z)-9-HODE | 6.403 | 297.24136 | [M+H]+ | 297.24219 | 55.01769:6296 55.05364:38162 57.03286:6669 57.06895:22936 67.05388:68804 69.06866:25840 71.08546:240090 79.05311:21698 81.06989:73087 83.04768:6649 83.08508:18579 91.05389:20236 93.06839:25258 95.08475:55215 96.08826:7224 97.06459:7201 97.09951:6251 97.78535:5515 99.07937:96992 105.06944:17698 107.08484:16617 109.10004:20267 111.07958:6676 111.77932:5732 117.06911:6391 119.08331:12852 121.09841:20724 123.11521:6782 127.11191:10806 131.08374:10706 135.11642:11025 147.11679:46501 151.26068:5220 153.12762:6190 163.11359:6140 165.12289:10164 | -2.7923E-06 |
| POS6620 | Agallochin G | 6.296 | 303.22992 | [M+H]+ | 303.23169 | 55.0177:6720 55.05366:68190 57.06896:30413 67.05389:114621 69.06869:67332 71.04816:13639 71.08419:8715 79.05313:53904 81.06834:86305 83.0851:8859 85.06337:7039 91.0539:110946 93.06841:60655 95.04894:7086 95.08478:61261 101.05931:8071 105.06946:126382 107.08487:37954 109.10007:12901 117.06913:51039 119.08334:83118 121.0641:8589 121.1013:23180 122.82823:6861 123.11524:9333 129.07022:37621 131.08377:93146 133.09863:39958 141.06726:16166 143.08574:15768 145.09904:47485 147.11682:11172 149.09483:6003 155.08328:7919 157.09775:23872 159.11723:19473 161.13199:14486 169.09862:10174 171.11407:8517 173.13153:16413 173.38622:6334 175.14503:12140 183.11301:11010 197.13113:7165 201.12592:11322 | -5.8371E-06 |
| POS2554 | ninovaleric acid be | 1.024 | 160.1326 | [M+H]+ | 160.13318 | 53.03857:8319 55.05365:1155093 55.0633:28922 56.05674:13139 57.03287:10381 58.06416:57207 59.04858:237063 59.07295:29511 60.08025:840096 70.0643:50259 73.06432:8252 83.0477:172410 84.07957:32714 100.07382:12056 101.0593:460148 102.06162:8367 115.05257:20736 116.04757:7478 117.05553:12796 132.07892:12704 160.07417:29256 160.13072:166982 | -3.622E-06 |
| POS3505 | lihydroquinoline-4 | 4.538 | 190.04877 | [M+H]+ | 190.04961 | 55.05365:20905 67.61636:6215 72.08047:142646 89.0373:16348 99.13448:5946 110.25202:6073 115.83308:6206 116.04757:39031 133.35954:6686 144.04256:8430 144.10197:15632 162.05399:418090 163.05547:10310 173.39601:13183 190.04633:30084 | -4.4199E-06 |
| POS1399 | Taurine | 1.128 | 126.02174 | [M+H]+ | 126.02198 | 53.03773:10197 55.05365:9503 56.04862:7360 66.53687:5755 68.0495:16281 70.0643:8011 78.98524:6630 80.04968:12502 84.07957:9237 98.05798:7485 108.00944:102751 108.9949:6815 109.07559:31331 126.02023:106282 | -1.9044E-06 |
| POS10356 | LysoPC(17:0/0:0) | 5.894 | 510.35648 | [M+H]+ | 510.35538 | 57.03287:86428 59.04859:50801 60.08025:54653 81.0699:7127 86.09541:122606 87.04335:5693 89.05896:8908 95.08477:7326 95.98923:6149 99.07939:13648 104.10697:742077 105.10879:18157 124.99956:30848 133.4489:5693 157.12312:11359 184.07466:368997 185.07635:9702 236.22951:5710 306.14078:5994 | 2.15536E-06 |

**Differences in metabolites between the WJW group, the Model group and the Control group**

| Alignment ID | Metabolite name | Rt(min) | Expreiment Mz | Adduct type | Reference m/z | MS/MS spectrum | PPM |
|---|---|---|---|---|---|---|---|
| POS6031 | Stearolic acid | 4.355 | 281.24564 | [M+H]+ | 281.24719 | 55.05364:45826 57.06895:26962 59.04857:6734 67.05388:17571 69.0699:49359 71.08546:9974 81.06989:18077 83.08508:33551 91.05389:5999 93.06839:8555 95.08475:26932 97.09951:17440 98.61504:5824 107.08485:10366 109.10004:9719 111.1173:7898 119.08331:8945 121.10127:8328 123.11521:7154 161.13194:5821 | -5.5112E-06 |
| POS1789 | -alpha-Phellandre | 2.777 | 137.13206 | [M+H]+ | 137.13251 | 50.26157:6555 53.03856:10255 55.05365:24128 56.94232:13348 57.06988:10135 67.05389:39521 69.06991:14085 72.93706:12012 79.05312:32040 81.06833:129502 89.90059:5602 90.94762:9906 91.05389:17447 93.0684:7249 95.08476:58768 103.24319:6600 136.06245:6346 | -3.2815E-06 |
| POS206 | Methacrylamide | 4.801 | 86.05978 | [M+H]+ | 86.06 | 58.06416:79400 69.03293:13792 84.03653:5447 86.05937:25114 | -2.5564E-06 |
| POS2847 | Indoleacrylic acid | 4.418 | 170.05942 | [M+H-H2O]+ | 170.06004 | 50.93715:6072 55.0177:6911 55.05365:19589 55.93345:27101 67.05389:12955 68.04829:6388 69.06868:8131 72.04369:11242 79.05463:9584 81.06989:11015 106.06411:6356 107.08485:7663 114.97045:25386 115.05257:138336 128.95062:7462 142.06203:38507 146.95981:6089 170.05659:71694 | -3.6458E-06 |
| POS1800 | Trigonelline | 4.613 | 138.05453 | [M+H]+ | 138.0551 | 53.03773:15270 55.05365:10182 65.03805:11336 67.05389:27633 68.04829:14746 78.0336:69964 79.04105:37440 79.05162:11264 80.04968:8100 81.06834:51981 94.06554:6644 95.08477:21912 96.04379:228644 108.0432:39978 110.05836:12946 124.03867:25923 138.05421:307052 | -4.1288E-06 |
| POS2015 | METHYLQUINOLI | 4.419 | 144.08061 | [M+H]+ | 144.0808 | 52.60244:6370 53.03774:28367 55.0177:33930 55.05366:27428 56.04863:16803 57.03287:18875 57.06989:8537 63.06548:5772 69.03294:31338 70.06431:164053 71.04816:39263 77.03802:7577 79.05313:6114 81.0323:11294 81.0699:16849 84.04482:7076 89.03731:7724 91.0539:29775 91.36633:5304 95.04894:11468 98.06007:87031 98.09554:47486 99.0434:52100 103.05422:41978 115.05257:81645 116.04757:10636 117.06913:139579 118.06566:6036 126.09015:33512 128.04655:8832 142.06567:6482 143.0343:41579 143.07104:94184 144.0797:393521 145.06528:22824 | -1.3187E-06 |
| POS1619 | 6-Methyl indole | 4.422 | 132.08081 | [M+H]+ | 132.0809 | 58.06512:12753 58.07272:6489 69.03294:22883 69.06992:48831 77.03802:12116 79.05313:14901 86.05939:13680 86.09541:283147 88.00349:10381 91.0539:15793 93.05684:11005 95.04894:24133 97.00919:8706 97.77084:5883 103.05422:70909 105.06946:50995 106.06412:5359 115.05257:103497 117.05553:226174 130.06477:29594 131.07088:29487 132.07892:233975 | -6.814E-07 |
| POS7887 | exapropylene glyc | 5.061 | 367.26733 | [M+H]+ | 367.26898 | 57.03286:154690 58.18789:5729 59.04858:1746998 60.05225:23401 73.06431:53570 85.02798:7284 87.04333:17183 89.05895:16939 97.06255:5646 99.07938:8648 103.07443:6641 115.07377:10087 117.0528:9869 117.09088:115985 131.07086:19714 167.31822:5942 173.3911:11814 175.13008:6860 226.5341:6140 333.52783:5814 | -4.4926E-06 |

**Differences in metabolites between the WJW group, the Model group and the Control group**

| Alignment ID | Metabolite name | Rt(min) | Expreiment Mz | Adduct type | Reference m/z | MS/MS spectrum | PPM |
|---|---|---|---|---|---|---|---|
| POS1231 | Hydroxybenzonitr | 4.774 | 120.04428 | [M+H]+ | 120.04438 | 56.04952:7750 56.05764:13294 61.03972:18567 65.03804:121006 70.39166:5450 72.08047:8558 73.08444:14919 91.0539:6993 92.0491:83508 103.05421:17259 111.67029:5583 120.04357:20325 120.0803:7590 | -8.3303E-07 |
| NEG253 | mino-2-methylbu | 0.908 | 97.07714 | [M-H]- | 97.0772 | 79.05368:126619 80.05277:612254 94.18431:5963 97.07645:2942376 | -6.1806E-07 |
| POS6248 | Testosterone | 5.518 | 289.21558 | [M+H]+ | 289.21619 | 55.05363:9679 67.05386:9397 69.06989:12175 79.05309:7681 81.06986:10555 83.04929:11954 84.03651:6250 86.09537:22617 93.06837:6272 97.06456:156759 98.06837:6410 104.10692:12893 105.0694:9938 109.06331:108771 110.33407:6009 123.07998:6579 130.99672:6339 261.53647:5972 | -2.1091E-06 |
| POS8198 | (+)-Diaeudesmin | 5.535 | 387.17865 | [M+H]+ | 387.18011 | 59.04858:7208 79.05312:18069 81.03229:7618 95.04893:7858 103.05421:12348 105.06945:1023412 106.07115:41636 119.04706:18835 121.0641:14413 124.34185:5209 141.08885:5042 163.98055:5395 197.54788:5811 281.13101:5592 | -3.7709E-06 |
| POS2409 | lethanolaminium p | 0.991 | 156.04137 | [M+H]+ | 156.0424 | 74.09583:71991 82.05225:11145 83.05907:63042 93.04334:39455 95.05889:27291 96.9661:7112 105.7131:5254 110.07076:223594 112.05141:10357 114.78535:5385 130.06158:42132 146.88715:6278 156.04167:102805 | -6.6008E-06 |
| POS9645 | oPE(18:2(9Z,12Z)/( | 6.153 | 478.29233 | [M+H]+ | 478.29269 | 51.34163:6667 55.05364:85019 55.21454:5493 57.03286:78181 57.06987:29910 62.06013:11470 67.05388:140674 69.06866:123864 71.08546:10086 79.05311:25298 81.06989:154548 83.08508:80852 85.06335:6763 87.29327:6183 91.05389:13545 93.06839:34365 95.08475:122181 97.06459:13284 97.09951:38596 105.06944:17190 107.08484:27814 109.10004:60872 111.1173:11633 119.0861:16510 121.10127:24420 123.11521:25988 133.0986:16699 135.11642:18706 137.13217:9333 147.11679:13918 149.1339:8839 161.13194:6938 162.26683:6997 163.14938:8087 | -7.5268E-07 |
| POS3818 | Dodecanamide | 6.145 | 200.19971 | [M+H]+ | 200.20081 | 55.05365:18749 57.06895:50255 69.06867:10385 71.08546:7644 74.06022:10785 75.65979:5727 81.06989:6977 88.07444:49109 102.09041:17858 116.10664:6624 124.89156:5987 148.61507:5308 200.19798:42427 | -5.4945E-06 |
| POS4316 | Propionylcarnitine | 1.092 | 218.13759 | [M+H]+ | 218.13869 | 57.03286:11116 60.08024:37522 61.03971:9927 84.07957:9433 85.02798:259449 | -5.0427E-06 |
| POS4575 | butyl 9-decenoate | 5.693 | 227.20029 | [M+H]+ | 227.20039 | 55.05365:75761 57.06896:54375 67.05389:21621 69.06992:84161 71.08547:15864 76.01581:5873 79.05312:13981 81.0699:28858 83.08509:31734 93.06841:15564 95.08477:25756 97.06461:14665 97.10159:16206 100.11038:9758 107.08486:19222 109.10006:20843 111.0796:8132 121.1013:13856 123.18568:6006 135.11646:11720 135.83438:5483 138.09952:5890 176.64301:6112 177.47789:7268 | -4.4014E-07 |

**Differences in metabolites between the WJW group, the Model group and the Control group**

| Alignment ID | Metabolite name | Rt(min) | Expreiment Mz | Adduct type | Reference m/z | MS/MS spectrum | PPM |
|---|---|---|---|---|---|---|---|
| POS6404 | (Z),9(Z),12(Z)-octa | 6.243 | 295.22681 | [M+H]+ | 295.22672 | 55.01769:6971 55.05364:28988 57.03286:55182 57.06987:16117 62.27805:5648 67.05388:51800 69.06866:22390 71.08418:17836 79.05311:28301 81.06831:65954 83.04931:7079 83.08508:9164 91.05389:13807 93.06839:42394 95.08475:49425 99.07937:7131 105.06944:8780 107.08484:31933 109.06334:6201 109.10004:20091 119.08331:6609 121.10127:25600 133.0986:8134 135.11642:19060 186.14619:5502 226.35844:5011 | 3.0485E-07 |
| POS6403 | ,4'Z,8E)-Colneleic | 5.647 | 295.22653 | [M+H]+ | 295.2265 | 52.0545:5624 55.01769:9634 55.05364:34234 57.03286:12621 57.06895:8575 59.04857:8780 67.05388:39854 69.0699:28580 71.04943:7208 71.08546:7824 79.05311:24591 81.06831:37881 83.08508:8951 91.05389:11435 93.06839:30809 93.93243:6190 95.08475:32607 97.06459:9142 97.09951:9384 105.06944:8362 107.08484:28725 109.06334:6243 109.10004:14948 119.08331:11954 121.09841:17574 133.1019:8700 135.11642:10539 145.09901:5857 146.10268:6223 173.39598:9476 | 1.01617E-07 |
| POS3359 | midopropyl)pyrrol | 4.424 | 185.12825 | [M+H]+ | 185.129 | 53.14906:6051 55.05365:12264 59.64061:5031 70.06429:11845 86.4879:5365 98.06005:86194 100.07381:18263 116.97124:6533 125.10766:20113 126.09013:189282 143.1188:8464 | -4.0512E-06 |
| POS5556 | -trans-Bergamoto | 5.875 | 263.20016 | [M+H]+ | 263.2002 | 53.03772:7535 55.01768:18061 55.05364:260452 57.03285:7077 57.06894:58783 59.04856:11671 61.48146:5472 67.05387:164706 69.06866:100524 71.04814:35224 71.08417:15772 77.03799:16605 79.0531:220578 81.06988:155633 82.065:17420 83.04767:15082 83.08507:24513 85.02797:7625 85.06503:19485 87.04332:8695 91.05387:214431 92.05666:5810 93.06838:282421 94.07336:8874 95.0489:18751 95.08474:72032 97.06458:6866 97.10155:11060 105.06942:235724 106.07346:13731 107.08483:105249 109.06332:14729 109.10003:25466 117.06909:49148 119.08329:197641 120.08875:12157 121.06406:5999 121.10126:52704 123.1152:9954 129.06702:29755 131.08372:58224 133.09859:121701 135.11641:21689 142.07654:5827 143.08569:20258 145.099:14880 147.07845:21436 147.11676:60528 149.09477:6374 149.12997:16558 157.10193:16064 161.09679:24467 161.13193:28054 163.1449:8970 171.11401:6749 175.11014:15110 179.10367:7485 203.17516:6551 | -1.5198E-07 |
| POS6563 | (+)-Cedronellone | 6.077 | 301.21539 | [M+H]+ | 301.21619 | 55.05366:25368 67.05389:56577 69.06868:18458 71.04816:7128 79.05313:39738 81.0699:50896 85.06506:5491 91.0539:61031 93.06841:58806 95.08477:29793 100.07598:8951 105.06946:56484 107.08486:28652 109.10007:13815 117.06913:59324 119.08334:41936 121.1013:28848 129.06706:5995 131.08377:59156 133.09863:46173 135.11646:18568 143.08574:10898 145.09904:38449 147.11682:18107 157.10197:7542 159.11723:35770 161.13197:10943 171.11888:11544 173.13153:21280 | -2.6559E-06 |
| POS1790 | methyl-1,3-buta- | 6.298 | 137.13211 | [M+H]+ | 137.1324 | 67.05386:16876 79.0546:6534 81.06986:23444 95.08472:7924 | -2.1147E-06 |

**Differences in metabolites between the WJW group, the Model group and the Control group**

| Alignment ID | Metabolite name | Rt(min) | Expreiment Mz | Adduct type | Reference m/z | MS/MS spectrum | PPM |
|---|---|---|---|---|---|---|---|
| POS441 | 3-Methylpyridine | 8.783 | 94.0649 | [M+H]+ | 94.065 | 51.02237:7674 53.03774:81905 59.52111:55294 60.02129:6491 65.03917:8304 67.05389:24286 68.52579:13854 71.02888:55394 71.52904:10201 78.0336:16339 80.0343:12790 82.53645:13303 93.05684:11112 94.06359:527473 96.04379:51254 | -1.0631E-06 |
| POS4539 | nzyldimethyl ammo | 6.524 | 226.15863 | [M]+ | 226.159 | 134.09351:18333 226.16113:12733 | -1.636E-06 |
| POS5559 | Farnesyl acetone | 6.02 | 263.23538 | [M+H]+ | 263.2366 | 55.05365:13059 57.06896:12734 65.03692:8294 67.05389:23489 69.06991:10162 71.08547:9394 79.05312:9136 81.0699:28906 91.0539:7192 93.06841:14666 95.08477:22257 105.06945:8580 107.08486:11834 109.10006:9848 116.69191:5808 119.08333:9577 121.1013:6421 133.09863:8001 | -4.6346E-06 |
| POS10531 | nitoylphosphatidylc | 8.79 | 518.31934 | [M+Na]+ | 518.32172 | 56.04953:8669 57.03287:23127 57.06896:76608 58.06512:6933 60.08025:35811 67.05389:11238 69.06992:16160 71.0726:25952 71.08547:60934 81.0699:18773 83.08509:27941 85.10045:39284 86.09541:370488 87.09918:9280 95.08477:28970 97.09953:9473 103.07444:6091 104.10696:1537859 105.10879:43020 109.10006:22270 123.11523:10211 126.02023:9904 146.97894:429330 164.99086:6845 260.09827:7101 313.26874:12736 337.16791:6369 | -4.5917E-06 |
| POS11971 | 2:5(4Z,7Z,10Z,13Z, | 6.306 | 570.35352 | [2M+H]+ | 570.35596 | 58.06512:26083 60.08025:237833 71.07261:29532 86.09541:566328 91.0539:27831 104.10697:2834781 105.10879:77117 124.99956:129786 184.07466:1870368 185.07635:54585 220.16205:20271 | -4.278E-06 |
| POS12629 | ecapropylene glyc | 5.72 | 599.43445 | [M+H]+ | 599.43646 | 57.03287:98999 59.04858:1006998 60.05225:10498 73.02811:17432 87.04334:26531 89.05895:57361 103.07444:19106 115.07378:13303 117.09089:142591 133.08543:25731 175.1301:24920 | -3.3531E-06 |
| POS7318 | Subamolide C | 6.06 | 337.27145 | [M+H]+ | 337.2731 | 55.05366:32691 57.03287:20626 57.06989:10268 59.04956:6694 67.05389:48472 69.06868:26923 79.05313:13135 81.06834:40722 83.0851:14560 91.05204:7645 93.07034:9117 95.08477:30326 97.09953:7279 107.08486:10438 109.10007:15247 121.1013:6150 | -4.8922E-06 |
| POS14450 | | 4.642 | 721.41571 | [M+2H]2+ | 721.41571 | 69.03294:64032 73.02811:159873 73.06432:77520 83.45062:47888 87.04334:536056 89.05896:2457454 90.0619:93104 95.04694:86163 98.23338:47352 111.04189:78648 111.44507:43274 113.05927:60756 121.66422:45778 131.07088:82096 133.08543:851508 137.0598:58032 177.11285:88833 200.87497:48388 327.85837:47698 656.01245:49795 | 0 |
| POS10376 | His Gln Val Lys | 4.662 | 511.29846 | [M+H]+ | 511.29861 | 69.03291:46719 73.02808:224037 73.06429:52427 80.05272:60468 87.0433:471413 88.04601:45781 89.05892:2417979 90.06187:116949 91.04639:54072 95.0489:109640 99.04335:56884 111.04436:90199 113.05922:59512 129.05441:36895 131.07082:54101 133.08539:702772 173.39104:87009 177.10771:62434 323.89127:41408 | -2.9337E-07 |
| POS4996 | alpha-Humulene | 4.604 | 243.15111 | [M+2H]2+ | 243.15096 | 73.02811:8617 104.56932:5620 136.52379:5669 173.43034:6491 207.27916:5990 227.04758:5414 | 6.16901E-07 |
| POS13566 | Dianthosaponin C | 4.577 | 652.40558 | [2M+H]+ | 652.40552 | 77.99068:37795 87.01893:36948 87.04334:142321 89.05896:1047998 131.06766:38560 133.08543:452202 166.69713:42909 177.11285:79337 400.16666:38883 | 9.19673E-08 |

**Differences in metabolites between the WJW group, the Model group and the Control group**

| Alignment ID | Metabolite name | Rt(min) | Expreiment Mz | Adduct type | Reference m/z | MS/MS spectrum | PPM |
|---|---|---|---|---|---|---|---|
| POS7122 | Boeravinone F | 0.879 | 327.04965 | [M+Na]+ | 327.04971 | 86.16064:5992 102.22125:5057 191.07452:111349 232.32516:5391 | -1.8346E-07 |
| POS12311 | Bilirubin | 4.692 | 584.67792 | [M+H]+ | 584.6781 | 73.02811:187672 73.06432:101464 80.05429:116505 87.04334:652252 89.05896:3177115 90.0619:109811 91.04457:60691 95.04893:140279 102.06606:66387 103.98616:58381 111.04441:87383 113.05927:81682 117.09089:58913 131.06766:103725 133.08543:1659876 134.08684:94500 137.05981:68955 155.0667:75801 175.09526:79543 177.11285:344461 210.66563:63524 | -3.0786E-07 |
| POS12919 | Puwainaphycin D | 4.706 | 613.35681 | [M+H]+ | 613.35699 | 73.02811:56583 87.04334:169634 89.05896:974096 90.0619:39072 102.56839:25109 113.05927:37699 117.20798:23434 130.75883:29066 133.08543:348689 177.11285:53095 202.06644:32968 | -2.9347E-07 |
| POS9758 | yl)oxolan-2-yl]oxyr | 4.813 | 483.18228 | [M+Na]+ | 483.1824 | 55.71933:7792 67.26189:6694 68.4661:6352 76.087:7454 81.38747:8313 95.49025:8769 132.05931:6142 142.10197:5732 162.06279:45742 189.08772:593454 190.09126:33628 207.09973:27956 230.1127:6781 253.08136:356252 254.08736:22405 | -2.4835E-07 |
| POS12917 | Austrobuxusin I | 4.569 | 613.33508 | [2M+H]+ | 613.33502 | 50.28071:22135 76.60421:18230 279.35773:16421 408.97412:18993 449.91245:18538 | 9.78258E-08 |
| POS6547 | Salidroside | 4.947 | 301.12735 | [M+Na]+ | 301.12729 | 51.83899:6899 55.01768:66130 57.06894:396680 71.01213:84376 81.06987:12902 83.01192:42239 83.04766:8314 85.06333:185974 86.06792:5770 109.06332:17662 111.04185:16356 113.02309:321047 114.0258:7122 115.03928:25477 125.02351:16579 127.07495:203994 128.07762:7579 139.0383:13773 139.07704:9267 159.09993:459001 160.10455:22396 167.035:13795 167.10457:8249 171.09958:19797 173.38614:7900 | 1.99251E-07 |
| POS13499 | Lotusine E | 4.542 | 648.37427 | [M+NH4]+ | 648.37433 | 69.03294:40275 83.04769:18316 87.04333:116802 89.05895:355918 99.04338:82046 111.04189:38023 118.28912:15999 125.05659:19656 131.07086:18570 133.08543:149936 155.07082:41003 173.43523:19753 177.11284:22214 377.14182:16945 | -9.2539E-08 |
| POS6948 | Leu-Ala-Asp | 4.4 | 318.16559 | [M+H-H2O]+ | 318.1655 | 56.04953:7642 60.04426:6621 60.60372:5468 69.04404:8689 84.07958:126264 85.06337:9106 85.0836:6364 86.09541:165517 87.09918:7101 89.96288:5351 91.0539:82896 99.62812:5429 110.07076:109186 110.98161:5239 111.07458:7410 119.04707:94549 120.05206:8496 120.08031:19854 129.10172:102908 130.1062:8360 132.10175:17803 136.07611:178726 137.07704:14016 147.04404:23887 320.41443:6412 | 2.82872E-07 |
| POS10612 | 180083-23-2 | 4.489 | 521.25366 | [M+H]+ | 521.25378 | 76.65031:7219 86.09541:8095 87.04334:12034 89.05896:183626 90.0619:14901 104.10696:28155 133.08543:76964 134.08684:9642 177.11285:11352 184.06929:12850 466.78955:6213 | -2.3021E-07 |
| POS9097 | Mutamicin 5 | 5.214 | 447.29263 | [M+H]+ | 447.29251 | 52.18702:7097 59.04856:8204 75.88783:5461 89.05891:10473 130.28481:6147 149.63971:5515 258.80569:5579 317.15057:5762 330.39728:5447 339.19794:5301 | 2.68281E-07 |
| POS14088 | an-2-yl)-17-oxa-1 | 4.554 | 692.4007 | [M+H]+ | 692.401 | 69.03294:34406 83.04932:19091 87.04334:79213 89.05896:243289 99.0434:58198 111.04441:37770 133.08545:97630 137.05981:16046 173.38622:28450 177.11285:22280 319.43127:17043 | -4.3327E-07 |

**Differences in metabolites between the WJW group, the Model group and the Control group**

| Alignment ID | Metabolite name | Rt(min) | Expreiment Mz | Adduct type | Reference m/z | MS/MS spectrum | PPM |
|---|---|---|---|---|---|---|---|
| POS8391 | Lintetralin | 5.686 | 401.19635 | [M+CH3OH+H]+ | 401.1962 | 57.06894:233706 58.07269:7607 61.01:15858 81.03226:61737 83.01192:78113 85.02796:10233 85.06333:169177 111.04436:9805 115.03928:45062 125.05955:8504 127.07495:13433 133.06891:58049 135.04222:15256 137.05975:9002 139.07352:21032 147.04396:13973 157.08499:7303 167.10457:243647 168.11116:14351 171.09958:19267 213.09639:6366 221.1192:22467 231.10547:21423 | 3.73882E-07 |
| POS11476 | Leueantine C | 8.805 | 552.32867 | [M+2H]2+ | 552.32867 | 60.08025:21437 86.0954:54921 104.10696:312401 184.07465:96514 | 0 |
| POS10703 | Pyrichalasin H | 8.806 | 524.30072 | [M+2H]2+ | 524.3006 | 60.08022:679045 80.43092:138712 86.09536:1490409 104.10692:7351334 124.99949:304914 184.07457:2358259 293.82486:116712 | 2.28876E-07 |
| POS5483 | 8-Deoxy-lactucin | 4.64 | 261.11411 | [M+H]+ | 261.11401 | 51.75893:6842 53.03854:10056 55.01768:12436 55.05363:7024 57.03377:15202 57.06894:491758 58.07269:11803 59.04856:6527 61.01:37998 67.05386:8968 69.06989:22516 71.01213:14176 79.05309:11797 81.03226:150374 81.06987:15602 83.04766:31896 85.06333:227721 95.0111:8694 97.06457:9527 107.04913:7432 111.04436:49075 125.05955:159609 127.07495:17739 133.0656:113285 137.09422:7017 167.10457:710584 168.11116:45533 179.10365:8088 185.0979:14064 195.10202:11404 197.1132:11825 213.09639:9178 231.10547:13816 | 3.82974E-07 |
| NEG6553 | etramethylheptade | 0.899 | 323.29568 | [M-H]- | 323.29559 | 77.07738:6812 79.05368:18879 90.50151:5857 103.07947:75414 104.78513:5409 113.12115:24053 119.0854:12567 124.15677:13453 187.15546:156768 | 2.78383E-07 |
| POS6609 | Cumyluron | 4.77 | 303.12589 | [M+H]+ | 303.12589 | 57.06894:245511 58.07269:8180 59.58425:5492 61.01:39462 81.03226:18130 81.06987:16570 85.06333:115257 95.0489:5669 109.06332:9289 111.04185:6901 113.05923:26071 125.05955:13986 127.07495:10276 133.0656:13185 137.05975:93128 167.10457:94935 221.1192:18987 | 0 |
| POS3472 | 4-Chlorobiphenyl | 0.753 | 189.04665 | [M+CH3OH+H]+ | 189.04649 | 59.59612:5875 68.98123:50815 78.67258:6030 83.00869:42062 86.99104:76636 100.03513:13540 101.01785:244130 102.01955:9461 105.0024:100551 118.0436:34416 119.02752:344739 120.02943:15424 123.0126:13613 129.01353:31462 131.08376:5520 137.03912:139074 138.04027:7743 144.02397:7307 147.02486:159008 161.05292:15694 165.03181:23271 179.06253:7655 | 8.46353E-07 |
| POS9531 | olic acid glycine co | 4.473 | 472.27298 | [M+H]+ | 472.27301 | 69.03294:38778 73.02811:13523 73.06432:6329 83.0477:15500 87.04334:72463 87.09045:5532 89.05896:142651 93.32733:6502 95.04694:6716 99.04339:53482 107.07059:5835 111.04189:35667 122.36179:5627 123.80167:6072 125.0596:10348 131.06766:7048 133.08543:49732 155.07083:13830 333.67181:5748 | -6.3523E-08 |
| POS13624 | beta-D-glucopyran | 4.717 | 657.38458 | [M+Na]+ | 657.3844 | 73.02678:32745 87.04334:110396 89.05896:562632 90.06374:31628 95.04893:28345 129.8802:24921 133.08543:277951 177.11285:50766 373.06967:24111 | 2.73812E-07 |
| POS9326 | BW A868C | 6.157 | 460.28043 | [M+H-H2O]+ | 460.28049 | 50.52047:6237 56.0153:6455 173.39111:6724 | -1.3036E-07 |
| POS5433 | ofumesate-2-hydr | 0.88 | 259.06332 | [M+Na]+ | 259.06339 | 75.5243:6317 80.34119:5069 119.49465:5860 150.53221:5378 153.20093:6466 191.07452:69247 | -2.702E-07 |

**Differences in metabolites between the WJW group, the Model group and the Control group**

| Alignment ID | Metabolite name | Rt(min) | Expreiment Mz | Adduct type | Reference m/z | MS/MS spectrum | PPM |
|---|---|---|---|---|---|---|---|
| POS1644 | THTC | 1.146 | 133.03159 | [2M+H]+ | 133.03169 | 53.03856:10928 55.05365:15188 56.04952:34333 57.05692:121539 58.06416:77048 58.99405:15342 59.04858:12006 61.01003:54938 67.05389:18755 69.06867:1093632 70.06934:24785 70.07311:93038 73.06432:9416 86.0954:2214347 87.0259:32303 87.09917:177128 90.05456:13203 | -7.517E-07 |
| POS7250 | Sarcostolide G | 5.29 | 333.20493 | [M+H]+ | 333.2049 | 72.03056:5233 93.06841:5945 97.06461:14230 107.08486:8269 109.06336:8815 114.88049:5409 121.06409:38398 123.08003:13336 147.11681:7411 180.61118:6398 245.93018:5880 324.12939:6920 | 9.00347E-08 |
| POS4246 | Atrazine | 4.819 | 215.08989 | [2M+H]+ | 215.09 | 50.42034:6599 55.05363:9548 57.06894:113538 58.07269:33225 61.01:8257 63.00633:9057 70.39037:6693 81.03226:25529 82.03626:7456 85.06333:15536 | -5.1141E-07 |
| POS7280 | dro-15-keto Prost | 5.614 | 335.21899 | [M+Na]+ | 335.21899 | 51.63123:6052 57.83962:6671 76.67337:5677 79.88229:5885 95.08477:6673 109.49762:5725 | 0 |
| POS7530 | -2-ylthio)-N-(4-r | 0.938 | 348.0777 | [M+CH3OH+H]+ | 348.07773 | 80.94776:10779 86.09541:34363 96.92101:111833 104.10697:163656 104.99086:7337 221.01341:33715 | -8.6188E-08 |
| POS9145 | Arnamiol | 4.456 | 451.18823 | [M+H]+ | 451.18811 | 55.0168:14414 55.05363:23165 57.03377:7112 59.04856:76132 61.01:9500 67.05386:6581 75.02494:11045 79.05309:8651 81.03226:82662 81.0683:8136 83.04766:73302 85.02796:17255 91.02029:179714 95.0489:37929 97.06457:14619 110.53846:6260 123.04187:33697 125.05955:827176 126.06273:33212 127.03804:14552 129.03552:11116 143.0526:34766 146.05714:18834 165.09097:73302 171.04672:35155 183.10228:34063 188.06996:43084 189.05423:202614 190.0575:11211 247.10213:10098 379.40112:6607 | 2.65964E-07 |
| POS6179 | Abacavir | 4.402 | 287.15973 | [M+2H]2+ | 287.15961 | 65.06059:7534 67.51974:6101 70.06431:642851 71.06746:23044 71.16019:5951 79.5765:6169 84.07957:14715 93.07034:8096 97.07488:6848 98.06007:16757 103.05197:23197 112.08709:52986 113.58521:38696 115.08704:10591 120.08031:498348 121.08413:37672 124.08617:13476 126.05367:11642 127.08732:15640 140.07935:15476 151.12099:15033 153.10732:7985 155.07913:10428 157.10622:19128 166.08408:38347 167.11856:8890 194.12837:8407 195.11382:30909 246.511:6159 254.16579:6773 | 4.17886E-07 |
| POS15004 | PC(16:0/18:1(9Z)) | 6.475 | 782.56622 | [M+H]+ | 782.56616 | 53.69582:11689 67.33069:12777 67.96278:10387 86.09541:15276 184.07466:34009 184.36464:11386 293.25256:10403 351.13647:10588 | 7.66708E-08 |
| POS3471 | 4-Chlorobiphenyl | 1.023 | 189.04648 | [M+H]+ | 189.04649 | 55.17931:6214 58.06416:19840 60.08024:33413 70.06429:25884 72.04369:29659 72.08047:435680 73.08309:21275 83.00869:10138 84.07957:203678 100.07381:216677 101.01785:73082 101.07893:15345 112.11002:5731 117.10176:10318 118.04636:10666 119.0303:116327 126.09013:8868 129.01353:10243 130.08388:20478 136.05563:5951 137.03912:40195 147.02486:57206 161.04852:7674 165.03636:13179 171.14771:6036 | -5.2897E-08 |
| POS6127 | lbut-3-en-2-yl)fur | 4.77 | 285.11392 | [M+H]+ | 285.11401 | 57.06986:9585 110.07567:6062 | -3.1566E-07 |
| POS10527 | Thr Thr Gly Leu Ile | 6.89 | 518.31836 | [M+H]+ | 518.31842 | 59.27237:6310 66.88557:6165 73.49677:5366 83.51614:5136 86.0954:10455 130.33278:5614 146.98276:20141 324.70667:6624 386.10718:5660 | -1.1576E-07 |

**Differences in metabolites between the WJW group, the Model group and the Control group**

| Alignment ID | Metabolite name | Rt(min) | Expreiment Mz | Adduct type | Reference m/z | MS/MS spectrum | PPM |
|---|---|---|---|---|---|---|---|
| POS4670 | Norpterphyllin III | 5.997 | 231.10387 | [M+H]+ | 231.104 | 53.69413:5942 57.06989:12373 74.0616:6065 108.67789:5870 167.10463:11847 179.73859:5568 | -5.6252E-07 |
| POS6976 | Tomelukast | 6.311 | 319.17535 | [M+NH4]2+ | 319.1752 | 55.0177:30555 55.05365:23184 57.03287:28828 64.85594:6493 67.05389:24674 69.06868:117672 69.80681:5353 71.04816:10347 79.05312:25104 81.06833:19993 91.0539:35686 93.06841:24821 95.04893:5465 95.08477:9970 97.06461:13405 105.03245:10543 105.06945:39949 107.04917:42940 107.08486:8338 107.37579:5076 117.06912:14013 117.28432:6209 119.08333:29494 121.1013:9240 122.76101:6073 131.08377:5982 133.09863:15446 135.11644:13885 139.11232:14733 143.08574:9419 145.09904:11977 147.07849:24923 161.09683:20508 163.10915:28111 165.09103:7358 206.39116:6086 | 4.69961E-07 |
| POS10232 | Lys Glu Asn Asp | 4.366 | 505.22525 | [2M+H]+ | 505.22519 | 60.04425:13615 72.08047:24447 89.88777:6027 115.0499:6708 118.08492:8113 129.1017:13294 154.0515:7791 175.10519:15609 189.08778:5882 | 1.18759E-07 |
| POS7539 | Mukoenine B | 6.445 | 348.19626 | [M+Na]2+ | 348.19635 | 57.03287:36244 59.67723:6330 74.65649:6367 252.33946:5698 | -2.5847E-07 |
| POS7516 | Argophyllin C | 4.821 | 347.18448 | [M+H]+ | 347.18451 | 53.47846:5443 90.58919:5692 102.95767:6152 105.06945:5447 113.36972:5229 121.06409:29767 170.89314:5683 | -8.6409E-08 |
| POS8931 | Nudicaulidine | 6.363 | 438.28555 | [M+2H]2+ | 438.2854 | 51.01141:6861 57.03287:7264 59.04858:31380 338.36777:6055 | 3.42243E-07 |
| POS14977 | -3-hydroxydecan | 5.943 | 780.54694 | [M+H]+ | 780.547 | 51.74374:10069 55.43724:8301 60.08022:27942 71.07257:10790 80.74166:8042 86.09537:79987 104.10693:11189 124.9995:22929 161.76192:7751 184.07458:204750 464.99683:8789 | -7.6869E-08 |
| NEG3711 | Sinapic acid | 1.325 | 223.08005 | [M-H]- | 223.08 | 71.09774:6503 79.05373:9090 84.60606:5587 92.02104:34617 97.07652:33898 106.00214:5626 136.06505:14081 140.30067:5684 165.03516:39688 | 2.24135E-07 |
| POS8562 | leliosupine N-oxid | 5.663 | 414.21255 | [M+Na]+ | 414.21259 | 77.00605:5821 85.02795:15976 119.08327:28298 173.38612:7537 | -9.6569E-08 |
| POS7490 | or-9-carboxy-Del | 5.167 | 345.20386 | [M+H]+ | 345.20389 | 55.05364:8745 57.03286:10758 57.06987:8631 59.04857:156108 60.08023:24029 67.05388:7682 73.0281:9297 79.05311:9225 81.06989:15609 83.04768:7036 85.02798:158970 86.03194:17410 87.04333:10279 89.05894:49265 93.06838:15565 95.08475:14207 97.06458:10748 101.05928:12799 103.07442:9886 105.06943:6111 107.08484:16588 121.06407:72357 123.08001:6829 131.08696:8149 133.1019:9366 135.07932:10998 143.08571:6090 144.25438:6320 145.10277:9176 147.07846:12472 155.08324:6498 157.10194:7401 163.1091:6871 173.09724:13813 297.94568:5678 | -8.6905E-08 |
| POS6795 | Estriol | 8.81 | 311.16177 | [M+2H]2+ | 311.16171 | 55.05365:58863 56.96447:7808 57.06895:432831 58.07271:19524 60.08024:27058 67.05389:88310 69.06867:69084 71.08546:254363 72.08836:10565 81.06832:104380 82.073:12499 83.08508:55048 85.10044:123869 86.0954:109906 87.09917:8681 89.05894:6052 95.08476:162792 96.08826:10354 97.09952:57875 102.09041:20834 104.10696:106566 109.10005:71073 111.1148:21223 115.96169:10215 116.98483:6866 123.11522:27162 124.99953:28113 130.99998:7779 150.03761:8254 166.54468:12846 173.43033:15135 181.02368:7195 184.06927:22644 240.09717:13120 | 1.92826E-07 |

**Differences in metabolites between the WJW group, the Model group and the Control group**

| Alignment ID | Metabolite name | Rt(min) | Expreiment Mz | Adduct type | Reference m/z | MS/MS spectrum | PPM |
|---|---|---|---|---|---|---|---|
| POS9465 | 28-Norbrassinolide | 6.426 | 467.33469 | [M+2H]2+ | 467.33459 | 57.03378:8878 57.53098:5601 59.04858:61853 87.04333:8149 122.83405:6319 128.02786:5926 173.42542:6550 | 2.13979E-07 |
| POS5599 | Enokipodin C | 6.059 | 265.14273 | [M+Na]+ | 265.1427 | 57.06894:271096 67.16013:5953 83.01192:16628 85.06333:68407 139.07352:6696 | 1.13147E-07 |
| POS11311 | , 24E)-Cholest-24 | 6.26 | 547.2934 | [M+2H]2+ | 547.29352 | 52.92344:5911 60.08025:8338 86.09541:12656 104.10696:89387 105.10879:9411 184.07466:15685 308.05939:6095 | -2.1926E-07 |
| POS14978 | -3-hydroxydecano | 6.997 | 780.54718 | [M+NH4]+ | 780.547 | 56.16686:6569 60.65643:4927 69.75047:6196 70.66898:5524 77.57815:5151 85.42:5496 92.73379:5286 148.68123:5243 152.18787:6049 173.3862:10346 345.00034:6225 772.16333:7117 | 2.30608E-07 |
| POS7879 | Bortezomib | 4.451 | 367.19409 | [M+H]+ | 367.194 | 55.05365:7737 62.02026:6111 69.03294:36387 74.89256:5499 83.04932:16016 86.05939:42416 87.04334:41179 89.05896:33192 93.07034:15025 98.05798:24268 98.65292:5798 99.04339:11100 111.04189:29575 112.07434:26726 113.07734:7178 128.07144:8579 133.08543:6665 152.10727:6122 173.34698:5856 178.11943:16843 317.81894:5571 | 2.45102E-07 |
| NEG4834 | ),5(R)-bis(hydroxy | 0.661 | 262.16595 | [M-H]- | 262.16611 | 84.03101:7416 100.04375:7708 | -6.103E-07 |
| POS3963 | -methyl-2-buteny | 6.297 | 205.1212 | [M+H]+ | 205.1221 | 69.90211:5326 72.95045:5817 91.0539:9489 | -4.3876E-06 |
| POS10645 | ysoPC(0:0/18:1(9Z | 6.902 | 522.35522 | [M+H]+ | 522.35522 | 128.86884:5368 184.0692:8379 | 0 |
| POS4448 | CHEMBL4283703 | 5.497 | 223.06314 | [M+H-H2O]+ | 223.0632 | 55.05365:8100 58.06512:6930 59.04858:16663 67.05389:6855 67.55789:6106 73.02811:8641 73.04555:15009 89.05896:9864 91.05576:12415 93.06841:16797 95.08477:8575 97.51802:7162 107.08486:6847 148.72409:5533 186.02084:6854 209.01401:7058 225.04044:77480 226.04431:8379 | -2.6898E-07 |
| POS12709 | ne-3alpha,29-diol | 7.894 | 603.40442 | [M+2H]2+ | 603.4043 | 76.80186:5699 145.42993:6409 173.43034:9974 296.44928:6733 | 1.98872E-07 |
| POS2609 | (R)-Boschniakine | 4.733 | 162.09058 | [M+CH3OH+H]+ | 162.0905 | 55.80161:6038 80.0266:7379 103.05195:17483 120.08028:56350 | 4.93551E-07 |
| POS14041 | E(16:1(9Z)/16:1(9Z | 8.694 | 688.4928 | [M+H]+ | 688.49115 | 116.11739:6684 173.43523:11648 324.71918:5487 384.25632:6124 | 2.39654E-06 |
| POS15090 | PG 38:7 | 6.733 | 793.5011 | [M+H]+ | 793.50128 | 55.14588:6653 60.88638:5143 66.33102:5834 78.19966:6200 143.06367:6466 160.10027:5317 243.58643:6061 359.39038:5954 611.99011:5960 | -2.2684E-07 |
| NEG1883 | Dihydroxynaphtha | 1.377 | 160.05307 | [M-H]- | 160.05243 | 51.09473:5957 57.65318:5568 58.00337:6717 59.08403:31394 70.57812:5376 71.09898:10088 73.11619:47205 73.99634:5682 85.13128:5671 101.14601:5693 150.50673:5630 | 3.99869E-06 |
| POS6562 | 4-Oxoretinol | 6.339 | 301.21536 | [M+H-H2O]+ | 301.21539 | 55.0177:11256 55.05365:7648 69.06867:18526 81.06832:6768 95.00317:5636 177.55406:6154 | -9.9597E-08 |
| POS6932 | Didemnilactone A | 5.088 | 317.21069 | [M+H-H2O]+ | 317.21069 | 55.01768:7347 55.05452:7069 57.06987:7703 67.05387:6094 69.06866:6327 71.08545:24519 79.05461:6074 81.06831:11657 83.75761:5774 99.07936:7885 105.06942:6155 108.6657:6482 117.06909:6488 129.07018:7550 143.08569:7294 175.81395:5798 | 0 |
| POS14521 | Javanicoside K | 7.456 | 729.29626 | [M+H]+ | 729.29639 | 53.35354:5571 | -1.7825E-07 |
| POS5266 | 2'-Methoxyflavone | 0.889 | 253.08517 | [M+Na]+ | 253.08521 | 70.98643:6400 79.20729:5666 90.97554:6469 91.85212:4897 173.38123:7053 178.11426:5697 216.29192:6822 225.03308:5615 | -1.5805E-07 |

**Differences in metabolites between the WJW group, the Model group and the Control group**

| Alignment ID | Metabolite name | Rt(min) | Expreiment Mz | Adduct type | Reference m/z | MS/MS spectrum | PPM |
|---|---|---|---|---|---|---|---|
| POS2874 | Azelaic acid | 5.981 | 171.10107 | [M+CH3OH+H]+ | 171.101 | 53.03772:6060 55.01768:58998 55.05364:20249 57.06894:642833 58.0727:7355 67.05387:8431 72.04367:23066 79.0531:14963 83.01192:449972 83.08669:5889 85.06334:206456 89.06976:6543 91.05387:11880 93.06838:20720 97.06458:7654 111.07957:14641 115.03664:25092 121.06406:8265 139.07353:9037 | 4.09115E-07 |
| POS5271 | 2'-Methoxyflavone | 8.761 | 253.08577 | [M+H]+ | 253.08549 | 70.40687:5541 73.03882:6438 133.87697:5330 213.12314:6659 242.1476:5910 | 1.10635E-06 |
| POS6668 | 3-Oxogeranyllinal | 6.936 | 305.24673 | [M+H]+ | 305.2467 | 52.71404:6652 115.55512:5844 | 9.82812E-08 |
| POS5270 | Dehydroeffusal | 6.458 | 253.08565 | [M+H]+ | 253.08569 | 53.29498:7137 95.92661:5268 129.79753:5642 150.35779:5993 251.99538:6043 | -1.5805E-07 |
| POS10586 | oPC(0:0/18:2(9Z,1 | 5.3 | 520.33905 | [M+H-H2O]+ | 520.33899 | 57.0338:18046 59.04859:455554 60.05226:11576 73.02812:27370 86.09541:8922 87.04335:53734 89.05897:271589 101.05931:34605 103.07445:89088 104.10697:36869 117.0909:11733 133.08545:61497 147.1015:32816 184.07468:44142 387.79099:10316 | 1.15309E-07 |
| POS3344 | orophenyl)prop-2 | 4.161 | 185.04076 | [M+CH3OH+H]+ | 185.0408 | 55.05363:9739 58.94248:9442 101.24716:5838 116.9712:31405 117.97749:26206 139.85522:5406 147.73953:4866 177.13303:5274 | -2.1617E-07 |
| POS1896 | Phosphonoacetate | 8.79 | 140.99532 | [M+NH4]+ | 140.99519 | 50.61313:7477 51.93935:6905 53.03856:8842 54.03373:7841 55.02909:45250 55.05365:9078 55.94513:44629 56.9414:14445 56.96447:9520 57.9342:627768 58.06511:187980 58.9425:28599 59.92951:71728 67.05389:9024 67.93391:26829 69.06991:20850 70.0643:38305 71.05973:16846 72.08047:9118 75.94611:12554 83.05907:36608 84.94554:12106 85.07516:57226 94.06358:6991 97.07692:10413 98.07049:20568 98.96088:40620 98.98203:8721 102.95542:6715 105.72243:7483 112.08707:64567 112.96638:9198 113.96311:9495 116.97124:453924 117.97478:22373 118.96619:46724 126.96738:10885 139.9868:49409 141.11403:45662 | 9.22017E-07 |
| POS6933 | Didemnilactone A | 5.508 | 317.21069 | [M+CH3OH+H]+ | 317.21069 | 78.49305:6069 86.90919:5969 97.06458:75463 109.06334:59271 153.79721:6272 173.39107:5731 184.94119:6312 232.74396:6119 | 0 |
| POS4735 | Alantolactone | 6.299 | 233.15237 | [M+Na]+ | 233.1524 | 152.50285:5564 | -1.2867E-07 |
| POS5496 | -octadeca-5,9,12- | 5.769 | 261.21991 | [M+H-H2O]+ | 261.22 | 67.05388:6690 91.05389:6161 | -3.4454E-07 |
| POS4643 | hydro-3H-pyrano[ | 4.824 | 230.11755 | [M+H]+ | 230.11758 | 55.01768:438388 55.05363:539904 57.03284:886288 57.06894:11108241 61.01:1178954 67.05386:506047 69.06989:1050960 79.05309:479473 81.03226:2900829 81.06987:401629 83.04766:1292620 85.02796:423432 85.06333:3656297 94.01654:219990 95.0489:253486 95.08473:223981 97.02761:297533 97.06457:372030 98.426:228350 107.04913:280327 111.04436:1397226 119.08328:196404 123.0448:183343 125.04754:457597 125.05955:6812293 133.06891:1254960 167.10457:14973471 | -1.3037E-07 |
| POS428 | Toluene | 4.381 | 93.06992 | [M+H]+ | 93.0698 | 57.03286:10207 70.25361:5628 95.04694:8249 | 1.28935E-06 |
| POS5956 | Prolyl-Tyrosine | 4.473 | 279.13336 | [M+H-H2O]+ | 279.13339 | 55.76759:6338 56.0486:70977 59.86378:5781 61.01:21312 67.05386:6587 68.04947:12414 70.06427:7602 70.77242:5663 81.06986:10469 84.04312:63320 86.05935:62849 88.02827:5406 95.08472:5874 102.05494:7477 103.05193:7343 104.05219:77849 120.08025:216265 130.04878:6549 132.06256:107539 133.02934:31081 150.0573:14199 | -1.0748E-07 |

**Differences in metabolites between the WJW group, the Model group and the Control group**

| Alignment ID | Metabolite name | Rt(min) | Expreiment Mz | Adduct type | Reference m/z | MS/MS spectrum | PPM |
|---|---|---|---|---|---|---|---|
| POS7916 | Cortol | 5.88 | 369.26291 | [M+H-H2O]+ | 369.26291 | 67.05388:11127 69.0699:11500 81.06989:13595 95.08475:10779 110.42126:6159 121.09841:8099 147.11679:7654 180.52777:6221 | 0 |
| NEG1495 | D-Lysine | 8.738 | 145.0985 | [M-H]- | 145.0985 | 50.06327:6758 64.09624:10247 65.09129:6982 77.10647:7974 84.03101:22843 100.0459:104530 101.05441:2798763 102.0561:244824 116.05927:96064 117.06683:50671 118.18127:60178 145.22426:7981 | 0 |
| POS194 | lethyl-3-buten-2- | 4.818 | 85.06468 | [M+Na]+ | 85.0647 | 56.0486:32294 84.04313:16884 84.07954:6052 | -2.3512E-07 |
| POS7956 | PARTEINE SULFAT | 5.765 | 371.1701 | [M+H]+ | 371.1702 | 76.54377:5973 173.39111:6509 182.17998:5526 | -2.6942E-07 |
| POS1076 | HLOROMETHYL) E | 9.304 | 114.97112 | [M+NH4]+ | 114.9711 | 55.0177:41067 55.05365:44699 58.06511:8999 59.04858:59962 60.04425:8361 61.01003:6709 62.98917:6479 68.04829:32860 69.03294:20927 69.06868:46368 70.02779:6245 70.0643:18437 72.04369:17074 72.08047:14898 72.93706:194456 73.04554:22057 73.93719:9384 79.05312:24758 80.05737:6212 86.05938:11508 87.04333:22870 90.94762:44448 91.05576:24234 96.08018:17580 97.0646:6942 108.9558:6241 113.96311:8090 114.06508:26326 114.09125:90865 115.09498:7965 | 1.73957E-07 |
| POS2871 | Azelaic acid | 5.671 | 171.10085 | [M+H]+ | 171.101 | 55.05363:20737 57.06986:32199 67.05386:6138 72.04366:30578 83.01192:20364 85.06502:6532 89.06976:12284 117.02013:5325 173.42537:8162 | -8.7668E-07 |
| POS3082 | 9E)-Trideca-3,5,7,9 | 6.409 | 177.16322 | [M+H-H2O]+ | 177.1633 | 55.05366:7912 81.0699:6023 83.68195:5881 88.02122:6673 92.61692:5662 93.06841:9043 102.63982:5740 107.08486:6140 121.1013:12055 | -4.5156E-07 |
| POS6235 | Sempervilam | 6.28 | 289.13318 | [M+H]+ | 289.1333 | 67.05389:10254 81.06989:6707 86.0954:23349 104.10696:26327 117.12083:5852 124.99955:8917 173.42543:11102 184.06929:12494 | -4.1503E-07 |
| POS4083 | Heteromine D | 4.832 | 209.12712 | [M+H]+ | 209.127 | 51.89836:5653 60.04323:6426 62.4356:5416 70.06427:22419 81.06829:5421 148.11047:10450 188.13643:5890 | 5.73814E-07 |
| NEG1489 | L-LYSINE | 6.807 | 145.09785 | [M-H]- | 145.09801 | 52.09783:7013 84.031:9349 100.04588:44311 101.05439:1161267 102.05608:101844 116.05926:45095 117.06681:15819 118.18125:15751 | -1.1027E-06 |
| POS337 | L-Alanine | 4.392 | 90.05499 | [M+CH3OH+H]+ | 90.055 | 61.02742:77191 64.32306:6811 69.61306:6290 72.08047:8526 | -1.1104E-07 |
| POS1833 | Suberic acid | 6.058 | 139.07501 | [M+H]+ | 139.075 | 50.5135:6387 55.01768:12965 55.05363:10633 57.06894:14914 58.06509:12052 73.30093:5932 83.01192:63402 93.0703:5907 95.04691:8212 | 7.19036E-08 |
| POS3147 | xy-5-phenyl-3-pe | 6.299 | 179.10619 | [M+NH4]+ | 179.1062 | 91.05389:11793 105.06944:10473 119.08332:10252 | -5.5833E-08 |
| POS4675 | Norpterphyllin III | 0.75 | 231.10406 | [M+NH4]+ | 231.104 | 55.93433:12000 57.03284:6762 57.06894:71414 69.06989:8521 79.05309:7886 81.03226:18224 83.04766:10137 85.06333:20585 111.04436:9389 125.05955:49088 133.06889:8965 167.10455:88462 | 2.59623E-07 |
| POS3367 | -2,2,6-trimethyl-1 | 6.467 | 185.15295 | [M+H]+ | 185.153 | 50.34662:5815 57.06896:31669 58.9425:10792 83.08509:6768 91.78413:5392 96.43512:5643 114.02061:6970 116.97125:33797 117.97753:35894 139.9868:8151 | -2.7005E-07 |
| POS1258 | Hydroxybenzoic ac | 6.414 | 121.02811 | [M+H]+ | 121.028 | 51.98441:6202 52.08194:5974 65.03805:30519 86.61761:5929 120.37191:5659 | 9.08881E-07 |
| POS1778 | 4-Propylphenol | 4.815 | 137.09586 | [M+NH4]+ | 137.0959 | 53.61392:6146 53.8126:5696 67.05386:18617 72.8742:5711 77.83262:6596 79.05309:11525 81.06987:39587 91.05386:9088 95.08473:16437 | -2.9177E-07 |
| POS7648 | Grandilobatin A | 6.612 | 353.26736 | [M+H]+ | 353.2674 | 67.05388:6123 91.09308:6433 107.08484:7637 119.67999:6221 140.46465:5768 | -1.1323E-07 |
| POS4854 | Palmitoleic acid | 6.324 | 237.22009 | [M+H-H2O]+ | 237.22 | 69.06869:6628 81.0699:9704 107.08487:6414 232.43169:6150 | 3.79395E-07 |
| POS247 | 3-Aminopyrrolidine | 8.756 | 87.09145 | [M+NH4]+ | 87.0916 | 86.05938:18741 87.09045:69839 | -1.7223E-06 |

**Differences in metabolites between the WJW group, the Model group and the Control group**

| Alignment ID | Metabolite name | Rt(min) | Expreiment Mz | Adduct type | Reference m/z | MS/MS spectrum | PPM |
|---|---|---|---|---|---|---|---|
| POS2178 | ialic acid 4-Me est | 2.683 | 149.04434 | [M+H]+ | 149.0444 | 65.03803:6116 91.05762:6280 103.0542:6043 103.80193:5951 | -4.0256E-07 |
| POS7699 | 2-Linoleoyl Glycerc | 6.877 | 355.28223 | [M+H]+ | 355.2821 | 101.92889:5706 142.24037:5452 153.16835:5057 173.43524:8239 | 3.65906E-07 |
| POS10736 | | 4.66 | 525.30688 | [M+H-H2O]+ | 525.30713 | 69.03294:67522 73.02811:187355 73.06432:73247 80.05429:75077 81.03385:33790 83.0477:34944 87.04334:431912 88.04604:27180 89.05896:2465756 90.0619:86112 91.04643:88865 95.04893:88450 99.04339:69196 102.06606:66841 107.06821:25147 111.04441:100871 113.05927:70640 117.08817:29401 124.08022:23947 131.06766:68756 133.08543:860051 134.08684:45384 137.0598:61376 155.07083:33039 177.11285:91886 | -4.7591E-07 |
| POS14851 | xy-12,19(29)-ursac | 4.666 | 765.43976 | [M+2H]2+ | 765.44 | 59.04858:189374 69.03294:107946 73.02811:457562 73.06432:231440 81.03229:104140 87.04334:1581831 89.05895:7716564 90.06374:158789 91.04643:139834 95.04893:226545 99.04339:207252 109.06335:110190 111.0444:307058 113.05927:264513 117.09089:189153 131.06764:339911 133.08543:4354042 134.09016:181612 137.0598:223439 146.80688:80855 155.07083:299185 175.09526:154709 177.11284:864362 178.11432:109079 199.09537:129018 221.14047:110403 243.12985:79798 | -3.1355E-07 |
| POS5546 | enyl beta-D-gluco | 4.585 | 263.14828 | [M+2H]2+ | 263.1485 | 69.03294:7268 87.04334:5508 171.10925:6106 191.00081:5391 253.66177:6313 | -8.3603E-07 |
| POS15066 | Okadaic acid (-H2C | 4.657 | 787.46161 | [M+2H]2+ | 787.46198 | 64.15166:115119 87.04332:165423 89.05893:744046 133.0854:434553 282.94159:115379 783.29553:120371 | -4.6986E-07 |
| POS3418 | hiophen]2-<Octat | 1.022 | 187.05727 | [M+CH3OH+H]+ | 187.0575 | 62.55975:6475 67.44471:5435 70.06427:6321 81.06986:8247 101.83167:6262 | -1.2296E-06 |
| POS8693 | Ugonin P | 5.456 | 423.1788 | [M+Na]+ | 423.17899 | 59.04856:6510 69.68794:6085 85.02796:14425 96.01747:71660 108.74114:5448 138.02629:170491 173.43518:6079 180.07538:49427 193.08113:254384 194.08763:12209 213.98772:5948 218.55022:5748 338.6218:5873 | -4.4898E-07 |
| POS10416 | e from Mulder Bioc | 5.345 | 513.34473 | [M+2H]2+ | 513.34497 | 57.03284:27899 59.04856:448896 60.05223:12018 69.06988:6563 73.02808:29925 73.06429:11442 85.06501:14269 87.0433:62729 87.07994:11068 89.05891:367724 90.06187:9711 94.06942:10331 101.05926:60205 103.07439:106553 104.10692:6321 115.07638:11671 117.09084:7292 129.08905:9081 133.08537:99502 147.10141:45184 191.1255:7629 333.50156:7036 379.29004:6238 | -4.6752E-07 |
| POS4666 | Norpterphyllin III | 5.591 | 231.10378 | [M+Na]+ | 231.104 | 53.03854:9765 55.01768:12449 55.05363:23575 57.03284:29823 57.06894:299791 61.01:36048 67.05386:14375 69.06865:26022 79.05309:12429 81.03226:83789 81.06987:16441 83.04766:36642 85.02796:14007 85.06333:103688 93.06837:6266 95.08473:8876 97.02761:13545 97.06457:11658 107.04913:7903 111.04436:33797 123.0448:6083 125.05955:244129 126.06273:6287 133.06891:40815 167.10457:465402 168.11116:22316 173.39104:7654 | -9.5195E-07 |
| POS11896 | Cinnamoyl-vulgarc | 4.745 | 567.33191 | [M+Na]+ | 567.3316 | 58.32636:6288 87.04335:6304 89.05896:14008 101.41589:5569 147.08617:5377 148.92294:5751 173.39111:10389 192.58664:6191 392.82523:6321 503.2316:5948 | 5.46418E-07 |

**Differences in metabolites between the WJW group, the Model group and the Control group**

| Alignment ID | Metabolite name | Rt(min) | Expreiment Mz | Adduct type | Reference m/z | MS/MS spectrum | PPM |
|---|---|---|---|---|---|---|---|
| POS6696 | 4-Gallocatechol | 3.027 | 307.08179 | [M+2H]2+ | 307.0816 | 56.04862:16784 68.02298:7664 76.0215:19080 76.03858:56598 84.04315:369127 96.04378:9515 102.05498:9126 130.04883:145558 131.04509:24300 140.01526:12904 177.03188:30486 179.0471:13981 | 6.18728E-07 |
| NEG2957 | Monuron | 1.216 | 197.049 | [M-H]- | 197.0488 | 55.23539:6281 68.33942:6047 73.52995:5450 81.53988:5503 161.03627:460728 162.03664:28802 163.0374:1032564 198.04526:168570 | 1.01498E-06 |
| POS12097 | ethyl]-10,14,16,16 | 4.692 | 575.00769 | [M+CH3OH+H]+ | 575.00739 | 73.02811:29511 87.04334:93472 89.05896:469726 90.0619:14744 95.04893:13062 99.04339:14744 99.79063:13882 113.05927:17085 131.07088:13450 133.08543:160500 177.11285:21906 366.98584:13541 | 5.21732E-07 |
| POS6773 | 6-Hydroxynobiline | 4.717 | 310.20068 | [M+Na]+ | 310.2009 | 55.05452:9349 57.09766:6312 59.04858:12269 60.08024:15333 85.02798:148494 93.06839:6424 121.10128:7372 131.23537:7547 | -7.0922E-07 |
| POS9402 | LysoPE(18:0/0:0) | 6.085 | 464.31125 | [M+H-H2O]+ | 464.311 | 57.03286:12124 59.04955:8892 342.63168:5391 | 5.38432E-07 |
| POS1671 | L-Alanine | 0.975 | 134.01877 | [M+NH4]+ | 134.019 | 62.98165:6876 70.06429:208419 71.06873:13251 74.02327:22845 90.05455:18754 91.05762:41642 92.05856:6291 93.26151:5477 115.08436:6481 116.06902:6879 133.07881:8893 | -1.7162E-06 |
| POS4659 | xo-1,2,3,4-tetrahy | 0.757 | 231.0336 | [M+H]+ | 231.0334 | 75.13534:6435 86.99104:18560 94.8403:5815 101.00695:12948 105.0024:19452 106.35088:5540 109.73916:5246 113.00764:8928 119.01637:16041 129.00409:13318 131.01932:16179 147.01338:23615 | 8.65676E-07 |
| POS4682 | Eremanthin | 4.376 | 231.13277 | [M+H-H2O]+ | 231.133 | 55.05365:24699 56.04862:40425 57.03286:18158 57.06988:19215 58.06511:8324 60.08024:104564 69.06991:17024 71.04816:10536 72.08047:78270 84.07957:178283 85.02798:890626 86.0954:77961 100.07381:50362 112.07433:16446 126.09013:85200 129.1017:10055 132.10173:9434 144.10196:9662 168.13461:19759 173.0777:11329 214.14249:8406 | -9.951E-07 |
| POS15383 | ,13-dimethylhexa | 7.329 | 1009.69946 | [M+H-H2O]+ | 1009.70001 | 50.41728:4625 51.16747:4355 67.85581:5258 68.19077:5133 69.09335:4538 69.98:5230 73.86624:4573 87.78227:5144 87.80347:4915 89.77258:4853 102.61972:5708 109.25687:4829 109.90973:5719 111.15759:5039 112.63448:5961 115.85182:6537 175.85406:6312 252.27922:8560 | -5.4472E-07 |
| POS2821 | -ureidobutyric aci | 1.049 | 169.05814 | [M+H]+ | 169.05832 | 52.11343:6093 55.02822:24863 67.05389:8183 68.7358:5188 69.00831:10412 70.03912:49662 79.05312:7897 81.06989:12314 96.0175:22582 98.03503:31566 114.06246:6020 116.72981:5296 124.01196:8099 126.02934:37045 141.03847:76945 151.04518:12385 152.00658:21390 169.0325:59061 | -1.0647E-06 |
| POS10245 | Yardenone A | 7.332 | 505.35059 | [M+2H]2+ | 505.35031 | 173.3911:17792 | 5.54071E-07 |
| POS5564 | Z)-Octadecadien | 6.288 | 263.23578 | [M+H-H2O]+ | 263.23599 | 55.05364:9969 57.06895:5869 62.58315:5822 67.05388:24081 69.0699:7106 81.06831:24764 83.08508:6428 93.06839:10323 95.08475:20730 107.08484:6287 109.10004:13846 121.10127:6250 193.25415:5595 | -7.9776E-07 |
| POS10783 | -Indocarbazostati | 1.459 | 527.15625 | [M+H]+ | 527.15601 | 55.55447:5626 127.59034:5621 144.60855:5867 163.10468:6399 | 4.55273E-07 |

**Differences in metabolites between the WJW group, the Model group and the Control group**

| Alignment ID | Metabolite name | Rt(min) | Expreiment Mz | Adduct type | Reference m/z | MS/MS spectrum | PPM |
|---|---|---|---|---|---|---|---|
| POS4668 | Norpterphyllin III | 8.925 | 231.10378 | [M+CH3OH+H]+ | 231.104 | 55.01768:8285 55.05363:16588 55.93343:27143 57.03284:14107 57.06894:178894 61.01:23672 61.75778:5917 67.05386:7067 69.06865:17043 73.63633:5686 79.05309:10705 81.03226:47980 81.06987:12417 83.04766:18076 85.02796:14311 85.06333:50272 97.02761:8872 97.06457:8835 111.04436:24819 114.97041:24398 123.0448:6417 125.05955:131348 133.0656:20110 167.10457:251841 168.11116:10679 | -9.5195E-07 |
| POS2428 | hosphoglycolic aci | 8.819 | 156.98994 | [M+CH3OH+H]+ | 156.98959 | 52.57622:5798 53.93831:20328 55.0177:6849 55.05365:18289 56.04952:11259 57.9342:16022 70.0643:8385 70.94153:8906 72.93706:21304 73.93719:8762 74.93712:198919 84.07957:7913 86.82748:5832 90.94576:6888 92.94704:32875 97.0646:12623 110.05835:7187 112.97669:6802 113.96311:7086 115.95633:28188 115.96169:84824 116.97124:8238 128.06833:6180 133.97353:17542 | 2.22945E-06 |
| POS1158 | -Ethylbutanoic aci | 5.974 | 117.09066 | [M+H]+ | 117.0909 | 55.05365:17360 64.34301:5615 65.67461:6123 70.0643:125691 71.06874:10479 72.08047:30359 | -2.0497E-06 |
| POS2896 | lole-3-propionic a | 5.061 | 172.07523 | [M+H-H2O]+ | 172.075 | 55.0177:67069 55.05366:45425 57.06989:8313 59.04859:10030 67.05389:105780 71.04816:8019 72.04369:39478 81.06834:15025 82.69934:6493 83.0851:13674 89.06979:11754 95.8057:5137 103.05422:7244 109.06336:6309 109.10007:35552 130.06477:157420 144.00543:6544 | 1.33663E-06 |
| POS3719 | Lactarazulene | 6.298 | 197.13197 | [M+Na]+ | 197.1322 | 68.17625:5403 80.64205:5329 100.70676:5930 127.82278:5754 136.63005:5533 161.47514:6497 167.6394:5486 | -1.1667E-06 |
| POS1881 | Na-Dimethylhistan | 8.768 | 140.11795 | [M+Na]+ | 140.1181 | 52.81611:7314 53.03773:8905 54.03287:5940 55.02822:65891 58.06416:292029 69.06868:37091 70.0643:46062 72.08047:17138 80.04967:6099 83.05907:65928 87.20751:5633 88.63091:6419 97.07487:7111 98.083:7589 123.09176:6408 | -1.0705E-06 |
| POS607 | mino-2-methylbu | 8.779 | 99.09141 | [M+H-H2O]+ | 99.0916 | 50.09744:5228 50.32975:5594 50.40268:5236 50.46883:5745 52.30545:4833 54.25877:4925 54.44379:5359 55.05365:23090 55.83027:6131 56.27641:5374 58.25276:5211 58.26422:5113 60.29585:5899 60.89658:5148 61.73696:4792 66.15387:3998 74.47533:4653 75.93189:5489 78.03951:4594 84.36342:4230 87.3108:4715 87.74342:4879 91.89187:4448 96.18139:4135 96.32538:3919 96.68372:5059 97.55318:4156 99.13871:4426 | -1.9174E-06 |
| POS5077 | 10,11,12,14,15,16,1 | 6.289 | 245.22614 | [2M+H]+ | 245.2263 | 57.05414:6045 59.16666:5253 67.25835:6028 85.66637:6272 113.07216:5864 125.9018:5442 132.26817:5515 214.60085:5742 | -6.5246E-07 |
| POS4443 | Aminoanthraquino | 4.347 | 223.06284 | [M+H-H2O]+ | 223.063 | 58.06414:18982 61.03867:16876 73.02809:6988 73.04685:17831 91.05573:21622 93.06644:6978 118.53261:5827 159.96542:6065 209.01393:10592 225.04034:69868 | -7.1729E-07 |
| POS3345 | phtho[2,1-b]thioph | 1.326 | 185.04166 | [M+H]+ | 185.0419 | 55.05363:8202 62.56081:6476 113.0902:5627 | -1.297E-06 |
| POS2945 | thyl-1,2-dihydron | 7.21 | 173.132 | [M+H-H2O]+ | 173.1322 | 50.28837:5925 88.32349:6088 | -1.1552E-06 |
| POS2649 | Geijeren | 7.249 | 163.1476 | [M+H]+ | 163.1478 | 63.15053:5460 63.16023:5160 119.3293:5345 | -1.2259E-06 |

**Differences in metabolites between the WJW group, the Model group and the Control group**

| Alignment ID | Metabolite name | Rt(min) | Expreiment Mz | Adduct type | Reference m/z | MS/MS spectrum | PPM |
|---|---|---|---|---|---|---|---|
| POS158 | )-methylbutanenit | 5.967 | 84.08052 | [M+H]+ | 84.0807 | 50.23862:6708 52.12395:4311 56.04863:4952 58.34167:3949 58.59901:4274 59.41376:5032 60.71431:4162 62.00978:4429 62.5035:4784 65.93027:5282 70.29284:4345 70.38533:4521 70.97234:3916 71.45245:4370 74.19456:4335 76.51501:4700 80.29636:5403 81.71966:4195 82.41099:4707 84.04317:8207 86.10914:4745 | -2.1408E-06 |
| POS4186 | Thiolactomycin ?? | 4.814 | 213.09402 | [M+H-H2O]+ | 213.0943 | 53.03772:39754 55.01768:27279 55.05363:8960 57.06894:755510 58.07269:14496 61.01:111636 64.67905:6122 67.05386:16043 68.99598:7803 73.01067:9754 81.03226:159019 85.06333:93695 87.02587:8795 91.02029:8021 95.0489:22413 95.08473:9010 97.02761:11801 101.03963:9227 109.10002:8852 123.04187:13448 129.03552:6515 133.0656:31378 137.09767:9771 166.09779:11435 | -1.314E-06 |
| POS13869 | vernonioside B3 | 4.562 | 675.37421 | [M+NH4]+ | 675.37378 | 59.04858:37087 69.03294:269041 73.02811:92499 73.06432:73920 81.03385:33829 83.0477:105986 85.02631:26682 87.04334:613385 89.05896:1780757 90.0619:30257 95.04893:48746 99.04339:436998 111.04441:239063 113.05927:40765 117.09089:47411 122.54804:27416 125.0596:104342 129.05446:51720 131.07088:62767 133.08543:703142 134.08684:34437 137.05981:51558 155.07083:146177 173.07771:30422 177.11285:80850 | 6.36684E-07 |
| NEG5237 | Fissoldhimine | 0.955 | 277.16745 | [M-H2O-H]- | 277.16711 | 90.03759:5693 93.03925:2699770 97.03745:10799 102.00297:5516 103.07946:40858 141.08502:6409 151.06711:63717 173.78252:6615 | 1.2267E-06 |
| POS12824 | Malyngamide J | 4.571 | 608.3797 | [M+NH4]+ | 608.37927 | 73.06432:37063 87.04334:182952 89.05896:1542307 90.0619:39641 117.09089:19249 131.07088:54281 133.08543:670589 134.08684:26788 177.11285:88397 553.66919:19689 | 7.06796E-07 |
| POS11942 | Hyperbrasilol A | 4.54 | 569.31042 | [M+Na]+ | 569.31079 | 243.29276:15178 | -6.4991E-07 |
| NEG5354 | Oprea1_249309 | 0.955 | 281.16565 | [M-H2O-H]- | 281.16599 | 65.52292:5964 75.06394:6474 93.03932:317154 95.0377:1006561 97.03753:253909 103.08179:10856 105.08054:8355 153.06766:13105 155.06664:13855 | -1.2093E-06 |
| POS10732 | etramethyl-12-oxo | 4.524 | 525.28528 | [M+Na]+ | 525.28497 | 104.10696:90124 116.99842:13251 184.06927:53077 | 5.90156E-07 |
| POS9325 | BW A868C | 4.53 | 460.28018 | [M+H]+ | 460.28049 | 70.35613:12729 71.03398:12288 72.08044:54452 82.99567:12845 84.07954:17718 86.09536:14624 87.0433:20802 89.05891:358132 90.06187:46690 120.08025:36316 133.08537:85632 173.42535:17198 457.95001:14539 | -6.735E-07 |
| POS12839 | ctyl)-1,4,7,10-tetra | 4.523 | 609.30444 | [M+H]+ | 609.30487 | 68.41991:7554 87.04333:35840 89.05895:261702 90.0619:17221 116.29226:8484 133.08543:93186 134.09016:11636 177.11284:16533 580.45636:8024 | -7.0572E-07 |

**Differences in metabolites between the WJW group, the Model group and the Control group**

| Alignment ID | Metabolite name | Rt(min) | Expreiment Mz | Adduct type | Reference m/z | MS/MS spectrum | PPM |
|---|---|---|---|---|---|---|---|
| POS3951 | ethylsulfanyl)propy | 4.535 | 205.05252 | [2M+H]+ | 205.0528 | 55.01768:78959 57.03284:19077 60.04324:9173 69.03291:7160 74.02325:8180 79.05309:53662 81.0683:31984 83.04929:7404 85.02796:34856 91.05386:8945 93.06837:10499 95.0489:7755 97.06457:63387 108.05521:12513 108.25813:6309 115.05253:9964 117.06908:12983 118.06285:184366 121.06405:7455 125.05955:449338 126.06273:14118 130.06154:7599 132.04301:8610 132.07886:32021 142.06561:8152 143.07098:14918 144.07964:52961 146.05714:223572 159.09131:17284 170.06129:9758 173.38614:9934 178.04791:8678 188.06996:43958 | -1.3655E-06 |
| NEG6992 | Ferocin | 0.963 | 339.19595 | [M-H2O-H]- | 339.19629 | 64.28277:5751 68.55454:6419 69.57709:5841 93.03925:226395 95.03763:278225 97.03745:46623 153.06755:27307 155.06651:12092 | -1.0024E-06 |
| POS2508 | Cleroindicin B | 4.932 | 159.10106 | [M+H]+ | 159.10139 | 53.68312:5904 55.05363:8822 55.93433:23564 56.0486:7073 57.06894:9653 58.07365:5503 66.47277:5330 69.06865:6803 70.06428:9016 71.01213:10431 71.04942:6172 76.93209:8708 104.37206:5536 | -2.0741E-06 |
| NEG5133 | labda-13(16),14-d | 1.039 | 273.25922 | [M-H]- | 273.25891 | 51.7709:5912 52.92688:7048 87.11259:26638 89.13132:620403 90.13493:24973 91.84886:6244 93.03932:615230 95.0377:199293 97.03753:78373 103.07954:38911 128.18695:6126 136.98956:5688 145.2356:10087 147.15926:225151 209.7704:5888 | 1.13446E-06 |
| POS1084 | Siphonodin | 6.062 | 115.03861 | [M+CH3OH+H]+ | 115.0389 | 55.01768:6203 55.05363:10908 68.04948:7986 69.06865:13654 70.06428:11377 75.8084:5719 83.01192:103439 96.24216:6652 107.414:5775 107.7495:5746 114.06504:6739 114.09121:16709 | -2.5209E-06 |
| NEG2413 | ,2,7,8-Octanetetro | 0.924 | 177.11366 | [M-H]- | 177.1133 | 74.1126:10026 93.03925:30520 128.69928:6126 161.08009:5411 | 2.0326E-06 |
| POS5264 | Ribose 5-phospha | 1.199 | 253.00798 | [M+H]+ | 253.00832 | 61.03971:9646 68.36646:5653 68.68076:5543 87.04333:5534 104.36064:5765 120.96402:9389 149.96265:5262 | -1.3438E-06 |
| POS9013 | Lovastatin | 4.419 | 443.23703 | [M+H]+ | 443.2373 | 70.06431:6500 82.91782:5885 89.05896:176541 118.06566:20414 132.07892:12359 133.08545:90295 136.07611:17369 144.0797:18937 146.061:96159 159.09138:16799 177.11285:8095 188.07004:385374 189.07106:24890 205.09822:73238 239.14922:15919 | -6.0915E-07 |
| POS11316 | yl-O-acetylbuxalo | 6.375 | 547.35266 | [M+H]+ | 547.35303 | 55.87151:5746 57.06989:10179 61.21729:5652 62.39856:5583 71.08547:7034 78.10174:6275 86.0954:23001 129.75313:5718 146.98277:20296 184.07465:16940 | -6.7598E-07 |
| POS7110 | kasarin | 4.438 | 326.17065 | [M+NH4]+ | 326.17099 | 57.06894:28962 70.06554:5563 74.73689:6539 85.06334:9850 87.04332:8115 89.05893:97753 98.51205:5351 110.07072:30468 122.05131:6042 133.0854:19736 | -1.0424E-06 |
| POS8621 | EnP(5,8) | 5.227 | 417.33533 | [M+H-H2O]+ | 417.3356 | 57.03286:18918 59.04858:302286 73.02811:18571 81.06832:25269 87.04333:25195 89.05895:135926 99.07938:32106 101.0593:16746 103.07443:36156 107.08485:7418 109.10005:8718 130.55034:6076 133.08543:19907 147.10147:9836 159.40642:5982 173.43523:10533 196.30745:5690 416.92136:6788 | -6.4696E-07 |
| POS4936 | Pirbuterol | 4.42 | 241.15355 | [M+H]+ | 241.1539 | 58.06511:13072 69.03294:8028 70.0643:11751 84.07957:7558 95.08477:10598 98.06007:17179 110.09556:5558 134.09351:8895 138.08907:38755 | -1.4514E-06 |

**Differences in metabolites between the WJW group, the Model group and the Control group**

| Alignment ID | Metabolite name | Rt(min) | Expreiment Mz | Adduct type | Reference m/z | MS/MS spectrum | PPM |
|---|---|---|---|---|---|---|---|
| POS13628 | Parisin | 6.429 | 657.49316 | [M+H]+ | 657.49353 | 57.03286:138037 58.03661:6859 58.22031:5838 59.04858:35475 78.02471:7108 99.07938:15568 115.07377:8433 126.9121:6505 126.93359:6413 140.10425:5512 145.29819:5482 157.11888:14588 346.10397:5472 461.44281:6575 652.27557:5618 | -5.6274E-07 |
| POS5044 | -11H-dibenzo[b,e | 0.877 | 245.0472 | [M+Na]+ | 245.0475 | 51.55165:7066 52.56477:6209 53.31757:6191 65.14406:6541 65.15309:6011 69.5333:5667 177.06224:34769 224.77242:5997 | -1.2243E-06 |
| POS5114 | hyl)methyl]-N-ethy | 1.172 | 247.09932 | [M+H]+ | 247.09959 | 72.08044:6734 95.30415:4812 101.79417:5815 111.04436:6252 125.05955:7002 160.12196:6138 167.10455:13925 | -1.0927E-06 |
| POS3846 | Lacinilene A | 6.296 | 201.12679 | [M+H-H2O]+ | 201.12711 | 64.187:5540 66.59401:6007 91.05389:9508 105.06944:6709 117.06911:7199 129.06705:13625 131.08376:29337 145.10278:14647 157.10196:18799 173.10216:6734 173.396:8191 | -1.591E-06 |
| POS9643 | hylphenylalanylarg | 6.193 | 478.28867 | [M+H]+ | 478.28839 | 54.62195:6208 62.57891:5577 134.27377:6357 397.29398:5879 | 5.85421E-07 |
| POS1062 | 5-Dimethylpiperidi | 4.488 | 114.12732 | [M+H]+ | 114.1276 | 53.03857:12167 54.03373:11727 55.0177:189439 55.05365:462522 58.02808:15578 67.05389:110907 68.04829:98581 69.06868:682557 70.06431:86380 71.04816:32870 71.08547:39319 72.08047:69497 77.03802:27084 79.05312:650159 81.05579:13126 84.07957:14653 86.09541:46098 95.04893:44642 96.08018:307102 97.06461:103540 105.04401:13777 114.09126:1768146 115.09234:18092 | -2.4534E-06 |
| POS5387 | 3-Deoxyestradiol | 6.297 | 257.18881 | [M+NH4]+ | 257.18909 | 57.53942:7335 57.62482:5391 83.3492:5597 142.84343:5835 | -1.0887E-06 |
| POS7211 | Sclareol | 6.5 | 331.26044 | [M+H]+ | 331.26071 | 55.05365:16712 57.06988:10107 65.03015:5403 67.05389:23387 69.06867:18376 79.05312:10309 81.06832:16159 83.08508:6287 91.05389:19068 93.0684:13151 95.08476:16426 105.06944:38936 119.08332:19632 121.10129:9555 131.08376:16476 133.10191:9633 145.10278:7045 155.82838:5614 | -8.1507E-07 |
| POS2187 | -Tetrahydro-2-na | 6.3 | 149.09537 | [M+H]+ | 149.0957 | 99.37657:5830 103.77468:5501 | -2.2133E-06 |
| POS1414 | SCHEMBL1137702 | 1.639 | 126.091 | [M+CH3OH+H]+ | 126.0913 | 53.03856:20679 55.0177:13306 56.04952:7110 70.06556:11281 80.04813:21980 81.03228:8774 98.05797:15555 108.04319:12716 111.99284:5768 126.09013:7626 127.03809:12474 | -2.3792E-06 |
| POS716 | Triethylamine | 8.459 | 102.12726 | [M+H]+ | 102.1276 | 50.32131:5604 50.48732:4526 51.19893:5189 51.50716:4412 52.29895:5871 52.71486:5036 53.88306:4640 54.18246:5254 55.00543:5181 59.37344:4488 60.23155:4397 60.9456:4849 61.22037:4370 68.22222:4167 70.48689:4791 70.78522:4244 71.1834:5263 71.56672:4789 87.3178:4485 87.85827:3923 88.16148:5554 91.93539:4267 92.01117:4186 92.1326:4756 92.31329:4411 93.96178:5724 96.02963:4223 96.6327:5515 98.03295:4386 | -3.3292E-06 |
| POS3840 | Matsutakic acid A | 9.749 | 201.11136 | [2M+H]+ | 201.11169 | 55.05365:227960 55.93435:129434 56.9414:111913 59.04858:14787 67.05389:7464 69.06991:6620 70.95692:13366 72.93706:49809 72.95981:12300 73.94402:8057 74.95245:24945 81.06989:9887 83.04769:43832 90.94762:32130 96.95995:11195 97.96836:93609 100.96772:13857 101.0593:69034 111.04189:43725 113.96311:482955 114.97044:68490 116.96038:12450 118.94112:14331 129.05446:14695 131.97466:90264 132.97011:21142 141.95662:110573 155.00035:7223 159.96982:69588 | -1.6409E-06 |

**Differences in metabolites between the WJW group, the Model group and the Control group**

| Alignment ID | Metabolite name | Rt(min) | Expreiment Mz | Adduct type | Reference m/z | MS/MS spectrum | PPM |
|---|---|---|---|---|---|---|---|
| POS7953 | trophenyl-L-Trypt | 6.984 | 371.09888 | [M+H]+ | 371.09857 | 173.44016:9933 | 8.35358E-07 |
| POS6849 | xo-nonadecanoic | 0.736 | 313.27216 | [M+CH3OH+H]+ | 313.27289 | 57.06988:10981 57.52068:6191 124.39247:6272 161.19786:5075 | -2.3302E-06 |
| POS7966 | conessine | 1.238 | 371.34229 | [M+H]+ | 371.34201 | 57.06989:9314 59.03105:42755 61.01003:14827 71.08548:6140 73.04555:31922 75.02498:34684 77.04093:20410 91.05577:23723 93.03564:23681 105.07177:9840 237.84406:5418 314.16376:6156 | 7.54022E-07 |
| POS9711 | 2-ylanilino)propar | 4.506 | 481.26016 | [M+Na]+ | 481.25977 | 133.00633:5343 173.39601:10702 190.73463:6381 353.4512:6282 | 8.10373E-07 |
| NEG7218 | oro-10-hydroxyo | 0.947 | 347.2363 | [M-H]- | 347.2359 | 50.12337:9028 60.17111:6402 76.71121:5469 82.79803:6263 93.03925:337442 95.03763:217009 97.03745:11363 103.07946:11923 151.06711:9117 153.06754:13092 338.8721:6308 | 1.15195E-06 |
| POS2926 | Xanthopappin A | 1.019 | 173.04153 | [M+Na]+ | 173.0419 | 70.06431:9695 173.04349:21661 175.89915:6307 | -2.1382E-06 |
| POS2777 | (E)-Lyratic acid | 8.914 | 167.10579 | [M+H]+ | 167.1062 | 53.93914:9285 84.95896:29778 103.05193:7594 112.97408:8555 119.39087:5587 120.08025:17724 125.05955:14703 | -2.4535E-06 |
| POS6152 | Myristoylglycine | 6.484 | 286.23709 | [M+H]+ | 286.23749 | 55.39032:5951 57.06896:39152 67.05389:7861 69.06992:7043 71.08548:23636 76.03859:91189 85.10045:6830 95.08477:13941 121.30764:5824 | -1.3974E-06 |
| POS9324 | yptophylvalylargini | 5.752 | 460.2662 | [M+H-H2O]+ | 460.2666 | 59.04858:13547 79.6314:5536 89.05895:13544 119.08332:58896 135.07935:9482 152.16771:5365 173.38618:5401 | -8.6906E-07 |
| POS3442 | 4-(pyridin-2-yl)bu | 4.12 | 188.06781 | [M+H]+ | 188.06822 | 55.05365:9874 83.91093:5305 113.4112:6295 131.41315:6112 | -2.1801E-06 |
| POS13937 | DTXSID60346618 | 8.778 | 679.47156 | [M+H]+ | 679.47198 | 65.3966:5920 198.67972:6253 272.09207:5880 290.05417:6865 | -6.1813E-07 |
| POS2031 | Ethephon | 6.504 | 144.9819 | [M+H-H2O]+ | 144.98151 | 53.03773:13579 55.01682:9119 55.05365:10583 57.03286:6811 62.92802:1139831 70.0643:47409 71.06874:6732 80.93837:9232 98.06006:32443 98.09552:15845 99.04338:23092 103.95428:45847 121.96451:462033 131.96162:6944 144.98274:31577 | 2.69E-06 |
| POS7164 | Incensole | 6.838 | 329.24548 | [M+H]+ | 329.24512 | 76.35284:6858 107.08486:6808 170.07565:5289 173.3911:10170 328.86789:6140 | 1.09341E-06 |
| POS6401 | ydroxyhexadecano | 5.946 | 295.2247 | [M+H]+ | 295.22433 | 59.04857:7603 67.05388:19576 69.06866:9868 79.05311:13119 81.06831:20876 83.08508:14072 91.05388:6351 93.06838:13704 95.08475:14161 107.08484:9073 121.0984:15495 135.11641:11977 165.01813:6026 258.12753:6279 258.19879:5911 275.03635:6959 | 1.25328E-06 |
| POS1349 | 2,4-Triaminobenze | 8.729 | 124.08641 | [M+H]+ | 124.0868 | 61.37498:5859 77.49311:6051 96.20368:6753 | -3.143E-06 |
| POS3075 | hyl-ribo-hexose-a | 4.594 | 177.11166 | [M+NH4]2+ | 177.11211 | 88.02121:34491 134.02684:9513 173.43524:6596 | -2.5408E-06 |
| POS10583 | Tenacibactin C | 4.524 | 520.33051 | [M+NH4]+ | 520.33002 | 73.06432:28839 87.04334:132120 89.05896:1572119 90.0619:33301 91.07443:10618 104.10696:19802 107.06821:11937 117.08817:18443 131.06766:32650 133.08543:696134 134.08684:18707 144.3028:8212 173.3813:8593 175.09526:11748 177.11285:97387 197.86429:8397 | 9.4171E-07 |
| POS14755 | PC(32:0) | 8.805 | 756.552 | [M+H]+ | 756.55133 | 60.08022:31450 67.54832:12374 73.45212:11172 86.09537:77100 94.06747:11228 101.42462:11288 101.90234:10882 104.10693:10989 111.52592:11379 124.9995:15745 184.06921:205498 222.45992:12398 253.30632:11679 542.04785:14291 | 8.85598E-07 |

**Differences in metabolites between the WJW group, the Model group and the Control group**

| Alignment ID | Metabolite name | Rt(min) | Expreiment Mz | Adduct type | Reference m/z | MS/MS spectrum | PPM |
|---|---|---|---|---|---|---|---|
| POS9624 | Loperamide | 4.491 | 477.23071 | [M+H]+ | 477.23029 | 72.08047:7460 73.06432:10341 86.05939:12046 86.09541:6800 87.04334:24231 89.05896:357135 90.0619:24425 98.06007:6841 112.07434:29439 119.08612:16395 120.0803:10075 133.08543:163641 134.08684:13166 136.52037:6027 157.00053:5902 177.11285:22950 291.63074:5845 | 8.80078E-07 |
| POS12102 | brujavanone D | 4.69 | 575.35828 | [M+CH3OH+H]+ | 575.35779 | 59.04859:11804 64.65227:7554 70.12607:6071 73.02812:13099 79.72459:6653 87.04335:34803 89.05896:179418 94.8423:6089 100.51604:5904 103.07444:6306 111.04441:6606 133.08545:69356 177.11285:11363 189.74847:6204 243.21132:6574 | 8.51644E-07 |
| POS12099 | raspiridole B_1300 | 4.457 | 575.31354 | [2M+H]+ | 575.31403 | 88.99577:8921 89.05891:64503 121.77953:7621 133.08537:31569 188.06995:14284 | -8.5171E-07 |
| POS5478 | 79966-13-5 | 0.759 | 261.00415 | [M+H]+ | 261.00372 | 55.08349:5608 61.03869:70612 62.50561:5753 162.99733:5917 | 1.64749E-06 |
| POS13415 | Cyclosquamosin A | 4.367 | 642.32507 | [M+H]+ | 642.32452 | 78.48708:5382 110.16753:6001 118.88821:6000 120.0803:11004 129.1017:14690 284.55325:5294 340.97604:5526 | 8.56265E-07 |
| POS1661 | minopropoxy)guan | 2.2 | 133.10878 | [M+H]+ | 133.10831 | 55.01769:9547 55.05364:22978 56.04951:32865 57.05691:117812 58.0651:67808 59.04856:11327 67.05387:14182 69.06989:1012020 70.0731:111514 73.0281:9696 73.0643:16475 86.09538:3664538 87.09916:413410 | 3.53096E-06 |
| POS8648 | ptan-2-yl)-10,13-d | 5.825 | 419.35056 | [M+NH4]+ | 419.35101 | 51.32425:6511 53.51543:5580 57.03286:8346 59.04858:18139 59.19405:6231 61.50322:5552 69.06991:15310 81.06832:74198 93.07033:7041 95.08476:13841 99.07938:92604 107.08485:5748 109.10005:24396 118.48554:6358 121.10129:10560 127.11192:42138 128.49895:5709 135.11644:16540 147.11679:11189 149.13:8978 161.13196:9149 177.72186:5742 | -1.0731E-06 |
| POS4255 | Oxododecanoic ac | 6.138 | 215.16316 | [M+H]+ | 215.1636 | 51.47779:6184 55.45854:6857 | -2.045E-06 |
| POS1648 | ethylthiopentan-3 | 4.817 | 133.06772 | [M+Na]+ | 133.06816 | 67.803:5709 69.06989:39772 82.45757:6350 86.09537:158292 87.09914:6806 104.04764:25055 105.03241:6749 133.05241:12601 | -3.3066E-06 |
| NEG1256 | -Butanesulfonic ac | 8.411 | 137.02744 | [M-H]- | 137.02789 | 93.14729:7280 114.20587:5520 114.93765:5388 | -3.284E-06 |
| POS6418 | oimidazole ribonu | 0.949 | 296.0636 | [M+H]+ | 296.06409 | 60.08025:23971 71.07261:19411 86.09541:204355 104.10697:776778 105.10879:24854 112.89422:14757 120.00122:13938 169.9852:20399 236.99554:7672 | -1.655E-06 |
| POS11866 | Leu Tyr Lys Glu | 6.195 | 566.31787 | [M+Na]+ | 566.31842 | 51.65913:10940 58.06512:15513 60.08025:31763 67.05272:14886 71.07261:14371 79.05313:11711 86.09541:230832 93.06841:17973 104.10697:1422411 105.10879:41028 119.08334:13797 146.97896:190666 180.48094:11372 354.95444:10709 | -9.7119E-07 |
| POS7905 | erone 3-sulfate so | 5.468 | 369.17148 | [M+H-H2O]+ | 369.17099 | 53.51121:5726 57.06894:147905 59.04856:9290 62.43773:6368 67.10578:5489 83.04766:6463 85.06333:84177 89.05892:7449 107.04913:6400 121.02687:5973 127.07495:12709 133.0656:7208 137.05975:15837 147.04396:10677 167.10457:23500 173.43027:10163 179.7023:6258 221.1192:18449 286.83472:5713 | 1.3273E-06 |
| POS5696 | ,12-Hexahydroaze | 4.825 | 269.06018 | [M+H]+ | 269.06067 | 53.90258:5675 183.7211:6412 | -1.8212E-06 |

**Differences in metabolites between the WJW group, the Model group and the Control group**

| Alignment ID | Metabolite name | Rt(min) | Expreiment Mz | Adduct type | Reference m/z | MS/MS spectrum | PPM |
|---|---|---|---|---|---|---|---|
| POS12499 | Podototarin | 6.134 | 593.43353 | [M+CH3OH+H]+ | 593.43292 | 50.67118:5669 54.03799:5682 57.03287:294743 59.04858:94944 67.95436:5881 81.06833:6117 99.07938:36638 101.09641:8090 107.9203:5749 115.07378:26630 117.08817:17043 137.96021:5530 157.12312:42351 173.43034:10610 | 1.02792E-06 |
| POS6069 | Helipandurin | 1.193 | 283.0184 | [M+Na]+ | 283.01889 | 55.05365:23136 55.37881:6720 57.06896:22459 61.0387:36270 67.05389:8412 69.06868:26057 71.08547:8629 81.0699:10292 83.08509:15330 93.06841:6457 95.08477:9999 97.09953:15408 109.10006:8774 110.8261:5504 111.11732:6136 121.1013:6617 162.05843:6212 201.10139:6503 276.58087:4927 | -1.7313E-06 |
| POS7357 | )-methylneoagaro | 4.731 | 339.12799 | [M+H]+ | 339.12851 | 55.01768:8163 57.06894:54867 59.04856:50167 65.94633:5708 83.01192:150502 85.06333:49011 87.04331:15571 89.05892:8066 101.02218:7596 103.03844:10054 103.07439:7378 111.07955:9845 115.03662:63315 139.07352:34259 141.03842:7937 152.00653:11651 157.08499:16901 158.7514:5029 169.03244:82317 171.09958:30901 217.64404:6682 | -1.5333E-06 |
| POS4658 | aureothricin | 1.204 | 231.02509 | [M+H]+ | 231.0256 | 51.60494:4860 52.34609:5040 52.40226:4955 53.57602:5516 55.51713:4676 59.27923:3988 59.67426:4210 61.0561:4726 61.12378:4139 61.50322:4601 64.35188:4912 70.01521:4499 71.72692:4475 71.8471:4437 79.13621:4928 82.53001:4615 86.48445:4756 90.88618:4138 91.6992:4865 100.39278:4164 101.92226:4489 102.37012:4400 103.79739:5268 105.51961:3989 106.1909:4007 106.89709:4513 119.68001:3814 125.68365:4764 141.86584:4666 158.9062:4620 159.09998:4177 159.43236:4009 161.65155:5065 162.84996:4407 168.66025:4075 180.60074:5156 215.51527:4641 | -2.2075E-06 |
| POS6750 | ripentyl phosphat | 6.215 | 309.21936 | [M+H]+ | 309.21887 | 55.05365:6885 59.04858:8074 67.05389:7179 70.24855:5875 72.41546:5413 81.06989:6388 97.10158:7561 138.85191:5487 173.396:6944 256.55826:5440 | 1.58464E-06 |
| NEG398 | Styrene | 8.114 | 103.05589 | [M-H]- | 103.0554 | 50.26:5653 57.10277:13123 58.51401:6316 59.08403:19211 60.29253:5787 61.07175:5855 64.04998:6164 73.11619:8279 88.9059:5414 103.16489:8181 | 4.75472E-06 |
| NEG1226 | ylpyridin-3-yl)bor | 7.106 | 136.05812 | [M-H]- | 136.0576 | 56.7561:5952 65.09239:8743 89.13125:21010 135.05537:9016 136.05812:8624 137.05487:11191 | 3.82191E-06 |
| POS7115 | (-)-Spectaline | 6.981 | 326.30392 | [M+H]+ | 326.30441 | 62.06014:7482 83.74775:5349 111.98013:5167 126.20273:5299 156.22183:5575 170.93153:6539 | -1.5017E-06 |
| POS12620 | ıctive peptide-1\|S( | 4.7 | 599.3504 | [M+H]+ | 599.35107 | 69.03294:38710 73.02811:181858 73.06432:104448 87.04334:467806 89.05896:2369354 90.0619:123719 91.04644:54510 95.04893:88861 99.04339:48918 102.06606:71614 111.04189:82690 113.05927:108419 117.09089:38395 131.06766:90357 133.08543:1302579 134.09018:66776 137.05981:60868 146.55127:41191 155.07083:76725 159.97418:38949 177.10779:196456 | -1.1179E-06 |

**Differences in metabolites between the WJW group, the Model group and the Control group**

| Alignment ID | Metabolite name | Rt(min) | Expreiment Mz | Adduct type | Reference m/z | MS/MS spectrum | PPM |
|---|---|---|---|---|---|---|---|
| POS6633 | 4,5-dimethyl-2-ox | 4.384 | 304.17487 | [M+CH3OH+H]+ | 304.17542 | 51.44688:6234 57.03287:16040 58.06511:8521 60.08025:219606 69.06868:93746 70.02779:12022 70.0643:6306 73.06432:25998 81.0699:7586 84.07957:23066 85.02799:547409 86.03196:12596 86.09541:67413 97.06461:74051 103.03849:8438 115.07378:62989 125.0596:302680 126.06278:8929 132.10173:10240 132.18655:6242 143.06735:108340 144.10197:24769 161.07927:24418 227.09167:8346 | -1.8082E-06 |
| POS2500 | Allantoin | 0.763 | 159.04947 | [M+H]+ | 159.05 | 52.15548:6852 55.93345:47177 56.94233:12875 68.98124:35509 69.98251:6275 72.93707:13179 73.06432:23608 86.99105:20833 105.00241:13277 108.27512:5459 113.96312:6903 131.01933:8361 | -3.3323E-06 |
| POS11544 | Juvenimicin A2 | 8.739 | 554.36932 | [M+2H]2+ | 554.36871 | 74.18494:6379 124.93654:5543 173.3911:12067 290.60663:5553 | 1.10035E-06 |
| POS678 | Homopiperazine | 8.743 | 101.10677 | [M+Na]+ | 101.10732 | 70.53394:6167 | -5.4398E-06 |
| POS3019 | densispicnin D | 5.978 | 175.13226 | [M+H]+ | 175.1328 | 54.46105:5937 59.02229:5374 59.04859:8480 69.72547:5990 133.93025:5405 146.03825:5677 157.05971:6021 165.42868:5228 173.43034:8800 | -3.0834E-06 |
| POS2883 | ramethyl-4-piperic | 1.175 | 171.14856 | [M+CH3OH+H]+ | 171.14909 | 55.05365:32473 58.06512:13894 67.05389:27210 69.06992:6107 72.04369:42975 83.08509:7684 89.06979:9185 109.10006:7372 118.74913:5687 | -3.0967E-06 |
| POS12377 | nagitide A | 4.535 | 587.32336 | [M+NH4]+ | 587.32269 | 59.04858:33429 69.03294:308951 73.02811:92996 73.06432:57698 81.03229:36420 83.0477:130652 85.02799:23950 87.04334:596637 89.05896:1689632 90.0619:30548 95.04893:60869 99.04339:492689 107.06821:29161 111.04189:250668 113.05927:37193 117.09089:32262 124.24364:22068 125.0596:122443 129.05446:44234 131.07088:65241 133.08543:647032 137.05981:42742 151.09705:22558 155.07083:154022 173.07771:39173 175.09526:23181 177.11285:43105 433.12009:19764 531.20953:18946 | 1.14077E-06 |
| POS10145 | 10,13-dimethylhex | 4.565 | 501.2886 | [M+2H]2+ | 501.28799 | 67.05389:10180 69.03294:24014 73.02811:78849 73.06432:11997 81.03229:36478 83.0477:31797 87.04334:139810 89.05896:695122 90.0619:36670 91.04643:15830 95.04893:52681 99.04339:36235 111.04441:88439 113.05927:24005 129.05132:9701 133.08543:208249 134.09018:9543 137.05981:22338 155.07083:22753 177.10779:13785 349.62927:8779 394.30075:8815 | 1.21687E-06 |
| NEG6949 |  | 0.963 | 337.19568 | [M-H2O-H]- | 337.19629 | 55.66968:5877 93.03925:612058 95.03763:385042 103.08171:6340 151.06711:31030 153.06754:44012 | -1.809E-06 |
| POS8025 | enzylidene,2-mesy | 4.811 | 375.11008 | [M+2H]2+ | 375.11069 | 50.22026:7631 57.06894:14736 81.03226:6824 85.06503:6672 109.48528:6124 122.25717:6253 125.05956:8737 144.07964:10290 149.09477:5550 166.09781:6158 167.10458:58280 168.11118:9279 173.4303:8431 221.47981:6724 259.06525:6603 260.06213:9506 | -1.6262E-06 |
| POS13576 | Gnididilatin | 4.539 | 653.33099 | [M+Na]+ | 653.33191 | 73.06432:16337 87.04335:81017 88.42162:21583 89.05896:508404 90.06375:26002 131.07088:22260 133.08545:261973 134.09018:19703 167.93817:14192 173.32739:16029 177.11285:33825 207.07423:17439 | -1.4082E-06 |
| POS14943 | xy-3-oxochol-4-e | 6.74 | 777.52911 | [M+H]+ | 777.53003 | 62.33298:5719 77.24027:5977 173.43034:14701 | -1.1832E-06 |
| POS8674 | SCHEMBL9230589 | 5.663 | 421.31528 | [M+H-H2O]+ | 421.31589 | 52.03271:6102 54.90479:5597 57.06893:21297 64.94799:5750 150.03362:6041 173.42535:9497 335.32715:5905 | -1.4478E-06 |

**Differences in metabolites between the WJW group, the Model group and the Control group**

| Alignment ID | Metabolite name | Rt(min) | Expreiment Mz | Adduct type | Reference m/z | MS/MS spectrum | PPM |
|---|---|---|---|---|---|---|---|
| NEG6305 | ACMC-20m5sy | 0.912 | 313.25443 | [M-H2O-H]- | 313.25381 | 93.03925:95947 94.70243:6657 95.03764:22058 97.03746:11298 103.07947:53240 124.15677:15567 131.55391:5587 170.74947:6080 177.1145:22416 | 1.97923E-06 |
| POS4414 | eta-glucosylbutan | 4.6 | 221.1377 | [M+2H]2+ | 221.13831 | 55.05365:18955 58.04042:7238 61.01003:10168 73.02811:12944 81.0699:6347 89.05896:7078 97.09953:9690 125.09565:8844 173.39111:11760 175.25459:6089 193.96582:5796 | -2.7585E-06 |
| POS7123 | riphenyl Phosphat | 6.096 | 327.07718 | [M+H]+ | 327.07779 | 57.78203:5585 95.04893:13692 115.42985:5442 121.75073:5463 131.258:5125 153.06662:12314 | -1.865E-06 |
| POS14067 | PS(P-16:0/14:1(9Z) | 8.704 | 690.46942 | [M+2H]2+ | 690.46863 | 62.46531:6290 118.79361:5989 173.3911:12671 | 1.14415E-06 |
| POS6316 | Epiandrosterone | 6.166 | 291.21527 | [M+H]+ | 291.21469 | 57.03287:30544 57.06989:8249 59.04858:14080 85.48107:5604 89.87497:5691 | 1.99166E-06 |
| POS10231 | Artoindonesianin L | 4.39 | 505.2211 | [M+H]+ | 505.22171 | 60.04426:13514 70.06556:5731 72.08047:15701 72.84618:5567 84.08123:5355 94.80859:6167 118.08494:8652 129.10172:8733 129.49957:5232 175.10521:10225 329.03448:6374 | -1.2074E-06 |
| POS6314 | Epiandrosterone | 5.973 | 291.21527 | [M+2H]2+ | 291.21469 | 57.03287:13229 57.06989:11761 59.04858:15163 94.53361:5705 99.57687:5565 104.10696:6784 208.52167:5827 | 1.99166E-06 |
| POS15338 | bicornutin A2 | 8.719 | 859.58398 | [M+2H]2+ | 859.5849 | 62.51623:5267 95.05093:4735 100.53985:5514 196.43747:5891 260.47702:5822 354.30902:5549 376.05884:5837 | -1.0703E-06 |
| POS8349 | ngoramine\|15-Ace | 6.689 | 398.23312 | [M+H]+ | 398.23251 | 50.02518:5903 53.62407:6384 65.30698:4998 67.58175:6532 102.40796:5349 | 1.53177E-06 |
| POS8523 | Risperidone | 4.471 | 411.22064 | [M+H]+ | 411.22 | 55.05365:17699 59.04858:11397 67.05389:5960 69.03294:127237 73.02811:24347 81.03229:11864 83.0477:60021 87.04334:171809 89.05896:220269 95.04893:19416 99.04339:81065 107.07059:11021 111.04189:87854 113.05927:8752 125.0596:10837 129.05446:12660 131.06766:18400 133.08543:57860 137.05981:5980 151.09705:10247 155.07083:21651 175.09526:6611 293.59796:5616 316.06183:5958 | 1.55634E-06 |
| POS14888 | HMS2873N16 | 4.419 | 770.33722 | [M+2H]2+ | 770.3382 | 72.08047:177076 86.09541:69139 118.08495:95567 129.09856:75262 305.85339:67544 | -1.2722E-06 |
| POS10614 | 16-Trihydroxygray | 4.36 | 521.2713 | [M+2H]2+ | 521.27197 | 60.04325:56165 84.04481:14779 84.07957:24844 86.0954:20948 86.4154:9828 102.05276:23592 120.0803:36031 126.05366:11459 129.1017:54961 154.04741:11480 189.0878:20782 217.07977:11623 269.75531:9991 | -1.2853E-06 |
| POS14376 | Mycolactone E | 6.036 | 715.5152 | [2M+H]+ | 715.51428 | 54.48004:11175 57.03287:49740 59.04858:412194 115.07642:12631 117.09089:66733 173.3911:19235 191.97964:12295 | 1.28579E-06 |
| POS11920 | Cholylhistidine | 6.474 | 568.33496 | [M+H]+ | 568.33569 | 53.90853:6958 104.10692:24584 130.73308:5764 208.30186:6079 316.26694:5760 355.9762:6039 | -1.2845E-06 |
| POS13730 | kanoyl-NBD Chole | 8.766 | 663.44861 | [M+NH4]+ | 663.44788 | 52.42753:6679 174.16846:5838 276.60065:6166 | 1.10031E-06 |
| POS14754 | PC(32:0) | 5.984 | 756.55035 | [2M+H]+ | 756.55133 | 60.08022:37579 86.09537:90868 124.9995:29152 184.07457:229178 278.94693:9358 | -1.2954E-06 |
| POS5519 | ydroxyvalerylcarni | 3.958 | 262.16397 | [M+H]+ | 262.16461 | 53.30585:6434 55.40891:6406 59.04858:11721 60.08024:13834 61.03869:45354 76.82643:5531 85.02798:102204 110.94395:5711 111.10222:5649 132.5201:5358 196.49069:5692 228.23517:5695 | -2.4412E-06 |
| NEG6724 | Hydroxyprogester | 0.904 | 329.25793 | [M-H]- | 329.25729 | 81.04115:6856 93.03925:49700 95.03764:24921 103.08172:13697 119.0826:16267 126.0732:6496 177.1145:11728 314.51971:6203 | 1.94377E-06 |

**Differences in metabolites between the WJW group, the Model group and the Control group**

| Alignment ID | Metabolite name | Rt(min) | Expreiment Mz | Adduct type | Reference m/z | MS/MS spectrum | PPM |
|---|---|---|---|---|---|---|---|
| POS939 | alpha-monochlor | 8.801 | 111.02007 | [M+CH3OH+H]2+ | 111.0207 | 54.03373:13309 55.04137:24637 55.93435:9953 56.96447:10873 57.9342:423490 67.49115:13985 69.99382:12383 71.05973:21944 72.93706:74402 74.93712:72777 76.49632:41026 77.99808:33349 78.9988:108420 79.49879:9418 84.94554:64956 85.50143:15059 87.00323:120458 88.00347:1912326 88.50381:137503 89.00122:26993 89.50647:13046 90.50591:56403 90.94576:21419 92.94704:11518 95.01113:30420 97.00507:278656 97.50974:30324 97.95379:29597 98.51207:25622 98.96088:91524 99.51075:403118 100.0115:33286 102.95542:46552 106.01485:14048 108.51748:32990 108.95824:12706 110.95901:9218 111.01927:40352 113.96311:112265 | -5.6746E-06 |
| POS7459 | nethylpent-4-enyl | 1.21 | 343.33655 | [M+Na]+ | 343.33591 | 55.05453:10758 57.06896:43638 59.03105:230090 61.01003:106223 62.98918:6912 67.05389:14750 69.06869:29415 70.83771:5507 71.08548:18494 73.04555:155497 75.02498:212522 77.00464:11970 77.04093:139452 79.01993:21569 83.0851:22905 85.10046:10091 87.06079:15150 91.05577:139447 93.03564:124450 95.01512:10207 97.09953:20636 105.07177:51413 111.11733:11258 119.08613:18341 120.30954:5504 125.13172:7294 133.10193:9567 252.17599:5722 343.06805:5855 | 1.86406E-06 |
| POS9109 | yr-pro-leu-gly-NH | 4.624 | 448.25616 | [M+2H]2+ | 448.25549 | 55.05365:43824 59.04858:50330 69.03294:750078 70.0366:21563 71.04816:16042 73.02811:185668 73.06432:84127 78.03804:19506 80.05429:70324 81.03229:24991 83.0477:139718 87.04334:621042 88.04782:23611 89.05896:2186922 90.0619:75419 91.04643:18226 95.04893:47437 99.04339:351123 100.05018:82056 102.06606:48598 107.07058:15750 109.06336:20714 111.04441:180290 113.05927:45693 117.09089:32455 122.06293:132622 122.56553:19196 125.0596:31456 129.05446:73320 131.07088:64470 133.08543:657783 134.08684:34734 137.0598:30580 144.07597:43365 155.07083:73250 166.08865:26780 173.42543:19873 177.11285:46147 | 1.49468E-06 |
| POS15254 | 15Z)/22:6(4Z,7Z,10 | 8.757 | 828.55261 | [M+H]+ | 828.55371 | 64.80882:5376 90.13166:5540 90.14452:6178 | -1.3276E-06 |
| POS2033 | Ethephon | 8.766 | 144.98216 | [M+H]+ | 144.98151 | 62.92802:382364 103.95428:12783 121.96451:111242 144.97899:15551 | 4.48333E-06 |
| POS5240 | roughanic acid | 5.861 | 251.19934 | [M+H]+ | 251.2 | 55.05366:8995 57.06896:10614 59.04859:13384 67.05389:49762 81.06834:33459 93.06841:13426 95.08478:16153 96.71436:5123 107.08487:8283 119.01638:5881 200.50862:5817 | -2.6274E-06 |
| POS7143 | earoylethanolamic | 7.516 | 328.31934 | [M+H]+ | 328.32001 | 74.28384:5185 83.54238:5423 168.10655:5885 292.4346:6312 | -2.0407E-06 |
| POS3651 | Cyclohexylalanine, | 0.996 | 194.11444 | [M+NH4]+ | 194.11513 | 52.16681:6394 58.21173:6940 65.0448:6380 105.28721:6138 135.04227:12472 | -3.5546E-06 |
| POS5715 | Vitamin A | 7.047 | 269.22433 | [M+H]+ | 269.22501 | 67.57101:5925 97.04408:5224 120.90405:6182 173.43034:10681 | -2.5258E-06 |
| POS5489 | Leucyl-Glutamate | 2.399 | 261.14291 | [M+H]+ | 261.14359 | 58.06512:6880 61.0387:112801 90.77464:6157 148.89172:5542 167.25319:5439 215.13528:10629 | -2.6039E-06 |

**Differences in metabolites between the WJW group, the Model group and the Control group**

| Alignment ID | Metabolite name | Rt(min) | Expreiment Mz | Adduct type | Reference m/z | MS/MS spectrum | PPM |
|---|---|---|---|---|---|---|---|
| POS10817 | Pro Lys Lys Val Gly | 5.236 | 528.34955 | [M+2H]2+ | 528.35028 | 57.03378:52435 59.04858:942759 60.05225:24259 73.02811:90964 73.06431:42004 80.05428:17471 85.06504:26185 87.04333:285945 87.06252:45317 87.07997:25489 88.04604:21069 89.05894:1175868 90.0619:30232 94.06945:36619 101.05929:186395 102.06604:24382 103.07442:277642 107.07057:16114 109.07558:18349 129.0891:30027 133.08542:384601 134.08682:17608 147.10147:95883 161.11877:16088 171.94873:14308 177.11282:23848 191.12556:20051 219.33647:14791 | -1.3817E-06 |
| POS15031 | PC(16:0e/12-HETE | 8.806 | 784.58392 | [M+Na]+ | 784.58502 | 53.95275:6330 55.18986:7167 57.91145:6853 104.49812:5708 134.72931:5113 151.41658:5085 164.64087:5531 | -1.402E-06 |
| POS10169 | Ala Leu Leu Ala Se | 5.369 | 502.32269 | [M+2H]2+ | 502.32339 | 57.03287:20505 59.04859:225785 60.05226:8017 67.015:26429 73.02812:7445 73.06432:10946 78.27695:5333 81.03072:15251 85.06506:7810 87.04335:27628 87.07999:12120 89.05896:175188 90.06374:7204 91.40009:5270 101.05931:14949 103.07444:66788 117.05281:5730 117.0909:6618 131.06766:5493 133.08545:48132 147.10149:28884 158.09198:14257 165.0956:8004 177.11285:8119 180.10141:7485 | -1.3935E-06 |
| NEG4606 | Tripelennamine | 2.717 | 254.16559 | [M-H]- | 254.16631 | 106.66814:6352 179.69743:5091 182.80327:5982 | -2.8328E-06 |
| NEG9857 | lihydro-3-[2-[[(2E) | 6.196 | 633.1239 | [M-H]- | 633.12299 | 89.1313:491831 90.13493:24984 171.80325:5295 | 1.43732E-06 |
| POS5913 | 13-OxoODE | 6.323 | 277.21069 | [M+H]+ | 277.20999 | 51.60017:5818 55.05365:14852 57.06895:8654 67.05389:11565 68.40289:5850 69.06991:7502 81.06989:8632 91.05389:7189 91.21084:5115 93.0684:13701 120.82984:5609 259.21762:5271 | 2.52516E-06 |
| POS8800 | Val Val Val Gly Gly | 4.948 | 430.26517 | [M+CH3OH+H]2+ | 430.2659 | 59.04858:42603 67.015:7871 82.13532:5643 87.04334:12522 87.1743:6597 89.05896:85351 103.07444:14768 122.2514:5931 133.08545:26483 221.2818:6249 | -1.6966E-06 |
| POS2234 | enzisothiazol-3(2H | 0.764 | 151.00845 | [M+Na]+ | 151.00919 | 56.74443:6504 67.05389:7281 68.98123:155552 82.01235:59734 86.99277:138376 100.02438:116854 105.0024:137716 110.00877:12313 118.03259:28004 123.0126:14057 128.01854:49666 | -4.9004E-06 |
| POS5109 | nyl)methyl]-N-ethy | 8.898 | 247.09886 | [2M+H]+ | 247.09959 | 57.06986:10568 69.06989:13126 80.83523:6072 111.04185:26675 125.05955:20055 167.10457:27673 168.4581:6972 229.63365:6215 239.8895:5286 | -2.9543E-06 |
| POS3883 | 3-benzodioxole-4 | 1.008 | 203.01422 | [M+CH3OH+H]+ | 203.015 | 57.03287:28735 60.08025:167617 62.98059:44775 70.0643:127962 71.04816:18884 71.05974:54954 82.94377:27979 84.07957:14710 85.02799:894229 87.97689:213884 88.0851:27716 100.07382:27565 105.0024:76477 116.06905:23099 117.00114:14605 135.00183:10803 142.98286:19252 144.10197:18137 145.04651:16065 158.12614:11853 | -3.8421E-06 |
| POS8135 | -methoxyphenyl}- | 1.034 | 383.11487 | [M+H]+ | 383.11569 | 53.2382:5895 53.43983:6521 82.94374:5768 203.05078:79829 238.1199:5854 | -2.1403E-06 |
| POS8839 | Istamycin C1 | 4.491 | 432.27838 | [M+NH4]+ | 432.27921 | 73.06432:19208 87.04334:58890 89.05896:1171961 90.0619:26224 107.06821:28428 117.08817:8395 130.08389:15416 131.07088:13409 133.08543:480616 134.09018:13108 177.11285:54497 | -1.9201E-06 |
| POS11841 | Aristophyll B | 4.506 | 565.2818 | [M+H]+ | 565.28088 | 66.95612:21347 67.26903:21225 87.04334:58740 89.05896:578913 90.0619:37662 98.06215:17836 131.07088:17316 133.08543:227625 140.56842:15987 166.01514:16742 167.74202:14961 173.39111:26395 177.11285:21075 | 1.62751E-06 |

**Differences in metabolites between the WJW group, the Model group and the Control group**

| Alignment ID | Metabolite name | Rt(min) | Expreiment Mz | Adduct type | Reference m/z | MS/MS spectrum | PPM |
|---|---|---|---|---|---|---|---|
| POS7995 | GS-2 | 5.299 | 373.17462 | [M+H-H2O]+ | 373.17542 | 55.01768:9674 57.06894:18595 59.04856:85272 71.1009:6022 73.0281:6516 73.14081:6523 83.04929:11086 85.06334:8841 89.05893:28226 95.08474:23769 101.05709:5455 103.07441:10789 122.09476:11174 130.06473:24991 131.57179:5379 132.04303:6133 146.06094:21617 150.0889:7699 158.05779:26437 160.07413:15754 168.10181:6410 168.88625:6034 175.08525:11309 182.11658:5953 184.0746:22478 186.05348:5870 199.00485:5375 | -2.1438E-06 |
| POS6605 | -hydroxy-6-methy | 0.755 | 303.02576 | [M+H]+ | 303.02655 | 53.82195:5647 61.97727:6437 128.94746:5700 132.68735:5070 173.69058:6055 218.98808:8075 236.06581:6082 | -2.607E-06 |
| POS4180 | thyl-1H-benzimid | 0.758 | 213.02388 | [M+Na]+ | 213.0247 | 86.99277:10620 94.43892:6952 105.0024:12319 180.54861:5459 185.02766:11004 | -3.8493E-06 |
| POS937 | alpha-monochlor | 6.48 | 111.01991 | [M+2H]2+ | 111.0207 | 55.0405:17427 55.93345:11104 57.9342:173904 67.49116:9696 72.93707:81535 74.93713:30982 75.49612:11274 76.4949:17358 77.99808:37259 78.99881:42211 84.94555:24951 85.49975:11244 87.00324:180801 87.50562:13106 88.00348:875251 88.50382:46455 89.00123:17917 89.50648:13792 90.50592:20519 90.94577:26361 96.00943:12156 97.00918:304637 97.50975:12290 98.51208:34880 98.96088:31845 99.51076:172240 100.01151:17327 102.95543:15439 106.01486:16401 108.51749:25855 108.95825:19281 111.01928:19849 113.96312:132436 | -7.1158E-06 |
| POS7454 | IC ACID DER (FR. L | 5.815 | 343.28339 | [M+H]+ | 343.28421 | 57.06893:9343 60.08022:5723 67.24886:5568 74.11497:5719 86.09536:13025 163.8858:5687 170.79715:6674 184.07457:13501 188.54721:5212 200.60612:6777 216.05299:5938 | -2.3887E-06 |
| POS938 | alpha-monochlor | 5.925 | 111.01991 | [2M+H]+ | 111.0207 | 55.04137:9377 55.93435:9040 57.9342:170588 72.93707:68652 74.93713:31886 76.49633:11064 77.99808:37110 78.99881:31105 84.94386:26990 85.50144:9992 87.00324:147205 87.50562:7534 88.00348:752935 88.50382:34626 89.00123:11667 89.50648:7362 90.94763:19535 92.94897:8194 95.01114:7544 97.00918:268649 97.50975:14659 98.50998:23719 98.96088:19921 99.51076:148819 100.01365:13214 102.95543:19751 106.01486:9065 108.51749:20082 108.95825:15304 111.01928:11217 113.96312:112127 | -7.1158E-06 |
| POS935 | alpha-monochlor | 5.758 | 111.0199 | [M+H]+ | 111.0207 | 55.04137:14360 55.93435:18317 57.9342:188736 68.04829:7364 72.93706:89086 74.93713:27482 75.49611:10279 76.49632:25918 77.99808:43985 78.99881:41352 84.94554:25301 85.49974:8835 87.00324:184402 87.50385:18900 88.00348:916425 88.50381:49343 89.00303:12289 89.50647:9252 90.50591:13615 90.94762:27374 92.94704:7548 95.01113:10101 96.00741:8333 97.00917:302326 97.50974:6819 97.95171:9592 98.51208:24318 98.96088:40993 99.51075:168294 100.0115:10693 102.95542:20264 106.01252:9103 108.51748:16989 108.95825:17323 111.01928:11515 113.96311:119832 | -7.2059E-06 |

**Differences in metabolites between the WJW group, the Model group and the Control group**

| Alignment ID | Metabolite name | Rt(min) | Expreiment Mz | Adduct type | Reference m/z | MS/MS spectrum | PPM |
|---|---|---|---|---|---|---|---|
| POS10447 | Hennoxazole A | 8.804 | 515.31238 | [M+NH4]2+ | 515.31152 | 57.06894:18004 60.08023:21856 75.85378:9829 84.26364:10607 86.09538:66938 104.10693:599104 105.10875:29289 112.04374:14156 173.38615:21450 183.12358:10484 312.70972:11045 | 1.66889E-06 |
| POS10242 | Eudesobovatol A | 5.401 | 505.33035 | [M+Na]+ | 505.33121 | 59.04858:22005 60.51869:6391 67.54954:5982 87.04334:9228 89.05896:22581 133.08543:7357 370.6825:5931 | -1.7019E-06 |
| POS6837 | nethyllumazine he | 0.878 | 313.03406 | [M+CH3OH+H]+ | 313.0332 | 51.59301:5858 57.06989:7570 64.0778:5520 86.3585:5997 95.34216:5905 145.91704:6573 177.06224:94869 | 2.74731E-06 |
| NEG3630 | H-Met-Ala-OH | 1.325 | 219.08076 | [M-H]- | 219.0816 | 58.04516:8286 97.07652:18103 99.07362:24309 118.92107:6107 161.03635:88578 | -3.8342E-06 |
| POS4740 | SCHEMBL4748090 | 6.31 | 233.17375 | [M+H]+ | 233.17461 | 50.02441:5649 51.9667:4592 55.5687:5839 57.06989:4761 58.42694:4201 63.52836:5329 65.09892:4186 69.69672:3886 70.41069:4354 71.44855:4437 73.02811:6086 73.10591:3917 73.56042:4965 75.60188:5517 79.3559:7422 82.78501:4928 83.87628:4915 89.71416:4409 92.87972:5020 104.80626:4991 111.92415:5047 123.71887:4982 125.3816:5402 130.29124:4211 136.30479:4052 140.81929:4339 144.29163:4038 147.57776:4511 151.09703:3968 165.68486:3732 179.49561:5680 195.94174:4385 196.09497:4181 210.6328:4450 216.47656:4519 218.19684:4031 230.56325:4532 | -3.6882E-06 |
| POS3864 | Pantothenic acid | 4.394 | 202.10612 | [M+H-H2O]+ | 202.10699 | 55.05365:27903 66.91496:6640 67.05389:5866 69.06868:5980 70.02779:6834 70.0643:12287 73.02811:6237 86.09541:9462 88.03895:19339 98.02254:6027 101.0593:7952 104.60148:5565 130.06477:6753 178.33917:5429 | -4.3047E-06 |
| POS8085 | DS-007819 | 4.879 | 379.23132 | [M+2H]2+ | 379.22781 | 59.04859:30062 67.015:11734 67.38055:5666 87.04335:11350 89.05896:48424 101.05931:13232 133.08545:14619 137.01158:5262 139.32047:5241 160.3878:5683 | 9.25565E-06 |
| NEG1917 | -2403;(E)-Metanico | 0.965 | 161.10941 | [2M-H]- | 161.10851 | 55.08503:9289 57.10279:31440 59.08405:50394 71.099:60111 73.11621:35802 74.11263:10004 85.1313:50907 87.11256:8759 93.03928:677611 95.12722:11837 99.16254:9323 101.14605:5754 103.0795:17885 104.15944:8033 113.15993:18230 | 5.5863E-06 |
| POS14377 | anosyl)-2,6-dihydr | 6.592 | 715.53668 | [M+NH4]+ | 715.5354 | 57.03287:232785 57.96547:6274 58.03662:7976 59.04858:58816 99.07938:17533 115.07378:17169 117.09089:8136 157.12312:17195 363.88025:5820 431.01767:5684 715.4892:6396 | 1.78887E-06 |
| NEG3318 | yl-1H-pyrazol-4-y | 1.228 | 208.0872 | [M-H]- | 208.0881 | 59.08407:39933 71.09774:8072 73.11623:69572 74.12088:6859 77.71171:5539 79.05373:15231 85.13134:6082 89.13131:33557 89.14215:7143 97.07652:565952 101.14608:10721 119.08548:7638 120.83963:5409 126.02468:11126 128.02199:17080 146.40347:5377 161.2383:8561 | -4.3251E-06 |
| POS3342 | phohydroxypyruvi | 8.789 | 184.9854 | [M+NH4]+ | 184.9845 | 55.8742:7477 57.9342:157519 58.9425:411639 59.94047:8544 75.94469:93117 76.95241:32865 84.94554:57664 86.0954:11358 93.95591:18053 98.96088:211388 99.96855:133494 100.96555:15784 102.95542:60805 114.95721:18958 115.96169:43297 116.97124:1264344 117.97753:1428314 120.96403:7764 125.97163:17274 126.96738:39663 127.975:18234 130.98712:9043 133.97353:7244 135.99091:9156 139.9868:162464 140.9953:104599 143.95718:16779 161.96983:30649 | 4.86527E-06 |

**Differences in metabolites between the WJW group, the Model group and the Control group**

| Alignment ID | Metabolite name | Rt(min) | Expreiment Mz | Adduct type | Reference m/z | MS/MS spectrum | PPM |
|---|---|---|---|---|---|---|---|
| POS8122 | JWH-370 | 4.589 | 382.21741 | [M+H]2+ | 382.21649 | 55.05365:38762 58.04137:25107 59.04858:48721 61.02743:24303 67.05389:16343 69.03294:728952 70.0366:20642 71.04816:21413 73.02811:188386 73.06432:52727 78.03804:44417 80.05429:38128 81.03229:25991 81.0699:16770 83.0477:123278 85.02799:19799 87.04334:542064 88.04604:20426 89.05896:1675804 90.0619:53117 91.04643:9790 95.04893:47874 97.06461:10190 99.04339:349853 100.05018:91888 102.06606:26695 103.03849:10777 107.07058:23191 109.06336:18528 111.04189:154115 113.05927:40171 117.09089:15015 122.06293:113519 122.56553:9929 125.0596:38116 127.075:14979 129.05446:73354 131.07088:42454 133.08543:414738 134.09018:15588 137.0598:18181 144.07597:47260 155.07083:57110 166.08865:15737 173.07771:18284 177.11285:28229 | 2.40701E-06 |
| POS15052 | 18:1(9Z)/18:2(9Z,1 | 8.722 | 786.52936 | [M+2H]2+ | 786.52789 | 52.01259:6679 60.08025:11626 71.0726:6657 86.09541:24623 100.38199:6010 104.10696:10800 124.99955:5935 184.07466:79999 518.55597:6718 | 1.86897E-06 |
| POS34 | FLUOROACETONE | 4.383 | 77.03867 | [M+H-H2O]+ | 77.0396 | 53.1757:4496 53.45075:5253 53.69243:4379 54.90918:5249 55.54824:5066 55.82938:5177 60.23557:4648 65.02227:4910 66.54385:3871 67.13178:3795 68.11464:4557 69.44248:4199 69.63427:4563 69.75673:4803 70.04163:4254 71.10993:3927 71.54332:4523 71.62527:4201 72.18309:3845 75.41446:4233 78.97468:5244 79.0169:4167 | -1.2072E-05 |
| NEG3633 | Dehydrocarissone | 0.952 | 219.13754 | [M-H2O-H]- | 219.1385 | 51.07359:5631 93.03929:2280891 103.07951:87869 103.76355:5961 123.10053:12025 151.06718:8102 | -4.3808E-06 |
| POS8010 | DIMBOA-Glc | 4.814 | 374.1109 | [M+2H]2+ | 374.10999 | 57.06894:43731 59.26746:6120 81.03226:19435 83.04929:13791 85.06334:20640 123.04189:8169 125.05956:16727 133.06561:19267 148.95413:8290 149.09477:15906 166.09781:17216 166.96548:11569 167.10458:129679 168.11118:10385 173.4303:11710 175.29941:6665 227.06221:21572 231.1055:9013 235.06412:6081 236.07355:23268 242.04243:11663 247.57834:7085 259.06525:22297 260.05313:5796 | 2.43244E-06 |
| POS6840 | Thymol glucoside | 5.258 | 313.16284 | [M+CH3OH+H]+ | 313.16379 | 55.01768:11829 55.05363:64392 57.06894:284785 69.06865:11020 71.01213:23866 81.0683:8650 83.01192:82153 83.04766:774122 84.0514:19074 85.06333:140535 97.06457:7544 99.00737:9122 111.08207:8130 115.03662:36950 127.07495:50870 137.05975:33459 139.07352:23369 155.07077:65777 157.08499:18404 159.09993:94667 171.09958:17877 179.06763:18703 197.07755:10811 | -3.0336E-06 |
| POS8848 | Glu Arg Glu | 4.46 | 433.20316 | [M+CH3OH+H]+ | 433.2041 | 62.05909:7454 85.02799:8503 87.04334:27083 89.05896:501474 90.0619:43329 110.23711:5445 127.03809:6320 131.06766:6017 133.08543:196828 134.08684:25018 173.43524:12361 177.11285:23861 | -2.1699E-06 |
| POS4286 | phenylphosphinam | 1.46 | 217.06667 | [M+H]+ | 217.0657 | 52.0803:7392 58.06319:6150 61.18333:6596 66.03267:5109 70.06427:6646 | 4.46869E-06 |
| POS13601 | Calenduloside E | 4.625 | 655.38031 | [M+2H]2+ | 655.38159 | 73.02811:264880 87.04334:687350 89.05896:3447120 113.05927:150994 133.08543:1321092 | -1.9531E-06 |
| POS7807 | nethoxy-8-(methy | 5.857 | 362.03809 | [M+CH3OH+H]+ | 362.03711 | 51.95142:6460 55.0177:5768 67.46735:6006 82.06343:12142 91.0539:100200 101.25815:5273 119.04706:16269 121.02692:16325 264.27393:7943 | 2.7069E-06 |

**Differences in metabolites between the WJW group, the Model group and the Control group**

| Alignment ID | Metabolite name | Rt(min) | Expreiment Mz | Adduct type | Reference m/z | MS/MS spectrum | PPM |
|---|---|---|---|---|---|---|---|
| POS3884 | yn-1-one\|trans-5- | 4.268 | 203.05142 | [M+H]+ | 203.0524 | 55.05363:12412 60.04424:5763 72.74344:6130 78.84068:5528 79.36346:5228 82.00914:5838 | -4.8263E-06 |
| NEG8216 | squalene | 0.812 | 395.36743 | [M-H]- | 395.36841 | 103.07276:11174 147.11324:14110 174.91417:5283 | -2.4787E-06 |
| POS5643 | no]-1,3,9-trimethy | 3.201 | 267.13202 | [M+H]+ | 267.133 | 67.05389:7067 78.88884:6173 84.07957:24893 94.57704:6600 95.29619:6594 96.04378:8462 136.06245:11875 | -3.6686E-06 |
| NEG9802 | 2'-Methoxyvinaxan | 5.522 | 605.05615 | [M-H]- | 605.05737 | 59.08407:15652 79.05373:33699 81.16663:5854 83.12411:15402 107.21418:10491 135.28148:17770 139.27896:9964 163.31036:9103 173.58612:7013 179.32071:34129 180.33003:7254 | -2.0163E-06 |
| POS6938 | -3-Hydroxypregn- | 6.683 | 317.24579 | [M+CH3OH+H]+ | 317.2468 | 67.05389:12161 69.06992:5708 105.06946:10786 119.08613:8711 131.08377:6719 164.12044:5913 173.39111:8186 | -3.1836E-06 |
| POS13644 | gamma-Truxilline | 4.462 | 659.33124 | [M+H]+ | 659.33258 | 62.52151:10420 86.09536:24341 87.0433:29724 89.05891:43916 95.96495:12038 99.04334:21490 133.08537:23260 166.3371:10479 173.43027:13733 188.06995:50777 205.09811:12467 | -2.0324E-06 |
| NEG3662 | oisobutyl phthalic | 1.325 | 221.08037 | [M-H]- | 221.08141 | 79.05373:19243 89.13132:6326 97.07652:16499 163.03299:71272 | -4.7041E-06 |
| POS8856 | Salicyloyl-conkurc | 7.323 | 433.28387 | [M+CH3OH+H]+ | 433.28491 | 53.10416:5657 59.07783:5143 62.33404:5331 94.48625:6398 152.66074:7117 | -2.4003E-06 |
| POS55 | Fluoroacetic acid | 8.803 | 79.01788 | [M+NH4]2+ | 79.0189 | 52.70664:5986 56.62345:5510 63.99746:10521 79.01992:7497 | -1.2908E-05 |
| POS4543 | Adalinine | 7.196 | 226.17897 | [M+NH4]+ | 226.17999 | 66.62321:5697 67.05389:9229 81.0699:8029 95.08477:9012 171.1862:5397 173.42545:6583 | -4.5097E-06 |
| POS14982 | -3-hydroxydecano | 8.793 | 780.54865 | [M+2H]2+ | 780.547 | 55.74166:5505 103.29499:5156 138.22501:5852 173.39102:13069 | 2.1139E-06 |
| POS15372 | Primycin B1 | 7.169 | 946.69574 | [M+NH4]+ | 946.69373 | 57.03287:19356 66.28696:5270 340.01797:5958 | 2.12318E-06 |
| POS13354 | Neomycin | 6.753 | 637.30286 | [M+2H]2+ | 637.30151 | 173.43524:9276 317.64862:5666 438.72989:7181 614.59955:6760 | 2.11831E-06 |
| POS5602 | ,6R)-2-hexoxy-6-( | 4.611 | 265.16293 | [M+2H]2+ | 265.164 | 53.46587:6831 67.6092:6033 72.95446:5688 73.02811:6498 173.43034:11133 | -4.0352E-06 |
| POS11810 | Ser Leu Pro Tyr Ala | 5.918 | 564.30145 | [M+Na]+ | 564.30267 | 57.03377:6989 59.04856:38234 60.08023:11190 86.09538:35400 87.04506:7924 89.05893:20376 103.0744:6286 104.10693:246669 105.10875:6849 126.15702:5745 146.98273:18779 173.37634:6494 | -2.162E-06 |
| POS7128 | ecylbenzenesulfon | 4.648 | 327.19986 | [M+H]+ | 327.19879 | 58.09455:6387 60.16131:5746 73.35352:6188 89.05891:10389 106.76425:5645 | 3.27018E-06 |
| POS4249 | Marindinin | 6.416 | 215.10892 | [M+H]+ | 215.11 | 55.90113:6879 57.98917:6670 60.19744:7268 61.5167:5501 62.53429:6215 70.83131:5585 123.52111:6118 | -5.0207E-06 |
| POS9231 | Tenovin-6 | 4.488 | 455.24631 | [M+H]+ | 455.24741 | 55.05365:22859 59.04858:15265 69.03294:202679 73.02811:51304 73.06432:14771 81.03229:23595 83.0477:123453 85.02799:13985 87.04334:346734 89.05896:690125 90.0619:9517 95.04893:21427 99.04339:216063 101.0593:10555 103.03849:11945 107.07059:18161 111.04189:187639 113.05927:21910 125.0596:55597 129.05446:26236 131.06766:37816 133.08543:222312 137.05637:19134 151.09305:16517 155.07083:70916 173.0826:12847 195.11966:10895 | -2.4163E-06 |
| POS6951 | uberoyl-L-carnitin | 4.365 | 318.18979 | [M+H]+ | 318.19089 | 55.05365:11102 57.03379:10806 60.08025:67546 69.06992:58223 70.0643:14150 71.04816:38446 72.08047:9082 83.08509:22174 84.07957:28562 85.02799:129011 86.09541:91104 91.0539:9058 93.06841:10767 95.08477:6639 97.06461:25073 102.05499:9713 110.07076:8873 111.0796:20090 115.07378:49827 116.06905:8732 136.07611:13535 139.07358:22015 144.10197:9751 157.08505:22809 175.09526:15938 | -3.457E-06 |

**Differences in metabolites between the WJW group, the Model group and the Control group**

| Alignment ID | Metabolite name | Rt(min) | Expreiment Mz | Adduct type | Reference m/z | MS/MS spectrum | PPM |
|---|---|---|---|---|---|---|---|
| POS7643 | Capillolide | 5.044 | 353.23419 | [M+2H]2+ | 353.23309 | 57.03287:115815 58.04137:39062 59.04859:2607516 60.05226:48531 65.04819:56027 67.05508:14948 69.06992:20878 73.02812:241521 73.06432:163613 80.0543:55733 85.06506:44910 87.04335:225480 87.06254:82433 87.07999:29597 89.05896:1496379 90.06191:47134 94.06947:27298 101.05931:145079 102.06606:28477 103.07444:367553 104.07732:15207 109.0756:38159 115.07643:17702 117.05281:192758 117.0909:49022 129.08911:26939 131.07088:105185 133.08545:425978 134.09018:17067 147.10149:87636 161.1188:22603 177.11285:32119 | 3.11409E-06 |
| POS3757 | octylpyrrolidin-2-c | 7.273 | 198.1841 | [M+H]+ | 198.1852 | 75.69515:7077 133.79388:6443 155.98309:6354 168.10655:6377 171.41733:6722 173.39111:11331 | -5.5504E-06 |
| POS15179 | xy-16,22,23-trihydı | 4.655 | 809.46619 | [M+2H]2+ | 809.46802 | 73.02811:130332 73.06432:64109 87.04334:322702 89.05896:1307563 131.06766:73103 133.08543:593855 177.895:58002 | -2.2607E-06 |
| POS5310 | Daidzein | 4.815 | 255.08171 | [M+H]+ | 255.08058 | 53.44067:6108 57.06986:7541 162.06279:6037 188.56947:5542 189.08772:11988 190.09126:9945 | 4.42997E-06 |
| POS14223 | hydroxy-6-(hydro | 4.476 | 703.36108 | [2M+H]+ | 703.36267 | 50.57987:12075 81.83556:12365 89.05891:26074 133.08537:21108 173.42535:18366 | -2.2606E-06 |
| POS12572 | Kadsuphilol C | 6.885 | 597.23431 | [M+H]+ | 597.23297 | 68.38469:5406 250.95917:6494 303.30765:6625 | 2.24368E-06 |
| POS5064 | Diethyl azelate | 7.266 | 245.17357 | [M+H]+ | 245.17468 | 60.35422:5795 69.06868:7381 72.65029:5639 83.32471:5613 177.83897:6060 | -4.5274E-06 |
| POS14414 | 2'-O-Acetylactein | 4.578 | 719.40167 | [M+NH4]+ | 719.40002 | 64.37184:55564 67.51974:55907 69.03294:237349 73.02811:120073 73.06297:67230 83.0477:116527 87.04334:450720 89.05896:1374356 95.04893:53932 99.04339:341638 111.04189:201892 122.10059:61744 125.0596:63125 133.08543:561076 150.88187:48487 155.07083:115493 174.20796:58532 362.51266:54235 | 2.29358E-06 |
| POS11018 | Phe Leu Asp Lys | 4.711 | 536.30658 | [M+2H]2+ | 536.3078 | 68.92713:6230 69.03295:5334 78.79263:5559 79.1211:5431 87.04335:8284 89.05897:32309 117.82632:5640 133.08545:6548 149.22003:5394 173.38622:8250 303.48926:5258 444.81195:5902 473.62137:6605 | -2.2748E-06 |
| POS10813 | Mls001140956 | 6.349 | 528.2981 | [M+H]+ | 528.29688 | 59.04856:8319 62.05906:16762 67.05386:6005 73.55632:5951 173.43027:7752 184.07457:6535 195.23669:5533 | 2.30931E-06 |
| POS5181 | Negamycin | 1.131 | 249.15454 | [M+H]+ | 249.15569 | 58.06512:12974 59.07198:13599 79.71541:6195 85.02799:11904 104.24855:6213 118.08494:229739 132.07565:5343 144.95276:6103 | -4.6156E-06 |
| POS8384 | Uoamine B | 4.787 | 400.28906 | [M+NH4]+ | 400.2879 | 57.03286:47673 59.04858:1348252 60.05225:17710 69.06867:7295 73.02811:10860 73.06431:28986 85.06335:14196 87.04333:55531 87.07997:12585 89.05894:612277 90.0619:10313 100.07596:9477 101.05929:76905 103.07442:379305 107.06819:16122 117.05279:116936 117.09087:34915 130.08707:9278 131.06764:18574 133.08542:88278 145.08401:9514 147.10147:149700 161.11438:29725 191.12556:10875 | 2.89791E-06 |
| POS12475 | -Tetradehydroasta | 4.575 | 592.35858 | [M+H]+ | 592.35999 | 87.04333:292582 87.73636:49559 89.05895:2113716 90.0619:265794 133.08543:920538 134.08682:187594 177.10777:66778 188.95941:48504 | -2.3803E-06 |
| POS7558 | Dodecyl glucoside | 6.14 | 349.2569 | [M+H]+ | 349.25809 | 57.03287:18914 59.04858:7832 90.3176:5956 93.2132:5617 165.29622:6592 347.04623:6512 | -3.4072E-06 |
| POS5607 | 4-ethylphenyl)-1- | 6.078 | 265.19382 | [M+H-H2O]+ | 265.19501 | 73.36568:5988 173.38618:9266 263.33527:5885 | -4.4873E-06 |

**Differences in metabolites between the WJW group, the Model group and the Control group**

| Alignment ID | Metabolite name | Rt(min) | Expreiment Mz | Adduct type | Reference m/z | MS/MS spectrum | PPM |
|---|---|---|---|---|---|---|---|
| POS4934 | enyl-3-(Pyridin-2- | 4.579 | 241.13478 | [M+H-H2O]2+ | 241.13358 | 55.05365:10048 57.06896:33395 58.06416:1074768 59.06808:27011 67.05389:24511 69.06868:13924 79.05312:12429 81.0699:142001 82.07301:10510 84.07957:53294 95.08477:23404 123.11523:84284 140.14345:14921 | 4.97649E-06 |
| POS5160 | -hydroxybutyrylcar | 2.256 | 248.1478 | [M+H]+ | 248.149 | 59.04858:11134 60.08024:14690 72.08047:40565 74.22613:5953 84.04315:15212 85.02799:85597 103.03848:12730 | -4.8358E-06 |
| POS7921 | Cholesterol | 0.727 | 369.34979 | [2M+H]+ | 369.35101 | 51.55879:6271 57.06894:6732 67.05387:10748 69.06989:6279 71.08417:7706 81.06831:12099 93.0703:6586 95.08474:9880 107.08483:9799 109.10003:11523 115.62182:6104 135.11641:11067 147.11678:9908 161.13193:8112 | -3.3031E-06 |
| POS15091 | S-3466-C | 6.728 | 793.50763 | [M+H]+ | 793.50958 | 87.02763:6464 118.83253:5497 627.29291:5952 661.3858:5900 | -2.4574E-06 |
| POS7263 | decatrienoic acid is | 7.48 | 334.30914 | [M+H]+ | 334.31039 | 199.73647:5709 | -3.739E-06 |
| POS7422 | Daphnilactone B | 6.078 | 342.24094 | [M+NH4]+ | 342.24219 | 57.06895:7702 57.4271:5912 58.06511:6050 59.05637:5598 67.05389:12496 75.02496:7322 81.06832:7854 84.07957:9365 91.05389:6026 105.06944:6899 107.2755:5846 119.08332:7047 122.09478:8318 131.08376:7998 134.09349:6883 147.11679:6070 171.90515:5738 179.28917:5348 217.66479:5314 | -3.6524E-06 |
| POS8229 | lidenebeyer-15-er | 5.054 | 389.24869 | [M+Na]+ | 389.24741 | 59.04858:19214 87.04334:6695 89.05896:9851 101.94215:5459 | 3.2884E-06 |
| POS11282 | anine-3beta,17,23 | 6.388 | 546.34113 | [M+H]+ | 546.34253 | 61.57684:6459 69.38287:7407 78.57826:5935 97.37959:6004 173.43524:12754 323.24142:5347 333.33173:6626 | -2.5625E-06 |
| POS11380 | beta-Scymnol sulfa | 5.398 | 549.31049 | [M+2H]2+ | 549.30908 | 59.04856:23963 70.72768:5438 86.09537:9585 87.0433:10775 89.05892:37861 104.10692:50456 133.08539:6636 184.06921:49416 270.05994:5654 | 2.56686E-06 |
| NEG968 | Flucytosine | 1.381 | 128.02403 | [M-H]- | 128.0253 | 89.13129:16069 127.55685:5690 128.19003:7317 | -9.9199E-06 |
| POS11944 | Antibiotic WF 3161 | 4.682 | 569.33484 | [M+H]+ | 569.33337 | 69.03294:49433 73.02811:178498 73.06432:57693 80.05429:70046 87.04334:376107 89.05896:1927869 90.0619:74864 91.04643:51479 95.04893:55157 99.04339:59499 111.04441:55463 113.05927:52859 124.08022:31021 131.06766:53555 133.08543:710807 134.09018:65579 155.07083:38486 177.11285:84402 | 2.58197E-06 |
| POS4060 | Polycanthisine | 7.199 | 208.1682 | [M+NH4]+ | 208.16949 | 52.88708:5961 109.24215:5320 110.50856:7136 124.29422:5920 163.05995:6082 | -6.1969E-06 |
| NEG6185 | | 0.908 | 309.28989 | [M-H]- | 309.2912 | 93.03925:25532 95.03763:17698 97.03951:8962 103.07947:6493 105.07814:127109 113.12114:13673 124.15676:19737 | -4.2355E-06 |
| POS13218 | baeckein F | 8.764 | 629.09088 | [M+Na]+ | 629.09253 | 62.18739:7329 70.11976:6194 87.02068:5993 91.66339:5579 141.59047:5065 535.01624:6253 561.61584:6028 | -2.6228E-06 |
| POS7610 | andamide (20:2, n | 6.825 | 352.3187 | [M+NH4]+ | 352.32001 | 86.98582:7061 267.80582:6085 | -3.7182E-06 |
| NEG3877 | Sarmentol A | 0.932 | 229.17865 | [M-H]- | 229.17999 | 79.05367:35423 93.03925:1344531 95.03763:52927 97.03745:12581 97.08466:13376 103.07946:280871 | -5.8469E-06 |
| POS3540 | 2H-3,4,5,6-tetrahy | 0.898 | 191.07605 | [M+Na]+ | 191.07739 | 60.08025:22959 61.08378:9675 62.98166:18938 68.05673:6062 72.67956:6599 72.68887:6035 84.07957:166945 85.08359:71411 90.58549:5456 130.08389:15437 131.09021:12874 173.02882:6747 191.0405:10460 191.07452:13864 | -7.0129E-06 |

**Differences in metabolites between the WJW group, the Model group and the Control group**

| Alignment ID | Metabolite name | Rt(min) | Expreiment Mz | Adduct type | Reference m/z | MS/MS spectrum | PPM |
|---|---|---|---|---|---|---|---|
| POS9171 | es-arg9)-Bradykin | 4.463 | 452.73608 | [2M+H]2+ | 452.73743 | 60.04426:33393 70.06431:764671 71.06746:21564 86.05939:5841 112.08709:15961 120.08031:56494 157.10199:11559 166.08408:6287 173.39111:10443 263.13351:20543 453.70743:8619 | -2.9819E-06 |
| POS6082 | 17_epimethanedie | 6.075 | 283.20364 | [M+H-H2O]+ | 283.20499 | 53.84824:5745 55.05365:19561 57.06895:16933 67.05389:6858 69.06867:20806 71.08546:11932 79.05312:6546 81.06989:8545 95.08476:9856 105.06944:6845 106.34853:5736 107.08485:6755 171.11885:7386 181.09694:6200 183.11298:8004 188.50836:6093 203.82985:5841 264.63409:5675 | -4.7669E-06 |
| POS5303 | Iodine | 1.063 | 254.81473 | [M+H]+ | 254.8161 | 61.03867:23704 67.29745:7271 80.94772:593200 138.90459:11091 | -5.3764E-06 |
| NEG2059 | Fluorocinnamic ac | 2.365 | 165.03433 | [M-H]- | 165.03572 | 55.9624:4941 58.11544:4593 59.91349:4666 60.95618:4588 63.27049:4503 64.56695:4678 65.45237:4508 66.11316:4603 70.95638:4896 71.53596:5115 74.69258:4643 76.7502:4013 81.75526:4291 84.90416:4658 88.45958:4754 89.58092:4436 93.14533:4997 94.02954:4403 95.4747:4193 97.07649:23371 97.18539:4177 109.36189:4477 111.2215:4581 115.00906:5117 119.16917:4485 125.9518:4829 136.76608:4327 137.42764:4511 156.04103:4502 159.0804:3900 165.81146:3944 | -8.4224E-06 |
| NEG9635 | H,2H-Perfluorode | 5.97 | 562.99481 | [M-H]- | 562.99323 | 59.08402:9092 79.05367:13711 93.85946:5239 95.63505:5906 102.93809:5184 277.54752:8793 | 2.80643E-06 |
| POS6239 | Asn Val Gly | 8.817 | 289.1492 | [M+NH4]+ | 289.1506 | 56.96448:7101 57.06989:10926 86.0954:11678 95.08476:7188 104.10696:10945 124.99955:7374 138.52921:7631 185.85204:6748 | -4.8418E-06 |
| POS10415 | Ala Leu Ala Pro Lys | 5.358 | 513.34088 | [M+2H]2+ | 513.33942 | 57.03379:102069 59.04956:2082748 60.05226:59262 65.04931:17320 69.06992:27439 73.02812:152526 73.06567:64356 80.0543:29008 83.04932:12528 85.06506:58920 87.04509:335997 87.06254:67164 87.07999:63058 89.05896:1542368 90.06374:50734 94.06947:63915 99.08151:17238 101.05931:272991 102.06606:21560 103.04074:17742 103.07444:472059 104.0796:14513 107.07059:25414 109.0756:32055 115.07378:21387 116.08248:25829 117.05553:19112 117.0909:50235 129.09227:45088 131.07088:37560 131.10634:27178 133.08545:580697 134.09018:35650 138.09604:15243 143.1078:17884 145.08403:28035 147.10149:303286 148.1028:18751 161.1188:25219 169.11278:15132 173.43034:24680 177.11285:64152 191.13127:40275 | 2.84412E-06 |
| NEG7454 | lupanacosmine | 1.079 | 356.27225 | [M-H]- | 356.27081 | 93.03925:573915 95.03763:34431 97.03745:8066 102.77911:6475 124.15676:56260 134.97455:6595 151.06711:23835 182.18414:14521 | 4.04187E-06 |
| NEG6505 | Aprindine | 6.288 | 321.23227 | [M-H]- | 321.2337 | 53.33165:5608 57.10276:13443 59.08403:341658 60.08905:44248 61.0902:19132 62.78073:6033 67.13445:11165 69.11722:49064 70.1209:15373 71.09898:13863 79.05216:7284 81.16814:10027 83.15008:13115 91.63754:6215 107.2141:39095 108.21955:32842 135.278:45323 136.28323:51715 139.27885:17981 140.28273:17285 153.31172:7172 154.31902:8632 163.30577:17887 164.31381:25905 179.32059:50916 180.32471:76803 181.33206:20822 204.42909:8547 258.54407:7895 280.51889:5748 | -4.4516E-06 |
| NEG4258 | -Undecanoylglycir | 9.352 | 242.17418 | [M-H]- | 242.17561 | 64.039:6632 90.04863:14065 | -5.9048E-06 |

**Differences in metabolites between the WJW group, the Model group and the Control group**

| Alignment ID | Metabolite name | Rt(min) | Expreiment Mz | Adduct type | Reference m/z | MS/MS spectrum | PPM |
|---|---|---|---|---|---|---|---|
| POS15316 | Antibiotic TM 531 | 6.872 | 851.54895 | [M+H]+ | 851.55139 | 53.28831:7958 54.97478:6765 105.15044:6230 254.47932:5572 438.78909:7185 631.97534:7426 | -2.8654E-06 |
| POS7836 | Inumakilactone A | 4.814 | 365.12537 | [M+2H]2+ | 365.1239 | 70.19419:5737 70.58992:6560 99.70287:6753 126.41608:6569 162.04507:6534 204.09253:9393 227.08423:22130 250.07343:9381 | 4.02603E-06 |
| POS12019 | Kinetensin 4-7 | 6.178 | 572.28857 | [M+2H]2+ | 572.29028 | 59.04859:6540 60.08025:10112 76.07848:6124 86.09541:27595 104.10697:101438 105.10879:10998 184.06931:69282 185.07635:9413 186.07535:7044 | -2.988E-06 |
| POS7757 | Ile-Arg-Ala | 6.022 | 359.23859 | [M+H]+ | 359.24011 | 52.452:5979 54.32751:8237 69.1946:5060 88.05315:6284 106.14863:6019 180.57471:6387 341.74814:5899 | -4.2312E-06 |
| POS15252 | Apramide G | 8.762 | 828.54401 | [M+H]+ | 828.5415 | 70.83127:5762 179.4697:5893 213.31702:6425 | 3.02942E-06 |
| NEG4801 | SCHEMBL14269370 | 0.906 | 261.18762 | [M-H]- | 261.1861 | 79.05368:19559 93.03925:9310 103.07947:17718 107.06415:5280 118.93213:5943 119.0854:144122 177.1145:7846 190.98895:5499 | 5.81961E-06 |
| POS8760 | ircinic acid A | 6.353 | 427.29388 | [M+2H]2+ | 427.29541 | 57.03287:29050 59.04858:206712 81.03072:22710 115.8304:5330 117.09089:26084 173.43034:8558 | -3.5807E-06 |
| POS13273 | palmitoyl-sn-glyce | 4.565 | 632.35535 | [M+H]+ | 632.35339 | 69.03294:129341 73.02811:35006 73.06431:30610 83.04769:63903 87.04333:227547 88.04781:24677 89.05895:619305 90.0619:60743 99.04338:155461 100.04802:31763 111.04189:78529 117.09088:32560 125.05659:34261 129.0513:30956 133.08543:238546 134.08682:38132 155.07082:38177 173.42543:33366 | 3.09953E-06 |
| NEG1302 | Myrothenone B | 7.705 | 138.05797 | [M-H]- | 138.0564 | 57.42459:5554 137.05487:7981 | 1.13722E-05 |
| POS9532 | Malyngamide O | 4.654 | 472.284 | [M+2H]2+ | 472.28241 | 58.04042:41917 69.03294:21966 71.04945:8981 73.02811:171275 73.06432:28295 80.05429:121145 87.04334:97696 89.05896:589103 90.0619:27539 95.04694:10836 102.06606:87038 107.07058:9726 111.06954:10968 117.08817:12382 124.08022:35440 133.08543:219018 134.08684:9092 146.09132:15891 155.09987:16375 173.43524:19122 177.11285:29597 | 3.36663E-06 |
| NEG7048 | Galactinol Dihydrat | 0.785 | 341.3584 | [M-H]- | 341.35999 | 80.71797:5797 119.32285:5836 196.84229:5703 | -4.6578E-06 |
| POS7824 | ne glycol monodo | 6.877 | 363.30881 | [M+H]+ | 363.31039 | 56.0234:6606 82.39974:5989 286.62631:5919 | -4.3489E-06 |
| POS4324 | mycin monophos | 0.779 | 218.98331 | [M+CH3OH+H]+ | 218.9817 | 51.71658:6445 61.03971:6312 68.98123:7856 73.06431:9481 86.99277:9904 98.97779:5810 101.02003:6761 107.0016:6625 119.0303:12751 121.0269:7406 131.01932:19292 145.04274:6936 149.02837:6261 159.04826:6278 162.99287:6641 190.98946:7110 194.38419:6124 | 7.35221E-06 |
| POS3176 | Phenprobamate | 0.891 | 180.0993 | [M+H]+ | 180.10091 | 50.5035:5940 56.24197:6611 160.02197:6206 | -8.9394E-06 |
| POS11350 | n E2 p-benzamido | 6.252 | 548.29883 | [M+2H]2+ | 548.3006 | 51.76935:6327 69.95988:6295 104.10697:31123 184.06931:8699 | -3.2282E-06 |
| POS14351 | -acetyltrichagmali | 7.121 | 713.31439 | [M+H]+ | 713.31671 | 110.02861:5819 173.39111:16903 193.88463:5749 | -3.2524E-06 |

**Differences in metabolites between the WJW group, the Model group and the Control group**

| Alignment ID | Metabolite name | Rt(min) | Expreiment Mz | Adduct type | Reference m/z | MS/MS spectrum | PPM |
|---|---|---|---|---|---|---|---|
| POS10090 | Halisulfate 10 | 4.503 | 499.27396 | [M+Na]+ | 499.27231 | 55.05365:28736 59.04858:18016 69.03294:236843 73.02811:68322 73.06432:23736 81.03229:38010 83.0477:126886 85.02799:18303 87.04334:463670 89.05896:1095471 90.0619:19337 95.04893:42461 99.04339:338329 101.0593:13923 107.06821:35729 111.04189:214311 113.05927:17531 125.0596:71784 129.05446:33904 131.07088:42278 133.08543:348351 137.05981:27478 151.09305:16868 155.07083:97815 173.07771:20199 173.39111:12653 175.09526:13257 177.11285:24897 383.6424:11174 | 3.30481E-06 |
| NEG3663 | SCHEMBL1388932 | 0.955 | 221.13783 | [M-H]- | 221.1395 | 93.03925:792254 95.03763:1454007 103.07947:55862 105.07814:25656 | -7.5518E-06 |
| POS9771 | aroten-4'-al; beta | 5.875 | 483.36038 | [M+H]+ | 483.36209 | 57.03284:903255 58.03659:71234 59.04856:265367 60.05223:21377 60.08022:42360 69.06989:10621 81.0683:24088 86.09537:44687 99.07935:82589 100.08238:14040 101.09636:14544 104.10692:476680 105.10874:72281 115.07373:53297 117.09084:31735 124.9995:17849 157.12306:60753 158.12607:17816 173.42537:15916 184.07457:19753 | -3.5377E-06 |
| POS7000 | tetrahydroisoquin | 8.805 | 320.1662 | [M+H]2+ | 320.16449 | 57.06989:10949 71.08547:6578 76.01012:5352 86.0954:5684 115.63253:6699 | 5.341E-06 |
| NEG4423 | dopargine | 1.353 | 248.13878 | [M-H]- | 248.1405 | 51.94239:6965 89.13124:21490 97.07645:7956 103.6411:5306 180.61613:5629 | -6.9316E-06 |
| POS8439 | yptophylalanyllysi | 4.601 | 404.23093 | [M+H]2+ | 404.22919 | 55.05365:69420 59.04858:82537 61.02743:37435 67.05389:27394 69.03294:1097856 70.0366:35509 71.04816:27370 73.02811:261407 73.06432:105466 78.03804:48174 80.05429:78280 81.03229:38017 81.0699:20906 83.0477:194623 85.02799:23581 87.04334:900676 88.04604:23622 89.05896:2799754 90.0619:89847 95.04893:56691 99.04339:524463 100.05018:130192 102.06606:47082 107.07058:27984 109.06336:26111 111.04189:254387 112.04632:16700 113.05927:72295 117.09089:31407 122.06293:201671 125.0596:59454 129.05446:97910 131.06766:64887 133.08543:726914 134.08684:40704 137.0598:32659 144.07597:79409 144.57867:17736 155.07083:88208 166.08865:22650 173.07771:23871 175.09526:27320 177.10779:45895 | 4.30449E-06 |
| POS8447 | Glu Lys Glu | 4.377 | 405.19617 | [2M+H]+ | 405.19791 | 53.77364:5498 60.04425:44760 70.06556:11702 86.0954:127568 87.0538:42449 88.03894:25101 90.05456:27388 115.48846:6684 124.03867:12775 128.10568:15187 170.0423:7647 173.12662:170473 205.07927:25285 | -4.2942E-06 |
| POS6131 | Tropisetron | 4.592 | 285.16193 | [2M+H]2+ | 285.16019 | 69.03294:11454 69.72296:6349 89.05896:7488 138.91872:5431 | 6.10183E-06 |
| POS10738 | 22alpha,23alpha-e | 6.78 | 525.34564 | [M+2H]2+ | 525.34747 | 55.72471:7451 69.15876:5473 154.17889:5150 171.15733:5928 206.71631:5893 207.01024:5952 | -3.4834E-06 |
| POS7880 | 2-Dihydro-(Z,Z)-1 | 4.827 | 367.22177 | [M+CH3OH+H]+ | 367.22351 | 59.04856:37874 70.06428:148208 73.02809:10618 84.07954:43110 87.04331:13396 89.05892:47022 94.06355:14508 96.08015:42066 116.069:13440 133.08539:8421 148.11049:302496 149.11432:17073 165.13654:37938 209.1243:8955 298.54297:5790 | -4.7383E-06 |
| POS5659 | tetramethyl-1,2-et | 6.296 | 267.20895 | [M+H-H2O]+ | 267.21069 | 55.05365:7454 67.05389:12529 122.52764:5775 141.06725:13587 143.08205:5119 155.08327:6205 159.31569:5686 177.96635:6350 183.1183:7326 | -6.5117E-06 |

**Differences in metabolites between the WJW group, the Model group and the Control group**

| Alignment ID | Metabolite name | Rt(min) | Expreiment Mz | Adduct type | Reference m/z | MS/MS spectrum | PPM |
|---|---|---|---|---|---|---|---|
| POS1262 | Hydroxyphenyletha | 1.133 | 121.07175 | [2M+H]+ | 121.07 | 56.04862:105910 57.03379:9938 60.90679:11689 61.0387:12084809 61.17308:10012 62.04229:11768 72.08047:9141 74.05886:38460 | 1.44544E-05 |
| POS10566 | Psychotrimine | 7.26 | 519.32117 | [M+H]+ | 519.323 | 50.16373:6887 173.39102:12142 190.7968:5946 313.43536:5778 443.14413:5879 | -3.5238E-06 |
| POS10472 | Conglobatin | 6.082 | 516.30499 | [M+Na]+ | 516.30682 | 50.25237:7851 53.78294:6299 53.80497:7313 60.08022:6032 86.09536:48983 95.5825:6341 100.53114:5675 104.10692:335300 105.10873:8662 146.97888:29680 171.22461:6543 173.43517:13877 280.15149:7285 499.54236:7580 | -3.5444E-06 |
| POS3415 | orosulfonyl)ethyl a | 8.788 | 186.98083 | [M+CH3OH+H]+ | 186.9826 | 55.93345:21983 57.9342:7293 58.94153:16957 59.92951:71616 59.94047:26872 60.93742:204263 68.94187:20280 75.94469:6384 77.94041:52171 78.94756:11954 81.06989:8968 86.94053:31410 100.95683:85024 101.96426:52086 104.95158:35621 116.97124:39376 117.95827:18006 117.97753:41988 118.96619:651313 119.97298:735529 120.97546:8423 127.96568:11266 128.96005:20319 129.96925:8824 137.98456:11401 141.98206:61670 142.9902:57285 145.9549:7332 163.96249:17008 | -9.4661E-06 |
| POS14170 | exanoyloxy-6-[(2S | 4.637 | 699.4054 | [M+2H]2+ | 699.4079 | 69.03294:61248 73.02811:190035 87.04334:549712 89.05896:2719911 91.04457:62847 95.04893:142404 111.04441:137877 113.05927:90196 131.06766:69592 133.08543:1072420 134.09018:86522 137.0598:90294 177.11285:104394 539.42859:63564 | -3.5745E-06 |
| POS11179 | hexoxy)pentan-2-y | 6.253 | 542.31909 | [M+Na]+ | 542.31714 | 51.5143:6374 54.3645:6571 57.03286:9626 59.04858:18997 60.08024:28518 67.05389:7207 69.0699:7761 71.07259:10684 81.06989:16238 86.09539:168554 95.08475:10387 104.10695:982143 105.10877:23540 146.97893:103052 150.06129:7659 167.15565:6181 173.43031:6585 393.3775:6571 | 3.59568E-06 |
| POS6904 | ctamide semisucc | 4.981 | 316.20999 | [M+2H]2+ | 316.21179 | 56.28908:6220 57.06894:21287 65.11468:5248 71.08544:10108 85.02796:11016 116.069:22489 144.06477:116778 155.23679:5802 158.0791:20443 204.08626:9544 | -5.6924E-06 |
| NEG4443 | Cadabicilone | 1.386 | 249.14725 | [M-H]- | 249.14906 | 50.33427:6968 51.7285:5831 56.11635:5995 89.12944:25875 126.02459:17056 126.341:5377 134.5883:4972 169.09492:6739 | -7.2647E-06 |
| POS1940 | yl]methyl 2-methy | 4.418 | 142.06476 | [M+H-H2O]+ | 142.063 | 51.89838:6403 55.0177:12582 55.05366:20095 56.9414:23620 57.68123:6654 62.30868:6446 64.73273:6578 67.05389:9188 69.03294:11704 69.06992:9261 70.06431:11796 70.44497:5969 72.93707:13392 95.08477:8545 96.08018:8232 113.96312:7614 115.05257:35396 143.0343:35726 | 1.23889E-05 |
| POS8459 | Dihydroroseoside | 4.626 | 406.24582 | [M+2H]2+ | 406.24399 | 58.04042:316049 69.03294:26840 71.04816:23644 73.02811:815241 73.06432:76618 74.03149:19535 80.05429:592291 80.55504:35823 87.04334:254484 89.05896:2323191 90.0619:75913 102.06606:456392 102.56839:31231 111.07206:34913 117.09089:24991 124.08022:170757 124.58041:21333 131.07088:32343 133.08543:732108 134.09018:31369 146.09132:51431 155.09572:46962 177.11285:66796 | 4.50468E-06 |

**Differences in metabolites between the WJW group, the Model group and the Control group**

| Alignment ID | Metabolite name | Rt(min) | Expreiment Mz | Adduct type | Reference m/z | MS/MS spectrum | PPM |
|---|---|---|---|---|---|---|---|
| NEG3825 | Pentadecanol | 0.798 | 227.23993 | [M-H]- | 227.2381 | 71.08354:41522 87.09683:59320 88.09991:9791 103.11093:93596 104.11378:13337 115.1256:84508 116.12636:9919 131.14032:348660 132.14209:51706 159.17082:77201 160.17122:17594 177.02853:5517 | 8.05323E-06 |
| NEG6902 | Istamycin KL1 | 0.963 | 335.19553 | [M-H2O-H]- | 335.1937 | 89.13124:24821 93.03925:542343 95.03763:47169 97.03745:32893 116.0619:6219 133.28078:6024 142.01402:5396 151.06711:47960 167.22272:11081 334.64075:5292 | 5.45953E-06 |
| NEG3179 | -coumaroyl-diketi | 1.208 | 204.04483 | [M-H]- | 204.043 | 79.01295:7636 89.12944:8651 93.03925:39450 124.02621:7196 148.73897:5526 167.03258:17470 | 8.9687E-06 |
| POS11349 | (-)-Detoxin D1 | 6.308 | 548.29456 | [M+H]+ | 548.29657 | 59.04859:7284 76.51501:6913 89.05896:7114 104.10697:43253 183.96208:5542 184.06931:9899 198.2594:6424 338.27423:5626 361.66922:5296 | -3.6659E-06 |
| NEG3583 | butylphosphine ox | 1.322 | 217.17096 | [M-H]- | 217.17281 | 65.3876:5879 79.05367:11310 97.07645:2343227 98.0778:15686 167.79976:6017 | -8.5186E-06 |
| POS4727 | ycero-pentulose) 1 | 4.815 | 233.10005 | [M+NH4]+ | 233.1019 | 53.03772:63084 54.04139:13798 55.01768:123942 55.05363:106245 56.02068:24513 56.05671:34031 57.03284:185372 57.06894:2077693 58.03659:32060 58.07269:616833 59.04856:33323 59.07586:10628 61.01:169423 62.01289:26131 63.00633:121529 67.01733:18516 67.05386:80745 68.05671:15839 68.99598:18621 69.03291:13742 69.06865:168111 70.0731:52136 71.01213:13405 71.04942:10780 73.00933:9853 75.00541:8774 79.05309:85527 80.05733:38082 81.03226:538095 81.06987:96583 82.03626:199772 82.07298:37309 83.04766:220530 84.0514:89592 85.02796:84541 85.06333:542437 86.03192:21388 86.06792:206682 87.06947:19218 91.05386:14044 93.06837:30923 94.07335:15087 95.0489:38375 95.08473:40722 96.05183:12069 96.08823:22827 97.02761:62571 97.06457:76830 98.03084:28852 98.06837:34211 99.04335:10673 103.03844:9134 107.04913:39429 108.0528:21900 109.06332:13291 109.10002:22181 111.04436:205374 112.04627:92888 113.0489:14727 121.10125:9485 123.0448:26997 124.05049:37016 125.05955:1076340 126.06273:628918 127.06264:86861 131.03217:11939 133.06891:78873 134.07011:101645 135.06244:119802 137.09422:21919 138.09946:13984 139.10875:21397 140.1149:18014 150.10074:9414 167.08138:22464 167.10457:1270805 168.11116:1294474 169.1127:210337 186.10252:11168 187.09335:14999 | -7.9364E-06 |
| POS2377 | e-desisopropyl-2- | 0.91 | 155.07883 | [M+H]+ | 155.0807 | 50.22104:6700 56.08018:6744 60.34113:6326 76.60997:7373 83.05907:17279 93.04334:12960 110.07076:74578 123.78096:5352 137.58165:5961 155.68228:5672 | -1.2058E-05 |

**Differences in metabolites between the WJW group, the Model group and the Control group**

| Alignment ID | Metabolite name | Rt(min) | Expreiment Mz | Adduct type | Reference m/z | MS/MS spectrum | PPM |
|---|---|---|---|---|---|---|---|
| POS979 | omethylphosphoni | 6.48 | 112.01766 | [M+2H]2+ | 112.01578 | 54.03373:103496 55.04137:15011 57.9342:140994 59.92951:82930 67.05389:22556 69.04403:12416 70.06431:13730 71.05974:323263 74.93713:25651 76.49633:17608 76.93212:12667 78.99881:19813 79.99587:19222 84.07957:13037 84.94555:17418 85.49975:9229 86.94054:7455 88.00348:610381 88.50382:41697 89.00123:340111 89.50102:31088 90.50592:8788 94.06358:11669 95.01114:12653 97.00918:209536 97.50975:10133 98.00587:132842 98.50788:10097 98.96088:29253 99.51076:99752 100.50954:66568 100.95683:13413 101.01132:12715 102.95543:16278 108.51749:16719 109.51485:11260 111.01928:11083 112.07434:12583 | 1.67833E-05 |
| POS4336 | Theobromine | 1.009 | 219.02596 | [M+H]+ | 219.02786 | 57.99675:6674 62.98166:16790 80.94775:20348 82.94376:622374 85.02799:6873 90.97557:117177 131.77626:6205 159.04828:7808 177.06223:11363 | -8.6747E-06 |
| POS14752 | Microcolin H | 5.991 | 756.54291 | [M+H]+ | 756.53998 | 60.04424:13397 60.08022:24316 86.09537:83658 124.9995:19123 184.06921:140597 | 3.8729E-06 |
| POS295 | Putrescine | 6.474 | 89.00195 | [M+H]+ | 89 | 50.37579:6720 55.05365:10358 55.93435:10232 56.04952:8560 61.02743:22531 61.0346:6141 70.0643:16085 72.93706:86397 74.99706:9477 77.99807:10994 87.00323:63718 88.07444:42139 90.94762:25573 | 2.19101E-05 |
| POS13082 | hydroxy-12-olean | 5.72 | 621.4173 | [M+Na]+ | 621.4151 | 55.77567:8051 59.04859:32506 73.02812:8587 87.04335:15535 89.05896:51955 90.06191:5907 100.78278:7247 101.05931:14746 103.07444:13967 133.08545:20985 147.10149:8119 173.39111:10285 314.7146:6119 320.94473:5878 322.51874:6938 | 3.54031E-06 |
| NEG9325 | Maltotriose | 6.334 | 502.90198 | [M-H]- | 502.89999 | 56.04784:6105 67.74068:6038 | 3.95705E-06 |
| POS7809 | Bacithrocin C 2 | 4.601 | 362.22061 | [M+NH4]2+ | 362.2186 | 58.04042:568294 69.03294:18110 71.04816:38377 73.02811:1280998 73.06432:84323 74.03149:32832 80.05429:736250 80.55659:42528 87.04334:300222 88.04782:18518 89.05896:3087766 90.0619:93126 102.06606:482204 102.56839:44585 111.07206:35091 115.07378:16474 117.09089:20523 124.08022:192208 124.58041:18554 131.07088:23712 133.08543:780054 134.08684:40726 146.09132:36484 155.09987:25200 177.11285:65140 | 5.54914E-06 |
| NEG3492 | N-Nonanoylglycine | 1.45 | 214.14711 | [M-H]- | 214.1451 | 55.67414:6437 65.62659:7043 84.0806:6578 89.13124:8733 126.02458:336706 127.0235:9115 165.97191:5996 | 9.38616E-06 |
| POS9506 | Ser His Val Lys | 4.633 | 470.27005 | [M+2H]2+ | 470.27209 | 59.04858:28327 61.02743:19094 69.03294:486273 70.0366:11048 71.04816:10393 73.02811:108277 73.06432:64282 78.03804:13932 80.05429:47826 81.03229:17258 83.0477:87647 87.04334:417368 88.04782:14229 89.05896:1414898 90.0619:50529 95.04893:30424 99.04339:216102 100.05018:36082 102.06606:30560 107.07058:14999 109.06336:9811 111.04189:108709 113.05927:36454 117.09089:16338 122.06293:73095 125.0596:25053 129.05446:37538 131.06766:34850 133.08543:424666 134.09018:26651 137.0598:14985 144.07597:36262 155.07083:45272 166.09326:19870 173.07771:12719 177.11285:37390 | -4.3379E-06 |

**Differences in metabolites between the WJW group, the Model group and the Control group**

| Alignment ID | Metabolite name | Rt(min) | Expreiment Mz | Adduct type | Reference m/z | MS/MS spectrum | PPM |
|---|---|---|---|---|---|---|---|
| POS13627 | ene\|21beta-angelo | 5.881 | 657.4751 | [M+H]+ | 657.47241 | 57.03287:56679 59.04858:643011 59.42949:8559 89.05895:18392 115.07378:17871 117.09089:104816 175.13507:13326 | 4.09143E-06 |
| POS11759 | Rhodoxanthin | 4.751 | 562.37769 | [M+NH4]+ | 562.38 | 59.04858:142885 73.02811:8122 73.06431:10740 87.04333:66884 89.05894:514488 90.0619:9773 101.05929:24945 103.07442:58253 129.0891:7427 131.07086:9705 133.08542:221969 147.10147:33828 173.38129:5567 177.11282:26216 | -4.1075E-06 |
| POS8156 | CHEMBL564302 | 4.614 | 384.23416 | [M+H]2+ | 384.23209 | 58.04136:306083 69.03294:18685 71.04816:28947 73.02811:754821 73.06432:61647 74.03149:13895 80.05429:564688 80.55503:36945 87.04334:225034 89.05895:2058075 90.0619:68475 102.06605:414678 102.56838:31943 111.07205:32339 117.09089:20394 124.08022:162777 131.07086:21136 133.08543:718377 134.09016:25768 146.09132:41706 155.09987:30189 173.40091:13649 177.11284:75759 | 5.38737E-06 |
| POS1825 | Fumaric acid | 1.867 | 139.00229 | [M+H-H2O]+ | 139.00021 | 52.35179:5638 55.0177:46251 55.05365:17776 62.64914:5860 67.05389:10667 68.996:233036 79.05312:9205 81.06989:9322 82.07301:11076 94.06554:12297 95.04893:8493 95.08476:6105 96.04379:6441 96.08827:7644 105.44981:6102 110.05835:8897 111.00671:27130 112.58314:5851 120.04356:7439 122.06003:9424 138.04724:13404 | 1.4964E-05 |
| POS11132 | yl-15-nonyl-9-pr | 5.581 | 540.36176 | [M+H]+ | 540.36401 | 51.79255:6873 57.03286:8263 59.04858:62176 60.08024:16632 70.71621:5496 83.32796:6701 86.0954:23095 87.04333:9356 89.05895:22252 104.10696:169093 117.09088:8898 173.43524:6501 184.07465:40385 339.21149:6492 | -4.1639E-06 |
| POS2107 | THTA | 0.766 | 147.04953 | [M+H]+ | 147.04739 | 55.05366:16760 56.94141:14929 58.06512:6982 68.98124:164801 69.98251:7418 72.08048:55584 72.93573:10611 74.95385:5586 84.07958:12888 86.9928:155495 89.81097:6217 100.0244:13701 101.02005:7847 105.00242:161160 106.00315:6326 118.03261:6972 119.03033:20612 123.01263:12071 128.01855:9989 132.57256:5371 137.03914:13565 | 1.45531E-05 |
| POS4492 | Cysteinyl-Cysteine | 0.99 | 225.03397 | [M+Na]+ | 225.0361 | 62.98166:93415 66.34612:5813 81.50273:5899 86.9684:6983 96.92101:46571 98.91862:42037 104.99086:895706 105.99377:9747 116.99028:108325 135.00185:16176 | -9.4651E-06 |
| POS13907 | eudoginsenoside R | 6.451 | 677.42059 | [M+H]+ | 677.42352 | 101.28879:8495 209.72971:9234 482.76108:9774 | -4.3252E-06 |
| NEG210 | 4-Pyridinol | 0.963 | 95.03929 | [M-H2O-H]- | 95.03712 | 58.87597:5863 90.95226:5704 95.03763:651935 | 2.28332E-05 |
| POS4681 | benzo<a>fluoren\| | 4.446 | 231.11897 | [2M+H]+ | 231.11681 | 51.75735:6450 56.04863:14775 60.08025:29441 72.08048:25120 84.07958:13355 85.028:226357 86.09541:26358 100.07383:16396 231.12067:29570 | 9.34592E-06 |
| POS12728 | Janthitrem E | 4.714 | 604.3606 | [M+2H]2+ | 604.36322 | 73.02811:11897 84.39672:9222 87.04334:26743 89.05896:141711 90.0619:9134 107.30415:9620 133.08543:52468 173.43034:19274 | -4.3351E-06 |
| NEG2568 | 1-Undecanolactor | 8.285 | 183.14153 | [M-H]- | 183.1393 | 55.92466:5540 83.12405:23497 116.58435:5303 142.57878:5698 | 1.21765E-05 |
| POS8802 | 7,8-didehydro-8'- | 5.794 | 430.28433 | [M+H]+ | 430.28662 | 59.04859:7332 60.08025:14503 76.14551:6421 85.028:73655 86.03025:20533 89.05896:6834 165.22324:5583 173.43036:6525 318.39163:7013 | -5.322E-06 |

**Differences in metabolites between the WJW group, the Model group and the Control group**

| Alignment ID | Metabolite name | Rt(min) | Expreiment Mz | Adduct type | Reference m/z | MS/MS spectrum | PPM |
|---|---|---|---|---|---|---|---|
| POS8593 | tadecanedioylcarn | 6.351 | 416.30289 | [M+2H]2+ | 416.3006 | 57.03287:79220 59.04858:714507 60.05225:16413 87.04334:48032 99.07938:16584 101.09422:6412 115.07378:14586 117.09089:53473 175.1301:8154 | 5.50083E-06 |
| POS9651 | cyclo[10.8.0.02,9.04 | 8.809 | 478.32684 | [M+H-H2O]+ | 478.32913 | 50.00464:6662 62.23794:5641 86.09537:7955 87.28099:5024 88.32702:6885 111.99535:7242 119.55073:5597 192.5636:6034 428.54907:6149 | -4.7875E-06 |
| POS10117 | Osimertinib | 6.157 | 500.27371 | [M+Na]+ | 500.276 | 59.04858:7297 60.0093:5919 89.05895:6451 120.96402:6568 | -4.5775E-06 |
| POS12122 | 2-yl]-18-norpregn | 4.835 | 576.39209 | [M+NH4]+ | 576.3894 | 59.04859:1065958 73.02678:55432 73.06432:42095 85.06337:36108 87.04335:323799 87.07999:47369 89.05896:2615279 90.06375:44507 101.05931:177454 101.09423:22210 103.07444:531423 105.08796:24071 117.05281:53286 117.0909:39218 129.08911:65461 131.06766:92997 131.10634:31421 133.08545:1296119 145.08403:55253 147.10149:480032 161.1188:34809 177.11285:190329 189.11574:24983 191.1256:129740 205.14238:31715 341.20605:24596 | 4.66698E-06 |
| POS10067 | Poricoic acid A | 5.496 | 498.33691 | [M+2H]2+ | 498.33453 | 57.03287:287249 59.04859:4624726 60.05226:121201 60.08025:118506 65.04819:79492 69.06992:81700 73.02812:449603 73.06432:126714 80.0543:89781 85.06337:157684 86.09541:264607 87.04335:567264 87.06079:199582 87.07999:106976 89.05896:3489491 90.06191:134469 94.06947:103107 101.05931:522818 102.06606:46573 103.07444:1024088 104.10697:1308416 105.10879:365901 106.11105:46620 109.0756:56872 115.07378:52093 117.05281:102652 117.0909:87329 124.99956:93343 129.08911:80034 131.06766:72005 133.08545:1056324 134.08684:51277 143.1078:55076 145.08778:54046 147.10149:505908 161.1188:83836 173.39111:83174 177.11285:126451 184.07466:896408 185.07635:309374 186.07535:146422 191.1256:114480 205.14238:48011 | 4.77591E-06 |
| POS8077 | 4-bromophenyl)ch | 0.882 | 378.89838 | [M+Na]+ | 378.896 | 90.97556:16093 106.94932:100680 174.93611:8732 | 6.28141E-06 |
| POS15273 | pteriatoxin B | 4.672 | 831.47833 | [M+2H]2+ | 831.4823 | 84.64203:28986 87.04334:84677 89.05896:302733 133.08543:119601 151.0771:30111 162.6404:26828 173.43524:53055 217.99625:28931 236.25288:28998 316.36374:26634 359.20026:28753 760.00513:29745 | -4.7746E-06 |
| POS1292 | -Mercaptolactic ac | 0.763 | 123.01339 | [M+Na]+ | 123.011 | 68.98124:26614 73.66351:6563 80.04815:9105 86.99279:29251 105.00241:38609 111.29868:5666 | 1.94292E-05 |
| POS1695 | Iodotyrosine | 8.763 | 135.00243 | [M+H]+ | 135 | 91.13419:5808 111.37939:6078 | 1.8E-05 |
| POS13903 | ahydrogambogic / | 4.624 | 677.39545 | [M+2H]2+ | 677.39215 | 73.02811:224730 77.35265:107361 87.04333:482475 89.05895:2727703 95.04893:120999 99.04338:114120 106.65066:121495 111.04189:180773 111.29109:94109 113.05926:113537 133.08543:1022934 177.11284:128923 182.38081:103717 432.59784:134147 | 4.87162E-06 |
| POS10433 | ydrocochlioquinor | 4.649 | 514.29553 | [M+2H]2+ | 514.29303 | 69.03294:203259 73.02811:75934 73.06432:45759 80.05429:31685 83.0477:45819 87.04334:208479 89.05896:751023 90.0619:31274 99.04339:103542 100.05018:21287 102.06606:17963 111.04189:58334 113.05927:26421 122.06293:37257 129.05446:20840 131.07088:19682 133.08543:257239 144.07597:23916 155.07083:25151 163.73279:16963 175.10023:16845 177.10779:34261 | 4.86104E-06 |

**Differences in metabolites between the WJW group, the Model group and the Control group**

| Alignment ID | Metabolite name | Rt(min) | Expreiment Mz | Adduct type | Reference m/z | MS/MS spectrum | PPM |
|---|---|---|---|---|---|---|---|
| POS15065 | indol-3-ylmethyl)- | 4.659 | 787.45392 | [2M+H]2+ | 787.45007 | 87.04334:482067 89.05896:1831671 97.71687:87547 113.05927:156148 131.07088:113006 133.08543:857033 177.11285:118860 | 4.8892E-06 |
| POS4291 | Longiflorone | 5.696 | 217.10446 | [M+NH4]+ | 217.1069 | 55.01768:16464 55.05363:17945 57.03284:43864 57.06986:19536 58.03659:7789 61.01:13364 63.00633:9905 67.01733:8009 67.05269:7271 69.06989:6962 75.02634:22691 77.02202:15905 79.05309:15500 80.05733:12623 81.06987:15537 82.07138:6386 83.04766:31121 84.0514:11510 85.02796:29634 86.03021:6940 97.06457:16772 107.04913:8384 111.04185:21558 112.04627:14442 124.05049:13923 125.05955:275719 126.06273:164950 127.06264:21246 167.10457:283170 168.11116:288651 169.1127:49499 | -1.1239E-05 |
| POS11752 | etraen-1-yl]-5,6-d | 5.605 | 562.33954 | [M+H]+ | 562.3374 | 59.04859:14791 60.08025:13667 86.09541:19358 89.05896:9053 104.10697:110067 133.08545:7633 184.07466:28801 | 3.80554E-06 |
| POS12764 | h_78_metenolone | 6.296 | 605.453 | [2M+H]+ | 605.45599 | 55.05365:18530 57.06895:14888 67.05389:44064 69.06867:27070 78.97769:6644 79.05312:16915 81.06989:42359 83.08508:13350 91.05389:13794 93.0684:29912 95.08476:34396 105.06944:15997 107.08485:21475 109.10005:12111 117.06911:14630 119.08332:20444 121.10129:15391 131.08376:16397 145.09903:7917 147.11296:8111 149.0948:7143 | -4.9384E-06 |
| POS9252 | Arachidyl carnitine | 5.93 | 456.4028 | [M+NH4]+ | 456.40527 | 57.06989:6800 60.08025:30331 85.02799:162764 372.31253:6191 | -5.4119E-06 |
| POS13883 | Stevastelin D3 | 4.552 | 676.38037 | [M+H]+ | 676.38373 | 69.03293:85440 87.04333:159569 89.05894:513421 89.77805:41723 99.04337:115176 133.08542:183022 155.06667:43362 173.43031:62566 270.54675:37789 544.49554:35321 | -4.9676E-06 |
| POS15117 | lucidanin | 6.195 | 795.54449 | [M+H]+ | 795.54053 | 51.78454:6742 59.04857:8131 | 4.97775E-06 |
| POS11691 | Homoamericin | 4.506 | 560.32593 | [M+H]+ | 560.32312 | 69.03294:55295 70.76349:11144 73.02811:25559 83.04932:15615 87.04334:117755 89.05896:361677 99.04339:115428 111.04189:54655 125.0596:21098 131.06766:19237 133.08543:129752 155.07083:33561 173.43034:15515 | 5.01496E-06 |
| POS13081 | 9)en-2,28-diol-3-y | 5.736 | 621.4118 | [M+2H]2+ | 621.41492 | 57.03287:56259 59.04859:169509 69.06992:13135 73.02812:43201 73.06432:20588 76.31847:9349 81.0699:10492 85.06337:40862 87.04335:72631 87.07999:14273 89.05896:238665 89.37024:10329 93.17069:9033 99.07939:11249 101.05931:59170 103.07444:35312 115.07378:15233 117.0909:11264 129.08911:29525 133.08545:84440 143.10413:12803 147.10149:18299 155.00865:10393 | -5.0208E-06 |
| POS8736 | alpha-rhamnopyra | 4.599 | 426.24603 | [M+2H]2+ | 426.2486 | 55.05363:41015 59.04856:44172 61.02843:28454 69.03291:717016 70.03657:16774 71.04813:16943 73.02808:176514 73.06429:74263 78.038:22960 80.05426:44187 81.03225:26480 83.04766:97931 87.0433:533648 88.04601:24610 89.05891:1840733 90.06187:73354 95.04889:44866 99.04334:301216 100.05013:64558 102.06601:27615 107.06815:17809 111.04185:146249 113.05922:39878 117.09084:31556 122.06287:110193 125.05954:30750 129.05441:61730 131.07082:39951 133.08537:474505 134.08678:16236 137.05974:15891 144.07591:34950 155.07077:56603 173.08252:20539 175.09518:15045 177.11278:38658 | -6.0293E-06 |

**Differences in metabolites between the WJW group, the Model group and the Control group**

| Alignment ID | Metabolite name | Rt(min) | Expreiment Mz | Adduct type | Reference m/z | MS/MS spectrum | PPM |
|---|---|---|---|---|---|---|---|
| NEG7495 | Sinigrin | 1.077 | 358.27255 | [M-H2O-H]- | 358.26999 | 52.36927:5581 68.06616:5950 93.03925:344479 95.03763:297239 97.03745:9331 124.15676:71886 153.06754:8821 182.18414:14831 239.79329:5500 | 7.14545E-06 |
| NEG3252 | droxytrihomometh | 1.228 | 206.08826 | [M-H2O-H]- | 206.08569 | 59.08407:15520 73.11623:33213 75.54928:5876 79.4505:5340 89.13131:7356 95.0377:12043 103.68422:6117 108.77026:5580 126.02468:113253 143.15802:6167 161.03633:9333 181.00244:6086 | 1.24705E-05 |
| POS2497 | enzenesulfonic aci | 0.759 | 159.01361 | [M+H-H2O]+ | 159.011 | 55.0177:21242 55.05365:12996 55.93434:80591 56.04952:8941 56.94232:9886 67.05389:20318 68.98123:145643 69.06991:10543 69.9825:8849 70.06429:15395 70.97875:7572 71.04815:9243 72.04369:6827 72.93706:22034 73.06431:35320 77.00898:6160 81.06989:6313 85.01283:17215 86.99277:124379 87.99284:8895 90.94576:6641 95.08475:21154 103.02276:78286 105.0024:157077 106.00548:8678 106.99923:15552 113.00764:17669 113.9631:12778 114.97044:11630 117.03919:20402 121.03262:38130 123.0126:23543 131.01932:140857 132.02023:6080 135.049:14033 147.04784:12385 149.02837:49767 149.06354:9678 | 1.6414E-05 |
| POS15116 | lucidanin | 6.192 | 795.53638 | [M+Na]+ | 795.54053 | 59.04858:13984 73.63771:5679 74.19592:5868 118.47446:5723 | -5.2166E-06 |
| POS10348 | Narbomycin | 8.759 | 510.34515 | [M+2H]2+ | 510.3425 | 70.88638:5356 87.14983:6076 108.37923:4924 313.50699:5615 353.19443:5993 | 5.19259E-06 |
| POS2928 | hyl-1,4-naphthoq | 4.922 | 173.06264 | [M+Na]+ | 173.06 | 55.05363:9834 67.05386:25503 71.71907:6162 86.84657:5966 109.10002:10689 125.05955:51217 173.39104:10989 | 1.52548E-05 |
| POS6820 | Adiphenine | 4.652 | 312.19916 | [M+NH4]+ | 312.19647 | 59.04859:833715 60.05226:67039 73.06432:8571 85.06506:15342 87.04335:65677 88.04605:6379 89.05896:1032050 90.06191:127384 91.06323:7664 101.05931:25769 103.07445:99740 104.07732:13123 107.07059:10487 117.05281:9556 133.08545:147176 134.09018:24780 147.10149:25984 | 8.61637E-06 |
| POS3354 | thyl-1-azulenecarb | 4.815 | 185.09868 | [M+H]+ | 185.09599 | 53.03854:7116 55.01768:6571 55.05363:8614 57.06894:89617 57.93418:13230 61.01:267803 63.99744:6028 67.05386:23601 75.56093:6006 81.03226:12174 81.06987:10338 85.06333:11562 87.02412:6764 91.05386:6057 95.0489:29208 109.10002:11649 | 1.4533E-05 |
| POS9356 | an-2-yl)-10,13-di | 5.677 | 461.32129 | [M+2H]2+ | 461.32401 | 57.03286:34728 59.04858:503341 60.05225:12222 63.82409:6740 73.02811:22106 73.06431:9679 85.06335:6543 87.04333:29484 87.07997:10785 89.05894:144769 90.0619:6752 94.06945:9436 101.05929:31698 103.07442:97789 117.09087:12654 133.08542:26507 147.10147:25154 161.11877:7539 | -5.8961E-06 |
| NEG3634 | rioxa-1,13-trideca | 1.32 | 219.16878 | [M-H2O-H]- | 219.17149 | 97.07645:143213 97.36658:5572 99.07355:118894 102.80595:5628 161.03622:6788 | -1.2365E-05 |
| POS7473 | Piperolein B | 4.464 | 344.22577 | [M+NH4]+ | 344.22299 | 87.04335:17462 89.05896:404679 90.06375:6687 97.43122:6500 133.08545:143591 177.11285:12752 | 8.07616E-06 |
| POS9869 | Pro-aMCA | 8.777 | 488.33322 | [M+2H]2+ | 488.336 | 91.88431:5876 149.21219:6338 | -5.6928E-06 |

**Differences in metabolites between the WJW group, the Model group and the Control group**

| Alignment ID | Metabolite name | Rt(min) | Expreiment Mz | Adduct type | Reference m/z | MS/MS spectrum | PPM |
|---|---|---|---|---|---|---|---|
| POS9870 | ydroxydocosa-4,7, | 4.81 | 488.33981 | [M+NH4]+ | 488.33701 | 57.03286:24280 59.04858:739728 60.05225:10445 73.02811:16689 73.06431:10252 85.06335:13911 87.04333:98766 87.07997:18103 89.05894:805009 90.0619:14342 101.05929:82125 103.03848:10202 103.07442:282660 103.96793:6582 107.07057:7414 117.05279:26650 117.09087:13071 129.0891:12148 131.07086:12641 131.10631:7236 133.08542:227288 145.08401:9229 147.10147:140270 160.1046:6894 161.11438:15337 177.11282:10423 191.12556:21180 | 5.73375E-06 |
| POS7672 | s-Cyclosuffrobuxi | 6.034 | 354.2821 | [M+H]+ | 354.2793 | 55.05366:20769 57.06897:29820 59.03105:17005 59.04859:185439 61.01003:7553 73.04689:9508 75.02637:12812 87.04335:29207 89.05897:6684 91.05764:9443 103.07445:209723 131.08055:5746 | 7.90337E-06 |
| POS1494 | 4-Thiouracil | 0.761 | 129.01442 | [M+H-H2O]+ | 129.0116 | 55.05365:41874 56.04952:7266 59.04858:6643 68.98123:66361 69.82184:6053 69.9825:11398 82.01235:14051 82.06502:12911 83.00869:7963 84.07957:34310 86.99104:60373 87.99284:8064 100.02438:32180 101.02003:30250 105.0024:83125 106.00314:10898 110.2371:6091 118.03534:9650 119.0303:75677 123.0126:11470 128.01854:10268 129.01982:7432 | 2.18585E-05 |
| POS8211 | eucylglutaminyllys | 4.476 | 388.25256 | [M+Na]+ | 388.2554 | 73.06432:11103 87.04334:33327 89.05896:786442 90.0619:16625 107.07059:7810 131.06766:9936 133.08543:294603 134.08684:8135 173.39601:8740 177.11285:39299 | -7.3148E-06 |
| POS12918 | melianol | 4.544 | 613.34045 | [M+Na]+ | 613.33698 | 52.72472:15674 57.68122:14530 59.89664:15405 64.38071:16020 69.0884:14292 86.0954:21295 135.45438:14429 148.67734:14215 311.24231:17300 | 5.65758E-06 |
| POS8206 | Neoenactin NL2 | 6.197 | 387.28244 | [M+2H]2+ | 387.28531 | 57.03287:41392 59.04858:357778 60.05225:7977 75.11298:5347 87.04334:34951 99.07938:8139 115.07378:10366 117.09089:21867 206.25749:6138 273.48544:5697 | -7.4106E-06 |
| POS1205 | -Nitrosothiazolidi | 0.763 | 119.03017 | [M+CH3OH+H]+ | 119.0273 | 53.03773:6029 55.05365:293886 55.93434:9302 56.05673:14151 56.94139:140906 57.05692:28162 58.5172:6224 59.04858:7664 72.08047:370484 72.93706:120281 73.08309:20993 74.95245:9104 77.8651:5377 90.94762:48955 91.34943:5251 101.02003:10175 119.0303:32603 | 2.41121E-05 |
| NEG9525 | Chlorfluazuron | 6.082 | 538.96613 | [M-H2O-H]- | 538.96301 | 173.6057:21503 253.52328:66828 254.53137:9394 284.3291:5692 | 5.78889E-06 |
| POS6728 | Paromomycin | 8.812 | 308.65833 | [M+Na]2+ | 308.65543 | 57.06989:43927 58.06511:11537 59.07198:10592 67.05271:10123 69.06868:6157 71.08418:22973 81.0699:6861 85.10045:8086 86.0954:13526 95.08476:15209 104.10696:9559 149.01665:5558 165.02727:9359 184.06929:6595 | 9.39559E-06 |
| POS14654 | -cyclopenta[a]phe | 4.648 | 743.42847 | [M+2H]2+ | 743.42413 | 72.4287:86873 80.15437:81887 87.04334:542368 89.05896:2429276 133.08543:902522 150.55998:93019 156.23862:78811 177.10779:106599 238.40434:82686 461.10242:89324 | 5.83785E-06 |
| POS15168 | PC(17:1/12-HEPE) | 8.623 | 808.54535 | [M+2H]2+ | 808.54858 | 50.21645:5920 54.8856:6263 57.75751:6358 173.39111:9749 497.72522:5572 | -3.9948E-06 |

**Differences in metabolites between the WJW group, the Model group and the Control group**

| Alignment ID | Metabolite name | Rt(min) | Expreiment Mz | Adduct type | Reference m/z | MS/MS spectrum | PPM |
|---|---|---|---|---|---|---|---|
| POS9130 | Malyngamide M | 4.648 | 450.27386 | [M+2H]2+ | 450.27689 | 58.04042:159330 69.03294:25197 71.04816:13779 73.02811:383167 73.06432:44608 74.03149:12087 80.05429:234713 80.55504:12762 87.04334:122674 89.05896:898052 90.0619:49972 102.06606:155110 102.56839:13871 111.07206:13246 117.09089:10217 124.08022:47892 133.08543:271057 134.09018:13289 146.09132:11754 173.43034:13484 177.11285:22689 289.82092:10072 | -6.7292E-06 |
| NEG4055 | Dropropizine | 0.932 | 235.14226 | [M-H2O-H]- | 235.14529 | 93.03925:684991 95.03764:5941 103.07947:15903 124.15677:24049 177.1145:28349 | -1.2886E-05 |
| POS781 | 2-Cyanopyridine | 0.767 | 105.00304 | [M+Na]+ | 105 | 58.06416:80101 58.99696:15260 61.1515:5169 68.98123:483576 77.00752:6727 86.99104:609953 87.99284:5427 90.89735:6337 105.0024:672241 | 2.89524E-05 |
| POS13108 | Antibiotic M 230B | 5.663 | 622.42749 | [M+H]+ | 622.43127 | 59.04856:123729 87.04331:19210 89.05893:108340 101.05927:22135 103.0744:32797 115.92144:9283 133.08539:26790 147.10143:9544 | -6.073E-06 |
| POS13219 | baeckein F | 8.78 | 629.09644 | [M+H]+ | 629.09253 | 67.19205:6851 69.94729:7347 95.36812:6350 100.12971:5204 282.84961:6618 | 6.2153E-06 |
| POS2846 | 2-Furoylglycine | 0.765 | 170.0405 | [M+NH4]+ | 170.04359 | 67.05389:5526 68.98123:128682 71.71257:6216 76.515:5693 81.06832:6663 82.01395:20593 83.00869:31886 86.99277:146306 100.02438:40441 101.01785:118621 102.01955:6718 105.0024:181483 118.03534:14167 119.0303:229980 120.02943:7976 123.0126:18484 123.46803:5846 128.01854:33603 129.01353:15591 137.03912:93193 142.03293:7856 147.02486:59909 165.03636:9211 | -1.8172E-05 |
| NEG1145 | 3-Trimethoxyprop | 8.741 | 133.08394 | [M-H]- | 133.0871 | 55.03414:5771 56.21574:6244 65.09239:15039 67.10966:7483 79.05367:8124 91.13873:7797 100.04587:14211 101.05437:131678 102.05606:12560 109.54619:6082 116.05923:190376 117.06135:15043 133.08284:8208 | -2.3744E-05 |
| POS12920 | limnantheoside A | 4.7 | 613.362 | [M+H]+ | 613.35822 | 73.0281:27054 73.06429:10545 79.63444:12220 80.05273:13791 87.04332:72595 89.05893:308858 90.06371:25293 95.0489:11224 113.05666:13771 133.0854:121564 | 6.16279E-06 |
| POS12665 | orodecane sulfoni | 7.882 | 600.93445 | [M+H]+ | 600.93829 | 68.48682:5486 90.24759:6837 173.43526:12346 293.1124:5001 369.65771:5913 554.7619:6490 | -6.39E-06 |
| NEG8825 | 3beta-ol\|4alpha,2 | 0.904 | 443.42911 | [M-H2O-H]- | 443.4259 | 55.6278:5822 93.03925:66331 95.03763:35097 97.03745:12013 103.07947:87112 113.12114:15535 257.57416:5848 | 7.23909E-06 |
| POS11664 | Sepaconitine | 6.214 | 559.29767 | [M+2H]2+ | 559.30127 | 50.40268:8965 52.1749:6583 53.81686:5946 68.18956:5778 104.10696:18576 224.66388:6301 240.11317:5511 245.61572:5266 266.03574:5919 502.1665:7462 | -6.4366E-06 |
| POS5741 | Diuron | 1.025 | 270.97675 | [M+Na]+ | 270.98001 | 62.98163:22976 74.09579:5637 76.16116:5345 80.94772:29281 82.94373:6478 90.97554:17180 96.92096:10587 104.99081:108276 116.99022:15700 135.00177:368785 136.00449:6799 140.06859:13752 | -1.203E-05 |

**Differences in metabolites between the WJW group, the Model group and the Control group**

| Alignment ID | Metabolite name | Rt(min) | Expreiment Mz | Adduct type | Reference m/z | MS/MS spectrum | PPM |
|---|---|---|---|---|---|---|---|
| POS8769 | Andrastin D | 4.637 | 428.25958 | [M+H]2+ | 428.25629 | 58.04042:189786 69.03294:19953 71.04816:16872 73.02811:452445 73.06432:55546 74.03149:12153 80.05429:357769 80.55504:20817 87.04334:174921 88.04782:9696 89.05896:1442791 90.0619:64847 91.06323:9601 99.04339:10211 102.06606:253790 102.56839:18867 107.06821:11122 111.07206:13912 113.05927:11019 115.07378:10454 117.09089:16175 124.08022:101969 124.58041:11349 131.07088:20308 133.08543:466748 134.08684:22188 146.09132:30048 155.09987:29479 177.11285:51265 | 7.68232E-06 |
| NEG8927 | .-Triacontanediol( | 0.864 | 453.47113 | [M-H2O-H]- | 453.4678 | 76.92368:6228 84.98986:6183 86.25851:6162 113.12118:1495906 114.12465:34477 172.76915:6133 181.19069:44372 249.2543:6909 | 7.34341E-06 |
| POS9287 | adecahydro-3,5-d | 4.85 | 458.29333 | [M+NH4]+ | 458.29001 | 59.04859:127912 62.9291:6736 87.04335:275102 89.05896:95178 101.05931:69613 103.0385:62724 103.07445:31513 110.84615:5616 117.05281:34988 131.07088:8119 133.08545:33611 136.56493:5515 147.10149:8298 180.89824:5683 | 7.24432E-06 |
| POS3236 | L-Tyrosine | 2.199 | 182.08022 | [M+H]+ | 182.08118 | 77.03802:15550 91.05391:637734 92.05669:18134 93.06841:8684 95.04894:297246 103.05422:18541 107.04918:17582 109.06337:19536 118.06567:14021 119.04707:396222 120.05206:9361 121.06411:9132 123.04193:399989 124.04758:7692 136.07611:448969 137.07706:13726 147.04404:69719 165.05461:33159 | -5.2724E-06 |
| POS2411 | Histidine | 1.055 | 156.07626 | [M+H]+ | 156.07678 | 56.04861:28400 68.04948:13371 81.04324:6644 82.05224:18332 83.05906:110927 93.04333:76341 95.05887:33226 100.90237:6143 104.79472:5957 110.07073:347611 | -3.3317E-06 |
| POS1770 | Hypoxanthine | 4.373 | 137.04532 | [M+H]+ | 137.04578 | 55.02822:373014 57.06896:12667 67.02796:86950 67.05389:58725 72.93707:26646 79.05313:37936 81.06834:159572 82.03949:271530 83.02332:40703 91.0539:79146 92.02255:44135 94.03812:523453 95.08477:94932 110.03357:1091621 112.04887:62715 119.03312:800626 120.01816:13134 128.04344:37191 137.04604:3745190 138.02985:30815 | -3.3565E-06 |
| POS1184 | L-Valine | 1.106 | 118.08609 | [M+H]+ | 118.08628 | 53.01368:8607 53.03856:65233 55.05365:3077097 56.04952:54851 57.05414:55938 57.05692:310822 58.06511:25899 59.04858:57039 59.07294:20713 71.06873:8607 72.08047:4019858 73.06431:7744 73.08443:10666 118.08493:16295 | -1.609E-06 |
| POS5832 | auryldiethanolamir | 5.023 | 274.27338 | [M+H]+ | 274.27408 | 55.05365:20481 57.06896:921924 58.06416:58013 58.07272:18651 60.04325:14176 62.05909:51561 68.04829:8100 69.06991:12514 70.0643:945401 71.06745:19545 71.08418:297450 72.08836:10263 74.05885:7639 85.10044:81364 86.05938:14506 88.07444:1278210 89.07881:29560 102.09042:352008 103.09467:7840 106.08522:681112 107.08961:13477 212.23726:7148 230.24779:8332 256.25845:32904 274.2739:217946 275.27167:24002 | -2.5522E-06 |
| POS4078 | Kynurenine | 4.336 | 209.09169 | [M+H]+ | 209.09209 | 74.02328:33601 91.0539:6350 94.06359:112013 99.00742:20398 104.04768:13858 118.0629:36202 120.04358:39052 132.04306:15007 136.07611:15142 146.05721:51480 150.05342:7705 174.05498:15598 | -1.913E-06 |

**Differences in metabolites between the WJW group, the Model group and the Control group**

| Alignment ID | Metabolite name | Rt(min) | Expreiment Mz | Adduct type | Reference m/z | MS/MS spectrum | PPM |
|---|---|---|---|---|---|---|---|
| POS3014 | DL-Arginine | 1.029 | 175.11815 | [M+H]+ | 175.11897 | 60.05526:769510 70.06431:2166182 71.04816:42605 71.06746:29367 72.08048:76556 84.07957:21869 88.07445:9948 97.07488:9417 98.06007:10224 112.08709:35482 113.0696:19749 115.08704:8718 116.06905:236146 130.09663:114071 134.01685:9323 158.09198:33378 175.12015:47736 | -4.6825E-06 |
| POS2215 | Methionine | 1.565 | 150.05785 | [M+H]+ | 150.05827 | 53.03773:28598 56.04862:4098100 57.03286:40299 57.05229:26356 58.99405:43779 61.01003:3042040 74.02328:200846 74.05885:141817 74.98869:16092 75.02496:27598 77.00462:19300 84.04315:60120 85.00947:23833 85.02799:62622 87.0259:334150 102.05498:226649 104.05223:569591 105.00008:19749 105.03476:23692 133.02939:316994 150.05736:14543 | -2.7989E-06 |
| POS161 | Tetrahydropyridine | 1.102 | 84.08079 | [M+H]+ | 84.08077 | 55.05365:23097 56.04862:215522 57.06988:9418 67.05389:28831 69.05635:8003 82.06502:37404 82.94376:11047 84.04315:141536 84.07957:511719 | 2.37867E-07 |
| POS3070 | Serotonin | 3.359 | 177.10153 | [M+H]+ | 177.10223 | 55.93435:49513 67.04092:27707 68.70155:5785 72.93707:17109 72.96115:9613 79.05313:7960 90.94764:6919 91.0539:13002 94.04205:7205 95.9206:5567 105.06946:43974 115.05257:174659 117.05553:76819 130.06477:10756 131.04832:11171 131.07088:9076 132.07892:159173 133.06236:28355 135.94325:6628 142.06567:27246 143.07104:15197 159.06984:16603 160.07417:785549 161.07928:20445 174.8517:5705 | -3.9525E-06 |
| POS6593 | adecyldiethanolan | 5.238 | 302.30368 | [M+H]+ | 302.30539 | 55.05366:13204 57.06897:611891 58.06512:28764 58.07272:12523 60.04426:8334 62.0591:34532 64.82231:6567 68.0483:9617 69.06869:8540 70.06431:594018 71.08548:215123 72.08837:7588 85.10046:75133 86.05939:9152 88.07445:808188 89.07883:13366 102.09042:211452 106.08524:437557 107.08725:6986 152.76207:6151 194.68724:6670 284.29575:9125 302.30051:69166 303.30765:7273 | -5.6565E-06 |
| POS2981 | Hexanoylglycine | 4.763 | 174.11218 | [M+H]+ | 174.11247 | 55.05365:13064 71.08547:378903 76.03858:429061 76.36717:6444 81.06833:31898 86.0954:13485 99.07938:93356 110.10796:5561 | -1.6656E-06 |
| POS2296 | Xanthine | 2.151 | 153.0399 | [M+H]+ | 153.04068 | 55.02822:218475 55.05366:12446 55.93435:8781 79.05313:12347 81.00723:29302 82.03949:98438 83.02332:23846 83.04932:7681 92.0491:17941 93.00867:15229 107.04918:12248 108.01909:12309 108.04321:9568 110.03357:754289 110.06084:19264 128.04344:78686 136.01477:29202 153.04224:170907 154.0228:243066 | -5.0967E-06 |
| POS10644 | LysoPC(18:1/0:0) | 5.57 | 522.35522 | [M+H]+ | 522.35541 | 58.06512:449133 60.08025:4693632 67.05389:258153 69.06992:217609 71.07261:665162 81.0699:254886 83.0851:210672 86.09541:11328304 89.05896:197113 95.08478:374162 104.10697:67285736 105.10879:1243121 124.99956:3462611 163.01524:388928 184.07466:61771484 185.07635:1554875 258.10977:355226 339.29205:229168 | -3.6374E-07 |
| POS5416 | ycerophosphochol | 1.149 | 258.10968 | [M+H]+ | 258.11008 | 60.08025:65541 71.07261:10895 74.4781:5594 86.09541:106029 98.98415:7132 104.10697:588962 105.10879:17453 124.99956:95822 184.06931:20027 | -1.5497E-06 |

**Differences in metabolites between the WJW group, the Model group and the Control group**

| Alignment ID | Metabolite name | Rt(min) | Expreiment Mz | Adduct type | Reference m/z | MS/MS spectrum | PPM |
|---|---|---|---|---|---|---|---|
| POS10646 | ysoPC(18:1(11Z)/0: | 8.803 | 522.35553 | [M+H]+ | 522.35596 | 60.08025:221595 71.07261:35196 86.09541:701630 104.10697:335647 124.99956:210444 184.07466:4791878 185.07635:136659 | -8.2319E-07 |
| POS11922 | 6(4Z,7Z,10Z,13Z,16 | 6.131 | 568.33942 | [M+CH3OH+H]+ | 568.34033 | 56.04953:11079 59.04858:26899 60.08025:262932 71.0726:47332 86.09541:749017 87.09918:21258 89.05896:15985 98.98203:14355 104.10696:143380 124.99955:252441 133.08543:14529 184.07466:3618798 185.07635:86007 | -1.6012E-06 |
| POS1185 | yl-a-aminoisobuty | 1.013 | 118.08609 | [M+H]+ | 118.0866 | 53.03773:43769 55.05365:2259418 56.04862:35861 57.05693:188243 58.06416:1108702 59.04858:40500 59.07198:1019120 70.0643:13165 71.06746:29623 72.08047:2998117 73.08444:9731 118.08494:671902 | -4.3189E-06 |
| POS1735 | Benzothiazole | 5.225 | 136.02126 | [M+H]+ | 136.02158 | 65.03805:7972 109.00957:24687 136.02158:109387 | -2.3526E-06 |
| POS1188 | D-Valine | 2.589 | 118.08617 | [M+H]+ | 118.08626 | 55.05363:412497 56.9423:10025 57.0569:33033 58.06509:10545 59.04856:11710 72.08044:483927 72.93703:7811 | -7.6215E-07 |
| POS5488 | mma-Glutamylleuc | 4.428 | 261.14288 | [M+H]+ | 261.14429 | 56.04952:47760 69.06991:56244 84.04315:1149629 85.02799:15357 85.04652:24617 86.05938:56530 86.0954:2495348 87.09917:64088 102.05498:30532 114.05461:34603 130.04884:207874 132.10173:511789 133.10521:15584 142.04747:64415 152.10725:34450 169.08443:25634 170.11852:16372 198.10959:74884 244.118:14015 | -5.3993E-06 |
| POS1120 | Proline | 1.038 | 116.0703 | [M+H]+ | 116.07058 | 52.41612:6575 54.56131:6288 56.04953:7301 56.55671:5899 58.06512:10102 59.07198:7702 68.0495:25256 70.0643:5008722 71.06874:10617 80.4635:5832 101.43783:6059 116.06905:173371 | -2.4123E-06 |
| POS2701 | nol-phenylpyruvat | 2.2 | 165.05429 | [M+H]+ | 165.05479 | 51.02237:6584 57.03287:7438 61.03972:6501 65.03805:19987 67.05389:14565 69.03294:14200 77.03802:40323 91.0539:159150 93.06841:8331 95.04894:567612 96.05188:7459 103.05422:24102 105.04402:33986 109.06337:24149 119.04707:243123 120.08031:7830 121.0641:7408 123.04193:323178 124.04758:6472 147.04404:25713 | -3.0293E-06 |
| POS2818 | Uric acid | 1.747 | 169.03532 | [M+H]+ | 169.03558 | 55.02822:192629 55.05365:10007 62.44093:5208 67.05389:13338 69.00832:71887 70.03912:370193 81.06833:14998 83.02332:8731 91.68036:5230 96.01751:146140 98.03504:216974 99.01799:24070 109.00224:8843 114.03108:5956 123.0302:7455 124.01198:48054 126.02935:290713 127.03194:7306 131.69183:5995 141.03847:532027 142.04385:5920 144.03883:5866 152.00659:160570 169.03252:387130 170.01851:46635 | -1.5381E-06 |
| POS8739 | Oleoylcarnitine | 5.6 | 426.35623 | [M+H]+ | 426.35779 | 55.05366:23248 57.03287:55325 57.06896:58528 59.04859:9346 60.08025:449291 67.05389:18680 69.06868:35283 71.08419:28706 81.06834:24527 83.0851:38575 85.028:2271848 85.10045:16305 86.03025:37104 93.07034:11432 95.08477:38242 97.09953:26196 107.08487:8926 109.10007:17784 111.11732:8733 121.1013:22005 135.11646:14656 144.10197:46088 149.13394:13348 173.43036:7350 | -3.6589E-06 |
| POS8577 | Nonaethylene glyco | 4.509 | 415.25272 | [M+H]+ | 415.25378 | 73.06432:16324 86.09541:11486 87.04334:57259 89.05896:974340 90.0619:14109 107.07059:8721 117.09089:6569 131.07088:8163 133.08543:338896 134.08684:10247 177.11285:27933 207.3625:5970 257.82501:6144 380.27573:5685 | -2.5527E-06 |

**Differences in metabolites between the WJW group, the Model group and the Control group**

| Alignment ID | Metabolite name | Rt(min) | Expreiment Mz | Adduct type | Reference m/z | MS/MS spectrum | PPM |
|---|---|---|---|---|---|---|---|
| POS7979 | yristoyllevocarnitir | 5.356 | 372.30908 | [M+H]+ | 372.31079 | 51.56357:6742 57.03287:15133 57.06896:18033 60.08025:185054 67.05389:6094 71.08547:16644 85.02799:971725 86.03024:16129 95.08477:8937 144.10197:17959 244.00337:6547 | -4.5929E-06 |
| POS5115 | N-Acetyltryptopha | 4.741 | 247.10725 | [M+H]+ | 247.10768 | 118.0629:26566 130.06477:64358 132.07892:48513 142.06567:9048 144.0797:20273 146.05721:74183 159.09138:159110 170.05661:15699 173.42545:8643 187.08794:9341 188.07004:58967 201.10141:15716 | -1.7401E-06 |
| POS8386 | .-Palmitoylcarnitin | 5.562 | 400.34106 | [M+H]+ | 400.34207 | 55.05366:9409 57.03287:67078 57.06896:102012 58.06512:12737 60.08025:814513 61.08379:10726 67.05389:14313 69.06868:21933 71.08547:66770 81.0699:39890 83.0851:33708 85.028:4222678 85.10045:39216 86.03025:64784 89.05896:8238 95.08477:49160 97.09953:14544 103.07444:7100 109.10007:31112 123.11524:16860 125.13172:6703 135.66116:6320 137.13222:10360 144.10197:90033 174.76732:6511 239.23662:17393 | -2.5228E-06 |
| POS10708 | telet-activating fac | 6.595 | 524.36841 | [M+H]+ | 524.37097 | 53.9196:8265 57.06989:10359 60.08025:51861 64.15609:7760 71.0726:8861 86.0954:182323 101.47952:7156 104.10696:45818 124.99955:59156 160.51437:7745 184.07465:693771 185.07634:14888 | -4.882E-06 |
| POS2119 | D-Lysine | 1.052 | 147.11206 | [M+H]+ | 147.1129 | 55.05365:21431 56.04862:311733 60.08025:127686 61.08378:10757 67.05389:41656 70.0643:13652 72.08047:8148 74.02328:13541 84.04316:1430594 84.07957:1365331 85.02799:28264 85.04652:15170 85.08359:10862 87.04334:174690 88.04782:11844 100.07382:9690 101.07021:11646 102.05499:25163 130.04884:137022 130.08389:54141 146.11787:20073 | -5.7099E-06 |
| POS4571 | cyclotetradecane- | 4.475 | 227.17447 | [M+H]+ | 227.17529 | 55.05365:298983 58.07272:6155 64.70701:6480 69.06992:11014 81.0699:6881 82.06343:5855 83.0851:96790 96.08018:20307 100.11039:1132751 101.11388:29135 109.10007:7135 110.10548:5919 112.84786:6464 114.09126:9909 117.13717:10401 139.08766:7866 209.16336:30335 227.17255:16373 | -3.6095E-06 |
| POS1673 | o-Tolyl isocyanate | 4.856 | 134.05936 | [M+H]+ | 134.06 | 77.03802:14145 78.04544:8286 79.05312:146398 79.23306:6396 87.09918:9982 88.02121:9864 95.04893:31499 104.04768:26491 105.03246:75496 106.06411:712941 107.06821:9029 120.69875:6478 133.05247:21390 134.06017:283645 | -4.774E-06 |
| POS2543 | 2721-59-7 | 3.357 | 160.0753 | [M+H]+ | 160.07561 | 52.64342:6544 55.0177:14037 55.05365:20891 55.93435:52322 56.94139:43783 57.06988:6318 65.03804:8303 67.04092:46275 67.05389:7590 69.06991:22072 71.04816:6935 72.08047:7876 72.93706:46772 74.95245:8165 79.05312:25691 90.94762:27540 91.05389:21823 94.04007:9009 95.04893:7258 96.08826:6989 103.05421:9639 105.06944:110273 107.04678:6672 113.9631:30824 114.09124:7417 115.05256:338584 116.97396:6430 117.05552:188538 118.06289:16734 130.06476:23657 131.04831:14488 131.07086:21686 131.97139:9239 132.0789:260377 132.97009:15510 133.06564:43156 142.06566:59329 143.07103:39725 159.06551:36453 160.07416:622196 161.07925:12631 162.91693:5901 | -1.9366E-06 |

**Differences in metabolites between the WJW group, the Model group and the Control group**

| Alignment ID | Metabolite name | Rt(min) | Expreiment Mz | Adduct type | Reference m/z | MS/MS spectrum | PPM |
|---|---|---|---|---|---|---|---|
| POS5704 | Inosine | 4.382 | 269.08707 | [M+H]+ | 269.08801 | 55.0177:37760 55.0291:33671 55.78193:7005 57.03287:64369 59.04859:7084 61.02846:9158 67.02914:10736 69.03294:15844 73.02812:24585 82.03949:29577 85.028:23898 94.04008:62874 110.03357:124679 115.03932:10195 119.0359:90606 133.04918:9042 137.04604:5792410 138.04726:214745 | -3.4933E-06 |
| POS4708 | Butyrylcarnitine | 4.389 | 232.15369 | [M+H]+ | 232.15421 | 57.03287:82378 58.06512:12276 60.08025:393758 71.04816:65210 72.08047:16666 84.07957:47991 85.028:3262740 86.03024:66696 86.09541:21691 126.09014:13972 144.10197:46354 173.07771:70441 232.15034:32028 | -2.2399E-06 |
| POS3508 | Acetyl-L-glutamic | 2.201 | 190.07033 | [M+H]+ | 190.07098 | 56.04861:6674 84.04314:94451 102.05497:10788 126.02325:5684 130.04883:35527 | -3.4198E-06 |
| POS1050 | Creatinine | 1.126 | 114.06573 | [M+H]+ | 114.06618 | 68.0495:10084 70.0643:10541 72.04369:19987 84.48338:6386 86.07139:35948 111.68296:5557 114.06509:147086 | -3.9451E-06 |
| POS9749 | yl-sn-glycero-3-p | 6.116 | 482.36169 | [M+H]+ | 482.36047 | 57.03286:28980 57.06895:63700 59.04858:22687 60.08024:71295 71.08547:41875 75.04311:10959 81.06833:9913 83.08509:8698 85.10044:24531 86.0954:111504 89.05895:12491 95.08476:8318 97.10158:6672 104.10696:1097444 105.10878:27839 109.10005:7273 124.99954:36928 173.43033:10886 184.07465:41126 | 2.52923E-06 |
| POS10032 | nitoylphosphatidyl | 5.109 | 496.33975 | [M+H]+ | 496.33978 | 57.06893:305017 58.06509:151177 60.08022:1846755 64.85479:109228 71.07257:252067 71.08544:138140 82.89832:114492 86.09536:4277546 95.08472:157403 104.10692:23310622 105.10873:612520 124.9995:934110 163.01515:138220 165.84062:115113 184.07457:11627109 185.07626:200921 308.44281:114198 | -6.0442E-08 |
| POS7339 | 13-Docosenamide | 8.427 | 338.34024 | [M+H]+ | 338.34167 | 55.0177:8204 55.05365:82802 57.03286:15973 57.06895:169688 58.06416:6364 67.05389:47175 69.06991:155934 71.04816:9116 71.08546:98556 72.08047:14585 73.52791:5775 79.05312:12140 81.06989:81173 83.08509:175609 85.10044:47719 86.05938:22227 93.0684:24825 95.08476:95549 97.0646:36675 97.09953:141619 100.07381:41903 107.08485:30694 109.10005:61223 111.07959:41175 111.1148:59469 114.09125:51521 121.10129:58797 123.11523:34047 125.09564:23458 125.13171:22326 128.10568:30349 135.11644:57188 137.12875:13189 139.11232:13424 142.12386:16195 149.13:41095 153.12764:6558 156.13799:12623 163.14493:14298 167.14174:7100 255.61653:6275 296.59183:5912 | -4.2265E-06 |
| POS5467 | -Hexanoylcarnitin | 4.523 | 260.18475 | [M+H]+ | 260.18558 | 57.03286:16321 60.08025:87561 71.08418:20259 85.02799:751638 86.03195:13108 99.07938:16217 144.10197:9767 201.11363:7309 | -3.19E-06 |
| POS8575 | Gelomulide N | 5.751 | 415.2103 | [M-H2O+H]+ | 415.211 | 65.03805:7164 69.03294:8960 77.03802:6531 79.05313:6739 81.03386:6738 91.0539:42167 91.11925:7534 104.06136:7053 105.06946:7622 107.08487:11777 117.06913:11433 119.08334:2012474 120.08878:79446 133.06236:23733 135.07936:19701 | -1.6859E-06 |

**Differences in metabolites between the WJW group, the Model group and the Control group**

| Alignment ID | Metabolite name | Rt(min) | Expreiment Mz | Adduct type | Reference m/z | MS/MS spectrum | PPM |
|---|---|---|---|---|---|---|---|
| POS7458 | ıramidopropylbeta | 5.351 | 343.29492 | [M]+ | 343.2955 | 57.06895:68496 58.06416:29621 71.08418:38504 76.07277:6028 81.06989:9508 83.08508:10148 85.10044:20196 95.08476:31625 109.10005:30042 123.11523:7812 155.90779:5375 173.43033:7572 183.17154:29559 240.23306:237476 241.23578:19036 | -1.6895E-06 |
| POS6062 | Oleamide | 8.825 | 282.27826 | [M+H]+ | 282.27908 | 51.70781:6117 55.05365:55737 57.03287:7605 57.06896:66412 57.61167:6213 67.05389:18045 69.06868:96769 71.08547:32424 81.0699:32367 83.08509:53084 85.10045:10326 86.05939:7345 86.2397:5472 93.06841:11368 95.08477:26417 97.06461:14262 97.09953:33756 100.07597:13617 107.08486:22620 109.10006:20293 111.0796:10966 111.11481:20975 114.08864:10737 121.1013:10895 123.11523:8425 135.11646:11592 149.13002:6796 | -2.9049E-06 |
| POS5032 | -Undecanoylglycir | 5.123 | 244.18991 | [M+H]+ | 244.19051 | 55.01682:31910 55.05365:249817 56.04862:22095 57.03286:23057 57.06895:138638 58.06416:22317 59.04858:6940 67.05389:128244 69.06867:175357 70.0643:10450 71.04816:25624 71.08418:61457 72.08047:27603 79.05312:63333 81.06833:253740 82.06342:11569 83.04932:10233 83.08508:255996 84.07957:14624 85.06336:23497 85.10044:9918 86.05938:35246 86.0954:13572 91.05389:14000 93.0684:96198 95.08476:202887 96.08018:17514 97.0646:54115 97.09953:140084 98.09552:13043 99.07938:11830 100.07381:44311 100.11037:11172 102.05498:12926 105.06944:10460 107.08485:147261 108.08662:8437 109.10005:39996 110.09555:34732 111.07959:47528 114.09124:36105 119.08332:7507 121.10129:108901 123.11523:22668 124.1099:51772 125.09564:30075 128.10568:13750 131.08376:34733 135.11644:27705 137.13219:8729 138.1274:37965 142.12386:7647 145.10278:5768 147.11681:7541 149.13:6646 156.17151:10797 163.14493:117767 164.15204:7861 167.74667:5342 173.1315:30440 180.17407:189833 181.15976:27629 190.15889:7151 198.18146:15198 226.17574:87363 | -2.4571E-06 |
| POS4651 | Lauramine oxide | 5.036 | 230.24738 | [M+H]+ | 230.2476 | 55.05365:16217 57.06896:495944 58.06416:66882 58.07177:10942 62.05909:265085 69.06991:8703 71.08418:155775 85.10044:44003 93.06841:10097 167.10463:8934 173.39601:7315 212.23726:40352 230.24779:246622 231.24898:15788 | -9.5549E-07 |
| POS3648 | Phenylacetylglycine | 4.656 | 194.08057 | [M+H]+ | 194.08118 | 53.29583:5855 65.03804:5998 76.03858:359475 91.05389:813066 92.05668:25266 120.0803:7288 135.04227:8015 177.25467:5430 | -3.143E-06 |
| POS2040 | -Methyleneglutara | 9.911 | 145.04929 | [M+H]+ | 145.04939 | 53.03774:301799 55.0177:188558 55.05366:29937 56.04953:11584 56.9414:7899 57.03287:138126 57.06989:6914 59.04858:13138 67.01736:6858 69.03294:30235 70.06431:62151 71.01216:17320 71.04816:385264 72.93707:11418 81.0323:93678 81.0699:14753 84.99097:13324 85.028:42224 98.06007:25728 98.09553:25813 99.0434:540241 101.02222:10793 127.0381:52379 145.04651:18737 | -6.8942E-07 |

**Differences in metabolites between the WJW group, the Model group and the Control group**

| Alignment ID | Metabolite name | Rt(min) | Expreiment Mz | Adduct type | Reference m/z | MS/MS spectrum | PPM |
|---|---|---|---|---|---|---|---|
| POS10710 | enantio-PAF C-16 | 5.717 | 524.36853 | [M+H]+ | 524.37018 | 60.08023:659119 71.0713:114821 86.09538:2224914 99.50859:76156 104.10693:924207 105.65473:68598 124.99951:644511 150.57184:80642 173.4303:120925 180.49649:74015 184.0746:13549719 185.07629:272102 235.66122:75223 | -3.1466E-06 |
| POS3510 | ole-3-methyl acet | 5.322 | 190.08531 | [M+H]+ | 190.08627 | 69.89706:5812 103.05418:6452 130.06473:289564 131.06761:12461 | -5.0503E-06 |
| POS6792 | nma-Glutamyltyro | 4.376 | 311.12238 | [M+H]+ | 311.1235 | 55.42663:5924 55.52425:6174 70.06431:37661 84.04317:139861 84.07958:22231 91.0539:11376 95.04894:7660 102.05277:7646 119.04707:56794 120.08031:10712 123.04193:85722 130.04886:25579 130.08708:7867 136.07611:194826 137.07704:12027 147.04404:37702 161.81059:5585 163.28825:5993 165.05461:131356 180.08583:8347 182.0797:35967 202.08495:11454 | -3.5999E-06 |
| POS10588 | oPC(0:0/18:2(9Z,12 | 6.252 | 520.33984 | [M+H]+ | 520.33929 | 56.04953:104351 58.06512:138990 60.08025:2771459 67.05389:276732 69.06868:175855 71.07261:293752 81.06834:329192 86.09541:6767502 87.09918:146255 95.08477:191886 104.10697:35008200 105.10879:669086 107.08487:89972 109.10007:124939 124.99956:1800818 163.01524:161429 173.39603:106986 181.02371:83013 184.07466:32080096 185.07635:720869 258.10977:180580 | 1.057E-06 |
| POS1054 | eridinecarboxalde | 4.523 | 114.09103 | [M+H]+ | 114.09189 | 53.03773:22129 54.03373:19684 55.0177:275908 55.05365:720196 56.04952:14814 58.02807:28138 58.06511:13280 65.03804:9965 67.05389:191692 68.04829:166504 69.03294:17596 69.06868:1196464 70.0643:116677 71.04816:59570 71.08547:74695 72.08047:136179 77.03802:45747 79.05312:1238748 81.05579:32851 84.07957:26762 86.05938:14861 86.0954:96980 91.05576:9702 95.04893:72789 96.08018:613010 97.0646:185251 105.04401:16672 114.09125:4150725 115.09233:15965 | -7.5378E-06 |
| POS1831 | Urocanic acid | 1.535 | 139.04982 | [M+H]+ | 139.05019 | 55.0177:9414 66.03385:11141 67.05389:7759 68.04949:6381 68.99723:18717 82.0746:5494 93.04333:35198 94.06358:6205 95.05888:8466 112.03867:5948 121.03835:36538 139.0489:13188 | -2.6609E-06 |
| POS3462 | ctyloxy)propan-1-a | 4.786 | 188.19987 | [M+H]+ | 188.2009 | 55.05364:8123 57.06987:499831 58.06511:847825 59.04857:20349 67.88462:6390 69.0699:7033 71.08546:249705 72.08835:5825 76.07561:347996 118.06287:19451 123.48865:5416 146.06096:11333 188.19746:7647 | -5.4729E-06 |
| POS1021 | Uracil | 1.557 | 113.03455 | [M+H]+ | 113.03458 | 67.0539:6326 69.04404:15747 70.0278:42926 96.00742:15749 113.03346:21848 | -2.6541E-07 |
| POS10030 | LysoPC(0:0/16:0) | 6.521 | 496.33655 | [M+H]+ | 496.3403 | 57.06896:6797 58.06607:7574 60.08025:92779 71.07261:9771 86.09541:301558 98.98415:8214 104.10697:91629 124.99956:81893 184.07466:2157294 185.07635:54194 | -7.5553E-06 |
| POS9747 | LysoPC(15:0/0:0) | 6.25 | 482.32437 | [M+H]+ | 482.32407 | 57.06989:8019 59.04858:8869 60.08025:54297 72.65694:7997 82.89674:6261 86.09541:112673 100.42736:5892 104.10696:681901 105.10879:16336 111.15255:5910 124.99955:23582 173.43034:7038 182.06386:7149 184.07466:284068 185.07635:9479 | 6.21988E-07 |

**Differences in metabolites between the WJW group, the Model group and the Control group**

| Alignment ID | Metabolite name | Rt(min) | Expreiment Mz | Adduct type | Reference m/z | MS/MS spectrum | PPM |
|---|---|---|---|---|---|---|---|
| POS6642 | methyl-dodecylan | 5.336 | 304.29819 | [M]+ | 304.2999 | 50.02972:7158 58.06416:92108 62.08849:6043 91.05389:147827 173.4254:6580 212.23723:15942 | -5.6195E-06 |
| POS11290 | PC(20:3(8Z,11Z,14Z | 6.473 | 546.35565 | [M+Na]+ | 546.35596 | 50.35427:5837 60.08022:15840 62.15261:4989 86.09536:55689 104.10692:20699 107.79275:5589 124.9995:12059 146.98271:13095 184.07457:167098 185.07626:7511 343.73755:6496 | -5.674E-07 |
| POS1545 | 2E)-Decenoyl-ACI | 1.07 | 130.08577 | [M+H]+ | 130.0864 | 56.04862:118858 58.06511:7521 67.05389:8363 70.0643:49894 83.05907:18019 84.04315:299249 84.07957:499412 85.02799:12389 86.05938:19031 130.04883:13762 130.08388:25923 | -4.8429E-06 |
| POS3045 | Indoleacetic acid | 4.908 | 176.07011 | [M+H]+ | 176.07001 | 103.05418:12802 112.7732:5731 130.06473:203262 | 5.67956E-07 |
| POS986 | Cytosine | 1.59 | 112.05036 | [M+H]+ | 112.05058 | 55.05365:13088 56.04862:9977 67.02913:10786 69.04403:32435 94.04007:8199 95.02306:57115 112.04886:82686 | -1.9634E-06 |
| POS630 | rolidinecarboxalde | 4.404 | 100.07545 | [M+H]+ | 100.07624 | 53.03773:15572 55.0177:33096 55.05365:79980 56.04862:75630 57.06896:14117 58.02807:34226 58.06512:9289 59.04858:12863 62.80278:6011 69.03294:9240 72.04369:11810 72.08047:11866 79.76434:5267 82.06503:14023 83.01195:6788 84.06467:6030 91.50716:5661 97.81029:5782 98.98415:8797 100.03944:6290 100.07382:172510 | -7.894E-06 |
| POS101 | 1,3,5-Hexatriene | 0.646 | 81.07 | [M+H]+ | 81.06988 | 53.03773:68020 55.0177:9358 55.05365:6707 55.92267:5853 56.96447:19258 65.03804:8525 66.04538:8879 67.50902:13644 79.05312:86752 80.04813:8423 81.04482:7777 81.06833:89005 | 1.4802E-06 |
| POS10358 | LysoPC(O-18:0/0:0 | 6.057 | 510.38947 | [M+H]+ | 510.39178 | 60.08025:20922 66.76708:5146 70.14877:6543 71.08547:5711 85.028:17314 86.09541:33705 104.10696:321132 105.10879:12888 124.99955:10268 184.06929:22176 297.80222:6396 | -4.5259E-06 |
| POS1539 | Isoquinoline | 5.322 | 130.06502 | [M+H]+ | 130.0654 | 55.05451:10840 56.0486:14816 67.05387:6220 77.03799:5908 84.04313:22135 84.07954:42714 95.0489:12653 103.05418:29486 105.04398:6228 130.06473:96836 | -2.9216E-06 |
| POS1967 | Kojic acid | 4.376 | 143.03334 | [M+H]+ | 143.03329 | 55.01768:35985 55.05363:31541 56.94137:14899 59.04856:6648 62.4123:6113 69.03291:59530 70.06427:12391 71.04813:16095 72.08044:6174 72.93703:11363 97.0276:9883 98.06002:6243 98.09548:5300 143.03423:122999 | 3.49569E-07 |
| POS6089 | adecadiene-1,18- | 0.723 | 283.26205 | [M+H]+ | 283.263 | 55.05365:51287 57.06895:74067 61.03869:14029 67.05389:17077 69.06867:58545 71.08546:26828 81.06832:30109 83.08508:26890 85.10044:10909 93.0684:11862 95.08475:26486 96.94559:5643 97.0646:9364 97.09952:16086 107.08485:12356 109.10005:7087 111.11479:7947 121.10128:11040 135.11642:11242 149.13:6480 171.68271:6646 | -3.3538E-06 |
| POS6212 | Octanoylcarnitine | 4.73 | 288.21643 | [M+H]+ | 288.21689 | 54.70615:6900 57.06896:15297 60.08025:41909 75.81551:5321 77.89019:6069 85.02799:316477 102.05719:7244 127.10885:8097 144.10197:9062 | -1.596E-06 |
| POS9770 | ctapropylene glyc | 5.388 | 483.35306 | [M+H]+ | 483.35278 | 57.03287:73776 59.04858:840745 60.05225:12790 60.13429:6466 64.94802:6318 70.09454:7101 76.96691:6548 83.1958:6334 87.04334:10053 89.05896:20193 99.07938:8483 115.07378:9116 117.09089:109783 175.1301:13269 198.19347:5897 305.05081:6387 325.36108:5759 336.14642:6711 | 5.79287E-07 |

**Differences in metabolites between the WJW group, the Model group and the Control group**

| Alignment ID | Metabolite name | Rt(min) | Expreiment Mz | Adduct type | Reference m/z | MS/MS spectrum | PPM |
|---|---|---|---|---|---|---|---|
| POS4456 | Tetraglyme | 4.517 | 223.15384 | [M+H]+ | 223.15398 | 57.03286:17644 59.04858:719164 61.02742:8630 73.02676:7417 84.0448:8180 87.04333:15449 89.05894:137329 90.16662:6172 93.0684:12612 103.07442:34124 117.05279:24353 | -6.2737E-07 |
| POS1177 | Indole | 4.441 | 118.06494 | [M+H]+ | 118.0653 | 53.03857:8410 55.05365:361485 57.05693:29432 58.06512:13322 59.04858:11113 59.07296:9243 70.06557:6328 72.04501:10182 72.08048:568553 77.03802:6876 84.00181:6311 91.0539:163299 95.04894:23475 105.04401:7866 117.05553:22955 118.0629:214646 | -3.0492E-06 |
| POS5283 | octadienyl ester 4- | 5.89 | 253.21492 | [M+H]+ | 253.2157 | 55.05366:44772 57.06896:14797 64.38515:5835 67.05389:25247 69.06869:39445 79.05313:6614 81.06834:26705 83.0851:16776 93.07034:10381 95.08478:27456 97.06461:7369 97.10159:8873 107.08487:10864 109.10007:10862 112.12533:5510 119.08334:10334 121.09844:7845 133.10193:7679 147.11682:6121 | -3.0804E-06 |
| POS9476 | LysoPC(14:0/0:0) | 5.948 | 468.30829 | [M+H]+ | 468.30847 | 57.03284:142001 57.06893:45390 58.03659:30210 59.04856:38273 60.08022:255437 71.08544:26823 86.09536:548739 87.09914:17012 95.08472:17725 104.10692:2605018 105.10873:68251 124.9995:104966 184.07457:971578 185.07626:30284 348.94217:16614 | -3.8436E-07 |
| POS1748 | 2-Phenylacetamide | 2.199 | 136.07529 | [M+H]+ | 136.0757 | 55.93345:61193 56.94233:8625 65.03805:41428 67.05389:6790 71.92827:16470 72.93707:55150 79.05313:8527 81.0699:20341 89.93906:9812 90.94764:24493 91.0539:664279 94.04008:7175 107.04918:41799 109.06581:10356 118.06566:13408 119.04707:61620 119.80384:6310 136.02159:13051 136.05907:20846 137.04604:7569 | -3.013E-06 |
| POS7477 | Lauroylcarnitine | 5.14 | 344.27841 | [M+H]+ | 344.27948 | 57.03287:14438 57.06896:18743 59.04858:78071 60.08025:121773 67.05389:10990 69.08101:8178 71.08547:7833 79.05463:9091 81.06833:11027 83.08509:7217 85.028:749086 86.03196:11393 86.61588:6665 88.03895:6369 89.05896:24823 93.06841:16163 95.08477:12929 103.07444:8760 107.08486:11118 109.10006:11058 135.07935:7396 144.10197:12672 163.14494:7803 183.17155:10126 | -3.1079E-06 |
| POS1238 | Isoindoline | 3.962 | 120.08053 | [M+NH4]+ | 120.08077 | 55.05366:9682 56.05764:7025 56.9414:19400 59.60601:5756 61.0387:28501 72.93573:19823 73.08311:14147 77.03802:38079 90.94764:8302 91.0539:63293 93.06841:61439 95.04894:121825 102.04613:6800 103.05422:433237 105.04402:28614 118.0629:7220 120.08031:292394 | -1.9987E-06 |
| POS1440 | -2H-pyran-3-carb | 0.182 | 127.03897 | [M+H]+ | 127.03952 | 50.26004:5651 53.03856:115390 53.93916:7725 55.0177:130637 55.05365:26134 56.04862:10501 56.96447:8503 57.03286:22978 67.05389:9915 70.06556:12345 71.04816:43129 79.05312:8435 80.04813:22549 81.03228:31319 81.06832:17377 82.06502:9874 84.04315:7402 84.95899:8129 99.04338:9133 108.04319:9438 109.02666:25826 127.03809:287876 | -4.3294E-06 |

**Differences in metabolites between the WJW group, the Model group and the Control group**

| Alignment ID | Metabolite name | Rt(min) | Expreiment Mz | Adduct type | Reference m/z | MS/MS spectrum | PPM |
|---|---|---|---|---|---|---|---|
| POS1537 | Indole-3-carbinol | 4.423 | 130.06456 | [M+H-H2O]+ | 130.0647 | 54.64017:6537 55.05366:26758 56.04863:30718 57.03287:8398 57.06896:80411 67.05389:15137 74.09583:23880 77.03802:23987 84.04317:38555 84.07957:69391 87.00325:14012 95.04894:33089 103.05422:81566 104.46147:6462 105.04402:6683 107.84089:5992 113.96312:8532 128.04967:9327 129.99472:6496 130.06477:306382 130.15721:27895 132.94377:5972 | -1.0764E-06 |
| POS2016 | cyclohexanecarbo | 1.135 | 144.1013 | [M+H]+ | 144.10181 | 53.03773:16288 55.0177:23838 55.05365:24757 56.04952:14732 58.06416:96575 61.03869:14347 69.03294:9637 70.06429:72063 71.04815:19953 72.08047:12728 81.06832:10240 84.04315:21596 84.07957:63766 98.06005:49924 98.09551:32076 99.04338:17761 102.05497:7220 112.20184:5816 125.72906:6288 126.09013:10892 143.03059:7747 144.10196:150958 | -3.5392E-06 |
| POS1628 | DL-Norleucine | 1.255 | 132.10962 | [M+H]+ | 132.11 | 55.0177:12744 55.05365:12696 56.04952:17870 57.05692:42131 58.06416:46069 61.01003:9893 69.03417:12424 69.06867:466009 72.04369:7878 73.06432:9978 86.0954:2907210 87.09917:12270 90.05456:82747 132.07564:17170 132.10173:22504 | -2.8764E-06 |
| POS5544 | Phenylalanylproline | 4.435 | 263.13757 | [M+H]+ | 263.13898 | 70.06431:91480 84.04317:22235 85.04652:13408 86.09541:49676 87.09918:29051 116.06905:174861 120.08031:205804 121.08413:11874 123.11524:9314 132.10175:5500 133.10522:7109 | -5.3584E-06 |
| POS211 | Cyclopentylamine | 2.203 | 86.09637 | [M+H]+ | 86.09643 | 50.39038:12062 56.04863:92328 57.05693:146946 58.06512:20445 67.05389:15821 69.06868:112689 86.05939:66632 86.09541:77074 | -6.9689E-07 |
| POS1475 | L-Baikiain | 1.179 | 128.07036 | [M+H]+ | 128.0708 | 53.03856:21195 54.03372:11950 55.0177:17257 55.05453:94553 56.04952:7843 57.04489:12639 58.06511:13196 62.47697:5464 67.0421:17684 70.06556:9392 71.04944:9132 72.58782:6505 82.06502:127867 86.05938:6760 100.07597:11830 110.05835:9058 127.03809:17037 128.07144:17484 | -3.4356E-06 |
| POS11231 | (20:4(8Z,11Z,14Z,1 | 6.192 | 544.33997 | [M+H]+ | 544.34033 | 60.08023:744682 86.09538:1786256 94.47636:236946 104.10693:15225237 124.99951:360103 126.55663:278578 184.0746:7584514 226.83458:285865 261.237:259828 341.89731:242410 381.40887:324009 531.4198:284034 | -6.6135E-07 |
| POS11420 | yl)-sn-glycero-3-[ | 6.402 | 550.38617 | [M+NH4]+ | 550.38672 | 60.08024:15426 86.0954:27092 104.10696:144095 105.10878:7529 124.99954:7469 184.07465:57400 | -9.993E-07 |
| POS6592 | -Aminooctadecane | 5.507 | 302.30365 | [M+H]+ | 302.30539 | 55.05365:26947 56.04862:6381 57.06896:15481 60.04425:231657 67.05389:30758 69.06868:25997 70.0643:9173 71.08547:6605 81.06989:26805 83.08509:14741 88.07444:7951 95.08476:27072 97.10158:11068 109.10005:11294 120.03226:5724 165.78566:5174 168.79677:5089 183.07042:7081 298.44339:6124 | -5.7558E-06 |
| POS6956 | Phytosphingosine | 5.034 | 318.29956 | [M+H]+ | 318.2999 | 55.05365:10813 57.06896:194439 58.06417:28443 69.06992:9206 70.06431:246436 71.08419:70457 72.08048:10266 84.07957:5835 85.10045:16514 86.09541:6291 88.07445:437164 95.08477:6332 102.09042:259378 132.10175:18678 146.11787:10681 150.11266:5960 256.25848:170371 257.26578:9467 318.29404:57728 | -1.0682E-06 |

**Differences in metabolites between the WJW group, the Model group and the Control group**

| Alignment ID | Metabolite name | Rt(min) | Expreiment Mz | Adduct type | Reference m/z | MS/MS spectrum | PPM |
|---|---|---|---|---|---|---|---|
| POS667 | -5-methyl-2(3H)-f | 0.659 | 101.05946 | [M+H]+ | 101.06026 | 53.03773:8754 55.0177:20315 55.05365:102916 56.04861:7906 57.06988:6898 59.04858:34741 60.04425:6534 72.04368:6808 83.04768:6817 100.07381:13531 | -7.9161E-06 |
| POS1743 | Adenine | 8.871 | 136.06134 | [M+H]+ | 136.0619 | 76.50495:5886 81.0699:10792 91.05389:60378 94.04008:8470 118.0629:7499 119.0359:17263 136.02158:20705 136.05905:64464 137.04602:8767 | -4.1158E-06 |
| POS10162 | (20:4(8Z,11Z,14Z,1 | 6.116 | 502.29059 | [M+H]+ | 502.2926 | 55.01769:23744 55.05364:63581 57.03286:67470 57.06895:32465 61.71925:6370 62.06013:14088 67.05388:168157 69.06866:79820 71.04815:6325 71.08418:14996 73.02811:11585 75.04311:7844 77.03801:6084 79.05311:98005 81.06831:127336 83.04768:6810 83.08508:26961 85.06335:24850 85.10043:7537 91.05389:118244 93.06839:110439 95.04892:7717 95.08475:122202 97.06459:31760 97.10156:10780 105.06944:84821 107.08484:69844 109.10004:38606 117.06911:40537 119.08331:103327 121.10127:61898 123.08001:13187 123.11521:9795 129.05444:51851 131.08374:35621 133.0986:52981 135.07933:6474 135.11642:19200 137.13217:8902 143.08571:12809 145.09901:19486 147.11679:20481 149.12999:8337 157.09772:19947 159.1172:6413 161.13194:9723 171.11403:13829 175.145:6167 203.17519:26631 210.12161:6896 240.12112:5812 | -4.0017E-06 |
| POS9474 | yl-sn-glycero-3-p | 5.96 | 468.3049 | [M+H]+ | 468.3078 | 57.03287:56976 57.06896:50087 58.06512:15779 59.04859:57555 60.08025:282939 67.05389:10296 71.07261:27548 71.08548:30422 73.02812:11311 81.0699:14310 85.10046:11116 86.09541:607746 89.05896:29623 95.08478:18964 97.67747:9594 101.05931:14140 104.10697:3224918 105.10879:76323 109.10007:10339 115.07378:8659 124.99956:154054 144.00543:8513 163.01524:17084 184.07466:1604689 185.07635:49195 285.23489:8885 348.21555:9886 | -6.1925E-06 |
| POS2275 | Guanine | 4.375 | 152.05612 | [M+H]+ | 152.05659 | 56.04953:6057 67.05389:8330 79.05312:6622 79.78424:6180 96.08018:5419 107.04917:9919 109.05113:5482 110.03356:28966 110.06084:6528 121.03835:5855 135.02879:17398 152.0549:30094 153.03816:28975 | -3.091E-06 |
| POS6299 | Inosine | 4.194 | 291.06769 | [M+Na]+ | 291.07001 | 103.31759:5482 138.38226:5620 159.02675:44285 | -7.9706E-06 |

**Differences in metabolites between the WJW group, the Model group and the Control group**

| Alignment ID | Metabolite name | Rt(min) | Expreiment Mz | Adduct type | Reference m/z | MS/MS spectrum | PPM |
|---|---|---|---|---|---|---|---|
| POS7731 | racosahexaenoic a | 5.942 | 357.2774 | [M+H]+ | 357.27859 | 55.05365:82340 57.06896:8984 62.52579:6246 67.05389:76133 69.06992:33518 79.05312:27449 81.0699:207516 82.07301:8125 83.04932:10956 83.08509:94266 85.06337:25760 91.0539:24546 93.06841:117068 95.08477:229277 96.08827:6720 99.04339:41540 101.0593:51827 105.06945:86914 107.08486:163615 109.10006:140914 115.07378:9366 119.08333:90308 121.1013:142247 123.08004:8885 123.11523:34384 131.08377:22886 133.09863:107850 135.11646:174219 137.09428:11864 145.09904:32009 147.11681:123491 149.13002:90343 150.13635:7744 151.10901:11390 159.11723:36301 161.13197:136473 162.13377:8170 163.11809:15359 163.14494:41743 167.10463:17815 173.13152:22906 175.14503:66743 177.12804:15544 179.10373:8325 181.12314:20871 187.14839:15478 189.166:24644 193.12155:9220 195.13722:18504 201.16266:44090 207.13823:11848 215.17596:30307 221.1546:10218 229.20091:12362 | -3.3307E-06 |
| POS2630 | -Hydroxychromon | 5.279 | 163.03874 | [M+H]+ | 163.039 | 56.07296:6261 57.974:7314 77.03802:18561 79.05312:11146 92.02633:7193 95.04893:39622 105.04401:13257 133.02939:17424 135.04227:13756 163.03757:33028 | -1.5947E-06 |
| POS4227 | Tetradecylamine | 5.134 | 214.25204 | [M+H]+ | 214.25288 | 57.06989:31070 71.08547:7581 81.71648:5671 192.96027:5735 214.25024:9807 | -3.9206E-06 |
| POS3477 | N-Acetylglutamine | 1.584 | 189.0865 | [M+H]+ | 189.08698 | 55.93435:12320 56.04862:10246 60.04426:12610 61.03972:5803 72.93707:6110 83.05907:8824 84.04316:249315 84.07957:88188 101.07021:7509 102.05499:7050 105.53591:5286 126.09014:23861 129.88974:5816 130.04884:153460 173.39601:7986 | -2.5385E-06 |
| POS1335 | Nicotinic acid | 2.61 | 124.03905 | [M+H]+ | 124.03928 | 53.03856:17751 78.03358:12060 80.04813:95435 83.04768:6157 95.04693:8071 96.04377:52175 112.03866:13916 123.05363:35771 124.03866:11491 | -1.8543E-06 |
| POS1232 | Hydroxybenzonitr | 4.466 | 120.04428 | [M+H]+ | 120.0447 | 56.04953:7283 56.05674:11034 59.04956:10983 61.03869:30175 61.92805:6124 65.03804:142129 72.08047:7094 73.0831:14251 73.53333:6277 77.03802:14026 91.0539:30732 92.0491:98533 93.06841:16735 95.04893:27270 103.05421:118034 110.06084:7522 120.04357:20188 120.0803:65183 | -3.4987E-06 |
| POS2555 | iino valeric acid be | 1.373 | 160.1326 | [M+H]+ | 160.13318 | 55.05365:321601 58.06512:7390 59.04858:53601 60.08025:180335 64.59314:6062 70.06557:14093 83.04932:34086 101.0593:79347 116.04757:13912 132.07892:6734 160.07417:6735 160.13509:16309 | -3.622E-06 |
| POS3580 | ydroxyindoleacetic | 2.976 | 192.06497 | [M+H]+ | 192.06599 | 57.31232:5637 146.05719:13051 | -5.3107E-06 |
| POS7939 | -Tetradecenoylcar | 5.229 | 370.29395 | [M+NH4]+ | 370.29575 | 55.05365:12123 57.03286:31568 57.06989:16170 59.04858:7729 60.08025:297746 67.05389:21597 69.06868:27238 81.06989:15191 83.08509:14885 85.02799:1552989 86.03024:22780 89.05895:7308 93.06841:11323 95.08476:17975 97.09953:10649 103.03848:7755 107.08485:12694 109.10006:25355 121.10129:28425 123.11523:8170 135.11644:19502 144.10197:31085 191.17667:11347 209.18933:10597 | -4.861E-06 |

**Differences in metabolites between the WJW group, the Model group and the Control group**

| Alignment ID | Metabolite name | Rt(min) | Expreiment Mz | Adduct type | Reference m/z | MS/MS spectrum | PPM |
|---|---|---|---|---|---|---|---|
| POS5679 | Adenosine | 3.861 | 268.10361 | [M+H]+ | 268.10397 | 55.0177:8560 55.05365:6074 57.03287:16050 84.84476:5419 136.05907:711106 137.04602:243689 180.67375:6017 | -1.3428E-06 |
| POS4408 | ıydroxy-L-tryptop | 3.106 | 221.09062 | [M+H]+ | 221.0918 | 55.93435:7421 58.06511:9653 74.00551:5496 79.55973:6350 89.96104:8886 102.58624:6205 116.97397:6505 130.06477:35503 132.04306:12425 157.0766:14875 158.05783:16085 175.08531:10770 | -5.3371E-06 |
| POS2089 | Spermidine | 0.914 | 146.16499 | [M+H]+ | 146.16518 | 56.04953:17737 58.06512:12252 67.0539:7871 72.08048:77083 73.6934:6841 79.67875:5981 84.04482:6788 84.08124:191994 112.11259:10999 | -1.2999E-06 |
| POS1431 | a-4,5-cyclopropyl- | 1.204 | 127.03858 | [M+H]+ | 127.0389 | 53.03773:67161 55.0177:76953 55.05365:16891 55.21807:6225 57.03286:15598 57.06896:6209 67.05389:7619 71.04816:11899 78.9069:5679 80.04814:13044 81.03229:20049 81.04325:8220 81.06989:6367 82.06503:15826 108.04319:6029 109.02667:13298 111.38697:4780 127.03809:65632 | -2.5189E-06 |
| POS12449 | 33,36-Dodecaoxa | 4.547 | 591.35876 | [M+H]+ | 591.35858 | 73.06432:125073 87.04334:743459 89.05896:5522730 90.0619:104323 117.09089:82210 130.08708:36141 131.06766:189581 133.08543:2375642 134.08684:68181 175.09526:47103 177.11285:303644 | 3.04384E-07 |
| POS11255 | asterone 22-O-su | 4.584 | 545.31409 | [M+2H]2+ | 545.31421 | 67.05389:29798 69.03294:42084 73.02811:194322 73.06432:63047 81.03229:54889 83.04932:51834 87.04334:399634 89.05896:2005170 90.0619:67636 91.04643:47910 95.04893:140429 99.04339:71219 107.07058:39913 108.77773:25939 109.02668:26574 109.06336:35887 111.04189:185371 113.05927:53829 131.07088:53610 133.08543:599344 134.08684:38149 137.0598:75205 155.06668:55882 177.11285:59448 274.02045:23581 311.28952:23464 | -2.2006E-07 |
| POS11312 | | 4.546 | 547.32977 | [M+H]+ | 547.32977 | 73.06432:77080 87.04334:310689 89.05896:3034282 90.0619:56261 91.07443:15872 107.06821:26790 117.09089:29974 131.07088:55766 133.08543:1045644 134.08684:29346 175.09526:16421 177.11285:105917 | 0 |
| POS8525 | ɔl 3-beta-D-gluco | 5.159 | 411.27438 | [M+2H]2+ | 411.27451 | 57.03287:106701 59.04859:2423900 60.05226:58860 65.04819:49767 69.06868:24429 73.02812:203056 73.06432:45534 80.0543:28441 81.0699:10650 83.04932:11338 85.06337:50911 87.04335:255244 87.06254:93632 87.07999:40629 89.05896:1724694 90.06191:50889 94.06947:59301 101.05931:186742 102.06606:20714 103.07444:447298 104.0796:11406 107.07059:15977 109.0756:40860 115.07378:18653 116.08248:24210 117.05281:16563 117.0909:23711 129.08911:29662 131.06766:24305 133.08545:471122 134.08684:21539 143.1078:10447 145.08403:14879 147.10149:192488 161.11441:12769 177.11285:35988 191.1256:28252 | -3.1609E-07 |
| POS10377 | CHEBI:68981 | 4.652 | 511.30283 | [M+H]+ | 511.30289 | 69.03294:54910 73.02811:290002 73.06432:126431 80.05429:70564 81.03229:42121 87.04334:557306 89.05896:2868972 90.0619:147150 91.04643:91641 92.64947:36836 95.04893:132782 97.53043:33536 99.04339:71888 102.06606:61822 104.827:36803 111.04441:53212 113.05927:114186 131.06766:70072 133.08543:985809 134.08684:71800 137.05637:55345 177.11285:107996 288.22748:36591 479.31699:35690 | -1.1735E-07 |

**Differences in metabolites between the WJW group, the Model group and the Control group**

| Alignment ID | Metabolite name | Rt(min) | Expreiment Mz | Adduct type | Reference m/z | MS/MS spectrum | PPM |
|---|---|---|---|---|---|---|---|
| POS8071 | Ile Val Phe | 5.563 | 378.23935 | [M+H]+ | 378.2392 | 53.43733:6285 55.05365:90186 56.04863:21368 57.06896:23453 67.04092:11340 67.05389:134907 69.06868:63033 70.06431:61804 75.76308:6507 79.05312:83881 80.04814:33789 81.06834:101400 82.06343:365326 83.06721:12639 83.08509:32106 84.07957:8502 91.0539:54314 93.06841:110549 94.06359:41593 95.04893:5641 95.08477:93567 96.08018:77879 97.10159:9417 98.98415:8204 105.06945:31175 107.08486:55798 108.07938:16346 109.10007:42081 110.09557:10276 119.08333:18474 119.29852:6083 121.1013:43555 123.11523:9738 124.10991:6794 133.09863:18013 135.11646:16218 138.67992:6402 147.11681:12248 149.13002:16616 161.13197:8614 166.16684:5424 196.27794:6256 208.3924:6424 262.25568:184124 263.25272:19304 | 3.96574E-07 |
| NEG3390 | dihydrocanadensc | 0.969 | 211.09708 | [M-H2O-H]- | 211.09711 | 79.05367:6003 93.03925:886889 95.03763:862836 131.2403:23563 154.2491:6626 | -1.4211E-07 |
| POS9633 | yonarasterol I | 5.152 | 477.31287 | [M+2H]2+ | 477.3129 | 57.03287:56982 58.04137:15518 59.04859:1226660 60.05226:30691 65.04819:23821 69.06868:19204 73.02812:145377 73.06432:36640 80.0543:48872 83.0477:10988 85.06337:37427 87.04335:209820 87.06079:61516 87.07999:30585 88.04605:9002 89.05896:1451464 90.06191:61655 94.06947:30623 101.05931:131752 102.06606:25430 103.03849:14083 103.07444:283960 104.07732:12882 105.09028:8596 107.07059:14672 109.0756:35061 115.07378:10308 116.08248:15275 117.0909:23098 124.08022:8919 127.07501:8106 129.08911:26603 131.06766:22858 133.08545:470848 134.08684:22171 140.09358:8084 145.08403:12423 147.10149:127214 161.11441:8142 177.1078:47142 191.1256:22526 | -6.2852E-08 |
| POS8061 | Desoximetasone | 4.592 | 377.21191 | [M+H]+ | 377.21201 | 74.06023:8026 89.05896:13965 91.90891:6126 377.22052:9604 | -2.651E-07 |
| POS2774 | thyl-2-prenylthiop | 3.379 | 167.08884 | [M+H]+ | 167.08881 | 77.03802:33641 79.05312:119463 80.05737:18155 91.0539:73080 92.05668:7771 93.06841:196037 94.07143:32746 95.04893:37463 103.05421:809050 104.05679:150822 105.04401:8951 107.04917:92188 108.05284:10313 118.0629:8015 120.06335:61653 120.0803:3048589 121.08412:632523 122.0861:7363 131.04832:35670 | 1.79545E-07 |
| POS4916 | Securinine | 8.805 | 240.09859 | [M+H-H2O]+ | 240.09869 | 103.37626:5837 116.44865:5990 118.20353:5371 124.99955:6414 173.43526:9234 196.63867:5118 | -4.165E-07 |
| POS8276 | Xanthoangelol I | 4.483 | 393.20648 | [M+Na]+ | 393.20639 | 80.38605:5492 179.43362:6023 183.31004:6171 289.0278:6119 | 2.28887E-07 |
| POS9123 | cetyl-leu-leu-tyr-a | 4.795 | 449.27011 | [M+H]+ | 449.26999 | 79.14378:5756 87.04335:5503 89.05896:16159 173.38622:10757 326.5238:6411 343.86096:5698 363.62698:6417 | 2.671E-07 |
| POS12359 | Leu Leu Lys Gln Gly | 5.427 | 586.39203 | [2M+H]2+ | 586.39221 | 57.03286:37116 59.04858:574104 65.04817:18765 73.02811:60325 73.06431:19873 85.06504:20511 87.04333:188581 87.06252:27189 87.07997:23055 89.05894:527083 101.05929:118601 103.07442:134199 115.07642:16951 117.09087:15496 129.0891:22851 133.08542:178858 145.08777:13629 147.10147:63098 177.11282:15666 191.12556:18190 192.67273:16708 234.40762:17853 | -3.0696E-07 |

**Differences in metabolites between the WJW group, the Model group and the Control group**

| Alignment ID | Metabolite name | Rt(min) | Expreiment Mz | Adduct type | Reference m/z | MS/MS spectrum | PPM |
|---|---|---|---|---|---|---|---|
| POS9460 | acetildenafil | 4.633 | 467.27637 | [M+H]+ | 467.2764 | 73.02811:107851 73.06432:42993 75.91343:15367 80.05275:28522 81.03229:26734 87.04334:210130 89.05895:984995 90.0619:60965 91.04643:42961 95.04694:39370 99.04339:29260 102.06827:17648 111.04189:42770 113.05927:26667 131.07086:17292 131.7048:16921 133.08543:356423 134.08684:39526 137.0598:27111 155.07083:15947 177.11284:33955 391.74057:15221 | -6.4202E-08 |
| POS8166 | sartorymensin | 5.686 | 385.16568 | [M+NH4]+ | 385.1658 | 55.01768:108421 57.06894:137002 83.01192:8474 85.06333:50239 111.49811:5934 155.18697:6350 204.95941:6868 | -3.1155E-07 |
| POS12534 | | 4.934 | 595.3631 | [M+Na]+ | 595.36292 | 59.82801:13607 62.53004:12277 100.68073:11281 135.33597:12776 148.35861:11993 420.52808:15110 569.88092:12102 | 3.02337E-07 |
| POS4287 | lpha-D-galactopy | 1.025 | 217.06792 | [2M+H]+ | 217.06799 | 52.17733:5754 62.98166:16407 70.06431:8139 75.71778:6086 80.94776:1450871 85.028:29720 90.97558:243299 134.10017:5273 160.03503:14015 | -3.2248E-07 |
| POS8731 | 3,4,6,7,8,8a-hexahy | 5.403 | 425.29007 | [M+2H]2+ | 425.29001 | 57.03287:84902 59.04859:1929612 59.05931:49421 60.05226:47566 65.04819:13475 69.06868:16412 73.02812:80386 73.06432:29815 81.0699:7680 83.0851:8116 85.06337:30340 87.04335:141455 87.06254:30547 87.07999:32010 89.05896:676639 90.06191:20312 94.06947:46752 99.08151:8114 101.05931:143792 103.07444:347040 104.07732:9876 115.07378:17640 116.08248:11319 117.05281:13965 117.0909:28290 129.08911:17986 131.06766:13510 133.08545:166479 134.08684:10288 143.1078:6873 147.10149:126930 148.1028:7550 161.11441:18634 191.1256:15710 | 1.4108E-07 |
| POS13123 | methylviny l)-17-o | 5.318 | 623.40717 | [M+2H]2+ | 623.40704 | 57.03287:38020 59.04859:691674 73.02812:112119 73.06432:31260 87.04335:166710 87.06254:35440 87.07999:27390 89.05896:1062317 90.06191:33289 94.06947:28494 101.05931:97398 103.07444:200628 107.06821:23051 109.0756:29367 126.93054:21131 130.08708:26378 131.06766:38358 133.08545:382463 147.10149:107107 147.47386:22019 177.1078:49877 | 2.08531E-07 |
| POS9694 | PC(15:1(9Z)/0:0) | 8.808 | 480.30655 | [2M+H]+ | 480.30661 | 54.75832:6275 57.06896:7381 62.05909:38228 63.97328:6278 69.06992:10406 83.08509:7427 89.78172:6752 100.4252:5806 104.10696:12415 112.92513:6587 153.58037:5681 155.00865:5704 173.44016:13028 174.6582:5647 204.03627:6123 410.89926:6566 | -1.2492E-07 |
| POS10797 | Hemiasterlin | 5.6 | 527.35992 | [M+2H]2+ | 527.35999 | 57.03287:145722 59.04859:2215140 59.05931:56826 60.05226:60017 69.06992:30441 73.02812:165016 73.06432:77054 85.06337:64190 87.04335:285695 87.06079:53136 87.07999:88955 89.05896:1224127 90.06191:73138 94.06947:39396 99.07939:22454 101.05931:325858 101.09641:21453 103.07444:665743 104.10697:119447 105.10879:49069 115.07378:60897 116.08248:44570 117.0909:69733 129.08911:65918 131.06766:44171 131.10634:36730 133.08545:425443 134.08684:38044 143.10413:30270 145.08403:24073 147.10149:350106 161.1188:60345 173.11685:32836 177.1078:49793 184.06931:55314 185.07635:21746 186.07535:28736 191.1256:59552 | -1.3274E-07 |

**Differences in metabolites between the WJW group, the Model group and the Control group**

| Alignment ID | Metabolite name | Rt(min) | Expreiment Mz | Adduct type | Reference m/z | MS/MS spectrum | PPM |
|---|---|---|---|---|---|---|---|
| POS10704 | PD173074 | 5.353 | 524.33429 | [M+2H]2+ | 524.33429 | 59.04859:344152 60.08025:793941 71.07261:123900 73.03751:126679 86.09541:1677589 89.05896:278884 104.10697:10252773 105.1111:233840 124.99956:361402 152.55547:117446 184.07466:3732591 248.77914:103774 | 0 |
| POS14421 | G(16:1(9Z)/16:1(9Z | 6.595 | 719.48578 | [M+2H]2+ | 719.48572 | 59.63764:5914 69.55946:7557 153.83411:6111 | 8.33929E-08 |
| POS9671 | bipinnatone A | 4.727 | 479.27853 | [M+H]+ | 479.2785 | 69.59312:5999 248.1651:5493 | 6.25941E-08 |
| POS9565 | droxy-5-[(3Z)-5-hy | 4.513 | 474.28506 | [M+2H]2+ | 474.28503 | 62.01709:7800 67.75507:8760 70.06428:9745 74.05883:14795 84.04313:19873 86.09538:115987 87.09915:8524 97.10977:7069 101.06799:8509 110.30173:7219 115.08699:8192 120.08026:150606 121.08408:12895 132.10168:25525 161.89456:9129 173.39105:17718 238.93481:8120 | 6.32531E-08 |
| POS1651 | Butoxyacetic acid | 4.956 | 133.08571 | [M+H]+ | 133.0858 | 58.06511:9057 69.06867:30295 86.09539:147379 87.09917:10505 89.00121:6080 132.52338:5562 | -6.7626E-07 |
| POS9695 | ysoPE(18:1(9Z)/0:0 | 5.375 | 480.30927 | [M+CH3OH+H]2+ | 480.3093 | 57.03287:22287 59.04859:253173 60.05226:7475 67.015:16773 73.02812:27236 73.06432:11574 78.30522:6307 81.03072:10941 85.06337:11317 87.04335:33369 87.06254:6825 87.07999:6875 89.05896:199777 90.06191:11231 101.05931:19575 103.07445:52426 117.05281:24502 117.0909:5638 131.07088:12578 133.08545:59241 136.66437:5460 147.10149:20882 347.26852:6725 | -6.246E-08 |
| POS10699 | Calcimycin | 5.474 | 524.27399 | [M+H]+ | 524.27399 | 59.04856:33682 60.08023:26828 62.05907:68444 67.05387:32624 69.06989:11172 79.05461:18944 81.06988:20294 86.09538:48259 87.04332:10912 89.05893:38101 91.05387:18221 93.06838:15104 95.08474:12806 104.10693:269808 104.94925:11291 105.06942:14786 184.0746:151074 278.50723:12180 421.39975:12577 | 0 |
| POS14554 | Argentinic acid G | 5.168 | 733.48761 | [M+H]+ | 733.48767 | 57.03379:50785 59.04859:896524 73.06432:52556 87.04335:127946 89.05896:811414 101.05931:153641 103.07444:313097 117.05281:38620 133.08545:230489 147.10149:106157 302.83173:31899 437.43027:38567 712.33484:42054 | -8.1801E-08 |
| POS10326 | Stellettin I | 5.493 | 509.32632 | [M+2H]2+ | 509.32639 | 57.03287:24173 59.04859:232241 67.015:16511 73.02812:19835 81.03072:14771 83.22678:5555 87.04335:25750 87.07999:8051 89.05896:150122 90.06191:7309 94.06555:6141 101.05931:30496 103.07444:67063 104.10697:9472 115.07643:5774 117.05281:6304 117.0909:6914 133.08545:34909 147.10149:21165 158.09198:7607 165.10016:7149 211.56157:5429 | -1.3744E-07 |
| POS9623 | hyl-but-2-enyl)-d | 4.476 | 477.22696 | [M+H]+ | 477.22711 | 80.10661:5961 87.04333:27250 89.05895:367847 90.0619:23458 112.07433:24636 119.08611:15831 133.08543:138518 134.08682:15450 177.11284:21139 178.11942:7388 | -3.1432E-07 |
| POS11020 | PC(16:1(9Z)/2:0) | 5.39 | 536.33325 | [M+H]+ | 536.33313 | 59.04859:185890 60.08025:33364 73.02812:25239 86.09541:77299 87.04335:36444 89.05896:239720 90.06191:16693 101.05931:26606 103.07445:42881 104.10697:422972 124.99956:16040 133.08545:71047 147.10149:19161 184.07466:172538 290.39401:12274 | 2.23742E-07 |

**Differences in metabolites between the WJW group, the Model group and the Control group**

| Alignment ID | Metabolite name | Rt(min) | Expreiment Mz | Adduct type | Reference m/z | MS/MS spectrum | PPM |
|---|---|---|---|---|---|---|---|
| POS8677 | Formacidine | 5.227 | 422.26428 | [M+2H]2+ | 422.26428 | 57.03286:10914 59.04858:108569 67.01499:17543 81.03072:6926 87.04334:7213 89.05895:80268 101.0593:8728 103.07443:22231 133.08543:26469 147.09764:8730 165.10014:6089 180.10658:5913 252.63242:5638 293.42517:5984 | 0 |
| NEG4912 | Hydroprene | 1.052 | 265.21741 | [M-H]- | 265.21741 | 54.30708:6028 59.08406:7086 89.13129:326629 90.13491:13540 93.0393:178610 95.03768:354553 97.0375:16669 121.99403:5086 147.15923:83595 149.16045:35272 154.19572:6567 169.77672:5477 220.4525:5858 | 0 |
| POS10094 | Cucurbitacin S | 4.964 | 499.30594 | [M+CH3OH+H]+ | 499.30579 | 57.03287:19243 59.04859:263512 60.08025:13730 73.02812:14774 73.06432:10015 85.06337:9587 86.09541:33442 87.04335:970332 87.09918:10552 88.04783:14431 89.05896:214713 101.05931:219429 103.03849:77055 103.07444:60662 104.10697:123746 105.10879:47566 106.11105:13063 115.07643:10213 117.05281:72333 131.06766:30221 133.08545:61663 147.10149:25608 184.07466:32565 185.07635:14610 186.07535:16191 | 3.00417E-07 |
| POS10120 | Aconine | 8.901 | 500.28616 | [M+H]+ | 500.2861 | 55.05453:11157 58.30054:10092 81.0699:10628 86.09541:10895 95.08477:10350 104.10696:42753 105.1111:21941 107.08486:12771 119.08333:15483 126.02023:87930 127.02272:11667 128.01544:40502 159.11723:14402 173.43034:13153 209.13087:13199 227.14313:10869 | 1.19931E-07 |
| POS9632 | yonarasterol I | 4.5 | 477.31284 | [M+H]+ | 477.3129 | 69.93977:14540 87.04334:26210 89.05896:391634 90.0619:30627 103.72703:8538 112.07434:12685 133.08543:137001 134.08684:29949 177.11285:12063 419.51126:10238 | -1.257E-07 |
| POS8640 | ethoxy-3-(3-meth | 4.839 | 419.22461 | [M+Na]+ | 419.22476 | 58.04042:12495 59.04859:189004 65.04932:9874 73.02812:51785 73.06432:10804 80.0543:7267 85.06337:8721 87.04335:51168 87.06254:10942 89.05896:257374 90.06375:7579 101.05931:18330 102.06606:13027 103.0385:19246 103.07445:23508 109.0756:6560 133.08545:66767 147.10149:7449 | -3.578E-07 |
| POS10619 | quaesitol | 5.068 | 521.32611 | [M+H]+ | 521.32611 | 59.04858:22204 60.08025:63739 86.09541:93852 87.04334:8058 89.05896:17883 104.10696:380594 108.16389:6635 124.99955:14995 173.39111:8455 182.94283:6709 184.07466:79071 239.22867:6583 282.12573:5632 | 0 |
| NEG8080 | o-7,8-dihydropyra | 1.054 | 387.18332 | [M-H]- | 387.1832 | 65.69628:5658 68.01559:5751 93.03928:352337 95.03767:298038 97.03749:33426 124.15681:11693 151.06717:22659 153.0676:49273 155.06657:11867 358.09082:5782 | 3.09931E-07 |
| POS9843 | 3,4,6,7,8,8a-hexahy | 4.43 | 487.26398 | [M+H-H2O]+ | 487.26401 | 64.9233:6323 77.04674:5750 87.04335:9197 89.05896:380439 107.06821:6794 111.12991:6008 118.06566:12199 133.08545:173386 144.0797:15248 146.061:74743 151.09705:6603 159.09138:11369 173.42545:8962 177.11285:29188 188.07004:307841 189.07106:13994 205.09822:63379 283.17694:10805 | -6.1568E-08 |
| POS9197 | 4-methyl-9,11-sec | 4.583 | 453.33994 | [M+H]+ | 453.34 | 61.49389:5160 96.08015:43369 100.1125:10595 111.08459:5769 113.10571:19788 114.09121:103674 173.39595:7814 209.16328:26759 228.16107:6962 | -1.3235E-07 |

**Differences in metabolites between the WJW group, the Model group and the Control group**

| Alignment ID | Metabolite name | Rt(min) | Expreiment Mz | Adduct type | Reference m/z | MS/MS spectrum | PPM |
|---|---|---|---|---|---|---|---|
| POS8645 | Denticulatin B | 5.002 | 419.27682 | [M+2H]2+ | 419.27682 | 59.04858:171300 73.02811:40529 73.06432:7607 80.0543:12196 87.04334:30343 87.06253:8494 89.05896:235891 90.06191:9288 101.0593:8606 102.06606:6905 103.07444:23919 111.18527:6119 133.08543:44807 137.5574:5651 147.10149:8191 177.11285:9764 188.64743:7025 | 0 |
| POS8140 | Lehualide D | 4.729 | 383.2251 | [M+H]+ | 383.22501 | 57.03287:10409 59.04859:284434 69.79429:8099 70.09203:5685 73.02812:18017 77.05836:5557 83.1274:6299 84.20721:5229 87.04335:481519 89.05896:167555 96.48599:5631 101.05931:64745 103.03849:237185 103.07444:42981 107.06821:7106 117.05281:78851 131.07088:12359 133.08545:40764 147.06319:6251 147.10149:13324 186.17351:5380 | 2.34849E-07 |
| POS9785 | malyngamide I | 6.112 | 484.28232 | [M+H-H2O]+ | 484.28238 | 59.04856:15166 62.05907:9682 71.34617:6384 104.10693:11735 184.07458:11252 190.59322:5859 | -1.2389E-07 |
| POS9100 | nethyl-7,11-dioxo | 5.643 | 447.30978 | [M+H]+ | 447.30981 | 57.06896:13752 59.04858:36661 89.05896:16814 97.06461:16142 101.0593:8015 103.07444:7241 121.0641:6520 159.11293:6657 | -6.7068E-08 |
| POS12414 | Nephilatoxin 1 | 5.693 | 589.38202 | [M+2H]2+ | 589.38202 | 57.03287:29722 57.52443:16027 59.04859:194931 65.56047:14424 67.015:16832 70.43989:14676 73.02812:27593 73.06432:18286 87.04335:26774 89.05896:153815 101.05931:29506 103.07444:60804 105.5429:13858 133.08545:52561 147.10149:17220 309.90234:13809 412.1004:13169 | 0 |
| POS7203 | a,4,5,8,9,12,13,15a | 5.222 | 331.18921 | [2M+H]+ | 331.18909 | 53.34515:5668 66.18044:7608 67.05388:8402 81.06989:11972 88.69724:5445 90.57806:4990 95.08475:11984 97.06458:6005 105.06943:6124 107.08484:6942 121.06407:28359 133.0986:6886 147.07846:6495 177.55913:6473 | 3.62331E-07 |
| POS10191 | droxy-6-methylhe | 6.221 | 503.29526 | [M+H]+ | 503.29541 | 59.04858:9200 65.70778:5439 86.0954:8650 89.05895:12061 104.10696:22086 484.58868:5896 | -2.9804E-07 |
| POS8041 | roxy-6-hydroxyme | 5.259 | 376.173 | [M+NH4]+ | 376.17303 | 55.0177:9572 55.05365:15226 57.03287:38159 59.04858:26096 61.02743:30522 69.03294:335076 71.04816:16089 73.02812:16384 79.05312:17888 81.03229:46270 83.0477:50525 85.028:47738 87.04334:12683 89.05896:9685 91.0539:660279 92.05669:16433 97.0297:10858 99.0434:14089 103.03849:8446 105.03246:62733 107.04917:113719 111.0419:53141 127.03809:14156 129.05447:72112 147.06317:130369 | -7.9751E-08 |
| POS12537 | e-(3beta,5beta,11 | 5.902 | 595.38391 | [M+2H]2+ | 595.38397 | 57.03287:38326 59.04859:187299 73.02812:62898 85.06337:25280 87.04509:37664 87.06254:22316 89.05896:185028 101.05931:43827 103.07444:31021 133.08545:49123 | -1.0078E-07 |
| POS10431 | hylhexadecahydro | 5.798 | 514.2829 | [M+H]+ | 514.28302 | 59.04857:35530 85.06335:16559 89.05894:28911 93.07031:6606 95.08475:16434 109.10004:9500 121.10126:9594 126.0202:184390 133.0854:11048 157.10194:9082 159.1172:8808 209.13083:7331 | -2.3333E-07 |
| POS7719 | Sterebin M | 4.85 | 356.27875 | [M+H]+ | 356.2789 | 58.06511:235047 59.04858:34692 60.32804:6778 73.06432:7429 81.06989:22804 82.43508:5463 84.07957:11302 89.05895:32250 104.79474:5929 108.885:6196 123.11523:24179 133.08543:7052 140.14343:9963 280.99945:5498 341.80237:6580 | -4.2102E-07 |

**Differences in metabolites between the WJW group, the Model group and the Control group**

| Alignment ID | Metabolite name | Rt(min) | Expreiment Mz | Adduct type | Reference m/z | MS/MS spectrum | PPM |
|---|---|---|---|---|---|---|---|
| POS9736 | nethyl-9-oxo-1,2, | 8.887 | 482.2749 | [M+H-H2O]+ | 482.27484 | 53.99451:6296 71.08418:5443 95.08477:8823 95.8601:5344 104.10696:30712 107.08486:6203 126.02023:43718 127.01965:6483 128.01855:16014 130.86165:5197 412.2981:5842 | 1.2441E-07 |
| NEG9815 | Cefpiramide | 5.385 | 611.112 | [M-H]- | 611.112 | 79.61639:5075 89.13125:759890 90.13486:35374 94.03929:5042 117.0695:29805 370.36819:6691 412.74951:5783 440.99393:6109 | 0 |
| POS9810 | Communesin H | 4.826 | 485.29111 | [M+H]+ | 485.29099 | 57.03287:9114 59.04859:208773 76.26552:5650 85.06506:6110 87.04335:26542 89.05897:78871 101.05931:16898 103.0385:10071 103.07445:43505 133.08545:10921 147.1015:9228 161.05295:5750 353.60825:5860 365.49567:5915 372.1275:5635 | 2.47274E-07 |
| POS8830 | Kuguacin E | 6.151 | 431.31509 | [M+2H]2+ | 431.31509 | 59.04858:9678 95.08477:12758 97.06461:61890 97.10159:10056 107.08486:11493 109.06336:6145 109.10006:12249 112.9767:5473 121.0641:6371 121.1013:7481 133.10193:8005 135.11646:7058 147.11681:6669 171.11887:5707 177.12804:19253 357.97528:5319 | 0 |
| POS8955 | Anisodorin 3 | 5.696 | 439.3053 | [M+2H]2+ | 439.3053 | 57.03287:46780 59.04859:698843 59.05931:17682 60.05226:21029 73.02812:27360 73.06432:29850 85.06337:9506 87.04335:41472 87.07999:7958 89.05896:167857 90.06191:6728 94.06947:8708 99.07939:6574 101.05931:62274 103.07444:111550 104.07732:7553 115.07378:17486 117.05281:7619 117.0909:20451 129.08911:6502 133.08545:32517 147.10149:33827 161.1188:7787 374.9805:6956 393.72964:6196 | 0 |
| POS12390 | yl-sn-glycero-3-pl | 5.747 | 588.36383 | [M+H]+ | 588.36401 | 59.04856:36489 60.08022:12272 86.09536:9160 87.0433:13002 89.05891:30567 101.05926:7093 103.07439:13733 104.10692:87875 133.08537:11196 173.39102:11056 184.07457:21898 412.29788:6117 | -3.0593E-07 |
| POS10255 | Talaroconvolutin E | 5.997 | 506.32382 | [M+H]+ | 506.32379 | 51.79336:6660 53.41721:6159 59.04859:22317 60.08025:12511 66.38443:5356 86.09541:31301 89.05896:18294 104.10697:144927 184.07466:37367 185.39581:4931 201.78914:5385 | 5.92506E-08 |
| POS1562 | lichloro-1,1-ethan | 9.248 | 130.96594 | [M+H-H2O]+ | 130.966 | 54.03373:7588 55.93435:6275 56.04952:26633 57.06988:6346 61.02845:6356 67.05389:23205 67.93391:13180 70.06556:7088 71.92826:29503 72.93706:41262 74.99706:7067 77.49896:7387 77.99808:64843 78.9988:8127 84.04315:36824 84.07957:76239 85.0061:22638 85.08359:6166 86.09711:6852 86.50518:17245 87.00323:285725 87.50385:31305 88.00347:191576 89.50647:9359 89.93904:12920 90.94762:9840 91.05389:5831 94.01266:8758 96.00942:20700 96.51448:7032 97.00917:70070 98.51207:53196 99.51075:59623 106.37916:6335 107.94919:7916 113.96311:105942 115.96169:38962 116.97124:12352 126.97046:6999 129.99471:7179 131.97466:30121 | -4.5813E-07 |
| POS12327 | kirkinine D | 4.852 | 585.27051 | [M+H]+ | 585.27039 | 55.4905:5598 89.05896:11618 106.74535:6542 133.08545:8463 179.12949:6616 299.14221:7438 | 2.05033E-07 |
| POS4447 | CHEMBL4283703 | 2.625 | 223.06311 | [M+H]+ | 223.0632 | 58.06511:13469 59.04858:7560 61.03868:29019 66.64188:6661 73.02811:9639 73.04553:12324 80.59386:5975 91.05576:17270 93.06839:6086 209.00749:6987 225.04041:63944 | -4.0347E-07 |

**Differences in metabolites between the WJW group, the Model group and the Control group**

| Alignment ID | Metabolite name | Rt(min) | Expreiment Mz | Adduct type | Reference m/z | MS/MS spectrum | PPM |
|---|---|---|---|---|---|---|---|
| POS4449 | CHEMBL4283703 | 0.702 | 223.06323 | [M+CH3OH+H]+ | 223.0632 | 58.02428:6139 58.06417:21920 59.04859:10298 73.02812:10576 73.04555:18931 76.81198:5763 79.05464:8297 90.48373:5205 91.05577:26695 93.06841:11778 119.04707:16973 155.97054:7677 192.70723:5645 209.00754:13773 225.04045:104121 226.04433:7698 | 1.34491E-07 |
| POS304 | Butyric acid | 4.952 | 89.05968 | [M+H]+ | 89.05968 | 61.02845:46841 68.67587:6086 72.93705:39696 87.00497:16898 90.94762:6676 | 0 |
| POS244 | Butanal, 2-oxo- | 2.532 | 87.04405 | [M+H]+ | 87.04405 | 69.03294:23471 69.06869:12170 86.05939:124827 87.04335:8207 87.06254:8704 | 0 |
| POS5757 | Doxylamine | 5.121 | 271.18137 | [M+H]+ | 271.1814 | 59.04859:7989 64.42622:5475 69.06868:5858 75.68808:5550 116.85452:5675 | -1.1063E-07 |
| POS10170 | Ala Leu Leu Ala Se | 6.274 | 502.32336 | [M+H]+ | 502.32339 | 74.3554:6617 95.82383:5824 183.62494:5457 | -5.9722E-08 |
| POS2584 | C)OC(C(CC(C)O)C): | 5.244 | 161.11703 | [M+H]+ | 161.1171 | 52.41368:6692 59.04859:8937 | -4.3447E-07 |
| POS9182 | Melleolide M | 5.746 | 453.16736 | [M+H]+ | 453.16739 | 57.03286:72567 57.94083:5677 59.04858:32735 62.03075:6005 85.02798:7600 99.07938:8882 | -6.6201E-08 |
| NEG6125 | Sclareol | 0.754 | 307.27808 | [M-H]- | 307.2782 | 76.37918:6321 76.39638:6059 189.36215:6130 | -3.9053E-07 |
| NEG1909 | Isatinoxim | 0.179 | 161.0356 | [M-H]- | 161.03571 | 55.08417:6740 57.10281:10574 59.08407:77697 67.31788:5529 71.09903:13234 73.11623:150130 82.68986:5427 85.13133:27001 101.14608:15075 110.22124:7649 113.15997:10825 | -6.8308E-07 |
| POS9809 | Ardisiaquinone E | 6.66 | 485.28726 | [M+H]+ | 485.28729 | 55.91729:6877 59.04858:18935 91.0539:15434 133.09863:54886 173.39111:11258 317.47849:6127 | -6.1819E-08 |
| POS7167 | naH-cheilantha-13 | 2.593 | 329.32031 | [M+H]+ | 329.32019 | 51.93132:6968 55.05365:11566 57.06896:23455 59.03105:90371 61.01003:62247 62.98918:11778 67.05389:7884 69.06992:13425 71.08419:13075 73.04555:73611 75.02498:108072 76.02578:6615 77.00463:7738 77.04093:83916 79.01993:16467 83.0851:9873 87.06253:6298 91.05577:87400 91.52596:5809 93.03564:90181 95.01511:5977 97.09953:15795 99.86346:5875 105.07177:29441 111.11732:14346 119.08891:6894 149.44341:5671 | 3.64387E-07 |
| POS5229 | lamino)phenyl]car | 4.8 | 251.10243 | [M+H]+ | 251.10258 | 59.04858:48481 60.04425:81896 69.06991:8564 70.02779:69771 70.09202:6754 74.02328:16012 81.21274:5624 87.04333:12693 88.03894:227375 89.04271:8569 94.06358:167546 98.02461:9232 99.0074:33563 104.04995:9980 116.03413:7494 117.0528:7389 120.04356:59548 123.03605:5659 130.04883:21732 132.04305:8725 136.0761:17339 146.05719:220239 147.06316:7815 150.05341:12704 158.04501:9386 174.05496:49223 192.06534:8392 214.37151:5709 | -5.9737E-07 |
| POS236 | Divinyl sulfide | 1.56 | 87.02609 | [M+H]+ | 87.0262 | 56.04863:17014 57.05693:39967 69.03294:10719 69.06868:27926 86.05939:34811 86.09541:16429 | -1.264E-06 |
| POS6698 | phenyl)-3,4-dihyd | 2.76 | 307.08212 | [M+2H]2+ | 307.082 | 76.03857:7299 84.04314:51760 130.04883:21051 | 3.90775E-07 |
| NEG1882 | Dihydroxynaphtha | 4.314 | 160.05246 | [M-H2O-H]- | 160.05243 | 57.10281:6266 59.08407:42864 62.57202:5617 73.11623:55262 97.1813:5084 154.52921:4889 | 1.87439E-07 |
| POS15135 | 11Z,14Z,17Z)/18:3 | 7.002 | 804.5509 | [M+H]+ | 804.55103 | 173.43526:10543 280.09122:6543 635.19501:6346 | -1.6158E-07 |
| POS10277 | Asn Phe Ala Arg | 6.663 | 507.26715 | [M+Na]+ | 507.2673 | 55.42575:7476 59.62183:6501 83.06232:5808 107.32325:7394 158.40836:5391 184.34851:5577 | -2.957E-07 |

**Differences in metabolites between the WJW group, the Model group and the Control group**

| Alignment ID | Metabolite name | Rt(min) | Expreiment Mz | Adduct type | Reference m/z | MS/MS spectrum | PPM |
|---|---|---|---|---|---|---|---|
| POS5047 | Pseudouridine | 1.585 | 245.07607 | [M+H]+ | 245.0761 | 54.03372:7226 57.3748:6140 61.03869:8235 68.04829:6953 79.05161:7372 80.04967:7066 82.02831:10593 100.03728:16503 125.03256:34265 155.04594:13881 167.04433:6999 189.8046:5950 191.04048:9591 | -1.2241E-07 |
| POS8795 | Leu Arg Leu | 6.871 | 429.31827 | [M+H]+ | 429.3183 | 70.0706:6749 171.49448:6082 | -6.9878E-08 |
| POS10278 | Amoritin | 6.663 | 507.27118 | [M+H]+ | 507.27121 | 76.2698:6746 149.75777:6022 | -5.914E-08 |
| POS15192 | PC(18:0/18:1(9Z)) | 6.665 | 810.59821 | [M+2H]2+ | 810.59833 | 62.37422:6728 207.28555:5488 | -1.4804E-07 |
| POS6798 | 3-hydroxypentane | 4.559 | 311.20444 | [M+H-H2O]+ | 311.20441 | 57.03287:52959 59.04859:2111908 60.05226:27540 73.02812:20430 73.06432:14659 85.06506:30420 87.04335:156446 87.07999:9067 89.05896:2519716 90.06191:43693 101.05931:56081 103.03849:67758 103.07444:277993 107.07059:27440 117.05281:22880 129.08911:11014 131.06766:9861 133.08545:399722 134.08684:16435 147.10149:78077 151.09705:15682 | 9.63997E-08 |
| POS13939 | (28),23-dien-3-oi | 8.716 | 679.47772 | [M+H]+ | 679.47791 | 53.85504:6025 129.86432:5667 529.3476:5951 | -2.7963E-07 |
| POS7967 | 5beta-Coprostano | 0.607 | 371.36737 | [M+H]+ | 371.36722 | 57.06989:9920 59.03105:40356 61.01003:10249 73.04689:37856 75.02498:32068 77.04093:20602 91.05577:34151 93.03564:23727 | 4.03913E-07 |
| POS4258 | Oxododecanoic ac | 5.797 | 215.16423 | [M+H]+ | 215.1642 | 57.03287:10104 66.7261:6671 67.05389:5776 122.57718:6670 142.00749:5780 | 1.39428E-07 |
| POS7208 | Tianshic acid | 6.156 | 331.24756 | [M+H]+ | 331.24759 | 55.36465:6892 178.25224:6519 | -9.0567E-08 |
| POS13403 | Filixic acid pbp | 6.87 | 641.25934 | [M+H]+ | 641.25922 | 67.85222:5791 86.21047:5133 151.98245:6514 223.58301:5499 | 1.87132E-07 |
| POS4256 | Putaminoxin E | 5.609 | 215.16414 | [M+H]+ | 215.16409 | 50.61698:4718 50.62858:5628 50.634:5520 52.61634:5988 59.28901:5307 61.05301:4706 63.6503:4244 65.26049:4134 68.36765:4736 69.36423:4484 70.8761:4970 74.7813:4590 77.10919:3789 79.32551:4591 88.21125:5039 88.39301:4773 92.35327:4313 92.43147:4989 96.87998:4409 98.64025:4532 100.98293:4280 103.63856:4294 104.2325:4271 112.76032:3816 116.427:4465 117.93621:4574 120.90114:4925 160.97826:4331 161.43983:4379 164.86342:5118 178.03259:5728 181.50056:4790 182.55013:3901 195.91809:4328 196.93501:4740 203.49271:4507 208.5345:4770 217.56131:4415 217.65092:4551 | 2.32381E-07 |
| POS4257 | Oxododecanoic ac | 5.971 | 215.16423 | [M+H-H2O]+ | 215.1642 | 64.30312:6434 69.00585:5486 74.06844:6177 173.3911:9235 | 1.39428E-07 |
| POS1486 | CHLOROACETIC AC | 9.179 | 128.95036 | [M+H]+ | 128.95039 | 53.03856:10805 54.03373:10138 55.0177:10933 55.05365:150397 55.93345:179525 56.02069:8500 56.04862:16413 57.03286:41085 57.04396:29066 58.06416:18311 59.04858:7804 60.04425:15010 65.03804:9745 67.04092:12917 67.05389:6669 69.03417:9779 69.06868:26954 69.93349:9102 70.0643:10771 71.04816:6777 72.04237:6227 80.04967:8994 82.06502:99688 83.04769:18093 83.0672:10035 84.04315:20834 84.07957:10450 84.58686:6181 86.05938:12259 100.07597:15920 101.0593:6259 101.36545:5561 110.06083:13659 114.97044:45719 128.04031:16387 128.06833:26616 128.10568:17720 | -2.3265E-07 |
| POS2454 | S-HNE | 5.797 | 157.12212 | [M+H-H2O]+ | 157.12219 | 59.829:6147 67.64503:6272 70.0643:11551 74.93713:42569 92.94704:7407 115.9617:16302 122.9072:5297 | -4.4551E-07 |
| POS763 | -2-Methylthiazolic | 1.565 | 104.05288 | [M+NH4]+ | 104.0528 | 56.04863:29277 57.03287:24803 58.06512:336618 60.08025:66032 61.01003:36530 61.02846:30831 62.05909:8009 69.03294:6299 86.05939:11487 104.10697:38356 | 7.6884E-07 |

**Differences in metabolites between the WJW group, the Model group and the Control group**

| Alignment ID | Metabolite name | Rt(min) | Expreiment Mz | Adduct type | Reference m/z | MS/MS spectrum | PPM |
|---|---|---|---|---|---|---|---|
| POS17 | Nitroethane | 4.635 | 76.03915 | [M+H]+ | 76.0392 | 51.24459:4285 55.05716:4903 55.30983:7160 57.15702:5153 57.30393:4660 57.6643:4205 57.95031:3573 58.80668:4248 60.95991:4197 61.88621:4240 65.07974:4101 66.60102:5484 69.31342:3955 69.56071:5039 70.01898:4557 71.18597:5216 72.46844:4568 74.39948:4665 | -6.5756E-07 |
| POS7696 | Erythrophloin D | 5.256 | 355.26282 | [M+H-H2O]+ | 355.26279 | 55.05365:12225 59.04858:19960 67.05389:9657 70.21569:5375 81.06833:32453 83.4408:5835 85.06506:7493 89.05896:19468 93.06841:22560 95.08477:27711 95.58656:5191 99.04339:10223 101.0593:7161 105.06945:25400 107.08486:16717 109.10006:22608 117.06913:6411 119.08333:19304 121.1013:21053 123.08004:16834 131.08699:13095 133.10193:25328 135.11646:18656 145.09904:14060 147.11681:21592 149.13393:7716 159.11723:32874 161.13197:16357 173.13152:12958 185.13043:8133 256.09998:6970 | 8.44445E-08 |
| POS3713 | L-Arginine | 0.948 | 197.10004 | [M+H]+ | 197.10001 | 95.17444:5217 173.43034:11655 | 1.52207E-07 |
| POS2606 | Aminopentanoic a | 1.019 | 162.04997 | [M+NH4]+ | 162.05 | 55.0177:68381 55.05365:34633 56.04862:40127 57.03286:476354 58.06511:99164 59.07295:127286 60.08024:2624557 61.02742:35746 70.0643:36310 84.07957:63394 85.02799:1124644 98.06006:93569 102.09041:716844 103.03848:1458833 162.11159:578657 | -1.8513E-07 |
| POS3956 | Eriosematin F | 2.594 | 205.08528 | [M+CH3OH+H]+ | 205.0854 | 55.93435:11112 57.06989:7322 60.04327:6456 109.32312:5950 149.02449:11007 | -5.8512E-07 |
| POS2457 | S-HNE | 5.411 | 157.12216 | [2M+H]+ | 157.12219 | 55.0177:8019 55.05365:8181 67.05389:7626 67.55908:5260 69.06992:7577 70.06556:10614 74.93713:27741 142.21487:5011 | -1.9093E-07 |
| POS2456 | S-HNE | 6.185 | 157.12212 | [M+H]+ | 157.12219 | 53.70765:6653 55.73544:6473 55.87062:7001 55.8778:7466 70.06431:7513 84.07958:5323 | -4.4551E-07 |
| POS3306 | Phosphorylcholine | 5.717 | 184.07246 | [M+H-H2O]+ | 184.07249 | 53.48685:5860 57.93419:6489 58.94249:11718 79.25425:5942 86.09538:11694 99.9063:7164 116.97122:31489 117.9775:41158 | -1.6298E-07 |
| POS3810 | cgonine methylest | 4.994 | 200.12769 | [M+H]+ | 200.12759 | 55.0177:20821 55.05365:509425 55.93435:26179 56.46365:6412 60.69907:6153 63.21632:5904 67.05389:54277 69.03294:20509 69.06991:45077 76.03859:206104 79.05312:29338 81.06989:55805 83.08509:62109 85.06336:6453 95.08476:21825 97.09953:121566 97.96837:6897 107.08485:17424 113.96311:40467 114.97045:23147 125.09565:43305 131.97139:7671 141.95662:13195 159.96982:8239 | 4.99681E-07 |
| POS6430 | trahydrobungeand | 6.202 | 296.25693 | [M+H]+ | 296.25681 | 50.40575:6657 55.05365:9155 57.03286:6456 57.06895:157124 59.75949:6013 67.05389:34264 69.06867:16648 71.08546:12163 81.06832:25536 89.05894:5649 93.0684:9678 95.08475:21343 107.08485:6420 109.10004:19765 112.94058:5981 127.10883:20812 135.11642:11814 169.12219:8839 | 4.05054E-07 |
| POS6447 | Nimbidiol | 5.318 | 297.14624 | [M+2H]2+ | 297.14612 | 55.05366:18863 57.06896:140606 67.05389:27585 69.06869:15980 71.08548:78878 81.06834:33064 83.0851:25721 85.10045:44797 86.09541:36444 89.05896:6026 95.08477:48806 97.09953:11422 102.09042:6365 104.10697:25479 109.10007:18973 123.11524:6592 124.99956:5615 184.07466:6961 | 4.03842E-07 |

**Differences in metabolites between the WJW group, the Model group and the Control group**

| Alignment ID | Metabolite name | Rt(min) | Expreiment Mz | Adduct type | Reference m/z | MS/MS spectrum | PPM |
|---|---|---|---|---|---|---|---|
| POS1077 | HLOROMETHYL) E | 0.146 | 114.97119 | [M+H]+ | 114.9711 | 55.0177:41439 55.05365:39687 55.93435:11379 59.04858:44006 60.04425:8325 61.01002:8971 65.69519:5897 68.04829:22913 69.03294:18476 69.06991:48364 70.02779:10448 70.0643:21124 72.04369:14221 72.08047:17478 72.93706:245053 73.04688:21064 73.93718:9329 76.96111:5797 79.05312:20383 86.06966:7235 87.04333:17434 90.94762:49751 91.05576:36055 96.08018:8058 97.06255:5899 97.50974:5642 113.9631:11952 114.06507:26415 114.09124:80031 115.09232:6779 | 7.82805E-07 |
| POS1575 | 3-Oxobutyl acetate | 4.882 | 131.0701 | [M+H]+ | 131.0701 | 56.04953:8861 59.04859:7283 67.05272:7041 69.06992:8089 73.02812:7622 73.06432:17796 84.04317:12866 84.07958:32323 86.09541:17493 87.00325:10920 88.00349:25491 91.0539:27177 97.00919:20774 103.05197:10683 115.96439:9271 130.06477:10960 130.08708:8023 131.08377:10556 131.97467:6433 | 0 |
| POS1564 | dichloro-1,1-ethan | 0.114 | 130.96603 | [M+H]+ | 130.966 | 55.05365:16079 55.93435:8576 56.04862:30593 57.03286:12117 57.06895:13709 58.05276:5832 60.04425:7659 67.05389:27151 69.03294:8992 70.0643:6230 70.76221:6356 71.04816:7946 71.92825:120858 72.93706:51136 74.02328:6586 75.02496:6958 77.06126:6536 83.04769:7578 84.04315:29053 84.07957:78908 85.06505:10994 85.08358:5625 86.05938:9231 86.0954:8645 89.93904:39558 90.94762:14004 93.03563:6575 107.94919:30535 111.93941:6010 112.07433:11074 125.95948:15501 129.99471:6324 130.08707:7610 130.96458:5561 | 2.29067E-07 |
| POS1148 | Ethyl pyruvate | 5.392 | 117.0544 | [M+H]+ | 117.0545 | 55.05366:16095 59.04859:15239 64.36852:5225 70.06431:169751 71.04945:5714 71.06875:8396 72.04501:6564 72.08048:16839 101.25597:5752 | -8.543E-07 |
| POS3068 | Allantoic acid | 1.006 | 177.06184 | [M+H]+ | 177.06183 | 60.05523:12147 70.06428:25050 71.06744:23278 82.464:6268 146.761:5011 146.91003:5418 | 5.64774E-08 |
| NEG5828 | hinidin 3-sambubi | 5.923 | 297.56561 | [M-H]- | 297.56546 | 92.23355:6520 94.94423:6029 101.05437:6192 173.62036:10440 182.25806:6476 292.43253:5709 | 5.04091E-07 |
| POS665 | hyltetrahydrofuran | 5.894 | 101.05945 | [M+H]+ | 101.0596 | 55.05364:12225 60.04425:40639 61.03868:12871 98.69289:5492 100.0738:7191 | -1.4843E-06 |
| POS4133 | leodomycin B | 5.797 | 211.13225 | [M+H]+ | 211.1324 | 57.03287:11300 69.06868:6229 70.42085:6023 71.04816:5428 76.21976:5535 81.0699:9033 95.08477:10395 109.06336:13332 119.08333:6266 125.03258:7347 133.10193:10793 151.10901:114635 165.12749:9258 | -7.1045E-07 |
| POS4451 | delta-Valerolactone | 0.889 | 223.09393 | [M+CH3OH+H]+ | 223.09399 | 58.27951:5910 62.98166:6700 78.99126:5440 80.94775:8679 96.921:226607 103.89967:5711 104.99084:6436 144.94151:5536 164.92259:37831 202.468:6480 | -2.6894E-07 |
| POS149 | al\|Nitrile-(()-3-Hyd | 9.005 | 84.04433 | [M+H-H2O]+ | 84.0443 | 56.0486:120292 56.2347:7436 62.64165:6270 84.04312:82510 | 3.56955E-07 |
| POS3959 | Anofinic acid | 1.176 | 205.0856 | [M+H]+ | 205.0856 | 57.03287:12657 57.06897:11826 60.08025:113265 85.02801:495511 86.03025:37113 149.02058:15997 | 0 |
| POS151 | al\|Nitrile-(()-3-Hyd | 0.618 | 84.04436 | [M+H]+ | 84.0443 | 55.05365:16638 56.04861:115979 84.04314:90317 84.07956:9447 | 7.13909E-07 |
| POS672 | hyltetrahydrofuran | 5.335 | 101.05948 | [M+Na]+ | 101.0596 | 55.05366:9573 60.04327:16959 64.96264:6955 70.27892:5149 96.75728:5504 103.46888:5735 | -1.1874E-06 |
| POS243 | Butanal, 2-oxo- | 4.902 | 87.04405 | [M+CH3OH+H]+ | 87.04405 | 54.09435:6368 86.05939:24522 87.04335:10728 | 0 |

**Differences in metabolites between the WJW group, the Model group and the Control group**

| Alignment ID | Metabolite name | Rt(min) | Expreiment Mz | Adduct type | Reference m/z | MS/MS spectrum | PPM |
|---|---|---|---|---|---|---|---|
| POS1740 | ydroxyanthranilic | 3.644 | 136.03891 | [M+H-H2O]+ | 136.039 | 55.93435:66812 71.92825:19297 72.93706:64380 86.21218:5189 89.93904:6074 90.94762:18292 91.05389:29653 108.99001:5988 136.02156:10530 136.05905:19517 | -6.6157E-07 |
| POS336 | L-Alanine | 2.49 | 90.05499 | [M+H]+ | 90.05498 | 61.02742:736144 62.03179:24408 72.04368:7129 72.08047:7254 | 1.11043E-07 |
| POS7346 | .3Z-Docosenamid | 6.315 | 338.34091 | [M+H]+ | 338.341 | 50.33126:5881 111.81992:5735 173.39104:5693 176.81448:5137 196.18338:6242 | -2.66E-07 |
| POS6847 | MG(16:0/0:0/0:0) | 6.322 | 313.27203 | [M+H]+ | 313.272 | 57.06987:13058 92.41432:6239 110.36397:5778 173.39598:13388 214.98621:5459 260.67572:6780 | 9.57634E-08 |
| POS6454 | -6,10,14-trimethyl | 5.097 | 297.2413 | [M+H]+ | 297.2413 | 55.05364:12699 57.06895:78902 67.05388:29424 69.0699:17559 69.81181:6200 71.08546:34550 81.06831:23723 83.08508:9349 84.08121:5730 85.10043:19462 86.09538:15480 89.05894:5805 95.08475:31751 95.21631:6185 104.10694:13999 109.10004:8792 172.32704:5568 | 0 |
| POS330 | Lactamide | 1.48 | 90.05469 | [M+H]+ | 90.05478 | 61.02742:529022 62.03075:13726 72.08047:8393 76.49201:5079 78.29926:5515 | -9.9939E-07 |
| POS1868 | BETAINE | 1.318 | 140.0679 | [M+H]+ | 140.06787 | 67.05389:6287 93.9344:5387 94.06358:19246 112.03867:7627 | 2.14182E-07 |
| POS4446 | Aminoanthraquin | 1.523 | 223.0631 | [M+H]+ | 223.063 | 58.06416:18318 59.04858:9237 61.03971:21594 73.02811:13800 73.04688:17204 89.05895:7088 91.05576:21940 93.07033:9530 119.04705:6844 209.00751:10711 225.04042:94361 | 4.48304E-07 |
| POS895 | octa-1,3,6-triene | 9.574 | 109.10102 | [M+H]+ | 109.1011 | 67.05386:20167 67.51138:5484 70.40051:5189 72.86618:6126 72.96379:5746 73.08709:7201 74.07662:5161 79.60238:5924 87.0032:8411 109.07554:8961 111.96735:6002 | -7.3326E-07 |
| POS593 | lethyl-2(5H)-furan | 9.001 | 99.04386 | [M+H]+ | 99.044 | 52.14494:6029 53.03854:7984 55.93433:11867 57.06894:21089 72.93703:45790 89.961:6771 90.94759:7406 98.9841:14208 | -1.4135E-06 |
| POS61 | Benzene | 3.996 | 79.05422 | [M+H]+ | 79.0542 | 51.36534:4811 51.44291:4633 51.60176:4579 55.41333:5178 59.86081:4222 60.2205:3878 61.02845:11184 61.59448:5402 61.78178:4911 64.48734:4628 68.45152:4331 70.77499:5100 71.3941:5594 76.17404:3930 78.70107:4524 | 2.52991E-07 |
| POS187 | -butenoic acid ga | 9.595 | 85.02846 | [M+Na]+ | 85.02838 | 53.01286:15435 56.04862:137267 67.05389:6919 84.04315:124379 84.07957:15886 85.02799:6883 85.04652:10727 | 9.40862E-07 |
| POS5118 | H-Leu-Asp-OH | 1.668 | 247.12817 | [M+NH4]+ | 247.1282 | 61.03972:6894 72.08047:42287 84.04316:21456 84.07957:9799 85.02799:14644 118.08494:10756 171.03236:5870 187.84866:5711 | -1.2139E-07 |
| POS181 | -butenoic acid ga | 9.903 | 85.02835 | [M+H]+ | 85.02838 | 53.77023:6062 55.05363:6463 56.0486:93024 84.04312:65379 | -3.5282E-07 |
| POS446 | 2-Methylpyridine | 3.062 | 94.06503 | [M+H]+ | 94.065 | 53.25908:4990 57.03286:6170 95.04893:8674 | 3.18928E-07 |
| POS930 | -propan-2-ylpyrro | 8.752 | 110.09641 | [M+H]+ | 110.0963 | 51.58027:6929 57.9342:33504 71.94005:11487 74.99706:25631 86.50518:14018 87.00323:7748 88.00347:108227 97.00917:47521 99.51075:24002 108.51748:9324 112.96638:11638 | 9.99125E-07 |
| POS631 | Pent-4-enamide | 2.585 | 100.07546 | [M+H]+ | 100.0756 | 55.01771:9721 55.05366:19322 58.02808:7424 70.41451:5645 76.19833:5992 98.98415:14159 | -1.3989E-06 |
| POS3955 | Eriosematin F | 1.524 | 205.08527 | [M+H]+ | 205.0854 | 57.03287:14839 57.06989:12885 60.08025:91444 85.028:404395 86.03025:32870 121.02692:7917 149.02058:22586 | -6.3388E-07 |

**Differences in metabolites between the WJW group, the Model group and the Control group**

| Alignment ID | Metabolite name | Rt(min) | Expreiment Mz | Adduct type | Reference m/z | MS/MS spectrum | PPM |
|---|---|---|---|---|---|---|---|
| POS8047 | -hydroxycrambesc | 5.802 | 376.25919 | [M+NH4]+ | 376.2594 | 57.06896:980735 58.07272:16000 80.38296:5747 83.32634:5984 125.07162:16895 126.05367:46383 209.12439:64788 210.11511:10225 218.12764:23013 219.11343:8727 275.17361:7848 292.19846:9008 293.18784:16395 297.95679:5695 302.18762:21385 343.06805:6336 376.26254:23085 | -5.5813E-07 |
| POS8735 | Nafoxidine | 4.614 | 426.24298 | [M+2H]2+ | 426.24277 | 55.05365:50348 59.04858:49053 61.02845:31074 67.05389:20798 69.03294:835472 70.0366:21185 71.04945:25943 73.02811:207204 73.06432:84852 78.03804:39467 80.05429:60806 81.03229:27254 81.0699:14234 83.04932:143834 85.02799:23662 87.04334:669015 88.04782:18084 89.05896:2239860 90.0619:79698 91.04643:17202 95.04893:43332 99.04339:386962 100.05018:102858 102.06606:49288 103.03849:20744 107.06821:25936 109.06336:23486 111.04441:191889 113.05927:53207 117.09089:26953 122.06293:147835 122.56553:16009 125.0596:38296 129.05446:69540 131.07088:66151 133.08543:619741 134.09018:31549 137.0598:20853 144.07597:61997 155.07083:74452 166.08865:23580 173.07771:22267 177.11285:50953 | 4.92677E-07 |
| POS13902 | )-dioic acid 29-me | 4.652 | 677.38928 | [M+2H]2+ | 677.38953 | 69.03294:73562 73.02811:197486 73.06432:65878 81.03229:39471 82.00758:33699 83.04932:46218 87.04334:562341 89.05896:2671234 90.0619:58131 91.04643:49146 95.04893:109715 99.04339:72157 111.04189:137158 113.05927:74273 117.09089:32831 131.07088:47371 133.08543:871621 134.09018:38962 137.0598:50855 155.07083:46456 177.11285:107687 | -3.6906E-07 |
| POS9607 | Netilmicin | 4.497 | 476.30658 | [M+H]+ | 476.30679 | 73.06431:16723 82.98758:9562 87.04333:88920 89.05895:1344325 90.0619:26633 107.52888:8753 117.09088:10341 130.08388:8904 131.06764:18442 133.08543:536390 134.09016:13500 175.09525:9280 177.11284:78839 177.44234:9902 | -4.4089E-07 |
| POS6878 | 2-methylphenyl)ch | 1.033 | 315.0018 | [M+CH3OH+H]+ | 315.00201 | 62.98163:10330 80.94772:43991 82.94373:11288 133.82373:5799 135.00177:798502 136.00449:11211 | -6.6666E-07 |
| POS1643 | THTC | 1.565 | 133.03145 | [M+H]+ | 133.03169 | 55.0177:11503 55.05365:19549 56.04863:22394 57.05693:74716 58.06512:49342 67.05389:8925 69.03294:13369 69.06868:753039 70.07312:57048 86.09541:3624676 87.09918:275236 132.10175:9428 | -1.8041E-06 |
| POS6474 | Stepharine | 8.809 | 298.14288 | [2M+H]2+ | 298.14447 | 55.05366:34647 57.06896:290541 58.07272:17207 60.08025:16111 67.05389:60108 69.06869:44183 71.08419:158150 72.08836:10216 81.06834:70186 83.0851:45791 85.10046:70404 86.09541:80791 95.08478:106599 96.08828:8833 97.09953:26441 99.07939:7069 102.09042:16028 103.09242:6131 104.10697:65978 109.10007:47414 110.10549:5637 123.11524:17088 124.99956:19890 152.28874:5179 173.39111:11392 181.02371:7528 184.07466:13722 189.81023:5380 240.09721:7437 | -5.333E-06 |

**Differences in metabolites between the WJW group, the Model group and the Control group**

| Alignment ID | Metabolite name | Rt(min) | Expreiment Mz | Adduct type | Reference m/z | MS/MS spectrum | PPM |
|---|---|---|---|---|---|---|---|
| POS10095 | alpha,6alpha,7bet | 5.146 | 499.3259 | [M+2H]2+ | 499.32571 | 57.03287:76137 59.04859:1558574 73.02812:180120 73.06432:77930 80.0543:78842 85.06506:35346 87.04335:275850 87.06079:126498 89.05896:1979272 90.06191:77094 101.05931:196932 102.06606:46237 103.07444:392972 104.10697:220163 105.10879:93223 109.0756:62332 129.08911:44561 131.07088:47159 133.08545:758990 134.09018:37677 138.09604:31302 147.10149:220400 177.11285:76722 184.06931:79134 191.1256:37782 344.14871:30997 | 3.80513E-07 |
| POS11993 | nodeoxycholylargi | 5.564 | 571.38263 | [M+2H]2+ | 571.38293 | 57.03287:54088 59.04859:910919 60.05226:26733 69.06992:16959 73.02812:81292 73.06432:36065 74.06708:9281 85.06337:30932 87.04335:141471 87.06254:28789 87.07999:32295 89.05896:563763 90.06191:22655 94.06947:26926 99.07939:13228 101.05931:142150 103.07444:250230 104.10469:18320 107.06821:11190 115.07378:18379 116.08248:9978 117.0909:23833 129.08911:15358 131.06766:15681 131.10634:9048 133.08545:194027 134.08684:17871 145.08403:13019 147.10149:105449 161.11441:12545 171.97781:9731 177.1078:10406 191.1256:13171 205.14238:9301 | -5.2504E-07 |
| POS8488 | Val Leu Tyr | 4.962 | 408.25195 | [M+CH3OH+H]2+ | 408.24921 | 59.04858:44387 87.04334:13409 89.05896:68683 95.42019:6092 101.0593:12186 103.07669:10578 133.08545:26958 | 6.71159E-06 |
| POS10966 | Roehybridine | 8.789 | 534.29517 | [M+Na]+ | 534.29541 | 60.08025:12284 65.02566:7665 86.09541:75514 104.10696:321832 105.66643:9116 112.49341:8183 | -4.4919E-07 |
| POS15389 | PC(18:2(2E,4E)/0:0) | 6.255 | 1039.67102 | [2M+H]+ | 1039.67053 | 56.0486:8800 58.06509:8734 60.08023:132038 63.92054:5359 67.05387:6755 71.07258:26955 86.09538:200052 95.08274:5910 98.98199:7908 104.10693:977993 105.11106:7441 116.77584:6002 121.29324:7112 121.79109:6253 124.9995:39813 173.38614:14816 184.07458:276262 185.0817:7575 914.8385:6452 | 4.71303E-07 |
| NEG5130 | Terezine A | 0.991 | 273.12494 | [M-H2O-H]- | 273.12469 | 87.11253:30944 89.13125:90150 93.03925:253037 95.03763:525814 97.03745:222648 145.23549:11469 147.15915:29333 153.06755:11910 155.06651:16051 | 9.15333E-07 |
| POS14049 | ethylbutyroyl),22-( | 5.172 | 689.46246 | [M+H]+ | 689.46222 | 57.03287:127973 59.04859:2489306 73.02812:97416 73.06432:81669 85.06337:72944 87.04335:451024 87.07999:84081 89.05896:2099070 99.07939:51889 101.05931:431129 103.07444:982473 115.07378:50248 117.05281:98589 117.0909:64985 131.06766:89739 133.08545:674337 145.08403:49569 147.10149:648273 161.1188:132124 173.39603:93594 177.11285:44225 191.1256:86610 205.14238:50020 355.71689:43311 | 3.48097E-07 |
| POS8879 | Suvanine | 4.698 | 435.2565 | [M+H]+ | 435.25629 | 51.50556:7580 67.73112:5330 89.05894:12640 123.58301:5278 183.53943:6587 | 4.82474E-07 |
| POS11844 | manzamine M | 5.053 | 565.35388 | [2M+H]+ | 565.35358 | 59.04858:259007 73.02811:50874 73.06431:30974 78.36626:16107 80.05274:24239 87.04333:86028 89.05894:518801 101.05929:33520 103.07442:72740 123.34731:18975 133.08542:191946 147.10147:40567 173.43033:40313 177.11282:31289 194.32018:20204 446.87405:18802 | 5.30641E-07 |

**Differences in metabolites between the WJW group, the Model group and the Control group**

| Alignment ID | Metabolite name | Rt(min) | Expreiment Mz | Adduct type | Reference m/z | MS/MS spectrum | PPM |
|---|---|---|---|---|---|---|---|
| POS8786 | Mifepristone | 4.548 | 429.267 | [M+H]+ | 429.26682 | 59.04859:101581 73.02812:9553 73.06432:14635 85.06506:7925 87.04335:104053 89.05896:1139310 90.06191:21204 101.05931:5715 103.07444:21527 107.06821:7909 129.56607:6863 131.06766:16384 133.08545:384629 134.08684:9803 147.10149:14268 154.65695:5826 177.11285:33393 287.56656:5856 | 4.1932E-07 |
| POS5025 | Agomelatine | 5.56 | 244.13278 | [M+NH4]+ | 244.133 | 67.05389:23319 69.06868:1558184 70.07312:40942 115.05522:13405 130.06477:303271 131.06766:12601 132.08218:16202 142.06566:259660 143.06737:13584 144.07968:12297 160.07417:332565 161.07927:17557 176.06961:21747 188.07004:192286 189.07663:13067 198.12756:12154 | -9.0115E-07 |
| POS11867 | ntibiotic FR 90084 | 5.929 | 566.32208 | [M+H]+ | 566.32239 | 59.04856:25629 60.08023:20202 71.07258:6320 73.02809:5629 86.09538:58974 87.04331:8500 89.05893:18621 104.10693:233342 124.9995:16304 184.07458:77241 355.55829:5667 | -5.4739E-07 |
| POS3675 | Hexylresorcinol | 4.373 | 195.12135 | [M+H]+ | 195.1216 | 58.06417:84777 89.05896:127199 95.26823:5724 107.04918:40419 133.08545:6906 135.04228:115350 136.04544:21897 194.11676:14281 | -1.2813E-06 |
| POS10211 | PE(0:0/20:3(5Z,8Z,1 | 6.328 | 504.30774 | [M+H]+ | 504.3075 | 55.84372:5639 55.87241:6045 57.03379:6226 60.15533:6699 62.05909:35627 67.05389:9008 81.0699:10454 89.05896:8405 95.08477:9910 107.08487:6805 115.67261:5821 | 4.759E-07 |
| POS12104 | a-Tocopherol succ | 5.451 | 575.36816 | [M+2H]2+ | 575.36798 | 59.04859:160598 67.015:20277 73.02812:13311 79.78577:10853 87.04335:32101 89.05896:181868 101.05931:14248 103.07444:47691 133.08545:56907 147.10149:15684 | 3.12843E-07 |
| POS3346 | phtho[2,1-b]thioph | 0.892 | 185.04167 | [M+NH4]+ | 185.0419 | 125.02051:21327 | -1.243E-06 |
| POS10477 | Ile Glu Leu Lys | 5.62 | 516.33887 | [M+2H]2+ | 516.33911 | 57.03287:26206 57.06896:9009 59.04859:219972 67.015:15101 73.02812:19965 73.06432:11710 81.03072:13328 85.06506:7129 87.04335:26028 87.07999:7319 89.05896:145585 90.06191:7778 101.05931:22095 103.07444:54508 117.05281:9146 126.02024:8756 129.08911:7557 133.08545:40360 147.10149:20887 149.97055:7802 161.11441:6022 165.10016:7441 194.85654:5915 340.77332:6237 | -4.6481E-07 |
| POS11755 | onjugated chenoc | 5.607 | 562.34882 | [M+H]+ | 562.349 | 57.97966:12413 59.04856:44377 60.08022:29247 67.61633:14164 86.09536:69963 89.05891:29635 91.32316:10827 104.10692:295921 105.11105:15458 184.07457:89546 | -3.2009E-07 |
| POS12674 | Gly Asn Leu Arg Ly | 5.094 | 601.37762 | [M+CH3OH+H]+ | 601.37793 | 57.03287:14339 59.04859:165112 73.02812:14904 73.06432:10785 87.04335:701428 89.05896:178052 101.05931:156728 103.03849:51479 103.07444:41422 115.07378:12363 117.05281:35948 131.06766:17236 133.08545:55064 147.10149:20260 217.73381:9698 | -5.1548E-07 |
| POS12835 | I9-Oxide ; Paecilot | 5.672 | 608.91724 | [M+2H]2+ | 608.91699 | 59.04859:265532 73.02812:20811 85.06337:17145 87.04335:54346 87.06079:14047 89.05896:228246 101.05931:63835 103.07444:93415 133.08545:72130 147.10149:36558 164.48682:14678 | 4.10565E-07 |
| POS10700 | Calcimycin | 6.108 | 524.27417 | [M+Na]+ | 524.27399 | 57.03286:1100542 58.03661:42022 59.04858:301784 60.08024:29131 73.58075:13876 81.06989:33230 86.0954:87303 99.07938:87036 101.09422:19774 104.10696:303568 115.07377:73317 117.09088:21560 157.11888:72431 173.43033:28487 184.06927:115013 462.50906:13869 522.58624:12674 | 3.43332E-07 |

**Differences in metabolites between the WJW group, the Model group and the Control group**

| Alignment ID | Metabolite name | Rt(min) | Expreiment Mz | Adduct type | Reference m/z | MS/MS spectrum | PPM |
|---|---|---|---|---|---|---|---|
| POS12148 | PA(15:1(9Z)/12:0) | 5.744 | 577.38403 | [M+2H]2+ | 577.38422 | 55.29747:20168 57.03287:71034 59.04859:217476 67.07867:19889 73.02812:56650 85.06337:37893 87.04335:72367 87.06079:26980 89.05896:315632 101.05931:70320 103.07444:46810 129.08911:41048 133.08545:103642 147.10149:30193 173.38622:22558 | -3.2907E-07 |
| POS8252 | Metazin | 4.667 | 391.22971 | [M+Na]+ | 391.22989 | 73.02812:6638 89.05896:17041 133.08545:9736 | -4.6009E-07 |
| POS11572 | Muscanone | 5.775 | 555.36932 | [M+2H]2+ | 555.36951 | 55.05366:11162 57.03287:75288 59.04859:163324 65.04931:8099 73.02812:47099 73.06432:9453 85.06337:39725 87.04335:75640 87.06254:15720 87.07999:16998 89.05896:198155 90.06191:8464 101.05931:57031 103.07444:31080 109.0756:6983 115.07378:7632 129.09227:12419 133.08545:57503 143.10413:8150 147.10149:12617 552.71912:6996 | -3.4211E-07 |
| POS13394 | cynthiaxanthin ac | 6.478 | 640.41254 | [M+H]+ | 640.41278 | 57.03287:116401 59.04859:51566 72.64896:22764 73.15427:20225 86.09541:26475 95.44221:21301 165.65282:20413 173.43034:39705 575.79932:22827 | -3.7476E-07 |
| NEG6716 | Thr Pro Asn | 1.086 | 329.14691 | [M-H]- | 329.1467 | 87.11256:12794 89.13128:25800 93.03928:344289 95.03767:312418 97.03749:31470 103.08175:9911 130.59087:5960 145.14165:6423 145.23553:7752 151.06717:7590 153.0676:22636 182.01541:5827 | 6.38013E-07 |
| NEG9755 | TAN-1496 E | 6.476 | 591.06091 | [M-H]- | 591.06061 | 79.0522:6446 79.66678:5497 | 5.07562E-07 |
| POS7358 | norpregna-1,3,5,7 | 4.714 | 339.19522 | [M+H]+ | 339.1954 | 57.03287:11041 59.04859:260958 72.71816:7359 73.02812:6847 87.04335:270072 88.04605:5926 89.05896:78140 101.05931:32140 103.03849:103628 103.07444:35871 117.05281:30389 131.06766:6514 133.08545:6862 147.10149:6776 290.02237:5411 | -5.3067E-07 |
| POS3669 | 01!(E)-4-hydroxyr | 0.887 | 195.09877 | [M+NH4]+ | 195.099 | 82.34032:6379 82.37885:6615 88.44839:5652 162.27571:6158 173.43031:6117 | -1.1789E-06 |
| POS5232 | Paucine | 4.416 | 251.13805 | [M+NH4]+ | 251.13831 | 69.03294:10049 72.77946:5754 73.02945:6132 86.05938:17064 88.03894:18168 101.0593:5852 103.05421:11782 120.04356:14019 120.08029:37074 123.67455:5556 131.04831:6292 147.0555:16864 171.53304:5898 188.10326:14079 | -1.0353E-06 |
| POS11802 | Rhodoxanthin | 7.672 | 563.39111 | [M+2H]2+ | 563.39081 | 82.57995:5554 136.10338:5762 136.28088:7128 345.74472:6147 | 5.3249E-07 |
| POS680 | Homopiperazine | 6.518 | 101.10713 | [M+H]+ | 101.10732 | 51.99327:6998 70.19422:6915 101.10733:5472 | -1.8792E-06 |
| POS3872 | metric dimethylarg | 5.069 | 202.14316 | [M+H-H2O]+ | 202.14296 | 54.00388:6561 55.05365:108846 57.06895:16962 69.06867:15266 76.7874:5737 97.09953:36648 97.24764:5777 101.0593:6591 114.12527:932384 115.12946:22465 130.06476:5500 156.13799:21795 160.13072:36610 | 9.89399E-07 |
| NEG4747 | ecyloxypropane-1 | 4.153 | 259.22815 | [M-H]- | 259.22791 | 53.9787:5890 59.0295:9226 60.40223:5661 64.27615:5905 70.36988:6031 92.55002:6256 103.07276:30071 | 9.25826E-07 |
| NEG2216 | Glycylproline | 8.782 | 171.07729 | [2M-H]- | 171.07751 | 56.02806:6761 56.26738:6083 61.21238:5515 84.03104:7884 109.19517:7395 136.52611:6194 | -1.286E-06 |
| POS506 | ,2-Dichloroethylen | 0.128 | 96.96085 | [M+H-H2O]+ | 96.9606 | 50.63479:6013 55.0177:9152 55.05365:24431 55.93344:20500 65.42387:6768 68.04949:8679 69.04403:10401 96.04378:19276 97.03996:9028 97.0646:9691 | 2.57837E-06 |
| POS502 | ,2-Dichloroethylen | 9.291 | 96.96079 | [M+H]+ | 96.9606 | 53.92981:6138 55.05365:20051 55.93435:14013 68.0495:6645 69.04403:7150 84.06301:5123 95.95288:6106 96.04378:13152 97.0379:12825 97.0646:7771 | 1.95956E-06 |

**Differences in metabolites between the WJW group, the Model group and the Control group**

| Alignment ID | Metabolite name | Rt(min) | Expreiment Mz | Adduct type | Reference m/z | MS/MS spectrum | PPM |
|---|---|---|---|---|---|---|---|
| POS1657 | t-butyl propyl sulf | 1.18 | 133.10469 | [M+H]+ | 133.10451 | 57.05693:13463 58.06512:8432 61.01003:14805 69.06869:113703 70.07313:16056 86.09541:579528 87.02591:7053 87.09918:66588 90.05457:23341 | 1.35232E-06 |
| POS1160 | -Ethylbutanoic aci | 4.938 | 117.09067 | [M+CH3OH+H]+ | 117.0909 | 55.05365:34633 70.0643:105869 71.61486:6049 72.08047:36936 98.5709:5404 103.54803:5433 | -1.9643E-06 |
| POS3201 | 80638-48-8 | 5.379 | 181.08533 | [M+Na]+ | 181.08549 | 135.07935:33604 | -8.8356E-07 |
| POS302 | Acetoin | 5.447 | 89.05939 | [M+H]+ | 89.0596 | 55.93345:7048 61.02845:15928 72.93707:41035 85.18988:5897 90.94763:7938 | -2.358E-06 |
| POS1482 | Galegine | 6.532 | 128.11792 | [M+Na]+ | 128.1181 | 58.06512:7710 62.30235:5532 71.10865:5107 88.24155:5415 128.11816:6668 | -1.405E-06 |
| POS157 | al\|Nitrile-(()-3-Hyc | 1.136 | 84.0445 | [M+NH4]+ | 84.0443 | 55.05365:24761 56.04862:287729 65.03804:6148 66.17352:6014 67.05389:15938 69.05635:7034 80.31335:6038 82.06502:31476 82.94376:8824 83.06069:10527 84.04315:222538 84.07957:435292 | 2.3797E-06 |
| POS929 | Brunfelsamidine | 0.855 | 110.07095 | [M+H]+ | 110.0712 | 55.55091:5496 56.04953:7725 62.15791:5484 68.98124:8315 81.96613:5329 86.99279:13087 88.0851:5737 100.02225:7363 105.0024:22770 | -2.2713E-06 |
| NEG1147 | ;_LEUCINE-5,5,5-D | 1.191 | 133.10646 | [M-H]- | 133.10622 | 59.08403:32480 71.09898:515055 73.07996:114427 75.09879:9345 79.05367:981327 79.06424:33752 87.11253:6641 88.14607:14457 89.13124:43366 97.08466:1245166 115.14149:329179 133.05977:14749 133.17186:63786 133.20816:7937 | 1.80307E-06 |
| NEG932 | imethylthiazol-2-a | 8.995 | 127.03379 | [M-H]- | 127.0336 | 53.92936:5989 65.28221:6656 | 1.49567E-06 |
| POS1854 | yl-2,5-dimethylpy | 6.462 | 139.12263 | [2M+H]+ | 139.12289 | 55.0291:14994 57.9342:17711 58.06417:68347 69.06869:10360 70.06431:14638 83.05908:18365 96.08018:7258 116.97126:9458 122.0948:18950 139.12291:11213 | -1.8689E-06 |
| POS3554 | bicyclo[4.3.0]nona | 7.201 | 191.14226 | [M+H]+ | 191.1425 | 55.68453:6124 140.74756:6479 | -1.2556E-06 |
| POS1025 | I-imidazol-5-yl)et | 6.475 | 113.07066 | [M+H]+ | 113.0709 | 54.03372:77373 59.92951:49646 61.927:7501 67.05389:20078 69.04403:443480 70.02779:36752 70.0643:9931 71.05973:242969 72.06339:12234 73.56719:6193 86.94053:6018 89.00122:68090 89.50283:10153 89.99953:24537 94.06554:8025 96.0074:17692 98.00586:131554 98.50787:22995 99.00529:32533 100.50954:32851 101.50806:6877 107.01112:12740 109.51484:20907 110.51353:8603 113.03345:19640 113.06959:128467 | -2.1226E-06 |
| POS735 | yne\|Ethynylbenzer | 3.787 | 103.05397 | [M+H]+ | 103.0542 | 53.01283:7468 56.0486:15841 57.03284:73042 60.04423:58404 61.0274:97448 85.02795:45406 95.04889:10935 102.05493:8132 103.03844:9101 | -2.2318E-06 |
| POS1481 | Galegine | 8.094 | 128.11789 | [M+H]+ | 128.1181 | 51.12348:4462 54.13879:4788 54.93365:4623 56.42904:4269 56.67014:5177 58.83768:4200 61.44732:4207 63.79562:5265 70.97746:5200 73.28477:4825 78.06468:6157 87.17079:5211 102.15912:5638 103.44626:4638 | -1.6391E-06 |
| POS3553 | cid, 3a,4,5,6,7,7a-h | 7.296 | 191.14224 | [M+H]+ | 191.142 | 55.69256:6028 60.02228:5774 67.29036:4872 70.4183:5808 | 1.25561E-06 |
| POS2 | 1-Propene-1-thio | 0.711 | 75.02599 | [M+H]+ | 75.0262 | 50.26234:6873 50.31902:6093 50.34586:4342 52.05128:4041 52.05774:5519 53.90685:4830 53.93237:6118 53.96725:5347 55.43372:4405 56.04863:5089 57.03379:6323 57.98443:4912 57.99202:4277 60.20647:4843 60.25968:5493 60.31799:5181 62.54278:4039 62.55872:5808 62.6055:4660 63.19151:4160 65.00201:4659 67.59607:4722 70.19422:3861 70.68302:4177 73.4035:4236 73.95222:4955 74.05886:5136 74.43671:4852 75.43698:4801 77.62954:4853 | -2.799E-06 |

**Differences in metabolites between the WJW group, the Model group and the Control group**

| Alignment ID | Metabolite name | Rt(min) | Expreiment Mz | Adduct type | Reference m/z | MS/MS spectrum | PPM |
|---|---|---|---|---|---|---|---|
| POS7653 | | 9.493 | 353.34113 | [M+CH3OH+H]+ | 353.34131 | 57.06989:8454 59.03007:18218 61.01003:11569 73.04688:11070 75.02497:19953 77.04092:10995 91.05576:16604 93.03564:21453 105.07176:6055 154.15424:5573 168.61324:5526 | -5.0942E-07 |
| POS2116 | -3,4-dimethylpent | 4.537 | 147.10121 | [M+NH4]+ | 147.1015 | 55.05366:16159 59.04859:11035 64.92667:10670 71.04816:8067 72.08048:7752 84.07957:11127 86.05939:7596 90.46526:5589 91.0539:13732 118.0629:12580 | -1.9714E-06 |
| POS9970 | Kermadecin C | 4.808 | 493.29453 | [M+H]+ | 493.2948 | 89.05896:14232 111.75651:8839 150.5957:6680 | -5.4734E-07 |
| POS7205 | Gly Arg Val | 4.894 | 331.20853 | [M+Na]+ | 331.2088 | 50.59146:6949 89.05891:7436 173.42535:7672 186.45195:6156 | -8.152E-07 |
| POS8912 | Drenison | 4.493 | 437.233 | [M+Na]+ | 437.23331 | 52.80128:6734 68.74682:5393 246.60245:5675 301.61307:5685 | -7.09E-07 |
| POS12827 | Roseocardin | 5.425 | 608.40125 | [M+2H]2+ | 608.40088 | 57.03287:54218 59.04859:969901 60.05226:32435 73.02812:94965 73.06432:38505 75.78008:13408 80.0543:18675 83.0477:14981 85.06337:49265 87.04335:169178 87.06079:40880 87.07999:43729 89.05896:1091298 90.06374:33013 94.06947:26629 101.05931:158968 103.07444:259107 116.08248:23405 117.0909:25837 129.08911:31419 131.06766:21780 133.08545:392229 134.08684:16872 147.10149:119249 161.11441:15337 177.11285:36405 191.1256:26122 | 6.08152E-07 |
| NEG2882 | pholino)-ethanesu | 1.208 | 194.04869 | [M-H]- | 194.0484 | 59.08403:9421 79.05367:22876 80.05276:8272 87.29256:5457 97.07645:5085384 159.03728:26253 | 1.49447E-06 |
| POS10867 | Ile Leu Ile Gly Thr | 5.137 | 530.35498 | [M+H]+ | 530.35468 | 59.04859:273368 73.02812:26540 73.06432:14036 87.04335:261049 87.06079:11436 89.05896:394889 90.06191:10841 101.05931:98209 103.03849:10424 103.07444:82931 117.05281:13607 131.06766:20291 133.08545:163177 147.10149:53517 177.11285:17248 191.13127:16924 200.78329:9637 | 5.65659E-07 |
| POS5104 | oxyacetaminopher | 1.038 | 247.01546 | [M+NH4]+ | 247.01511 | 62.98163:7152 64.72823:6041 101.51242:6272 104.10692:24282 134.01678:5969 135.00177:247258 | 1.41692E-06 |
| POS2118 | -3,4-dimethylpent | 5.027 | 147.10121 | [M+H]+ | 147.1015 | 55.05366:11502 56.04773:6884 59.04859:11893 64.92667:21080 67.82102:6960 69.06869:7496 70.45259:6235 72.08048:7240 76.67482:6051 86.05939:8729 91.0539:9974 100.07383:8196 118.0629:19497 130.08708:5411 | -1.9714E-06 |
| POS8878 | Balaenonol | 4.701 | 435.25327 | [M+Na]+ | 435.25299 | 89.05894:16135 133.08542:6624 159.1517:5039 163.68777:5218 252.45139:5891 | 6.43304E-07 |
| POS11213 | Val Ile Pro Lys Ser | 5.146 | 543.35034 | [M+2H]2+ | 543.34998 | 59.04858:120187 73.02811:23326 80.05428:13558 87.04333:28569 87.06078:11620 89.05894:233452 101.05929:19089 102.06604:7752 103.07442:30258 131.06763:8023 133.08542:75491 147.10146:16462 173.42542:14482 285.71133:8254 | 6.62556E-07 |
| POS13154 | -3,4-dehydro-apo | 4.842 | 625.37299 | [M+Na]+ | 625.37341 | 585.47443:14342 | -6.716E-07 |
| POS13470 | lbutanoyl)-13-O-( | 5.195 | 645.43555 | [M+H]+ | 645.43597 | 59.04859:242310 66.66176:9597 73.02812:18954 87.04335:24629 89.05896:120566 101.05931:29662 103.07444:54756 117.05281:11269 133.08545:31894 147.10149:17486 155.30751:11078 599.66156:11795 | -6.5072E-07 |
| POS10854 | Ala Ala Gln Asn Leu | 4.468 | 530.29352 | [M+2H]2+ | 530.29321 | 65.25937:5692 70.02776:17986 70.06428:5914 86.09537:38615 87.05377:74064 88.03892:7436 89.05892:49134 107.32559:6541 122.51595:5492 133.08539:30002 146.05714:7555 172.06981:10437 188.06996:26492 205.09813:8059 344.03888:5638 | 5.84582E-07 |

**Differences in metabolites between the WJW group, the Model group and the Control group**

| Alignment ID | Metabolite name | Rt(min) | Expreiment Mz | Adduct type | Reference m/z | MS/MS spectrum | PPM |
|---|---|---|---|---|---|---|---|
| POS9165 | Cytochalasin II | 4.969 | 452.27783 | [M+CH3OH+H]2+ | 452.2775 | 52.35993:6926 59.04859:26203 87.04335:17260 89.05896:52371 101.05931:18825 133.08545:17198 173.43036:7924 321.76129:5831 | 7.29641E-07 |
| POS7897 | yltricaprylylammo | 6.231 | 368.42371 | [M+H]+ | 368.42401 | 53.41133:6012 57.06989:9046 60.08025:22375 71.08418:8467 126.59031:6735 | -8.1428E-07 |
| POS8027 | Acetoxypregnenol | 5.196 | 375.1463 | [M+CH3OH+H]+ | 375.14603 | 55.01768:11003 57.06894:23731 59.04856:18964 83.01192:36653 85.06333:16538 89.05892:15921 115.03928:16748 171.09958:11834 | 7.1972E-07 |
| POS2503 | lethylene-L-glutar | 2.278 | 159.07599 | [M+H]+ | 159.07629 | 55.05365:7750 55.93345:25019 56.04862:12251 61.03972:9412 62.29601:5395 67.05389:13226 70.0643:38752 72.93572:6851 81.07617:6065 95.08477:17780 115.05257:5547 117.05824:7372 | -1.8859E-06 |
| POS11453 | nino)-3beta-phtha | 4.923 | 551.34827 | [M+2H]2+ | 551.3479 | 50.11341:5893 61.73277:5959 89.05892:11118 104.10693:18044 104.21423:6262 133.08539:5544 | 6.71083E-07 |
| POS12812 | lloxanthin 3-acetat | 5.801 | 607.41406 | [M+2H]2+ | 607.41449 | 57.03287:71227 59.04859:547655 69.06868:16950 73.02812:65250 73.06432:23441 85.06337:27886 87.04335:75830 87.07999:18021 89.05896:323129 94.06947:22078 101.05931:67832 103.07444:165300 115.07643:17031 117.0909:26454 129.08911:15801 131.10312:15080 133.08545:87414 147.10149:65990 154.42589:15842 161.1188:17895 568.3938:14728 | -7.0792E-07 |
| POS9908 | Mmv676063 | 5.958 | 490.28867 | [M+Na]+ | 490.289 | 59.04859:7609 60.08025:7720 69.77051:5489 86.09541:33568 104.10697:183850 146.97896:17678 190.10825:6852 345.91049:5415 | -6.7307E-07 |
| POS13396 | propan-2-yl)-1,7,1 | 5.778 | 640.41736 | [M+2H]2+ | 640.41699 | 57.03287:18330 59.04859:110224 73.02812:21293 73.06432:10910 85.06506:9571 87.04335:33746 87.06254:11364 89.05896:105997 101.05931:33457 103.07445:20257 117.05281:10544 117.0909:9995 133.08545:43526 147.10149:14077 173.38622:9218 213.08313:8803 280.85791:9059 | 5.77749E-07 |
| POS7300 | Pipericine | 6.74 | 336.32523 | [M+H]+ | 336.3255 | 50.50196:6109 112.50879:5338 156.31415:6028 218.8768:6308 | -8.0279E-07 |
| NEG9902 | Oligomycin F | 8.8 | 803.53241 | [M-H]- | 803.53162 | 105.20542:5352 116.96082:5538 120.79679:5685 146.04282:5948 302.20255:6442 | 9.8316E-07 |
| POS6713 | rophenyl)naphtha | 2.993 | 308.08005 | [M+2H]2+ | 308.07971 | 56.04862:7136 59.54773:6225 74.0055:5790 76.0215:8827 76.03858:24778 84.04315:132124 85.04819:7381 130.04883:46154 131.04509:11298 | 1.10361E-06 |
| POS6641 | Fenpropimorph | 0.898 | 304.26031 | [M+NH4]+ | 304.26059 | 52.64424:6420 73.14485:5706 115.68862:6183 306.7052:6369 | -9.2026E-07 |
| POS4707 | carnitine (isomer | 2.62 | 232.15359 | [M+H]+ | 232.1539 | 51.53652:7216 57.06986:11963 60.08022:18059 73.56039:5627 81.03226:7065 85.02796:114643 167.10455:17058 193.27718:5915 | -1.3353E-06 |
| POS3967 | Dihydroxydecanoic | 5.167 | 205.14304 | [M+H]+ | 205.14339 | 59.04859:23058 89.05896:11043 146.061:6498 | -1.7061E-06 |
| POS3968 | Dihydroxydecanoic | 5.045 | 205.14304 | [M+2H]2+ | 205.14339 | 53.84231:6575 54.14479:6940 88.4502:5964 102.45471:5068 108.92896:6128 110.70092:5999 173.43036:7271 | -1.7061E-06 |
| NEG1818 | ndoleacetaldehyde | 1.412 | 158.06174 | [M-H]- | 158.0614 | 51.81568:5803 58.04419:57416 59.08405:10121 68.85773:6177 71.09771:5955 89.13128:114845 90.1349:6742 99.78008:6799 104.08418:5829 | 2.15106E-06 |
| POS747 | lydroxy-2-pentan | 6.013 | 103.075 | [M+H]+ | 103.0753 | 59.04859:14124 61.0387:35706 | -2.9105E-06 |
| POS369 | p-Cresol | 3.189 | 91.05404 | [M+H]+ | 91.0537 | 50.38117:6380 61.02845:9666 62.0318:14637 64.66679:6087 | 3.73406E-06 |
| POS10761 | eryl-tyrosyl-leucyl- | 6.064 | 526.29065 | [M+H]+ | 526.29108 | 55.05363:43554 57.03284:78227 62.05907:419133 62.19157:40989 67.05386:109530 79.05309:72262 81.06987:91738 91.05386:77026 93.06837:65164 98.98411:42704 104.10693:54144 105.06941:73745 105.22224:45939 119.08607:63324 131.08371:45324 173.39104:98496 | -8.1704E-07 |

**Differences in metabolites between the WJW group, the Model group and the Control group**

| Alignment ID | Metabolite name | Rt(min) | Expreiment Mz | Adduct type | Reference m/z | MS/MS spectrum | PPM |
|---|---|---|---|---|---|---|---|
| POS8037 | l!21-Acetoxypregr | 4.957 | 375.24762 | [M+H]2+ | 375.24799 | 57.03287:52016 58.04042:47946 59.04859:1374553 60.05226:30239 65.04819:56027 69.06992:15300 73.02812:258559 73.06432:31160 80.0543:63820 85.06337:34716 87.04335:197259 87.06079:104272 87.07999:20165 89.05896:1562559 90.06191:56718 94.06947:24597 101.05931:83424 102.06606:31793 103.07444:194860 104.07732:12680 107.07059:12543 109.07315:48915 116.08248:10905 124.08022:10182 129.08911:28066 131.06766:18310 131.08699:17165 133.08545:442284 134.09018:16067 147.10149:67507 173.42545:13363 177.1078:37213 191.1256:10836 | -9.8601E-07 |
| NEG3335 | Dimethyl-9H-xant | 0.972 | 209.09691 | [M-H2O-H]- | 209.09731 | 59.08403:16279 71.09898:6542 89.13125:14394 93.03925:1463321 97.03745:18826 97.07645:21359 131.2403:46894 | -1.913E-06 |
| POS12382 | Ganoderic acid Mc | 5.054 | 587.39465 | [M+H]+ | 587.39423 | 57.03286:51786 59.04858:891911 60.05225:20113 73.02811:19863 87.04333:179564 87.07997:23421 89.05894:500972 100.07381:17644 101.05929:119738 103.03848:19811 103.07442:257044 117.09087:24642 129.09224:14715 130.08388:13756 133.08542:91030 147.10147:107219 161.11438:27688 191.12556:17070 203.61127:13223 247.26077:12099 | 7.15022E-07 |
| POS8033 | paragylasparagyllys | 4.88 | 375.19821 | [M+H]+ | 375.19861 | 59.04859:47938 73.02812:17314 79.26034:5756 87.04335:17922 87.06254:7271 89.05896:59470 101.05931:8757 103.0385:20065 131.06766:5695 133.08545:12704 168.01765:6012 185.73782:5824 311.60825:5348 374.63766:5259 | -1.0661E-06 |
| POS10474 | ;(18:4(6Z,9Z,12Z,15 | 4.654 | 516.30945 | [M+2H]2+ | 516.30902 | 73.02811:42732 73.06432:11415 80.05429:34236 87.04334:48191 89.05896:279765 90.06374:11849 91.04643:6372 102.06606:18262 124.08022:9198 133.08543:106621 162.54245:7142 177.11285:11255 309.0488:8241 | 8.32835E-07 |
| POS5829 | ylquinoline 1-oxide | 4.868 | 274.18237 | [M+NH4]+ | 274.18201 | 55.05365:155349 57.03287:30183 57.06896:44646 61.01003:30787 67.05389:217736 68.05673:8080 69.06868:142022 71.04816:36076 79.05312:36470 81.06833:454771 82.07301:19347 83.0477:24995 83.08509:56492 85.00948:11202 85.06337:16910 91.0539:49244 91.93919:5980 93.06841:63061 95.08477:403021 96.08827:17496 97.06461:18138 97.09953:30213 105.06945:67244 106.07349:6510 107.08486:32951 109.06336:18807 109.10006:28658 111.0796:6349 113.05927:8230 115.07378:6267 117.06913:5785 119.08333:131978 120.08878:8567 121.1013:42594 123.08004:34238 127.01965:9520 127.075:10520 133.09863:54900 135.11646:87970 137.09427:11368 137.1322:413534 138.13437:30462 151.14491:5836 161.13197:163509 162.1382:11314 169.10333:28795 173.39111:12062 179.13977:50426 | 1.313E-06 |
| POS8643 | scutione | 4.921 | 419.25839 | [M+H]+ | 419.258 | 57.03287:7353 59.04859:32413 73.02812:7236 87.04335:17096 89.05896:45059 101.05931:9469 103.0385:17268 124.16041:5615 133.08545:14093 173.42545:10300 303.42111:5687 317.40564:5096 | 9.30215E-07 |
| POS13578 | ethyl-5-methylide | 5.052 | 653.40283 | [M+H]+ | 653.40234 | 59.04859:22567 70.18664:14842 87.04335:20137 89.05896:97236 95.55244:12756 131.36464:13900 133.08545:17226 268.88205:15103 618.28107:17286 | 7.49921E-07 |

**Differences in metabolites between the WJW group, the Model group and the Control group**

| Alignment ID | Metabolite name | Rt(min) | Expreiment Mz | Adduct type | Reference m/z | MS/MS spectrum | PPM |
|---|---|---|---|---|---|---|---|
| POS11926 | hyl-2-[6-(3-methy | 6.186 | 568.4364 | [M+H]+ | 568.43597 | 55.4754:85672 57.03287:352190 58.06511:125360 59.04858:93726 60.08025:1130365 67.05389:84753 69.93349:73463 71.0726:139182 81.06833:84518 86.0954:2759882 87.09918:96145 93.07034:89869 104.10696:12050610 105.10878:331944 109.23234:74300 124.99955:658444 175.14503:80713 184.07465:4111756 185.07634:120884 | 7.56462E-07 |
| POS10623 | Phe Val Lys Lys | 8.815 | 521.34406 | [M+2H]2+ | 521.34448 | 60.08022:28979 86.09537:76667 87.09914:6393 104.10693:159350 105.10874:18973 124.9995:13415 159.69203:5840 173.42537:6516 184.06921:172363 318.78229:6125 | -8.0561E-07 |
| POS14708 | Muronic acid | 5.168 | 750.51654 | [2M+H]+ | 750.51599 | 59.04859:215859 73.06432:32288 87.04335:32286 89.05896:250885 92.87973:18245 101.05931:39315 101.62239:17896 103.07445:71098 117.05281:55112 131.07088:20529 133.08545:96551 147.10149:54381 204.50024:21937 | 7.32829E-07 |
| POS12580 | ethylanhydrovilang | 5.437 | 597.38049 | [M+2H]2+ | 597.38 | 59.04859:141038 67.015:20370 73.02812:16096 81.03072:18390 87.04335:27664 88.08155:12357 89.05896:193138 90.06191:13491 101.05931:41205 103.07444:39391 133.08545:75424 147.10149:13880 194.0645:14267 | 8.20248E-07 |
| POS11348 | Eurycomaoside | 6.062 | 548.27057 | [M+Na]+ | 548.27014 | 53.59624:5870 59.04858:26783 89.05896:10466 91.0539:6945 104.10696:15133 120.96689:22884 | 7.84285E-07 |
| POS4239 | Citric acid | 1.219 | 215.01562 | [M+H-H2O]+ | 215.01601 | 52.46179:6029 69.16247:5332 72.08047:8938 75.66121:6218 81.0699:19912 88.72058:5424 95.08477:6798 | -1.8138E-06 |
| POS12323 | 8a-pentamethyl-4 | 4.865 | 585.26538 | [M+H]+ | 585.26581 | 58.47013:5769 89.05896:14428 173.43034:11705 299.1311:5905 357.3071:6222 | -7.3471E-07 |
| POS8335 | a,8-dimethyl-6-ox | 5.75 | 397.19965 | [M+H-H2O]+ | 397.20001 | 59.04858:23504 69.03294:8009 87.04333:5658 89.05895:12635 119.08332:75640 | -9.0634E-07 |
| POS10498 | ylnaringenin7-O-g | 6.21 | 516.99963 | [M+H]+ | 517 | 52.44873:4856 56.27097:4187 57.98917:5108 58.19838:4594 60.07725:4665 60.36026:5265 60.95378:5076 63.24981:4456 64.00186:4692 64.86604:4564 67.67013:4577 68.34462:4437 68.53918:4719 69.43006:5691 70.26247:4358 70.37391:4217 70.41577:4417 70.42593:4039 70.43608:4755 74.89813:4712 77.04238:4995 80.67318:5405 83.44244:4438 87.48277:4251 89.29951:5035 89.67585:4541 94.42513:4738 95.21034:4112 95.22631:3790 98.21039:4061 98.22293:4514 100.78278:4159 102.74039:4042 102.99358:4351 103.7225:4083 104.58311:3632 107.09676:4168 107.84329:4670 109.57148:4830 111.35164:4010 111.56139:3858 113.12126:4108 136.55806:4286 140.75113:4023 143.19608:5018 153.78905:3666 167.38799:5310 179.18613:4192 179.24796:4594 181.7372:4760 192.49483:5029 195.91818:4660 204.91539:4892 205.36974:5037 220.06386:4828 241.96165:4268 262.63007:4140 266.41821:4031 284.85248:4063 328.84229:4778 330.48773:4968 354.36633:4168 382.86893:4410 403.22705:5486 406.07657:4964 411.92078:4195 432.1152:4902 448.90985:6243 462.74417:4983 480.55896:4326 487.76578:5462 497.17715:4657 501.85251:4493 | -7.1567E-07 |
| POS14575 | Mubenin B | 6.597 | 735.4624 | [M+H]+ | 735.46301 | 94.29323:5399 115.30742:6495 117.48097:6924 201.29752:6080 | -8.2941E-07 |

**Differences in metabolites between the WJW group, the Model group and the Control group**

| Alignment ID | Metabolite name | Rt(min) | Expreiment Mz | Adduct type | Reference m/z | MS/MS spectrum | PPM |
|---|---|---|---|---|---|---|---|
| POS13936 | DTXSID60346618 | 8.667 | 679.47144 | [M+2H]2+ | 679.47198 | 70.29156:5858 92.95089:5664 105.49634:6093 215.87585:5333 286.19946:6859 304.34259:5523 | -7.9473E-07 |
| POS6151 | ancuronium bromi | 7.046 | 286.22839 | [M+NH4]+ | 286.228 | 73.09652:5308 113.8013:6307 | 1.36255E-06 |
| POS370 | p-Cresol | 4.389 | 91.05412 | [M+Na]+ | 91.0537 | 51.59062:7082 57.30673:5757 57.93421:6222 62.03075:6327 65.03805:19051 66.99615:6298 91.0539:28271 | 4.61266E-06 |
| NEG1560 | Citramalic acid | 8.811 | 147.10043 | [M-H2O-H]- | 147.10001 | 59.08403:8234 74.67177:5613 101.05437:9917 102.05606:16315 103.05476:36319 | 2.8552E-06 |
| NEG1185 | Phenyl vinyl sulfide | 7.569 | 135.02791 | [M-H]- | 135.0275 | 65.09129:31817 66.07739:17039 66.08893:7004 79.05219:10746 89.13127:23061 92.12341:6997 92.13479:10820 100.0459:6214 102.05609:9050 107.15221:13537 117.06139:10774 135.0251:24814 135.05542:20732 | 3.03642E-06 |
| POS6410 | oleic acid, methyl | 1.207 | 295.26163 | [M+H]+ | 295.26199 | 51.64319:7048 55.05366:19076 57.06989:11474 67.05389:13073 69.06992:20634 69.89835:6757 81.06834:20028 83.0851:14047 95.08477:10860 97.09953:8127 107.08487:7368 121.1013:7297 133.09863:10153 | -1.2193E-06 |
| POS12448 | Austrobuxusin I | 4.568 | 591.35358 | [M+2H]2+ | 591.35303 | 73.06431:106240 87.04333:602119 89.05895:4109172 90.0619:73522 117.08816:56878 131.06764:114271 133.08543:1918621 134.08682:60913 173.43523:48508 175.09525:51397 177.11284:220419 296.69055:30865 570.67053:33803 581.78009:35843 | 9.3007E-07 |
| POS12196 | odeoxycholyltrypto | 5.331 | 579.38055 | [M+2H]2+ | 579.38 | 57.03287:49203 59.04859:1127110 60.05226:37743 73.02812:124180 73.06432:32703 80.0543:27308 85.06506:35434 87.04335:219358 87.06079:43940 87.07999:42551 89.05896:1293560 90.06191:46038 94.06947:25213 101.05931:167521 103.07444:325522 109.0756:35468 116.08248:25592 117.0909:33581 129.08911:42456 131.06766:25643 133.08545:482817 134.08684:31299 147.10149:172306 161.11441:30490 177.11285:48489 191.1256:36244 | 9.49291E-07 |
| POS11050 | ydrocelastryl diace | 4.821 | 537.32159 | [M+Na]+ | 537.32111 | 51.77975:10735 53.44824:10138 172.19113:9965 384.88806:10550 | 8.93321E-07 |
| POS11667 | oxy-22,25-oxido-1 | 4.872 | 559.3634 | [M+H]+ | 559.36292 | 59.04858:164065 87.04334:24241 89.05896:243012 101.0593:19786 103.07444:37613 133.08545:52739 147.10149:11455 184.7793:10282 252.30504:9418 | 8.58119E-07 |
| POS11611 | Gln Val Leu Leu Gly | 5.352 | 557.36609 | [M+2H]2+ | 557.3656 | 57.03287:13623 59.04858:238876 69.06992:8568 73.02811:21019 73.06432:8827 85.06505:6937 85.42169:5890 87.04334:38228 87.06078:12802 87.07998:9133 89.05896:238150 90.0619:8125 101.0593:34692 103.07444:61192 109.07559:6788 109.61583:5807 110.70842:6597 129.08911:6293 133.08543:81501 147.10149:31376 173.39601:8739 226.88602:5403 359.24414:5833 | 8.79136E-07 |

**Differences in metabolites between the WJW group, the Model group and the Control group**

| Alignment ID | Metabolite name | Rt(min) | Expreiment Mz | Adduct type | Reference m/z | MS/MS spectrum | PPM |
|---|---|---|---|---|---|---|---|
| POS10524 | lemonnierin | 8.857 | 518.29767 | [M+H]+ | 518.29718 | 65.91531:6574 81.0699:13970 85.06506:15727 93.07034:7524 95.08477:15201 104.10696:29663 105.06945:8505 107.08486:11646 109.10006:13271 121.09843:9822 126.02023:100495 127.02272:19039 128.01544:50965 131.08377:7190 133.09863:8719 135.11646:6467 145.09904:8818 147.11681:9320 157.10197:6557 159.11723:15079 160.12202:11101 199.14969:12618 200.14934:6864 208.06328:12433 209.13087:10801 213.1633:7460 227.14313:9550 505.616:6482 | 9.45404E-07 |
| POS7395 | Myrsinoic acid B | 4.502 | 341.21442 | [M+H]+ | 341.21399 | 59.04859:420374 73.06432:26204 85.06506:15104 87.04335:166315 89.05896:2371431 90.06191:49744 96.08018:10842 101.05931:8375 103.03849:13936 103.07444:64185 107.07059:21353 114.09126:11675 129.08911:9317 131.07088:15901 133.08545:565314 134.08684:17578 147.10149:24456 173.43036:8183 177.11285:26297 | 1.26021E-06 |
| POS11845 | ¥æ¨ å å 4 | 4.528 | 565.35852 | [M+2H]2+ | 565.35822 | 87.04333:35172 89.05895:298107 90.0619:48146 133.08543:136004 134.08684:29761 140.82646:19314 173.3911:40907 176.78931:20847 314.63065:18759 487.51135:21257 | 5.30637E-07 |
| POS9389 | Stellettin B | 4.912 | 463.28534 | [M+H]+ | 463.2858 | 59.04859:17473 73.02812:6506 80.0543:6441 87.04335:9064 89.05896:27470 103.0385:7367 133.08545:13761 234.66223:5886 | -9.9291E-07 |
| POS8470 | rihydroxy-6-methy | 4.528 | 407.22345 | [M+Na]+ | 407.22299 | 62.14423:6016 86.05939:5706 163.52153:5868 258.34149:6538 | 1.1296E-06 |
| POS12380 | yloxy)icosanoyl]oxy | 5.143 | 587.37946 | [M+2H]2+ | 587.37891 | 59.04859:361571 72.83016:28106 73.02812:91944 73.06432:36273 87.04335:142063 87.06254:31344 89.05896:826186 90.06191:26877 101.05931:88199 103.07444:125138 106.91372:28114 129.08911:28422 133.08545:281272 147.10149:61475 173.42545:34963 177.11285:64965 185.83572:27589 | 9.36363E-07 |
| POS11321 | Cholylarginine | 4.913 | 547.38452 | [M+H]+ | 547.38501 | 59.04856:201737 87.0433:19741 89.05891:174987 101.05926:35441 103.07439:54084 117.05275:24089 133.08537:41112 147.10141:25965 173.38612:22581 185.46078:14998 267.18558:15823 | -8.9517E-07 |
| POS12977 | Leu Gln Lys Ser Leu | 5.217 | 616.40222 | [M+2H]2+ | 616.40277 | 57.03287:12818 59.04858:170867 73.02812:34173 87.04334:107092 87.06079:10222 88.04782:9812 89.05896:280759 101.0593:50900 102.06606:10401 103.07444:31834 133.08545:113618 145.04276:8984 147.10149:18346 177.11285:11702 348.84436:9617 | -8.9227E-07 |
| POS9288 | Gly Val Ile Gly Ile | 5.385 | 458.29684 | [M+CH3OH+H]2+ | 458.29721 | 57.03286:11500 59.04858:162045 60.05225:6547 61.36362:5549 67.01499:11342 81.03072:11099 87.04333:13940 89.05895:83040 101.0593:12643 103.07443:36718 125.31528:5654 133.08543:16914 141.435:5171 147.10147:11008 158.09196:7295 165.41951:5772 | -8.0734E-07 |
| POS13697 | ,18-hexa(propan-2 | 6.477 | 662.3974 | [M+Na]+ | 662.39801 | 51.71899:51994 74.05202:54767 155.46558:50627 173.39111:118150 367.89346:58816 | -9.209E-07 |
| NEG6605 | bicyclo[4.1.0]hept- | 1.021 | 325.14819 | [M-H]- | 325.14774 | 55.93273:5822 64.72199:5727 89.13125:15144 93.03925:471780 95.03763:46740 97.03745:13607 105.56303:5625 124.46915:5651 127.20192:7845 134.22708:7003 145.23547:44896 151.06712:33643 | 1.38399E-06 |
| POS7904 | erone 3-sulfate so | 5.752 | 369.17142 | [M+H]+ | 369.17099 | 57.06894:21290 59.04953:11239 79.15736:5939 79.33614:5442 85.06333:11476 89.05892:10233 | 1.16477E-06 |

**Differences in metabolites between the WJW group, the Model group and the Control group**

| Alignment ID | Metabolite name | Rt(min) | Expreiment Mz | Adduct type | Reference m/z | MS/MS spectrum | PPM |
|---|---|---|---|---|---|---|---|
| POS9047 | Difenacoum | 3.357 | 445.18066 | [M+H]+ | 445.18109 | 50.11115:6874 137.04604:132977 145.24556:5233 160.07417:39036 179.40268:6166 | -9.659E-07 |
| POS3783 | Tacrine | 4.594 | 199.12416 | [M+H]2+ | 199.1246 | 73.51437:5165 75.64143:6344 81.06833:8050 | -2.2097E-06 |
| POS7800 | [12]-Shogaol | 6.047 | 361.27246 | [2M+H]+ | 361.27289 | 55.05366:8866 57.03287:8714 57.06896:5744 57.37107:5850 67.05389:19133 67.47569:5805 69.06869:6543 79.05313:12329 81.0699:19388 91.0539:16121 93.06841:10870 95.08478:10353 105.06946:8554 119.08613:10188 173.39111:10269 | -1.1902E-06 |
| POS2369 | ethyl propyl trisulfi | 9.136 | 155.00212 | [M+H]+ | 155.00169 | 51.94016:10242 55.05365:17822 55.93345:133211 67.05389:30009 67.93391:7764 71.94005:16588 72.93706:1124896 73.93582:6224 79.05463:6605 81.06989:14737 90.94762:214569 97.5863:6159 108.08179:9968 108.95824:27118 113.96311:308913 114.97044:26824 126.97046:6325 131.97466:103719 | 2.77416E-06 |
| NEG1006 | Agmatine | 0.889 | 129.11505 | [M-H]- | 129.11459 | 62.06304:9145 88.1461:11047 | 3.56273E-06 |
| POS4898 | OPC-4:0 | 6.191 | 239.16335 | [M+H]+ | 239.1638 | 55.01769:7056 55.05452:11522 57.06987:16397 67.05388:6518 81.06988:9457 95.08475:19134 107.1801:6125 109.06334:22417 109.10004:6309 119.08609:11449 123.08001:29059 179.13972:130632 | -1.8816E-06 |
| POS1215 | ide, methyl tert-pe | 1.053 | 119.08923 | [M+H]+ | 119.0888 | 53.03773:49580 55.05365:2183662 56.04951:38541 56.05673:209583 57.05692:204000 59.04858:37663 72.08047:2599418 73.08309:235070 | 3.61075E-06 |
| POS7456 | IC ACID DER (FR. I | 6.406 | 343.28378 | [M+2H]2+ | 343.28421 | 60.08125:10091 69.71296:6009 75.02497:9170 82.06503:9029 86.0954:24935 124.99955:8473 145.3998:6233 184.06929:46920 264.26468:14336 | -1.2526E-06 |
| NEG1561 | Citramalic acid | 0.646 | 147.10046 | [M-H]- | 147.10001 | 59.0295:16415 59.08405:16129 65.60266:6277 73.20889:6079 79.5448:5428 | 3.05914E-06 |
| POS5562 | Z)-Octadecadienc | 6.776 | 263.23553 | [M+H]+ | 263.23599 | 201.77066:5538 257.78946:5736 | -1.7475E-06 |
| NEG471 | 1,2,4-Butanetriol | 0.337 | 106.06342 | [M-H]- | 106.063 | 53.45897:4091 54.47842:4136 56.19134:5904 59.97625:4719 60.3217:4124 62.3273:4462 62.51997:3862 62.68903:4419 64.66617:4102 64.67733:4571 65.33318:4541 65.69171:4110 68.71078:4153 68.82339:5140 68.90435:4902 69.84071:4193 70.78477:4560 74.1647:4320 76.61039:4261 76.85421:4962 81.62371:4848 82.3423:4234 86.58962:5238 90.25441:4335 92.34967:4441 95.33667:4001 96.94727:5738 101.89474:4618 109.04345:4326 | 3.95991E-06 |
| POS12057 | SNF-4794-12 | 4.934 | 573.38245 | [M+H]+ | 573.3819 | 57.03287:155241 59.04859:3207844 60.05226:53694 73.02812:126239 73.06432:67632 85.06337:85421 87.04335:737397 87.07999:92912 89.05896:3782734 90.06191:78200 101.05931:518869 101.09423:32070 103.03849:110417 103.07444:1274112 104.07732:51424 105.09028:30692 107.06821:33453 115.07378:44584 117.05281:62599 117.0909:75995 129.08911:65900 131.06766:93031 131.10634:47827 133.08545:1309605 134.08684:47921 143.10413:30559 145.08403:79916 147.10149:917573 148.1028:29181 161.1188:78016 177.11285:104586 191.1256:143377 205.14238:58272 | 9.59221E-07 |

**Differences in metabolites between the WJW group, the Model group and the Control group**

| Alignment ID | Metabolite name | Rt(min) | Expreiment Mz | Adduct type | Reference m/z | MS/MS spectrum | PPM |
|---|---|---|---|---|---|---|---|
| POS8339 | Rollicosin | 4.953 | 397.259 | [M+2H]2+ | 397.25851 | 57.03284:27986 59.04856:732509 60.05223:15843 65.04928:7615 69.06865:6942 73.02808:110844 73.06429:38310 80.05426:15877 85.06501:17184 87.0433:98442 87.06249:24442 87.07994:13465 89.05891:741093 90.06187:22602 94.06942:8094 100.07593:7046 101.05926:50173 102.06822:11634 103.07439:120326 104.07727:7604 107.06815:8568 109.07554:12543 117.05275:34792 117.09084:10051 129.09219:7225 130.08702:20660 131.07082:25295 133.08537:161299 134.08678:9455 147.10141:40354 161.11433:8025 173.39594:6746 177.11278:11449 | 1.23345E-06 |
| POS12516 | Destruxin B | 5.228 | 594.38672 | [M+2H]2+ | 594.38611 | 57.03287:26272 59.04859:538786 73.02812:101193 73.06432:24663 73.49814:17151 80.0543:19289 85.06337:28720 87.04335:178714 87.06254:36547 87.07999:24217 89.05896:1022678 90.06191:35777 101.05931:79315 102.06606:39869 103.07444:150524 109.0756:24944 114.26157:13530 129.08911:22954 131.06766:27389 133.08545:384610 134.09018:27651 147.10149:90130 177.11285:48414 230.37541:13202 233.37059:13654 262.24658:15602 276.94675:14044 | 1.02627E-06 |
| POS9151 | -5 trisulfate hexah | 5.267 | 451.28793 | [M+2H]2+ | 451.28741 | 57.03287:15085 59.04859:185668 67.015:17826 73.02812:7320 73.06432:9437 73.48596:7111 81.03072:14132 87.04335:17803 89.05896:148284 101.05931:29580 103.07444:35713 115.07378:6390 133.08545:38886 147.10149:13744 158.08772:5226 226.99617:5283 | 1.15226E-06 |
| POS10896 | Ser Ile Arg Arg | 4.69 | 531.33557 | [M+H]+ | 531.33612 | 59.04858:43160 87.04333:65942 87.26875:22683 89.05894:465193 133.08542:152319 173.43033:28266 177.11282:27108 259.75623:19243 530.31659:22636 | -1.0351E-06 |
| POS11729 | ntibiotic Sch 2383 | 5.238 | 561.35284 | [M+2H]2+ | 561.35339 | 59.04859:87533 67.015:18922 73.06432:11342 87.04509:14094 89.05896:127367 93.83868:7235 103.07445:20350 117.05553:9711 133.08545:51224 147.10149:11926 428.1304:7850 477.49673:7311 | -9.7977E-07 |
| POS13779 | -Dibenzoyl karoun | 5.304 | 667.43494 | [M+2H]2+ | 667.43561 | 59.04859:242078 73.02812:68694 84.68888:23790 87.04335:82863 89.05896:540016 101.05931:60546 103.07444:63152 117.0909:28913 130.72673:29701 133.08545:202787 225.53427:29601 | -1.0038E-06 |
| POS8754 | Arg Asp His | 4.437 | 427.2052 | [M+Na]+ | 427.20471 | 59.04956:6979 86.05939:7605 87.04335:5502 89.05896:15923 103.03849:13665 111.0419:5450 118.0629:26205 132.07892:13821 134.09685:11988 144.0797:16425 146.061:110536 147.06319:5783 159.09138:14633 188.07004:314637 189.07664:16750 205.09822:59622 | 1.14699E-06 |
| POS5814 | Alismorientol A | 5.97 | 273.20508 | [M+CH3OH+H]+ | 273.2056 | 55.32309:6444 55.35403:5615 57.03287:8367 57.06989:6506 82.75266:6326 | -1.9033E-06 |
| POS4818 | ethoxyphenyl)isoq | 5.91 | 236.10951 | [M+H]+ | 236.11 | 57.06896:21774 141.5977:5960 174.85666:6375 | -2.0753E-06 |
| POS5817 | Alismorientol A | 6.138 | 273.20511 | [M+H-H2O]+ | 273.2056 | 180.81987:6144 273.90359:6008 | -1.7935E-06 |
| NEG9489 | PFSA-ether | 5.373 | 530.91949 | [M-H]- | 530.92004 | 56.03255:8255 75.12117:5831 123.12397:138146 256.54803:6117 364.29367:5817 | -1.0359E-06 |
| POS6744 | -glucopyranosyl-1 | 4.867 | 309.19022 | [M+H]+ | 309.1907 | 55.0177:10556 57.03287:103329 59.04859:1483114 60.05226:15732 71.04816:26645 73.02812:31427 73.06432:408673 87.04335:42986 89.05896:10118 103.07445:11926 117.05281:211554 117.0909:37128 131.06766:139032 | -1.5524E-06 |

**Differences in metabolites between the WJW group, the Model group and the Control group**

| Alignment ID | Metabolite name | Rt(min) | Expreiment Mz | Adduct type | Reference m/z | MS/MS spectrum | PPM |
|---|---|---|---|---|---|---|---|
| POS5815 | Alismorientol A | 5.797 | 273.20508 | [M+H]+ | 273.2056 | 50.07689:6175 72.35727:6141 82.85947:5386 87.14983:5554 182.26979:5537 | -1.9033E-06 |
| POS7078 | xanecarbonyltetra( | 8.014 | 324.32507 | [M+H]+ | 324.32559 | 51.92889:6355 62.49498:5815 65.37954:5827 87.17076:5573 105.47303:6335 144.18367:5420 221.77029:6139 | -1.6033E-06 |
| POS3888 | Hydroxy-L-tryptop | 3.068 | 203.08051 | [M+H-H2O]+ | 203.08099 | 55.05365:15338 56.05764:6963 60.04426:13778 61.03972:9841 98.06007:7464 130.06477:8421 134.57501:6771 157.07237:7125 | -2.3636E-06 |
| POS4165 | Elaeokanine C | 4.978 | 212.16383 | [M+H-H2O]+ | 212.16431 | 55.05365:14712 57.06988:6819 60.26771:6333 67.05389:7738 69.06991:12367 81.06989:8879 83.08508:10264 93.0684:12155 105.40099:6211 107.08485:9791 | -2.2624E-06 |
| POS1585 | )-2-Pentanol aceta | 5.696 | 131.10608 | [M+H]+ | 131.1066 | 56.04953:7138 66.73078:6452 84.04482:8608 84.07957:26514 86.09541:23548 88.00349:46180 91.0539:15445 97.00918:17650 99.51076:6357 105.87435:5223 115.96171:8913 131.08377:8452 | -3.9662E-06 |
| POS6104 | adecanoyloxyprop | 7.722 | 284.29349 | [M+H]+ | 284.29401 | 50.15384:6115 55.83028:5610 70.08572:6014 73.98637:5237 173.38622:15165 205.2939:6562 | -1.8291E-06 |
| POS477 | 5Z)-1,3,5-Heptatri | 0.704 | 95.08558 | [M+H]+ | 95.08607 | 55.05365:40419 60.81196:6018 65.03804:9508 67.05389:59072 95.04893:12522 95.08476:16068 | -5.1532E-06 |
| POS5136 | Estrane | 7.14 | 247.24088 | [M+H-H2O]+ | 247.24139 | 60.68689:7013 68.0495:6114 101.14882:5995 130.32001:6294 189.62503:5234 | -2.0628E-06 |
| POS10064 | -1a,4,4a,5,6,7,7a,10 | 5.543 | 498.28558 | [M+H-H2O]+ | 498.28503 | 57.06989:52798 59.04858:117315 60.08025:319774 61.08378:61635 71.0726:40468 80.26237:38436 86.09541:637366 87.04334:89207 87.09918:212624 89.05896:111737 104.10696:3389616 105.10879:901230 106.11105:71854 124.42527:38870 124.99955:143348 126.02023:198528 143.13353:36345 173.43034:49780 184.07466:1275888 185.07635:350369 186.07533:109538 | 1.10379E-06 |
| POS6421 | clopropylethenyl)- | 8.844 | 296.14386 | [M+2H]2+ | 296.14441 | 55.0177:7234 55.05365:61963 57.06896:421288 58.06511:67268 58.07177:17376 59.07198:43990 60.08025:34325 67.05389:84423 69.06868:48493 71.0726:10686 71.08547:201446 72.08836:9254 76.53945:6980 81.06833:84160 83.08509:50908 85.10045:85449 86.0954:100834 95.08477:125856 97.09953:24544 102.09042:12012 104.10696:104389 109.10006:38765 123.11816:12654 124.99955:18668 137.5089:10278 149.01665:18663 156.51999:16360 157.01743:7404 165.52467:12755 184.07465:21663 193.53142:9053 256.00317:23735 | -1.8572E-06 |
| POS13106 | homodestcardin | 5.66 | 622.41663 | [M+2H]2+ | 622.4173 | 57.03287:119903 59.04859:1589836 69.06868:36093 73.02812:141734 73.06432:68803 85.06337:71467 87.04335:301380 87.06254:61232 87.07999:69568 89.05896:1353998 90.06191:32242 94.06947:52451 101.05931:317615 102.06384:30548 103.07444:550777 115.07378:36005 116.08248:32758 117.08817:47840 129.08911:66206 131.07088:55611 131.10634:49848 133.08545:577301 134.09018:40175 143.10413:33472 145.08403:32688 147.10149:335780 161.1188:45661 173.11685:31273 177.11285:74070 191.1256:68499 205.14238:45497 | -1.0764E-06 |
| POS10119 | tidylethanolamine | 5.495 | 500.27765 | [M+H-H2O]+ | 500.2771 | 59.04857:49635 62.06013:11238 67.21217:5362 87.04333:8936 89.05894:37492 103.07442:9126 104.10694:12132 126.02021:44426 127.02269:6110 128.01852:20755 | 1.09939E-06 |

Differences in metabolites between the WJW group, the Model group and the Control group

| Alignment ID | Metabolite name | Rt(min) | Expreiment Mz | Adduct type | Reference m/z | MS/MS spectrum | PPM |
|---|---|---|---|---|---|---|---|
| POS3197 | Nicotinuric acid | 4.505 | 181.05997 | [M+H]+ | 181.0605 | 51.02236:7006 65.03804:6560 68.75417:5609 78.0336:77533 96.04379:539695 97.04613:9670 97.21471:5970 106.02893:12081 115.702:6127 124.03867:318359 135.05576:213309 173.3862:8053 | -2.9272E-06 |
| NEG1756 | lethylpseudoconhy | 1.56 | 156.14005 | [M-H]- | 156.1395 | 78.18732:5748 82.16454:20570 89.13125:113313 97.07645:9456 111.15351:7432 151.103:5303 | 3.52249E-06 |
| POS11732 | -)-Strempeliopidir | 6.844 | 561.39569 | [M+Na]+ | 561.39508 | 53.71611:5663 95.83591:6661 158.69135:6209 261.57294:5663 273.44662:6200 | 1.08658E-06 |
| POS4068 | phenyl)-5-(Methylt | 1.583 | 209.05473 | [M+H]+ | 209.0542 | 77.12376:5258 102.64651:6055 162.43123:5468 180.68938:5230 | 2.53523E-06 |
| POS5134 | Estrane | 5.843 | 247.24084 | [M+H]+ | 247.24139 | 79.05312:6558 93.06841:11853 107.08486:7921 | -2.2245E-06 |
| POS3017 | densispicnin D | 4.881 | 175.13225 | [M+H-H2O]+ | 175.1328 | 53.40379:6508 59.04858:7105 70.78779:5501 94.23231:6106 104.95851:5791 106.06411:14832 130.06477:32586 134.06017:17831 173.39111:13848 | -3.1405E-06 |
| POS2917 | anethiosulfonic ac | 9.369 | 172.97636 | [M+H]+ | 172.9758 | 55.05366:23586 55.05892:6892 55.93345:32149 57.73019:6022 67.05389:78850 70.06431:6554 71.04945:6566 71.92827:70420 72.65827:5430 72.93707:64732 81.0699:10823 87.03812:6491 89.93906:65184 90.90295:65891 90.94764:22434 95.08477:6469 107.9492:38478 109.10007:25251 114.97046:10998 125.9595:24131 126.09015:8907 130.9646:25725 131.92909:12921 132.86481:5588 148.97369:38969 149.93901:14493 | 3.23745E-06 |
| POS8789 | 4'-Diapolycopenec | 5.797 | 429.27969 | [2M+H]+ | 429.27911 | 57.03287:30988 57.06896:36714 60.08025:325791 71.08547:35847 85.02799:1805681 86.03024:139674 95.08477:25251 144.10197:24246 246.25351:11792 | 1.3511E-06 |
| POS10669 | LPE 19:0-d5 | 5.401 | 523.35559 | [M+H]+ | 523.35498 | 60.08022:1087168 86.09537:1949797 87.09914:264323 91.09866:145234 104.10692:9567680 105.10874:1173202 105.7948:130571 124.9995:376109 145.09148:136445 173.43027:289912 184.06921:3053278 185.07626:403079 204.46875:140514 208.62509:134079 261.91843:153349 | 1.16556E-06 |
| NEG4961 | -/-)-Apomorphine | 0.986 | 267.12653 | [M-H2O-H]- | 267.12592 | 87.11253:36435 89.13125:15772 93.03925:2156657 95.03763:9071 97.03951:6007 122.80492:5907 151.06712:82487 | 2.28357E-06 |
| POS8243 | Bacithrocin A 2 | 4.878 | 390.25101 | [M+2H]2+ | 390.2504 | 57.03287:28517 58.04042:61799 59.04859:810843 60.05226:18014 65.04819:32205 69.06992:12328 73.02812:311303 73.06432:37208 80.0543:152726 85.06337:31220 87.04335:212354 87.06079:100912 87.07999:18149 87.56366:9811 89.05896:1659588 90.06191:47156 94.06947:11847 101.05931:59372 102.06606:90634 103.07444:111592 107.07059:17137 109.0756:40923 111.07206:7925 117.0909:9068 118.07944:8454 124.08022:36657 129.08911:21721 131.06766:22242 131.08699:17347 133.08545:518030 134.08684:24749 140.09358:8830 147.10149:50140 162.10718:10227 173.11685:8866 173.39111:15223 177.11285:46600 191.1256:7760 | 1.5631E-06 |
| POS11315 | 34-oxamanzamine | 6.191 | 547.34375 | [M+H]+ | 547.34308 | 50.72232:13760 60.08022:29099 72.07124:11772 76.34278:12631 76.75559:11365 86.09536:55042 104.10692:204612 105.10873:83869 106.111:29289 112.98438:11424 166.01965:10978 184.06921:58662 185.07626:30063 292.86453:11391 381.84113:11651 419.16068:13625 531.78821:11194 | 1.2241E-06 |

**Differences in metabolites between the WJW group, the Model group and the Control group**

| Alignment ID | Metabolite name | Rt(min) | Expreiment Mz | Adduct type | Reference m/z | MS/MS spectrum | PPM |
|---|---|---|---|---|---|---|---|
| POS8790 | 4'-Diapolycopened | 5.995 | 429.27969 | [M+Na]+ | 429.27911 | 67.01499:23138 68.83137:5620 85.02631:6440 97.0256:21060 107.27312:5940 140.07933:9545 156.05841:5539 166.37865:6072 213.10983:6838 251.07872:6323 | 1.3511E-06 |
| POS10243 | ctapropylene glyc | 5.391 | 505.33411 | [M+Na]+ | 505.33472 | 53.72115:6428 59.04856:11800 87.04504:5356 89.05891:18520 106.9778:6131 307.71118:5652 | -1.2071E-06 |
| POS10853 | Lucidamine B | 4.455 | 530.28937 | [M+H]+ | 530.28998 | 70.02779:27691 70.25867:6630 86.0954:72075 87.0538:163077 88.03895:13858 88.05669:7621 89.05895:42678 133.08543:20422 136.39027:6852 144.07225:8666 172.06985:29347 188.07002:17350 331.34097:9057 344.17609:6824 | -1.1503E-06 |
| POS8201 | Sufentanil | 5.612 | 387.21133 | [M+H]+ | 387.21075 | 66.28812:5293 149.63585:5504 | 1.49789E-06 |
| POS6552 | -oxo-3,4,4a,5,6,7- | 1.043 | 301.13873 | [M+H]+ | 301.13931 | 67.55789:6126 74.91763:5349 94.50796:6279 | -1.926E-06 |
| POS6126 | )-Isopropylidene u | 8.945 | 285.10867 | [M+2H]2+ | 285.10809 | 50.45882:5582 54.38173:5837 57.06989:9494 108.00944:7526 126.02023:100702 133.29341:6084 230.44301:5340 | 2.03432E-06 |
| POS5487 | ,6,7-hexahydro-2 | 5.97 | 261.13385 | [2M+H]2+ | 261.13327 | 57.03287:50469 57.54692:5185 61.26566:5423 109.75152:6913 | 2.22109E-06 |
| POS4547 | xy-10-methylunde | 8.399 | 226.21539 | [M+H]+ | 226.216 | 67.77902:6710 87.61996:5760 194.30267:6060 201.20547:6926 | -2.6965E-06 |
| POS3548 | SCHEMBL536577 | 4.957 | 191.12712 | [M+2H]2+ | 191.1277 | 59.04859:20309 89.05896:14193 169.03723:6139 | -3.0346E-06 |
| POS3547 | SCHEMBL536577 | 5.284 | 191.12712 | [M+CH3OH+H]+ | 191.1277 | 59.04859:16667 78.08692:5601 130.06477:33273 131.21602:5188 131.92909:5869 | -3.0346E-06 |
| POS6874 | 4,16-dimethylocta | 5.678 | 314.34061 | [M+H]+ | 314.34119 | 57.06989:29811 58.06417:9153 62.05909:18598 62.34779:5756 67.25955:5549 71.08548:13255 95.08478:6751 162.15594:5457 | -1.8451E-06 |
| POS4798 | Callicarpenal | 5.882 | 235.20441 | [M+H-H2O]+ | 235.205 | 55.05365:12582 57.06896:10346 67.05389:19811 69.06992:6651 81.0699:20363 93.06841:13292 95.08477:16563 107.08486:10424 109.10006:8951 121.1013:6810 133.09863:9261 173.39601:9357 189.166:6362 202.80254:6188 | -2.5085E-06 |
| POS5612 | Vaccenic acid | 0.723 | 265.2514 | [M+H-H2O]+ | 265.25201 | 55.05364:6929 57.06895:7314 64.0822:5705 67.05388:8099 95.08475:7276 128.43639:5448 156.52417:4948 173.39107:8108 | -2.2997E-06 |
| NEG6662 | Neurocil | 1.052 | 327.15433 | [M-H2O-H]- | 327.15369 | 52.07363:6494 93.03928:872312 95.03767:416150 112.81192:5661 148.88708:6056 151.06717:36099 153.0676:44578 211.09386:6706 | 1.95627E-06 |
| NEG6606 | HYDROQUINIDINE | 1.056 | 325.15448 | [M-H]- | 325.15384 | 64.7287:5833 89.13125:11698 93.03925:628532 95.03763:54615 97.03745:20711 145.23549:26894 151.06712:32256 224.36078:6202 | 1.9683E-06 |
| POS9945 | LysoPC 16:2 | 5.8 | 492.30777 | [M+Na]+ | 492.30841 | 59.04859:14322 60.08025:17792 86.09541:33976 89.05896:11013 104.10697:195092 124.99956:13507 184.06931:80539 | -1.3E-06 |
| POS9490 | olin-1-yl)-17-nor | 5.121 | 469.29675 | [M+H]+ | 469.29611 | 59.04858:36831 87.04334:65710 89.05896:30551 101.0593:18277 | 1.36374E-06 |
| NEG9467 | Sulfluramid | 5.826 | 526.98547 | [M-H]- | 526.9848 | 56.92723:6923 68.16396:5792 79.05373:35127 98.51506:5653 158.53918:7858 168.244:8458 216.10568:5340 | 1.27138E-06 |
| NEG4913 | OCTADEC-16-ENA | 0.878 | 265.25446 | [M-H]- | 265.25381 | 77.54872:6374 101.10021:5444 113.12118:131760 134.21043:6877 143.93689:5017 | 2.45048E-06 |
| POS14103 | coumaroyltetracos | 6.817 | 693.47156 | [M+H]+ | 693.47241 | 62.44623:4860 69.6243:6494 87.19528:6082 184.61758:5548 207.8765:6022 248.67805:5908 | -1.2257E-06 |

**Differences in metabolites between the WJW group, the Model group and the Control group**

| Alignment ID | Metabolite name | Rt(min) | Expreiment Mz | Adduct type | Reference m/z | MS/MS spectrum | PPM |
|---|---|---|---|---|---|---|---|
| POS8606 | Epigomisin O | 5.68 | 417.19174 | [2M+H]+ | 417.19241 | 55.01768:174743 57.06894:393506 69.06989:9178 79.05309:16715 83.01192:882887 84.015:11910 85.06333:274529 93.0703:31073 97.06457:46170 101.02218:44803 111.04436:16485 111.07955:46585 115.03928:542901 116.04215:7881 121.06405:26848 137.05975:7557 139.07352:234912 140.07928:11300 149.09476:8913 150.10469:6037 153.09097:10085 157.08499:131414 167.10457:37600 171.09958:352444 172.10374:14078 221.1192:19923 222.12537:16691 247.10213:10619 | -1.606E-06 |
| POS13381 | deferrioxamine E | 4.942 | 639.39178 | [M+NH4]+ | 639.39093 | 51.69823:15774 52.24539:16019 57.26204:16845 57.49446:15405 63.10206:14911 64.38737:17272 66.84683:15535 67.72396:16179 69.98:15275 70.89279:14630 72.48169:14052 81.48377:15286 83.18766:14148 85.52522:14213 106.00784:15084 116.15504:19389 119.128:16230 126.05975:15786 127.80418:13953 130.4415:14237 131.46817:16566 137.12877:14484 145.22301:15836 146.56651:14491 148.21899:17379 149.40419:14346 159.72244:14941 172.44861:17416 181.18074:19832 183.76932:19344 193.33499:17767 198.01981:17442 216.79158:16968 219.92371:18493 251.43799:14919 278.60718:15006 294.77921:16229 316.50882:18242 316.65399:16131 321.06827:17036 323.32883:17358 373.3949:20685 379.43304:18899 503.52267:15397 504.54337:16705 548.30841:15379 584.229:16901 | 1.32939E-06 |
| POS8548 | Arg His Thr | 4.42 | 413.22482 | [M+H]+ | 413.22549 | 59.04859:17241 70.06431:7509 72.08048:8153 87.04335:7051 89.05896:45647 103.07444:10459 118.06566:25692 132.07892:8927 133.08545:19008 144.0797:18346 146.061:92134 151.09705:6329 159.09138:12523 188.07004:310236 189.07106:20113 205.09822:54513 209.13737:7734 363.82065:6967 | -1.6214E-06 |
| POS2769 | Methylglutaconic a | 0.946 | 167.03081 | [M+CH3OH+H]+ | 167.03148 | 51.87749:7152 53.48768:5939 141.06718:5550 | -4.0112E-06 |
| POS5062 | Aspergilliamide B | 4.38 | 245.14882 | [M+NH4]+ | 245.14951 | 50.74328:5710 60.53485:5917 68.04947:19806 69.06865:9987 70.06427:6873 84.08119:7743 85.02796:14854 86.05935:80919 86.09537:175775 132.06581:129317 176.54716:5792 | -2.8146E-06 |
| POS12762 | -caffeoyloxyfriede | 6.835 | 605.42078 | [M+H]+ | 605.41998 | 59.86679:6420 | 1.3214E-06 |
| POS474 | 5Z)-1,3,5-Heptatri | 1.206 | 95.08541 | [M+H-H2O]+ | 95.08607 | 55.05365:31397 65.03804:11693 67.05389:46139 81.05109:6681 94.06358:28792 95.04893:9914 95.08476:12078 | -6.9411E-06 |
| POS4088 | Ethirimol | 7.201 | 209.15213 | [M+H]+ | 209.1528 | 62.28967:5904 95.08477:7367 156.20926:5810 | -3.2034E-06 |
| POS1888 | methylthio)methyl | 9.436 | 140.98665 | [M+H]+ | 140.98599 | 54.94678:16057 55.0177:7569 55.05365:10468 55.93345:120086 55.94513:11345 56.9414:107108 57.9342:29721 58.06511:11469 67.05389:25965 67.93391:10623 70.0643:12048 70.94153:9884 72.93706:54805 90.94762:10952 94.06358:21162 95.04694:7073 95.08476:17165 98.98203:6037 111.96739:9636 113.96311:43964 114.97044:33110 116.97124:22432 131.97466:8216 | 4.68132E-06 |

**Differences in metabolites between the WJW group, the Model group and the Control group**

| Alignment ID | Metabolite name | Rt(min) | Expreiment Mz | Adduct type | Reference m/z | MS/MS spectrum | PPM |
|---|---|---|---|---|---|---|---|
| POS7891 | UNII-5BCD1RO66X | 4.87 | 368.23901 | [M+2H]2+ | 368.23831 | 57.03287:32946 58.04042:81847 59.04859:845961 60.05226:21212 65.04819:47242 69.06869:8507 71.04816:9491 73.02812:288399 73.06432:31626 80.0543:109304 80.55504:8430 85.06337:33913 87.04335:206760 87.06079:92957 87.07999:13240 89.05896:1404834 90.06191:46269 94.06947:13907 101.05931:73293 102.06606:60752 103.07444:122821 107.07059:12879 109.07315:40397 111.07206:7955 115.07378:9574 117.05281:7930 124.08022:18934 129.08911:15292 131.06766:20340 133.08545:402344 134.09018:21942 147.10149:42058 177.11285:31739 191.1256:9253 | 1.90094E-06 |
| POS11452 | A 108836 | 4.929 | 551.33923 | [M+H]+ | 551.34003 | 59.04859:39169 63.23145:11485 73.02812:16131 74.31548:11144 80.0543:16151 87.04335:26564 89.05896:154180 104.10697:19400 133.08545:72250 173.38622:14575 495.42001:11316 | -1.451E-06 |
| POS7519 | reonylalanylarginir | 4.648 | 347.203 | [M+Na]+ | 347.2037 | 60.6027:5934 80.86337:6667 89.05896:7305 92.63606:5017 173.39111:15290 | -2.0161E-06 |
| POS10834 | methylhexadecahy | 8.806 | 529.32574 | [M+NH4]2+ | 529.32501 | 60.08025:7840 63.63507:6441 86.09541:16018 104.10697:162165 105.11111:9431 132.62503:6264 | 1.37911E-06 |
| POS11695 | Stellettacholine A | 4.67 | 560.33539 | [M+2H]2+ | 560.33459 | 69.03294:15177 73.02811:35386 73.06432:11459 80.05429:14448 87.04334:85223 88.04604:6778 89.05896:404118 90.0619:15946 91.04643:14813 95.04893:11870 102.06606:13250 111.04189:9243 113.05927:20331 131.06766:11306 133.08543:167499 134.09018:9578 137.0598:8958 155.06668:6020 177.11285:24578 190.08008:7159 223.32474:6305 | 1.42772E-06 |
| POS14395 | ulomo'opunalide- | 5.697 | 717.47858 | [2M+H]2+ | 717.47961 | 57.03287:19891 59.04859:221207 73.02812:35528 73.06432:17061 85.06337:12306 87.04335:77540 87.06079:20207 89.05896:270835 94.06947:13494 101.05931:63349 103.07444:78962 129.08911:11617 131.10634:12235 133.08545:89934 147.10149:47843 396.90311:11015 669.2359:11689 | -1.4356E-06 |
| NEG4967 | )-6-octadecen-5- | 1.74 | 267.26871 | [M-H]- | 267.26941 | 97.07645:17103 99.07355:76917 122.14449:23477 126.07927:8655 135.1936:9014 165.20354:11966 183.59097:6420 | -2.6191E-06 |
| POS13938 | -(28),23-dien-3-oi | 8.648 | 679.47693 | [M+2H]2+ | 679.47791 | 155.16624:5615 316.72656:5510 389.63431:6421 | -1.4423E-06 |
| POS13533 | -en-28-ol\|3??-trar | 6.826 | 649.44531 | [M+H]+ | 649.44623 | 55.45766:7692 66.63255:7260 66.88206:5923 91.21459:5691 110.99668:6065 130.05522:6720 291.0647:5703 517.97321:7532 | -1.4166E-06 |
| POS6778 | ythro-Sphingosine | 7.672 | 310.30829 | [M+H]+ | 310.30899 | 50.68978:8626 276.54135:5621 | -2.2558E-06 |
| POS12710 | ne-3alpha,29-dio | 6.299 | 603.40521 | [M+Na]+ | 603.4043 | 295.258:5816 | 1.50811E-06 |
| POS12993 | lene-4-(2-methyl- | 4.941 | 617.40582 | [M+H]+ | 617.40491 | 57.03287:111732 59.04859:2946029 73.02812:137628 73.06432:76402 85.06337:118003 87.04335:783200 87.07999:105717 89.05896:4837258 90.06191:77426 101.05931:594982 103.03849:81983 103.07444:1201891 115.07378:47468 117.05281:77075 117.0909:61153 129.08911:124756 131.06766:120960 131.10634:77159 133.08545:1871124 134.09018:72977 145.08403:98662 147.10149:957866 161.11441:76372 177.11285:189428 191.1256:146797 304.17166:44505 | 1.47391E-06 |

**Differences in metabolites between the WJW group, the Model group and the Control group**

| Alignment ID | Metabolite name | Rt(min) | Expreiment Mz | Adduct type | Reference m/z | MS/MS spectrum | PPM |
|---|---|---|---|---|---|---|---|
| POS8016 | Leucocrystal Violet | 5.293 | 374.25827 | [M+2H]2+ | 374.259 | 57.03287:93167 59.04859:2046332 60.05226:51596 65.04819:14339 69.06992:11142 73.02812:116829 73.06432:24672 81.0699:9899 85.06337:32496 87.04335:91908 87.06254:28085 87.07999:22968 89.05896:688187 90.06191:16894 94.06947:42386 99.07939:11622 101.05931:108683 103.07444:305256 104.07732:10915 115.07378:16776 116.08248:17429 117.05281:13299 117.0909:22477 129.08911:17999 131.07088:14247 133.08545:155401 143.1078:10089 147.10149:123765 161.11441:10648 191.1256:11440 | -1.9505E-06 |
| POS10667 | schiprolactone A | 4.747 | 523.30438 | [M+H]+ | 523.30359 | 60.08025:21171 86.09541:53744 87.04335:5583 87.49507:7251 89.05896:20147 104.10697:262831 133.08545:6003 142.02203:6853 184.07466:122621 | 1.50964E-06 |
| POS8668 | Erythrophloin A | 4.606 | 421.23813 | [M+Na]+ | 421.2374 | 89.05896:20748 118.57424:5939 139.98682:6236 173.43526:6276 | 1.73299E-06 |
| POS10171 | LysoPC(P-16:0/0:0 | 6.253 | 502.32748 | [M+H-H2O]+ | 502.32672 | 62.06014:11393 68.02178:6959 86.09712:10834 318.39163:5746 | 1.51296E-06 |
| POS13012 | Veralosine | 5.804 | 618.40094 | [M+2H]2+ | 618.40002 | 57.03287:16195 59.04859:82528 60.16235:11541 70.74816:10102 73.02812:15396 73.06432:12980 87.04335:28027 89.05896:96925 101.05931:29693 103.07445:22539 129.08911:12432 133.08545:33699 135.46794:9780 147.10149:14249 173.38132:13274 196.63869:10859 | 1.48771E-06 |
| POS4339 | mma-Glutamylalan | 1.483 | 219.09666 | [M+H]+ | 219.0974 | 56.0495:7304 83.04604:5690 84.04313:118986 84.07954:10805 85.02796:8517 90.05453:44206 128.0714:7891 130.0488:15023 156.06255:19326 170.79237:5928 | -3.3775E-06 |
| NEG1779 | Pelargonic acid | 1.51 | 157.20074 | [M-H]- | 157.2 | 69.08023:6205 79.71713:5218 89.13125:312981 90.1367:6657 97.07645:7737 113.19607:6950 115.14149:7846 141.94867:5416 144.71094:5617 147.59695:5893 | 4.70738E-06 |
| POS13290 | n-3alpha-yl-O-be | 4.616 | 633.3642 | [M+2H]2+ | 633.36322 | 67.05389:62726 69.03294:62783 73.02811:263251 73.06432:93764 81.03229:85335 83.0477:79886 87.04334:608498 89.05896:3244339 90.0619:123708 95.04893:174189 99.04339:100212 111.04189:266453 113.05927:113170 113.1652:62702 133.08543:1007164 137.0598:88154 | 1.5473E-06 |
| POS8382 | Cryptomaldamide | 5.19 | 400.25473 | [M+2H]2+ | 400.25549 | 59.04858:46073 67.015:6651 87.04334:8280 89.05896:46653 101.0593:5604 103.07444:10843 127.95014:5198 133.08545:11379 262.60263:6014 307.50278:7369 335.51199:6585 | -1.8988E-06 |
| POS8644 | Roxithromycin | 4.963 | 419.27377 | [2M+H]2+ | 419.27301 | 57.03287:36771 58.04042:44168 59.04859:1046584 60.05226:24137 65.04819:44839 67.05389:10654 69.06869:13857 73.02812:213131 73.06432:37940 80.0543:98672 85.06337:40000 87.04335:222274 87.06079:104870 87.07999:21985 89.05896:1663653 90.06191:45792 94.06947:14388 101.05931:74030 102.06606:50977 103.07444:168670 104.0796:10383 107.06821:13701 109.07315:62752 116.0798:9014 117.0909:12747 124.07726:21506 129.08911:25685 131.06766:24749 133.08545:543876 134.08684:29226 138.09604:10695 140.09358:12090 147.10149:69791 153.09918:12457 177.11285:48734 191.1256:13042 | 1.81266E-06 |

**Differences in metabolites between the WJW group, the Model group and the Control group**

| Alignment ID | Metabolite name | Rt(min) | Expreiment Mz | Adduct type | Reference m/z | MS/MS spectrum | PPM |
|---|---|---|---|---|---|---|---|
| POS11613 | CHEBI:69627 | 5.196 | 557.38458 | [M+H]+ | 557.38373 | 57.03287:68192 59.04859:1426531 60.05226:22336 69.06868:16224 73.02812:30907 73.06432:66693 85.06506:16565 87.04335:101496 87.07999:14345 89.05896:331344 101.05931:133579 103.07444:374703 115.07378:27471 117.05281:73537 117.0909:59987 131.07088:55951 133.08545:42112 147.10149:77117 161.11441:46865 | 1.52498E-06 |
| POS8199 | Alstovine | 5.725 | 387.19226 | [M+CH3OH+H]+ | 387.1915 | 51.54529:6444 51.69024:6817 55.05365:6559 57.06896:36001 59.04858:6559 71.04816:33403 74.83275:5981 83.0477:43632 101.0593:21270 173.43034:13159 175.01567:53935 211.91226:5915 214.52664:6403 | 1.96285E-06 |
| POS12628 | Coriandrinol | 5.713 | 599.42914 | [M+H]+ | 599.42822 | 57.03286:45848 59.04858:470607 67.46854:7742 69.06991:8637 70.37264:7815 70.79802:7299 73.02811:19200 87.04334:21683 89.05895:85190 91.19027:8508 99.07938:9185 101.0593:21007 103.07443:21528 115.07377:11938 117.09089:73108 133.08543:18864 147.10147:8020 155.0584:7607 173.3911:16437 200.63062:7164 480.31015:8204 563.1051:7694 | 1.5348E-06 |
| POS13032 | 7',8'-Tetradehydro | 6.297 | 619.37714 | [M+H]+ | 619.37811 | 51.86788:5622 530.44775:5263 | -1.5661E-06 |
| POS13958 | cheilocline F | 5.492 | 681.45233 | [M+2H]2+ | 681.45129 | 59.04859:306302 73.02812:48139 87.04335:65890 89.05896:393891 101.05931:62129 103.07445:79381 129.08911:27142 133.08545:134914 147.10149:48421 147.89014:27807 173.43526:39266 | 1.52615E-06 |
| POS5228 | Deoxyadenosine | 4.68 | 251.10112 | [M+NH4]+ | 251.10181 | 59.04858:52639 60.04426:70014 69.06992:10529 70.02779:77145 74.02328:15442 87.04334:14689 88.03896:207029 94.06359:180511 95.06884:6101 98.02254:9480 99.00742:32831 104.04768:16240 107.43317:5637 116.03146:11602 117.05281:8812 118.06566:5322 120.04358:56815 130.04884:28260 132.04306:17934 136.07611:13891 146.05721:230585 147.06317:13812 150.05342:17259 158.04503:14454 163.19865:6458 173.38622:10070 174.05498:45442 177.6405:5237 192.06537:12410 | -2.7479E-06 |
| POS10530 | Thr Thr Gly Leu Ile | 6.514 | 518.31921 | [M+Na]+ | 518.31842 | 59.04858:12918 60.10926:5981 62.53428:5966 76.55672:6150 80.40308:5481 86.09541:25027 146.97894:35421 331.8078:5767 361.46249:5952 | 1.52416E-06 |
| POS4891 | 3,HCl-(??)-N5-Hyd | 4.856 | 239.13814 | [2M+H]+ | 239.1389 | 68.04949:10488 80.04967:18785 84.07957:100441 89.05895:10258 130.08707:9249 134.09683:383236 135.09956:16940 197.1311:14065 | -3.1781E-06 |
| POS7961 | Val-Arg-Pro | 5.612 | 371.23929 | [M+H]+ | 371.24011 | 52.73953:5807 59.03397:5750 193.61818:5194 329.18835:6615 371.23489:8925 | -2.2088E-06 |
| POS7819 | 11-epoxy-9-fluoro | 4.505 | 363.1973 | [M+Na]+ | 363.1965 | 173.39111:8444 281.01971:6255 289.36584:6283 363.1955:12277 | 2.20266E-06 |
| POS9516 | pirost-9(11)-en-1 | 4.84 | 471.31198 | [M+H]+ | 471.31119 | 57.03287:12458 59.04859:331752 73.02812:9897 83.38518:5882 87.04335:30374 89.05896:269482 90.06375:6392 95.44421:5320 101.05931:24970 103.03849:9236 103.07444:66995 133.08545:42039 147.10149:21312 388.28345:5701 | 1.67617E-06 |
| POS7551 | Arg Ser Ser | 4.509 | 349.1821 | [M+Na]+ | 349.18289 | 62.11793:5892 67.20391:5960 70.06431:10778 86.05939:8665 165.3967:6089 166.37866:6413 173.43036:6489 | -2.2624E-06 |

**Differences in metabolites between the WJW group, the Model group and the Control group**

| Alignment ID | Metabolite name | Rt(min) | Expreiment Mz | Adduct type | Reference m/z | MS/MS spectrum | PPM |
|---|---|---|---|---|---|---|---|
| POS11797 | -yl)-1H-cyclopent | 5.556 | 563.37207 | [M+Na]+ | 563.37299 | 57.03287:9100 59.04858:24525 66.65124:6273 72.53472:7669 87.04334:11665 89.05896:33263 103.07444:7267 104.10696:14628 104.63825:5839 133.08543:8499 141.77881:5799 165.37384:6538 184.06929:7804 344.60162:5601 350.31827:6336 | -1.633E-06 |
| POS14256 | ta-D-glucopyrand | 4.955 | 705.45593 | [M+H]+ | 705.45709 | 59.04858:88066 73.02811:7844 87.04334:40200 89.05895:236352 101.0593:25592 103.07443:34789 133.08543:94486 147.10147:21517 156.65466:6609 177.11284:12295 184.07465:10663 399.92606:7569 | -1.6443E-06 |
| POS12084 | Sevcorine | 5.201 | 574.41101 | [M+H]+ | 574.4101 | 59.04859:415155 73.02812:15506 73.06432:81514 87.04335:36123 89.05896:199439 101.05931:36242 103.07445:124824 117.05281:154756 117.0909:25395 131.06766:75721 133.08545:53150 147.10149:37025 | 1.58423E-06 |
| NEG4249 | ethachlor CGA369 | 1.208 | 242.05011 | [M-H]- | 242.0493 | 59.53994:5499 62.06197:10364 71.2459:6079 79.05367:23326 83.19241:5742 97.08672:5531 161.03622:21782 163.03287:17835 | 3.34643E-06 |
| POS6274 | )-3-(4-hydroxyph | 6.146 | 290.15469 | [M+H]2+ | 290.1539 | 55.57048:6563 57.03287:35246 110.31171:5599 175.7039:5876 | 2.72269E-06 |
| NEG1775 | de, cyclopentyl iso | 1.259 | 157.1049 | [M-H2O-H]- | 157.1057 | 59.09475:28573 71.10927:39130 72.09502:6311 79.05367:106869 97.07645:129535 97.11958:205100 99.07355:13615 114.16649:92565 136.50891:5947 159.91479:5870 | -5.0921E-06 |
| POS5650 | eta-D-glucopyran | 4.483 | 267.14297 | [M+H-H2O]+ | 267.1438 | 59.04859:209303 71.04816:7917 73.02812:13696 73.06432:9664 77.05981:7144 87.04335:97005 88.21841:5677 89.05896:187607 101.05931:15789 103.03849:332742 103.07444:17763 104.04312:7029 110.05836:6470 117.05281:11657 119.06938:7902 133.08545:20337 165.7536:5287 173.42545:10359 266.04507:5893 | -3.1069E-06 |
| POS6244 | Arginylasparagine | 4.53 | 289.16101 | [M+Na]+ | 289.1618 | 51.45718:6340 70.02779:12580 86.09541:38435 88.03896:7095 156.97519:5914 163.43175:5297 173.39111:5979 232.69829:5819 289.1651:48759 | -2.732E-06 |
| POS2582 | C)OC(C(CC(C)O)C) | 5.496 | 161.11629 | [M+CH3OH+H]+ | 161.1171 | 67.86302:6189 123.64797:5879 | -5.0274E-06 |
| POS4237 | Citric acid | 1.01 | 215.01515 | [M+H]+ | 215.01601 | 70.0643:6076 96.92099:12553 98.9186:10567 110.9314:6271 131.91931:6299 134.01683:6754 | -3.9997E-06 |
| POS13649 | )-alpha-D-acetylri | 5.318 | 659.45282 | [M+H]+ | 659.45172 | 57.03379:66876 59.04956:789822 73.06432:41776 83.54895:27459 87.04335:72894 89.05896:328663 101.05931:96236 103.07444:238795 115.07378:26832 117.05553:55923 131.07088:29420 133.08545:57155 147.10149:64623 161.11441:26692 190.06322:24163 | 1.66805E-06 |
| POS12888 | dyl pseudopterane | 5.672 | 611.39319 | [M+2H]2+ | 611.39423 | 57.03286:18838 59.04858:107939 73.02811:18743 85.06504:14420 87.04333:15714 89.05894:100135 101.05929:24600 103.07442:41330 133.08542:26485 147.10147:14782 160.2352:10512 | -1.701E-06 |
| NEG4833 | (-)-Epipolasin A | 0.488 | 262.16446 | [M-H]- | 262.1636 | 84.25964:5170 100.0459:11700 153.12859:5074 | 3.28039E-06 |
| POS12675 | olestane-3beta,6a | 5.323 | 601.39343 | [M+2H]2+ | 601.39447 | 57.03287:54435 59.04859:859064 60.05226:35649 73.02812:102762 73.06432:50234 85.06337:38465 87.04335:181364 87.06254:44298 87.07999:44771 89.05896:1238278 90.06374:36665 101.05931:153091 102.06606:21323 103.07444:270837 109.0756:24811 115.07378:19880 117.0909:34380 129.08911:28073 131.06766:23646 133.08545:446052 134.09018:29466 147.10149:143483 177.11285:44140 191.1256:28717 307.53751:16936 | -1.7293E-06 |

**Differences in metabolites between the WJW group, the Model group and the Control group**

| Alignment ID | Metabolite name | Rt(min) | Expreiment Mz | Adduct type | Reference m/z | MS/MS spectrum | PPM |
|---|---|---|---|---|---|---|---|
| POS11521 | His Ile Lys Arg | 5.463 | 553.35583 | [M+2H]2+ | 553.35681 | 59.04859:215099 61.68283:11550 61.80683:13336 67.015:20031 73.02812:24862 73.06432:19312 87.04335:31828 87.07999:13960 89.05896:199596 101.05931:40023 103.07444:60091 104.10697:50848 133.08545:65203 147.10149:36903 158.09198:12769 184.07466:16179 314.30731:14461 | -1.771E-06 |
| NEG2746 | Diethyl L-malate | 1.319 | 189.08087 | [M-H]- | 189.08 | 73.0612:12532 109.03119:7235 125.04041:6868 145.13034:14383 189.08273:7862 | 4.60123E-06 |
| POS4736 | SCHEMBL4748090 | 5.798 | 233.17374 | [2M+H]+ | 233.17461 | 55.24187:5609 57.03287:17170 59.04858:21868 | -3.7311E-06 |
| POS11284 | anine-3beta,17,23 | 6.111 | 546.34155 | [M+Na]+ | 546.34253 | 86.0954:20507 93.39707:5656 97.71894:5703 104.10696:40334 105.10878:7465 184.06927:20814 273.52429:5481 315.59171:5348 340.05838:6352 | -1.7937E-06 |
| POS12054 | a-Hydroxymilbem | 4.867 | 573.33826 | [2M+H]+ | 573.33929 | 57.03287:19053 59.04859:212199 73.02812:12020 73.06432:7762 87.04335:295959 87.07999:9202 89.05896:269303 101.05931:92747 103.03849:134737 103.07444:61656 104.3103:7845 115.07378:9094 117.05281:35589 131.06766:13682 131.87703:8857 133.08545:86616 147.10149:33234 173.43036:10327 177.1078:10853 | -1.7965E-06 |
| NEG9811 | allocatechin 3,4'-d | 6.248 | 609.0907 | [M-H]- | 609.0896 | 89.1313:907206 90.13492:34712 156.12057:6322 409.70682:8289 591.94617:5607 | 1.80597E-06 |
| POS7398 | tadecenyl)-1,3-be | 6.602 | 341.246 | [M+H]+ | 341.24512 | 82.54128:5672 175.02563:6388 305.6582:5038 | 2.57879E-06 |
| POS2880 | ramethyl-4-piperi | 0.682 | 171.14819 | [M+H]+ | 171.14909 | 55.05365:61066 57.03287:11399 67.05389:44024 69.06992:11642 70.06431:6126 71.04816:6819 71.92826:6813 72.04369:57363 72.08047:5949 79.05463:8663 81.0699:12644 83.08509:18180 89.06979:19943 93.06841:5858 109.06336:5644 109.10007:18048 123.59779:6135 166.5724:5543 | -5.2586E-06 |
| POS7334 | Istamycin AO | 4.561 | 338.19119 | [M+NH4]2+ | 338.19211 | 55.05365:49468 58.04137:31543 59.04858:61426 61.02845:24162 67.05389:26368 69.03294:776238 70.03661:25734 71.04816:22373 73.02811:184930 73.06432:46091 78.03804:83888 80.05429:48495 81.03229:32872 81.0699:20190 83.0477:151707 85.02799:22720 87.04334:640153 88.04604:21083 89.05896:1486336 90.0619:42664 95.04893:39479 97.06461:14050 99.04339:321315 100.05018:94695 101.0593:10558 102.06606:15836 103.03849:8502 107.07059:20344 109.06336:23678 111.04189:174394 112.04632:10697 113.05927:31595 117.09089:10199 122.06293:140023 122.56553:18337 125.0596:42861 129.05446:59089 131.07088:37711 133.08543:289594 134.09018:15252 137.05981:20468 144.07597:41692 151.09305:14173 155.07083:64386 166.08865:9512 173.07771:20122 175.09526:10805 177.11285:16091 | -2.7203E-06 |

**Differences in metabolites between the WJW group, the Model group and the Control group**

| Alignment ID | Metabolite name | Rt(min) | Expreiment Mz | Adduct type | Reference m/z | MS/MS spectrum | PPM |
|---|---|---|---|---|---|---|---|
| POS9493 | Simplexin B | 5.384 | 469.31497 | [M+2H]2+ | 469.31589 | 57.03287:117896 59.04859:2300633 59.05931:59214 60.05226:60339 65.04931:24666 69.06868:28764 73.02812:140915 73.06432:49915 83.0851:15798 85.06337:48771 87.04335:271250 87.06254:50152 87.07999:61435 89.05896:1297080 90.06191:46940 94.06947:73494 99.07939:11795 101.05931:263789 101.09641:11767 103.03849:20538 103.07444:482551 104.07732:28994 105.09028:13361 107.06821:11344 109.07315:13916 115.07378:41262 116.08248:19653 117.05281:23109 117.0909:50237 127.07501:11211 129.08911:37463 130.08708:12384 131.07088:29078 131.10634:18719 133.08545:433862 134.09018:23569 145.08403:24398 147.10149:244411 148.10667:9928 161.1188:28471 177.11285:37578 191.1256:32772 205.14238:12414 | -1.9603E-06 |
| POS11697 | Arg Thr Lys Arg | 4.996 | 560.36365 | [M+H]+ | 560.36261 | 59.04856:97321 73.02808:7807 73.06429:9247 76.54948:6580 87.0433:167181 89.05891:92453 101.05926:51732 103.03844:19276 103.07439:28034 117.05275:17101 131.0676:11931 133.08537:28163 145.85263:6712 177.02168:6394 | 1.85594E-06 |
| NEG8213 | Kazinol F | 0.992 | 395.22391 | [M-H2O-H]- | 395.22299 | 65.39328:5279 67.67252:5287 80.98167:5345 87.53817:5223 89.13125:10868 93.03925:321897 95.03763:159660 97.03745:18025 101.79763:5129 147.15915:8848 151.06712:23521 153.06755:21269 186.0121:5720 208.17513:5711 333.99783:5874 | 2.3278E-06 |
| POS9687 | PC-M5' | 4.896 | 480.27539 | [M+H]+ | 480.27448 | 53.64094:6458 65.62093:6215 126.02025:18856 150.00212:6125 | 1.89475E-06 |
| POS6422 | Pizotifen | 5.074 | 296.1456 | [M+2H]2+ | 296.14651 | 55.05364:16814 57.06895:174141 58.06415:8749 59.04857:8589 59.07294:6187 60.08023:13534 67.05388:33682 67.2394:5981 68.82522:5669 69.0699:22609 71.08546:90873 75.26418:6891 78.54684:5994 81.06831:35008 83.08508:24588 85.10043:38935 86.09538:38307 95.08475:60267 96.01749:5656 97.09951:13160 104.10694:37141 109.10004:25526 114.91485:6385 156.51996:8986 245.69836:5553 256.00311:7771 290.06473:6318 | -3.0728E-06 |
| POS6860 | anthraniloyl-beta- | 1.621 | 314.12149 | [M+H]+ | 314.12241 | 51.39856:6408 54.99667:6656 57.06988:8968 91.59369:6293 121.06409:133554 | -2.9288E-06 |
| POS174 | Dichloromethane | 0.763 | 84.95968 | [M+H]+ | 84.9606 | 56.04863:22764 56.96448:23144 59.79324:6337 84.04316:16629 | -1.0829E-05 |
| POS175 | Dichloromethane | 4.25 | 84.95968 | [M+H-H2O]+ | 84.9606 | 54.61502:5320 56.04863:26433 56.091:6003 79.57497:5269 84.04482:11852 | -1.0829E-05 |
| POS5611 | Vaccenic acid | 7.137 | 265.2511 | [M+H]+ | 265.25201 | 61.67659:5453 84.07957:6347 111.40971:6016 114.95457:6407 173.39601:5858 201.17491:5557 | -3.4307E-06 |
| POS7377 | Bursin | 4.589 | 340.20816 | [M+H]2+ | 340.20911 | 58.04137:707509 69.03294:21970 71.04816:62924 73.02811:1839392 73.06432:132891 74.03149:43183 80.05429:962667 80.55504:76281 87.04334:361428 89.05896:3789226 90.0619:124674 102.06606:490082 102.56839:53624 107.07058:26618 111.07206:64520 115.07378:31855 117.09089:22842 124.08022:176716 131.07088:41564 133.08543:1136328 134.09018:34958 146.09511:56660 155.09987:31976 177.11285:87227 | -2.7924E-06 |

**Differences in metabolites between the WJW group, the Model group and the Control group**

| Alignment ID | Metabolite name | Rt(min) | Expreiment Mz | Adduct type | Reference m/z | MS/MS spectrum | PPM |
|---|---|---|---|---|---|---|---|
| POS4947 | -(4-acetylamino-b | 4.534 | 241.20316 | [M+2H]2+ | 241.20219 | 55.05365:7736 57.06896:68681 58.06416:2763218 59.06808:89643 67.05389:63876 69.06868:17389 79.05312:24329 81.06833:379873 82.07301:25212 84.07957:123835 85.06337:14082 95.08477:49800 121.09843:11305 123.11523:306716 124.11882:29187 138.54323:7262 140.14345:69996 | 4.02152E-06 |
| POS6395 | purin-6-yl)carbam | 5.751 | 295.11584 | [2M+H]+ | 295.1149 | 55.01682:6428 57.06895:13921 59.04858:10698 67.05389:10906 73.02811:6042 78.6007:4934 81.06989:8065 83.08508:6707 85.02798:8604 93.07033:6373 95.08277:8775 97.09952:5395 104.02032:5922 105.06944:5813 133.06235:21022 163.47661:5188 179.13976:17382 | 3.1852E-06 |
| POS3043 | uanidinosuccinic a | 1.091 | 176.06526 | [M+H]+ | 176.06621 | 60.05523:190390 70.06429:845409 71.04814:19765 71.06744:54596 72.08046:24009 84.08121:8979 112.08705:8669 113.06956:61703 114.05197:10024 116.06902:51195 117.07182:6868 130.0966:17345 159.0741:9593 175.12009:8320 | -5.3957E-06 |
| NEG3943 | dehydroschoberine | 0.757 | 231.18767 | [M-H]- | 231.18671 | 67.90976:5778 97.15044:6255 127.79837:6022 | 4.15249E-06 |
| POS14929 | Oligomycin C | 8.719 | 775.53864 | [M+NH4]+ | 775.5354 | 78.02028:7026 78.65312:6033 79.51556:6056 82.88864:7619 100.9307:4764 111.60696:5977 130.14764:5777 221.2182:5478 306.94766:6098 353.1517:5441 752.54596:6685 | 4.17776E-06 |
| NEG9405 | 6,7,7,8,8-hexadeca | 5.174 | 514.90167 | [M-H]- | 514.9007 | 80.47415:6165 84.43423:5234 106.70364:5642 143.62971:5224 | 1.88386E-06 |
| POS4498 | SCHEMBL2268896 | 2.598 | 225.05943 | [M+H]+ | 225.06039 | 58.06512:12460 74.39811:5599 79.05313:11946 91.0539:9945 93.06841:25297 115.93221:5744 121.06696:6799 123.70115:5058 163.1002:5060 227.04024:10378 | -4.2655E-06 |
| POS7795 | serylvalylarginine | 4.769 | 361.21835 | [M+Na]+ | 361.2193 | 58.04136:9454 59.04858:48441 73.02811:30879 80.05428:9866 87.04333:12174 89.05894:100509 90.0619:7012 133.08542:19612 253.44498:4951 318.4404:5197 | -2.63E-06 |
| POS6986 | Allopregnanolone | 9.536 | 319.26096 | [M+H]+ | 319.26001 | 51.78613:7408 67.05386:6215 81.0683:6781 93.06837:17456 95.08472:8177 107.08482:7951 119.08607:6210 121.10124:10538 212.5829:6045 316.8718:5836 | 2.97563E-06 |
| POS8281 | DTXSID40693955 | 5.056 | 393.24561 | [M+CH3OH+H]2+ | 393.2446 | 57.03286:8746 59.04858:35580 70.68684:5779 76.29126:5243 87.04333:9185 87.39496:6144 89.05895:49405 101.0593:13576 103.07443:7488 133.08543:18297 138.69392:5703 155.00864:7420 | 2.56838E-06 |
| POS12429 | 386093-01_C34H4 | 6.131 | 590.32086 | [M+Na]+ | 590.32202 | 59.04859:6348 59.73867:5925 67.05389:17007 73.79539:5505 79.05313:13892 81.0699:9701 86.09541:86286 91.0539:12814 93.06841:12853 104.10697:20830 107.08487:7944 119.08334:7685 120.9869:5889 123.31204:5899 146.97896:104460 460.44394:5660 | -1.965E-06 |
| POS12351 | Val-Glu-Ile-Pro-Gl | 8.782 | 586.30707 | [2M+H]+ | 586.30823 | 86.09541:42111 104.10696:279897 105.1111:10117 146.97894:40326 173.39111:14345 | -1.9785E-06 |
| POS8787 | Palau'imide | 5.276 | 429.27338 | [M+2H]2+ | 429.27429 | 57.03286:8078 59.04858:169278 60.05225:6477 67.01499:14280 69.03294:6572 73.02811:5794 81.03072:10236 85.06505:5593 87.04333:13633 89.05895:97862 91.05389:12442 101.0593:10057 103.07443:36596 133.08543:32376 147.10147:13437 387.87292:7334 | -2.1199E-06 |
| POS7299 | Pipericine | 7.722 | 336.32452 | [M+NH4]+ | 336.3255 | 55.48339:6753 55.876:6022 64.58645:6061 66.81867:5411 157.44508:5415 173.39111:13440 335.26138:5837 | -2.9138E-06 |

**Differences in metabolites between the WJW group, the Model group and the Control group**

| Alignment ID | Metabolite name | Rt(min) | Expreiment Mz | Adduct type | Reference m/z | MS/MS spectrum | PPM |
|---|---|---|---|---|---|---|---|
| POS10865 | Cholylvaline | 5.123 | 530.34625 | [M+H]+ | 530.34521 | 57.03287:11319 59.04859:222796 69.06992:5704 73.02812:23499 73.06432:14832 85.06506:9730 87.04335:222920 87.06254:12243 87.07999:17550 89.05896:359254 90.06375:11755 101.05931:83294 103.0385:13641 103.07445:70159 109.0756:6347 117.05281:16226 117.08818:9561 129.08911:11479 131.07088:19808 131.10634:6099 133.08545:146562 134.08685:6598 145.08403:8277 147.10149:34862 173.43036:11283 177.11287:21267 191.1256:7692 282.47211:5720 529.29529:6452 | 1.96099E-06 |
| POS9219 | :)-Eicoseneoylcarn | 5.772 | 454.38867 | [M+H]+ | 454.38965 | 57.03286:16430 57.06895:6063 57.78485:5819 59.04857:12049 60.08024:26214 61.65057:5446 85.02798:138083 90.2034:5032 152.38158:5777 157.5979:6596 | -2.1567E-06 |
| NEG9741 | Bartramiaflavone | 5.139 | 585.06635 | [M-H]- | 585.0675 | 51.88619:5547 57.33037:6239 61.31321:5860 74.51412:6798 89.13125:1512759 90.13486:55324 363.81647:7398 | -1.9656E-06 |
| POS8348 | Napelline N-oxide | 6.748 | 398.23111 | [M+H]+ | 398.23013 | 149.02057:11000 214.37152:5354 267.62708:5763 355.32816:6300 | 2.46089E-06 |
| POS8046 | ıtibiotic KA 6606 X | 5.699 | 376.25641 | [M+H-H2O]+ | 376.2554 | 57.06893:699280 58.07269:10234 67.26308:5204 70.55553:5401 116.75143:6984 125.07156:8673 126.0536:21966 140.03654:5666 208.147:6492 209.12428:43279 210.11501:7913 213.08969:6199 218.12753:8382 219.11333:6674 275.17349:7102 293.17691:7592 302.17621:13491 376.26236:14500 | 2.68435E-06 |
| POS9507 | Ebastine | 5.062 | 470.3064 | [M+2H]2+ | 470.30539 | 57.03287:43096 58.04042:26597 59.04859:972238 60.05226:23087 65.04819:28614 69.06992:16341 73.02812:179898 73.06432:46282 80.0543:63123 83.04932:11665 85.06337:33685 87.04335:232396 87.06079:83016 87.07999:23623 89.05896:1608846 90.06191:47124 94.06947:17818 101.05931:142289 102.06606:46435 103.07444:213219 109.07315:44856 109.57641:10420 115.07378:11588 117.05281:14388 117.0909:18327 124.08022:10414 127.07501:10495 129.08911:26812 131.06766:25568 133.08545:525393 134.08684:26022 140.09358:10795 145.08403:11520 147.10149:103445 177.11285:52604 191.1256:9549 | 2.14754E-06 |
| POS12233 | Manzamine X | 4.833 | 581.34735 | [M+Na]+ | 581.34851 | 104.79475:14431 | -1.9954E-06 |
| POS7958 | Octaethylene glyco | 4.499 | 371.2265 | [M+H]+ | 371.22751 | 59.04859:14098 73.06432:6886 87.04335:41143 89.05896:878039 90.06191:15323 101.05931:6933 107.07059:8062 131.08699:6423 133.08545:237512 177.11285:17984 285.99164:5706 | -2.7207E-06 |
| POS8891 | hosphoryl]oxy-2-h | 6.451 | 436.27899 | [M+H-H2O]+ | 436.28 | 84.57517:5229 90.81367:5393 139.2675:5804 226.54878:6242 235.64577:5658 327.74368:6693 | -2.315E-06 |
| POS8678 | (-)-Terpendole B | 5.173 | 422.2674 | [M+2H]2+ | 422.2684 | 57.03286:13210 59.04858:182542 67.01499:21936 81.02915:8017 83.87628:5473 87.04333:18935 89.05895:114294 101.0593:18614 103.07443:42228 117.05552:5882 131.06764:6691 133.08543:28848 147.10147:10643 173.42543:9310 177.11284:6188 191.12558:5809 257.68277:5035 | -2.3682E-06 |

**Differences in metabolites between the WJW group, the Model group and the Control group**

| Alignment ID | Metabolite name | Rt(min) | Expreiment Mz | Adduct type | Reference m/z | MS/MS spectrum | PPM |
|---|---|---|---|---|---|---|---|
| POS10892 | Leu Asp Asn Arg | 4.473 | 531.28741 | [M+H-H2O]+ | 531.28851 | 79.6421:9188 86.09712:12182 87.05381:26044 89.05896:169138 133.08545:96417 143.89046:9582 146.05721:17239 173.42545:10498 177.1078:12586 188.07004:101378 205.09822:21849 247.72908:9404 248.81287:9322 | -2.0704E-06 |
| POS10216 | (4R,5R)-antillatoxin | 5.567 | 504.34421 | [M+H-H2O]+ | 504.34317 | 59.04859:55591 89.05896:23572 101.05931:9029 103.07444:12691 133.08545:9579 175.37425:6049 | 2.06209E-06 |
| POS12556 | Dihydroxycanthaxa | 5.831 | 596.38879 | [M+2H]2+ | 596.39001 | 57.03379:25446 59.04859:152492 67.015:12316 73.02812:25803 73.06432:22756 87.04335:42468 89.05896:127194 101.05931:15587 103.07445:19902 133.08545:37316 156.90764:11128 166.20825:10197 271.25543:11374 | -2.0456E-06 |
| NEG4399 | TADECA-3,6,9-TRI | 0.839 | 247.24217 | [M-H]- | 247.24319 | 64.0225:5693 113.12118:40005 133.25772:5213 | -4.1255E-06 |
| POS3868 | AMOZ | 2.237 | 202.11746 | [M+H]+ | 202.1185 | 55.05365:119164 56.05764:6675 59.04858:9776 67.05389:6586 70.0643:136839 71.04816:29730 71.05974:76454 83.0477:21601 101.0593:34106 110.72093:5593 111.04189:13509 113.0696:13377 114.05462:7124 157.88727:6214 | -5.1455E-06 |
| POS9718 | Xestobergsterol B | 6.512 | 481.31287 | [M+H]+ | 481.3139 | 135.48819:5754 342.19617:6394 | -2.14E-06 |
| POS11481 | PE 22:0 | 5.89 | 552.36475 | [M+2H]2+ | 552.36591 | 57.03287:32364 59.04859:160838 73.02812:48004 73.06432:8898 85.06506:15372 86.09541:13243 87.04335:41960 87.06079:6514 87.07999:12448 89.05896:125523 101.05931:29687 103.07444:27711 104.10697:58575 115.07378:7187 129.08911:6066 133.08545:36473 147.10149:14657 173.39111:10247 184.06931:19733 234.51561:6047 | -2.1001E-06 |
| POS15149 | LacCer(d18:1/12:0) | 7.051 | 806.56409 | [M+H]+ | 806.56238 | 96.4697:6680 | 2.12011E-06 |
| POS13933 | Cimiracemoside D | 4.602 | 679.40662 | [M+2H]2+ | 679.40515 | 87.04334:261744 89.05896:1951863 133.08543:766098 194.5706:85597 342.42755:85462 469.50879:108563 | 2.16366E-06 |
| NEG4861 | idecylbenzene-1,3 | 1.051 | 263.21698 | [M-H]- | 263.21805 | 87.11257:8198 89.13129:313336 90.13491:14096 93.0393:531192 95.03768:119175 147.15923:120996 148.16444:6571 151.0672:6294 | -4.0651E-06 |
| POS10430 | nethyl-1-oxo-2-pi | 5.784 | 514.27881 | [M+H]+ | 514.27991 | 59.04858:37228 73.06432:26921 85.06506:7012 87.04334:9315 89.05896:29110 95.08477:6550 101.05712:9332 104.10696:6575 106.33912:5185 126.02023:53819 131.10634:7123 | -2.1389E-06 |
| POS11941 | phlomiside | 8.96 | 569.20874 | [M+2H]2+ | 569.20752 | 62.99562:5907 79.55058:5110 104.10696:25395 138.2146:5137 184.07466:10556 366.74438:6801 | 2.14333E-06 |
| NEG2214 | Glycylproline | 8.998 | 171.07645 | [M-H]- | 171.07751 | 65.77522:6130 79.0537:8499 91.19855:6752 100.76936:5625 127.03277:51219 171.30643:8510 | -6.196E-06 |
| NEG2215 | Glycylproline | 0.143 | 171.07645 | [M-H2O-H]- | 171.07751 | 109.19514:6443 127.03276:23681 134.32732:5374 171.30641:5507 | -6.196E-06 |
| POS5938 | Obscuraminol A | 6.585 | 278.24634 | [M+H]+ | 278.24741 | 98.85948:6284 111.15255:5710 149.02057:21243 | -3.8455E-06 |
| POS5106 | methoxyphenyl)-2 | 4.905 | 247.08507 | [M+H]+ | 247.084 | 61.811:5658 66.62203:5495 130.02655:5917 173.43034:9058 | 4.33051E-06 |
| POS9849 | caryolanemagnolo | 5.971 | 487.32169 | [M+H]+ | 487.32059 | 59.04858:18644 66.91378:5588 89.05895:19841 105.14582:6132 138.83788:5569 168.3127:5941 170.74451:5189 | 2.25724E-06 |

**Differences in metabolites between the WJW group, the Model group and the Control group**

| Alignment ID | Metabolite name | Rt(min) | Expreiment Mz | Adduct type | Reference m/z | MS/MS spectrum | PPM |
|---|---|---|---|---|---|---|---|
| POS9374 | -3,4-dehydro-apo | 5.261 | 462.3089 | [M+2H]2+ | 462.31 | 57.03287:68483 59.04859:1640159 60.05226:37212 65.04819:28519 67.05389:7945 69.06992:18982 73.02812:108145 73.06432:26699 80.0543:17455 81.06834:9411 85.06337:37671 87.04335:212219 87.06254:50748 87.07999:35953 89.05896:1176095 90.06191:47001 94.06947:35528 99.07939:10329 101.05931:146547 103.03849:16839 103.07444:333822 104.07732:14685 107.07059:12041 109.0756:14204 115.07378:13589 116.08248:20526 117.05281:12288 117.0909:25986 127.07501:9358 129.08911:28441 131.06766:20109 131.10634:11361 133.08545:327602 134.09018:19521 145.08403:8459 147.10149:147866 161.1188:9065 177.11285:24585 191.1256:24974 | -2.3794E-06 |
| POS7883 | Betaenone A | 5.159 | 367.24951 | [M+2H]2+ | 367.24841 | 57.03287:82888 59.04859:1792934 60.05226:41220 65.04819:30358 69.06992:10700 73.02812:124861 73.06432:25247 85.06337:32324 87.04335:136399 87.06254:32476 87.07999:23482 89.05896:839407 90.06191:26550 94.06947:35327 101.05931:117040 103.07444:240294 104.07732:10586 109.0756:15513 116.08248:11466 117.05281:10242 117.0909:19812 129.09227:10554 131.07088:12461 133.08545:185155 134.08684:11321 147.10149:97569 177.11285:12571 191.1256:10145 | 2.99525E-06 |
| NEG9457 | pferol 3,7,4'-tri-O- | 5.894 | 524.90985 | [M-H]- | 524.91101 | 59.08406:64413 67.13567:8066 79.05222:24424 107.21417:9070 119.23064:5283 140.17944:10809 147.16307:5463 352.116:5702 434.19601:6421 | -2.2099E-06 |
| POS5577 | Protriptyline | 4.97 | 264.17349 | [M+Na]+ | 264.17459 | 59.04859:30915 73.06432:6336 77.01915:5714 87.04335:18298 89.05896:14100 101.05931:9161 173.38622:6861 | -4.1639E-06 |
| POS12378 | ganorbiformin A | 4.969 | 587.35907 | [2M+H]+ | 587.35779 | 57.03287:19218 59.04859:262800 73.02812:16307 73.06432:11398 85.06337:13281 87.04335:528738 89.05896:240711 101.05931:136286 103.03849:102338 103.07444:68206 115.07378:13795 117.05281:40507 131.06766:19995 133.08545:75808 147.10149:36730 478.21463:10321 | 2.17925E-06 |
| POS9696 | ahydro-1H-cyclop | 5.367 | 480.3129 | [M+2H]2+ | 480.314 | 59.04857:103646 60.05124:6194 62.05908:6808 67.01498:9006 73.0281:10117 73.13141:6883 81.06831:8845 85.06335:9736 87.04333:12205 89.05894:73041 90.91968:7216 101.05928:6046 103.07442:20538 105.06943:5431 126.02021:107504 133.0854:12086 159.1172:9335 161.12755:8955 205.14232:6011 205.30017:5483 208.05679:6945 359.60992:5718 401.25186:6022 | -2.2902E-06 |
| NEG3105 | Spermine | 0.809 | 201.2074 | [M-H]- | 201.2085 | 61.06563:9276 62.6869:5420 64.61039:6008 71.497:6145 81.87756:5654 89.09516:25801 89.13128:10589 103.21892:6510 111.10325:24071 149.19171:5498 | -5.467E-06 |
| POS11162 | pxy-5??-cholest-3 | 5.876 | 541.37463 | [M+2H]2+ | 541.37341 | 57.03287:55586 59.04859:377973 60.08025:175833 73.02812:49737 86.09541:411670 87.04335:44070 89.05896:165272 101.05931:55497 103.07444:74900 104.10697:1493231 124.99956:88707 133.08545:44281 184.07466:902685 | 2.25353E-06 |

**Differences in metabolites between the WJW group, the Model group and the Control group**

| Alignment ID | Metabolite name | Rt(min) | Expreiment Mz | Adduct type | Reference m/z | MS/MS spectrum | PPM |
|---|---|---|---|---|---|---|---|
| POS11897 | Hydroxymanzamin | 5.713 | 567.37061 | [M+2H]2+ | 567.36932 | 57.03287:32090 59.04859:225088 65.11697:7883 67.015:9217 69.06868:7730 73.02812:27208 73.06432:12085 80.0543:10143 81.03072:14445 85.06337:6232 87.04335:42018 87.06254:8100 87.07999:15385 89.05896:147403 90.06191:7741 90.55772:6238 101.05931:32599 102.42354:6623 103.07444:62652 115.28882:6132 117.0909:11533 133.08545:48468 147.10149:20078 165.10016:10555 187.26393:6734 224.00723:7255 242.62587:6749 395.24411:6288 | 2.27365E-06 |
| POS9944 | -Acetylgentamicin | 5.973 | 492.30383 | [M+H]+ | 492.3027 | 53.31924:7416 59.04858:17883 60.08025:11439 86.09541:18227 89.05896:10776 104.10696:100376 168.57562:4870 173.04839:5391 184.07466:34363 | 2.29534E-06 |
| POS4790 | tylidene-1-Deoxy | 5.301 | 235.15277 | [M+H]+ | 235.1539 | 59.04859:36308 89.05896:12673 101.05931:7281 | -4.8054E-06 |
| POS4789 | tylidene-1-Deoxy | 4.962 | 235.15277 | [M+2H]2+ | 235.1539 | 57.03287:10776 59.04859:40011 75.04173:5838 86.09541:6763 87.04335:6496 89.05896:15004 99.54275:6028 101.05931:12771 104.31946:5300 173.38622:6893 | -4.8054E-06 |
| POS7363 | -1-ylidene)-5alpha | 5.958 | 339.26678 | [M+H-H2O]+ | 339.26791 | 55.05365:8739 57.06989:9879 59.04858:13318 67.05389:7170 69.06868:11374 76.29413:8746 81.0699:14839 83.08509:6751 93.06841:7072 95.08477:11768 121.09843:6574 180.99232:6405 192.80493:6095 | -3.3307E-06 |
| POS11698 | Arg Thr Lys Arg | 5.588 | 560.36389 | [M+2H]2+ | 560.36261 | 57.03287:22512 59.04859:242637 67.015:24194 81.03072:13634 85.90186:12964 87.04335:32407 89.05896:164672 101.05931:33101 103.07444:66674 104.10697:90582 133.08545:45681 147.10149:30558 161.25945:12601 165.10016:17400 168.84389:12040 184.07466:29869 191.1256:13898 239.59464:13538 424.36945:13016 | 2.28424E-06 |
| POS14079 | 25-trihydroxy-21al | 4.862 | 691.44312 | [M+H]+ | 691.44153 | 59.04859:232466 73.02812:26440 73.06432:24661 87.04335:121594 89.05896:857079 101.05931:58881 103.07444:87512 131.07088:26065 133.08545:322000 147.10149:51095 173.44016:17372 177.11285:41023 258.35931:15898 268.91992:15496 | 2.29954E-06 |
| POS6281 | no-1,3,4-hexadeca | 5.048 | 290.26773 | [M+H]+ | 290.26889 | 55.05366:10786 56.04863:189825 57.06897:135063 58.06417:132218 58.42599:5736 60.04426:25540 68.04951:8877 68.50143:5538 71.08419:38979 74.05887:115193 78.92046:5853 85.10046:11041 86.05939:13852 88.07445:175317 118.08495:35197 122.08031:36038 164.62282:5251 242.24483:35748 290.26651:13312 | -3.9963E-06 |
| POS11285 | Arg Ser Lys Arg | 5.349 | 546.3457 | [M+H]+ | 546.34698 | 57.03287:20336 59.04859:239650 67.015:23160 73.02812:21084 81.03072:13272 86.09541:10981 87.04335:27798 87.07999:9468 89.05896:203904 90.06191:10843 101.05931:24012 103.07444:61977 104.10697:83116 117.0909:9549 130.85844:8922 133.08545:77872 147.07468:10073 147.10149:27853 151.08508:14899 158.09198:14052 184.07466:18004 251.92677:8982 466.18372:8502 | -2.3428E-06 |
| POS9691 | PC(15:1(9Z)/0:0) | 5.383 | 480.30545 | [M+2H]2+ | 480.30661 | 57.03379:14320 59.04956:151841 67.01499:11221 73.02811:13915 80.43406:5472 81.03072:10483 87.04508:20484 89.05895:111443 101.0593:14821 103.07668:35478 133.08543:33690 147.10147:11830 158.09198:7813 | -2.4151E-06 |
| POS3920 | yl)oxy]pyridine-3- | 2.187 | 204.06197 | [M+Na]+ | 204.06313 | 55.93345:6871 60.04426:49156 61.03972:8693 91.0539:6082 204.9343:5201 | -5.6845E-06 |

**Differences in metabolites between the WJW group, the Model group and the Control group**

| Alignment ID | Metabolite name | Rt(min) | Expreiment Mz | Adduct type | Reference m/z | MS/MS spectrum | PPM |
|---|---|---|---|---|---|---|---|
| NEG9707 | Epicatechin | 6.194 | 579.00317 | [M-H]- | 579.00452 | 51.52467:7674 115.89837:5631 125.34409:5780 173.16458:5215 320.50644:7472 | -2.3316E-06 |
| POS12842 | idyl pseudopteran | 5.051 | 609.37994 | [M+H]+ | 609.37848 | 59.04859:157183 73.02812:36917 80.0543:26331 85.06337:19243 87.04335:74354 89.05896:483808 90.90853:15706 101.05713:40824 103.07444:43739 133.08545:215034 147.10149:33676 549.27478:18568 | 2.39588E-06 |
| POS7674 | handamide (20:l, n- | 7.171 | 354.33459 | [M+H]+ | 354.33578 | 51.79896:6119 64.54077:6654 199.06522:5344 352.11353:6186 | -3.3584E-06 |
| POS4432 | Carbofuran | 0.688 | 222.11081 | [M+CH3OH+H]+ | 222.112 | 58.06417:33664 62.63849:5800 73.04688:8793 91.0539:29708 114.88578:5683 119.04706:16113 135.04564:8455 209.00752:8800 225.04044:76549 | -5.3577E-06 |
| POS13262 | oxowitha-3,5,24-tr | 4.548 | 631.34912 | [M+H]+ | 631.3476 | 69.03294:531950 73.02811:243743 73.06432:134227 76.79319:61089 81.03229:96327 83.0477:276069 87.04334:1322209 89.05896:3918546 90.0619:65491 95.04893:152763 99.04339:1144402 107.07059:83202 109.06336:74333 111.04189:549180 113.05927:78300 125.0596:289524 129.05132:87907 131.07088:170648 133.08543:1947140 137.05981:128580 151.09705:71775 155.07083:464980 173.07771:106829 177.11285:207110 356.07733:73384 | 2.40755E-06 |
| POS6902 | xaden-TP NOA 40 | 4.548 | 316.17993 | [M+2H]2+ | 316.17871 | 55.05365:18529 59.04858:24176 67.05389:9614 69.03294:248663 70.03661:6902 71.04816:7926 73.02811:59434 73.06432:11612 78.03804:38009 81.03229:11849 81.0699:28225 83.0477:58645 85.02799:10159 87.04334:192220 88.04782:6438 89.05896:448073 90.0619:13322 94.71547:6485 95.04893:10730 99.04339:104753 99.07938:42340 100.05018:33996 107.07059:6587 109.06336:9850 111.04189:70949 113.05927:7981 117.09089:5997 122.06293:40646 125.0596:14454 129.05446:21948 131.07088:13397 133.08543:80573 137.05981:9095 142.13843:6809 144.07597:11241 155.06668:21540 173.0826:6402 238.23842:6823 | 3.85858E-06 |
| NEG1676 | 5-propanoic acid, | 0.976 | 153.06822 | [M-H2O-H]- | 153.067 | 58.00906:6838 79.05367:6097 93.03925:706937 93.15687:31392 95.03763:1316380 154.24498:6898 | 7.97037E-06 |
| POS10794 | l-7-oxo-3-oxaper | 5.25 | 527.33826 | [M+2H]2+ | 527.33698 | 57.03287:46245 59.04859:531889 73.02812:105948 73.06432:31040 87.04335:496867 87.06079:42824 89.05896:707738 101.05931:232147 103.07444:118606 104.10697:100247 105.10879:35413 133.08545:221115 147.10149:52471 157.54272:26623 160.78989:23542 173.44016:29715 184.06931:34472 225.7818:24805 | 2.42729E-06 |
| POS6384 | Lanyuamide I | 6.014 | 294.24127 | [M+NH4]+ | 294.24249 | 51.58743:5684 55.05365:13873 57.06895:6691 58.06416:16018 59.04858:13208 67.05389:9580 69.06867:6520 69.51213:5415 71.08418:19877 72.06339:5677 79.05312:5969 81.06989:16726 93.0684:10649 95.04893:10753 95.08475:10952 99.07938:9041 103.45982:5507 107.08485:6496 121.10128:6063 151.10899:10128 173.42542:10887 | -4.1462E-06 |
| POS3835 | minic acid; Dihydr | 2.265 | 201.08569 | [M+H]+ | 201.0869 | 55.05365:208072 56.94232:6443 59.04858:17433 70.0643:107285 71.05974:20129 73.06432:7664 81.06833:8008 83.0477:29349 101.0593:58942 111.04189:31051 113.0696:30268 113.96312:18595 114.05462:13930 129.05447:6787 | -6.0173E-06 |

**Differences in metabolites between the WJW group, the Model group and the Control group**

| Alignment ID | Metabolite name | Rt(min) | Expreiment Mz | Adduct type | Reference m/z | MS/MS spectrum | PPM |
|---|---|---|---|---|---|---|---|
| POS7557 | Dodecyl glucoside | 5.611 | 349.25684 | [2M+H]+ | 349.25809 | 53.00208:46898 55.05365:21485 57.03287:2262521 58.03662:29273 59.04858:645513 60.05225:8515 69.06868:16523 81.0699:61309 97.06461:35786 99.07938:81290 101.09641:13722 115.07378:94912 117.09089:27878 157.12312:40542 | -3.579E-06 |
| POS9429 | R-tetrol 1-O-alpha | 5.97 | 465.34085 | [2M+H]+ | 465.3421 | 53.00125:7212 55.05365:6134 57.03287:1001863 58.03662:16164 59.04858:291038 69.06991:8673 81.06833:29495 84.86993:5864 97.06461:18596 99.07938:66823 101.09641:14815 115.07378:76797 117.09089:25959 157.12312:56319 | -2.6862E-06 |
| POS5586 | nethyl-4-n-tridec- | 5.843 | 264.26724 | [M+NH4]+ | 264.26849 | 55.05365:17531 56.04863:6359 57.06896:12214 67.05389:25964 69.06868:16706 70.06431:10714 79.05312:28424 81.05736:7540 81.06834:23025 82.06503:166526 83.08509:8547 87.04334:5763 93.06841:22611 95.07282:8271 95.08477:12848 96.08018:30769 107.08486:9970 107.43556:6034 123.45921:5364 244.9557:5570 264.26468:36412 | -4.73E-06 |
| POS6358 | -beta-D-glucopyr | 5.031 | 293.19446 | [M+NH4]2+ | 293.19571 | 57.03287:7399 59.04859:55202 73.02678:6749 87.04335:11890 89.05896:26747 101.05931:26250 168.88631:5777 184.97366:6137 | -4.2634E-06 |
| POS6357 | -beta-D-glucopyr | 5.388 | 293.19446 | [2M+H]+ | 293.19571 | 59.04859:38699 76.22691:6115 87.04335:6678 89.05896:19281 101.05931:11689 122.48106:5725 173.43034:9639 222.31757:5454 | -4.2634E-06 |
| POS6356 | -beta-D-glucopyr | 5.168 | 293.19446 | [M+Na]+ | 293.19571 | 57.03287:9277 59.04859:39097 79.92984:5719 87.04335:8527 89.05896:20142 100.119:5361 101.05931:9225 | -4.2634E-06 |
| POS7262 | decatrienoic acid is | 7.32 | 334.30914 | [M+NH4]+ | 334.31039 | 73.8131:5978 173.42545:10635 | -3.739E-06 |
| POS4314 | pha-Acetyl-L-citru | 2.242 | 218.11223 | [M+NH4]+ | 218.11348 | 60.04326:10332 60.08025:11494 70.0643:172582 85.02799:88013 103.29053:5246 103.88376:5663 113.0696:76240 114.05462:66520 115.08438:28031 159.07413:29905 218.09305:6247 | -5.731E-06 |
| NEG1677 | 5-propanoic acid, | 1.081 | 153.06828 | [M-H]- | 153.067 | 60.17114:7239 79.0537:13233 93.03928:807346 93.15691:14844 95.03767:1450379 154.24504:7592 | 8.36235E-06 |
| POS11281 | anine-3beta,17,23 | 5.349 | 546.34113 | [M+2H]2+ | 546.34253 | 57.03287:20336 59.04859:239650 67.015:23160 73.02812:21084 81.03072:13272 86.09541:10981 87.04335:27798 87.07999:9468 89.05896:203904 90.06191:10843 101.05931:24012 103.07444:61977 104.10697:83116 117.0909:9549 130.85844:8922 133.08545:77872 147.07468:10073 147.10149:27853 151.08508:14899 158.09198:14052 184.07466:18004 251.92677:8982 466.18372:8502 | -2.5625E-06 |
| POS8915 | legonoxamine D | 5.059 | 437.27228 | [M+CH3OH+H]2+ | 437.271 | 59.04858:51499 64.85931:7350 67.015:13694 70.60906:5529 73.06432:7510 87.04334:12699 89.05896:82936 90.06191:6787 101.0593:13977 103.07444:11380 109.40909:5558 133.08545:20776 256.47888:5178 | 2.92725E-06 |
| POS3073 | nyl-ribo-hexose-a | 5.004 | 177.11082 | [M+H]+ | 177.11211 | 57.03287:6878 59.04859:13795 75.04312:6009 87.04335:6016 89.05896:7373 130.06477:5905 132.07892:18046 173.39111:13727 | -7.2835E-06 |
| POS11965 | 4S,5S,6R)-3,4,5-tril | 8.947 | 570.21643 | [M+2H]2+ | 570.2179 | 60.08025:8675 78.17443:5727 86.09541:10618 104.10696:59038 124.64912:5634 184.07466:25470 276.23544:5963 318.97809:6314 | -2.578E-06 |
| NEG7219 | Dodecyl glucoside | 0.951 | 347.24557 | [M-H]- | 347.24429 | 53.43378:6734 93.03925:329943 95.03763:217753 97.03745:17129 103.07946:12491 147.41237:5261 153.06754:11603 164.71239:5910 173.62527:5953 | 3.68617E-06 |

**Differences in metabolites between the WJW group, the Model group and the Control group**

| Alignment ID | Metabolite name | Rt(min) | Expreiment Mz | Adduct type | Reference m/z | MS/MS spectrum | PPM |
|---|---|---|---|---|---|---|---|
| POS13100 | tra-Ac-Glucoeryso | 8.651 | 622.09558 | [M+H]+ | 622.09277 | 57.30859:7273 163.38239:5280 173.3911:15519 184.22493:5325 283.61749:5576 432.82986:5820 | 4.51701E-06 |
| POS12383 | tenacibactin G | 5.045 | 587.39984 | [M+H]+ | 587.40137 | 57.03379:49528 59.04859:1150583 73.02812:174833 73.06432:106459 80.0543:36403 85.06337:68389 87.04335:364272 87.06254:45358 87.07999:39762 89.05896:2128959 90.06191:78061 101.05931:212338 102.06606:25877 103.07444:373822 109.0756:31911 115.07378:27184 117.05281:75104 117.08817:28534 129.08911:36362 131.06766:60847 133.08545:963873 134.09018:26638 147.10149:256485 161.11441:42884 173.39111:45294 177.11285:102401 191.1256:39949 205.57848:24855 336.53061:25361 | -2.6047E-06 |
| POS12353 | Leu Ser Leu Pro Glu | 5.604 | 586.34613 | [M+H]+ | 586.3446 | 55.58558:7301 59.04856:39906 60.08022:16137 86.09536:32586 87.04504:8769 89.05891:19117 101.47509:6543 104.10692:144929 113.55135:5751 124.99949:8850 137.26674:5675 145.44116:5577 184.07457:48053 | 2.60939E-06 |
| NEG818 | 4-Methylcatechol | 1.229 | 123.0424 | [M-H]- | 123.0437 | 80.0528:71794 87.12827:5272 94.14119:14123 124.15681:26277 | -1.0565E-05 |
| NEG2861 | ±)-trans-Nerolido | 0.647 | 193.16119 | [M-H]- | 193.1599 | 55.54954:6353 85.23931:6047 103.05704:52750 | 6.6784E-06 |
| POS9494 | Malonganenone C | 5.369 | 469.31851 | [M+2H]2+ | 469.3172 | 57.03286:56965 59.04858:1142699 59.0593:28750 60.05225:31066 69.0699:12710 73.02811:61570 73.06431:25556 85.06335:27969 87.04333:133959 87.06252:19345 87.07997:25264 89.05894:648978 90.0619:25966 94.06945:23606 101.05929:119793 102.06383:7071 103.07442:246499 107.07057:10209 115.07376:9573 117.05279:11042 117.09087:15522 129.0891:18761 130.08705:6924 131.06764:10362 131.10631:10108 133.08542:178867 134.08682:7983 143.1041:8150 145.08401:10484 147.10147:95276 161.11438:12011 173.396:12762 177.11282:10385 191.12556:12121 | 2.79129E-06 |
| POS11700 | gamma-Solanine | 5.05 | 560.39716 | [M+H]+ | 560.39569 | 57.03287:43652 59.04859:819372 73.02812:35597 73.06432:39036 87.04335:92548 87.07999:22864 89.05896:397039 90.06191:14676 101.05931:102712 103.07444:286688 115.07378:16524 117.05281:52576 117.0909:29847 126.69132:14541 131.06766:27036 133.08545:98508 134.34398:12696 147.10149:104265 161.1188:38344 214.75627:13536 325.95432:13449 | 2.62315E-06 |
| POS8841 | Fumifungin | 5.541 | 432.2977 | [M+2H]2+ | 432.29639 | 57.03287:73422 59.04859:1232213 60.05226:30514 61.02846:7108 65.04931:8454 69.06992:8820 73.02812:48709 73.06432:18833 85.06337:16643 87.04335:61455 87.06079:17964 87.07999:12595 89.05896:345651 90.06191:9339 94.06947:18745 101.05931:80332 101.1816:6440 103.07444:219893 104.0796:11684 115.07378:15155 117.0909:21544 129.08911:6620 131.07088:11849 133.08545:69052 147.10149:65316 161.11441:15682 191.1256:7175 215.3116:6205 | 3.03033E-06 |
| POS9544 | Betnesol | 4.411 | 473.17471 | [2M+H]+ | 473.1734 | 53.73895:6162 72.08048:11154 79.14983:5657 86.09541:8131 119.82919:5778 137.04604:1404390 138.04726:40284 188.07004:12017 | 2.76854E-06 |
| NEG9081 | -2-yl-2,3,4,5,6,7,7 | 0.865 | 469.4657 | [M-H]- | 469.46701 | 113.12114:215686 181.19063:8714 226.34309:5786 | -2.7904E-06 |

**Differences in metabolites between the WJW group, the Model group and the Control group**

| Alignment ID | Metabolite name | Rt(min) | Expreiment Mz | Adduct type | Reference m/z | MS/MS spectrum | PPM |
|---|---|---|---|---|---|---|---|
| POS15336 | Erythrosine | 6.873 | 858.64624 | [M+H]+ | 858.64398 | 53.67301:5841 90.49667:5979 176.32578:6196 278.14832:5738 287.21078:5204 779.2157:6077 | 2.63206E-06 |
| POS12957 | pha-cholestane-3l | 5.537 | 615.41187 | [M+2H]2+ | 615.41022 | 57.03287:44080 59.04859:834913 73.02812:47441 73.06432:44656 85.06337:39158 87.04335:140754 87.06254:22146 87.07999:33512 89.05896:715880 90.06191:22219 94.06947:23019 98.14563:19219 101.05931:127727 103.07444:256990 110.08812:18889 115.07378:23200 117.0909:25901 129.09227:24319 131.06766:23162 133.08545:219415 136.89111:17692 147.10149:101774 335.16913:18091 387.0867:18684 611.11322:18948 | 2.68114E-06 |
| POS13648 | leptasteroside L | 5.508 | 659.43463 | [M+2H]2+ | 659.4364 | 57.03379:45047 59.04859:610398 73.02812:59204 73.06432:39891 85.06506:23196 87.04335:119176 87.06254:32428 87.07999:21949 89.05896:665859 90.06191:27727 101.05931:133712 103.07444:178434 117.0909:37598 121.28181:21722 129.08911:30531 133.08545:245690 147.10149:94194 170.73015:25919 415.89944:21779 | -2.6841E-06 |
| POS7792 | anylaspartylarginin | 4.765 | 361.18155 | [M+Na]+ | 361.18289 | 58.04042:23351 59.04859:104682 65.04819:12460 73.02812:91447 80.0543:31045 80.41393:6032 87.04335:34073 87.06254:8323 89.05896:240925 90.06191:5522 101.05931:7642 102.06606:16955 124.07726:8817 131.06766:8963 131.90631:5727 133.08545:60119 173.43036:8546 300.88321:5418 | -3.71E-06 |
| POS4086 | epoxy-15R-hydro | 4.436 | 209.13698 | [M+NH4]+ | 209.13831 | 59.04859:329315 70.06431:58044 84.07958:16457 85.06506:6163 87.04335:14626 89.05896:314430 94.06359:27752 96.08018:9538 103.07445:14247 107.06821:7843 118.06566:9683 133.08545:25822 146.061:9335 148.11055:35581 187.04947:5586 | -6.3594E-06 |
| POS5183 | 5-methylsulfinylhe | 4.956 | 249.16536 | [M+H]+ | 249.164 | 59.04859:32759 73.02812:6702 89.05896:12184 208.26324:6382 | 5.45825E-06 |
| POS11847 | Valnemulin | 5.139 | 565.36841 | [2M+H]2+ | 565.36688 | 59.04859:518046 73.02812:56453 87.04335:163613 89.05896:1044131 96.30509:36186 101.05931:63679 103.07444:124619 109.0756:44440 133.08545:456674 147.10149:79674 177.11285:51930 | 2.70621E-06 |
| POS5280 | 3,4,6-Penta-Me-A | 4.464 | 253.16315 | [M+H]+ | 253.16451 | 59.04859:545051 85.06337:9005 87.04335:61951 89.05896:1103382 90.06191:21774 103.03849:19756 103.07444:43522 107.07059:20743 112.07435:6289 133.08545:122674 146.87569:6869 151.09306:11128 177.1078:5932 | -5.372E-06 |
| POS11227 | Thr Ile Pro Ile Thr | 7.036 | 544.33527 | [M+H]+ | 544.33398 | 50.03354:5940 100.52903:6793 284.80084:6514 | 2.36987E-06 |
| NEG744 | Dimethoxypropan- | 9.109 | 119.07005 | [M-H]- | 119.0714 | 59.08403:37295 61.75145:6227 73.11752:6672 97.57933:4913 100.04586:6139 101.04565:74731 102.04721:49298 105.74018:5590 118.07099:59549 119.06866:51500 | -1.1338E-05 |
| POS8909 | isovaleryl-blastmy | 5.754 | 437.19043 | [M+Na]+ | 437.1918 | 59.04858:13381 99.09844:5843 115.8652:5791 173.44014:7355 | -3.1336E-06 |

**Differences in metabolites between the WJW group, the Model group and the Control group**

| Alignment ID | Metabolite name | Rt(min) | Expreiment Mz | Adduct type | Reference m/z | MS/MS spectrum | PPM |
|---|---|---|---|---|---|---|---|
| NEG3376 | Zalcitabine | 4.322 | 210.08705 | [M-H]- | 210.08842 | 66.15359:4819 68.75729:6831 69.07782:4508 74.63445:4580 85.09258:4703 86.00461:4641 93.35798:4824 94.57208:3693 95.05959:4105 95.53884:4481 104.05004:4949 105.25879:5141 106.06308:4313 106.9953:4225 107.84846:5108 110.26845:4463 115.0144:4439 119.9493:4524 122.31573:4279 125.98219:5389 126.38684:4694 126.5578:4420 126.81796:4924 138.25093:4513 141.14272:4189 151.39058:4107 155.47362:3909 167.15324:4146 181.72069:4445 189.89471:4348 198.03937:5441 198.21295:4317 202.14603:4163 207.77614:5262 | -6.5211E-06 |
| POS12436 | -2,3,5,6,7,11,12,15 | 4.934 | 590.40765 | [M+NH4]+ | 590.40601 | 59.04859:838808 87.04335:134216 89.05896:939187 101.05931:114236 103.07444:326499 114.22485:21803 117.05281:51142 117.0909:25332 131.07088:30137 133.08545:324380 147.10149:179621 161.11441:25466 177.11285:34271 191.1256:33417 298.89792:23738 347.28244:25441 393.26031:24812 | 2.77775E-06 |
| POS12415 | dro-1H-cyclopent | 4.749 | 589.38702 | [M+H]+ | 589.38867 | 59.04858:22575 87.04333:30022 89.05894:167008 91.48834:9489 103.07442:10588 133.08542:58874 165.20952:8194 186.57237:8493 | -2.7995E-06 |
| POS15029 | PC(16:0e/12-HETE | 5.901 | 784.5824 | [M+H]+ | 784.58502 | 50.02442:27649 60.08025:49787 86.09541:132816 105.14583:23793 124.99956:27804 173.43526:46783 184.06931:433022 623.297:25599 688.9975:26388 690.32001:28995 | -3.3393E-06 |
| POS11693 | Leu Ser Ala Leu Gl | 5.52 | 560.33051 | [M+2H]2+ | 560.32892 | 53.19652:9330 57.84054:8613 59.04856:55066 60.08023:59508 71.07258:14030 86.09538:143371 87.04332:23037 89.05893:58555 101.05927:12562 103.07441:9952 104.10693:430735 105.11107:11428 124.99951:32392 133.0854:13643 184.0746:298269 325.12164:7536 560.04681:8055 | 2.83762E-06 |
| POS11127 | R,4R)-3,4-dihydro | 5.978 | 540.30292 | [M+Na]+ | 540.30139 | 57.77541:5576 59.04856:15019 60.08023:6218 86.09538:23974 89.05893:6018 96.25027:5780 104.10693:110546 146.98273:12025 | 2.83175E-06 |
| POS5185 | COOH-2But-A7EC | 5.148 | 249.16818 | [M+2H]2+ | 249.1696 | 59.04859:43293 80.82903:5246 89.05896:13447 120.44286:6777 173.39111:7545 | -5.6989E-06 |
| POS5184 | COOH-2But-A7EC | 5.326 | 249.16818 | [M+H]+ | 249.1696 | 59.04859:34217 73.56042:5763 89.05896:14240 102.36123:6026 | -5.6989E-06 |
| POS9048 | Mevinolinic acid | 5.796 | 445.25461 | [M+H]+ | 445.25601 | 59.04858:9104 86.5484:5603 89.05895:5840 149.04791:5048 173.42543:11955 268.03186:5439 326.53644:5422 | -3.1443E-06 |
| POS14930 | PG 36:2 | 8.745 | 775.54608 | [M+2H]2+ | 775.54828 | 68.68565:6044 75.29504:4935 | -2.8367E-06 |
| NEG1524 | ,2,3,4-tetrahydrois | 7.486 | 146.09901 | [M-H]- | 146.0976 | 100.04586:8539 101.05436:159156 102.05606:168511 103.057:27012 144.21524:6429 | 9.65108E-06 |
| NEG5078 | Sempervirine | 0.991 | 271.12555 | [M-H2O-H]- | 271.12411 | 89.13125:22548 90.1367:8192 93.03925:648444 95.03763:1175372 97.03745:192475 145.23549:9570 153.06755:50462 155.06651:25311 | 5.31122E-06 |
| POS6843 | Ficellomycin | 5.412 | 313.19678 | [M+Na]+ | 313.19821 | 57.06989:6684 62.84341:5471 271.15952:10932 313.1973:23428 | -4.5658E-06 |
| POS8109 | -1-(2,4,6-trimeth | 5.259 | 381.12848 | [M+Na]+ | 381.12991 | 59.04858:24008 70.1021:6931 73.06432:6414 73.32253:5950 87.04334:10393 89.05896:18119 101.0593:15519 255.73947:5295 | -3.752E-06 |

**Differences in metabolites between the WJW group, the Model group and the Control group**

| Alignment ID | Metabolite name | Rt(min) | Expreiment Mz | Adduct type | Reference m/z | MS/MS spectrum | PPM |
|---|---|---|---|---|---|---|---|
| POS9766 | Euryspongiol A1 | 5.653 | 483.33017 | [M+2H]2+ | 483.3316 | 57.03287:14812 59.04858:207015 60.05225:6586 62.06014:6340 73.02812:13751 87.04334:13053 89.05896:90974 94.06947:7405 101.0593:21132 103.07444:45253 104.10696:78496 115.07378:7008 133.08545:19303 147.10149:17951 147.60472:5190 173.43034:12231 199.8517:6654 308.36151:5312 | -2.9586E-06 |
| POS9769 | Complanadine A | 6.313 | 483.34955 | [M+H]+ | 483.34811 | 59.04856:76678 95.27219:5765 104.10693:24816 117.09086:9704 161.69566:5716 173.4303:10129 184.0746:7882 | 2.97922E-06 |
| POS12886 | I-3,12-epoxy[1,2]c | 4.61 | 611.35553 | [M+2H]2+ | 611.35376 | 69.03294:60483 73.02811:301618 73.06432:88196 81.03229:85977 83.0477:56965 87.04334:632549 89.05896:3386812 90.0619:114237 91.04457:79164 95.04893:177748 99.04339:106397 111.04189:192093 113.05927:149944 131.06766:63385 133.08543:1148567 134.08684:76949 137.0598:73368 155.07083:93591 164.50946:43316 173.39111:50584 175.10023:43853 177.11285:137209 442.12436:47115 | 2.89521E-06 |
| POS10705 | dimine B\|gymnody | 5.355 | 524.3385 | [M+2H]2+ | 524.33698 | 60.08024:562938 71.07259:89143 72.72482:79104 75.99161:84194 86.0954:1219011 89.05895:152949 100.47708:77300 104.10696:6883640 105.10877:170021 118.89655:80900 124.99954:278323 172.39996:81033 184.06927:2543607 253.02956:86323 360.09375:93576 390.51123:104774 | 2.8989E-06 |
| POS10859 | leopolic acid A | 6.498 | 530.32086 | [M+H]+ | 530.32239 | 59.04858:7175 67.40431:6374 86.38091:6041 183.17155:5455 277.32327:5659 | -2.885E-06 |
| POS8983 | communesin F\|com | 4.842 | 441.26627 | [M+H]+ | 441.2648 | 57.03287:16432 59.04859:379092 73.02812:16020 73.06432:10834 85.06337:7376 87.04335:950837 88.04605:19752 89.05896:261383 97.06461:6118 101.05931:178688 103.03849:176796 103.07444:67373 115.07378:15655 117.05281:107968 131.07088:19277 133.08545:62974 145.08403:6911 147.06319:6605 147.10149:23804 173.38622:6797 | 3.33133E-06 |
| POS13122 | 3-methoxybenzoy | 5.184 | 623.39606 | [M+Na]+ | 623.39423 | 53.5634:6773 57.68123:8495 59.04859:10286 87.04335:9461 89.05896:24055 115.29946:6531 122.35307:7124 418.62692:6913 | 2.93554E-06 |
| POS6626 | )-3,15-Docosadier | 8.426 | 303.30313 | [M+H]+ | 303.3046 | 78.00248:5855 173.43027:11384 | -4.8466E-06 |
| POS13362 | Antioxidant 1098 | 6.565 | 637.49188 | [M+H]+ | 637.49377 | 51.36454:6747 53.10913:5163 53.34431:4667 55.26744:4753 55.4603:4592 57.16814:4529 57.60226:4841 59.38129:5185 59.70594:4757 59.73666:4807 60.54902:4772 61.71716:4371 62.1821:4951 63.79452:4673 67.00437:4054 67.07275:4297 68.12308:4600 72.93169:4974 79.71844:4418 81.57861:4400 93.94611:4260 95.96295:4347 96.63064:5221 99.46171:4827 100.33014:4970 101.19031:4252 107.37337:4320 109.60594:4511 110.68337:3835 111.24065:3892 111.91395:4893 116.4918:4230 119.59566:4429 121.4167:4181 126.0597:4394 127.47894:4810 138.11342:3916 149.29829:4425 154.5083:4636 155.32823:4680 168.06438:3975 171.64886:5563 173.42538:3826 174.58382:5302 186.85178:4110 190.22644:4278 199.62126:4291 204.95943:4169 224.4037:4408 230.90944:5263 276.89719:3772 288.78519:4333 289.20724:5025 311.63177:4714 321.98441:4252 326.27039:5364 337.58038:4794 337.66031:4594 339.78909:4637 342.22339:4357 365.4505:4522 383.93292:4938 418.79239:4649 445.98264:4205 453.12668:4803 552.55151:5532 555.49207:5100 609.55859:5993 | -2.9647E-06 |

**Differences in metabolites between the WJW group, the Model group and the Control group**

| Alignment ID | Metabolite name | Rt(min) | Expreiment Mz | Adduct type | Reference m/z | MS/MS spectrum | PPM |
|---|---|---|---|---|---|---|---|
| POS10837 | etyl-7,8-didehydro | 4.929 | 529.35529 | [M+H]+ | 529.3537 | 57.03287:122787 59.04859:2515289 60.05226:35061 73.02812:49060 73.06432:43538 83.04932:16974 85.06337:48027 87.04335:323380 87.07999:46630 89.05896:1727090 90.06191:39242 99.07939:18467 101.05931:253110 101.09641:16173 103.03849:52425 103.07444:806262 105.09028:15142 115.07378:33323 117.05281:46176 117.0909:45960 129.08911:28993 131.06766:25724 131.10634:22433 133.08545:409248 143.10413:16687 145.08403:30187 147.10149:320582 161.11441:51390 177.1078:18989 191.1256:41225 205.14238:19176 | 3.00366E-06 |
| POS6680 | Benzosimuline | 8.807 | 306.1503 | [M+H]2+ | 306.1488 | 103.46206:5460 218.17601:6104 | 4.89958E-06 |
| POS9447 | ethanolamine lyso | 6.406 | 466.32761 | [M+H]+ | 466.3291 | 51.75655:5860 57.03287:13613 59.04858:45287 | -3.1952E-06 |
| NEG1638 | Ribitol | 1.081 | 151.0685 | [M-H2O-H]- | 151.07001 | 90.1349:28099 93.03928:2218054 108.14954:29810 136.36186:5906 151.20676:17480 | -9.9954E-06 |
| POS10620 | nigramide I | 5.149 | 521.33887 | [M+2H]2+ | 521.33728 | 59.04859:1043720 60.05226:27521 65.04931:28196 73.02812:149483 73.06432:49144 80.0543:55029 85.06506:39103 87.04335:217216 87.06079:100088 87.07999:44362 89.05896:1615024 90.06191:61559 94.06947:20830 101.05931:118585 102.06606:39513 103.07444:265622 104.10697:19272 109.07315:34636 116.08248:18047 117.0909:18739 129.08911:30466 131.07088:34386 133.08545:555306 134.09018:31190 145.08403:23317 147.10149:146270 173.43034:27456 177.11285:53251 184.06931:72285 191.1256:33343 448.9303:17013 | 3.04985E-06 |
| POS8173 | Persicaxanthin | 6.014 | 385.27136 | [M+H]+ | 385.27289 | 67.05389:19140 79.05312:16632 81.06989:7775 91.05389:11767 93.0684:7734 105.06713:5753 180.40804:5330 | -3.9712E-06 |
| POS9848 | Ile Val Leu Glu | 5.51 | 487.31403 | [M+2H]2+ | 487.3125 | 57.03287:11754 59.04859:178044 67.015:12952 73.02812:7761 81.03072:9289 87.04335:14631 87.07999:6587 89.05896:107639 101.05931:16636 103.07444:35327 133.08545:34823 147.10149:13984 221.12637:5452 | 3.13967E-06 |
| NEG6661 | CCG-47657 | 1.066 | 327.14783 | [M-H]- | 327.1463 | 54.53023:5918 93.03925:570753 95.03763:280756 151.06711:26429 153.06754:25377 211.29141:6546 267.29996:5797 291.4368:5469 | 4.67681E-06 |
| POS8932 | hoethanolamine (F | 6.689 | 438.29608 | [M+H]+ | 438.29761 | 52.20243:4490 53.61563:5270 53.66457:4977 60.84661:4453 62.38481:4801 65.33078:4671 65.99124:5305 66.74718:4625 67.60562:4701 70.60014:4795 76.86985:4876 78.83771:5031 82.89027:4843 83.98693:4172 86.43439:4401 87.24602:3800 92.00359:4432 95.965:4148 103.80876:4389 105.82056:4366 112.09728:4502 116.57019:4230 118.66303:4819 120.83271:4774 128.8532:4697 129.61676:4410 132.38916:3731 134.03683:4933 134.87729:3885 137.11153:4585 137.99503:4415 139.85175:4222 141.37001:4694 155.07085:5265 155.99983:4850 156.95407:4549 158.97508:4801 173.45488:5520 174.03526:4117 175.35431:4045 175.96431:4479 198.67374:4813 203.77364:4810 205.12976:5112 222.55981:5364 242.70706:4240 257.70947:3820 271.49545:4172 273.60205:4675 278.77704:4638 293.27414:3851 313.62628:4549 314.39111:7065 352.8526:5080 401.8049:4986 423.26389:4703 | -3.4908E-06 |
| POS1260 | Hydroxyphenyletha | 1.517 | 121.07151 | [M+H]+ | 121.07 | 56.04952:14227 61.03869:6216516 72.08047:22252 | 1.24721E-05 |

**Differences in metabolites between the WJW group, the Model group and the Control group**

| Alignment ID | Metabolite name | Rt(min) | Expreiment Mz | Adduct type | Reference m/z | MS/MS spectrum | PPM |
|---|---|---|---|---|---|---|---|
| POS13319 | 3,36,39-Tridecaoxa | 4.563 | 635.38287 | [M+2H]2+ | 635.38483 | 87.04334:469308 89.05896:3297967 90.06374:82939 117.09089:67942 131.07088:76644 133.08543:1125547 177.11285:102442 216.40135:69223 369.65768:67791 | -3.0847E-06 |
| POS12958 | achidonic acid-bio | 5.328 | 615.42828 | [M+H]+ | 615.43018 | 55.33458:16317 57.03287:42628 59.04859:680263 69.15135:12526 73.02812:17714 73.06432:47268 83.45882:10977 87.04335:62743 89.05896:223262 101.05931:64144 103.07444:201452 117.05281:48062 117.0909:33547 131.06766:22156 133.08545:41216 147.10149:33225 161.1188:18984 580.7569:13279 | -3.0873E-06 |
| POS14295 | -4a-methyl-3,4,4a | 4.861 | 708.47028 | [2M+H]+ | 708.46808 | 59.04956:128458 67.2335:25062 83.81858:19368 87.04335:67545 89.05896:420225 101.05931:36987 103.07444:52472 131.06766:19661 133.08545:208250 147.10149:30929 177.11285:29017 490.83408:20972 | 3.10529E-06 |
| POS8788 | Palau'imide | 5.276 | 429.27655 | [M+H]+ | 429.27509 | 59.04858:78891 62.70562:6008 67.01499:7043 81.03072:6086 87.04334:7795 89.05895:63827 89.54285:5633 101.0593:6206 103.07443:18299 133.08543:19503 133.1481:6079 147.10147:5292 169.53391:6042 296.42734:6526 | 3.40108E-06 |
| POS14893 | oline-3r-carboxyl | 4.581 | 771.29462 | [M+H]+ | 771.297 | 66.49142:46233 223.17427:48411 310.88882:50981 | -3.0857E-06 |
| POS14229 | thyl)oxan-2-yl]oxy | 5.306 | 703.48169 | [M+H]+ | 703.47949 | 57.03287:69866 59.04859:845872 65.52287:39937 68.4929:35657 87.04335:78830 89.05896:398245 98.69503:47696 101.05931:115384 103.07444:239877 117.05281:55079 133.08545:119557 147.10149:122421 184.06931:55139 | 3.12731E-06 |
| NEG296 | heamino)-N-methy | 7.709 | 99.05807 | [M-H]- | 99.0565 | 52.83277:6460 58.19922:5807 | 1.58495E-05 |
| POS9971 | 1-(prop-1-en-2-y | 4.799 | 493.2984 | [M+H]+ | 493.29999 | 51.77015:6467 89.05895:10948 109.86272:5593 | -3.2232E-06 |
| POS8733 | eptapropylene glyc | 5.228 | 425.3092 | [M+H]+ | 425.31079 | 57.03287:102488 59.04858:1181776 60.05225:14621 87.04334:10245 89.05896:20238 103.96567:5605 115.07378:10823 117.09089:124968 173.3862:6020 175.1301:13398 274.86017:6186 | -3.7384E-06 |
| POS9235 | Blazeispirol Z | 4.956 | 455.2811 | [M+H]+ | 455.27951 | 59.04858:74319 73.02812:7384 73.06566:6187 73.29153:6055 87.04334:236414 89.05896:49675 98.93763:4991 101.0593:62978 103.03849:17002 103.07444:13917 115.07378:5845 117.05281:10844 133.08545:14029 161.34744:5438 172.78468:5846 231.72554:5815 345.12427:5506 | 3.49236E-06 |
| POS11082 | Val Leu His Arg | 5.602 | 538.34418 | [M+2H]2+ | 538.34589 | 57.03286:9390 59.04858:114031 67.01499:6481 73.02811:14208 76.64165:6153 87.04333:18041 87.06252:9217 89.05895:88988 101.0593:11741 103.07443:25937 104.10696:45449 115.07377:5394 133.08543:26685 147.09764:9660 184.06927:14473 | -3.1764E-06 |
| POS8892 | Leu Leu Tyr | 6.411 | 436.28207 | [M+H]+ | 436.28049 | 51.60652:6803 160.33104:6165 173.43027:13779 198.89635:5400 | 3.62152E-06 |
| NEG3683 | pyrazin-2-yl)methy | 1.238 | 222.10211 | [M-H]- | 222.1037 | 97.07645:144827 195.65027:6966 | -7.1588E-06 |
| POS13777 | | 5.18 | 667.42255 | [2M+H]+ | 667.42041 | 50.11649:13235 59.04859:12614 89.05896:21060 490.27408:12572 | 3.20637E-06 |

**Differences in metabolites between the WJW group, the Model group and the Control group**

| Alignment ID | Metabolite name | Rt(min) | Expreiment Mz | Adduct type | Reference m/z | MS/MS spectrum | PPM |
|---|---|---|---|---|---|---|---|
| POS8967 | (?)-agelasidine E | 5.267 | 440.29572 | [M+2H]2+ | 440.2941 | 57.03287:114316 59.04859:2189595 60.05226:54308 65.04819:34113 69.06992:23163 73.02812:163661 73.06432:40475 80.0543:20518 85.06337:43061 87.04335:292393 87.06254:57682 87.07999:43790 88.04783:13107 89.05896:1435500 90.06191:45631 94.06947:51671 99.07939:14391 101.05931:236192 102.06384:21944 103.03849:23308 103.07444:457049 104.07732:18845 107.06821:11388 109.0756:26532 115.07378:19339 116.08248:16834 117.05281:13209 117.0909:40443 127.07501:9301 129.08911:31106 131.06766:23576 131.10634:12972 133.08545:406086 134.09018:23270 138.09604:14415 143.10413:10586 145.08403:17173 147.10149:185349 161.11441:13617 177.11285:33361 191.1256:26315 | 3.67936E-06 |
| POS10008 | Norselic acid E | 5.253 | 495.31253 | [M+2H]2+ | 495.31091 | 57.03287:31592 57.06896:126130 58.06512:54802 59.04859:95775 60.08025:892944 67.05389:28807 71.07261:99655 71.08419:76540 81.06834:31475 83.0851:34036 85.10045:65301 86.09541:1777225 89.05896:124465 95.08477:35028 104.10697:9197985 109.10007:26533 124.99956:409778 133.08545:25773 143.24394:21408 163.01524:25461 184.07466:3315038 424.03168:20732 | 3.27067E-06 |
| POS9355 | navirine | 5.671 | 461.31781 | [M+2H]2+ | 461.31619 | 57.03287:59935 59.04859:897027 60.05226:17523 65.04819:8818 69.06868:11381 73.02812:41278 73.06432:16588 87.04335:50952 87.06254:8955 87.07999:14598 89.05896:251265 94.06947:19965 101.05931:62550 103.07444:172294 115.07378:10213 117.0909:25079 129.08911:8562 133.08545:51831 147.10149:63720 161.11441:14604 173.42545:8420 | 3.51169E-06 |
| POS9772 | aroten-4'-al; beta | 5.39 | 483.36047 | [2M+H]+ | 483.36209 | 57.03286:60035 59.04857:639142 60.05224:10273 69.8093:6211 73.0643:7539 87.04333:13400 89.05894:31742 99.07937:5985 101.0571:7897 103.07442:7199 115.07375:8980 117.09087:72617 118.09319:5757 133.0854:7041 175.13007:7639 277.59119:5874 418.71881:5733 | -3.3515E-06 |
| POS10701 | ɔylglycerophosphc | 8.804 | 524.29651 | [M+2H]2+ | 524.29822 | 50.22102:49994 60.08023:159852 69.78801:43132 86.09538:275146 99.47875:45190 104.10693:1657209 105.10875:61902 184.06923:1175168 185.07629:113468 343.16348:49365 428.68253:52781 | -3.2615E-06 |
| POS8170 | MLS001074091-01 | 4.518 | 385.24164 | [M+H]+ | 385.23999 | 59.04859:198724 73.06432:23292 85.06506:9454 87.04335:135996 89.05896:1684771 90.06191:27626 101.05931:10071 103.07444:42261 107.07059:17644 118.46617:7133 129.08911:8882 131.06766:22653 133.08545:532233 134.09018:12455 147.10149:19174 177.11285:33121 | 4.28304E-06 |
| POS6992 | 3-Hexadecylphenc | 7.728 | 319.29785 | [M+H]+ | 319.2995 | 55.44436:6228 152.06296:5795 | -5.1676E-06 |
| POS5965 | 2-Heptanol glucos | 4.96 | 279.17847 | [M+H]+ | 279.18011 | 57.03287:7884 59.04859:34718 60.22052:7271 87.04335:20299 89.05896:26744 101.05931:55446 103.07444:8419 133.08545:7181 149.02057:9892 | -5.8743E-06 |
| NEG9735 | PFSM-amine | 5.075 | 585.06122 | [M-H]- | 585.05927 | 89.13124:1344418 90.13486:49481 91.69405:5157 173.586:6204 236.26202:6039 238.19133:5688 | 3.333E-06 |
| NEG2818 | Cyprotene | 5.414 | 191.17894 | [M-H]- | 191.1806 | 59.02949:33858 87.11256:5863 103.07276:50878 111.14096:13856 134.77959:5929 173.61551:12363 | -8.6829E-06 |

**Differences in metabolites between the WJW group, the Model group and the Control group**

| Alignment ID | Metabolite name | Rt(min) | Expreiment Mz | Adduct type | Reference m/z | MS/MS spectrum | PPM |
|---|---|---|---|---|---|---|---|
| POS9238 | Palmonine A | 5.153 | 455.30197 | [M+2H]2+ | 455.30029 | 57.03287:68998 59.04859:1527596 60.05226:45674 65.04819:25475 69.06868:17124 73.02812:146998 73.06432:42980 80.0543:33719 85.06337:37843 87.04335:245819 87.06079:71965 87.07999:33428 88.04783:10096 89.05896:1523596 90.06191:55922 94.06947:27000 101.05931:149161 102.06606:22085 103.03849:15588 103.07444:321698 104.07732:17927 107.06821:15137 109.07315:36192 115.07378:17028 116.08248:17508 117.05281:25268 117.0909:22044 127.07501:9960 129.08911:27742 131.06766:29751 131.10634:16879 133.08545:448558 134.08684:21470 138.09604:13135 145.08403:11129 147.10149:139436 177.11285:40433 191.1256:18424 | 3.68987E-06 |
| POS10068 | Tumonoic Acid I | 5.466 | 498.34079 | [M+2H]2+ | 498.34247 | 57.03286:38459 57.06988:22198 59.04858:684673 60.08024:89957 61.08377:15770 65.0493:16494 69.0699:12064 71.07259:12954 71.7504:10678 73.02811:62351 73.06431:11042 75.86941:10828 85.06335:15657 86.09539:198595 87.04333:149263 87.06252:30276 87.07997:13244 87.09917:39303 89.05894:384471 90.0619:13226 94.06945:16235 101.05929:79931 103.07442:110724 104.10695:892489 105.10877:292476 106.11103:40248 111.16512:11085 124.99953:42653 133.08542:94051 147.10147:40128 184.07463:249382 185.07632:98694 186.07532:41872 | -3.3712E-06 |
| POS13361 | milicifoline C | 5.521 | 637.42297 | [M+2H]2+ | 637.42511 | 57.03287:62586 59.04859:825992 73.02812:69609 87.04335:158241 87.06079:40926 89.05896:817897 101.05931:144391 103.07444:246986 122.71721:27694 133.08545:284362 147.10149:133885 177.11285:41117 298.98672:31467 | -3.3573E-06 |
| POS9308 | SCHEMBL1271357 | 5.06 | 459.28525 | [M+2H]2+ | 459.2869 | 59.04858:34977 59.05443:7569 67.01499:6994 87.04334:11196 89.05895:55091 95.59659:5571 103.07443:11088 133.08543:19402 147.10147:7609 173.3911:11204 270.74762:5613 | -3.5925E-06 |
| NEG5611 | ethyl-4-n-pentade | 1.872 | 290.28708 | [M-H]- | 290.2854 | 50.17372:5483 74.03466:7673 90.04866:10248 99.07362:6553 101.05444:6378 104.0432:7910 105.05279:24208 106.2744:5517 114.11422:8284 128.07486:6858 128.10909:6768 130.12769:71969 146.14146:21199 156.13734:53912 157.14728:6726 162.1563:5780 166.15576:7130 174.17671:6195 200.18018:12158 | 5.78741E-06 |
| POS6825 | SCHEMBL1021325 | 8.576 | 312.32361 | [M+H]+ | 312.32529 | 55.52958:5945 59.48562:5526 130.17635:5828 236.2997:5516 | -5.379E-06 |
| POS10621 | nigramide I | 6.135 | 521.33905 | [M+H]+ | 521.33728 | 56.04953:14607 60.08025:237305 61.08378:19303 67.05389:10716 71.0726:40524 86.09541:677397 87.09918:72619 98.98415:28602 104.10696:130559 105.1111:17278 124.99955:215033 126.00201:11723 173.43034:12835 184.07466:2453926 185.07635:387950 186.07533:29304 | 3.39511E-06 |
| POS14227 | ulomo'opunalide- | 5.483 | 703.46161 | [M+2H]2+ | 703.46399 | 59.04859:390085 73.02946:39071 87.04335:113928 89.05896:523676 101.05931:107492 103.07444:96810 104.81318:28701 116.56748:27100 133.08545:200510 147.10149:61869 177.11285:34186 184.06931:41318 211.92552:23765 328.8551:31348 | -3.3833E-06 |
| POS3138 | se, 6-deoxy-3-O- | 0.947 | 179.0896 | [M+H-H2O]+ | 179.09129 | 64.6556:6685 69.68045:5681 79.08632:5474 | -9.4365E-06 |

**Differences in metabolites between the WJW group, the Model group and the Control group**

| Alignment ID | Metabolite name | Rt(min) | Expreiment Mz | Adduct type | Reference m/z | MS/MS spectrum | PPM |
|---|---|---|---|---|---|---|---|
| POS9788 | Poricoic acid B | 5.253 | 484.32059 | [M+2H]2+ | 484.31888 | 57.03287:68054 58.04042:17563 59.04859:1518611 59.05931:38277 60.05226:44010 65.04819:26666 67.05389:10380 69.06992:24349 73.02812:140028 73.06432:39950 80.0543:31676 85.06337:40551 87.04335:266839 87.06079:73682 87.07999:38629 89.05896:1348141 90.06191:47112 94.06947:36153 99.07939:9958 101.05931:204703 102.06384:22097 103.03849:13150 103.07444:343966 104.07732:14918 107.07059:14090 109.0756:29523 115.07378:19859 116.08248:13343 117.05281:17225 117.0909:23356 129.08911:28205 131.06766:22913 131.10634:13401 133.08545:420011 134.08684:29316 143.10413:9866 145.08403:14646 147.10149:155668 148.10667:10781 161.11441:15132 177.11285:33982 191.1256:14469 | 3.53073E-06 |
| POS10282 | ethyl-2H-chromer | 4.92 | 507.31213 | [M+Na]+ | 507.31039 | 89.05896:28389 133.08545:8527 328.20291:8787 | 3.42985E-06 |
| POS10506 | abloxime / IC202C | 5.249 | 517.32721 | [M+2H]2+ | 517.32898 | 57.03287:9752 59.04859:166769 67.015:27063 73.02812:11806 73.06432:9301 81.03072:6908 86.09541:9540 87.04335:20078 87.07999:7269 89.05896:186589 90.06374:9987 101.05931:11965 103.07444:48519 104.10697:66890 117.93352:5956 133.08545:62091 147.10149:28620 151.08508:9196 177.11285:5900 | -3.4214E-06 |
| POS7642 | syl-(1->4)-2,6-dic | 4.817 | 353.21518 | [M+Na]+ | 353.21689 | 57.03287:14908 59.04859:426669 60.05226:7114 71.04816:6970 73.02812:14269 73.06432:9159 87.04335:1004828 88.04605:21520 89.05896:117182 94.90382:7466 101.05931:210115 103.03849:70058 103.07444:29014 117.05281:109733 131.07088:17596 133.08545:19675 | -4.8412E-06 |
| POS7524 | oscr#20 | 5.046 | 347.2446 | [M+H]+ | 347.24289 | 55.05364:6974 67.05388:22365 69.0699:10099 79.05311:9369 81.06831:25682 83.08508:9356 93.06838:14687 95.08475:23665 105.06943:9346 107.08484:10309 109.10004:18336 121.10127:13071 293.7276:5209 | 4.92451E-06 |
| POS11799 | DTXSID10275904 | 5.859 | 563.38623 | [M+2H]2+ | 563.38831 | 57.03287:26132 59.04859:208545 73.02812:12773 73.06432:14312 87.04335:28288 87.07824:12119 89.05896:134492 101.05931:35914 103.07445:56449 104.10697:22666 112.35517:11952 117.05281:12725 117.0909:14243 133.08545:35059 173.43036:22893 184.81708:13305 244.06889:10416 | -3.6919E-06 |
| POS10281 | -8-methoxy-10,12 | 4.779 | 507.30383 | [M+H]+ | 507.3056 | 59.04857:10016 73.0281:6517 89.05894:28411 133.0854:6429 143.883:5278 177.48293:7160 | -3.489E-06 |
| NEG6279 | Arachidic acid | 4.943 | 312.30453 | [M-H]- | 312.3028 | 80.05276:7612 84.76994:6113 97.07439:6550 100.04586:23098 101.05437:45655 167.23665:6156 180.09624:28321 | 5.5395E-06 |
| POS8429 | Enactin Ib | 5.415 | 403.27847 | [M+2H]2+ | 403.28021 | 57.03287:82442 59.04859:1698368 60.05226:36274 61.02743:9456 65.04819:10553 69.06868:10839 73.02812:79429 73.06432:24878 85.06337:21941 87.04335:72156 87.06254:16414 87.07999:20441 89.05896:561635 90.06191:17750 94.06947:39409 99.07939:9733 101.05931:95659 103.07444:279788 104.07732:12398 115.07378:14869 117.0909:23914 131.07088:12124 133.08545:117425 147.10149:89823 161.11441:14249 177.1078:7988 | -4.3146E-06 |

**Differences in metabolites between the WJW group, the Model group and the Control group**

| Alignment ID | Metabolite name | Rt(min) | Expreiment Mz | Adduct type | Reference m/z | MS/MS spectrum | PPM |
|---|---|---|---|---|---|---|---|
| POS8441 | an-2-yl]propanoyl | 5.07 | 404.26877 | [M+2H]2+ | 404.267 | 57.03287:58837 58.04137:29486 59.04859:1616627 60.05226:35988 65.04819:36403 69.06992:16574 73.02812:189531 73.06432:37543 80.0543:43942 85.06337:37458 87.04335:213136 87.06079:98341 87.07999:25048 87.56366:9658 89.05896:1617776 90.06191:48771 94.06947:38166 99.07939:10110 101.05931:120398 102.06606:31638 103.03849:13374 103.07444:284408 107.07059:17747 109.07315:50609 116.08248:18777 117.0909:19918 129.08911:33830 131.06766:24497 131.08699:18428 133.08545:484706 134.09018:23158 140.09358:10265 147.10149:112636 177.11285:42992 191.1256:16952 | 4.37829E-06 |
| POS4702 | hylideneamino)-5 | 5.789 | 232.11319 | [M+2H]2+ | 232.11142 | 57.03287:26397 59.04858:8583 73.45755:5578 104.44542:5183 | 7.62565E-06 |
| POS10501 | (2S,3R,4R,5R,6S)-3 | 6.171 | 517.01233 | [M+H]+ | 517.01416 | 57.88688:5908 66.74015:5527 173.43526:8482 259.29834:5302 323.72876:5833 345.17938:6032 | -3.5396E-06 |
| POS6132 | Boldione | 5.072 | 285.18777 | [M+H]+ | 285.186 | 55.05453:6606 55.1802:5646 64.45844:6128 67.05389:7664 81.06833:10869 104.02033:5184 106.8283:5606 144.31024:5418 | 6.20648E-06 |
| POS5070 | 10,11,12,14,15,16,1 | 6.775 | 245.22453 | [M+H]+ | 245.2263 | 107.41644:6377 | -7.2178E-06 |
| NEG1727 | -2-Hexenyl propa | 1.257 | 155.10521 | [M-H]- | 155.10699 | 57.10276:10240 59.08403:14502 69.11722:22841 79.05367:640348 80.05276:9872 81.14304:13137 83.15008:8291 93.15687:46848 95.03763:308253 96.13688:8700 97.03745:157327 97.08466:514540 97.10725:17037 109.17062:13483 111.1535:9824 111.17866:27566 119.17749:7602 137.08243:25627 147.46233:5920 154.24498:8477 | -1.1476E-05 |
| POS10668 | Methylepiamauron | 4.723 | 523.30859 | [M+H]+ | 523.3067 | 60.08024:39206 86.09539:83178 89.05894:24975 104.10695:432274 124.99953:18719 131.77299:5289 184.06927:171092 | 3.61165E-06 |
| NEG8043 | Arg Pro Asp | 1.051 | 385.1824 | [M-H2O-H]- | 385.1842 | 59.79719:5582 93.03928:417381 94.34543:5411 95.03767:189130 97.03749:6539 151.06717:41243 153.0676:34035 211.09386:6038 249.88901:7290 296.67526:5707 374.81418:5789 | -4.6731E-06 |
| POS14140 | odermin glycolipi | 5.389 | 696.45532 | [M+2H]2+ | 696.45282 | 59.04859:140904 68.52091:34557 87.04335:55010 89.05896:318027 101.05931:43050 103.07444:68044 133.08545:143489 242.30151:31183 382.90112:33882 512.59747:36556 623.69818:38147 | 3.58962E-06 |
| POS13845 | inaginsenoside R1 | 5.733 | 673.44965 | [M+2H]2+ | 673.45209 | 57.03287:85024 59.04859:784371 60.05126:27631 64.73385:24077 69.06868:27228 73.02812:76311 73.06432:39863 80.05276:20891 82.95837:15141 85.06337:45652 87.04335:179937 87.06079:38342 87.07999:55866 89.05896:756533 90.06374:30229 94.06947:43568 99.07939:21152 101.05931:189201 103.07444:251639 115.07378:31749 117.05281:32922 117.08817:36493 129.08911:41739 131.07088:26483 131.10634:31873 133.08545:338218 134.09018:24566 147.10149:190411 161.11441:20361 177.1078:45478 181.53217:18532 191.1256:51214 205.1487:16869 211.9454:17643 226.3:16723 340.80035:16961 380.6423:17731 397.44708:19957 | -3.6231E-06 |
| NEG948 | yl-2,3-dimethylpe | 1.102 | 127.15112 | [M-H]- | 127.1493 | 82.05115:58658 83.85929:5676 89.13124:245074 90.13486:13532 97.09287:11531 99.88496:5881 126.15527:9329 128.18996:18336 | 1.43139E-05 |

**Differences in metabolites between the WJW group, the Model group and the Control group**

| Alignment ID | Metabolite name | Rt(min) | Expreiment Mz | Adduct type | Reference m/z | MS/MS spectrum | PPM |
|---|---|---|---|---|---|---|---|
| POS12302 | Shearinine F | 5.495 | 584.33398 | [M+NH4]+ | 584.33612 | 59.04858:61152 60.08025:92369 62.04125:18600 74.02055:19986 86.09541:196362 89.05896:85023 103.07444:23999 104.10696:871256 124.99955:34437 173.43034:42850 184.07466:412600 | -3.6623E-06 |
| POS10435 | Maraviroc | 5.065 | 514.33313 | [M+2H]2+ | 514.33502 | 58.04137:18704 59.04859:457688 60.05226:19809 69.06992:13175 73.02812:109044 73.06432:33504 80.0543:49272 85.06506:17945 87.04335:222579 87.06254:43340 87.07999:14094 88.04605:17976 89.05896:978529 90.06191:29299 94.06947:10053 101.05931:128383 102.06606:39990 103.07444:120581 107.06821:9358 109.0756:27888 116.08248:11130 117.05281:18551 117.0909:14302 124.07726:18119 129.08911:17802 131.06766:31383 133.08545:392702 134.08684:18607 140.09358:9025 147.10149:54001 153.09918:9300 177.11285:40338 191.1256:12368 | -3.6746E-06 |
| POS13977 | Rubescensin M | 4.947 | 683.41779 | [M+H]+ | 683.41528 | 71.70737:10859 86.33093:11542 88.84815:11253 105.48704:12829 150.76653:13188 173.39111:27333 | 3.67273E-06 |
| POS6704 | Ruscopine | 5.437 | 307.21088 | [M+NH4]+ | 307.21271 | 57.03286:9458 59.04858:17416 | -5.9568E-06 |
| POS6451 | dodeca-2,6,10-tri | 4.487 | 297.19003 | [M+H]+ | 297.1882 | 59.04859:673313 73.06432:21185 85.06337:18211 87.04335:153095 89.05896:2518169 90.06191:56779 103.03849:14835 103.07444:72495 107.06821:28876 131.06766:10369 133.08545:485030 134.08684:16965 147.10149:19674 151.09705:15676 177.11285:10663 | 6.15771E-06 |
| POS7026 | e-(3Z,6Z,9Z)-3,6,9 | 8.429 | 321.31326 | [M+H]+ | 321.31509 | 70.24474:7366 76.22115:6057 158.60115:5338 319.11258:6577 | -5.6953E-06 |
| POS13677 | Eupassofilin | 4.946 | 661.43335 | [M+H]+ | 661.43091 | 57.03287:41420 59.04859:805266 73.02812:28269 73.06432:22540 85.06337:22492 87.04335:219622 87.07999:37638 89.05896:1216031 90.06375:24636 101.05931:162048 103.07444:273206 117.05281:18477 129.08911:26497 131.10634:29001 133.08545:406824 134.09018:20388 147.10149:153326 161.1188:17418 177.11285:22391 191.1256:28133 271.96683:16540 | 3.68897E-06 |
| POS10414 | rynamine A\|Pseud | 5.1 | 513.32422 | [M+Na]+ | 513.32233 | 57.03287:25815 59.04859:295939 73.02812:54974 73.06432:16633 80.05276:16281 85.06337:12561 87.04335:987143 87.06254:29520 88.04605:16722 89.05896:348376 101.05931:308362 102.06606:14154 103.03849:24348 103.07444:75383 115.07643:9455 117.05281:44277 117.0909:8690 130.0807:9060 131.07088:24750 131.10634:7846 133.08545:113121 147.10149:23943 177.1078:11328 406.83331:7597 | 3.6819E-06 |
| POS7608 | 2-(3-furanyl)ethyl] | 5.516 | 352.25009 | [M+H]+ | 352.24823 | 57.06894:50991 71.08544:19760 83.08505:5694 84.07954:64985 85.10041:5780 86.09537:12881 88.0744:5945 166.05182:15901 184.06384:35843 | 5.28037E-06 |
| POS6075 | CHEBI:70570 | 4.972 | 283.17126 | [M+H]+ | 283.1694 | 55.05365:31218 57.06896:33562 67.05389:13975 69.06868:42773 71.08547:9772 79.05463:10750 81.06833:18709 83.08509:22840 89.05896:7788 93.06841:26898 95.08477:16820 97.06461:7051 97.09953:16189 100.07597:6934 107.08486:13321 109.10006:7549 111.0796:6733 121.1013:12432 135.11307:6930 163.14494:5752 173.43034:16278 | 6.56851E-06 |
| NEG1977 | ylsulfanyl-propyl)- | 0.192 | 163.03513 | [M-H]- | 163.037 | 59.08501:6264 101.95877:5287 102.12029:6051 112.62181:5924 | -1.147E-05 |

**Differences in metabolites between the WJW group, the Model group and the Control group**

| Alignment ID | Metabolite name | Rt(min) | Expreiment Mz | Adduct type | Reference m/z | MS/MS spectrum | PPM |
|---|---|---|---|---|---|---|---|
| NEG298 | neamino)-N-methy | 7.485 | 99.05838 | [M-H2O-H]- | 99.0565 | 50.45867:5282 50.63686:4731 52.5896:5478 54.41459:5216 54.5553:4341 55.59398:5477 55.64116:4879 57.96262:4576 60.51724:5215 62.46269:4877 63.83049:4119 66.61794:5225 67.90253:3883 70.18143:4460 70.47387:6502 72.58765:4299 72.85787:4634 75.44221:4565 75.67907:4878 77.01496:4716 81.56989:4984 87.99705:3960 89.88375:5045 94.98595:4363 96.0257:5005 96.65898:4448 | 1.89791E-05 |
| POS9605 | Progeldanamycin | 4.507 | 476.30289 | [M+NH4]+ | 476.30099 | 73.06432:18860 87.04334:121661 89.05896:1662491 90.0619:35266 107.07059:10722 112.07434:13270 117.08817:17685 131.06766:26392 133.08543:736677 134.09018:30300 173.39111:23393 177.11285:97579 | 3.98907E-06 |
| POS11816 | anoyl]-hydroxyami | 5.455 | 564.37366 | [M+2H]2+ | 564.37152 | 57.03379:24448 59.04956:417829 60.05325:13891 73.02812:45138 73.06566:15355 85.06506:15845 87.04334:70351 87.06253:15152 87.07999:14180 89.05896:356656 90.06374:14936 99.72215:8955 101.0593:66806 103.07444:125605 109.07559:10320 123.51226:8200 129.09225:11722 130.08708:11693 133.08545:136882 147.10149:68488 177.11285:14320 191.13127:15101 551.74365:8931 | 3.79183E-06 |
| POS13171 | Enniatin-B2 | 5.54 | 626.39862 | [M+2H]2+ | 626.401 | 57.03287:25847 59.04859:309793 67.015:22678 69.06992:13608 73.02812:35115 73.06432:25919 85.06337:17718 87.04335:68475 89.05896:400755 90.06191:14581 99.92779:12315 101.05931:62306 103.07444:92552 109.0756:12974 117.08817:21424 131.06766:20017 133.08545:188559 147.10149:65896 173.39111:28819 177.11285:17788 225.6507:12906 | -3.7995E-06 |
| NEG2933 | noethyl methyl ph | 1.223 | 196.04738 | [M-H]- | 196.0493 | 56.43259:5803 59.08403:5648 79.05368:6140 81.55566:6182 97.07645:1401613 98.0778:45117 99.07355:179528 117.06951:30539 124.15677:12949 153.07974:7956 159.03728:9905 161.03622:529928 162.03658:8897 196.04443:52826 197.04991:6995 | -9.7935E-06 |
| POS7380 | cyclohenicosane-2 | 4.542 | 340.25748 | [M+H]+ | 340.2594 | 59.04856:31007 69.06866:84019 71.08545:8408 73.06429:9314 79.0531:16901 79.1861:6788 87.04331:11348 89.05893:184400 96.08015:372017 97.06458:18211 97.08306:9037 113.10571:162547 114.09121:358557 115.09229:11966 133.08539:41046 173.43028:8567 174.75732:5879 195.7886:6075 209.16328:129619 210.16083:8006 226.19028:7461 227.17245:35459 321.50101:7261 322.24512:7990 | -5.6428E-06 |
| POS14697 | zamamidine A | 4.955 | 749.48724 | [M+CH3OH+H]+ | 749.49011 | 59.04859:193782 87.04335:65241 89.05896:529070 101.05713:36432 103.07444:51241 109.67995:36455 133.08545:207760 147.09766:35842 404.62183:33904 474.819:35804 | -3.8293E-06 |
| NEG3341 | pentadec-2-ene | 1.342 | 209.22568 | [M-H]- | 209.2276 | 59.08403:13174 79.05367:23100 80.04047:5185 89.13125:25835 93.50523:6111 97.07645:787088 119.08539:8472 128.02501:9076 | -9.1766E-06 |
| POS1263 | Dimethoxypropan- | 3.17 | 121.08389 | [M+H]+ | 121.0858 | 56.96447:10960 61.03869:542954 77.03802:17207 84.959:22201 91.05389:21343 93.03563:11806 93.06841:25035 95.04893:44023 103.05421:116503 104.05679:19980 120.0803:84862 121.08412:12230 | -1.5774E-05 |

**Differences in metabolites between the WJW group, the Model group and the Control group**

| Alignment ID | Metabolite name | Rt(min) | Expreiment Mz | Adduct type | Reference m/z | MS/MS spectrum | PPM |
|---|---|---|---|---|---|---|---|
| POS2235 | Aminobenzothiazo | 0.763 | 151.03493 | [M+H]+ | 151.033 | 58.99696:9747 67.05389:7667 68.98123:290371 72.04237:6093 81.06989:9024 82.01395:151616 86.99278:262824 87.99461:6195 91.05389:5746 95.08476:6405 95.43819:6763 96.04378:6050 100.02438:330463 101.02657:9117 105.0024:291039 105.06944:9573 110.00877:47923 110.03355:8945 118.03534:92609 123.0126:44635 123.03898:8102 128.01854:202653 129.01982:7852 132.99644:9000 146.02687:21046 151.00931:6500 151.06114:22776 | 1.27787E-05 |
| POS11894 | Thr Tyr Arg Lys | 4.592 | 567.32703 | [M+2H]2+ | 567.32483 | 69.03417:53726 73.02811:220069 73.06432:42869 81.03385:46702 83.0477:46845 87.04334:445389 89.05896:2303357 90.06374:59990 95.04893:148172 99.04339:77710 111.04189:168038 113.05927:63343 131.06766:65612 133.08543:796132 134.09018:48601 137.0598:39987 155.06668:80457 173.39111:53708 177.11285:72286 294.94229:40929 562.07343:45543 | 3.87785E-06 |
| POS11924 | His Gly Lys Lys Val | 5.341 | 568.3587 | [M+2H]2+ | 568.35651 | 57.03379:15383 59.04859:190882 67.015:20860 73.02812:32846 73.06432:15990 81.03072:14575 85.06337:15280 87.04335:34936 89.05896:244115 90.06374:13454 97.61116:9353 101.05931:32276 103.07444:65960 133.08545:91691 147.10149:32142 157.22046:9896 158.09198:12284 177.11285:14461 409.98849:9933 | 3.85322E-06 |
| POS10589 | Fenestin B | 5.471 | 520.34729 | [M+2H]2+ | 520.3493 | 57.03284:30269 59.04856:486377 60.05223:17744 65.04928:8935 73.02808:33974 73.06429:9876 81.70692:6584 85.06333:12744 86.09536:9245 87.0433:50634 87.06075:10561 87.07994:7390 89.05891:288461 90.06187:7405 96.95786:7399 101.05926:37328 103.07439:85359 104.10692:41217 117.09084:11496 133.08537:66665 147.10141:31501 168.12987:6619 184.07457:22551 240.41701:6519 | -3.8628E-06 |
| POS5068 | 10,11,12,14,15,16,1 | 9.918 | 245.22437 | [M+H-H2O]+ | 245.2263 | 109.76384:5522 194.7397:5349 | -7.8703E-06 |
| POS9517 | Terfenadine | 4.807 | 471.31564 | [M+H]+ | 471.31369 | 57.03287:65988 59.04859:1819779 60.05226:33917 61.02846:12331 69.06992:14219 73.02812:38060 73.06432:24521 83.04932:12352 85.06337:36044 87.04335:223799 87.07999:33804 89.05896:1708307 90.06191:29641 99.07939:9761 101.05931:180879 103.03849:32028 103.07444:524725 104.07732:13567 105.09028:11927 107.07059:14424 115.07378:10759 117.05281:32226 117.0909:27327 129.08911:23706 131.07088:27981 131.10634:12667 133.08545:416039 134.08684:13790 145.08403:18900 147.10149:247631 161.11441:20221 177.11285:18828 191.1256:24494 | 4.13737E-06 |
| POS11351 | asterone C 22-nic | 4.537 | 548.33484 | [M+H]+ | 548.33698 | 73.06431:42322 87.04333:107792 87.3213:27235 89.05895:1227742 90.0619:142684 117.09088:27070 131.07086:28767 133.08543:418955 134.09016:65235 177.11284:46088 | -3.9027E-06 |
| POS10869 | Lys Lys Leu Ala Gly | 5.359 | 530.3681 | [M+2H]2+ | 530.36603 | 59.04858:93051 69.77426:6928 73.02812:7024 73.06432:11558 85.06337:6334 87.04334:36853 87.07998:5721 89.05896:121367 101.0593:25695 103.07444:31208 117.0909:7133 133.08545:41338 147.10149:13551 364.68661:6553 | 3.90296E-06 |

**Differences in metabolites between the WJW group, the Model group and the Control group**

| Alignment ID | Metabolite name | Rt(min) | Expreiment Mz | Adduct type | Reference m/z | MS/MS spectrum | PPM |
|---|---|---|---|---|---|---|---|
| NEG3004 | N-Glyceryltaurine | 1.205 | 198.04619 | [M-H]- | 198.0442 | 79.05367:22192 81.06151:5960 161.03622:312986 163.03734:726720 164.03838:13700 198.04518:121900 | 1.00483E-05 |
| NEG242 | mino-2H-pyrrol-2 | 0.962 | 97.03882 | [M-H2O-H]- | 97.0408 | 50.8479:6218 79.05367:208009 80.05276:81134 81.286:4924 88.2065:5166 97.03745:211881 97.07645:435261 | -2.0404E-05 |
| POS6589 | CHEMBL2229478 | 5.069 | 302.21338 | [M+H]+ | 302.2114 | 55.05365:129180 57.03287:9962 57.06896:49742 59.04858:7324 61.01003:8355 67.05389:185932 69.06868:156592 71.04816:31271 71.08547:23748 79.05312:30386 81.06833:309800 82.07301:7658 83.0477:20895 83.08509:116967 85.06337:28484 85.10045:13174 91.0539:49071 93.06841:48134 95.08477:318163 96.08827:11507 97.06461:12086 97.09953:65192 99.07939:5623 105.06945:32571 107.08486:75967 109.06336:16497 109.10006:243459 110.103:11340 111.0796:8286 111.11481:15745 114.59279:5540 119.08333:76117 121.1013:35163 123.08004:23880 123.11523:69731 125.09565:11049 128.06834:25619 129.07336:16376 133.09863:98182 134.10684:6890 135.11646:15927 137.09427:30717 142.08385:6653 144.09827:8149 147.11681:47253 148.11829:5573 149.13002:19925 151.10901:10614 161.13197:18900 163.14494:33349 165.16396:237099 166.16684:15773 167.3136:6215 170.48605:6220 173.39111:6772 178.52345:5523 189.16042:86633 190.16454:6522 207.17024:25460 | 6.55171E-06 |
| POS9812 | Tobramycin | 4.847 | 485.29492 | [M+Na]+ | 485.29294 | 57.03287:15398 59.04859:273494 73.02812:14205 73.06432:10434 76.4834:5527 87.04335:649412 88.04605:9919 89.05896:235728 101.05931:130095 103.03849:171420 103.07444:66416 110.9791:5381 115.07378:9879 117.05281:83480 131.06766:16572 133.08545:75681 147.06319:8732 147.10149:25839 173.43526:10832 196.16579:6213 202.72192:6580 | 4.08001E-06 |
| POS12173 | NBD-dihydro-Cera | 5.691 | 578.39343 | [M+2H]2+ | 578.39111 | 55.05366:14753 57.03287:53323 59.04859:671995 60.05226:17840 69.06992:10379 73.02812:68294 73.06432:42721 80.0543:8628 81.0699:9920 85.06337:35636 87.04335:91351 87.06079:22020 87.07999:30506 89.05896:411190 90.06375:17210 94.06947:16227 96.50431:9577 99.07939:15179 101.05931:104704 103.07445:194301 115.07378:22064 117.05281:52219 117.0909:29782 119.23139:8095 129.08911:19768 131.06766:41126 131.10634:13377 133.08545:172080 143.10413:9771 147.10149:98957 161.11441:21054 177.11287:21305 191.1256:12890 205.14238:11902 429.96268:8644 | 4.01113E-06 |

**Differences in metabolites between the WJW group, the Model group and the Control group**

| Alignment ID | Metabolite name | Rt(min) | Expreiment Mz | Adduct type | Reference m/z | MS/MS spectrum | PPM |
|---|---|---|---|---|---|---|---|
| POS9714 | Pharboside B | 4.645 | 481.28281 | [M+NH4]+ | 481.28079 | 58.04041:23872 59.04858:35887 69.03294:54111 73.02811:189832 73.06432:62878 80.05429:73051 81.03229:41889 83.04769:28918 87.04334:390027 88.04782:21281 89.05895:2047538 90.0619:89012 91.04643:68448 91.07442:10828 95.04893:82738 99.04339:69170 101.0593:11472 102.06605:41337 107.0682:24784 109.06335:12856 111.0444:82514 113.05927:81471 117.09089:25129 124.08022:17131 125.05959:13639 131.07086:51757 133.08543:782893 134.09016:43064 137.0598:43303 151.09703:24787 155.06668:39977 157.08505:17409 173.39601:11618 177.11284:87990 | 4.19713E-06 |
| POS10293 | Val Phe Lys Asp | 6.067 | 508.27853 | [M+H-H2O]+ | 508.27649 | 60.08022:57792 69.06989:10630 86.09537:75225 104.10693:815147 105.10874:13218 124.9995:27646 158.71704:6638 184.07458:32309 335.11627:9683 | 4.01356E-06 |
| POS7450 | Nostocyclyne A | 7.302 | 343.26077 | [M+H]+ | 343.26279 | 55.31514:6243 56.21209:5842 70.43735:6475 92.06237:5683 124.23769:5623 | -5.8847E-06 |
| POS13266 | ethyl-5-methylide | 5.051 | 631.42297 | [2M+H]+ | 631.42041 | 57.03287:150773 59.04859:2764982 69.06869:38455 73.02812:91875 73.06432:60500 85.06337:66679 85.2186:31195 87.04335:632992 87.07999:84893 89.05896:2537144 90.06191:53195 99.08151:36128 101.05931:507606 103.03849:76130 103.07444:947555 105.09028:34377 107.07059:32935 115.07378:40858 117.05281:76758 117.0909:69998 129.08911:67531 131.06766:89552 133.08545:787532 145.08403:54317 147.10149:647766 161.11441:72138 177.1078:56457 191.1256:119385 264.8284:34663 | 4.05435E-06 |
| POS4831 | ene-1,3-disulfona | 1.009 | 236.99767 | [M+Na]+ | 236.99969 | 51.28637:5659 62.98058:33092 69.63676:6189 103.01153:22380 132.98657:34966 143.15192:5232 168.37367:5230 169.98517:21779 | -8.5232E-06 |
| POS11985 | Cymarine | 4.436 | 571.28479 | [M+Na]+ | 571.28717 | 67.41739:6578 132.3401:5945 146.061:6037 188.07004:25305 205.09822:8072 231.13576:6047 | -4.166E-06 |
| POS6528 | xy-C12 homoserin | 5.084 | 300.21484 | [M+NH4]+ | 300.21689 | 55.53489:6784 57.06894:103329 61.96259:6471 70.06429:18750 71.08545:62549 82.065:18059 95.22827:5435 100.07379:159104 128.0683:699485 129.07332:21185 142.0838:174223 188.09213:79339 254.20921:7240 275.32065:6325 | -6.8284E-06 |
| POS9670 | -8,10-dioxapenta | 4.554 | 479.27475 | [M+2H]2+ | 479.27682 | 67.05389:8360 69.03294:12926 73.02811:43288 73.06432:9890 81.03229:12823 83.0477:9371 86.09541:21134 87.04334:67828 89.05896:387738 90.0619:11879 95.04893:30411 99.04339:18889 111.04189:45463 113.05927:14954 133.08543:146203 134.08684:9891 137.05981:10721 144.71318:6682 155.07083:10098 173.39111:9454 177.11285:7148 195.11966:8453 388.89212:7036 406.26993:7750 449.97394:7982 | -4.319E-06 |
| POS12677 | tenacibactin H | 5.182 | 601.41455 | [M+H]+ | 601.41705 | 57.03287:78289 59.04859:1548720 73.02812:30158 73.06432:42032 85.06506:26493 87.04335:130026 87.07824:25916 89.05896:502687 101.05931:146764 103.07444:383406 115.07378:48419 117.05281:41343 117.08817:63891 131.07088:41107 133.08545:66329 147.10149:124154 161.11441:40724 173.42545:23346 417.47043:20268 | -4.1568E-06 |

**Differences in metabolites between the WJW group, the Model group and the Control group**

| Alignment ID | Metabolite name | Rt(min) | Expreiment Mz | Adduct type | Reference m/z | MS/MS spectrum | PPM |
|---|---|---|---|---|---|---|---|
| POS7201 | Epijasminoside A | 4.879 | 331.17203 | [M+Na]+ | 331.1741 | 59.04859:12242 81.48219:5523 89.05896:10799 98.88059:5631 107.7904:5492 161.48398:5151 331.17267:9964 | -6.2505E-06 |
| POS13453 | NPTX-643 | 5.638 | 644.42688 | [M+2H]2+ | 644.42419 | 59.04856:99508 73.02808:12471 87.0433:26044 89.05892:105064 95.53635:9875 101.05926:14035 103.07439:34219 133.08539:24962 | 4.17427E-06 |
| NEG5909 | netamine L | 0.999 | 300.24249 | [M-H]- | 300.2446 | 72.52529:5988 84.68958:5811 93.03925:186879 95.03763:283775 97.07645:7399 110.58968:5112 124.15676:183779 182.18414:20774 291.46884:5531 | -7.0276E-06 |
| POS9813 | Pubeside B | 4.98 | 485.3064 | [M+2H]2+ | 485.3085 | 59.04858:79486 60.05225:6663 61.8371:6356 73.02812:27787 73.06432:7785 80.0543:15872 87.04334:33021 89.05896:162685 92.46779:5702 101.0593:23513 102.06606:12967 103.07444:18479 124.07726:7576 133.08545:59366 147.10149:10895 177.11285:5607 | -4.3271E-06 |
| NEG3613 | Cimaterol | 9.081 | 218.1279 | [M-H]- | 218.13 | 128.02194:71183 129.03217:6533 133.70459:5598 | -9.6273E-06 |
| POS5059 | l-5-hydroxy-1-hep | 4.501 | 245.1351 | [M+Na]+ | 245.133 | 58.06512:9572 86.09541:13427 122.86917:5221 143.72749:5680 146.99809:6036 173.43526:6088 218.00317:6136 237.56071:5529 245.13695:37659 | 8.56678E-06 |
| POS4725 | ycero-pentulose) 1 | 5.566 | 233.09978 | [M+H]+ | 233.1019 | 55.01768:8584 57.03377:7507 57.06894:49350 57.18206:6569 58.07269:10056 67.05505:6206 81.03226:14436 83.08506:8660 85.06333:15404 103.12611:5356 111.04185:6677 112.5395:5665 121.06405:13022 125.05955:25938 126.06273:14620 167.10457:32994 167.83998:6425 168.11116:38598 173.43027:7378 183.91914:5373 | -9.0947E-06 |
| POS9459 | farneside A | 4.614 | 467.27295 | [M+H]+ | 467.27509 | 73.02812:51321 80.0543:16473 87.04335:104693 89.05896:517633 90.06191:29742 95.04894:19450 107.06821:13535 111.0419:32094 113.05927:15459 133.08545:160752 137.05637:15123 173.39111:37168 173.87758:17191 399.6514:12759 | -4.5797E-06 |
| POS13563 | hermoactinoamide | 6.305 | 651.4762 | [M+CH3OH+H]+ | 651.47998 | 57.03379:42684 59.04956:13713 164.73628:5198 650.09869:5839 | -5.8022E-06 |
| POS13492 | xy]-19-hydroxyurs | 4.853 | 647.41809 | [M+H]+ | 647.41528 | 59.04859:528102 73.02812:37374 85.06337:36406 87.04335:215381 89.05896:1493702 95.46825:25189 101.05931:125037 103.07444:212478 124.09804:30390 131.07088:38314 131.10634:28086 133.08545:545537 147.10149:101782 161.69572:23650 227.7915:22995 446.18515:25857 | 4.34034E-06 |
| POS7512 | s-19-Ene-1,2,4-Tr | 5.478 | 346.32935 | [M+H]+ | 346.33151 | 56.04953:18614 57.06989:9248 58.06512:15486 74.05886:12861 88.07445:24774 99.30854:6438 | -6.2368E-06 |
| POS12731 | 4,9,13,14-pentame | 5.551 | 604.38763 | [M+2H]2+ | 604.38501 | 59.04859:78126 67.015:9397 81.03072:6475 87.04335:8618 87.07824:6789 89.05896:85814 99.40212:5879 101.05931:13335 103.07445:23943 133.08545:24141 154.89267:5869 | 4.33499E-06 |
| POS10946 | Taurocholic acid | 8.928 | 533.32312 | [M+NH4]+ | 533.32544 | 85.06337:13517 104.10696:45688 126.02023:120155 145.09904:11422 160.20474:11112 184.07466:33281 208.06328:16524 209.13087:24366 227.13577:17589 | -4.3501E-06 |
| POS9391 | yonarasterol G | 6.563 | 463.29947 | [M+H]+ | 463.2973 | 55.12037:6647 147.88623:5357 180.24678:5464 | 4.68382E-06 |

**Differences in metabolites between the WJW group, the Model group and the Control group**

| Alignment ID | Metabolite name | Rt(min) | Expreiment Mz | Adduct type | Reference m/z | MS/MS spectrum | PPM |
|---|---|---|---|---|---|---|---|
| POS11637 | taumycin A | 5.068 | 558.35614 | [M+2H]2+ | 558.3537 | 59.04859:317628 73.02812:77858 73.06432:28561 76.76718:17249 80.0543:37095 87.04335:342822 87.06254:27695 88.04605:28998 89.05896:611285 90.06191:18244 96.08221:17910 101.05931:127267 102.06606:28792 103.03849:18756 103.07444:54981 109.07315:19595 117.05281:42712 133.08545:194131 147.10149:32335 161.22427:18152 170.08043:20278 173.21472:22817 | 4.36999E-06 |
| POS8857 | Salicyloyl-conkurc | 5.156 | 433.28711 | [M+2H]2+ | 433.28491 | 57.03287:122648 59.04859:3019070 59.05931:77019 60.05226:74211 65.04931:33097 69.06992:24835 73.02812:264160 73.06432:66059 80.0543:46306 85.06337:83297 87.04335:410670 87.06254:135765 87.07999:58686 89.05896:2636898 90.06191:104028 94.06947:95750 101.05931:266741 102.06606:26984 103.07444:610018 109.0756:42692 115.07378:33155 116.08248:45168 117.0909:43486 129.08911:70544 131.06766:38930 133.08545:929040 134.09018:38699 138.09604:22047 145.08403:24259 147.10149:303882 177.11285:83305 191.1256:74282 | 5.07749E-06 |
| POS9946 | Morusimic acid C | 5.061 | 492.31949 | [M+2H]2+ | 492.31729 | 58.04042:32820 59.04859:673719 60.05226:22571 65.04819:30212 69.06992:13995 73.02812:150374 73.06432:40802 80.0543:70867 85.06337:26951 87.04335:162683 87.06254:76816 87.07999:25394 89.05896:1259871 90.06191:47778 101.05931:88979 102.06606:40417 103.07444:144945 107.06821:13208 109.07315:43866 117.0909:22869 124.08022:12762 129.08911:22447 131.06766:24791 133.08545:464620 134.09018:26379 138.09604:11087 140.09358:16099 147.10149:74442 169.11278:10513 177.11285:54784 191.1256:15896 | 4.46866E-06 |
| POS12237 | copadiol decanoat | 6.3 | 581.42255 | [2M+H]+ | 581.41998 | 57.03287:583029 59.04858:187706 81.0699:15293 97.06461:6372 99.07938:49343 101.09422:14097 115.07378:50363 117.09089:20887 157.12312:40594 173.3911:10101 | 4.42021E-06 |
| POS13872 | Giganteumgenin D | 5.053 | 675.44958 | [M+H]+ | 675.44659 | 57.03287:53724 59.04859:1120360 73.02812:38305 87.04335:224956 87.07999:22732 89.05896:1106176 90.06191:21493 101.05931:168575 103.07444:380565 107.0254:19690 117.05553:24756 118.83812:18633 129.08911:21403 131.06766:41651 133.08545:349481 145.08403:19166 147.10149:165995 161.1188:22588 177.1078:20334 205.86365:21003 231.24901:24255 | 4.4267E-06 |
| NEG1724 | Histidine | 0.976 | 155.06726 | [M-H]- | 155.06947 | 58.04514:5773 59.83095:5668 79.0537:106586 80.66509:5785 81.14307:7922 87.11256:70161 93.15691:34894 95.03767:465257 97.03749:232436 97.07648:103337 111.15354:35922 120.89378:6292 121.51244:5309 137.19968:6037 151.60265:6598 154.24504:10632 | -1.4252E-05 |
| NEG1725 | Histidine | 1.079 | 155.06726 | [M-H2O-H]- | 155.06947 | 64.06541:5847 79.0537:191622 93.15691:22481 95.03767:519248 97.03749:252310 97.07648:109035 111.15354:21238 137.08247:9393 156.05357:9694 | -1.4252E-05 |
| NEG1570 | ropenyl)-5-vinylcy | 8.966 | 147.11578 | [M-H]- | 147.118 | 57.0112:14206 59.0295:55214 59.08405:27695 103.07276:20890 103.67512:5608 112.83764:5624 119.54141:5832 | -1.509E-05 |

**Differences in metabolites between the WJW group, the Model group and the Control group**

| Alignment ID | Metabolite name | Rt(min) | Expreiment Mz | Adduct type | Reference m/z | MS/MS spectrum | PPM |
|---|---|---|---|---|---|---|---|
| POS11021 | Leu Tyr Val Lys | 5.063 | 536.34656 | [M+2H]2+ | 536.34418 | 58.04137:17266 59.04859:301936 73.02812:94452 73.06432:21392 80.0543:43703 85.06337:21301 87.04335:130609 87.06079:36121 87.07999:15848 89.05896:783413 90.06191:27599 101.05931:45697 102.06606:45551 103.07444:85751 109.0756:23540 117.0909:18548 124.08022:22265 129.08911:16082 131.06766:18971 133.08545:313664 134.08684:17615 147.10149:39057 173.42545:16622 177.11285:32828 | 4.43745E-06 |
| POS13228 | Ferensimycin A | 5.773 | 629.42297 | [M+2H]2+ | 629.42578 | 57.03287:57295 59.04859:461322 69.06992:14182 73.02812:52708 73.06432:29320 85.06337:26788 87.04335:101916 87.06254:20498 87.07999:18603 89.05896:318566 94.06947:24179 101.05931:72536 103.07444:132810 115.07378:17623 129.08911:19577 133.08545:91906 141.32309:13970 147.10149:54603 193.21381:14327 | -4.4644E-06 |
| NEG9602 | PFCA-H | 5.373 | 556.98877 | [M-H]- | 556.98627 | 62.79358:5873 79.05221:8913 81.14308:5793 89.53181:5702 181.16978:6607 | 4.48844E-06 |
| POS9449 | voretin C\|olovoreti | 5.967 | 466.34497 | [M+H]+ | 466.34271 | 53.00208:10126 55.05365:9939 57.03287:1568713 58.03662:138237 59.04858:423905 60.05225:27359 69.06992:14513 81.0699:41265 87.04334:8140 97.06461:21210 99.07939:98160 100.08458:16083 101.09641:17642 115.07378:97125 116.0771:20054 117.0909:35484 157.12312:76096 158.12614:31178 | 4.84622E-06 |
| POS8539 | Lys Lys His | 4.885 | 412.26434 | [M+2H]2+ | 412.2666 | 57.03287:26233 58.04042:51039 59.04859:530500 65.04819:22388 69.06869:9260 71.04816:10028 73.02812:211329 73.06432:27828 80.0543:117431 85.06337:26355 87.04335:191352 87.06079:60341 87.07999:11348 88.04783:9405 89.05896:1205068 90.06191:36132 101.05931:68762 102.06606:69439 103.03849:14021 103.07444:110287 107.07059:8640 109.0756:34035 111.07206:13151 117.08817:12291 124.08022:28268 129.08911:16022 131.06766:24895 133.08545:404612 134.08684:21362 140.09358:15864 147.10149:40341 173.43036:11455 177.11285:39558 | -5.4819E-06 |
| POS10244 | Ergosterol Acetate | 5.62 | 505.34656 | [M+2H]2+ | 505.34427 | 57.03287:66480 59.04859:1008826 60.05226:28304 69.06868:10573 73.02812:56892 73.06432:28375 85.06337:26026 87.04335:95828 87.06079:24889 87.07999:30373 89.05896:400585 90.06191:14719 94.06947:29000 99.07939:8330 101.05931:99646 103.07444:229806 104.07732:12755 109.0756:7315 115.07378:20926 117.05281:15195 117.0909:28429 129.08911:15724 131.07088:11503 131.10634:13149 133.08545:109593 147.10149:84254 161.11441:18592 205.14238:9040 | 4.53156E-06 |
| NEG6244 | 5-Diamino-eikosa | 1.852 | 311.34546 | [M-H]- | 311.3432 | 85.13134:7977 87.1126:11482 111.14101:55249 | 7.25887E-06 |
| POS9814 | Liquoric acid | 4.921 | 485.32938 | [M+H]+ | 485.32709 | 57.03287:125270 59.04859:2843541 59.05931:71617 60.05226:39477 69.06992:16250 73.02812:37788 73.06432:36680 81.0699:15102 85.06337:51433 87.04335:258376 87.07999:53761 89.05896:1359929 90.06191:23239 99.07939:14334 101.05931:256138 101.09423:18458 103.03849:49684 103.07444:781300 104.07732:24005 105.09028:15188 115.07378:46380 117.05281:66066 117.0909:57051 129.08911:19021 131.06766:26963 133.08545:268187 143.1078:19690 145.08403:15776 147.10149:276400 161.11441:52580 | 4.71847E-06 |

**Differences in metabolites between the WJW group, the Model group and the Control group**

| Alignment ID | Metabolite name | Rt(min) | Expreiment Mz | Adduct type | Reference m/z | MS/MS spectrum | PPM |
|---|---|---|---|---|---|---|---|
| POS5532 | promethyl)chrome | 1.033 | 262.94507 | [M+H]+ | 262.94278 | 62.57782:6309 80.94772:11668 82.94373:53067 85.02795:14817 103.21838:7255 105.81583:6000 110.58839:5851 135.00177:100440 173.43027:5741 | 8.70912E-06 |
| POS9672 | Terragine D | 4.717 | 479.28229 | [M+H]+ | 479.28 | 62.54703:5555 89.05896:11415 98.65503:5455 193.53143:7135 243.06476:5573 | 4.778E-06 |
| POS2839 | minoethylarsonic | 9.075 | 169.97691 | [M+H]+ | 169.9792 | 50.41959:6511 55.0177:8175 55.05365:12372 55.93345:449948 65.27977:5757 67.05389:7732 69.93349:5878 70.95692:38693 72.93706:21210 79.05312:9070 81.06989:5857 87.94499:8464 88.96877:12257 96.95995:72113 100.07382:6120 110.05835:6676 111.98266:7744 113.96311:13822 114.97044:527374 128.95062:102406 137.98456:17548 146.95981:36415 | -1.3472E-05 |
| POS10622 | nigramide I | 8.81 | 521.33966 | [2M+H]+ | 521.33728 | 50.45496:6193 51.83419:6802 60.08023:43348 67.37577:5809 86.09538:89886 95.44818:6482 104.10693:163753 105.10874:19782 124.9995:22076 128.15237:5616 184.07458:227376 185.07628:8154 195.01427:6031 | 4.56518E-06 |
| POS12076 | Cycloposine | 5.121 | 574.3764 | [M+H]+ | 574.37378 | 59.04858:79125 69.92596:5668 73.02811:8315 87.04333:146374 89.05895:88243 101.0593:47183 103.03848:7634 103.07443:16938 117.0528:7801 131.06764:7907 133.08543:24716 147.10147:7331 175.0853:6157 180.86687:5702 198.75192:5900 270.37485:5190 | 4.56149E-06 |
| NEG5636 | -2-enyl]cyclopent | 1.985 | 291.22513 | [M-H]- | 291.22742 | 60.69548:5592 77.46813:7272 108.77018:6596 126.02459:14571 163.03734:12093 167.73914:5356 285.31952:6500 | -7.8633E-06 |
| POS8630 | anodipyrido[1,2-A | 5.274 | 418.28296 | [M+H]2+ | 418.28528 | 55.05366:22078 57.03287:125034 59.04859:2525705 60.05226:57428 65.04819:25872 67.05389:10159 69.06868:20235 73.02812:148140 73.06432:31841 80.0543:9936 81.06834:12473 85.06337:42060 87.04335:225535 87.06254:50750 87.07999:43845 89.05896:1302131 90.06191:37053 94.06947:61475 99.07939:9880 101.05931:181628 103.07444:478159 104.07732:19685 107.06821:12884 109.07315:21801 115.07378:23777 116.08248:26758 117.05281:13252 117.0909:36600 129.08911:35198 130.08389:12682 131.07088:18517 131.10634:10495 133.08545:347346 134.09018:17711 143.1078:13060 145.08403:17356 147.10149:199987 148.1028:10108 161.11441:21964 173.11685:8694 177.1078:26012 191.1256:18382 | -5.5465E-06 |
| POS9392 | yonarasterol G | 4.971 | 463.29962 | [M+2H]2+ | 463.2973 | 58.04042:63295 59.04859:504811 65.04819:21997 73.02812:167294 73.06432:25138 80.0543:96970 85.06506:25089 87.04335:177305 87.06079:66151 89.05896:1213248 90.06191:44246 101.05931:69166 102.06606:88313 103.07444:117189 109.07315:38830 111.07206:15858 117.08817:17058 124.08022:34782 129.08911:15408 131.06766:18088 133.08545:425593 134.08684:19498 147.10149:65143 154.63217:12540 173.43036:18277 177.11285:50763 | 5.00758E-06 |
| POS8411 | Leu Lys Gln | 4.526 | 402.26868 | [M+NH4]+ | 402.271 | 59.04859:82599 73.06432:9263 85.06337:7422 87.04335:61379 89.05896:874148 90.06375:11985 101.05931:6852 103.07444:22527 107.06821:8809 124.58938:5510 130.08389:5964 131.06766:16379 133.08545:359856 134.09018:8764 147.10149:17029 177.11285:35710 241.19559:6116 | -5.7673E-06 |

**Differences in metabolites between the WJW group, the Model group and the Control group**

| Alignment ID | Metabolite name | Rt(min) | Expreiment Mz | Adduct type | Reference m/z | MS/MS spectrum | PPM |
|---|---|---|---|---|---|---|---|
| POS9847 | Ile Val Leu Glu | 4.639 | 487.31018 | [M+H]+ | 487.3125 | 59.04859:292906 73.02812:30849 73.06432:28687 79.13319:12788 85.06506:12807 87.04335:175797 89.05896:1561067 90.06191:30701 101.05931:34060 103.07444:83540 107.60078:10197 117.6151:9986 129.08911:19638 131.06766:23717 133.08545:549733 134.09018:23234 147.10149:41072 173.43036:22546 177.11285:47516 | -4.7608E-06 |
| POS13468 | Fucoxanthin | 5.319 | 645.41791 | [M+2H]2+ | 645.41492 | 59.04859:479514 73.02812:81248 73.06432:51689 87.04335:120059 89.05896:855098 101.05931:131167 103.07444:167082 131.10312:32544 133.08545:362154 134.09018:28192 147.10149:79929 173.44016:26547 177.11285:50734 | 4.63268E-06 |
| POS12195 | Hugonone B | 5.191 | 579.37061 | [M+Na]+ | 579.36792 | 59.04859:32268 87.04335:11722 89.05896:47033 96.43513:8825 114.28255:8750 133.08545:19594 274.88956:8878 | 4.64299E-06 |
| POS5381 | 4-hydroxyphenyl)p | 4.875 | 257.15601 | [M+H]+ | 257.15369 | 55.05365:14169 57.06896:19251 67.05389:17654 69.06868:10464 71.08547:6875 77.8651:5469 81.06833:33341 88.07445:17903 95.08477:27614 102.09264:9084 119.08333:8294 137.1322:7120 | 9.02184E-06 |
| NEG6396 | cavernene B | 1.849 | 316.26703 | [M-H]- | 316.26471 | 96.82237:6153 148.11794:11831 182.01009:5761 | 7.33563E-06 |
| POS10173 | oxypoly-angioic ac | 4.92 | 502.35495 | [M+NH4]+ | 502.3526 | 57.03287:43776 59.04859:1181274 73.02812:24017 73.06432:28593 81.0699:9594 85.06337:19759 87.04335:112047 87.07999:18800 89.05896:666070 90.06191:15021 101.05931:129104 101.09423:14091 103.07444:446719 104.07732:10150 115.07378:16237 117.05281:87278 117.0909:38393 129.08911:10081 131.06766:36632 131.10634:12713 133.08545:169742 145.08403:12324 147.10149:206987 148.1028:8780 161.11441:54314 163.5305:8100 167.5183:10008 173.43036:15006 191.1256:24685 205.14238:19739 485.25378:8436 | 4.67799E-06 |
| POS12078 | Cycloposine | 5.857 | 574.37646 | [M+2H]2+ | 574.37378 | 57.03287:33910 59.04859:136280 73.02812:28360 81.03072:9795 85.06506:14046 87.04335:41005 87.07999:8481 89.05896:132471 101.05931:27133 103.07444:28370 115.07378:8781 129.08911:12956 133.08545:35650 147.10149:15380 | 4.66595E-06 |
| POS9634 | (-)-Asbestinine 2 | 5.142 | 477.31635 | [M+2H]2+ | 477.3187 | 59.04856:188902 60.05123:8391 73.02808:14107 73.06429:6009 85.06333:6872 87.0433:35492 87.06249:7482 89.05891:211438 90.06187:7440 101.05926:18100 103.07439:35720 117.0854:5972 118.5326:5493 133.08537:62620 147.10141:15339 256.90271:6885 462.67981:5708 | -4.9233E-06 |
| POS9491 | Ebastine | 5.095 | 469.30042 | [M+Na]+ | 469.29807 | 57.03287:26413 58.04137:13386 59.04859:331842 65.04819:7736 69.06992:9934 73.02812:68830 73.06432:19481 80.0543:23919 85.06337:13467 87.04335:816974 87.06254:31098 88.04605:14580 89.05896:377655 101.05931:311944 102.06384:21137 103.03849:9401 103.07444:63120 109.0756:10877 115.07378:10911 117.05281:29543 117.08817:7011 131.06766:15910 133.08545:112635 145.08403:6826 147.10149:23166 | 5.00748E-06 |

**Differences in metabolites between the WJW group, the Model group and the Control group**

| Alignment ID | Metabolite name | Rt(min) | Expreiment Mz | Adduct type | Reference m/z | MS/MS spectrum | PPM |
|---|---|---|---|---|---|---|---|
| POS9112 | CHEMBL470965 | 5.06 | 448.29343 | [M+2H]2+ | 448.29581 | 57.03287:68804 58.04137:27871 59.04859:1628296 60.05226:39454 65.04819:30631 69.06868:25025 73.02812:244775 73.06432:63531 80.0543:67590 85.06337:54377 87.04335:304086 87.06079:140329 87.07999:39268 87.56366:13261 88.04605:17000 89.05896:2262140 90.06191:80290 94.06947:36830 101.05931:156011 102.06606:47961 103.07444:331285 104.07732:16371 105.09028:10549 107.07059:17179 109.07315:66728 115.07378:15284 116.08248:23736 117.0909:23972 124.08022:24049 129.08911:43356 130.08389:22054 131.06766:42495 131.10634:17943 133.08545:784664 134.08684:42267 138.09604:9933 140.09358:13738 145.08403:15352 146.09132:11834 147.10149:176709 177.11285:79602 191.1256:33857 | -5.309E-06 |
| POS8759 | Crustecdysone | 4.798 | 427.28738 | [M+H]+ | 427.285 | 57.03287:96412 59.04859:2660545 60.05226:41071 69.06992:13467 73.02812:38638 73.06432:30020 81.0699:8670 85.06337:43292 87.04335:254559 87.07999:30664 89.05896:1612168 90.06191:30564 99.07939:9460 101.05931:212539 101.09641:11841 103.03849:74057 103.07444:696734 104.07732:15618 105.09028:12907 107.07059:17053 115.07378:13781 117.05281:58399 117.0909:30087 129.08911:22180 131.06766:23475 131.10634:9323 133.08545:323802 143.10413:11097 145.08403:20325 147.10149:280810 161.11441:35992 191.1256:26189 | 5.57005E-06 |
| POS8592 | Salmeterol | 4.605 | 416.28442 | [M+NH4]+ | 416.2821 | 59.04859:257618 69.03294:18634 73.02812:10737 73.06432:9467 85.06337:8628 87.04335:70807 89.05896:1019619 90.06191:24733 101.05931:23922 103.07444:79191 107.07059:9047 117.05281:10679 131.07088:14556 133.08545:402513 134.09018:12916 147.10149:58041 173.39111:8539 177.11285:42085 191.1256:10972 | 5.57314E-06 |
| NEG5800 | ZINC2171653 | 8.938 | 296.97531 | [M-2H]2- | 296.97769 | 63.70482:5931 97.07649:38057 227.58353:6403 | -8.0141E-06 |
| POS1363 | lroxy-1,4-benzoqu | 0.991 | 125.02081 | [M+Na]+ | 125.0232 | 101.17503:6058 117.59318:5031 | -1.9116E-05 |
| POS11968 | Leu Ala Pro Leu Glu | 5.879 | 570.35242 | [M+H]+ | 570.34967 | 59.04858:23768 66.82805:6713 72.18837:5650 86.0954:18019 87.04334:7782 89.05895:14613 94.83833:6230 101.0593:7966 104.10696:112184 146.98277:12615 249.88678:6454 301.56808:5957 | 4.8216E-06 |
| POS11188 | Pro Lys Val Lys Ala | 5.459 | 542.3634 | [2M+H]2+ | 542.36603 | 57.03287:86391 59.04859:1612797 60.05226:37101 65.04819:23288 69.06868:20449 73.02812:122944 73.06432:40164 80.0543:32205 83.04932:16424 85.06337:38825 87.04335:284572 87.06254:57144 87.07999:39721 89.05896:1251753 90.06191:29175 91.494:19192 94.06947:41465 101.05931:275969 102.06606:14246 103.07444:386000 104.07732:23684 109.07315:36497 116.0798:27127 117.05281:20293 117.0909:43102 129.09227:40122 131.06766:24424 133.08545:350447 134.08684:14581 147.10149:157161 148.1028:22169 161.1188:29090 177.11285:31342 191.1256:33280 267.83408:14932 311.62006:14301 328.30508:16016 407.45084:15379 | -4.8491E-06 |
| NEG3242 | droxytrihomometh | 3.201 | 206.0881 | [M-H]- | 206.08569 | 59.08403:43411 69.11722:14541 73.11618:107827 80.05276:11726 99.1286:9273 101.14601:9415 126.02459:50621 161.03183:6211 161.23819:8132 | 1.16942E-05 |

**Differences in metabolites between the WJW group, the Model group and the Control group**

| Alignment ID | Metabolite name | Rt(min) | Expreiment Mz | Adduct type | Reference m/z | MS/MS spectrum | PPM |
|---|---|---|---|---|---|---|---|
| NEG2671 | Gongrine | 1.501 | 187.08127 | [M-H]- | 187.08369 | 58.0983:58602 62.21653:5617 79.05367:93919 89.09513:6340 97.08466:229408 97.18124:5655 114.16125:6013 115.15741:29718 123.03893:6622 125.22068:17593 125.24475:15819 127.20192:17880 145.23547:7066 | -1.2935E-05 |
| POS10256 | Ile Phe Val Lys | 5.247 | 506.33606 | [M+2H]2+ | 506.33359 | 57.03287:71605 59.04859:1823837 60.05226:56884 65.04819:17257 69.06992:25750 73.02812:167496 73.06432:57882 80.05276:35937 85.06337:52209 87.04335:315450 87.06079:100667 87.07999:59629 89.05896:1945502 90.06191:67040 94.06947:47589 99.07939:16286 101.05931:212129 103.03849:24585 103.07444:477548 104.07732:22879 105.09028:12167 107.06821:14019 109.0756:51116 115.07378:18939 116.08248:25256 117.0909:37772 127.07501:16344 129.08911:43925 131.06766:37075 133.08545:727718 134.08684:52970 138.09604:23348 140.09358:12996 143.1078:13173 145.08403:14221 147.10149:272258 148.1028:14220 161.1188:20799 173.11685:15015 177.11285:68870 191.1256:53325 | 4.87821E-06 |
| POS10996 | Cyclomicrosin | 5.97 | 535.39191 | [2M+H]+ | 535.38928 | 57.03287:347741 59.04858:111809 75.69939:6349 81.0699:7431 86.09711:7236 87.04334:6061 89.05895:13908 99.07938:32438 104.10696:6865 115.07378:24910 117.09089:13262 157.12312:41837 184.06929:20632 192.94875:5606 | 4.91231E-06 |
| POS10168 | Delaminomycin A | 5.361 | 502.31866 | [M+2H]2+ | 502.31619 | 57.03287:11288 59.04859:205091 60.05226:11892 67.015:23236 73.02812:8479 73.06432:15896 81.03072:8298 87.04335:23478 89.05896:155828 101.05931:16909 103.07445:53432 117.05281:17865 131.07088:13840 133.08545:41663 147.10149:13157 151.0811:10240 177.11287:6631 204.758:6350 316.92041:6433 | 4.91722E-06 |
| POS2577 | Monomethyl adipat | 2.601 | 161.07834 | [M+H]+ | 161.0808 | 56.94137:11350 67.67966:6202 72.93703:15146 105.0694:7747 115.05251:19043 117.05547:13777 118.06284:12438 132.07886:15843 132.97005:6489 160.0741:24901 161.07481:6579 | -1.5272E-05 |
| POS8986 | Grevirobstol B | 4.913 | 441.30276 | [M+H]+ | 441.30029 | 57.03287:91869 59.04859:2216200 60.05226:38474 69.06869:14908 73.02812:28853 73.06432:28137 85.06337:23678 87.04335:285232 87.07999:26585 89.05896:622334 90.06191:14456 99.07939:13362 101.05931:162838 101.09423:9388 103.03849:55808 103.07444:518486 104.07732:11131 107.07059:9778 115.07378:27765 117.05281:52568 117.0909:56797 129.09227:9778 131.06766:18558 133.08545:70772 147.10149:131341 161.11441:37325 | 5.5971E-06 |
| POS7812 | N-Palmitoyltaurine | 5.844 | 362.24283 | [M+H-H2O]+ | 362.2453 | 57.06989:6537 67.05389:14997 69.06992:8783 79.05312:19566 81.06834:11710 82.06503:43602 91.0539:13094 93.06841:20613 95.08477:9569 96.08018:11956 107.08486:11704 214.23677:6474 264.26468:32965 264.41238:5526 350.88223:5713 | -6.8186E-06 |
| POS13018 | -D-glucosyl betuli | 5.182 | 618.43945 | [M+NH4]+ | 618.4364 | 59.04859:448789 73.06432:32302 87.04335:50417 89.05896:304779 90.06191:27372 101.05931:63464 103.07444:146189 117.05281:60982 117.0909:24036 131.07088:26206 133.08545:75904 147.10149:72940 163.85435:20030 173.39603:31478 474.44147:19340 | 4.93179E-06 |

**Differences in metabolites between the WJW group, the Model group and the Control group**

| Alignment ID | Metabolite name | Rt(min) | Expreiment Mz | Adduct type | Reference m/z | MS/MS spectrum | PPM |
|---|---|---|---|---|---|---|---|
| POS9508 | tumonoic acid G | 5.05 | 470.31366 | [M+H]+ | 470.31119 | 59.04856:210663 64.28428:6020 73.02809:39989 73.06429:12336 80.05426:16006 87.04331:50212 87.0625:18074 87.0782:6990 89.05892:304845 90.06371:9022 101.05927:28035 102.06602:6567 103.07439:47648 109.07555:7757 133.08539:95973 147.10143:14785 177.11278:14535 | 5.25184E-06 |
| NEG9597 | efpodoxime?proxe | 8.148 | 556.11475 | [M-H]- | 556.11749 | 58.03277:6804 95.15706:5698 370.90424:6061 | -4.927E-06 |
| POS1464 | 5-dihydrooxazole | 0.764 | 128.01889 | [M+CH3OH+H]+ | 128.0164 | 53.03773:43432 54.03372:13280 55.0177:42032 55.05365:56029 57.03286:7783 57.04396:19937 58.99794:11420 67.04092:11162 68.98123:315590 69.06991:9599 69.9913:6332 71.04815:11644 80.04813:7127 81.06989:7155 82.01235:106684 82.06342:70860 86.05938:6850 86.99104:288728 100.02438:233782 101.02003:12023 105.0024:308647 109.02666:6951 110.00877:16486 118.03259:66900 119.02752:30303 123.0126:31600 127.03808:34422 128.01854:61966 128.06833:12319 | 1.94506E-05 |
| POS9269 | Chaxalactin B | 4.713 | 457.29761 | [M+H]+ | 457.2951 | 57.03287:16353 59.04859:529559 73.02812:14687 73.06432:12692 85.06506:15007 87.04335:97771 87.07999:15167 89.05896:994740 90.06191:21788 101.05931:51281 103.03849:11047 103.07444:149086 104.07732:7846 107.06821:9425 129.08911:11107 131.06766:10006 131.10634:8119 133.08545:293362 134.09018:9588 147.10149:73929 177.11285:19039 191.1256:8962 | 5.4888E-06 |
| POS12032 | PC-M4 | 5.227 | 572.37628 | [M+2H]2+ | 572.37341 | 57.03286:9966 59.04858:221267 73.02811:28340 73.06431:7596 85.06505:8617 87.04333:77892 87.06252:8328 87.07997:6421 89.05895:322147 90.0619:11336 94.06946:7232 99.61956:6140 101.0593:44944 102.06383:8446 103.07443:60197 104.10696:21277 109.07558:9611 115.77686:5817 131.06764:7148 133.08543:104042 134.08682:6520 147.10147:28222 177.11284:14357 184.07465:6833 415.7355:5473 | 5.01421E-06 |
| POS8799 | Mebeverine | 4.958 | 430.26196 | [M+2H]2+ | 430.25946 | 59.04859:51989 67.015:8720 86.36885:5594 87.04335:13277 89.05896:76175 101.05931:6941 103.07445:10649 131.06766:6788 133.08545:16632 135.28865:6161 173.43036:7527 | 5.81045E-06 |
| POS13293 | Scaphopetalumate | 5.659 | 633.40973 | [M+2H]2+ | 633.4129 | 59.04859:127985 80.32882:16383 87.04335:24626 89.05896:122135 101.05931:27054 103.07444:37583 103.53445:14955 133.08545:45083 147.10149:17551 162.54245:15598 173.39111:24998 189.11574:16272 209.53416:14642 | -5.0046E-06 |
| POS12430 | ngeloylzygadenin | 5.333 | 590.3717 | [2M+H]2+ | 590.36871 | 59.04859:142035 87.04335:29038 89.05896:142146 103.07444:33614 130.18591:17652 133.08545:65233 308.32666:18721 436.48868:16883 | 5.06463E-06 |
| NEG6717 | Schultesine | 1.055 | 329.15332 | [M-H]- | 329.15079 | 80.64178:6422 93.03928:74284 95.03767:32222 124.15681:27274 177.93195:6010 | 7.68645E-06 |
| POS13718 | NAD | 8.769 | 663.11243 | [M+H]+ | 663.10907 | 126.32156:5312 166.99335:5442 173.43034:13072 256.35541:5511 | 5.06704E-06 |
| POS11488 | Aliskiren-[d3] | 6.453 | 552.39777 | [M+Na]+ | 552.40057 | 60.08122:5983 72.73411:5317 73.3144:6683 76.72816:5385 104.10692:20045 139.75227:5697 184.06921:7460 | -5.0688E-06 |
| NEG9583 | (E)-Ceftriaxone | 6.016 | 553.03607 | [M-H]- | 553.03888 | 76.75741:7106 79.0537:6830 121.28275:5667 172.79352:5907 210.16214:6500 250.67091:6197 270.48953:5435 | -5.081E-06 |

**Differences in metabolites between the WJW group, the Model group and the Control group**

| Alignment ID | Metabolite name | Rt(min) | Expreiment Mz | Adduct type | Reference m/z | MS/MS spectrum | PPM |
|---|---|---|---|---|---|---|---|
| POS7510 | thylnonanedioylca | 4.862 | 346.22552 | [M+2H]2+ | 346.22296 | 57.03287:29942 58.04042:76672 59.04859:875504 60.05226:16977 65.04819:46132 69.06992:7833 73.02812:301787 73.06432:24861 80.0543:87657 85.06337:31198 87.04335:155382 87.06079:98721 89.05896:1272129 90.06191:48954 94.06947:11967 101.05931:49615 102.06606:58963 103.07444:119498 107.07059:17606 109.07315:34147 124.08022:24388 129.08911:18888 131.06766:12967 133.08545:358139 134.08684:19133 147.10149:39424 177.11285:33961 | 7.39408E-06 |
| POS9239 | Ilekudinol A | 5.051 | 455.31937 | [M+H]+ | 455.3168 | 57.03287:102228 59.04859:1811090 60.05226:29341 69.06992:11679 73.02812:24976 73.06432:91429 85.06337:16261 87.04335:99042 87.07999:12841 89.05896:216625 90.06374:9159 99.07939:10766 101.05931:96706 101.09423:12097 103.03849:13055 103.07444:357020 115.07378:35759 117.05281:92245 117.0909:73378 131.06766:64145 133.08545:19462 147.10149:47996 161.11441:43254 | 5.64442E-06 |
| POS9028 | Nakijiquinone B | 5.157 | 444.27756 | [M+CH3OH+H]2+ | 444.27499 | 57.03286:10691 59.04858:120785 67.01498:18566 74.60526:5510 81.03071:8738 87.04333:11223 89.05894:108782 101.05929:7136 103.07442:25807 126.29716:5232 133.08542:32302 147.10147:10585 156.52419:6353 177.11282:6748 | 5.78471E-06 |
| POS7359 | U 6796 | 4.768 | 339.23547 | [M+H]+ | 339.23291 | 57.03287:107158 59.04859:2537525 60.05226:41069 69.06992:7865 73.02812:29093 73.06432:57512 85.06337:17847 87.04335:129956 87.07999:13310 89.05896:412178 90.06375:8792 99.07939:7426 101.05931:68198 103.03849:39782 103.07444:344578 104.07732:8444 115.07378:11308 117.05281:145545 117.0909:37751 131.07088:31810 133.08545:17737 147.10149:47546 161.11441:15716 | 7.54644E-06 |
| POS9551 | nyl apo-6'-lycoper | 6.863 | 473.34387 | [M+H]+ | 473.34131 | 51.72378:6127 53.48267:6634 91.91269:5447 97.12214:6040 219.6438:5558 337.36746:5935 381.37701:5360 | 5.40836E-06 |
| POS10297 | PC(17:1(10Z)/0:0) | 6.412 | 508.33618 | [M+H]+ | 508.33881 | 55.6239:5924 59.04858:6614 60.08025:18263 86.09541:45672 104.10697:293443 105.1111:10635 124.99956:13625 148.10667:5865 173.38622:11353 184.06931:103155 295.92383:6376 | -5.1737E-06 |
| NEG4148 | opentadecanone C | 2.49 | 238.22028 | [M-H]- | 238.2177 | 101.31654:6837 108.03613:9828 108.22439:5969 124.04993:7581 180.11699:6444 | 1.08304E-05 |
| POS8350 | thylethyl)phenyl]su | 5.664 | 398.23859 | [M+Na]+ | 398.23599 | 59.04856:6062 71.14339:6012 130.37427:5976 | 6.52879E-06 |
| POS9933 | Herbasterol | 5.372 | 491.33173 | [M+2H]2+ | 491.33432 | 55.05366:26013 57.03287:120930 59.04859:2295520 60.05226:72763 65.04931:26636 69.06868:24696 73.02812:137599 73.06432:47595 80.05276:16213 81.06834:12172 83.0851:15285 85.06337:63016 87.04335:313542 87.06254:72873 87.07999:72223 88.04605:17042 89.05896:1561190 90.06191:56459 94.06947:70155 99.07939:11920 101.05931:283891 103.04074:16838 103.07444:528793 104.07732:29033 107.07059:11558 109.07315:26968 115.07378:31105 116.08248:30635 117.05281:20081 117.0909:46183 123.08884:12106 129.08911:44046 131.07088:32080 131.10634:29423 133.08545:521258 134.09018:28042 138.09604:18277 145.08403:25715 147.10149:286550 148.1028:17335 161.11441:33870 177.11285:45030 191.1256:49261 205.14238:11468 | -5.2714E-06 |

**Differences in metabolites between the WJW group, the Model group and the Control group**

| Alignment ID | Metabolite name | Rt(min) | Expreiment Mz | Adduct type | Reference m/z | MS/MS spectrum | PPM |
|---|---|---|---|---|---|---|---|
| POS8528 | Flabellinone | 5.051 | 411.2923 | [M+CH3OH+H]+ | 411.2897 | 57.03287:44003 59.04859:859429 59.05931:20887 60.05226:12857 71.04816:8536 73.02812:24879 73.06432:171605 85.06337:7432 87.04335:100162 87.07999:7724 89.05896:129800 101.05931:40620 103.0385:28408 103.07445:135499 115.07378:9791 117.05281:193132 117.0909:28669 131.06766:88047 133.08545:30173 161.11441:9903 | 6.32158E-06 |
| NEG1841 | Tricholomic acid | 1.226 | 159.03804 | [M-H]- | 159.04059 | 55.08413:10747 59.08403:18302 69.11721:6679 73.11618:8054 99.07355:8781 159.03728:13510 | -1.6034E-05 |
| POS11608 | Revandchinone 4 | 5.097 | 557.34216 | [M+H]+ | 557.34509 | 57.03287:23495 59.04859:229775 73.02812:38939 73.06432:14431 80.05276:16103 87.04335:946634 88.04783:16418 89.05896:252195 101.05931:259222 103.03849:54702 103.07444:55777 115.07378:17740 117.05281:41938 131.07088:24598 133.08545:89166 147.10149:29268 | -5.2571E-06 |
| POS5314 | Russulanorol | 5.197 | 255.15587 | [M+Na]+ | 255.15849 | 57.06988:6670 78.09728:6172 98.81725:5879 149.29048:5845 216.18271:5881 255.16071:8834 | -1.0268E-05 |
| POS10624 | cdysone-20,22-m | 6.142 | 521.35156 | [M+H]+ | 521.34882 | 51.77413:6985 56.04951:9219 60.08023:146083 69.06989:7297 71.07258:29433 86.09538:428126 87.09915:50395 95.08474:5568 98.98412:10856 104.10693:69240 105.10875:9331 124.99951:119508 126.00197:6355 184.0746:1123078 185.07629:177466 186.07527:13060 | 5.2556E-06 |
| POS7978 | lysylprolyllysine | 4.576 | 372.25784 | [M+H]+ | 372.2605 | 59.04859:448099 69.03294:16725 73.02812:11457 73.06432:9359 85.06337:15701 87.04335:73439 87.07999:7755 89.05896:1144522 90.06191:28135 101.05931:27742 103.07444:127799 105.09028:7020 107.06821:9930 117.05281:19430 129.08911:7743 131.07088:12052 133.08545:401910 134.08684:12480 147.10149:78159 151.09705:8489 177.11285:28023 191.1256:15673 | -7.1455E-06 |
| NEG4220 | decyltetrahydrothi | 1.413 | 241.20226 | [M-H]- | 241.1996 | 55.8771:6330 70.7528:6757 73.42717:5996 74.81602:5965 82.7948:5707 87.11253:6721 89.07528:10927 161.14594:13424 204.73056:5733 232.00209:6128 | 1.10282E-05 |
| NEG2928 | -Phenyl-L-cystein | 3.148 | 196.04639 | [M-H]- | 196.04372 | 99.32591:5903 153.19771:5391 153.42589:6134 161.03622:34233 161.28214:5408 | 1.36194E-05 |
| POS13541 | Quinovin | 4.775 | 650.42969 | [M+NH4]+ | 650.42621 | 59.04858:50737 87.04333:31364 89.05894:259203 103.07442:10850 133.08542:111104 147.10147:18945 147.1283:10857 173.38618:22166 191.7171:12443 192.78191:12033 473.44424:10940 | 5.35034E-06 |
| NEG2806 | hyl-6-(2-methylbu | 5.066 | 191.15807 | [M-H]- | 191.1554 | 57.01118:21492 59.02947:116695 85.13128:6248 87.04276:7689 87.11253:11088 91.05847:6309 101.05437:13474 103.07272:208186 104.07503:7912 111.14092:14668 135.65366:5751 147.117:10377 | 1.39677E-05 |
| POS9867 | SCHEMBL1643131 | 5.969 | 488.32437 | [M+H]+ | 488.32169 | 59.04858:23033 64.29315:5737 82.54288:7031 89.05895:11043 94.77686:5356 101.0593:6085 151.42064:6293 | 5.48819E-06 |
| POS9650 | cyclo[10.8.0.02,9.04 | 6.48 | 478.32645 | [M+2H]2+ | 478.32913 | 92.10029:5338 271.37048:5825 308.74551:5511 | -5.6028E-06 |
| POS6640 | Salicin | 0.892 | 304.25275 | [M+H]+ | 304.2554 | 110.79853:5377 118.67968:5474 | -8.7098E-06 |
| POS11187 | Pro Lys Val Lys Ala | 5.675 | 542.3631 | [M+H]+ | 542.36603 | 57.03284:35229 59.04856:470812 73.02808:47687 73.06429:21024 85.06333:14921 87.0433:71874 89.05891:394650 94.06942:17086 101.05926:67976 103.07439:118733 129.08905:13049 133.08537:92892 147.10141:43828 177.11278:14554 | -5.4023E-06 |

**Differences in metabolites between the WJW group, the Model group and the Control group**

| Alignment ID | Metabolite name | Rt(min) | Expreiment Mz | Adduct type | Reference m/z | MS/MS spectrum | PPM |
|---|---|---|---|---|---|---|---|
| POS14038 | MONENSIN | 5.599 | 688.45917 | [M+2H]2+ | 688.46289 | 59.04859:447999 73.02812:75560 85.06506:27413 87.04335:96532 87.06254:39067 89.05896:532809 101.05931:67741 103.07444:129273 133.08545:188542 147.10149:60605 177.11285:27077 | -5.4033E-06 |
| POS9549 | Val Ile Leu Asp | 5.261 | 473.29962 | [M+CH3OH+H]2+ | 473.29691 | 57.03287:13304 59.04859:167174 67.015:23341 73.02812:6643 81.03072:7557 87.04335:29208 89.05896:170959 101.05931:12395 103.07444:44551 133.08545:57064 147.10149:8679 158.09198:8681 | 5.72579E-06 |
| POS10743 | Axillarine C | 6.387 | 525.37146 | [M+H]+ | 525.36859 | 60.08024:31618 70.04918:5882 71.07259:7051 86.09539:95816 87.09917:9695 104.10695:40976 120.02661:5949 124.99953:23066 163.24342:5148 167.61609:5305 184.07463:315921 185.07632:48014 293.36038:6355 | 5.46283E-06 |
| POS11994 | bengamide G | 5.561 | 571.39209 | [M+2H]2+ | 571.3952 | 59.04856:119837 87.0433:16278 89.05892:99058 101.05926:20124 103.07439:30982 133.08539:21094 145.95105:6874 146.86797:6536 147.09758:7523 173.43027:15425 280.6557:6597 311.86816:7253 | -5.4428E-06 |
| POS11320 | seudotrienic acid | 6.138 | 547.37109 | [M+H]+ | 547.37408 | 69.62679:6764 86.0954:16902 88.90752:6739 104.10696:19850 116.29496:6737 168.67908:6925 184.07465:34999 185.07634:14910 186.07532:14030 318.24524:6938 476.40054:8121 | -5.4624E-06 |
| POS8117 | Methoxybutyrfenta | 5.846 | 381.25635 | [M+H]+ | 381.2536 | 55.05366:59197 56.04863:36764 57.06896:87857 58.07272:9338 67.04092:12889 67.05389:116800 68.04951:8904 68.05674:18427 69.06868:54600 70.06431:37694 70.07313:11344 71.08419:35277 79.05313:141014 80.05737:23471 81.05579:16697 81.06834:107621 82.06343:589187 83.06721:102276 83.0851:42505 84.08786:9734 85.10045:12190 91.0539:21022 93.06841:205623 94.07143:43152 95.04894:10832 95.08477:71566 96.08018:105786 97.0831:24824 97.09953:25755 98.98415:8786 107.08487:72153 108.08663:20951 109.10007:29213 110.09557:9670 121.09844:49051 122.10349:12156 123.11524:11544 135.11646:42561 136.11703:15726 149.13002:7634 263.25275:9608 264.26471:164125 265.27322:189890 266.2782:7267 | 7.21305E-06 |
| POS8449 | Celerioside E | 4.781 | 405.24515 | [M+Na]+ | 405.24789 | 59.04858:12415 73.02811:5900 87.04333:5505 89.05894:18713 108.92649:5637 133.08542:6137 173.58739:6753 176.6682:5636 216.32614:5671 354.49524:6265 | -6.7613E-06 |
| POS9215 | o-teleocidin A1\|JB | 5.548 | 454.30905 | [M+2H]2+ | 454.3063 | 57.03287:18774 59.04859:295508 73.02812:18046 87.04335:17452 89.05896:89480 101.05931:15349 102.5617:5680 103.07444:37998 133.08545:14549 147.10149:7192 158.81593:5572 173.43036:11397 | 6.05318E-06 |
| POS13742 | voninin 6\|pavoninin | 4.854 | 664.44543 | [M+NH4]+ | 664.44177 | 59.04859:245674 73.02812:21700 85.06506:16253 87.04335:104584 87.07999:14111 87.48628:13562 89.05896:727168 90.06375:20733 101.05931:49600 103.07444:99762 131.06766:14181 133.08545:315046 147.10149:67503 173.38132:15537 177.1078:44899 191.13127:18117 | 5.50838E-06 |
| NEG1702 | Antibiotic BN 227 | 1.207 | 154.05396 | [M-H]- | 154.05119 | 67.07545:6313 79.05367:305536 95.03962:13577 97.07645:243799 137.08243:14250 154.05191:25417 | 1.7981E-05 |
| NEG3554 | Maybridge4_00094 | 4.314 | 216.12827 | [M-H]- | 216.1255 | 54.79307:5975 62.37378:5944 62.44574:6431 126.02459:83638 127.03272:8735 128.0219:33050 131.66733:5889 | 1.28166E-05 |

**Differences in metabolites between the WJW group, the Model group and the Control group**

| Alignment ID | Metabolite name | Rt(min) | Expreiment Mz | Adduct type | Reference m/z | MS/MS spectrum | PPM |
|---|---|---|---|---|---|---|---|
| POS10096 | o-ethyl)-1H-indol- | 5.052 | 499.34589 | [M+H]+ | 499.34311 | 57.03287:86547 59.04859:1682228 60.05226:28452 60.08025:22141 73.02812:27972 73.06432:34939 85.06506:20956 86.09541:22343 87.04335:225468 87.07999:22317 87.09918:10002 89.05896:368289 99.08151:16498 101.05931:141151 101.09641:13981 103.03849:26235 103.07444:394975 104.10697:155502 105.10879:48503 106.11105:12970 115.07378:30682 117.05281:36056 117.0909:66113 131.07088:29743 133.08545:50670 147.10149:88991 161.1188:40566 184.07466:38011 185.07635:29515 186.07535:21842 | 5.56731E-06 |
| POS9492 | Simplexin B | 5.947 | 469.31122 | [M+H]+ | 469.314 | 57.03286:13280 59.04857:8582 60.08023:42136 61.08377:5509 86.09539:78112 87.09917:7598 101.05711:5882 104.10695:351710 105.10876:52893 124.99953:13642 158.75143:5535 184.07462:122027 185.07631:24648 215.35905:5728 | -5.9235E-06 |
| POS9371 | S,14aS)-4b-deoxy | 8.922 | 462.26657 | [M+Na]+ | 462.26379 | 55.05365:11330 67.05389:7267 79.05312:6647 81.0699:21159 85.06337:14778 91.85783:5756 93.06841:17016 95.08477:15604 105.06945:18444 107.08486:28719 109.10006:12051 117.06913:8309 119.08612:16590 121.09843:11999 126.02023:204008 131.08377:6468 133.09863:20057 135.11646:11219 143.08206:5647 145.09904:15259 147.11681:16331 157.10197:13389 159.11723:23023 161.13197:7431 173.43034:8036 189.12691:6211 199.14366:13544 201.16266:6546 208.06328:19880 209.13087:18352 210.6722:5690 213.1633:9412 227.13577:9477 | 6.01388E-06 |
| NEG1138 | nopropyl)amino)e | 8.349 | 133.08339 | [M-H]- | 133.0806 | 59.12987:5186 65.09239:9815 73.30846:5403 74.24288:5666 101.05437:61494 102.54467:6000 116.05923:91887 117.06135:6233 133.08284:6874 | 2.09647E-05 |
| POS10148 | o-20(29)-en-3-ox | 4.727 | 501.3248 | [M+H]+ | 501.32199 | 59.04859:428126 73.02812:17540 73.06432:15485 85.06337:15767 87.04335:140079 87.07999:11619 89.05896:1095919 90.06191:26964 101.05931:57188 103.03849:10138 103.07444:128774 104.68195:7630 107.06821:11142 117.05281:7244 129.08911:13689 131.06766:23962 133.08545:371861 134.08684:7068 145.08403:7231 147.10149:74325 173.39111:7151 177.11285:22029 191.1256:8284 | 5.60518E-06 |
| POS11210 | Milbemycin A4 | 4.968 | 543.33673 | [M+H]+ | 543.33368 | 57.03287:14949 59.04859:239886 73.06432:11215 87.04335:780433 89.05896:221587 95.14056:9272 101.05931:168653 102.06384:12893 103.03849:121933 103.07444:70587 115.07378:11425 117.05281:73307 131.07088:21478 133.08545:66765 147.10149:23043 | 5.61349E-06 |
| POS4373 | hyl-4-oxochromer | 9.18 | 220.01317 | [M+H]+ | 220.01598 | 55.93344:24593 69.45864:6515 70.35236:5317 72.93571:6290 72.9598:36824 89.96286:5966 90.96998:15094 99.74139:5356 116.97396:24667 132.97009:16945 133.97685:15029 134.96141:6939 146.40657:5172 160.96077:8092 178.97508:7638 | -1.2772E-05 |
| POS10886 | ,6R)-3,4,5-trihydr | 4.653 | 530.98499 | [M+H]+ | 530.98798 | 70.42718:6286 72.86754:5890 73.02811:7430 87.04333:17348 89.05894:109585 113.05925:5994 133.08542:36838 173.39598:9305 364.62674:6157 | -5.631E-06 |
| POS10893 | Leu Asp Asn Arg | 4.448 | 531.2915 | [M+2H]2+ | 531.28851 | 72.4194:7773 86.09536:17901 87.05376:24342 89.05891:81013 133.08537:43158 146.06093:8085 188.06995:30052 303.14871:8317 | 5.62783E-06 |
| NEG166 | Glycerol | 0.634 | 91.04296 | [M-H]- | 91.0401 | 90.13486:71847 91.13872:16344 | 3.14147E-05 |

**Differences in metabolites between the WJW group, the Model group and the Control group**

| Alignment ID | Metabolite name | Rt(min) | Expreiment Mz | Adduct type | Reference m/z | MS/MS spectrum | PPM |
|---|---|---|---|---|---|---|---|
| POS9268 | Communesin A | 4.548 | 457.2626 | [M+2H]2+ | 457.25977 | 69.03294:7465 73.02811:24149 83.04932:7884 87.04334:40421 89.05896:183975 95.04893:15484 99.04339:10666 111.04189:25470 113.05927:14135 133.08543:49846 155.07083:7130 168.79677:5700 173.42543:5884 | 6.18904E-06 |
| POS13291 | Rubuside D | 4.768 | 633.40399 | [M+H]+ | 633.40039 | 59.04859:129463 73.02812:15925 73.06432:14343 87.04335:100421 89.05896:635802 90.06191:15554 101.05931:27993 103.07444:34781 109.3968:10547 129.08911:10945 131.06766:19760 133.08545:258954 146.70763:11522 147.10149:23064 171.92455:11422 177.11285:28269 | 5.68361E-06 |
| POS10666 | Azadironol | 4.574 | 523.30011 | [M+2H]2+ | 523.3031 | 65.42842:22264 69.03294:42168 73.02811:122011 73.06432:36758 81.03229:60085 83.0477:36457 87.04334:258228 89.05896:1561065 90.0619:25113 91.04643:26116 95.04893:103995 99.04339:36849 111.04189:117303 113.05927:50583 131.07088:41791 133.08543:537202 134.08684:40688 137.0598:44873 151.09305:23499 155.07083:50347 157.08505:26813 177.10779:46777 178.6362:20443 | -5.7137E-06 |
| POS10476 | Delaminomycin B | 5.631 | 516.3349 | [M+2H]2+ | 516.33191 | 57.03379:10046 59.04858:86059 73.02812:6818 87.04334:6345 89.05896:65314 101.0593:11654 103.07444:22657 117.05553:6404 126.02024:28145 133.08545:22148 137.23573:6146 137.4812:5640 147.10149:9745 166.02432:6052 170.09471:5838 319.87314:5954 | 5.79085E-06 |
| POS8367 | lihydroxy-4,6-dim | 4.604 | 399.25629 | [M+H]+ | 399.25339 | 59.04859:1010931 73.02812:28224 73.06432:32070 85.06337:29569 87.04335:253725 87.07999:24881 89.05896:3000530 90.06191:61240 101.05931:73680 103.03849:27828 103.07444:237082 107.06821:20239 117.05281:16405 129.08911:20548 131.07088:33861 133.08545:866283 134.08684:26756 147.10149:118198 177.11285:54567 | 7.26356E-06 |
| POS9327 | Verdine | 4.611 | 460.30869 | [M+H]+ | 460.30579 | 59.04859:114620 69.03294:12691 73.06432:6779 74.03423:6222 87.04335:50910 89.05896:563675 90.06191:12397 98.87637:6070 101.05931:12596 103.07444:33731 107.07059:8782 117.36894:5773 131.06766:15377 133.08545:233407 134.09018:11356 147.10149:29190 173.43036:12408 177.11285:25475 | 6.30016E-06 |
| POS8566 | Metachromin S | 4.905 | 414.30331 | [M+NH4]+ | 414.30042 | 57.03287:50975 59.04859:1296740 60.05226:20845 73.02812:16496 73.06432:173960 85.06506:6581 87.04335:32499 87.07999:13341 89.05896:289834 90.06191:7439 99.07939:11105 101.05931:68599 101.09423:10172 103.07445:356011 104.07732:7444 115.07378:12790 117.05281:405628 117.0909:62243 118.0574:9386 131.06766:159921 132.0724:6890 133.08545:18985 145.08403:11452 147.10149:83947 161.07928:16601 161.11441:45642 173.39111:11217 175.09528:13656 | 6.97561E-06 |
| POS12083 | esteryl-Phosphoch | 5.341 | 574.39618 | [M+2H]2+ | 574.39954 | 59.04858:74523 65.13841:8808 87.04334:16033 89.05896:105291 101.0593:19257 103.07444:17919 104.18001:8297 117.97754:7694 133.08545:39980 147.10149:14681 177.11285:9324 | -5.8496E-06 |

**Differences in metabolites between the WJW group, the Model group and the Control group**

| Alignment ID | Metabolite name | Rt(min) | Expreiment Mz | Adduct type | Reference m/z | MS/MS spectrum | PPM |
|---|---|---|---|---|---|---|---|
| POS12413 | 1alpha,25-dimeth | 4.757 | 589.37689 | [2M+H]+ | 589.37341 | 59.04859:207411 73.02812:28959 73.06432:17706 83.31817:11252 87.04335:127032 87.07999:13074 89.05896:909512 90.06191:18586 92.93743:10793 101.05931:55264 103.07444:76697 115.78223:12321 131.07088:21915 133.08545:333416 147.10149:45428 173.43526:12231 177.11285:26196 183.19818:10830 265.44962:12614 358.28094:12755 | 5.90458E-06 |
| POS8184 | cosahexaenoyl Gly | 4.669 | 386.27216 | [M+NH4]+ | 386.2692 | 59.04859:166957 87.04335:13037 89.05896:192528 99.55555:6222 101.05931:10841 103.07444:50844 117.05281:11954 133.08545:55275 147.10149:27949 277.32327:6236 | 7.66305E-06 |
| POS11846 | Valnemulin | 5.139 | 565.36353 | [M+2H]2+ | 565.36688 | 59.04858:259007 73.02811:50874 73.06431:30974 78.36626:16107 80.05274:24239 87.04333:86028 89.05894:518801 101.05929:33520 103.07442:72740 123.34731:18975 133.08542:191946 147.10147:40567 173.43033:40313 177.11282:31289 194.32018:20204 446.87405:18802 | -5.9254E-06 |
| POS8515 | Marineosin A | 5.563 | 410.285 | [M+2H]2+ | 410.28201 | 57.03287:49031 59.04859:876259 60.05226:18268 65.04931:7682 67.85582:5822 73.02812:41810 73.06432:19938 85.06506:12180 87.04335:46344 87.06254:8629 87.07999:9658 89.05896:216433 90.06191:9081 94.06947:14511 101.05931:57353 103.07444:141593 105.61979:6301 115.07378:9548 116.08248:7404 117.0909:18444 131.07088:6858 133.08545:42086 147.10149:37057 161.1188:8715 173.39111:10399 185.21703:6740 195.57704:6851 211.2645:5911 | 7.28767E-06 |
| NEG2901 | ethyl-1-cyclohexer | 1.277 | 195.17252 | [M-H]- | 195.17551 | 59.08406:6652 75.09884:7763 79.05372:22833 97.07651:682352 159.03737:12622 161.03632:573850 196.04454:63048 | -1.532E-05 |
| POS13957 | horosyloxydocosar | 5.5 | 681.44598 | [M+2H]2+ | 681.44189 | 57.03287:42599 59.04859:477045 73.02812:59066 74.02876:28013 87.04335:110902 89.05896:615241 101.05931:83280 103.07444:203065 133.08545:260993 147.10149:91056 153.90381:33434 200.93614:29460 332.91391:27498 387.02127:27199 551.54889:30427 555.54846:31138 | 6.00198E-06 |
| POS11256 | ]-1-oxopropan-2- | 5.527 | 545.33667 | [M+H]+ | 545.33337 | 59.04856:17063 60.08022:9496 69.95734:5940 86.09537:31472 89.05892:17490 104.10693:201760 105.10874:26047 146.98271:20803 191.11984:5359 319.55377:6838 | 6.05134E-06 |
| NEG2410 | ylsulfanyl-butyl)-t | 1.209 | 177.04959 | [M-H]- | 177.0526 | 79.05367:6822 119.17749:7333 142.03583:7854 | -1.7001E-05 |
| POS6925 | 2,6-Dimethyl-2,5-h | 4.716 | 317.15616 | [M+Na]+ | 317.15921 | 58.04042:9487 59.04859:41583 73.02812:39470 80.0543:10378 89.05896:57821 133.08545:13578 173.43036:13731 173.4745:5458 176.86003:5563 207.79285:5325 | -9.6166E-06 |
| POS11727 | side, 3,17-dihydro | 6.137 | 561.33856 | [M+H]+ | 561.34198 | 59.04858:6808 71.33195:6263 89.05895:8051 98.42394:5281 104.10696:5405 539.50934:6287 | -6.0925E-06 |
| POS7737 | Songorine | 4.504 | 358.24142 | [M+NH4]+ | 358.23837 | 59.04859:187338 73.06432:14123 87.04335:81153 89.05896:1400826 90.06191:26297 101.05931:5973 103.07444:37700 107.07059:15584 129.08911:7096 131.07088:12867 133.08545:486213 134.09018:12227 147.10149:31980 173.39603:8242 177.1078:51863 | 8.51388E-06 |

**Differences in metabolites between the WJW group, the Model group and the Control group**

| Alignment ID | Metabolite name | Rt(min) | Expreiment Mz | Adduct type | Reference m/z | MS/MS spectrum | PPM |
|---|---|---|---|---|---|---|---|
| POS12976 | Arg Leu Lys Trp | 5.233 | 616.39667 | [M+2H]2+ | 616.39288 | 57.03287:32646 59.04859:466780 73.02812:84005 73.06432:23669 80.05276:22272 85.06337:22002 87.04335:305077 87.07999:25052 88.04783:31059 89.05896:844076 90.06374:38897 101.05931:189509 103.07444:144583 117.0909:21604 133.08545:324054 134.08684:20407 145.08778:22085 147.10149:57823 173.43034:32907 175.88412:21732 177.11285:45332 191.1256:28014 424.63242:19913 | 6.14868E-06 |
| POS7780 | Lavoltidine | 5.053 | 360.24237 | [M+2H]2+ | 360.23929 | 57.03287:60582 58.04137:15193 59.04859:1675242 60.05226:41959 65.04819:49136 69.06868:12856 71.04945:7803 73.02812:166575 73.06432:21791 80.0543:17844 85.06337:32565 87.04335:150591 87.06079:69315 87.07999:19379 89.05896:1115052 90.06191:37919 94.06947:40386 101.05931:95564 102.06606:9818 103.07444:234132 104.07732:12819 105.09259:7544 107.07059:9096 109.07315:31309 116.08248:19999 117.0909:14133 129.08911:19810 131.08699:10183 133.08545:285434 147.10149:86939 177.11285:15536 191.1256:13700 | 8.54987E-06 |
| POS11259 | spicatin | 4.74 | 545.35059 | [M+H]+ | 545.34723 | 59.04859:622712 73.02812:57318 73.06432:44968 85.06506:20813 87.04335:345886 87.07999:33732 89.05896:2375766 89.07882:41091 90.06191:41952 101.05931:115064 103.07444:226217 107.07059:34198 129.08911:32429 130.08708:18221 131.07088:54070 133.08545:1103812 134.09018:32979 147.10149:182907 173.43526:23820 177.11285:122377 191.1256:26938 | 6.16121E-06 |
| POS12732 | a-D-glucopyranos | 5.051 | 604.42444 | [M+H]+ | 604.42072 | 57.03287:33024 59.04859:715256 73.02812:24066 73.06432:21369 87.04335:79713 89.05896:540829 90.06191:24670 101.05931:93361 103.07444:301644 106.69563:20633 117.05281:69426 117.07185:24236 117.0909:29699 131.06766:39433 131.10634:25272 133.08545:178801 147.10149:147013 147.56622:18270 161.11441:37347 173.43034:49754 183.02257:18996 342.37314:23317 343.1636:21411 | 6.15465E-06 |
| POS9216 | )-Deoxymethymyc | 5.52 | 454.31253 | [M+2H]2+ | 454.31561 | 57.03287:81565 59.04859:1490384 60.05226:42113 65.04819:17652 69.06992:15052 73.02812:92610 73.06432:32519 80.0543:7485 83.0851:9630 85.06337:28999 87.04335:111027 87.06079:23074 87.07999:26781 89.05896:621674 90.06191:21255 94.06947:40236 99.07939:11675 101.05931:127551 103.07444:278122 104.07732:11744 115.07378:21818 117.05553:9391 117.0909:23259 129.08911:15064 131.06766:18057 131.10312:8823 133.08545:152206 145.08403:8057 147.10149:98787 161.11441:19346 173.43034:8036 177.1078:10809 191.1256:10621 | -6.7794E-06 |
| POS13580 | opyranosyl-6'-pim | 5.044 | 653.40875 | [M+H]+ | 653.40472 | 57.87836:7624 74.77715:7412 173.43031:12962 565.78345:6817 | 6.16769E-06 |
| POS10665 | Azadironol | 4.732 | 523.29987 | [M+H]+ | 523.3031 | 60.08022:20622 69.03291:6328 81.09808:5146 86.09536:34227 89.05891:9720 104.10692:177748 124.9995:9959 173.43027:11599 184.06921:65670 293.67346:5616 | -6.1723E-06 |
| POS1957 | Iodomethane | 1.23 | 142.9382 | [M+H]+ | 142.9351 | 55.01682:24101 55.05365:43290 56.94231:9903 57.06895:7810 64.52962:5774 68.04829:7093 69.03294:65214 70.06555:11197 70.07311:6937 71.04815:20688 83.04768:6460 97.02763:12463 98.05796:6868 143.03427:91947 | 2.16882E-05 |

**Differences in metabolites between the WJW group, the Model group and the Control group**

| Alignment ID | Metabolite name | Rt(min) | Expreiment Mz | Adduct type | Reference m/z | MS/MS spectrum | PPM |
|---|---|---|---|---|---|---|---|
| NEG4317 | imycin: Oxazinom | 1.208 | 244.04961 | [M-H]- | 244.0464 | 79.05367:6909 97.07645:11114 163.03734:11778 175.2373:5440 | 1.31532E-05 |
| NEG635 | Isocaproic acid | 5.528 | 115.07261 | [M-H2O-H]- | 115.0757 | 59.08403:7579 69.07899:6232 98.04861:26594 100.04587:57877 103.87704:5463 115.07258:24219 116.05923:61702 | -2.6852E-05 |
| NEG5018 | -)-Quinolactacin A | 0.985 | 269.12595 | [M-H2O-H]- | 269.12909 | 59.08403:22810 89.13125:479299 90.13486:14166 93.03925:1773201 95.03763:1125557 151.06712:48152 153.06755:73754 | -1.1667E-05 |
| POS11669 | Cholyllysine | 4.829 | 559.36816 | [M+H]+ | 559.3717 | 57.03287:53343 59.04859:1266598 73.02812:47890 73.06432:36520 83.0477:18243 85.06337:49893 87.04335:307887 87.07999:49722 89.05896:2215204 90.06191:44290 101.05931:198639 103.07444:401994 107.07059:17771 117.05281:25782 129.08911:34704 131.06766:55594 133.08545:772419 134.08684:22090 145.08403:25622 147.10149:237818 164.1385:16358 173.43036:39536 177.1078:47093 191.1256:39333 372.55954:16853 | -6.3285E-06 |
| POS10994 | )10-natafuranamin | 5.346 | 535.35645 | [2M+H]2+ | 535.35303 | 57.03287:58664 59.04859:1185472 60.05226:28537 69.06992:18596 73.02812:105121 73.06432:35161 85.06337:35816 87.04335:220489 87.06254:56060 87.07999:54007 89.05896:1072132 90.06191:31375 94.06947:34509 101.05931:145346 103.03849:19681 103.07444:333188 104.07732:15184 109.0756:17790 117.0909:30649 129.08911:23306 131.06766:17923 133.08545:354329 134.08684:24095 143.10413:15855 147.10149:166337 173.38622:19003 177.11285:38861 191.1256:25034 314.09201:16848 | 6.38831E-06 |
| POS10449 | 11-Ketofusidic acid | 4.823 | 515.34082 | [M+H]+ | 515.33752 | 57.03287:49450 59.04859:1449929 60.05226:28917 73.02812:46166 73.06432:39569 83.0477:12608 85.06337:38266 87.04335:237243 87.07999:34396 89.05896:1809246 90.06191:40554 101.05931:169273 103.03849:32397 103.07444:433003 104.07732:12532 115.07378:14554 117.05281:30054 117.0909:22782 129.08911:30253 131.07088:40680 133.08545:538518 134.08684:22924 145.08403:18900 147.10149:231683 161.11441:17324 177.11285:35046 191.1256:24259 338.48813:11314 | 6.40357E-06 |
| POS10097 | o-ethyl)-1H-indol- | 5.375 | 499.34631 | [M+H]2+ | 499.34311 | 57.06989:6828 59.04859:39692 60.08025:63374 73.02812:7840 86.09541:121291 87.04335:54077 87.09918:40902 89.05896:41583 101.05931:12329 103.07445:11368 104.10697:595170 105.10879:221811 106.11105:73269 110.11542:7478 124.99956:26496 127.00428:7786 131.83476:7331 133.08545:14332 184.07466:188160 185.07635:108954 186.07535:100589 187.07695:9257 318.11121:7126 | 6.40842E-06 |
| POS11690 | Chrysopentamine | 6.214 | 560.29828 | [M+2H]2+ | 560.30188 | 84.90689:5295 104.10469:17228 133.38271:6134 171.91002:5442 405.91846:5953 | -6.4251E-06 |
| POS3633 | Vitispirane | 5.393 | 193.16249 | [M+H]+ | 193.15924 | 55.05365:9474 58.06512:6990 60.2657:5893 67.05389:17243 79.05312:6183 81.06834:19681 93.07034:7061 95.08477:11928 105.06945:11578 109.10007:9376 147.11681:15425 147.72034:5289 | 1.68255E-05 |
| POS13035 | hydroxyl acyl DFC | 5.428 | 619.39294 | [M+2H]2+ | 619.39697 | 59.04859:85033 81.03072:14718 87.04335:23911 89.05896:107737 103.07445:20833 109.73177:12931 133.08545:35925 173.39111:25291 | -6.5063E-06 |
| POS12658 | yl)-deoxysphing-4 | 6.837 | 600.46539 | [M+H]+ | 600.46149 | 55.53491:6757 83.89444:5594 89.05896:6132 199.65767:5901 203.06952:5435 | 6.495E-06 |

**Differences in metabolites between the WJW group, the Model group and the Control group**

| Alignment ID | Metabolite name | Rt(min) | Expreiment Mz | Adduct type | Reference m/z | MS/MS spectrum | PPM |
|---|---|---|---|---|---|---|---|
| POS5740 | Diuron | 0.883 | 270.97675 | [M+CH3OH+H]+ | 270.98001 | 57.06986:13553 71.08544:8173 79.61764:5963 135.00177:139311 263.71185:6056 | -1.203E-05 |
| NEG3854 | Axinynitrile A | 2.089 | 228.17908 | [M-H]- | 228.1758 | 79.05367:26955 84.02768:5510 97.08672:9662 224.09412:6638 | 1.43749E-05 |
| POS5588 | Pentachloropheno | 0.958 | 264.85098 | [M+CH3OH+H]+ | 264.85428 | 50.29755:6809 62.46424:5260 77.06998:5493 80.94775:1146872 81.04482:13340 82.94376:26061 83.67702:6314 84.91025:6728 90.97557:17328 104.10696:13956 122.07161:108671 138.90465:7942 144.04625:26688 | -1.246E-05 |
| POS9168 | Z)-Eicosadienoylc | 5.645 | 452.3707 | [M+H]+ | 452.37399 | 50.29372:6425 55.05452:8946 60.08024:22619 69.0699:11333 83.08508:9886 85.02798:101310 173.43521:8354 196.08905:5390 260.33258:6729 | -7.2727E-06 |
| POS12412 | rmectin B2b aglyc | 4.601 | 589.341 | [2M+H]2+ | 589.3371 | 69.03294:85645 73.02811:270464 79.66347:74724 81.03229:100442 81.9725:68091 87.04334:605848 89.05896:3143008 90.0619:150195 95.04893:184828 99.04339:77337 111.04189:266398 113.05927:86412 133.08543:931530 137.0598:126393 155.07083:95403 155.12476:64704 199.10141:68832 | 6.6176E-06 |
| POS10671 | ydrokarounidiol dia | 6.136 | 523.38159 | [M+H]+ | 523.37811 | 53.00208:11481 55.05365:13411 57.03287:1944042 58.03662:30775 59.04858:574645 69.06868:19389 81.06833:58211 86.0954:20230 97.06461:30371 99.07938:156839 101.09422:33453 104.10696:110452 115.07378:141694 117.09089:61504 157.12312:161664 184.06929:48328 | 6.64911E-06 |
| POS8125 | obutyryl bakkenoli | 5.055 | 382.25488 | [M+H]2+ | 382.25821 | 57.03287:59844 58.04137:17722 59.04859:1631943 59.05931:39530 60.05226:41665 65.04819:48386 69.06868:14164 71.04816:8664 73.02812:166879 73.06432:24983 74.03149:8137 80.0543:25562 85.06337:41089 87.04335:187063 87.06079:65798 87.07999:23344 87.56366:8138 89.05896:1267394 90.06191:33992 94.06947:29465 101.05931:98242 102.06606:12479 103.03849:10363 103.07444:233574 104.07732:12470 107.07059:12391 109.07315:29079 116.08248:14366 117.0909:17535 129.08911:21471 131.06766:19420 131.10634:10398 133.08545:342321 134.08684:11035 147.10149:90922 177.11285:26646 191.1256:12000 | -8.7114E-06 |
| POS9032 | Yibeissine | 4.8 | 444.31442 | [M+NH4]+ | 444.3111 | 57.03286:35610 59.04858:1024461 59.0593:26431 60.05225:16286 73.02811:12469 73.06431:18139 83.04768:7020 85.06504:16909 87.04333:86153 87.07997:17599 89.05894:779869 90.0619:15131 99.07938:6169 101.05929:79470 103.03848:14076 103.07442:363566 105.09026:8277 115.07376:6370 117.05279:68029 117.09087:20251 129.09224:7563 131.07086:11202 131.10631:6301 133.08542:179811 145.08401:9892 147.10147:178089 161.11438:20425 173.396:8609 191.12556:18245 205.14235:7523 | 7.47224E-06 |
| POS9286 | adecahydro-3,5-d | 5.395 | 458.29333 | [M+2H]2+ | 458.29001 | 57.03379:7869 59.04859:141324 67.015:16456 73.06432:13343 81.02916:6543 87.04335:13792 89.05896:83653 101.05931:11880 103.07445:29819 117.05281:18938 131.06766:11334 133.08545:21246 147.10149:13494 158.092:5847 191.1256:6630 439.38196:6118 | 7.24432E-06 |
| POS15209 | Ganosinensin C | 6.569 | 815.55103 | [M+2H]2+ | 815.54559 | 52.05935:5830 397.22595:5531 610.95099:6742 | 6.67038E-06 |

**Differences in metabolites between the WJW group, the Model group and the Control group**

| Alignment ID | Metabolite name | Rt(min) | Expreiment Mz | Adduct type | Reference m/z | MS/MS spectrum | PPM |
|---|---|---|---|---|---|---|---|
| POS13386 | njaoamine B | 6.449 | 639.46747 | [2M+H]+ | 639.4632 | 53.00208:6937 57.03287:435875 59.04858:140883 73.37244:5391 81.0699:18838 97.06461:7991 99.07938:30953 101.09641:6964 110.6359:6188 115.07378:34385 117.09089:10878 157.12312:27488 489.76151:5649 | 6.67748E-06 |
